# Altered microRNA Transcriptome in Cultured Human Liver Cells upon Infection with Ebola Virus

**DOI:** 10.3390/ijms22073792

**Published:** 2021-04-06

**Authors:** Idrissa Diallo, Jeffrey Ho, Benoit Laffont, Jonathan Laugier, Abderrahim Benmoussa, Marine Lambert, Zeinab Husseini, Geoff Soule, Robert Kozak, Gary P. Kobinger, Patrick Provost

**Affiliations:** 1 CHU de Québec Research Center, Department of Microbiology, Infectious Diseases and Immunology, Faculty of Medicine, Université Laval, Quebec, QC G1V 4G2, Canada; Idrissa.Diallo@crchudequebec.ulaval.ca (I.D.); jeffrey.ho@crchudequebec.ulaval.ca (J.H.); laffont.benoit@gmail.com (B.L.); laugierjonathan@yahoo.fr (J.L.); abderrahim.benmoussa@umontreal.ca (A.B.); Marine.Lambert@crchudequebec.ulaval.ca (M.L.); zeinab.husseini@crchudequebec.ulaval.ca (Z.H.); Gary.Kobinger@crchudequebec.ulaval.ca (G.P.K.); 2 Special Pathogens Program, National Microbiology Laboratory, Public Health Agency of Canada, Winnipeg, MB R3B 3M9, Canada; geoff.soule@phac-aspc.gc.ca (G.S.); rob.kozak@sunnybrook.ca (R.K.); 3 Division of Microbiology, Department of Laboratory Medicine & Molecular Diagnostics, Sunnybrook Health Sciences Centre, Toronto, ON M4N 3M5, Canada; 4 Département de Microbiologie Médicale, Université du Manitoba, Winnipeg, MB R3E 0J9, Canada; 5 CHUQ Research Center/CHUL Pavilion, 2705 Blvd Laurier, Room T1-65, Quebec, QC G1V 4G2, Canada

**Keywords:** Ebola virus, microRNA, liver cell, transcriptome, small RNA sequencing

## Abstract

Ebola virus (EBOV) is a virulent pathogen, notorious for inducing life-threatening hemorrhagic fever, that has been responsible for several outbreaks in Africa and remains a public health threat. Yet, its pathogenesis is still not completely understood. Although there have been numerous studies on host transcriptional response to EBOV, with an emphasis on the clinical features, the impact of EBOV infection on post-transcriptional regulatory elements, such as microRNAs (miRNAs), remains largely unexplored. MiRNAs are involved in inflammation and immunity and are believed to be important modulators of the host response to viral infection. Here, we have used small RNA sequencing (sRNA-Seq), qPCR and functional analyses to obtain the first comparative miRNA transcriptome (miRNome) of a human liver cell line (Huh7) infected with one of the following three EBOV strains: Mayinga (responsible for the first Zaire outbreak in 1976), Makona (responsible for the West Africa outbreak in 2013–2016) and the epizootic Reston (presumably innocuous to humans). Our results highlight specific miRNA-based immunity pathways and substantial differences between the strains beyond their clinical manifestation and pathogenicity. These analyses shed new light into the molecular signature of liver cells upon EBOV infection and reveal new insights into miRNA-based virus attack and host defense strategy.

## 1. Introduction

The largest and longest outbreak of Ebola virus disease (EVD) since 1976 occurred between 2013 and 2016 in West Africa and caused over 28,000 infections and 11,000 deaths, highlighting its potential as a global public health threat [1,2]. Ebolavirus, along with Cuevavirus and Marburgvirus, constitute the three main genera of Filoviridae family. The genus Ebolavirus (EBOV) consists of six known species, which include Zaire ebolavirus (virus type: ZEBOV), Sudan ebolavirus (SUDV), Bundibugyo ebolavirus (BDBV), Taï Forest ebolavirus (TAFV), Bombala ebolavirus (BOMV) and Reston ebolavirus (RESTV) [3]. Except for RESTV and BOMV, which are presumably nonpathogenic in humans, the four others cause life-threatening disease [4,5]. Notably, ZEBOV has garnered the most attention out of the viral hemorrhagic fevers due to its high case fatality rate (up to 90%), making the virus one of the most virulent and deadly pathogens.

EBOV can be transmitted among humans mainly through direct contact with the body fluids of an infected individual or surfaces and materials contaminated with these fluids [2,6]. The molecular mechanisms governing EBOV pathogenesis have been gradually uncovered [7]. After entering host cells via clathrin-mediated endocytosis and, to a larger extent, through micropinocytosis [8,9], the virus impairs the immune system by targeting macrophages and dendritic cells [10,11]. The proinflammatory molecules secreted by these cells lead to a potentially fatal “cytokine storm” [11]. The virus also travels through the lymphatic system, where it infects secondary lymphoid organs and reaches the liver where it replicates uncontrollably [12].

EBOV has developed several mechanisms to counteract host innate immune responses [13] mainly through its nine encoded proteins, namely Nucleoprotein (NP), Glycoprotein (GP), soluble GP (sGP), small soluble GP (ssGP), RNA-dependent RNA polymerase (L), and the structural viral proteins VP24, VP30, VP35 and VP40 [14]. VP24 and VP35 are mainly associated with viral pathogenicity, as they prevent the nuclear import of Signal Transducer and Activator of Transcription 1 (STAT1) [15] and the recognition by Retinoic acid Inducible Gene 1 (RIG-I) and Melanoma Differentiation-Associated protein 5 (MDA5) binding to viral dsRNA, respectively [16]; these interactions subsequently block the production of signaling antiviral interferons [17].

MicroRNAs (miRNAs) are short, 19 to 24-nucleotide (nt) long noncoding RNA sequences engaged in the post-transcriptional regulation of gene expression [18]. More than 60% of the human protein-coding genes have been under selective pressure to maintain pairing to miRNAs [19], which participate in many selectively conserved regulatory interactions [18].

Host-encoded miRNAs are believed to be critical modulators of viral infection, as their regulation is altered by the host inflammatory response [20,21]. They may exhibit an inhibitory effect on the EBOV genome [22,23]. The inhibition of certain miRNAs, such as hsa-miR-1246, hsa-miR320a and hsa-miR-196b-5p, in human umbilical vein endothelial cells (HUVEC) is believed to reduce EBOV GP-mediated cytotoxicity [23]. Duy et al. [24] showed changes induced by EBOV in circulating miRNA profiles, highlighting the importance of exploring miRNAs as both therapeutic and diagnostic tools.

miR-150-3p was shown to inhibit viral GP and VP40 protein expression through a viruslike particle system in 293T cells [22]. miR-29b-3p, a suppressor of the transcription factor nuclear factor kappa-light-chain-enhancer of activated β cells (NF-κB) and an actor in the antiviral interferon response, was shown to be upregulated during EBOV infection [25]. Other human miRNAs have been predicted in silico to target EBOV RNA [26]. Recent evidence suggests that, in addition to its viral proteins, EBOV can presumably encode its own miRNAs to subvert host immune defenses [27,28,29].

Many attempts have been made to compare EBOV strains [30,31,32] and/or to rationalize the lack of pathogenicity in humans infected with RESTV compared to the other strains [4,15,33,34]. All Ebolavirus genomes are quite similar. The Ebola and Reston viruses contain seven linearly arranged genes (coding for the nine proteins described above). Variations are commonly noted in the intergenic regions and within specific areas of the genes encoding the GP, NP and L [35]. In their genomic sequences, the Makona and Mayinga strains showed 97% identity [36]. The specific characteristics of each strain were often analyzed in regard to the host and not the pathogen. Interferon-stimulated genes have been found to be more highly expressed in the RESTV, and the inability of the RESTV to attenuate the interferon response contributes to its reduced pathogenicity [33]. Notably, RESTV does not trigger TLR4 signaling, which is required to activate NF-κB and, subsequently, generate a “cytokine storm” [37].

Histological studies from autopsies indicated that the liver is where the viral particles are the most concentrated [38]. In addition to presenting indications of significant hepatic damage [39], liver provides the best histopathological features characterizing filovirus infections [40]. Furthermore, the challenge of isolating emerging viruses appears to be overcome thanks to liver cell lines, like Huh7 cells (hepatocellular carcinoma cells, HCC), which give methodological advantages and comparable sensitivity to viral isolation using other cell types or suckling BALB/c laboratory mice [41]. The whole-genome expression profiling of liver samples (HepG2 and animal tissue) with EBOV infection has provided precious data on the extensive dysregulation of the metabolism and adaptive immunity through modulation by viral proteins [42,43].

However, to the best of our knowledge, in the EVD context, comparative genomewide analyses of miRNA expression in human liver cells have not yet been reported. In this study, we used small RNA-sequencing (sRNA-Seq) to analyze the modulation of Huh7 cells miRNA transcriptome (miRNome) following infection with the prototype EBOV Mayinga, responsible for the first Zaire outbreak in 1976 [44]; the EBOV Makona, responsible for the most recent West Africa outbreak [36]; and the epizootic RESTV, presumably innocuous to humans [45]. To better understand and characterize the actors and dynamics of host–virus interactions in this context, we investigated the differentially expressed (DE) miRNAs at the early (24 h) and late (96 h) stages of infection, followed by Kyoto Encyclopedia of Genes and Genomes (KEGG) and Gene Ontology (GO) term enrichment analyses of their target genes. The profiling of Huh7 cell miRNAs over time prior to infection showed selective and virus-specific modulations of the three most abundantly expressed miRNAs: miR-122-5p, miR-148a-3p and miR-21-5p. DE miRNAs and monitoring the expression level of key players in coagulation (factors F2, F3, F8 and F10), apoptosis (Bax, Bcl2 and Casp-3) and cellular adhesion molecules Intercellular Adhesion Molecule-1 (ICAM-1) and Vascular Cell Adhesion Molecule-1 (VCAM-1) unraveled an Achilles heel for RESTV and, most importantly, fundamental differences in the biological processes and pathways induced by the strains.

## 2. Results

Prior to conducting our experiments, we established the appropriate multiplicity of infection (MOI) to use and the best time to harvest the cells. RNA extracted at the two time points (24 h and 96 h) was divided in two parts; one for Reverse Transcription-quantitative Polymerase Chain Reaction (RT-qPCR) and the other for sRNA-seq experiments.

Analysis of the human small RNA library by sRNA-Seq unveiled the existence of an average of 6 million adaptor-trimmed reads on average in the 16 to 30 nt window of RNA sizes. Reads of 21 to 23 nt, corresponding to the average length of miRNAs, were found to be the most abundant, with a notable peak at 22 nt (Figure S1). We observed the same trend independently of the stage of infection. Furthermore, the strains do not seem to alter the patterns of the reads.

Using Novoalign software (Novocraft Technologies), the adaptor-trimmed reads were aligned to the human pre-miRNA in miRbase (http://www.mirbase.org/, accessed on 1 October 2020; Release 22.1). In samples collected at 24 h, an average of 46% of the reads were annotated to known human pre-miRNA sequences. There were 10% more reads (to 56%) at 96 h. Furthermore, from 24 h to 96 h, the rate of coverage increased for both control (uninfected, 24%) and experimental conditions of infection with human pathogenic strains of EBOV (7% for Mayinga; 19% for Makona). In the case of the nonpathogenic RESTV, however, the rate of coverage decreased by about 10% (Table S1).

Unless otherwise stated, EBOV refers to the three strains Mayinga, Makona and RESTV; and ZEBOV includes only the Mayinga and Makona strains.

### 2.1. EBOV Relative Viral Replication

**Figure 1:**
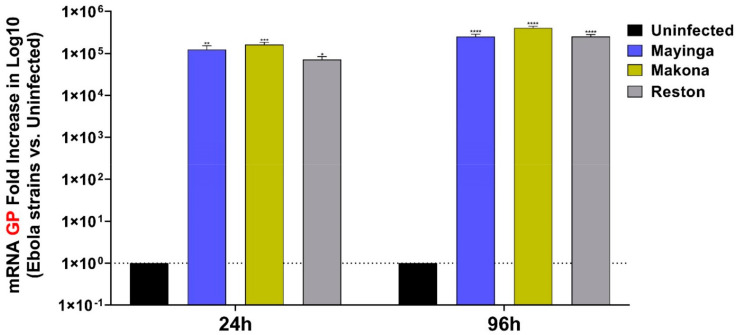
Relative viral replication of EBOV variants. The relative viral replication of Mayinga (blue), Makona (gold), and Reston (gray) post-infection (24 h, 96 h) were approximated by RT-qPCR using the log fold increase of GP mRNA in Huh7-infected cells, versus uninfected cells (black). Data were normalized with a reference gene (ACTB), reported to control uninfected cells and expressed with a relative quantitation method (ddCT). Data presented were calculated from three biological replicate (*n = 3*) measurements. The two-way analysis of variance (ANOVA) and Sidak multiple comparisons were used for statistical analysis. Statistically significant differences (uninfected vs. strains-infected cells) are indicated by stars (*), * *p* < 0.05; ** *p* < 0.01; *** *p* < 0.001, **** *p* < 0.0001.

Using RT-qPCR, we monitored viral infection by quantitating viral GP RNA levels (Figure 1). Several reasons make GP a target of choice often used to assess relative viral replication: it is the only virally expressed protein on the virion surface, the main viral antigenic determinant, a critical actor for attachment to the host cell (through receptor binding) and a promoter of the expression and internalization (catalysis of membrane fusion) of the virus.

The viral loads were similar for all strains at 24 and 96 h in Huh7 cells infected with an equal (1) MOI (Figure 1). Viral replication appeared to be high from the early stage of infection. In linear scale, the viral load of the three strains displayed a fold increase greater than 120,000 and 300,000 on average between 24 h and 96 h.

### 2.2. Makona May Elicit Proinflammatory Reaction Earlier Than Mayinga

The ability of EBOV to induce widespread inflammation and cellular damage has already been reported [30,46]. Here, in a comparative approach between the strains, we tested the mRNA level of certain classes of inflammatory mediators such as the cytokines interleukin (IL) IL-1b, IL-6, IL-8, TNF and adhesion molecules (ICAM-1, VCAM-1).

mRNA monitoring showed that IL-6 was commonly expressed only in the late phase of infection and exclusively for ZEBOV (not for RESTV). Similarly to IL-6, the IL-8 chemokine, which is known for its role in neutrophil recruitment, was expressed specifically in the late stage for all three strains with, nevertheless, important variations between samples.

**Figure 2:**
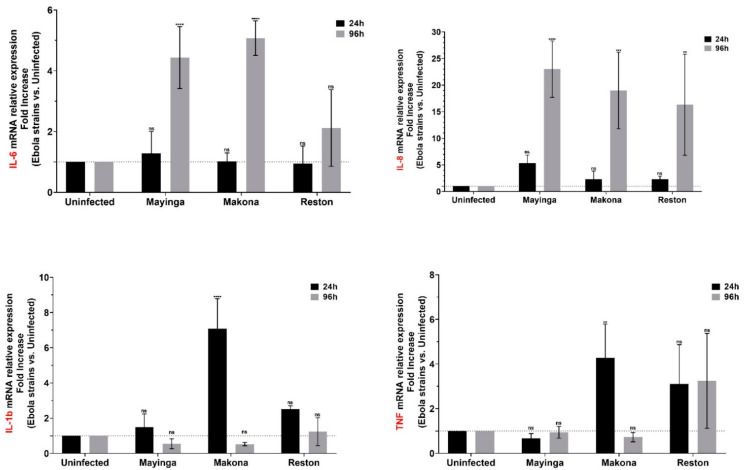
Modulation of cytokines (IL-6, IL-8, IL-1b, TNF) in Huh7 cells infected or not with Ebola virus. The relative mRNA levels of four cytokines (IL-6, IL-8, IL-1b, TNF) were measured for three EBOV variants at 24 h (black) and 96 h (gray) post-infection based on the linear fold increase in Huh7-infected cells using RT-qPCR. Data are normalized with a reference gene (ACTB), reported to control (uninfected), and expressed with a relative quantitation method (ddCT). Data presented were calculated from three biological replicate measurements (*n* = 3). The two-way analysis of variance (ANOVA) and Dunnett’s multiple comparisons were used for statistical analysis. Statistically significant differences (uninfected vs. strains-infected cells) are indicated by stars (*), ** *p* < 0.01; *** *p* < 0.001, **** *p* < 0.0001; non significant differences are indicated by the abbreviation “ns”.

IL-1β and TNF, two of the most important proinflammatory cytokines, were expressed at the early stage of infection and exclusively with the Makona strain of ZEBOV, suggesting that the Makona strain may induce an inflammatory reaction earlier than the other strains, possibly through a better recognition of and binding to the liver cells (Figure 2).

**Figure 3:**
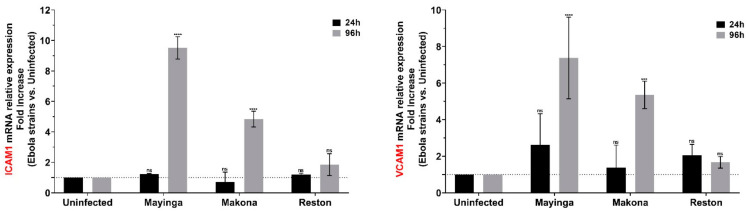
Modulation of IgSF Cell Adhesion Molecules (ICAM1, VCAM1) in Huh7 cells infected or not with Ebola virus. The relative levels of cell adhesion molecules ICAM-1 and VCAM-1 in EBOV-infected Huh7 cells were measured by RT-qPCR at 24 h (black) and 96 h (gray) post-infection using the linear fold increase. Data were normalized with a reference gene (ACTB), reported to control (uninfected), and expressed with a relative quantitation method (ddCT). Data presented were calculated from three biological replicate measurements (*n* = 3). The two-way analysis of variance (ANOVA) and Dunnett’s multiple comparisons were used for statistical analysis. Statistically significant differences (uninfected vs. variants-infected) are indicated by stars (*), *** *p* < 0.001, **** *p* < 0.0001; non significant differences are indicated by the abbreviation “ns”.

Our analyses revealed that the levels of ICAM-1 and VCAM-1 were particularly increased in the presence of the ZEBOV strains but remained unchanged with RESTV (Figure 3). Moreover, ICAM-1 levels with Mayinga were more than two times higher than with Makona.

### 2.3. Mayinga and Makona Haemorrhagic Phenotype May Result from an Imbalance of Coagulation Factors

Next, the hemorrhagic phenotype associated with EBOV prompted us to document its effect on clotting factor gene expression. Although EVD is associated with coagulopathy [47], little is known about the molecular signature of EBOV infection on hepatocytes, which are involved in the synthesis of most blood coagulation factors [48].

**Figure 4:**
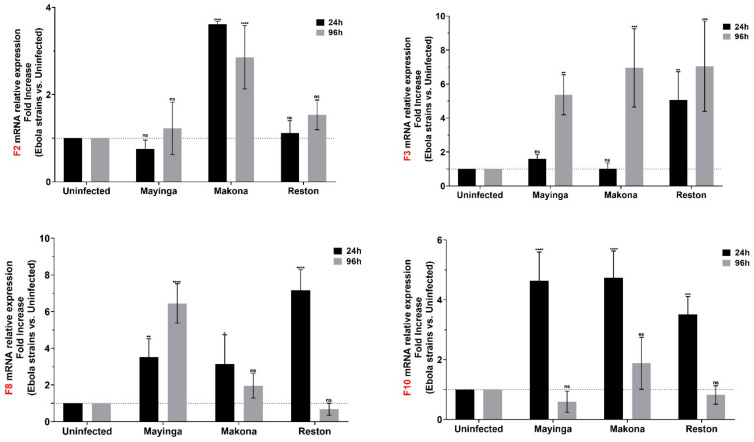
Modulation of coagulation factors (F2, F3, F8, F10) in Huh7 cells infected or not with Ebola virus. The relative levels of coagulation factor 2, factor 3, factor 8 and factor 10 mRNAs in EBOV-infected Huh7 cells were measured by RT-qPCR at 24 h (black) and 96 h (gray) post-infection using the linear fold increase. Data were normalized with a reference gene (ACTB), reported to control (uninfected), and expressed with a relative quantitation method (ddCT). Data presented were calculated from three biological replicate measurements (*n* = 3). The two-way analysis of variance (ANOVA) and Dunnett’s multiple comparisons were used for statistical analysis. Statistically significant differences (uninfected vs. variants-infected) are indicated by stars (*), * *p* < 0.05; ** *p* < 0.01; *** *p* < 0.001, **** *p* < 0.0001; non significant differences are indicated by the abbreviation “ns”.

We thus monitored the relative mRNA level of the factor 2 (F2, FII or prothrombin), F3 (FIII tissue factor), F8 (FVIII) and F10 (FX) factors (Figure 4).

F2 transcript, encoding the prothrombin instructor, was only expressed in presence of the Makona strain. Expressed on the cell surface, the potent initiator F3 was increased in the two ZEBOV strains after 96 h, whereas the levels increased in RESTV after just 24 h of infection.

Additionally, known as antihemophilic factor (AHF) or FIXa cofactor, F8 transcript was found to be upregulated during the course of infection with Mayinga strain, but this increase was transient with the Makona and RESTV strains. In addition, their expression remained two times higher in Huh7 cells when infected with RESTV compared to the ZEBOV strains. Finally, regarding F10, the converter of prothrombin (F2) into thrombin, it presented a transient expression regardless of the strain with comparatively similar levels.

### 2.4. Mayinga and Makona Do Not Seem to Promote Apoptosis in Cultured Hepatocytes

**Figure 5:**
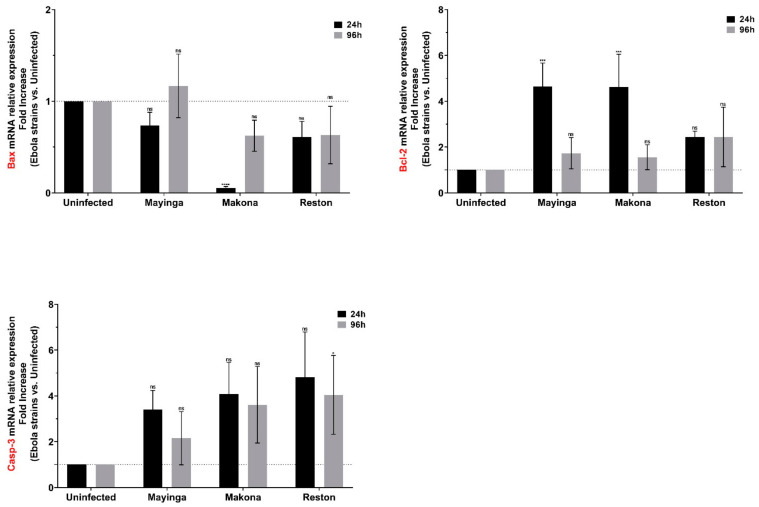
Modulation of apoptosis players (Bax, Bcl-2, Casp3) in Huh7 cells infected or not with Ebola virus. The relative expression of Bax, Bcl-2, and Casp3 mRNAs in EBOV-infected Huh7 cells were measured by RT-qPCR at 24 h (black) and 96 h (gray) post-infection using the linear fold increase. Data were normalized with a reference gene (ACTB), reported to control (uninfected), and expressed with a relative quantitation method (ddCT). Data presented were calculated from three biological replicate measurements (*n* = 3). The two-way analysis of variance (ANOVA) and Dunnett’s multiple comparisons were used for statistical analysis. Statistically significant differences (uninfected vs. variants-infected) are indicated by stars (*), * *p* < 0.05; *** *p* < 0.001; **** *p* < 0.0001; non significant differences are indicated by the abbreviation “ns”.

To further understand the programmed cell death behavior in the hepatocyte cell model Huh7, we measured the mRNA expression of Bax, Bcl-2 and Casp-3 (Figure 5). We assessed whether the actors of apoptosis are modulated in the early and late stages of infection.

The Mayinga strain induced a transient, but (just at the initial stage) significant, expression of the antiapoptosis Bcl-2 gene. This effect was confirmed in Makona, accompanied this time with a significant repression of the proapoptotic gene, Bax. The Casp-3 gene, one of the effectors of apoptosis, was markedly increased only in the late stage of infection with RESTV. These results suggest that the pathogenicity of the ZEBOV strains may not be related to interference with host cell apoptosis.

### 2.5. One Fifth of the miRNome Is Differentially Expressed over Time upon EBOV Infection

The modulation of the actors of apoptosis, coagulation and inflammation serves as a common explanation for the clinical manifestations of EVD. We sought to explore whether the dynamics at the miRNome scale may underlie the observations made above.

We first compared all the identified miRNAs at the early and late phases of infection with the three strains of EBOV (Figure S2). The results show that 82% of early phase miRNAs and 90% of late phase miRNAs were shared among the three strains. Ebola virus infection does not significantly alter the miRNA repertoire.

**Figure 6:**
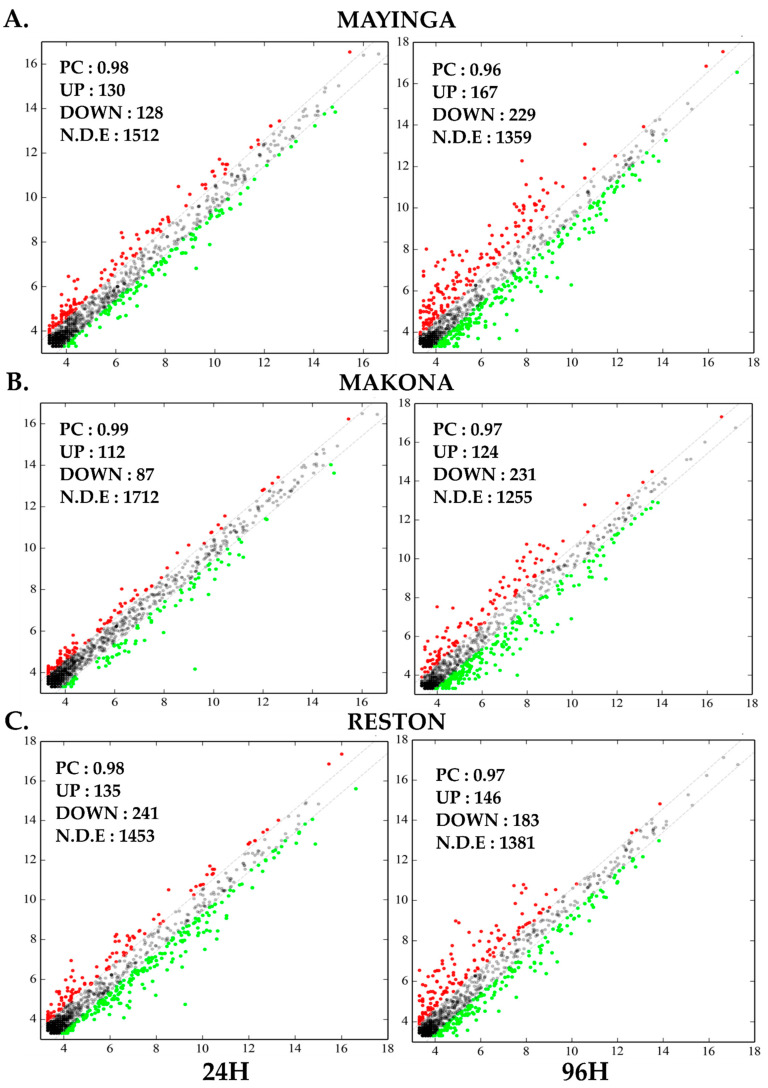
Scatter plot showing Huh7 miRNome modulated by Ebola variants over time. X-axis shows the controls (uninfected) at the indicated time, Y-axis is the experimental condition using the EBOV variant Mayinga (**A**), Makona (**B**) or Reston (**C**). Only a few miRNAs were modulated by the variants. PC: Pearson correlation. UP (red dots): Upregulated miRNAs, DOWN (green dots): Downregulated miRNAs. N.D.E (grey dots): Not differentially expressed.

To obtain an initial panoramic view of the miRNome modulation, we plotted each dataset of DE miRNAs (Supplementary File A) on scatter graph (uninfected vs. infected with EBOV strains, Figure 6). Virus presence effect in DE miRNAs is appreciated by the Pearson correlation (PC).

Between 24 h and 96 h, nearly 1800 (range: 1610–1911) miRNAs were identified in the different experimental conditions. At 24 h, most of the identified miRNAs remained non-DE in the presence of the virus, i.e., 1512 out of 1770 miRNAs with Mayinga (Figure 6A), 1712 out of 1911 miRNAs with Makona (Figure 6B) and the 1453 out of 1828 miRNAs with RESTV (Figure 6C). At 96 h, we observe the same trend; thus, 1359 out of 1755 miRNAs identified with Mayinga (Figure 6A), 1255 out of 1610 miRNAs identified with Makona (Figure 6B), 1381 out of 1710 miRNAs identified with RESTV (Figure 6C) remained unaffected, respectively. On average, some 139 (range: 87–241) miRNAs at the early stage and 180 (range: 124–229) miRNAs at the late stage were differentially expressed (upregulated or downregulated). The PC was overall close to one, indicating a strong commonality in the expression of miRNAs independently of virus infection.

Indeed, regardless of the stage, the EBOV strains did not affect the expression of more than 80% of the cellular miRNAs (nondifferentially expressed) during infection (Figure 6) and seem to modulate in a specific and selective manner only one out of five miRNAs.

### 2.6. The Transient Downregulation of Certain Host miRNAs May Be an Achilles Heel for the RESTV

**Figure 7:**
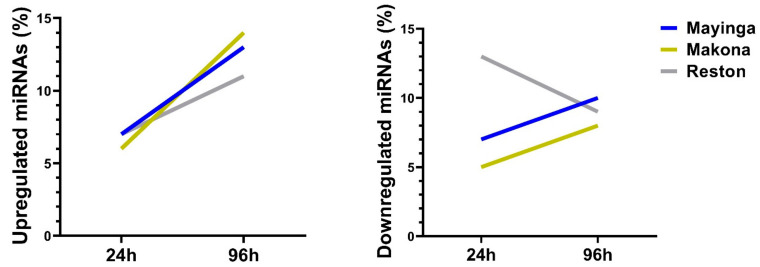
Selective modulation of the DE miRNA expression (%) induced by the EBOV variant over time. Based on the total number of miRNAs expressed in each condition, we estimated the percentage of miRNAs that were upregulated, downregulated or no longer regulated. This graph shows the percentage (%) of upregulated and downregulated miRNAs at 24 h and 96 h, highlighting a reduction in the number of downregulated miRNAs induced by the EBOV Reston variant over time.

We investigated the dynamics of the miRNAs affected by the EBOV strains. For this purpose we computed the upregulated and downregulated proportions of miRNAs with each of the strains from the scatter graph data (Figure 7).

We found that between 24 h and 96 h the number of upregulated miRNAs increased in all EBOV strains: from 130 to 229 with Mayinga (+99 miRNAs), from 112 to 231 with Makona (+119 miRNAs) and from 135 to 183 with RESTV (+48 miRNAs). Notably, RESTV exhibited the lowest modulation among the three strains (Figure 7).

A similar trend was observed for downregulated miRNAs (Mayinga: +39 miRNAs; Makona: +37 miRNAs) except for RESTV (−95 miRNAs) where miRNA number at the initial stage was higher than at the late stage (Figure 7). In other words, over time, the ZEBOV strains maintain and increase (~3%) the number of downregulated miRNAs while the RESTV displayed an opposite situation, losing about 5% of the downregulated miRNAs between 24 h and 96 h. Could the reduced number of miRNAs undergoing downregulation over time contribute to the lower level of pathogenicity of the RESTV strain?

### 2.7. GO and KEGG Analyses of Differentially Expressed MiRNAs

**Figure 8:**
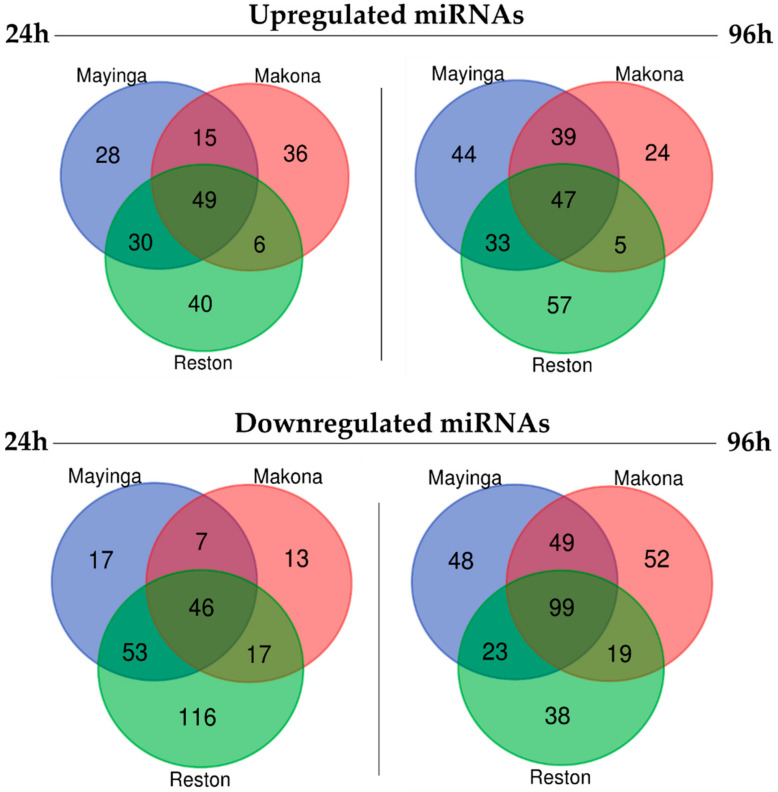
Venn diagrams showing unique and common DE (upregulated and downregulated) miRNAs detected (by RNA-Seq) in Huh7 cells infected with 3 ebolavirus strains (Mayinga, Makona, Reston).

Next, we examined in detail the nature of the DE miRNAs. The relationships between and among all DE miRNAs were illustrated by a Venn diagram (Figure 8, Supplementary File E). Reston shared more similarity with Mayinga than with Makona and concentrated more specific DE miRNAs particularly in the category of downregulated miRNAs. Mayinga and Makona share more DE miRNAs in common in the late phase than in the early stage of infection. The Venn diagram has evidenced that the strains share about 40% of DE miRNAs in common.

The top 20 upregulated (fold change, FC ≥ 1.5) and downregulated (FC ≤ 1/1.5 = 0.67) miRNAs are listed in Table 1, Table 2, Table 3 and Table 4 and reflected the impact of the three EBOV strains on the miRNA profile of Huh7 cells at 24 h and 96 h.

To further deepen our understanding of the cellular consequences of these profiles, the virus operating mode and the host defense mechanism under the control of miRNAs, GO (biological process = BP, molecular functions = MF) [49,50] and KEGG pathways [51] analyses were performed on mRNA targets of all DE miRNAs (see Supplementary File B). These were predicted with Targetscan7.1 (http://www.targetscan.org/vert_71, accessed on 1 October 2020) and mirdbV6 (http://mirdb.org/miRDB/, accessed on 1 October 2020) databases.

#### 2.7.1. Upregulated miRNAs

##### Early Stage

A considerable commonality was observed when comparing the profile of the 20 most abundant miRNAs at the early stage (24 h) of infection (Table 1, Supplementary File A). Fifty per cent of the miRNAs overexpressed with RESTV were also identified with Makona and/or Mayinga. However, each of the strains preserved a distinctive characteristic effect on the host miRNA profile. For example, miR-363-3p, miR-200a-3p, miR-216a-5p were exclusively overexpressed with Mayinga, miR-132-3p, miR-4483, miR-221-5p exclusively overexpressed with Makona and miR-3128, miR-95-3p, miR-582-5p exclusively overexpressed with RESTV. Both miR-19a-3p and miR-20a-5p were preferentially overexpressed with the ZEBOV strains and were absent from RESTV’s top 20 profile. Among the 20 most abundant miRNAs, there were also novel unclassified miRNAs, 2 with Mayinga, 4 with Makona and none with RESTV (Table 1).

The GO analysis of the miRNA targets (complete data, Supplementary File C) revealed that very similar biological processes were affected at the early stage of infection in the three strains of EBOV. The top 10-fold enrichment value of the significant enrichment terms showed similar patterns between all viruses, as shown in Figure S3. The molecular function annotations revealed a significant representation of the “protein binding” category in the ZEBOV strains, whereas it is completely absent with RESTV (Figure S4). DNA binding transcription factor activity and RNA polymerase regulatory sequence-specific DNA binding were the molecular function categories that remained predominant.

Interestingly, analysis using the KEGG database identified different pathways (complete data, Supplementary File D). In fact, in the 10 most significant signaling pathways (enrichment score) for each condition at the early stage, we specifically identified P13K-Akt and Erbb (also named Epidermal Growth Factor Receptor, EGFR) signaling pathways associated with Mayinga, TGF-β and Wnt signaling pathways associated with Makona and p53, T-Cell receptor and RNA degradation signaling pathways associated with RESTV. The transcription factor FOXO was significantly enriched with ZEBOV strains. RESTV shared with Mayinga and Makona the mechanistic target of the Rapamycin (mTOR) and MAPK pathways, respectively (Figure S5, left panel, 24 h).

Functional analysis of these miRNAs revealed that they regulate important cellular signaling pathways (PI3K-Akt, HIF-1, Ras, Rap1, ErbB, and MAPK signaling pathways), that are involved in HCC carcinogenesis [52,53,54].

##### Later Stage

Except for miR-26a-2-3p in RESTV and miR-novel-chr20_29712 in Makona, surprisingly none of the most abundant miRNAs from the early phase remained as abundant in the late phase of infection. New profiles emerged with a relative diversity between them. Nevertheless, RESTV shared with Mayinga more than 40% of the most expressed miRNAs and only 20% with Makona. Mayinga and Makona, belonging to the same family, shared 40% of the most abundant miRNAs, compared to only 10% in the early phase. The late phase is marked by the over-representation of novel miRNAs, including 9 with Mayinga, 13 with Makona and 12 with RESTV (Table 2, Supplementary File A).

In contrast to the observation reported in the early stage, the late stage showed a greater diversity between the three strains in displayed biological processes (complete data, Supplementary File C). With Mayinga, the most enriched terms among others were “positive regulation nucleobase-containing compound metabolic process”, “cell cycle” and “transcription by RNA polymerase II”, while with Makona, we observed “stress-activated protein kinase cascade”, “regulation of apoptotic process” and “chronic inflammatory response”. Lastly, “response to tumor necrosis factor”, “positive regulation of apoptotic process” and “regulation of B-cell activation” were among the most enriched terms with RESTV (Figure S6).

The molecular function annotations exhibited a diversity of terms between the three strains. “Transcription factor activity”, “protein dimerization activity” and “endonuclease activity” were associated with Mayinga, and “DNA binding transcription, peptide antigen binding” and “transcription regulator activity” with Makona. Specifically, with RESTV, we found terms related to the master mediator of inflammatory responses and innate immunity, including “death receptor activity”, “tumor necrosis factor-activated receptor activity” or “MAP kinase activity” (Figure S7).

KEGG pathway analysis (Figure S5, right panel, 96 h, complete data, Supplementary File D) pointed to a parallel with the molecular function annotation for the RESTV condition by showing the recurrence of terms such as “natural killer cell mediated cytotoxicity”, “EGFR signaling pathway” and “T-cell receptor signaling pathway”. The redundancy of KEGG pathway was observed in the ZEBOV strains (e.g., phagosome, phosphatidylinositol signaling system, inositol phosphate metabolism and amino acids degradation). However, we noted the specific occurrence of Tumor Necrosis Factor (TNF) pathway only with Makona.

#### 2.7.2. Downregulated miRNA

##### Early Stage

We also investigated the significantly downregulated miRNAs (Top 20) in the context of EBOV infection. At the early stage of infection (24 h), there was a remarkable similarity of the profiles irrespective of the virus. We found 60% of the downregulated miRNAs of the RESTV condition among Mayinga and/or Makona. The latter two also have in common 60% of the miRNAs. Despite the similarities, there were specific miRNAs for each condition, such as miR-491-5p in Mayinga, miR-199a-5p in Makona or miR-940 in RESTV. Among the 20 lowest expressed miRNAs, approximately 10 to 15 are novel miRNA candidates (9 with Mayinga, 10 with Makona and 15 with RESTV) (Table 3, Supplementary File A).

The ontology of the target genes of miRNAs downregulated in the context of EBOV infection, are listed in Figure S8 (complete data, Supplementary File C). The annotation of molecular function, irrespective of the EBOV strains, showed “binding” term as one of the most redundant (Figure S9).

Consistent with the molecular functions data, the primary biological pathways cataloged on KEGG (complete data, Supplementary File D) also unveiled the connection of the host defense pathways to the downregulated miRNAs during the initial stage of ZEBOV strains infection (Mayinga, PI3k-Akt signaling pathway, cAMP signaling pathway; Makona, T-cell receptor signaling pathway, proteasome, Fc gamma R-mediated phagocytosis) (Figure S10, left panel, 24 h). “Adherens junction”, “glyoxylate and dicarboxylate metabolism” and “glycosaminoglycan biosynthesis” were, among others, over-represented terms in the RESTV strain.

##### Later Stage

Similarly, in the late phase, numerous miRNAs were downregulated regardless of the virus. Of the 20 most affected miRNAs in each of the experimental conditions, at least 25% were common to all three strains. The human pathogenic strains Mayinga and Makona generated different profiles, having in common only 2 miRNAs out of the 20 most severely repressed. The nonpathogenic strain RESTV caused a drastic decrease in miRNAs, half of which were also under-expressed in the pathogenic strains Mayinga and/or Makona. As observed in the late phase of the infection, for miRNAs which were upregulated, there was also an overabundance of novel miRNA candidates among the 20 most affected miRNA candidates: 50% with Mayinga, 35% with Makona and 15% with RESTV (Table 4, Supplementary File A).

GO functional analysis (complete data, Supplementary File C) of the genes targeted by these downregulated miRNAs revealed that the most significant biological processes that emerged with ZEBOV strains were “signaling” and “biological regulation” terms. In contrast, “proteasomal protein catabolic process”, “cell surface receptor signaling pathway” and “Positive regulation of organelle organization” were observed with RESTV (Figure S11).

As for molecular functions, they were essentially related to “protein binding” (Figure S12). The top hits among the KEGG pathways (Figure S10, right panel, 96 h, (complete data, Supplementary File D) featured pathways including “Rap1 signaling pathway” (Mayinga), “Ras signaling pathway” (Makona) and “Wnt-signaling pathway” (RESTV).

### 2.8. miR-122-5p, miR-148a-3p, miR-21-5p Are Selectively Modulated by EBOV

**Figure 9:**
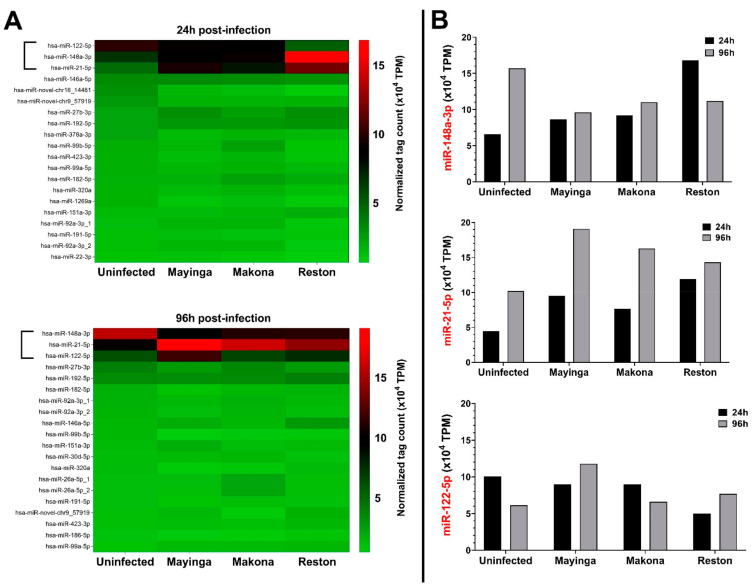
Modulation of the most abundant isoform miRNAs in Huh7 cells infected or not with Ebola virus. (**A**) Heatmap of the 20 most abundant Huh7 miRNAs expression profiling tracked during EBOV infections with the Mayinga, Makona or Reston variant. TPM, transcripts per million. (**B**) Variation over time of the 3 most abundant miRNAs in Huh7 cells in the presence of the EBOV Mayinga, Makona or Reston variants. The figure was generated from the data of (**A**): **[** . TPM, transcripts per million.

Lastly, to further characterize the selective and restrained modulation of the miRNome observed above, we examined the influence of EBOV strains on the basal expression of the most highly expressed miRNAs in Huh7 cells grown under normal conditions (Figure 9).

Remarkably, in the absence of EBOV infection, we noted slight variations in terms of miRNAs ranking, but collectively all the abundant miRNAs (top 20) at 24 h maintained their levels after 96 h. These 20 first miRNAs represent more than 57% of total miRNAs (Figure 9A). Notably, three miRNAs were displaced off the top 20 list, but remained among the top 30. More specifically, among the 20 most abundant miRNAs at 24 h and 96 h, only miR-122-5p, miR-148a-3p and miR-21-5p appeared to be markedly modulated by the virus (Figure 9B). Together, these three miRNAs represent more than 22% of total miRNAs.

*hsa-miR-122-5p.* At the early stage (24 h), the ZEBOV strains (Mayinga, Makona) did not affect the level of miR-122-5p, while in the RESTV strain, it was reduced by half. At a later stage (96 h), with no virus present, miR-122-5p decreased by about 1.6 times, but the expression level reached twice as high with Mayinga. However, neither Makona nor RESTV showed remarkable changes.

*hsa-miR-148a-3p.* At the initial stage, miR-148a-3p displayed to a certain extent similar expression level among the ZEBOV strains but was about three times higher in the RESTV strain.

At the late stage, miR-148a-3p increased by 2.4 times without infection before dropping to 1.5 times lower in the ZEBOV and RESTV strains.

*hsa-miR-21-5p.* At the initial stage, the level of miR-21-5p increased by ~2-fold in the ZEBOV strains and by 3-fold in the RESTV strain. At the late stage, miR-21-5p expression increased by 2-fold in the Mayinga strain and ~1.5-fold in the Makona and RESTV.

Each strain typically affected the expression of miR-122-5p, miR-148a-3p and miR-21-5p. The two ZEBOV strains seem to induce the same trend of miRNA modulation, which is opposite to that triggered by the RESTV strain. Taken together, these results underline the selective and specific nature of miRNA modulation by the EBOV strains.

### 2.9. Filovirus-Targeting MiRNA

Several studies have reported that, once released in cells, viral genomes may be the target of host miRNAs [22,55,56,57]. As hsa-miR-122-5p, hsa-miR-148a-3p and hsa-miR-21-5p were markedly modulated (Figure 9) during EBOV infection (early and late stages), we investigated whether they could interact directly in the viral genome and/or compete with the most upregulated miRNAs by EBOV (Table 1 and Table 2). For this purpose, we used bioinformatics analyses to find putative binding sites for host miRNAs in EBOV genome. We submitted the reference genome of ZEBOV and RESTV strains for analysis with a total of 21 miRNAs, i.e., these three most abundant uninfected-Huh7 miRNAs and the six most upregulated miRNAs over time in the presence of each EBOV strain (3 miRNAs for 24 h and 3 miRNAs for 96 h/strain).

The output showed that hsa-miR-122-5p may target the viral genome of ZEBOV and RESTV; miR-148a-3p did not seem to interact with none of the EBOV and miR-21-5p were specific to ZEBOV.

At the early stage of infection, the Makona-associated hsa-miR-454 (FC: 3.36) was the only one found to potentially interact with the viral genome of the corresponding EBOV strain (Mayinga and Makona). The other miRNAs did not present potential binding sites on the reference genomes or the *p*-value associated with heteroduplex formation exceeded 0.05 by far. At the late stage, hsa-miR-27a (FC: 22.35), hsa-miR-443 (FC: 21.58), hsa-miR-195-3p (FC: 11.18) associated with Mayinga (Table 2) and hsa-miR-193-5p (FC: 14.33) associated with RESTV (Table 2) also showed potential interactions with their respective viral genome.

**Figure 10:**
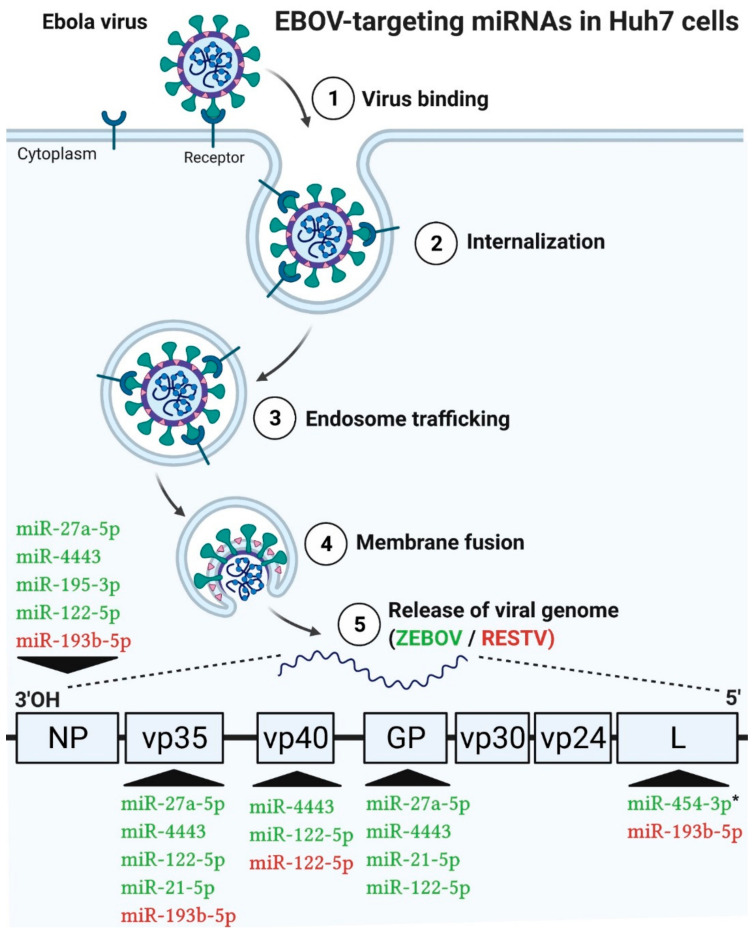
Model of EBOV cell entry and of the host miRNAs targeting the EBOV RNA genome. The steps leading to the cytosolic release of the viral genome into Huh7 cells are presented. The 3 most abundant uninfected Huh7 cell miRNAs and the 6 most EBOV-upregulated miRNAs (3 miRNAs for 24 h and 3 miRNAs for 96 h for each variant) over time are predicted to target various genes of the viral genome (VP35, VP40, GP, L) in the ZEBOV (Mayinga/Makona, green) variant and the Reston (RESTV, red) variant. The predictions were performed with RNA22. Only significant (≤0.05) hits were retained. * = miRNA expressed in the early phase, (the others being expressed in the late phase).

**Figure 11:**
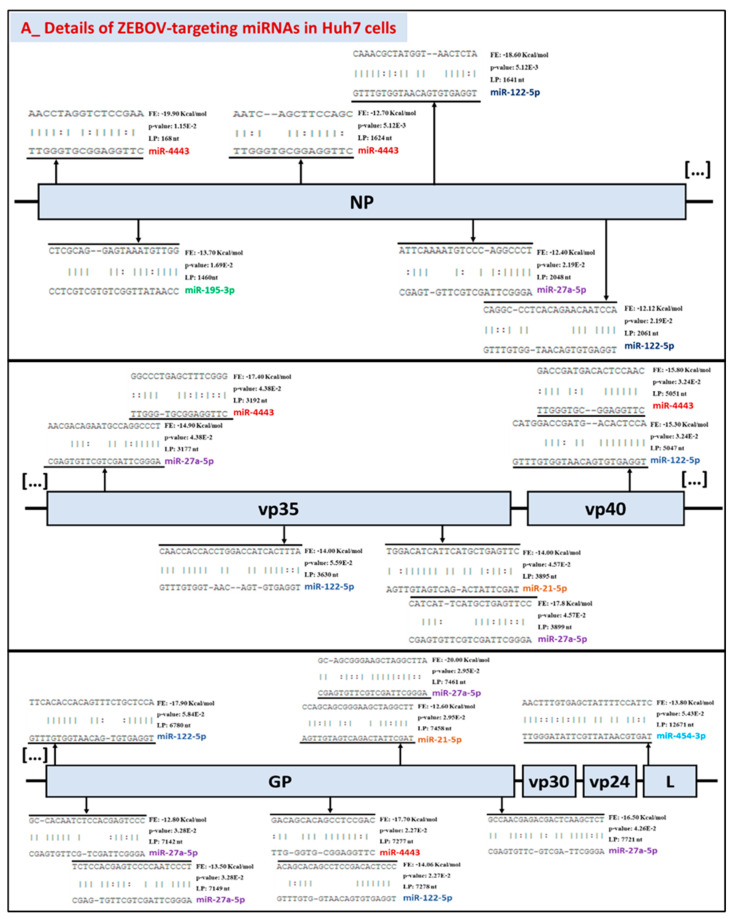

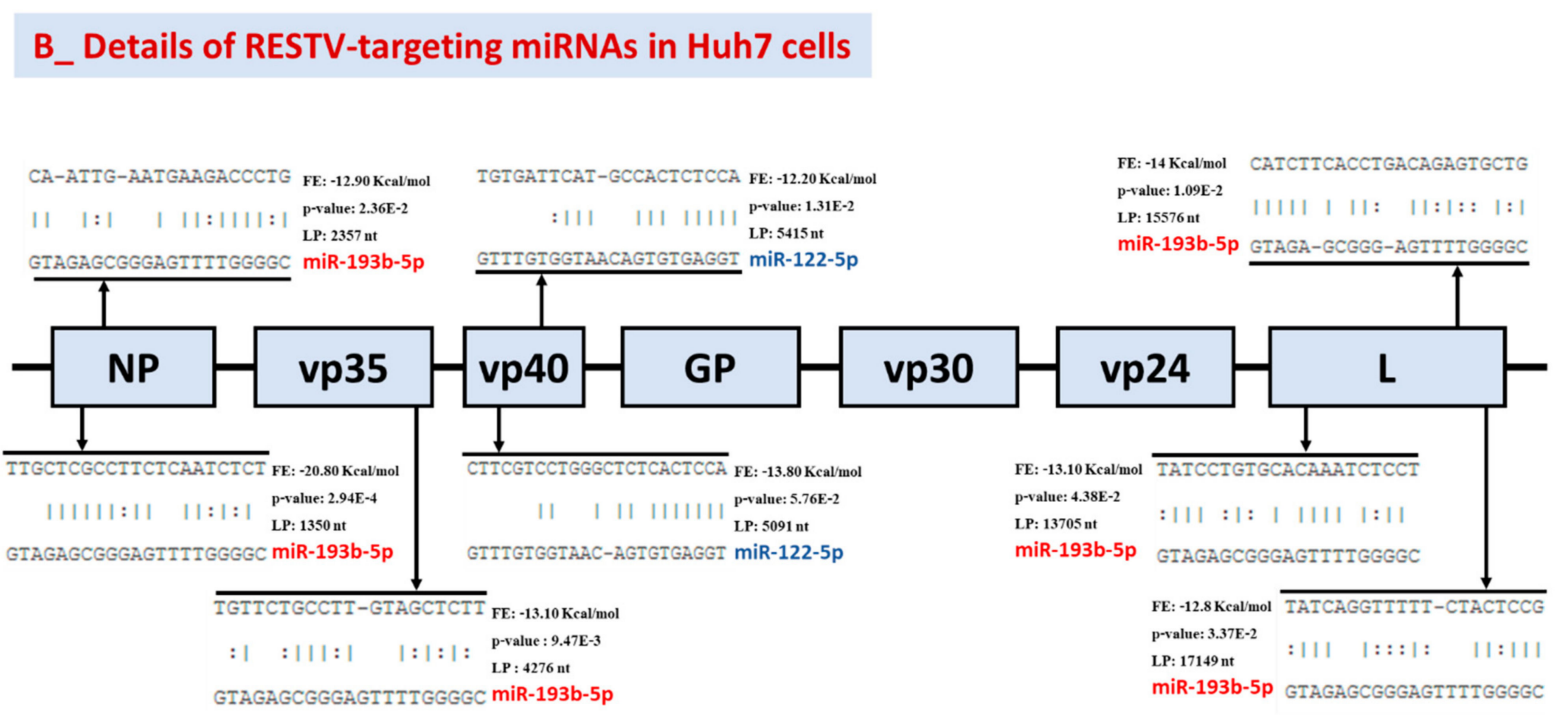
Alignment of the top upregulated host miRNAs on the EBOV RNA genome for both ZEBOV and RESTV Scheme 7. Cells before and after infection with (**A**) ZEBOV (Mayinga, Makona) and (**B**) RESTV (Reston) were aligned with their respective genome using RNA22. Alignments with the lowest free energy (FE) in kcal/mol and the highest probability (*p* ≤ 0.05) were reported along with the leftmost position (LP) in nt of the alignment on the EBOV RNA genome.

Detailed analysis of ZEBOV/RESTV-miRNAs interactions highlighted specificity and selectivity of host miRNAs (Figure 10). In fact, host miRNAs were found to target ZEBOV in its GP (7 hits), NP (6 hits), vp35 (5 hits), VP40 (2 hits) and L (1 hit) genes. For RESTV, miR-193b may bind to several genes, such as L (3 hits), NP (2 hits), vp35 (1 hit), while miR-122 exhibited a preference for vp40 (2 hits). The vp30 and vp24 of all the strains, as well as GP of the RESTV strain, did not display any binding site for the chosen miRNAs (Figure 10 and Figure 11). Therefore, certain miRNAs may target several viral genes at the same time (miR-27a-5p, miR-4443, miR-21-5p, miR-193b-5p, miR-122-5p), while others may target only one (miR-454-3p, miR-195-3p).

In addition, possible competition may also emerge between miRNAs naturally abundant in Huh7 (miR-122-5p, miR-148a-3p, miR-21-5p) and those upregulated during infection (Table 1 and Table 2, top 3 miRNAs) with either of the strains (Figure 11). On the NP viral gene, the binding sites of miR-122 (leftmost position: nt 1641 and nt 2061) are located nearby those of miR-4443 (nt 1624) and miR-27a-5p (nt 2048). Similarly, we observed a very high proximity of the miR-122 (nt 5047) and miR-4443 (nt 5051) binding sites on the vp35 gene. On the same gene, miR-27a-5p (nt 3177 and nt 3899) may also compete with miR-4443 (nt 3192) and miR-21-5p (nt 3895). Lastly, on the GP gene, miR-122 (nt 7278) and miR-21-5p (nt 7458) were predicted to bind sites which could also be targeted by miR-4443 (nt 7277) and miR-27a-5p (nt 7461).

During EBOV infection, miRNAs that were the most severely repressed were also subjected to an in-depth analysis of their possible interaction with the viral genomes (summarized in Figure S13). Overall, the in silico predictions suggested that EBOV, once released, may be targeted by host miRNAs, especially those that are naturally abundant in Huh7 and those that, through mechanisms to be elucidated, become strongly expressed or repressed during infection.

## 3. Discussion

As yet, little is known about the miRNA transcriptome in filovirus infection, especially in the liver, which is an important target of filovirus infection [42]. We report here the first high throughput sequencing analysis of the impact of viral infection on Huh7 cells miRNome. The sRNA-Seq samples were pooled from biological triplicates for each condition, nevertheless the ranks and abundance of Huh7 miRNAs reported were consistent with the findings of similar studies [58,59]. The pooled-design study presents a statistical weakness and affects the individual expression of the genes but also reduces biological variation between replicates and offers many practical advantages [60,61,62]. These limitations also apply to the GO and KEGG data designed through sRNA-Seq outputs.

We generated an average depth of 9 (range: 6.87–9.39) million reads (Figure S1 and Table S1) which is believed to be sufficient to identify moderate-to-low-abundance miRNAs and modest expression differences between samples [63]. Besides providing highly accurate quantification, the sRNA-Seq’s unbiased nature allows detection of novel small RNAs [64]. However, approximately 3 million reads were removed from the data due to the target biotype (in this case miRNAs). Considering that “size” does not define the biological function of an RNA sequence [65,66], this lower limit (<16 nt = exclude) can be arbitrary if we are interested in the discovery of new (very) small functional RNAs.

Based on the total number of reads aligned to known human pre-miRNAs in miRBase21, we found an average of 970,000 transcripts per million (TPM) representing the normalized tag number of all annotated miRNA isoforms, which include 7% of predicted novel miRNAs whose functions remain to be determined.

NovoAlign, used in this study, showed higher sensitivity towards both short and long read mapping even for complex genomes [67]. However, still, the variation in percentage of reads aligned to known pre-miRNAs may result in inherent mapping bias of the most commonly used short read mapping software [68] regardless of the parsimonious and suitable selection of the aligners. Putting aside any inevitable technical variability introduced in the library preparation [69], the lowest depth of coverage observed in the RESTV condition (Table S1) between the samples collected at early and late stages remains puzzling. These observations could be the result of a specific modulation of the genome, which may reduce exposure to miRNAs and emphasize, speculatively, the primary impotence to modulate the host genome.

Differences in the host response to infection with the three strains could not be awarded to the replication kinetics as these were quite similar for each of the measurement points (Figure 1). The comparative evaluation of the expression levels of coagulation, inflammation and apoptosis players in the Huh7 cells confirmed the specific molecular signatures of the viral strains and highlighted new ones.

In our study, two (IL-1b, TNF) of the four proinflammatory cytokines tested were increased transiently, not lasting over time, while the other two (IL-6, IL-8) were delayed and detected on the fourth day (Figure 2). The “cytokine storm” described in inflammatory cells [70] does not seem to take place in liver cells or seems to be quickly “quenched” by viral mechanisms. Furthermore, the features do not seem to be sufficient to confer avirulent properties to Reston.

Even though the liver is an organ taking part in the systemic defense mechanisms initiated by the host after infection [71,72], the study of cytokines remains illustrative in an HCC cell model. Inflammatory data analysis may be more suitable in cell models associated with the immune system, including B cells, T cells, macrophages, mast cells, neutrophils, basophils and eosinophils [73]. It appears then more relevant to integrate these datasets with those obtained in the measurement of the other parameters.

Indeed, there seems to be a positive feedback loop between cytokines and coagulation factors, whereby blood coagulation factors might induce inflammation [74] and inflammatory mediators (such as IL-6) may effectively upregulate coagulation factors (such as F3 expression in monocytes) [75,76]. ZEBOV-infected cells display a positive correlation between IL-6, IL-8 and F3, which is the initiator of blood coagulation cascades [77]. The early activation of F3 in Reston samples may enable early adjustment mechanisms in human cells and contribute to its lack of pathogenicity. In ZEBOV, the F3 transcript increased on day four, which is consistent with the hemorrhagic complications often observed in EVD and also shown previously by Geisbert et al. [47] in nonhuman primate peripheral blood mononuclear cells (PBMC).

Safety concerns and access to patient samples complicate the characterization of the basic coagulation changes [78]. Our study brings new insights at the transcriptional level of four essential coagulation factors (F2, F3, F8, F10—Figure 4) that need to be further investigated for a better understanding of hemorrhagic fever diseases.

The absence of significant expression of cell-adhesion molecules (Figure 3) that belong to the immunoglobulinlike superfamily of proteins (IgSF CAMs) with Reston can be added to the table of factors explaining the nonpathogenicity of this strain. Indeed, the IgSF shape the viral tropism [79] and therefore might enhances EBOV infection. Moreover, the IgSF TIM-1 (T-cell immunoglobulin and mucin domain 1) was recently well characterized as a receptor for Ebolavirus [80].

Our results indicate that the Mayinga and Makona strains seem to have evolved to adopt mechanisms that allow them to efficiently infect cells without triggering apoptosis, allowing their replication. The most recent strain, Makona, looks more efficient in this respect (Figure 5). Moreover, 5 of the 10 most enriched GO terms in Makona are related to the regulation of programmed cell death. In contrast, the GO term “positive regulation of apoptosis” was among the most enriched terms for RESTV (Figure S6) and Caspase-3, an early marker of apoptosis, was only detected in RESTV (Figure 5). These data provide additional support for the nonpathogenicity of the strain, which may be related to its inability to prevent cell death and allow sufficient time to replicate effectively.

Our data do not argue in favor of apoptosis in view of the profiles obtained (Figure 5); however, other groups, such as Bradfute et al., [81] reported the apoptotic cell death of hepatocytes in EBOV-infected mice. The model used (cultured cells vs. tissue) and the method of assessing apoptosis were different. Their examinations were performed at day seven of livers from EBOV-infected mice via conventional brightfield microscopy, TUNEL staining and electron microscopic analysis. It is also necessary to point out that our qPCR data capture the level of mRNA expression, but not that of the protein effectors, which would need to be further confirmed and validated over longer durations (>96 h).

The documented harmful inflammatory responses and direct tissue damage caused by EVD [82] suggest potential major dysregulation of the miRNome. Interestingly, our data showed the involvement of a very small number of miRNAs (Figure 6). However, a limitation of our study is that it was performed in cell culture, and further investigation in animal models and human clinical samples is required. Notably, only a few cellular miRNAs (20%) were differentially expressed by EBOV in Huh7 cells, suggesting a limited and selective, rather than a global, modulation of the host miRNAs, resulting from EBOV interaction with the host cells, contributing to its pathogenesis. For the RESTV strain, more than 95 miRNAs escaped downregulation (24–96 h) (Figure 7), inferring that the strain failed to silence miRNAs clusters. Since the changes in the pool of miRNAs were dynamic over time, these 95 miRNAs may be of variable composition and potentially include antiviral miRNAs, which may explain why RESTV lacks virulence. The identification of 116 RESTV-specific downregulated miRNAs (versus 17 and 13 with Mayinga and Makona, respectively) in the early phase by the Venn diagram (Figure 8) strengthens this possibility. These “miRNomic” features of RESTV could explain the slower replication and cytopathic effect of RESTV in some experimental models [83] (REF) and introduce a new dimension of distinction between RESTV and ZEBOV. Future studies in nonhuman primate cell lines will provide further insight.

Our study indicates that, among the 20 most abundant miRNAs found in HCC Huh7 (uninfected) cells, only miR-122-5p, miR-148a-3p and miR-21-5p are deregulated by the EBOV strains (Figure 9). These are to be distinguished from the miRNAs that are found upregulated because of the infection (Table 1 and Table 2). The limited and specific modulation of miRNAs in hepatocytes (Figure 6) provides an easy identification of the miRNomic signature of EBOV and gives a promising prospect for their use as biomarkers.

The liver-specific miR-122-5p is well known to facilitate replication of Hepatitis C Virus (HCV) viral RNA by interacting with the 5′ untranslated region [84], by hiding the viral genome from exoribonuclease Xrn2 [85] or by altering the balance of viral RNAs engaged in replication versus translation [86]. miR-122-5p may play a similar role and favor EBOV replication, given that its expression levels (Figure 9B) were reduced by half (vs. uninfected, 24 h) with the nonpathogenic RESTV and doubled with Mayinga (vs. uninfected, 96 h).

miR-148a was repressed in the presence of the ZEBOV strains and transiently increased with the RESTV strain (Figure 9B). miR-148a has been shown to suppress the proliferation of HCC cells infected with HCV by targeting *c-Jun* mRNA [87]. Therefore, the transient increase observed with RESTV could activate MAPK signaling pathways and thus allow the early establishment of defense mechanisms that would limit the proliferation of this strain and reduce its pathogenicity.

In contrast to miR-148a, miR-21-5p was more increased with ZEBOV strains (notably with Mayinga) than with the RESTV strain (Figure 9B). Its crucial role in promoting the HCV replication [88], however, also suggests that miR-21-5p may be a pro-EBOV regulator and could probably enhance some stages of the virus life cycle. In fact, miR-21-5p inhibition decreased HCV replication and release of infectious virions by Huh-7 cells [88].

EBOV and HCV differ in size [6,89], structure [90,91,92], and transcription/translation mode [6,93], but are both single-stranded RNA viruses [6,94] with a fairly similar mode of intrusion [8,9,95]. The comparison with HCV takes root from the fact that this virus is widely studied in the cellular model used in our research. Assuming that both viruses may interact similarly with host miRNAs, we posit that the gene regulatory effects of miR-122-5p, miR-148a-3p and miR-21-5p demonstrated in HCV may be transposed to Ebola virus replication and serve as valuable clues to better understand viral pathogenesis, especially in the absence of direct experimental validation. The therapeutic silencing of miR-122 in primates with chronic HCV infection [96] could inspire a “triple therapy” against EBOV based on the modulation of these three miRNAs.

Regarding miRNAs upregulated following EBOV infection (Table 1 and Table 2), it is interesting to note the existence of strain-specific miRNAs that can be detected during the early stage of infection and that may serve as precocious biomarkers, and potentially contribute either positively or negatively to the virulence and pathogenicity of the EBOV strains. For example, Mayinga seemed to correlate in a specific manner with miR-363-3p (belonging to miR-106-363 cluster), a regulator of apoptosis and proliferation in various cell lines, including those derived from the liver [97,98,99]. The Makona strain was associated with miR-132-3p which is known to promote H1N1 Influenza A Virus replication by suppressing type I interferon response through targeting Interferon Regulatory Factor 1 (IRF1) [100]. Makona was also associated with the upregulation of miR-221, which is known for its antiviral activity through the NF-κB pathway [101]. RESTV was associated with upregulation of miR-95-3p, which is known to promote tumorigenesis in HCC by targeting the universal inhibitor of cyclin-dependent kinases p21 [102]. In parallel, this nonpathogenic strain was also correlated with miR-582-5p, which is involved in host antiviral responses in influenza virus infection [103]. These findings suggest that DE miRNAs are most likely the active result of host–pathogen interactions.

MiRNAs may play distinctive regulatory roles in the immune system [20], either antiviral [104,105,106,107] or proviral [84,108], which may involve direct interactions with the viral genome, as suggested by the presence of the predicted miRNA binding sites in the EBOV genome featured in our results (Figure 10 and Figure 11). Relative viral replication data indicated an early and rapid replication of the three EBOV strains in Huh7 cells (Figure 1), in which miRNAs were effectively upregulated at the early stage, but the other abundant miRNAs appeared to interact with the viral genome at the late stage (Figure 10 and Figure 11, Table 1 and Table 2), except for miR-454-3p, associated with Makona. Indeed, higher miR-454-3p expression is characteristic of a favorable prognosis in HCC [109] and may also be involved in the delayed disease progression seen in Makona infection [31,110]. Among other factors, the transient expression of miR-454-3p (Table 1 vs. Table 2) combined with the transient expression of TNF (Figure 2) that it can modulate [111] could explain why we observe decreased rather than increased virulence of this most recent EBOV Makona strain.

Since bioinformatics analyses based on RNA22 involve miRNA sequences and not 3′UTR sequences [112], the method offers more flexibility; however, this comes at the cost of more false positives. Not all interactions mediated by miRNAs are canonical, but the Watson and Crick base pairings observed (Figure 11) do not all systematically meet the “classic” topology of miRNA binding. Therefore, despite the rigorous selection of miRNAs that can bind to viral genomes, additional studies are fundamentally necessary to elucidate these in silico predictions.

The KEGG data provides an additional perspective about the distinction between the strains. For example, they indicate that, compared to Mayinga, Makona seems to be less successful in its escape strategy. In fact, the Makona strain is correlated with the activation of the signaling pathways related to immune system. This is also the case for RESTV, with more important players, such as the p53 [113], mTOR [114], MAPK [115] signaling pathways (Figure S5). Finally, it is noteworthy that the RESTV does not repress key actors of the immune defense in the KEGG data related to downregulated miRNAs (Figure S10).

The construction of miRNA-centric multiplex networks using miRNet [116] illustrates that host miRNAs are, during Ebolavirus infection, at the crossroads of many essential pathways (cell cycle, apoptosis, transcription, immune defense) and therefore are pivotal modulators requiring attention to understand EVD. The network analysis of upregulated (Figure S14) and downregulated (Figure S15) miRNAs confirms the GO/KEGG data and unveil miRNA hubs (Supplementary File F). The possible interactions with the viral genomes of these miRNAs introduce an additional level of complexity to the host–pathogen relationship in the EVD context. Combined with possible interactions with viral genomes, these miRNAs provide an additional layer of complexity to the host–pathogen relationship in the EVD context but also new perspectives.

## 4. Conclusions

Our study represents the first, comprehensive differential analysis of miRNA expression in HCC cells infected with EBOV. We have documented the miRNA profile of three strains including Mayinga from the first outbreak, Makona from the second most important one and RESTV, which is nonpathogenic to humans. For each of them, we unveiled a particular early and late molecular signature, which may be investigated more thoroughly by experts in the field.

Since miRNAs may serve as useful biomarkers for prediction of vaccine-induced immunogenicity [117] and may be thought of as the sculptors of the transcriptome [18] and important modulators of immune defense [20], we deem it especially important to integrate them in the analysis of clinical and pathological observations of diseases [118].

Considering the current COVID-19 pandemic [119], with ecosystems unbalanced by global warming in a globalized world [120], and in the light of the two recent EBOV epidemic outbreaks, a pandemic of EVD would not be a “black swan”. In consequence, fundamental research on these viruses deserves more than ever to be pursued and planned for.

## 5. Materials and Methods

### 5.1. Cell Culture

The hepatocyte-derived cellular carcinoma cell line Huh7 was grown in Dulbecco’s modified Eagle’s medium (DMEM), supplemented with 10% fetal bovine serum, 1 mM L-glutamine, 100 units/mL penicillin, and 100 µg/mL streptomycin. Cells were grown and maintained in tissue culture plates and incubated at 37 °C in a humidified atmosphere containing 5% CO_2_. Cells were kept in the exponential growth phase and subcultured every 2–3 days. For transfection, 300,000 cells were transferred to 6-well plates and transfected the following day at 70–80% confluency using polyethylenimine (PEI; MilliporeSigma, Oakville, ON, Canada), as described previously with minor modifications [121].

### 5.2. Viruses

Experiments involving the manipulation of EBOV were conducted with all precautions in the Biosafety Level 4 laboratory facility at the National Microbiology Laboratory of Canada (Winnipeg, Manitoba). This study utilized the following three EBOV isolates: EBOV/May (Ebola virus/H.sapiens-tc/COD/1976/Yambuku-Mayinga; NC_002549.1), EBOV/Mak-C07 (Ebola virus/H. sapiens-tc/GIN/2014/Makona-WPGC07; KJ660347.2) and RESTV/XX (Reston virus/M.fascicularis-tc/USA/1989/Philippines89-Pennsylvania; NC_004161). Huh7 cells were infected separately with the three strains at a multiplicity of infection (MOI) of 1.0. Cells were harvested at early (24 h) and late (96 h) stage of infection in biological triplicate (*n* = 3). Viral replication was assessed by RT-qPCR detection of GP mRNA (See Table S1).

### 5.3. RNA Isolation

Total RNA was extracted from Huh7 cells (24 h and 96 h) using TRIzol reagent (Invitrogen, Cat No.: 15596026, Burlington, ON, Canada) following the manufacturer’s recommendations. All RNA samples were subjected to treatment with DNase I and their concentration was determined by OD_260_ using a NanoDrop ND-1000 instrument. The OD_260_/OD_280_ ratios were inspected as an indication of potential impurities.

### 5.4. RT-qPCR

cDNA was generated by reverse transcription with the HiFlex miScript II RT Kit using 1 µg of DNase-treated RNA, following the manufacturer’s protocol. After diluting the cDNA 1/10, qPCR was performed using the SsoAdvanced Universal SYBR Green Supermix (Bio-Rad, Cat No.: 1725271, Hercules, CA, USA) in 0.1 mL MicroAmp™ Fast Optical 96-Well Reaction Plate (Applied Biosystem™, Cat No.: 4346907, Foster city, CA, USA), as described previously [122]. The final concentration of the primers (Integrated DNA Technologies, Inc., Coralville, IA, USA) was 100 nM. The primers used are listed in Table S2. To restrict the amplification to mRNAs of interest, primers were designed [123] to span exon–exon junction and tests were carried out to determine the best annealing temperatures associated with each pair of primers.

Unless otherwise stated, all data obtained were normalized with the reference gene ACTB and reported to the controls (uninfected, 24 h, 96 h) [124]. miRNA expression was defined based on the threshold cycle (Ct) and relative quantitation was calculated using the ∆∆Ct method [125].

### 5.5. Statistical Analysis of qPCR Data

The main objective was to evaluate the significance of the relative expression obtained following EBOV infection with the Mayinga, Makona or RESTV, compared to uninfected control. Unless otherwise specified, all qPCR results are expressed as mean ± standard error of the mean (SEM). Statistical significance was assessed using one-way analysis of variance (ANOVA) with Dunnett’s multiple comparisons test. When applicable, the nonparametric alternative Kruskal–Wallis one-way ANOVA was used. All statistical analyses were carried out with GraphPad Prism version 9.0.1 (GraphPad Software, Inc., La Jolla, CA, USA), with statistical significance set at *p* < 0.05.

### 5.6. Illumina Nextseq Sequencing

For each biological condition, an RNA sample was prepared by pooling equivalent amounts of total RNA isolated from 3 samples. In order to obtain the most representative data, we opted for a pooling strategy so to minimize the influence of interindividual variability. Previous studies have shown it to be a valid alternative to biological replicates at much reduced cost for large-scale gene expression approaches [60,61,62].

Total RNA was shipped on dry ice to the ArrayStar sequencing platform (Rockville, MD, USA). Total RNA of each sample was used to prepare the miRNA sequencing library, which included the following steps: (1) 3′-adaptor ligation with T4 RNA ligase 2 (truncated); (2) 5′-adaptor ligation with T4 RNA ligase; (3) cDNA synthesis with RT primer; (4) PCR amplification; (5) extraction and purification of ~130–150 bp PCR amplified fragments (corresponding to ~16–30 nt small RNAs) from the PAGE gel. After the completed libraries were quantified with Agilent 2100 Bioanalyzer, the DNA fragments in the libraries were denatured with 0.1 M NaOH to generate single-stranded DNA molecules, captured on Illumina flow cells, amplified in situ and finally sequenced for 51 cycles on Illumina Nextseq, according to the manufacturer’s instructions. The experiment workflow is summarized in the Supplementary Materials.

#### 5.6.1. Trimmed Reads

Subsequently, the 3′ adaptor sequence was trimmed from the clean reads, and the reads shorter than 16 nt were discarded. As the 5′ adaptor was also used as the sequencing primer site, the 5′-adaptor sequence is not present in the sequencing reads.

#### 5.6.2. miRNA Expression Profiles

For miRNA alignment, the maximum number of mismatches allowed was 1. When calculating miRNA expression, reads with counts less than 2 were discarded. miRNA expression levels were measured and normalized as transcripts per million (TPM) of total aligned miRNA reads [126]. miRNA read counts were used to estimate the expression level of each miRNA.

#### 5.6.3. Differentially Expressed miRNA

When comparing two groups of samples of profile differences (such as EBOV Mayinga versus Uninfected), the “fold change” (i.e., the ratio of the group averages) and *p*-value between each group are computed. miRNAs with fold changes ≥ 1.5, *p*-value ≤ 0.05 are considered as differentially expressed miRNAs. One can filter the analysis outputs and rank the differentially expressed genes according to fold change, using Microsoft Excel’s Data/Sort and Filter functionalities.

#### 5.6.4. Novel miRNAs

In order to report novel miRNAs with high confidence and recover the known miRNAs present in our sequenced samples, we used miRDeep [127] algorithms. For novel miRNA prediction, we pooled all sequence data from all 3′ adaptor-trimmed files. All adaptor trimmed sequences with length < 17 bp and mismatch > 1 were excluded from the prediction pipeline.

#### 5.6.5. GO and KEGG Annotation/Enrichment

We used two databases to predict human (hsa) DE miRNAs’ target genes: Targetscan 7.1 (http://www.targetscan.org/vert_71, accessed on 1 October 2020) and mirdbV6 (http://mirdb.org/miRDB/, accessed on 1 October 2020) with the following parameters: species: hsa; score: ≥ 70 (miRdbV6); Cumulative Weighted Context Score: <−0.3; Total Context ++ Score: <−0.3. The details of each prediction in the databases are in Supplementary Files A–D. The filtered input was used to calculate over-represented biological pathways by following the recommended guidelines [49,50,51]. The ID of GO term equals (−log10 (*p*-value)).

### 5.7. RNA22

To analyze the possible interactions between miRNAs and viral genomes (ZEBOV NC_002549 and RESTV NC_004161), we used RNA22 [112] with the following parameters: sensitivity vs. specificity setting: 63% vs. 61%; seed/nucleus region: seed size of 7, allow max of 1 UN-paired bases in seed; *p*-value (≤0.05), i.e., the likelihood that the target site loci is random/a lower *p*-value = greater chance that the loci contains a valid miRNA response elements.

## Figures and Tables

**Table 1:** Top 20 upregulated miRNAs in Huh7 cells 24 h post-EBOV infection, compared to uninfected control (C). FC, fold change.

Mayinga		Makona		Reston	
Mature miRNA	FC vs. C	Mature miRNA	FC vs. C	Mature miRNA	FC vs. C
miR-363-3p	5.18	miR-454-3p	3.36	miR-374a-3p	6.15
miR-374a-5p	4.57	miR-374a-3p	2.80	miR-26a-2-3p	4.14
miR-374b-5p	3.90	miR-374b-5p	2.37	miR-374b-5p	3.92
miR-374a-3p	3.80	miR-132-3p	2.36	miR-454-3p	3.90
miR-454-3p	3.78	miR-4483	2.31	miR-374a-5p	3.79
miR-32-5p	3.64	miR-novel-chr1_24913	2.23	miR-3613-5p	3.71
miR-429	3.57	miR-221-5p	2.20	miR-664a-3p	3.50
miR-20a-5p	2.90	miR-19a-3p	2.16	miR-3128	3.23
miR-26a-2-3p	2.86	miR-664a-3p	2.15	miR-95-3p	3.17
miR-200a-3p	2.81	miR-429	2.14	miR-126-5p	3.00
miR-126-5p	2.50	miR-652-5p	2.14	miR-652-5p	3.00
miR-216a-5p	2.50	miR-4521	2.14	miR-582-5p	2.87
miR-3613-5p	2.47	miR-374a-5p	2.13	miR-429	2.79
miR-novelchr16_13983	2.47	miR-6894-3p	2.00	miR-32-5p	2.77
miR-16-5p	2.41	miR-novel-chr20_29712	2.00	miR-582-3p	2.69
miR-16-5p	2.39	miR-novel-chr2_37626	2.00	miR-450b-5p	2.67
miR-548-3p	2.36	miR-32-5p	1.95	miR-21-5p	2.65
miR-18a-5p	2.32	miR-20a-5p	1.92	miR-30e-5p	2.62
miR-novelchr4_42996	2.31	miR-novel-chr10_1855	1.90	miR-148a-3p	2.55
miR-19a-3p	2.30	miR-4455	1.90	miR-548x-3p	2.45

**Table 2:** Top 20 upregulated miRNAs in Huh7 cells 96 h post-EBOV infection, compared to uninfected control (C). FC, fold change.

Mayinga		Makona		Reston	
Mature miRNA	FC vs. C	Mature miRNA	FC vs. C	Mature miRNA	FC vs. C
miR-27a-5p	22.35	miR-novel-chr1_24417	11.50	miR-novel-chr1_23305	16.80
miR-4443	21.58	miR-novel-chr7_52141	6.83	miR-193b-5p	14.33
miR-195-3p	11.18	miR-novel-chr4_43591	6.77	miR-novel-chr19_22180	9.70
miR-132-5p	9.82	miR-novel-chr1_26062	5.03	miR-novel-chr3_39440	9.30
miR-145-3p	9.73	miR-novel-chr14_11806	4.69	miR-novel-chr19_21743	8.00
miR-4485-3p	9.55	miR-novel-chr12_7186	4.67	miR-novel-chr5_47105	7.56
miR-novel-chr1_24417	9.19	miR-novel-chr19_21089	4.64	miR-1246	7.53
miR-novel-chr4_43591	9.12	miR-4443	4.50	miR-novel-chr17_17480	6.45
miR-novel-chr7_52141	8.87	miR-novel-chr6_48321	4.46	miR-novel-chr4_43591	6.23
miR-181b-3p	8.62	miR-novel-chr22_33060	4.26	miR-novel-chr7_52141	6.20
miR-novel-chr19_21743	7.89	miR-27a-5p	4.23	miR-27a-5p	5.98
miR-92a-1-5p	6.95	miR-92a-1-5p	4.05	miR-novel-chr12_8312	5.80
miR-181a-3p	6.91	miR-novel-chr20_29712	4.01	miR-novel-chr17_17167	5.77
miR-novel-chr19_22180	6.65	miR-195-3p	4.00	miR-4792	5.63
miR-novel-chr17_17480	6.60	miR-215-3p	3.95	miR-novel-chr12_6127	5.41
miR-193b-5p	6.39	miR-novel-chr10_1111	3.94	miR-26a-2-3p	5.40
miR-200a-5p	5.88	miR-novel-chr3_38884	3.81	miR-4485-3p	5.18
miR-novel-chr12_7186	5.72	miR-181b-3p	3.67	miR-1290	5.13
miR-novel-chr1_26062	5.68	miR-4497	3.64	miR-146a-3p	5.11
miR-novel-chr19_21089	5.30	miR-novel-chr1_24751	3.56	miR-novel-chr20_30085	5.07

**Table 3:** Top 20 downregulated miRNAs in Huh7 cells 24 h post-EBOV infection, compared to uninfected control (C). FC, fold change.

Mayinga		Makona		Reston	
Mature miRNA	FC vs. C	Mature miRNA	FC vs. C	Mature miRNA	FC vs. C
hsa-miR-novel-chr16_14823	0.19	hsa-miR-novel-chr16_14823	0.03	hsa-miR-novel-chr16_14823	0.04
hsa-miR-451a	0.27	hsa-miR-novel-chr5_47105	0.24	hsa-miR-novel-chr5_47105	0.15
hsa-miR-novel-chr2_35636	0.30	hsa-miR-451a	0.25	hsa-miR-novel-chr12_6591	0.17
hsa-miR-210-5p	0.31	hsa-miR-novel-chr15_12117	0.32	hsa-miR-3182	0.17
hsa-miR-486-5p	0.35	hsa-miR-143-3p	0.32	hsa-miR-novel-chr19_20368	0.18
hsa-miR-486-5p	0.35	hsa-miR-novel-chr2_36744	0.33	hsa-miR-novel-chr2_36744	0.18
hsa-miR-novel-chr5_47105	0.37	hsa-miR-novel-chr9_56521	0.34	hsa-miR-novel-chr2_34829	0.21
hsa-miR-novel-chr2_36744	0.39	hsa-miR-novel-chr8_54829	0.34	hsa-miR-novel-chr4_41778	0.21
hsa-miR-210-3p	0.40	hsa-miR-novel-chr2_34829	0.37	hsa-miR-novel-chr16_14481	0.24
hsa-miR-7974	0.40	hsa-miR-3182	0.38	hsa-miR-940	0.24
hsa-miR-491-5p	0.43	hsa-miR-210-5p	0.39	hsa-miR-novel-chr3_38187	0.25
hsa-miR-novel-chr17_18292	0.44	hsa-miR-210-3p	0.39	hsa-miR-210-3p	0.26
hsa-miR-novel-chr15_12117	0.44	hsa-miR-novel-chr2_35636	0.40	hsa-miR-novel-chr2_35636	0.26
hsa-miR-novel-chr4_41778	0.45	hsa-miR-199a-5p	0.40	hsa-miR-novel-chr1_26376	0.26
hsa-miR-novel-chr19_20494	0.45	hsa-miR-199a-5p	0.40	hsa-miR-novel-chr17_16473	0.26
hsa-miR-novel-chr9_56521	0.46	hsa-miR-199b-5p	0.40	hsa-miR-1291	0.27
hsa-miR-3182	0.47	hsa-miR-486-5p	0.42	hsa-miR-novel-chr3_39344	0.27
hsa-let-7i-5p	0.47	hsa-miR-novel-chr16_14481	0.42	hsa-miR-novel-chr3_38154	0.27
hsa-miR-224-5p	0.48	hsa-miR-novel-chr3_38187	0.42	hsa-miR-671-5p	0.28
hsa-miR-1908-3p	0.48	hsa-miR-486-5p	0.43	hsa-miR-novel-chr17_16481	0.29

**Table 4:** Top 20 downregulated miRNAs in Huh7 cells 96 h post-EBOV infection, compared to uninfected control (C). FC, fold change.

Mayinga		Makona		Reston	
Mature miRNA	FC vs. C	Mature miRNA	FC vs. C	Mature miRNA	FC vs. C
hsa-miR-671-5p	0.08	hsa-miR-7974	0.08	hsa-miR-4454	0.18
hsa-miR-7974	0.09	hsa-miR-671-5p	0.12	hsa-miR-582-5p	0.19
hsa-miR-novel-chr15_13530	0.12	hsa-miR-582-3p	0.13	hsa-miR-7974	0.20
hsa-miR-582-5p	0.15	hsa-miR-582-5p	0.15	hsa-miR-novel-chr15_13530	0.21
hsa-miR-326	0.16	hsa-miR-novel-chr15_13530	0.17	hsa-miR-novel-chr21_31339	0.27
hsa-miR-3182	0.21	hsa-miR-574-5p	0.17	hsa-miR-1306-3p	0.28
hsa-miR-novel-chr12_5743	0.21	hsa-miR-326	0.18	hsa-miR-1303	0.29
hsa-miR-novel-chr18_19540	0.22	hsa-miR-novel-chr4_41778	0.20	hsa-miR-326	0.29
hsa-miR-4454	0.22	hsa-miR-novel-chr12_8466	0.20	hsa-miR-582-3p	0.30
hsa-miR-1257	0.22	hsa-miR-1291	0.23	hsa-miR-5591-3p	0.30
hsa-miR-novel-chr16_14823	0.03	hsa-miR-novel-chr17_16082	0.23	hsa-miR-589-3p	0.32
hsa-miR-novel-chr5_47105	0.24	hsa-miR-novel-chr21_31339	0.23	hsa-miR-573	0.32
hsa-miR-451a	0.25	hsa-miR-novel-chr18_19540	0.24	hsa-miR-671-5p	0.33
hsa-miR-novel-chr15_12117	0.32	hsa-miR-1257	0.26	hsa-miR-342-3p	0.36
hsa-miR-143-3p	0.32	hsa-miR-3679-5p	0.27	hsa-miR-500a-5p	0.36
hsa-miR-novel-chr2_36744	0.33	hsa-miR-3182	0.27	hsa-miR-1304-3p	0.37
hsa-miR-novel-chr9_56521	0.34	hsa-miR-3187-3p	0.28	hsa-miR-653-5p	0.39
hsa-miR-novel-chr8_54829	0.34	hsa-miR-652-3p	0.28	hsa-miR-574-5p	0.39
hsa-miR-novel-chr2_34829	0.37	hsa-miR-5591-3p	0.29	hsa-miR-3201	0.39
hsa-miR-3182	0.38	hsa-miR-novel-chr12_5743	0.29	hsa-miR-novel-chr12_5743	0.40

## Data Availability

The data presented in this study are available on request from the corresponding author. The data are not publicly available due to the temporary unavailability of the mirbase repository system. However, the key raw data related to the DE miRNAs as well as the GO and KEGG outputs are attached in the Supplementary Files.

## Supplementary Materials

The following are available online at https://www.mdpi.com/article/10.3390/ijms22073792/s1.

## Abbreviations

EGFR	Epidermal Growth Factor Receptor
GO	Gene Ontology
HCV	Hepatitis C Virus
ICAM-1	Intercellular Adhesion Molecule-1
IL	Interleukin
KEGG	Kyoto Encyclopedia of Genes and Genomes
mTOR	mechanistic Target of Rapamycin
MOI	Multiplicity of infection
NF-kB	Nuclear factor kappa-light-chain-enhancer of activated β cells
RT-qPCR	Reverse Transcription-quantitative Polymerase Chain Reaction
TNF	Tumor Necrosis Factor
VCAM-1	Vascular Cell Adhesion Molecule-1
