# Supplementary material for: Altered microRNA Transcriptome in Cultured Human Liver Cells upon Infection with Ebola Virus
Source: Int J Mol Sci. 2021 Apr 6;22(7):3792. doi: 10.3390/ijms22073792 (PMC8038836; doi:10.3390/ijms22073792)
Supplement: Supplementary file 1 [file ijms-22-03792-s001.zip › Supplementary_File/C_ GO_Analysis_Results/16-30nt_go_Makona-24h-Huh7_vs_Control-24h-Huh7_down.mature_mirna_targets/BP_result(Human).html]

| GO.ID | Term | Ontology | Count | Pop.Hits | List.Total | Pop.Total | Fold.Enrichment | Pvalue | FDR | Enrichment.Score | Gene.Ratio | GENES |
| --- | --- | --- | --- | --- | --- | --- | --- | --- | --- | --- | --- | --- |
| GO:0090342 | regulation of cell aging | Biological process | 4 | 53 | 115 | 17653 | 11.5852337981952 | 0.000391266658989643 | 1 | 3.40752715806338 | 0.0347826086956522 | MIF//VASH1//KRAS//EEF1E1// |
| GO:0002720 | positive regulation of cytokine production involved in immune response | Biological process | 3 | 36 | 115 | 17653 | 12.7920289855072 | 0.0016441132066601 | 1 | 2.78406828209091 | 0.0260869565217391 | MAP3K7//MIF//FFAR3// |
| GO:0002367 | cytokine production involved in immune response | Biological process | 4 | 85 | 115 | 17653 | 7.22373401534527 | 0.00230635106952961 | 1 | 2.63707458431162 | 0.0347826086956522 | KDELR1//FFAR3//MAP3K7//MIF// |
| GO:0038166 | angiotensin-activated signaling pathway | Biological process | 2 | 12 | 115 | 17653 | 25.5840579710145 | 0.00266072852010825 | 1 | 2.57499943519444 | 0.0173913043478261 | AHCYL1//MAS1// |
| GO:0006662 | glycerol ether metabolic process | Biological process | 2 | 13 | 115 | 17653 | 23.6160535117057 | 0.00313114338179917 | 1 | 2.50429704464804 | 0.0173913043478261 | FAR1//TXNDC8// |
| GO:0044794 | positive regulation by host of viral process | Biological process | 2 | 13 | 115 | 17653 | 23.6160535117057 | 0.00313114338179917 | 1 | 2.50429704464804 | 0.0173913043478261 | PC//VAPA// |
| GO:2000772 | regulation of cellular senescence | Biological process | 3 | 45 | 115 | 17653 | 10.2336231884058 | 0.00313158129450028 | 1 | 2.50423630971882 | 0.0260869565217391 | KRAS//EEF1E1//VASH1// |
| GO:0051353 | positive regulation of oxidoreductase activity | Biological process | 3 | 47 | 115 | 17653 | 9.79814986123959 | 0.00354490308366069 | 1 | 2.45039563376913 | 0.0260869565217391 | KRAS//NPR3//ABL2// |
| GO:0001946 | lymphangiogenesis | Biological process | 2 | 15 | 115 | 17653 | 20.4672463768116 | 0.0041793032627315 | 1 | 2.37889611400778 | 0.0173913043478261 | VASH1//PROX2// |
| GO:1902188 | positive regulation of viral release from host cell | Biological process | 2 | 15 | 115 | 17653 | 20.4672463768116 | 0.0041793032627315 | 1 | 2.37889611400778 | 0.0173913043478261 | PC//VAPA// |
| GO:0032526 | response to retinoic acid | Biological process | 4 | 104 | 115 | 17653 | 5.90401337792642 | 0.00476613753646075 | 1 | 2.32183342937015 | 0.0347826086956522 | OSR1//ABL2//CDKN2D//IGFBP7// |
| GO:2000774 | positive regulation of cellular senescence | Biological process | 2 | 17 | 115 | 17653 | 18.0593350383632 | 0.00536739371191964 | 1 | 2.27023654694459 | 0.0173913043478261 | KRAS//EEF1E1// |
| GO:0038093 | Fc receptor signaling pathway | Biological process | 6 | 246 | 115 | 17653 | 3.7440084835631 | 0.00548525484863753 | 1 | 2.26080319000616 | 0.0521739130434783 | VAV3//KRAS//PSMB8//PSMC6//MAP3K7//ARPC3// |
| GO:0032722 | positive regulation of chemokine production | Biological process | 3 | 55 | 115 | 17653 | 8.37296442687747 | 0.00552353623595287 | 1 | 2.25778279255207 | 0.0260869565217391 | TSLP//MIF//FFAR3// |
| GO:0007569 | cell aging | Biological process | 4 | 111 | 115 | 17653 | 5.53168820994908 | 0.00599635965559669 | 1 | 2.22211232649809 | 0.0347826086956522 | MIF//VASH1//KRAS//EEF1E1// |
| GO:0007250 | activation of NF-kappaB-inducing kinase activity | Biological process | 2 | 18 | 115 | 17653 | 17.056038647343 | 0.00601273901112247 | 1 | 2.22092764672647 | 0.0173913043478261 | MAS1//MAP3K7// |
| GO:0018904 | ether metabolic process | Biological process | 2 | 18 | 115 | 17653 | 17.056038647343 | 0.00601273901112247 | 1 | 2.22092764672647 | 0.0173913043478261 | TXNDC8//FAR1// |
| GO:0036303 | lymph vessel morphogenesis | Biological process | 2 | 18 | 115 | 17653 | 17.056038647343 | 0.00601273901112247 | 1 | 2.22092764672647 | 0.0173913043478261 | PROX2//VASH1// |
| GO:1903902 | positive regulation of viral life cycle | Biological process | 3 | 57 | 115 | 17653 | 8.079176201373 | 0.00610279440460175 | 1 | 2.21447126062688 | 0.0260869565217391 | PC//VAPA//VAPB// |
| GO:0038095 | Fc-epsilon receptor signaling pathway | Biological process | 5 | 179 | 115 | 17653 | 4.28783094486276 | 0.00634282160839712 | 1 | 2.19771750294563 | 0.0434782608695652 | VAV3//KRAS//PSMB8//PSMC6//MAP3K7// |
| GO:0090343 | positive regulation of cell aging | Biological process | 2 | 19 | 115 | 17653 | 16.158352402746 | 0.00669166736545584 | 1 | 2.17446565541697 | 0.0173913043478261 | KRAS//EEF1E1// |
| GO:0006555 | methionine metabolic process | Biological process | 2 | 20 | 115 | 17653 | 15.3504347826087 | 0.00740372163633793 | 1 | 2.13054991813024 | 0.0173913043478261 | MTAP//AHCYL1// |
| GO:0051000 | positive regulation of nitric-oxide synthase activity | Biological process | 2 | 20 | 115 | 17653 | 15.3504347826087 | 0.00740372163633793 | 1 | 2.13054991813024 | 0.0173913043478261 | KRAS//NPR3// |
| GO:2001020 | regulation of response to DNA damage stimulus | Biological process | 5 | 186 | 115 | 17653 | 4.12646096306685 | 0.00743038506465157 | 1 | 2.12898867922461 | 0.0434782608695652 | POLH//PPP4R2//EEF1E1//MIF//CDKN2D// |
| GO:0033554 | cellular response to stress | Biological process | 22 | 1962 | 115 | 17653 | 1.7212516066126 | 0.00757980806963829 | 1 | 2.12034179111627 | 0.191304347826087 | CDKN2D//POLH//POLI//INO80D//VAV3//VAPB//OSR1//MAP3K7//PPP4R2//MIF//PSMC6//GIGYF2//SRXN1//TXNDC8//STAC//UFM1//TMX1//MAPK7//EEF1E1//PSMB8//VASH1//KRAS// |
| GO:0043687 | post-translational protein modification | Biological process | 8 | 442 | 115 | 17653 | 2.77835923667126 | 0.00834472347921287 | 1 | 2.07858805005025 | 0.0695652173913043 | TECTB//KLHL2//IGFBP5//IGFBP7//GOLM1//PSMB8//PSMC6//RAB2A// |
| GO:0070555 | response to interleukin-1 | Biological process | 5 | 192 | 115 | 17653 | 3.99750905797101 | 0.00846099489768142 | 1 | 2.07257856684646 | 0.0434782608695652 | PSMB8//PSMC6//MAP3K7//CCL16//SLC30A8// |
| GO:0002223 | stimulatory C-type lectin receptor signaling pathway | Biological process | 4 | 123 | 115 | 17653 | 4.99201131141746 | 0.0085663807223169 | 1 | 2.06720262782329 | 0.0347826086956522 | KRAS//PSMB8//PSMC6//MAP3K7// |
| GO:0002718 | regulation of cytokine production involved in immune response | Biological process | 3 | 66 | 115 | 17653 | 6.97747035573123 | 0.00914962168269055 | 1 | 2.03859686271132 | 0.0260869565217391 | FFAR3//MAP3K7//MIF// |
| GO:0002220 | innate immune response activating cell surface receptor signaling pathway | Biological process | 4 | 126 | 115 | 17653 | 4.87315389924086 | 0.00930558975632695 | 1 | 2.03125609756792 | 0.0347826086956522 | KRAS//PSMB8//PSMC6//MAP3K7// |
| GO:0038061 | NIK/NF-kappaB signaling | Biological process | 4 | 126 | 115 | 17653 | 4.87315389924086 | 0.00930558975632695 | 1 | 2.03125609756792 | 0.0347826086956522 | MAS1//MAP3K7//PSMB8//PSMC6// |
| GO:0071577 | zinc ion transmembrane transport | Biological process | 2 | 23 | 115 | 17653 | 13.3482041587902 | 0.00973413494193359 | 1 | 2.01170263753252 | 0.0173913043478261 | SLC30A8//SLC39A11// |
| GO:1904385 | cellular response to angiotensin | Biological process | 2 | 23 | 115 | 17653 | 13.3482041587902 | 0.00973413494193359 | 1 | 2.01170263753252 | 0.0173913043478261 | AHCYL1//MAS1// |
| GO:0001945 | lymph vessel development | Biological process | 2 | 24 | 115 | 17653 | 12.7920289855072 | 0.0105742097371406 | 1 | 1.97575207970928 | 0.0173913043478261 | PROX2//VASH1// |
| GO:0070227 | lymphocyte apoptotic process | Biological process | 3 | 70 | 115 | 17653 | 6.57875776397515 | 0.0107420732767137 | 1 | 1.96891188942432 | 0.0260869565217391 | MIF//KDELR1//TSC22D3// |
| GO:0006888 | ER to Golgi vesicle-mediated transport | Biological process | 5 | 205 | 115 | 17653 | 3.7440084835631 | 0.011026117790398 | 1 | 1.95757737232776 | 0.0434782608695652 | VAPB//VAPA//KDELR1//TRAPPC8//ZW10// |
| GO:0090398 | cellular senescence | Biological process | 3 | 71 | 115 | 17653 | 6.48609920391917 | 0.0111636356579107 | 1 | 1.95219434580764 | 0.0260869565217391 | VASH1//KRAS//EEF1E1// |
| GO:0006829 | zinc ion transport | Biological process | 2 | 26 | 115 | 17653 | 11.8080267558528 | 0.0123466465137428 | 1 | 1.90845098559023 | 0.0173913043478261 | SLC30A8//SLC39A11// |
| GO:0044788 | modulation by host of viral process | Biological process | 2 | 26 | 115 | 17653 | 11.8080267558528 | 0.0123466465137428 | 1 | 1.90845098559023 | 0.0173913043478261 | PC//VAPA// |
| GO:1990776 | response to angiotensin | Biological process | 2 | 26 | 115 | 17653 | 11.8080267558528 | 0.0123466465137428 | 1 | 1.90845098559023 | 0.0173913043478261 | AHCYL1//MAS1// |
| GO:0002702 | positive regulation of production of molecular mediator of immune response | Biological process | 3 | 74 | 115 | 17653 | 6.22314923619271 | 0.0124851949550797 | 1 | 1.90360467179447 | 0.0260869565217391 | FFAR3//MAP3K7//MIF// |
| GO:0032642 | regulation of chemokine production | Biological process | 3 | 74 | 115 | 17653 | 6.22314923619271 | 0.0124851949550797 | 1 | 1.90360467179447 | 0.0260869565217391 | FFAR3//TSLP//MIF// |
| GO:0000731 | DNA synthesis involved in DNA repair | Biological process | 3 | 75 | 115 | 17653 | 6.14017391304348 | 0.0129447810335891 | 1 | 1.88790529144064 | 0.0260869565217391 | POLI//POLH//CDKN2D// |
| GO:0007030 | Golgi organization | Biological process | 4 | 139 | 115 | 17653 | 4.41739130434783 | 0.0129859978744651 | 1 | 1.88652467254814 | 0.0347826086956522 | UBXN2B//TRAPPC8//RAB2A//ZW10// |
| GO:0032770 | positive regulation of monooxygenase activity | Biological process | 2 | 27 | 115 | 17653 | 11.370692431562 | 0.0132781506037863 | 1 | 1.87686240979496 | 0.0173913043478261 | KRAS//NPR3// |
| GO:0006464 | cellular protein modification process | Biological process | 38 | 4192 | 115 | 17653 | 1.39149933620976 | 0.0147199738394812 | 1 | 1.83209296183134 | 0.330434782608696 | CDKN2D//KRAS//MAPK7//PSMB8//PSMC6//MAP3K7//UBE2E1//MAS1//CDK2AP1//UBE2E3//KLHL2//PAN3//PPP4R2//METTL21A//PCMT1//ASPH//MOGS//TET1//TECTB//VAPB//GXYLT1//INO80D//OSR1//ABL2//ZDHHC20//NDFIP2//MIF//TSLP//RIT2//UBXN2B//IGFBP5//IGFBP7//GOLM1//RAB2A//PRDM4//LRP8//CCL16//UFM1// |
| GO:0036211 | protein modification process | Biological process | 38 | 4192 | 115 | 17653 | 1.39149933620976 | 0.0147199738394812 | 1 | 1.83209296183134 | 0.330434782608696 | CDKN2D//KRAS//MAPK7//PSMB8//PSMC6//MAP3K7//UBE2E1//MAS1//CDK2AP1//UBE2E3//KLHL2//PPP4R2//ABL2//PAN3//METTL21A//PCMT1//ASPH//MOGS//TET1//TECTB//VAPB//GXYLT1//INO80D//OSR1//ZDHHC20//NDFIP2//MIF//TSLP//RIT2//UBXN2B//IGFBP5//IGFBP7//GOLM1//RAB2A//PRDM4//LRP8//CCL16//UFM1// |
| GO:1902230 | negative regulation of intrinsic apoptotic signaling pathway in response to DNA damage | Biological process | 2 | 29 | 115 | 17653 | 10.5865067466267 | 0.0152296168860232 | 1 | 1.81731102157393 | 0.0173913043478261 | MIF//CDKN2D// |
| GO:0031145 | anaphase-promoting complex-dependent catabolic process | Biological process | 3 | 80 | 115 | 17653 | 5.75641304347826 | 0.0153869683109759 | 1 | 1.81284694063081 | 0.0260869565217391 | PSMB8//PSMC6//UBE2E1// |
| GO:0032602 | chemokine production | Biological process | 3 | 81 | 115 | 17653 | 5.685346215781 | 0.0159044213720567 | 1 | 1.79848212658768 | 0.0260869565217391 | FFAR3//TSLP//MIF// |
| GO:0043516 | regulation of DNA damage response, signal transduction by p53 class mediator | Biological process | 2 | 30 | 115 | 17653 | 10.2336231884058 | 0.0162487461789468 | 1 | 1.78918014536653 | 0.0173913043478261 | EEF1E1//MIF// |
| GO:0048873 | homeostasis of number of cells within a tissue | Biological process | 2 | 30 | 115 | 17653 | 10.2336231884058 | 0.0162487461789468 | 1 | 1.78918014536653 | 0.0173913043478261 | GIGYF2//KRAS// |
| GO:0044070 | regulation of anion transport | Biological process | 3 | 83 | 115 | 17653 | 5.54834992142483 | 0.0169684994115682 | 1 | 1.77035656228402 | 0.0260869565217391 | MIF//OSR1//AHCYL1// |
| GO:0021522 | spinal cord motor neuron differentiation | Biological process | 2 | 31 | 115 | 17653 | 9.90350631136045 | 0.0172962577920478 | 1 | 1.76204785040109 | 0.0173913043478261 | GIGYF2//LMO4// |
| GO:0070229 | negative regulation of lymphocyte apoptotic process | Biological process | 2 | 31 | 115 | 17653 | 9.90350631136045 | 0.0172962577920478 | 1 | 1.76204785040109 | 0.0173913043478261 | MIF//TSC22D3// |
| GO:1902186 | regulation of viral release from host cell | Biological process | 2 | 31 | 115 | 17653 | 9.90350631136045 | 0.0172962577920478 | 1 | 1.76204785040109 | 0.0173913043478261 | PC//VAPA// |
| GO:0002369 | T cell cytokine production | Biological process | 2 | 34 | 115 | 17653 | 9.02966751918159 | 0.0206050438151642 | 1 | 1.686026457642 | 0.0173913043478261 | MAP3K7//KDELR1// |
| GO:0030815 | negative regulation of cAMP metabolic process | Biological process | 2 | 34 | 115 | 17653 | 9.02966751918159 | 0.0206050438151642 | 1 | 1.686026457642 | 0.0173913043478261 | NPR3//MAPK7// |
| GO:0045070 | positive regulation of viral genome replication | Biological process | 2 | 34 | 115 | 17653 | 9.02966751918159 | 0.0206050438151642 | 1 | 1.686026457642 | 0.0173913043478261 | VAPA//VAPB// |
| GO:0051453 | regulation of intracellular pH | Biological process | 3 | 91 | 115 | 17653 | 5.0605828953655 | 0.0216156680083183 | 1 | 1.6652313385322 | 0.0260869565217391 | TTPA//ATP6V1A//SLC9B1// |
| GO:0019076 | viral release from host cell | Biological process | 2 | 35 | 115 | 17653 | 8.77167701863354 | 0.0217620618740782 | 1 | 1.66229995924443 | 0.0173913043478261 | PC//VAPA// |
| GO:0035890 | exit from host | Biological process | 2 | 35 | 115 | 17653 | 8.77167701863354 | 0.0217620618740782 | 1 | 1.66229995924443 | 0.0173913043478261 | PC//VAPA// |
| GO:0035891 | exit from host cell | Biological process | 2 | 35 | 115 | 17653 | 8.77167701863354 | 0.0217620618740782 | 1 | 1.66229995924443 | 0.0173913043478261 | PC//VAPA// |
| GO:0052126 | movement in host environment | Biological process | 2 | 35 | 115 | 17653 | 8.77167701863354 | 0.0217620618740782 | 1 | 1.66229995924443 | 0.0173913043478261 | PC//VAPA// |
| GO:0052192 | movement in environment of other organism involved in symbiotic interaction | Biological process | 2 | 35 | 115 | 17653 | 8.77167701863354 | 0.0217620618740782 | 1 | 1.66229995924443 | 0.0173913043478261 | PC//VAPA// |
| GO:0051341 | regulation of oxidoreductase activity | Biological process | 3 | 92 | 115 | 17653 | 5.00557655954631 | 0.0222406716578658 | 1 | 1.65285210140202 | 0.0260869565217391 | KRAS//NPR3//ABL2// |
| GO:0042127 | regulation of cell proliferation | Biological process | 18 | 1668 | 115 | 17653 | 1.65652173913043 | 0.0224047435689285 | 1 | 1.64966002239306 | 0.156521739130435 | VASH1//KRAS//MAB21L1//MAS1//TET1//TSLP//CDKN2D//IGFBP7//ASPH//EEF1E1//VAV3//MIF//NPR3//HLA-DPA1//FOSL2//IGFBP5//OSR1//ABL2// |
| GO:0030800 | negative regulation of cyclic nucleotide metabolic process | Biological process | 2 | 36 | 115 | 17653 | 8.5280193236715 | 0.0229454706192204 | 1 | 1.63930303035396 | 0.0173913043478261 | NPR3//MAPK7// |
| GO:1902229 | regulation of intrinsic apoptotic signaling pathway in response to DNA damage | Biological process | 2 | 37 | 115 | 17653 | 8.29753231492362 | 0.0241548834757939 | 1 | 1.61699505322784 | 0.0173913043478261 | MIF//CDKN2D// |
| GO:0030641 | regulation of cellular pH | Biological process | 3 | 95 | 115 | 17653 | 4.8475057208238 | 0.0241745661615397 | 1 | 1.61664131105427 | 0.0260869565217391 | TTPA//SLC9B1//ATP6V1A// |
| GO:2001243 | negative regulation of intrinsic apoptotic signaling pathway | Biological process | 3 | 96 | 115 | 17653 | 4.79701086956522 | 0.0248388204346214 | 1 | 1.60486903212261 | 0.0260869565217391 | MIF//MAPK7//CDKN2D// |
| GO:0071347 | cellular response to interleukin-1 | Biological process | 4 | 170 | 115 | 17653 | 3.61186700767263 | 0.0251571971568438 | 1 | 1.59933774665774 | 0.0347826086956522 | PSMB8//PSMC6//MAP3K7//CCL16// |
| GO:0000096 | sulfur amino acid metabolic process | Biological process | 2 | 38 | 115 | 17653 | 8.079176201373 | 0.0253899177083388 | 1 | 1.59533870672267 | 0.0173913043478261 | MTAP//AHCYL1// |
| GO:0019048 | modulation by virus of host morphology or physiology | Biological process | 2 | 38 | 115 | 17653 | 8.079176201373 | 0.0253899177083388 | 1 | 1.59533870672267 | 0.0173913043478261 | VAPA//VAPB// |
| GO:1901988 | negative regulation of cell cycle phase transition | Biological process | 5 | 256 | 115 | 17653 | 2.99813179347826 | 0.0261218239525076 | 1 | 1.58299650179002 | 0.0434782608695652 | ZW10//PSMB8//PSMC6//GIGYF2//CDKN2D// |
| GO:0048009 | insulin-like growth factor receptor signaling pathway | Biological process | 2 | 39 | 115 | 17653 | 7.87201783723523 | 0.0266501943878334 | 1 | 1.57429961886047 | 0.0173913043478261 | IGFBP5//GIGYF2// |
| GO:0010948 | negative regulation of cell cycle process | Biological process | 6 | 350 | 115 | 17653 | 2.63150310559006 | 0.027042609887338 | 1 | 1.56795139686604 | 0.0521739130434783 | ZW10//PSMB8//PSMC6//GIGYF2//MIF//CDKN2D// |
| GO:0070498 | interleukin-1-mediated signaling pathway | Biological process | 3 | 100 | 115 | 17653 | 4.60513043478261 | 0.0275938309412873 | 1 | 1.5591880008197 | 0.0260869565217391 | PSMB8//PSMC6//MAP3K7// |
| GO:0043902 | positive regulation of multi-organism process | Biological process | 4 | 175 | 115 | 17653 | 3.50867080745342 | 0.0275965969315018 | 1 | 1.55914446956493 | 0.0347826086956522 | MIF//PC//VAPA//VAPB// |
| GO:0002429 | immune response-activating cell surface receptor signaling pathway | Biological process | 7 | 450 | 115 | 17653 | 2.38784541062802 | 0.0278751790058857 | 1 | 1.55478233502831 | 0.0608695652173913 | KRAS//PSMB8//PSMC6//MAP3K7//HLA-DPA1//ARPC3//VAV3// |
| GO:0006885 | regulation of pH | Biological process | 3 | 102 | 115 | 17653 | 4.51483375959079 | 0.0290300242118915 | 1 | 1.53715260195384 | 0.0260869565217391 | TTPA//SLC9B1//ATP6V1A// |
| GO:0071887 | leukocyte apoptotic process | Biological process | 3 | 102 | 115 | 17653 | 4.51483375959079 | 0.0290300242118915 | 1 | 1.53715260195384 | 0.0260869565217391 | MIF//KDELR1//TSC22D3// |
| GO:0043412 | macromolecule modification | Biological process | 38 | 4389 | 115 | 17653 | 1.32904197252023 | 0.0295669953186444 | 1 | 1.52919280746019 | 0.330434782608696 | CDKN2D//KRAS//MAPK7//PSMB8//PSMC6//MAP3K7//UBE2E1//MAS1//CDK2AP1//UBE2E3//KLHL2//PPP4R2//ABL2//PAN3//METTL21A//PCMT1//ASPH//MOGS//TET1//TECTB//VAPB//GXYLT1//INO80D//OSR1//ZDHHC20//NDFIP2//MIF//TSLP//RIT2//UBXN2B//IGFBP5//IGFBP7//GOLM1//RAB2A//PRDM4//LRP8//CCL16//UFM1// |
| GO:0048524 | positive regulation of viral process | Biological process | 3 | 103 | 115 | 17653 | 4.47100042211904 | 0.0297627572270047 | 1 | 1.52632683814612 | 0.0260869565217391 | PC//VAPA//VAPB// |
| GO:0006479 | protein methylation | Biological process | 4 | 180 | 115 | 17653 | 3.4112077294686 | 0.03017289779501 | 1 | 1.52038297832525 | 0.0347826086956522 | METTL21A//TET1//PRDM4//PCMT1// |
| GO:0008213 | protein alkylation | Biological process | 4 | 180 | 115 | 17653 | 3.4112077294686 | 0.03017289779501 | 1 | 1.52038297832525 | 0.0347826086956522 | METTL21A//PCMT1//TET1//PRDM4// |
| GO:0019985 | translesion synthesis | Biological process | 2 | 42 | 115 | 17653 | 7.30973084886128 | 0.0305787462307475 | 1 | 1.51458032520985 | 0.0173913043478261 | POLI//POLH// |
| GO:0030890 | positive regulation of B cell proliferation | Biological process | 2 | 42 | 115 | 17653 | 7.30973084886128 | 0.0305787462307475 | 1 | 1.51458032520985 | 0.0173913043478261 | VAV3//MIF// |
| GO:0080135 | regulation of cellular response to stress | Biological process | 9 | 672 | 115 | 17653 | 2.05586180124224 | 0.0314743125205072 | 1 | 1.50204374721886 | 0.0782608695652174 | POLH//PPP4R2//MAP3K7//EEF1E1//MIF//MAPK7//CDKN2D//VASH1//KRAS// |
| GO:0010470 | regulation of gastrulation | Biological process | 2 | 43 | 115 | 17653 | 7.13973710819009 | 0.0319362783996114 | 1 | 1.49571569448065 | 0.0173913043478261 | MAPK7//OSR1// |
| GO:1901224 | positive regulation of NIK/NF-kappaB signaling | Biological process | 2 | 43 | 115 | 17653 | 7.13973710819009 | 0.0319362783996114 | 1 | 1.49571569448065 | 0.0173913043478261 | MAS1//MAP3K7// |
| GO:0051817 | modification of morphology or physiology of other organism involved in symbiotic interaction | Biological process | 3 | 107 | 115 | 17653 | 4.303860219423 | 0.0327909519970323 | 1 | 1.48424597457625 | 0.0260869565217391 | VAPB//PC//VAPA// |
| GO:0031349 | positive regulation of defense response | Biological process | 7 | 466 | 115 | 17653 | 2.3058593021086 | 0.0327986750865507 | 1 | 1.48414369940483 | 0.0608695652173913 | KRAS//PSMB8//PSMC6//MAP3K7//FFAR3//CCL16//TSLP// |
| GO:0006520 | cellular amino acid metabolic process | Biological process | 6 | 367 | 115 | 17653 | 2.50960786636654 | 0.0330017764012675 | 1 | 1.48146268253107 | 0.0521739130434783 | IARS//EEF1E1//PSMB8//PSMC6//MTAP//AHCYL1// |
| GO:0006790 | sulfur compound metabolic process | Biological process | 6 | 367 | 115 | 17653 | 2.50960786636654 | 0.0330017764012675 | 1 | 1.48146268253107 | 0.0521739130434783 | EEF1E1//PC//MTAP//XYLT2//AHCYL1//FAR1// |
| GO:0021517 | ventral spinal cord development | Biological process | 2 | 44 | 115 | 17653 | 6.97747035573123 | 0.0333172143334968 | 1 | 1.47733131729422 | 0.0173913043478261 | LMO4//GIGYF2// |
| GO:0080134 | regulation of response to stress | Biological process | 16 | 1500 | 115 | 17653 | 1.63737971014493 | 0.0338601822823585 | 1 | 1.47031070824689 | 0.139130434782609 | KRAS//PSMB8//PSMC6//MAP3K7//FFAR3//POLH//PPP4R2//EEF1E1//MIF//MAS1//MAPK7//TNFAIP8L2//CCL16//TSLP//CDKN2D//VASH1// |
| GO:0000041 | transition metal ion transport | Biological process | 3 | 110 | 115 | 17653 | 4.18648221343873 | 0.0351637697037157 | 1 | 1.45390457291102 | 0.0260869565217391 | SLC30A8//ATP6V1A//SLC39A11// |
| GO:0019221 | cytokine-mediated signaling pathway | Biological process | 10 | 798 | 115 | 17653 | 1.92361338127929 | 0.0354583926358641 | 1 | 1.45028095528887 | 0.0869565217391304 | TSLP//PSMB8//PSMC6//MIF//MTAP//HLA-DPA1//CCL16//MAP3K7//KRAS//LRP8// |
| GO:0044003 | modification by symbiont of host morphology or physiology | Biological process | 2 | 46 | 115 | 17653 | 6.67410207939508 | 0.0361478740122011 | 1 | 1.44191724005467 | 0.0173913043478261 | VAPB//VAPA// |
| GO:0042327 | positive regulation of phosphorylation | Biological process | 12 | 1032 | 115 | 17653 | 1.78493427704752 | 0.0364806717857321 | 1 | 1.43793717280661 | 0.104347826086957 | MAP3K7//KRAS//CDK2AP1//VAPB//MAS1//MIF//LMO4//TSLP//RIT2//VAV3//LRP8//CCL16// |
| GO:0050851 | antigen receptor-mediated signaling pathway | Biological process | 5 | 282 | 115 | 17653 | 2.72170829478878 | 0.0373090867577703 | 1 | 1.42818538137769 | 0.0434782608695652 | HLA-DPA1//PSMB8//PSMC6//MAP3K7//VAV3// |
| GO:0035722 | interleukin-12-mediated signaling pathway | Biological process | 2 | 47 | 115 | 17653 | 6.53209990749306 | 0.0375968949423585 | 1 | 1.42484802116499 | 0.0173913043478261 | MIF//MTAP// |
| GO:0050999 | regulation of nitric-oxide synthase activity | Biological process | 2 | 47 | 115 | 17653 | 6.53209990749306 | 0.0375968949423585 | 1 | 1.42484802116499 | 0.0173913043478261 | KRAS//NPR3// |
| GO:0071349 | cellular response to interleukin-12 | Biological process | 2 | 47 | 115 | 17653 | 6.53209990749306 | 0.0375968949423585 | 1 | 1.42484802116499 | 0.0173913043478261 | MIF//MTAP// |
| GO:0002768 | immune response-regulating cell surface receptor signaling pathway | Biological process | 7 | 482 | 115 | 17653 | 2.2293162547357 | 0.0382752711446702 | 1 | 1.41708172404663 | 0.0608695652173913 | KRAS//PSMB8//PSMC6//MAP3K7//HLA-DPA1//VAV3//ARPC3// |
| GO:0030004 | cellular monovalent inorganic cation homeostasis | Biological process | 3 | 114 | 115 | 17653 | 4.0395881006865 | 0.0384620922599398 | 1 | 1.4149670947145 | 0.0260869565217391 | TTPA//SLC9B1//ATP6V1A// |
| GO:0033674 | positive regulation of kinase activity | Biological process | 8 | 590 | 115 | 17653 | 2.08141488577745 | 0.0390325044085392 | 1 | 1.40857358256668 | 0.0695652173913043 | MAP3K7//VAPB//MAS1//KRAS//MIF//VAV3//LRP8//LMO4// |
| GO:0051452 | intracellular pH reduction | Biological process | 2 | 48 | 115 | 17653 | 6.39601449275362 | 0.0390679139467103 | 1 | 1.40817977755483 | 0.0173913043478261 | ATP6V1A//TTPA// |
| GO:0070231 | T cell apoptotic process | Biological process | 2 | 48 | 115 | 17653 | 6.39601449275362 | 0.0390679139467103 | 1 | 1.40817977755483 | 0.0173913043478261 | TSC22D3//KDELR1// |
| GO:0002700 | regulation of production of molecular mediator of immune response | Biological process | 3 | 115 | 115 | 17653 | 4.00446124763705 | 0.039310552792512 | 1 | 1.4054908490049 | 0.0260869565217391 | FFAR3//MAP3K7//MIF// |
| GO:0031347 | regulation of defense response | Biological process | 10 | 813 | 115 | 17653 | 1.88812235948446 | 0.0394625252593028 | 1 | 1.403815127115 | 0.0869565217391304 | KRAS//PSMB8//PSMC6//MAP3K7//FFAR3//MAS1//MAPK7//TNFAIP8L2//CCL16//TSLP// |
| GO:0050715 | positive regulation of cytokine secretion | Biological process | 3 | 116 | 115 | 17653 | 3.96994002998501 | 0.0401685160433188 | 1 | 1.39611421223403 | 0.0260869565217391 | TSLP//CLEC9A//MIF// |
| GO:0006418 | tRNA aminoacylation for protein translation | Biological process | 2 | 49 | 115 | 17653 | 6.26548358473824 | 0.0405605884073722 | 1 | 1.39189575337341 | 0.0173913043478261 | IARS//EEF1E1// |
| GO:0045851 | pH reduction | Biological process | 2 | 49 | 115 | 17653 | 6.26548358473824 | 0.0405605884073722 | 1 | 1.39189575337341 | 0.0173913043478261 | TTPA//ATP6V1A// |
| GO:0070671 | response to interleukin-12 | Biological process | 2 | 49 | 115 | 17653 | 6.26548358473824 | 0.0405605884073722 | 1 | 1.39189575337341 | 0.0173913043478261 | MIF//MTAP// |
| GO:2000107 | negative regulation of leukocyte apoptotic process | Biological process | 2 | 49 | 115 | 17653 | 6.26548358473824 | 0.0405605884073722 | 1 | 1.39189575337341 | 0.0173913043478261 | MIF//TSC22D3// |
| GO:0007188 | adenylate cyclase-modulating G-protein coupled receptor signaling pathway | Biological process | 4 | 199 | 115 | 17653 | 3.08551452916758 | 0.0412272575569196 | 1 | 1.38481555356899 | 0.0347826086956522 | RIT2//FFAR3//NPR3//GNA14// |
| GO:0050852 | T cell receptor signaling pathway | Biological process | 4 | 200 | 115 | 17653 | 3.07008695652174 | 0.0418648749262258 | 1 | 1.37815020193404 | 0.0347826086956522 | HLA-DPA1//PSMB8//PSMC6//MAP3K7// |
| GO:0032890 | regulation of organic acid transport | Biological process | 2 | 50 | 115 | 17653 | 6.14017391304348 | 0.042074579167735 | 1 | 1.37598021916781 | 0.0173913043478261 | MIF//OSR1// |
| GO:0043618 | regulation of transcription from RNA polymerase II promoter in response to stress | Biological process | 3 | 119 | 115 | 17653 | 3.86985750822068 | 0.0427991552290828 | 1 | 1.3685648030188 | 0.0260869565217391 | MAPK7//PSMB8//PSMC6// |
| GO:0050871 | positive regulation of B cell activation | Biological process | 3 | 119 | 115 | 17653 | 3.86985750822068 | 0.0427991552290828 | 1 | 1.3685648030188 | 0.0260869565217391 | VAV3//MIF//ATP11C// |
| GO:0021515 | cell differentiation in spinal cord | Biological process | 2 | 51 | 115 | 17653 | 6.01977834612106 | 0.0436095505025577 | 1 | 1.36041838971421 | 0.0173913043478261 | LMO4//GIGYF2// |
| GO:0070936 | protein K48-linked ubiquitination | Biological process | 2 | 51 | 115 | 17653 | 6.01977834612106 | 0.0436095505025577 | 1 | 1.36041838971421 | 0.0173913043478261 | UBE2E3//UBE2E1// |
| GO:0055085 | transmembrane transport | Biological process | 16 | 1550 | 115 | 17653 | 1.58456100981767 | 0.0436675064808768 | 1 | 1.35984160662489 | 0.139130434782609 | ATP6V1A//AHCYL1//SLC9B1//TSC22D3//ATP11C//GRIA3//ASPH//SLC30A8//SLC39A11//STAC//OSR1//SLC25A15//FFAR3//PSMB8//PSMC6//SLC16A2// |
| GO:0043039 | tRNA aminoacylation | Biological process | 2 | 52 | 115 | 17653 | 5.90401337792642 | 0.0451651700882984 | 1 | 1.34519634991135 | 0.0173913043478261 | IARS//EEF1E1// |
| GO:0007568 | aging | Biological process | 5 | 298 | 115 | 17653 | 2.57557630580683 | 0.0454615110521254 | 1 | 1.34235613326803 | 0.0434782608695652 | MIF//VASH1//KRAS//EEF1E1//IGFBP5// |
| GO:0006301 | postreplication repair | Biological process | 2 | 53 | 115 | 17653 | 5.79261689909762 | 0.0467411089736808 | 1 | 1.33030098780522 | 0.0173913043478261 | POLI//POLH// |
| GO:0043038 | amino acid activation | Biological process | 2 | 53 | 115 | 17653 | 5.79261689909762 | 0.0467411089736808 | 1 | 1.33030098780522 | 0.0173913043478261 | IARS//EEF1E1// |
| GO:0048146 | positive regulation of fibroblast proliferation | Biological process | 2 | 53 | 115 | 17653 | 5.79261689909762 | 0.0467411089736808 | 1 | 1.33030098780522 | 0.0173913043478261 | FOSL2//MIF// |
| GO:0050729 | positive regulation of inflammatory response | Biological process | 3 | 124 | 115 | 17653 | 3.71381486676017 | 0.0473709869967814 | 1 | 1.3244875664481 | 0.0260869565217391 | FFAR3//CCL16//TSLP// |
| GO:0043620 | regulation of DNA-templated transcription in response to stress | Biological process | 3 | 125 | 115 | 17653 | 3.68410434782609 | 0.0483131898650012 | 1 | 1.31593428739376 | 0.0260869565217391 | MAPK7//PSMB8//PSMC6// |
| GO:0008219 | cell death | Biological process | 21 | 2216 | 115 | 17653 | 1.45468921676346 | 0.0486763417226443 | 1 | 1.31268206867881 | 0.182608695652174 | MIF//SMNDC1//FAM32A//CDKN2D//OSR1//UFM1//TSLP//ABL2//TNFAIP8L2//ANP32E//VAV3//EEF1E1//MAP3K7//KRAS//TTPA//KDELR1//TSC22D3//ITM2B//MAPK7//FOSL2//VAPA// |
| GO:0048662 | negative regulation of smooth muscle cell proliferation | Biological process | 2 | 55 | 115 | 17653 | 5.58197628458498 | 0.0499526455246286 | 1 | 1.30144150631041 | 0.0173913043478261 | IGFBP5//NPR3// |
| GO:0070228 | regulation of lymphocyte apoptotic process | Biological process | 2 | 55 | 115 | 17653 | 5.58197628458498 | 0.0499526455246286 | 1 | 1.30144150631041 | 0.0173913043478261 | MIF//TSC22D3// |
