# Supplementary material for: Altered microRNA Transcriptome in Cultured Human Liver Cells upon Infection with Ebola Virus
Source: Int J Mol Sci. 2021 Apr 6;22(7):3792. doi: 10.3390/ijms22073792 (PMC8038836; doi:10.3390/ijms22073792)
Supplement: Supplementary file 1 [file ijms-22-03792-s001.zip › Supplementary_File/C_ GO_Analysis_Results/16-30nt_go_Makona-24h-Huh7_vs_Control-24h-Huh7_down.mature_mirna_targets/CC_result(Human).html]

| GO.ID | Term | Ontology | Count | Pop.Hits | List.Total | Pop.Total | Fold.Enrichment | Pvalue | FDR | Enrichment.Score | Gene.Ratio | GENES |
| --- | --- | --- | --- | --- | --- | --- | --- | --- | --- | --- | --- | --- |
| GO:0017101 | aminoacyl-tRNA synthetase multienzyme complex | Cellular component | 2 | 12 | 118 | 18698 | 26.409604519774 | 0.00250082039848561 | 1 | 2.60191749689302 | 0.0169491525423729 | IARS//EEF1E1// |
| GO:0005622 | intracellular | Cellular component | 104 | 14673 | 118 | 18698 | 1.1231236434498 | 0.00485162189797052 | 1 | 2.31411305241046 | 0.88135593220339 | KDELR1//HLA-DPA1//NDFIP2//RAB2A//XYLT2//VAPB//VAPA//ITM2B//UBE2E1//GNA14//TRAPPC8//PSMB8//PSMC6//ZW10//PHF6//GABPA//GATAD2B//ANP32E//PAN3//TXNDC8//CCDC58//SLC9B1//KRAS//PC//ATP6V1A//ARPC3//CDKN2D//VAV3//UBE2E3//AHCYL1//KLHL2//KLF12//OSR1//UBXN2B//SRXN1//TSC22D3//NECAP1//GIGYF2//ABL2//IARS//MIF//MTAP//PCMT1//POLH//ASAP3//MAPK7//DDX55//STAC//MAP3K7//TTPA//CDK2AP1//FBP2//EEF1E1//FAR1//SMNDC1//WDR3//PPP4R2//SLC39A11//FOSL2//FAM32A//PROX2//MAB21L1//UFM1//INO80D//RIT2//SNAPC1//IFRD2//TET1//TIGD5//ZNF682//SRSF11//EFS//IFI44//PRDM4//MSI2//METTL21A//DNAH12//VASH1//ABHD14A//SGTB//BRD2//TNFAIP8L2//MEDAG//SNRNP27//POLI//LMO4//SLC25A15//ATP11C//ANKRD50//RASGEF1B//ZDHHC20//ASPH//MOGS//TMX1//IGFBP5//IGFBP7//GOLM1//SLC30A8//TMOD2//LRP8//FAM206A//GRIA3//MAS1//NPR3// |
| GO:0005783 | endoplasmic reticulum | Cellular component | 21 | 1859 | 118 | 18698 | 1.79000009117349 | 0.00598276519873338 | 1 | 2.2230980412655 | 0.177966101694915 | ASPH//IGFBP5//IGFBP7//GOLM1//AHCYL1//KDELR1//ATP11C//RAB2A//XYLT2//MOGS//TMX1//ZW10//VAPB//VAPA//HLA-DPA1//UBXN2B//VASH1//ZDHHC20//GIGYF2//UFM1//NDFIP2// |
| GO:0044424 | intracellular part | Cellular component | 102 | 14395 | 118 | 18698 | 1.1227980525253 | 0.00716836360030992 | 1 | 2.1445799741044 | 0.864406779661017 | UBE2E1//GNA14//PSMB8//PSMC6//EFS//SMNDC1//CDKN2D//IFI44//AHCYL1//PRDM4//KLHL2//MSI2//SRXN1//METTL21A//PPP4R2//TSC22D3//SLC39A11//DNAH12//VASH1//TXNDC8//ABHD14A//IARS//KRAS//MTAP//PC//PCMT1//UFM1//SGTB//NDFIP2//ASAP3//MAPK7//DDX55//RIT2//BRD2//TNFAIP8L2//MEDAG//FBP2//VAPB//EEF1E1//LMO4//TRAPPC8//ABL2//SLC25A15//HLA-DPA1//FAR1//ITM2B//CCDC58//SLC9B1//ATP6V1A//ARPC3//VAV3//UBE2E3//KLF12//OSR1//UBXN2B//PAN3//NECAP1//GIGYF2//MIF//POLH//RAB2A//STAC//MAP3K7//TTPA//CDK2AP1//ZW10//ZDHHC20//ATP11C//ASPH//MOGS//TMX1//VAPA//KDELR1//GOLM1//SLC30A8//ANP32E//POLI//IGFBP5//IGFBP7//TMOD2//SNAPC1//WDR3//FOSL2//GABPA//FAM32A//PROX2//MAB21L1//INO80D//IFRD2//TET1//PHF6//TIGD5//ZNF682//SRSF11//ANKRD50//RASGEF1B//GATAD2B//SNRNP27//LRP8//XYLT2//GRIA3//FAM206A// |
| GO:0031982 | vesicle | Cellular component | 39 | 4372 | 118 | 18698 | 1.41350582287903 | 0.0105276096011983 | 1 | 1.97767022851631 | 0.330508474576271 | PCMT1//SLC30A8//TRAPPC8//ANP32E//ARPC3//VAV3//AHCYL1//BROX//TXNDC8//ABHD14A//IARS//IGFBP7//MIF//MTAP//NPR3//GOLM1//UFM1//ATP6V1A//PSMB8//PSMC6//RAB2A//MOGS//ABHD8//FBP2//ITM2B//EEF1E1//GNA14//GIGYF2//ANKRD50//RASGEF1B//TTPA//KDELR1//ATP11C//NDFIP2//HLA-DPA1//GRIA3//VAPA//MAP3K7//NECAP1// |
| GO:0044432 | endoplasmic reticulum part | Cellular component | 15 | 1319 | 118 | 18698 | 1.802020020303 | 0.0188755678034856 | 1 | 1.7240999753167 | 0.127118644067797 | IGFBP5//IGFBP7//GOLM1//AHCYL1//KDELR1//ATP11C//ASPH//RAB2A//XYLT2//MOGS//TMX1//ZW10//VAPB//VAPA//HLA-DPA1// |
| GO:0043226 | organelle | Cellular component | 96 | 13597 | 118 | 18698 | 1.11877121448774 | 0.0193731006948191 | 1 | 1.71280086421221 | 0.813559322033898 | DNAH12//HLA-DPA1//NDFIP2//ASAP3//FAR1//ITM2B//MIF//VAPA//PCMT1//LRP8//CCDC58//SLC9B1//KRAS//PC//ATP6V1A//SMNDC1//CDKN2D//WDR3//OSR1//UBXN2B//PPP4R2//TSC22D3//SLC39A11//FOSL2//GABPA//FAM32A//PROX2//MAB21L1//MTAP//UFM1//INO80D//MAPK7//PSMB8//PSMC6//DDX55//RAB2A//RIT2//SNAPC1//MAP3K7//UBE2E1//IFRD2//TET1//CDK2AP1//ANP32E//PHF6//TIGD5//ZNF682//ZW10//SRSF11//EEF1E1//VASH1//ZDHHC20//GIGYF2//ATP11C//ASPH//MOGS//TMX1//VAPB//KDELR1//TXNDC8//GOLM1//SLC30A8//TRAPPC8//ARPC3//VAV3//AHCYL1//BROX//ABHD14A//IARS//IGFBP7//NPR3//ABHD8//FBP2//GNA14//TMOD2//KLHL2//ABL2//POLI//ANKRD50//RASGEF1B//TTPA//PAN3//GRIA3//XYLT2//GATAD2B//UBE2E3//SNRNP27//KLF12//METTL21A//POLH//BRD2//SLC25A15//IGFBP5//FAM206A//NECAP1//PRDM4// |
| GO:0043227 | membrane-bounded organelle | Cellular component | 90 | 12616 | 118 | 18698 | 1.13040475908988 | 0.0234842386316484 | 1 | 1.62922351525224 | 0.76271186440678 | HLA-DPA1//NDFIP2//ASAP3//FAR1//ITM2B//MIF//VAPA//CCDC58//SLC9B1//KRAS//PC//ATP6V1A//SMNDC1//CDKN2D//WDR3//OSR1//UBXN2B//PPP4R2//TSC22D3//SLC39A11//FOSL2//GABPA//FAM32A//PROX2//MAB21L1//MTAP//UFM1//INO80D//MAPK7//PSMB8//PSMC6//DDX55//RAB2A//RIT2//SNAPC1//MAP3K7//UBE2E1//IFRD2//TET1//CDK2AP1//ANP32E//PHF6//TIGD5//ZNF682//ZW10//SRSF11//EEF1E1//VASH1//ZDHHC20//GIGYF2//ATP11C//ASPH//MOGS//TMX1//VAPB//KDELR1//TXNDC8//GOLM1//PCMT1//SLC30A8//TRAPPC8//ARPC3//VAV3//AHCYL1//BROX//ABHD14A//IARS//IGFBP7//NPR3//ABHD8//FBP2//GNA14//ANKRD50//RASGEF1B//TTPA//GRIA3//XYLT2//SLC25A15//GATAD2B//UBE2E3//SNRNP27//POLI//KLF12//METTL21A//POLH//BRD2//FAM206A//PRDM4//IGFBP5//NECAP1// |
| GO:0031234 | extrinsic component of cytoplasmic side of plasma membrane | Cellular component | 3 | 110 | 118 | 18698 | 4.32157164869029 | 0.0324795122980852 | 1 | 1.48839050060822 | 0.0254237288135593 | GNA14//ABL2//KRAS// |
| GO:0005789 | endoplasmic reticulum membrane | Cellular component | 12 | 1050 | 118 | 18698 | 1.81094430992736 | 0.0333245435909177 | 1 | 1.4772357899202 | 0.101694915254237 | ASPH//HLA-DPA1//AHCYL1//KDELR1//ATP11C//RAB2A//XYLT2//MOGS//TMX1//ZW10//VAPB//VAPA// |
| GO:0016607 | nuclear speck | Cellular component | 6 | 381 | 118 | 18698 | 2.49539570265581 | 0.0338464746911215 | 1 | 1.4704865589411 | 0.0508474576271186 | SMNDC1//POLI//FAM206A//GATAD2B//BRD2//SRSF11// |
| GO:0098827 | endoplasmic reticulum subcompartment | Cellular component | 12 | 1054 | 118 | 18698 | 1.80407165599974 | 0.0341643796598807 | 1 | 1.46642646057679 | 0.101694915254237 | AHCYL1//KDELR1//ATP11C//ASPH//RAB2A//XYLT2//MOGS//TMX1//ZW10//VAPB//VAPA//HLA-DPA1// |
| GO:0030660 | Golgi-associated vesicle membrane | Cellular component | 3 | 113 | 118 | 18698 | 4.2068396580171 | 0.0347654809996542 | 1 | 1.45885175753249 | 0.0254237288135593 | HLA-DPA1//KDELR1//ITM2B// |
| GO:0042175 | nuclear outer membrane-endoplasmic reticulum membrane network | Cellular component | 12 | 1071 | 118 | 18698 | 1.775435597968 | 0.0379040896818391 | 1 | 1.42131392907061 | 0.101694915254237 | AHCYL1//KDELR1//ATP11C//ASPH//RAB2A//XYLT2//MOGS//TMX1//ZW10//VAPB//VAPA//HLA-DPA1// |
| GO:0005654 | nucleoplasm | Cellular component | 30 | 3472 | 118 | 18698 | 1.36916152464266 | 0.0395442047122286 | 1 | 1.4029171542881 | 0.254237288135593 | MAP3K7//SMNDC1//GATAD2B//MAPK7//POLI//FAM206A//BRD2//SRSF11//PRDM4//CDKN2D//UBE2E3//WDR3//SNRNP27//KLF12//METTL21A//PPP4R2//FOSL2//GABPA//MIF//POLH//INO80D//ASAP3//PSMB8//PSMC6//SNAPC1//UBE2E1//CDK2AP1//PHF6//FBP2//EEF1E1// |
| GO:0005737 | cytoplasm | Cellular component | 82 | 11544 | 118 | 18698 | 1.12556526539577 | 0.0486295664560588 | 1 | 1.31309960225129 | 0.694915254237288 | KDELR1//HLA-DPA1//NDFIP2//RAB2A//XYLT2//VAPB//VAPA//ITM2B//TRAPPC8//PAN3//TXNDC8//CCDC58//SLC9B1//KRAS//PC//ATP6V1A//ARPC3//CDKN2D//VAV3//UBE2E3//AHCYL1//KLHL2//KLF12//OSR1//UBXN2B//SRXN1//TSC22D3//NECAP1//GIGYF2//ABL2//IARS//MIF//MTAP//PCMT1//POLH//ASAP3//MAPK7//PSMB8//PSMC6//DDX55//STAC//MAP3K7//TTPA//UBE2E1//CDK2AP1//FBP2//ZW10//EEF1E1//FAR1//SLC25A15//ATP11C//ANKRD50//RASGEF1B//VASH1//ZDHHC20//ASPH//UFM1//MOGS//TMX1//IGFBP5//IGFBP7//GOLM1//SLC39A11//SLC30A8//TMOD2//GRIA3//ANP32E//POLI//EFS//SMNDC1//IFI44//PRDM4//MSI2//METTL21A//PPP4R2//DNAH12//ABHD14A//SGTB//RIT2//BRD2//TNFAIP8L2//MEDAG// |
