# Supplementary material for: Altered microRNA Transcriptome in Cultured Human Liver Cells upon Infection with Ebola Virus
Source: Int J Mol Sci. 2021 Apr 6;22(7):3792. doi: 10.3390/ijms22073792 (PMC8038836; doi:10.3390/ijms22073792)
Supplement: Supplementary file 1 [file ijms-22-03792-s001.zip › Supplementary_File/C_ GO_Analysis_Results/16-30nt_go_Makona-24h-Huh7_vs_Control-24h-Huh7_down.mature_mirna_targets/MF_result(Human).html]

| GO.ID | Term | Ontology | Count | Pop.Hits | List.Total | Pop.Total | Fold.Enrichment | Pvalue | FDR | Enrichment.Score | Gene.Ratio | GENES |
| --- | --- | --- | --- | --- | --- | --- | --- | --- | --- | --- | --- | --- |
| GO:0033218 | amide binding | Molecular function | 7 | 308 | 114 | 17548 | 3.49840510366826 | 0.00398512271003212 | 1 | 2.39955830125173 | 0.0614035087719298 | GRIA3//ITM2B//KDELR1//PC//MAS1//NPR3//HLA-DPA1// |
| GO:0016763 | transferase activity, transferring pentosyl groups | Molecular function | 3 | 52 | 114 | 17548 | 8.88056680161943 | 0.00468218727402406 | 1 | 2.32955121976184 | 0.0263157894736842 | MTAP//XYLT2//GXYLT1// |
| GO:0016667 | oxidoreductase activity, acting on a sulfur group of donors | Molecular function | 3 | 61 | 114 | 17548 | 7.57031924072476 | 0.00731016703390057 | 1 | 2.13607269950281 | 0.0263157894736842 | TXNDC8//TMX1//SRXN1// |
| GO:0042277 | peptide binding | Molecular function | 6 | 273 | 114 | 17548 | 3.38307306728359 | 0.00883964736794393 | 1 | 2.05356505955827 | 0.0526315789473684 | GRIA3//ITM2B//KDELR1//MAS1//NPR3//HLA-DPA1// |
| GO:0005385 | zinc ion transmembrane transporter activity | Molecular function | 2 | 22 | 114 | 17548 | 13.993620414673 | 0.00887764524185152 | 1 | 2.05170221373559 | 0.0175438596491228 | SLC30A8//SLC39A11// |
| GO:0072509 | divalent inorganic cation transmembrane transporter activity | Molecular function | 2 | 26 | 114 | 17548 | 11.8407557354926 | 0.0122811839717656 | 1 | 1.91075976286763 | 0.0175438596491228 | SLC30A8//SLC39A11// |
| GO:0035639 | purine ribonucleoside triphosphate binding | Molecular function | 20 | 1810 | 114 | 17548 | 1.70088203935252 | 0.0123579763317848 | 1 | 1.90805264088913 | 0.175438596491228 | UBE2E3//SRXN1//DNAH12//PAN3//ABL2//ATP11C//IARS//MAB21L1//PC//ATP6V1A//MAPK7//PSMC6//DDX55//MAP3K7//UBE2E1//ITM2B//KRAS//RAB2A//RIT2//GNA14// |
| GO:0061631 | ubiquitin conjugating enzyme activity | Molecular function | 2 | 27 | 114 | 17548 | 11.4022092267706 | 0.0132079098887667 | 1 | 1.87916590271807 | 0.0175438596491228 | UBE2E3//UBE2E1// |
| GO:0061650 | ubiquitin-like protein conjugating enzyme activity | Molecular function | 2 | 28 | 114 | 17548 | 10.9949874686717 | 0.0141641273293873 | 1 | 1.84881017777655 | 0.0175438596491228 | UBE2E3//UBE2E1// |
| GO:0003887 | DNA-directed DNA polymerase activity | Molecular function | 2 | 29 | 114 | 17548 | 10.615849969752 | 0.0151494210250093 | 1 | 1.81960396455101 | 0.0175438596491228 | POLI//POLH// |
| GO:0005520 | insulin-like growth factor binding | Molecular function | 2 | 29 | 114 | 17548 | 10.615849969752 | 0.0151494210250093 | 1 | 1.81960396455101 | 0.0175438596491228 | IGFBP5//IGFBP7// |
| GO:0032555 | purine ribonucleotide binding | Molecular function | 20 | 1854 | 114 | 17548 | 1.66051590681126 | 0.0157633488334974 | 1 | 1.80235151366052 | 0.175438596491228 | UBE2E3//SRXN1//DNAH12//PAN3//ABL2//ATP11C//IARS//MAB21L1//PC//ATP6V1A//MAPK7//PSMC6//DDX55//MAP3K7//UBE2E1//ITM2B//KRAS//RAB2A//RIT2//GNA14// |
| GO:0017076 | purine nucleotide binding | Molecular function | 20 | 1868 | 114 | 17548 | 1.64807092678162 | 0.0169909386607515 | 1 | 1.76978262796297 | 0.175438596491228 | UBE2E3//SRXN1//DNAH12//PAN3//ABL2//ATP11C//IARS//MAB21L1//PC//ATP6V1A//MAPK7//PSMC6//DDX55//MAP3K7//UBE2E1//ITM2B//KRAS//RAB2A//RIT2//GNA14// |
| GO:0032553 | ribonucleotide binding | Molecular function | 20 | 1869 | 114 | 17548 | 1.6471891338834 | 0.0170814408593506 | 1 | 1.76747549835004 | 0.175438596491228 | UBE2E3//SRXN1//DNAH12//PAN3//ABL2//ATP11C//IARS//MAB21L1//PC//ATP6V1A//MAPK7//PSMC6//DDX55//MAP3K7//UBE2E1//ITM2B//KRAS//RAB2A//RIT2//GNA14// |
| GO:0042056 | chemoattractant activity | Molecular function | 2 | 34 | 114 | 17548 | 9.0546955624355 | 0.0204977817404834 | 1 | 1.68829313552931 | 0.0175438596491228 | MIF//CCL16// |
| GO:0019208 | phosphatase regulator activity | Molecular function | 3 | 91 | 114 | 17548 | 5.07460960092539 | 0.0214581734413484 | 1 | 1.66840724873094 | 0.0263157894736842 | ANP32E//UBXN2B//PPP4R2// |
| GO:0046915 | transition metal ion transmembrane transporter activity | Molecular function | 2 | 40 | 114 | 17548 | 7.69649122807017 | 0.0277919211038393 | 1 | 1.55608143177751 | 0.0175438596491228 | SLC30A8//SLC39A11// |
| GO:0005524 | ATP binding | Molecular function | 16 | 1474 | 114 | 17548 | 1.6708800495132 | 0.0287560446872669 | 1 | 1.54127085016071 | 0.140350877192982 | UBE2E3//SRXN1//DNAH12//PAN3//ABL2//ATP11C//IARS//MAB21L1//PC//ATP6V1A//MAPK7//PSMC6//DDX55//MAP3K7//UBE2E1//ITM2B// |
| GO:0017046 | peptide hormone binding | Molecular function | 2 | 42 | 114 | 17548 | 7.32999164578112 | 0.0304224855190833 | 1 | 1.51680530694555 | 0.0175438596491228 | MAS1//NPR3// |
| GO:0034061 | DNA polymerase activity | Molecular function | 2 | 42 | 114 | 17548 | 7.32999164578112 | 0.0304224855190833 | 1 | 1.51680530694555 | 0.0175438596491228 | POLI//POLH// |
| GO:0015036 | disulfide oxidoreductase activity | Molecular function | 2 | 43 | 114 | 17548 | 7.15952672378621 | 0.0317734597788667 | 1 | 1.49793549272522 | 0.0175438596491228 | TXNDC8//TMX1// |
| GO:0016706 | oxidoreductase activity, acting on paired donors, with incorporation or reduction of molecular oxygen, 2-oxoglutarate as one donor, and incorporation of one atom each of oxygen into both donors | Molecular function | 2 | 45 | 114 | 17548 | 6.84132553606238 | 0.0345450035670997 | 1 | 1.46161475813506 | 0.0175438596491228 | ASPH//TET1// |
| GO:0032559 | adenyl ribonucleotide binding | Molecular function | 16 | 1510 | 114 | 17548 | 1.63104449866388 | 0.0348495387698903 | 1 | 1.45780296537134 | 0.140350877192982 | UBE2E3//SRXN1//DNAH12//PAN3//ABL2//ATP11C//IARS//MAB21L1//PC//ATP6V1A//MAPK7//PSMC6//DDX55//MAP3K7//UBE2E1//ITM2B// |
| GO:0030554 | adenyl nucleotide binding | Molecular function | 16 | 1522 | 114 | 17548 | 1.61818475228808 | 0.0370788862686325 | 1 | 1.43087331915136 | 0.140350877192982 | UBE2E3//SRXN1//DNAH12//PAN3//ABL2//ATP11C//IARS//MAB21L1//PC//ATP6V1A//MAPK7//PSMC6//DDX55//MAP3K7//UBE2E1//ITM2B// |
| GO:0016860 | intramolecular oxidoreductase activity | Molecular function | 2 | 49 | 114 | 17548 | 6.2828499820981 | 0.0403566785422504 | 1 | 1.39408458450974 | 0.0175438596491228 | TMX1//MIF// |
