# Supplementary material for: Altered microRNA Transcriptome in Cultured Human Liver Cells upon Infection with Ebola Virus
Source: Int J Mol Sci. 2021 Apr 6;22(7):3792. doi: 10.3390/ijms22073792 (PMC8038836; doi:10.3390/ijms22073792)
Supplement: Supplementary file 1 [file ijms-22-03792-s001.zip › Supplementary_File/C_ GO_Analysis_Results/16-30nt_go_Makona-24h-Huh7_vs_Control-24h-Huh7_up.mature_mirna_targets/BP_result(Human).html]

| GO.ID | Term | Ontology | Count | Pop.Hits | List.Total | Pop.Total | Fold.Enrichment | Pvalue | FDR | Enrichment.Score | Gene.Ratio | GENES |
| --- | --- | --- | --- | --- | --- | --- | --- | --- | --- | --- | --- | --- |
| GO:0006366 | transcription by RNA polymerase II | Biological process | 207 | 2405 | 902 | 17653 | 1.68448538936344 | 4.33137798386934e-15 | 2.92931093049083e-11 | 14.3633739152898 | 0.229490022172949 | TSHZ1//ZBTB18//CEBPB//TCERG1//STRAP//KLF12//GSC//ARX//DNMT3B//EDN1//EDNRB//EFNA1//CC2D1B//EN1//EP300//ESR1//JAZF1//CPEB3//FOXF1//MYT1L//SIN3B//SIRT1//ZFPM2//DNAJB5//GLI3//CNOT7//HIC1//FOXA1//HSBP1//ID2//IFNG//IRF2//AR//ISL1//JARID2//JUN//MAF//MDM4//MEF2A//MYB//NFIA//NFIB//NFIC//NR4A2//PAX5//KLF3//POU4F1//PPARG//ZBTB4//RB1//RBBP8//CCND1//RNF2//SARS//PRDM16//SMARCA2//SUV39H1//ZEB1//TFAP2A//TGIF1//KLF10//TNF//UBE2I//WT1//ZNF217//LRP8//BHLHE41//TBL1XR1//E2F8//ARID5B//BHLHE40//TP63//RUNX3//NOG//FOXP2//PHF14//GTF2E1//SRSF2//SRSF6//SLBP//CREBL2//ZNF367//ELK3//EMX2//ETS1//ETV5//ATF5//FOXF2//FLI1//ADNP//EPC2//HBP1//FOXP1//ATAD2//HIVEP2//HOXA3//HOXD1//BOLA3//NR3C2//MYBL1//MYCN//NEUROG1//NFE2//NHLH2//KLF13//ARID4B//BRWD1//POU3F2//MED9//TRERF1//FOXJ2//SLC2A4RG//MIER1//PURA//HIVEP3//RFX7//TRAK2//SMARCD2//SOX5//MED22//TXK//ZNF3//ZNF708//ZNF131//ZSCAN5A//FOSL1//LCOR//KLF7//CBFB//CLOCK//NR5A2//GTF2A1//GTF2H1//NRBP1//NRBF2//RORA//TEAD2//ELL2//CCNT2//TOB1//SMURF1//BMP3//BMPR2//GDF5//ACVR1//SULF1//LEMD3//CTDSPL2//INSIG2//ZMYND11//FLNA//MYOG//C5AR1//INTS8//NABP1//ADCYAP1//PPARGC1B//CREBRF//E2F5//S1PR1//MED19//KPNA6//AGO1//DLL1//TBK1//H2AFZ//NRG1//IGF1//IL2//IRF1//LRP6//MEOX2//MET//PPP1R12A//NCK1//NEUROD1//NPAS2//OTX2//IER5//ARMCX3//PKNOX1//CYTL1//EGLN1//PPP3R1//CCNL1//MAVS//RAF1//ATXN7//SOX4//SS18//TFE3//WNT7A//YES1//PCGF5//FUBP3//RPS6KA5//ST18//EGLN3//PSMA2//PSMD12//ZMYND8//TMBIM6//FBXW7//NPNT//BTF3//NMI//ZNF516// |
| GO:0006357 | regulation of transcription by RNA polymerase II | Biological process | 193 | 2227 | 902 | 17653 | 1.69609071095814 | 2.58309528632337e-14 | 8.73473671070248e-11 | 13.5878595730907 | 0.213968957871397 | TSHZ1//ZBTB18//CEBPB//TCERG1//STRAP//KLF12//GSC//ARX//DNMT3B//EDN1//EDNRB//EFNA1//CC2D1B//EN1//EP300//ESR1//JAZF1//CPEB3//FOXF1//MYT1L//SIN3B//SIRT1//ZFPM2//DNAJB5//GLI3//CNOT7//HIC1//FOXA1//HSBP1//ID2//IFNG//IRF2//AR//ISL1//JARID2//JUN//MAF//MDM4//MEF2A//MYB//NFIA//NFIB//NFIC//NR4A2//PAX5//KLF3//POU4F1//PPARG//ZBTB4//RB1//RBBP8//CCND1//RNF2//SARS//PRDM16//SMARCA2//SUV39H1//ZEB1//TFAP2A//TGIF1//KLF10//TNF//UBE2I//WT1//ZNF217//LRP8//BHLHE41//TBL1XR1//E2F8//ARID5B//BHLHE40//TP63//RUNX3//NOG//FOXP2//PHF14//TOB1//SMURF1//BMP3//BMPR2//GDF5//ACVR1//SULF1//LEMD3//CTDSPL2//INSIG2//ZMYND11//ADCYAP1//PPARGC1B//CREBRF//E2F5//S1PR1//ELK3//ETS1//ETV5//MED19//ATF5//FOXF2//FLI1//KPNA6//NR5A2//AGO1//DLL1//ATAD2//TBK1//GTF2H1//H2AFZ//NRG1//IGF1//IL2//IRF1//LRP6//MEOX2//MET//MYBL1//MYCN//MYOG//PPP1R12A//NCK1//NEUROD1//NEUROG1//NHLH2//NPAS2//OTX2//IER5//ARMCX3//KLF13//ARID4B//PKNOX1//CYTL1//POU3F2//EGLN1//PPP3R1//FOXJ2//CCNL1//MAVS//RAF1//RORA//ATXN7//SOX4//SS18//TFE3//TXK//WNT7A//YES1//FOSL1//PCGF5//TEAD2//KLF7//CBFB//FUBP3//CCNT2//RPS6KA5//CLOCK//ST18//EGLN3//PSMA2//PSMD12//ZMYND8//TMBIM6//FBXW7//NPNT//CREBL2//ZNF367//EMX2//ADNP//EPC2//HBP1//FOXP1//GTF2E1//HIVEP2//HOXA3//HOXD1//BOLA3//NR3C2//NFE2//BRWD1//MED9//TRERF1//SLC2A4RG//MIER1//PURA//HIVEP3//RFX7//TRAK2//SMARCD2//SOX5//MED22//ZNF3//ZNF708//ZNF131//ZSCAN5A//LCOR// |
| GO:0048468 | cell development | Biological process | 180 | 2045 | 902 | 17653 | 1.72262670837422 | 6.06287109784737e-14 | 1.36677324115806e-10 | 13.2173216651625 | 0.199556541019956 | EREG//YTHDF2//AGFG1//EDNRB//ISL1//SEMA4C//ACVR1//CCM2//CLIC4//MET//GLI3//FZD3//SULF1//BMPR2//ESR1//TP63//GJA1//PTHLH//DLL1//PALLD//AR//FRMD6//TGFBR2//CELF1//DZIP1//RNF2//BOLL//WT1//TDRD5//SMARCA2//QKI//SLITRK3//OGN//POU4F1//PRELP//WNT7A//SLIT2//RANBP9//ARX//DPYSL2//EFNA1//EFNB2//OTX2//PIK3CA//PIK3CB//ENAH//MAPK1//ROBO2//SCN1B//WNT3//KLF7//RPS6KA5//NRXN3//FEZ2//NOG//GSTM3//FRZB//PTEN//SNAP25//STMN2//ADCYAP1//NPTN//NCK1//SERPINI1//RAP1A//RAPGEF2//LPAR1//MYLIP//PTPRG//SPOCK1//TSC1//BAG5//ROR1//POU3F2//WASF3//ID2//RDH10//EDN1//TAPT1//NRG1//KBTBD8//IGF1//GNAQ//STRN//SYNGAP1//CD3E//SOX4//PLXNA4//ATF5//FLNA//NR4A2//HPRT1//NFIB//KCNJ10//PPP3R1//EP300//WDR1//TMOD3//TMOD1//RAB21//JUN//NREP//CAMSAP1//SAMD14//GPM6A//LRP12//PSD//RB1//UNC5B//VAMP3//CASP2//FLI1//FOXP1//CFL2//MYCN//NTF3//YWHAG//DDX6//DNMT3B//ETV5//MMD//FOXA1//NEUROD1//NEUROG1//ZEB1//GDF5//ADNP//TNFRSF12A//PPARG//CCL21//CBFB//INHBB//SDC1//EN1//MYT1L//FMR1//FXR1//ULK2//SLC9A6//TNFRSF21//MYOG//HOMER1//SGK1//MEF2A//TRAK2//HECW2//RUNX3//DBN1//CAMSAP2//GABRA5//MYB//PDE5A//YIPF6//ACSL4//ARC//DOCK10//CPEB3//ZMYND8//IL2//MAPK6//DLG5//LRP8//PPP1R16B//MSN//RAP1B//LDLR//RAP2C//ARID4B//NANOS1//CRKL//TNF//SMURF1//SYT1//PLAA//CLOCK//FBXW7//GJC1//MAF//BMP3//ARID5B// |
| GO:0031325 | positive regulation of cellular metabolic process | Biological process | 243 | 3074 | 902 | 17653 | 1.54708577916204 | 1.55218168840447e-13 | 2.62435118966986e-10 | 12.8090574443838 | 0.26940133037694 | GADD45A//TNF//CRKL//TAOK1//RAF1//RAP1A//C1QTNF2//LPAR1//NRG1//IGF1//NTF3//PIK3CB//PRKAA1//MAPK1//C5AR1//UBE2V1//EPS15//NSG1//VAMP3//EDNRB//EFNA1//FMR1//SIRT1//NPTN//IFNG//KDR//MAVS//CCND1//PRR5L//CCND2//EREG//FBXW7//EGLN3//F3//PMAIP1//PPARG//CASP2//EP300//COPS8//MDFIC//SOCS1//CCL21//TGFBR2//PPARGC1B//NPNT//CALM2//INHBB//BMP3//BMPR2//TTK//GDF5//ACVR1//CNEP1R1//LDLR//EDN1//ABHD5//MTMR9//ARPP19//CPEB3//SERP1//PRR16//SOX4//FXR1//RAB12//SESN1//TBK1//TSC1//CREBL2//ESR1//ETS1//ATF5//FOXF1//FOXF2//FLI1//PHF8//NR5A2//BAMBI//GLI3//ATAD2//ID2//IRF1//AR//JUN//LRP6//MYB//MYBL1//MYCN//NEUROD1//NFE2//NPAS2//NPAT//OTX2//TRERF1//RB1//HIVEP3//RORA//PRDM16//SMARCA2//SS18//TFAP2A//WNT7A//WT1//TBL1XR1//TEAD2//TP63//RUNX3//FUBP3//CLOCK//ZNF516//CXCL9//ADRB1//PTHLH//ADNP//NDFIP2//RAP2C//RASSF2//PIK3CA//PAK6//SLK//RICTOR//CCT6A//ANKIB1//LONRF3//LONRF1//SOCS5//PPP1R15B//MID1//SEMA4C//E2F8//INSIG2//RPS6KA5//PFN2//IFNA1//RWDD3//MET//ROR1//ADCY3//PRKACB//PRKAR2B//ISL1//SPPL3//PPP1R16B//PPP1R12A//NCK1//PTGES3//ITSN1//MOB1B//IL2//SOCS3//PPM1F//PDE5A//IGFBP3//CD3E//MID1IP1//ELOVL5//CCNY//CCNL1//CCNT2//ADCYAP1//CACUL1//MMD//RAPGEF2//ZBTB18//CEBPB//CREBRF//E2F5//S1PR1//ELK3//EN1//ETV5//MED19//ZFPM2//KPNA6//AGO1//DLL1//GTF2H1//CNOT7//H2AFZ//FOXA1//IRF2//MAF//MEF2A//MEOX2//MYOG//NEUROG1//NFIA//NFIB//NFIC//NHLH2//NR4A2//PAX5//IER5//ARMCX3//KLF13//ARID4B//PKNOX1//CYTL1//POU3F2//POU4F1//EGLN1//PPP3R1//FOXJ2//ATXN7//ZEB1//TFE3//KLF10//TXK//YES1//FOSL1//PCGF5//KLF7//CBFB//NOG//ST18//BOLL//HPRT1//LRP8//SH3D19//DCUN1D4//ARRDC3//DNMT3B//JARID2//TOB1//TNRC6B//GTPBP1//CNOT6L//YTHDF2//QKI//SMURF1//PTEN//CCL2//CCL8//GPR55//ACSL1//PPP2CA//NANOS1//NMI//RAB3GAP1//MSN//TNFAIP3//UBE2I//BAG6//FAM168A// |
| GO:0007275 | multicellular organism development | Biological process | 373 | 5351 | 902 | 17653 | 1.36422456212466 | 3.46003121894692e-13 | 4.6800382267476e-10 | 12.4609199826653 | 0.413525498891353 | WNT7A//HAPLN1//EN1//EVC//FRZB//SERP1//GNAQ//IGF1//PRELP//PTHLH//BMP3//SOX4//KLF10//TP63//NOG//RASSF2//MYCN//S1PR1//EFNA1//ELK3//EREG//UNC5B//SIRT1//LEMD3//CLIC4//HOXA3//JUN//KDR//RHOB//MEOX2//TNFRSF12A//PIK3CA//PKNOX1//PTEN//RORA//CCL2//VEGFB//CEBPB//ADCYAP1//INHBB//IMMP2L//ESR1//CASP2//CRKL//FOXF1//SPHK2//TGFBR2//RAPGEF2//EDN1//PPP3R1//ACVR1//GJC1//ZFPM2//RASA1//WT1//CCM2//TEAD2//QKI//RAB18//SMOC1//RDH10//GLI3//ID2//ROBO2//NPNT//SDC1//SLIT2//TAPT1//TANC2//GJA1//AR//MBNL1//BTF3//FOSL1//LRP6//RNF2//DUSP1//BMPR2//WNT3//EDNRB//ISL1//SEMA4C//EP300//DLL1//GPM6A//LRP12//NR4A2//SPOCK1//FZD3//SULF1//TFAP2A//TSC1//BAG6//ARID5B//RBBP8//FOXF2//FAM83D//DLG5//PRKACB//FZD6//JARID2//MET//PPARG//E2F8//STOX2//APCDD1//WNT10A//FBXW7//EFNB2//RECK//SNX19//NFIB//CYTL1//GDF5//RUNX3//ESCO2//KCNAB2//DOCK10//CD3E//ARID2//DHRS3//NRG1//POU4F1//KCNK2//SNX17//DCTN5//NEUROD1//PALLD//CXXC4//NEUROG1//GSC//MID1//ZEB1//SYNGAP1//E2F5//FLI1//PAX5//BHLHE41//FUT9//CHRM2//CNTFR//RBFOX3//DPYSL2//MYT1L//FMR1//GLRB//MYLIP//NTF3//POU3F2//TAOK1//PCDH10//PURA//SMARCA2//MPPED2//ZIC5//NAV3//FEZ2//SLITRK3//OGN//RANBP9//ARX//OTX2//PIK3CB//ENAH//MAPK1//SCN1B//KLF7//RPS6KA5//NRXN3//PCDHB10//BSN//NHLH2//NPAS2//HPCAL4//SERPINI1//FXR1//RAB3GAP1//PHF8//GABRA5//PTPRG//ATXN1//SLC6A11//SYT1//MEF2A//RAF1//ADIPOR2//SGCE//ZBTB18//CFL2//MYOG//GPCPD1//CCNT2//FOXP2//CCND1//GSTM3//RNF38//CSDE1//ATF5//MORC3//CELF1//RAI2//NR5A2//RICTOR//TSHZ1//EMX2//ARC//GRSF1//BAMBI//STRAP//FOXA1//PPP2CA//LDLRAD4//SNAP25//STMN2//NPTN//NCK1//RAP1A//LPAR1//BAG5//ROR1//WASF3//KBTBD8//STRN//SPRED1//AGO1//FOXJ2//SARS//C5AR1//LRP8//PLXNA4//HPRT1//CALM2//ETS1//FLNA//TACC2//PRDM16//TRAK2//KCNJ10//MSN//CBFB//ACSL4//HOXD1//RB1//CR2//IFNA1//DNAJB9//TGM3//IL2//TXK//WDR1//FOXP1//OSTM1//TNF//RAB21//TDRD5//IFNG//NREP//CAMSAP1//SAMD14//PSD//KRT80//KRTAP5-6//KRTAP2-4//TNFRSF21//MAF//SOX5//LRIG1//IGSF3//MGST1//HIVEP3//HSBP1//INSIG2//NFIC//SBF2//IRF1//BNC2//ARRDC3//ERRFI1//CD8A//HMGB3//SOCS1//BTG1//SERPINB13//SRSF6//MYB//SOCS5//KLF13//TNRC6B//NFE2//YWHAG//DDX6//DNMT3B//ETV5//MMD//TFE3//GPR55//PPARGC1B//TMEM64//EGLN1//VASH2//F3//ITGB8//ADAM12//ADNP//EDN2//PHF14//YTHDF2//PRKAA1//ZNF521//ULK2//SLC9A6//DBN1//SLC35D1//HOMER1//SGK1//HECW2//TMOD3//CAMSAP2//TNNI1//TUB//PRICKLE2//PSMA2//SMURF1//PSMD12//NFIA//SYBU//MFAP5//YIPF6//ZNF516//TBL1XR1//SOCS3//BTBD7//DNAJB6//GCNT4//CPEB3//ZMYND8//MAPK6//TNFAIP3//LDLR//TMOD1//KLHL3//ARID4B//NANOS1//NLN//PPP1R16B//PLAA//CLOCK//CFDP1//FAT3//EDARADD//DZIP1//WDR47//WDFY3//FLII//LCLAT1//CDH20//HIC1//HIVEP2//AGFG1//SHISA2//PI15//KLF3//ANO1//TRERF1//NXF2//BOLL//TGIF1//NXF2B//ZNF3//ZNF217//PTP4A1//SPATA9//ADAM18// |
| GO:0048856 | anatomical structure development | Biological process | 398 | 5836 | 902 | 17653 | 1.33468805137924 | 7.97084596929185e-13 | 8.98447188172013e-10 | 12.0984955832089 | 0.441241685144124 | WNT7A//VASH2//CDH20//NRG1//SS18//WNT3//HAPLN1//EN1//EVC//FRZB//SERP1//GNAQ//IGF1//PRELP//PTHLH//BMP3//SOX4//KLF10//TP63//NOG//RASSF2//MYCN//S1PR1//EFNA1//ELK3//EREG//UNC5B//SIRT1//LEMD3//CLIC4//HOXA3//JUN//KDR//RHOB//MEOX2//TNFRSF12A//PIK3CA//PKNOX1//PTEN//RORA//CCL2//VEGFB//CEBPB//ADCYAP1//INHBB//IMMP2L//ESR1//CASP2//YTHDF2//CRKL//FOXF1//SPHK2//TGFBR2//RAPGEF2//EDN1//PPP3R1//ACVR1//GJC1//ZFPM2//RASA1//WT1//CCM2//TEAD2//QKI//RAB18//SMOC1//RDH10//GLI3//ID2//ROBO2//NPNT//SDC1//SLIT2//AGFG1//TAPT1//TANC2//GJA1//AR//MBNL1//BTF3//FOSL1//LRP6//RNF2//DUSP1//BMPR2//FZD3//EDNRB//ISL1//SEMA4C//EP300//DLL1//GPM6A//LRP12//NR4A2//SPOCK1//SULF1//TFAP2A//TSC1//BAG6//ARID5B//RBBP8//FOXF2//FAM83D//DLG5//PRKACB//FZD6//MET//JARID2//PPARG//E2F8//STOX2//APCDD1//WNT10A//FBXW7//EFNB2//RECK//ARHGAP12//SNX19//NFIB//CYTL1//GDF5//RUNX3//ESCO2//KCNAB2//DOCK10//TNFAIP3//CD3E//ARID2//DHRS3//POU4F1//KCNK2//SNX17//DCTN5//NEUROD1//PALLD//CNTFR//FRMD6//TSHZ1//CFDP1//FAT3//EDARADD//DZIP1//WDR47//WDFY3//FLII//LCLAT1//TNFRSF21//HIC1//HIVEP2//HMGB3//SHISA2//MEF2A//NFE2//ROR1//PAX5//PI15//KLF3//ANO1//BTBD7//TRERF1//NXF2//RAF1//HIVEP3//BOLL//TGIF1//NXF2B//ZNF3//ZNF217//PTP4A1//SPATA9//ADAM18//ZNF516//CELF1//TDRD5//SMARCA2//RCAN3//PHLDA3//FOXP1//FOXA1//FOXJ2//FOXP2//CXXC4//NEUROG1//GSC//MID1//ZEB1//SYNGAP1//E2F5//FLI1//BHLHE41//NR5A2//SMURF1//FUT9//CHRM2//RBFOX3//DPYSL2//MYT1L//FMR1//GLRB//MYLIP//NTF3//POU3F2//TAOK1//PCDH10//PURA//MPPED2//ZIC5//NAV3//FEZ2//SLITRK3//OGN//RANBP9//ARX//OTX2//PIK3CB//ENAH//MAPK1//SCN1B//KLF7//RPS6KA5//NRXN3//PCDHB10//BSN//NHLH2//NPAS2//HPCAL4//SERPINI1//FXR1//RAB3GAP1//PHF8//GABRA5//PTPRG//ATXN1//SLC6A11//SYT1//ARC//PPP2CA//ADIPOR2//SGCE//ZBTB18//CFL2//MYOG//GPCPD1//CCNT2//ADAM12//CCND1//GSTM3//WASF3//PALM2//LPAR1//EPS8//BAMBI//ANXA7//RND3//MSN//BRWD1//PLXNA4//WDR1//ATP2C1//RNF38//CSDE1//ATF5//MORC3//RAI2//RICTOR//EMX2//GRSF1//STRAP//LDLRAD4//SNAP25//STMN2//NPTN//NCK1//RAP1A//BAG5//KBTBD8//STRN//SPRED1//AGO1//SARS//C5AR1//LRP8//HPRT1//CALM2//ETS1//FLNA//TACC2//PRDM16//TRAK2//KCNJ10//SH3D19//CAMSAP1//CBFB//ACSL4//HOXD1//RB1//CR2//IFNA1//DNAJB9//TGM3//IL2//TXK//TMOD3//TMOD1//OSTM1//TNF//RAB21//CNN3//GSTA2//IFNG//NREP//SAMD14//PSD//KRT80//KRTAP5-6//KRTAP2-4//MAF//SOX5//LRIG1//IGSF3//MGST1//VAMP3//HSBP1//INSIG2//NFIC//SBF2//IRF1//BNC2//ARRDC3//ERRFI1//TMEFF2//CD8A//SOCS1//BTG1//SERPINB13//SRSF6//MYB//SOCS5//KLF13//TNRC6B//IGFBP3//CXCL9//YWHAG//DDX6//DNMT3B//ETV5//MMD//TFE3//GPR55//PPARGC1B//TMEM64//EGLN1//F3//ITGB8//ADNP//EDN2//PHF14//CCL21//PRKAA1//ZNF521//ULK2//SLC9A6//DBN1//SLC35D1//GCNT4//HOMER1//SGK1//HECW2//CAMSAP2//BNIP2//TNNI1//TUB//PRICKLE2//PSMA2//PSMD12//NFIA//SYBU//MFAP5//PDE5A//YIPF6//TBL1XR1//SOCS3//DNAJB6//CPEB3//ZMYND8//MAPK6//PPP1R16B//RAP1B//LDLR//KLHL3//SNX2//MIEF1//RAP2C//ARID4B//NANOS1//NLN//PLAA//CLOCK// |
| GO:0051173 | positive regulation of nitrogen compound metabolic process | Biological process | 233 | 2965 | 902 | 17653 | 1.53795350785027 | 1.17198327818284e-12 | 1.13230327290722e-09 | 11.9310785847725 | 0.258314855875831 | GADD45A//TNF//CRKL//TAOK1//RAF1//RAP1A//C1QTNF2//LPAR1//NRG1//IGF1//NTF3//PIK3CB//PRKAA1//MAPK1//C5AR1//UBE2V1//EDNRB//EFNA1//FMR1//SIRT1//NPTN//IFNG//KDR//MAVS//CCND1//PRR5L//CCND2//EREG//FBXW7//EGLN3//F3//PMAIP1//PPARG//CASP2//EP300//COPS8//MDFIC//SOCS1//CCL21//CALM2//INHBB//BMP3//BMPR2//TTK//GDF5//ACVR1//CPEB3//SERP1//PRR16//SOX4//FXR1//CREBL2//ESR1//ETS1//ATF5//FOXF1//FOXF2//FLI1//PHF8//NR5A2//BAMBI//GLI3//ATAD2//ID2//IRF1//AR//JUN//LRP6//MYB//MYBL1//MYCN//NEUROD1//NFE2//NPAS2//NPAT//OTX2//TRERF1//RB1//HIVEP3//RORA//PRDM16//SMARCA2//SS18//TFAP2A//WNT7A//WT1//TBL1XR1//TEAD2//TP63//RUNX3//FUBP3//CLOCK//ZNF516//CXCL9//ADRB1//PTHLH//ADNP//NDFIP2//RAP2C//RASSF2//PIK3CA//PAK6//TGFBR2//SLK//RICTOR//CCT6A//ANKIB1//LONRF3//LONRF1//SOCS5//PPP1R15B//MID1//SEMA4C//E2F8//INSIG2//RPS6KA5//TBK1//PFN2//IFNA1//RWDD3//ADCY3//PRKACB//PRKAR2B//ISL1//SPPL3//CNEP1R1//PPP1R16B//PPP1R12A//NCK1//IL2//SOCS3//PPM1F//EDN1//PDE5A//IGFBP3//MET//ROR1//CD3E//GJA1//MYLIP//SMURF1//HECW2//TNFAIP3//CCNY//CCNL1//CCNT2//ADCYAP1//CACUL1//MMD//RAPGEF2//ZBTB18//CEBPB//PPARGC1B//CREBRF//E2F5//S1PR1//ELK3//EN1//ETV5//MED19//ZFPM2//KPNA6//AGO1//DLL1//GTF2H1//CNOT7//H2AFZ//FOXA1//IRF2//MAF//MEF2A//MEOX2//MYOG//NEUROG1//NFIA//NFIB//NFIC//NHLH2//NR4A2//PAX5//IER5//ARMCX3//KLF13//ARID4B//PKNOX1//CYTL1//POU3F2//POU4F1//EGLN1//PPP3R1//FOXJ2//ATXN7//ZEB1//TFE3//KLF10//TXK//YES1//FOSL1//PCGF5//KLF7//CBFB//NOG//ST18//BOLL//HPRT1//LRP8//SH3D19//DCUN1D4//ARRDC3//DNMT3B//JARID2//PTGES3//TOB1//TNRC6B//GTPBP1//CNOT6L//YTHDF2//QKI//NPNT//PTEN//CCL2//CCL8//GPR55//ACSL1//PPP2CA//NANOS1//NMI//RAB3GAP1//MSN//UBE2I//BAG6//FAM168A//LDLR// |
| GO:0009893 | positive regulation of metabolic process | Biological process | 253 | 3329 | 902 | 17653 | 1.48736894548279 | 3.37309098983179e-12 | 2.85152679552905e-09 | 11.4719719434687 | 0.280487804878049 | GADD45A//TNF//CRKL//TAOK1//RAF1//RAP1A//C1QTNF2//LPAR1//NRG1//IGF1//NTF3//PIK3CB//PRKAA1//MAPK1//C5AR1//UBE2V1//EPS15//NSG1//VAMP3//EDNRB//EFNA1//FMR1//SIRT1//NPTN//IFNG//KDR//MAVS//CCND1//PRR5L//CCND2//EREG//FBXW7//EGLN3//F3//PMAIP1//PPARG//CASP2//EP300//COPS8//MDFIC//SOCS1//CCL21//ARX//DNMT3B//ETS1//RAB3GAP1//GJA1//ID2//AR//ITGB8//LDLR//MSN//MYCN//POU4F1//PTEN//TFAP2A//WNT3//WNT10A//FUBP3//CD3E//NOG//QKI//PPM1F//TGFBR2//PPARGC1B//NPNT//CALM2//INHBB//BMP3//BMPR2//TTK//GDF5//ACVR1//CNEP1R1//EDN1//ABHD5//MTMR9//ARPP19//CPEB3//SERP1//PRR16//SOX4//FXR1//CREBL2//RAB12//SESN1//TBK1//TSC1//ESR1//ATF5//FOXF1//FOXF2//FLI1//PHF8//NR5A2//BAMBI//GLI3//ATAD2//IRF1//JUN//LRP6//MYB//MYBL1//NEUROD1//NFE2//NPAS2//NPAT//OTX2//TRERF1//RB1//HIVEP3//RORA//PRDM16//SMARCA2//SS18//WNT7A//WT1//TBL1XR1//TEAD2//TP63//RUNX3//CLOCK//ZNF516//CXCL9//ADRB1//PTHLH//ADNP//NDFIP2//RAP2C//RASSF2//PIK3CA//PAK6//SLK//RICTOR//CCT6A//ANKIB1//LONRF3//LONRF1//SOCS5//PPP1R15B//MID1//SEMA4C//E2F8//INSIG2//RPS6KA5//PFN2//IFNA1//RWDD3//MET//ROR1//ADCY3//PRKACB//PRKAR2B//ISL1//SPPL3//PPP1R16B//PPP1R12A//NCK1//PTGES3//ITSN1//MOB1B//IL2//SOCS3//PDE5A//IGFBP3//MID1IP1//ELOVL5//MYLIP//SMURF1//HECW2//TNFAIP3//CCNY//CCNL1//CCNT2//ADCYAP1//CACUL1//MMD//RAPGEF2//ZBTB18//CEBPB//CREBRF//E2F5//S1PR1//ELK3//EN1//ETV5//MED19//ZFPM2//KPNA6//AGO1//DLL1//GTF2H1//CNOT7//H2AFZ//FOXA1//IRF2//MAF//MEF2A//MEOX2//MYOG//NEUROG1//NFIA//NFIB//NFIC//NHLH2//NR4A2//PAX5//IER5//ARMCX3//KLF13//ARID4B//PKNOX1//CYTL1//POU3F2//EGLN1//PPP3R1//FOXJ2//ATXN7//ZEB1//TFE3//KLF10//TXK//YES1//FOSL1//PCGF5//KLF7//CBFB//ST18//BOLL//HPRT1//LRP8//SH3D19//DCUN1D4//ARRDC3//JARID2//CCL2//TOB1//TNRC6B//EDN2//GTPBP1//CNOT6L//YTHDF2//CCL8//GPR55//ACSL1//PPP2CA//NANOS1//NMI//UBE2I//BAG6//FAM168A//PYURF//PIGA// |
| GO:0048731 | system development | Biological process | 333 | 4760 | 902 | 17653 | 1.36914443160856 | 1.24317032592301e-11 | 9.34173434913035e-09 | 10.9054693648894 | 0.369179600886918 | HAPLN1//EN1//EVC//FRZB//SERP1//GNAQ//IGF1//PRELP//PTHLH//BMP3//SOX4//KLF10//TP63//NOG//RASSF2//MYCN//WNT7A//S1PR1//EFNA1//ELK3//EREG//UNC5B//SIRT1//LEMD3//CLIC4//HOXA3//JUN//KDR//RHOB//MEOX2//TNFRSF12A//PIK3CA//PKNOX1//PTEN//RORA//CCL2//VEGFB//CEBPB//ADCYAP1//INHBB//IMMP2L//ESR1//CASP2//CRKL//FOXF1//SPHK2//TGFBR2//RAPGEF2//EDN1//PPP3R1//ACVR1//GJC1//ZFPM2//RASA1//WT1//CCM2//TEAD2//QKI//RAB18//SMOC1//RDH10//GLI3//ID2//ROBO2//NPNT//SDC1//SLIT2//EDNRB//ISL1//SEMA4C//GJA1//GPM6A//LRP12//NR4A2//SPOCK1//FZD3//SULF1//TFAP2A//TSC1//BAG6//ARID5B//FOXF2//FAM83D//DLG5//LRP6//PRKACB//FZD6//JARID2//MET//PPARG//E2F8//STOX2//BMPR2//APCDD1//WNT10A//FBXW7//EFNB2//DLL1//RECK//SNX19//NFIB//CYTL1//GDF5//RUNX3//ESCO2//KCNAB2//DOCK10//CD3E//ARID2//DHRS3//NRG1//POU4F1//KCNK2//SNX17//DCTN5//NEUROD1//PALLD//E2F5//EP300//FLI1//PAX5//BHLHE41//FUT9//CHRM2//CNTFR//RBFOX3//DPYSL2//MYT1L//FMR1//GLRB//MYLIP//MBNL1//NEUROG1//NTF3//POU3F2//TAOK1//PCDH10//PURA//SMARCA2//MPPED2//ZIC5//NAV3//FEZ2//SLITRK3//OGN//RANBP9//ARX//OTX2//PIK3CB//ENAH//MAPK1//SCN1B//WNT3//KLF7//RPS6KA5//NRXN3//PCDHB10//BSN//NHLH2//NPAS2//HPCAL4//SERPINI1//ZEB1//FXR1//RAB3GAP1//PHF8//GABRA5//PTPRG//ATXN1//SLC6A11//SYT1//MEF2A//RAF1//ADIPOR2//SGCE//ZBTB18//CFL2//MYOG//GPCPD1//CCNT2//FOXP2//CCND1//GSTM3//RNF38//CSDE1//BAMBI//STRAP//FOXA1//PPP2CA//LDLRAD4//SNAP25//STMN2//NPTN//NCK1//RAP1A//LPAR1//BAG5//ROR1//WASF3//KBTBD8//TAPT1//GSC//STRN//SYNGAP1//SPRED1//AGO1//FOXJ2//SARS//C5AR1//LRP8//EMX2//PLXNA4//HPRT1//CALM2//ETS1//ATF5//FLNA//TACC2//PRDM16//TRAK2//KCNJ10//MSN//CBFB//ACSL4//HOXD1//RB1//CR2//IFNA1//DNAJB9//TGM3//IL2//TXK//WDR1//FOXP1//OSTM1//TNF//RAB21//AR//NREP//CAMSAP1//SAMD14//PSD//KRT80//KRTAP5-6//KRTAP2-4//TNFRSF21//MAF//SOX5//LRIG1//IGSF3//IFNG//MGST1//HIVEP3//INSIG2//TSHZ1//NFIC//SBF2//IRF1//BNC2//ARRDC3//ERRFI1//CD8A//HMGB3//SOCS1//SERPINB13//SRSF6//MYB//SOCS5//KLF13//TNRC6B//NFE2//YWHAG//DDX6//DNMT3B//ETV5//MMD//TFE3//GPR55//PPARGC1B//TMEM64//EGLN1//VASH2//F3//ITGB8//BTG1//ADAM12//ADNP//EDN2//PHF14//PRKAA1//ZNF521//ULK2//SLC9A6//DBN1//SLC35D1//HOMER1//SGK1//HECW2//TMOD3//CAMSAP2//TNNI1//TUB//PRICKLE2//PSMA2//SMURF1//PSMD12//NFIA//SYBU//MFAP5//YIPF6//ZNF516//TBL1XR1//SOCS3//FOSL1//BTBD7//DNAJB6//GCNT4//ARC//CPEB3//ZMYND8//MAPK6//TNFAIP3//NR5A2//LDLR//TMOD1//KLHL3//ARID4B//NANOS1//YTHDF2//NLN//PPP1R16B//PLAA//CLOCK// |
| GO:0045944 | positive regulation of transcription by RNA polymerase II | Biological process | 111 | 1132 | 902 | 17653 | 1.91905992180706 | 1.5570979151262e-11 | 9.76680735863925e-09 | 10.8076840767948 | 0.123059866962306 | EP300//INSIG2//ESR1//JUN//CEBPB//FBXW7//NPNT//ZBTB18//ADCYAP1//PPARGC1B//CREBRF//E2F5//S1PR1//EDN1//ELK3//EN1//ETS1//ETV5//MED19//ATF5//FOXF1//FOXF2//FLI1//SIRT1//ZFPM2//KPNA6//NR5A2//AGO1//GLI3//DLL1//ATAD2//TBK1//GTF2H1//CNOT7//H2AFZ//NRG1//FOXA1//IGF1//IL2//IRF1//IRF2//AR//ISL1//LRP6//MAF//MEF2A//MEOX2//MET//MYB//MYBL1//MYCN//MYOG//PPP1R12A//NCK1//NEUROD1//NEUROG1//NFIA//NFIB//NFIC//NHLH2//NPAS2//NR4A2//OTX2//PAX5//IER5//ARMCX3//KLF13//ARID4B//PKNOX1//CYTL1//POU3F2//POU4F1//EGLN1//PPARG//PPP3R1//FOXJ2//CCNL1//MAVS//RAF1//RB1//RORA//ATXN7//PRDM16//BMPR2//SMARCA2//SOX4//SS18//ZEB1//TFAP2A//TFE3//KLF10//TNF//TXK//WNT7A//WT1//YES1//TBL1XR1//E2F8//FOSL1//PCGF5//TEAD2//KLF7//TP63//CBFB//FUBP3//ACVR1//CCNT2//NOG//RPS6KA5//CLOCK//ST18// |
| GO:0050793 | regulation of developmental process | Biological process | 204 | 2563 | 902 | 17653 | 1.55773488143139 | 1.61021691649933e-11 | 9.76680735863925e-09 | 10.7931156150905 | 0.226164079822616 | GLI3//FZD3//TNFAIP3//TGFBR2//CD3E//WT1//BMPR2//ISL1//CFDP1//WASF3//PALM2//LPAR1//EPS8//BAMBI//ANXA7//KDR//RHOB//RND3//MSN//BRWD1//RASA1//CCL2//PLXNA4//WDR1//STRAP//EFNA1//FOXA1//PPP2CA//PTEN//LDLRAD4//FRZB//PPARG//MYOG//BHLHE41//SNAP25//STMN2//ADCYAP1//NPTN//NCK1//SERPINI1//RAP1A//SCN1B//RAPGEF2//EFNB2//MYLIP//PTPRG//SPOCK1//TSC1//BAG5//EDNRB//DLL1//SPRED1//SULF1//AGO1//FOXJ2//SARS//SH3D19//CAMSAP1//ARC//S1PR1//GJA1//TFAP2A//ACVR1//DPYSL2//RAB21//IFNG//CCND1//WNT7A//TNFRSF21//NRG1//MAF//PTHLH//GDF5//SOX5//ZEB1//IGF1//PIK3CA//ADRB1//POU3F2//OTX2//EDN1//WNT10A//HMGB3//ID2//IL2//CBFB//IRF1//SOCS1//RAF1//RUNX3//MAPK1//AR//JUN//NEUROD1//SOCS3//DLK2//SIRT1//ZFPM2//RORA//TNF//CEBPB//CREBL2//TMEM64//BTG1//MYCN//SERPINB13//ERRFI1//SRSF6//TP63//MYB//SOCS5//GNAQ//KLF13//ETS1//RB1//EP300//TNRC6B//NFE2//FOXP1//IGFBP3//CXCL9//NTF3//YWHAG//NREP//DDX6//DNMT3B//ETV5//MMD//NEUROG1//SMOC1//RASSF2//TOB1//NOG//NPNT//TFE3//GPR55//PPARGC1B//POU4F1//KLF10//TNFRSF12A//EGLN1//VASH2//F3//ITGB8//C5AR1//VEGFB//ADAM12//ADNP//ARX//SERP1//PRKAA1//ULK2//WNT3//FMR1//HECW2//FXR1//SEMA4C//TRAK2//SYNGAP1//ROBO2//SLIT2//DBN1//CAMSAP2//MEF2A//BNIP2//FOXF1//EREG//SLITRK3//DLG5//JARID2//KCNK2//PRICKLE2//ROR1//PSMA2//SMURF1//PSMD12//FZD6//PDE5A//FOXP2//ESR1//BTBD7//CPEB3//ZMYND8//MAPK6//LRP8//TAPT1//LDLR//MEOX2//MIEF1//PRDM16//CRKL//FLNA//PRKACB//YTHDF2//NLN//PPP1R16B//SYT1//PLAA//LRP6//CLOCK//FBXW7//TEAD2//NFIB//PHF14// |
| GO:0031323 | regulation of cellular metabolic process | Biological process | 425 | 6454 | 902 | 17653 | 1.28875971655454 | 1.73298370994634e-11 | 9.76680735863925e-09 | 10.7612055196367 | 0.471175166297118 | GADD45A//GTF2H1//PTEN//CCNT2//DIRAS3//NPAT//TSHZ1//ZBTB18//CEBPB//TCERG1//STRAP//KLF12//GSC//ARX//DNMT3B//EDN1//EDNRB//EFNA1//CC2D1B//EN1//EP300//ESR1//JAZF1//CPEB3//FOXF1//MYT1L//SIN3B//SIRT1//ZFPM2//DNAJB5//GLI3//CNOT7//HIC1//FOXA1//HSBP1//ID2//IFNG//IRF2//AR//ISL1//JARID2//JUN//MAF//MDM4//MEF2A//MYB//NFIA//NFIB//NFIC//NR4A2//PAX5//KLF3//POU4F1//PPARG//ZBTB4//RB1//RBBP8//CCND1//RNF2//SARS//PRDM16//SMARCA2//SUV39H1//ZEB1//TFAP2A//TGIF1//KLF10//TNF//UBE2I//WT1//ZNF217//LRP8//BHLHE41//TBL1XR1//E2F8//ARID5B//BHLHE40//TP63//RUNX3//NOG//FOXP2//PHF14//CRKL//TAOK1//RAF1//RAP1A//C1QTNF2//LPAR1//NRG1//IGF1//NTF3//PIK3CB//PRKAA1//MAPK1//C5AR1//UBE2V1//SPRED1//DUSP1//PPP2CA//MBNL2//RBFOX3//FMR1//MBNL1//RBM25//SRSF2//SRSF6//SEPSECS//SECISBP2L//EPS15//NSG1//VAMP3//SOCS1//IGFBP3//IL2//LRP6//PRR5L//PPP1R15B//SLIT2//NPTN//KDR//MAVS//CCND2//EREG//FBXW7//ERRFI1//GPRC5A//SOCS5//NUP54//NLN//ESCO2//BAHD1//H2AFZ//HIPK3//ZMYND11//PNRC1//ZNF526//PPARGC1B//ZNF800//ARID2//ELAVL2//ATF5//ZFP30//ELL2//KDM2A//MYCBP2//FLII//ZMYND8//CNOT6L//NR5A2//ZNF521//ZBTB11//GTF2A1//GTF2E1//NRBF2//HIVEP2//HMGB3//ZNF680//ZNF662//AFF1//MLLT6//OGG1//ASCC1//PHF20L1//COMMD10//BNC2//ZNF532//SLC2A4RG//PAK6//PHTF2//CNOT6//ZBTB26//HIVEP3//RORA//SOX4//BTF3//BTG1//ZNF3//ZNF131//ZNF227//CSDE1//ZNF655//SAP30L//SNIP1//ANP32A//TEAD2//RPS6KA5//CLOCK//ZNF516//CREBL2//ZNF367//ELK3//EMX2//ETS1//ETV5//FOXF2//FLI1//ADNP//EPC2//HBP1//FOXP1//ATAD2//HOXA3//HOXD1//BOLA3//NR3C2//MYBL1//MYCN//NEUROG1//NFE2//NHLH2//KLF13//ARID4B//BRWD1//POU3F2//MED9//TRERF1//FOXJ2//MIER1//PURA//RFX7//TRAK2//SMARCD2//SOX5//MED22//TXK//ZNF708//ZSCAN5A//FOSL1//LCOR//KLF7//CBFB//BDP1//DDX6//TSC1//KBTBD8//GNAQ//NCK1//GSKIP//YWHAG//SOCS3//PPM1F//PSMA2//PSMD12//EGLN3//F3//PMAIP1//CASP2//GRM3//COPS8//G3BP2//MDFIC//AGO1//PI15//DRAM1//RRAGD//ULK2//MET//MTMR9//CCL21//PPP4R2//CRHR1//TGFBR2//NPNT//CALM2//INHBB//BMP3//BMPR2//TTK//GDF5//ACVR1//CNEP1R1//LDLR//ABHD5//FKBP15//PPP1R12A//PLEKHF2//SERPINI1//SERPINB13//SPOCK1//RECK//ARPP19//SERP1//PRR16//FXR1//RAB12//SESN1//TBK1//SNX5//PIK3CA//FLNA//PHF8//BAMBI//IRF1//NEUROD1//NPAS2//OTX2//SS18//WNT7A//FUBP3//DNAJB6//RASD1//ATXN1//BTAF1//TOB1//CELF1//NANOS1//TIA1//MEX3B//SMURF1//SULF1//LEMD3//CTDSPL2//CXCL9//ADRB1//PTHLH//EGLN1//PDE5A//SPOPL//TNFAIP3//BAG5//NDFIP2//MOB1B//RAP2C//RASSF2//RWDD3//SLK//RICTOR//DCP2//CCT6A//BAG6//ANKIB1//LONRF3//LONRF1//PPP1R1B//MYLIP//MID1//SEMA4C//INSIG2//PFN2//IFNA1//ROR1//ADCY3//PRKACB//PRKAR2B//TNRC6B//PPP1R16B//SPPL3//ADIPOR2//ATXN7//PTGES3//ITSN1//ARL6IP1//SYNGAP1//FZD6//FASTK//YTHDF2//RCAN3//UBXN2B//PPP2R5E//CD3E//NMI//MID1IP1//ELOVL5//CCNY//CCNL1//MYOG//ADCYAP1//CACUL1//MMD//RAPGEF2//CREBRF//E2F5//S1PR1//MED19//KPNA6//DLL1//MEOX2//IER5//ARMCX3//PKNOX1//CYTL1//PPP3R1//TFE3//YES1//PCGF5//ST18//PAIP2//BOLL//HPRT1//GRIK2//SH3D19//C8ORF44-SGK3//SGK3//SGK1//RHEBL1//IRAK4//NOVA1//RASA1//ZER1//DCUN1D4//ARRDC3//PMEPA1//LDLRAD4//TMBIM6//GTPBP1//QKI//FAM83D//RANBP9//CCL2//CCL8//GPR55//ACSL1//CAST//PLAA//CPEB2//CRTAP//FEZ2//RAB3GAP1//MSN//HSBP1L1//FAM168A//GJA1//KCNK2//CPEB1//TBC1D12// |
| GO:0045595 | regulation of cell differentiation | Biological process | 153 | 1761 | 902 | 17653 | 1.7003724451059 | 2.01603497995627e-11 | 1.04880342841879e-08 | 10.695501936775 | 0.169623059866962 | GLI3//FZD3//BAMBI//ISL1//TGFBR2//STRAP//EFNA1//FOXA1//PPP2CA//PTEN//LDLRAD4//FRZB//PPARG//MYOG//BHLHE41//SNAP25//STMN2//ADCYAP1//NPTN//NCK1//SERPINI1//RAP1A//SCN1B//RAPGEF2//LPAR1//EFNB2//MYLIP//PTPRG//SPOCK1//TSC1//BAG5//EDNRB//DPYSL2//RAB21//DLL1//IFNG//CCND1//WNT7A//MAF//PTHLH//GDF5//SOX5//ZEB1//HMGB3//ID2//IL2//CBFB//IRF1//SOCS1//MAPK1//AR//JUN//NEUROD1//SOCS3//DLK2//SIRT1//ZFPM2//RORA//TNF//CEBPB//CREBL2//TMEM64//BTG1//MYCN//SERPINB13//ERRFI1//SRSF6//TP63//MYB//SOCS5//GNAQ//KLF13//ETS1//RB1//EP300//TNRC6B//AGO1//NFE2//FOXP1//IGFBP3//CXCL9//NTF3//YWHAG//NREP//DDX6//DNMT3B//ETV5//MMD//NEUROG1//SMOC1//RASSF2//TOB1//NOG//NPNT//GJA1//IGF1//BMPR2//ACVR1//TFE3//GPR55//PPARGC1B//POU4F1//KLF10//ADNP//NRG1//TNFRSF12A//ULK2//WNT3//TNFRSF21//WDR1//FMR1//HECW2//FXR1//PLXNA4//SEMA4C//POU3F2//TRAK2//SYNGAP1//ROBO2//SLIT2//DBN1//CAMSAP2//MEF2A//BNIP2//FOXF1//EREG//PDE5A//EDN1//CPEB3//ZMYND8//MAPK6//DLG5//ARC//LRP8//LDLR//PRDM16//FOXJ2//CRKL//FLNA//YTHDF2//PSMA2//PSMD12//NLN//SMURF1//SYT1//PLAA//CLOCK//TEAD2//FBXW7//KDR//RAF1//TFAP2A//RUNX3// |
| GO:0009653 | anatomical structure morphogenesis | Biological process | 209 | 2656 | 902 | 17653 | 1.54003361004996 | 2.42000515369531e-11 | 1.16903534674581e-08 | 10.6161837091357 | 0.231707317073171 | VASH2//CDH20//NRG1//SS18//WNT3//MYCN//WNT7A//S1PR1//EFNA1//ELK3//EREG//UNC5B//SIRT1//LEMD3//CLIC4//HOXA3//JUN//KDR//RHOB//MEOX2//TNFRSF12A//PIK3CA//PKNOX1//PTEN//RORA//CCL2//VEGFB//EDN1//PPP3R1//TGFBR2//ACVR1//GJC1//FOXF1//ZFPM2//RASA1//WT1//CCM2//TEAD2//QKI//NPNT//GLI3//AGFG1//LRP6//RNF2//DUSP1//NOG//BMPR2//FZD3//TP63//EP300//DLL1//SOX4//SEMA4C//PRKACB//TSC1//FZD6//MET//GJA1//ARHGAP12//E2F8//EFNB2//SLIT2//TNFAIP3//ARID2//ISL1//ID2//DHRS3//POU4F1//PALLD//AR//FRMD6//TFAP2A//GSC//CRKL//E2F5//FLI1//PAX5//BHLHE41//SLITRK3//OGN//PRELP//RANBP9//ARX//DPYSL2//OTX2//PIK3CB//ENAH//MAPK1//ROBO2//SCN1B//KLF7//RPS6KA5//NRXN3//FEZ2//ADAM12//CFDP1//WASF3//PALM2//LPAR1//EPS8//BAMBI//ANXA7//RND3//MSN//BRWD1//PLXNA4//WDR1//FOXA1//KBTBD8//GRSF1//SPRED1//SULF1//AGO1//PPARG//FOXJ2//SARS//NEUROG1//FLNA//NR4A2//NFIB//SH3D19//CAMSAP1//ARC//TMOD3//TMOD1//MBNL1//GDF5//RAB21//DLG5//TGM3//WNT10A//LRIG1//VAMP3//RDH10//EN1//RECK//HSBP1//FOXF2//INSIG2//TSHZ1//NFIC//SDC1//GNAQ//ERRFI1//TMEFF2//CFL2//ETS1//EGLN1//F3//ITGB8//BTG1//C5AR1//ADNP//NEUROD1//ZEB1//YTHDF2//SERP1//ARID5B//RB1//ULK2//SLC9A6//GCNT4//GPM6A//NTF3//SGK1//HPRT1//MEF2A//TRAK2//FMR1//HECW2//FXR1//POU3F2//SYNGAP1//RAPGEF2//TNNI1//FRZB//PRICKLE2//ROR1//PSMA2//SMURF1//PSMD12//RAB3GAP1//PPARGC1B//SRSF6//FOXP2//ESR1//TNF//SOCS3//BTBD7//DNAJB6//ETV5//DOCK10//LRP8//ADIPOR2//NR5A2//KLHL3//CEBPB//SNX2//MIEF1//FOXP1//MYOG//CXCL9//PPP1R16B//SYT1//PLAA//FBXW7//RAP1A//RCAN3//PHLDA3// |
| GO:0010604 | positive regulation of macromolecule metabolic process | Biological process | 236 | 3106 | 902 | 17653 | 1.48703960434207 | 2.64664562261737e-11 | 1.19328428971742e-08 | 10.5773042054478 | 0.261640798226164 | GADD45A//TNF//CRKL//TAOK1//RAF1//RAP1A//C1QTNF2//LPAR1//NRG1//IGF1//NTF3//PIK3CB//PRKAA1//MAPK1//C5AR1//UBE2V1//EPS15//NSG1//VAMP3//EDNRB//EFNA1//FMR1//SIRT1//NPTN//IFNG//KDR//MAVS//CCND1//PRR5L//CCND2//EREG//FBXW7//EGLN3//F3//PMAIP1//PPARG//CASP2//EP300//COPS8//MDFIC//SOCS1//CCL21//ARX//DNMT3B//ETS1//RAB3GAP1//GJA1//ID2//AR//ITGB8//LDLR//MSN//MYCN//POU4F1//PTEN//TFAP2A//WNT3//WNT10A//FUBP3//CD3E//NOG//QKI//PPM1F//CALM2//INHBB//BMP3//BMPR2//TTK//GDF5//ACVR1//EDN1//CPEB3//SERP1//PRR16//SOX4//FXR1//CREBL2//ESR1//ATF5//FOXF1//FOXF2//FLI1//PHF8//NR5A2//BAMBI//GLI3//ATAD2//IRF1//JUN//LRP6//MYB//MYBL1//NEUROD1//NFE2//NPAS2//NPAT//OTX2//TRERF1//RB1//HIVEP3//RORA//PRDM16//SMARCA2//SS18//WNT7A//WT1//TBL1XR1//TEAD2//TP63//RUNX3//CLOCK//ZNF516//NDFIP2//RAP2C//RASSF2//ADNP//PIK3CA//PAK6//TGFBR2//SLK//RICTOR//CCT6A//ANKIB1//LONRF3//LONRF1//SOCS5//PPP1R15B//MID1//SEMA4C//E2F8//INSIG2//RPS6KA5//TBK1//PFN2//IFNA1//RWDD3//ADCY3//PRKACB//PRKAR2B//ISL1//SPPL3//CNEP1R1//PPP1R16B//PPP1R12A//NCK1//IL2//SOCS3//PDE5A//IGFBP3//MET//ROR1//MYLIP//SMURF1//HECW2//TNFAIP3//CCNY//CCNL1//CCNT2//ADCYAP1//CACUL1//MMD//RAPGEF2//ZBTB18//CEBPB//PPARGC1B//CREBRF//E2F5//S1PR1//ELK3//EN1//ETV5//MED19//ZFPM2//KPNA6//AGO1//DLL1//GTF2H1//CNOT7//H2AFZ//FOXA1//IRF2//MAF//MEF2A//MEOX2//MYOG//NEUROG1//NFIA//NFIB//NFIC//NHLH2//NR4A2//PAX5//IER5//ARMCX3//KLF13//ARID4B//PKNOX1//CYTL1//POU3F2//EGLN1//PPP3R1//FOXJ2//ATXN7//ZEB1//TFE3//KLF10//TXK//YES1//FOSL1//PCGF5//KLF7//CBFB//ST18//BOLL//LRP8//SH3D19//DCUN1D4//ARRDC3//JARID2//CCL2//PTGES3//TOB1//TNRC6B//GTPBP1//CNOT6L//YTHDF2//NPNT//CCL8//GPR55//ACSL1//PPP2CA//NANOS1//NMI//UBE2I//BAG6//FAM168A// |
| GO:0048519 | negative regulation of biological process | Biological process | 361 | 5303 | 902 | 17653 | 1.3322862890227 | 3.5977660646378e-11 | 1.52073074344659e-08 | 10.4439670789393 | 0.400221729490022 | USP28//CLOCK//TSHZ1//ZBTB18//CEBPB//TCERG1//STRAP//KLF12//GSC//ARX//DNMT3B//EDN1//EDNRB//EFNA1//CC2D1B//EN1//EP300//ESR1//JAZF1//CPEB3//FOXF1//MYT1L//SIN3B//SIRT1//ZFPM2//DNAJB5//GLI3//CNOT7//HIC1//FOXA1//HSBP1//ID2//IFNG//IRF2//AR//ISL1//JARID2//JUN//MAF//MDM4//MEF2A//MYB//NFIA//NFIB//NFIC//NR4A2//PAX5//KLF3//POU4F1//PPARG//ZBTB4//RB1//RBBP8//CCND1//RNF2//SARS//PRDM16//SMARCA2//SUV39H1//ZEB1//TFAP2A//TGIF1//KLF10//TNF//UBE2I//WT1//ZNF217//LRP8//BHLHE41//TBL1XR1//E2F8//ARID5B//BHLHE40//TP63//RUNX3//NOG//FOXP2//PHF14//PNRC1//DCP2//PPP2CA//SPRED1//DUSP1//CNOT6L//PAN3//CNOT6//PATL1//AGO1//RPS6KA5//IGFBP3//IL2//LRP6//PTEN//PRR5L//PPP1R15B//SLIT2//SULF1//GJA1//RASA1//TNFAIP3//ADCYAP1//FOXP1//BMPR2//EPS8//ERRFI1//GPRC5A//SOCS5//BAHD1//H2AFZ//GADD45A//GNAQ//NCK1//GSKIP//YWHAG//SOCS1//SOCS3//PPM1F//SOX4//CASP2//STMN2//CAMSAP1//CAMSAP2//MID1//MID1IP1//NAV3//HBP1//IRF1//PRKAA1//RRAGD//TSC1//CUL5//NABP1//TTK//TAOK1//CBLL1//PLXNA4//GRM3//G3BP2//HIPK3//ATF5//FLNA//ARL6IP1//KLHL20//IGF1//KDR//MET//ROR1//OGG1//ZFAND6//SPHK2//RAF1//BCL2A1//BNIP2//TMBIM6//WNT7A//BAG6//CCND2//TOB1//COPS8//ARID2//EREG//ETS1//FRZB//TES//DLL1//MYOG//KLF13//PTHLH//STRN//BTG1//FOSL1//SKAP2//DLG5//RAPGEF2//ST18//HS3ST5//RGS7BP//ACVR1//SNIP1//PI15//MTMR9//MYBL1//ADNP//TBK1//ANXA7//ITGB8//LDLR//MYCN//POU3F2//NDFIP2//CD3E//PFN2//PTPRG//LDLRAD4//C5ORF30//CALM2//SPOCK1//EFNB2//FBXW7//ABHD5//INSIG2//FKBP15//PPP1R12A//PLEKHF2//SERPINI1//SERPINB13//RECK//PSMA2//PSMD12//SKP1//LPAR1//MYLIP//BAG5//PMEPA1//PIK3CA//CELF1//IQGAP2//CPEB2//FOXJ2//DNAJB6//PPARGC1B//ELK3//FOXF2//MDFIC//NRG1//RASD1//PURA//ATXN1//BTAF1//INHBB//FMR1//NANOS1//TIA1//FXR1//MEX3B//APCDD1//SHISA2//CXXC4//SMURF1//ADIPOR2//EI24//ZMYND8//TMEFF2//CLIC4//RHOB//RAP2C//TSN//LEMD3//FAM89B//BAMBI//TGFBR2//CTDSPL2//EGLN1//TNFRSF21//TNRC6B//SPOPL//ARRDC3//ATAD2//MIER1//RWDD3//SLC6A1//GDF5//PCBP2//MAVS//ARPP19//PPP1R1B//CD2AP//RAP1A//RASSF2//CCL2//PPP1R16B//ADRB1//EPS15//PDE5A//ATXN7//ZMYND11//RORA//PXK//FZD6//YTHDF2//ANP32A//CNTFR//UNC5B//GABRA5//GRIK2//NTF3//ITSN1//C5AR1//SYNGAP1//DDX6//NMI//HMGB3//MAPK1//SRSF6//TMEM64//GPR55//DLK2//RNF167//PAIP2//FZD3//NEUROD1//PPP2CB//KCNB1//MORC3//DHRS3//OGN//ULK2//SEMA4C//WNT3//TRAK2//ROBO2//ARHGAP12//CCL21//CXCL9//PRKACB//S1PR1//ARHGAP6//PPFIA1//TMOD3//TMOD1//OTUD3//PHLDA3//KCNK2//NPAS2//TEAD2//VPS4B//NUP54//GTPBP1//QKI//PHF8//RANBP9//CCL8//BOLL//CSDE1//CDC14A//MEOX2//ANAPC15//KCNE1//CAST//PLAA//CREBRF//NEUROG1//CRHR1//CRTAP//CHMP3//ABHD17C//FEZ2//HSBP1L1//NPAT//SRSF2//VAMP3//DNAJB9//SESN1//SESTD1//ZNF655//CFDP1//LYPD6//PRKAR2B//HECW2//CPEB1//ARHGAP1// |
| GO:0009888 | tissue development | Biological process | 165 | 1969 | 902 | 17653 | 1.64002403101735 | 4.95134200697542e-11 | 1.96976035253969e-08 | 10.3052770743528 | 0.182926829268293 | MYCN//WNT7A//EDN1//PPP3R1//TGFBR2//ACVR1//NPNT//ROBO2//SDC1//WT1//SLIT2//GLI3//DUSP1//NOG//BMPR2//WNT3//FZD3//TP63//EDNRB//ISL1//SEMA4C//EP300//FOXF1//DLL1//MEOX2//FOXF2//FAM83D//DLG5//SOX4//LRP6//PRKACB//TSC1//FZD6//TEAD2//CCM2//CLIC4//MET//APCDD1//WNT10A//GJA1//ARHGAP12//SNX19//NFIB//CYTL1//GDF5//RUNX3//SULF1//ESR1//KDR//NRG1//ID2//EFNA1//ZFPM2//POU4F1//S1PR1//NEUROD1//PALLD//AR//FRMD6//TFAP2A//SMURF1//ARC//PPP2CA//ZBTB18//CFL2//MYOG//GPCPD1//CCNT2//FOXP2//GSTM3//ATP2C1//POU3F2//PTHLH//BAMBI//STRAP//FOXA1//PTEN//LDLRAD4//KBTBD8//RDH10//TAPT1//FRZB//GSC//IGF1//GRSF1//EREG//TGM3//KLF10//CNN3//NR5A2//GSTA2//ANXA7//PPARG//IFNG//CCND1//EN1//KRT80//KRTAP5-6//KRTAP2-4//MAF//SOX5//CEBPB//MAPK1//JUN//PAX5//RB1//HIVEP3//HSBP1//TXK//WDR1//FLNA//TMEFF2//MSN//ZEB1//BTG1//SERPINB13//ERRFI1//CBFB//SRSF6//PRKAA1//GCNT4//GJC1//HOMER1//EVC//HOXA3//ITGB8//BMP3//TNF//MEF2A//EGLN1//TNNI1//ARID2//JARID2//KCNK2//PRICKLE2//ROR1//PSMA2//PSMD12//BNC2//YIPF6//PIK3CA//ARID5B//ZNF516//ARRDC3//TBL1XR1//SOCS3//BTBD7//ETV5//PPP1R16B//RAP1A//RAP1B//RAPGEF2//RHOB//TMOD1//E2F8//ARX//KLHL3//EFNB2//RAP2C//ARID4B//FOXJ2//FOXP1//NLN//SIRT1//CLOCK// |
| GO:0048522 | positive regulation of cellular process | Biological process | 351 | 5142 | 902 | 17653 | 1.33594022876688 | 6.08740864623207e-11 | 2.28717470413708e-08 | 10.2155675431876 | 0.389135254988914 | GADD45A//TNF//CRKL//TAOK1//RAF1//RAP1A//C1QTNF2//LPAR1//NRG1//IGF1//NTF3//PIK3CB//PRKAA1//MAPK1//C5AR1//UBE2V1//EPS15//NSG1//VAMP3//EDNRB//EFNA1//FMR1//SIRT1//NPTN//IFNG//KDR//MAVS//CCND1//PRR5L//CCND2//F3//RICTOR//PPP1R16B//JUN//BMPR2//VEGFB//VASH2//GLI3//FZD3//FOXF1//MYCN//TGFBR2//TP63//FOXP2//CCL8//EDN2//CXCL9//EREG//FBXW7//GTF2H1//EGLN3//PMAIP1//PPARG//CASP2//EP300//CNOT6L//CNOT7//MDM4//CNOT6//SOX4//E2F8//COPS8//MDFIC//DNAJA2//ADCYAP1//CNTFR//CACUL1//EDN1//EFNB2//ETS1//BAMBI//DLL1//HOXA3//IL2//AR//ISL1//POU3F2//SPHK2//PTEN//PTHLH//PURA//TTK//FOSL1//TOB1//EPS8//ARHGAP1//LASP1//ARHGAP6//SH3BGRL//SKAP2//HOMER1//TRPC3//SOCS1//CCL21//FOXP1//RHOB//WNT7A//PAN3//PPM1F//GJA1//PPARGC1B//NPNT//KCNB1//CALM2//MYOG//INHBB//BMP3//GDF5//ACVR1//CNEP1R1//LDLR//ABHD5//MTMR9//KCNK2//VPS4B//STMN2//NCK1//SERPINI1//SCN1B//RAPGEF2//UNC5B//ARPP19//CPEB3//SERP1//PRR16//FXR1//RAB12//SESN1//TBK1//TSC1//CREBL2//ESR1//ATF5//FOXF2//FLI1//PHF8//NR5A2//ATAD2//ID2//IRF1//LRP6//MYB//MYBL1//NEUROD1//NFE2//NPAS2//NPAT//OTX2//TRERF1//RB1//HIVEP3//RORA//PRDM16//SMARCA2//SS18//TFAP2A//WT1//TBL1XR1//TEAD2//RUNX3//FUBP3//CLOCK//ZNF516//SULF1//WNT3//S1PR1//FAM89B//SEMA4C//PTP4A1//CBLL1//ADRB1//ADNP//CFL2//WDR1//PFN2//MET//CLIP1//NAV3//PIK3CA//YES1//CD3E//SYT1//C2CD5//RAB8B//NDFIP2//NEUROG1//WASF3//MIER1//RAP2C//RASSF2//RRAGD//PAK6//SLK//CCT6A//SOX5//ANKIB1//LONRF3//LONRF1//SOCS5//PPP1R15B//MID1//INSIG2//RPS6KA5//IFNA1//FOXA1//RWDD3//ROR1//ADCY3//PRKACB//PRKAR2B//IQGAP2//ARFIP1//GPR55//SPPL3//DLG5//PPP1R12A//ANO1//SYBU//PTGES3//ITSN1//MOB1B//SOCS3//DUSP1//CDK19//PHLDA3//FRZB//IGFBP3//USP27X//ARHGEF4//TNFRSF12A//POU4F1//BCL2A1//ZMAT3//SOS2//SLIT2//FLNA//ATP2C1//IRAK4//UBE2I//PDE5A//SPRED1//HIC1//GRIK2//CEBPB//TMEM64//BTG1//DNMT3B//ETV5//MMD//ZEB1//KLF10//MID1IP1//ELOVL5//TAPT1//CCNY//CCNL1//CCNT2//EVC//ZBTB18//CREBRF//E2F5//ELK3//EN1//MED19//ZFPM2//KPNA6//AGO1//H2AFZ//IRF2//MAF//MEF2A//MEOX2//NFIA//NFIB//NFIC//NHLH2//NR4A2//PAX5//IER5//ARMCX3//KLF13//ARID4B//PKNOX1//CYTL1//EGLN1//PPP3R1//FOXJ2//ATXN7//TFE3//TXK//PCGF5//KLF7//CBFB//NOG//ST18//BOLL//HPRT1//RASGEF1A//RAB21//LRP8//SIRPA//TUB//ROBO2//PLXNA4//RIMS4//CCL2//SH3D19//BNIP2//DCUN1D4//ARRDC3//ZMYND8//GPM6A//JARID2//SLITRK3//CHRNB4//TNRC6B//ARC//SNAP25//SRSF6//MAPK6//GTPBP1//YTHDF2//QKI//RAB3GAP1//SMURF1//MSN//ACSL1//PPP2CA//MIEF1//GSKIP//PSMA2//PSMD12//NANOS1//PAIP2//DYNLL2//YWHAG//KCNE1//KCNJ2//TMOD3//SERINC3//NMI//FAM83D//SEPT9//TNFAIP3//SDC1//PLAA//NMUR1//SNIP1//TIA1//BAG6//FAM168A// |
| GO:0048523 | negative regulation of cellular process | Biological process | 333 | 4821 | 902 | 17653 | 1.35182067920696 | 6.56137923682459e-11 | 2.32967072005452e-08 | 10.1830048600174 | 0.369179600886918 | USP28//CLOCK//TSHZ1//ZBTB18//CEBPB//TCERG1//STRAP//KLF12//GSC//ARX//DNMT3B//EDN1//EDNRB//EFNA1//CC2D1B//EN1//EP300//ESR1//JAZF1//CPEB3//FOXF1//MYT1L//SIN3B//SIRT1//ZFPM2//DNAJB5//GLI3//CNOT7//HIC1//FOXA1//HSBP1//ID2//IFNG//IRF2//AR//ISL1//JARID2//JUN//MAF//MDM4//MEF2A//MYB//NFIA//NFIB//NFIC//NR4A2//PAX5//KLF3//POU4F1//PPARG//ZBTB4//RB1//RBBP8//CCND1//RNF2//SARS//PRDM16//SMARCA2//SUV39H1//ZEB1//TFAP2A//TGIF1//KLF10//TNF//UBE2I//WT1//ZNF217//LRP8//BHLHE41//TBL1XR1//E2F8//ARID5B//BHLHE40//TP63//RUNX3//NOG//FOXP2//PHF14//SPRED1//DUSP1//PPP2CA//IGFBP3//IL2//LRP6//PTEN//PRR5L//PPP1R15B//SLIT2//SULF1//GJA1//RASA1//FOXP1//BMPR2//EPS8//ERRFI1//GPRC5A//SOCS5//BAHD1//H2AFZ//GADD45A//GNAQ//NCK1//GSKIP//YWHAG//SOCS1//SOCS3//PPM1F//CNOT6L//CNOT6//SOX4//CASP2//STMN2//CAMSAP1//CAMSAP2//MID1//MID1IP1//NAV3//HBP1//IRF1//PRKAA1//RRAGD//TSC1//CUL5//NABP1//TTK//TAOK1//CBLL1//PLXNA4//GRM3//HIPK3//ATF5//FLNA//ARL6IP1//KLHL20//IGF1//KDR//MET//ROR1//OGG1//ZFAND6//SPHK2//RAF1//BCL2A1//BNIP2//TMBIM6//WNT7A//BAG6//CCND2//TOB1//COPS8//ARID2//EREG//ETS1//FRZB//TES//DLL1//MYOG//KLF13//PTHLH//STRN//BTG1//FOSL1//SKAP2//DLG5//RAPGEF2//ST18//RGS7BP//ACVR1//AGO1//SNIP1//PI15//MTMR9//MYBL1//PFN2//PTPRG//ADCYAP1//MYCN//LDLRAD4//C5ORF30//CALM2//SPOCK1//EFNB2//FBXW7//FKBP15//PPP1R12A//PLEKHF2//SERPINI1//SERPINB13//RECK//PSMA2//PSMD12//SKP1//LPAR1//MYLIP//BAG5//PMEPA1//PIK3CA//DNAJB6//PPARGC1B//ELK3//FOXF2//MDFIC//NRG1//RASD1//PURA//ATXN1//BTAF1//RPS6KA5//INHBB//CELF1//FMR1//NANOS1//TIA1//FXR1//MEX3B//APCDD1//SHISA2//CXXC4//ADIPOR2//EI24//ZMYND8//TMEFF2//CLIC4//RHOB//RAP2C//LEMD3//FAM89B//BAMBI//SMURF1//TGFBR2//CTDSPL2//EGLN1//TNFRSF21//SPOPL//TNFAIP3//ATAD2//MIER1//DCP2//SLC6A1//GDF5//ARPP19//PPP1R1B//RASSF2//CCL2//TNRC6B//PPP1R16B//EPS15//PDE5A//ATXN7//ZMYND11//RORA//CNTFR//UNC5B//ADNP//GABRA5//GRIK2//NTF3//ITSN1//C5AR1//SYNGAP1//NMI//HMGB3//MAPK1//SRSF6//DDX6//TMEM64//GPR55//INSIG2//YTHDF2//DLK2//RNF167//CD3E//PAIP2//FZD3//NEUROD1//PPP2CB//KCNB1//MORC3//DHRS3//OGN//ULK2//SEMA4C//WNT3//TRAK2//CD2AP//ARHGAP12//CCL21//CXCL9//PRKACB//S1PR1//ARHGAP6//PPFIA1//TMOD3//TMOD1//OTUD3//PHLDA3//ROBO2//KCNK2//NPAS2//TEAD2//VPS4B//NUP54//QKI//PHF8//LDLR//RANBP9//CCL8//BOLL//CDC14A//MEOX2//FZD6//ANAPC15//CAST//FOXJ2//PLAA//CREBRF//CPEB2//CRTAP//KCNE1//FEZ2//HSBP1L1//NPAT//SRSF2//VAMP3//DNAJB9//SESN1//ZNF655//CFDP1//LYPD6//RAP1A//PRKAR2B//CPEB1//ARHGAP1// |
| GO:0032502 | developmental process | Biological process | 412 | 6267 | 902 | 17653 | 1.28661765054484 | 6.88945947081035e-11 | 2.32967072005452e-08 | 10.1618148503787 | 0.456762749445676 | WNT7A//VASH2//CDH20//NRG1//SS18//WNT3//TP63//HAPLN1//EN1//EVC//FRZB//SERP1//GNAQ//IGF1//PRELP//PTHLH//BMP3//SOX4//KLF10//NOG//RASSF2//MYCN//S1PR1//EFNA1//ELK3//EREG//UNC5B//SIRT1//LEMD3//CLIC4//HOXA3//JUN//KDR//RHOB//MEOX2//TNFRSF12A//PIK3CA//PKNOX1//PTEN//RORA//CCL2//VEGFB//CEBPB//ADCYAP1//INHBB//IMMP2L//ESR1//CASP2//YTHDF2//CRKL//FOXF1//SPHK2//TGFBR2//RAPGEF2//EDN1//PPP3R1//ACVR1//GJC1//ZFPM2//RASA1//WT1//CCM2//TEAD2//QKI//GJA1//IGFBP3//RDH14//CBFB//RAB18//SMOC1//RDH10//GLI3//ID2//ROBO2//NPNT//SDC1//SLIT2//AGFG1//TAPT1//TANC2//AR//MBNL1//BTF3//FOSL1//LRP6//RNF2//DUSP1//BMPR2//DLL1//FZD3//EDNRB//ISL1//SEMA4C//EP300//GPM6A//LRP12//NR4A2//SPOCK1//SULF1//TFAP2A//TSC1//BAG6//ARID5B//RBBP8//FOXF2//FAM83D//DLG5//PRKACB//FZD6//MET//JARID2//PPARG//E2F8//STOX2//APCDD1//WNT10A//FBXW7//EFNB2//RECK//ARHGAP12//SNX19//NFIB//CYTL1//GDF5//RUNX3//ESCO2//KCNAB2//DOCK10//TNFAIP3//CD3E//ARID2//DHRS3//POU4F1//KCNK2//SNX17//DCTN5//NEUROD1//PALLD//CNTFR//FRMD6//BNC2//TSHZ1//CFDP1//FAT3//EDARADD//DZIP1//WDR47//WDFY3//FLII//LCLAT1//TNFRSF21//HIC1//HIVEP2//HMGB3//SHISA2//MEF2A//NFE2//ROR1//PAX5//PI15//KLF3//ANO1//BTBD7//TRERF1//NXF2//RAF1//HIVEP3//BOLL//TGIF1//NXF2B//ZNF3//ZNF217//PTP4A1//SPATA9//ADAM18//ZNF516//CELF1//TDRD5//SMARCA2//RCAN3//PHLDA3//FOXP1//FOXA1//FOXJ2//FOXP2//CXXC4//NEUROG1//GSC//MID1//ZEB1//SYNGAP1//E2F5//FLI1//BHLHE41//NR5A2//SMURF1//FUT9//CHRM2//RBFOX3//DPYSL2//MYT1L//FMR1//GLRB//MYLIP//NTF3//POU3F2//TAOK1//PCDH10//PURA//MPPED2//ZIC5//NAV3//FEZ2//SLITRK3//OGN//RANBP9//ARX//OTX2//PIK3CB//ENAH//MAPK1//SCN1B//KLF7//RPS6KA5//NRXN3//PCDHB10//BSN//NHLH2//NPAS2//HPCAL4//SERPINI1//FXR1//RAB3GAP1//PHF8//GABRA5//PTPRG//ATXN1//SLC6A11//SYT1//ARC//PPP2CA//ADIPOR2//SGCE//ZBTB18//CFL2//MYOG//GPCPD1//CCNT2//ADAM12//OGG1//MORC3//CCND1//GSTM3//WASF3//PALM2//LPAR1//EPS8//BAMBI//ANXA7//RND3//MSN//BRWD1//PLXNA4//WDR1//ATP2C1//RNF38//CSDE1//ATF5//RAI2//RICTOR//EMX2//GRSF1//STRAP//LDLRAD4//SNAP25//STMN2//NPTN//NCK1//RAP1A//BAG5//KBTBD8//STRN//SPRED1//AGO1//SARS//DDX6//C5AR1//LRP8//HPRT1//CALM2//ETS1//FLNA//TACC2//PRDM16//TRAK2//KCNJ10//SH3D19//CAMSAP1//CREBL2//ETV5//MYB//MYBL1//ABHD5//RB1//SUV39H1//TXK//UBE2V1//YES1//ACSL4//HOXD1//CR2//IFNA1//DNAJB9//TGM3//IL2//TMOD3//TMOD1//OSTM1//TNF//RAB21//CNN3//GSTA2//IFNG//NREP//SAMD14//PSD//KRT80//KRTAP5-6//KRTAP2-4//MAF//SOX5//LRIG1//IGSF3//MGST1//VAMP3//TBL1XR1//HSBP1//ADRB1//INSIG2//NFIC//SBF2//IRF1//ARRDC3//ERRFI1//TMEFF2//TMEM64//CD8A//BBS9//SOCS1//SOCS3//DLK2//BTG1//SERPINB13//SRSF6//SOCS5//KLF13//TNRC6B//CXCL9//YWHAG//DNMT3B//MMD//TOB1//TFE3//GPR55//PPARGC1B//EGLN1//F3//ITGB8//ADNP//EDN2//PHF14//CCL21//PRKAA1//ZNF521//ULK2//SLC9A6//DBN1//SLC35D1//GCNT4//HOMER1//SGK1//HECW2//CAMSAP2//BNIP2//TNNI1//TUB//PRICKLE2//PSMA2//PSMD12//NFIA//SYBU//MFAP5//PDE5A//YIPF6//DNAJB6//CPEB3//ZMYND8//MAPK6//PPP1R16B//RAP1B//LDLR//KLHL3//SNX2//MIEF1//RAP2C//ARID4B//NANOS1//NLN//PLAA//CLOCK//MBNL2//RBM41// |
| GO:0051252 | regulation of RNA metabolic process | Biological process | 285 | 3984 | 902 | 17653 | 1.40003055459087 | 8.93032749246453e-11 | 2.8759907062637e-08 | 10.0491326143968 | 0.315964523281596 | NPAT//TSHZ1//ZBTB18//CEBPB//TCERG1//STRAP//KLF12//GSC//ARX//DNMT3B//EDN1//EDNRB//EFNA1//CC2D1B//EN1//EP300//ESR1//JAZF1//CPEB3//FOXF1//MYT1L//SIN3B//SIRT1//ZFPM2//DNAJB5//GLI3//CNOT7//HIC1//FOXA1//HSBP1//ID2//IFNG//IRF2//AR//ISL1//JARID2//JUN//MAF//MDM4//MEF2A//MYB//NFIA//NFIB//NFIC//NR4A2//PAX5//KLF3//POU4F1//PPARG//ZBTB4//RB1//RBBP8//CCND1//RNF2//SARS//PRDM16//SMARCA2//SUV39H1//ZEB1//TFAP2A//TGIF1//KLF10//TNF//UBE2I//WT1//ZNF217//LRP8//BHLHE41//TBL1XR1//E2F8//ARID5B//BHLHE40//TP63//RUNX3//NOG//FOXP2//PHF14//MBNL2//RBFOX3//FMR1//MBNL1//RBM25//SRSF2//SRSF6//BAHD1//H2AFZ//HIPK3//ZMYND11//PNRC1//ZNF526//PPARGC1B//ZNF800//ARID2//ELAVL2//ATF5//ZFP30//ELL2//KDM2A//MYCBP2//FLII//ZMYND8//CNOT6L//NR5A2//ZNF521//ZBTB11//GTF2A1//GTF2E1//NRBF2//HIVEP2//HMGB3//ZNF680//ZNF662//AFF1//MLLT6//OGG1//ASCC1//PHF20L1//COMMD10//BNC2//PPP2CA//ZNF532//SLC2A4RG//PAK6//PHTF2//CNOT6//ZBTB26//HIVEP3//RORA//SOX4//BTF3//BTG1//UBE2V1//ZNF3//ZNF131//ZNF227//CSDE1//ZNF655//SAP30L//SNIP1//ANP32A//TEAD2//RPS6KA5//CLOCK//ZNF516//CREBL2//ZNF367//ELK3//EMX2//ETS1//ETV5//FOXF2//FLI1//ADNP//EPC2//HBP1//FOXP1//ATAD2//HOXA3//HOXD1//BOLA3//NR3C2//MYBL1//MYCN//NEUROG1//NFE2//NHLH2//KLF13//ARID4B//BRWD1//POU3F2//MED9//TRERF1//FOXJ2//MIER1//PURA//RFX7//TRAK2//SMARCD2//SOX5//MED22//TXK//ZNF708//ZSCAN5A//FOSL1//LCOR//KLF7//CBFB//BDP1//G3BP2//FLNA//PHF8//BAMBI//MDFIC//IGF1//IRF1//LRP6//NEUROD1//NPAS2//OTX2//MAPK1//SS18//WNT7A//FUBP3//ACVR1//DNAJB6//EREG//NRG1//RASD1//ATXN1//BTAF1//PPM1F//TOB1//SMURF1//BMP3//BMPR2//GDF5//SULF1//LEMD3//CTDSPL2//RWDD3//TNFAIP3//INSIG2//EGLN1//FZD6//CELF1//FASTK//DCP2//YTHDF2//PSMA2//PSMD12//FXR1//ADCYAP1//CREBRF//E2F5//S1PR1//MED19//KPNA6//AGO1//DLL1//TBK1//GTF2H1//IL2//MEOX2//MET//MYOG//PPP1R12A//NCK1//IER5//ARMCX3//PKNOX1//CYTL1//PPP3R1//CCNL1//MAVS//RAF1//ATXN7//TFE3//YES1//PCGF5//CCNT2//ST18//TIA1//C8ORF44-SGK3//SGK3//SGK1//PTEN//RHEBL1//ROR1//IRAK4//TNRC6B//TMBIM6//PRR5L//GTPBP1//QKI//EGLN3//BOLL//NANOS1//PRKAA1//RAP2C//NMI//HSBP1L1//FBXW7//NPNT//NOVA1//RASA1// |
| GO:2000026 | regulation of multicellular organismal development | Biological process | 166 | 2008 | 902 | 17653 | 1.61791746539342 | 1.2378677049675e-10 | 3.71547756547095e-08 | 9.90732576732938 | 0.184035476718404 | GLI3//FZD3//TGFBR2//CD3E//WT1//BMPR2//ISL1//BAMBI//STRAP//EFNA1//FOXA1//PPP2CA//PTEN//LDLRAD4//SNAP25//STMN2//ADCYAP1//NPTN//NCK1//SERPINI1//RAP1A//SCN1B//RAPGEF2//LPAR1//EFNB2//MYLIP//PTPRG//SPOCK1//TSC1//BAG5//EDNRB//DLL1//SPRED1//SULF1//AGO1//PPARG//FOXJ2//SARS//S1PR1//GJA1//TFAP2A//ACVR1//DPYSL2//RAB21//IFNG//CCND1//WNT7A//TNFRSF21//WASF3//NRG1//MAF//PTHLH//GDF5//SOX5//ZEB1//OTX2//EDN1//WNT10A//HMGB3//ID2//IL2//CBFB//IRF1//SOCS1//BTG1//MYCN//SERPINB13//ERRFI1//SRSF6//TP63//MYB//SOCS5//KLF13//ETS1//RB1//EP300//TNRC6B//NFE2//FOXP1//JUN//NTF3//YWHAG//NREP//DDX6//DNMT3B//ETV5//MMD//NEUROD1//NEUROG1//CEBPB//TFE3//RASSF2//GPR55//PPARGC1B//TMEM64//POU4F1//KLF10//TNF//TNFRSF12A//EGLN1//VASH2//F3//SIRT1//ITGB8//KDR//RHOB//C5AR1//VEGFB//ADAM12//ADNP//PRKAA1//ULK2//WNT3//NOG//WDR1//MYOG//FMR1//HECW2//FXR1//PLXNA4//SEMA4C//POU3F2//TRAK2//SYNGAP1//ROBO2//SLIT2//DBN1//CAMSAP2//SLITRK3//DLG5//JARID2//KCNK2//ZFPM2//PRICKLE2//ROR1//PSMA2//SMURF1//PSMD12//FZD6//FOXP2//AR//ESR1//BTBD7//CPEB3//ZMYND8//MAPK6//ARC//LRP8//TAPT1//FRZB//TNFAIP3//IGF1//LDLR//MEOX2//PRKACB//YTHDF2//NLN//PPP1R16B//SYT1//PLAA//LRP6//CLOCK//FBXW7//NFIB//PHF14//FLNA// |
| GO:0007399 | nervous system development | Biological process | 184 | 2296 | 902 | 17653 | 1.56840393396015 | 1.26358101442898e-10 | 3.71547756547095e-08 | 9.89839690808398 | 0.203991130820399 | GJA1//GPM6A//LRP12//NR4A2//SPOCK1//FZD3//RAPGEF2//SOX4//LRP6//SEMA4C//PRKACB//TSC1//FZD6//TEAD2//NOG//GLI3//SLITRK3//OGN//POU4F1//PRELP//WNT7A//SLIT2//RANBP9//ARX//DPYSL2//EFNA1//EFNB2//OTX2//PIK3CA//PIK3CB//ENAH//MAPK1//ROBO2//SCN1B//WNT3//KLF7//RPS6KA5//NRXN3//FEZ2//NRG1//PCDHB10//PTEN//BSN//HAPLN1//FMR1//JARID2//NHLH2//NPAS2//HPCAL4//SERPINI1//ZEB1//FXR1//S1PR1//RAB3GAP1//RAB18//PHF8//GABRA5//SPHK2//PTPRG//ATXN1//SLC6A11//BMPR2//SYT1//TGFBR2//BAG6//CASP2//IMMP2L//EDNRB//NTF3//GSTM3//NFIB//SNAP25//STMN2//ADCYAP1//NPTN//NCK1//RAP1A//LPAR1//MYLIP//BAG5//DLL1//ROR1//POU3F2//WASF3//ID2//GNAQ//STRN//SYNGAP1//CD3E//ISL1//C5AR1//LRP8//EMX2//NEUROD1//EN1//FOXP2//NEUROG1//TFAP2A//PLXNA4//HOXA3//PAX5//RORA//HPRT1//CALM2//ETS1//ATF5//GSC//FOXA1//FLNA//TACC2//PRDM16//TRAK2//KCNJ10//PPP3R1//CEBPB//ACSL4//MYT1L//HOXD1//RB1//WNT10A//RUNX3//RAB21//DLG5//JUN//NREP//CAMSAP1//SAMD14//PSD//TNFRSF21//UNC5B//SBF2//QKI//APCDD1//CCL2//MYCN//YWHAG//DDX6//DNMT3B//ETV5//MMD//GDF5//ADNP//TNFRSF12A//TP63//ZNF521//ULK2//SLC9A6//DBN1//WDR1//PPARG//SGK1//MEF2A//HECW2//CAMSAP2//SOX5//NFIA//SYBU//MYB//SULF1//LRIG1//ARC//DOCK10//CPEB3//ZMYND8//IL2//MAPK6//LDLR//NANOS1//SMURF1//PLAA//FUT9//CHRM2//CNTFR//RBFOX3//EP300//GLRB//MBNL1//TAOK1//PCDH10//PURA//SMARCA2//MPPED2//ZIC5//NAV3// |
| GO:0030154 | cell differentiation | Biological process | 292 | 4124 | 902 | 17653 | 1.38572221230545 | 1.46780101924844e-10 | 4.1361409554905e-08 | 9.83333281506 | 0.323725055432373 | EREG//YTHDF2//GJC1//FOXF1//ZFPM2//KDR//RASA1//TGFBR2//WT1//CCM2//TEAD2//QKI//GJA1//IGFBP3//RDH14//BMP3//CBFB//NOG//AGFG1//DLL1//EDNRB//ISL1//SEMA4C//ACVR1//GPM6A//LRP12//NR4A2//SPOCK1//FZD3//RAPGEF2//ID2//FOXF2//PPP3R1//FAM83D//DLG5//CLIC4//MET//GLI3//SNX19//NFIB//CYTL1//WNT7A//GDF5//RUNX3//SULF1//BMPR2//ESR1//TP63//PTHLH//ESCO2//KCNAB2//DOCK10//SOX4//EFNA1//NEUROD1//PALLD//AR//FRMD6//CELF1//DZIP1//RNF2//BOLL//TDRD5//SMARCA2//SLITRK3//OGN//POU4F1//PRELP//SLIT2//RANBP9//ARX//DPYSL2//EFNB2//OTX2//PIK3CA//PIK3CB//ENAH//MAPK1//ROBO2//SCN1B//WNT3//KLF7//RPS6KA5//NRXN3//FEZ2//ADAM12//GSTM3//BAMBI//STRAP//FOXA1//PPP2CA//PTEN//LDLRAD4//FRZB//EP300//PPARG//MYOG//BHLHE41//SNAP25//STMN2//ADCYAP1//NPTN//NCK1//SERPINI1//RAP1A//LPAR1//MYLIP//PTPRG//TSC1//BAG5//ROR1//POU3F2//WASF3//LRP6//KBTBD8//RDH10//EDN1//TAPT1//NRG1//WNT10A//GSC//IGF1//GNAQ//STRN//SYNGAP1//CD3E//RORA//PLXNA4//ATF5//HPRT1//FLNA//PRDM16//TRAK2//KCNJ10//CEBPB//S1PR1//EMX2//ACSL4//MYT1L//HOXD1//NEUROG1//RB1//CR2//IFNA1//DNAJB9//TGM3//IL2//PKNOX1//TXK//WDR1//JUN//SIRT1//TMOD3//TMOD1//FOXP1//OSTM1//TNF//RAB21//CNN3//NR5A2//GSTA2//ANXA7//IFNG//CCND1//NREP//CAMSAP1//SAMD14//PSD//KRT80//KRTAP5-6//KRTAP2-4//MAF//SOX5//ZEB1//MGST1//UNC5B//VAMP3//CASP2//FLI1//PAX5//HIVEP3//HSBP1//IRF1//APCDD1//CCL2//TMEM64//CD8A//MSN//CFL2//BBS9//INHBB//ARID5B//SOCS1//MBNL1//HMGB3//RAF1//TFAP2A//SOCS3//DLK2//CREBL2//BTG1//MYCN//SERPINB13//ERRFI1//SRSF6//MYB//SOCS5//KLF13//ETS1//TNRC6B//AGO1//NFE2//CXCL9//NTF3//YWHAG//DDX6//DNMT3B//ETV5//MMD//SMOC1//RASSF2//TOB1//NPNT//TFE3//GPR55//PPARGC1B//KLF10//ADNP//TNFRSF12A//CCL21//SDC1//ZNF521//EN1//FMR1//FXR1//ULK2//SLC9A6//DBN1//TNFRSF21//HOMER1//SGK1//MEF2A//HECW2//JARID2//CAMSAP2//TBL1XR1//ADRB1//ZNF516//BNIP2//GABRA5//PDE5A//YIPF6//E2F8//DNAJB6//SPRED1//ARC//CPEB3//ZMYND8//MAPK6//LRP8//PPP1R16B//RAP1B//LDLR//RAP2C//ARID4B//NANOS1//FOXJ2//CRKL//PSMA2//PSMD12//NLN//SMURF1//SYT1//PLAA//CLOCK//FBXW7//EDARADD//ELK3//RHOB//MYBL1//NHLH2//ABHD5//PURA//SUV39H1//UBE2V1//YES1//ZNF3//BAG6//SPATA9//ZIC5//ADAM18//FOXP2// |
| GO:0051254 | positive regulation of RNA metabolic process | Biological process | 134 | 1523 | 902 | 17653 | 1.72193549608152 | 1.98068011401482e-10 | 5.20189579737891e-08 | 9.70318565870476 | 0.148558758314856 | EP300//CREBL2//ESR1//ETS1//ATF5//FOXF1//FOXF2//FLI1//PHF8//NR5A2//BAMBI//GLI3//ATAD2//MDFIC//ID2//IGF1//IRF1//AR//JUN//LRP6//MYB//MYBL1//MYCN//NEUROD1//NFE2//NPAS2//NPAT//OTX2//PPARG//TRERF1//MAPK1//RB1//HIVEP3//RORA//PRDM16//SMARCA2//SOX4//SS18//TFAP2A//TNF//UBE2V1//WNT7A//WT1//TBL1XR1//TEAD2//TP63//RUNX3//FUBP3//ACVR1//CLOCK//ZNF516//INSIG2//ZBTB18//CEBPB//ADCYAP1//PPARGC1B//CREBRF//E2F5//S1PR1//EDN1//ELK3//EN1//ETV5//MED19//SIRT1//ZFPM2//KPNA6//AGO1//DLL1//TBK1//GTF2H1//CNOT7//H2AFZ//NRG1//FOXA1//IL2//IRF2//ISL1//MAF//MEF2A//MEOX2//MET//MYOG//PPP1R12A//NCK1//NEUROG1//NFIA//NFIB//NFIC//NHLH2//NR4A2//PAX5//IER5//ARMCX3//KLF13//ARID4B//PKNOX1//CYTL1//POU3F2//POU4F1//EGLN1//PPP3R1//FOXJ2//CCNL1//MAVS//RAF1//ATXN7//BMPR2//ZEB1//TFE3//KLF10//TXK//YES1//E2F8//FOSL1//PCGF5//KLF7//CBFB//CCNT2//NOG//RPS6KA5//ST18//TOB1//CPEB3//TNRC6B//PRR5L//GTPBP1//CNOT6L//YTHDF2//QKI//NANOS1//PRKAA1//FBXW7//NPNT// |
| GO:0035295 | tube development | Biological process | 103 | 1065 | 902 | 17653 | 1.8927776563297 | 2.02094231255747e-10 | 5.20189579737891e-08 | 9.6944460831673 | 0.114190687361419 | S1PR1//EFNA1//ELK3//EREG//UNC5B//SIRT1//LEMD3//CLIC4//HOXA3//JUN//KDR//RHOB//MEOX2//TNFRSF12A//PIK3CA//PKNOX1//PTEN//RORA//CCL2//VEGFB//WNT7A//EDN1//PPP3R1//TGFBR2//ACVR1//GJC1//FOXF1//ZFPM2//RASA1//WT1//CCM2//TEAD2//QKI//NPNT//ROBO2//SDC1//SLIT2//GLI3//SOX4//LRP6//SEMA4C//PRKACB//TSC1//FZD3//FZD6//NOG//GJA1//DLL1//E2F8//EFNB2//EDNRB//SPRED1//SULF1//AGO1//PPARG//FOXJ2//SARS//GSC//FOXA1//EP300//MYCN//BAG6//EN1//ETS1//EGLN1//VASH2//F3//ISL1//ITGB8//BTG1//C5AR1//ADAM12//EDN2//ERRFI1//BMPR2//FOXP2//PHF14//ID2//RB1//FOXF2//TNF//ZEB1//MET//PIK3CB//TP63//MAPK1//RDH10//DLG5//NFIB//SRSF6//YIPF6//AR//ESR1//ETV5//ARID2//ADIPOR2//TNFAIP3//KLHL3//NFIA//FOXP1//PPP1R16B//RAP1A//RAPGEF2// |
| GO:0060255 | regulation of macromolecule metabolic process | Biological process | 418 | 6425 | 902 | 17653 | 1.27325424693936 | 2.07675863565327e-10 | 5.20189579737891e-08 | 9.68261397495987 | 0.463414634146341 | GADD45A//GTF2H1//PTEN//CCNT2//DIRAS3//NPAT//TSHZ1//ZBTB18//CEBPB//TCERG1//STRAP//KLF12//GSC//ARX//DNMT3B//EDN1//EDNRB//EFNA1//CC2D1B//EN1//EP300//ESR1//JAZF1//CPEB3//FOXF1//MYT1L//SIN3B//SIRT1//ZFPM2//DNAJB5//GLI3//CNOT7//HIC1//FOXA1//HSBP1//ID2//IFNG//IRF2//AR//ISL1//JARID2//JUN//MAF//MDM4//MEF2A//MYB//NFIA//NFIB//NFIC//NR4A2//PAX5//KLF3//POU4F1//PPARG//ZBTB4//RB1//RBBP8//CCND1//RNF2//SARS//PRDM16//SMARCA2//SUV39H1//ZEB1//TFAP2A//TGIF1//KLF10//TNF//UBE2I//WT1//ZNF217//LRP8//BHLHE41//TBL1XR1//E2F8//ARID5B//BHLHE40//TP63//RUNX3//NOG//FOXP2//PHF14//PNRC1//DCP2//PPP2CA//CRKL//TAOK1//RAF1//RAP1A//C1QTNF2//LPAR1//NRG1//IGF1//NTF3//PIK3CB//PRKAA1//MAPK1//C5AR1//UBE2V1//SPRED1//DUSP1//CNOT6L//PAN3//CNOT6//PATL1//MBNL2//RBFOX3//FMR1//MBNL1//RBM25//SRSF2//SRSF6//AGO1//SEPSECS//SECISBP2L//EPS15//NSG1//VAMP3//SOCS1//IGFBP3//IL2//LRP6//PRR5L//PPP1R15B//SLIT2//NPTN//KDR//MAVS//CCND2//EREG//FBXW7//ERRFI1//GPRC5A//SOCS5//ESCO2//BAHD1//H2AFZ//ARID4B//HIPK3//ZMYND11//ZNF526//PPARGC1B//ZNF800//ARID2//ELAVL2//ATF5//ZFP30//ELL2//KDM2A//MYCBP2//FLII//ZMYND8//NR5A2//ZNF521//ZBTB11//GTF2A1//GTF2E1//NRBF2//HIVEP2//HMGB3//ZNF680//ZNF662//AFF1//MLLT6//OGG1//ASCC1//PHF20L1//COMMD10//BNC2//ZNF532//SLC2A4RG//PAK6//PHTF2//ZBTB26//HIVEP3//RORA//SOX4//BTF3//BTG1//ZNF3//ZNF131//ZNF227//CSDE1//ZNF655//SAP30L//SNIP1//ANP32A//TEAD2//RPS6KA5//CLOCK//ZNF516//CREBL2//ZNF367//ELK3//EMX2//ETS1//ETV5//FOXF2//FLI1//ADNP//EPC2//HBP1//FOXP1//ATAD2//HOXA3//HOXD1//BOLA3//NR3C2//MYBL1//MYCN//NEUROG1//NFE2//NHLH2//KLF13//BRWD1//POU3F2//MED9//TRERF1//FOXJ2//MIER1//PURA//RFX7//TRAK2//SMARCD2//SOX5//MED22//TXK//ZNF708//ZSCAN5A//FOSL1//LCOR//KLF7//CBFB//BDP1//DDX6//TSC1//KBTBD8//GNAQ//NCK1//GSKIP//YWHAG//SOCS3//PPM1F//EGLN3//F3//PMAIP1//CASP2//COPS8//G3BP2//MDFIC//PI15//TOB1//RICTOR//PIK3CA//PPP2CB//MAPK6//PTHLH//TGFBR2//DDX3Y//CCL21//PPP4R2//NANOS1//RAB3GAP1//GJA1//ITGB8//LDLR//MSN//WNT3//WNT10A//FUBP3//CD3E//QKI//TBK1//ANXA7//NDFIP2//CALM2//INHBB//BMP3//BMPR2//TTK//GDF5//ACVR1//SERPINI1//SERPINB13//SPOCK1//RECK//SERP1//PRR16//FXR1//CELF1//FLNA//PHF8//BAMBI//IRF1//NEUROD1//NPAS2//OTX2//SS18//WNT7A//DNAJB6//RASD1//ATXN1//BTAF1//TIA1//MEX3B//SPOPL//TSN//CR2//SMURF1//SULF1//LEMD3//CTDSPL2//TNRC6B//TNFAIP3//BAG5//MOB1B//RAP2C//RASSF2//RWDD3//SLK//CCT6A//BAG6//ANKIB1//LONRF3//LONRF1//ARPP19//PPP1R1B//MYLIP//MID1//SEMA4C//GFPT1//INSIG2//PFN2//IFNA1//ADCY3//PRKACB//PRKAR2B//PPP1R16B//SPPL3//CNEP1R1//PPP1R12A//FAM83D//ARL6IP1//PDE5A//SYNGAP1//MET//ROR1//EGLN1//FZD6//FASTK//YTHDF2//PSMA2//PSMD12//RCAN3//UBXN2B//PPP2R5E//NMI//TMEFF2//HECW2//CCNY//CCNL1//ADCYAP1//CACUL1//MMD//RAPGEF2//CREBRF//E2F5//S1PR1//MED19//KPNA6//DLL1//MEOX2//MYOG//IER5//ARMCX3//PKNOX1//CYTL1//PPP3R1//ATXN7//TFE3//YES1//PCGF5//ST18//PAIP2//BOLL//GRIK2//SH3D19//C8ORF44-SGK3//SGK3//SGK1//RHEBL1//IRAK4//NOVA1//RASA1//ZER1//DCUN1D4//ARRDC3//CCL2//PTGES3//MTMR9//PMEPA1//LDLRAD4//TMBIM6//NUP54//GTPBP1//RANBP9//NPNT//CCL8//GPR55//ACSL1//CAST//PLAA//CPEB2//CRTAP//HSBP1L1//FAM168A//KCNK2//CPEB1// |
| GO:0019222 | regulation of metabolic process | Biological process | 444 | 6920 | 902 | 17653 | 1.25570857311306 | 2.33366590036282e-10 | 5.63663660148348e-08 | 9.63196131967659 | 0.492239467849224 | GADD45A//GTF2H1//PTEN//CCNT2//DIRAS3//NPAT//TSHZ1//ZBTB18//CEBPB//TCERG1//STRAP//KLF12//GSC//ARX//DNMT3B//EDN1//EDNRB//EFNA1//CC2D1B//EN1//EP300//ESR1//JAZF1//CPEB3//FOXF1//MYT1L//SIN3B//SIRT1//ZFPM2//DNAJB5//GLI3//CNOT7//HIC1//FOXA1//HSBP1//ID2//IFNG//IRF2//AR//ISL1//JARID2//JUN//MAF//MDM4//MEF2A//MYB//NFIA//NFIB//NFIC//NR4A2//PAX5//KLF3//POU4F1//PPARG//ZBTB4//RB1//RBBP8//CCND1//RNF2//SARS//PRDM16//SMARCA2//SUV39H1//ZEB1//TFAP2A//TGIF1//KLF10//TNF//UBE2I//WT1//ZNF217//LRP8//BHLHE41//TBL1XR1//E2F8//ARID5B//BHLHE40//TP63//RUNX3//NOG//FOXP2//PHF14//PNRC1//DCP2//PPP2CA//CRKL//TAOK1//RAF1//RAP1A//C1QTNF2//LPAR1//NRG1//IGF1//NTF3//PIK3CB//PRKAA1//MAPK1//C5AR1//UBE2V1//SPRED1//DUSP1//CNOT6L//PAN3//CNOT6//PATL1//MBNL2//RBFOX3//FMR1//MBNL1//RBM25//SRSF2//SRSF6//AGO1//SEPSECS//SECISBP2L//EPS15//NSG1//VAMP3//SOCS1//IGFBP3//IL2//LRP6//PRR5L//PPP1R15B//SLIT2//NPTN//KDR//MAVS//CCND2//EREG//FBXW7//ERRFI1//GPRC5A//SOCS5//NUP54//NLN//ESCO2//BAHD1//H2AFZ//ARID4B//HIPK3//ZMYND11//ZNF526//PPARGC1B//ZNF800//ARID2//ELAVL2//ATF5//ZFP30//ELL2//KDM2A//MYCBP2//FLII//ZMYND8//NR5A2//ZNF521//ZBTB11//GTF2A1//GTF2E1//NRBF2//HIVEP2//HMGB3//ZNF680//ZNF662//AFF1//MLLT6//OGG1//ASCC1//PHF20L1//COMMD10//BNC2//ZNF532//SLC2A4RG//PAK6//PHTF2//ZBTB26//HIVEP3//RORA//SOX4//BTF3//BTG1//ZNF3//ZNF131//ZNF227//CSDE1//ZNF655//SAP30L//SNIP1//ANP32A//TEAD2//RPS6KA5//CLOCK//ZNF516//CREBL2//ZNF367//ELK3//EMX2//ETS1//ETV5//FOXF2//FLI1//ADNP//EPC2//HBP1//FOXP1//ATAD2//HOXA3//HOXD1//BOLA3//NR3C2//MYBL1//MYCN//NEUROG1//NFE2//NHLH2//KLF13//BRWD1//POU3F2//MED9//TRERF1//FOXJ2//MIER1//PURA//RFX7//TRAK2//SMARCD2//SOX5//MED22//TXK//ZNF708//ZSCAN5A//FOSL1//LCOR//KLF7//CBFB//BDP1//DDX6//TSC1//KBTBD8//GNAQ//NCK1//GSKIP//YWHAG//SOCS3//PPM1F//PSMA2//PSMD12//EGLN3//F3//PMAIP1//CASP2//GRM3//COPS8//G3BP2//MDFIC//PYURF//PIGA//PI15//TOB1//RICTOR//PIK3CA//PPP2CB//MAPK6//PTHLH//TGFBR2//DDX3Y//DRAM1//RRAGD//ULK2//MET//MTMR9//CCL21//PPP4R2//CRHR1//NANOS1//RAB3GAP1//GJA1//ITGB8//LDLR//MSN//WNT3//WNT10A//FUBP3//CD3E//QKI//TBK1//ANXA7//NDFIP2//NPNT//CALM2//INHBB//BMP3//BMPR2//TTK//GDF5//ACVR1//CNEP1R1//INSIG2//ABHD5//FKBP15//PPP1R12A//PLEKHF2//SERPINI1//SERPINB13//SPOCK1//RECK//ARPP19//SERP1//PRR16//FXR1//RAB12//SESN1//SNX5//CELF1//FLNA//PHF8//BAMBI//IRF1//NEUROD1//NPAS2//OTX2//SS18//WNT7A//DNAJB6//RASD1//ATXN1//BTAF1//TIA1//MEX3B//ACSL1//RGL1//TNFRSF21//SPOPL//TSN//CR2//SMURF1//SULF1//LEMD3//CTDSPL2//CXCL9//ADRB1//EGLN1//PDE5A//TNRC6B//TNFAIP3//BAG5//MOB1B//RAP2C//RASSF2//RWDD3//SLK//CCT6A//BAG6//ANKIB1//LONRF3//LONRF1//PPP1R1B//MYLIP//MID1//SEMA4C//GFPT1//PFN2//IFNA1//ROR1//ADCY3//PRKACB//PRKAR2B//PPP1R16B//SPPL3//FAM83D//ADIPOR2//ATXN7//PTGES3//ITSN1//ARL6IP1//SYNGAP1//FZD6//FASTK//YTHDF2//RCAN3//UBXN2B//PPP2R5E//NMI//TMEFF2//MID1IP1//ELOVL5//HECW2//CCNY//CCNL1//MYOG//ADCYAP1//CACUL1//MMD//RAPGEF2//CREBRF//E2F5//S1PR1//MED19//KPNA6//DLL1//MEOX2//IER5//ARMCX3//PKNOX1//CYTL1//PPP3R1//TFE3//YES1//PCGF5//ST18//PAIP2//BOLL//HPRT1//GRIK2//SH3D19//C8ORF44-SGK3//SGK3//SGK1//RHEBL1//IRAK4//NOVA1//RASA1//ZER1//DCUN1D4//ARRDC3//CCL2//PMEPA1//LDLRAD4//EDN2//TMBIM6//GTPBP1//RANBP9//CCL8//GPR55//CAST//PLAA//CPEB2//CRTAP//FEZ2//HSBP1L1//FAM168A//KCNK2//CPEB1//TBC1D12// |
| GO:0030182 | neuron differentiation | Biological process | 119 | 1303 | 902 | 17653 | 1.78737026782812 | 2.52616008820664e-10 | 5.891179543635e-08 | 9.59753913073171 | 0.131929046563193 | SOX4//SLITRK3//OGN//POU4F1//PRELP//WNT7A//SLIT2//RANBP9//ARX//DPYSL2//EFNA1//EFNB2//GLI3//OTX2//PIK3CA//PIK3CB//ENAH//MAPK1//ROBO2//SCN1B//WNT3//KLF7//RPS6KA5//NRXN3//FEZ2//NOG//PTEN//SNAP25//STMN2//ADCYAP1//NPTN//NCK1//SERPINI1//RAP1A//RAPGEF2//LPAR1//MYLIP//PTPRG//SPOCK1//TSC1//BAG5//EDNRB//GNAQ//STRN//SYNGAP1//CD3E//ISL1//RORA//PLXNA4//ATF5//HPRT1//NR4A2//NFIB//RAB21//JUN//NREP//CAMSAP1//SAMD14//GPM6A//LRP12//PSD//RB1//UNC5B//NEUROD1//FZD3//MYCN//DLL1//NTF3//YWHAG//DDX6//ID2//DNMT3B//ETV5//MMD//FOXA1//NEUROG1//ZEB1//GDF5//ADNP//NRG1//TNFRSF12A//MYT1L//ZNF521//POU3F2//EN1//FMR1//FXR1//ULK2//SLC9A6//GJA1//SGK1//MEF2A//TRAK2//HECW2//BMPR2//SEMA4C//RUNX3//DBN1//CAMSAP2//GABRA5//ACSL4//ARC//DOCK10//CPEB3//ZMYND8//IL2//MAPK6//DLG5//LRP8//LRP6//NANOS1//SMURF1//SYT1//PLAA//CEBPB//S1PR1//EMX2//HOXD1//WNT10A// |
| GO:0048869 | cellular developmental process | Biological process | 302 | 4323 | 902 | 17653 | 1.36720516722548 | 2.74899257334111e-10 | 6.19714559116864e-08 | 9.56082643344185 | 0.334811529933481 | CDH20//NRG1//SS18//WNT3//TP63//EREG//YTHDF2//GJC1//FOXF1//ZFPM2//KDR//RASA1//TGFBR2//WT1//CCM2//TEAD2//QKI//GJA1//IGFBP3//RDH14//BMP3//CBFB//NOG//AGFG1//DLL1//EDNRB//ISL1//SEMA4C//ACVR1//GPM6A//LRP12//NR4A2//SPOCK1//FZD3//RAPGEF2//ID2//FOXF2//PPP3R1//FAM83D//DLG5//CLIC4//MET//GLI3//SNX19//NFIB//CYTL1//WNT7A//GDF5//RUNX3//SULF1//BMPR2//ESR1//PTHLH//ESCO2//KCNAB2//DOCK10//SOX4//EFNA1//NEUROD1//PALLD//AR//FRMD6//CELF1//DZIP1//RNF2//BOLL//TDRD5//SMARCA2//SLITRK3//OGN//POU4F1//PRELP//SLIT2//RANBP9//ARX//DPYSL2//EFNB2//OTX2//PIK3CA//PIK3CB//ENAH//MAPK1//ROBO2//SCN1B//KLF7//RPS6KA5//NRXN3//FEZ2//ADAM12//SIRT1//MORC3//GSTM3//CFDP1//WASF3//PALM2//LPAR1//EPS8//BAMBI//ANXA7//RHOB//RND3//MSN//BRWD1//CCL2//PLXNA4//WDR1//STRAP//FOXA1//PPP2CA//PTEN//LDLRAD4//FRZB//EP300//PPARG//MYOG//BHLHE41//SNAP25//STMN2//ADCYAP1//NPTN//NCK1//SERPINI1//RAP1A//MYLIP//PTPRG//TSC1//BAG5//ROR1//POU3F2//LRP6//KBTBD8//RDH10//EDN1//TAPT1//WNT10A//GSC//IGF1//GNAQ//STRN//SYNGAP1//CD3E//RORA//ATF5//HPRT1//FLNA//PRDM16//TRAK2//KCNJ10//SH3D19//CAMSAP1//ARC//EDARADD//CREBL2//ELK3//ETS1//ETV5//FLI1//FOXP1//INHBB//MYB//MYBL1//NHLH2//ABHD5//FOXJ2//SMURF1//PURA//RAF1//RB1//SMOC1//SUV39H1//TXK//UBE2V1//YES1//ZNF3//BAG6//FXR1//SPATA9//ZIC5//ADAM18//FOXP2//CEBPB//S1PR1//EMX2//ACSL4//MYT1L//HOXD1//NEUROG1//CR2//IFNA1//DNAJB9//TGM3//IL2//PKNOX1//JUN//TMOD3//TMOD1//OSTM1//TNF//RAB21//CNN3//NR5A2//GSTA2//IFNG//CCND1//NREP//SAMD14//PSD//KRT80//KRTAP5-6//KRTAP2-4//MAF//SOX5//ZEB1//MGST1//UNC5B//VAMP3//CASP2//PAX5//HIVEP3//HSBP1//IRF1//APCDD1//TMEM64//CD8A//CFL2//BBS9//ARID5B//SOCS1//MBNL1//HMGB3//TFAP2A//SOCS3//DLK2//BTG1//MYCN//SERPINB13//ERRFI1//SRSF6//SOCS5//KLF13//TNRC6B//AGO1//NFE2//CXCL9//NTF3//YWHAG//DDX6//DNMT3B//MMD//RASSF2//TOB1//NPNT//TFE3//GPR55//PPARGC1B//KLF10//ADNP//TNFRSF12A//CCL21//SDC1//ZNF521//EN1//FMR1//ULK2//SLC9A6//DBN1//TNFRSF21//HOMER1//SGK1//MEF2A//HECW2//JARID2//CAMSAP2//TBL1XR1//ADRB1//ZNF516//BNIP2//GABRA5//PDE5A//YIPF6//E2F8//DNAJB6//SPRED1//CPEB3//ZMYND8//MAPK6//LRP8//PPP1R16B//RAP1B//LDLR//SNX2//RAP2C//ARID4B//NANOS1//CRKL//PSMA2//PSMD12//NLN//SYT1//PLAA//CLOCK//FBXW7// |
| GO:0019219 | regulation of nucleobase-containing compound metabolic process | Biological process | 302 | 4328 | 902 | 17653 | 1.36562567881602 | 3.14362428798812e-10 | 6.85817130956892e-08 | 9.50256936447863 | 0.334811529933481 | NPAT//TSHZ1//ZBTB18//CEBPB//TCERG1//STRAP//KLF12//GSC//ARX//DNMT3B//EDN1//EDNRB//EFNA1//CC2D1B//EN1//EP300//ESR1//JAZF1//CPEB3//FOXF1//MYT1L//SIN3B//SIRT1//ZFPM2//DNAJB5//GLI3//CNOT7//HIC1//FOXA1//HSBP1//ID2//IFNG//IRF2//AR//ISL1//JARID2//JUN//MAF//MDM4//MEF2A//MYB//NFIA//NFIB//NFIC//NR4A2//PAX5//KLF3//POU4F1//PPARG//ZBTB4//RB1//RBBP8//CCND1//RNF2//SARS//PRDM16//SMARCA2//SUV39H1//ZEB1//TFAP2A//TGIF1//KLF10//TNF//UBE2I//WT1//ZNF217//LRP8//BHLHE41//TBL1XR1//E2F8//ARID5B//BHLHE40//TP63//RUNX3//NOG//FOXP2//PHF14//MBNL2//RBFOX3//FMR1//MBNL1//RBM25//SRSF2//SRSF6//NUP54//ESCO2//PPP2CA//UBE2V1//BAHD1//H2AFZ//HIPK3//ZMYND11//PNRC1//ZNF526//PPARGC1B//ZNF800//ARID2//ELAVL2//ATF5//ZFP30//ELL2//KDM2A//MYCBP2//FLII//ZMYND8//CNOT6L//NR5A2//ZNF521//ZBTB11//GTF2A1//GTF2E1//NRBF2//HIVEP2//HMGB3//ZNF680//ZNF662//AFF1//MLLT6//OGG1//ASCC1//PHF20L1//COMMD10//BNC2//ZNF532//SLC2A4RG//PAK6//PHTF2//CNOT6//ZBTB26//HIVEP3//RORA//SOX4//BTF3//BTG1//ZNF3//ZNF131//ZNF227//CSDE1//ZNF655//SAP30L//SNIP1//ANP32A//TEAD2//RPS6KA5//CLOCK//ZNF516//CREBL2//ZNF367//ELK3//EMX2//ETS1//ETV5//FOXF2//FLI1//ADNP//EPC2//HBP1//FOXP1//ATAD2//HOXA3//HOXD1//BOLA3//NR3C2//MYBL1//MYCN//NEUROG1//NFE2//NHLH2//KLF13//ARID4B//BRWD1//POU3F2//MED9//TRERF1//FOXJ2//MIER1//PURA//RFX7//TRAK2//SMARCD2//SOX5//MED22//TXK//ZNF708//ZSCAN5A//FOSL1//LCOR//KLF7//CBFB//BDP1//GRM3//G3BP2//PPP4R2//CRHR1//FLNA//PHF8//BAMBI//MDFIC//IGF1//IRF1//LRP6//NEUROD1//NPAS2//OTX2//MAPK1//SS18//WNT7A//FUBP3//ACVR1//DNAJB6//EREG//NRG1//RASD1//ATXN1//BTAF1//PPM1F//TOB1//SMURF1//BMP3//BMPR2//GDF5//SULF1//LEMD3//CTDSPL2//CALM2//CXCL9//ADRB1//PTHLH//EGLN1//PDE5A//RWDD3//TNFAIP3//DCP2//CCT6A//FBXW7//INSIG2//FZD6//CELF1//FASTK//YTHDF2//PSMA2//PSMD12//FXR1//PIK3CA//MYOG//PRKAA1//ADCYAP1//CREBRF//E2F5//S1PR1//MED19//KPNA6//AGO1//DLL1//TBK1//GTF2H1//IL2//MEOX2//MET//PPP1R12A//NCK1//IER5//ARMCX3//PKNOX1//CYTL1//PPP3R1//CCNL1//MAVS//RAF1//ATXN7//TFE3//YES1//PCGF5//CCNT2//ST18//TIA1//C8ORF44-SGK3//SGK3//SGK1//PTEN//RHEBL1//ROR1//IRAK4//NOVA1//RASA1//PTGES3//TNRC6B//TMBIM6//PRR5L//GTPBP1//QKI//EGLN3//BOLL//NANOS1//RAP2C//NMI//HSBP1L1//FAM168A//DUSP1//GJA1//KCNK2//NPNT// |
| GO:0051171 | regulation of nitrogen compound metabolic process | Biological process | 407 | 6240 | 902 | 17653 | 1.27650289243277 | 3.379824763986e-10 | 7.14304839963666e-08 | 9.4711058162924 | 0.451219512195122 | GADD45A//GTF2H1//PTEN//CCNT2//DIRAS3//NPAT//TSHZ1//ZBTB18//CEBPB//TCERG1//STRAP//KLF12//GSC//ARX//DNMT3B//EDN1//EDNRB//EFNA1//CC2D1B//EN1//EP300//ESR1//JAZF1//CPEB3//FOXF1//MYT1L//SIN3B//SIRT1//ZFPM2//DNAJB5//GLI3//CNOT7//HIC1//FOXA1//HSBP1//ID2//IFNG//IRF2//AR//ISL1//JARID2//JUN//MAF//MDM4//MEF2A//MYB//NFIA//NFIB//NFIC//NR4A2//PAX5//KLF3//POU4F1//PPARG//ZBTB4//RB1//RBBP8//CCND1//RNF2//SARS//PRDM16//SMARCA2//SUV39H1//ZEB1//TFAP2A//TGIF1//KLF10//TNF//UBE2I//WT1//ZNF217//LRP8//BHLHE41//TBL1XR1//E2F8//ARID5B//BHLHE40//TP63//RUNX3//NOG//FOXP2//PHF14//CRKL//TAOK1//RAF1//RAP1A//C1QTNF2//LPAR1//NRG1//IGF1//NTF3//PIK3CB//PRKAA1//MAPK1//C5AR1//UBE2V1//SPRED1//DUSP1//PPP2CA//MBNL2//RBFOX3//FMR1//MBNL1//RBM25//SRSF2//SRSF6//SEPSECS//SECISBP2L//SOCS1//IGFBP3//IL2//LRP6//PRR5L//PPP1R15B//SLIT2//NPTN//KDR//MAVS//CCND2//EREG//FBXW7//ERRFI1//GPRC5A//SOCS5//NUP54//ESCO2//BAHD1//H2AFZ//HIPK3//ZMYND11//PNRC1//ZNF526//PPARGC1B//ZNF800//ARID2//ELAVL2//ATF5//ZFP30//ELL2//KDM2A//MYCBP2//FLII//ZMYND8//CNOT6L//NR5A2//ZNF521//ZBTB11//GTF2A1//GTF2E1//NRBF2//HIVEP2//HMGB3//ZNF680//ZNF662//AFF1//MLLT6//OGG1//ASCC1//PHF20L1//COMMD10//BNC2//ZNF532//SLC2A4RG//PAK6//PHTF2//CNOT6//ZBTB26//HIVEP3//RORA//SOX4//BTF3//BTG1//ZNF3//ZNF131//ZNF227//CSDE1//ZNF655//SAP30L//SNIP1//ANP32A//TEAD2//RPS6KA5//CLOCK//ZNF516//CREBL2//ZNF367//ELK3//EMX2//ETS1//ETV5//FOXF2//FLI1//ADNP//EPC2//HBP1//FOXP1//ATAD2//HOXA3//HOXD1//BOLA3//NR3C2//MYBL1//MYCN//NEUROG1//NFE2//NHLH2//KLF13//ARID4B//BRWD1//POU3F2//MED9//TRERF1//FOXJ2//MIER1//PURA//RFX7//TRAK2//SMARCD2//SOX5//MED22//TXK//ZNF708//ZSCAN5A//FOSL1//LCOR//KLF7//CBFB//BDP1//DDX6//TSC1//KBTBD8//GNAQ//NCK1//GSKIP//YWHAG//SOCS3//PPM1F//PSMA2//PSMD12//EGLN3//F3//PMAIP1//CASP2//GRM3//COPS8//G3BP2//MDFIC//AGO1//PI15//CCL21//PPP4R2//CRHR1//CALM2//INHBB//BMP3//BMPR2//TTK//GDF5//ACVR1//LDLR//SERPINI1//SERPINB13//SPOCK1//RECK//SERP1//PRR16//FXR1//FLNA//PHF8//BAMBI//IRF1//NEUROD1//NPAS2//OTX2//SS18//WNT7A//FUBP3//DNAJB6//RASD1//ATXN1//BTAF1//TOB1//CELF1//NANOS1//TIA1//MEX3B//SPOPL//CR2//SMURF1//SULF1//LEMD3//CTDSPL2//CXCL9//ADRB1//PTHLH//EGLN1//PDE5A//TNFAIP3//BAG5//NDFIP2//MOB1B//RAP2C//RASSF2//RWDD3//PIK3CA//TGFBR2//SLK//RICTOR//DCP2//CCT6A//BAG6//ANKIB1//LONRF3//LONRF1//ARPP19//PPP1R1B//MYLIP//MID1//SEMA4C//INSIG2//TBK1//PFN2//IFNA1//ADCY3//PRKACB//PRKAR2B//TNRC6B//PPP1R16B//SPPL3//CNEP1R1//PPP1R12A//FAM83D//ARL6IP1//SYNGAP1//MET//ROR1//FZD6//FASTK//YTHDF2//RCAN3//UBXN2B//PPP2R5E//CD3E//NMI//GJA1//HECW2//CCNY//CCNL1//MYOG//ADCYAP1//CACUL1//MMD//RAPGEF2//CREBRF//E2F5//S1PR1//MED19//KPNA6//DLL1//MEOX2//IER5//ARMCX3//PKNOX1//CYTL1//PPP3R1//ATXN7//TFE3//YES1//PCGF5//ST18//PAIP2//BOLL//HPRT1//GRIK2//SH3D19//C8ORF44-SGK3//SGK3//SGK1//RHEBL1//IRAK4//NOVA1//RASA1//ZER1//DCUN1D4//ARRDC3//PTGES3//PMEPA1//LDLRAD4//TMBIM6//GTPBP1//QKI//RANBP9//NPNT//CCL2//CCL8//GPR55//ACSL1//CAST//PLAA//CPEB2//CRTAP//RAB3GAP1//MSN//HSBP1L1//FAM168A//KCNK2//CPEB1// |
| GO:0080090 | regulation of primary metabolic process | Biological process | 415 | 6394 | 902 | 17653 | 1.27024486648029 | 3.75389510514156e-10 | 7.69320987759769e-08 | 9.42551786700894 | 0.460088691796009 | GADD45A//GTF2H1//PTEN//CCNT2//DIRAS3//NPAT//TSHZ1//ZBTB18//CEBPB//TCERG1//STRAP//KLF12//GSC//ARX//DNMT3B//EDN1//EDNRB//EFNA1//CC2D1B//EN1//EP300//ESR1//JAZF1//CPEB3//FOXF1//MYT1L//SIN3B//SIRT1//ZFPM2//DNAJB5//GLI3//CNOT7//HIC1//FOXA1//HSBP1//ID2//IFNG//IRF2//AR//ISL1//JARID2//JUN//MAF//MDM4//MEF2A//MYB//NFIA//NFIB//NFIC//NR4A2//PAX5//KLF3//POU4F1//PPARG//ZBTB4//RB1//RBBP8//CCND1//RNF2//SARS//PRDM16//SMARCA2//SUV39H1//ZEB1//TFAP2A//TGIF1//KLF10//TNF//UBE2I//WT1//ZNF217//LRP8//BHLHE41//TBL1XR1//E2F8//ARID5B//BHLHE40//TP63//RUNX3//NOG//FOXP2//PHF14//CRKL//TAOK1//RAF1//RAP1A//C1QTNF2//LPAR1//NRG1//IGF1//NTF3//PIK3CB//PRKAA1//MAPK1//C5AR1//UBE2V1//SPRED1//DUSP1//PPP2CA//MBNL2//RBFOX3//FMR1//MBNL1//RBM25//SRSF2//SRSF6//SEPSECS//SECISBP2L//SOCS1//IGFBP3//IL2//LRP6//PRR5L//PPP1R15B//SLIT2//NPTN//KDR//MAVS//CCND2//EREG//FBXW7//ERRFI1//GPRC5A//SOCS5//NUP54//NLN//ESCO2//BAHD1//H2AFZ//HIPK3//ZMYND11//PNRC1//ZNF526//PPARGC1B//ZNF800//ARID2//ELAVL2//ATF5//ZFP30//ELL2//KDM2A//MYCBP2//FLII//ZMYND8//CNOT6L//NR5A2//ZNF521//ZBTB11//GTF2A1//GTF2E1//NRBF2//HIVEP2//HMGB3//ZNF680//ZNF662//AFF1//MLLT6//OGG1//ASCC1//PHF20L1//COMMD10//BNC2//ZNF532//SLC2A4RG//PAK6//PHTF2//CNOT6//ZBTB26//HIVEP3//RORA//SOX4//BTF3//BTG1//ZNF3//ZNF131//ZNF227//CSDE1//ZNF655//SAP30L//SNIP1//ANP32A//TEAD2//RPS6KA5//CLOCK//ZNF516//CREBL2//ZNF367//ELK3//EMX2//ETS1//ETV5//FOXF2//FLI1//ADNP//EPC2//HBP1//FOXP1//ATAD2//HOXA3//HOXD1//BOLA3//NR3C2//MYBL1//MYCN//NEUROG1//NFE2//NHLH2//KLF13//ARID4B//BRWD1//POU3F2//MED9//TRERF1//FOXJ2//MIER1//PURA//RFX7//TRAK2//SMARCD2//SOX5//MED22//TXK//ZNF708//ZSCAN5A//FOSL1//LCOR//KLF7//CBFB//BDP1//DDX6//TSC1//KBTBD8//GNAQ//NCK1//GSKIP//YWHAG//SOCS3//PPM1F//PSMA2//PSMD12//EGLN3//F3//PMAIP1//CASP2//GRM3//COPS8//G3BP2//MDFIC//AGO1//PI15//CCL21//PPP4R2//CRHR1//CALM2//INHBB//BMP3//BMPR2//TTK//GDF5//ACVR1//CNEP1R1//LDLR//INSIG2//ABHD5//SERPINI1//SERPINB13//SPOCK1//RECK//ARPP19//SERP1//PRR16//FXR1//FLNA//PHF8//BAMBI//IRF1//NEUROD1//NPAS2//OTX2//SS18//WNT7A//FUBP3//DNAJB6//RASD1//ATXN1//BTAF1//TOB1//CELF1//NANOS1//TIA1//MEX3B//ACSL1//RGL1//TNFRSF21//SPOPL//CR2//SMURF1//SULF1//LEMD3//CTDSPL2//CXCL9//ADRB1//PTHLH//EGLN1//PDE5A//TNFAIP3//BAG5//NDFIP2//MOB1B//RAP2C//RASSF2//RWDD3//PIK3CA//TGFBR2//SLK//RICTOR//DCP2//CCT6A//BAG6//ANKIB1//LONRF3//LONRF1//PPP1R1B//MYLIP//MID1//SEMA4C//TBK1//PFN2//IFNA1//ADCY3//PRKACB//PRKAR2B//TNRC6B//PPP1R16B//SPPL3//PPP1R12A//FAM83D//ADIPOR2//ARL6IP1//SYNGAP1//MET//ROR1//FZD6//FASTK//YTHDF2//RCAN3//UBXN2B//PPP2R5E//CD3E//NMI//MID1IP1//ELOVL5//GJA1//HECW2//CCNY//CCNL1//MYOG//ADCYAP1//CACUL1//MMD//RAPGEF2//CREBRF//E2F5//S1PR1//MED19//KPNA6//DLL1//MEOX2//IER5//ARMCX3//PKNOX1//CYTL1//PPP3R1//ATXN7//TFE3//YES1//PCGF5//ST18//PAIP2//BOLL//HPRT1//GRIK2//SH3D19//C8ORF44-SGK3//SGK3//SGK1//RHEBL1//IRAK4//NOVA1//RASA1//ZER1//DCUN1D4//ARRDC3//PTGES3//MTMR9//PMEPA1//LDLRAD4//TMBIM6//GTPBP1//QKI//RANBP9//NPNT//CCL2//CCL8//GPR55//CAST//PLAA//CPEB2//CRTAP//RAB3GAP1//MSN//HSBP1L1//FAM168A//KCNK2//CPEB1// |
| GO:0048518 | positive regulation of biological process | Biological process | 384 | 5841 | 902 | 17653 | 1.28663689774592 | 6.55240100762889e-10 | 1.29986586536787e-07 | 9.18359953156645 | 0.425720620842572 | GADD45A//TNF//CRKL//TAOK1//RAF1//RAP1A//C1QTNF2//LPAR1//NRG1//IGF1//NTF3//PIK3CB//PRKAA1//MAPK1//C5AR1//UBE2V1//EREG//EPS15//NSG1//VAMP3//EDNRB//EFNA1//FMR1//SIRT1//NPTN//IFNG//KDR//MAVS//CCND1//PRR5L//CCND2//F3//RICTOR//PPP1R16B//JUN//BMPR2//VEGFB//VASH2//CCL21//ADRB1//ARL6IP1//GLI3//FZD3//FOXF1//MYCN//TGFBR2//TP63//FOXP2//EP300//PRKACB//PSMA2//PSMD12//SKP1//RPS6KA5//IRAK4//BAG6//CR2//CD3E//CCL8//EDN2//CXCL9//SKAP2//EDN1//GJA1//FBXW7//GTF2H1//EGLN3//PMAIP1//PPARG//CASP2//CNOT6L//CNOT7//MDM4//CNOT6//SOX4//E2F8//COPS8//MDFIC//DNAJA2//ADCYAP1//CNTFR//CACUL1//EFNB2//ETS1//BAMBI//DLL1//HOXA3//IL2//AR//ISL1//POU3F2//SPHK2//PTEN//PTHLH//PURA//TTK//FOSL1//NPNT//PYURF//PIGA//TOB1//EPS8//ARHGAP1//LASP1//ARHGAP6//SH3BGRL//HOMER1//TRPC3//SOCS1//SULF1//RORA//FOXP1//RHOB//WNT7A//PAN3//MEF2A//PDE5A//ARX//DNMT3B//RAB3GAP1//ID2//ITGB8//LDLR//MSN//POU4F1//TFAP2A//WNT3//WNT10A//FUBP3//NOG//QKI//PPM1F//PPARGC1B//KCNB1//SCN1B//CALM2//SMOC2//C2CD5//MYOG//INHBB//BMP3//GDF5//ACVR1//CNEP1R1//ABHD5//MTMR9//KCNK2//VPS4B//STMN2//NCK1//SERPINI1//RAPGEF2//SLC6A1//UNC5B//ARPP19//CPEB3//SERP1//PRR16//FXR1//CREBL2//ADIPOR2//RAB12//SESN1//TBK1//TSC1//ESR1//ATF5//FOXF2//FLI1//PHF8//NR5A2//ATAD2//IRF1//LRP6//MYB//MYBL1//NEUROD1//NFE2//NPAS2//NPAT//OTX2//TRERF1//RB1//HIVEP3//PRDM16//SMARCA2//SS18//WT1//TBL1XR1//TEAD2//RUNX3//CLOCK//ZNF516//SIRPA//SPPL3//DENND1B//TNFRSF21//PIK3CA//TXK//S1PR1//FAM89B//SEMA4C//PTP4A1//CBLL1//ADNP//CFL2//WDR1//PFN2//MET//CLIP1//NAV3//YES1//SYT1//RAB8B//NDFIP2//NEUROG1//WASF3//MIER1//RAP2C//RASSF2//RRAGD//PAK6//SLK//CCT6A//STIM2//SOX5//C8ORF44-SGK3//SGK2//SGK3//SGK1//ANKIB1//LONRF3//LONRF1//SOCS5//POLR3H//PPP1R15B//CEBPB//LRP8//MID1//INSIG2//IFNA1//FOXA1//RWDD3//ROR1//TNFAIP3//ADCY3//PRKAR2B//IQGAP2//ARFIP1//GPR55//DLG5//PPP1R12A//ANO1//SYBU//PTGES3//ITSN1//MOB1B//SOCS3//DUSP1//CDK19//PHLDA3//FRZB//IGFBP3//USP27X//ARHGEF4//TNFRSF12A//BCL2A1//ZMAT3//SOS2//SLIT2//FLNA//ATP2C1//UBE2I//SPRED1//HIC1//GRIK2//TMEM64//BTG1//ETV5//MMD//ZEB1//KLF10//MID1IP1//ELOVL5//TAPT1//MYLIP//SMURF1//HECW2//CCNY//CCNL1//CCNT2//ADAM12//TFE3//EVC//ZBTB18//CREBRF//E2F5//ELK3//EN1//MED19//ZFPM2//KPNA6//AGO1//H2AFZ//IRF2//MAF//MEOX2//NFIA//NFIB//NFIC//NHLH2//NR4A2//PAX5//IER5//ARMCX3//KLF13//ARID4B//PKNOX1//CYTL1//EGLN1//PPP3R1//FOXJ2//ATXN7//PCGF5//KLF7//CBFB//ST18//BOLL//RAET1E//HPRT1//RASGEF1A//SLC25A6//CTDSPL2//RAB21//TUB//ROBO2//PLXNA4//RIMS4//GCSAML//CCL2//SH3D19//ARID5B//RHEBL1//BNIP2//DCUN1D4//ARRDC3//ZMYND8//GPM6A//JARID2//SLITRK3//CHRNB4//TNRC6B//ARC//SNAP25//SRSF6//MAPK6//GTPBP1//YTHDF2//ACSL1//PPP2CA//MIEF1//GSKIP//NANOS1//CD2AP//PAIP2//DYNLL2//YWHAG//KCNE1//KCNJ2//TMOD3//CHMP3//SERINC3//NMI//FAM83D//SEPT9//PLAA//SDC1//NMUR1//SNIP1//TIA1//FAM168A//ABHD17C//DENND6A//GLRX// |
| GO:0022008 | neurogenesis | Biological process | 133 | 1536 | 902 | 17653 | 1.69462031711936 | 6.72708935204428e-10 | 1.29986586536787e-07 | 9.17217280379601 | 0.147450110864745 | GJA1//GPM6A//LRP12//NR4A2//SPOCK1//FZD3//RAPGEF2//GLI3//SOX4//SLITRK3//OGN//POU4F1//PRELP//WNT7A//SLIT2//RANBP9//ARX//DPYSL2//EFNA1//EFNB2//OTX2//PIK3CA//PIK3CB//ENAH//MAPK1//ROBO2//SCN1B//WNT3//KLF7//RPS6KA5//NRXN3//FEZ2//NOG//NFIB//PTEN//SNAP25//STMN2//ADCYAP1//NPTN//NCK1//SERPINI1//RAP1A//LPAR1//MYLIP//PTPRG//TSC1//BAG5//DLL1//ROR1//POU3F2//WASF3//ID2//EDNRB//GNAQ//STRN//SYNGAP1//CD3E//ISL1//RORA//NRG1//PLXNA4//ATF5//HPRT1//KCNJ10//PPP3R1//CEBPB//S1PR1//EMX2//ACSL4//MYT1L//HOXD1//NEUROG1//RB1//WNT10A//RUNX3//RAB21//JUN//NREP//CAMSAP1//SAMD14//PSD//UNC5B//NEUROD1//APCDD1//CCL2//MYCN//NTF3//YWHAG//DDX6//DNMT3B//ETV5//MMD//FOXA1//ZEB1//GDF5//ADNP//TNFRSF12A//ZNF521//EN1//FMR1//FXR1//ULK2//SLC9A6//DBN1//TNFRSF21//WDR1//PPARG//SGK1//MEF2A//TRAK2//HECW2//BMPR2//SEMA4C//CAMSAP2//SOX5//GABRA5//MYB//ARC//DOCK10//CPEB3//ZMYND8//IL2//MAPK6//DLG5//LRP8//LDLR//LRP6//NANOS1//SMURF1//SYT1//PLAA//FLNA//PRDM16// |
| GO:0048699 | generation of neurons | Biological process | 126 | 1437 | 902 | 17653 | 1.71603349550292 | 9.39350886414382e-10 | 1.76467501245013e-07 | 9.02717215047257 | 0.139689578713969 | GJA1//GPM6A//LRP12//NR4A2//SPOCK1//FZD3//RAPGEF2//GLI3//SOX4//SLITRK3//OGN//POU4F1//PRELP//WNT7A//SLIT2//RANBP9//ARX//DPYSL2//EFNA1//EFNB2//OTX2//PIK3CA//PIK3CB//ENAH//MAPK1//ROBO2//SCN1B//WNT3//KLF7//RPS6KA5//NRXN3//FEZ2//NOG//PTEN//SNAP25//STMN2//ADCYAP1//NPTN//NCK1//SERPINI1//RAP1A//LPAR1//MYLIP//PTPRG//TSC1//BAG5//EDNRB//GNAQ//STRN//SYNGAP1//CD3E//ISL1//RORA//PLXNA4//ATF5//HPRT1//NFIB//CEBPB//S1PR1//EMX2//ACSL4//MYT1L//HOXD1//ID2//NEUROG1//POU3F2//RB1//WNT10A//RUNX3//RAB21//JUN//NREP//CAMSAP1//SAMD14//PSD//UNC5B//NEUROD1//MYCN//DLL1//NTF3//YWHAG//DDX6//DNMT3B//ETV5//MMD//FOXA1//ZEB1//GDF5//ADNP//NRG1//TNFRSF12A//ZNF521//EN1//FMR1//FXR1//ULK2//SLC9A6//TNFRSF21//WDR1//PPARG//SGK1//MEF2A//TRAK2//HECW2//BMPR2//SEMA4C//DBN1//CAMSAP2//SOX5//GABRA5//MYB//ARC//DOCK10//CPEB3//ZMYND8//IL2//MAPK6//DLG5//LRP8//LDLR//LRP6//NANOS1//SMURF1//SYT1//PLAA//FLNA// |
| GO:0060284 | regulation of cell development | Biological process | 86 | 858 | 902 | 17653 | 1.96165733748882 | 1.30508297824859e-09 | 2.33788769357722e-07 | 8.88436187464767 | 0.0953436807095344 | GLI3//FZD3//FRZB//PTEN//SNAP25//STMN2//ADCYAP1//NPTN//NCK1//SERPINI1//RAP1A//SCN1B//RAPGEF2//LPAR1//EFNB2//MYLIP//PTPRG//SPOCK1//TSC1//BAG5//EDNRB//DPYSL2//RAB21//WNT7A//MYCN//DLL1//NTF3//YWHAG//NREP//DDX6//ID2//ISL1//DNMT3B//ETV5//MMD//FOXA1//NEUROD1//NEUROG1//ZEB1//GDF5//ADNP//NRG1//TNFRSF12A//ULK2//WNT3//NOG//TNFRSF21//WDR1//PPARG//MYOG//FMR1//HECW2//FXR1//PLXNA4//BMPR2//SEMA4C//POU4F1//EFNA1//POU3F2//TRAK2//SYNGAP1//ROBO2//SLIT2//DBN1//CAMSAP2//MYB//PDE5A//IGF1//EDN1//CPEB3//ZMYND8//IL2//MAPK6//DLG5//ARC//LRP8//LDLR//CRKL//FLNA//TNF//YTHDF2//SMURF1//SYT1//PLAA//CLOCK//FBXW7// |
| GO:0045664 | regulation of neuron differentiation | Biological process | 67 | 600 | 902 | 17653 | 2.18542313377679 | 1.31361425929224e-09 | 2.33788769357722e-07 | 8.88153214592932 | 0.0742793791574279 | PTEN//SNAP25//STMN2//ADCYAP1//NPTN//NCK1//SERPINI1//RAP1A//SCN1B//RAPGEF2//LPAR1//EFNB2//MYLIP//PTPRG//SPOCK1//TSC1//BAG5//EDNRB//DPYSL2//RAB21//WNT7A//MYCN//DLL1//DDX6//GLI3//ID2//ISL1//DNMT3B//ETV5//MMD//FOXA1//NEUROD1//NEUROG1//ZEB1//GDF5//ADNP//NRG1//TNFRSF12A//ULK2//WNT3//FMR1//HECW2//FXR1//PLXNA4//BMPR2//SEMA4C//EFNA1//POU3F2//TRAK2//SYNGAP1//ROBO2//SLIT2//DBN1//CAMSAP2//CPEB3//ZMYND8//IL2//MAPK6//DLG5//ARC//LRP8//SMURF1//SYT1//PLAA//NTF3//YWHAG//NREP// |
| GO:0009891 | positive regulation of biosynthetic process | Biological process | 151 | 1841 | 902 | 17653 | 1.60522214500699 | 1.80833253433369e-09 | 3.13583408453814e-07 | 8.74272170408917 | 0.167405764966741 | EP300//IGF1//CCL21//CNEP1R1//LDLR//EDN1//ARPP19//CPEB3//FMR1//SERP1//PRR16//MAPK1//SOX4//FXR1//CREBL2//ESR1//ETS1//ATF5//FOXF1//FOXF2//FLI1//PHF8//NR5A2//BAMBI//GLI3//ATAD2//MDFIC//ID2//IRF1//AR//JUN//LRP6//MYB//MYBL1//MYCN//NEUROD1//NFE2//NPAS2//NPAT//OTX2//PPARG//TRERF1//RB1//HIVEP3//RORA//PRDM16//SMARCA2//SS18//TFAP2A//TNF//UBE2V1//WNT7A//WT1//TBL1XR1//TEAD2//TP63//RUNX3//FUBP3//ACVR1//CLOCK//ZNF516//ADRB1//PTHLH//CALM2//ADNP//CCT6A//E2F8//INSIG2//NCK1//EREG//CD3E//SIRT1//TBK1//IFNG//PRKAA1//MID1IP1//ELOVL5//C1QTNF2//ZBTB18//CEBPB//ADCYAP1//PPARGC1B//CREBRF//E2F5//S1PR1//ELK3//EN1//ETV5//MED19//ZFPM2//KPNA6//AGO1//DLL1//GTF2H1//CNOT7//H2AFZ//NRG1//FOXA1//IL2//IRF2//ISL1//MAF//MEF2A//MEOX2//MET//MYOG//PPP1R12A//NEUROG1//NFIA//NFIB//NFIC//NHLH2//NR4A2//PAX5//IER5//ARMCX3//KLF13//ARID4B//PKNOX1//CYTL1//POU3F2//POU4F1//EGLN1//PPP3R1//FOXJ2//CCNL1//MAVS//RAF1//ATXN7//BMPR2//ZEB1//TFE3//KLF10//TXK//YES1//FOSL1//PCGF5//KLF7//CBFB//CCNT2//NOG//RPS6KA5//ST18//BOLL//KDR//CCL2//PTGES3//RAB3GAP1//YTHDF2//FBXW7//NPNT// |
| GO:0031328 | positive regulation of cellular biosynthetic process | Biological process | 149 | 1812 | 902 | 17653 | 1.60931129254098 | 1.99454428237176e-09 | 3.35595319497572e-07 | 8.70015631714997 | 0.165188470066519 | EP300//IGF1//CCL21//CNEP1R1//LDLR//ARPP19//CPEB3//FMR1//SERP1//PRR16//MAPK1//SOX4//FXR1//CREBL2//ESR1//ETS1//ATF5//FOXF1//FOXF2//FLI1//PHF8//NR5A2//BAMBI//GLI3//ATAD2//MDFIC//ID2//IRF1//AR//JUN//LRP6//MYB//MYBL1//MYCN//NEUROD1//NFE2//NPAS2//NPAT//OTX2//PPARG//TRERF1//RB1//HIVEP3//RORA//PRDM16//SMARCA2//SS18//TFAP2A//TNF//UBE2V1//WNT7A//WT1//TBL1XR1//TEAD2//TP63//RUNX3//FUBP3//ACVR1//CLOCK//ZNF516//ADRB1//PTHLH//CALM2//ADNP//CCT6A//E2F8//INSIG2//NCK1//EREG//CD3E//TBK1//EDN1//IFNG//MID1IP1//ELOVL5//C1QTNF2//PRKAA1//ZBTB18//CEBPB//ADCYAP1//PPARGC1B//CREBRF//E2F5//S1PR1//ELK3//EN1//ETV5//MED19//SIRT1//ZFPM2//KPNA6//AGO1//DLL1//GTF2H1//CNOT7//H2AFZ//NRG1//FOXA1//IL2//IRF2//ISL1//MAF//MEF2A//MEOX2//MET//MYOG//PPP1R12A//NEUROG1//NFIA//NFIB//NFIC//NHLH2//NR4A2//PAX5//IER5//ARMCX3//KLF13//ARID4B//PKNOX1//CYTL1//POU3F2//POU4F1//EGLN1//PPP3R1//FOXJ2//CCNL1//MAVS//RAF1//ATXN7//BMPR2//ZEB1//TFE3//KLF10//TXK//YES1//FOSL1//PCGF5//KLF7//CBFB//CCNT2//NOG//RPS6KA5//ST18//BOLL//PTGES3//RAB3GAP1//YTHDF2//FBXW7//NPNT// |
| GO:0050794 | regulation of cellular process | Biological process | 648 | 11074 | 902 | 17653 | 1.14520298239579 | 2.03451250915281e-09 | 3.35595319497572e-07 | 8.69153963555297 | 0.71840354767184 | USP28//CLOCK//GADD45A//GTF2H1//PTEN//CCNT2//DIRAS3//NPAT//TSHZ1//ZBTB18//CEBPB//TCERG1//STRAP//KLF12//GSC//ARX//DNMT3B//EDN1//EDNRB//EFNA1//CC2D1B//EN1//EP300//ESR1//JAZF1//CPEB3//FOXF1//MYT1L//SIN3B//SIRT1//ZFPM2//DNAJB5//GLI3//CNOT7//HIC1//FOXA1//HSBP1//ID2//IFNG//IRF2//AR//ISL1//JARID2//JUN//MAF//MDM4//MEF2A//MYB//NFIA//NFIB//NFIC//NR4A2//PAX5//KLF3//POU4F1//PPARG//ZBTB4//RB1//RBBP8//CCND1//RNF2//SARS//PRDM16//SMARCA2//SUV39H1//ZEB1//TFAP2A//TGIF1//KLF10//TNF//UBE2I//WT1//ZNF217//LRP8//BHLHE41//TBL1XR1//E2F8//ARID5B//BHLHE40//TP63//RUNX3//NOG//FOXP2//PHF14//KCNH4//RANBP9//SPRED1//EREG//RASGEF1A//NRG1//IL2//MET//MAPK1//MAPK6//PSMA2//PSMD12//RAF1//RASA1//CCL2//SYNGAP1//RAPGEF2//CRKL//TAOK1//RAP1A//C1QTNF2//LPAR1//IGF1//NTF3//PIK3CB//PRKAA1//C5AR1//UBE2V1//DUSP1//PPP2CA//MBNL2//RBFOX3//FMR1//MBNL1//RBM25//SRSF2//SRSF6//SEPSECS//SECISBP2L//C8ORF44-SGK3//SGK2//SGK3//NANOS1//IGFBP3//SGK1//SPOCK1//PMAIP1//BCL2A1//EPS15//NSG1//VAMP3//SOCS1//LRP6//PRR5L//PPP1R15B//SLIT2//NPTN//KDR//MAVS//CCND2//SULF1//GJA1//F3//RICTOR//PPP1R16B//BMPR2//VEGFB//VASH2//CBFB//FZD3//MYCN//TGFBR2//PRKACB//SKP1//RPS6KA5//IRAK4//BAG6//CR2//CCL8//EDN2//CXCL9//SKAP2//FOXP1//S1PR1//SPHK2//E2F5//IRF1//CDK15//TSC1//EPS8//APCDD1//CCNY//HBP1//GRK6//STRN//WNT3//WNT7A//CXXC4//WNT10A//TNRC6B//AGO1//PPP3R1//FZD6//CNIH1//CDS1//PTGES3//IQGAP2//ADCY3//RALBP1//TMED1//RASSF8//CHRNB4//ARAP2//CLCN6//CNGA3//CNTFR//CREBL2//DPYSL2//DTNA//ELK3//CD2AP//GABRA5//APPL1//ATP2C1//GLRB//LRP12//HIVEP2//NR3C2//PPP1R12A//HPCAL4//GULP1//RASD1//KCNK10//CYTL1//NDFIP2//PPP2R5E//PSD//ARRDC3//MIER1//HIVEP3//SMOC1//SMOC2//ZYX//GLRA3//SNX27//SYDE2//PPP1R1B//PPFIA1//OR6A2//PDE5A//GPRC5A//DLG5//PLAA//ARHGAP12//GTPBP1//FEZ2//ZNF516//CD69//ULK2//SNX17//ROR1//PTPRG//TXK//YES1//CD3E//CD8A//AKAP11//DGKH//NRBP1//ARHGEF4//PRKAR2B//STK33//SS18//ANP32A//DGKE//RALGPS1//PALM2//FBXW7//ERRFI1//SOCS5//NUP54//NLN//DNAJC16//GLRX//NHLRC2//ESCO2//BAHD1//H2AFZ//HIPK3//ZMYND11//PNRC1//ZNF526//PPARGC1B//ZNF800//ARID2//ELAVL2//ATF5//ZFP30//ELL2//KDM2A//MYCBP2//FLII//ZMYND8//CNOT6L//NR5A2//ZNF521//ZBTB11//GTF2A1//GTF2E1//NRBF2//HMGB3//ZNF680//ZNF662//AFF1//MLLT6//OGG1//ASCC1//PHF20L1//COMMD10//BNC2//ZNF532//SLC2A4RG//PAK6//PHTF2//CNOT6//ZBTB26//RORA//SOX4//BTF3//BTG1//ZNF3//ZNF131//ZNF227//CSDE1//ZNF655//SAP30L//SNIP1//TEAD2//ZNF367//EMX2//ETS1//ETV5//FOXF2//FLI1//ADNP//EPC2//ATAD2//HOXA3//HOXD1//BOLA3//MYBL1//NEUROG1//NFE2//NHLH2//KLF13//ARID4B//BRWD1//POU3F2//MED9//TRERF1//FOXJ2//PURA//RFX7//TRAK2//SMARCD2//SOX5//MED22//ZNF708//ZSCAN5A//FOSL1//LCOR//KLF7//BDP1//DDX6//KBTBD8//GNAQ//NCK1//GSKIP//YWHAG//SOCS3//PPM1F//EGLN3//CASP2//STMN2//CAMSAP1//CAMSAP2//MID1//MID1IP1//NAV3//RRAGD//CUL5//NABP1//TTK//CDC14A//CBLL1//PLXNA4//CRHR1//IGSF3//TSPAN11//PIK3CA//SMURF1//GDF5//ACVR1//NMUR1//RAPGEF4//CHRM2//ADCYAP1//ADRB1//TAPT1//FRZB//OR4N4//GRM3//KCNK2//RHOB//RGS7BP//PTHLH//CCL21//ITSN1//SOS2//CALM2//GPR55//FLNA//ANO1//HOMER1//GRIK2//KCNB1//NXPH3//SORCS1//SORCS2//SUSD5//DLL1//TMEM17//EVC//DZIP1//ITGB8//CCM2//RCAN3//SAMD14//TBK1//COPS8//G3BP2//MDFIC//NMI//NEUROD1//RAB18//RGL1//RND3//ARHGAP1//RIN2//DOCK10//ARHGAP6//CDK19//SLK//TRPC3//SLC25A6//MARCKS//SNAP25//ARL6IP1//KLHL20//ZFAND6//BNIP2//TMBIM6//GIT2//DNAJA2//CACUL1//EFNB2//BAMBI//TOB1//TES//MYOG//ST18//CFDP1//WASF3//ANXA7//MSN//WDR1//TIA1//SHCBP1//OTX2//ADIPOR2//RWDD3//LASP1//SH3BGRL//NEDD1//CEP41//PI15//TXLNA//IFNA1//INHBB//OGN//BMP3//DRAM1//MTMR9//PPP4R2//PAN3//PFN2//NPNT//LDLRAD4//C5ORF30//TNFAIP3//CHMP3//VPS4B//CNEP1R1//LDLR//ABHD5//FKBP15//PLEKHF2//SERPINI1//SERPINB13//RECK//SCN1B//MYLIP//BAG5//PMEPA1//SYT1//UNC5B//ARPP19//SERP1//PRR16//FXR1//RAB12//SESN1//SNX5//TNFRSF21//PHF8//NPAS2//FUBP3//DNAJB6//ATXN1//BTAF1//NREP//CELF1//MEX3B//RAB21//RAB8B//SYT6//PLP2//SDC1//SH3D19//ARC//SPPL3//DENND1B//HECW2//SHISA2//EI24//FAM89B//SEMA4C//PTP4A1//TMEFF2//CLIC4//RAP2C//LEMD3//CTDSPL2//EGLN1//CFL2//CREBRF//CLIP1//TBC1D12//C2CD5//SPOPL//SLC9A6//RHEBL1//MOB1B//RASSF2//FAM83D//IQSEC2//FBXO8//AGFG1//DCP2//CCT6A//SLC6A1//ANKIB1//LONRF3//LONRF1//KIF13A//RAB30//RAB9B//RAB28//RAP1B//MEMO1//KLHL42//INSIG2//FRMD6//UBA5//EDARADD//TNFRSF12A//ACSL1//ARFIP1//PLEKHG4B//ROBO2//GSTA2//SYBU//GFPT1//DNAJB9//ASNA1//SEC61A2//IER5//ATXN7//PHLDA3//USP27X//ZMAT3//FASTK//YTHDF2//UBXN2B//PEX5L//TMEM64//DLK2//MMD//TFE3//ELOVL5//CCNL1//RNF167//MED19//KPNA6//MEOX2//ARMCX3//PKNOX1//PCGF5//PAIP2//BOLL//HPRT1//PPP2CB//GIGYF1//MORC3//RIMS4//DBN1//KCNJ10//RAB3GAP1//DHRS3//SIRPA//TUB//PXK//GCSAML//NOVA1//ZER1//DCUN1D4//GPM6A//TMOD3//TMOD1//OTUD3//SLITRK3//PRICKLE2//SLC30A6//QKI//FIBIN//KCNJ2//MIEF1//ANAPC15//SIVA1//CAST//BSN//LYPD6//CPEB2//DYNLL2//KCNE1//KCNIP4//KCNAB2//CRTAP//SERINC3//SEPT9//HSBP1L1//FAM168A//IER3IP1//CPEB1// |
| GO:0050767 | regulation of neurogenesis | Biological process | 76 | 732 | 902 | 17653 | 2.03195691420402 | 2.72176397750211e-09 | 4.38268804282066e-07 | 8.56514953810134 | 0.0842572062084257 | GLI3//FZD3//PTEN//SNAP25//STMN2//ADCYAP1//NPTN//NCK1//SERPINI1//RAP1A//SCN1B//RAPGEF2//LPAR1//EFNB2//MYLIP//PTPRG//SPOCK1//TSC1//BAG5//EDNRB//DPYSL2//RAB21//WNT7A//MYCN//DLL1//NTF3//YWHAG//NREP//DDX6//ID2//ISL1//DNMT3B//ETV5//MMD//FOXA1//NEUROD1//NEUROG1//ZEB1//GDF5//ADNP//NRG1//TNFRSF12A//ULK2//WNT3//NOG//TNFRSF21//WDR1//PPARG//FMR1//HECW2//FXR1//PLXNA4//BMPR2//SEMA4C//EFNA1//POU3F2//TRAK2//SYNGAP1//ROBO2//SLIT2//DBN1//CAMSAP2//MYB//CPEB3//ZMYND8//IL2//MAPK6//DLG5//ARC//LRP8//LDLR//SMURF1//SYT1//PLAA//FLNA//POU4F1// |
| GO:0048666 | neuron development | Biological process | 99 | 1060 | 902 | 17653 | 1.8278531983433 | 3.04496802155435e-09 | 4.6155585209669e-07 | 8.51641726399512 | 0.109756097560976 | SLITRK3//OGN//POU4F1//PRELP//WNT7A//SLIT2//RANBP9//ARX//DPYSL2//EFNA1//EFNB2//GLI3//OTX2//PIK3CA//PIK3CB//ENAH//MAPK1//ROBO2//SCN1B//WNT3//KLF7//RPS6KA5//NRXN3//FEZ2//NOG//PTEN//SNAP25//STMN2//ADCYAP1//NPTN//NCK1//SERPINI1//RAP1A//RAPGEF2//LPAR1//MYLIP//PTPRG//SPOCK1//TSC1//BAG5//EDNRB//GNAQ//STRN//SYNGAP1//CD3E//PLXNA4//ATF5//NR4A2//HPRT1//NFIB//RAB21//ISL1//JUN//NREP//CAMSAP1//SAMD14//GPM6A//LRP12//PSD//RB1//UNC5B//FZD3//ADNP//NRG1//TNFRSF12A//ID2//ULK2//SLC9A6//GJA1//NTF3//SGK1//MEF2A//TRAK2//FMR1//HECW2//FXR1//BMPR2//SEMA4C//RUNX3//POU3F2//DBN1//CAMSAP2//GABRA5//ACSL4//ARC//DOCK10//CPEB3//ZMYND8//IL2//MAPK6//DLG5//LRP8//NANOS1//SMURF1//SYT1//PLAA//EN1//MYT1L//NEUROD1// |
| GO:0010557 | positive regulation of macromolecule biosynthetic process | Biological process | 141 | 1698 | 902 | 17653 | 1.62514984369246 | 3.1364676699267e-09 | 4.6155585209669e-07 | 8.50355918472311 | 0.156319290465632 | EP300//IGF1//CCL21//EDN1//CPEB3//FMR1//SERP1//PRR16//MAPK1//SOX4//FXR1//CREBL2//ESR1//ETS1//ATF5//FOXF1//FOXF2//FLI1//PHF8//NR5A2//BAMBI//GLI3//ATAD2//MDFIC//ID2//IRF1//AR//JUN//LRP6//MYB//MYBL1//MYCN//NEUROD1//NFE2//NPAS2//NPAT//OTX2//PPARG//TRERF1//RB1//HIVEP3//RORA//PRDM16//SMARCA2//SS18//TFAP2A//TNF//UBE2V1//WNT7A//WT1//TBL1XR1//TEAD2//TP63//RUNX3//FUBP3//ACVR1//CLOCK//ZNF516//CCT6A//E2F8//INSIG2//NCK1//EREG//CD3E//SIRT1//TBK1//C1QTNF2//ZBTB18//CEBPB//ADCYAP1//PPARGC1B//CREBRF//E2F5//S1PR1//ELK3//EN1//ETV5//MED19//ZFPM2//KPNA6//AGO1//DLL1//GTF2H1//CNOT7//H2AFZ//NRG1//FOXA1//IL2//IRF2//ISL1//MAF//MEF2A//MEOX2//MET//MYOG//PPP1R12A//NEUROG1//NFIA//NFIB//NFIC//NHLH2//NR4A2//PAX5//IER5//ARMCX3//KLF13//ARID4B//PKNOX1//CYTL1//POU3F2//POU4F1//EGLN1//PPP3R1//FOXJ2//CCNL1//MAVS//RAF1//ATXN7//BMPR2//ZEB1//TFE3//KLF10//TXK//YES1//FOSL1//PCGF5//KLF7//CBFB//CCNT2//NOG//RPS6KA5//ST18//BOLL//KDR//CCL2//PTGES3//RAB3GAP1//PRKAA1//YTHDF2//FBXW7//NPNT// |
| GO:0045893 | positive regulation of transcription, DNA-templated | Biological process | 125 | 1450 | 902 | 17653 | 1.68715115834544 | 3.13937146184352e-09 | 4.6155585209669e-07 | 8.50315729395772 | 0.138580931263858 | EP300//ATAD2//INSIG2//ESR1//PHF8//ZBTB18//CEBPB//ADCYAP1//PPARGC1B//CREBRF//E2F5//S1PR1//EDN1//ELK3//EN1//ETS1//ETV5//MED19//ATF5//FOXF1//FOXF2//FLI1//SIRT1//ZFPM2//KPNA6//NR5A2//AGO1//GLI3//DLL1//TBK1//GTF2H1//CNOT7//H2AFZ//NRG1//FOXA1//IGF1//IL2//IRF1//IRF2//AR//ISL1//JUN//LRP6//MAF//MEF2A//MEOX2//MET//MYB//MYBL1//MYCN//MYOG//PPP1R12A//NCK1//NEUROD1//NEUROG1//NFIA//NFIB//NFIC//NHLH2//NPAS2//NR4A2//OTX2//PAX5//IER5//ARMCX3//KLF13//ARID4B//PKNOX1//CYTL1//POU3F2//POU4F1//EGLN1//PPARG//PPP3R1//FOXJ2//CCNL1//MAVS//RAF1//RB1//RORA//ATXN7//PRDM16//BMPR2//SMARCA2//SOX4//SS18//ZEB1//TFAP2A//TFE3//KLF10//TNF//TXK//WNT7A//WT1//YES1//TBL1XR1//E2F8//FOSL1//PCGF5//TEAD2//KLF7//TP63//CBFB//FUBP3//ACVR1//CCNT2//NOG//RPS6KA5//CLOCK//ST18//ID2//PRKAA1//FBXW7//NPNT//CREBL2//BAMBI//MDFIC//NFE2//NPAT//TRERF1//MAPK1//HIVEP3//UBE2V1//RUNX3//ZNF516// |
| GO:1903508 | positive regulation of nucleic acid-templated transcription | Biological process | 125 | 1450 | 902 | 17653 | 1.68715115834544 | 3.13937146184352e-09 | 4.6155585209669e-07 | 8.50315729395772 | 0.138580931263858 | EP300//CREBL2//ESR1//ETS1//ATF5//FOXF1//FOXF2//FLI1//PHF8//NR5A2//BAMBI//GLI3//ATAD2//MDFIC//ID2//IGF1//IRF1//AR//JUN//LRP6//MYB//MYBL1//MYCN//NEUROD1//NFE2//NPAS2//NPAT//OTX2//PPARG//TRERF1//MAPK1//RB1//HIVEP3//RORA//PRDM16//SMARCA2//SOX4//SS18//TFAP2A//TNF//UBE2V1//WNT7A//WT1//TBL1XR1//TEAD2//TP63//RUNX3//FUBP3//ACVR1//CLOCK//ZNF516//INSIG2//ZBTB18//CEBPB//ADCYAP1//PPARGC1B//CREBRF//E2F5//S1PR1//EDN1//ELK3//EN1//ETV5//MED19//SIRT1//ZFPM2//KPNA6//AGO1//DLL1//TBK1//GTF2H1//CNOT7//H2AFZ//NRG1//FOXA1//IL2//IRF2//ISL1//MAF//MEF2A//MEOX2//MET//MYOG//PPP1R12A//NCK1//NEUROG1//NFIA//NFIB//NFIC//NHLH2//NR4A2//PAX5//IER5//ARMCX3//KLF13//ARID4B//PKNOX1//CYTL1//POU3F2//POU4F1//EGLN1//PPP3R1//FOXJ2//CCNL1//MAVS//RAF1//ATXN7//BMPR2//ZEB1//TFE3//KLF10//TXK//YES1//E2F8//FOSL1//PCGF5//KLF7//CBFB//CCNT2//NOG//RPS6KA5//ST18//PRKAA1//FBXW7//NPNT// |
| GO:1902680 | positive regulation of RNA biosynthetic process | Biological process | 125 | 1451 | 902 | 17653 | 1.68598840771943 | 3.2734718618737e-09 | 4.63877982765134e-07 | 8.48499138783669 | 0.138580931263858 | EP300//CREBL2//ESR1//ETS1//ATF5//FOXF1//FOXF2//FLI1//PHF8//NR5A2//BAMBI//GLI3//ATAD2//MDFIC//ID2//IGF1//IRF1//AR//JUN//LRP6//MYB//MYBL1//MYCN//NEUROD1//NFE2//NPAS2//NPAT//OTX2//PPARG//TRERF1//MAPK1//RB1//HIVEP3//RORA//PRDM16//SMARCA2//SOX4//SS18//TFAP2A//TNF//UBE2V1//WNT7A//WT1//TBL1XR1//TEAD2//TP63//RUNX3//FUBP3//ACVR1//CLOCK//ZNF516//INSIG2//ZBTB18//CEBPB//ADCYAP1//PPARGC1B//CREBRF//E2F5//S1PR1//EDN1//ELK3//EN1//ETV5//MED19//SIRT1//ZFPM2//KPNA6//AGO1//DLL1//TBK1//GTF2H1//CNOT7//H2AFZ//NRG1//FOXA1//IL2//IRF2//ISL1//MAF//MEF2A//MEOX2//MET//MYOG//PPP1R12A//NCK1//NEUROG1//NFIA//NFIB//NFIC//NHLH2//NR4A2//PAX5//IER5//ARMCX3//KLF13//ARID4B//PKNOX1//CYTL1//POU3F2//POU4F1//EGLN1//PPP3R1//FOXJ2//CCNL1//MAVS//RAF1//ATXN7//BMPR2//ZEB1//TFE3//KLF10//TXK//YES1//E2F8//FOSL1//PCGF5//KLF7//CBFB//CCNT2//NOG//RPS6KA5//ST18//PRKAA1//FBXW7//NPNT// |
| GO:1903506 | regulation of nucleic acid-templated transcription | Biological process | 265 | 3766 | 902 | 17653 | 1.37713825298828 | 3.29234706087926e-09 | 4.63877982765134e-07 | 8.48249439014261 | 0.293791574279379 | NPAT//TSHZ1//ZBTB18//CEBPB//TCERG1//STRAP//KLF12//GSC//ARX//DNMT3B//EDN1//EDNRB//EFNA1//CC2D1B//EN1//EP300//ESR1//JAZF1//CPEB3//FOXF1//MYT1L//SIN3B//SIRT1//ZFPM2//DNAJB5//GLI3//CNOT7//HIC1//FOXA1//HSBP1//ID2//IFNG//IRF2//AR//ISL1//JARID2//JUN//MAF//MDM4//MEF2A//MYB//NFIA//NFIB//NFIC//NR4A2//PAX5//KLF3//POU4F1//PPARG//ZBTB4//RB1//RBBP8//CCND1//RNF2//SARS//PRDM16//SMARCA2//SUV39H1//ZEB1//TFAP2A//TGIF1//KLF10//TNF//UBE2I//WT1//ZNF217//LRP8//BHLHE41//TBL1XR1//E2F8//ARID5B//BHLHE40//TP63//RUNX3//NOG//FOXP2//PHF14//BAHD1//H2AFZ//HIPK3//ZMYND11//PNRC1//ZNF526//PPARGC1B//ZNF800//ARID2//ELAVL2//ATF5//ZFP30//ELL2//KDM2A//MYCBP2//FLII//ZMYND8//CNOT6L//NR5A2//ZNF521//ZBTB11//GTF2A1//GTF2E1//NRBF2//HIVEP2//HMGB3//ZNF680//ZNF662//AFF1//MLLT6//OGG1//ASCC1//PHF20L1//COMMD10//BNC2//PPP2CA//ZNF532//SLC2A4RG//PAK6//PHTF2//CNOT6//ZBTB26//HIVEP3//RORA//SOX4//BTF3//BTG1//UBE2V1//ZNF3//ZNF131//ZNF227//CSDE1//ZNF655//SAP30L//SNIP1//ANP32A//TEAD2//RPS6KA5//CLOCK//ZNF516//CREBL2//ZNF367//ELK3//EMX2//ETS1//ETV5//FOXF2//FLI1//ADNP//EPC2//HBP1//FOXP1//ATAD2//HOXA3//HOXD1//BOLA3//NR3C2//MYBL1//MYCN//NEUROG1//NFE2//NHLH2//KLF13//ARID4B//BRWD1//POU3F2//MED9//TRERF1//FOXJ2//MIER1//PURA//RFX7//TRAK2//SMARCD2//SOX5//MED22//TXK//ZNF708//ZSCAN5A//FOSL1//LCOR//KLF7//CBFB//BDP1//G3BP2//FLNA//PHF8//BAMBI//MDFIC//IGF1//IRF1//LRP6//NEUROD1//NPAS2//OTX2//MAPK1//SS18//WNT7A//FUBP3//ACVR1//DNAJB6//EREG//NRG1//RASD1//ATXN1//BTAF1//PPM1F//TOB1//SMURF1//BMP3//BMPR2//GDF5//SULF1//LEMD3//CTDSPL2//RWDD3//TNFAIP3//INSIG2//EGLN1//FZD6//ADCYAP1//CREBRF//E2F5//S1PR1//MED19//KPNA6//AGO1//DLL1//TBK1//GTF2H1//IL2//MEOX2//MET//MYOG//PPP1R12A//NCK1//IER5//ARMCX3//PKNOX1//CYTL1//PPP3R1//CCNL1//MAVS//RAF1//ATXN7//TFE3//YES1//PCGF5//CCNT2//ST18//C8ORF44-SGK3//SGK3//SGK1//PTEN//RHEBL1//ROR1//IRAK4//EGLN3//PSMA2//PSMD12//PRKAA1//HSBP1L1//SRSF2//TMBIM6//FBXW7//NPNT//RAP2C//NMI// |
| GO:2001141 | regulation of RNA biosynthetic process | Biological process | 265 | 3774 | 902 | 17653 | 1.37421904100527 | 4.06986972177725e-09 | 5.61725080171011e-07 | 8.3904194924993 | 0.293791574279379 | NPAT//TSHZ1//ZBTB18//CEBPB//TCERG1//STRAP//KLF12//GSC//ARX//DNMT3B//EDN1//EDNRB//EFNA1//CC2D1B//EN1//EP300//ESR1//JAZF1//CPEB3//FOXF1//MYT1L//SIN3B//SIRT1//ZFPM2//DNAJB5//GLI3//CNOT7//HIC1//FOXA1//HSBP1//ID2//IFNG//IRF2//AR//ISL1//JARID2//JUN//MAF//MDM4//MEF2A//MYB//NFIA//NFIB//NFIC//NR4A2//PAX5//KLF3//POU4F1//PPARG//ZBTB4//RB1//RBBP8//CCND1//RNF2//SARS//PRDM16//SMARCA2//SUV39H1//ZEB1//TFAP2A//TGIF1//KLF10//TNF//UBE2I//WT1//ZNF217//LRP8//BHLHE41//TBL1XR1//E2F8//ARID5B//BHLHE40//TP63//RUNX3//NOG//FOXP2//PHF14//BAHD1//H2AFZ//HIPK3//ZMYND11//PNRC1//ZNF526//PPARGC1B//ZNF800//ARID2//ELAVL2//ATF5//ZFP30//ELL2//KDM2A//MYCBP2//FLII//ZMYND8//CNOT6L//NR5A2//ZNF521//ZBTB11//GTF2A1//GTF2E1//NRBF2//HIVEP2//HMGB3//ZNF680//ZNF662//AFF1//MLLT6//OGG1//ASCC1//PHF20L1//COMMD10//BNC2//PPP2CA//ZNF532//SLC2A4RG//PAK6//PHTF2//CNOT6//ZBTB26//HIVEP3//RORA//SOX4//BTF3//BTG1//UBE2V1//ZNF3//ZNF131//ZNF227//CSDE1//ZNF655//SAP30L//SNIP1//ANP32A//TEAD2//RPS6KA5//CLOCK//ZNF516//CREBL2//ZNF367//ELK3//EMX2//ETS1//ETV5//FOXF2//FLI1//ADNP//EPC2//HBP1//FOXP1//ATAD2//HOXA3//HOXD1//BOLA3//NR3C2//MYBL1//MYCN//NEUROG1//NFE2//NHLH2//KLF13//ARID4B//BRWD1//POU3F2//MED9//TRERF1//FOXJ2//MIER1//PURA//RFX7//TRAK2//SMARCD2//SOX5//MED22//TXK//ZNF708//ZSCAN5A//FOSL1//LCOR//KLF7//CBFB//BDP1//G3BP2//FLNA//PHF8//BAMBI//MDFIC//IGF1//IRF1//LRP6//NEUROD1//NPAS2//OTX2//MAPK1//SS18//WNT7A//FUBP3//ACVR1//DNAJB6//EREG//NRG1//RASD1//ATXN1//BTAF1//PPM1F//TOB1//SMURF1//BMP3//BMPR2//GDF5//SULF1//LEMD3//CTDSPL2//RWDD3//TNFAIP3//INSIG2//EGLN1//FZD6//ADCYAP1//CREBRF//E2F5//S1PR1//MED19//KPNA6//AGO1//DLL1//TBK1//GTF2H1//IL2//MEOX2//MET//MYOG//PPP1R12A//NCK1//IER5//ARMCX3//PKNOX1//CYTL1//PPP3R1//CCNL1//MAVS//RAF1//ATXN7//TFE3//YES1//PCGF5//CCNT2//ST18//C8ORF44-SGK3//SGK3//SGK1//PTEN//RHEBL1//ROR1//IRAK4//EGLN3//PSMA2//PSMD12//PRKAA1//RAP2C//NMI//HSBP1L1//SRSF2//TMBIM6//FBXW7//NPNT// |
| GO:0032774 | RNA biosynthetic process | Biological process | 274 | 3940 | 902 | 17653 | 1.36102569585917 | 5.04982478865478e-09 | 6.70898383094395e-07 | 8.29672369012698 | 0.303769401330377 | NPAT//TSHZ1//ZBTB18//CEBPB//TCERG1//STRAP//KLF12//GSC//ARX//DNMT3B//EDN1//EDNRB//EFNA1//CC2D1B//EN1//EP300//ESR1//JAZF1//CPEB3//FOXF1//MYT1L//SIN3B//SIRT1//ZFPM2//DNAJB5//GLI3//CNOT7//HIC1//FOXA1//HSBP1//ID2//IFNG//IRF2//AR//ISL1//JARID2//JUN//MAF//MDM4//MEF2A//MYB//NFIA//NFIB//NFIC//NR4A2//PAX5//KLF3//POU4F1//PPARG//ZBTB4//RB1//RBBP8//CCND1//RNF2//SARS//PRDM16//SMARCA2//SUV39H1//ZEB1//TFAP2A//TGIF1//KLF10//TNF//UBE2I//WT1//ZNF217//LRP8//BHLHE41//TBL1XR1//E2F8//ARID5B//BHLHE40//TP63//RUNX3//NOG//FOXP2//PHF14//GTF2E1//GTF2H1//CREBRF//ELK3//ETV5//ATF5//FOXF2//FLI1//GTF2A1//HIVEP2//IRF1//MEOX2//MYBL1//MYCN//NEUROD1//NHLH2//OTX2//KLF13//PKNOX1//POU3F2//FOXJ2//HIVEP3//BMPR2//SOX4//SOX5//BTF3//TFE3//TXK//FOSL1//KLF7//CBFB//FUBP3//CCNT2//NMI//CLOCK//ZNF516//POLR3H//SRSF2//SRSF6//SLBP//BAHD1//H2AFZ//ZMYND11//PNRC1//ZNF526//CREBL2//ZNF800//E2F5//ZNF367//ARID2//MED19//ZFP30//KDM2A//MYCBP2//PHF8//FLII//ADNP//ZMYND8//CNOT6L//ZNF521//EPC2//AGO1//HBP1//FOXP1//ZBTB11//ATAD2//MDFIC//HMGB3//HOXA3//HOXD1//ZNF680//ZNF662//NEUROG1//NFE2//NPAS2//ASCC1//IER5//COMMD10//ARID4B//BRWD1//BNC2//MED9//ZNF532//PRKAA1//TRERF1//MAPK1//SLC2A4RG//CCNL1//PHTF2//CNOT6//ZBTB26//MIER1//PURA//ATXN1//ATXN7//SMARCD2//SS18//MED22//ZNF3//ZNF708//ZNF131//ZNF227//ZNF655//ZSCAN5A//SAP30L//ANP32A//PPP1R1B//PCGF5//LCOR//ST18//HIPK3//PPARGC1B//ELAVL2//ELL2//NR5A2//NRBF2//AFF1//MLLT6//OGG1//PHF20L1//PPP2CA//PAK6//RORA//BTG1//UBE2V1//CSDE1//SNIP1//TEAD2//RPS6KA5//EMX2//ETS1//BOLA3//NR3C2//RFX7//TRAK2//BDP1//NRBP1//G3BP2//EREG//FLNA//BAMBI//IGF1//LRP6//WNT7A//ACVR1//DNAJB6//NRG1//RASD1//BTAF1//PPM1F//TOB1//SMURF1//BMP3//GDF5//SULF1//LEMD3//CTDSPL2//RWDD3//TNFAIP3//INSIG2//MYOG//C5AR1//INTS8//NABP1//EGLN1//FZD6//ADCYAP1//S1PR1//KPNA6//DLL1//TBK1//IL2//MET//PPP1R12A//NCK1//ARMCX3//CYTL1//PPP3R1//MAVS//RAF1//YES1//C8ORF44-SGK3//SGK3//SGK1//PTEN//RHEBL1//ROR1//IRAK4//EGLN3//PSMA2//PSMD12//SEPSECS//RAP2C//HSBP1L1//TMBIM6//FBXW7//NPNT// |
| GO:0045935 | positive regulation of nucleobase-containing compound metabolic process | Biological process | 143 | 1742 | 902 | 17653 | 1.60657080451402 | 5.05926623359665e-09 | 6.70898383094395e-07 | 8.29591246612619 | 0.158536585365854 | EP300//CREBL2//ESR1//ETS1//ATF5//FOXF1//FOXF2//FLI1//PHF8//NR5A2//BAMBI//GLI3//ATAD2//MDFIC//ID2//IGF1//IRF1//AR//JUN//LRP6//MYB//MYBL1//MYCN//NEUROD1//NFE2//NPAS2//NPAT//OTX2//PPARG//TRERF1//MAPK1//RB1//HIVEP3//RORA//PRDM16//SMARCA2//SOX4//SS18//TFAP2A//TNF//UBE2V1//WNT7A//WT1//TBL1XR1//TEAD2//TP63//RUNX3//FUBP3//ACVR1//CLOCK//ZNF516//CALM2//CXCL9//ADRB1//PTHLH//ADNP//CCT6A//E2F8//INSIG2//SIRT1//EREG//PRKAA1//ZBTB18//CEBPB//ADCYAP1//PPARGC1B//CREBRF//E2F5//S1PR1//EDN1//ELK3//EN1//ETV5//MED19//ZFPM2//KPNA6//AGO1//DLL1//TBK1//GTF2H1//CNOT7//H2AFZ//NRG1//FOXA1//IL2//IRF2//ISL1//MAF//MEF2A//MEOX2//MET//MYOG//PPP1R12A//NCK1//NEUROG1//NFIA//NFIB//NFIC//NHLH2//NR4A2//PAX5//IER5//ARMCX3//KLF13//ARID4B//PKNOX1//CYTL1//POU3F2//POU4F1//EGLN1//PPP3R1//FOXJ2//CCNL1//MAVS//RAF1//ATXN7//BMPR2//ZEB1//TFE3//KLF10//TXK//YES1//FOSL1//PCGF5//KLF7//CBFB//CCNT2//NOG//RPS6KA5//ST18//PTGES3//TOB1//CPEB3//TNRC6B//PRR5L//GTPBP1//CNOT6L//YTHDF2//QKI//NANOS1//FAM168A//FBXW7//NPNT// |
| GO:0097659 | nucleic acid-templated transcription | Biological process | 273 | 3924 | 902 | 17653 | 1.36158773910508 | 5.25930639355714e-09 | 6.76904632724031e-07 | 8.27907152757881 | 0.302660753880266 | NPAT//TSHZ1//ZBTB18//CEBPB//TCERG1//STRAP//KLF12//GSC//ARX//DNMT3B//EDN1//EDNRB//EFNA1//CC2D1B//EN1//EP300//ESR1//JAZF1//CPEB3//FOXF1//MYT1L//SIN3B//SIRT1//ZFPM2//DNAJB5//GLI3//CNOT7//HIC1//FOXA1//HSBP1//ID2//IFNG//IRF2//AR//ISL1//JARID2//JUN//MAF//MDM4//MEF2A//MYB//NFIA//NFIB//NFIC//NR4A2//PAX5//KLF3//POU4F1//PPARG//ZBTB4//RB1//RBBP8//CCND1//RNF2//SARS//PRDM16//SMARCA2//SUV39H1//ZEB1//TFAP2A//TGIF1//KLF10//TNF//UBE2I//WT1//ZNF217//LRP8//BHLHE41//TBL1XR1//E2F8//ARID5B//BHLHE40//TP63//RUNX3//NOG//FOXP2//PHF14//GTF2E1//GTF2H1//CREBRF//ELK3//ETV5//ATF5//FOXF2//FLI1//GTF2A1//HIVEP2//IRF1//MEOX2//MYBL1//MYCN//NEUROD1//NHLH2//OTX2//KLF13//PKNOX1//POU3F2//FOXJ2//HIVEP3//BMPR2//SOX4//SOX5//BTF3//TFE3//TXK//FOSL1//KLF7//CBFB//FUBP3//CCNT2//NMI//CLOCK//ZNF516//POLR3H//SRSF2//SRSF6//SLBP//BAHD1//H2AFZ//ZMYND11//PNRC1//ZNF526//CREBL2//ZNF800//E2F5//ZNF367//ARID2//MED19//ZFP30//KDM2A//MYCBP2//PHF8//FLII//ADNP//ZMYND8//CNOT6L//ZNF521//EPC2//AGO1//HBP1//FOXP1//ZBTB11//ATAD2//MDFIC//HMGB3//HOXA3//HOXD1//ZNF680//ZNF662//NEUROG1//NFE2//NPAS2//ASCC1//IER5//COMMD10//ARID4B//BRWD1//BNC2//MED9//ZNF532//PRKAA1//TRERF1//MAPK1//SLC2A4RG//CCNL1//PHTF2//CNOT6//ZBTB26//MIER1//PURA//ATXN1//ATXN7//SMARCD2//SS18//MED22//ZNF3//ZNF708//ZNF131//ZNF227//ZNF655//ZSCAN5A//SAP30L//ANP32A//PPP1R1B//PCGF5//LCOR//ST18//HIPK3//PPARGC1B//ELAVL2//ELL2//NR5A2//NRBF2//AFF1//MLLT6//OGG1//PHF20L1//PPP2CA//PAK6//RORA//BTG1//UBE2V1//CSDE1//SNIP1//TEAD2//RPS6KA5//EMX2//ETS1//BOLA3//NR3C2//RFX7//TRAK2//BDP1//NRBP1//G3BP2//EREG//FLNA//BAMBI//IGF1//LRP6//WNT7A//ACVR1//DNAJB6//NRG1//RASD1//BTAF1//PPM1F//TOB1//SMURF1//BMP3//GDF5//SULF1//LEMD3//CTDSPL2//RWDD3//TNFAIP3//INSIG2//MYOG//C5AR1//INTS8//NABP1//EGLN1//FZD6//ADCYAP1//S1PR1//KPNA6//DLL1//TBK1//IL2//MET//PPP1R12A//NCK1//ARMCX3//CYTL1//PPP3R1//MAVS//RAF1//YES1//C8ORF44-SGK3//SGK3//SGK1//PTEN//RHEBL1//ROR1//IRAK4//EGLN3//PSMA2//PSMD12//RAP2C//HSBP1L1//TMBIM6//FBXW7//NPNT// |
| GO:0009889 | regulation of biosynthetic process | Biological process | 317 | 4707 | 902 | 17653 | 1.31803531749901 | 5.30473836084188e-09 | 6.76904632724031e-07 | 8.27533603141292 | 0.351441241685144 | NPAT//TSHZ1//ZBTB18//CEBPB//TCERG1//STRAP//KLF12//GSC//ARX//DNMT3B//EDN1//EDNRB//EFNA1//CC2D1B//EN1//EP300//ESR1//JAZF1//CPEB3//FOXF1//MYT1L//SIN3B//SIRT1//ZFPM2//DNAJB5//GLI3//CNOT7//HIC1//FOXA1//HSBP1//ID2//IFNG//IRF2//AR//ISL1//JARID2//JUN//MAF//MDM4//MEF2A//MYB//NFIA//NFIB//NFIC//NR4A2//PAX5//KLF3//POU4F1//PPARG//ZBTB4//RB1//RBBP8//CCND1//RNF2//SARS//PRDM16//SMARCA2//SUV39H1//ZEB1//TFAP2A//TGIF1//KLF10//TNF//UBE2I//WT1//ZNF217//LRP8//BHLHE41//TBL1XR1//E2F8//ARID5B//BHLHE40//TP63//RUNX3//NOG//FOXP2//PHF14//SEPSECS//SECISBP2L//NUP54//NLN//ESCO2//PPP2CA//BAHD1//H2AFZ//HIPK3//ZMYND11//PNRC1//ZNF526//PPARGC1B//ZNF800//ARID2//ELAVL2//ATF5//ZFP30//ELL2//KDM2A//MYCBP2//FLII//ZMYND8//CNOT6L//NR5A2//ZNF521//ZBTB11//GTF2A1//GTF2E1//NRBF2//HIVEP2//HMGB3//ZNF680//ZNF662//AFF1//MLLT6//OGG1//ASCC1//PHF20L1//COMMD10//BNC2//ZNF532//SLC2A4RG//PAK6//PHTF2//CNOT6//ZBTB26//HIVEP3//RORA//SOX4//BTF3//BTG1//UBE2V1//ZNF3//ZNF131//ZNF227//CSDE1//ZNF655//SAP30L//SNIP1//ANP32A//TEAD2//RPS6KA5//CLOCK//ZNF516//CREBL2//ZNF367//ELK3//EMX2//ETS1//ETV5//FOXF2//FLI1//ADNP//EPC2//HBP1//FOXP1//ATAD2//HOXA3//HOXD1//BOLA3//NR3C2//MYBL1//MYCN//NEUROG1//NFE2//NHLH2//KLF13//ARID4B//BRWD1//POU3F2//MED9//TRERF1//FOXJ2//MIER1//PURA//RFX7//TRAK2//SMARCD2//SOX5//MED22//TXK//ZNF708//ZSCAN5A//FOSL1//LCOR//KLF7//CBFB//BDP1//DDX6//TSC1//KBTBD8//GRM3//G3BP2//AGO1//IGF1//CCL21//CRHR1//CNEP1R1//LDLR//FBXW7//INSIG2//ARPP19//FMR1//SERP1//PRR16//MAPK1//FXR1//FLNA//PHF8//BAMBI//MDFIC//IRF1//LRP6//NEUROD1//NPAS2//OTX2//SS18//WNT7A//FUBP3//ACVR1//DNAJB6//EREG//NRG1//RASD1//ATXN1//BTAF1//PPM1F//TOB1//CELF1//NANOS1//TIA1//MEX3B//SMURF1//BMP3//BMPR2//GDF5//SULF1//LEMD3//CTDSPL2//ADRB1//PTHLH//CALM2//EGLN1//RWDD3//TNFAIP3//DCP2//CCT6A//ERRFI1//RAP1A//TNRC6B//NCK1//ADIPOR2//FZD6//CD3E//NMI//TBK1//PRKAA1//TMEFF2//MID1IP1//ELOVL5//C1QTNF2//MYOG//ADCYAP1//CREBRF//E2F5//S1PR1//MED19//KPNA6//DLL1//GTF2H1//IL2//MEOX2//MET//PPP1R12A//IER5//ARMCX3//PKNOX1//CYTL1//PPP3R1//CCNL1//MAVS//RAF1//ATXN7//TFE3//YES1//PCGF5//CCNT2//ST18//PAIP2//BOLL//RAPGEF2//INHBB//C8ORF44-SGK3//SGK3//SGK1//PTEN//RHEBL1//ROR1//IRAK4//KDR//CCL2//PTGES3//YTHDF2//QKI//EGLN3//PSMA2//PSMD12//CPEB2//RAB3GAP1//RAP2C//HSBP1L1//SRSF2//PPP1R15B//TMBIM6//DUSP1//GJA1//KCNK2//NPNT//CPEB1// |
| GO:0035239 | tube morphogenesis | Biological process | 86 | 887 | 902 | 17653 | 1.89752197921692 | 6.4263990301931e-09 | 8.04846974836962e-07 | 8.19203231158396 | 0.0953436807095344 | S1PR1//EFNA1//ELK3//EREG//UNC5B//SIRT1//LEMD3//CLIC4//HOXA3//JUN//KDR//RHOB//MEOX2//TNFRSF12A//PIK3CA//PKNOX1//PTEN//RORA//CCL2//VEGFB//WNT7A//EDN1//PPP3R1//TGFBR2//ACVR1//GJC1//FOXF1//ZFPM2//RASA1//WT1//CCM2//TEAD2//QKI//NPNT//GLI3//SOX4//LRP6//SEMA4C//PRKACB//TSC1//FZD3//FZD6//NOG//GJA1//DLL1//E2F8//EFNB2//SLIT2//SPRED1//SULF1//AGO1//PPARG//FOXJ2//SARS//ETS1//EGLN1//VASH2//F3//ISL1//ITGB8//BTG1//C5AR1//ADAM12//ID2//ZEB1//MET//MYCN//PIK3CB//TP63//RDH10//FOXA1//DLG5//AR//NFIB//ESR1//ETV5//ARID2//ADIPOR2//TNFAIP3//TNF//BMPR2//KLHL3//FOXP1//PPP1R16B//RAP1A//RAPGEF2// |
| GO:0010556 | regulation of macromolecule biosynthetic process | Biological process | 303 | 4467 | 902 | 17653 | 1.32751262398759 | 6.94406523175747e-09 | 8.53867512043196e-07 | 8.15838620809953 | 0.335920177383592 | NPAT//TSHZ1//ZBTB18//CEBPB//TCERG1//STRAP//KLF12//GSC//ARX//DNMT3B//EDN1//EDNRB//EFNA1//CC2D1B//EN1//EP300//ESR1//JAZF1//CPEB3//FOXF1//MYT1L//SIN3B//SIRT1//ZFPM2//DNAJB5//GLI3//CNOT7//HIC1//FOXA1//HSBP1//ID2//IFNG//IRF2//AR//ISL1//JARID2//JUN//MAF//MDM4//MEF2A//MYB//NFIA//NFIB//NFIC//NR4A2//PAX5//KLF3//POU4F1//PPARG//ZBTB4//RB1//RBBP8//CCND1//RNF2//SARS//PRDM16//SMARCA2//SUV39H1//ZEB1//TFAP2A//TGIF1//KLF10//TNF//UBE2I//WT1//ZNF217//LRP8//BHLHE41//TBL1XR1//E2F8//ARID5B//BHLHE40//TP63//RUNX3//NOG//FOXP2//PHF14//SEPSECS//SECISBP2L//ESCO2//PPP2CA//BAHD1//H2AFZ//HIPK3//ZMYND11//PNRC1//ZNF526//PPARGC1B//ZNF800//ARID2//ELAVL2//ATF5//ZFP30//ELL2//KDM2A//MYCBP2//FLII//ZMYND8//CNOT6L//NR5A2//ZNF521//ZBTB11//GTF2A1//GTF2E1//NRBF2//HIVEP2//HMGB3//ZNF680//ZNF662//AFF1//MLLT6//OGG1//ASCC1//PHF20L1//COMMD10//BNC2//ZNF532//SLC2A4RG//PAK6//PHTF2//CNOT6//ZBTB26//HIVEP3//RORA//SOX4//BTF3//BTG1//UBE2V1//ZNF3//ZNF131//ZNF227//CSDE1//ZNF655//SAP30L//SNIP1//ANP32A//TEAD2//RPS6KA5//CLOCK//ZNF516//CREBL2//ZNF367//ELK3//EMX2//ETS1//ETV5//FOXF2//FLI1//ADNP//EPC2//HBP1//FOXP1//ATAD2//HOXA3//HOXD1//BOLA3//NR3C2//MYBL1//MYCN//NEUROG1//NFE2//NHLH2//KLF13//ARID4B//BRWD1//POU3F2//MED9//TRERF1//FOXJ2//MIER1//PURA//RFX7//TRAK2//SMARCD2//SOX5//MED22//TXK//ZNF708//ZSCAN5A//FOSL1//LCOR//KLF7//CBFB//BDP1//DDX6//TSC1//KBTBD8//G3BP2//AGO1//IGF1//CCL21//FMR1//SERP1//PRR16//MAPK1//FXR1//FLNA//PHF8//BAMBI//MDFIC//IRF1//LRP6//NEUROD1//NPAS2//OTX2//SS18//WNT7A//FUBP3//ACVR1//DNAJB6//EREG//NRG1//RASD1//ATXN1//BTAF1//PPM1F//TOB1//CELF1//NANOS1//TIA1//MEX3B//SMURF1//BMP3//BMPR2//GDF5//SULF1//LEMD3//CTDSPL2//RWDD3//TNFAIP3//DCP2//CCT6A//FBXW7//INSIG2//TNRC6B//NCK1//ERRFI1//EGLN1//FZD6//CD3E//NMI//TBK1//TMEFF2//C1QTNF2//ADCYAP1//CREBRF//E2F5//S1PR1//MED19//KPNA6//DLL1//GTF2H1//IL2//MEOX2//MET//MYOG//PPP1R12A//IER5//ARMCX3//PKNOX1//CYTL1//PPP3R1//CCNL1//MAVS//RAF1//ATXN7//TFE3//YES1//PCGF5//CCNT2//ST18//PAIP2//BOLL//PRKAA1//INHBB//C8ORF44-SGK3//SGK3//SGK1//PTEN//RHEBL1//ROR1//IRAK4//KDR//CCL2//PTGES3//NUP54//YTHDF2//QKI//EGLN3//PSMA2//PSMD12//CPEB2//RAB3GAP1//RAP2C//HSBP1L1//SRSF2//PPP1R15B//TMBIM6//DUSP1//GJA1//KCNK2//NPNT//CPEB1// |
| GO:0010646 | regulation of cell communication | Biological process | 249 | 3522 | 902 | 17653 | 1.3836364014097 | 8.23853223910692e-09 | 9.94035262455978e-07 | 8.08415015447228 | 0.276053215077605 | GADD45A//TNF//CRKL//TAOK1//RAF1//RAP1A//C1QTNF2//LPAR1//NRG1//IGF1//NTF3//PIK3CB//PRKAA1//MAPK1//C5AR1//UBE2V1//SPRED1//DUSP1//PPP2CA//CBFB//EREG//FBXW7//ERRFI1//GPRC5A//SOCS5//NCK1//CD3E//COPS8//MDFIC//RAPGEF4//SLC25A6//IFNG//KCNB1//MARCKS//NEUROD1//SNAP25//CLOCK//ADCYAP1//GRK6//GIT2//OTX2//RORA//RWDD3//YWHAG//TOB1//EPS8//ARHGAP1//LASP1//ARHGAP6//SH3BGRL//SKAP2//HOMER1//IGFBP3//KDR//RGS7BP//MET//ROR1//PRR5L//ACVR1//EDN1//EDN2//TXLNA//IFNA1//IL2//INHBB//CXCL9//OGN//PTHLH//CCL2//CCL8//CCL21//BMP3//VEGFB//WNT3//WNT7A//WNT10A//GDF5//SOCS1//F3//GJA1//AKAP11//TNFAIP3//BMPR2//TTK//PMEPA1//LDLRAD4//PPP1R16B//PTEN//UNC5B//SIRT1//ZEB1//NREP//ESR1//SULF1//SOX4//GSC//APCDD1//FRZB//HIC1//SHISA2//CCND1//CXXC4//NPNT//STRAP//LEMD3//FAM89B//BAMBI//SMURF1//PRDM16//TGFBR2//CTDSPL2//NOG//FAM83D//TSC1//RICTOR//PIK3CA//RRAGD//ARAP2//IQSEC2//APPL1//FBXO8//AGFG1//PSD//SERP1//ISL1//SLC6A1//RALGPS1//MID1//SEMA4C//UBA5//TP63//FOXA1//AR//IRF1//PLEKHG4B//ARHGEF4//ITSN1//SOS2//GPR55//DLG5//ANO1//SYBU//EPS15//CNOT7//SOCS3//ZFAND6//FLNA//ATP2C1//TBK1//IRAK4//NDFIP2//MAVS//MIER1//UBE2I//ZMYND11//PDE5A//PAK6//SYNGAP1//EFNA1//HIPK3//PMAIP1//ATXN7//PEX5L//ADRB1//RAPGEF2//NPTN//YTHDF2//DLK2//EP300//DLL1//GLI3//RB1//EVC//GRIK2//RASSF2//RASGEF1A//PPP2CB//RASA1//CHRNB4//FMR1//RIMS4//CPEB3//DBN1//ARC//KCNJ10//RAB3GAP1//DHRS3//PXK//LRP8//ADNP//SYT1//GCSAML//PAX5//RALBP1//RHOB//SYDE2//ARHGAP12//CD2AP//SLIT2//SLC9A6//RAB8B//OTUD3//PHLDA3//GRM3//CALM2//TXK//PPARG//FOXP1//CCNY//LRP6//RANBP9//JUN//RCAN3//SPPL3//ARRDC3//TMEM64//PSMA2//PSMD12//FZD6//GSKIP//TBL1XR1//ZMYND8//FXR1//LYPD6//CREBRF//NSG1//PAIP2//DYNLL2//PPP3R1//AGO1//PRKACB//MDM4//POU4F1//RBBP8//BAG5//TMBIM6//SERINC3//DNAJB9//PPP1R15B//SESN1//PFN2//PHF14//MYB//CRHR1//NR4A2//CASP2//TNFRSF12A//BCL2A1//ST18// |
| GO:0006351 | transcription, DNA-templated | Biological process | 271 | 3907 | 902 | 17653 | 1.35749382681718 | 8.3779402572809e-09 | 9.94035262455978e-07 | 8.07686274089838 | 0.300443458980044 | NPAT//TSHZ1//ZBTB18//CEBPB//TCERG1//STRAP//KLF12//GSC//ARX//DNMT3B//EDN1//EDNRB//EFNA1//CC2D1B//EN1//EP300//ESR1//JAZF1//CPEB3//FOXF1//MYT1L//SIN3B//SIRT1//ZFPM2//DNAJB5//GLI3//CNOT7//HIC1//FOXA1//HSBP1//ID2//IFNG//IRF2//AR//ISL1//JARID2//JUN//MAF//MDM4//MEF2A//MYB//NFIA//NFIB//NFIC//NR4A2//PAX5//KLF3//POU4F1//PPARG//ZBTB4//RB1//RBBP8//CCND1//RNF2//SARS//PRDM16//SMARCA2//SUV39H1//ZEB1//TFAP2A//TGIF1//KLF10//TNF//UBE2I//WT1//ZNF217//LRP8//BHLHE41//TBL1XR1//E2F8//ARID5B//BHLHE40//TP63//RUNX3//NOG//FOXP2//PHF14//GTF2E1//GTF2H1//CREBRF//ELK3//ETV5//ATF5//FOXF2//FLI1//GTF2A1//HIVEP2//IRF1//MEOX2//MYBL1//MYCN//NEUROD1//NHLH2//OTX2//KLF13//PKNOX1//POU3F2//FOXJ2//HIVEP3//BMPR2//SOX4//SOX5//BTF3//TFE3//TXK//FOSL1//KLF7//CBFB//FUBP3//CCNT2//NMI//CLOCK//ZNF516//POLR3H//SRSF2//SRSF6//SLBP//BAHD1//H2AFZ//HIPK3//ZMYND11//PNRC1//ZNF526//PPARGC1B//ZNF800//ARID2//ELAVL2//ZFP30//ELL2//KDM2A//MYCBP2//FLII//ZMYND8//CNOT6L//NR5A2//ZNF521//ZBTB11//NRBF2//HMGB3//ZNF680//ZNF662//AFF1//MLLT6//OGG1//ASCC1//PHF20L1//COMMD10//BNC2//PPP2CA//ZNF532//SLC2A4RG//PAK6//PHTF2//CNOT6//ZBTB26//RORA//BTG1//UBE2V1//ZNF3//ZNF131//ZNF227//CSDE1//ZNF655//SAP30L//SNIP1//ANP32A//TEAD2//RPS6KA5//CREBL2//ZNF367//EMX2//ETS1//ADNP//EPC2//HBP1//FOXP1//ATAD2//HOXA3//HOXD1//BOLA3//NR3C2//NEUROG1//NFE2//ARID4B//BRWD1//MED9//TRERF1//MIER1//PURA//RFX7//TRAK2//SMARCD2//MED22//ZNF708//ZSCAN5A//LCOR//BDP1//NRBP1//G3BP2//EREG//FLNA//PHF8//BAMBI//MDFIC//IGF1//LRP6//NPAS2//MAPK1//SS18//WNT7A//ACVR1//DNAJB6//NRG1//RASD1//ATXN1//BTAF1//PPM1F//TOB1//SMURF1//BMP3//GDF5//SULF1//LEMD3//CTDSPL2//RWDD3//TNFAIP3//INSIG2//MYOG//C5AR1//INTS8//NABP1//EGLN1//FZD6//ADCYAP1//E2F5//S1PR1//MED19//KPNA6//AGO1//DLL1//TBK1//IL2//MET//PPP1R12A//NCK1//IER5//ARMCX3//CYTL1//PPP3R1//CCNL1//MAVS//RAF1//ATXN7//YES1//PCGF5//ST18//C8ORF44-SGK3//SGK3//SGK1//PTEN//RHEBL1//ROR1//IRAK4//EGLN3//PSMA2//PSMD12//PRKAA1//TMBIM6//FBXW7//NPNT//PPP1R1B// |
| GO:0031175 | neuron projection development | Biological process | 89 | 936 | 902 | 17653 | 1.86091330755965 | 8.77834770233193e-09 | 1.02358561225639e-06 | 8.05658722114273 | 0.098669623059867 | SLITRK3//OGN//POU4F1//PRELP//WNT7A//SLIT2//RANBP9//ARX//DPYSL2//EFNA1//EFNB2//GLI3//OTX2//PIK3CA//PIK3CB//ENAH//MAPK1//ROBO2//SCN1B//WNT3//KLF7//RPS6KA5//NRXN3//FEZ2//NOG//PTEN//SNAP25//STMN2//ADCYAP1//NPTN//NCK1//SERPINI1//RAP1A//RAPGEF2//LPAR1//MYLIP//PTPRG//SPOCK1//TSC1//BAG5//STRN//SYNGAP1//CD3E//PLXNA4//NR4A2//NFIB//RAB21//ISL1//JUN//NREP//UNC5B//FZD3//ADNP//NRG1//TNFRSF12A//ULK2//SLC9A6//GJA1//GPM6A//NTF3//SGK1//HPRT1//MEF2A//TRAK2//FMR1//HECW2//FXR1//BMPR2//SEMA4C//POU3F2//DBN1//CAMSAP2//ACSL4//ARC//DOCK10//CPEB3//ZMYND8//IL2//MAPK6//DLG5//LRP8//SMURF1//SYT1//PLAA//CAMSAP1//SAMD14//LRP12//PSD//RB1// |
| GO:0031326 | regulation of cellular biosynthetic process | Biological process | 312 | 4640 | 902 | 17653 | 1.31597790350944 | 9.17871040217276e-09 | 1.0521291262694e-06 | 8.03721833236767 | 0.34589800443459 | NPAT//TSHZ1//ZBTB18//CEBPB//TCERG1//STRAP//KLF12//GSC//ARX//DNMT3B//EDN1//EDNRB//EFNA1//CC2D1B//EN1//EP300//ESR1//JAZF1//CPEB3//FOXF1//MYT1L//SIN3B//SIRT1//ZFPM2//DNAJB5//GLI3//CNOT7//HIC1//FOXA1//HSBP1//ID2//IFNG//IRF2//AR//ISL1//JARID2//JUN//MAF//MDM4//MEF2A//MYB//NFIA//NFIB//NFIC//NR4A2//PAX5//KLF3//POU4F1//PPARG//ZBTB4//RB1//RBBP8//CCND1//RNF2//SARS//PRDM16//SMARCA2//SUV39H1//ZEB1//TFAP2A//TGIF1//KLF10//TNF//UBE2I//WT1//ZNF217//LRP8//BHLHE41//TBL1XR1//E2F8//ARID5B//BHLHE40//TP63//RUNX3//NOG//FOXP2//PHF14//SEPSECS//SECISBP2L//NUP54//ESCO2//PPP2CA//BAHD1//H2AFZ//HIPK3//ZMYND11//PNRC1//ZNF526//PPARGC1B//ZNF800//ARID2//ELAVL2//ATF5//ZFP30//ELL2//KDM2A//MYCBP2//FLII//ZMYND8//CNOT6L//NR5A2//ZNF521//ZBTB11//GTF2A1//GTF2E1//NRBF2//HIVEP2//HMGB3//ZNF680//ZNF662//AFF1//MLLT6//OGG1//ASCC1//PHF20L1//COMMD10//BNC2//ZNF532//SLC2A4RG//PAK6//PHTF2//CNOT6//ZBTB26//HIVEP3//RORA//SOX4//BTF3//BTG1//UBE2V1//ZNF3//ZNF131//ZNF227//CSDE1//ZNF655//SAP30L//SNIP1//ANP32A//TEAD2//RPS6KA5//CLOCK//ZNF516//CREBL2//ZNF367//ELK3//EMX2//ETS1//ETV5//FOXF2//FLI1//ADNP//EPC2//HBP1//FOXP1//ATAD2//HOXA3//HOXD1//BOLA3//NR3C2//MYBL1//MYCN//NEUROG1//NFE2//NHLH2//KLF13//ARID4B//BRWD1//POU3F2//MED9//TRERF1//FOXJ2//MIER1//PURA//RFX7//TRAK2//SMARCD2//SOX5//MED22//TXK//ZNF708//ZSCAN5A//FOSL1//LCOR//KLF7//CBFB//BDP1//DDX6//TSC1//KBTBD8//GRM3//G3BP2//AGO1//IGF1//CCL21//CRHR1//CNEP1R1//LDLR//FBXW7//ARPP19//FMR1//SERP1//PRR16//MAPK1//FXR1//FLNA//PHF8//BAMBI//MDFIC//IRF1//LRP6//NEUROD1//NPAS2//OTX2//SS18//WNT7A//FUBP3//ACVR1//DNAJB6//EREG//NRG1//RASD1//ATXN1//BTAF1//PPM1F//TOB1//CELF1//NANOS1//TIA1//MEX3B//SMURF1//BMP3//BMPR2//GDF5//SULF1//LEMD3//CTDSPL2//ADRB1//PTHLH//CALM2//EGLN1//RWDD3//TNFAIP3//DCP2//CCT6A//INSIG2//TNRC6B//NCK1//ADIPOR2//ERRFI1//FZD6//CD3E//NMI//TBK1//MID1IP1//ELOVL5//C1QTNF2//MYOG//PRKAA1//ADCYAP1//CREBRF//E2F5//S1PR1//MED19//KPNA6//DLL1//GTF2H1//IL2//MEOX2//MET//PPP1R12A//IER5//ARMCX3//PKNOX1//CYTL1//PPP3R1//CCNL1//MAVS//RAF1//ATXN7//TFE3//YES1//PCGF5//CCNT2//ST18//PAIP2//BOLL//RAPGEF2//INHBB//C8ORF44-SGK3//SGK3//SGK1//PTEN//RHEBL1//ROR1//IRAK4//PTGES3//YTHDF2//QKI//EGLN3//PSMA2//PSMD12//CPEB2//RAB3GAP1//RAP2C//HSBP1L1//SRSF2//PPP1R15B//TMBIM6//DUSP1//GJA1//KCNK2//NPNT//CPEB1// |
| GO:0006355 | regulation of transcription, DNA-templated | Biological process | 261 | 3745 | 902 | 17653 | 1.3639569684931 | 1.18234773997314e-08 | 1.33270296090639e-06 | 7.92725477443544 | 0.289356984478936 | NPAT//TSHZ1//ZBTB18//CEBPB//TCERG1//STRAP//KLF12//GSC//ARX//DNMT3B//EDN1//EDNRB//EFNA1//CC2D1B//EN1//EP300//ESR1//JAZF1//CPEB3//FOXF1//MYT1L//SIN3B//SIRT1//ZFPM2//DNAJB5//GLI3//CNOT7//HIC1//FOXA1//HSBP1//ID2//IFNG//IRF2//AR//ISL1//JARID2//JUN//MAF//MDM4//MEF2A//MYB//NFIA//NFIB//NFIC//NR4A2//PAX5//KLF3//POU4F1//PPARG//ZBTB4//RB1//RBBP8//CCND1//RNF2//SARS//PRDM16//SMARCA2//SUV39H1//ZEB1//TFAP2A//TGIF1//KLF10//TNF//UBE2I//WT1//ZNF217//LRP8//BHLHE41//TBL1XR1//E2F8//ARID5B//BHLHE40//TP63//RUNX3//NOG//FOXP2//PHF14//BAHD1//H2AFZ//CREBL2//ZNF367//ELK3//EMX2//ETS1//ETV5//ATF5//FOXF2//FLI1//ADNP//EPC2//HBP1//FOXP1//ATAD2//GTF2E1//HIVEP2//HOXA3//HOXD1//BOLA3//NR3C2//MYBL1//MYCN//NEUROG1//NFE2//NHLH2//KLF13//ARID4B//BRWD1//POU3F2//MED9//TRERF1//FOXJ2//SLC2A4RG//MIER1//PURA//HIVEP3//RFX7//TRAK2//SMARCD2//SOX5//MED22//TXK//ZNF3//ZNF708//ZNF131//ZSCAN5A//FOSL1//LCOR//KLF7//CBFB//CLOCK//BDP1//G3BP2//FLNA//PHF8//NR5A2//BAMBI//MDFIC//IGF1//IRF1//LRP6//NEUROD1//NPAS2//OTX2//MAPK1//RORA//SOX4//SS18//UBE2V1//WNT7A//TEAD2//FUBP3//ACVR1//ZNF516//DNAJB6//PPARGC1B//EREG//NRG1//RASD1//ATXN1//BTAF1//RPS6KA5//PPM1F//TOB1//SMURF1//BMP3//BMPR2//GDF5//SULF1//LEMD3//CTDSPL2//RWDD3//TNFAIP3//INSIG2//ZMYND11//EGLN1//FZD6//ADCYAP1//CREBRF//E2F5//S1PR1//MED19//KPNA6//AGO1//DLL1//TBK1//GTF2H1//IL2//MEOX2//MET//MYOG//PPP1R12A//NCK1//IER5//ARMCX3//PKNOX1//CYTL1//PPP3R1//CCNL1//MAVS//RAF1//ATXN7//TFE3//YES1//PCGF5//CCNT2//ST18//C8ORF44-SGK3//SGK3//SGK1//PTEN//RHEBL1//ROR1//IRAK4//EGLN3//PSMA2//PSMD12//PRKAA1//ZMYND8//TMBIM6//FBXW7//NPNT//HIPK3//PNRC1//ZNF526//ZNF800//ARID2//ELAVL2//ZFP30//ELL2//KDM2A//MYCBP2//FLII//CNOT6L//ZNF521//ZBTB11//GTF2A1//NRBF2//HMGB3//ZNF680//ZNF662//AFF1//MLLT6//OGG1//ASCC1//PHF20L1//COMMD10//BNC2//PPP2CA//ZNF532//PAK6//PHTF2//CNOT6//ZBTB26//BTF3//BTG1//ZNF227//CSDE1//ZNF655//SAP30L//SNIP1//ANP32A// |
| GO:0003158 | endothelium development | Biological process | 24 | 123 | 902 | 17653 | 3.81872262181602 | 1.23957879175365e-08 | 1.35787147237852e-06 | 7.90672586281607 | 0.0266075388026608 | CCM2//CLIC4//MET//GSTM3//WNT7A//S1PR1//ZEB1//BTG1//BMPR2//DLL1//NRG1//PPP1R16B//MSN//RAP1A//RAP1B//RAPGEF2//RHOB//ACVR1//RAP2C//FOXJ2//FOXP1//TNF//GJA1//KDR// |
| GO:0000122 | negative regulation of transcription by RNA polymerase II | Biological process | 79 | 800 | 902 | 17653 | 1.9326316518847 | 1.2448326376973e-08 | 1.35787147237852e-06 | 7.90488903363682 | 0.0875831485587583 | NFIB//ZMYND8//JUN//NCK1//TMBIM6//TSHZ1//ZBTB18//CEBPB//TCERG1//STRAP//KLF12//GSC//ARX//DNMT3B//EDN1//EDNRB//EFNA1//CC2D1B//EN1//EP300//ESR1//JAZF1//CPEB3//FOXF1//MYT1L//SIN3B//SIRT1//ZFPM2//DNAJB5//GLI3//CNOT7//HIC1//FOXA1//HSBP1//ID2//IFNG//IRF2//AR//ISL1//JARID2//MAF//MDM4//MEF2A//MYB//NFIA//NFIC//NR4A2//PAX5//KLF3//POU4F1//PPARG//ZBTB4//RB1//RBBP8//CCND1//RNF2//SARS//PRDM16//SMARCA2//SUV39H1//ZEB1//TFAP2A//TGIF1//KLF10//TNF//UBE2I//WT1//ZNF217//LRP8//BHLHE41//TBL1XR1//E2F8//ARID5B//BHLHE40//TP63//RUNX3//NOG//FOXP2//PHF14// |
| GO:0048729 | tissue morphogenesis | Biological process | 67 | 639 | 902 | 17653 | 2.05204050119887 | 1.68596993243087e-08 | 1.79951828971824e-06 | 7.7731501748452 | 0.0742793791574279 | EDN1//PPP3R1//TGFBR2//ACVR1//NPNT//GLI3//WT1//BMPR2//WNT3//NOG//FZD3//TP63//SOX4//LRP6//SEMA4C//PRKACB//TSC1//FZD6//TEAD2//GJA1//DLL1//ARHGAP12//ISL1//ZFPM2//NRG1//POU4F1//S1PR1//TFAP2A//GRSF1//DLG5//TGM3//WNT10A//WNT7A//WDR1//FOXF2//FLNA//TMEFF2//FOXF1//MSN//CLIC4//MET//MYCN//SLIT2//EGLN1//TNNI1//FRZB//PRICKLE2//ROR1//PSMA2//SMURF1//PSMD12//RDH10//FOXA1//AR//ESR1//SOCS3//SULF1//BTBD7//TNF//ETV5//RHOB//CCM2//KLHL3//EFNB2//FOXP1//PTEN//GCNT4// |
| GO:0019220 | regulation of phosphate metabolic process | Biological process | 144 | 1791 | 902 | 17653 | 1.57354399491916 | 1.70293021650107e-08 | 1.79951828971824e-06 | 7.76880314840232 | 0.159645232815965 | GADD45A//GTF2H1//PTEN//CCNT2//DIRAS3//TNF//CRKL//TAOK1//RAF1//RAP1A//C1QTNF2//LPAR1//NRG1//IGF1//NTF3//PIK3CB//PRKAA1//MAPK1//C5AR1//UBE2V1//SPRED1//DUSP1//PPP2CA//SOCS1//IGFBP3//IL2//LRP6//PRR5L//PPP1R15B//SLIT2//EDNRB//EFNA1//FMR1//SIRT1//NPTN//IFNG//KDR//MAVS//CCND1//CCND2//EREG//FBXW7//ERRFI1//GPRC5A//SOCS5//NUP54//GNAQ//NCK1//GSKIP//RB1//YWHAG//SOCS3//PPM1F//GRM3//COPS8//MDFIC//CRHR1//PPARGC1B//NPNT//CALM2//INHBB//BMP3//BMPR2//TTK//GDF5//ACVR1//LDLR//MTMR9//FKBP15//PPP1R12A//PLEKHF2//TBL1XR1//CXCL9//ADRB1//PTHLH//EGLN1//PDE5A//ADNP//MOB1B//JUN//RAP2C//RASSF2//PIK3CA//PAK6//TGFBR2//SLK//RICTOR//ARPP19//PPP1R1B//MID1//SEMA4C//RPS6KA5//CREBL2//TBK1//PFN2//IFNA1//MET//ROR1//ADCY3//PRKACB//PRKAR2B//PPP1R16B//SPPL3//CNEP1R1//ATXN7//PTGES3//AR//ITSN1//CNOT7//ISL1//EDN1//SYNGAP1//HIPK3//NRBF2//CCL21//RCAN3//UBXN2B//PPP4R2//PPP2R5E//TSC1//TNFAIP3//CCNY//CCNL1//MYOG//ADCYAP1//CACUL1//MMD//RAPGEF2//GRIK2//ZMYND11//WNT7A//YES1//LRP8//CD3E//STRAP//PMEPA1//LDLRAD4//NOG//FAM83D//RANBP9//CCL2//CCL8//GPR55//ACSL1// |
| GO:0050789 | regulation of biological process | Biological process | 673 | 11709 | 902 | 17653 | 1.12488271098908 | 2.05583375780183e-08 | 2.13901595446366e-06 | 7.68701200688899 | 0.746119733924612 | USP28//CLOCK//GADD45A//GTF2H1//PTEN//CCNT2//DIRAS3//NPAT//TSHZ1//ZBTB18//CEBPB//TCERG1//STRAP//KLF12//GSC//ARX//DNMT3B//EDN1//EDNRB//EFNA1//CC2D1B//EN1//EP300//ESR1//JAZF1//CPEB3//FOXF1//MYT1L//SIN3B//SIRT1//ZFPM2//DNAJB5//GLI3//CNOT7//HIC1//FOXA1//HSBP1//ID2//IFNG//IRF2//AR//ISL1//JARID2//JUN//MAF//MDM4//MEF2A//MYB//NFIA//NFIB//NFIC//NR4A2//PAX5//KLF3//POU4F1//PPARG//ZBTB4//RB1//RBBP8//CCND1//RNF2//SARS//PRDM16//SMARCA2//SUV39H1//ZEB1//TFAP2A//TGIF1//KLF10//TNF//UBE2I//WT1//ZNF217//LRP8//BHLHE41//TBL1XR1//E2F8//ARID5B//BHLHE40//TP63//RUNX3//NOG//FOXP2//PHF14//KCNH4//RANBP9//SPRED1//EREG//RASGEF1A//NRG1//IL2//MET//MAPK1//MAPK6//PSMA2//PSMD12//RAF1//RASA1//CCL2//SYNGAP1//RAPGEF2//PNRC1//DCP2//PPP2CA//CRKL//TAOK1//RAP1A//C1QTNF2//LPAR1//IGF1//NTF3//PIK3CB//PRKAA1//C5AR1//UBE2V1//DUSP1//CNOT6L//PAN3//CNOT6//PATL1//MBNL2//RBFOX3//FMR1//MBNL1//RBM25//SRSF2//SRSF6//AGO1//SEPSECS//SECISBP2L//C8ORF44-SGK3//SGK2//SGK3//NANOS1//IGFBP3//SGK1//SPOCK1//RPS6KA5//PMAIP1//BCL2A1//NSG1//EPS15//VAMP3//SOCS1//LRP6//PRR5L//PPP1R15B//SLIT2//NPTN//KDR//MAVS//CCND2//SULF1//GJA1//F3//RICTOR//PPP1R16B//BMPR2//VEGFB//VASH2//TSC1//CCL21//CBFB//ADRB1//CALM2//ARL6IP1//FZD3//MYCN//TGFBR2//NUP54//PRKACB//SKP1//IRAK4//BAG6//CR2//TNFAIP3//CD3E//CCL8//EDN2//CXCL9//SKAP2//IRF1//ADCYAP1//FOXP1//S1PR1//SPHK2//E2F5//CDK15//EPS8//APCDD1//CCNY//HBP1//GRK6//STRN//WNT3//WNT7A//CXXC4//WNT10A//TNRC6B//PPP3R1//FZD6//CNIH1//CDS1//PTGES3//IQGAP2//ADCY3//RALBP1//TMED1//RASSF8//CHRNB4//ARAP2//CLCN6//CNGA3//CNTFR//CREBL2//DPYSL2//DTNA//ELK3//CD2AP//GABRA5//APPL1//ATP2C1//GLRB//LRP12//HIVEP2//NR3C2//PPP1R12A//HPCAL4//GULP1//RASD1//KCNK10//CYTL1//NDFIP2//PPP2R5E//PSD//ARRDC3//MIER1//HIVEP3//SMOC1//SMOC2//ZYX//GLRA3//SNX27//SYDE2//PPP1R1B//PPFIA1//OR6A2//PDE5A//GPRC5A//DLG5//PLAA//ARHGAP12//GTPBP1//FEZ2//ZNF516//CD69//ULK2//SNX17//ROR1//PTPRG//TXK//YES1//CD8A//AKAP11//DGKH//NRBP1//ARHGEF4//PRKAR2B//STK33//SS18//ANP32A//DGKE//RALGPS1//PALM2//FBXW7//ERRFI1//SOCS5//NLN//DNAJC16//GLRX//NHLRC2//ESCO2//BAHD1//H2AFZ//ARID4B//HIPK3//ZMYND11//ZNF526//PPARGC1B//ZNF800//ARID2//ELAVL2//ATF5//ZFP30//ELL2//KDM2A//MYCBP2//FLII//ZMYND8//NR5A2//ZNF521//ZBTB11//GTF2A1//GTF2E1//NRBF2//HMGB3//ZNF680//ZNF662//AFF1//MLLT6//OGG1//ASCC1//PHF20L1//COMMD10//BNC2//ZNF532//SLC2A4RG//PAK6//PHTF2//ZBTB26//RORA//SOX4//BTF3//BTG1//ZNF3//ZNF131//ZNF227//CSDE1//ZNF655//SAP30L//SNIP1//TEAD2//ZNF367//EMX2//ETS1//ETV5//FOXF2//FLI1//ADNP//EPC2//ATAD2//HOXA3//HOXD1//BOLA3//MYBL1//NEUROG1//NFE2//NHLH2//KLF13//BRWD1//POU3F2//MED9//TRERF1//FOXJ2//PURA//RFX7//TRAK2//SMARCD2//SOX5//MED22//ZNF708//ZSCAN5A//FOSL1//LCOR//KLF7//BDP1//DDX6//KBTBD8//GNAQ//NCK1//GSKIP//YWHAG//SOCS3//PPM1F//EGLN3//CASP2//CHRM2//TNNI1//STMN2//CAMSAP1//CAMSAP2//MID1//MID1IP1//NAV3//RRAGD//CUL5//NABP1//TTK//CDC14A//CBLL1//PLXNA4//CRHR1//IGSF3//TSPAN11//PIK3CA//SMURF1//GDF5//ACVR1//NMUR1//RAPGEF4//TAPT1//FRZB//OR4N4//GRM3//KCNK2//RHOB//RGS7BP//PTHLH//ITSN1//SOS2//GPR55//FLNA//ANO1//HOMER1//GRIK2//KCNB1//NXPH3//SORCS1//SORCS2//SUSD5//DLL1//TMEM17//EVC//DZIP1//ITGB8//CCM2//RCAN3//SAMD14//TBK1//COPS8//G3BP2//MDFIC//NMI//NEUROD1//RAB18//RGL1//RND3//ARHGAP1//RIN2//DOCK10//ARHGAP6//CDK19//SLK//TRPC3//SLC25A6//MARCKS//SNAP25//KLHL20//ZFAND6//BNIP2//TMBIM6//GIT2//DNAJA2//CACUL1//EFNB2//BAMBI//TOB1//TES//MYOG//ST18//CFDP1//WASF3//ANXA7//MSN//WDR1//HS3ST5//TIA1//SHCBP1//OTX2//NPNT//ADIPOR2//PYURF//PIGA//RWDD3//LASP1//SH3BGRL//NEDD1//CEP41//PI15//PPP2CB//DDX3Y//TXLNA//IFNA1//INHBB//OGN//BMP3//DRAM1//MTMR9//PPP4R2//RAB3GAP1//LDLR//FUBP3//QKI//PFN2//LDLRAD4//C5ORF30//SCN1B//CHMP3//VPS4B//C2CD5//CNEP1R1//ABHD5//INSIG2//FKBP15//PLEKHF2//SERPINI1//SERPINB13//RECK//MYLIP//BAG5//PMEPA1//SLC6A1//SYT1//UNC5B//KCNJ2//ARPP19//SERP1//PRR16//FXR1//RAB12//SESN1//SNX5//CELF1//CPEB2//TNFRSF21//PHF8//NPAS2//DNAJB6//ATXN1//BTAF1//NREP//MEX3B//RAB21//RAB8B//SYT6//ACSL1//PLP2//SDC1//SIRPA//SH3D19//ARC//SPPL3//DENND1B//HECW2//SPOPL//SHISA2//DHRS3//EI24//FAM89B//SEMA4C//PTP4A1//TMEFF2//CLIC4//RAP2C//TSN//LEMD3//CTDSPL2//EGLN1//CFL2//CREBRF//CLIP1//TBC1D12//SLC9A6//RHEBL1//MOB1B//RASSF2//FAM83D//IQSEC2//FBXO8//AGFG1//CCT6A//STIM2//ANKIB1//LONRF3//LONRF1//KIF13A//PCBP2//POLR3H//RAB30//RAB9B//RAB28//RAP1B//STX6//MMD//SLC26A4//MEMO1//KLHL42//GFPT1//FRMD6//UBA5//EDARADD//TNFRSF12A//ARFIP1//KCNA4//KCNJ3//KCNJ10//PLEKHG4B//ROBO2//GSTA2//SYBU//DNAJB9//ASNA1//SEC61A2//ZMAT3//IER5//ATXN7//PHLDA3//USP27X//PXK//FASTK//YTHDF2//UBXN2B//PEX5L//TMEM64//DLK2//TFE3//ELOVL5//CCNL1//ADAM12//RNF167//MED19//KPNA6//MEOX2//ARMCX3//PKNOX1//PCGF5//PAIP2//BOLL//RAET1E//HPRT1//GIGYF1//MORC3//RIMS4//DBN1//ELMOD2//TUB//NPDC1//GCSAML//NOVA1//ZER1//DCUN1D4//GPM6A//TMOD3//TMOD1//OTUD3//SLITRK3//PRICKLE2//KCNE1//BTBD7//SLC30A6//KCNIP4//FIBIN//GJC1//MIEF1//ANAPC15//LPCAT3//SIVA1//CAST//BSN//LYPD6//DYNLL2//KCNAB2//CRTAP//SERINC3//SLC31A2//ABHD17C//SEPT9//HSBP1L1//SESTD1//FAM168A//DENND6A//IER3IP1//CPEB1// |
| GO:0023051 | regulation of signaling | Biological process | 250 | 3575 | 902 | 17653 | 1.36859814243406 | 2.09142627064618e-08 | 2.14307816187578e-06 | 7.67955744105988 | 0.277161862527716 | GADD45A//TNF//CRKL//TAOK1//RAF1//RAP1A//C1QTNF2//LPAR1//NRG1//IGF1//NTF3//PIK3CB//PRKAA1//MAPK1//C5AR1//UBE2V1//SPRED1//DUSP1//PPP2CA//NSG1//EPS15//VAMP3//CBFB//EREG//FBXW7//ERRFI1//GPRC5A//SOCS5//NCK1//CD3E//COPS8//MDFIC//RAPGEF4//SLC25A6//IFNG//KCNB1//MARCKS//NEUROD1//SNAP25//CLOCK//ADCYAP1//GRK6//GIT2//OTX2//RORA//RWDD3//YWHAG//TOB1//EPS8//ARHGAP1//LASP1//ARHGAP6//SH3BGRL//SKAP2//HOMER1//IGFBP3//KDR//RGS7BP//MET//ROR1//PRR5L//ACVR1//EDN1//EDN2//TXLNA//IFNA1//IL2//INHBB//CXCL9//OGN//PTHLH//CCL2//CCL8//CCL21//BMP3//VEGFB//WNT3//WNT7A//WNT10A//GDF5//SOCS1//F3//AKAP11//TNFAIP3//BMPR2//TTK//PMEPA1//LDLRAD4//PPP1R16B//PTEN//UNC5B//SIRT1//ZEB1//NREP//ESR1//SULF1//SOX4//GSC//APCDD1//FRZB//HIC1//SHISA2//CCND1//CXXC4//NPNT//STRAP//LEMD3//FAM89B//BAMBI//SMURF1//PRDM16//TGFBR2//CTDSPL2//NOG//FAM83D//TSC1//RICTOR//PIK3CA//RRAGD//ARAP2//IQSEC2//APPL1//FBXO8//AGFG1//PSD//GJA1//SERP1//ISL1//SLC6A1//RALGPS1//MID1//SEMA4C//UBA5//TP63//FOXA1//AR//IRF1//PLEKHG4B//ARHGEF4//ITSN1//SOS2//GPR55//DLG5//ANO1//SYBU//CNOT7//SOCS3//ZFAND6//FLNA//ATP2C1//TBK1//IRAK4//NDFIP2//MAVS//MIER1//UBE2I//ZMYND11//PDE5A//PAK6//SYNGAP1//EFNA1//HIPK3//PMAIP1//ATXN7//PEX5L//ADRB1//RAPGEF2//NPTN//YTHDF2//DLK2//EP300//DLL1//GLI3//RB1//EVC//GRIK2//RASSF2//RASGEF1A//PPP2CB//RASA1//CHRNB4//FMR1//RIMS4//CPEB3//DBN1//ARC//KCNJ10//RAB3GAP1//DHRS3//PXK//LRP8//ADNP//SYT1//GCSAML//PAX5//RALBP1//RHOB//SYDE2//ARHGAP12//CD2AP//SLIT2//SLC9A6//RAB8B//OTUD3//PHLDA3//GRM3//CALM2//TXK//PPARG//FOXP1//CCNY//LRP6//RANBP9//JUN//RCAN3//SPPL3//ARRDC3//TMEM64//PSMA2//PSMD12//FZD6//GSKIP//TBL1XR1//ZMYND8//FXR1//LYPD6//CREBRF//PAIP2//DYNLL2//PPP3R1//AGO1//PRKACB//MDM4//POU4F1//RBBP8//BAG5//TMBIM6//SERINC3//DNAJB9//PPP1R15B//SESN1//PFN2//PHF14//MYB//CRHR1//NR4A2//CASP2//TNFRSF12A//BCL2A1//ST18// |
| GO:0006915 | apoptotic process | Biological process | 154 | 1958 | 902 | 17653 | 1.53928847255786 | 2.13460902511944e-08 | 2.1546807219228e-06 | 7.67068165871032 | 0.170731707317073 | TNFRSF21//JUN//PMAIP1//BCL2A1//PTEN//FOXP1//IL2//EGLN3//F3//PPARG//TNF//CASP2//HIPK3//DUSP1//EDNRB//ATF5//FLNA//ARL6IP1//SIRT1//KLHL20//GLI3//IGF1//KDR//MDM4//MET//ROR1//OGG1//ZFAND6//PRKAA1//SPHK2//RAF1//RASA1//BNIP2//TMBIM6//TFAP2A//WNT7A//WT1//BAG6//TP63//CCND2//SOCS3//HIC1//GSKIP//PPP2CB//ADCYAP1//C8ORF44-SGK3//SGK2//ESR1//SGK3//IGFBP3//INHBB//NTF3//PAK6//TAOK1//RBM25//SGK1//BMP3//ANP32A//GDF5//CD3E//DLG5//SLK//PIK3CB//RB1//DLL1//CCL2//EDN1//LRP6//IFNG//EP300//PHLDA3//USP28//GADD45A//LPAR1//CDK19//FRZB//FOXA1//RHOB//USP27X//NEUROD1//ARHGEF4//TNFRSF12A//POU4F1//ZMAT3//ITSN1//SOS2//SOX4//FOSL1//SLIT2//RASSF2//DNAJB6//PIK3CA//PPM1F//CEBPB//CNTFR//EN1//UNC5B//ADNP//GABRA5//GRIK2//ISL1//NR4A2//C5AR1//SYNGAP1//MYB//ROBO2//EFNA1//XKR8//BMPR2//FASTK//SIVA1//APPL1//CUL5//FZD3//DYNLL2//PPP3R1//YWHAG//TNFAIP3//SERPINB13//BAG5//SERINC3//NCK1//FBXW7//TIA1//TGFBR2//IER3IP1//CFDP1//BTG1//CCL21//RAPGEF2//KCNB1//SRSF6//CAST//NOG//ZMYND11//AR//ACVR1//ST18//RTN3//PRUNE2//SULF1//GJA1//NSG1//SLC25A6//IRF1//C6ORF120//MEF2A//GULP1//CHMP3//PPP2CA//DRAM1//MAPK1//FXR1//EI24// |
| GO:0051128 | regulation of cellular component organization | Biological process | 181 | 2403 | 902 | 17653 | 1.47413340493636 | 2.32510843353286e-08 | 2.29640412008319e-06 | 7.63355678858613 | 0.200665188470067 | C8ORF44-SGK3//SGK2//SGK3//NANOS1//IGFBP3//RB1//SGK1//SPOCK1//FMR1//NTF3//EPS8//GTF2H1//STMN2//CAMSAP1//CAMSAP2//MID1//MID1IP1//NAV3//TTK//CDC14A//CFDP1//WASF3//PALM2//LPAR1//BAMBI//ANXA7//KDR//RHOB//RND3//MSN//BRWD1//RASA1//CCL2//PLXNA4//WDR1//SLIT2//CNOT6L//PAN3//CNOT6//ETS1//PPARGC1B//CHMP3//VPS4B//PTEN//SNAP25//ADCYAP1//NPTN//NCK1//SERPINI1//RAP1A//SCN1B//RAPGEF2//EFNB2//MYLIP//PTPRG//TSC1//BAG5//PMEPA1//LDLRAD4//SH3D19//ARC//HECW2//RAB21//SNX17//IL2//SIRT1//FRZB//GJA1//PPARG//PPP2CA//BMPR2//SMARCA2//BTG1//WT1//ADIPOR2//EI24//DPYSL2//PFN2//CFL2//RICTOR//CCL21//JARID2//MET//CLIP1//WNT7A//RAF1//IFNG//TNF//TBC1D12//SYT1//C2CD5//RAB8B//NEUROG1//ATAD2//MIER1//DCP2//MAPK1//CCT6A//EP300//PMAIP1//TAOK1//RPS6KA5//IQGAP2//ARFIP1//ISL1//TMEFF2//AR//TAPT1//ADNP//NRG1//TNFRSF12A//DLL1//LDLR//CBLL1//EDN1//EREG//IGF1//ESR1//ATF5//CD2AP//ULK2//WNT3//FXR1//SEMA4C//SIRPA//TUB//EFNA1//POU3F2//TRAK2//SYNGAP1//ROBO2//DBN1//ETV5//PPP1R16B//ZMYND8//GPM6A//CLIC4//PPM1F//S1PR1//ARHGAP6//PPFIA1//DNMT3B//MYB//PAX5//TMOD3//TMOD1//SLK//SLITRK3//DLG5//CPEB3//MAPK6//LRP8//PHF8//SMURF1//DNAJB6//MIEF1//DUSP1//ANAPC15//CRKL//FLNA//MAVS//DYNLL2//PPP3R1//YWHAG//TP63//MYOG//CXCL9//RAP1B//SEPT9//FEZ2//FBXW7//RAB3GAP1//SDC1//PLAA//NMUR1//PRKAA1//SNIP1//JUN//FOSL1//PIK3CB//CBFB// |
| GO:0031399 | regulation of protein modification process | Biological process | 144 | 1800 | 902 | 17653 | 1.56567627494457 | 2.34292302655242e-08 | 2.29640412008319e-06 | 7.6302419793043 | 0.159645232815965 | GADD45A//GTF2H1//PTEN//CCNT2//DIRAS3//TNF//CRKL//TAOK1//RAF1//RAP1A//C1QTNF2//LPAR1//NRG1//IGF1//NTF3//PIK3CB//PRKAA1//MAPK1//C5AR1//UBE2V1//SPRED1//DUSP1//PPP2CA//SOCS1//IGFBP3//IL2//LRP6//PRR5L//PPP1R15B//SLIT2//EDNRB//EFNA1//FMR1//SIRT1//NPTN//IFNG//KDR//MAVS//CCND1//CCND2//EREG//FBXW7//ERRFI1//GPRC5A//SOCS5//GNAQ//NCK1//GSKIP//RB1//YWHAG//SOCS3//PPM1F//COPS8//MDFIC//CALM2//ADNP//INHBB//BMP3//BMPR2//TTK//GDF5//ACVR1//JARID2//SPOPL//SOX4//TNFAIP3//BAG5//NDFIP2//MOB1B//JUN//RAP2C//RASSF2//PIK3CA//PAK6//TGFBR2//SLK//RICTOR//ARPP19//PPP1R1B//MID1//SEMA4C//RPS6KA5//CREBL2//TBK1//PFN2//IFNA1//RWDD3//ADCY3//PRKACB//PRKAR2B//ISL1//PPP1R16B//SPPL3//CNEP1R1//PPP1R12A//CNOT7//EDN1//PDE5A//SYNGAP1//AR//MET//ROR1//HIPK3//RCAN3//UBXN2B//PPP4R2//PPP2R5E//TSC1//CCNY//CCNL1//ADCYAP1//CACUL1//MMD//CCL21//RAPGEF2//GRIK2//ZMYND11//WNT7A//YES1//LRP8//CD3E//ZER1//DCUN1D4//ARRDC3//DNMT3B//MYB//PAX5//STRAP//PMEPA1//LDLRAD4//NOG//FAM83D//RANBP9//NPNT//CCL2//CCL8//GPR55//ACSL1//EP300//PLAA//CRTAP//NMI//RAB3GAP1//UBE2I// |
| GO:1903507 | negative regulation of nucleic acid-templated transcription | Biological process | 107 | 1224 | 902 | 17653 | 1.71085949161631 | 2.41370831847542e-08 | 2.33198705112132e-06 | 7.61731521283447 | 0.118625277161863 | TSHZ1//ZBTB18//CEBPB//TCERG1//STRAP//KLF12//GSC//ARX//DNMT3B//EDN1//EDNRB//EFNA1//CC2D1B//EN1//EP300//ESR1//JAZF1//CPEB3//FOXF1//MYT1L//SIN3B//SIRT1//ZFPM2//DNAJB5//GLI3//CNOT7//HIC1//FOXA1//HSBP1//ID2//IFNG//IRF2//AR//ISL1//JARID2//JUN//MAF//MDM4//MEF2A//MYB//NFIA//NFIB//NFIC//NR4A2//PAX5//KLF3//POU4F1//PPARG//ZBTB4//RB1//RBBP8//CCND1//RNF2//SARS//PRDM16//SMARCA2//SUV39H1//ZEB1//TFAP2A//TGIF1//KLF10//TNF//UBE2I//WT1//ZNF217//LRP8//BHLHE41//TBL1XR1//E2F8//ARID5B//BHLHE40//TP63//RUNX3//NOG//FOXP2//PHF14//BAHD1//H2AFZ//FLNA//DNAJB6//PPARGC1B//ELK3//EREG//ATF5//FOXF2//FOXP1//MDFIC//NRG1//IRF1//RASD1//PURA//ATXN1//BTAF1//RPS6KA5//CLOCK//PPM1F//ATAD2//MIER1//PHF8//ZMYND8//NCK1//TMBIM6//TOB1//ZMYND11//HSBP1L1//NPAT//SRSF2// |
| GO:1902679 | negative regulation of RNA biosynthetic process | Biological process | 107 | 1226 | 902 | 17653 | 1.70806852996604 | 2.62928948905783e-08 | 2.5044908189434e-06 | 7.58016159473086 | 0.118625277161863 | TSHZ1//ZBTB18//CEBPB//TCERG1//STRAP//KLF12//GSC//ARX//DNMT3B//EDN1//EDNRB//EFNA1//CC2D1B//EN1//EP300//ESR1//JAZF1//CPEB3//FOXF1//MYT1L//SIN3B//SIRT1//ZFPM2//DNAJB5//GLI3//CNOT7//HIC1//FOXA1//HSBP1//ID2//IFNG//IRF2//AR//ISL1//JARID2//JUN//MAF//MDM4//MEF2A//MYB//NFIA//NFIB//NFIC//NR4A2//PAX5//KLF3//POU4F1//PPARG//ZBTB4//RB1//RBBP8//CCND1//RNF2//SARS//PRDM16//SMARCA2//SUV39H1//ZEB1//TFAP2A//TGIF1//KLF10//TNF//UBE2I//WT1//ZNF217//LRP8//BHLHE41//TBL1XR1//E2F8//ARID5B//BHLHE40//TP63//RUNX3//NOG//FOXP2//PHF14//BAHD1//H2AFZ//FLNA//DNAJB6//PPARGC1B//ELK3//EREG//ATF5//FOXF2//FOXP1//MDFIC//NRG1//IRF1//RASD1//PURA//ATXN1//BTAF1//RPS6KA5//CLOCK//PPM1F//ATAD2//MIER1//PHF8//TOB1//ZMYND11//HSBP1L1//NPAT//SRSF2//ZMYND8//NCK1//TMBIM6// |
| GO:0048513 | animal organ development | Biological process | 241 | 3428 | 902 | 17653 | 1.37590425270435 | 2.68006011445916e-08 | 2.51739535473435e-06 | 7.57185546452155 | 0.267184035476718 | MYCN//WNT7A//CEBPB//ADCYAP1//INHBB//IMMP2L//SIRT1//ESR1//EREG//CASP2//RAB18//SMOC1//RDH10//GLI3//ID2//ROBO2//NPNT//SDC1//WT1//SLIT2//EDNRB//ISL1//SEMA4C//ACVR1//SULF1//TFAP2A//TSC1//BAG6//ARID5B//FOXF2//PPP3R1//FAM83D//DLG5//NOG//JARID2//JUN//MET//PIK3CA//PPARG//E2F8//STOX2//BMPR2//APCDD1//FZD3//WNT10A//FZD6//GJA1//DLL1//TGFBR2//SNX19//NFIB//CYTL1//GDF5//RUNX3//ESCO2//KCNAB2//DOCK10//SOX4//ARID2//TEAD2//ZFPM2//DHRS3//NRG1//EFNA1//FOXF1//POU4F1//KCNK2//S1PR1//SNX17//DCTN5//NEUROD1//PALLD//LRP6//GPM6A//CRKL//E2F5//EFNB2//EP300//FLI1//PAX5//CCL2//BHLHE41//DPYSL2//RAB3GAP1//PHF8//GABRA5//SPHK2//PTPRG//ATXN1//SLC6A11//SYT1//EDN1//GNAQ//MEF2A//PTEN//RAF1//ADIPOR2//EVC//IGF1//FXR1//SGCE//ZBTB18//CFL2//MEOX2//MYOG//GPCPD1//CCNT2//FOXP2//CCND1//RNF38//CSDE1//HOXA3//BAMBI//STRAP//FOXA1//PPP2CA//LDLRAD4//KBTBD8//TAPT1//FRZB//GSC//C5AR1//LRP8//EMX2//LPAR1//EN1//CD3E//RAPGEF2//RORA//HPRT1//ARX//CALM2//POU3F2//ETS1//ATF5//FLNA//PLXNA4//NR4A2//TACC2//MSN//CBFB//CR2//IFNA1//DNAJB9//CLIC4//TGM3//TP63//IL2//PKNOX1//TXK//WDR1//PTHLH//KLF10//FOXP1//OSTM1//TNF//AR//MAPK1//OTX2//KRT80//KRTAP5-6//KRTAP2-4//MAF//SOX5//LRIG1//IGSF3//IFNG//ZEB1//MGST1//NEUROG1//KDR//RB1//HIVEP3//INSIG2//TSHZ1//NFIC//IRF1//BNC2//PRDM16//ARRDC3//ERRFI1//CD8A//HMGB3//SOCS1//SERPINB13//SRSF6//MYB//SOCS5//KLF13//TNRC6B//AGO1//NFE2//TFE3//RASSF2//GPR55//PPARGC1B//TMEM64//EDN2//PHF14//PRKAA1//SERP1//GJC1//HOMER1//TMOD3//ROR1//CCM2//ITGB8//BMP3//EGLN1//TNNI1//TUB//PRICKLE2//PSMA2//SMURF1//PSMD12//MFAP5//CNTFR//ZNF516//TBL1XR1//SOCS3//FOSL1//BTBD7//DNAJB6//VASH2//ETV5//VEGFB//SPRED1//GCNT4//NR5A2//WNT3//TMOD1//KLHL3//NFIA//ARID4B//RAP1A//NANOS1//YTHDF2//NLN//CLOCK//FBXW7//TNFAIP3// |
| GO:0051174 | regulation of phosphorus metabolic process | Biological process | 144 | 1806 | 902 | 17653 | 1.56047469263578 | 2.89161851926626e-08 | 2.67890630764352e-06 | 7.53885900249285 | 0.159645232815965 | GADD45A//GTF2H1//PTEN//CCNT2//DIRAS3//TNF//CRKL//TAOK1//RAF1//RAP1A//C1QTNF2//LPAR1//NRG1//IGF1//NTF3//PIK3CB//PRKAA1//MAPK1//C5AR1//UBE2V1//SPRED1//DUSP1//PPP2CA//SOCS1//IGFBP3//IL2//LRP6//PRR5L//PPP1R15B//SLIT2//EDNRB//EFNA1//FMR1//SIRT1//NPTN//IFNG//KDR//MAVS//CCND1//CCND2//EREG//FBXW7//ERRFI1//GPRC5A//SOCS5//NUP54//GNAQ//NCK1//GSKIP//RB1//YWHAG//SOCS3//PPM1F//GRM3//COPS8//MDFIC//CRHR1//PPARGC1B//NPNT//CALM2//INHBB//BMP3//BMPR2//TTK//GDF5//ACVR1//LDLR//MTMR9//FKBP15//PPP1R12A//PLEKHF2//TBL1XR1//CXCL9//ADRB1//PTHLH//EGLN1//PDE5A//ADNP//MOB1B//JUN//RAP2C//RASSF2//PIK3CA//PAK6//TGFBR2//SLK//RICTOR//ARPP19//PPP1R1B//MID1//SEMA4C//RPS6KA5//CREBL2//TBK1//PFN2//IFNA1//MET//ROR1//ADCY3//PRKACB//PRKAR2B//PPP1R16B//SPPL3//CNEP1R1//ATXN7//PTGES3//AR//ITSN1//CNOT7//ISL1//EDN1//SYNGAP1//HIPK3//NRBF2//CCL21//RCAN3//UBXN2B//PPP4R2//PPP2R5E//TSC1//TNFAIP3//CCNY//CCNL1//MYOG//ADCYAP1//CACUL1//MMD//RAPGEF2//GRIK2//ZMYND11//WNT7A//YES1//LRP8//CD3E//STRAP//PMEPA1//LDLRAD4//NOG//FAM83D//RANBP9//CCL2//CCL8//GPR55//ACSL1// |
| GO:0001944 | vasculature development | Biological process | 75 | 761 | 902 | 17653 | 1.92880618628191 | 3.22992858644959e-08 | 2.95189284191332e-06 | 7.4908070797908 | 0.0831485587583149 | S1PR1//EFNA1//ELK3//EREG//UNC5B//SIRT1//LEMD3//CLIC4//HOXA3//JUN//KDR//RHOB//MEOX2//TNFRSF12A//PIK3CA//PKNOX1//PTEN//RORA//CCL2//VEGFB//WNT7A//CRKL//FOXF1//SPHK2//TGFBR2//RAPGEF2//EDN1//PPP3R1//ACVR1//GJC1//ZFPM2//RASA1//WT1//CCM2//TEAD2//QKI//EFNB2//BMPR2//RECK//E2F8//SLIT2//SPRED1//SULF1//AGO1//PPARG//FOXJ2//SARS//DCTN5//SNX17//SOX4//ETS1//EGLN1//VASH2//F3//ISL1//ITGB8//BTG1//C5AR1//ADAM12//GJA1//PIK3CB//ERRFI1//FOSL1//SOCS3//MAPK1//GLI3//DLL1//ARID2//ADIPOR2//TNFAIP3//IMMP2L//NOG//PPP1R16B//RAP1A//FBXW7// |
| GO:0010562 | positive regulation of phosphorus metabolic process | Biological process | 100 | 1126 | 902 | 17653 | 1.73809533186564 | 3.34385904210088e-08 | 2.97559456601688e-06 | 7.47575203820911 | 0.110864745011086 | GADD45A//TNF//CRKL//TAOK1//RAF1//RAP1A//C1QTNF2//LPAR1//NRG1//IGF1//NTF3//PIK3CB//PRKAA1//MAPK1//C5AR1//UBE2V1//EDNRB//EFNA1//FMR1//SIRT1//NPTN//IFNG//KDR//MAVS//CCND1//PRR5L//CCND2//EREG//FBXW7//COPS8//MDFIC//SOCS1//PPARGC1B//NPNT//CALM2//INHBB//BMP3//BMPR2//TTK//GDF5//ACVR1//MTMR9//CXCL9//ADRB1//PTHLH//ADNP//RAP2C//RASSF2//PIK3CA//PAK6//TGFBR2//SLK//RICTOR//PPP1R15B//MID1//SEMA4C//RPS6KA5//CREBL2//TBK1//PFN2//IFNA1//MET//ROR1//ADCY3//PRKACB//PRKAR2B//SPPL3//CNEP1R1//PPP1R16B//PPP1R12A//PTGES3//AR//ITSN1//MOB1B//IL2//ISL1//SOCS3//EDN1//PDE5A//IGFBP3//CCL21//CCNY//CCNL1//CCNT2//ADCYAP1//CACUL1//MMD//RAPGEF2//WNT7A//YES1//LRP8//CD3E//JUN//PTEN//CCL2//CCL8//GPR55//ACSL1//PPP2CA//MYOG// |
| GO:0045937 | positive regulation of phosphate metabolic process | Biological process | 100 | 1126 | 902 | 17653 | 1.73809533186564 | 3.34385904210088e-08 | 2.97559456601688e-06 | 7.47575203820911 | 0.110864745011086 | GADD45A//TNF//CRKL//TAOK1//RAF1//RAP1A//C1QTNF2//LPAR1//NRG1//IGF1//NTF3//PIK3CB//PRKAA1//MAPK1//C5AR1//UBE2V1//EDNRB//EFNA1//FMR1//SIRT1//NPTN//IFNG//KDR//MAVS//CCND1//PRR5L//CCND2//EREG//FBXW7//COPS8//MDFIC//SOCS1//PPARGC1B//NPNT//CALM2//INHBB//BMP3//BMPR2//TTK//GDF5//ACVR1//MTMR9//CXCL9//ADRB1//PTHLH//ADNP//RAP2C//RASSF2//PIK3CA//PAK6//TGFBR2//SLK//RICTOR//PPP1R15B//MID1//SEMA4C//RPS6KA5//CREBL2//TBK1//PFN2//IFNA1//MET//ROR1//ADCY3//PRKACB//PRKAR2B//SPPL3//CNEP1R1//PPP1R16B//PPP1R12A//PTGES3//AR//ITSN1//MOB1B//IL2//ISL1//SOCS3//EDN1//PDE5A//IGFBP3//CCL21//CCNY//CCNL1//CCNT2//ADCYAP1//CACUL1//MMD//RAPGEF2//WNT7A//YES1//LRP8//CD3E//JUN//PTEN//CCL2//CCL8//GPR55//ACSL1//PPP2CA//MYOG// |
| GO:0060429 | epithelium development | Biological process | 109 | 1264 | 902 | 17653 | 1.68768506693985 | 3.61911494954554e-08 | 3.12415823996295e-06 | 7.44139762270542 | 0.120842572062084 | EDN1//PPP3R1//TGFBR2//ACVR1//NPNT//ROBO2//SDC1//WT1//SLIT2//GLI3//FZD3//TP63//EP300//FOXF1//DLL1//MEOX2//NOG//SOX4//LRP6//SEMA4C//PRKACB//TSC1//FZD6//TEAD2//CCM2//CLIC4//MET//APCDD1//WNT10A//GJA1//ARHGAP12//ESR1//KDR//NEUROD1//PALLD//AR//FRMD6//TFAP2A//GSTM3//GRSF1//GSC//FOXA1//WNT7A//EREG//TGM3//CNN3//NR5A2//GSTA2//ANXA7//PPARG//IFNG//CCND1//DLG5//EN1//KRT80//KRTAP5-6//KRTAP2-4//CEBPB//ID2//MAPK1//JUN//WDR1//FOXF2//FLNA//TMEFF2//MSN//S1PR1//ZEB1//BTG1//MYCN//SERPINB13//ERRFI1//CBFB//SRSF6//TNF//FRZB//PRICKLE2//ROR1//PSMA2//SMURF1//PSMD12//RDH10//NFIB//FOXP2//YIPF6//SOCS3//SULF1//BTBD7//ETV5//BMPR2//NRG1//PPP1R16B//RAP1A//RAP1B//RAPGEF2//RHOB//WNT3//MAF//TMOD1//E2F8//ARX//KLHL3//EFNB2//RAP2C//ARID4B//FOXJ2//FOXP1//PTEN//CLOCK// |
| GO:0051960 | regulation of nervous system development | Biological process | 79 | 820 | 902 | 17653 | 1.88549429452166 | 3.64186431868357e-08 | 3.12415823996295e-06 | 7.43867623831723 | 0.0875831485587583 | GLI3//FZD3//PTEN//SNAP25//STMN2//ADCYAP1//NPTN//NCK1//SERPINI1//RAP1A//SCN1B//RAPGEF2//LPAR1//EFNB2//MYLIP//PTPRG//SPOCK1//TSC1//BAG5//EDNRB//DPYSL2//RAB21//WNT7A//TNFRSF21//WASF3//NRG1//MYCN//DLL1//NTF3//YWHAG//NREP//DDX6//ID2//ISL1//DNMT3B//ETV5//MMD//FOXA1//NEUROD1//NEUROG1//ZEB1//GDF5//ADNP//TNFRSF12A//ULK2//WNT3//NOG//WDR1//PPARG//FMR1//HECW2//FXR1//PLXNA4//BMPR2//SEMA4C//POU4F1//EFNA1//POU3F2//TRAK2//SYNGAP1//ROBO2//SLIT2//DBN1//CAMSAP2//SLITRK3//DLG5//MYB//CPEB3//ZMYND8//IL2//MAPK6//ARC//LRP8//LDLR//PRKACB//SMURF1//SYT1//PLAA//FLNA// |
| GO:0010628 | positive regulation of gene expression | Biological process | 148 | 1877 | 902 | 17653 | 1.54315455974824 | 3.64939377431721e-08 | 3.12415823996295e-06 | 7.43777927316963 | 0.164079822616408 | EP300//CPEB3//FMR1//SERP1//PRR16//MAPK1//SOX4//FXR1//CREBL2//ESR1//ETS1//ATF5//FOXF1//FOXF2//FLI1//PHF8//NR5A2//BAMBI//GLI3//ATAD2//MDFIC//ID2//IGF1//IRF1//AR//JUN//LRP6//MYB//MYBL1//MYCN//NEUROD1//NFE2//NPAS2//NPAT//OTX2//PPARG//TRERF1//RB1//HIVEP3//RORA//PRDM16//SMARCA2//SS18//TFAP2A//TNF//UBE2V1//WNT7A//WT1//TBL1XR1//TEAD2//TP63//RUNX3//FUBP3//ACVR1//CLOCK//ZNF516//INSIG2//NCK1//ZBTB18//CEBPB//ADCYAP1//PPARGC1B//CREBRF//E2F5//S1PR1//EDN1//ELK3//EN1//ETV5//MED19//SIRT1//ZFPM2//KPNA6//AGO1//DLL1//TBK1//GTF2H1//CNOT7//H2AFZ//NRG1//FOXA1//IL2//IRF2//ISL1//MAF//MEF2A//MEOX2//MET//MYOG//PPP1R12A//NEUROG1//NFIA//NFIB//NFIC//NHLH2//NR4A2//PAX5//IER5//ARMCX3//KLF13//ARID4B//PKNOX1//CYTL1//POU3F2//POU4F1//EGLN1//PPP3R1//FOXJ2//CCNL1//MAVS//RAF1//ATXN7//BMPR2//ZEB1//TFE3//KLF10//TXK//YES1//E2F8//FOSL1//PCGF5//KLF7//CBFB//CCNT2//NOG//RPS6KA5//ST18//BOLL//TOB1//PRKAA1//YTHDF2//FBXW7//NPNT//ARX//DNMT3B//RAB3GAP1//GJA1//IFNG//ITGB8//LDLR//MSN//PIK3CB//PTEN//WNT3//WNT10A//CD3E//QKI//PPM1F// |
| GO:0032270 | positive regulation of cellular protein metabolic process | Biological process | 127 | 1546 | 902 | 17653 | 1.60770445438195 | 4.09763568922527e-08 | 3.46403877077881e-06 | 7.38746665627947 | 0.14079822616408 | GADD45A//TNF//CRKL//TAOK1//RAF1//RAP1A//C1QTNF2//LPAR1//NRG1//IGF1//NTF3//PIK3CB//PRKAA1//MAPK1//C5AR1//UBE2V1//EDNRB//EFNA1//FMR1//SIRT1//NPTN//IFNG//KDR//MAVS//CCND1//PRR5L//CCND2//EREG//FBXW7//EGLN3//F3//PMAIP1//PPARG//CASP2//COPS8//MDFIC//SOCS1//CALM2//INHBB//BMP3//BMPR2//TTK//GDF5//ACVR1//CPEB3//SERP1//PRR16//SOX4//FXR1//NDFIP2//RAP2C//RASSF2//ADNP//PIK3CA//PAK6//TGFBR2//SLK//RICTOR//ANKIB1//LONRF3//LONRF1//SOCS5//PPP1R15B//MID1//SEMA4C//RPS6KA5//CREBL2//TBK1//PFN2//IFNA1//RWDD3//ADCY3//PRKACB//PRKAR2B//ISL1//SPPL3//CNEP1R1//PPP1R16B//PPP1R12A//NCK1//IL2//SOCS3//PPM1F//EDN1//PDE5A//IGFBP3//AR//MET//ROR1//CCNY//CCNL1//CCNT2//ADCYAP1//CACUL1//MMD//CCL21//RAPGEF2//BOLL//WNT7A//YES1//LRP8//CD3E//SH3D19//DCUN1D4//ARRDC3//DNMT3B//MYB//JARID2//NPNT//JUN//PTEN//CCL2//CCL8//GPR55//ACSL1//PPP2CA//NMI//RAB3GAP1//MSN//TNFAIP3//YTHDF2//UBE2I//ESR1//BAG6//LDLR//SMURF1//ST18// |
| GO:0000902 | cell morphogenesis | Biological process | 91 | 997 | 902 | 17653 | 1.78631570987908 | 4.17023320485863e-08 | 3.48188730425419e-06 | 7.37983965803551 | 0.100886917960089 | CLIC4//MET//PALLD//AR//FRMD6//SLITRK3//OGN//POU4F1//PRELP//WNT7A//SLIT2//RANBP9//ARX//DPYSL2//EFNA1//EFNB2//GLI3//OTX2//PIK3CA//PIK3CB//ENAH//MAPK1//ROBO2//SCN1B//WNT3//KLF7//RPS6KA5//NRXN3//FEZ2//NOG//CFDP1//WASF3//PALM2//LPAR1//EPS8//BAMBI//ANXA7//KDR//RHOB//RND3//MSN//BRWD1//RASA1//CCL2//PLXNA4//WDR1//FLNA//NR4A2//PTEN//NFIB//SH3D19//CAMSAP1//ARC//EP300//RAB21//ISL1//UNC5B//VAMP3//FZD3//ADNP//NRG1//TNFRSF12A//ID2//RB1//ULK2//SLC9A6//GJA1//GPM6A//NTF3//SGK1//HPRT1//MEF2A//TRAK2//FMR1//HECW2//FXR1//BMPR2//SEMA4C//POU3F2//SYNGAP1//RAPGEF2//DOCK10//LRP8//SNX2//YTHDF2//CRKL//SMURF1//SYT1//PLAA//CDH20//SS18// |
| GO:0010468 | regulation of gene expression | Biological process | 321 | 4873 | 902 | 17653 | 1.28920091385493 | 4.62144576392863e-08 | 3.81156557334748e-06 | 7.33522213936323 | 0.355875831485588 | NPAT//TSHZ1//ZBTB18//CEBPB//TCERG1//STRAP//KLF12//GSC//ARX//DNMT3B//EDN1//EDNRB//EFNA1//CC2D1B//EN1//EP300//ESR1//JAZF1//CPEB3//FOXF1//MYT1L//SIN3B//SIRT1//ZFPM2//DNAJB5//GLI3//CNOT7//HIC1//FOXA1//HSBP1//ID2//IFNG//IRF2//AR//ISL1//JARID2//JUN//MAF//MDM4//MEF2A//MYB//NFIA//NFIB//NFIC//NR4A2//PAX5//KLF3//POU4F1//PPARG//ZBTB4//RB1//RBBP8//CCND1//RNF2//SARS//PRDM16//SMARCA2//SUV39H1//ZEB1//TFAP2A//TGIF1//KLF10//TNF//UBE2I//WT1//ZNF217//LRP8//BHLHE41//TBL1XR1//E2F8//ARID5B//BHLHE40//TP63//RUNX3//NOG//FOXP2//PHF14//PNRC1//DCP2//PPP2CA//CNOT6L//PAN3//CNOT6//PATL1//MBNL2//RBFOX3//FMR1//MBNL1//RBM25//SRSF2//SRSF6//AGO1//SEPSECS//SECISBP2L//BAHD1//H2AFZ//ARID4B//DIRAS3//HIPK3//ZMYND11//ZNF526//PPARGC1B//ZNF800//ARID2//ELAVL2//ATF5//ZFP30//ELL2//KDM2A//MYCBP2//FLII//ZMYND8//NR5A2//ZNF521//ZBTB11//GTF2A1//GTF2E1//NRBF2//HIVEP2//HMGB3//ZNF680//ZNF662//AFF1//MLLT6//OGG1//ASCC1//PHF20L1//COMMD10//BNC2//ZNF532//SLC2A4RG//PAK6//PHTF2//ZBTB26//HIVEP3//RORA//SOX4//BTF3//BTG1//UBE2V1//ZNF3//ZNF131//ZNF227//CSDE1//ZNF655//SAP30L//SNIP1//ANP32A//TEAD2//RPS6KA5//CLOCK//ZNF516//CREBL2//ZNF367//ELK3//EMX2//ETS1//ETV5//FOXF2//FLI1//ADNP//EPC2//HBP1//FOXP1//ATAD2//HOXA3//HOXD1//BOLA3//NR3C2//MYBL1//MYCN//NEUROG1//NFE2//NHLH2//KLF13//BRWD1//POU3F2//MED9//TRERF1//FOXJ2//MIER1//PURA//RFX7//TRAK2//SMARCD2//SOX5//MED22//TXK//ZNF708//ZSCAN5A//FOSL1//LCOR//KLF7//CBFB//BDP1//DDX6//TSC1//KBTBD8//G3BP2//NANOS1//RAB3GAP1//GJA1//IGF1//ITGB8//LDLR//MSN//PIK3CB//PRKAA1//MAPK1//PTEN//WNT3//WNT10A//FUBP3//CD3E//QKI//PPM1F//TBK1//ANXA7//KDR//NDFIP2//SERP1//PRR16//FXR1//CELF1//FLNA//PHF8//BAMBI//MDFIC//IRF1//LRP6//NEUROD1//NPAS2//OTX2//SS18//WNT7A//ACVR1//DNAJB6//EREG//NRG1//RASD1//ATXN1//BTAF1//TOB1//TIA1//MEX3B//TSN//CR2//C5AR1//SMURF1//BMP3//BMPR2//GDF5//SULF1//LEMD3//CTDSPL2//TNRC6B//RWDD3//TNFAIP3//GFPT1//INSIG2//NCK1//EGLN1//FZD6//FASTK//YTHDF2//PSMA2//PSMD12//ADCYAP1//CREBRF//E2F5//S1PR1//MED19//KPNA6//DLL1//GTF2H1//IL2//MEOX2//MET//MYOG//PPP1R12A//IER5//ARMCX3//PKNOX1//CYTL1//PPP3R1//CCNL1//MAVS//RAF1//ATXN7//TFE3//YES1//PCGF5//CCNT2//ST18//PAIP2//BOLL//C8ORF44-SGK3//SGK3//SGK1//RHEBL1//ROR1//IRAK4//NUP54//PRR5L//GTPBP1//EGLN3//PRKACB//CAST//CPEB2//PPP1R15B//TMBIM6//FBXW7//NPNT//CPEB1//RICTOR//PIK3CA//PPP2CB//MAPK6//PTHLH//TGFBR2//DDX3Y// |
| GO:0072358 | cardiovascular system development | Biological process | 75 | 769 | 902 | 17653 | 1.90874058226337 | 4.98209727638255e-08 | 4.05950890122593e-06 | 7.30258779703562 | 0.0831485587583149 | S1PR1//EFNA1//ELK3//EREG//UNC5B//SIRT1//LEMD3//CLIC4//HOXA3//JUN//KDR//RHOB//MEOX2//TNFRSF12A//PIK3CA//PKNOX1//PTEN//RORA//CCL2//VEGFB//WNT7A//CRKL//FOXF1//SPHK2//TGFBR2//RAPGEF2//EDN1//PPP3R1//ACVR1//GJC1//ZFPM2//RASA1//WT1//CCM2//TEAD2//QKI//FBXW7//EFNB2//BMPR2//RECK//E2F8//SLIT2//SPRED1//SULF1//AGO1//PPARG//FOXJ2//SARS//DCTN5//SNX17//SOX4//ETS1//EGLN1//VASH2//F3//ISL1//ITGB8//BTG1//C5AR1//ADAM12//GJA1//PIK3CB//ERRFI1//FOSL1//SOCS3//MAPK1//GLI3//DLL1//ARID2//ADIPOR2//TNFAIP3//IMMP2L//NOG//PPP1R16B//RAP1A// |
| GO:0044093 | positive regulation of molecular function | Biological process | 139 | 1742 | 902 | 17653 | 1.56163176103111 | 5.05142305653265e-08 | 4.06699692039647e-06 | 7.29658625781438 | 0.15410199556541 | GADD45A//TNF//CRKL//TAOK1//RAF1//RAP1A//C1QTNF2//LPAR1//NRG1//IGF1//NTF3//PIK3CB//PRKAA1//MAPK1//C5AR1//UBE2V1//EREG//FBXW7//EGLN3//F3//PMAIP1//PPARG//CASP2//NMUR1//GNAQ//TXK//GPR55//COPS8//MDFIC//SOCS1//PPARGC1B//NPNT//ESR1//MTMR9//SPPL3//EP300//BAMBI//RAPGEF2//ADNP//PIK3CA//PAK6//TGFBR2//SLK//RICTOR//STIM2//C8ORF44-SGK3//SGK2//SGK3//SGK1//PLAA//CALM2//PPP1R15B//DNAJB6//DNAJA2//PFN2//LRP8//RPS6KA5//RALBP1//ADCYAP1//ARAP2//ADRB1//DENND1B//S1PR1//RAB3GAP1//ELMOD2//AGFG1//JUN//ARHGAP1//ARHGAP6//ERRFI1//RIN2//DOCK10//RASA1//CCL2//CCL8//CCL21//BNIP2//SYNGAP1//ARHGAP12//GIT2//KDR//MET//ROR1//ADCY3//PRKACB//PRKAR2B//PPP1R12A//TRIM23//NR4A2//SIRT1//PPM1F//ISL1//EDN1//PDE5A//CCNY//CCNL1//CCND1//CCND2//CCNT2//CACUL1//MMD//RASSF2//ABHD5//FOXA1//LRP6//NEUROD1//NEUROG1//NHLH2//CYTL1//PTEN//MAVS//FOSL1//ARID5B//RHEBL1//AR//IRAK4//CLOCK//MID1IP1//DCUN1D4//ARRDC3//PTGES3//IFNG//EDN2//EFNA1//ACSL1//PPP2CA//RB1//TBC1D12//TSC1//SYDE2//FMR1//UBE2I//PPP2CB//CNOT6//DYNLL2//GLRX//POU4F1//ARC//ST18// |
| GO:0065007 | biological regulation | Biological process | 701 | 12351 | 902 | 17653 | 1.11077956110451 | 5.43006100564418e-08 | 4.32041206837313e-06 | 7.26519529117252 | 0.777161862527716 | USP28//CLOCK//GADD45A//GTF2H1//PTEN//CCNT2//DIRAS3//NPAT//TSHZ1//ZBTB18//CEBPB//TCERG1//STRAP//KLF12//GSC//ARX//DNMT3B//EDN1//EDNRB//EFNA1//CC2D1B//EN1//EP300//ESR1//JAZF1//CPEB3//FOXF1//MYT1L//SIN3B//SIRT1//ZFPM2//DNAJB5//GLI3//CNOT7//HIC1//FOXA1//HSBP1//ID2//IFNG//IRF2//AR//ISL1//JARID2//JUN//MAF//MDM4//MEF2A//MYB//NFIA//NFIB//NFIC//NR4A2//PAX5//KLF3//POU4F1//PPARG//ZBTB4//RB1//RBBP8//CCND1//RNF2//SARS//PRDM16//SMARCA2//SUV39H1//ZEB1//TFAP2A//TGIF1//KLF10//TNF//UBE2I//WT1//ZNF217//LRP8//BHLHE41//TBL1XR1//E2F8//ARID5B//BHLHE40//TP63//RUNX3//NOG//FOXP2//PHF14//KCNH4//RANBP9//SPRED1//EREG//RASGEF1A//NRG1//IL2//MET//MAPK1//MAPK6//PSMA2//PSMD12//RAF1//RASA1//CCL2//SYNGAP1//RAPGEF2//PNRC1//DCP2//PPP2CA//CRKL//TAOK1//RAP1A//C1QTNF2//LPAR1//IGF1//NTF3//PIK3CB//PRKAA1//C5AR1//UBE2V1//DUSP1//CNOT6L//PAN3//CNOT6//PATL1//MBNL2//RBFOX3//FMR1//MBNL1//RBM25//SRSF2//SRSF6//PTGES3//AGO1//SNAP25//CHRNB4//GNAQ//KCNB1//SEPSECS//SECISBP2L//C8ORF44-SGK3//SGK2//SGK3//NANOS1//IGFBP3//SGK1//SPOCK1//ADRB1//EDN2//ARRDC3//DOCK10//RPS6KA5//PMAIP1//BCL2A1//NSG1//EPS15//VAMP3//TNFAIP3//SOCS1//LRP6//PRR5L//PPP1R15B//SLIT2//NPTN//KDR//MAVS//CCND2//SULF1//GJA1//F3//RICTOR//PPP1R16B//BMPR2//VEGFB//VASH2//TSC1//CCL21//CBFB//CALM2//ARL6IP1//FZD3//MYCN//TGFBR2//RDH10//NUP54//PRKACB//SKP1//IRAK4//BAG6//CR2//CD3E//CCL8//CXCL9//SKAP2//IRF1//ADCYAP1//FOXP1//ADCY3//PRKAR2B//S1PR1//SPHK2//E2F5//CDK15//EPS8//IFNA1//NFE2//APCDD1//CCNY//HBP1//GRK6//STRN//WNT3//WNT7A//CXXC4//WNT10A//TNRC6B//PPP3R1//FZD6//CNIH1//CDS1//IQGAP2//RALBP1//TMED1//RASSF8//ARAP2//CLCN6//CNGA3//CNTFR//CREBL2//DPYSL2//DTNA//ELK3//CD2AP//GABRA5//APPL1//ATP2C1//GLRB//LRP12//HIVEP2//NR3C2//PPP1R12A//HPCAL4//GULP1//RASD1//KCNK10//CYTL1//NDFIP2//PPP2R5E//PSD//MIER1//HIVEP3//SMOC1//SMOC2//ZYX//GLRA3//SNX27//SYDE2//PPP1R1B//PPFIA1//OR6A2//PDE5A//GPRC5A//DLG5//PLAA//ARHGAP12//GTPBP1//FEZ2//ZNF516//CD69//ULK2//SNX17//ROR1//PTPRG//TXK//YES1//CD8A//AKAP11//DGKH//NRBP1//ARHGEF4//STK33//SS18//ANP32A//DGKE//RALGPS1//PALM2//FBXW7//ERRFI1//SOCS5//NLN//DNAJC16//GLRX//NHLRC2//ESCO2//BAHD1//H2AFZ//ARID4B//HIPK3//ZMYND11//ZNF526//PPARGC1B//ZNF800//ARID2//ELAVL2//ATF5//ZFP30//ELL2//KDM2A//MYCBP2//FLII//ZMYND8//NR5A2//ZNF521//ZBTB11//GTF2A1//GTF2E1//NRBF2//HMGB3//ZNF680//ZNF662//AFF1//MLLT6//OGG1//ASCC1//PHF20L1//COMMD10//BNC2//ZNF532//SLC2A4RG//PAK6//PHTF2//ZBTB26//RORA//SOX4//BTF3//BTG1//ZNF3//ZNF131//ZNF227//CSDE1//ZNF655//SAP30L//SNIP1//TEAD2//ZNF367//EMX2//ETS1//ETV5//FOXF2//FLI1//ADNP//EPC2//ATAD2//HOXA3//HOXD1//BOLA3//MYBL1//NEUROG1//NHLH2//KLF13//BRWD1//POU3F2//MED9//TRERF1//FOXJ2//PURA//RFX7//TRAK2//SMARCD2//SOX5//MED22//ZNF708//ZSCAN5A//FOSL1//LCOR//KLF7//BDP1//DDX6//KBTBD8//NCK1//GSKIP//YWHAG//SOCS3//PPM1F//GRIK2//ANXA7//SLC24A3//STIM2//SLC31A2//ABCB7//EGLN1//SLC26A4//EGLN3//CASP2//CHRM2//TNNI1//STMN2//CAMSAP1//CAMSAP2//MID1//MID1IP1//NAV3//CLIC4//RRAGD//CUL5//NABP1//TTK//CDC14A//CBLL1//PLXNA4//CRHR1//IGSF3//TSPAN11//PIK3CA//SMURF1//GDF5//ACVR1//NMUR1//RAPGEF4//TAPT1//FRZB//OR4N4//GRM3//KCNK2//RHOB//RGS7BP//PTHLH//ITSN1//SOS2//GPR55//FLNA//ANO1//SPPL3//RIC3//HOMER1//NXPH3//SORCS1//SORCS2//SUSD5//DLL1//TMEM17//EVC//DZIP1//ITGB8//CCM2//RCAN3//SAMD14//TBK1//COPS8//G3BP2//MDFIC//NMI//NEUROD1//RAB18//RGL1//RND3//ARHGAP1//RIN2//ARHGAP6//SYT1//CDK19//SLK//TRPC3//SLC25A6//MARCKS//KLHL20//ZFAND6//BNIP2//TMBIM6//UGT2B7//GIT2//DNAJA2//CACUL1//EFNB2//BAMBI//TOB1//TES//MYOG//ST18//CFDP1//WASF3//MSN//WDR1//HS3ST5//TIA1//SHCBP1//OTX2//NPNT//ADIPOR2//PYURF//PIGA//RWDD3//LASP1//SH3BGRL//NEDD1//CEP41//PI15//PPP2CB//DDX3Y//TXLNA//INHBB//OGN//BMP3//DRAM1//MTMR9//PPP4R2//RAB3GAP1//LDLR//FUBP3//QKI//PFN2//LDLRAD4//C5ORF30//SCN1B//CHMP3//VPS4B//C2CD5//CNEP1R1//ABHD5//INSIG2//FKBP15//PLEKHF2//SERPINI1//SERPINB13//RECK//SLC41A1//MYLIP//BAG5//PMEPA1//SLC6A1//UNC5B//KCNJ2//ARPP19//SERP1//PRR16//FXR1//RAB12//SESN1//SNX5//CELF1//CPEB2//TNFRSF21//PHF8//NPAS2//DNAJB6//ATXN1//BTAF1//NREP//CRTAP//OTUD3//MORC3//USP27X//CCT6A//RASSF2//MEX3B//RAB21//RAB8B//SYT6//ACSL1//PLP2//SDC1//SIRPA//SH3D19//ARC//DENND1B//CPD//HECW2//SNX19//SPOPL//SHISA2//PKNOX1//DHRS3//EI24//FAM89B//SEMA4C//PTP4A1//TMEFF2//RAP2C//TSN//LEMD3//CTDSPL2//GPCPD1//CFL2//CREBRF//CLIP1//TBC1D12//SLC9A6//RHEBL1//MOB1B//FAM83D//IQSEC2//FBXO8//AGFG1//ANKIB1//LONRF3//LONRF1//KIF13A//PCBP2//POLR3H//RAB30//RAB9B//RAB28//RAP1B//DBN1//CNN3//PXK//STX6//MMD//MEMO1//KLHL42//GFPT1//FRMD6//SBF2//ELMOD2//UBA5//EDARADD//TNFRSF12A//ARFIP1//KCNA4//KCNJ3//KCNJ10//PLEKHG4B//ROBO2//GSTA2//SYBU//DNAJB9//ASNA1//SEC61A2//ZMAT3//IER5//ATXN7//SLC26A7//RIMS4//GCNT4//SLC44A1//ADH4//RDH14//PHLDA3//TRIM23//TMEM64//FASTK//YTHDF2//UBXN2B//PEX5L//TUB//DLK2//TFE3//ELOVL5//CCNL1//ADAM12//RNF167//MED19//KPNA6//MEOX2//ARMCX3//PCGF5//PAIP2//BOLL//RAET1E//HPRT1//KLHL3//GIGYF1//TMOD3//NPDC1//GCSAML//NOVA1//ZER1//DCUN1D4//GPM6A//TMOD1//SLITRK3//CUTC//STEAP2//SLC25A28//PRICKLE2//KCNE1//BTBD7//SLC30A6//KCNIP4//DENND6A//TRAPPC8//KCNAB2//SLC16A2//FIBIN//XKR8//GJC1//MIEF1//ANAPC15//LPCAT3//SIVA1//CAST//FAM155A//GPHN//BSN//LYPD6//DYNLL2//SERINC3//ABHD17C//SEPT9//HSBP1L1//MTRR//SPTSSB//SESTD1//FAM168A//IER3IP1//CPEB1// |
| GO:2000112 | regulation of cellular macromolecule biosynthetic process | Biological process | 293 | 4376 | 902 | 17653 | 1.31039519126702 | 5.56356038868887e-08 | 4.33714729859165e-06 | 7.25464719361697 | 0.324833702882483 | NPAT//TSHZ1//ZBTB18//CEBPB//TCERG1//STRAP//KLF12//GSC//ARX//DNMT3B//EDN1//EDNRB//EFNA1//CC2D1B//EN1//EP300//ESR1//JAZF1//CPEB3//FOXF1//MYT1L//SIN3B//SIRT1//ZFPM2//DNAJB5//GLI3//CNOT7//HIC1//FOXA1//HSBP1//ID2//IFNG//IRF2//AR//ISL1//JARID2//JUN//MAF//MDM4//MEF2A//MYB//NFIA//NFIB//NFIC//NR4A2//PAX5//KLF3//POU4F1//PPARG//ZBTB4//RB1//RBBP8//CCND1//RNF2//SARS//PRDM16//SMARCA2//SUV39H1//ZEB1//TFAP2A//TGIF1//KLF10//TNF//UBE2I//WT1//ZNF217//LRP8//BHLHE41//TBL1XR1//E2F8//ARID5B//BHLHE40//TP63//RUNX3//NOG//FOXP2//PHF14//SEPSECS//SECISBP2L//ESCO2//PPP2CA//BAHD1//H2AFZ//HIPK3//ZMYND11//PNRC1//ZNF526//PPARGC1B//ZNF800//ARID2//ELAVL2//ATF5//ZFP30//ELL2//KDM2A//MYCBP2//FLII//ZMYND8//CNOT6L//NR5A2//ZNF521//ZBTB11//GTF2A1//GTF2E1//NRBF2//HIVEP2//HMGB3//ZNF680//ZNF662//AFF1//MLLT6//OGG1//ASCC1//PHF20L1//COMMD10//BNC2//ZNF532//SLC2A4RG//PAK6//PHTF2//CNOT6//ZBTB26//HIVEP3//RORA//SOX4//BTF3//BTG1//UBE2V1//ZNF3//ZNF131//ZNF227//CSDE1//ZNF655//SAP30L//SNIP1//ANP32A//TEAD2//RPS6KA5//CLOCK//ZNF516//CREBL2//ZNF367//ELK3//EMX2//ETS1//ETV5//FOXF2//FLI1//ADNP//EPC2//HBP1//FOXP1//ATAD2//HOXA3//HOXD1//BOLA3//NR3C2//MYBL1//MYCN//NEUROG1//NFE2//NHLH2//KLF13//ARID4B//BRWD1//POU3F2//MED9//TRERF1//FOXJ2//MIER1//PURA//RFX7//TRAK2//SMARCD2//SOX5//MED22//TXK//ZNF708//ZSCAN5A//FOSL1//LCOR//KLF7//CBFB//BDP1//DDX6//TSC1//KBTBD8//G3BP2//AGO1//IGF1//CCL21//FMR1//SERP1//PRR16//MAPK1//FXR1//FLNA//PHF8//BAMBI//MDFIC//IRF1//LRP6//NEUROD1//NPAS2//OTX2//SS18//WNT7A//FUBP3//ACVR1//DNAJB6//EREG//NRG1//RASD1//ATXN1//BTAF1//PPM1F//TOB1//CELF1//NANOS1//TIA1//MEX3B//SMURF1//BMP3//BMPR2//GDF5//SULF1//LEMD3//CTDSPL2//RWDD3//TNFAIP3//DCP2//CCT6A//FBXW7//INSIG2//TNRC6B//NCK1//EGLN1//FZD6//C1QTNF2//ADCYAP1//CREBRF//E2F5//S1PR1//MED19//KPNA6//DLL1//TBK1//GTF2H1//IL2//MEOX2//MET//MYOG//PPP1R12A//IER5//ARMCX3//PKNOX1//CYTL1//PPP3R1//CCNL1//MAVS//RAF1//ATXN7//TFE3//YES1//PCGF5//CCNT2//ST18//PAIP2//BOLL//C8ORF44-SGK3//SGK3//SGK1//PTEN//RHEBL1//ROR1//IRAK4//PTGES3//NUP54//YTHDF2//QKI//EGLN3//PSMA2//PSMD12//CPEB2//RAB3GAP1//PRKAA1//PPP1R15B//TMBIM6//DUSP1//GJA1//KCNK2//NPNT//CPEB1// |
| GO:0009890 | negative regulation of biosynthetic process | Biological process | 143 | 1809 | 902 | 17653 | 1.54706818212461 | 5.57935553715028e-08 | 4.33714729859165e-06 | 7.25341596285874 | 0.158536585365854 | TSHZ1//ZBTB18//CEBPB//TCERG1//STRAP//KLF12//GSC//ARX//DNMT3B//EDN1//EDNRB//EFNA1//CC2D1B//EN1//EP300//ESR1//JAZF1//CPEB3//FOXF1//MYT1L//SIN3B//SIRT1//ZFPM2//DNAJB5//GLI3//CNOT7//HIC1//FOXA1//HSBP1//ID2//IFNG//IRF2//AR//ISL1//JARID2//JUN//MAF//MDM4//MEF2A//MYB//NFIA//NFIB//NFIC//NR4A2//PAX5//KLF3//POU4F1//PPARG//ZBTB4//RB1//RBBP8//CCND1//RNF2//SARS//PRDM16//SMARCA2//SUV39H1//ZEB1//TFAP2A//TGIF1//KLF10//TNF//UBE2I//WT1//ZNF217//LRP8//BHLHE41//TBL1XR1//E2F8//ARID5B//BHLHE40//TP63//RUNX3//NOG//FOXP2//PHF14//BAHD1//H2AFZ//GRM3//AGO1//SNIP1//FBXW7//INSIG2//FLNA//DNAJB6//PPARGC1B//ELK3//EREG//ATF5//FOXF2//FOXP1//MDFIC//NRG1//IRF1//RASD1//PURA//ATXN1//BTAF1//RPS6KA5//CLOCK//PPM1F//TOB1//CELF1//FMR1//NANOS1//TIA1//TSC1//FXR1//MEX3B//EGLN1//ATAD2//MIER1//DCP2//ERRFI1//RAP1A//CNOT6//TNRC6B//NMI//TMEFF2//MYOG//PAIP2//PRKAA1//RAPGEF2//INHBB//NUP54//CNOT6L//YTHDF2//QKI//PHF8//CPEB2//ZMYND11//HSBP1L1//NPAT//SRSF2//ZMYND8//MYCN//NCK1//TMBIM6//DUSP1//GJA1//KCNK2//BMPR2//CPEB1// |
| GO:0002009 | morphogenesis of an epithelium | Biological process | 57 | 528 | 902 | 17653 | 2.11277338238258 | 7.56344511497828e-08 | 5.81267946734069e-06 | 7.12128034026362 | 0.0631929046563193 | EDN1//PPP3R1//TGFBR2//ACVR1//NPNT//GLI3//WT1//FZD3//TP63//NOG//SOX4//LRP6//SEMA4C//PRKACB//TSC1//FZD6//TEAD2//GJA1//DLL1//ARHGAP12//TFAP2A//GRSF1//DLG5//TGM3//WNT10A//WNT7A//WDR1//FOXF2//FLNA//TMEFF2//FOXF1//MSN//CLIC4//MET//MYCN//SLIT2//FRZB//PRICKLE2//ROR1//PSMA2//SMURF1//PSMD12//RDH10//FOXA1//AR//ESR1//SOCS3//SULF1//BTBD7//TNF//ETV5//RHOB//CCM2//KLHL3//EFNB2//FOXP1//PTEN// |
| GO:0045892 | negative regulation of transcription, DNA-templated | Biological process | 102 | 1176 | 902 | 17653 | 1.69748065523327 | 7.85957568358233e-08 | 5.97239442113116e-06 | 7.10460089966708 | 0.113082039911308 | TSHZ1//ZBTB18//CEBPB//TCERG1//STRAP//KLF12//GSC//ARX//DNMT3B//EDN1//EDNRB//EFNA1//CC2D1B//EN1//EP300//ESR1//JAZF1//CPEB3//FOXF1//MYT1L//SIN3B//SIRT1//ZFPM2//DNAJB5//GLI3//CNOT7//HIC1//FOXA1//HSBP1//ID2//IFNG//IRF2//AR//ISL1//JARID2//JUN//MAF//MDM4//MEF2A//MYB//NFIA//NFIB//NFIC//NR4A2//PAX5//KLF3//POU4F1//PPARG//ZBTB4//RB1//RBBP8//CCND1//RNF2//SARS//PRDM16//SMARCA2//SUV39H1//ZEB1//TFAP2A//TGIF1//KLF10//TNF//UBE2I//WT1//ZNF217//LRP8//BHLHE41//TBL1XR1//E2F8//ARID5B//BHLHE40//TP63//RUNX3//NOG//FOXP2//PHF14//BAHD1//H2AFZ//FLNA//ATAD2//MIER1//PHF8//ZMYND8//NCK1//TMBIM6//DNAJB6//PPARGC1B//ELK3//EREG//ATF5//FOXF2//FOXP1//MDFIC//NRG1//IRF1//RASD1//PURA//ATXN1//BTAF1//RPS6KA5//CLOCK//PPM1F// |
| GO:0001568 | blood vessel development | Biological process | 72 | 736 | 902 | 17653 | 1.91454979273113 | 8.28981393298026e-08 | 6.22933462541617e-06 | 7.08145521719241 | 0.0798226164079823 | S1PR1//EFNA1//ELK3//EREG//UNC5B//SIRT1//LEMD3//CLIC4//HOXA3//JUN//KDR//RHOB//MEOX2//TNFRSF12A//PIK3CA//PKNOX1//PTEN//RORA//CCL2//VEGFB//WNT7A//EDN1//PPP3R1//TGFBR2//ACVR1//GJC1//FOXF1//ZFPM2//RASA1//WT1//CCM2//TEAD2//QKI//RECK//E2F8//EFNB2//SLIT2//SPRED1//SULF1//AGO1//PPARG//FOXJ2//SARS//DCTN5//SNX17//SOX4//ETS1//EGLN1//VASH2//F3//ISL1//ITGB8//BTG1//C5AR1//ADAM12//GJA1//PIK3CB//FOSL1//SOCS3//MAPK1//GLI3//BMPR2//DLL1//ARID2//ADIPOR2//TNFAIP3//NOG//PPP1R16B//RAP1A//RAPGEF2//CRKL//SPHK2// |
| GO:0031327 | negative regulation of cellular biosynthetic process | Biological process | 141 | 1789 | 902 | 17653 | 1.54248431223577 | 8.43362039059705e-08 | 6.24562646605473e-06 | 7.07398595110148 | 0.156319290465632 | TSHZ1//ZBTB18//CEBPB//TCERG1//STRAP//KLF12//GSC//ARX//DNMT3B//EDN1//EDNRB//EFNA1//CC2D1B//EN1//EP300//ESR1//JAZF1//CPEB3//FOXF1//MYT1L//SIN3B//SIRT1//ZFPM2//DNAJB5//GLI3//CNOT7//HIC1//FOXA1//HSBP1//ID2//IFNG//IRF2//AR//ISL1//JARID2//JUN//MAF//MDM4//MEF2A//MYB//NFIA//NFIB//NFIC//NR4A2//PAX5//KLF3//POU4F1//PPARG//ZBTB4//RB1//RBBP8//CCND1//RNF2//SARS//PRDM16//SMARCA2//SUV39H1//ZEB1//TFAP2A//TGIF1//KLF10//TNF//UBE2I//WT1//ZNF217//LRP8//BHLHE41//TBL1XR1//E2F8//ARID5B//BHLHE40//TP63//RUNX3//NOG//FOXP2//PHF14//BAHD1//H2AFZ//GRM3//AGO1//SNIP1//FBXW7//FLNA//DNAJB6//PPARGC1B//ELK3//EREG//ATF5//FOXF2//FOXP1//MDFIC//NRG1//IRF1//RASD1//PURA//ATXN1//BTAF1//RPS6KA5//CLOCK//PPM1F//TOB1//CELF1//FMR1//NANOS1//TIA1//TSC1//FXR1//MEX3B//EGLN1//ATAD2//MIER1//DCP2//CNOT6//TNRC6B//ERRFI1//NMI//INSIG2//MYOG//PAIP2//PRKAA1//RAPGEF2//INHBB//NUP54//CNOT6L//YTHDF2//QKI//PHF8//CPEB2//ZMYND11//HSBP1L1//NPAT//SRSF2//ZMYND8//MYCN//NCK1//TMBIM6//DUSP1//GJA1//KCNK2//BMPR2//CPEB1// |
| GO:0010558 | negative regulation of macromolecule biosynthetic process | Biological process | 136 | 1709 | 902 | 17653 | 1.55743105172953 | 8.49619451245062e-08 | 6.24562646605473e-06 | 7.07077555338161 | 0.150776053215078 | TSHZ1//ZBTB18//CEBPB//TCERG1//STRAP//KLF12//GSC//ARX//DNMT3B//EDN1//EDNRB//EFNA1//CC2D1B//EN1//EP300//ESR1//JAZF1//CPEB3//FOXF1//MYT1L//SIN3B//SIRT1//ZFPM2//DNAJB5//GLI3//CNOT7//HIC1//FOXA1//HSBP1//ID2//IFNG//IRF2//AR//ISL1//JARID2//JUN//MAF//MDM4//MEF2A//MYB//NFIA//NFIB//NFIC//NR4A2//PAX5//KLF3//POU4F1//PPARG//ZBTB4//RB1//RBBP8//CCND1//RNF2//SARS//PRDM16//SMARCA2//SUV39H1//ZEB1//TFAP2A//TGIF1//KLF10//TNF//UBE2I//WT1//ZNF217//LRP8//BHLHE41//TBL1XR1//E2F8//ARID5B//BHLHE40//TP63//RUNX3//NOG//FOXP2//PHF14//BAHD1//H2AFZ//AGO1//SNIP1//FLNA//DNAJB6//PPARGC1B//ELK3//EREG//ATF5//FOXF2//FOXP1//MDFIC//NRG1//IRF1//RASD1//PURA//ATXN1//BTAF1//RPS6KA5//CLOCK//PPM1F//TOB1//CELF1//FMR1//NANOS1//TIA1//TSC1//FXR1//MEX3B//ATAD2//MIER1//DCP2//FBXW7//CNOT6//TNRC6B//ERRFI1//NMI//TMEFF2//PAIP2//INHBB//NUP54//CNOT6L//YTHDF2//QKI//PHF8//CPEB2//ZMYND11//HSBP1L1//NPAT//SRSF2//ZMYND8//MYCN//NCK1//TMBIM6//DUSP1//GJA1//KCNK2//BMPR2//CPEB1// |
| GO:0045934 | negative regulation of nucleobase-containing compound metabolic process | Biological process | 120 | 1457 | 902 | 17653 | 1.61188360495323 | 8.91560492844389e-08 | 6.48346625065226e-06 | 7.04984918438182 | 0.133037694013304 | TSHZ1//ZBTB18//CEBPB//TCERG1//STRAP//KLF12//GSC//ARX//DNMT3B//EDN1//EDNRB//EFNA1//CC2D1B//EN1//EP300//ESR1//JAZF1//CPEB3//FOXF1//MYT1L//SIN3B//SIRT1//ZFPM2//DNAJB5//GLI3//CNOT7//HIC1//FOXA1//HSBP1//ID2//IFNG//IRF2//AR//ISL1//JARID2//JUN//MAF//MDM4//MEF2A//MYB//NFIA//NFIB//NFIC//NR4A2//PAX5//KLF3//POU4F1//PPARG//ZBTB4//RB1//RBBP8//CCND1//RNF2//SARS//PRDM16//SMARCA2//SUV39H1//ZEB1//TFAP2A//TGIF1//KLF10//TNF//UBE2I//WT1//ZNF217//LRP8//BHLHE41//TBL1XR1//E2F8//ARID5B//BHLHE40//TP63//RUNX3//NOG//FOXP2//PHF14//BAHD1//H2AFZ//GRM3//FLNA//DNAJB6//PPARGC1B//ELK3//EREG//ATF5//FOXF2//FOXP1//MDFIC//NRG1//IRF1//RASD1//PURA//ATXN1//BTAF1//RPS6KA5//CLOCK//PPM1F//EGLN1//ATAD2//MIER1//DCP2//FBXW7//MYOG//SRSF6//TOB1//TMBIM6//PHF8//BOLL//OGG1//FMR1//ZMYND11//HSBP1L1//NPAT//SRSF2//ZMYND8//NCK1//DUSP1//GJA1//KCNK2//BMPR2// |
| GO:0051253 | negative regulation of RNA metabolic process | Biological process | 110 | 1306 | 902 | 17653 | 1.64839577185971 | 1.06295186322008e-07 | 7.64759941591213e-06 | 6.97348640246753 | 0.121951219512195 | TSHZ1//ZBTB18//CEBPB//TCERG1//STRAP//KLF12//GSC//ARX//DNMT3B//EDN1//EDNRB//EFNA1//CC2D1B//EN1//EP300//ESR1//JAZF1//CPEB3//FOXF1//MYT1L//SIN3B//SIRT1//ZFPM2//DNAJB5//GLI3//CNOT7//HIC1//FOXA1//HSBP1//ID2//IFNG//IRF2//AR//ISL1//JARID2//JUN//MAF//MDM4//MEF2A//MYB//NFIA//NFIB//NFIC//NR4A2//PAX5//KLF3//POU4F1//PPARG//ZBTB4//RB1//RBBP8//CCND1//RNF2//SARS//PRDM16//SMARCA2//SUV39H1//ZEB1//TFAP2A//TGIF1//KLF10//TNF//UBE2I//WT1//ZNF217//LRP8//BHLHE41//TBL1XR1//E2F8//ARID5B//BHLHE40//TP63//RUNX3//NOG//FOXP2//PHF14//BAHD1//H2AFZ//FLNA//DNAJB6//PPARGC1B//ELK3//EREG//ATF5//FOXF2//FOXP1//MDFIC//NRG1//IRF1//RASD1//PURA//ATXN1//BTAF1//RPS6KA5//CLOCK//PPM1F//ATAD2//MIER1//SRSF6//TOB1//TMBIM6//PHF8//BOLL//FMR1//ZMYND11//HSBP1L1//NPAT//SRSF2//ZMYND8//NCK1// |
| GO:0051239 | regulation of multicellular organismal process | Biological process | 211 | 2969 | 902 | 17653 | 1.39086263899168 | 1.24536317110847e-07 | 8.86567486969114e-06 | 6.90470398173399 | 0.233924611973392 | RPS6KA5//EREG//TNF//ADRB1//CALM2//GLI3//FZD3//F3//TGFBR2//CD3E//EDN2//EDN1//EDNRB//GJA1//WT1//BMPR2//ISL1//CHRM2//CHRNB4//TNNI1//HS3ST5//NPNT//TXK//SULF1//RORA//C5AR1//ETS1//FOXP1//KDR//RHOB//WNT7A//SLIT2//IGF1//MEF2A//PDE5A//JARID2//STRAP//PFN2//PTPRG//IFNG//JUN//PPM1F//BAMBI//EFNA1//FOXA1//PPP2CA//PTEN//LDLRAD4//SNAP25//STMN2//ADCYAP1//NPTN//NCK1//SERPINI1//RAP1A//SCN1B//RAPGEF2//LPAR1//EFNB2//MYLIP//SPOCK1//TSC1//BAG5//LDLR//MYOG//DLL1//KCNJ2//SPRED1//AGO1//PPARG//FOXJ2//SARS//INHBB//LRP6//MAPK1//ACVR1//DHRS3//SMURF1//S1PR1//TFAP2A//DPYSL2//RAB21//CCND1//TNFRSF21//WASF3//NRG1//ARRDC3//MAF//PTHLH//GDF5//SOX5//TBK1//PCBP2//MAVS//TNFAIP3//POLR3H//EP300//IRF1//ERRFI1//NAV3//SOCS5//IL2//CEBPB//CD2AP//ZEB1//PIK3CA//POU3F2//OTX2//TIA1//WNT10A//CLOCK//SIRT1//NR4A2//NMI//HMGB3//ID2//CBFB//SOCS1//BTG1//MYCN//SERPINB13//SRSF6//TP63//MYB//KLF13//RB1//TNRC6B//NFE2//NTF3//YWHAG//NREP//DDX6//DNMT3B//ETV5//MMD//NEUROD1//NEUROG1//SMOC1//RASSF2//TOB1//TMEM64//NOG//TFE3//GPR55//PPARGC1B//POU4F1//KLF10//TNFRSF12A//EGLN1//VASH2//ITGB8//VEGFB//ADAM12//ADNP//ARX//SERP1//PRKAA1//ULK2//WNT3//WDR1//FMR1//HECW2//FXR1//PLXNA4//SEMA4C//TRAK2//SYNGAP1//ROBO2//DBN1//CAMSAP2//RAB8B//SLITRK3//DLG5//KCNK2//ZFPM2//PRICKLE2//ROR1//PSMA2//PSMD12//FZD6//SGK1//FOXP2//AR//ESR1//BTBD7//CPEB3//ZMYND8//MAPK6//ARC//LRP8//TAPT1//FRZB//KCNE1//KCNIP4//GJC1//KCNJ3//MEOX2//C5ORF30//PRKACB//YTHDF2//NLN//TRPC3//PPP1R16B//SYT1//PLAA//FLNA//FBXW7//NFIB//PHF14//CRHR1//MET// |
| GO:0008283 | cell proliferation | Biological process | 158 | 2078 | 902 | 17653 | 1.48807056930487 | 1.26084053739289e-07 | 8.88235891082095e-06 | 6.8993398365907 | 0.175166297117517 | PIK3CB//BMPR2//SULF1//GJA1//TNF//F3//SIRT1//RICTOR//PPP1R16B//JUN//KDR//VEGFB//VASH2//GLI3//FZD3//FOXF1//MYCN//TGFBR2//TP63//FOXP2//ISL1//DNAJA2//ADCYAP1//CNTFR//CRKL//CACUL1//EDN1//EDN2//EDNRB//EFNB2//EREG//ETS1//CNOT6L//BAMBI//DLL1//CNOT7//NRG1//HOXA3//IFNG//IGF1//IL2//AR//NTF3//POU3F2//PRKAA1//MAPK1//SPHK2//PTEN//PTHLH//CNOT6//PURA//SOX4//TTK//FOSL1//CCND2//TOB1//COPS8//ARID2//ATF5//FRZB//TES//IGFBP3//IRF1//MDM4//MYOG//KLF13//RAF1//SMARCA2//STRN//ZEB1//BTG1//TFAP2A//KLF10//TSC1//WT1//CUL5//SKAP2//DLG5//RAPGEF2//ST18//SRSF6//C5AR1//ARX//EMX2//WNT7A//RORA//TNFRSF21//FZD6//CEBPB//ID2//CCND1//MSN//CR2//IFNA1//NCK1//CD3E//C8ORF44-SGK3//SGK2//CFDP1//EGLN3//EPS15//SGK3//NR5A2//CXCL9//IER5//SGK1//TXK//YES1//BAG6//PDE5A//LRP6//TBK1//HPRT1//IMPDH1//ESR1//MORC3//S1PR1//FOXP1//MYB//IRAK4//OGN//PPARG//TNFAIP3//NOG//STRAP//RB1//GDF5//RUNX3//SOX5//JARID2//KCNK2//ZFPM2//ETV5//DBN1//ERRFI1//CCL8//WNT3//FOXJ2//SHCBP1//FLNA//FBXW7//NFIB//PHF14//CMC4//TACC2//ZMYND11//TXLNA//EPS8//APPL1//ANXA7//IRF2//MET//USP28//GMNC//BHLHE41//E2F8//FAM83D//CDC14A// |
| GO:0051247 | positive regulation of protein metabolic process | Biological process | 131 | 1641 | 902 | 17653 | 1.56233692883713 | 1.28223391976188e-07 | 8.92489146714134e-06 | 6.89203273862795 | 0.145232815964523 | GADD45A//TNF//CRKL//TAOK1//RAF1//RAP1A//C1QTNF2//LPAR1//NRG1//IGF1//NTF3//PIK3CB//PRKAA1//MAPK1//C5AR1//UBE2V1//EDNRB//EFNA1//FMR1//SIRT1//NPTN//IFNG//KDR//MAVS//CCND1//PRR5L//CCND2//EREG//FBXW7//EGLN3//F3//PMAIP1//PPARG//CASP2//COPS8//MDFIC//SOCS1//CCL21//CALM2//INHBB//BMP3//BMPR2//TTK//GDF5//ACVR1//CPEB3//SERP1//PRR16//SOX4//FXR1//NDFIP2//RAP2C//RASSF2//ADNP//PIK3CA//PAK6//TGFBR2//SLK//RICTOR//WNT7A//ANKIB1//LONRF3//LONRF1//SOCS5//PPP1R15B//MID1//SEMA4C//RPS6KA5//CREBL2//TBK1//PFN2//IFNA1//RWDD3//ADCY3//PRKACB//PRKAR2B//ISL1//SPPL3//CNEP1R1//PPP1R16B//PPP1R12A//NCK1//IL2//SOCS3//PPM1F//EDN1//PDE5A//IGFBP3//AR//MET//ROR1//IRF1//CD3E//GJA1//MYLIP//SMURF1//HECW2//TNFAIP3//CCNY//CCNL1//CCNT2//ADCYAP1//CACUL1//MMD//RAPGEF2//BOLL//YES1//LRP8//SH3D19//DCUN1D4//ARRDC3//DNMT3B//MYB//JARID2//NPNT//JUN//PTEN//CCL2//CCL8//GPR55//ACSL1//PPP2CA//NMI//RAB3GAP1//MSN//YTHDF2//UBE2I//ESR1//BAG6//LDLR//ST18// |
| GO:0031401 | positive regulation of protein modification process | Biological process | 102 | 1188 | 902 | 17653 | 1.68033438598849 | 1.2932712757354e-07 | 8.92489146714134e-06 | 6.88831036823805 | 0.113082039911308 | GADD45A//TNF//CRKL//TAOK1//RAF1//RAP1A//C1QTNF2//LPAR1//NRG1//IGF1//NTF3//PIK3CB//PRKAA1//MAPK1//C5AR1//UBE2V1//EDNRB//EFNA1//FMR1//SIRT1//NPTN//IFNG//KDR//MAVS//CCND1//PRR5L//CCND2//EREG//FBXW7//COPS8//MDFIC//SOCS1//CALM2//INHBB//BMP3//BMPR2//TTK//GDF5//ACVR1//NDFIP2//RAP2C//RASSF2//ADNP//PIK3CA//PAK6//TGFBR2//SLK//RICTOR//PPP1R15B//MID1//SEMA4C//RPS6KA5//CREBL2//TBK1//PFN2//IFNA1//RWDD3//ADCY3//PRKACB//PRKAR2B//ISL1//SPPL3//CNEP1R1//PPP1R16B//PPP1R12A//IL2//SOCS3//EDN1//PDE5A//IGFBP3//AR//MET//ROR1//CCNY//CCNL1//CCNT2//ADCYAP1//CACUL1//MMD//CCL21//RAPGEF2//WNT7A//YES1//LRP8//CD3E//DCUN1D4//ARRDC3//DNMT3B//MYB//JARID2//NPNT//JUN//PTEN//CCL2//CCL8//GPR55//ACSL1//PPP2CA//NMI//RAB3GAP1//UBE2I//SOX4// |
| GO:0001932 | regulation of protein phosphorylation | Biological process | 117 | 1421 | 902 | 17653 | 1.61140151450136 | 1.34387997596448e-07 | 9.18046492671493e-06 | 6.87163951707384 | 0.129711751662971 | GADD45A//GTF2H1//PTEN//CCNT2//DIRAS3//TNF//CRKL//TAOK1//RAF1//RAP1A//C1QTNF2//LPAR1//NRG1//IGF1//NTF3//PIK3CB//PRKAA1//MAPK1//C5AR1//UBE2V1//SPRED1//DUSP1//PPP2CA//IGFBP3//IL2//LRP6//PRR5L//PPP1R15B//SLIT2//EDNRB//EFNA1//FMR1//SIRT1//NPTN//IFNG//KDR//MAVS//CCND1//CCND2//EREG//FBXW7//ERRFI1//GPRC5A//SOCS5//GNAQ//NCK1//GSKIP//RB1//YWHAG//SOCS1//SOCS3//PPM1F//COPS8//MDFIC//CALM2//INHBB//BMP3//BMPR2//TTK//GDF5//ACVR1//MOB1B//JUN//RAP2C//RASSF2//ADNP//PIK3CA//PAK6//TGFBR2//SLK//RICTOR//MID1//SEMA4C//RPS6KA5//CREBL2//TBK1//PFN2//IFNA1//ADCY3//PRKACB//PRKAR2B//CNOT7//ISL1//EDN1//PDE5A//SYNGAP1//AR//MET//ROR1//HIPK3//TNFAIP3//CCNY//CCNL1//TSC1//ADCYAP1//CACUL1//MMD//CCL21//RAPGEF2//GRIK2//ZMYND11//WNT7A//YES1//LRP8//CD3E//STRAP//PMEPA1//LDLRAD4//NOG//FAM83D//RANBP9//NPNT//CCL2//CCL8//GPR55//PPP1R1B//ACSL1// |
| GO:0065009 | regulation of molecular function | Biological process | 234 | 3373 | 902 | 17653 | 1.35772401547965 | 1.45410777292264e-07 | 9.83413086827581e-06 | 6.83740340403334 | 0.259423503325942 | GADD45A//GTF2H1//PTEN//CCNT2//DIRAS3//TNF//CRKL//TAOK1//RAF1//RAP1A//C1QTNF2//LPAR1//NRG1//IGF1//NTF3//PIK3CB//PRKAA1//MAPK1//C5AR1//UBE2V1//SPRED1//DUSP1//PPP2CA//EREG//FBXW7//ERRFI1//GPRC5A//SOCS5//GNAQ//LRP6//NCK1//GSKIP//RB1//YWHAG//SOCS1//SOCS3//PPM1F//EGLN3//F3//PMAIP1//PPARG//CASP2//ADCY3//ADCYAP1//CRHR1//ADRB1//EDNRB//GRM3//NMUR1//TXK//GPR55//COPS8//G3BP2//MDFIC//PI15//EDN1//EDN2//TXLNA//IFNA1//IFNG//IL2//INHBB//CXCL9//OGN//PTHLH//CCL2//CCL8//CCL21//BMP3//VEGFB//WNT3//WNT7A//WNT10A//GDF5//PPARGC1B//NPNT//ESR1//MTMR9//FKBP15//PPP1R12A//PLEKHF2//SERPINI1//SERPINB13//SPOCK1//RECK//IQGAP2//CPEB2//TSC1//SLIT2//GJA1//SIRT1//RWDD3//TNFAIP3//ADNP//TMBIM6//SPPL3//EP300//BAMBI//RAPGEF2//PIK3CA//PAK6//TGFBR2//SLK//RICTOR//STIM2//NDFIP2//C8ORF44-SGK3//SGK2//SGK3//SGK1//PLAA//ARPP19//PPP1R1B//CALM2//PPP1R15B//CNN3//PXK//DNAJB6//DNAJA2//PFN2//LRP8//RPS6KA5//RALBP1//RAB3GAP1//ARHGAP1//SBF2//SYDE2//PLXNA4//ARAP2//DENND1B//S1PR1//ELMOD2//AGFG1//JUN//ARHGAP6//RIN2//DOCK10//RASA1//BNIP2//SYNGAP1//ARHGAP12//GIT2//KDR//MET//ROR1//PRKACB//PRKAR2B//BTAF1//TRIM23//NR4A2//ARL6IP1//TP63//ISL1//ID2//PDE5A//FLNA//EGLN1//RNF2//FZD6//BHLHE40//TMEM64//HIPK3//NRBF2//RCAN3//UBXN2B//PPP4R2//PPP1R16B//IGFBP3//PPP2R5E//CCNY//CCNL1//CCND1//CCND2//CACUL1//MMD//RASSF2//AR//ABHD5//POU4F1//FOXA1//NEUROD1//NEUROG1//NHLH2//CYTL1//MAVS//FOSL1//ARID5B//RHEBL1//IRAK4//CLOCK//MID1IP1//ZER1//BAG5//DCUN1D4//ARRDC3//PTGES3//NOG//RAP2C//EFNA1//ACSL1//TBC1D12//CAST//LYPD6//FMR1//KCNE1//UBE2I//MTRR//SPTSSB//PPP2CB//CNOT6//DYNLL2//SCN1B//HECW2//GLRX//ARC//HOMER1//ST18//RANBP9//RAPGEF4//PLEKHG4B//DENND6A//RASGEF1A//TRAPPC8//IQSEC2//RGL1//FBXO8//ARHGEF4//HPCAL4//PSD//ITSN1//SOS2//KCNIP4//KCNAB2//RALGPS1// |
| GO:0008219 | cell death | Biological process | 166 | 2216 | 902 | 17653 | 1.46605517622291 | 1.50855400259852e-07 | 1.01013373461127e-05 | 6.82143913851467 | 0.184035476718404 | TNFRSF21//JUN//PMAIP1//BCL2A1//PTEN//FOXP1//IL2//HIPK3//RTN3//EGLN3//PRUNE2//GADD45A//EP300//UNC5B//ARL6IP1//SULF1//GJA1//NSG1//SLC25A6//IFNG//IGFBP3//IRF1//C6ORF120//RHOB//MEF2A//GULP1//CHMP3//ZFAND6//PPP2CA//DRAM1//MAPK1//PAK6//RAF1//SGK1//BNIP2//TGFBR2//TIA1//TNFAIP3//C5AR1//BAG6//FXR1//CASP2//TP63//EI24//SLK//F3//PPARG//TNF//DUSP1//EDNRB//ATF5//FLNA//SIRT1//KLHL20//GLI3//IGF1//KDR//MDM4//MET//ROR1//OGG1//PRKAA1//SPHK2//RASA1//TMBIM6//TFAP2A//WNT7A//WT1//CCND2//SOCS3//HIC1//GSKIP//PPP2CB//ADCYAP1//KCNK2//MYCN//C8ORF44-SGK3//SGK2//ESR1//SGK3//INHBB//NTF3//TAOK1//RBM25//BMP3//ANP32A//GDF5//CD3E//DLG5//PIK3CB//RB1//DLL1//CCL2//EDN1//LRP6//PHLDA3//USP28//LPAR1//CDK19//FRZB//FOXA1//USP27X//NEUROD1//ARHGEF4//TNFRSF12A//POU4F1//ZMAT3//ITSN1//SOS2//SOX4//FOSL1//SLIT2//RASSF2//DNAJB6//PIK3CA//PPM1F//CEBPB//CNTFR//EN1//ADNP//GABRA5//GRIK2//ISL1//NR4A2//SYNGAP1//MYB//ZFPM2//NCK1//NPAS2//SRSF6//TEAD2//VPS4B//ROBO2//EFNA1//KRT80//XKR8//MEOX2//BAG5//BMPR2//FASTK//SIVA1//APPL1//CUL5//FZD3//DYNLL2//PPP3R1//YWHAG//TBK1//EGLN1//TSC1//EFNB2//SERPINB13//SERINC3//FBXW7//IER3IP1//CFDP1//BTG1//CCL21//RAPGEF2//KCNB1//CAST//NOG//ZMYND11//AR//ACVR1//ST18// |
| GO:0032989 | cellular component morphogenesis | Biological process | 96 | 1101 | 902 | 17653 | 1.70645915525293 | 1.54001280545034e-07 | 1.02108888267261e-05 | 6.81247566792113 | 0.106430155210643 | CDH20//NRG1//SS18//WNT3//AGFG1//CLIC4//MET//PALLD//AR//FRMD6//SLITRK3//OGN//POU4F1//PRELP//WNT7A//SLIT2//RANBP9//ARX//DPYSL2//EFNA1//EFNB2//GLI3//OTX2//PIK3CA//PIK3CB//ENAH//MAPK1//ROBO2//SCN1B//KLF7//RPS6KA5//NRXN3//FEZ2//NOG//CFDP1//WASF3//PALM2//LPAR1//EPS8//BAMBI//ANXA7//KDR//RHOB//RND3//MSN//BRWD1//RASA1//CCL2//PLXNA4//WDR1//FLNA//NR4A2//PTEN//NFIB//SH3D19//CAMSAP1//ARC//EP300//TMOD3//TMOD1//RAB21//ISL1//UNC5B//VAMP3//FZD3//CFL2//ADNP//TNFRSF12A//ID2//RB1//ULK2//SLC9A6//GJA1//GPM6A//NTF3//SGK1//HPRT1//MEF2A//TRAK2//FMR1//HECW2//FXR1//BMPR2//SEMA4C//POU3F2//SYNGAP1//RAPGEF2//EDN1//DOCK10//LRP8//SNX2//YTHDF2//CRKL//SMURF1//SYT1//PLAA// |
| GO:0010941 | regulation of cell death | Biological process | 134 | 1696 | 902 | 17653 | 1.54628995314396 | 1.63064263547489e-07 | 1.07068312074919e-05 | 6.78764120661557 | 0.148558758314856 | PTEN//FOXP1//IL2//EGLN3//F3//PMAIP1//PPARG//TNF//CASP2//HIPK3//DUSP1//EDNRB//ATF5//FLNA//ARL6IP1//SIRT1//KLHL20//GLI3//IGF1//KDR//MDM4//MET//ROR1//OGG1//ZFAND6//PRKAA1//SPHK2//RAF1//RASA1//BCL2A1//BNIP2//TMBIM6//TFAP2A//WNT7A//WT1//BAG6//TP63//CCND2//SOCS3//ADCYAP1//KCNK2//MYCN//C8ORF44-SGK3//SGK2//ESR1//SGK3//TNFRSF21//IGFBP3//INHBB//NTF3//PAK6//TAOK1//RBM25//SGK1//BMP3//ANP32A//GDF5//CD3E//DLG5//SLK//PIK3CB//DLL1//CCL2//EDN1//LRP6//IFNG//GADD45A//LPAR1//CDK19//PHLDA3//FRZB//FOXA1//JUN//RHOB//USP27X//NEUROD1//ARHGEF4//TNFRSF12A//POU4F1//ZMAT3//ITSN1//SOS2//SOX4//FOSL1//SLIT2//RASSF2//DNAJB6//PPM1F//CEBPB//CNTFR//EN1//UNC5B//ADNP//GABRA5//GRIK2//ISL1//NR4A2//PIK3CA//C5AR1//SYNGAP1//MYB//ZFPM2//NCK1//NPAS2//SRSF6//TEAD2//VPS4B//EFNA1//FZD3//DYNLL2//PPP3R1//YWHAG//TBK1//EGLN1//TSC1//EFNB2//TNFAIP3//SERPINB13//BAG5//SERINC3//FBXW7//TIA1//IER3IP1//CFDP1//BTG1//CCL21//RAPGEF2//KCNB1//CAST//NOG//ZMYND11//AR//ACVR1//ST18// |
| GO:0006464 | cellular protein modification process | Biological process | 280 | 4192 | 902 | 17653 | 1.30722017230582 | 1.68544945701563e-07 | 1.08558996931397e-05 | 6.77328426650007 | 0.310421286031042 | GADD45A//GTF2H1//PTEN//CCNT2//DIRAS3//RANBP9//SPRED1//EREG//RASGEF1A//NRG1//IL2//MEF2A//MET//MAPK1//MAPK6//PSMA2//PSMD12//RAF1//RASA1//CCL2//TNF//SYNGAP1//RAPGEF2//CRKL//TAOK1//RAP1A//C1QTNF2//LPAR1//IGF1//NTF3//PIK3CB//PRKAA1//C5AR1//UBE2V1//DUSP1//PPP2CA//FBXO41//RNF145//ZNRF2//RNF167//MYLIP//ANKIB1//UBE2W//FBXW7//SMURF1//HECW2//KLHL42//SKP1//THOP1//LONRF3//FBXO30//CBFB//UBE3B//LONRF1//FBXO44//COPS8//NKTR//SOCS1//IGFBP3//LRP6//PRR5L//PPP1R15B//SLIT2//EDNRB//EFNA1//FMR1//SIRT1//NPTN//IFNG//KDR//MAVS//CCND1//CCND2//ABHD17C//ERRFI1//GPRC5A//SOCS5//ZER1//CACUL1//RNF38//MARCH10//PDZRN3//MYCBP2//FBXO33//KLHL3//RNF11//KLHL20//SPOPL//TRIM23//MDM4//DCAF8//CBLL1//CUL5//KBTBD8//SOCS3//C8ORF44-SGK3//SGK2//HIPK3//FASTK//PDIK1L//CDK19//MORC3//MMD//SGK3//PAN3//GRK6//TBK1//NRBP1//CDK17//PIK3CA//PRKACB//CCL8//SGK1//CDK15//TGFBR2//TXK//MEX3B//RUNX3//ACVR1//RPS6KA5//RASSF2//GNAQ//NCK1//GSKIP//RB1//YWHAG//PPM1F//PTPN21//PPP4R2//PPTC7//PPP1R12A//CTDSPL2//PPP2CB//PPP3R1//EP300//CLOCK//HS3ST5//PCMTD1//BTG1//FUT9//B3GALNT2//FUT2//FUT5//SERP1//ST8SIA5//GFPT1//KCNE1//CCDC126//ALG2//EOGT//GCNT4//TRAK2//VEGFB//WDR45B//ALPI//RECK//PYURF//PIGP//PIGA//IRAK4//MDFIC//CALM2//ADNP//INHBB//BMP3//BMPR2//TTK//GDF5//EPC2//SIN3B//ARID4B//MIER1//SAP30L//TBL1XR1//ARID5B//ATXN7//MBD6//ESR1//OTUD3//AR//USP27X//USP28//TNFAIP3//USP38//WDR20//NUP54//UBE2I//CEP41//RICTOR//STK33//ROR1//EGLN3//TGM3//ZDHHC22//ZDHHC7//ZDHHC18//ST3GAL5//MCFD2//GPHN//BAG6//CRTAP//EGLN1//DCUN1D4//KCNH4//PAK6//SLK//JARID2//SOX4//BAG5//NDFIP2//MOB1B//JUN//RAP2C//ARPP19//PPP1R1B//MID1//SEMA4C//CREBL2//PFN2//IFNA1//PHF8//RWDD3//ADCY3//PRKAR2B//ESCO2//PRDM16//ISL1//PPP1R16B//SPPL3//CNEP1R1//PTPRG//PTP4A1//PTP4A2//CDC14A//RNF2//SUV39H1//PCGF5//YES1//CNOT7//EDN1//PDE5A//RCAN3//UBXN2B//PPP2R5E//TSC1//CKAP4//CALU//CCNY//CCNL1//ADCYAP1//CCL21//GRIK2//ZMYND11//WNT7A//ULK2//LRP8//CD3E//CCM2//ARRDC3//DNMT3B//MYB//PAX5//STRAP//PMEPA1//LDLRAD4//NOG//ATG16L1//FAM83D//NPNT//GPR55//OTUD1//KDM2A//UBA5//ACSL1//GLRX//PLAA//NMI//RAB3GAP1//ZMYND8//RIMKLB// |
| GO:0036211 | protein modification process | Biological process | 280 | 4192 | 902 | 17653 | 1.30722017230582 | 1.68544945701563e-07 | 1.08558996931397e-05 | 6.77328426650007 | 0.310421286031042 | GADD45A//GTF2H1//PTEN//CCNT2//DIRAS3//RANBP9//SPRED1//EREG//RASGEF1A//NRG1//IL2//MEF2A//MET//MAPK1//MAPK6//PSMA2//PSMD12//RAF1//RASA1//CCL2//TNF//SYNGAP1//RAPGEF2//CRKL//TAOK1//RAP1A//C1QTNF2//LPAR1//IGF1//NTF3//PIK3CB//PRKAA1//C5AR1//UBE2V1//DUSP1//PPP2CA//FBXO41//RNF145//ZNRF2//RNF167//MYLIP//ANKIB1//UBE2W//FBXW7//SMURF1//HECW2//KLHL42//SKP1//THOP1//LONRF3//FBXO30//CBFB//UBE3B//LONRF1//FBXO44//COPS8//NKTR//SOCS1//IGFBP3//LRP6//PRR5L//PPP1R15B//SLIT2//EDNRB//EFNA1//FMR1//SIRT1//NPTN//IFNG//KDR//MAVS//CCND1//CCND2//ABHD17C//ERRFI1//GPRC5A//SOCS5//ZER1//CACUL1//RNF38//MARCH10//PDZRN3//MYCBP2//FBXO33//KLHL3//RNF11//KLHL20//SPOPL//TRIM23//MDM4//DCAF8//CBLL1//CUL5//KBTBD8//SOCS3//PPP4R2//SERP1//RIMKLB//TGM3//UBE2I//YES1//C8ORF44-SGK3//SGK2//HIPK3//FASTK//PDIK1L//CDK19//MORC3//MMD//SGK3//PAN3//GRK6//TBK1//NRBP1//CDK17//PIK3CA//PRKACB//CCL8//SGK1//CDK15//TGFBR2//TXK//MEX3B//RUNX3//ACVR1//RPS6KA5//RASSF2//GNAQ//NCK1//GSKIP//RB1//YWHAG//PPM1F//PTPN21//PPTC7//PPP1R12A//CTDSPL2//PPP2CB//PPP3R1//EP300//CLOCK//HS3ST5//PCMTD1//BTG1//FUT9//B3GALNT2//FUT2//FUT5//ST8SIA5//GFPT1//KCNE1//CCDC126//ALG2//EOGT//GCNT4//TRAK2//VEGFB//WDR45B//ALPI//RECK//PYURF//PIGP//PIGA//IRAK4//MDFIC//CALM2//ADNP//INHBB//BMP3//BMPR2//TTK//GDF5//EPC2//SIN3B//ARID4B//MIER1//SAP30L//TBL1XR1//ARID5B//ATXN7//MBD6//ESR1//OTUD3//AR//USP27X//USP28//TNFAIP3//USP38//WDR20//NUP54//CEP41//RICTOR//STK33//ROR1//EGLN3//ZDHHC22//ZDHHC7//ZDHHC18//ST3GAL5//MCFD2//GPHN//BAG6//CRTAP//EGLN1//DCUN1D4//KCNH4//PAK6//SLK//JARID2//SOX4//BAG5//NDFIP2//MOB1B//JUN//RAP2C//ARPP19//PPP1R1B//MID1//SEMA4C//CREBL2//PFN2//IFNA1//PHF8//RWDD3//ADCY3//PRKAR2B//ESCO2//PRDM16//ISL1//PPP1R16B//SPPL3//CNEP1R1//PTPRG//PTP4A1//PTP4A2//CDC14A//RNF2//SUV39H1//PCGF5//CNOT7//EDN1//PDE5A//RCAN3//UBXN2B//PPP2R5E//TSC1//CKAP4//CALU//CCNY//CCNL1//ADCYAP1//CCL21//GRIK2//ZMYND11//WNT7A//ULK2//LRP8//CD3E//CCM2//ARRDC3//DNMT3B//MYB//PAX5//STRAP//PMEPA1//LDLRAD4//NOG//ATG16L1//FAM83D//NPNT//GPR55//OTUD1//KDM2A//UBA5//ACSL1//GLRX//PLAA//NMI//RAB3GAP1//ZMYND8// |
| GO:0044260 | cellular macromolecule metabolic process | Biological process | 512 | 8555 | 902 | 17653 | 1.17128324484456 | 1.79600453716578e-07 | 1.14588478158983e-05 | 6.74569257052864 | 0.567627494456763 | SIRT1//IGF1//NFIA//NFIB//NFIC//RBBP8//RBMS1//GADD45A//GTF2H1//PTEN//CCNT2//DIRAS3//NPAT//TSHZ1//ZBTB18//CEBPB//TCERG1//STRAP//KLF12//GSC//ARX//DNMT3B//EDN1//EDNRB//EFNA1//CC2D1B//EN1//EP300//ESR1//JAZF1//CPEB3//FOXF1//MYT1L//SIN3B//ZFPM2//DNAJB5//GLI3//CNOT7//HIC1//FOXA1//HSBP1//ID2//IFNG//IRF2//AR//ISL1//JARID2//JUN//MAF//MDM4//MEF2A//MYB//NR4A2//PAX5//KLF3//POU4F1//PPARG//ZBTB4//RB1//CCND1//RNF2//SARS//PRDM16//SMARCA2//SUV39H1//ZEB1//TFAP2A//TGIF1//KLF10//TNF//UBE2I//WT1//ZNF217//LRP8//BHLHE41//TBL1XR1//E2F8//ARID5B//BHLHE40//TP63//RUNX3//NOG//FOXP2//PHF14//RANBP9//SPRED1//EREG//RASGEF1A//NRG1//IL2//MET//MAPK1//MAPK6//PSMA2//PSMD12//RAF1//RASA1//CCL2//SYNGAP1//RAPGEF2//PNRC1//DCP2//PPP2CA//CRKL//TAOK1//RAP1A//C1QTNF2//LPAR1//NTF3//PIK3CB//PRKAA1//C5AR1//UBE2V1//DUSP1//FBXO41//RNF145//ZNRF2//RNF167//MYLIP//ANKIB1//UBE2W//FBXW7//SMURF1//HECW2//KLHL42//SKP1//THOP1//LONRF3//FBXO30//CBFB//UBE3B//LONRF1//FBXO44//CNOT6L//PAN3//CNOT6//PATL1//COPS8//NKTR//PTGES3//NABP1//AGO1//GTF2E1//SEPSECS//SECISBP2L//NSG1//EPS15//VAMP3//SOCS1//IGFBP3//LRP6//PRR5L//PPP1R15B//SLIT2//FMR1//NPTN//KDR//MAVS//CCND2//ABHD17C//CREBRF//ELK3//ETV5//ATF5//FOXF2//FLI1//GTF2A1//HIVEP2//IRF1//MEOX2//MYBL1//MYCN//NEUROD1//NHLH2//OTX2//KLF13//PKNOX1//POU3F2//FOXJ2//HIVEP3//BMPR2//SOX4//SOX5//BTF3//TFE3//TXK//FOSL1//KLF7//FUBP3//NMI//CLOCK//ZNF516//POLR3H//SRSF2//SRSF6//SLBP//ERRFI1//GPRC5A//SOCS5//ZER1//CACUL1//RNF38//MARCH10//PDZRN3//MYCBP2//FBXO33//KLHL3//RNF11//KLHL20//SPOPL//TRIM23//DCAF8//CBLL1//CUL5//KBTBD8//SOCS3//PURA//GMNC//ESCO2//EPC2//ASTE1//USP28//SMUG1//OGG1//KDM2A//MTRR//ASCC1//HMGB3//TSN//BAHD1//H2AFZ//ZMYND11//ZNF526//CREBL2//ZNF800//E2F5//ZNF367//ARID2//MED19//ZFP30//PHF8//FLII//ADNP//ZMYND8//ZNF521//HBP1//FOXP1//ZBTB11//ATAD2//MDFIC//HOXA3//HOXD1//ZNF680//ZNF662//NEUROG1//NFE2//NPAS2//IER5//COMMD10//ARID4B//BRWD1//BNC2//MED9//ZNF532//TRERF1//SLC2A4RG//CCNL1//PHTF2//ZBTB26//MIER1//ATXN1//ATXN7//SMARCD2//SS18//MED22//ZNF3//ZNF708//ZNF131//ZNF227//ZNF655//ZSCAN5A//SAP30L//ANP32A//PPP1R1B//PCGF5//LCOR//ST18//HIPK3//PPARGC1B//ELAVL2//ELL2//NR5A2//NRBF2//AFF1//MLLT6//PHF20L1//PAK6//RORA//BTG1//CSDE1//SNIP1//TEAD2//RPS6KA5//EMX2//ETS1//BOLA3//NR3C2//RFX7//TRAK2//BDP1//NRBP1//CPEB2//CPEB1//DDX3Y//GTPBP1//DDX6//TSC1//PPP4R2//SERP1//RIMKLB//TGM3//YES1//SPPL3//IMMP2L//C8ORF44-SGK3//SGK2//FASTK//PDIK1L//CDK19//MORC3//MMD//SGK3//GRK6//TBK1//CDK17//PIK3CA//PRKACB//CCL8//SGK1//CDK15//TGFBR2//MEX3B//ACVR1//RASSF2//GNAQ//NCK1//GSKIP//YWHAG//PPM1F//PTPN21//PPTC7//PPP1R12A//CTDSPL2//PPP2CB//PPP3R1//HS3ST5//PCMTD1//FUT9//B3GALNT2//FUT2//FUT5//ST8SIA5//GFPT1//KCNE1//CCDC126//ALG2//EOGT//GCNT4//VEGFB//WDR45B//ALPI//RECK//PYURF//PIGP//PIGA//CTSF//FBXO8//USP27X//BAG6//USP38//GPHN//EGLN3//F3//PMAIP1//CASP2//DLL1//G3BP2//IRAK4//PI15//CCL21//CALM2//INHBB//BMP3//TTK//GDF5//SERPINI1//SERPINB13//SPOCK1//PRR16//FXR1//GNPTG//FLNA//BAMBI//WNT7A//DNAJB6//RASD1//BTAF1//MBD6//OTUD3//TNFAIP3//WDR20//NUP54//TOB1//CELF1//NANOS1//TIA1//CEP41//RICTOR//STK33//ROR1//ZDHHC22//ZDHHC7//ZDHHC18//ST3GAL5//MCFD2//CRTAP//EGLN1//DCUN1D4//KCNH4//SLK//SULF1//DSEL//SLC35D1//DNAJB9//JKAMP//LEMD3//ANAPC15//BAG5//NDFIP2//MOB1B//RAP2C//RWDD3//CCT6A//ARPP19//MID1//SEMA4C//INSIG2//PFN2//IFNA1//ADCY3//PRKAR2B//TNRC6B//PPP1R16B//CNEP1R1//PTPRG//PTP4A1//PTP4A2//CDC14A//MYOG//INTS8//TDRD5//ARL6IP1//UBXN2B//CD2AP//ARMC8//PCBP2//PLAA//VPS37A//VPS4B//PDE5A//FZD6//YTHDF2//RCAN3//PPP2R5E//CKAP4//CALU//RAB12//CCNY//ADCYAP1//S1PR1//KPNA6//ARMCX3//CYTL1//PAIP2//BOLL//GRIK2//ULK2//CD3E//SH3D19//RHEBL1//CCM2//ARRDC3//PMEPA1//LDLRAD4//QKI//ATG16L1//FAM83D//NPNT//GPR55//OTUD1//UBA5//ACSL1//GLRX//CAST//SNAP25//RAB3GAP1//MSN//FAM168A//LDLR//TMBIM6//GJA1//KCNK2// |
| GO:0042327 | positive regulation of phosphorylation | Biological process | 91 | 1032 | 902 | 17653 | 1.72573329723783 | 2.02352121574949e-07 | 1.27738734716153e-05 | 6.69389223785772 | 0.100886917960089 | GADD45A//TNF//CRKL//TAOK1//RAF1//RAP1A//C1QTNF2//LPAR1//NRG1//IGF1//NTF3//PIK3CB//PRKAA1//MAPK1//C5AR1//UBE2V1//EDNRB//EFNA1//FMR1//SIRT1//NPTN//IFNG//KDR//MAVS//CCND1//PRR5L//CCND2//EREG//FBXW7//COPS8//MDFIC//SOCS1//CALM2//INHBB//BMP3//BMPR2//TTK//GDF5//ACVR1//RAP2C//RASSF2//ADNP//PIK3CA//PAK6//TGFBR2//SLK//RICTOR//MID1//SEMA4C//RPS6KA5//CREBL2//TBK1//PFN2//IFNA1//MET//ROR1//ADCY3//PRKACB//PRKAR2B//IL2//ISL1//SOCS3//EDN1//PDE5A//IGFBP3//AR//CCL21//CCNY//CCNL1//CCNT2//ADCYAP1//CACUL1//MMD//RAPGEF2//WNT7A//YES1//LRP8//CD3E//NPNT//JUN//PTEN//CCL2//CCL8//GPR55//ACSL1//PPP2CA//MYOG//PTGES3//PPARGC1B//ITSN1//MOB1B// |
| GO:0061061 | muscle structure development | Biological process | 64 | 640 | 902 | 17653 | 1.95709534368071 | 2.03989107634845e-07 | 1.27738734716153e-05 | 6.69039302188967 | 0.0709534368070953 | ZFPM2//NRG1//POU4F1//NOG//S1PR1//EVC//SIRT1//IGF1//MEF2A//FXR1//SGCE//ZBTB18//CFL2//EP300//MEOX2//MYOG//GPCPD1//CCNT2//FOXP2//ADAM12//BHLHE41//TMOD3//TMOD1//EDNRB//PAX5//RB1//HIVEP3//SEMA4C//RORA//QKI//WDR1//MBNL1//DLL1//TNF//IGFBP3//CXCL9//BTG1//GJA1//SDC1//NEUROG1//PRKAA1//GSC//SERP1//ARID5B//HOMER1//FOXF1//ACVR1//BNIP2//ZEB1//EREG//TSC1//EGLN1//ISL1//TNNI1//JARID2//KCNK2//PTEN//TGFBR2//EDN1//CNTFR//WT1//NLN//NPNT//EFNB2// |
| GO:0009892 | negative regulation of metabolic process | Biological process | 216 | 3075 | 902 | 17653 | 1.37474014385377 | 2.08172901665306e-07 | 1.29162691189217e-05 | 6.68157580423552 | 0.239467849223947 | TSHZ1//ZBTB18//CEBPB//TCERG1//STRAP//KLF12//GSC//ARX//DNMT3B//EDN1//EDNRB//EFNA1//CC2D1B//EN1//EP300//ESR1//JAZF1//CPEB3//FOXF1//MYT1L//SIN3B//SIRT1//ZFPM2//DNAJB5//GLI3//CNOT7//HIC1//FOXA1//HSBP1//ID2//IFNG//IRF2//AR//ISL1//JARID2//JUN//MAF//MDM4//MEF2A//MYB//NFIA//NFIB//NFIC//NR4A2//PAX5//KLF3//POU4F1//PPARG//ZBTB4//RB1//RBBP8//CCND1//RNF2//SARS//PRDM16//SMARCA2//SUV39H1//ZEB1//TFAP2A//TGIF1//KLF10//TNF//UBE2I//WT1//ZNF217//LRP8//BHLHE41//TBL1XR1//E2F8//ARID5B//BHLHE40//TP63//RUNX3//NOG//FOXP2//PHF14//PNRC1//DCP2//PPP2CA//SPRED1//DUSP1//CNOT6L//PAN3//CNOT6//PATL1//AGO1//IGFBP3//IL2//LRP6//PTEN//PRR5L//PPP1R15B//SLIT2//ERRFI1//GPRC5A//SOCS5//BAHD1//H2AFZ//GADD45A//GNAQ//NCK1//GSKIP//YWHAG//SOCS1//SOCS3//PPM1F//GRM3//SNIP1//PI15//MET//MTMR9//ADNP//GJA1//TBK1//ANXA7//ITGB8//KDR//LDLR//MYCN//POU3F2//NDFIP2//CD3E//CALM2//FBXW7//INSIG2//FKBP15//PPP1R12A//PLEKHF2//SERPINI1//SERPINB13//SPOCK1//RECK//PIK3CA//TSC1//CELF1//FLNA//DNAJB6//PPARGC1B//ELK3//EREG//ATF5//FOXF2//FOXP1//MDFIC//NRG1//IRF1//RASD1//PURA//ATXN1//BTAF1//RPS6KA5//CLOCK//TOB1//FMR1//NANOS1//TIA1//FXR1//MEX3B//TSN//EGLN1//TNRC6B//SPOPL//SOX4//TNFAIP3//BAG5//ATAD2//MIER1//BAG6//ARPP19//PPP1R1B//RAP1A//RASSF2//PPP1R16B//ATXN7//ARL6IP1//RAF1//YTHDF2//PSMA2//PSMD12//ANP32A//HIPK3//DDX6//NMI//TMEFF2//MYOG//PAIP2//PRKAA1//ZMYND11//RAPGEF2//SRSF6//INHBB//NTF3//PMEPA1//LDLRAD4//TMBIM6//NUP54//GTPBP1//QKI//PHF8//RANBP9//BOLL//CSDE1//CAST//PLAA//CPEB2//OGG1//CRTAP//FEZ2//HSBP1L1//NPAT//SRSF2//ZMYND8//KCNK2//BMPR2//PRKAR2B//CPEB1// |
| GO:0042325 | regulation of phosphorylation | Biological process | 124 | 1544 | 902 | 17653 | 1.57176050917363 | 2.12267555919039e-07 | 1.2996049416827e-05 | 6.67311638059657 | 0.137472283813747 | GADD45A//GTF2H1//PTEN//CCNT2//DIRAS3//TNF//CRKL//TAOK1//RAF1//RAP1A//C1QTNF2//LPAR1//NRG1//IGF1//NTF3//PIK3CB//PRKAA1//MAPK1//C5AR1//UBE2V1//SPRED1//DUSP1//PPP2CA//SOCS1//IGFBP3//IL2//LRP6//PRR5L//PPP1R15B//SLIT2//EDNRB//EFNA1//FMR1//SIRT1//NPTN//IFNG//KDR//MAVS//CCND1//CCND2//EREG//FBXW7//ERRFI1//GPRC5A//SOCS5//NUP54//GNAQ//NCK1//GSKIP//RB1//YWHAG//SOCS3//PPM1F//COPS8//MDFIC//CALM2//INHBB//BMP3//BMPR2//TTK//GDF5//ACVR1//MOB1B//JUN//RAP2C//RASSF2//ADNP//PIK3CA//PAK6//TGFBR2//SLK//RICTOR//MID1//SEMA4C//RPS6KA5//CREBL2//TBK1//PFN2//IFNA1//MET//ROR1//ADCY3//PRKACB//PRKAR2B//ATXN7//PTGES3//PPARGC1B//AR//ITSN1//CNOT7//ISL1//EDN1//PDE5A//SYNGAP1//HIPK3//NRBF2//CCL21//TNFAIP3//CCNY//CCNL1//MYOG//TSC1//ADCYAP1//CACUL1//MMD//RAPGEF2//GRIK2//ZMYND11//WNT7A//YES1//LRP8//CD3E//STRAP//PMEPA1//LDLRAD4//NOG//FAM83D//RANBP9//NPNT//CCL2//CCL8//GPR55//PPP1R1B//ACSL1// |
| GO:0061448 | connective tissue development | Biological process | 33 | 240 | 902 | 17653 | 2.69100609756098 | 2.13302008763536e-07 | 1.2996049416827e-05 | 6.67100505457959 | 0.0365853658536585 | MYCN//WNT7A//SNX19//NFIB//CYTL1//GDF5//RUNX3//SULF1//BMPR2//TGFBR2//MAF//PTHLH//GLI3//SOX5//EDN1//TAPT1//EVC//HOXA3//ITGB8//BMP3//ZEB1//NOG//LRP6//ID2//PIK3CA//ARID5B//ZNF516//ARRDC3//TBL1XR1//FOXA1//FRZB//WT1//SIRT1// |
| GO:0009790 | embryo development | Biological process | 86 | 959 | 902 | 17653 | 1.75505943228927 | 2.18382870644953e-07 | 1.31868156622484e-05 | 6.66078142950266 | 0.0953436807095344 | WNT7A//RDH10//EDN1//TAPT1//FOXF1//ZFPM2//TANC2//GJA1//GLI3//AR//MBNL1//BTF3//TGFBR2//FOSL1//CCM2//ACVR1//NOG//LRP6//RNF2//DUSP1//BMPR2//WNT3//EP300//DLL1//MEOX2//RBBP8//SOX4//SEMA4C//PRKACB//TSC1//FZD3//FZD6//TEAD2//CEBPB//TFAP2A//CXXC4//NEUROG1//WT1//GSC//CELF1//RAI2//NR5A2//RICTOR//STOX2//EFNA1//KBTBD8//GRSF1//FOXA1//GDF5//TDRD5//EN1//LRIG1//RECK//TP63//KDR//HSBP1//PIK3CB//OTX2//INSIG2//MAPK1//TSHZ1//GNAQ//MYCN//ID2//NEUROD1//FOXF2//TNF//ARID2//ZEB1//YTHDF2//PAX5//HOXA3//SLC35D1//SULF1//HOXD1//ISL1//ACSL4//SOCS3//E2F8//DNAJB6//EGLN1//VASH2//FRZB//IGF1//NRG1//BAG6// |
| GO:0035556 | intracellular signal transduction | Biological process | 204 | 2871 | 902 | 17653 | 1.39062156081806 | 2.21745448204399e-07 | 1.32713669575783e-05 | 6.65414528622003 | 0.226164079822616 | KCNH4//RANBP9//SPRED1//EREG//RASGEF1A//NRG1//IL2//MEF2A//MET//MAPK1//MAPK6//PSMA2//PSMD12//RAF1//RASA1//CCL2//TNF//SYNGAP1//RAPGEF2//GADD45A//CRKL//TAOK1//RAP1A//C1QTNF2//LPAR1//IGF1//NTF3//PIK3CB//PRKAA1//C5AR1//UBE2V1//DUSP1//PPP2CA//EP300//CNOT6L//CNOT7//MDM4//CNOT6//SOX4//E2F8//CASP2//TP63//ADCY3//ADCYAP1//CRHR1//GNAQ//PTHLH//NMUR1//RCAN3//EDN1//EDN2//EDNRB//SAMD14//TBK1//SNIP1//COPS8//IRAK4//MDFIC//SOCS1//SOCS3//NMI//SOCS5//NEUROD1//RASD1//RALBP1//RAPGEF4//RAB18//RGL1//RND3//ARHGAP1//RIN2//DOCK10//ITSN1//SOS2//DIRAS3//RALGPS1//JUN//USP28//RB1//G3BP2//EPS8//RHOB//ARHGAP6//PAK6//HIC1//BCL2A1//GSKIP//AKAP11//CALM2//PIK3CA//PPP1R16B//PTEN//UNC5B//SIRT1//KDR//PRR5L//SLK//RHEBL1//RICTOR//FAM83D//TSC1//RRAGD//ARAP2//IQSEC2//APPL1//FBXO8//AGFG1//PSD//GIT2//RAB12//RAB21//RAB30//RAB9B//RAB8B//RAB28//RAP2C//RAP1B//MID1//SEMA4C//IFNA1//IFNG//PPP3R1//PLEKHG4B//ARHGEF4//GPR55//TEAD2//MOB1B//DLG5//SKP1//ISL1//PHLDA3//BAG6//ZFAND6//FLNA//GJA1//ATP2C1//ROR1//NDFIP2//MAVS//MIER1//CCL21//UBE2I//ZMYND11//ESR1//RORA//TNFAIP3//PDE5A//INHBB//BMP3//GDF5//EFNA1//IGFBP3//AR//HIPK3//PMAIP1//PEX5L//ADRB1//CXCL9//GRIK2//WNT7A//RASSF2//PPP2CB//CD3E//SYDE2//ARHGAP12//CD2AP//SLIT2//CCM2//F3//OTUD3//CEBPB//TMBIM6//ERRFI1//NPNT//NPTN//FBXW7//CCL8//FIBIN//SPPL3//POU4F1//ZMAT3//CUL5//PPM1F//NR5A2//AGO1//RBBP8//BAG5//SERINC3//NCK1//LEMD3//SESN1//C8ORF44-SGK3//SGK2//DGKH//SGK3//NRBP1//PRKAR2B//SGK1//STK33//SS18//ANP32A//PPP1R1B//DGKE//RPS6KA5// |
| GO:0051094 | positive regulation of developmental process | Biological process | 110 | 1329 | 902 | 17653 | 1.61986823028501 | 2.57717788922773e-07 | 1.52889947937256e-05 | 6.58885560331808 | 0.121951219512195 | GLI3//FZD3//TGFBR2//CD3E//BAMBI//ISL1//MYOG//STMN2//ADCYAP1//NPTN//NCK1//SERPINI1//RAP1A//SCN1B//RAPGEF2//BMPR2//TFAP2A//ACVR1//WASF3//NRG1//SOX5//GDF5//IFNG//CCND1//POU3F2//OTX2//EDN1//IL2//SOCS1//AR//JUN//NEUROD1//SOCS3//CEBPB//CREBL2//TMEM64//FRZB//ID2//PPARG//BTG1//MYB//SOCS5//ETS1//RB1//IGFBP3//CXCL9//DNMT3B//ETV5//MMD//FOXA1//NEUROG1//PTEN//ZEB1//NPNT//GJA1//IGF1//TP63//PPARGC1B//POU4F1//KLF10//TNF//F3//SIRT1//ITGB8//KDR//RHOB//C5AR1//VEGFB//VASH2//ADAM12//ADNP//TNFRSF12A//ARX//SERP1//DLL1//PRKAA1//WNT3//ROBO2//PLXNA4//SLIT2//RAB21//MEF2A//BNIP2//SLITRK3//WNT7A//DLG5//ZFPM2//PDE5A//WT1//SRSF6//FOXP2//LPAR1//CPEB3//FMR1//ZMYND8//MAPK6//LRP8//TAPT1//MIEF1//NOG//PRDM16//CRKL//FLNA//PPP1R16B//EFNB2//SMURF1//SYT1//PLAA//LRP6//TNFAIP3// |
| GO:0034654 | nucleobase-containing compound biosynthetic process | Biological process | 295 | 4488 | 902 | 17653 | 1.28641516574378 | 3.0045610532927e-07 | 1.76694316551465e-05 | 6.52221896660873 | 0.327050997782705 | NPAT//TSHZ1//ZBTB18//CEBPB//TCERG1//STRAP//KLF12//GSC//ARX//DNMT3B//EDN1//EDNRB//EFNA1//CC2D1B//EN1//EP300//ESR1//JAZF1//CPEB3//FOXF1//MYT1L//SIN3B//SIRT1//ZFPM2//DNAJB5//GLI3//CNOT7//HIC1//FOXA1//HSBP1//ID2//IFNG//IRF2//AR//ISL1//JARID2//JUN//MAF//MDM4//MEF2A//MYB//NFIA//NFIB//NFIC//NR4A2//PAX5//KLF3//POU4F1//PPARG//ZBTB4//RB1//RBBP8//CCND1//RNF2//SARS//PRDM16//SMARCA2//SUV39H1//ZEB1//TFAP2A//TGIF1//KLF10//TNF//UBE2I//WT1//ZNF217//LRP8//BHLHE41//TBL1XR1//E2F8//ARID5B//BHLHE40//TP63//RUNX3//NOG//FOXP2//PHF14//GTF2E1//GTF2H1//CREBRF//ELK3//ETV5//ATF5//FOXF2//FLI1//GTF2A1//HIVEP2//IRF1//MEOX2//MYBL1//MYCN//NEUROD1//NHLH2//OTX2//KLF13//PKNOX1//POU3F2//FOXJ2//HIVEP3//BMPR2//SOX4//SOX5//BTF3//TFE3//TXK//FOSL1//KLF7//CBFB//FUBP3//CCNT2//NMI//CLOCK//ZNF516//POLR3H//SRSF2//SRSF6//SLBP//GFPT1//SLC35D1//NUP54//HPRT1//ADSS//ADCY3//IMPDH1//CMPK1//BAHD1//H2AFZ//ZMYND11//PNRC1//ZNF526//CREBL2//ZNF800//E2F5//ZNF367//ARID2//MED19//ZFP30//KDM2A//MYCBP2//PHF8//FLII//ADNP//ZMYND8//CNOT6L//ZNF521//EPC2//AGO1//HBP1//FOXP1//ZBTB11//ATAD2//MDFIC//HMGB3//HOXA3//HOXD1//ZNF680//ZNF662//NEUROG1//NFE2//NPAS2//ASCC1//IER5//COMMD10//ARID4B//BRWD1//BNC2//MED9//ZNF532//PRKAA1//TRERF1//MAPK1//SLC2A4RG//CCNL1//PHTF2//CNOT6//ZBTB26//MIER1//PURA//ATXN1//ATXN7//SMARCD2//SS18//MED22//ZNF3//ZNF708//ZNF131//ZNF227//ZNF655//ZSCAN5A//SAP30L//ANP32A//PPP1R1B//PCGF5//LCOR//ST18//HIPK3//PPARGC1B//ELAVL2//ELL2//NR5A2//NRBF2//AFF1//MLLT6//OGG1//PHF20L1//PPP2CA//PAK6//RORA//BTG1//UBE2V1//CSDE1//SNIP1//TEAD2//RPS6KA5//EMX2//ETS1//BOLA3//NR3C2//RFX7//TRAK2//BDP1//NRBP1//PTGES3//GRM3//G3BP2//EREG//CRHR1//FLNA//BAMBI//IGF1//LRP6//WNT7A//ACVR1//DNAJB6//NRG1//RASD1//BTAF1//PPM1F//TOB1//SMURF1//BMP3//GDF5//SULF1//LEMD3//CTDSPL2//ADRB1//PTHLH//CALM2//EGLN1//RWDD3//TNFAIP3//DCP2//CCT6A//INSIG2//MYOG//C5AR1//INTS8//NABP1//FZD6//SMUG1//ADCYAP1//S1PR1//KPNA6//DLL1//TBK1//IL2//MET//PPP1R12A//NCK1//ARMCX3//CYTL1//PPP3R1//MAVS//RAF1//YES1//PDE5A//C8ORF44-SGK3//SGK3//SGK1//PTEN//RHEBL1//ROR1//IRAK4//EGLN3//PSMA2//PSMD12//SEPSECS//RAP2C//HSBP1L1//TMBIM6//DUSP1//GJA1//KCNK2//FBXW7//NPNT// |
| GO:0045446 | endothelial cell differentiation | Biological process | 20 | 105 | 902 | 17653 | 3.72780065462992 | 3.03644395778007e-07 | 1.77029917986781e-05 | 6.51763473003672 | 0.0221729490022173 | CCM2//CLIC4//MET//GSTM3//WNT7A//ZEB1//BTG1//BMPR2//DLL1//NRG1//PPP1R16B//MSN//RAP1A//RAP1B//RAPGEF2//ACVR1//RAP2C//FOXJ2//TNF//S1PR1// |
| GO:0042127 | regulation of cell proliferation | Biological process | 131 | 1668 | 902 | 17653 | 1.5370473022912 | 3.21396263179031e-07 | 1.85778028023913e-05 | 6.49295917701313 | 0.145232815964523 | SULF1//GJA1//TNF//F3//SIRT1//RICTOR//PPP1R16B//JUN//KDR//BMPR2//VEGFB//VASH2//GLI3//FZD3//FOXF1//MYCN//TGFBR2//TP63//FOXP2//ISL1//DNAJA2//ADCYAP1//CNTFR//CRKL//CACUL1//EDN1//EDN2//EDNRB//EFNB2//EREG//ETS1//CNOT6L//BAMBI//DLL1//CNOT7//NRG1//HOXA3//IFNG//IGF1//IL2//AR//NTF3//POU3F2//PRKAA1//MAPK1//SPHK2//PTEN//PTHLH//CNOT6//PURA//SOX4//TTK//FOSL1//CCND2//TOB1//COPS8//ARID2//ATF5//FRZB//TES//IGFBP3//IRF1//MDM4//MYOG//KLF13//RAF1//SMARCA2//STRN//ZEB1//BTG1//TFAP2A//KLF10//TSC1//WT1//CUL5//SKAP2//DLG5//RAPGEF2//ST18//SRSF6//TNFRSF21//CCND1//NCK1//CD3E//CEBPB//PDE5A//LRP6//ESR1//MORC3//S1PR1//FOXP1//ID2//MYB//IRAK4//OGN//PPARG//TNFAIP3//C5AR1//NOG//STRAP//RB1//GDF5//RUNX3//JARID2//KCNK2//ZFPM2//WNT7A//ETV5//ERRFI1//CCL8//FOXJ2//WNT3//SHCBP1//FLNA//FBXW7//NFIB//PHF14//C8ORF44-SGK3//SGK2//CFDP1//EGLN3//ARX//EPS15//SGK3//NR5A2//CXCL9//IER5//SGK1//TXK//YES1//BAG6// |
| GO:0022603 | regulation of anatomical structure morphogenesis | Biological process | 96 | 1123 | 902 | 17653 | 1.6730289669933 | 3.83256226481284e-07 | 2.19657784719739e-05 | 6.41651078075409 | 0.106430155210643 | TNFAIP3//WT1//BMPR2//ISL1//CFDP1//WASF3//PALM2//LPAR1//EPS8//BAMBI//ANXA7//KDR//RHOB//RND3//MSN//BRWD1//RASA1//CCL2//PLXNA4//WDR1//DLL1//SPRED1//SULF1//AGO1//PPARG//FOXJ2//SARS//SH3D19//CAMSAP1//ARC//DPYSL2//RAB21//WNT7A//EDN1//WNT10A//EFNA1//ETS1//TNFRSF12A//EGLN1//VASH2//F3//SIRT1//ITGB8//BTG1//TGFBR2//C5AR1//VEGFB//ADAM12//ADNP//NRG1//ULK2//WNT3//FMR1//HECW2//FXR1//SEMA4C//POU3F2//PTEN//TRAK2//SYNGAP1//ROBO2//SLIT2//RAPGEF2//PRICKLE2//ROR1//PSMA2//SMURF1//PSMD12//FZD3//FZD6//SRSF6//FOXP2//AR//ESR1//BTBD7//TNF//ETV5//LRP8//TFAP2A//MEOX2//MIEF1//NOG//CRKL//FLNA//FOXP1//MYOG//CXCL9//PPP1R16B//EFNB2//SYT1//PLAA//ACVR1//FBXW7//OTX2//NFIB//RAP1A// |
| GO:0010605 | negative regulation of macromolecule metabolic process | Biological process | 201 | 2842 | 902 | 17653 | 1.38415258296912 | 3.96268704641756e-07 | 2.25207163822874e-05 | 6.4020102247591 | 0.222838137472284 | TSHZ1//ZBTB18//CEBPB//TCERG1//STRAP//KLF12//GSC//ARX//DNMT3B//EDN1//EDNRB//EFNA1//CC2D1B//EN1//EP300//ESR1//JAZF1//CPEB3//FOXF1//MYT1L//SIN3B//SIRT1//ZFPM2//DNAJB5//GLI3//CNOT7//HIC1//FOXA1//HSBP1//ID2//IFNG//IRF2//AR//ISL1//JARID2//JUN//MAF//MDM4//MEF2A//MYB//NFIA//NFIB//NFIC//NR4A2//PAX5//KLF3//POU4F1//PPARG//ZBTB4//RB1//RBBP8//CCND1//RNF2//SARS//PRDM16//SMARCA2//SUV39H1//ZEB1//TFAP2A//TGIF1//KLF10//TNF//UBE2I//WT1//ZNF217//LRP8//BHLHE41//TBL1XR1//E2F8//ARID5B//BHLHE40//TP63//RUNX3//NOG//FOXP2//PHF14//PNRC1//DCP2//PPP2CA//SPRED1//DUSP1//CNOT6L//PAN3//CNOT6//PATL1//AGO1//IGFBP3//IL2//LRP6//PTEN//PRR5L//PPP1R15B//SLIT2//ERRFI1//GPRC5A//SOCS5//BAHD1//H2AFZ//GADD45A//GNAQ//NCK1//GSKIP//YWHAG//SOCS1//SOCS3//PPM1F//SNIP1//PI15//ADNP//GJA1//TBK1//ANXA7//ITGB8//KDR//LDLR//MYCN//POU3F2//NDFIP2//CD3E//CALM2//SERPINI1//SERPINB13//SPOCK1//RECK//CELF1//FLNA//DNAJB6//PPARGC1B//ELK3//EREG//ATF5//FOXF2//FOXP1//MDFIC//NRG1//IRF1//RASD1//PURA//ATXN1//BTAF1//RPS6KA5//CLOCK//TOB1//FMR1//NANOS1//TIA1//TSC1//FXR1//MEX3B//TSN//TNRC6B//SPOPL//SOX4//TNFAIP3//BAG5//ATAD2//MIER1//BAG6//ARPP19//PPP1R1B//FBXW7//RASSF2//PPP1R16B//ARL6IP1//RAF1//YTHDF2//PSMA2//PSMD12//ANP32A//HIPK3//DDX6//NMI//TMEFF2//PAIP2//ZMYND11//SRSF6//INHBB//NTF3//PMEPA1//LDLRAD4//TMBIM6//NUP54//GTPBP1//QKI//PHF8//RANBP9//BOLL//CSDE1//CAST//PLAA//CPEB2//OGG1//CRTAP//HSBP1L1//NPAT//SRSF2//ZMYND8//KCNK2//BMPR2//PRKAR2B//CPEB1// |
| GO:0051172 | negative regulation of nitrogen compound metabolic process | Biological process | 186 | 2591 | 902 | 17653 | 1.40493915061602 | 4.53606682152113e-07 | 2.53673064859763e-05 | 6.343320556407 | 0.206208425720621 | TSHZ1//ZBTB18//CEBPB//TCERG1//STRAP//KLF12//GSC//ARX//DNMT3B//EDN1//EDNRB//EFNA1//CC2D1B//EN1//EP300//ESR1//JAZF1//CPEB3//FOXF1//MYT1L//SIN3B//SIRT1//ZFPM2//DNAJB5//GLI3//CNOT7//HIC1//FOXA1//HSBP1//ID2//IFNG//IRF2//AR//ISL1//JARID2//JUN//MAF//MDM4//MEF2A//MYB//NFIA//NFIB//NFIC//NR4A2//PAX5//KLF3//POU4F1//PPARG//ZBTB4//RB1//RBBP8//CCND1//RNF2//SARS//PRDM16//SMARCA2//SUV39H1//ZEB1//TFAP2A//TGIF1//KLF10//TNF//UBE2I//WT1//ZNF217//LRP8//BHLHE41//TBL1XR1//E2F8//ARID5B//BHLHE40//TP63//RUNX3//NOG//FOXP2//PHF14//SPRED1//DUSP1//PPP2CA//IGFBP3//IL2//LRP6//PTEN//PRR5L//PPP1R15B//SLIT2//ERRFI1//GPRC5A//SOCS5//BAHD1//H2AFZ//GADD45A//GNAQ//NCK1//GSKIP//YWHAG//SOCS1//SOCS3//PPM1F//GRM3//AGO1//SNIP1//PI15//CALM2//SERPINI1//SERPINB13//SPOCK1//RECK//FLNA//DNAJB6//PPARGC1B//ELK3//EREG//ATF5//FOXF2//FOXP1//MDFIC//NRG1//IRF1//RASD1//PURA//ATXN1//BTAF1//RPS6KA5//CLOCK//TOB1//CELF1//FMR1//NANOS1//TIA1//TSC1//FXR1//MEX3B//EGLN1//SPOPL//SOX4//TNFAIP3//BAG5//ATAD2//MIER1//DCP2//BAG6//ARPP19//PPP1R1B//FBXW7//RASSF2//CNOT6//TNRC6B//PPP1R16B//ARL6IP1//RAF1//HIPK3//NMI//MYOG//PAIP2//PRKAA1//ZMYND11//SRSF6//INHBB//NTF3//LDLR//PMEPA1//LDLRAD4//TMBIM6//NUP54//CNOT6L//YTHDF2//QKI//PHF8//RANBP9//BOLL//CAST//PLAA//CPEB2//OGG1//CRTAP//HSBP1L1//NPAT//SRSF2//ZMYND8//MYCN//GJA1//KCNK2//BMPR2//PRKAR2B//CPEB1// |
| GO:0042981 | regulation of apoptotic process | Biological process | 124 | 1566 | 902 | 17653 | 1.54967958248026 | 4.53858359426753e-07 | 2.53673064859763e-05 | 6.34307966105701 | 0.137472283813747 | PTEN//FOXP1//IL2//EGLN3//F3//PMAIP1//PPARG//TNF//CASP2//HIPK3//DUSP1//EDNRB//ATF5//FLNA//ARL6IP1//SIRT1//KLHL20//GLI3//IGF1//KDR//MDM4//MET//ROR1//OGG1//ZFAND6//PRKAA1//SPHK2//RAF1//RASA1//BCL2A1//BNIP2//TMBIM6//TFAP2A//WNT7A//WT1//BAG6//TP63//CCND2//SOCS3//ADCYAP1//PIK3CB//DLL1//CCL2//EDN1//LRP6//IFNG//GADD45A//LPAR1//CDK19//PHLDA3//FRZB//FOXA1//IGFBP3//JUN//RHOB//USP27X//NEUROD1//ARHGEF4//TNFRSF12A//POU4F1//ZMAT3//ITSN1//SOS2//SOX4//FOSL1//SLIT2//RASSF2//DNAJB6//PPM1F//CEBPB//CNTFR//EN1//UNC5B//ADNP//GABRA5//GRIK2//ISL1//NTF3//NR4A2//PIK3CA//C5AR1//GDF5//SYNGAP1//MYB//EFNA1//FZD3//DYNLL2//PPP3R1//YWHAG//TNFAIP3//SERPINB13//BAG5//SERINC3//NCK1//FBXW7//TIA1//IER3IP1//CFDP1//BTG1//CCL21//RAPGEF2//KCNB1//SRSF6//CAST//NOG//INHBB//ZMYND11//AR//ACVR1//ST18//C8ORF44-SGK3//SGK2//ESR1//SGK3//TNFRSF21//PAK6//TAOK1//RBM25//SGK1//BMP3//ANP32A//CD3E//DLG5//SLK// |
| GO:0007417 | central nervous system development | Biological process | 83 | 932 | 902 | 17653 | 1.74290679748389 | 4.80728626407622e-07 | 2.66489155770061e-05 | 6.31810001575215 | 0.0920177383592018 | DPYSL2//S1PR1//RAB3GAP1//RAB18//PHF8//GABRA5//SPHK2//PTPRG//ROBO2//ATXN1//SLC6A11//BMPR2//SYT1//TGFBR2//BAG6//CASP2//IMMP2L//DLL1//ROR1//POU3F2//WASF3//LPAR1//ID2//SOX4//NOG//ISL1//C5AR1//POU4F1//SEMA4C//LRP8//EMX2//NEUROD1//PTEN//EN1//CD3E//FOXP2//LRP6//RAPGEF2//PLXNA4//TFAP2A//PAX5//RORA//WNT7A//NFIB//HPRT1//ARX//CALM2//GLI3//TSC1//SLIT2//NRG1//ETS1//GNAQ//ATF5//FLNA//NR4A2//SPOCK1//SCN1B//ADCYAP1//TACC2//KCNJ10//GSC//OTX2//DLG5//FZD3//FZD6//MYCN//TNFRSF21//WDR1//PPARG//MAPK1//LDLR//NANOS1//WNT3//HAPLN1//FMR1//JARID2//NHLH2//NPAS2//HPCAL4//SERPINI1//ZEB1//FXR1// |
| GO:0010975 | regulation of neuron projection development | Biological process | 49 | 450 | 902 | 17653 | 2.13105937423011 | 4.86330066825495e-07 | 2.67402458694376e-05 | 6.31306887981955 | 0.0543237250554324 | STMN2//ADCYAP1//NPTN//NCK1//SERPINI1//RAP1A//SCN1B//RAPGEF2//LPAR1//EFNB2//MYLIP//PTPRG//SPOCK1//TSC1//BAG5//DPYSL2//RAB21//WNT7A//ADNP//NRG1//TNFRSF12A//ULK2//PTEN//WNT3//FMR1//HECW2//FXR1//PLXNA4//BMPR2//SEMA4C//EFNA1//POU3F2//TRAK2//SYNGAP1//ROBO2//SLIT2//DBN1//CAMSAP2//CPEB3//ZMYND8//IL2//MAPK6//DLG5//ARC//LRP8//SMURF1//SYT1//PLAA//SNAP25// |
| GO:0031324 | negative regulation of cellular metabolic process | Biological process | 195 | 2748 | 902 | 17653 | 1.38876852990443 | 4.9731128812152e-07 | 2.71235180771439e-05 | 6.30337168290884 | 0.216186252771619 | TSHZ1//ZBTB18//CEBPB//TCERG1//STRAP//KLF12//GSC//ARX//DNMT3B//EDN1//EDNRB//EFNA1//CC2D1B//EN1//EP300//ESR1//JAZF1//CPEB3//FOXF1//MYT1L//SIN3B//SIRT1//ZFPM2//DNAJB5//GLI3//CNOT7//HIC1//FOXA1//HSBP1//ID2//IFNG//IRF2//AR//ISL1//JARID2//JUN//MAF//MDM4//MEF2A//MYB//NFIA//NFIB//NFIC//NR4A2//PAX5//KLF3//POU4F1//PPARG//ZBTB4//RB1//RBBP8//CCND1//RNF2//SARS//PRDM16//SMARCA2//SUV39H1//ZEB1//TFAP2A//TGIF1//KLF10//TNF//UBE2I//WT1//ZNF217//LRP8//BHLHE41//TBL1XR1//E2F8//ARID5B//BHLHE40//TP63//RUNX3//NOG//FOXP2//PHF14//SPRED1//DUSP1//PPP2CA//IGFBP3//IL2//LRP6//PTEN//PRR5L//PPP1R15B//SLIT2//ERRFI1//GPRC5A//SOCS5//BAHD1//H2AFZ//GADD45A//GNAQ//NCK1//GSKIP//YWHAG//SOCS1//SOCS3//PPM1F//GRM3//AGO1//SNIP1//PI15//MET//MTMR9//MYCN//CALM2//FBXW7//FKBP15//PPP1R12A//PLEKHF2//SERPINI1//SERPINB13//SPOCK1//RECK//PIK3CA//TSC1//FLNA//DNAJB6//PPARGC1B//ELK3//EREG//ATF5//FOXF2//FOXP1//MDFIC//NRG1//IRF1//RASD1//PURA//ATXN1//BTAF1//RPS6KA5//CLOCK//TOB1//CELF1//FMR1//NANOS1//TIA1//FXR1//MEX3B//EGLN1//SPOPL//SOX4//TNFAIP3//BAG5//ATAD2//MIER1//DCP2//BAG6//ARPP19//PPP1R1B//RASSF2//CNOT6//TNRC6B//PPP1R16B//ATXN7//ARL6IP1//RAF1//HIPK3//NMI//INSIG2//MYOG//PAIP2//PRKAA1//ZMYND11//RAPGEF2//SRSF6//INHBB//NTF3//PMEPA1//LDLRAD4//TMBIM6//NUP54//CNOT6L//YTHDF2//QKI//PHF8//RANBP9//BOLL//CAST//PLAA//CPEB2//OGG1//CRTAP//FEZ2//HSBP1L1//NPAT//SRSF2//ZMYND8//GJA1//KCNK2//BMPR2//PRKAR2B//CPEB1// |
| GO:0018130 | heterocycle biosynthetic process | Biological process | 297 | 4550 | 902 | 17653 | 1.27748860895202 | 5.1882548821872e-07 | 2.80705342145856e-05 | 6.28497869658018 | 0.329268292682927 | NPAT//TSHZ1//ZBTB18//CEBPB//TCERG1//STRAP//KLF12//GSC//ARX//DNMT3B//EDN1//EDNRB//EFNA1//CC2D1B//EN1//EP300//ESR1//JAZF1//CPEB3//FOXF1//MYT1L//SIN3B//SIRT1//ZFPM2//DNAJB5//GLI3//CNOT7//HIC1//FOXA1//HSBP1//ID2//IFNG//IRF2//AR//ISL1//JARID2//JUN//MAF//MDM4//MEF2A//MYB//NFIA//NFIB//NFIC//NR4A2//PAX5//KLF3//POU4F1//PPARG//ZBTB4//RB1//RBBP8//CCND1//RNF2//SARS//PRDM16//SMARCA2//SUV39H1//ZEB1//TFAP2A//TGIF1//KLF10//TNF//UBE2I//WT1//ZNF217//LRP8//BHLHE41//TBL1XR1//E2F8//ARID5B//BHLHE40//TP63//RUNX3//NOG//FOXP2//PHF14//GTF2E1//GTF2H1//CREBRF//ELK3//ETV5//ATF5//FOXF2//FLI1//GTF2A1//HIVEP2//IRF1//MEOX2//MYBL1//MYCN//NEUROD1//NHLH2//OTX2//KLF13//PKNOX1//POU3F2//FOXJ2//HIVEP3//BMPR2//SOX4//SOX5//BTF3//TFE3//TXK//FOSL1//KLF7//CBFB//FUBP3//CCNT2//NMI//CLOCK//ZNF516//POLR3H//SRSF2//SRSF6//SLBP//GFPT1//SLC35D1//NUP54//HPRT1//ADSS//ADCY3//IMPDH1//CMPK1//BAHD1//H2AFZ//ZMYND11//PNRC1//ZNF526//CREBL2//ZNF800//E2F5//ZNF367//ARID2//MED19//ZFP30//KDM2A//MYCBP2//PHF8//FLII//ADNP//ZMYND8//CNOT6L//ZNF521//EPC2//AGO1//HBP1//FOXP1//ZBTB11//ATAD2//MDFIC//HMGB3//HOXA3//HOXD1//ZNF680//ZNF662//NEUROG1//NFE2//NPAS2//ASCC1//IER5//COMMD10//ARID4B//BRWD1//BNC2//MED9//ZNF532//PRKAA1//TRERF1//MAPK1//SLC2A4RG//CCNL1//PHTF2//CNOT6//ZBTB26//MIER1//PURA//ATXN1//ATXN7//SMARCD2//SS18//MED22//ZNF3//ZNF708//ZNF131//ZNF227//ZNF655//ZSCAN5A//SAP30L//ANP32A//PPP1R1B//PCGF5//LCOR//ST18//HIPK3//PPARGC1B//ELAVL2//ELL2//NR5A2//NRBF2//AFF1//MLLT6//OGG1//PHF20L1//PPP2CA//PAK6//RORA//BTG1//UBE2V1//CSDE1//SNIP1//TEAD2//RPS6KA5//EMX2//ETS1//BOLA3//NR3C2//RFX7//TRAK2//BDP1//NRBP1//GPHN//PTGES3//GRM3//G3BP2//PSAT1//EREG//CRHR1//FLNA//BAMBI//IGF1//LRP6//WNT7A//ACVR1//DNAJB6//NRG1//RASD1//BTAF1//PPM1F//TOB1//SMURF1//BMP3//GDF5//SULF1//LEMD3//CTDSPL2//ADRB1//PTHLH//CALM2//EGLN1//RWDD3//TNFAIP3//DCP2//CCT6A//INSIG2//MYOG//C5AR1//INTS8//NABP1//FZD6//SMUG1//ADCYAP1//S1PR1//KPNA6//DLL1//TBK1//IL2//MET//PPP1R12A//NCK1//ARMCX3//CYTL1//PPP3R1//MAVS//RAF1//YES1//PDE5A//C8ORF44-SGK3//SGK3//SGK1//PTEN//RHEBL1//ROR1//IRAK4//EGLN3//PSMA2//PSMD12//SEPSECS//RAP2C//HSBP1L1//TMBIM6//DUSP1//GJA1//KCNK2//FBXW7//NPNT// |
| GO:0023052 | signaling | Biological process | 414 | 6724 | 902 | 17653 | 1.20499326633524 | 5.4489244805456e-07 | 2.92468859221666e-05 | 6.26368921116537 | 0.458980044345898 | KCNH4//RANBP9//SPRED1//EREG//RASGEF1A//NRG1//IL2//MEF2A//MET//MAPK1//MAPK6//PSMA2//PSMD12//RAF1//RASA1//CCL2//TNF//SYNGAP1//RAPGEF2//GADD45A//CRKL//TAOK1//RAP1A//C1QTNF2//LPAR1//IGF1//NTF3//PIK3CB//PRKAA1//C5AR1//UBE2V1//DUSP1//PPP2CA//JUN//PMAIP1//BCL2A1//EPS15//NSG1//VAMP3//CBFB//EP300//PRKACB//SKP1//RPS6KA5//IRAK4//BAG6//CR2//SKAP2//TMED1//ADCYAP1//ADRB1//EDN1//EFNA1//EFNB2//GJA1//AR//CXCL9//PCDHB10//PTHLH//PCDH10//CCL8//CCL21//BMP3//KLF10//ZYX//GDF5//S1PR1//SPHK2//APCDD1//CCNY//HBP1//GRK6//HIC1//LRP6//STRN//WNT3//WNT7A//CXXC4//WNT10A//TNRC6B//AGO1//PPP3R1//FZD3//FZD6//CNIH1//CDS1//PTGES3//IQGAP2//ADCY3//RALBP1//RASSF8//CHRNB4//ARAP2//CLCN6//CNGA3//CNTFR//CREBL2//DPYSL2//DTNA//ELK3//EPS8//ESR1//CD2AP//GABRA5//APPL1//ATP2C1//GLRB//CNOT7//LRP12//HIVEP2//NR3C2//PPP1R12A//NR4A2//HPCAL4//GULP1//RASD1//KCNK10//CYTL1//NDFIP2//PPARG//PPP2R5E//PSD//MAVS//ARRDC3//MIER1//HIVEP3//SMOC1//SMOC2//SPOCK1//ZNF217//LRP8//GLRA3//SNX27//SYDE2//PPP1R1B//PPFIA1//OR6A2//PDE5A//GPRC5A//DLG5//PLAA//ARHGAP12//GTPBP1//CLOCK//FEZ2//ZNF516//CD69//ULK2//SNX17//KDR//ROR1//PTPRG//TXK//YES1//CD3E//CD8A//C8ORF44-SGK3//SGK2//AKAP11//DGKH//SGK3//NRBP1//ARHGEF4//PRKAR2B//SGK1//STK33//SS18//ANP32A//DGKE//RALGPS1//PALM2//BMPR2//FBXW7//ERRFI1//SOCS5//CNOT6L//MDM4//CNOT6//SOX4//E2F8//CASP2//TP63//PPP1R15B//CRHR1//EDN2//EDNRB//IGSF3//IFNG//TSPAN11//NCK1//PIK3CA//SIRT1//SMURF1//TGFBR2//ACVR1//NMUR1//RAPGEF4//CHRM2//TAPT1//FRZB//GNAQ//OR4N4//GRM3//KCNK2//RHOB//RGS7BP//ITSN1//SOS2//CALM2//GPR55//FLNA//ANO1//HOMER1//FMR1//GRIK2//KCNB1//NXPH3//SORCS1//SORCS2//SUSD5//DLL1//FOXA1//TMEM17//EVC//DZIP1//FOXF1//GLI3//ITGB8//CCM2//RCAN3//SAMD14//TBK1//SNIP1//COPS8//MDFIC//SOCS1//SOCS3//NMI//NEUROD1//RAB18//RGL1//RND3//ARHGAP1//RIN2//DOCK10//DIRAS3//USP28//RB1//G3BP2//ARHGAP6//PAK6//GJC1//NOVA1//SCN1B//SLC6A1//SNAP25//SYT1//BSN//PTEN//RIC3//ETV5//TRPC3//SLC25A6//MARCKS//GIT2//TIA1//SHCBP1//OTX2//RORA//GSKIP//NR5A2//ADIPOR2//RWDD3//YWHAG//TOB1//LASP1//SH3BGRL//IGFBP3//PRR5L//TXLNA//IFNA1//INHBB//OGN//VEGFB//F3//TNFAIP3//TTK//PMEPA1//LDLRAD4//PPP1R16B//UNC5B//ZEB1//NREP//PLP2//CCND1//SDC1//SPPL3//DENND1B//TNFRSF21//SLK//GSC//SNX19//SULF1//SHISA2//NOG//NPNT//STRAP//LEMD3//FAM89B//BAMBI//PRDM16//CTDSPL2//PPARGC1B//CREBRF//SERP1//SLC9A6//RHEBL1//RICTOR//FAM83D//TSC1//RRAGD//IQSEC2//FBXO8//AGFG1//ISL1//RAB12//RAB21//RAB30//RAB9B//RAB8B//RAB28//RAP2C//RAP1B//MID1//SEMA4C//INSIG2//UBA5//EDARADD//TNFRSF12A//ST18//ACSL1//IRF1//PLEKHG4B//TEAD2//MOB1B//ROBO2//SLIT2//C2CD5//GSTA2//MSN//SYBU//GFPT1//DNAJB9//ASNA1//SEC61A2//BRWD1//CUL5//RASSF2//PHLDA3//ZFAND6//UBE2I//ZMYND11//HIPK3//ATXN7//PEX5L//TMEM64//NPTN//YTHDF2//DLK2//PPP2CB//ARID5B//GIGYF1//RIMS4//CPEB3//DBN1//ARC//KCNJ10//RAB3GAP1//DHRS3//SYT6//PXK//ADNP//GCSAML//PAX5//OTUD3//PRICKLE2//IRF2//FOXP1//KCNE1//KCNJ2//KCNIP4//CEBPB//TMBIM6//FIBIN//PLXNA4//POU4F1//ZMAT3//KCNJ3//TBL1XR1//FASTK//SIVA1//PPM1F//ZMYND8//FXR1//LYPD6//PAIP2//DYNLL2//RBBP8//BAG5//SERINC3//SESN1//PFN2//PHF14//MYB// |
| GO:2000113 | negative regulation of cellular macromolecule biosynthetic process | Biological process | 128 | 1636 | 902 | 17653 | 1.53122374077708 | 5.50994336888391e-07 | 2.93415330738283e-05 | 6.25885286479751 | 0.141906873614191 | TSHZ1//ZBTB18//CEBPB//TCERG1//STRAP//KLF12//GSC//ARX//DNMT3B//EDN1//EDNRB//EFNA1//CC2D1B//EN1//EP300//ESR1//JAZF1//CPEB3//FOXF1//MYT1L//SIN3B//SIRT1//ZFPM2//DNAJB5//GLI3//CNOT7//HIC1//FOXA1//HSBP1//ID2//IFNG//IRF2//AR//ISL1//JARID2//JUN//MAF//MDM4//MEF2A//MYB//NFIA//NFIB//NFIC//NR4A2//PAX5//KLF3//POU4F1//PPARG//ZBTB4//RB1//RBBP8//CCND1//RNF2//SARS//PRDM16//SMARCA2//SUV39H1//ZEB1//TFAP2A//TGIF1//KLF10//TNF//UBE2I//WT1//ZNF217//LRP8//BHLHE41//TBL1XR1//E2F8//ARID5B//BHLHE40//TP63//RUNX3//NOG//FOXP2//PHF14//BAHD1//H2AFZ//AGO1//SNIP1//FLNA//DNAJB6//PPARGC1B//ELK3//EREG//ATF5//FOXF2//FOXP1//MDFIC//NRG1//IRF1//RASD1//PURA//ATXN1//BTAF1//RPS6KA5//CLOCK//PPM1F//TOB1//CELF1//FMR1//NANOS1//TIA1//TSC1//FXR1//MEX3B//ATAD2//MIER1//DCP2//FBXW7//CNOT6//TNRC6B//PAIP2//NUP54//CNOT6L//YTHDF2//QKI//PHF8//CPEB2//ZMYND8//MYCN//NCK1//TMBIM6//DUSP1//GJA1//KCNK2//BMPR2//CPEB1// |
| GO:0019438 | aromatic compound biosynthetic process | Biological process | 297 | 4558 | 902 | 17653 | 1.27524641744882 | 6.1279847597356e-07 | 3.23777819766343e-05 | 6.2126823234621 | 0.329268292682927 | NPAT//TSHZ1//ZBTB18//CEBPB//TCERG1//STRAP//KLF12//GSC//ARX//DNMT3B//EDN1//EDNRB//EFNA1//CC2D1B//EN1//EP300//ESR1//JAZF1//CPEB3//FOXF1//MYT1L//SIN3B//SIRT1//ZFPM2//DNAJB5//GLI3//CNOT7//HIC1//FOXA1//HSBP1//ID2//IFNG//IRF2//AR//ISL1//JARID2//JUN//MAF//MDM4//MEF2A//MYB//NFIA//NFIB//NFIC//NR4A2//PAX5//KLF3//POU4F1//PPARG//ZBTB4//RB1//RBBP8//CCND1//RNF2//SARS//PRDM16//SMARCA2//SUV39H1//ZEB1//TFAP2A//TGIF1//KLF10//TNF//UBE2I//WT1//ZNF217//LRP8//BHLHE41//TBL1XR1//E2F8//ARID5B//BHLHE40//TP63//RUNX3//NOG//FOXP2//PHF14//GTF2E1//GTF2H1//CREBRF//ELK3//ETV5//ATF5//FOXF2//FLI1//GTF2A1//HIVEP2//IRF1//MEOX2//MYBL1//MYCN//NEUROD1//NHLH2//OTX2//KLF13//PKNOX1//POU3F2//FOXJ2//HIVEP3//BMPR2//SOX4//SOX5//BTF3//TFE3//TXK//FOSL1//KLF7//CBFB//FUBP3//CCNT2//NMI//CLOCK//ZNF516//POLR3H//SRSF2//SRSF6//SLBP//GFPT1//SLC35D1//NUP54//HPRT1//ADSS//ADCY3//IMPDH1//CMPK1//BAHD1//H2AFZ//ZMYND11//PNRC1//ZNF526//CREBL2//ZNF800//E2F5//ZNF367//ARID2//MED19//ZFP30//KDM2A//MYCBP2//PHF8//FLII//ADNP//ZMYND8//CNOT6L//ZNF521//EPC2//AGO1//HBP1//FOXP1//ZBTB11//ATAD2//MDFIC//HMGB3//HOXA3//HOXD1//ZNF680//ZNF662//NEUROG1//NFE2//NPAS2//ASCC1//IER5//COMMD10//ARID4B//BRWD1//BNC2//MED9//ZNF532//PRKAA1//TRERF1//MAPK1//SLC2A4RG//CCNL1//PHTF2//CNOT6//ZBTB26//MIER1//PURA//ATXN1//ATXN7//SMARCD2//SS18//MED22//ZNF3//ZNF708//ZNF131//ZNF227//ZNF655//ZSCAN5A//SAP30L//ANP32A//PPP1R1B//PCGF5//LCOR//ST18//HIPK3//PPARGC1B//ELAVL2//ELL2//NR5A2//NRBF2//AFF1//MLLT6//OGG1//PHF20L1//PPP2CA//PAK6//RORA//BTG1//UBE2V1//CSDE1//SNIP1//TEAD2//RPS6KA5//EMX2//ETS1//BOLA3//NR3C2//RFX7//TRAK2//BDP1//NRBP1//PTGES3//GRM3//G3BP2//PSAT1//EREG//CRHR1//FLNA//BAMBI//IGF1//LRP6//WNT7A//ACVR1//DNAJB6//NRG1//RASD1//BTAF1//PPM1F//TOB1//SMURF1//BMP3//GDF5//SULF1//LEMD3//CTDSPL2//ADRB1//PTHLH//CALM2//EGLN1//RWDD3//TNFAIP3//DCP2//CCT6A//INSIG2//MYOG//C5AR1//INTS8//NABP1//FZD6//SMUG1//ADCYAP1//S1PR1//KPNA6//DLL1//TBK1//IL2//MET//PPP1R12A//NCK1//ARMCX3//CYTL1//PPP3R1//MAVS//RAF1//YES1//PDE5A//RAPGEF2//C8ORF44-SGK3//SGK3//SGK1//PTEN//RHEBL1//ROR1//IRAK4//EGLN3//PSMA2//PSMD12//SEPSECS//RAP2C//HSBP1L1//TMBIM6//DUSP1//GJA1//KCNK2//FBXW7//NPNT// |
| GO:0070887 | cellular response to chemical stimulus | Biological process | 219 | 3175 | 902 | 17653 | 1.34993348115299 | 6.78196872090459e-07 | 3.55553910538587e-05 | 6.16864421756145 | 0.242793791574279 | CBFB//CCL21//CCL2//CCL8//SLIT2//EDN2//CXCL9//PTGES3//MGST1//RORA//EP300//SIRT1//JUN//SMURF1//TGFBR2//KLF10//ZYX//GDF5//ACVR1//CHRM2//GNAQ//APPL1//SPRED1//MAPK1//TIA1//SHCBP1//GSKIP//NR5A2//PPARG//ADIPOR2//AGO1//SNIP1//C5AR1//TNF//TNFAIP3//ZEB1//NREP//CNTFR//CRKL//S1PR1//TXLNA//EREG//F3//IFNA1//IL2//IRAK4//PIK3CA//PIK3CB//PLP2//CCND1//SDC1//LRP8//SOCS1//SOCS3//SOCS5//PLXNA4//CEBPB//CPEB3//SESN1//RRAGD//CPEB1//TSN//TOB1//BMP3//BMPR2//NOG//NPNT//STRAP//LEMD3//FAM89B//BAMBI//PMEPA1//PRDM16//LDLRAD4//SULF1//CTDSPL2//ESR1//PPARGC1B//AR//RB1//CREBRF//SERP1//CPEB2//INHBB//RAB8B//ERRFI1//PTEN//YWHAG//C2CD5//DUSP1//ROBO2//UBA5//ISL1//TP63//FOXA1//EDARADD//TNFRSF21//TNFRSF12A//PSMA2//PSMD12//ST18//ACSL1//ETV5//NR4A2//PRR5L//HSPA13//IRF1//EDN1//PPP1R12A//RAP1A//RAP1B//PPM1F//CD69//GSTA2//IFNG//MSN//RAF1//ANO1//SYBU//KDR//YES1//GFPT1//DNAJB9//ASNA1//SEC61A2//BRWD1//NTF3//RAPGEF2//OTX2//SHISA2//GSTM3//NR3C2//NPTN//NCK1//TSC1//IGF1//EDNRB//SLC9A6//UGT2B28//LPAR1//IRF2//MID1//NMI//TXK//CNOT7//MAVS//VEGFB//FOXP1//EGLN3//EGLN1//ETS1//RHOB//MYB//PRKAA1//SKP1//UBE2V1//RPS6KA5//UBE2W//CDK19//PLAA//OGG1//CLIC4//MEF2A//SYT1//TFAP2A//ID2//MYOG//SLC41A1//WNT3//TEAD2//POU3F2//MAPK6//KCNE1//WT1//KCNB1//NEUROD1//SOX4//FOXF1//TBK1//POU4F1//RAB12//VAMP3//ZFAND6//TGIF1//GJA1//CRHR1//ADCY3//PRKACB//PRKAR2B//ADCYAP1//H2AFZ//LRP6//LDLR//DNMT3B//KCNK2//MDM4//PMAIP1//SUV39H1//SOX5//WNT7A//WNT10A//BAG6//FMR1//DLL1//MET//KLF3//RWDD3//BAG5//TMBIM6//FBXW7//MYCN//PPP1R15B//STMN2//DYNC1LI2//EPS8//JARID2//PIGA//ARID5B//CLOCK// |
| GO:0043067 | regulation of programmed cell death | Biological process | 124 | 1579 | 902 | 17653 | 1.53692097920461 | 7.01925199783341e-07 | 3.65163086625749e-05 | 6.15370916572228 | 0.137472283813747 | PTEN//FOXP1//IL2//EGLN3//F3//PMAIP1//PPARG//TNF//CASP2//HIPK3//DUSP1//EDNRB//ATF5//FLNA//ARL6IP1//SIRT1//KLHL20//GLI3//IGF1//KDR//MDM4//MET//ROR1//OGG1//ZFAND6//PRKAA1//SPHK2//RAF1//RASA1//BCL2A1//BNIP2//TMBIM6//TFAP2A//WNT7A//WT1//BAG6//TP63//CCND2//SOCS3//ADCYAP1//C8ORF44-SGK3//SGK2//ESR1//SGK3//TNFRSF21//IGFBP3//INHBB//NTF3//PAK6//TAOK1//RBM25//SGK1//BMP3//ANP32A//GDF5//CD3E//DLG5//SLK//PIK3CB//DLL1//CCL2//EDN1//LRP6//IFNG//GADD45A//LPAR1//CDK19//PHLDA3//FRZB//FOXA1//JUN//RHOB//USP27X//NEUROD1//ARHGEF4//TNFRSF12A//POU4F1//ZMAT3//ITSN1//SOS2//SOX4//FOSL1//SLIT2//RASSF2//DNAJB6//PPM1F//CEBPB//CNTFR//EN1//UNC5B//ADNP//GABRA5//GRIK2//ISL1//NR4A2//PIK3CA//C5AR1//SYNGAP1//MYB//EFNA1//FZD3//DYNLL2//PPP3R1//YWHAG//TNFAIP3//SERPINB13//BAG5//SERINC3//NCK1//FBXW7//TIA1//IER3IP1//CFDP1//BTG1//CCL21//RAPGEF2//KCNB1//SRSF6//CAST//NOG//ZMYND11//AR//ACVR1//ST18// |
| GO:0012501 | programmed cell death | Biological process | 155 | 2088 | 902 | 17653 | 1.45282460857524 | 7.48975297574608e-07 | 3.86665644083746e-05 | 6.12553250580381 | 0.171840354767184 | TNFRSF21//JUN//PMAIP1//BCL2A1//PTEN//FOXP1//IL2//HIPK3//RTN3//EGLN3//PRUNE2//GADD45A//EP300//UNC5B//ARL6IP1//SULF1//GJA1//NSG1//SLC25A6//IFNG//IGFBP3//IRF1//C6ORF120//RHOB//MEF2A//GULP1//CHMP3//ZFAND6//PPP2CA//DRAM1//MAPK1//PAK6//RAF1//SGK1//BNIP2//TGFBR2//TIA1//TNFAIP3//C5AR1//BAG6//FXR1//CASP2//TP63//EI24//SLK//F3//PPARG//TNF//DUSP1//EDNRB//ATF5//FLNA//SIRT1//KLHL20//GLI3//IGF1//KDR//MDM4//MET//ROR1//OGG1//PRKAA1//SPHK2//RASA1//TMBIM6//TFAP2A//WNT7A//WT1//CCND2//SOCS3//HIC1//GSKIP//PPP2CB//ADCYAP1//C8ORF44-SGK3//SGK2//ESR1//SGK3//INHBB//NTF3//TAOK1//RBM25//BMP3//ANP32A//GDF5//CD3E//DLG5//PIK3CB//RB1//DLL1//CCL2//EDN1//LRP6//PHLDA3//USP28//LPAR1//CDK19//FRZB//FOXA1//USP27X//NEUROD1//ARHGEF4//TNFRSF12A//POU4F1//ZMAT3//ITSN1//SOS2//SOX4//FOSL1//SLIT2//RASSF2//DNAJB6//PIK3CA//PPM1F//CEBPB//CNTFR//EN1//ADNP//GABRA5//GRIK2//ISL1//NR4A2//SYNGAP1//MYB//ROBO2//EFNA1//KRT80//XKR8//BMPR2//FASTK//SIVA1//APPL1//CUL5//FZD3//DYNLL2//PPP3R1//YWHAG//SERPINB13//BAG5//SERINC3//NCK1//FBXW7//IER3IP1//CFDP1//BTG1//CCL21//RAPGEF2//KCNB1//SRSF6//CAST//NOG//ZMYND11//AR//ACVR1//ST18// |
| GO:0023056 | positive regulation of signaling | Biological process | 134 | 1743 | 902 | 17653 | 1.50459424012172 | 7.57530997052822e-07 | 3.88119858565775e-05 | 6.12059959177144 | 0.148558758314856 | GADD45A//TNF//CRKL//TAOK1//RAF1//RAP1A//C1QTNF2//LPAR1//NRG1//IGF1//NTF3//PIK3CB//PRKAA1//MAPK1//C5AR1//UBE2V1//EPS15//NSG1//VAMP3//EREG//FBXW7//COPS8//MDFIC//TOB1//EPS8//ARHGAP1//LASP1//ARHGAP6//SH3BGRL//SKAP2//HOMER1//SOCS1//F3//INHBB//BMP3//BMPR2//TTK//GDF5//ACVR1//UNC5B//SIRT1//KDR//PRR5L//EDN1//SULF1//SOX4//WNT3//NPNT//RICTOR//PIK3CA//RRAGD//GJA1//SERP1//ISL1//MID1//SEMA4C//FOXA1//AR//GPR55//DLG5//ANO1//SYBU//IFNG//IL2//SOCS3//FLNA//ATP2C1//TBK1//ROR1//IRAK4//NDFIP2//MAVS//MIER1//CCL21//UBE2I//PDE5A//EFNA1//IGFBP3//MET//SPRED1//HIC1//PMAIP1//ADCYAP1//ADRB1//CXCL9//RAPGEF2//NPTN//EP300//DLL1//TP63//EVC//WNT7A//RASSF2//RASGEF1A//RIMS4//GRIK2//SYT1//CD3E//SOS2//RAB8B//ESR1//ITSN1//CHRNB4//ARC//PTEN//SNAP25//CALM2//TXK//RAB3GAP1//JUN//CCL2//CCL8//SPPL3//ARRDC3//BAMBI//LRP6//GSKIP//PSMA2//PSMD12//TBL1XR1//PAIP2//FMR1//DYNLL2//PPP3R1//YWHAG//AGO1//RWDD3//CREBRF//SERINC3//NCK1//MYB//CASP2//TNFRSF12A//ST18// |
| GO:0009966 | regulation of signal transduction | Biological process | 221 | 3215 | 902 | 17653 | 1.3453128178956 | 7.6613492980244e-07 | 3.89576731598038e-05 | 6.11569473675361 | 0.245011086474501 | GADD45A//TNF//CRKL//TAOK1//RAF1//RAP1A//C1QTNF2//LPAR1//NRG1//IGF1//NTF3//PIK3CB//PRKAA1//MAPK1//C5AR1//UBE2V1//SPRED1//DUSP1//PPP2CA//CBFB//EREG//FBXW7//ERRFI1//GPRC5A//SOCS5//NCK1//CD3E//COPS8//MDFIC//ADCYAP1//GRK6//GIT2//OTX2//RORA//TOB1//EPS8//ARHGAP1//LASP1//ARHGAP6//SH3BGRL//SKAP2//HOMER1//IGFBP3//KDR//RGS7BP//MET//ROR1//PRR5L//ACVR1//EDN1//EDN2//TXLNA//IFNA1//IFNG//IL2//INHBB//CXCL9//OGN//PTHLH//CCL2//CCL8//CCL21//BMP3//VEGFB//WNT3//WNT7A//WNT10A//GDF5//SOCS1//F3//AKAP11//TNFAIP3//BMPR2//TTK//PMEPA1//LDLRAD4//PPP1R16B//PTEN//UNC5B//SIRT1//ZEB1//NREP//ESR1//SULF1//SOX4//GSC//APCDD1//FRZB//HIC1//SHISA2//CCND1//CXXC4//NPNT//STRAP//LEMD3//FAM89B//BAMBI//SMURF1//PRDM16//TGFBR2//CTDSPL2//NOG//FAM83D//TSC1//RICTOR//PIK3CA//RRAGD//ARAP2//IQSEC2//APPL1//FBXO8//AGFG1//PSD//RALGPS1//MID1//SEMA4C//UBA5//ISL1//TP63//FOXA1//AR//IRF1//PLEKHG4B//ARHGEF4//ITSN1//SOS2//GPR55//DLG5//EPS15//CNOT7//SOCS3//ZFAND6//FLNA//GJA1//ATP2C1//TBK1//IRAK4//NDFIP2//MAVS//MIER1//UBE2I//ZMYND11//PDE5A//PAK6//SYNGAP1//EFNA1//HIPK3//PMAIP1//ATXN7//PEX5L//ADRB1//RAPGEF2//NPTN//YTHDF2//DLK2//EP300//DLL1//GLI3//RB1//EVC//GRIK2//RASSF2//NEUROD1//RASGEF1A//PPP2CB//RASA1//DHRS3//GCSAML//PAX5//RALBP1//RHOB//SYDE2//ARHGAP12//CD2AP//SLIT2//SLC9A6//OTUD3//PHLDA3//CALM2//TXK//PPARG//FOXP1//CCNY//LRP6//RANBP9//JUN//RCAN3//SPPL3//ARRDC3//TMEM64//PSMA2//PSMD12//FZD6//GSKIP//TBL1XR1//ZMYND8//LYPD6//CREBRF//DYNLL2//PPP3R1//YWHAG//AGO1//PRKACB//MDM4//POU4F1//RBBP8//RWDD3//BAG5//TMBIM6//SERINC3//DNAJB9//PPP1R15B//SESN1//CLOCK//PHF14//ARC//NR4A2//CASP2//TNFRSF12A//BCL2A1//ST18// |
| GO:0007154 | cell communication | Biological process | 414 | 6744 | 902 | 17653 | 1.20141973944812 | 7.85892255277645e-07 | 3.96640994212143e-05 | 6.10463699104183 | 0.458980044345898 | KCNH4//RANBP9//SPRED1//EREG//RASGEF1A//NRG1//IL2//MEF2A//MET//MAPK1//MAPK6//PSMA2//PSMD12//RAF1//RASA1//CCL2//TNF//SYNGAP1//RAPGEF2//GADD45A//CRKL//TAOK1//RAP1A//C1QTNF2//LPAR1//IGF1//NTF3//PIK3CB//PRKAA1//C5AR1//UBE2V1//DUSP1//PPP2CA//JUN//PMAIP1//BCL2A1//CBFB//EP300//PRKACB//SKP1//RPS6KA5//IRAK4//BAG6//CR2//SKAP2//TMED1//ADCYAP1//ADRB1//EDN1//EFNA1//EFNB2//GJA1//AR//CXCL9//PCDHB10//PTHLH//PCDH10//CCL8//CCL21//BMP3//KLF10//ZYX//GDF5//S1PR1//SPHK2//APCDD1//CCNY//HBP1//GRK6//HIC1//LRP6//STRN//WNT3//WNT7A//CXXC4//WNT10A//TNRC6B//AGO1//PPP3R1//FZD3//FZD6//CNIH1//CDS1//PTGES3//IQGAP2//ADCY3//RALBP1//RASSF8//CHRNB4//ARAP2//CLCN6//CNGA3//CNTFR//CREBL2//DPYSL2//DTNA//ELK3//EPS8//ESR1//CD2AP//GABRA5//APPL1//ATP2C1//GLRB//CNOT7//LRP12//HIVEP2//NR3C2//PPP1R12A//NR4A2//HPCAL4//GULP1//RASD1//KCNK10//CYTL1//NDFIP2//PPARG//PPP2R5E//PSD//MAVS//ARRDC3//MIER1//HIVEP3//SMOC1//SMOC2//SPOCK1//ZNF217//LRP8//GLRA3//SNX27//SYDE2//PPP1R1B//PPFIA1//OR6A2//PDE5A//GPRC5A//DLG5//PLAA//ARHGAP12//GTPBP1//CLOCK//FEZ2//ZNF516//CD69//ULK2//SNX17//KDR//ROR1//PTPRG//TXK//YES1//CD3E//CD8A//C8ORF44-SGK3//SGK2//AKAP11//DGKH//SGK3//NRBP1//ARHGEF4//PRKAR2B//SGK1//STK33//SS18//ANP32A//DGKE//RALGPS1//PALM2//BMPR2//FBXW7//ERRFI1//SOCS5//CNOT6L//MDM4//CNOT6//SOX4//E2F8//CASP2//TP63//PPP1R15B//GABARAPL1//CRHR1//EDN2//EDNRB//IGSF3//IFNG//TSPAN11//NCK1//EPS15//PIK3CA//SIRT1//SMURF1//TGFBR2//ACVR1//NMUR1//RAPGEF4//CHRM2//TAPT1//FRZB//GNAQ//OR4N4//GRM3//KCNK2//RHOB//RGS7BP//ITSN1//SOS2//CALM2//GPR55//FLNA//ANO1//HOMER1//NSG1//FMR1//GRIK2//KCNB1//NXPH3//SORCS1//SORCS2//SUSD5//DLL1//FOXA1//TMEM17//EVC//DZIP1//FOXF1//GLI3//ITGB8//CCM2//RCAN3//SAMD14//TBK1//SNIP1//COPS8//MDFIC//SOCS1//SOCS3//NMI//NEUROD1//RAB18//RGL1//RND3//ARHGAP1//RIN2//DOCK10//DIRAS3//USP28//RB1//G3BP2//ARHGAP6//PAK6//GJC1//NOVA1//SCN1B//SLC6A1//SNAP25//SYT1//BSN//PTEN//RIC3//ETV5//TRPC3//SLC25A6//MARCKS//GIT2//TIA1//SHCBP1//OTX2//RORA//GSKIP//INHBB//RRAGD//PLEKHF2//NR5A2//ADIPOR2//RWDD3//YWHAG//TOB1//LASP1//SH3BGRL//IGFBP3//PRR5L//TXLNA//IFNA1//OGN//VEGFB//F3//DBN1//TNFAIP3//TTK//PMEPA1//LDLRAD4//PPP1R16B//UNC5B//ZEB1//NREP//PLP2//CCND1//SDC1//SPPL3//DENND1B//TNFRSF21//SLK//GSC//SNX19//SULF1//SHISA2//NOG//NPNT//STRAP//LEMD3//FAM89B//BAMBI//PRDM16//CTDSPL2//PPARGC1B//CREBRF//SERP1//SLC9A6//ADNP//FOSL1//RHEBL1//RICTOR//FAM83D//TSC1//IQSEC2//FBXO8//AGFG1//ISL1//RAB12//RAB21//RAB30//RAB9B//RAB8B//RAB28//RAP2C//RAP1B//MID1//SEMA4C//INSIG2//UBA5//EDARADD//TNFRSF12A//ST18//ACSL1//IRF1//SESN1//PLEKHG4B//TEAD2//MOB1B//ROBO2//SLIT2//C2CD5//GSTA2//MSN//SYBU//GFPT1//DNAJB9//ASNA1//SEC61A2//BRWD1//CUL5//RASSF2//PHLDA3//ZFAND6//UBE2I//ZMYND11//HIPK3//ATXN7//PEX5L//TMEM64//NPTN//YTHDF2//DLK2//PPP2CB//ARID5B//GIGYF1//RIMS4//CPEB3//ARC//KCNJ10//RAB3GAP1//DHRS3//SYT6//PXK//GCSAML//PAX5//OTUD3//PRICKLE2//IRF2//FOXP1//CEBPB//TMBIM6//FIBIN//PLXNA4//POU4F1//ZMAT3//TBL1XR1//FASTK//SIVA1//PPM1F//ZMYND8//KCNJ3//FXR1//LYPD6//PAIP2//DYNLL2//RBBP8//BAG5//SERINC3//PFN2//PHF14//MYB//ENPP5// |
| GO:0009059 | macromolecule biosynthetic process | Biological process | 349 | 5526 | 902 | 17653 | 1.23602293692466 | 8.15561832036974e-07 | 4.08566271856745e-05 | 6.08854310722396 | 0.386917960088692 | SIRT1//IGF1//NFIA//NFIB//NFIC//RBBP8//RBMS1//NPAT//TSHZ1//ZBTB18//CEBPB//TCERG1//STRAP//KLF12//GSC//ARX//DNMT3B//EDN1//EDNRB//EFNA1//CC2D1B//EN1//EP300//ESR1//JAZF1//CPEB3//FOXF1//MYT1L//SIN3B//ZFPM2//DNAJB5//GLI3//CNOT7//HIC1//FOXA1//HSBP1//ID2//IFNG//IRF2//AR//ISL1//JARID2//JUN//MAF//MDM4//MEF2A//MYB//NR4A2//PAX5//KLF3//POU4F1//PPARG//ZBTB4//RB1//CCND1//RNF2//SARS//PRDM16//SMARCA2//SUV39H1//ZEB1//TFAP2A//TGIF1//KLF10//TNF//UBE2I//WT1//ZNF217//LRP8//BHLHE41//TBL1XR1//E2F8//ARID5B//BHLHE40//TP63//RUNX3//NOG//FOXP2//PHF14//GTF2E1//SEPSECS//SECISBP2L//GTF2H1//CREBRF//ELK3//ETV5//ATF5//FOXF2//FLI1//GTF2A1//HIVEP2//IRF1//MEOX2//MYBL1//MYCN//NEUROD1//NHLH2//OTX2//KLF13//PKNOX1//POU3F2//FOXJ2//HIVEP3//BMPR2//SOX4//SOX5//BTF3//TFE3//TXK//FOSL1//KLF7//CBFB//FUBP3//CCNT2//NMI//CLOCK//ZNF516//POLR3H//SRSF2//SRSF6//SLBP//HS3ST5//SDC1//HS3ST3A1//HS3ST1//PURA//GMNC//ESCO2//PPP2CA//BAHD1//H2AFZ//ZMYND11//PNRC1//ZNF526//CREBL2//ZNF800//E2F5//ZNF367//ARID2//MED19//ZFP30//KDM2A//MYCBP2//PHF8//FLII//ADNP//ZMYND8//CNOT6L//ZNF521//EPC2//AGO1//HBP1//FOXP1//ZBTB11//ATAD2//MDFIC//HMGB3//HOXA3//HOXD1//ZNF680//ZNF662//NEUROG1//NFE2//NPAS2//ASCC1//IER5//COMMD10//ARID4B//BRWD1//BNC2//MED9//ZNF532//PRKAA1//TRERF1//MAPK1//SLC2A4RG//CCNL1//PHTF2//CNOT6//ZBTB26//MIER1//ATXN1//ATXN7//SMARCD2//SS18//MED22//ZNF3//ZNF708//ZNF131//ZNF227//ZNF655//ZSCAN5A//SAP30L//ANP32A//PPP1R1B//PCGF5//LCOR//ST18//HIPK3//PPARGC1B//ELAVL2//ELL2//NR5A2//NRBF2//AFF1//MLLT6//OGG1//PHF20L1//PAK6//RORA//BTG1//UBE2V1//CSDE1//SNIP1//TEAD2//RPS6KA5//EMX2//ETS1//BOLA3//NR3C2//RFX7//TRAK2//BDP1//NRBP1//CPEB2//CPEB1//DDX3Y//GTPBP1//DDX6//TSC1//KBTBD8//FUT9//B3GALNT2//FUT2//FUT5//SERP1//ST8SIA5//GFPT1//KCNE1//CCDC126//ALG2//EOGT//GCNT4//VEGFB//WDR45B//ALPI//RECK//PYURF//PIGP//PIGA//PTGES3//G3BP2//EREG//CCL21//FMR1//PRR16//FXR1//FLNA//BAMBI//LRP6//WNT7A//ACVR1//DNAJB6//NRG1//RASD1//BTAF1//PPM1F//TOB1//CELF1//NANOS1//TIA1//MEX3B//OGN//PRELP//CHST1//ZDHHC22//ZDHHC7//ZDHHC18//ST3GAL5//MCFD2//ABHD17C//SLC35D1//DSEL//SMURF1//BMP3//GDF5//SULF1//LEMD3//CTDSPL2//RWDD3//TNFAIP3//DCP2//CCT6A//FBXW7//INSIG2//TNRC6B//NCK1//ERRFI1//MYOG//C5AR1//INTS8//NABP1//EGLN1//FZD6//CD3E//TBK1//TMEFF2//C1QTNF2//ADCYAP1//S1PR1//KPNA6//DLL1//IL2//MET//PPP1R12A//ARMCX3//CYTL1//PPP3R1//MAVS//RAF1//YES1//PAIP2//BOLL//INHBB//C8ORF44-SGK3//SGK3//SGK1//PTEN//RHEBL1//ROR1//IRAK4//KDR//CCL2//NUP54//YTHDF2//QKI//EGLN3//PSMA2//PSMD12//ATG16L1//RAB3GAP1//RAP2C//HSBP1L1//PPP1R15B//TMBIM6//DUSP1//GJA1//KCNK2//NPNT// |
| GO:0001934 | positive regulation of protein phosphorylation | Biological process | 86 | 990 | 902 | 17653 | 1.70010302582365 | 8.39348827386145e-07 | 4.17390891147978e-05 | 6.07605751196665 | 0.0953436807095344 | GADD45A//TNF//CRKL//TAOK1//RAF1//RAP1A//C1QTNF2//LPAR1//NRG1//IGF1//NTF3//PIK3CB//PRKAA1//MAPK1//C5AR1//UBE2V1//EREG//FBXW7//COPS8//MDFIC//SOCS1//CALM2//INHBB//BMP3//BMPR2//TTK//GDF5//ACVR1//RAP2C//RASSF2//ADNP//PIK3CA//PAK6//TGFBR2//SLK//RICTOR//MID1//SEMA4C//FMR1//RPS6KA5//CREBL2//TBK1//PFN2//IFNA1//IFNG//ADCY3//PRKACB//PRKAR2B//IL2//ISL1//SOCS3//EDN1//PDE5A//EFNA1//IGFBP3//AR//KDR//MET//ROR1//CCNY//CCNL1//CCND1//CCND2//CCNT2//ADCYAP1//CACUL1//MMD//CCL21//RAPGEF2//WNT7A//YES1//LRP8//CD3E//NPNT//NPTN//JUN//PTEN//CCL2//CCL8//GPR55//ACSL1//PPP2CA//SIRT1//EDNRB//MAVS//PRR5L// |
| GO:0009719 | response to endogenous stimulus | Biological process | 125 | 1606 | 902 | 17653 | 1.52326848044887 | 1.00356419787702e-06 | 4.95409100017685e-05 | 5.99845484052382 | 0.138580931263858 | SIRT1//JUN//SMURF1//TGFBR2//KLF10//ZYX//GDF5//ACVR1//CHRM2//GNAQ//APPL1//SPRED1//MAPK1//TIA1//SHCBP1//STEAP2//TUB//NR5A2//PPARG//ADIPOR2//ZEB1//NREP//CEBPB//CPEB3//SESN1//RRAGD//CPEB1//TNF//SOCS1//TOB1//BMP3//BMPR2//NOG//NPNT//STRAP//LEMD3//FAM89B//BAMBI//PMEPA1//PRDM16//LDLRAD4//SULF1//CTDSPL2//ESR1//PPARGC1B//AR//RB1//CNGA3//DNMT3B//DUSP1//ETS1//FOXA1//OGG1//PTEN//RBBP8//CCND1//SLC6A1//WNT7A//FOSL1//IGF1//INSIG2//SRSF6//TSC1//CPEB2//INHBB//RAB8B//ERRFI1//YWHAG//C2CD5//ROBO2//SLIT2//UBA5//CBFB//ISL1//TP63//ACSL1//EDN1//FOXP2//CCL21//PIK3CA//YES1//NTF3//RAP1A//RAPGEF2//OTX2//SHISA2//NR3C2//NR4A2//RORA//EREG//GJA1//LRP6//BTG1//CCL2//NPTN//NCK1//SOCS3//SDC1//EP300//FOXP1//KCNE1//RAP1B//WT1//CRHR1//ADCY3//PRKACB//PRKAR2B//PRKAA1//ADCYAP1//H2AFZ//MYOG//POU4F1//MSN//LDLR//SOCS5//FIBIN//RUNX3//SOX5//WNT10A//CREBRF//KLF3//STMN2//DYNC1LI2//EDNRB//CLOCK// |
| GO:0048514 | blood vessel morphogenesis | Biological process | 63 | 656 | 902 | 17653 | 1.87952754042507 | 1.03590813054678e-06 | 5.07670049774484e-05 | 5.98467875826427 | 0.0698447893569845 | S1PR1//EFNA1//ELK3//EREG//UNC5B//SIRT1//LEMD3//CLIC4//HOXA3//JUN//KDR//RHOB//MEOX2//TNFRSF12A//PIK3CA//PKNOX1//PTEN//RORA//CCL2//VEGFB//WNT7A//EDN1//PPP3R1//TGFBR2//ACVR1//GJC1//FOXF1//ZFPM2//RASA1//WT1//CCM2//TEAD2//QKI//E2F8//EFNB2//SLIT2//SPRED1//SULF1//AGO1//PPARG//FOXJ2//SARS//SOX4//ETS1//EGLN1//VASH2//F3//ISL1//ITGB8//BTG1//C5AR1//ADAM12//PIK3CB//ARID2//ADIPOR2//TNFAIP3//BMPR2//NOG//DLL1//PPP1R16B//RAP1A//RAPGEF2//GJA1// |
| GO:0060485 | mesenchyme development | Biological process | 33 | 258 | 902 | 17653 | 2.50326148610323 | 1.13698538240149e-06 | 5.51419134274922e-05 | 5.94424511876273 | 0.0365853658536585 | EDNRB//ISL1//SEMA4C//ACVR1//FOXF2//PPP3R1//FAM83D//DLG5//NOG//FOXF1//BMPR2//EFNA1//TGFBR2//ZFPM2//BAMBI//STRAP//FOXA1//PPP2CA//PTEN//LDLRAD4//LRP6//KBTBD8//RDH10//EDN1//TAPT1//NRG1//FRZB//WNT10A//GSC//TEAD2//MAPK1//WT1//BNC2// |
| GO:0043412 | macromolecule modification | Biological process | 286 | 4389 | 902 | 17653 | 1.27530022395234 | 1.14148571341844e-06 | 5.51419134274922e-05 | 5.94252951967297 | 0.317073170731707 | GADD45A//GTF2H1//PTEN//CCNT2//DIRAS3//RANBP9//SPRED1//EREG//RASGEF1A//NRG1//IL2//MEF2A//MET//MAPK1//MAPK6//PSMA2//PSMD12//RAF1//RASA1//CCL2//TNF//SYNGAP1//RAPGEF2//CRKL//TAOK1//RAP1A//C1QTNF2//LPAR1//IGF1//NTF3//PIK3CB//PRKAA1//C5AR1//UBE2V1//DUSP1//PPP2CA//FBXO41//RNF145//ZNRF2//RNF167//MYLIP//ANKIB1//UBE2W//FBXW7//SMURF1//HECW2//KLHL42//SKP1//THOP1//LONRF3//FBXO30//CBFB//UBE3B//LONRF1//FBXO44//COPS8//NKTR//SOCS1//IGFBP3//LRP6//PRR5L//PPP1R15B//SLIT2//EDNRB//EFNA1//FMR1//SIRT1//NPTN//IFNG//KDR//MAVS//CCND1//CCND2//ABHD17C//ERRFI1//GPRC5A//SOCS5//ZER1//CACUL1//RNF38//MARCH10//PDZRN3//MYCBP2//FBXO33//KLHL3//RNF11//KLHL20//SPOPL//TRIM23//MDM4//DCAF8//CBLL1//CUL5//KBTBD8//SOCS3//DNMT3B//MTRR//ASCC1//THG1L//PPP4R2//SERP1//RIMKLB//TGM3//UBE2I//YES1//C8ORF44-SGK3//SGK2//HIPK3//FASTK//PDIK1L//CDK19//MORC3//MMD//SGK3//PAN3//GRK6//TBK1//NRBP1//CDK17//PIK3CA//PRKACB//CCL8//SGK1//CDK15//TGFBR2//TXK//MEX3B//RUNX3//ACVR1//RPS6KA5//RASSF2//GNAQ//NCK1//GSKIP//RB1//YWHAG//PPM1F//PTPN21//PPTC7//PPP1R12A//CTDSPL2//PPP2CB//PPP3R1//EP300//CLOCK//HS3ST5//PCMTD1//BTG1//FUT9//B3GALNT2//FUT2//FUT5//ST8SIA5//GFPT1//KCNE1//CCDC126//ALG2//EOGT//GCNT4//TRAK2//VEGFB//WDR45B//ALPI//RECK//PYURF//PIGP//PIGA//IRAK4//MDFIC//CALM2//ADNP//INHBB//BMP3//BMPR2//TTK//GDF5//EPC2//SIN3B//ARID4B//MIER1//SAP30L//TBL1XR1//ARID5B//ATXN7//MBD6//ESR1//OTUD3//AR//USP27X//USP28//TNFAIP3//USP38//WDR20//NUP54//CEP41//RICTOR//STK33//ROR1//EGLN3//ZDHHC22//ZDHHC7//ZDHHC18//ST3GAL5//MCFD2//GPHN//BAG6//CRTAP//EGLN1//DCUN1D4//KCNH4//PAK6//SLK//JARID2//SOX4//BAG5//NDFIP2//MOB1B//JUN//RAP2C//ARPP19//PPP1R1B//MID1//SEMA4C//CREBL2//PFN2//IFNA1//PHF8//RWDD3//ADCY3//PRKAR2B//ESCO2//PRDM16//ISL1//PPP1R16B//SPPL3//CNEP1R1//PTPRG//PTP4A1//PTP4A2//CDC14A//RNF2//SUV39H1//PCGF5//CNOT7//TDRD5//EDN1//PDE5A//RCAN3//UBXN2B//PPP2R5E//TSC1//CKAP4//CALU//OGG1//SMUG1//CCNY//CCNL1//ADCYAP1//CCL21//GRIK2//ZMYND11//WNT7A//ULK2//LRP8//CD3E//CCM2//ARRDC3//MYB//PAX5//STRAP//PMEPA1//LDLRAD4//NOG//ATG16L1//FAM83D//NPNT//GPR55//OTUD1//KDM2A//UBA5//ACSL1//GLRX//PLAA//NMI//RAB3GAP1//ZMYND8// |
| GO:0010033 | response to organic substance | Biological process | 220 | 3218 | 902 | 17653 | 1.33797692855735 | 1.23744321451598e-06 | 5.93533933317133e-05 | 5.90747472143537 | 0.24390243902439 | CBFB//HPRT1//NR4A2//PPP1R1B//TNFAIP3//DNAJB5//JKAMP//HSPA13//TMBIM6//EP300//SIRT1//JUN//SMURF1//TGFBR2//KLF10//ZYX//GDF5//ACVR1//CHRM2//GNAQ//APPL1//HOMER1//SPRED1//MAPK1//TIA1//SHCBP1//STEAP2//TUB//ADNP//RAP1B//SLC6A1//GJA1//IGF1//NEUROD1//PTEN//NR5A2//PPARG//ADIPOR2//AGO1//SNIP1//MGST1//CCND1//TNF//EDNRB//ACSL1//ANXA7//ADSS//ZEB1//NREP//CNTFR//CRKL//S1PR1//EDN2//TXLNA//EREG//F3//IFNA1//IL2//IRAK4//PIK3CA//PIK3CB//PLP2//RORA//CCL2//SDC1//LRP8//SOCS1//SOCS3//SOCS5//CEBPB//CPEB3//SESN1//RRAGD//CPEB1//TSN//DNAJB9//BAG6//TOB1//BMP3//BMPR2//NOG//NPNT//STRAP//LEMD3//FAM89B//BAMBI//PMEPA1//PRDM16//LDLRAD4//SULF1//CTDSPL2//ESR1//PPARGC1B//AR//RB1//CREBRF//SERP1//DNMT3B//PRKAA1//CNGA3//DUSP1//ETS1//FOXA1//OGG1//RBBP8//WNT7A//C5AR1//EDN1//FOXP1//TNFRSF21//CXCL9//FOSL1//INSIG2//SRSF6//TSC1//CPEB2//INHBB//RAB8B//ERRFI1//YWHAG//C2CD5//ROBO2//SLIT2//UBA5//ISL1//TP63//SRSF2//TRPC3//EDARADD//TNFRSF12A//PSMA2//PSMD12//ST18//FOXP2//VPS4B//CCL21//KLHL20//IRF1//GSTA2//IFNG//MSN//RAF1//ANO1//SYBU//KDR//YES1//GFPT1//ASNA1//SEC61A2//BRWD1//NTF3//RAP1A//RAPGEF2//OTX2//SHISA2//GLRB//GLRA3//PMAIP1//NR3C2//LRP6//BTG1//ADCYAP1//EPS8//NPTN//NCK1//SLC9A6//IRF2//MID1//NMI//TXK//CNOT7//MAVS//CCL8//SKP1//UBE2V1//RPS6KA5//ACSL4//UBE2W//CDK19//PLAA//MYB//MYOG//WNT3//TEAD2//POU3F2//MAPK6//KCNE1//WT1//KCNB1//SOX4//FOXF1//TBK1//POU4F1//RAB12//VAMP3//ZFAND6//TGIF1//CRHR1//ADCY3//PRKACB//PRKAR2B//H2AFZ//LDLR//FIBIN//RUNX3//SOX5//WNT10A//FMR1//DLL1//KLF3//MYCN//PPP1R15B//LPAR1//STMN2//DYNC1LI2//JARID2//PIGA//ARID5B//CLOCK//PPP2CA// |
| GO:0045597 | positive regulation of cell differentiation | Biological process | 81 | 926 | 902 | 17653 | 1.7119300522477 | 1.35959498187359e-06 | 6.47531046648668e-05 | 5.86659044702266 | 0.08980044345898 | GLI3//FZD3//BAMBI//ISL1//TGFBR2//MYOG//STMN2//ADCYAP1//NPTN//NCK1//SERPINI1//RAP1A//SCN1B//RAPGEF2//SOX5//GDF5//IFNG//IL2//SOCS1//CEBPB//CREBL2//TMEM64//FRZB//ID2//PPARG//BTG1//MYB//SOCS5//ETS1//RB1//JUN//IGFBP3//CXCL9//DNMT3B//ETV5//MMD//FOXA1//NEUROD1//NEUROG1//PTEN//ZEB1//NPNT//GJA1//IGF1//BMPR2//TP63//ACVR1//PPARGC1B//POU4F1//KLF10//TNF//ADNP//NRG1//TNFRSF12A//WNT3//ROBO2//PLXNA4//SLIT2//RAB21//MEF2A//BNIP2//SIRT1//PDE5A//EDN1//LPAR1//CPEB3//FMR1//ZMYND8//MAPK6//DLG5//LRP8//PRDM16//CRKL//FLNA//SMURF1//SYT1//PLAA//EFNB2//KDR//AR//SOCS3// |
| GO:0007165 | signal transduction | Biological process | 384 | 6210 | 902 | 17653 | 1.21018456034363 | 1.43182435940556e-06 | 6.77162807179007e-05 | 5.84411025327073 | 0.425720620842572 | KCNH4//RANBP9//SPRED1//EREG//RASGEF1A//NRG1//IL2//MEF2A//MET//MAPK1//MAPK6//PSMA2//PSMD12//RAF1//RASA1//CCL2//TNF//SYNGAP1//RAPGEF2//GADD45A//CRKL//TAOK1//RAP1A//C1QTNF2//LPAR1//IGF1//NTF3//PIK3CB//PRKAA1//C5AR1//UBE2V1//DUSP1//PPP2CA//JUN//PMAIP1//BCL2A1//CBFB//EP300//PRKACB//SKP1//RPS6KA5//IRAK4//BAG6//CR2//SKAP2//S1PR1//SPHK2//APCDD1//CCNY//HBP1//GRK6//HIC1//LRP6//STRN//WNT3//WNT7A//CXXC4//WNT10A//TNRC6B//AGO1//PPP3R1//FZD3//FZD6//KDR//ROR1//PTPRG//TXK//YES1//CD3E//CD8A//C8ORF44-SGK3//SGK2//AKAP11//DGKH//SGK3//NRBP1//ARHGEF4//PRKAR2B//SGK1//STK33//SS18//ANP32A//PPP1R1B//DGKE//DLG5//RALGPS1//PALM2//BMPR2//FBXW7//ERRFI1//GPRC5A//SOCS5//CNOT6L//CNOT7//MDM4//CNOT6//SOX4//E2F8//CASP2//TP63//PPP1R15B//CRHR1//EDN1//EDN2//EDNRB//IGSF3//IFNG//TSPAN11//NCK1//EPS8//EPS15//PIK3CA//SIRT1//SMURF1//TGFBR2//KLF10//ZYX//GDF5//ACVR1//NMUR1//ADCY3//RAPGEF4//CHRM2//ADCYAP1//ADRB1//TAPT1//FRZB//GNAQ//OR4N4//GRM3//KCNK2//RHOB//RGS7BP//CXCL9//RASD1//PPARG//PTHLH//CCL8//CCL21//ITSN1//SOS2//CALM2//OR6A2//GPR55//FLNA//ESR1//ANO1//HOMER1//NSG1//GABRA5//FMR1//GRIK2//KCNB1//NXPH3//SORCS1//GLRB//SORCS2//GLRA3//SUSD5//DLL1//FOXA1//TMEM17//EVC//DZIP1//FOXF1//GLI3//ITGB8//CCM2//RCAN3//SAMD14//TBK1//SNIP1//COPS8//MDFIC//SOCS1//SOCS3//NMI//NEUROD1//RALBP1//RAB18//RGL1//RND3//ARHGAP1//RIN2//DOCK10//DIRAS3//USP28//RB1//G3BP2//ARHGAP6//PAK6//CDS1//TRPC3//GIT2//APPL1//TIA1//SHCBP1//OTX2//RORA//GSKIP//NR5A2//ADIPOR2//RWDD3//YWHAG//TOB1//LASP1//SH3BGRL//IGFBP3//PRR5L//TXLNA//IFNA1//INHBB//OGN//BMP3//VEGFB//F3//TNFAIP3//TTK//PMEPA1//LDLRAD4//PPP1R16B//PTEN//UNC5B//ZEB1//NREP//CNTFR//PLP2//CCND1//SDC1//LRP8//SPPL3//DENND1B//TNFRSF21//SLK//GSC//SULF1//SHISA2//NOG//NPNT//STRAP//LEMD3//FAM89B//BAMBI//PRDM16//CTDSPL2//PPARGC1B//AR//NR4A2//CREBRF//SERP1//SLC9A6//RHEBL1//RICTOR//FAM83D//TSC1//RRAGD//ARAP2//IQSEC2//FBXO8//AGFG1//PSD//RAB12//RAB21//RAB30//RAB9B//RAB8B//RAB28//RAP2C//RAP1B//MID1//SEMA4C//INSIG2//UBA5//ISL1//EDARADD//TNFRSF12A//ST18//ACSL1//IRF1//PLEKHG4B//TEAD2//MOB1B//ROBO2//SLIT2//C2CD5//GSTA2//MSN//GFPT1//DNAJB9//ASNA1//SEC61A2//BRWD1//CUL5//RASSF2//PHLDA3//ZFAND6//GJA1//ATP2C1//NDFIP2//MAVS//MIER1//UBE2I//ZMYND11//NR3C2//PDE5A//EFNA1//HIPK3//ATXN7//PEX5L//TMEM64//NPTN//YTHDF2//DLK2//PPP2CB//ARID5B//GIGYF1//EFNB2//DHRS3//GCSAML//PAX5//SYDE2//ARHGAP12//CD2AP//OTUD3//PRICKLE2//CHRNB4//RAB3GAP1//IRF2//FOXP1//CEBPB//TMBIM6//IQGAP2//FIBIN//PLXNA4//ARRDC3//POU4F1//ZMAT3//TBL1XR1//FASTK//SIVA1//PPM1F//ZMYND8//BSN//LYPD6//DYNLL2//RBBP8//BAG5//SERINC3//SESN1//CLOCK//PHF14//ARC//CNIH1//PTGES3//TMED1//RASSF8//CLCN6//CNGA3//CREBL2//DPYSL2//DTNA//ELK3//LRP12//HIVEP2//PPP1R12A//HPCAL4//GULP1//KCNK10//CYTL1//PPP2R5E//HIVEP3//SMOC1//SMOC2//SPOCK1//ZNF217//SNX27//PPFIA1//PLAA//GTPBP1//FEZ2//ZNF516//CD69//ULK2//SNX17// |
| GO:0006468 | protein phosphorylation | Biological process | 145 | 1946 | 902 | 17653 | 1.45826734241368 | 1.49302790399882e-06 | 6.96280441149136e-05 | 5.82593207543675 | 0.160753880266075 | GADD45A//GTF2H1//PTEN//CCNT2//DIRAS3//RANBP9//SPRED1//EREG//RASGEF1A//NRG1//IL2//MEF2A//MET//MAPK1//MAPK6//PSMA2//PSMD12//RAF1//RASA1//CCL2//TNF//SYNGAP1//RAPGEF2//CRKL//TAOK1//RAP1A//C1QTNF2//LPAR1//IGF1//NTF3//PIK3CB//PRKAA1//C5AR1//UBE2V1//DUSP1//PPP2CA//SOCS1//IGFBP3//LRP6//PRR5L//PPP1R15B//SLIT2//EDNRB//EFNA1//FMR1//SIRT1//NPTN//IFNG//KDR//MAVS//CCND1//CCND2//FBXW7//ERRFI1//GPRC5A//SOCS5//GNAQ//NCK1//GSKIP//RB1//YWHAG//SOCS3//PPM1F//TGFBR2//COPS8//IRAK4//MDFIC//CALM2//INHBB//BMP3//BMPR2//TTK//GDF5//ACVR1//RPS6KA5//C8ORF44-SGK3//SGK2//HIPK3//MORC3//SGK3//RICTOR//TBK1//SGK1//STK33//ROR1//KCNH4//PAK6//SLK//MOB1B//JUN//RAP2C//RASSF2//ADNP//PIK3CA//MID1//SEMA4C//CREBL2//PFN2//IFNA1//ADCY3//PRKACB//PRKAR2B//TXK//YES1//CNOT7//ISL1//EDN1//PDE5A//AR//PPP1R1B//TNFAIP3//CCNY//CCNL1//TSC1//ADCYAP1//CACUL1//MMD//CCL21//GRIK2//ZMYND11//WNT7A//MEX3B//ULK2//LRP8//CD3E//SKP1//CCM2//STRAP//PMEPA1//LDLRAD4//NOG//FAM83D//NPNT//CCL8//GPR55//ACSL1//FASTK//PDIK1L//CDK19//PAN3//GRK6//NRBP1//CDK17//CDK15//RUNX3// |
| GO:0032268 | regulation of cellular protein metabolic process | Biological process | 195 | 2792 | 902 | 17653 | 1.36688249290021 | 1.50284499146892e-06 | 6.96280441149136e-05 | 5.8230858117094 | 0.216186252771619 | GADD45A//GTF2H1//PTEN//CCNT2//DIRAS3//TNF//CRKL//TAOK1//RAF1//RAP1A//C1QTNF2//LPAR1//NRG1//IGF1//NTF3//PIK3CB//PRKAA1//MAPK1//C5AR1//UBE2V1//SPRED1//DUSP1//PPP2CA//SEPSECS//SECISBP2L//SOCS1//IGFBP3//IL2//LRP6//PRR5L//PPP1R15B//SLIT2//EDNRB//EFNA1//FMR1//SIRT1//NPTN//IFNG//KDR//MAVS//CCND1//CCND2//EREG//FBXW7//ERRFI1//GPRC5A//SOCS5//DDX6//TSC1//KBTBD8//GNAQ//NCK1//GSKIP//RB1//YWHAG//SOCS3//PPM1F//EGLN3//F3//PMAIP1//PPARG//CASP2//COPS8//MDFIC//AGO1//SNIP1//PI15//CALM2//ADNP//INHBB//BMP3//BMPR2//TTK//GDF5//ACVR1//SERPINI1//SERPINB13//SPOCK1//RECK//CPEB3//SERP1//PRR16//SOX4//FXR1//TOB1//CELF1//CNOT7//NANOS1//PURA//TIA1//MEX3B//JARID2//SPOPL//TNFAIP3//BAG5//NDFIP2//MOB1B//JUN//RAP2C//RASSF2//PIK3CA//PAK6//TGFBR2//SLK//RICTOR//EDN1//WNT7A//BAG6//ANKIB1//LONRF3//LONRF1//ARPP19//PPP1R1B//MYLIP//MID1//SEMA4C//RPS6KA5//CREBL2//TBK1//PFN2//IFNA1//RWDD3//ADCY3//PRKACB//PRKAR2B//ISL1//CNOT6//TNRC6B//PPP1R16B//SPPL3//CNEP1R1//PPP1R12A//DNAJB6//ARL6IP1//PDE5A//SYNGAP1//AR//MET//ROR1//HIPK3//RCAN3//UBXN2B//PPP4R2//PPP2R5E//CCNY//CCNL1//ADCYAP1//CACUL1//MMD//CCL21//RAPGEF2//PAIP2//BOLL//GRIK2//ZMYND11//YES1//LRP8//CD3E//SH3D19//ZER1//DCUN1D4//ARRDC3//DNMT3B//MYB//PAX5//STRAP//PMEPA1//LDLRAD4//NOG//NUP54//CNOT6L//YTHDF2//QKI//FAM83D//RANBP9//NPNT//CCL2//CCL8//GPR55//ACSL1//EP300//CAST//PLAA//CPEB2//CRTAP//NMI//RAB3GAP1//MSN//UBE2I//ESR1//MYCN//LDLR//SMURF1//CPEB1//ST18// |
| GO:0016070 | RNA metabolic process | Biological process | 309 | 4821 | 902 | 17653 | 1.25439216178664 | 1.50313388152852e-06 | 6.96280441149136e-05 | 5.82300233583385 | 0.342572062084257 | NPAT//TSHZ1//ZBTB18//CEBPB//TCERG1//STRAP//KLF12//GSC//ARX//DNMT3B//EDN1//EDNRB//EFNA1//CC2D1B//EN1//EP300//ESR1//JAZF1//CPEB3//FOXF1//MYT1L//SIN3B//SIRT1//ZFPM2//DNAJB5//GLI3//CNOT7//HIC1//FOXA1//HSBP1//ID2//IFNG//IRF2//AR//ISL1//JARID2//JUN//MAF//MDM4//MEF2A//MYB//NFIA//NFIB//NFIC//NR4A2//PAX5//KLF3//POU4F1//PPARG//ZBTB4//RB1//RBBP8//CCND1//RNF2//SARS//PRDM16//SMARCA2//SUV39H1//ZEB1//TFAP2A//TGIF1//KLF10//TNF//UBE2I//WT1//ZNF217//LRP8//BHLHE41//TBL1XR1//E2F8//ARID5B//BHLHE40//TP63//RUNX3//NOG//FOXP2//PHF14//PNRC1//DCP2//PPP2CA//CNOT6L//PAN3//CNOT6//PATL1//SRSF6//MBNL2//RBFOX3//FMR1//MBNL1//RBM25//SRSF2//ELAVL2//NOVA1//PCBP2//RBM41//RRP15//AGO1//GTF2E1//RPP14//RPP25//GTF2H1//CREBRF//ELK3//ETV5//ATF5//FOXF2//FLI1//GTF2A1//HIVEP2//IRF1//MEOX2//MYBL1//MYCN//NEUROD1//NHLH2//OTX2//KLF13//PKNOX1//POU3F2//FOXJ2//HIVEP3//BMPR2//SOX4//SOX5//BTF3//TFE3//TXK//FOSL1//KLF7//CBFB//FUBP3//CCNT2//NMI//CLOCK//ZNF516//POLR3H//SLBP//BAHD1//H2AFZ//ZMYND11//ZNF526//CREBL2//ZNF800//E2F5//ZNF367//ARID2//MED19//ZFP30//KDM2A//MYCBP2//PHF8//FLII//ADNP//ZMYND8//ZNF521//EPC2//HBP1//FOXP1//ZBTB11//ATAD2//MDFIC//HMGB3//HOXA3//HOXD1//ZNF680//ZNF662//NEUROG1//NFE2//NPAS2//ASCC1//IER5//COMMD10//ARID4B//BRWD1//BNC2//MED9//ZNF532//PRKAA1//TRERF1//MAPK1//SLC2A4RG//CCNL1//PHTF2//ZBTB26//MIER1//PURA//ATXN1//ATXN7//SMARCD2//SS18//MED22//ZNF3//ZNF708//ZNF131//ZNF227//ZNF655//ZSCAN5A//SAP30L//ANP32A//PPP1R1B//PCGF5//LCOR//ST18//HIPK3//PPARGC1B//ELL2//NR5A2//NRBF2//AFF1//MLLT6//OGG1//PHF20L1//PAK6//RORA//BTG1//UBE2V1//CSDE1//SNIP1//TEAD2//RPS6KA5//EMX2//ETS1//BOLA3//NR3C2//RFX7//TRAK2//BDP1//NRBP1//CELF1//GRSF1//RBMS1//RTCA//PPP4R2//CPEB1//QKI//THG1L//G3BP2//EREG//DDX6//DDX3Y//INTS8//FLNA//BAMBI//IGF1//LRP6//WNT7A//ACVR1//DNAJB6//NRG1//RASD1//BTAF1//PPM1F//TSN//TOB1//SMURF1//BMP3//GDF5//SULF1//LEMD3//CTDSPL2//RWDD3//TNFAIP3//INSIG2//MYOG//C5AR1//NABP1//EGLN1//FZD6//FASTK//YTHDF2//PSMA2//PSMD12//FXR1//ADCYAP1//S1PR1//KPNA6//DLL1//TBK1//IL2//MET//PPP1R12A//NCK1//ARMCX3//CYTL1//PPP3R1//MAVS//RAF1//YES1//TIA1//C8ORF44-SGK3//SGK3//SGK1//PTEN//RHEBL1//ROR1//IRAK4//RASA1//TNRC6B//TMBIM6//PRR5L//GTPBP1//EGLN3//BOLL//SEPSECS//NANOS1//RAP2C//HSBP1L1//FBXW7//NPNT// |
| GO:0051130 | positive regulation of cellular component organization | Biological process | 100 | 1221 | 902 | 17653 | 1.60286268933719 | 1.54000978407565e-06 | 7.08509263245144e-05 | 5.81247651997096 | 0.110864745011086 | FMR1//NTF3//GTF2H1//CNOT6L//PAN3//CNOT6//PPARGC1B//STMN2//ADCYAP1//NPTN//NCK1//SERPINI1//RAP1A//SCN1B//RAPGEF2//CFL2//WDR1//RICTOR//PFN2//CCL21//MET//CLIP1//NAV3//IFNG//TNF//SYT1//C2CD5//RAB8B//NEUROG1//SIRT1//MIER1//MAPK1//CCT6A//PMAIP1//RPS6KA5//IQGAP2//ARFIP1//ISL1//TAPT1//AR//ADNP//NRG1//TNFRSF12A//DLL1//LDLR//CBLL1//EDN1//EREG//IGF1//RB1//ESR1//RAB21//WNT3//BMPR2//SIRPA//TUB//ROBO2//PLXNA4//SLIT2//ZMYND8//GPM6A//LPAR1//TSC1//PPM1F//DNMT3B//MYB//JARID2//KDR//SLITRK3//WNT7A//DLG5//PPARG//CPEB3//IL2//MAPK6//LRP8//SMURF1//MSN//MIEF1//CRKL//FLNA//EPS8//DYNLL2//PPP3R1//YWHAG//TP63//CXCL9//MYOG//SEPT9//RAB3GAP1//SDC1//VPS4B//PLAA//NMUR1//FBXW7//PRKAA1//SNIP1//JUN//FOSL1//CCL2// |
| GO:0120036 | plasma membrane bounded cell projection organization | Biological process | 114 | 1444 | 902 | 17653 | 1.54507527132688 | 1.6264705237568e-06 | 7.43231091362651e-05 | 5.78875380295438 | 0.126385809312639 | CD2AP//NCK1//SLITRK3//OGN//POU4F1//PRELP//WNT7A//SLIT2//RANBP9//ARX//DPYSL2//EFNA1//EFNB2//GLI3//OTX2//PIK3CA//PIK3CB//ENAH//MAPK1//ROBO2//SCN1B//WNT3//KLF7//RPS6KA5//NRXN3//FEZ2//NOG//SEPT9//DYNLL2//TMEM17//DZIP1//FLNA//BBS9//GMNC//CDC14A//C5ORF30//CEP41//PTEN//SNAP25//STMN2//ADCYAP1//NPTN//SERPINI1//RAP1A//RAPGEF2//LPAR1//MYLIP//PTPRG//SPOCK1//TSC1//BAG5//STRN//SYNGAP1//CD3E//PLXNA4//NR4A2//NFIB//WASF3//S1PR1//ARHGEF4//RAB21//ISL1//JUN//NREP//CAMSAP1//SAMD14//GPM6A//LRP12//PSD//RB1//CCL21//UNC5B//ZMYND10//FZD3//TAPT1//ADNP//NRG1//TNFRSF12A//ULK2//SLC9A6//GJA1//NTF3//SGK1//HPRT1//MEF2A//TRAK2//FMR1//HECW2//FXR1//BMPR2//SEMA4C//POU3F2//DBN1//CAMSAP2//PPP1R16B//ZMYND8//ACSL4//ARC//DOCK10//CPEB3//IL2//MAPK6//DLG5//LRP8//SNX2//NEDD1//PRKAR2B//YWHAG//PFN2//EPS8//SMURF1//SYT1//PLAA//NAV3// |
| GO:0030030 | cell projection organization | Biological process | 116 | 1477 | 902 | 17653 | 1.53705524622182 | 1.68060252519477e-06 | 7.6281307905317e-05 | 5.77453498824172 | 0.12860310421286 | CD2AP//NCK1//SLITRK3//OGN//POU4F1//PRELP//WNT7A//SLIT2//RANBP9//ARX//DPYSL2//EFNA1//EFNB2//GLI3//OTX2//PIK3CA//PIK3CB//ENAH//MAPK1//ROBO2//SCN1B//WNT3//KLF7//RPS6KA5//NRXN3//FEZ2//NOG//SEPT9//DYNLL2//TMEM17//DZIP1//FLNA//BBS9//GMNC//CDC14A//C5ORF30//CEP41//PTEN//SNAP25//STMN2//ADCYAP1//NPTN//SERPINI1//RAP1A//RAPGEF2//LPAR1//MYLIP//PTPRG//SPOCK1//TSC1//BAG5//STRN//SYNGAP1//CD3E//PLXNA4//NR4A2//NFIB//WASF3//S1PR1//ARHGEF4//RAB21//ISL1//JUN//NREP//CAMSAP1//SAMD14//GPM6A//LRP12//PSD//RB1//CCL21//RAB8B//UNC5B//ZMYND10//FZD3//TAPT1//ADNP//NRG1//TNFRSF12A//ULK2//SLC9A6//GJA1//NTF3//SGK1//HPRT1//MEF2A//TRAK2//FMR1//HECW2//FXR1//BMPR2//SEMA4C//POU3F2//DBN1//CAMSAP2//PPP1R16B//ZMYND8//ACSL4//ARC//DOCK10//CPEB3//IL2//MAPK6//DLG5//LRP8//SNX2//NEDD1//PRKAR2B//YWHAG//PFN2//EPS8//SMURF1//SYT1//PLAA//NAV3//E2F5// |
| GO:0010647 | positive regulation of cell communication | Biological process | 132 | 1737 | 902 | 17653 | 1.48725725599225 | 1.72718488874264e-06 | 7.78730093504432e-05 | 5.76266117032899 | 0.146341463414634 | GADD45A//TNF//CRKL//TAOK1//RAF1//RAP1A//C1QTNF2//LPAR1//NRG1//IGF1//NTF3//PIK3CB//PRKAA1//MAPK1//C5AR1//UBE2V1//EREG//FBXW7//COPS8//MDFIC//TOB1//EPS8//ARHGAP1//LASP1//ARHGAP6//SH3BGRL//SKAP2//HOMER1//SOCS1//F3//GJA1//INHBB//BMP3//BMPR2//TTK//GDF5//ACVR1//UNC5B//SIRT1//KDR//PRR5L//EDN1//SULF1//SOX4//WNT3//NPNT//RICTOR//PIK3CA//RRAGD//SERP1//ISL1//MID1//SEMA4C//FOXA1//AR//GPR55//DLG5//ANO1//SYBU//IFNG//IL2//SOCS3//FLNA//ATP2C1//TBK1//ROR1//IRAK4//NDFIP2//MAVS//MIER1//CCL21//UBE2I//PDE5A//EFNA1//IGFBP3//MET//SPRED1//HIC1//PMAIP1//ADCYAP1//ADRB1//CXCL9//RAPGEF2//NPTN//EP300//DLL1//TP63//EVC//WNT7A//RASSF2//RASGEF1A//RIMS4//GRIK2//SYT1//CD3E//SOS2//RAB8B//ESR1//ITSN1//CHRNB4//ARC//PTEN//SNAP25//CALM2//TXK//RAB3GAP1//JUN//CCL2//CCL8//SPPL3//ARRDC3//BAMBI//LRP6//GSKIP//PSMA2//PSMD12//TBL1XR1//NSG1//PAIP2//FMR1//DYNLL2//PPP3R1//YWHAG//AGO1//RWDD3//CREBRF//SERINC3//NCK1//MYB//CASP2//TNFRSF12A//ST18// |
| GO:0007267 | cell-cell signaling | Biological process | 127 | 1658 | 902 | 17653 | 1.4991019821921 | 1.86843191138073e-06 | 8.36834769315753e-05 | 5.72852272390502 | 0.14079822616408 | APCDD1//CCNY//HBP1//GRK6//HIC1//LRP6//PRKAA1//SKP1//STRN//WNT3//WNT7A//CXXC4//WNT10A//TNRC6B//AGO1//PPP3R1//FZD3//FZD6//GJC1//CHRNB4//DTNA//GABRA5//GLRB//GRIK2//GRM3//NOVA1//MAPK1//PCDHB10//SCN1B//SLC6A1//SNAP25//SYT1//GLRA3//BSN//HOMER1//PPFIA1//PTEN//CHRM2//RIC3//ETV5//TP63//RAPGEF4//SLC25A6//IFNG//KCNB1//MARCKS//NEUROD1//RAP1A//TNF//CLOCK//INHBB//EDN1//ADCYAP1//SNX19//ESR1//MDFIC//PPP2CA//CBFB//SULF1//SOX4//GSC//FRZB//SHISA2//CCND1//GJA1//SERP1//ISL1//RAF1//ANO1//SYBU//TMEM64//DLL1//FMR1//RIMS4//CPEB3//YWHAG//SYNGAP1//RAPGEF2//DBN1//ARC//KCNJ10//NPTN//RAB3GAP1//SYT6//NTF3//PXK//LRP8//ADNP//CRHR1//RAB8B//NR4A2//SDC1//PRICKLE2//ROR1//PSMA2//SMURF1//PSMD12//FOXA1//GLI3//NOG//BAMBI//GSKIP//TBL1XR1//NSG1//ZMYND8//KCNJ3//FXR1//PAIP2//FLNA//PFN2//MYB//TMED1//ADRB1//EFNA1//EFNB2//EREG//IL2//AR//CXCL9//PTHLH//PCDH10//CCL8//CCL21//BMP3//KLF10//ZYX//GDF5// |
| GO:0016358 | dendrite development | Biological process | 29 | 216 | 902 | 17653 | 2.62758171142317 | 1.88081798602241e-06 | 8.36840265754576e-05 | 5.72565323076054 | 0.0321507760532151 | HPRT1//MEF2A//TRAK2//KLF7//FMR1//HECW2//FXR1//DBN1//CAMSAP2//RAPGEF2//RAB21//SLC9A6//ACSL4//ARC//DOCK10//PTEN//WNT7A//CPEB3//LPAR1//ZMYND8//NRG1//IL2//MAPK6//DLG5//EFNA1//LRP8//STRN//SYNGAP1//CD3E// |
| GO:0010629 | negative regulation of gene expression | Biological process | 148 | 2005 | 902 | 17653 | 1.4446389569314 | 1.94322925269319e-06 | 8.58958133069545e-05 | 5.71147596043656 | 0.164079822616408 | TSHZ1//ZBTB18//CEBPB//TCERG1//STRAP//KLF12//GSC//ARX//DNMT3B//EDN1//EDNRB//EFNA1//CC2D1B//EN1//EP300//ESR1//JAZF1//CPEB3//FOXF1//MYT1L//SIN3B//SIRT1//ZFPM2//DNAJB5//GLI3//CNOT7//HIC1//FOXA1//HSBP1//ID2//IFNG//IRF2//AR//ISL1//JARID2//JUN//MAF//MDM4//MEF2A//MYB//NFIA//NFIB//NFIC//NR4A2//PAX5//KLF3//POU4F1//PPARG//ZBTB4//RB1//RBBP8//CCND1//RNF2//SARS//PRDM16//SMARCA2//SUV39H1//ZEB1//TFAP2A//TGIF1//KLF10//TNF//UBE2I//WT1//ZNF217//LRP8//BHLHE41//TBL1XR1//E2F8//ARID5B//BHLHE40//TP63//RUNX3//NOG//FOXP2//PHF14//PNRC1//DCP2//PPP2CA//CNOT6L//PAN3//CNOT6//PATL1//AGO1//BAHD1//H2AFZ//SNIP1//CELF1//FLNA//DNAJB6//PPARGC1B//ELK3//EREG//ATF5//FOXF2//FOXP1//MDFIC//NRG1//IRF1//RASD1//PURA//ATXN1//BTAF1//RPS6KA5//CLOCK//PPM1F//TOB1//FMR1//NANOS1//TIA1//TSC1//FXR1//MEX3B//TSN//TNRC6B//ATAD2//MIER1//YTHDF2//PSMA2//PSMD12//ANP32A//DDX6//PAIP2//SRSF6//NUP54//PRR5L//GTPBP1//QKI//PHF8//BOLL//CSDE1//CAST//CPEB2//ZMYND8//MYCN//TMBIM6//NCK1//CPEB1//ADNP//GJA1//TBK1//ANXA7//ITGB8//KDR//LDLR//POU3F2//NDFIP2//CD3E// |
| GO:0007166 | cell surface receptor signaling pathway | Biological process | 207 | 3016 | 902 | 17653 | 1.34323188375964 | 2.20527647500991e-06 | 9.68460051980001e-05 | 5.65653695538245 | 0.229490022172949 | CBFB//EP300//PRKACB//PSMA2//PSMD12//RAF1//SKP1//UBE2V1//RPS6KA5//BAG6//CR2//C5AR1//APCDD1//CCNY//HBP1//GRK6//HIC1//LRP6//PRKAA1//STRN//WNT3//WNT7A//CXXC4//WNT10A//TNRC6B//AGO1//PPP3R1//FZD3//FZD6//KDR//NTF3//ROR1//PIK3CB//PTPRG//TXK//YES1//CD3E//CD8A//PALM2//BMPR2//EREG//FBXW7//ERRFI1//GPRC5A//SOCS5//NRG1//EPS8//EPS15//PIK3CA//SIRT1//JUN//SMURF1//TGFBR2//KLF10//ZYX//GDF5//ACVR1//GRM3//HOMER1//FMR1//GNAQ//GRIK2//KCNB1//SUSD5//DLL1//FOXA1//TP63//TMEM17//EVC//DZIP1//FOXF1//GLI3//ITGB8//CCM2//APPL1//SPRED1//MAPK1//TIA1//SHCBP1//OTX2//RORA//TNF//F3//TNFAIP3//INHBB//BMP3//TTK//PMEPA1//LDLRAD4//ZEB1//NREP//CNTFR//CRKL//S1PR1//EDN2//TXLNA//IFNA1//IL2//IRAK4//PLP2//CCND1//CCL2//SDC1//LRP8//SOCS1//SOCS3//SPPL3//DENND1B//TNFRSF21//NCK1//ESR1//MDFIC//PPP2CA//SULF1//SOX4//GSC//FRZB//SHISA2//TOB1//NOG//NPNT//STRAP//LEMD3//FAM89B//BAMBI//PRDM16//CTDSPL2//SLC9A6//RAPGEF2//EDARADD//TNFRSF12A//ST18//ACSL1//ADIPOR2//ROBO2//SLIT2//C2CD5//GSTA2//IFNG//MSN//RAP1B//UNC5B//BRWD1//CUL5//RASSF2//RAP1A//IGFBP3//IGF1//AR//ATXN7//TMEM64//NPTN//YTHDF2//DLK2//RB1//DLG5//TSC1//PTEN//ARID5B//GIGYF1//VEGFB//MET//EFNA1//EFNB2//RASA1//ITSN1//SS18//GCSAML//PAX5//NR4A2//PRICKLE2//CHRNB4//RAB3GAP1//GLRB//GLRA3//IRF1//IRF2//MID1//NMI//PPARG//CNOT7//MAVS//CXCL9//CCL8//CCL21//EDN1//FLNA//SEMA4C//PLXNA4//ISL1//GSKIP//TBL1XR1//SIVA1//BCL2A1//CASP2//ZMYND8//GABRA5//PMAIP1//CREBRF//PHF14//ARC//ZMYND11//CRHR1//EDNRB//IGSF3//TSPAN11//MAPK6// |
| GO:0000904 | cell morphogenesis involved in differentiation | Biological process | 65 | 701 | 902 | 17653 | 1.81471037573818 | 2.32634138897674e-06 | 0.000101503527829998 | 5.63332655251837 | 0.0720620842572062 | CLIC4//MET//PALLD//AR//FRMD6//SLITRK3//OGN//POU4F1//PRELP//WNT7A//SLIT2//RANBP9//ARX//DPYSL2//EFNA1//EFNB2//GLI3//OTX2//PIK3CA//PIK3CB//ENAH//MAPK1//ROBO2//SCN1B//WNT3//KLF7//RPS6KA5//NRXN3//FEZ2//NOG//PLXNA4//FLNA//NR4A2//PTEN//NFIB//EP300//WDR1//RAB21//ISL1//UNC5B//VAMP3//FZD3//ADNP//NRG1//TNFRSF12A//ID2//RB1//ULK2//SLC9A6//HPRT1//MEF2A//TRAK2//FMR1//HECW2//FXR1//BMPR2//SEMA4C//POU3F2//SYNGAP1//RAPGEF2//ARC//DOCK10//LRP8//YTHDF2//CRKL// |
| GO:0071310 | cellular response to organic substance | Biological process | 186 | 2656 | 902 | 17653 | 1.3705562271258 | 2.38853064240498e-06 | 0.000103548927785801 | 5.62186918279091 | 0.206208425720621 | CBFB//EP300//SIRT1//JUN//SMURF1//TGFBR2//KLF10//ZYX//GDF5//ACVR1//CHRM2//GNAQ//APPL1//SPRED1//MAPK1//TIA1//SHCBP1//NR5A2//PPARG//ADIPOR2//AGO1//SNIP1//TNF//TNFAIP3//ZEB1//NREP//CNTFR//CRKL//S1PR1//EDN2//TXLNA//EREG//F3//IFNA1//IL2//IRAK4//PIK3CA//PIK3CB//PLP2//CCND1//RORA//CCL2//SDC1//LRP8//SOCS1//SOCS3//SOCS5//CEBPB//CPEB3//SESN1//RRAGD//CPEB1//TSN//TOB1//BMP3//BMPR2//NOG//NPNT//STRAP//LEMD3//FAM89B//BAMBI//PMEPA1//PRDM16//LDLRAD4//SULF1//CTDSPL2//ESR1//PPARGC1B//AR//RB1//CREBRF//SERP1//CPEB2//INHBB//RAB8B//ERRFI1//PTEN//YWHAG//C2CD5//DUSP1//ROBO2//SLIT2//UBA5//ISL1//TP63//FOXA1//EDARADD//TNFRSF21//TNFRSF12A//PSMA2//PSMD12//ST18//ACSL1//HSPA13//IRF1//GSTA2//IFNG//MSN//RAP1B//RAF1//ANO1//SYBU//KDR//YES1//GFPT1//DNAJB9//ASNA1//SEC61A2//BRWD1//NTF3//RAP1A//RAPGEF2//OTX2//SHISA2//NR3C2//NR4A2//NPTN//NCK1//TSC1//IGF1//SLC9A6//IRF2//MID1//NMI//TXK//CNOT7//MAVS//FOXP1//CXCL9//CCL8//CCL21//EDN1//SKP1//UBE2V1//RPS6KA5//UBE2W//EDNRB//CDK19//PLAA//MYB//MYOG//WNT3//TEAD2//KCNE1//WT1//KCNB1//NEUROD1//SOX4//FOXF1//TBK1//POU4F1//RAB12//VAMP3//ZFAND6//PRKAA1//TGIF1//GJA1//CRHR1//ADCY3//PRKACB//PRKAR2B//ADCYAP1//H2AFZ//LRP6//LDLR//DNMT3B//SOX5//WNT7A//WNT10A//BAG6//DLL1//KLF3//MYCN//PPP1R15B//LPAR1//TMBIM6//STMN2//DYNC1LI2//EPS8//JARID2//PIGA//ARID5B//CLOCK//POU3F2//MAPK6// |
| GO:0060996 | dendritic spine development | Biological process | 17 | 90 | 902 | 17653 | 3.69673564917467 | 2.619074926645e-06 | 0.000112820405916561 | 5.58185207707572 | 0.0188470066518847 | ARC//DOCK10//PTEN//WNT7A//CPEB3//FMR1//FXR1//LPAR1//ZMYND8//NRG1//IL2//MAPK6//DLG5//EFNA1//LRP8//SLC9A6//ACSL4// |
| GO:1901362 | organic cyclic compound biosynthetic process | Biological process | 301 | 4704 | 902 | 17653 | 1.25230803241474 | 2.65172907058736e-06 | 0.000113504074078369 | 5.57647085023693 | 0.33370288248337 | NPAT//TSHZ1//ZBTB18//CEBPB//TCERG1//STRAP//KLF12//GSC//ARX//DNMT3B//EDN1//EDNRB//EFNA1//CC2D1B//EN1//EP300//ESR1//JAZF1//CPEB3//FOXF1//MYT1L//SIN3B//SIRT1//ZFPM2//DNAJB5//GLI3//CNOT7//HIC1//FOXA1//HSBP1//ID2//IFNG//IRF2//AR//ISL1//JARID2//JUN//MAF//MDM4//MEF2A//MYB//NFIA//NFIB//NFIC//NR4A2//PAX5//KLF3//POU4F1//PPARG//ZBTB4//RB1//RBBP8//CCND1//RNF2//SARS//PRDM16//SMARCA2//SUV39H1//ZEB1//TFAP2A//TGIF1//KLF10//TNF//UBE2I//WT1//ZNF217//LRP8//BHLHE41//TBL1XR1//E2F8//ARID5B//BHLHE40//TP63//RUNX3//NOG//FOXP2//PHF14//GTF2E1//GTF2H1//CREBRF//ELK3//ETV5//ATF5//FOXF2//FLI1//GTF2A1//HIVEP2//IRF1//MEOX2//MYBL1//MYCN//NEUROD1//NHLH2//OTX2//KLF13//PKNOX1//POU3F2//FOXJ2//HIVEP3//BMPR2//SOX4//SOX5//BTF3//TFE3//TXK//FOSL1//KLF7//CBFB//FUBP3//CCNT2//NMI//CLOCK//ZNF516//POLR3H//SRSF2//SRSF6//SLBP//GFPT1//SLC35D1//NUP54//HPRT1//ADSS//ADCY3//IMPDH1//CMPK1//BAHD1//H2AFZ//ZMYND11//PNRC1//ZNF526//CREBL2//ZNF800//E2F5//ZNF367//ARID2//MED19//ZFP30//KDM2A//MYCBP2//PHF8//FLII//ADNP//ZMYND8//CNOT6L//ZNF521//EPC2//AGO1//HBP1//FOXP1//ZBTB11//ATAD2//MDFIC//HMGB3//HOXA3//HOXD1//ZNF680//ZNF662//NEUROG1//NFE2//NPAS2//ASCC1//IER5//COMMD10//ARID4B//BRWD1//BNC2//MED9//ZNF532//PRKAA1//TRERF1//MAPK1//SLC2A4RG//CCNL1//PHTF2//CNOT6//ZBTB26//MIER1//PURA//ATXN1//ATXN7//SMARCD2//SS18//MED22//ZNF3//ZNF708//ZNF131//ZNF227//ZNF655//ZSCAN5A//SAP30L//ANP32A//PPP1R1B//PCGF5//LCOR//ST18//HIPK3//PPARGC1B//ELAVL2//ELL2//NR5A2//NRBF2//AFF1//MLLT6//OGG1//PHF20L1//PPP2CA//PAK6//RORA//BTG1//UBE2V1//CSDE1//SNIP1//TEAD2//RPS6KA5//EMX2//ETS1//BOLA3//NR3C2//RFX7//TRAK2//BDP1//NRBP1//ACBD3//LBR//INSIG2//MSMO1//GPHN//PTGES3//GRM3//G3BP2//PSAT1//EREG//CRHR1//FLNA//BAMBI//IGF1//LRP6//WNT7A//ACVR1//DNAJB6//NRG1//RASD1//BTAF1//PPM1F//TOB1//SMURF1//BMP3//GDF5//SULF1//LEMD3//CTDSPL2//ADRB1//PTHLH//CALM2//EGLN1//RWDD3//TNFAIP3//DCP2//CCT6A//MYOG//C5AR1//INTS8//NABP1//FZD6//SMUG1//ADCYAP1//S1PR1//KPNA6//DLL1//TBK1//IL2//MET//PPP1R12A//NCK1//ARMCX3//CYTL1//PPP3R1//MAVS//RAF1//YES1//PDE5A//RAPGEF2//C8ORF44-SGK3//SGK3//SGK1//PTEN//RHEBL1//ROR1//IRAK4//EGLN3//PSMA2//PSMD12//SEPSECS//RAP2C//HSBP1L1//TMBIM6//DUSP1//GJA1//KCNK2//FBXW7//NPNT// |
| GO:0009792 | embryo development ending in birth or egg hatching | Biological process | 56 | 578 | 902 | 17653 | 1.89614773782214 | 3.18372570222945e-06 | 0.000135418471221244 | 5.49706435649333 | 0.0620842572062084 | RDH10//EDN1//TAPT1//FOXF1//ZFPM2//TANC2//GJA1//GLI3//AR//MBNL1//BTF3//TGFBR2//FOSL1//CCM2//ACVR1//NOG//EP300//DLL1//MEOX2//RBBP8//SOX4//LRP6//SEMA4C//PRKACB//TSC1//FZD3//FZD6//TEAD2//CEBPB//KBTBD8//GSC//FOXA1//EN1//PAX5//TFAP2A//HOXA3//MYCN//ZEB1//SLC35D1//SULF1//HOXD1//ISL1//ACSL4//SOCS3//E2F8//DNAJB6//EGLN1//MAPK1//VASH2//BMPR2//IGF1//CELF1//RAI2//NR5A2//RICTOR//STOX2// |
| GO:0045859 | regulation of protein kinase activity | Biological process | 71 | 797 | 902 | 17653 | 1.74346009286487 | 3.32353709833545e-06 | 0.000140481758725267 | 5.47839946913523 | 0.0787139689578714 | GADD45A//GTF2H1//PTEN//CCNT2//DIRAS3//TNF//CRKL//TAOK1//RAF1//RAP1A//C1QTNF2//LPAR1//NRG1//IGF1//NTF3//PIK3CB//PRKAA1//MAPK1//C5AR1//UBE2V1//SPRED1//DUSP1//PPP2CA//EREG//FBXW7//ERRFI1//GPRC5A//SOCS5//GNAQ//LRP6//NCK1//GSKIP//RB1//YWHAG//SOCS1//SOCS3//PPM1F//COPS8//MDFIC//ADNP//PIK3CA//PAK6//TGFBR2//SLK//RICTOR//ADCY3//PRKACB//PRKAR2B//EDN1//PDE5A//HIPK3//TNFAIP3//CCNY//CCNL1//CCND1//CCND2//ADCYAP1//CACUL1//MMD//CCL21//RAPGEF2//RASSF2//RAP2C//EFNA1//LRP8//SIRT1//PPP1R1B//ACSL1//IFNG//CALM2//TSC1// |
| GO:0031344 | regulation of cell projection organization | Biological process | 58 | 612 | 902 | 17653 | 1.85476356100459 | 4.22281810684543e-06 | 0.000177384589171402 | 5.37439762492002 | 0.0643015521064302 | SLIT2//PTEN//SNAP25//STMN2//ADCYAP1//NPTN//NCK1//SERPINI1//RAP1A//SCN1B//RAPGEF2//LPAR1//EFNB2//MYLIP//PTPRG//SPOCK1//TSC1//BAG5//DPYSL2//RAB21//WNT7A//CCL21//RAB8B//TAPT1//ADNP//NRG1//TNFRSF12A//ULK2//WNT3//FMR1//HECW2//FXR1//PLXNA4//BMPR2//SEMA4C//EFNA1//POU3F2//TRAK2//SYNGAP1//ROBO2//DBN1//CAMSAP2//PPP1R16B//ZMYND8//GPM6A//CPEB3//IL2//MAPK6//DLG5//ARC//LRP8//PFN2//EPS8//SEPT9//SMURF1//SYT1//PLAA//NAV3// |
| GO:0009887 | animal organ morphogenesis | Biological process | 84 | 1001 | 902 | 17653 | 1.64231777092088 | 4.45871992740046e-06 | 0.000185870472738125 | 5.35078980681716 | 0.0931263858093126 | MYCN//WNT7A//NPNT//GLI3//WT1//GJA1//DLL1//TGFBR2//ARID2//ISL1//TEAD2//ACVR1//ZFPM2//BMPR2//ID2//NOG//JUN//DHRS3//EFNA1//SOX4//FOXF1//NRG1//POU4F1//S1PR1//TFAP2A//HOXA3//MSN//LRIG1//NEUROG1//LRP6//INSIG2//FZD3//FZD6//MAPK1//TSHZ1//GSC//EDN1//NFIC//TP63//SDC1//WNT10A//ERRFI1//NEUROD1//FOXF2//ZEB1//SERP1//ARID5B//AR//PAX5//RDH10//PTEN//EGLN1//TNNI1//PRICKLE2//ROR1//PSMA2//SMURF1//PSMD12//PPARGC1B//FOXA1//DLG5//SRSF6//FOXP2//ESR1//NFIB//TNF//SULF1//BTBD7//ETV5//GCNT4//NR5A2//KLHL3//CEBPB//FRZB//FBXW7//TNFAIP3//CRKL//E2F5//EFNB2//EP300//EREG//FLI1//CCL2//BHLHE41// |
| GO:0120039 | plasma membrane bounded cell projection morphogenesis | Biological process | 60 | 642 | 902 | 17653 | 1.82906106886048 | 4.47979994918148e-06 | 0.000185870472738125 | 5.34874137950776 | 0.0665188470066519 | SLITRK3//OGN//POU4F1//PRELP//WNT7A//SLIT2//RANBP9//ARX//DPYSL2//EFNA1//EFNB2//GLI3//OTX2//PIK3CA//PIK3CB//ENAH//MAPK1//ROBO2//SCN1B//WNT3//KLF7//RPS6KA5//NRXN3//FEZ2//NOG//PLXNA4//NR4A2//PTEN//NFIB//RAB21//ISL1//UNC5B//FZD3//ADNP//NRG1//TNFRSF12A//ULK2//SLC9A6//GJA1//GPM6A//NTF3//SGK1//HPRT1//MEF2A//TRAK2//FMR1//HECW2//FXR1//BMPR2//SEMA4C//POU3F2//SYNGAP1//RAPGEF2//ARC//DOCK10//LRP8//SNX2//SMURF1//SYT1//PLAA// |
| GO:0048812 | neuron projection morphogenesis | Biological process | 59 | 628 | 902 | 17653 | 1.83867237702487 | 4.57785364428723e-06 | 0.000188780635343381 | 5.33933809599841 | 0.065410199556541 | SLITRK3//OGN//POU4F1//PRELP//WNT7A//SLIT2//RANBP9//ARX//DPYSL2//EFNA1//EFNB2//GLI3//OTX2//PIK3CA//PIK3CB//ENAH//MAPK1//ROBO2//SCN1B//WNT3//KLF7//RPS6KA5//NRXN3//FEZ2//NOG//PLXNA4//NR4A2//PTEN//NFIB//RAB21//ISL1//UNC5B//FZD3//ADNP//NRG1//TNFRSF12A//ULK2//SLC9A6//HPRT1//MEF2A//TRAK2//FMR1//HECW2//FXR1//BMPR2//SEMA4C//POU3F2//SYNGAP1//RAPGEF2//ARC//DOCK10//LRP8//SMURF1//SYT1//PLAA//GJA1//GPM6A//NTF3//SGK1// |
| GO:0051716 | cellular response to stimulus | Biological process | 451 | 7556 | 902 | 17653 | 1.16814452091053 | 4.72691678502538e-06 | 0.00019374629222501 | 5.32542204316406 | 0.5 | SIRT1//USP28//CLOCK//KCNH4//RANBP9//SPRED1//EREG//RASGEF1A//NRG1//IL2//MEF2A//MET//MAPK1//MAPK6//PSMA2//PSMD12//RAF1//RASA1//CCL2//TNF//SYNGAP1//RAPGEF2//GADD45A//CRKL//TAOK1//RAP1A//C1QTNF2//LPAR1//IGF1//NTF3//PIK3CB//PRKAA1//C5AR1//UBE2V1//DUSP1//PPP2CA//COPS8//GTF2H1//RBBP8//NABP1//JUN//PMAIP1//BCL2A1//CBFB//EP300//PRKACB//SKP1//RPS6KA5//IRAK4//CCL21//BAG6//CR2//CCL8//SLIT2//EDN2//CXCL9//SKAP2//S1PR1//SPHK2//APCDD1//CCNY//HBP1//GRK6//HIC1//LRP6//STRN//WNT3//WNT7A//CXXC4//WNT10A//TNRC6B//AGO1//PPP3R1//FZD3//FZD6//CNIH1//CDS1//PTGES3//IQGAP2//ADCY3//RALBP1//TMED1//RASSF8//CHRNB4//ARAP2//CLCN6//CNGA3//CNTFR//CREBL2//DPYSL2//DTNA//ELK3//EPS8//ESR1//CD2AP//GABRA5//APPL1//GJA1//ATP2C1//GLRB//CNOT7//LRP12//HIVEP2//AR//NR3C2//PPP1R12A//NR4A2//HPCAL4//GULP1//RASD1//KCNK10//CYTL1//NDFIP2//PPARG//PPP2R5E//PSD//MAVS//ARRDC3//MIER1//HIVEP3//SMOC1//SMOC2//SPOCK1//ZNF217//ZYX//LRP8//GLRA3//SNX27//SYDE2//PPP1R1B//PPFIA1//OR6A2//PDE5A//GPRC5A//DLG5//PLAA//ARHGAP12//GTPBP1//FEZ2//ZNF516//CD69//ULK2//SNX17//KDR//ROR1//PTPRG//TXK//YES1//CD3E//CD8A//C8ORF44-SGK3//SGK2//AKAP11//DGKH//SGK3//NRBP1//ARHGEF4//PRKAR2B//SGK1//STK33//SS18//ANP32A//DGKE//RALGPS1//PALM2//BMPR2//FBXW7//ERRFI1//SOCS5//EPC2//ASTE1//UBE2W//SMUG1//OGG1//ESCO2//KDM2A//ASCC1//MGST1//RORA//EGLN3//CACUL1//FMR1//NPAS2//ZBTB4//CCND1//ZMAT3//SUV39H1//CUL5//IMMP2L//CNOT6L//MDM4//CNOT6//SOX4//E2F8//CASP2//TP63//PPP1R15B//GABARAPL1//CRHR1//EDN1//EDNRB//IGSF3//IFNG//TSPAN11//NCK1//EPS15//PIK3CA//SMURF1//TGFBR2//KLF10//GDF5//ACVR1//NMUR1//RAPGEF4//CHRM2//ADCYAP1//ADRB1//TAPT1//FRZB//GNAQ//OR4N4//GRM3//KCNK2//RHOB//RGS7BP//PTHLH//ITSN1//SOS2//CALM2//GPR55//FLNA//ANO1//HOMER1//NSG1//GRIK2//KCNB1//NXPH3//SORCS1//SORCS2//SUSD5//DLL1//FOXA1//TMEM17//EVC//DZIP1//FOXF1//GLI3//ITGB8//CCM2//RCAN3//SAMD14//TBK1//SNIP1//MDFIC//SOCS1//SOCS3//NMI//NEUROD1//RAB18//RGL1//RND3//ARHGAP1//RIN2//DOCK10//DIRAS3//RB1//G3BP2//ARHGAP6//PAK6//TRPC3//GIT2//TIA1//SHCBP1//OTX2//GSKIP//INHBB//RRAGD//PLEKHF2//NR5A2//ADIPOR2//RWDD3//YWHAG//TOB1//LASP1//SH3BGRL//IGFBP3//PRR5L//TXLNA//IFNA1//OGN//BMP3//VEGFB//PPP4R2//F3//TNFAIP3//TTK//PMEPA1//LDLRAD4//PPP1R16B//PTEN//UNC5B//ZEB1//NREP//PLP2//SDC1//PLXNA4//CEBPB//CPEB3//SESN1//CPEB1//SPPL3//DENND1B//TNFRSF21//SLK//GSC//IRF1//KCNJ2//SULF1//SHISA2//TSN//DNAJB9//JKAMP//NOG//NPNT//STRAP//LEMD3//FAM89B//BAMBI//PRDM16//CTDSPL2//PPARGC1B//CREBRF//SERP1//ISL1//SLC9A6//ADNP//FOSL1//RHEBL1//RICTOR//FAM83D//TSC1//IQSEC2//FBXO8//AGFG1//RAB12//RAB21//RAB30//RAB9B//RAB8B//RAB28//RAP2C//RAP1B//CPEB2//C2CD5//ROBO2//MID1//SEMA4C//INSIG2//UBA5//EDARADD//TNFRSF12A//ST18//ACSL1//ETV5//IER5//HSPA13//NRBF2//PPP2CB//PLEKHG4B//TEAD2//MOB1B//PPM1F//GSTA2//MSN//SYBU//GFPT1//ASNA1//SEC61A2//BRWD1//RASSF2//GSTM3//PHLDA3//ZFAND6//UBE2I//ZMYND11//EFNA1//HIPK3//ATXN7//PEX5L//TMEM64//NPTN//YTHDF2//DLK2//ARID5B//GIGYF1//EFNB2//DHRS3//GCSAML//PAX5//OTUD3//UGT2B28//PRICKLE2//RAB3GAP1//IRF2//FOXP1//EGLN1//TMBIM6//ETS1//MYB//HSBP1//HSBP1L1//FIBIN//CDK19//ADSS//MYOG//CLIC4//SYT1//TFAP2A//ID2//SLC41A1//POU3F2//KCNE1//WT1//POU4F1//VAMP3//TGIF1//H2AFZ//LDLR//DNMT3B//CCND2//SOX5//TBL1XR1//FASTK//SIVA1//ZMYND8//BSN//LYPD6//DNAJB6//NUP54//BAG5//DYNLL2//KLF3//SERINC3//MYCN//FAM168A//STMN2//DYNC1LI2//JARID2//PIGA//PHF14//ARC// |
| GO:0051338 | regulation of transferase activity | Biological process | 83 | 988 | 902 | 17653 | 1.64411855795039 | 4.88954713173081e-06 | 0.000199204862963226 | 5.31073136322775 | 0.0920177383592018 | GADD45A//GTF2H1//PTEN//CCNT2//DIRAS3//TNF//CRKL//TAOK1//RAF1//RAP1A//C1QTNF2//LPAR1//NRG1//IGF1//NTF3//PIK3CB//PRKAA1//MAPK1//C5AR1//UBE2V1//SPRED1//DUSP1//PPP2CA//EREG//FBXW7//ERRFI1//GPRC5A//SOCS5//GNAQ//LRP6//NCK1//GSKIP//RB1//YWHAG//SOCS1//SOCS3//PPM1F//COPS8//MDFIC//ADNP//PIK3CA//PAK6//TGFBR2//SLK//RICTOR//KDR//MET//ROR1//ADCY3//PRKACB//PRKAR2B//EDN1//PDE5A//HIPK3//NRBF2//CCL21//TNFAIP3//CCNY//CCNL1//CCND1//CCND2//TSC1//ADCYAP1//CACUL1//MMD//RAPGEF2//RASSF2//ZER1//BAG5//DCUN1D4//ARRDC3//PTGES3//PPARG//RAP2C//EFNA1//LRP8//SIRT1//PPP1R1B//ACSL1//IFNG//CALM2//UBE2I//SPTSSB// |
| GO:0048858 | cell projection morphogenesis | Biological process | 60 | 644 | 902 | 17653 | 1.82338075498203 | 4.93871097023062e-06 | 0.000200003007734549 | 5.3063863894503 | 0.0665188470066519 | SLITRK3//OGN//POU4F1//PRELP//WNT7A//SLIT2//RANBP9//ARX//DPYSL2//EFNA1//EFNB2//GLI3//OTX2//PIK3CA//PIK3CB//ENAH//MAPK1//ROBO2//SCN1B//WNT3//KLF7//RPS6KA5//NRXN3//FEZ2//NOG//PLXNA4//NR4A2//PTEN//NFIB//RAB21//ISL1//UNC5B//FZD3//ADNP//NRG1//TNFRSF12A//ULK2//SLC9A6//GJA1//GPM6A//NTF3//SGK1//HPRT1//MEF2A//TRAK2//FMR1//HECW2//FXR1//BMPR2//SEMA4C//POU3F2//SYNGAP1//RAPGEF2//ARC//DOCK10//LRP8//SNX2//SMURF1//SYT1//PLAA// |
| GO:0048762 | mesenchymal cell differentiation | Biological process | 27 | 203 | 902 | 17653 | 2.60303321573296 | 5.04831949302148e-06 | 0.000203224909114906 | 5.29685316769707 | 0.0299334811529933 | EDNRB//ISL1//SEMA4C//ACVR1//FOXF2//PPP3R1//FAM83D//DLG5//NOG//EFNA1//BAMBI//TGFBR2//STRAP//FOXA1//PPP2CA//PTEN//LDLRAD4//LRP6//KBTBD8//RDH10//EDN1//TAPT1//NRG1//FRZB//WNT10A//GSC//MAPK1// |
| GO:0120035 | regulation of plasma membrane bounded cell projection organization | Biological process | 57 | 602 | 902 | 17653 | 1.85306369750499 | 5.25579009827033e-06 | 0.000210324901979895 | 5.27936198762317 | 0.0631929046563193 | SLIT2//PTEN//SNAP25//STMN2//ADCYAP1//NPTN//NCK1//SERPINI1//RAP1A//SCN1B//RAPGEF2//LPAR1//EFNB2//MYLIP//PTPRG//SPOCK1//TSC1//BAG5//DPYSL2//RAB21//WNT7A//CCL21//TAPT1//ADNP//NRG1//TNFRSF12A//ULK2//WNT3//FMR1//HECW2//FXR1//PLXNA4//BMPR2//SEMA4C//EFNA1//POU3F2//TRAK2//SYNGAP1//ROBO2//DBN1//CAMSAP2//PPP1R16B//ZMYND8//GPM6A//CPEB3//IL2//MAPK6//DLG5//ARC//LRP8//PFN2//EPS8//SEPT9//SMURF1//SYT1//PLAA//NAV3// |
| GO:0043523 | regulation of neuron apoptotic process | Biological process | 26 | 194 | 902 | 17653 | 2.62291128534528 | 6.53605061626026e-06 | 0.00026001947245746 | 5.1846845932141 | 0.0288248337028825 | CEBPB//CNTFR//EN1//UNC5B//ADNP//GABRA5//GRIK2//ISL1//JUN//NTF3//NR4A2//PIK3CA//POU4F1//RASA1//CCL2//ITSN1//C5AR1//GDF5//SYNGAP1//MYB//TFAP2A//CASP2//FBXW7//KCNB1//EGLN3//TP63// |
| GO:0001501 | skeletal system development | Biological process | 49 | 493 | 902 | 17653 | 1.94518604138651 | 6.58194283848341e-06 | 0.000260313914717329 | 5.18164589369753 | 0.0543237250554324 | MYCN//WNT7A//SNX19//NFIB//CYTL1//GDF5//RUNX3//SULF1//BMPR2//TGFBR2//MAF//PTHLH//GLI3//SOX5//IGF1//EP300//FLI1//FOXP1//PAX5//TFAP2A//RDH10//GSC//HOXA3//ZEB1//ARID5B//TAPT1//SLC35D1//HOXD1//NOG//EDN1//EVC//ITGB8//BMP3//PPARGC1B//DHRS3//INSIG2//LRP6//FRZB//NEUROG1//FBXW7//HAPLN1//EN1//SERP1//GNAQ//PRELP//SOX4//KLF10//TP63//RASSF2// |
| GO:0007517 | muscle organ development | Biological process | 42 | 398 | 902 | 17653 | 2.0652764933314 | 7.0037020296579e-06 | 0.000275383935038235 | 5.1546723391203 | 0.0465631929046563 | ZFPM2//NRG1//POU4F1//NOG//S1PR1//ZBTB18//CFL2//EP300//MEOX2//MYOG//GPCPD1//CCNT2//FOXP2//PAX5//RB1//HIVEP3//GJA1//DLL1//NEUROG1//PRKAA1//GSC//SERP1//ARID5B//HOMER1//EGLN1//ISL1//TNNI1//JARID2//KCNK2//PTEN//TGFBR2//CNTFR//WT1//EDN1//IGF1//NLN//EFNB2//EVC//SIRT1//MEF2A//FXR1//SGCE// |
| GO:0051402 | neuron apoptotic process | Biological process | 28 | 219 | 902 | 17653 | 2.50222235721735 | 7.31338162473586e-06 | 0.000285898265480281 | 5.13588176375712 | 0.0310421286031042 | EGLN3//TP63//CEBPB//CNTFR//EN1//UNC5B//ADNP//GABRA5//GRIK2//ISL1//JUN//NTF3//NR4A2//PIK3CA//POU4F1//RASA1//CCL2//ITSN1//C5AR1//GDF5//SYNGAP1//MYB//TFAP2A//CASP2//FBXW7//KCNB1//TNFRSF21//RB1// |
| GO:0051240 | positive regulation of multicellular organismal process | Biological process | 121 | 1606 | 902 | 17653 | 1.47452388907451 | 7.5214226655566e-06 | 0.000292011967162989 | 5.12370000550358 | 0.134146341463415 | EREG//TNF//ADRB1//GLI3//FZD3//F3//TGFBR2//CD3E//EDN1//EDNRB//EDN2//GJA1//NPNT//SULF1//ISL1//RORA//C5AR1//ETS1//FOXP1//KDR//RHOB//BMPR2//WNT7A//IGF1//MEF2A//PDE5A//IFNG//JUN//PPM1F//BAMBI//STMN2//ADCYAP1//NPTN//NCK1//SERPINI1//RAP1A//SCN1B//RAPGEF2//LDLR//MYOG//INHBB//TFAP2A//ACVR1//WASF3//NRG1//SOX5//GDF5//POLR3H//EP300//TBK1//IRF1//MAVS//IL2//TXK//CEBPB//CCND1//POU3F2//OTX2//SIRT1//SOCS1//MYB//SOCS5//ID2//RB1//DNMT3B//ETV5//MMD//FOXA1//NEUROD1//NEUROG1//PTEN//ZEB1//TP63//PPARGC1B//TMEM64//POU4F1//KLF10//ITGB8//BTG1//VEGFB//VASH2//ADAM12//ADNP//TNFRSF12A//ARX//SERP1//DLL1//PRKAA1//WNT3//PPARG//ROBO2//PLXNA4//SLIT2//RAB21//RAB8B//SLITRK3//DLG5//CHRNB4//ZFPM2//WT1//SRSF6//FOXP2//AR//LPAR1//CPEB3//FMR1//ZMYND8//MAPK6//LRP8//TAPT1//NOG//TRPC3//PPP1R16B//EFNB2//SMURF1//SYT1//PLAA//LRP6//TNFAIP3//MET//FLNA// |
| GO:0050790 | regulation of catalytic activity | Biological process | 162 | 2292 | 902 | 17653 | 1.3832872848005 | 7.55612808716886e-06 | 0.000292011967162989 | 5.12170068877573 | 0.17960088691796 | GADD45A//GTF2H1//PTEN//CCNT2//DIRAS3//TNF//CRKL//TAOK1//RAF1//RAP1A//C1QTNF2//LPAR1//NRG1//IGF1//NTF3//PIK3CB//PRKAA1//MAPK1//C5AR1//UBE2V1//SPRED1//DUSP1//PPP2CA//EREG//FBXW7//ERRFI1//GPRC5A//SOCS5//GNAQ//LRP6//NCK1//GSKIP//RB1//YWHAG//SOCS1//SOCS3//PPM1F//EGLN3//F3//PMAIP1//PPARG//CASP2//ADCY3//ADCYAP1//CRHR1//ADRB1//EDNRB//GRM3//NMUR1//TXK//GPR55//COPS8//MDFIC//PI15//PPARGC1B//NPNT//ESR1//MTMR9//FKBP15//PPP1R12A//PLEKHF2//SERPINI1//SERPINB13//SPOCK1//RECK//IQGAP2//CPEB2//TSC1//SLIT2//SIRT1//ADNP//PIK3CA//PAK6//TGFBR2//SLK//RICTOR//PLAA//ARPP19//PPP1R1B//CALM2//PPP1R15B//CNN3//PXK//DNAJB6//DNAJA2//PFN2//RALBP1//RAB3GAP1//ARHGAP1//SBF2//SYDE2//PLXNA4//ARAP2//DENND1B//S1PR1//ELMOD2//AGFG1//JUN//ARHGAP6//RIN2//DOCK10//RASA1//CCL2//CCL8//CCL21//BNIP2//SYNGAP1//ARHGAP12//RAPGEF2//GIT2//KDR//MET//ROR1//PRKACB//PRKAR2B//TRIM23//NR4A2//ARL6IP1//TP63//EDN1//PDE5A//TMEM64//HIPK3//NRBF2//RCAN3//UBXN2B//PPP4R2//PPP1R16B//IGFBP3//PPP2R5E//TNFAIP3//CCNY//CCNL1//CCND1//CCND2//CACUL1//MMD//RASSF2//ABHD5//EGLN1//MID1IP1//ZER1//BAG5//DCUN1D4//ARRDC3//PTGES3//IFNG//EDN2//TMBIM6//RAP2C//EFNA1//LRP8//ACSL1//TBC1D12//CAST//UBE2I//MTRR//SPTSSB//DYNLL2//ST18//AR//SGK1// |
| GO:0016043 | cellular component organization | Biological process | 382 | 6269 | 902 | 17653 | 1.19255131805078 | 7.73929882907982e-06 | 0.000297391352165152 | 5.11129838407879 | 0.42350332594235 | MEF2A//UBXN2B//TRAPPC8//GABARAPL1//ATG16L1//WDR45B//ULK2//SEPT9//SIRT1//SUV39H1//TACC2//CAMSAP1//DYNC1LI2//CAMSAP2//MID1//ATXN7//SS18//MIEF1//STRAP//COPS8//GTF2H1//PTGES3//CDH20//NRG1//WNT3//GTF2E1//C8ORF44-SGK3//SGK2//SGK3//NANOS1//IGFBP3//RB1//SGK1//SPOCK1//CLIP1//AGFG1//JUN//PMAIP1//BCL2A1//CLIC4//MET//FMR1//NTF3//PALLD//AR//FRMD6//NAP1L3//HIST2H2BE//EPS8//RHOB//RND3//MARCKS//CFL2//WDR1//DNAJB6//IQSEC2//FLII//PFN2//PAK6//WASF3//SH3D19//DPYSL2//ARC//KLHL20//MSN//BRWD1//CCL2//CCDC6//STX6//SYT6//SNAP25//VAMP1//SYT1//VAMP3//C2CD5//ZFAND6//PURA//ZMYND11//PHF13//ATAD2//H2AFZ//JARID2//BAG6//ARID2//NFE2//SMARCD2//ESR1//HMGB3//FOXA1//MYB//SMARCA2//ANP32A//TP63//BAHD1//CELF1//SRSF6//TIMM9//SLC25A6//MTX3//IMMP2L//CLCN6//GULP1//ARHGAP12//CD2AP//NCK1//VPS4B//REEP3//LEMD3//NUP54//SERP1//PALM2//PPARGC1B//DBN1//SAMD14//TMOD3//TMOD1//STMN2//RANBP9//VBP1//MID1IP1//NAV3//SPTSSB//TMED1//RAB30//GOLGA6A//TJAP1//BAG5//GJC1//TNS1//TTK//DYNLL2//FBXW7//TAOK1//CENPO//CNEP1R1//PPP2CA//CDC14A//GADD45A//PPP1R12A//SYCE1//MYBL1//EREG//CD3E//FOSL1//SLITRK3//OGN//POU4F1//PRELP//WNT7A//SLIT2//ARX//EFNA1//EFNB2//GLI3//OTX2//PIK3CA//PIK3CB//ENAH//MAPK1//ROBO2//SCN1B//KLF7//RPS6KA5//NRXN3//FEZ2//GPM6A//PCDHB10//PTEN//BSN//PDZRN3//GPHN//NOG//KATNAL1//CFDP1//LPAR1//BAMBI//ANXA7//KDR//RASA1//PLXNA4//PPP2CB//ARHGAP6//TMEM17//DZIP1//FLNA//BBS9//GMNC//C5ORF30//CEP41//SKP1//CNOT6L//PAN3//CNOT6//ETS1//CHMP3//ADCYAP1//NPTN//SERPINI1//RAP1A//RAPGEF2//MYLIP//PTPRG//TSC1//PMEPA1//LDLRAD4//EPS15//SNX5//SNX2//GJA1//STRN//SYNGAP1//PEX5L//EP300//EPC2//CLOCK//SIN3B//ARID4B//MIER1//SAP30L//TBL1XR1//PPM1F//ARID5B//NR4A2//NFIB//E2F5//TAPT1//S1PR1//ARHGEF4//HECW2//RAB21//SNX17//HAPLN1//FOXF1//FOXF2//NPNT//ITGB8//SMOC2//TNF//ADAM12//MFAP5//RECK//IL2//FRZB//PPARG//BMPR2//BTG1//WT1//ADIPOR2//EI24//FAM118B//TDRD5//RICTOR//CCL21//CNN3//LIMCH1//ISL1//NREP//SLK//LRP12//PSD//RAF1//IFNG//TBC1D12//RAB8B//ATP2C1//NEUROG1//DCP2//CCT6A//KIF13A//SULF1//PHF8//UNC5B//DDX6//PATL1//CNOT7//G3BP2//IQGAP2//ARFIP1//RASSF8//PRKACB//RAB3GAP1//RAB18//PRDM16//AGO1//PRKAA1//RNF2//ZMYND10//VPS37A//PCGF5//FZD3//APOO//C1QTNF2//KCNA4//KCNB1//SLC6A1//GLRA3//ZYX//TGM3//XKR8//PPP1R1B//VASH2//DLG5//CCM2//TMEFF2//ADNP//TNFRSF12A//EDN1//PRR16//DLL1//LDLR//CBLL1//IGF1//ATF5//CNIH1//SEC23A//MCFD2//NSG1//VTI1A//TRAK2//ID2//SLC9A6//HPRT1//FXR1//SEMA4C//SIRPA//TUB//POU3F2//ETV5//CUTC//SBF2//DGKH//TNFAIP3//KCNJ2//THG1L//CHRNB4//GLRB//MCCC2//MZT1//PPP1R16B//ZMYND8//PPFIA1//DNMT3B//PAX5//NFIA//SYBU//ACSL4//DOCK10//CPEB3//MAPK6//LRP8//CHRM2//ITSN1//YWHAG//SGCE//SMURF1//MDM4//TEAD2//SKAP2//MGST1//C1QTNF6//KDM2A//BDP1//RTN3//ARL6IP1//DUSP1//ANAPC15//NEDD1//PRKAR2B//YTHDF2//CRKL//MAVS//PPP3R1//MYOG//CXCL9//RAP1B//SDC1//PLAA//NMUR1//SNIP1//LRP6//SNX19//CBFB// |
| GO:0048667 | cell morphogenesis involved in neuron differentiation | Biological process | 54 | 567 | 902 | 17653 | 1.86390032731496 | 7.86584064599063e-06 | 0.0003005462163211 | 5.10425485620595 | 0.0598669623059867 | SLITRK3//OGN//POU4F1//PRELP//WNT7A//SLIT2//RANBP9//ARX//DPYSL2//EFNA1//EFNB2//GLI3//OTX2//PIK3CA//PIK3CB//ENAH//MAPK1//ROBO2//SCN1B//WNT3//KLF7//RPS6KA5//NRXN3//FEZ2//NOG//PLXNA4//NR4A2//PTEN//NFIB//RAB21//ISL1//UNC5B//FZD3//ADNP//NRG1//TNFRSF12A//ULK2//SLC9A6//HPRT1//MEF2A//TRAK2//FMR1//HECW2//FXR1//BMPR2//SEMA4C//POU3F2//SYNGAP1//RAPGEF2//ARC//DOCK10//LRP8//ID2//RB1// |
| GO:0043085 | positive regulation of catalytic activity | Biological process | 109 | 1414 | 902 | 17653 | 1.50865199760394 | 8.31573229014383e-06 | 0.00031595110942833 | 5.08009950043541 | 0.120842572062084 | GADD45A//TNF//CRKL//TAOK1//RAF1//RAP1A//C1QTNF2//LPAR1//NRG1//IGF1//NTF3//PIK3CB//PRKAA1//MAPK1//C5AR1//UBE2V1//EREG//FBXW7//EGLN3//F3//PMAIP1//PPARG//CASP2//NMUR1//GNAQ//TXK//GPR55//COPS8//MDFIC//SOCS1//PPARGC1B//NPNT//ESR1//MTMR9//ADNP//PIK3CA//PAK6//TGFBR2//SLK//RICTOR//PLAA//CALM2//PPP1R15B//DNAJB6//DNAJA2//PFN2//RALBP1//ADCYAP1//ARAP2//ADRB1//DENND1B//S1PR1//RAB3GAP1//ELMOD2//AGFG1//JUN//ARHGAP1//ARHGAP6//ERRFI1//RIN2//DOCK10//RASA1//CCL2//CCL8//CCL21//BNIP2//SYNGAP1//ARHGAP12//RAPGEF2//GIT2//KDR//MET//ROR1//ADCY3//PRKACB//PRKAR2B//PPP1R12A//SIRT1//PPM1F//EDN1//PDE5A//CCNY//CCNL1//CCND1//CCND2//CCNT2//CACUL1//MMD//RASSF2//ABHD5//MID1IP1//DCUN1D4//ARRDC3//PTGES3//IFNG//EDN2//EFNA1//LRP8//ACSL1//PPP2CA//TBC1D12//TSC1//SYDE2//UBE2I//PTEN//DYNLL2//ST18//TRIM23//NR4A2// |
| GO:0061138 | morphogenesis of a branching epithelium | Biological process | 25 | 185 | 902 | 17653 | 2.64472343740636 | 8.44680826249652e-06 | 0.000319138347928849 | 5.07330736393087 | 0.0277161862527716 | EDN1//PPP3R1//TGFBR2//ACVR1//NPNT//GLI3//WT1//CLIC4//MET//MYCN//SLIT2//RDH10//FOXF1//FOXA1//DLG5//LRP6//TP63//NOG//AR//ESR1//SOCS3//SULF1//BTBD7//TNF//ETV5// |
| GO:0007610 | behavior | Biological process | 55 | 583 | 902 | 17653 | 1.84631636196293 | 8.55355716304387e-06 | 0.000321376150520365 | 5.06785323790254 | 0.0609756097560976 | ADCYAP1//GABRA5//GRIK2//CNTFR//POU4F1//GCNT4//MAPK1//PTEN//ARC//JUN//PRKAR2B//ATXN1//SLC6A1//FOSL1//NRXN3//CEBPB//KCNK2//NTF3//PAIP2//KCNK10//ADNP//CPEB3//LDLR//SGK1//CCND2//NHLH2//PPP1R1B//HPRT1//CHRNB4//ETV5//NRG1//NOVA1//SNAP25//STRN//GLRB//KCNJ10//PAX5//HOMER1//EN1//EPS8//ID2//NR4A2//TMOD1//TSC1//ADCY3//NPTN//SYNGAP1//NEUROG1//ANXA7//FOXP2//CREBRF//GNAQ//ARRDC3//GJA1//ADRB1// |
| GO:0034645 | cellular macromolecule biosynthetic process | Biological process | 335 | 5401 | 902 | 17653 | 1.21389916706728 | 9.595308120141e-06 | 0.000358525242080185 | 5.01794107483904 | 0.37139689578714 | SIRT1//IGF1//NFIA//NFIB//NFIC//RBBP8//RBMS1//NPAT//TSHZ1//ZBTB18//CEBPB//TCERG1//STRAP//KLF12//GSC//ARX//DNMT3B//EDN1//EDNRB//EFNA1//CC2D1B//EN1//EP300//ESR1//JAZF1//CPEB3//FOXF1//MYT1L//SIN3B//ZFPM2//DNAJB5//GLI3//CNOT7//HIC1//FOXA1//HSBP1//ID2//IFNG//IRF2//AR//ISL1//JARID2//JUN//MAF//MDM4//MEF2A//MYB//NR4A2//PAX5//KLF3//POU4F1//PPARG//ZBTB4//RB1//CCND1//RNF2//SARS//PRDM16//SMARCA2//SUV39H1//ZEB1//TFAP2A//TGIF1//KLF10//TNF//UBE2I//WT1//ZNF217//LRP8//BHLHE41//TBL1XR1//E2F8//ARID5B//BHLHE40//TP63//RUNX3//NOG//FOXP2//PHF14//GTF2E1//SEPSECS//SECISBP2L//GTF2H1//CREBRF//ELK3//ETV5//ATF5//FOXF2//FLI1//GTF2A1//HIVEP2//IRF1//MEOX2//MYBL1//MYCN//NEUROD1//NHLH2//OTX2//KLF13//PKNOX1//POU3F2//FOXJ2//HIVEP3//BMPR2//SOX4//SOX5//BTF3//TFE3//TXK//FOSL1//KLF7//CBFB//FUBP3//CCNT2//NMI//CLOCK//ZNF516//POLR3H//SRSF2//SRSF6//SLBP//PURA//GMNC//ESCO2//PPP2CA//BAHD1//H2AFZ//ZMYND11//PNRC1//ZNF526//CREBL2//ZNF800//E2F5//ZNF367//ARID2//MED19//ZFP30//KDM2A//MYCBP2//PHF8//FLII//ADNP//ZMYND8//CNOT6L//ZNF521//EPC2//AGO1//HBP1//FOXP1//ZBTB11//ATAD2//MDFIC//HMGB3//HOXA3//HOXD1//ZNF680//ZNF662//NEUROG1//NFE2//NPAS2//ASCC1//IER5//COMMD10//ARID4B//BRWD1//BNC2//MED9//ZNF532//PRKAA1//TRERF1//MAPK1//SLC2A4RG//CCNL1//PHTF2//CNOT6//ZBTB26//MIER1//ATXN1//ATXN7//SMARCD2//SS18//MED22//ZNF3//ZNF708//ZNF131//ZNF227//ZNF655//ZSCAN5A//SAP30L//ANP32A//PPP1R1B//PCGF5//LCOR//ST18//HIPK3//PPARGC1B//ELAVL2//ELL2//NR5A2//NRBF2//AFF1//MLLT6//OGG1//PHF20L1//PAK6//RORA//BTG1//UBE2V1//CSDE1//SNIP1//TEAD2//RPS6KA5//EMX2//ETS1//BOLA3//NR3C2//RFX7//TRAK2//BDP1//NRBP1//CPEB2//CPEB1//DDX3Y//GTPBP1//DDX6//TSC1//KBTBD8//FUT9//B3GALNT2//FUT2//FUT5//SERP1//ST8SIA5//GFPT1//KCNE1//CCDC126//ALG2//EOGT//GCNT4//VEGFB//WDR45B//ALPI//RECK//PYURF//PIGP//PIGA//PTGES3//G3BP2//EREG//CCL21//HS3ST5//FMR1//PRR16//FXR1//FLNA//BAMBI//LRP6//WNT7A//ACVR1//DNAJB6//NRG1//RASD1//BTAF1//PPM1F//TOB1//CELF1//NANOS1//TIA1//MEX3B//ZDHHC22//ZDHHC7//ZDHHC18//ST3GAL5//MCFD2//ABHD17C//SLC35D1//DSEL//SMURF1//BMP3//GDF5//SULF1//LEMD3//CTDSPL2//RWDD3//TNFAIP3//DCP2//CCT6A//FBXW7//INSIG2//TNRC6B//NCK1//MYOG//C5AR1//INTS8//NABP1//EGLN1//FZD6//C1QTNF2//ADCYAP1//S1PR1//KPNA6//DLL1//TBK1//IL2//MET//PPP1R12A//ARMCX3//CYTL1//PPP3R1//MAVS//RAF1//YES1//PAIP2//BOLL//C8ORF44-SGK3//SGK3//SGK1//PTEN//RHEBL1//ROR1//IRAK4//NUP54//YTHDF2//QKI//EGLN3//PSMA2//PSMD12//ATG16L1//RAB3GAP1//PPP1R15B//TMBIM6//DUSP1//GJA1//KCNK2//NPNT// |
| GO:0008285 | negative regulation of cell proliferation | Biological process | 65 | 732 | 902 | 17653 | 1.73785788714817 | 9.66526483973189e-06 | 0.000359154868742345 | 5.01478624128796 | 0.0720620842572062 | SULF1//GJA1//TNF//BMPR2//EFNB2//TNFRSF21//CEBPB//PDE5A//DLG5//MORC3//IFNG//IGFBP3//OGN//PPARG//TNFAIP3//IL2//STRAP//EREG//AR//ISL1//PTEN//RB1//GDF5//RUNX3//JARID2//KCNK2//TGFBR2//NOG//ADCYAP1//CCL8//WT1//ID2//FBXW7//NFIB//PHF14//TOB1//COPS8//ARID2//ETS1//ATF5//FRZB//TES//GLI3//DLL1//CNOT7//IRF1//JUN//MDM4//MYOG//KLF13//PTHLH//RAF1//SMARCA2//SOX4//STRN//ZEB1//BTG1//TFAP2A//KLF10//TSC1//FOSL1//CUL5//SKAP2//RAPGEF2//ST18// |
| GO:0043170 | macromolecule metabolic process | Biological process | 554 | 9622 | 902 | 17653 | 1.12682479775422 | 9.86873764817729e-06 | 0.000364711872757503 | 5.00573839621513 | 0.614190687361419 | SIRT1//IGF1//NFIA//NFIB//NFIC//RBBP8//RBMS1//GADD45A//GTF2H1//PTEN//CCNT2//DIRAS3//NPAT//TSHZ1//ZBTB18//CEBPB//TCERG1//STRAP//KLF12//GSC//ARX//DNMT3B//EDN1//EDNRB//EFNA1//CC2D1B//EN1//EP300//ESR1//JAZF1//CPEB3//FOXF1//MYT1L//SIN3B//ZFPM2//DNAJB5//GLI3//CNOT7//HIC1//FOXA1//HSBP1//ID2//IFNG//IRF2//AR//ISL1//JARID2//JUN//MAF//MDM4//MEF2A//MYB//NR4A2//PAX5//KLF3//POU4F1//PPARG//ZBTB4//RB1//CCND1//RNF2//SARS//PRDM16//SMARCA2//SUV39H1//ZEB1//TFAP2A//TGIF1//KLF10//TNF//UBE2I//WT1//ZNF217//LRP8//BHLHE41//TBL1XR1//E2F8//ARID5B//BHLHE40//TP63//RUNX3//NOG//FOXP2//PHF14//RANBP9//SPRED1//EREG//RASGEF1A//NRG1//IL2//MET//MAPK1//MAPK6//PSMA2//PSMD12//RAF1//RASA1//CCL2//SYNGAP1//RAPGEF2//PNRC1//DCP2//PPP2CA//CRKL//TAOK1//RAP1A//C1QTNF2//LPAR1//NTF3//PIK3CB//PRKAA1//C5AR1//UBE2V1//DUSP1//FBXO41//RNF145//ZNRF2//RNF167//MYLIP//ANKIB1//UBE2W//FBXW7//SMURF1//HECW2//KLHL42//SKP1//THOP1//LONRF3//FBXO30//CBFB//UBE3B//LONRF1//FBXO44//CNOT6L//PAN3//CNOT6//PATL1//COPS8//SRSF6//MBNL2//RBFOX3//FMR1//MBNL1//RBM25//SRSF2//ELAVL2//NOVA1//PCBP2//RBM41//NKTR//RRP15//PTGES3//NABP1//AGO1//GTF2E1//SEPSECS//SECISBP2L//RPP14//RPP25//NSG1//EPS15//VAMP3//SOCS1//IGFBP3//LRP6//PRR5L//PPP1R15B//SLIT2//NPTN//KDR//MAVS//CCND2//ABHD17C//F3//CREBRF//ELK3//ETV5//ATF5//FOXF2//FLI1//GTF2A1//HIVEP2//IRF1//MEOX2//MYBL1//MYCN//NEUROD1//NHLH2//OTX2//KLF13//PKNOX1//POU3F2//FOXJ2//HIVEP3//BMPR2//SOX4//SOX5//BTF3//TFE3//TXK//FOSL1//KLF7//FUBP3//NMI//CLOCK//ZNF516//POLR3H//SLBP//ERRFI1//GPRC5A//SOCS5//ZER1//CACUL1//RNF38//MARCH10//PDZRN3//MYCBP2//FBXO33//KLHL3//RNF11//KLHL20//SPOPL//TRIM23//DCAF8//CBLL1//CUL5//KBTBD8//SOCS3//CHST1//HS3ST5//SDC1//HS3ST3A1//HS3ST1//PURA//GMNC//ESCO2//EPC2//ASTE1//USP28//SMUG1//OGG1//KDM2A//MTRR//ASCC1//HMGB3//TSN//BAHD1//H2AFZ//ARID4B//ZMYND11//ZNF526//CREBL2//ZNF800//E2F5//ZNF367//ARID2//MED19//ZFP30//PHF8//FLII//ADNP//ZMYND8//ZNF521//HBP1//FOXP1//ZBTB11//ATAD2//MDFIC//HOXA3//HOXD1//ZNF680//ZNF662//NEUROG1//NFE2//NPAS2//IER5//COMMD10//BRWD1//BNC2//MED9//ZNF532//TRERF1//SLC2A4RG//CCNL1//PHTF2//ZBTB26//MIER1//ATXN1//ATXN7//SMARCD2//SS18//MED22//ZNF3//ZNF708//ZNF131//ZNF227//ZNF655//ZSCAN5A//SAP30L//ANP32A//PPP1R1B//PCGF5//LCOR//ST18//HIPK3//PPARGC1B//ELL2//NR5A2//NRBF2//AFF1//MLLT6//PHF20L1//PAK6//RORA//BTG1//CSDE1//SNIP1//TEAD2//RPS6KA5//EMX2//ETS1//BOLA3//NR3C2//RFX7//TRAK2//BDP1//NRBP1//CELF1//GRSF1//RTCA//PPP4R2//CPEB1//QKI//THG1L//AGFG1//NUP54//NXF2//NXF2B//CPEB2//DDX3Y//GTPBP1//DDX6//TSC1//SERP1//RIMKLB//TGM3//YES1//SPPL3//IMMP2L//C8ORF44-SGK3//SGK2//FASTK//PDIK1L//CDK19//MORC3//MMD//SGK3//GRK6//TBK1//CDK17//PIK3CA//PRKACB//CCL8//SGK1//CDK15//TGFBR2//MEX3B//ACVR1//RASSF2//GNAQ//NCK1//GSKIP//YWHAG//PPM1F//PTPN21//PPTC7//PPP1R12A//CTDSPL2//PPP2CB//PPP3R1//PCMTD1//FUT9//B3GALNT2//FUT2//FUT5//ST8SIA5//GFPT1//KCNE1//CCDC126//ALG2//EOGT//GCNT4//VEGFB//WDR45B//ALPI//RECK//PYURF//PIGP//PIGA//AMZ1//ADAMTS17//PRSS55//MEP1A//KLK15//NRIP3//NLN//NPEPL1//ADAM12//CTSF//ADAM18//LMLN//CPD//CASP2//FBXO8//USP27X//BAG6//USP38//GPHN//EGLN3//PMAIP1//CR2//DLL1//G3BP2//IRAK4//PI15//TOB1//RICTOR//PTHLH//CCL21//NANOS1//RAB3GAP1//GJA1//ITGB8//LDLR//MSN//WNT3//WNT10A//CD3E//ANXA7//NDFIP2//CALM2//INHBB//BMP3//TTK//GDF5//SERPINI1//SERPINB13//SPOCK1//PRR16//FXR1//INTS8//GNPTG//FLNA//BAMBI//WNT7A//DNAJB6//RASD1//BTAF1//MBD6//OTUD3//TNFAIP3//WDR20//TIA1//CEP41//STK33//ROR1//OGN//PRELP//ZDHHC22//ZDHHC7//ZDHHC18//ST3GAL5//MCFD2//CRTAP//EGLN1//DCUN1D4//KCNH4//SLK//SULF1//DSEL//SLC35D1//DNAJB9//JKAMP//LEMD3//TNRC6B//ANAPC15//BAG5//MOB1B//RAP2C//RWDD3//CCT6A//ARPP19//MID1//SEMA4C//INSIG2//PFN2//IFNA1//ADCY3//PRKAR2B//PPP1R16B//CNEP1R1//PTPRG//PTP4A1//PTP4A2//CDC14A//FAM83D//MYOG//TDRD5//ARL6IP1//UBXN2B//CD2AP//ARMC8//PLAA//VPS37A//VPS4B//PDE5A//FZD6//YTHDF2//RCAN3//PPP2R5E//CKAP4//CALU//RAB12//TMEFF2//CCNY//ADCYAP1//S1PR1//KPNA6//ARMCX3//CYTL1//PAIP2//BOLL//GRIK2//ULK2//SH3D19//RHEBL1//CCM2//ARRDC3//MTMR9//PMEPA1//LDLRAD4//TMBIM6//ATG16L1//NPNT//GPR55//OTUD1//UBA5//ACSL1//GLRX//CAST//SNAP25//HSBP1L1//FAM168A//KCNK2// |
| GO:0009725 | response to hormone | Biological process | 81 | 977 | 902 | 17653 | 1.62256625218155 | 1.04137506336469e-05 | 0.000382761932257359 | 4.98239282609645 | 0.08980044345898 | CHRM2//GNAQ//APPL1//NR5A2//PPARG//ADIPOR2//ESR1//PPARGC1B//AR//PMEPA1//RB1//CNGA3//DNMT3B//DUSP1//ETS1//FOXA1//OGG1//PTEN//RBBP8//CCND1//SLC6A1//WNT7A//FOSL1//SIRT1//IGF1//INSIG2//SRSF6//TSC1//CPEB2//INHBB//RAB8B//ERRFI1//CPEB1//YWHAG//C2CD5//JUN//ROBO2//SLIT2//UBA5//CBFB//ISL1//TP63//ACSL1//EDN1//FOXP2//CCL21//PIK3CA//NR3C2//NR4A2//RORA//EREG//GJA1//LRP6//BTG1//NCK1//SOCS1//SOCS3//TGFBR2//SDC1//TNF//MAPK1//EP300//FOXP1//WT1//CRHR1//ADCY3//PRKACB//PRKAR2B//PRKAA1//ADCYAP1//H2AFZ//MYOG//POU4F1//MSN//FIBIN//RAP1B//CREBRF//EDNRB//CLOCK//STEAP2//TUB// |
| GO:0072359 | circulatory system development | Biological process | 89 | 1105 | 902 | 17653 | 1.5763030369917 | 1.16417669703633e-05 | 0.000425585243354416 | 4.93398109810201 | 0.098669623059867 | S1PR1//EFNA1//ELK3//EREG//UNC5B//SIRT1//LEMD3//CLIC4//HOXA3//JUN//KDR//RHOB//MEOX2//TNFRSF12A//PIK3CA//PKNOX1//PTEN//RORA//CCL2//VEGFB//WNT7A//CRKL//FOXF1//SPHK2//TGFBR2//RAPGEF2//EDN1//PPP3R1//ACVR1//GJC1//ZFPM2//RASA1//WT1//CCM2//TEAD2//QKI//FBXW7//EFNB2//BMPR2//GJA1//DLL1//RECK//E2F8//SLIT2//ARID2//ISL1//ID2//NOG//DHRS3//NRG1//SOX4//POU4F1//KCNK2//SNX17//DCTN5//LRP6//EP300//GLI3//GNAQ//MEF2A//PPARG//RAF1//ADIPOR2//SPRED1//SULF1//AGO1//FOXJ2//SARS//ETS1//EGLN1//VASH2//F3//ITGB8//BTG1//C5AR1//ADAM12//TSC1//TNNI1//JARID2//PIK3CB//ERRFI1//FOSL1//SOCS3//MAPK1//TNFAIP3//IGF1//IMMP2L//PPP1R16B//RAP1A// |
| GO:0032990 | cell part morphogenesis | Biological process | 60 | 663 | 902 | 17653 | 1.77112700785585 | 1.20893053824375e-05 | 0.000439569743556047 | 4.9175986517646 | 0.0665188470066519 | SLITRK3//OGN//POU4F1//PRELP//WNT7A//SLIT2//RANBP9//ARX//DPYSL2//EFNA1//EFNB2//GLI3//OTX2//PIK3CA//PIK3CB//ENAH//MAPK1//ROBO2//SCN1B//WNT3//KLF7//RPS6KA5//NRXN3//FEZ2//NOG//PLXNA4//NR4A2//PTEN//NFIB//RAB21//ISL1//UNC5B//FZD3//ADNP//NRG1//TNFRSF12A//ULK2//SLC9A6//GJA1//GPM6A//NTF3//SGK1//HPRT1//MEF2A//TRAK2//FMR1//HECW2//FXR1//BMPR2//SEMA4C//POU3F2//SYNGAP1//RAPGEF2//ARC//DOCK10//LRP8//SNX2//SMURF1//SYT1//PLAA// |
| GO:0043549 | regulation of kinase activity | Biological process | 75 | 889 | 902 | 17653 | 1.65109280962939 | 1.23307209351651e-05 | 0.000445950083874447 | 4.90901153094596 | 0.0831485587583149 | GADD45A//GTF2H1//PTEN//CCNT2//DIRAS3//TNF//CRKL//TAOK1//RAF1//RAP1A//C1QTNF2//LPAR1//NRG1//IGF1//NTF3//PIK3CB//PRKAA1//MAPK1//C5AR1//UBE2V1//SPRED1//DUSP1//PPP2CA//EREG//FBXW7//ERRFI1//GPRC5A//SOCS5//GNAQ//LRP6//NCK1//GSKIP//RB1//YWHAG//SOCS1//SOCS3//PPM1F//COPS8//MDFIC//ADNP//PIK3CA//PAK6//TGFBR2//SLK//RICTOR//KDR//MET//ROR1//ADCY3//PRKACB//PRKAR2B//EDN1//PDE5A//HIPK3//NRBF2//CCL21//TNFAIP3//CCNY//CCNL1//CCND1//CCND2//TSC1//ADCYAP1//CACUL1//MMD//RAPGEF2//RASSF2//RAP2C//EFNA1//LRP8//SIRT1//PPP1R1B//ACSL1//IFNG//CALM2// |
| GO:0010648 | negative regulation of cell communication | Biological process | 106 | 1380 | 902 | 17653 | 1.50327613355185 | 1.30690613548545e-05 | 0.00047013862735575 | 4.88375560316512 | 0.117516629711752 | SPRED1//DUSP1//PPP2CA//ERRFI1//GPRC5A//SOCS5//IGFBP3//KDR//RGS7BP//MET//ROR1//RAF1//PRR5L//ACVR1//PMEPA1//LDLRAD4//PTEN//INHBB//GSC//APCDD1//FRZB//HIC1//SHISA2//CCND1//CXXC4//STRAP//SIRT1//LEMD3//FAM89B//BAMBI//SMURF1//PRDM16//TGFBR2//TOB1//CTDSPL2//NOG//PRKAA1//TSC1//SLC6A1//ISL1//TP63//TNFAIP3//DLG5//SULF1//EPS15//SOCS1//SOCS3//ZMYND11//ESR1//RORA//EFNA1//HIPK3//ATXN7//LPAR1//YTHDF2//FBXW7//DLK2//GLI3//RB1//CD3E//NEUROD1//PPP2CB//RASA1//SYNGAP1//NCK1//KCNB1//EDN1//DHRS3//ADNP//CD2AP//SLIT2//ARHGAP12//OTUD3//PHLDA3//GRIK2//CALM2//PPARG//CNOT7//FOXP1//RANBP9//TMEM64//LRP6//PSMA2//PSMD12//FZD6//IGF1//CREBRF//FMR1//EREG//NRG1//RASSF2//PRKACB//BAG5//TMBIM6//DNAJB9//PPP1R15B//SESN1//LYPD6//RAP1A//CLOCK//PHF14//NR4A2//AR//UNC5B//TNF//BCL2A1// |
| GO:0048583 | regulation of response to stimulus | Biological process | 271 | 4243 | 902 | 17653 | 1.24999490487267 | 1.33624066794967e-05 | 0.000478147917319768 | 4.87411531480252 | 0.300443458980044 | GADD45A//TNF//CRKL//TAOK1//RAF1//RAP1A//C1QTNF2//LPAR1//NRG1//IGF1//NTF3//PIK3CB//PRKAA1//MAPK1//C5AR1//UBE2V1//SPRED1//DUSP1//PPP2CA//CBFB//ARL6IP1//MAVS//EP300//PRKACB//PSMA2//PSMD12//SKP1//RPS6KA5//IRAK4//BAG6//CR2//F3//TNFAIP3//PPARG//SLIT2//EDN2//CXCL9//SKAP2//IRF1//SIRT1//ADCYAP1//EREG//FBXW7//ERRFI1//GPRC5A//SOCS5//NCK1//CD3E//COPS8//MDFIC//GRK6//GIT2//OTX2//RORA//RWDD3//YWHAG//TOB1//EPS8//ARHGAP1//LASP1//ARHGAP6//SH3BGRL//HOMER1//IGFBP3//KDR//RGS7BP//MET//ROR1//PRR5L//ACVR1//EDN1//TXLNA//IFNA1//IFNG//IL2//INHBB//OGN//PTHLH//CCL2//CCL8//CCL21//BMP3//VEGFB//WNT3//WNT7A//WNT10A//GDF5//SOCS1//TXK//PPP4R2//MEF2A//PDE5A//JARID2//AKAP11//BMPR2//TTK//PMEPA1//LDLRAD4//SYT1//PPP1R16B//PTEN//UNC5B//MYOG//TNNI1//ZEB1//NREP//SPPL3//DENND1B//TNFRSF21//PIK3CA//ESR1//SULF1//SOX4//GSC//APCDD1//FRZB//HIC1//SHISA2//CCND1//CXXC4//NPNT//STRAP//LEMD3//FAM89B//BAMBI//SMURF1//PRDM16//TGFBR2//CTDSPL2//NOG//EDNRB//FAM83D//TSC1//RICTOR//RRAGD//ARAP2//IQSEC2//APPL1//FBXO8//AGFG1//PSD//RALGPS1//MID1//SEMA4C//UBA5//ISL1//TP63//FOXA1//AR//PLEKHG4B//ARHGEF4//ITSN1//SOS2//GPR55//DLG5//TBK1//ANO1//SYBU//JUN//PPP3R1//YES1//EPS15//CNOT7//SOCS3//ZFAND6//FLNA//GJA1//ATP2C1//NDFIP2//MIER1//UBE2I//ZMYND11//FOXF1//PAK6//SYNGAP1//EFNA1//HIPK3//PMAIP1//ATXN7//PEX5L//ADRB1//RAPGEF2//MYB//NPAS2//NPTN//YTHDF2//DLK2//DLL1//NMI//GLI3//RB1//EVC//RAET1E//GRIK2//RASSF2//NEUROD1//RASGEF1A//PPP2CB//RASA1//SLC25A6//DHRS3//PLXNA4//PCBP2//ELMOD2//FOXP1//ZYX//ETS1//CDK19//LDLR//CLOCK//NPDC1//CD8A//GCSAML//PAX5//EFNB2//PPM1F//ROBO2//S1PR1//RALBP1//RHOB//SYDE2//ARHGAP12//CD2AP//SLC9A6//OTUD3//PHLDA3//CALM2//CCNY//LRP6//TNFRSF12A//SRSF6//RANBP9//RCAN3//ARRDC3//TMEM64//FZD6//GSKIP//TBL1XR1//ZMYND8//LYPD6//C5ORF30//DNAJB6//PTGES3//HSBP1//NUP54//BAG5//IER5//KCNK2//CREBRF//DYNLL2//SESN1//AGO1//OGG1//MDM4//POU4F1//RBBP8//TMBIM6//SERINC3//TRPC3//MYCN//DNAJB9//PPP1R15B//FAM168A//PHF14//ARC//FMR1//NR4A2//CASP2//BCL2A1//ST18//C2CD5// |
| GO:0007264 | small GTPase mediated signal transduction | Biological process | 54 | 578 | 902 | 17653 | 1.82842817575706 | 1.36138383209341e-05 | 0.000484580992444617 | 4.86601941141785 | 0.0598669623059867 | CRKL//IGF1//JUN//USP28//RB1//SYNGAP1//G3BP2//EPS8//RHOB//RND3//ARHGAP1//ARHGAP6//PAK6//ARAP2//IQSEC2//APPL1//FBXO8//AGFG1//PSD//GIT2//RAB12//RAB18//RAB21//RAB30//RAB9B//RAB8B//RAB28//RALGPS1//RAP2C//RAP1A//RAP1B//RAPGEF2//PLEKHG4B//ARHGEF4//RAF1//ITSN1//SOS2//MET//LPAR1//GPR55//RASGEF1A//NRG1//PPP2CB//RASA1//RALBP1//SYDE2//ARHGAP12//CD2AP//SLIT2//RAPGEF4//RGL1//RIN2//DOCK10//DIRAS3// |
| GO:0001885 | endothelial cell development | Biological process | 12 | 54 | 902 | 17653 | 4.34910076373491 | 1.38238904089481e-05 | 0.000489481522700084 | 4.85936971778892 | 0.0133037694013304 | CLIC4//MET//GSTM3//WNT7A//PPP1R16B//MSN//RAP1A//RAP1B//RAPGEF2//RAP2C//TNF//CCM2// |
| GO:0060562 | epithelial tube morphogenesis | Biological process | 35 | 316 | 902 | 17653 | 2.16766889331724 | 1.43964646001084e-05 | 0.000504473005650431 | 4.8417441463198 | 0.0388026607538803 | EDN1//PPP3R1//TGFBR2//ACVR1//NPNT//GLI3//WT1//SOX4//LRP6//SEMA4C//PRKACB//TSC1//FZD3//FZD6//TEAD2//NOG//GJA1//DLL1//CLIC4//MET//MYCN//SLIT2//RDH10//FOXF1//FOXA1//DLG5//AR//ESR1//ETV5//TNF//RHOB//CCM2//KLHL3//EFNB2//FOXP1// |
| GO:0070997 | neuron death | Biological process | 35 | 316 | 902 | 17653 | 2.16766889331724 | 1.43964646001084e-05 | 0.000504473005650431 | 4.8417441463198 | 0.0388026607538803 | EGLN3//TP63//CEBPB//CNTFR//EN1//UNC5B//ADNP//GABRA5//GRIK2//ISL1//JUN//NTF3//NR4A2//PIK3CA//POU4F1//RASA1//CCL2//ITSN1//C5AR1//GDF5//SYNGAP1//MYB//TFAP2A//CASP2//TNFRSF21//RB1//TBK1//EGLN1//TSC1//SIRT1//EFNB2//FBXW7//KCNB1//MEOX2//BAG5// |
| GO:0045860 | positive regulation of protein kinase activity | Biological process | 50 | 522 | 902 | 17653 | 1.8746123981616 | 1.45958084331144e-05 | 0.000508821919758519 | 4.83577184529282 | 0.0554323725055432 | GADD45A//TNF//CRKL//TAOK1//RAF1//RAP1A//C1QTNF2//LPAR1//NRG1//IGF1//NTF3//PIK3CB//PRKAA1//MAPK1//C5AR1//UBE2V1//EREG//FBXW7//COPS8//MDFIC//SOCS1//ADNP//PIK3CA//PAK6//TGFBR2//SLK//RICTOR//ADCY3//PRKACB//PRKAR2B//EDN1//PDE5A//CCNY//CCNL1//CCND1//CCND2//CCNT2//EFNA1//LRP8//ACSL1//IFNG//PPP2CA//CALM2//SIRT1//RAPGEF2//ADCYAP1//CACUL1//MMD//CCL21//RASSF2// |
| GO:0023057 | negative regulation of signaling | Biological process | 106 | 1384 | 902 | 17653 | 1.49893140484216 | 1.4786034216275e-05 | 0.000512809996947014 | 4.83014829313903 | 0.117516629711752 | SPRED1//DUSP1//PPP2CA//ERRFI1//GPRC5A//SOCS5//IGFBP3//KDR//RGS7BP//MET//ROR1//RAF1//PRR5L//ACVR1//PMEPA1//LDLRAD4//PTEN//INHBB//GSC//APCDD1//FRZB//HIC1//SHISA2//CCND1//CXXC4//STRAP//SIRT1//LEMD3//FAM89B//BAMBI//SMURF1//PRDM16//TGFBR2//TOB1//CTDSPL2//NOG//PRKAA1//TSC1//SLC6A1//ISL1//TP63//TNFAIP3//DLG5//SULF1//EPS15//SOCS1//SOCS3//ZMYND11//ESR1//RORA//EFNA1//HIPK3//ATXN7//LPAR1//YTHDF2//FBXW7//DLK2//GLI3//RB1//CD3E//NEUROD1//PPP2CB//RASA1//SYNGAP1//NCK1//KCNB1//EDN1//DHRS3//ADNP//CD2AP//SLIT2//ARHGAP12//OTUD3//PHLDA3//GRIK2//CALM2//PPARG//CNOT7//FOXP1//RANBP9//TMEM64//LRP6//PSMA2//PSMD12//FZD6//IGF1//CREBRF//FMR1//EREG//NRG1//RASSF2//PRKACB//BAG5//TMBIM6//DNAJB9//PPP1R15B//SESN1//LYPD6//RAP1A//CLOCK//PHF14//NR4A2//AR//UNC5B//TNF//BCL2A1// |
| GO:0045666 | positive regulation of neuron differentiation | Biological process | 37 | 343 | 902 | 17653 | 2.11115241155062 | 1.49812474313073e-05 | 0.000516929471315976 | 4.82445202308663 | 0.041019955654102 | STMN2//ADCYAP1//NPTN//NCK1//SERPINI1//RAP1A//SCN1B//RAPGEF2//ADNP//NRG1//TNFRSF12A//WNT3//BMPR2//ROBO2//PLXNA4//SLIT2//RAB21//LPAR1//CPEB3//FMR1//ZMYND8//IL2//MAPK6//DLG5//LRP8//SMURF1//SYT1//PLAA//DNMT3B//ETV5//MMD//FOXA1//NEUROD1//NEUROG1//PTEN//ZEB1//GDF5// |
| GO:0051246 | regulation of protein metabolic process | Biological process | 203 | 3033 | 902 | 17653 | 1.3098923665255 | 1.52360261364881e-05 | 0.000522946684145681 | 4.81712829100434 | 0.225055432372506 | GADD45A//GTF2H1//PTEN//CCNT2//DIRAS3//TNF//CRKL//TAOK1//RAF1//RAP1A//C1QTNF2//LPAR1//NRG1//IGF1//NTF3//PIK3CB//PRKAA1//MAPK1//C5AR1//UBE2V1//SPRED1//DUSP1//PPP2CA//SEPSECS//SECISBP2L//SOCS1//IGFBP3//IL2//LRP6//PRR5L//PPP1R15B//SLIT2//EDNRB//EFNA1//FMR1//SIRT1//NPTN//IFNG//KDR//MAVS//CCND1//CCND2//EREG//FBXW7//ERRFI1//GPRC5A//SOCS5//DDX6//TSC1//KBTBD8//GNAQ//NCK1//GSKIP//RB1//YWHAG//SOCS3//PPM1F//EGLN3//F3//PMAIP1//PPARG//CASP2//COPS8//MDFIC//AGO1//SNIP1//PI15//CCL21//CALM2//ADNP//INHBB//BMP3//BMPR2//TTK//GDF5//ACVR1//SERPINI1//SERPINB13//SPOCK1//RECK//CPEB3//SERP1//PRR16//SOX4//FXR1//TOB1//CELF1//CNOT7//NANOS1//PURA//TIA1//MEX3B//SPOPL//CR2//JARID2//TNFAIP3//BAG5//NDFIP2//MOB1B//JUN//RAP2C//RASSF2//PIK3CA//PAK6//TGFBR2//SLK//RICTOR//EDN1//WNT7A//BAG6//ANKIB1//LONRF3//LONRF1//ARPP19//PPP1R1B//MYLIP//MID1//SEMA4C//RPS6KA5//CREBL2//TBK1//PFN2//IFNA1//RWDD3//ADCY3//PRKACB//PRKAR2B//ISL1//CNOT6//TNRC6B//PPP1R16B//SPPL3//CNEP1R1//PPP1R12A//FAM83D//FLNA//MDM4//DNAJB6//ARL6IP1//TP63//PDE5A//SYNGAP1//AR//MET//ROR1//HIPK3//RCAN3//UBXN2B//PPP4R2//PPP2R5E//IRF1//CD3E//NMI//CEBPB//GJA1//SMURF1//HECW2//CCNY//CCNL1//ADCYAP1//CACUL1//MMD//RAPGEF2//PAIP2//BOLL//GRIK2//ZMYND11//YES1//LRP8//SH3D19//LDLR//ZER1//DCUN1D4//ARRDC3//DNMT3B//MYB//PAX5//STRAP//PMEPA1//LDLRAD4//NOG//NUP54//CNOT6L//YTHDF2//QKI//RANBP9//NPNT//CCL2//CCL8//GPR55//ACSL1//EP300//CAST//PLAA//CPEB2//CRTAP//RAB3GAP1//MSN//UBE2I//ESR1//MYCN//CPEB1//ST18// |
| GO:0051961 | negative regulation of nervous system development | Biological process | 32 | 278 | 902 | 17653 | 2.25277161862528 | 1.5310282930777e-05 | 0.000522946684145681 | 4.81501678355779 | 0.0354767184035477 | STMN2//LPAR1//EFNB2//MYLIP//PTPRG//SPOCK1//TSC1//BAG5//EDNRB//TNFRSF21//PTEN//DLL1//DDX6//GLI3//ID2//ISL1//ULK2//MYCN//NOG//SEMA4C//WNT3//WNT7A//TRAK2//SYNGAP1//RAPGEF2//ROBO2//ADCYAP1//EFNA1//LDLR//SLIT2//PRKACB//NRG1// |
| GO:0022604 | regulation of cell morphogenesis | Biological process | 46 | 468 | 902 | 17653 | 1.92364072242121 | 1.67054121234003e-05 | 0.00056773217181184 | 4.77714280582586 | 0.0509977827050998 | CFDP1//WASF3//PALM2//LPAR1//EPS8//BAMBI//ANXA7//KDR//RHOB//RND3//MSN//BRWD1//RASA1//CCL2//PLXNA4//WDR1//DPYSL2//RAB21//WNT7A//ADNP//NRG1//TNFRSF12A//ULK2//WNT3//FMR1//HECW2//FXR1//BMPR2//SEMA4C//EFNA1//POU3F2//PTEN//TRAK2//SYNGAP1//ROBO2//SLIT2//RAPGEF2//ARC//LRP8//CRKL//FLNA//SMURF1//SYT1//PLAA//SH3D19//CAMSAP1// |
| GO:1902531 | regulation of intracellular signal transduction | Biological process | 137 | 1901 | 902 | 17653 | 1.41042641811813 | 1.70268118553525e-05 | 0.000575761642888745 | 4.76886666285638 | 0.151884700665188 | GADD45A//TNF//CRKL//TAOK1//RAF1//RAP1A//C1QTNF2//LPAR1//NRG1//IGF1//NTF3//PIK3CB//PRKAA1//MAPK1//C5AR1//UBE2V1//SPRED1//DUSP1//PPP2CA//COPS8//MDFIC//SOCS1//AKAP11//PPP1R16B//PTEN//UNC5B//SIRT1//KDR//PRR5L//FAM83D//TSC1//RICTOR//PIK3CA//RRAGD//ARAP2//IQSEC2//APPL1//FBXO8//AGFG1//PSD//GIT2//RALGPS1//MID1//SEMA4C//IFNA1//IFNG//PLEKHG4B//EPS8//ARHGEF4//ITSN1//SOS2//MET//GPR55//DLG5//CNOT7//IL2//ISL1//SOCS3//ZFAND6//FLNA//GJA1//ATP2C1//TBK1//ROR1//IRAK4//NDFIP2//MAVS//MIER1//CCL21//UBE2I//ZMYND11//ESR1//RORA//TNFAIP3//EDN1//PDE5A//INHBB//PAK6//BMP3//GDF5//SYNGAP1//EFNA1//IGFBP3//AR//HIPK3//HIC1//PMAIP1//PEX5L//ADCYAP1//ADRB1//CXCL9//RAPGEF2//GRIK2//WNT7A//RASSF2//NEUROD1//SOCS5//RASGEF1A//PPP2CB//RASA1//CD3E//RALBP1//RHOB//ARHGAP1//ARHGAP6//SYDE2//ARHGAP12//CD2AP//SLIT2//EREG//F3//OTUD3//PHLDA3//CALM2//RANBP9//ERRFI1//NPNT//NPTN//JUN//FBXW7//CCL2//CCL8//RCAN3//SPPL3//AGO1//EP300//MDM4//POU4F1//RBBP8//TP63//BAG5//TMBIM6//SERINC3//NCK1//SESN1//BCL2A1//LEMD3// |
| GO:0009967 | positive regulation of signal transduction | Biological process | 118 | 1586 | 902 | 17653 | 1.45609867940936 | 1.75875880685925e-05 | 0.000591765463223339 | 4.75479371485312 | 0.130820399113082 | GADD45A//TNF//CRKL//TAOK1//RAF1//RAP1A//C1QTNF2//LPAR1//NRG1//IGF1//NTF3//PIK3CB//PRKAA1//MAPK1//C5AR1//UBE2V1//EREG//FBXW7//COPS8//MDFIC//SOCS1//F3//INHBB//BMP3//BMPR2//TTK//GDF5//ACVR1//UNC5B//SIRT1//KDR//PRR5L//SULF1//SOX4//WNT3//NPNT//RICTOR//PIK3CA//RRAGD//MID1//SEMA4C//FOXA1//AR//GPR55//DLG5//IFNG//IL2//ISL1//SOCS3//FLNA//GJA1//ATP2C1//TBK1//ROR1//IRAK4//NDFIP2//MAVS//MIER1//CCL21//UBE2I//EDN1//PDE5A//EFNA1//IGFBP3//MET//SPRED1//HIC1//PMAIP1//ADCYAP1//ADRB1//CXCL9//RAPGEF2//NPTN//EP300//DLL1//TP63//EVC//WNT7A//RASSF2//RASGEF1A//CD3E//SOS2//ESR1//ITSN1//CALM2//TXK//JUN//PTEN//CCL2//CCL8//SPPL3//ARRDC3//BAMBI//LRP6//GSKIP//PSMA2//PSMD12//TBL1XR1//DYNLL2//PPP3R1//YWHAG//AGO1//RWDD3//CREBRF//SERINC3//NCK1//ARC//CASP2//TNFRSF12A//ST18//TOB1//EPS8//ARHGAP1//LASP1//ARHGAP6//SH3BGRL//SKAP2//HOMER1// |
| GO:0044267 | cellular protein metabolic process | Biological process | 334 | 5421 | 902 | 17653 | 1.20581044971289 | 1.85410915428508e-05 | 0.000620759416357921 | 4.73186470184834 | 0.370288248337029 | GADD45A//GTF2H1//PTEN//CCNT2//DIRAS3//RANBP9//SPRED1//EREG//RASGEF1A//NRG1//IL2//MEF2A//MET//MAPK1//MAPK6//PSMA2//PSMD12//RAF1//RASA1//CCL2//TNF//SYNGAP1//RAPGEF2//CRKL//TAOK1//RAP1A//C1QTNF2//LPAR1//IGF1//NTF3//PIK3CB//PRKAA1//C5AR1//UBE2V1//DUSP1//PPP2CA//FBXO41//RNF145//ZNRF2//RNF167//MYLIP//ANKIB1//UBE2W//FBXW7//SMURF1//HECW2//KLHL42//SKP1//THOP1//LONRF3//FBXO30//CBFB//UBE3B//LONRF1//FBXO44//COPS8//NKTR//SEPSECS//SECISBP2L//SOCS1//IGFBP3//LRP6//PRR5L//PPP1R15B//SLIT2//EDNRB//EFNA1//FMR1//SIRT1//NPTN//IFNG//KDR//MAVS//CCND1//CCND2//ABHD17C//ERRFI1//GPRC5A//SOCS5//ZER1//CACUL1//RNF38//MARCH10//PDZRN3//MYCBP2//FBXO33//KLHL3//RNF11//KLHL20//SPOPL//TRIM23//MDM4//DCAF8//CBLL1//CUL5//KBTBD8//SOCS3//CPEB2//CPEB3//SARS//CPEB1//DDX3Y//GTPBP1//DDX6//TSC1//PPP4R2//SERP1//RIMKLB//TGM3//UBE2I//YES1//SPPL3//IMMP2L//C8ORF44-SGK3//SGK2//HIPK3//FASTK//PDIK1L//CDK19//MORC3//MMD//SGK3//PAN3//GRK6//TBK1//NRBP1//CDK17//PIK3CA//PRKACB//CCL8//SGK1//CDK15//TGFBR2//TXK//MEX3B//RUNX3//ACVR1//RPS6KA5//RASSF2//GNAQ//NCK1//GSKIP//RB1//YWHAG//PPM1F//PTPN21//PPTC7//PPP1R12A//CTDSPL2//PPP2CB//PPP3R1//EP300//CLOCK//HS3ST5//PCMTD1//BTG1//FUT9//B3GALNT2//FUT2//FUT5//ST8SIA5//GFPT1//KCNE1//CCDC126//ALG2//EOGT//GCNT4//TRAK2//VEGFB//WDR45B//ALPI//RECK//PYURF//PIGP//PIGA//CTSF//FBXO8//USP27X//USP28//BAG6//USP38//GPHN//EGLN3//F3//PMAIP1//PPARG//CASP2//DLL1//IRAK4//MDFIC//PI15//CALM2//ADNP//INHBB//BMP3//BMPR2//TTK//GDF5//SERPINI1//SERPINB13//SPOCK1//PRR16//SOX4//FXR1//EPC2//SIN3B//ARID4B//MIER1//SAP30L//TBL1XR1//ARID5B//ATXN7//MBD6//ESR1//OTUD3//AR//TNFAIP3//WDR20//NUP54//TOB1//CELF1//CNOT7//NANOS1//PURA//TIA1//CEP41//RICTOR//STK33//ROR1//ZDHHC22//ZDHHC7//ZDHHC18//ST3GAL5//MCFD2//CRTAP//EGLN1//DCUN1D4//KCNH4//PAK6//SLK//DNAJB9//JKAMP//JARID2//ANAPC15//BAG5//NDFIP2//MOB1B//JUN//RAP2C//EDN1//WNT7A//ARPP19//PPP1R1B//MID1//SEMA4C//CREBL2//PFN2//IFNA1//PHF8//RWDD3//ADCY3//PRKAR2B//ESCO2//PRDM16//ISL1//CNOT6//TNRC6B//AGO1//PPP1R16B//CNEP1R1//PTPRG//PTP4A1//PTP4A2//CDC14A//RNF2//SUV39H1//PCGF5//DNAJB6//ARL6IP1//UBXN2B//CD2AP//ARMC8//PCBP2//PLAA//VPS37A//VPS4B//PDE5A//RCAN3//PPP2R5E//CKAP4//CALU//RAB12//CCNY//CCNL1//ADCYAP1//CCL21//PAIP2//BOLL//GRIK2//ZMYND11//ULK2//LRP8//CD3E//SH3D19//CCM2//ARRDC3//DNMT3B//MYB//PAX5//STRAP//PMEPA1//LDLRAD4//NOG//CNOT6L//YTHDF2//QKI//ATG16L1//FAM83D//NPNT//GPR55//OTUD1//KDM2A//UBA5//ACSL1//GLRX//CAST//NMI//RAB3GAP1//MSN//ZMYND8//MYCN//LDLR//ST18// |
| GO:0061014 | positive regulation of mRNA catabolic process | Biological process | 10 | 39 | 902 | 17653 | 5.0181931889249 | 1.89289019015568e-05 | 0.000630621495370584 | 4.7228745794819 | 0.0110864745011086 | TOB1//CPEB3//TNRC6B//CNOT7//CNOT6L//YTHDF2//QKI//NANOS1//PRR5L//GTPBP1// |
| GO:0048608 | reproductive structure development | Biological process | 43 | 430 | 902 | 17653 | 1.95709534368071 | 2.07031690399081e-05 | 0.000679687049596595 | 4.68396317187619 | 0.0476718403547672 | CEBPB//ADCYAP1//INHBB//IMMP2L//SIRT1//ESR1//EREG//CASP2//PPARG//E2F8//STOX2//BMPR2//ZFPM2//RDH10//WT1//RNF38//CSDE1//ARID5B//GLI3//AR//MGST1//CCND1//NEUROG1//LRP6//GJA1//FOXF2//TP63//SDC1//WNT7A//NOG//FOXA1//SOCS3//ITGB8//FOSL1//SULF1//DNAJB6//EGLN1//MAPK1//VASH2//PTEN//ROBO2//SLIT2//ARID4B// |
| GO:0048732 | gland development | Biological process | 43 | 430 | 902 | 17653 | 1.95709534368071 | 2.07031690399081e-05 | 0.000679687049596595 | 4.68396317187619 | 0.0476718403547672 | JARID2//JUN//MET//PIK3CA//ARID5B//CCND1//ADCYAP1//ETS1//ISL1//NOG//POU3F2//MSN//WT1//GLI3//AR//HOXA3//MAPK1//RAF1//NRG1//IGSF3//CEBPB//ID2//CRKL//WNT10A//GJA1//TGFBR2//LRP6//TP63//ESR1//FOXA1//NFIB//TNF//SULF1//BTBD7//PTEN//ETV5//FOXF1//WNT3//E2F8//FRZB//RAP1A//FBXW7//TNFAIP3// |
| GO:0050769 | positive regulation of neurogenesis | Biological process | 43 | 430 | 902 | 17653 | 1.95709534368071 | 2.07031690399081e-05 | 0.000679687049596595 | 4.68396317187619 | 0.0476718403547672 | GLI3//FZD3//STMN2//ADCYAP1//NPTN//NCK1//SERPINI1//RAP1A//SCN1B//RAPGEF2//DNMT3B//ETV5//MMD//FOXA1//NEUROD1//NEUROG1//PTEN//ZEB1//GDF5//ADNP//NRG1//TNFRSF12A//WNT3//ID2//PPARG//BMPR2//ROBO2//PLXNA4//SLIT2//RAB21//MYB//LPAR1//CPEB3//FMR1//ZMYND8//IL2//MAPK6//DLG5//LRP8//SMURF1//SYT1//PLAA//FLNA// |
| GO:0031331 | positive regulation of cellular catabolic process | Biological process | 36 | 335 | 902 | 17653 | 2.10314723500017 | 2.11377498131906e-05 | 0.00069060194196429 | 4.67494124672245 | 0.0399113082039911 | IFNG//PIK3CB//PRKAA1//ABHD5//RAB12//SIRT1//SESN1//TBK1//KDR//TSC1//ANKIB1//LONRF3//LONRF1//SOCS5//IGF1//SH3D19//TNF//TOB1//CPEB3//TNRC6B//CNOT7//PRR5L//GTPBP1//CNOT6L//YTHDF2//QKI//SMURF1//NANOS1//FMR1//FBXW7//MSN//TNFAIP3//BAG6//LDLR//PTEN//RAB3GAP1// |
| GO:0006793 | phosphorus metabolic process | Biological process | 220 | 3352 | 902 | 17653 | 1.28448978403865 | 2.21146695579601e-05 | 0.000719045722213866 | 4.65531954556531 | 0.24390243902439 | GADD45A//GTF2H1//PTEN//CCNT2//DIRAS3//RANBP9//SPRED1//EREG//RASGEF1A//NRG1//IL2//MEF2A//MET//MAPK1//MAPK6//PSMA2//PSMD12//RAF1//RASA1//CCL2//TNF//SYNGAP1//RAPGEF2//CRKL//TAOK1//RAP1A//C1QTNF2//LPAR1//IGF1//NTF3//PIK3CB//PRKAA1//C5AR1//UBE2V1//DUSP1//PPP2CA//SOCS1//IGFBP3//LRP6//PRR5L//PPP1R15B//SLIT2//EDNRB//EFNA1//FMR1//SIRT1//NPTN//IFNG//KDR//MAVS//CCND1//CCND2//FBXW7//ERRFI1//GPRC5A//SOCS5//GFPT1//SLC35D1//NUP54//COX8C//HPRT1//CMPK1//ADSS//ADCY3//IMPDH1//C8ORF44-SGK3//SGK2//HIPK3//FASTK//PDIK1L//CDK19//MORC3//MMD//SGK3//PAN3//GRK6//TBK1//NRBP1//CDK17//PIK3CA//PRKACB//CCL8//SGK1//CDK15//TGFBR2//TXK//MEX3B//RUNX3//ACVR1//RPS6KA5//RASSF2//GNAQ//NCK1//GSKIP//RB1//YWHAG//SOCS3//PPM1F//PTPN21//PPP4R2//PPTC7//PPP1R12A//CTDSPL2//PPP2CB//PPP3R1//PYURF//PIGP//PIGA//PLAA//ALPI//LCLAT1//ABHD5//CDS1//ABHD3//SLC44A1//SERINC3//MTMR9//SPHK2//GPHN//PRUNE2//BNIP2//GRM3//COPS8//IRAK4//MDFIC//PTHLH//LPCAT3//DGKE//CRHR1//PPARGC1B//NPNT//CALM2//INHBB//BMP3//BMPR2//TTK//GDF5//LDLR//FKBP15//PLEKHF2//MCCC2//DGKH//HDHD2//RICTOR//STK33//ROR1//KCNH4//PAK6//SLK//TBL1XR1//CXCL9//ADRB1//EGLN1//PDE5A//ADNP//MOB1B//JUN//RAP2C//ARPP19//PPP1R1B//MID1//SEMA4C//CREBL2//PFN2//IFNA1//PRKAR2B//PPP1R16B//SPPL3//CNEP1R1//PTPRG//PTP4A1//PTP4A2//CDC14A//ACSL1//ACSL4//ELOVL5//ELOVL7//YES1//ATXN7//PTGES3//AR//ITSN1//CNOT7//ISL1//EDN1//NRBF2//CCL21//RCAN3//UBXN2B//PPP2R5E//TSC1//SMUG1//OGG1//TNFAIP3//CCNY//CCNL1//MYOG//ADCYAP1//CACUL1//GTPBP1//RORA//GRIK2//ZMYND11//WNT7A//GPCPD1//ULK2//GNPTG//ESR1//EFR3A//LRP8//CD3E//SKP1//CCM2//STRAP//PMEPA1//LDLRAD4//NOG//FAM83D//GPR55//KCNAB2// |
| GO:0071495 | cellular response to endogenous stimulus | Biological process | 103 | 1350 | 902 | 17653 | 1.49319126221565 | 2.31305874346933e-05 | 0.000745138930552207 | 4.63581333755337 | 0.114190687361419 | SIRT1//JUN//SMURF1//TGFBR2//KLF10//ZYX//GDF5//ACVR1//CHRM2//GNAQ//APPL1//SPRED1//MAPK1//TIA1//SHCBP1//NR5A2//PPARG//ADIPOR2//ZEB1//NREP//CEBPB//CPEB3//SESN1//RRAGD//CPEB1//TNF//SOCS1//TOB1//BMP3//BMPR2//NOG//NPNT//STRAP//LEMD3//FAM89B//BAMBI//PMEPA1//PRDM16//LDLRAD4//SULF1//CTDSPL2//ESR1//PPARGC1B//AR//RB1//CPEB2//INHBB//RAB8B//ERRFI1//PTEN//YWHAG//C2CD5//DUSP1//ROBO2//SLIT2//UBA5//CBFB//ISL1//TP63//FOXA1//ACSL1//PIK3CA//YES1//NTF3//RAP1A//RAPGEF2//OTX2//SHISA2//NR3C2//NR4A2//RORA//CCL2//NPTN//NCK1//TSC1//SOCS3//IGF1//EP300//FOXP1//KCNE1//RAP1B//WT1//GJA1//EDN1//CRHR1//ADCY3//PRKACB//PRKAR2B//PRKAA1//ADCYAP1//H2AFZ//MYOG//POU4F1//MSN//DNMT3B//SOX5//WNT7A//WNT10A//CREBRF//KLF3//STMN2//DYNC1LI2//CLOCK// |
| GO:0051216 | cartilage development | Biological process | 24 | 184 | 902 | 17653 | 2.55273305697484 | 2.31375388756415e-05 | 0.000745138930552207 | 4.6356828385378 | 0.0266075388026608 | MYCN//WNT7A//SNX19//NFIB//CYTL1//GDF5//RUNX3//SULF1//BMPR2//TGFBR2//MAF//PTHLH//GLI3//SOX5//LRP6//TAPT1//FRZB//NOG//EDN1//EVC//HOXA3//ITGB8//BMP3//ZEB1// |
| GO:0061458 | reproductive system development | Biological process | 43 | 433 | 902 | 17653 | 1.94353579164597 | 2.44761358708655e-05 | 0.000784512354951011 | 4.61125714463198 | 0.0476718403547672 | CEBPB//ADCYAP1//INHBB//IMMP2L//SIRT1//ESR1//EREG//CASP2//PPARG//E2F8//STOX2//BMPR2//ZFPM2//RDH10//WT1//RNF38//CSDE1//ARID5B//GLI3//AR//MGST1//CCND1//NEUROG1//LRP6//GJA1//FOXF2//TP63//SDC1//WNT7A//NOG//FOXA1//SOCS3//ITGB8//FOSL1//SULF1//DNAJB6//EGLN1//MAPK1//VASH2//PTEN//ROBO2//SLIT2//ARID4B// |
| GO:0010720 | positive regulation of cell development | Biological process | 48 | 504 | 902 | 17653 | 1.86390032731496 | 2.5015303889287e-05 | 0.000798011793411547 | 4.60179421688056 | 0.0532150776053215 | GLI3//FZD3//STMN2//ADCYAP1//NPTN//NCK1//SERPINI1//RAP1A//SCN1B//RAPGEF2//DNMT3B//ETV5//MMD//FOXA1//NEUROD1//NEUROG1//PTEN//ZEB1//GDF5//ADNP//NRG1//TNFRSF12A//WNT3//ID2//PPARG//MYOG//BMPR2//ROBO2//PLXNA4//SLIT2//RAB21//MYB//PDE5A//EDN1//LPAR1//CPEB3//FMR1//ZMYND8//IL2//MAPK6//DLG5//LRP8//IGF1//CRKL//FLNA//SMURF1//SYT1//PLAA// |
| GO:1901576 | organic substance biosynthetic process | Biological process | 399 | 6665 | 902 | 17653 | 1.17161446680961 | 2.54775569756956e-05 | 0.000808942337214222 | 4.59384221852043 | 0.442350332594235 | SIRT1//IGF1//NFIA//NFIB//NFIC//RBBP8//RBMS1//NPAT//TSHZ1//ZBTB18//CEBPB//TCERG1//STRAP//KLF12//GSC//ARX//DNMT3B//EDN1//EDNRB//EFNA1//CC2D1B//EN1//EP300//ESR1//JAZF1//CPEB3//FOXF1//MYT1L//SIN3B//ZFPM2//DNAJB5//GLI3//CNOT7//HIC1//FOXA1//HSBP1//ID2//IFNG//IRF2//AR//ISL1//JARID2//JUN//MAF//MDM4//MEF2A//MYB//NR4A2//PAX5//KLF3//POU4F1//PPARG//ZBTB4//RB1//CCND1//RNF2//SARS//PRDM16//SMARCA2//SUV39H1//ZEB1//TFAP2A//TGIF1//KLF10//TNF//UBE2I//WT1//ZNF217//LRP8//BHLHE41//TBL1XR1//E2F8//ARID5B//BHLHE40//TP63//RUNX3//NOG//FOXP2//PHF14//GTF2E1//SEPSECS//SECISBP2L//PTGES3//EDN2//MGST1//ST3GAL5//RDH10//GTF2H1//CREBRF//ELK3//ETV5//ATF5//FOXF2//FLI1//GTF2A1//HIVEP2//IRF1//MEOX2//MYBL1//MYCN//NEUROD1//NHLH2//OTX2//KLF13//PKNOX1//POU3F2//FOXJ2//HIVEP3//BMPR2//SOX4//SOX5//BTF3//TFE3//TXK//FOSL1//KLF7//CBFB//FUBP3//CCNT2//NMI//CLOCK//ZNF516//POLR3H//SRSF2//SRSF6//SLBP//HS3ST5//SDC1//HS3ST3A1//HS3ST1//GFPT1//SLC35D1//NUP54//NLN//HPRT1//ADSS//ADCY3//IMPDH1//CMPK1//PURA//GMNC//ESCO2//PPP2CA//BAHD1//H2AFZ//ZMYND11//PNRC1//ZNF526//CREBL2//ZNF800//E2F5//ZNF367//ARID2//MED19//ZFP30//KDM2A//MYCBP2//PHF8//FLII//ADNP//ZMYND8//CNOT6L//ZNF521//EPC2//AGO1//HBP1//FOXP1//ZBTB11//ATAD2//MDFIC//HMGB3//HOXA3//HOXD1//ZNF680//ZNF662//NEUROG1//NFE2//NPAS2//ASCC1//IER5//COMMD10//ARID4B//BRWD1//BNC2//MED9//ZNF532//PRKAA1//TRERF1//MAPK1//SLC2A4RG//CCNL1//PHTF2//CNOT6//ZBTB26//MIER1//ATXN1//ATXN7//SMARCD2//SS18//MED22//ZNF3//ZNF708//ZNF131//ZNF227//ZNF655//ZSCAN5A//SAP30L//ANP32A//PPP1R1B//PCGF5//LCOR//ST18//HIPK3//PPARGC1B//ELAVL2//ELL2//NR5A2//NRBF2//AFF1//MLLT6//OGG1//PHF20L1//PAK6//RORA//BTG1//UBE2V1//CSDE1//SNIP1//TEAD2//RPS6KA5//EMX2//ETS1//BOLA3//NR3C2//RFX7//TRAK2//BDP1//NRBP1//CPEB2//CPEB1//DDX3Y//GTPBP1//DDX6//TSC1//KBTBD8//FUT9//B3GALNT2//FUT2//FUT5//SERP1//ST8SIA5//KCNE1//CCDC126//ALG2//EOGT//GCNT4//VEGFB//WDR45B//ALPI//RECK//PYURF//PIGP//PIGA//SERINC3//PSAT1//ELOVL5//LCLAT1//ABHD5//CDS1//ABHD3//SLC44A1//PIK3CA//PIK3CB//PTEN//MTMR9//SPHK2//ACBD3//LBR//INSIG2//MSMO1//GPHN//GRM3//G3BP2//ACSL1//RIMKLB//LPCAT3//DGKE//MTRR//GLT6D1//EREG//CCL21//CRHR1//CNEP1R1//LDLR//FBXW7//ARPP19//FMR1//PRR16//FXR1//FLNA//BAMBI//LRP6//WNT7A//ACVR1//DNAJB6//NRG1//RASD1//BTAF1//PPM1F//TOB1//CELF1//NANOS1//TIA1//MEX3B//OGN//PRELP//CHST1//ZDHHC22//ZDHHC7//ZDHHC18//MCFD2//ABHD17C//ELOVL7//SPTSSB//SPTSSA//DSEL//SMURF1//BMP3//GDF5//SULF1//LEMD3//CTDSPL2//ADRB1//PTHLH//CALM2//EGLN1//RWDD3//TNFAIP3//DCP2//CCT6A//TNRC6B//ACSL4//NCK1//ADIPOR2//ERRFI1//QKI//MYOG//C5AR1//INTS8//NABP1//FZD6//SMUG1//CD3E//TBK1//TMEFF2//MID1IP1//C1QTNF2//ADCYAP1//S1PR1//KPNA6//DLL1//IL2//MET//PPP1R12A//ARMCX3//CYTL1//PPP3R1//MAVS//RAF1//YES1//PAIP2//BOLL//PDE5A//RAPGEF2//INHBB//C8ORF44-SGK3//SGK3//SGK1//RHEBL1//ROR1//IRAK4//KDR//CCL2//YTHDF2//EGLN3//PSMA2//PSMD12//ATG16L1//GSTA2//GSTM3//RAB3GAP1//RAP2C//HSBP1L1//PPP1R15B//TMBIM6//DUSP1//GJA1//KCNK2//NPNT// |
| GO:1901698 | response to nitrogen compound | Biological process | 85 | 1065 | 902 | 17653 | 1.56200097852451 | 2.58173580332746e-05 | 0.000815900898967459 | 4.58808820243661 | 0.0942350332594235 | HPRT1//NR4A2//PPP1R1B//CHRM2//GNAQ//APPL1//HOMER1//AGO1//SNIP1//MGST1//CCND1//ADSS//SLC6A1//CEBPB//CPEB3//SESN1//RRAGD//CPEB1//ZEB1//TNF//SOCS1//TSN//DNAJB9//JKAMP//BAG6//DNMT3B//PPARG//PRKAA1//C5AR1//TNFAIP3//SIRT1//IGF1//INSIG2//SRSF6//TSC1//CPEB2//INHBB//RAB8B//ERRFI1//PTEN//YWHAG//C2CD5//TRPC3//PIK3CA//EDN1//GLRB//GLRA3//IFNA1//MAPK1//PMAIP1//EREG//GJA1//LRP6//BTG1//NCK1//SOCS3//CNGA3//PPARGC1B//DUSP1//JUN//SDC1//FOSL1//OGG1//GSTM3//KCNE1//RAP1A//RAP1B//WT1//RAPGEF2//MAVS//CRHR1//ADCY3//PRKACB//PRKAR2B//LDLR//SOCS5//SLIT2//EGLN1//FMR1//KLF3//KLF10//TMBIM6//ESR1//MYCN//EDNRB// |
| GO:0060322 | head development | Biological process | 64 | 741 | 902 | 17653 | 1.6903387583747 | 2.67723925093037e-05 | 0.000839608357289826 | 4.57231281644251 | 0.0709534368070953 | DPYSL2//S1PR1//RAB3GAP1//RAB18//PHF8//GABRA5//SPHK2//PTPRG//ROBO2//ATXN1//SLC6A11//BMPR2//SYT1//TGFBR2//BAG6//CASP2//IMMP2L//NOG//C5AR1//POU4F1//SEMA4C//LRP8//EMX2//NEUROD1//PTEN//LPAR1//EN1//CD3E//FOXP2//LRP6//RAPGEF2//PAX5//DLL1//RORA//WNT7A//NFIB//HPRT1//ARX//CALM2//GLI3//TSC1//ID2//POU3F2//SLIT2//NRG1//ETS1//GNAQ//ATF5//FLNA//PLXNA4//ADCYAP1//ISL1//NR4A2//TACC2//GSC//OTX2//DLG5//FZD3//FZD6//WNT3//MAPK1//RAF1//ARID5B//NANOS1// |
| GO:0006796 | phosphate-containing compound metabolic process | Biological process | 214 | 3255 | 902 | 17653 | 1.28669248401742 | 2.68158221461781e-05 | 0.000839608357289826 | 4.57160888346411 | 0.237250554323725 | GADD45A//GTF2H1//PTEN//CCNT2//DIRAS3//RANBP9//SPRED1//EREG//RASGEF1A//NRG1//IL2//MEF2A//MET//MAPK1//MAPK6//PSMA2//PSMD12//RAF1//RASA1//CCL2//TNF//SYNGAP1//RAPGEF2//CRKL//TAOK1//RAP1A//C1QTNF2//LPAR1//IGF1//NTF3//PIK3CB//PRKAA1//C5AR1//UBE2V1//DUSP1//PPP2CA//SOCS1//IGFBP3//LRP6//PRR5L//PPP1R15B//SLIT2//EDNRB//EFNA1//FMR1//SIRT1//NPTN//IFNG//KDR//MAVS//CCND1//CCND2//FBXW7//ERRFI1//GPRC5A//SOCS5//GFPT1//NUP54//COX8C//HPRT1//CMPK1//ADSS//ADCY3//IMPDH1//C8ORF44-SGK3//SGK2//HIPK3//FASTK//PDIK1L//CDK19//MORC3//MMD//SGK3//PAN3//GRK6//TBK1//NRBP1//CDK17//PIK3CA//PRKACB//CCL8//SGK1//CDK15//TGFBR2//TXK//MEX3B//RUNX3//ACVR1//RPS6KA5//RASSF2//GNAQ//NCK1//GSKIP//RB1//YWHAG//SOCS3//PPM1F//PTPN21//PPP4R2//PPTC7//PPP1R12A//CTDSPL2//PPP2CB//PPP3R1//PYURF//PIGP//PIGA//PLAA//ALPI//LCLAT1//ABHD5//CDS1//ABHD3//SLC44A1//SERINC3//MTMR9//SPHK2//GPHN//GRM3//COPS8//IRAK4//MDFIC//PTHLH//LPCAT3//DGKE//CRHR1//PPARGC1B//NPNT//CALM2//INHBB//BMP3//BMPR2//TTK//GDF5//LDLR//FKBP15//PLEKHF2//MCCC2//DGKH//HDHD2//RICTOR//STK33//ROR1//KCNH4//PAK6//SLK//TBL1XR1//CXCL9//ADRB1//EGLN1//PDE5A//ADNP//MOB1B//JUN//RAP2C//ARPP19//PPP1R1B//MID1//SEMA4C//CREBL2//PFN2//IFNA1//PRKAR2B//PPP1R16B//SPPL3//CNEP1R1//PTPRG//PTP4A1//PTP4A2//CDC14A//YES1//ATXN7//PTGES3//AR//ITSN1//CNOT7//ISL1//EDN1//NRBF2//CCL21//RCAN3//UBXN2B//PPP2R5E//TSC1//SMUG1//OGG1//TNFAIP3//CCNY//CCNL1//MYOG//ADCYAP1//CACUL1//GTPBP1//RORA//GRIK2//ZMYND11//WNT7A//GPCPD1//ULK2//GNPTG//ESR1//EFR3A//LRP8//CD3E//SKP1//CCM2//STRAP//PMEPA1//LDLRAD4//NOG//FAM83D//GPR55//KCNAB2//ACSL1// |
| GO:0001763 | morphogenesis of a branching structure | Biological process | 25 | 198 | 902 | 17653 | 2.47107997939484 | 2.75597119300676e-05 | 0.000855098884396135 | 4.55972532623596 | 0.0277161862527716 | EDN1//PPP3R1//TGFBR2//ACVR1//NPNT//GLI3//WT1//CLIC4//MET//MYCN//SLIT2//RDH10//FOXF1//FOXA1//DLG5//LRP6//TP63//NOG//AR//ESR1//SOCS3//SULF1//BTBD7//TNF//ETV5// |
| GO:0010638 | positive regulation of organelle organization | Biological process | 57 | 637 | 902 | 17653 | 1.75124701082889 | 2.75634417859467e-05 | 0.000855098884396135 | 4.55966655398801 | 0.0631929046563193 | CNOT6L//PAN3//CNOT6//PPARGC1B//CFL2//WDR1//RICTOR//NCK1//PFN2//CCL21//MET//CLIP1//NAV3//STMN2//SYT1//C2CD5//NEUROG1//SIRT1//MIER1//MAPK1//CCT6A//FMR1//RPS6KA5//IQGAP2//ARFIP1//ISL1//TAPT1//EDN1//EREG//IGF1//RB1//LPAR1//TSC1//PPM1F//DNMT3B//MYB//JARID2//SMURF1//MSN//TNF//KDR//MIEF1//PMAIP1//DYNLL2//PPP3R1//YWHAG//TP63//SEPT9//RAB3GAP1//SDC1//VPS4B//NMUR1//NRG1//FBXW7//PRKAA1//SNIP1//NTF3// |
| GO:0040012 | regulation of locomotion | Biological process | 79 | 973 | 902 | 17653 | 1.58900855242319 | 2.80222767201227e-05 | 0.000861700382255222 | 4.55249658258279 | 0.0875831485587583 | CCL8//SLIT2//EDN2//CXCL9//EDN1//ETS1//FOXP1//KDR//RHOB//BMPR2//WNT7A//STRAP//PFN2//PTPRG//IFNG//JUN//TGFBR2//PPM1F//C5AR1//PRR5L//C5ORF30//IGF1//IGFBP3//NRG1//C8ORF44-SGK3//FLNA//SGK3//RND3//NCK1//DOCK10//SGK1//PLXNA4//SLK//S1PR1//F3//FOXF1//FAM89B//NTF3//SEMA4C//MAPK1//PTP4A1//CBLL1//ACVR1//ARID2//SULF1//ZMYND8//TMEFF2//CLIC4//PTEN//RAP2C//LDLRAD4//RECK//NAV3//DLG5//NOG//EFNA1//SIRT1//PPARG//WNT3//EFNB2//CCL21//ROBO2//VEGFB//LPAR1//TNF//SPRED1//MEOX2//ARRDC3//CHMP3//VPS4B//EREG//MEMO1//RAF1//GCSAML//MSN//CCL2//MET//PLAA//RAPGEF2// |
| GO:0045596 | negative regulation of cell differentiation | Biological process | 60 | 682 | 902 | 17653 | 1.7217847598364 | 2.80310637433312e-05 | 0.000861700382255222 | 4.552360421002 | 0.0665188470066519 | STRAP//EFNA1//FOXA1//PPP2CA//PTEN//LDLRAD4//FRZB//PPARG//BHLHE41//STMN2//LPAR1//EFNB2//MYLIP//PTPRG//SPOCK1//TSC1//BAG5//EDNRB//DLL1//IFNG//CCND1//PTHLH//GDF5//HMGB3//ID2//IRF1//SIRT1//ZFPM2//RORA//TNF//ZEB1//SRSF6//TP63//SOCS5//KLF13//DDX6//GLI3//ISL1//TOB1//TMEM64//NOG//GPR55//ULK2//MYCN//SEMA4C//WNT3//WNT7A//TRAK2//SYNGAP1//RAPGEF2//EREG//ADCYAP1//IGF1//LDLR//SLIT2//FOXJ2//IL2//FBXW7//NRG1//MAPK1// |
| GO:0009968 | negative regulation of signal transduction | Biological process | 98 | 1276 | 902 | 17653 | 1.50309830470775 | 2.8623971803048e-05 | 0.00087594534526703 | 4.54327010459206 | 0.108647450110865 | SPRED1//DUSP1//PPP2CA//ERRFI1//GPRC5A//SOCS5//PMEPA1//LDLRAD4//PTEN//GSC//APCDD1//FRZB//HIC1//SHISA2//CCND1//CXXC4//STRAP//SIRT1//LEMD3//FAM89B//BAMBI//SMURF1//PRDM16//TGFBR2//TOB1//CTDSPL2//NOG//PRKAA1//TSC1//ACVR1//ISL1//TP63//TNFAIP3//MET//DLG5//SULF1//EPS15//SOCS1//SOCS3//ZMYND11//ESR1//RORA//EFNA1//HIPK3//ATXN7//LPAR1//YTHDF2//FBXW7//DLK2//GLI3//RB1//CD3E//NEUROD1//PPP2CB//RASA1//SYNGAP1//NCK1//DHRS3//CD2AP//SLIT2//ARHGAP12//OTUD3//PHLDA3//CALM2//PPARG//CNOT7//FOXP1//RANBP9//TMEM64//LRP6//PSMA2//PSMD12//FZD6//IGF1//CREBRF//EREG//NRG1//RASSF2//PRKACB//RAF1//BAG5//TMBIM6//DNAJB9//PPP1R15B//SESN1//LYPD6//CLOCK//PHF14//NR4A2//AR//UNC5B//TNF//BCL2A1//IGFBP3//KDR//RGS7BP//ROR1//PRR5L// |
| GO:0050673 | epithelial cell proliferation | Biological process | 41 | 409 | 902 | 17653 | 1.96188041787064 | 3.029681123595e-05 | 0.000922960965714999 | 4.51860307894018 | 0.0454545454545455 | PIK3CB//BMPR2//SULF1//GJA1//TNF//F3//SIRT1//RICTOR//PPP1R16B//JUN//KDR//VEGFB//VASH2//SRSF6//TP63//EFNB2//CEBPB//ID2//MAPK1//CCND1//EREG//IGFBP3//EDNRB//IGF1//C5AR1//NOG//STRAP//AR//ISL1//PTEN//RB1//GDF5//RUNX3//WNT7A//FOXP2//ESR1//ERRFI1//PPARG//FBXW7//TNFAIP3//NFIB// |
| GO:0007420 | brain development | Biological process | 61 | 700 | 902 | 17653 | 1.70546879949319 | 3.18600811542889e-05 | 0.000966231967921327 | 4.49675312229462 | 0.0676274944567627 | NOG//C5AR1//POU4F1//SEMA4C//LRP8//EMX2//NEUROD1//PTEN//LPAR1//EN1//CD3E//FOXP2//LRP6//RAPGEF2//PAX5//DLL1//RORA//WNT7A//NFIB//HPRT1//ARX//CALM2//GLI3//TSC1//ID2//POU3F2//SLIT2//NRG1//ETS1//RAB3GAP1//GNAQ//ATF5//ROBO2//FLNA//PLXNA4//ADCYAP1//ISL1//NR4A2//TACC2//GSC//OTX2//DLG5//FZD3//FZD6//NANOS1//WNT3//DPYSL2//S1PR1//RAB18//PHF8//GABRA5//SPHK2//PTPRG//ATXN1//SLC6A11//BMPR2//SYT1//TGFBR2//BAG6//CASP2//IMMP2L// |
| GO:0060998 | regulation of dendritic spine development | Biological process | 13 | 68 | 902 | 17653 | 3.74150580409547 | 3.40524327706907e-05 | 0.00102810983405438 | 4.46785185581742 | 0.0144124168514412 | LPAR1//CPEB3//FMR1//ZMYND8//NRG1//IL2//MAPK6//DLG5//ARC//EFNA1//PTEN//LRP8//FXR1// |
| GO:0048754 | branching morphogenesis of an epithelial tube | Biological process | 21 | 153 | 902 | 17653 | 2.68620929524803 | 3.44414333459479e-05 | 0.00103523294986065 | 4.46291878284897 | 0.0232815964523282 | EDN1//PPP3R1//TGFBR2//ACVR1//NPNT//GLI3//WT1//RDH10//FOXF1//FOXA1//DLG5//LRP6//AR//ESR1//ETV5//TNF//NOG//CLIC4//MET//MYCN//SLIT2// |
| GO:0090304 | nucleic acid metabolic process | Biological process | 327 | 5329 | 902 | 17653 | 1.20091982995607 | 3.4974770331678e-05 | 0.00104661226439442 | 4.45624512870211 | 0.362527716186253 | SIRT1//IGF1//NFIA//NFIB//NFIC//RBBP8//RBMS1//NPAT//TSHZ1//ZBTB18//CEBPB//TCERG1//STRAP//KLF12//GSC//ARX//DNMT3B//EDN1//EDNRB//EFNA1//CC2D1B//EN1//EP300//ESR1//JAZF1//CPEB3//FOXF1//MYT1L//SIN3B//ZFPM2//DNAJB5//GLI3//CNOT7//HIC1//FOXA1//HSBP1//ID2//IFNG//IRF2//AR//ISL1//JARID2//JUN//MAF//MDM4//MEF2A//MYB//NR4A2//PAX5//KLF3//POU4F1//PPARG//ZBTB4//RB1//CCND1//RNF2//SARS//PRDM16//SMARCA2//SUV39H1//ZEB1//TFAP2A//TGIF1//KLF10//TNF//UBE2I//WT1//ZNF217//LRP8//BHLHE41//TBL1XR1//E2F8//ARID5B//BHLHE40//TP63//RUNX3//NOG//FOXP2//PHF14//PNRC1//DCP2//PPP2CA//CNOT6L//PAN3//CNOT6//PATL1//SRSF6//MBNL2//RBFOX3//FMR1//MBNL1//RBM25//SRSF2//ELAVL2//NOVA1//PCBP2//RBM41//RRP15//COPS8//GTF2H1//PTGES3//NABP1//AGO1//GTF2E1//RPP14//RPP25//CREBRF//ELK3//ETV5//ATF5//FOXF2//FLI1//GTF2A1//HIVEP2//IRF1//MEOX2//MYBL1//MYCN//NEUROD1//NHLH2//OTX2//KLF13//PKNOX1//POU3F2//FOXJ2//HIVEP3//BMPR2//SOX4//SOX5//BTF3//TFE3//TXK//FOSL1//KLF7//CBFB//FUBP3//CCNT2//NMI//CLOCK//ZNF516//POLR3H//SLBP//PURA//GMNC//ESCO2//GADD45A//EPC2//ASTE1//UBE2W//TAOK1//USP28//UBE2V1//SMUG1//OGG1//KDM2A//MTRR//ASCC1//HMGB3//TSN//BAHD1//H2AFZ//ZMYND11//ZNF526//CREBL2//ZNF800//E2F5//ZNF367//ARID2//MED19//ZFP30//MYCBP2//PHF8//FLII//ADNP//ZMYND8//ZNF521//HBP1//FOXP1//ZBTB11//ATAD2//MDFIC//HOXA3//HOXD1//ZNF680//ZNF662//NEUROG1//NFE2//NPAS2//IER5//COMMD10//ARID4B//BRWD1//BNC2//MED9//ZNF532//PRKAA1//TRERF1//MAPK1//SLC2A4RG//CCNL1//PHTF2//ZBTB26//MIER1//ATXN1//ATXN7//SMARCD2//SS18//MED22//ZNF3//ZNF708//ZNF131//ZNF227//ZNF655//ZSCAN5A//SAP30L//ANP32A//PPP1R1B//PCGF5//LCOR//ST18//HIPK3//PPARGC1B//ELL2//NR5A2//NRBF2//AFF1//MLLT6//PHF20L1//PAK6//RORA//BTG1//CSDE1//SNIP1//TEAD2//RPS6KA5//EMX2//ETS1//BOLA3//NR3C2//RFX7//TRAK2//BDP1//NRBP1//CELF1//GRSF1//RTCA//PPP4R2//CPEB1//QKI//THG1L//G3BP2//EREG//DDX6//DDX3Y//INTS8//FLNA//BAMBI//LRP6//WNT7A//ACVR1//DNAJB6//NRG1//RASD1//BTAF1//PPM1F//TOB1//SMURF1//BMP3//GDF5//SULF1//LEMD3//CTDSPL2//RWDD3//TNFAIP3//CCT6A//FBXW7//INSIG2//MYOG//C5AR1//TDRD5//EGLN1//FZD6//FASTK//YTHDF2//PSMA2//PSMD12//FXR1//PIK3CA//ADCYAP1//S1PR1//KPNA6//DLL1//TBK1//IL2//MET//PPP1R12A//NCK1//ARMCX3//CYTL1//PPP3R1//MAVS//RAF1//YES1//TIA1//C8ORF44-SGK3//SGK3//SGK1//PTEN//RHEBL1//ROR1//IRAK4//RASA1//TNRC6B//TMBIM6//PRR5L//GTPBP1//EGLN3//BOLL//SEPSECS//NANOS1//RAP2C//HSBP1L1//FAM168A//DUSP1//GJA1//KCNK2//NPNT// |
| GO:0002064 | epithelial cell development | Biological process | 25 | 201 | 902 | 17653 | 2.4341981886576 | 3.55621733315242e-05 | 0.00105950210678898 | 4.44901170560266 | 0.0277161862527716 | CCM2//CLIC4//MET//GJA1//DLL1//PALLD//AR//FRMD6//GSTM3//WNT7A//SDC1//YIPF6//FOXA1//PPP1R16B//MSN//RAP1A//RAP1B//RAPGEF2//TMOD1//RAP2C//ARID4B//TNF//CLOCK//ESR1//TP63// |
| GO:0001570 | vasculogenesis | Biological process | 14 | 78 | 902 | 17653 | 3.51273523224743 | 3.60252884835513e-05 | 0.00106392587779152 | 4.44339253275373 | 0.0155210643015521 | WNT7A//SPRED1//KDR//RAP1A//RAPGEF2//GJC1//FOXF1//ZFPM2//RASA1//TGFBR2//WT1//CCM2//TEAD2//QKI// |
| GO:0014033 | neural crest cell differentiation | Biological process | 14 | 78 | 902 | 17653 | 3.51273523224743 | 3.60252884835513e-05 | 0.00106392587779152 | 4.44339253275373 | 0.0155210643015521 | EDNRB//ISL1//SEMA4C//ACVR1//RDH10//EDN1//TAPT1//NRG1//KBTBD8//GSC//LRP6//MAPK1//FRZB//WNT10A// |
| GO:0021954 | central nervous system neuron development | Biological process | 13 | 69 | 902 | 17653 | 3.68728108229699 | 4.00204494095261e-05 | 0.00117677521459402 | 4.39771803876374 | 0.0144124168514412 | ARX//SLIT2//GNAQ//RAPGEF2//ATF5//ROBO2//NR4A2//PTEN//NFIB//PLXNA4//SCN1B//NANOS1//HPRT1// |
| GO:0007611 | learning or memory | Biological process | 28 | 240 | 902 | 17653 | 2.28327790096083 | 4.02352736432538e-05 | 0.0011779703707763 | 4.39539304065613 | 0.0310421286031042 | ARC//JUN//PRKAR2B//ATXN1//SLC6A1//FOSL1//NRXN3//CEBPB//KCNK2//NTF3//PAIP2//KCNK10//PTEN//ADNP//CPEB3//LDLR//SGK1//CCND2//GABRA5//SNAP25//ADCY3//NPTN//PPP1R1B//SYNGAP1//FOXP2//EN1//NEUROG1//MAPK1// |
| GO:0051093 | negative regulation of developmental process | Biological process | 78 | 968 | 902 | 17653 | 1.57699831412289 | 4.08036359024161e-05 | 0.00118946116210362 | 4.38930113637087 | 0.0864745011086474 | BMPR2//STRAP//EFNA1//FOXA1//PPP2CA//PTEN//LDLRAD4//FRZB//PPARG//BHLHE41//STMN2//LPAR1//EFNB2//MYLIP//PTPRG//SPOCK1//TSC1//BAG5//EDNRB//SPRED1//SULF1//AGO1//FOXJ2//SARS//DLL1//IFNG//CCND1//TNFRSF21//PTHLH//GDF5//ADRB1//HMGB3//ID2//IRF1//MAPK1//SIRT1//ZFPM2//RORA//TNF//ZEB1//SRSF6//TP63//SOCS5//KLF13//DDX6//GLI3//ISL1//TOB1//TMEM64//NOG//GPR55//FZD3//ULK2//MYCN//SEMA4C//WNT3//WNT7A//TRAK2//SYNGAP1//RAPGEF2//EREG//ROBO2//GJA1//JARID2//KCNK2//TGFBR2//ADCYAP1//IGF1//LDLR//WT1//MEOX2//SLIT2//PRKACB//IL2//FBXW7//NFIB//PHF14//NRG1// |
| GO:0009058 | biosynthetic process | Biological process | 401 | 6735 | 902 | 17653 | 1.16524904649735 | 4.15690408549513e-05 | 0.00120657263219758 | 4.38122999609055 | 0.444567627494457 | SIRT1//IGF1//NFIA//NFIB//NFIC//RBBP8//RBMS1//NPAT//TSHZ1//ZBTB18//CEBPB//TCERG1//STRAP//KLF12//GSC//ARX//DNMT3B//EDN1//EDNRB//EFNA1//CC2D1B//EN1//EP300//ESR1//JAZF1//CPEB3//FOXF1//MYT1L//SIN3B//ZFPM2//DNAJB5//GLI3//CNOT7//HIC1//FOXA1//HSBP1//ID2//IFNG//IRF2//AR//ISL1//JARID2//JUN//MAF//MDM4//MEF2A//MYB//NR4A2//PAX5//KLF3//POU4F1//PPARG//ZBTB4//RB1//CCND1//RNF2//SARS//PRDM16//SMARCA2//SUV39H1//ZEB1//TFAP2A//TGIF1//KLF10//TNF//UBE2I//WT1//ZNF217//LRP8//BHLHE41//TBL1XR1//E2F8//ARID5B//BHLHE40//TP63//RUNX3//NOG//FOXP2//PHF14//GTF2E1//SEPSECS//SECISBP2L//PTGES3//EDN2//MGST1//ST3GAL5//RDH10//GTF2H1//CREBRF//ELK3//ETV5//ATF5//FOXF2//FLI1//GTF2A1//HIVEP2//IRF1//MEOX2//MYBL1//MYCN//NEUROD1//NHLH2//OTX2//KLF13//PKNOX1//POU3F2//FOXJ2//HIVEP3//BMPR2//SOX4//SOX5//BTF3//TFE3//TXK//FOSL1//KLF7//CBFB//FUBP3//CCNT2//NMI//CLOCK//ZNF516//POLR3H//SRSF2//SRSF6//SLBP//HS3ST5//SDC1//HS3ST3A1//HS3ST1//GFPT1//SLC35D1//NUP54//NLN//HPRT1//ADSS//ADCY3//IMPDH1//CMPK1//PURA//GMNC//ESCO2//PPP2CA//BAHD1//H2AFZ//ZMYND11//PNRC1//ZNF526//CREBL2//ZNF800//E2F5//ZNF367//ARID2//MED19//ZFP30//KDM2A//MYCBP2//PHF8//FLII//ADNP//ZMYND8//CNOT6L//ZNF521//EPC2//AGO1//HBP1//FOXP1//ZBTB11//ATAD2//MDFIC//HMGB3//HOXA3//HOXD1//ZNF680//ZNF662//NEUROG1//NFE2//NPAS2//ASCC1//IER5//COMMD10//ARID4B//BRWD1//BNC2//MED9//ZNF532//PRKAA1//TRERF1//MAPK1//SLC2A4RG//CCNL1//PHTF2//CNOT6//ZBTB26//MIER1//ATXN1//ATXN7//SMARCD2//SS18//MED22//ZNF3//ZNF708//ZNF131//ZNF227//ZNF655//ZSCAN5A//SAP30L//ANP32A//PPP1R1B//PCGF5//LCOR//ST18//HIPK3//PPARGC1B//ELAVL2//ELL2//NR5A2//NRBF2//AFF1//MLLT6//OGG1//PHF20L1//PAK6//RORA//BTG1//UBE2V1//CSDE1//SNIP1//TEAD2//RPS6KA5//EMX2//ETS1//BOLA3//NR3C2//RFX7//TRAK2//BDP1//NRBP1//CPEB2//CPEB1//DDX3Y//GTPBP1//DDX6//TSC1//KBTBD8//FUT9//B3GALNT2//FUT2//FUT5//SERP1//ST8SIA5//KCNE1//CCDC126//ALG2//EOGT//GCNT4//VEGFB//WDR45B//ALPI//RECK//PYURF//PIGP//PIGA//SERINC3//PSAT1//ELOVL5//LCLAT1//ABHD5//CDS1//ABHD3//SLC44A1//PIK3CA//PIK3CB//PTEN//MTMR9//SPHK2//ACBD3//LBR//INSIG2//MSMO1//GPHN//GRM3//G3BP2//ACSL1//RIMKLB//LPCAT3//DGKE//MTRR//GLT6D1//EREG//CCL21//CRHR1//CNEP1R1//LDLR//FBXW7//ARPP19//FMR1//PRR16//FXR1//FLNA//BAMBI//LRP6//WNT7A//ACVR1//DNAJB6//NRG1//RASD1//BTAF1//PPM1F//TOB1//CELF1//NANOS1//TIA1//MEX3B//OGN//PRELP//CHST1//ZDHHC22//ZDHHC7//ZDHHC18//MCFD2//ABHD17C//ELOVL7//SPTSSB//SPTSSA//DSEL//SMURF1//BMP3//GDF5//SULF1//LEMD3//CTDSPL2//ADRB1//PTHLH//CALM2//EGLN1//RWDD3//TNFAIP3//DCP2//CCT6A//ERRFI1//RAP1A//TNRC6B//ACSL4//NCK1//ADIPOR2//QKI//MYOG//C5AR1//INTS8//NABP1//FZD6//SMUG1//CD3E//TBK1//TMEFF2//MID1IP1//C1QTNF2//ADCYAP1//S1PR1//KPNA6//DLL1//IL2//MET//PPP1R12A//ARMCX3//CYTL1//PPP3R1//MAVS//RAF1//YES1//PAIP2//BOLL//PDE5A//RAPGEF2//INHBB//C8ORF44-SGK3//SGK3//SGK1//RHEBL1//ROR1//IRAK4//KDR//CCL2//YTHDF2//EGLN3//PSMA2//PSMD12//ATG16L1//GSTA2//GSTM3//RAB3GAP1//RAP2C//HSBP1L1//PPP1R15B//TMBIM6//DUSP1//GJA1//KCNK2//NPNT//ACCS// |
| GO:0040007 | growth | Biological process | 77 | 954 | 902 | 17653 | 1.57962622079051 | 4.32950403953973e-05 | 0.00125130067604304 | 4.36356185081238 | 0.0853658536585366 | ESR1//EREG//AR//SOCS5//C8ORF44-SGK3//SGK2//SGK3//NANOS1//IGFBP3//RB1//SGK1//SPOCK1//S1PR1//EVC//BNC2//TGFBR2//IGF1//WNT7A//IL2//SIRT1//FRZB//GJA1//PPARG//PPP2CA//BMPR2//SMARCA2//BTG1//WT1//ADIPOR2//SLIT2//EI24//DPYSL2//RAB21//TNF//EN1//ATF5//CLIC4//TBL1XR1//CCM2//ARID5B//DLL1//LRP12//IFNG//ZMAT3//SOCS1//SOCS3//PIK3CA//GDF5//ADRB1//POU3F2//TXK//MYOG//ADNP//NRG1//TNFRSF12A//PPM1F//PTEN//ARX//SERP1//GLI3//ULK2//SLC9A6//WNT3//PLXNA4//SEMA4C//ARID2//JARID2//KCNK2//NOG//ZFPM2//RDH10//EDN1//LRP6//SMURF1//SYT1//PLAA//FOXP2// |
| GO:0051090 | regulation of DNA binding transcription factor activity | Biological process | 41 | 416 | 902 | 17653 | 1.92886800699301 | 4.46963346886392e-05 | 0.00128630345318837 | 4.34972808961449 | 0.0454545454545455 | G3BP2//SIRT1//RWDD3//TNFAIP3//LRP8//RPS6KA5//ESR1//FLNA//ID2//EGLN1//RB1//RNF2//FZD6//BHLHE40//PPARGC1B//EDN1//EP300//FOXA1//LRP6//NEUROD1//NEUROG1//NHLH2//CYTL1//PPARG//PTEN//MAVS//TNF//FOSL1//ARID5B//RHEBL1//AR//ROR1//IRAK4//UBE2V1//CLOCK//C8ORF44-SGK3//SGK3//JUN//POU4F1//MAPK1//SGK1// |
| GO:0051241 | negative regulation of multicellular organismal process | Biological process | 90 | 1162 | 902 | 17653 | 1.5158225553465 | 4.55401842961828e-05 | 0.00130503502709781 | 4.34160521617469 | 0.0997782705099778 | RPS6KA5//TNF//BMPR2//HS3ST5//SLIT2//JARID2//STRAP//PFN2//PTPRG//EFNA1//FOXA1//PPP2CA//PTEN//LDLRAD4//STMN2//LPAR1//EFNB2//MYLIP//SPOCK1//TSC1//BAG5//EDNRB//SPRED1//SULF1//AGO1//PPARG//FOXJ2//SARS//INHBB//EDN1//SMURF1//TNFRSF21//ARRDC3//PTHLH//GDF5//TBK1//PCBP2//MAVS//TNFAIP3//ERRFI1//DLL1//IFNG//NAV3//SOCS5//CD2AP//ADRB1//TIA1//NMI//HMGB3//ID2//IRF1//SRSF6//TP63//KLF13//DDX6//GLI3//ISL1//TOB1//TMEM64//NOG//GPR55//IL2//FZD3//ULK2//MYCN//SEMA4C//WNT3//WNT7A//TRAK2//SYNGAP1//RAPGEF2//ROBO2//PDE5A//GJA1//KCNK2//TGFBR2//ADCYAP1//IGF1//FRZB//LDLR//WT1//MEOX2//C5ORF30//NEUROG1//PRKACB//ZFPM2//FBXW7//NFIB//PHF14//NRG1// |
| GO:0051347 | positive regulation of transferase activity | Biological process | 58 | 664 | 902 | 17653 | 1.70951099297411 | 4.6583858188463e-05 | 0.00132528745940206 | 4.33176454498619 | 0.0643015521064302 | GADD45A//TNF//CRKL//TAOK1//RAF1//RAP1A//C1QTNF2//LPAR1//NRG1//IGF1//NTF3//PIK3CB//PRKAA1//MAPK1//C5AR1//UBE2V1//EREG//FBXW7//COPS8//MDFIC//SOCS1//ADNP//PIK3CA//PAK6//TGFBR2//SLK//RICTOR//KDR//MET//ROR1//ADCY3//PRKACB//PRKAR2B//EDN1//PDE5A//CCL21//CCNY//CCNL1//CCND1//CCND2//CCNT2//ADCYAP1//CACUL1//MMD//RAPGEF2//RASSF2//DCUN1D4//ARRDC3//PTGES3//EFNA1//LRP8//ACSL1//IFNG//PPP2CA//CALM2//UBE2I//PTEN//SIRT1// |
| GO:0032879 | regulation of localization | Biological process | 181 | 2700 | 902 | 17653 | 1.31197873039336 | 4.66388311899588e-05 | 0.00132528745940206 | 4.33125234191353 | 0.200665188470067 | ARL6IP1//FMR1//NTF3//NUP54//CCL8//SLIT2//EDN2//CXCL9//TNF//EDN1//EDNRB//ADCYAP1//RAPGEF4//SLC25A6//IFNG//KCNB1//MARCKS//NEUROD1//RAP1A//SNAP25//CLOCK//TMBIM6//TRPC3//ETS1//FOXP1//KDR//RHOB//BMPR2//WNT7A//STRAP//PFN2//PTPRG//JUN//TGFBR2//PPM1F//C5AR1//PRR5L//C5ORF30//SCN1B//C2CD5//SIRT1//CALM2//FBXW7//PPARG//ABHD5//SLC6A1//SYT1//IGF1//IGFBP3//ARPP19//C1QTNF2//CREBL2//ADIPOR2//INHBB//RAB21//RAB8B//SYT6//NRG1//GJA1//SNX17//C8ORF44-SGK3//FLNA//SGK3//RND3//NCK1//DOCK10//SGK1//PLXNA4//SLK//S1PR1//F3//FOXF1//FAM89B//SEMA4C//MAPK1//PTP4A1//CBLL1//ACVR1//ARID2//SULF1//ZMYND8//TMEFF2//CLIC4//PTEN//RAP2C//LDLRAD4//RECK//NAV3//DLG5//NOG//TBC1D12//SERP1//ISL1//SOX4//STIM2//NDFIP2//SGK2//DNAJB6//STX6//MMD//SLC26A4//OGG1//MAVS//KCNH4//KCNA4//KCNJ2//KCNJ3//KCNJ10//KCNK10//ANO1//SYBU//GLI3//MDFIC//PXK//EFNA1//DLL1//LDLR//APPL1//PPP1R12A//CTDSPL2//CHRNB4//CD2AP//CCL21//WNT3//SOCS1//ARFIP1//SIRPA//TUB//IL2//HOMER1//KCNE1//WDR1//PRKAA1//VEGFB//SLC30A6//RAB3GAP1//LPAR1//RB1//SPRED1//MEOX2//CCL2//MIEF1//DYNLL2//PMAIP1//PPP3R1//YWHAG//TP63//KCNIP4//KCNAB2//CRHR1//SLC31A2//NPTN//ABHD17C//MSN//AR//PPFIA1//PLAA//VAMP3//VPS4B//SDC1//NMUR1//SNIP1//CCT6A//DCP2//SESTD1//NEUROG1//EREG//MEMO1//RAF1//RIMS4//PIK3CB//GCSAML//CHMP3//HECW2//GLRX//TNFRSF21//MYB//ARC//MET//ARHGAP1//RAPGEF2// |
| GO:1901214 | regulation of neuron death | Biological process | 31 | 281 | 902 | 17653 | 2.15907315495025 | 4.72026983880629e-05 | 0.00133538412493215 | 4.32603317379072 | 0.0343680709534368 | EGLN3//TP63//CEBPB//CNTFR//EN1//UNC5B//ADNP//GABRA5//GRIK2//ISL1//JUN//NTF3//NR4A2//PIK3CA//POU4F1//RASA1//CCL2//ITSN1//C5AR1//GDF5//SYNGAP1//MYB//TFAP2A//CASP2//SIRT1//EFNB2//TSC1//FBXW7//KCNB1//TBK1//EGLN1// |
| GO:0033674 | positive regulation of kinase activity | Biological process | 53 | 590 | 902 | 17653 | 1.75806869856064 | 4.73890566292645e-05 | 0.00133538412493215 | 4.32432193668534 | 0.0587583148558758 | GADD45A//TNF//CRKL//TAOK1//RAF1//RAP1A//C1QTNF2//LPAR1//NRG1//IGF1//NTF3//PIK3CB//PRKAA1//MAPK1//C5AR1//UBE2V1//EREG//FBXW7//COPS8//MDFIC//SOCS1//ADNP//PIK3CA//PAK6//TGFBR2//SLK//RICTOR//ADCY3//PRKACB//PRKAR2B//EDN1//PDE5A//CCL21//CCNY//CCNL1//CCND1//CCND2//CCNT2//ADCYAP1//CACUL1//MMD//RAPGEF2//RASSF2//EFNA1//LRP8//ACSL1//IFNG//PPP2CA//CALM2//SIRT1//KDR//MET//ROR1// |
| GO:0060425 | lung morphogenesis | Biological process | 11 | 52 | 902 | 17653 | 4.14000938086304 | 5.12352705735336e-05 | 0.00143777649331455 | 4.29043096594165 | 0.0121951219512195 | RDH10//FOXF1//FOXA1//DLG5//TGFBR2//SRSF6//FOXP2//TNF//NFIB//MAPK1//NOG// |
| GO:0007265 | Ras protein signal transduction | Biological process | 44 | 463 | 902 | 17653 | 1.85987462466417 | 5.68076450114291e-05 | 0.00158756240996816 | 4.24559321423868 | 0.0487804878048781 | EPS8//RHOB//RND3//ARHGAP1//ARHGAP6//PAK6//ARAP2//IQSEC2//APPL1//FBXO8//AGFG1//PSD//GIT2//RAB12//RAB18//RAB21//RAB30//RAB9B//RAB8B//RAB28//RALGPS1//RAP2C//RAP1A//RAP1B//RAPGEF2//PLEKHG4B//ARHGEF4//RAF1//ITSN1//SOS2//MET//LPAR1//GPR55//RASGEF1A//NRG1//IGF1//PPP2CB//RASA1//SYNGAP1//CRKL//JUN//USP28//RB1//G3BP2// |
| GO:0030334 | regulation of cell migration | Biological process | 70 | 853 | 902 | 17653 | 1.60605714018347 | 5.81997198284413e-05 | 0.00161704034799052 | 4.23507910602459 | 0.0776053215077605 | CCL8//SLIT2//EDN2//CXCL9//EDN1//ETS1//FOXP1//KDR//RHOB//BMPR2//WNT7A//STRAP//PFN2//PTPRG//IFNG//JUN//TGFBR2//PPM1F//C5AR1//PRR5L//C5ORF30//IGF1//IGFBP3//NRG1//S1PR1//F3//FOXF1//FAM89B//NTF3//SEMA4C//MAPK1//PTP4A1//CBLL1//ACVR1//ARID2//SULF1//ZMYND8//TMEFF2//CLIC4//PTEN//RAP2C//LDLRAD4//RECK//NAV3//DLG5//NOG//EFNA1//SIRT1//PPARG//VEGFB//LPAR1//TNF//CCL21//SPRED1//MEOX2//GCSAML//MSN//CCL2//MET//FLNA//PLAA//RAPGEF2//C8ORF44-SGK3//SGK3//RND3//NCK1//DOCK10//SGK1//PLXNA4//SLK// |
| GO:0014706 | striated muscle tissue development | Biological process | 38 | 379 | 902 | 17653 | 1.96225918363765 | 5.83406542820771e-05 | 0.00161704034799052 | 4.2340287046366 | 0.0421286031042129 | NRG1//ID2//ZFPM2//POU4F1//NOG//S1PR1//ZBTB18//CFL2//EP300//MEOX2//MYOG//GPCPD1//CCNT2//FOXP2//PAX5//RB1//HIVEP3//GJA1//DLL1//PRKAA1//GJC1//PTEN//HOMER1//MEF2A//TSC1//EGLN1//ISL1//TNNI1//ARID2//JARID2//KCNK2//TGFBR2//WT1//ACVR1//EDN1//IGF1//NLN//EFNB2// |
| GO:0009896 | positive regulation of catabolic process | Biological process | 39 | 393 | 902 | 17653 | 1.94215568456864 | 5.86619534986702e-05 | 0.00161930935310819 | 4.23164347869664 | 0.0432372505543237 | IFNG//PIK3CB//PRKAA1//ABHD5//RAB12//SIRT1//SESN1//TBK1//KDR//TSC1//ANKIB1//LONRF3//LONRF1//SOCS5//GJA1//MYLIP//SMURF1//HECW2//TNF//TNFAIP3//IGF1//SH3D19//TOB1//CPEB3//TNRC6B//CNOT7//PRR5L//GTPBP1//CNOT6L//YTHDF2//QKI//NANOS1//FMR1//FBXW7//MSN//BAG6//LDLR//PTEN//RAB3GAP1// |
| GO:0051962 | positive regulation of nervous system development | Biological process | 46 | 493 | 902 | 17653 | 1.82609301844448 | 6.05074357404084e-05 | 0.00166346255248936 | 4.2181912517509 | 0.0509977827050998 | GLI3//FZD3//STMN2//ADCYAP1//NPTN//NCK1//SERPINI1//RAP1A//SCN1B//RAPGEF2//WASF3//NRG1//DNMT3B//ETV5//MMD//FOXA1//NEUROD1//NEUROG1//PTEN//ZEB1//GDF5//ADNP//TNFRSF12A//WNT3//ID2//PPARG//BMPR2//ROBO2//PLXNA4//SLIT2//RAB21//SLITRK3//WNT7A//DLG5//MYB//LPAR1//CPEB3//FMR1//ZMYND8//IL2//MAPK6//LRP8//SMURF1//SYT1//PLAA//FLNA// |
| GO:0050821 | protein stabilization | Biological process | 21 | 159 | 902 | 17653 | 2.58484290674811 | 6.12048796626617e-05 | 0.00167582429618859 | 4.2132139516112 | 0.0232815964523282 | CRTAP//PTGES3//CREBL2//EFNA1//EP300//FLNA//OTUD3//MORC3//GNAQ//IGF1//USP27X//MDM4//PFN2//FBXW7//PTEN//MTMR9//SOX4//TSC1//BAG6//CCT6A//RASSF2// |
| GO:0021953 | central nervous system neuron differentiation | Biological process | 22 | 171 | 902 | 17653 | 2.51790044216232 | 6.16880688990384e-05 | 0.00168224358856531 | 4.20979882482197 | 0.024390243902439 | ISL1//SOX4//RORA//WNT7A//GLI3//ARX//SLIT2//GNAQ//RAPGEF2//ATF5//ROBO2//HPRT1//NR4A2//PTEN//NFIB//PLXNA4//SCN1B//NANOS1//LRP6//WNT3//POU4F1//SPOCK1// |
| GO:0007167 | enzyme linked receptor protein signaling pathway | Biological process | 81 | 1028 | 902 | 17653 | 1.54206928830873 | 6.2514633149954e-05 | 0.00169756496222772 | 4.20401831301699 | 0.08980044345898 | KDR//NTF3//ROR1//PIK3CB//PTPRG//TXK//YES1//CD3E//CD8A//PALM2//BMPR2//EREG//FBXW7//ERRFI1//GPRC5A//SOCS5//NRG1//EPS8//EPS15//PIK3CA//RPS6KA5//SIRT1//JUN//SMURF1//TGFBR2//KLF10//ZYX//GDF5//ACVR1//APPL1//SPRED1//MAPK1//TIA1//SHCBP1//F3//INHBB//BMP3//TTK//PMEPA1//LDLRAD4//ZEB1//NREP//TOB1//NOG//NPNT//STRAP//LEMD3//FAM89B//BAMBI//PRDM16//SULF1//CTDSPL2//SLC9A6//RAPGEF2//C2CD5//CUL5//RASSF2//OTX2//SHISA2//IGFBP3//IGF1//AR//ATXN7//NPTN//NCK1//TSC1//SOCS1//SOCS3//PTEN//ARID5B//GIGYF1//VEGFB//RAF1//MET//EFNA1//EFNB2//RASA1//ITSN1//SS18//DLL1//PHF14// |
| GO:0050768 | negative regulation of neurogenesis | Biological process | 29 | 259 | 902 | 17653 | 2.19134227670813 | 6.27519208275809e-05 | 0.00169756496222772 | 4.20237297594969 | 0.0321507760532151 | STMN2//LPAR1//EFNB2//MYLIP//PTPRG//SPOCK1//TSC1//BAG5//EDNRB//DLL1//DDX6//GLI3//ID2//ISL1//ULK2//PTEN//MYCN//NOG//SEMA4C//WNT3//TRAK2//SYNGAP1//RAPGEF2//ADCYAP1//EFNA1//LDLR//SLIT2//NRG1//WNT7A// |
| GO:2000145 | regulation of cell motility | Biological process | 73 | 904 | 902 | 17653 | 1.58039778859172 | 6.75921654651938e-05 | 0.00182121838661795 | 4.170103639745 | 0.0809312638580931 | CCL8//SLIT2//EDN2//CXCL9//EDN1//ETS1//FOXP1//KDR//RHOB//BMPR2//WNT7A//STRAP//PFN2//PTPRG//IFNG//JUN//TGFBR2//PPM1F//C5AR1//PRR5L//C5ORF30//IGF1//IGFBP3//NRG1//C8ORF44-SGK3//FLNA//SGK3//RND3//NCK1//DOCK10//SGK1//PLXNA4//SLK//S1PR1//F3//FOXF1//FAM89B//NTF3//SEMA4C//MAPK1//PTP4A1//CBLL1//ACVR1//ARID2//SULF1//ZMYND8//TMEFF2//CLIC4//PTEN//RAP2C//LDLRAD4//RECK//NAV3//DLG5//NOG//EFNA1//SIRT1//PPARG//VEGFB//LPAR1//TNF//CCL21//SPRED1//MEOX2//GCSAML//MSN//CCL2//MET//PLAA//RAPGEF2//EREG//MEMO1//RAF1// |
| GO:0060537 | muscle tissue development | Biological process | 39 | 396 | 902 | 17653 | 1.92744238392797 | 6.91848248639993e-05 | 0.00185673401013979 | 4.15998915410623 | 0.0432372505543237 | NRG1//ID2//ZFPM2//POU4F1//NOG//S1PR1//ZBTB18//CFL2//EP300//MEOX2//MYOG//GPCPD1//CCNT2//FOXP2//PAX5//RB1//HIVEP3//GJA1//DLL1//PRKAA1//GJC1//PTEN//HOMER1//TP63//MEF2A//TSC1//EGLN1//ISL1//TNNI1//ARID2//JARID2//KCNK2//TGFBR2//WT1//ACVR1//EDN1//IGF1//NLN//EFNB2// |
| GO:0014070 | response to organic cyclic compound | Biological process | 75 | 937 | 902 | 17653 | 1.56651174787677 | 7.13824944497605e-05 | 0.00190814154135862 | 4.14640827976453 | 0.0831485587583149 | PPP1R1B//HOMER1//AGO1//SNIP1//ADSS//SLC6A1//TSN//ESR1//PPARGC1B//AR//PMEPA1//RB1//DNMT3B//PPARG//PRKAA1//CNGA3//DUSP1//ETS1//FOXA1//OGG1//PTEN//RBBP8//CCND1//WNT7A//FOSL1//UBA5//CBFB//ISL1//TP63//SRSF2//TRPC3//EDN1//FOXP2//RORA//IFNA1//MAPK1//PMAIP1//NR5A2//NR3C2//NR4A2//TGFBR2//SDC1//TNF//SLIT2//JUN//EP300//SIRT1//FOXP1//KCNE1//RAP1A//RAP1B//WT1//RAPGEF2//MAVS//ADCYAP1//H2AFZ//MYOG//POU4F1//MSN//LRP6//LRP8//CEBPB//FOXF1//CCL2//FIBIN//ERRFI1//PRKACB//PRKAR2B//CREBRF//MYCN//ADCY3//CLOCK//EDNRB//ACSL1//ANXA7// |
| GO:0061564 | axon development | Biological process | 46 | 497 | 902 | 17653 | 1.81139609274271 | 7.3426937755337e-05 | 0.00194744023686201 | 4.13414458351343 | 0.0509977827050998 | SLITRK3//OGN//POU4F1//PRELP//WNT7A//SLIT2//RANBP9//ARX//DPYSL2//EFNA1//EFNB2//GLI3//OTX2//PIK3CA//PIK3CB//ENAH//MAPK1//ROBO2//SCN1B//WNT3//KLF7//RPS6KA5//NRXN3//FEZ2//NOG//PLXNA4//NR4A2//PTEN//NFIB//RAB21//ISL1//JUN//NREP//UNC5B//FZD3//ADNP//NRG1//TNFRSF12A//ULK2//SLC9A6//BMPR2//SEMA4C//POU3F2//TRAK2//SYNGAP1//CAMSAP2// |
| GO:0010721 | negative regulation of cell development | Biological process | 32 | 301 | 902 | 17653 | 2.08063292351438 | 7.3428546562149e-05 | 0.00194744023686201 | 4.13413506809187 | 0.0354767184035477 | STMN2//LPAR1//EFNB2//MYLIP//PTPRG//SPOCK1//TSC1//BAG5//EDNRB//DLL1//DDX6//GLI3//ID2//ISL1//ULK2//PTEN//MYCN//NOG//SEMA4C//WNT3//WNT7A//TRAK2//SYNGAP1//RAPGEF2//ADCYAP1//IGF1//EFNA1//LDLR//SLIT2//FBXW7//NRG1//FRZB// |
| GO:0048598 | embryonic morphogenesis | Biological process | 52 | 586 | 902 | 17653 | 1.73667163603066 | 7.65154216092879e-05 | 0.00202127995631928 | 4.11625102439107 | 0.057649667405765 | LRP6//RNF2//ACVR1//DUSP1//NOG//BMPR2//WNT3//SOX4//SEMA4C//PRKACB//TSC1//FZD3//FZD6//TEAD2//GJA1//DLL1//TGFBR2//TFAP2A//GSC//EFNA1//GRSF1//MBNL1//GDF5//LRIG1//RDH10//EN1//WNT7A//RECK//TP63//HSBP1//NEUROG1//INSIG2//MAPK1//TSHZ1//EDN1//GLI3//GNAQ//MYCN//FOXF1//ID2//NEUROD1//FOXF2//ZEB1//PAX5//HOXA3//AR//SOCS3//SULF1//DNAJB6//OTX2//FRZB//YTHDF2// |
| GO:0043524 | negative regulation of neuron apoptotic process | Biological process | 19 | 138 | 902 | 17653 | 2.69455156014011 | 7.68104315797806e-05 | 0.00202127995631928 | 4.11457979468348 | 0.0210643015521064 | CEBPB//CNTFR//EN1//UNC5B//ADNP//GABRA5//GRIK2//ISL1//JUN//NTF3//NR4A2//PIK3CA//POU4F1//RASA1//CCL2//ITSN1//C5AR1//GDF5//SYNGAP1// |
| GO:0033002 | muscle cell proliferation | Biological process | 25 | 211 | 902 | 17653 | 2.31883334559326 | 7.95799622286723e-05 | 0.00208604373857562 | 4.09919627132036 | 0.0277161862527716 | MYOG//S1PR1//EDN1//EREG//FOXP1//ID2//IGF1//MYB//IRAK4//TGFBR2//TNF//IFNG//IGFBP3//OGN//PPARG//TNFAIP3//ARID2//GJA1//JARID2//KCNK2//PTEN//NOG//ZFPM2//JUN//FOXJ2// |
| GO:0040008 | regulation of growth | Biological process | 57 | 662 | 902 | 17653 | 1.68511230498188 | 8.00960344496463e-05 | 0.00209146517754038 | 4.09638898527992 | 0.0631929046563193 | C8ORF44-SGK3//SGK2//SGK3//NANOS1//IGFBP3//RB1//SGK1//SPOCK1//IL2//SIRT1//FRZB//GJA1//PPARG//PPP2CA//BMPR2//SMARCA2//BTG1//WT1//ADIPOR2//SLIT2//EI24//DPYSL2//RAB21//TNF//IGF1//PIK3CA//GDF5//ADRB1//POU3F2//TGFBR2//ADNP//NRG1//TNFRSF12A//PPM1F//PTEN//ARX//SERP1//DLL1//AR//ULK2//WNT3//PLXNA4//SEMA4C//JARID2//KCNK2//NOG//ZFPM2//EDN1//SMURF1//SYT1//PLAA//LRP12//IFNG//ZMAT3//SOCS1//SOCS3//SOCS5// |
| GO:0040011 | locomotion | Biological process | 130 | 1845 | 902 | 17653 | 1.37898316898912 | 8.11829870737338e-05 | 0.00211169439069101 | 4.09053497314964 | 0.144124168514412 | EDNRB//ISL1//SEMA4C//ACVR1//GJA1//GPM6A//LRP12//NR4A2//SPOCK1//FZD3//RAPGEF2//ID2//EFNB2//KDR//SLIT2//CCL21//CCL2//CCL8//EDN2//CXCL9//CD2AP//NCK1//TNFRSF12A//RALBP1//S1PR1//PIK3CB//PLP2//MAPK1//C5AR1//FOSL1//RANBP9//ARX//DPYSL2//EFNA1//GLI3//OTX2//PIK3CA//ENAH//ROBO2//SCN1B//WNT3//KLF7//RPS6KA5//NRXN3//FEZ2//NOG//EDN1//ETS1//FOXP1//RHOB//BMPR2//WNT7A//NANOS1//STRAP//PFN2//PTPRG//IFNG//JUN//TGFBR2//PPM1F//TMEM201//TNS1//ARID5B//PRR5L//C5ORF30//IGF1//IGFBP3//RASGEF1A//PALLD//ARC//BAMBI//RND3//PAK6//PTEN//SDC1//BTG1//YES1//FAM83D//VPS4B//POU4F1//PLXNA4//POU3F2//NRG1//EMX2//FLNA//ADCY3//C8ORF44-SGK3//SGK3//DOCK10//SGK1//SLK//F3//FOXF1//FAM89B//NTF3//PTP4A1//CBLL1//ARID2//SULF1//ZMYND8//TMEFF2//CLIC4//RAP2C//LDLRAD4//RECK//NAV3//DLG5//UNC5B//EPS8//SIRT1//PPARG//APCDD1//SIRPA//MSN//TNF//MET//VEGFB//LPAR1//NFIB//SPRED1//MEOX2//ARRDC3//CHMP3//IRAK4//WDR1//EREG//MEMO1//RAF1//GCSAML//PLAA// |
| GO:0007409 | axonogenesis | Biological process | 43 | 456 | 902 | 17653 | 1.84550657408488 | 8.21027420328165e-05 | 0.00212743618531777 | 4.08564233825406 | 0.0476718403547672 | RANBP9//ARX//DPYSL2//EFNA1//EFNB2//GLI3//OTX2//PIK3CA//PIK3CB//ENAH//MAPK1//ROBO2//SCN1B//WNT3//KLF7//RPS6KA5//SLIT2//NRXN3//FEZ2//NOG//PLXNA4//NR4A2//PTEN//NFIB//RAB21//WNT7A//ISL1//UNC5B//FZD3//ADNP//NRG1//TNFRSF12A//ULK2//SLC9A6//BMPR2//SEMA4C//POU3F2//TRAK2//SYNGAP1//SLITRK3//OGN//POU4F1//PRELP// |
| GO:0060548 | negative regulation of cell death | Biological process | 79 | 1005 | 902 | 17653 | 1.5384132552316 | 8.34644691504004e-05 | 0.00215446643077923 | 4.07849836446744 | 0.0875831485587583 | FOXP1//IL2//HIPK3//DUSP1//EDNRB//ATF5//FLNA//ARL6IP1//SIRT1//KLHL20//GLI3//IGF1//KDR//MDM4//MET//ROR1//OGG1//ZFAND6//PRKAA1//SPHK2//PTEN//RAF1//RASA1//BCL2A1//BNIP2//TMBIM6//TFAP2A//WNT7A//WT1//BAG6//CASP2//TP63//CCND2//SOCS3//ADCYAP1//DLL1//CCL2//EDN1//LRP6//POU4F1//DNAJB6//CEBPB//CNTFR//EN1//UNC5B//ADNP//GABRA5//GRIK2//ISL1//JUN//NTF3//NR4A2//PIK3CA//ITSN1//C5AR1//GDF5//SYNGAP1//EFNA1//FZD3//TNFAIP3//SERPINB13//BAG5//TSC1//CFDP1//CCL21//NEUROD1//SRSF6//CAST//NOG//ZMYND11//AR//ACVR1//TNF//ZFPM2//NCK1//NPAS2//SOX4//TEAD2//VPS4B// |
| GO:0010467 | gene expression | Biological process | 347 | 5761 | 902 | 17653 | 1.17880938076238 | 8.64780497122935e-05 | 0.00222376825172715 | 4.06309411332787 | 0.38470066518847 | NPAT//TSHZ1//ZBTB18//CEBPB//TCERG1//STRAP//KLF12//GSC//ARX//DNMT3B//EDN1//EDNRB//EFNA1//CC2D1B//EN1//EP300//ESR1//JAZF1//CPEB3//FOXF1//MYT1L//SIN3B//SIRT1//ZFPM2//DNAJB5//GLI3//CNOT7//HIC1//FOXA1//HSBP1//ID2//IFNG//IRF2//AR//ISL1//JARID2//JUN//MAF//MDM4//MEF2A//MYB//NFIA//NFIB//NFIC//NR4A2//PAX5//KLF3//POU4F1//PPARG//ZBTB4//RB1//RBBP8//CCND1//RNF2//SARS//PRDM16//SMARCA2//SUV39H1//ZEB1//TFAP2A//TGIF1//KLF10//TNF//UBE2I//WT1//ZNF217//LRP8//BHLHE41//TBL1XR1//E2F8//ARID5B//BHLHE40//TP63//RUNX3//NOG//FOXP2//PHF14//PNRC1//DCP2//PPP2CA//CNOT6L//PAN3//CNOT6//PATL1//SRSF6//MBNL2//RBFOX3//FMR1//MBNL1//RBM25//SRSF2//ELAVL2//NOVA1//PCBP2//RBM41//RRP15//AGO1//GTF2E1//SEPSECS//SECISBP2L//RPP14//RPP25//F3//GTF2H1//CREBRF//ELK3//ETV5//ATF5//FOXF2//FLI1//GTF2A1//HIVEP2//IRF1//MEOX2//MYBL1//MYCN//NEUROD1//NHLH2//OTX2//KLF13//PKNOX1//POU3F2//FOXJ2//HIVEP3//BMPR2//SOX4//SOX5//BTF3//TFE3//TXK//FOSL1//KLF7//CBFB//FUBP3//CCNT2//NMI//CLOCK//ZNF516//POLR3H//SLBP//BAHD1//H2AFZ//ARID4B//DIRAS3//ZMYND11//ZNF526//CREBL2//ZNF800//E2F5//ZNF367//ARID2//MED19//ZFP30//KDM2A//MYCBP2//PHF8//FLII//ADNP//ZMYND8//ZNF521//EPC2//HBP1//FOXP1//ZBTB11//ATAD2//MDFIC//HMGB3//HOXA3//HOXD1//ZNF680//ZNF662//NEUROG1//NFE2//NPAS2//ASCC1//IER5//COMMD10//BRWD1//BNC2//MED9//ZNF532//PRKAA1//TRERF1//MAPK1//SLC2A4RG//CCNL1//PHTF2//ZBTB26//MIER1//PURA//ATXN1//ATXN7//SMARCD2//SS18//MED22//ZNF3//ZNF708//ZNF131//ZNF227//ZNF655//ZSCAN5A//SAP30L//ANP32A//PPP1R1B//PCGF5//LCOR//ST18//HIPK3//PPARGC1B//ELL2//NR5A2//NRBF2//AFF1//MLLT6//OGG1//PHF20L1//PAK6//RORA//BTG1//UBE2V1//CSDE1//SNIP1//TEAD2//RPS6KA5//EMX2//ETS1//BOLA3//NR3C2//RFX7//TRAK2//BDP1//NRBP1//CELF1//GRSF1//RBMS1//RTCA//PPP4R2//CPEB1//QKI//THG1L//AGFG1//NUP54//NXF2//NXF2B//CPEB2//DDX3Y//GTPBP1//DDX6//TSC1//KBTBD8//SPPL3//IMMP2L//CPD//CASP2//G3BP2//EREG//TOB1//RICTOR//IGF1//PIK3CA//PPP2CB//MAPK6//PTHLH//TGFBR2//NANOS1//RAB3GAP1//GJA1//ITGB8//LDLR//MSN//PIK3CB//PTEN//WNT3//WNT10A//CD3E//PPM1F//TBK1//ANXA7//KDR//NDFIP2//SERP1//PRR16//FXR1//INTS8//FLNA//BAMBI//LRP6//WNT7A//ACVR1//DNAJB6//NRG1//RASD1//BTAF1//TIA1//MEX3B//TSN//CR2//C5AR1//SMURF1//BMP3//GDF5//SULF1//LEMD3//CTDSPL2//TNRC6B//RWDD3//TNFAIP3//GFPT1//INSIG2//NCK1//MYOG//NABP1//EGLN1//FZD6//FASTK//YTHDF2//PSMA2//PSMD12//ADCYAP1//S1PR1//KPNA6//DLL1//IL2//MET//PPP1R12A//ARMCX3//CYTL1//PPP3R1//MAVS//RAF1//YES1//PAIP2//BOLL//C8ORF44-SGK3//SGK3//SGK1//RHEBL1//ROR1//IRAK4//PRR5L//EGLN3//PRKACB//CAST//PPP1R15B//TMBIM6//FBXW7//NPNT// |
| GO:0043009 | chordate embryonic development | Biological process | 50 | 560 | 902 | 17653 | 1.74740655685778 | 8.98723221402454e-05 | 0.00230229740391848 | 4.04637403677085 | 0.0554323725055432 | RDH10//EDN1//TAPT1//FOXF1//ZFPM2//TANC2//GJA1//GLI3//AR//MBNL1//BTF3//TGFBR2//FOSL1//CCM2//ACVR1//NOG//EP300//DLL1//MEOX2//RBBP8//SOX4//LRP6//SEMA4C//PRKACB//TSC1//FZD3//FZD6//TEAD2//CEBPB//KBTBD8//GSC//FOXA1//EN1//PAX5//TFAP2A//HOXA3//MYCN//ZEB1//SLC35D1//SULF1//HOXD1//ISL1//SOCS3//E2F8//DNAJB6//EGLN1//MAPK1//VASH2//BMPR2//IGF1// |
| GO:0065008 | regulation of biological quality | Biological process | 239 | 3770 | 902 | 17653 | 1.24070500567557 | 9.25408679946287e-05 | 0.00236171279338745 | 4.03366643131646 | 0.264966740576497 | PTGES3//SNAP25//CHRNB4//GNAQ//KCNB1//ADRB1//EDN2//ARRDC3//SIRT1//DOCK10//PMAIP1//NANOS1//RB1//TNFAIP3//CALM2//ARL6IP1//RDH10//F3//AR//EDN1//EDNRB//BMPR2//ADCY3//PRKACB//PRKAR2B//GJA1//EPS8//ZFPM2//IFNA1//IRF1//IRF2//NFE2//DNAJC16//GLRX//NHLRC2//RORA//ATP2C1//GRIK2//ANXA7//PIK3CB//SLC24A3//STIM2//CCL8//SLC31A2//ABCB7//EGLN1//SKP1//CLCN6//SLC26A4//CLIC4//ADCYAP1//SPPL3//LPAR1//ESR1//NPTN//IL2//LRP6//C5AR1//RIC3//G3BP2//SYT1//WNT7A//PPFIA1//CCND1//FLI1//PPP1R1B//RAPGEF4//SLC25A6//IFNG//MARCKS//NEUROD1//RAP1A//TNF//CLOCK//UGT2B7//DLL1//PPARG//SGK1//CFDP1//WASF3//PALM2//BAMBI//KDR//RHOB//RND3//MSN//BRWD1//RASA1//CCL2//PLXNA4//WDR1//TMBIM6//TRPC3//TXK//FBXW7//ABHD5//SLC41A1//PLAA//KCNJ2//INHBB//CRTAP//CREBL2//EFNA1//EP300//FLNA//OTUD3//MORC3//IGF1//USP27X//MDM4//PFN2//PTEN//MTMR9//SOX4//TSC1//BAG6//CCT6A//RASSF2//SCN1B//CPD//SNX19//DGKH//PIK3CA//MAPK1//RAF1//FZD6//DGKE//PKNOX1//STRAP//KCNK2//KCNK10//DPYSL2//RAB21//GPCPD1//CFL2//SLIT2//RICTOR//NCK1//CCL21//USP28//MYLIP//SERP1//ISL1//DCP2//DBN1//ADNP//IQGAP2//ARFIP1//TAPT1//ANO1//SYBU//SLC26A7//CNGA3//RIMS4//KCNH4//GABRA5//GLRB//PXK//GLRA3//GCNT4//SLC44A1//FOXA1//ADH4//RDH14//DHRS3//ARID2//NR5A2//TRERF1//PRKAA1//ADIPOR2//LDLR//YES1//ID2//FMR1//YTHDF2//PSMA2//PSMD12//FXR1//ANP32A//S1PR1//GPR55//TUB//KLF13//ETS1//NRG1//TNFRSF12A//PPARGC1B//TMEM64//PRR16//KLHL3//CPEB3//YWHAG//SYNGAP1//RAPGEF2//ARC//KCNJ10//RAB3GAP1//ULK2//WNT3//SYT6//TMOD3//SEMA4C//HOMER1//ETV5//DZIP1//CXCL9//SLC9A6//CRHR1//RAB8B//TMOD1//JUN//ROBO2//SLITRK3//DLG5//CUTC//STEAP2//SLC25A28//KCNE1//LRP8//SLC30A6//CNOT6L//QKI//MET//TP63//SLC16A2//XKR8//BOLL//GJC1//KCNJ3//FAM155A//ZMYND8//NSG1//GPHN//PAIP2//DYNLL2//PPP3R1//NEUROG1//CYTL1//MYB// |
| GO:0016310 | phosphorylation | Biological process | 160 | 2367 | 902 | 17653 | 1.32292038440606 | 9.43833925097779e-05 | 0.00239968001332191 | 4.02510441646333 | 0.177383592017738 | GADD45A//GTF2H1//PTEN//CCNT2//DIRAS3//RANBP9//SPRED1//EREG//RASGEF1A//NRG1//IL2//MEF2A//MET//MAPK1//MAPK6//PSMA2//PSMD12//RAF1//RASA1//CCL2//TNF//SYNGAP1//RAPGEF2//CRKL//TAOK1//RAP1A//C1QTNF2//LPAR1//IGF1//NTF3//PIK3CB//PRKAA1//C5AR1//UBE2V1//DUSP1//PPP2CA//SOCS1//IGFBP3//LRP6//PRR5L//PPP1R15B//SLIT2//EDNRB//EFNA1//FMR1//SIRT1//NPTN//IFNG//KDR//MAVS//CCND1//CCND2//FBXW7//ERRFI1//GPRC5A//SOCS5//NUP54//COX8C//CMPK1//C8ORF44-SGK3//SGK2//HIPK3//FASTK//PDIK1L//CDK19//MORC3//MMD//SGK3//PAN3//GRK6//TBK1//NRBP1//CDK17//PIK3CA//PRKACB//CCL8//SGK1//CDK15//TGFBR2//TXK//MEX3B//RUNX3//ACVR1//RPS6KA5//RASSF2//GNAQ//NCK1//GSKIP//RB1//YWHAG//SOCS3//PPM1F//COPS8//IRAK4//MDFIC//CALM2//INHBB//BMP3//BMPR2//TTK//GDF5//RICTOR//STK33//ROR1//KCNH4//PAK6//SLK//MOB1B//JUN//RAP2C//ADNP//MID1//SEMA4C//CREBL2//PFN2//IFNA1//ADCY3//PRKAR2B//YES1//ATXN7//PTGES3//PPARGC1B//AR//ITSN1//CNOT7//ISL1//EDN1//PDE5A//NRBF2//CCL21//PPP1R1B//TNFAIP3//CCNY//CCNL1//MYOG//TSC1//ADCYAP1//CACUL1//GRIK2//ZMYND11//WNT7A//ULK2//SPHK2//DGKE//GNPTG//ESR1//EFR3A//LRP8//CD3E//SKP1//CCM2//STRAP//PMEPA1//LDLRAD4//NOG//FAM83D//NPNT//GPR55//ACSL1//DGKH// |
| GO:0071840 | cellular component organization or biogenesis | Biological process | 385 | 6486 | 902 | 17653 | 1.16170475997082 | 9.49822345871156e-05 | 0.00240586087083394 | 4.0223576172615 | 0.426829268292683 | MEF2A//UBXN2B//TRAPPC8//GABARAPL1//ATG16L1//WDR45B//ULK2//SEPT9//SIRT1//SUV39H1//TACC2//CAMSAP1//DYNC1LI2//CAMSAP2//MID1//ATXN7//SS18//MIEF1//STRAP//RRP15//COPS8//GTF2H1//PTGES3//CDH20//NRG1//WNT3//GTF2E1//C8ORF44-SGK3//SGK2//SGK3//NANOS1//IGFBP3//RB1//SGK1//SPOCK1//CLIP1//AGFG1//JUN//PMAIP1//BCL2A1//CLIC4//MET//FMR1//NTF3//PALLD//AR//FRMD6//NAP1L3//HIST2H2BE//EPS8//RHOB//RND3//MARCKS//CFL2//WDR1//DNAJB6//IQSEC2//FLII//PFN2//PAK6//WASF3//SH3D19//DPYSL2//ARC//KLHL20//MSN//BRWD1//CCL2//CCDC6//STX6//SYT6//SNAP25//VAMP1//SYT1//VAMP3//C2CD5//ZFAND6//PURA//ZMYND11//PHF13//ATAD2//H2AFZ//JARID2//BAG6//ARID2//NFE2//SMARCD2//ESR1//HMGB3//FOXA1//MYB//SMARCA2//ANP32A//TP63//BAHD1//RPP14//RPP25//CELF1//SRSF6//TSC1//TIMM9//SLC25A6//MTX3//IMMP2L//CLCN6//GULP1//ARHGAP12//CD2AP//NCK1//VPS4B//REEP3//LEMD3//NUP54//SERP1//PALM2//PPARGC1B//DBN1//SAMD14//TMOD3//TMOD1//STMN2//RANBP9//VBP1//MID1IP1//NAV3//SPTSSB//TMED1//RAB30//GOLGA6A//TJAP1//BAG5//GJC1//TNS1//TTK//DYNLL2//FBXW7//TAOK1//CENPO//CNEP1R1//PPP2CA//CDC14A//GADD45A//PPP1R12A//SYCE1//MYBL1//EREG//CD3E//FOSL1//SLITRK3//OGN//POU4F1//PRELP//WNT7A//SLIT2//ARX//EFNA1//EFNB2//GLI3//OTX2//PIK3CA//PIK3CB//ENAH//MAPK1//ROBO2//SCN1B//KLF7//RPS6KA5//NRXN3//FEZ2//GPM6A//PCDHB10//PTEN//BSN//PDZRN3//GPHN//NOG//KATNAL1//CFDP1//LPAR1//BAMBI//ANXA7//KDR//RASA1//PLXNA4//PPP2CB//ARHGAP6//TMEM17//DZIP1//FLNA//BBS9//GMNC//C5ORF30//CEP41//SKP1//CNOT6L//PAN3//CNOT6//ETS1//CHMP3//ADCYAP1//NPTN//SERPINI1//RAP1A//RAPGEF2//MYLIP//PTPRG//PMEPA1//LDLRAD4//EPS15//SNX5//SNX2//GJA1//STRN//SYNGAP1//PEX5L//EP300//EPC2//CLOCK//SIN3B//ARID4B//MIER1//SAP30L//TBL1XR1//PPM1F//ARID5B//NR4A2//NFIB//E2F5//TAPT1//S1PR1//ARHGEF4//HECW2//RAB21//SNX17//HAPLN1//FOXF1//FOXF2//NPNT//ITGB8//SMOC2//TNF//ADAM12//MFAP5//RECK//IL2//FRZB//PPARG//BMPR2//BTG1//WT1//ADIPOR2//EI24//FAM118B//TDRD5//RICTOR//CCL21//CNN3//LIMCH1//ISL1//NREP//SLK//LRP12//PSD//RAF1//IFNG//TBC1D12//RAB8B//ATP2C1//NEUROG1//DCP2//CCT6A//KIF13A//SULF1//PHF8//UNC5B//DDX6//PATL1//CNOT7//G3BP2//IQGAP2//ARFIP1//RASSF8//PRKACB//RAB3GAP1//RAB18//PRDM16//AGO1//PRKAA1//RNF2//ZMYND10//VPS37A//PCGF5//FZD3//APOO//C1QTNF2//KCNA4//KCNB1//SLC6A1//GLRA3//ZYX//TGM3//XKR8//PPP1R1B//VASH2//DLG5//CCM2//TMEFF2//ADNP//TNFRSF12A//EDN1//PRR16//DLL1//LDLR//CBLL1//IGF1//ATF5//CNIH1//SEC23A//MCFD2//NSG1//VTI1A//TRAK2//ID2//SLC9A6//HPRT1//FXR1//SEMA4C//SIRPA//TUB//POU3F2//ETV5//CUTC//SBF2//DGKH//TNFAIP3//KCNJ2//THG1L//CHRNB4//GLRB//MCCC2//MZT1//PPP1R16B//ZMYND8//PPFIA1//DNMT3B//PAX5//NFIA//SYBU//ACSL4//DOCK10//CPEB3//MAPK6//LRP8//CHRM2//ITSN1//YWHAG//SGCE//SMURF1//MDM4//TEAD2//SKAP2//MGST1//C1QTNF6//KDM2A//BDP1//RTN3//ARL6IP1//DUSP1//ANAPC15//NEDD1//PRKAR2B//YTHDF2//CRKL//MAVS//PPP3R1//MYOG//CXCL9//RAP1B//SDC1//PLAA//NMUR1//SNIP1//LRP6//SNX19//CBFB// |
| GO:0032870 | cellular response to hormone stimulus | Biological process | 59 | 698 | 902 | 17653 | 1.65427829909974 | 0.000100300013155383 | 0.0025310783170517 | 3.99869901001737 | 0.065410199556541 | CHRM2//GNAQ//APPL1//NR5A2//PPARG//ADIPOR2//ESR1//PPARGC1B//AR//PMEPA1//RB1//CPEB2//INHBB//RAB8B//ERRFI1//PTEN//CPEB1//YWHAG//C2CD5//UBA5//CBFB//ISL1//TP63//FOXA1//SIRT1//ACSL1//PIK3CA//NR3C2//NR4A2//RORA//NCK1//TSC1//SOCS1//SOCS3//IGF1//MAPK1//EP300//FOXP1//WT1//GJA1//EDN1//CRHR1//ADCY3//PRKACB//PRKAR2B//PRKAA1//ADCYAP1//H2AFZ//MYOG//POU4F1//MSN//DNMT3B//RAP1B//CREBRF//CLOCK//DUSP1//JUN//ROBO2//SLIT2// |
| GO:0030855 | epithelial cell differentiation | Biological process | 63 | 760 | 902 | 17653 | 1.62232903489322 | 0.000102643569576611 | 0.00258058907452275 | 3.98866825316013 | 0.0698447893569845 | CCM2//CLIC4//MET//ESR1//TP63//GJA1//DLL1//NEUROD1//PALLD//AR//FRMD6//GSTM3//WNT7A//EREG//TGM3//IFNG//CCND1//DLG5//KRT80//KRTAP5-6//KRTAP2-4//JUN//FOXF1//MSN//S1PR1//ZEB1//BTG1//MYCN//SERPINB13//ERRFI1//CBFB//SRSF6//SDC1//WT1//NFIB//FOXA1//PPP3R1//SOX4//YIPF6//CEBPB//BMPR2//NRG1//PPP1R16B//RAP1A//RAP1B//RAPGEF2//ID2//ACVR1//MAF//TMOD1//E2F8//FRZB//ARX//RAP2C//ARID4B//FOXJ2//TNF//CLOCK//CNN3//NR5A2//GSTA2//ANXA7//PPARG// |
| GO:0003006 | developmental process involved in reproduction | Biological process | 57 | 669 | 902 | 17653 | 1.66748033766518 | 0.000106240691772519 | 0.0026611325868798 | 3.97370911011452 | 0.0631929046563193 | CEBPB//ADCYAP1//INHBB//IMMP2L//SIRT1//ESR1//EREG//CASP2//YTHDF2//AGFG1//PPARG//E2F8//STOX2//BMPR2//CELF1//DZIP1//RNF2//BOLL//WT1//ACVR1//TDRD5//SMARCA2//QKI//ZFPM2//CNTFR//AR//WNT7A//RDH10//RNF38//CSDE1//ARID5B//GLI3//MGST1//CCND1//NEUROG1//LRP6//GJA1//ETV5//FOXF2//TP63//SDC1//PDE5A//IGF1//NOG//FOXA1//SOCS3//ITGB8//FOSL1//SULF1//DNAJB6//EGLN1//MAPK1//VASH2//PTEN//ROBO2//SLIT2//ARID4B// |
| GO:0050804 | modulation of chemical synaptic transmission | Biological process | 34 | 335 | 902 | 17653 | 1.9863057219446 | 0.000111103521038118 | 0.00276247467934115 | 3.95427217739402 | 0.0376940133037694 | SNAP25//PTEN//SLC6A1//EDN1//CHRNB4//FMR1//RIMS4//CPEB3//YWHAG//SYNGAP1//RAPGEF2//DBN1//ARC//GRIK2//KCNJ10//NPTN//RAB3GAP1//ADNP//SYT1//GRM3//ADCYAP1//SYBU//MAPK1//ZMYND8//FXR1//NSG1//PAIP2//KCNB1//PFN2//RAP1A//WNT7A//NTF3//PXK//LRP8// |
| GO:0099177 | regulation of trans-synaptic signaling | Biological process | 34 | 335 | 902 | 17653 | 1.9863057219446 | 0.000111103521038118 | 0.00276247467934115 | 3.95427217739402 | 0.0376940133037694 | SNAP25//PTEN//SLC6A1//EDN1//CHRNB4//FMR1//RIMS4//CPEB3//YWHAG//SYNGAP1//RAPGEF2//DBN1//ARC//GRIK2//KCNJ10//NPTN//RAB3GAP1//NTF3//PXK//LRP8//ADNP//SYT1//GRM3//ADCYAP1//SYBU//MAPK1//ZMYND8//FXR1//NSG1//PAIP2//KCNB1//PFN2//RAP1A//WNT7A// |
| GO:0022612 | gland morphogenesis | Biological process | 17 | 119 | 902 | 17653 | 2.79585049097244 | 0.000113757522267344 | 0.00281810301499651 | 3.94401987580329 | 0.0188470066518847 | TGFBR2//LRP6//TP63//NOG//AR//ESR1//GLI3//NFIB//TNF//SULF1//BTBD7//FOXA1//ETV5//CEBPB//FBXW7//TNFAIP3//MSN// |
| GO:0044249 | cellular biosynthetic process | Biological process | 389 | 6579 | 902 | 17653 | 1.15718207735491 | 0.000121110527715224 | 0.00298930839028489 | 3.9168181035134 | 0.431263858093126 | SIRT1//IGF1//NFIA//NFIB//NFIC//RBBP8//RBMS1//NPAT//TSHZ1//ZBTB18//CEBPB//TCERG1//STRAP//KLF12//GSC//ARX//DNMT3B//EDN1//EDNRB//EFNA1//CC2D1B//EN1//EP300//ESR1//JAZF1//CPEB3//FOXF1//MYT1L//SIN3B//ZFPM2//DNAJB5//GLI3//CNOT7//HIC1//FOXA1//HSBP1//ID2//IFNG//IRF2//AR//ISL1//JARID2//JUN//MAF//MDM4//MEF2A//MYB//NR4A2//PAX5//KLF3//POU4F1//PPARG//ZBTB4//RB1//CCND1//RNF2//SARS//PRDM16//SMARCA2//SUV39H1//ZEB1//TFAP2A//TGIF1//KLF10//TNF//UBE2I//WT1//ZNF217//LRP8//BHLHE41//TBL1XR1//E2F8//ARID5B//BHLHE40//TP63//RUNX3//NOG//FOXP2//PHF14//GTF2E1//SEPSECS//SECISBP2L//PTGES3//EDN2//MGST1//ST3GAL5//RDH10//GTF2H1//CREBRF//ELK3//ETV5//ATF5//FOXF2//FLI1//GTF2A1//HIVEP2//IRF1//MEOX2//MYBL1//MYCN//NEUROD1//NHLH2//OTX2//KLF13//PKNOX1//POU3F2//FOXJ2//HIVEP3//BMPR2//SOX4//SOX5//BTF3//TFE3//TXK//FOSL1//KLF7//CBFB//FUBP3//CCNT2//NMI//CLOCK//ZNF516//POLR3H//SRSF2//SRSF6//SLBP//GFPT1//SLC35D1//NUP54//HPRT1//ADSS//ADCY3//IMPDH1//CMPK1//PURA//GMNC//ESCO2//PPP2CA//BAHD1//H2AFZ//ZMYND11//PNRC1//ZNF526//CREBL2//ZNF800//E2F5//ZNF367//ARID2//MED19//ZFP30//KDM2A//MYCBP2//PHF8//FLII//ADNP//ZMYND8//CNOT6L//ZNF521//EPC2//AGO1//HBP1//FOXP1//ZBTB11//ATAD2//MDFIC//HMGB3//HOXA3//HOXD1//ZNF680//ZNF662//NEUROG1//NFE2//NPAS2//ASCC1//IER5//COMMD10//ARID4B//BRWD1//BNC2//MED9//ZNF532//PRKAA1//TRERF1//MAPK1//SLC2A4RG//CCNL1//PHTF2//CNOT6//ZBTB26//MIER1//ATXN1//ATXN7//SMARCD2//SS18//MED22//ZNF3//ZNF708//ZNF131//ZNF227//ZNF655//ZSCAN5A//SAP30L//ANP32A//PPP1R1B//PCGF5//LCOR//ST18//HIPK3//PPARGC1B//ELAVL2//ELL2//NR5A2//NRBF2//AFF1//MLLT6//OGG1//PHF20L1//PAK6//RORA//BTG1//UBE2V1//CSDE1//SNIP1//TEAD2//RPS6KA5//EMX2//ETS1//BOLA3//NR3C2//RFX7//TRAK2//BDP1//NRBP1//CPEB2//CPEB1//DDX3Y//GTPBP1//DDX6//TSC1//KBTBD8//FUT9//B3GALNT2//FUT2//FUT5//SERP1//ST8SIA5//KCNE1//CCDC126//ALG2//EOGT//GCNT4//VEGFB//WDR45B//ALPI//RECK//PYURF//PIGP//PIGA//SERINC3//PSAT1//ELOVL5//LCLAT1//ABHD5//CDS1//ABHD3//SLC44A1//PIK3CA//PIK3CB//PTEN//MTMR9//SPHK2//GPHN//GRM3//G3BP2//RIMKLB//LPCAT3//DGKE//MTRR//GLT6D1//EREG//CCL21//CRHR1//CNEP1R1//LDLR//FBXW7//HS3ST5//ARPP19//FMR1//PRR16//FXR1//FLNA//BAMBI//LRP6//WNT7A//ACVR1//DNAJB6//NRG1//RASD1//BTAF1//PPM1F//TOB1//CELF1//NANOS1//TIA1//MEX3B//OGN//PRELP//CHST1//ZDHHC22//ZDHHC7//ZDHHC18//MCFD2//ABHD17C//ELOVL7//SPTSSB//SPTSSA//DSEL//SMURF1//BMP3//GDF5//SULF1//LEMD3//CTDSPL2//ADRB1//PTHLH//CALM2//EGLN1//RWDD3//TNFAIP3//DCP2//CCT6A//INSIG2//TNRC6B//ACSL1//ACSL4//NCK1//ADIPOR2//ERRFI1//QKI//MYOG//C5AR1//INTS8//NABP1//FZD6//SMUG1//CD3E//TBK1//MID1IP1//C1QTNF2//ADCYAP1//S1PR1//KPNA6//DLL1//IL2//MET//PPP1R12A//ARMCX3//CYTL1//PPP3R1//MAVS//RAF1//YES1//PAIP2//BOLL//PDE5A//RAPGEF2//INHBB//C8ORF44-SGK3//SGK3//SGK1//RHEBL1//ROR1//IRAK4//YTHDF2//EGLN3//PSMA2//PSMD12//ATG16L1//GSTA2//GSTM3//RAB3GAP1//RAP2C//HSBP1L1//PPP1R15B//TMBIM6//DUSP1//GJA1//KCNK2//NPNT// |
| GO:0030324 | lung development | Biological process | 21 | 167 | 902 | 17653 | 2.46101809684401 | 0.000125014574681785 | 0.00307444934026513 | 3.90303935231283 | 0.0232815964523282 | EDN2//FOXF1//ERRFI1//BMPR2//FOXP2//PHF14//MAPK1//NOG//RDH10//FOXA1//DLG5//TGFBR2//NFIB//PPP3R1//SRSF6//TNF//EP300//ZFPM2//GLI3//MYCN//BAG6// |
| GO:0034613 | cellular protein localization | Biological process | 124 | 1762 | 902 | 17653 | 1.37729751768677 | 0.000127481582968302 | 0.00312376067251676 | 3.89455255242979 | 0.137472283813747 | TNPO2//IFNG//TNF//PMAIP1//NUP54//ZFAND6//AGFG1//NXF2//SRSF2//SRSF6//NXF2B//SLBP//TSC1//PAN3//TRAK2//YWHAG//KPNA6//SMURF1//ZDHHC22//ZDHHC7//ZDHHC18//ARL6IP1//VTI1A//TIMM9//SLC25A6//MTX3//IMMP2L//STX6//SEC23A//TMED1//RAB12//RAB18//RAB21//TBC1D12//SNX5//ARFIP1//RAB30//GRIK2//RHOB//RAB9B//RAB8B//KIF13A//SNX2//SNX27//AP1S2//RAB28//SNX17//GNPTG//PEX5L//TRAPPC8//DBN1//SIRT1//OGG1//IGF1//MAPK1//MAVS//MZT1//FLNA//RIC3//ATG16L1//WDR45B//TTK//DNAJB6//CNEP1R1//MID1//TAPT1//GLI3//MDFIC//C16ORF70//VAMP3//SYNGAP1//CLIP1//SOX4//CTDSPL2//MORC3//SKP1//BBS9//TUB//C2CD5//ESCO2//ESR1//ETV5//RB1//NEDD1//BAG6//GPHN//EFR3A//KCNB1//LRP6//KCNIP4//RAPGEF2//TNFAIP3//MIEF1//KCNE1//PRR5L//RAB3GAP1//GLRB//NSG1//SNAP25//CD2AP//DYNLL2//PPP3R1//TP63//FAM83D//ABHD17C//ZMYND8//MSN//APPL1//AR//PPFIA1//ARL5A//TIA1//NPTN//NMUR1//NRG1//FBXW7//PRKAA1//SNIP1//CCT6A//KCNAB2//SEPT9//FRMD6//ARMCX3//RRAGD// |
| GO:0048646 | anatomical structure formation involved in morphogenesis | Biological process | 86 | 1133 | 902 | 17653 | 1.48552691576823 | 0.000135476155793968 | 0.0033076723524715 | 3.86813713518325 | 0.0953436807095344 | VASH2//S1PR1//EFNA1//ELK3//EREG//UNC5B//SIRT1//LEMD3//CLIC4//HOXA3//JUN//KDR//RHOB//MEOX2//TNFRSF12A//PIK3CA//PKNOX1//PTEN//RORA//CCL2//VEGFB//WNT7A//EDN1//PPP3R1//TGFBR2//ACVR1//AGFG1//DUSP1//NOG//BMPR2//WNT3//EP300//FOXF1//DLL1//SOX4//LRP6//SEMA4C//PRKACB//TSC1//FZD3//FZD6//TEAD2//E2F8//EFNB2//SLIT2//TNFAIP3//ISL1//WT1//TFAP2A//ADAM12//KBTBD8//GSC//SPRED1//SULF1//AGO1//PPARG//FOXJ2//SARS//NEUROG1//GLI3//WDR1//TMOD3//TMOD1//HSBP1//CFL2//ETS1//EGLN1//F3//ITGB8//BTG1//C5AR1//AR//MEF2A//PIK3CB//PPARGC1B//RDH10//MAPK1//TP63//NFIB//ADIPOR2//OTX2//MYOG//CXCL9//PPP1R16B//TNF//FOXA1// |
| GO:0060541 | respiratory system development | Biological process | 23 | 193 | 902 | 17653 | 2.33228978780603 | 0.00013871170591525 | 0.00337448657231955 | 3.85788688716117 | 0.0254988913525499 | EP300//FOXF1//ZFPM2//GLI3//MYCN//BAG6//RDH10//EDN2//ERRFI1//BMPR2//FOXP2//PHF14//MAPK1//NOG//TGFBR2//FOXA1//DLG5//NFIB//PPP3R1//SRSF6//LRP6//WT1//TNF// |
| GO:1902533 | positive regulation of intracellular signal transduction | Biological process | 79 | 1021 | 902 | 17653 | 1.51430491822503 | 0.000139336588090529 | 0.00337753887188619 | 3.85593482816056 | 0.0875831485587583 | GADD45A//TNF//CRKL//TAOK1//RAF1//RAP1A//C1QTNF2//LPAR1//NRG1//IGF1//NTF3//PIK3CB//PRKAA1//MAPK1//C5AR1//UBE2V1//COPS8//MDFIC//SOCS1//UNC5B//SIRT1//KDR//PRR5L//RICTOR//PIK3CA//RRAGD//MID1//SEMA4C//GPR55//DLG5//IFNG//IL2//ISL1//SOCS3//FLNA//GJA1//ATP2C1//TBK1//ROR1//IRAK4//NDFIP2//MAVS//MIER1//CCL21//UBE2I//EDN1//PDE5A//EFNA1//IGFBP3//AR//MET//SPRED1//HIC1//PMAIP1//ADCYAP1//ADRB1//CXCL9//RAPGEF2//WNT7A//RASSF2//RASGEF1A//CD3E//SOS2//EREG//ESR1//F3//ITSN1//CALM2//NPNT//NPTN//JUN//FBXW7//PTEN//CCL2//CCL8//SPPL3//AGO1//SERINC3//NCK1// |
| GO:0031346 | positive regulation of cell projection organization | Biological process | 35 | 353 | 902 | 17653 | 1.94046280534915 | 0.000140560597597675 | 0.00339504043411813 | 3.85213640509258 | 0.0388026607538803 | STMN2//ADCYAP1//NPTN//NCK1//SERPINI1//RAP1A//SCN1B//RAPGEF2//CCL21//TAPT1//ADNP//NRG1//TNFRSF12A//WNT3//BMPR2//ROBO2//PLXNA4//SLIT2//RAB21//FMR1//ZMYND8//GPM6A//LPAR1//CPEB3//IL2//MAPK6//DLG5//LRP8//EPS8//SEPT9//SMURF1//SYT1//PLAA//NAV3//RAB8B// |
| GO:0048584 | positive regulation of response to stimulus | Biological process | 157 | 2334 | 902 | 17653 | 1.31646944712027 | 0.000141506784806105 | 0.00340573090976401 | 3.84922273658343 | 0.174057649667406 | GADD45A//TNF//CRKL//TAOK1//RAF1//RAP1A//C1QTNF2//LPAR1//NRG1//IGF1//NTF3//PIK3CB//PRKAA1//MAPK1//C5AR1//UBE2V1//ARL6IP1//MAVS//EP300//PRKACB//PSMA2//PSMD12//SKP1//RPS6KA5//IRAK4//BAG6//CR2//F3//EDN2//CXCL9//SKAP2//SIRT1//EREG//FBXW7//COPS8//MDFIC//TOB1//EPS8//ARHGAP1//LASP1//ARHGAP6//SH3BGRL//HOMER1//SOCS1//INHBB//BMP3//BMPR2//TTK//GDF5//ACVR1//UNC5B//KDR//PRR5L//MYOG//SPPL3//DENND1B//TNFRSF21//NCK1//PIK3CA//TXK//CD3E//SULF1//SOX4//WNT3//NPNT//RICTOR//RRAGD//IFNG//MID1//SEMA4C//FOXA1//AR//ESR1//IRF1//TNFAIP3//GPR55//DLG5//TBK1//ANO1//SYBU//YES1//IL2//ISL1//SOCS3//FLNA//GJA1//ATP2C1//ROR1//NDFIP2//MIER1//CCL21//UBE2I//EDN1//PDE5A//EFNA1//IGFBP3//MET//TGFBR2//SPRED1//HIC1//PMAIP1//ADCYAP1//ADRB1//RAPGEF2//MYB//SOCS5//NPAS2//NPTN//DLL1//TP63//EVC//RAET1E//WNT7A//RASSF2//RASGEF1A//SLC25A6//ETS1//CDK19//LDLR//CLOCK//GCSAML//PAX5//CBFB//PPM1F//S1PR1//SLIT2//VEGFB//SOS2//ITSN1//CALM2//JUN//PTEN//CCL2//CCL8//ARRDC3//BAMBI//LRP6//GSKIP//TBL1XR1//IER5//KCNK2//DYNLL2//PPP3R1//YWHAG//AGO1//RWDD3//CREBRF//SERINC3//TRPC3//MYCN//FAM168A//ARC//FMR1//CASP2//TNFRSF12A//ST18//C2CD5// |
| GO:1901700 | response to oxygen-containing compound | Biological process | 112 | 1565 | 902 | 17653 | 1.40060497439131 | 0.000145546702375134 | 0.00349054024171288 | 3.8369976298439 | 0.124168514412417 | CHRM2//GNAQ//APPL1//PPP1R1B//HOMER1//ADNP//RAP1B//SLC6A1//GJA1//IGF1//NEUROD1//PTEN//TGFBR2//EDN1//CEBPB//CPEB3//SESN1//RRAGD//CPEB1//ZEB1//TNF//SOCS1//MAPK1//CCL2//DNMT3B//DUSP1//ESR1//ETS1//FOXA1//OGG1//RBBP8//CCND1//WNT7A//C5AR1//TNFAIP3//FOXP1//TNFRSF21//JUN//MGST1//CXCL9//PPARG//FOSL1//SIRT1//INSIG2//SRSF6//TSC1//CPEB2//INHBB//RAB8B//ERRFI1//YWHAG//C2CD5//SRSF2//TRPC3//FOXP2//ACSL1//PPARGC1B//CCL21//PIK3CA//RAF1//ANO1//SYBU//RORA//PPP2CB//SDC1//PPP1R15B//GLRB//GLRA3//EREG//LRP6//BTG1//ADCYAP1//IL2//EPS8//NCK1//SOCS3//SLIT2//CNGA3//RHOB//MYB//PRKAA1//EDNRB//CDK19//PLAA//MYOG//WNT3//YES1//TEAD2//KCNE1//RAP1A//WT1//RAPGEF2//KCNB1//SOX4//CRHR1//NR4A2//ADCY3//PRKACB//PRKAR2B//H2AFZ//POU4F1//AR//MSN//LRP8//LDLR//FIBIN//EGLN1//MET//KLF3//KLF10//TMBIM6//LPAR1// |
| GO:0051179 | localization | Biological process | 384 | 6497 | 902 | 17653 | 1.15672558407479 | 0.000146154312715331 | 0.00349272656146213 | 3.83518836497411 | 0.425720620842572 | TNPO2//IFNG//TNF//SNAP25//SLC26A7//EDNRB//ISL1//SEMA4C//ACVR1//GJA1//GPM6A//LRP12//NR4A2//SPOCK1//FZD3//RAPGEF2//ADCYAP1//EDN1//PMAIP1//ARL6IP1//EFNB2//KDR//SLIT2//FMR1//NTF3//STIM2//NUP54//CCL21//CCL2//CCL8//FLNA//IGF1//NHLRC2//VEGFB//WDR1//EDN2//CXCL9//SLC6A15//SLC6A6//RTN3//KIF1C//ARAP2//APPL1//AGFG1//HSPA13//AP1S2//VAMP3//GIT2//TRAK2//STX6//SYT6//VAMP1//SYT1//C2CD5//ZFAND6//NXF2//SRSF2//SRSF6//NXF2B//SLBP//TSC1//PAN3//YWHAG//KPNA6//SMURF1//ZDHHC22//ZDHHC7//ZDHHC18//VTI1A//TIMM9//SLC25A6//MTX3//IMMP2L//SLC6A1//SLC6A8//SLC6A11//SLC9A6//CHRNB4//GLRB//LASP1//SLC26A4//PLP2//TMEM63B//SLC24A3//CNGA3//ANO1//KCNH4//KCNA4//KCNJ2//KCNJ3//KCNJ10//VPS4B//SGK1//SLC10A2//SLC23A2//NMUR1//ATP2C1//TRPC3//CLCN6//CLIC4//NDFIP2//SLC31A2//CUTC//SLC14A1//SLC25A34//SLC25A28//MICU3//MID1IP1//RALBP1//GULP1//APOO//SEC23A//TMED1//RAB12//RAB18//RAB21//TBC1D12//SNX5//ARFIP1//RAB30//GRIK2//RHOB//RAB9B//RAB8B//KIF13A//SNX2//SNX27//RAB28//SNX17//TXLNA//SNX19//CNIH1//DCTN6//TEX261//DYNLL2//DYNC1LI2//TRAPPC8//NRBP1//IER3IP1//DCTN5//MCFD2//STEAP2//EPS15//KLHL20//DENND1B//DPYSL2//ARC//FKBP15//LDLR//RIN2//LRP8//TGFBR2//KCNB1//TUB//ARHGAP12//NEUROD1//ANP32A//CD2AP//NCK1//TNFRSF12A//TMEM201//G3BP2//WNT7A//PPFIA1//CCND1//RAPGEF4//MARCKS//RAP1A//CLOCK//FXR1//SLC35D1//TMBIM6//ETS1//FOXP1//BMPR2//NANOS1//STRAP//PFN2//PTPRG//JUN//PPM1F//C5AR1//TNS1//ARID5B//PRR5L//C5ORF30//SCN1B//SIRT1//CALM2//FBXW7//PPARG//ABHD5//IGFBP3//VPS37A//LCA5//SERP1//BBS9//CHMP3//AFTPH//ATG16L1//SEC61A2//ZMAT3//BTF3//PLEKHF2//CEP41//SLC41A1//MMGT1//ASNA1//SLC22A23//SLC16A2//SERINC3//SLC44A1//ABCB7//ACSL4//ARPP19//C1QTNF2//CREBL2//ADIPOR2//NSG1//ARHGAP1//GNPTG//S1PR1//EFNA1//RASGEF1A//PALLD//BAMBI//RND3//PIK3CA//PIK3CB//PAK6//PTEN//SDC1//BTG1//YES1//FAM83D//PEX5L//INHBB//SYBU//POU4F1//POU3F2//ARX//GLI3//NRG1//EMX2//ADCY3//C8ORF44-SGK3//SGK3//DOCK10//PLXNA4//SLK//F3//FOXF1//FAM89B//MAPK1//PTP4A1//CBLL1//ARID2//SULF1//ZMYND8//TMEFF2//RAP2C//LDLRAD4//RECK//NAV3//DLG5//NOG//SOX4//SGK2//DBN1//DNAJB6//MMD//OGG1//MAVS//MZT1//GJC1//CLCN5//GABRA5//OSTM1//RAF1//GLRA3//RIC3//WDR45B//TTK//CNEP1R1//SEPT9//FRMD6//ARMCX3//RRAGD//KCNK10//MID1//BSN//TAPT1//EPS8//MDFIC//C16ORF70//SYNGAP1//PXK//IQGAP2//CKAP4//SIRPA//BRI3//ARMC8//IMPDH1//C6ORF120//MGST1//NEU1//PSMA2//KCMF1//PSMD12//RAP1B//KCNAB2//APCDD1//XKR8//ACSL1//CLIP1//DZIP1//NPNT//DLL1//PPP1R12A//CTDSPL2//MEF2A//ITSN1//RIMS4//WNT3//SOCS1//MSN//IL2//QKI//ATXN1//MORC3//SKP1//CRHR1//HOMER1//SLC35F1//BCL2A1//KCNE1//LPAR1//PRKAA1//SLC30A6//RAB3GAP1//LEMD3//KLHL3//CUL5//ESCO2//ESR1//ETV5//RB1//NEDD1//KCNK2//KCNIP4//BAG6//GPHN//EFR3A//LRP6//TNFAIP3//SPRED1//MEOX2//MIEF1//PRKAR2B//FAM155A//PPP3R1//TP63//COX7A2//COX8C//NPTN//ABHD17C//AR//ARL5A//PLAA//TIA1//SNIP1//CCT6A//DCP2//SESTD1//NEUROG1//IRAK4//EREG//MEMO1//GCSAML//HECW2//GLRX//TNFRSF21//MYB//MET// |
| GO:0043066 | negative regulation of apoptotic process | Biological process | 72 | 911 | 902 | 17653 | 1.54677129248091 | 0.000146809687422952 | 0.00349603491563882 | 3.8332452860036 | 0.0798226164079823 | FOXP1//IL2//ADCYAP1//DLL1//CCL2//EDN1//IGF1//LRP6//DNAJB6//ARL6IP1//RAF1//CEBPB//CNTFR//EN1//UNC5B//ADNP//GABRA5//GRIK2//ISL1//JUN//NTF3//NR4A2//PIK3CA//POU4F1//RASA1//ITSN1//C5AR1//GDF5//SYNGAP1//EFNA1//FZD3//TNFAIP3//SIRT1//SERPINB13//BAG5//TMBIM6//CFDP1//KDR//CCL21//NEUROD1//SRSF6//CAST//NOG//ZMYND11//AR//ACVR1//TNF//BCL2A1//HIPK3//DUSP1//EDNRB//ATF5//FLNA//KLHL20//GLI3//MDM4//MET//ROR1//OGG1//ZFAND6//PRKAA1//SPHK2//PTEN//BNIP2//TFAP2A//WNT7A//WT1//BAG6//CASP2//TP63//CCND2//SOCS3// |
| GO:0032501 | multicellular organismal process | Biological process | 436 | 7496 | 902 | 17653 | 1.13833187012379 | 0.00015063068764994 | 0.00357443979149665 | 3.82208654128704 | 0.483370288248337 | WNT7A//HAPLN1//EN1//EVC//FRZB//SERP1//GNAQ//IGF1//PRELP//PTHLH//BMP3//SOX4//KLF10//TP63//NOG//RASSF2//MYCN//PPARGC1B//TAPT1//MYOG//RUNX3//S1PR1//EFNA1//ELK3//EREG//UNC5B//SIRT1//LEMD3//CLIC4//HOXA3//JUN//KDR//RHOB//MEOX2//TNFRSF12A//PIK3CA//PKNOX1//PTEN//RORA//CCL2//VEGFB//CEBPB//ADCYAP1//INHBB//IMMP2L//ESR1//CASP2//YTHDF2//CRKL//FOXF1//SPHK2//TGFBR2//RAPGEF2//EDN1//PPP3R1//ACVR1//GJC1//ZFPM2//RASA1//WT1//CCM2//TEAD2//QKI//GJA1//IGFBP3//RDH14//CBFB//RAB18//SMOC1//RDH10//GLI3//ID2//ROBO2//NPNT//SDC1//SLIT2//ADRB1//EDN2//ARRDC3//GABRA5//GRIK2//AGFG1//SLC26A7//TANC2//AR//MBNL1//BTF3//FOSL1//LRP6//RNF2//DUSP1//BMPR2//WNT3//EDNRB//ISL1//SEMA4C//EP300//DLL1//GPM6A//LRP12//NR4A2//SPOCK1//FZD3//MAF//IRAK4//TXK//RPS6KA5//TNF//SULF1//TFAP2A//TSC1//BAG6//ARID5B//RBBP8//FOXF2//FAM83D//DLG5//PRKACB//FZD6//JARID2//MET//PPARG//E2F8//STOX2//NANOS1//RB1//APCDD1//WNT10A//FBXW7//EFNB2//RECK//GLRB//NRG1//TBL1XR1//CALM2//SNX19//NFIB//CYTL1//GDF5//ESCO2//KCNAB2//DOCK10//F3//CD3E//ARID2//TNNI1//HOMER1//SGCE//ADCY3//PRKAR2B//DHRS3//POU4F1//KCNK2//SNX17//DCTN5//NEUROD1//PALLD//BNC2//TMOD3//HSBP1//SLC6A8//TMOD1//IFNA1//IRF1//IRF2//NFE2//NMUR1//CHRNB4//CHRM2//DTNA//MYBL1//TSHZ1//CFDP1//FAT3//EDARADD//DZIP1//WDR47//WDFY3//FLII//LCLAT1//TNFRSF21//CDH20//HIC1//HIVEP2//HMGB3//SHISA2//MEF2A//ROR1//PAX5//PI15//KLF3//ANO1//BTBD7//TRERF1//NXF2//RAF1//HIVEP3//BOLL//TGIF1//NXF2B//ZNF3//ZNF217//PTP4A1//SPATA9//ADAM18//ZNF516//CELF1//CRTAP//DDX6//PAIP2//ARID4B//BTG1//KATNAL1//CLOCK//TDRD5//SMARCA2//CXXC4//NEUROG1//GSC//MID1//ZEB1//SYNGAP1//E2F5//FLI1//BHLHE41//FUT9//CNTFR//RBFOX3//DPYSL2//MYT1L//FMR1//MYLIP//NTF3//POU3F2//TAOK1//PCDH10//PURA//MPPED2//ZIC5//NAV3//FEZ2//GLRA3//SLITRK3//OGN//RANBP9//ARX//OTX2//PIK3CB//ENAH//MAPK1//SCN1B//KLF7//NRXN3//PCDHB10//BSN//NHLH2//NPAS2//HPCAL4//SERPINI1//FXR1//RAB3GAP1//PHF8//PTPRG//ATXN1//SLC6A11//SYT1//ADIPOR2//ZBTB18//CFL2//GPCPD1//CCNT2//FOXP2//CRHR1//ETS1//ALPI//CLCN5//CCND1//CNGA3//BBS9//KCNJ10//ATXN7//LRIG1//TIMM9//KCNE1//SLC26A4//TUB//WDR1//C5AR1//OR6A2//ARC//SLC6A1//KCNK10//ADNP//CPEB3//LDLR//SGK1//CCND2//PPP1R1B//GSTM3//SNAP25//EPS8//HS3ST5//NPTN//RNF38//CSDE1//ATF5//MORC3//RAI2//NR5A2//RICTOR//EMX2//GRSF1//FOXP1//PDE5A//STRAP//PFN2//IFNG//PPM1F//BAMBI//FOXA1//PPP2CA//LDLRAD4//STMN2//NCK1//RAP1A//LPAR1//BAG5//WASF3//KBTBD8//KCNJ2//TTK//STRN//SPRED1//AGO1//FOXJ2//SARS//HOXD1//LRP8//PLXNA4//HPRT1//FLNA//TACC2//PRDM16//TRAK2//MSN//DGKH//TRPC3//DGKE//ACSL4//CR2//DNAJB9//TGM3//IL2//SMURF1//OSTM1//RAB21//NREP//CAMSAP1//SAMD14//PSD//KRT80//KRTAP5-6//KRTAP2-4//SOX5//TBK1//PCBP2//MAVS//TNFAIP3//POLR3H//ERRFI1//SOCS5//IGSF3//CD2AP//GFPT1//BHLHE40//MGST1//DENND1B//TIA1//INSIG2//NFIC//SBF2//CREBRF//YES1//TMEM64//CD8A//NMI//GPR55//SOCS1//SERPINB13//SRSF6//MYB//KLF13//TNRC6B//YWHAG//DNMT3B//ETV5//MMD//TOB1//TFE3//EGLN1//VASH2//ITGB8//ADAM12//PHF14//PRKAA1//ZNF521//ULK2//SLC9A6//DBN1//SLC35D1//HECW2//CAMSAP2//VTI1A//OR4N4//RAB8B//PRICKLE2//PSMA2//PSMD12//NFIA//SYBU//KPNA6//MFAP5//YIPF6//SOCS3//DNAJB6//GCNT4//ZMYND8//MAPK6//KCNIP4//KLHL3//KCNJ3//SS18//C5ORF30//NLN//PPP1R16B//PLAA// |
| GO:0070727 | cellular macromolecule localization | Biological process | 124 | 1772 | 902 | 17653 | 1.36952495833187 | 0.00016173784799944 | 0.00382459113993081 | 3.79118833968581 | 0.137472283813747 | TNPO2//IFNG//TNF//PMAIP1//NUP54//ZFAND6//AGFG1//NXF2//SRSF2//SRSF6//NXF2B//SLBP//TSC1//PAN3//TRAK2//YWHAG//KPNA6//SMURF1//ZDHHC22//ZDHHC7//ZDHHC18//ARL6IP1//VTI1A//TIMM9//SLC25A6//MTX3//IMMP2L//STX6//SEC23A//TMED1//RAB12//RAB18//RAB21//TBC1D12//SNX5//ARFIP1//RAB30//GRIK2//RHOB//RAB9B//RAB8B//KIF13A//SNX2//SNX27//AP1S2//RAB28//SNX17//GNPTG//PEX5L//TRAPPC8//DBN1//SIRT1//OGG1//IGF1//MAPK1//MAVS//MZT1//FLNA//RIC3//ATG16L1//WDR45B//TTK//DNAJB6//CNEP1R1//SEPT9//FRMD6//ARMCX3//RRAGD//MID1//TAPT1//GLI3//MDFIC//C16ORF70//VAMP3//SYNGAP1//CLIP1//SOX4//CTDSPL2//MORC3//SKP1//BBS9//TUB//C2CD5//ESCO2//ESR1//ETV5//RB1//NEDD1//BAG6//GPHN//EFR3A//KCNB1//LRP6//KCNIP4//RAPGEF2//TNFAIP3//MIEF1//KCNE1//PRR5L//RAB3GAP1//GLRB//NSG1//SNAP25//CD2AP//DYNLL2//PPP3R1//TP63//FAM83D//ABHD17C//ZMYND8//MSN//APPL1//AR//PPFIA1//ARL5A//TIA1//NPTN//NMUR1//NRG1//FBXW7//PRKAA1//SNIP1//CCT6A//KCNAB2// |
| GO:0010976 | positive regulation of neuron projection development | Biological process | 28 | 260 | 902 | 17653 | 2.10764113934846 | 0.000163040783739688 | 0.0038419680154408 | 3.78770374567202 | 0.0310421286031042 | ADNP//NRG1//TNFRSF12A//WNT3//BMPR2//ROBO2//PLXNA4//SLIT2//RAB21//LPAR1//CPEB3//FMR1//ZMYND8//IL2//MAPK6//DLG5//LRP8//SMURF1//SYT1//PLAA//STMN2//ADCYAP1//NPTN//NCK1//SERPINI1//RAP1A//SCN1B//RAPGEF2// |
| GO:0043583 | ear development | Biological process | 24 | 208 | 902 | 17653 | 2.2581869350162 | 0.000165560472776056 | 0.00388779679647384 | 3.78104334209407 | 0.0266075388026608 | LRIG1//NEUROG1//INSIG2//TFAP2A//FZD3//FZD6//MAPK1//TSHZ1//GSC//EDN1//NOG//MYCN//DLL1//ZEB1//MAF//NEUROD1//ROR1//CYTL1//CCM2//GABRA5//GLI3//KCNK2//FRZB//RDH10// |
| GO:0051050 | positive regulation of transport | Biological process | 74 | 947 | 902 | 17653 | 1.52930364764913 | 0.000168427159468215 | 0.00394142864873197 | 3.77358787568603 | 0.082039911308204 | ARL6IP1//FMR1//NTF3//EDN1//EDNRB//TRPC3//KCNB1//SCN1B//C2CD5//SIRT1//SLC6A1//ARPP19//C1QTNF2//CREBL2//IGF1//RAP1A//ADIPOR2//INHBB//SYT1//GJA1//SERP1//ISL1//SOX4//STIM2//C8ORF44-SGK3//SGK2//SGK3//SGK1//IFNG//MAPK1//MAVS//ANO1//SYBU//GLI3//DLL1//LDLR//CBLL1//CTDSPL2//RAB21//CCL21//TNF//SIRPA//TUB//IL2//CXCL9//RAB8B//HOMER1//PPARG//ADCYAP1//CALM2//RAB3GAP1//CCL2//MIEF1//PRR5L//DYNLL2//PMAIP1//PPP3R1//YWHAG//TP63//FLNA//KCNE1//KCNJ2//PLAA//SDC1//VPS4B//NMUR1//NRG1//FBXW7//PRKAA1//SNIP1//MSN//GLRX//MYB//ARC// |
| GO:0033993 | response to lipid | Biological process | 71 | 900 | 902 | 17653 | 1.54393077112589 | 0.000172363759748097 | 0.00401964174888407 | 3.76355404127175 | 0.0787139689578714 | ESR1//PPARGC1B//AR//PMEPA1//RB1//MAPK1//CCL2//TNF//CNGA3//DNMT3B//DUSP1//ETS1//FOXA1//OGG1//PTEN//RBBP8//CCND1//SLC6A1//WNT7A//EDN1//GJA1//FOXP1//TNFRSF21//JUN//MGST1//CXCL9//C5AR1//PPARG//FOSL1//UBA5//CBFB//ISL1//TP63//FOXP2//ACSL1//CCL21//RORA//NR5A2//NR3C2//NR4A2//TGFBR2//SDC1//SLIT2//EP300//SIRT1//INSIG2//CEBPB//EDNRB//CDK19//TNFAIP3//PLAA//MYB//MYOG//WNT3//YES1//TEAD2//PRKAA1//ADCYAP1//H2AFZ//POU4F1//MSN//LRP6//LRP8//LDLR//FIBIN//ERRFI1//CREBRF//ADCY3//LPAR1//CLOCK//VPS4B// |
| GO:0048568 | embryonic organ development | Biological process | 40 | 428 | 902 | 17653 | 1.82906106886048 | 0.000173732745037564 | 0.00403764451783177 | 3.76011831828947 | 0.0443458980044346 | CEBPB//GJA1//DLL1//TGFBR2//TEAD2//ACVR1//TFAP2A//EFNA1//RDH10//LRIG1//KDR//NEUROG1//INSIG2//FZD3//FZD6//MAPK1//TSHZ1//GSC//EDN1//NOG//FOXF1//GLI3//ID2//NEUROD1//FOXF2//TNF//LRP6//ZEB1//PAX5//HOXA3//MYCN//SOCS3//E2F8//DNAJB6//EGLN1//VASH2//FRZB//EN1//ARID2//ZFPM2// |
| GO:0030323 | respiratory tube development | Biological process | 21 | 171 | 902 | 17653 | 2.40345042206403 | 0.000175002472015935 | 0.0040397013297131 | 3.75695581659772 | 0.0232815964523282 | EP300//FOXF1//ZFPM2//GLI3//MYCN//BAG6//EDN2//ERRFI1//BMPR2//FOXP2//PHF14//MAPK1//NOG//RDH10//TGFBR2//FOXA1//DLG5//NFIB//PPP3R1//SRSF6//TNF// |
| GO:0050678 | regulation of epithelial cell proliferation | Biological process | 35 | 357 | 902 | 17653 | 1.91872092517717 | 0.00017501589377583 | 0.0040397013297131 | 3.75692250979538 | 0.0388026607538803 | SULF1//GJA1//TNF//F3//SIRT1//RICTOR//PPP1R16B//JUN//KDR//BMPR2//VEGFB//VASH2//SRSF6//TP63//EFNB2//CCND1//IGF1//C5AR1//NOG//STRAP//EREG//AR//ISL1//PTEN//RB1//GDF5//RUNX3//WNT7A//FOXP2//ERRFI1//PPARG//FBXW7//TNFAIP3//NFIB//EDNRB// |
| GO:0007411 | axon guidance | Biological process | 27 | 248 | 902 | 17653 | 2.13070864029755 | 0.000177160250172886 | 0.00407528833986132 | 3.7516337151076 | 0.0299334811529933 | NOG//SLIT2//ROBO2//PLXNA4//SCN1B//ISL1//UNC5B//FZD3//BMPR2//SEMA4C//WNT3//NFIB//RANBP9//ARX//DPYSL2//EFNA1//EFNB2//GLI3//OTX2//PIK3CA//PIK3CB//ENAH//MAPK1//KLF7//RPS6KA5//NRXN3//FEZ2// |
| GO:0060999 | positive regulation of dendritic spine development | Biological process | 9 | 41 | 902 | 17653 | 4.29606294954302 | 0.000182131694774251 | 0.00416394638909302 | 3.73961447118584 | 0.00997782705099778 | LRP8//LPAR1//CPEB3//FMR1//ZMYND8//NRG1//IL2//MAPK6//DLG5// |
| GO:0044271 | cellular nitrogen compound biosynthetic process | Biological process | 322 | 5342 | 902 | 17653 | 1.17967933482813 | 0.000182245768323456 | 0.00416394638909302 | 3.7393425470435 | 0.356984478935698 | NPAT//TSHZ1//ZBTB18//CEBPB//TCERG1//STRAP//KLF12//GSC//ARX//DNMT3B//EDN1//EDNRB//EFNA1//CC2D1B//EN1//EP300//ESR1//JAZF1//CPEB3//FOXF1//MYT1L//SIN3B//SIRT1//ZFPM2//DNAJB5//GLI3//CNOT7//HIC1//FOXA1//HSBP1//ID2//IFNG//IRF2//AR//ISL1//JARID2//JUN//MAF//MDM4//MEF2A//MYB//NFIA//NFIB//NFIC//NR4A2//PAX5//KLF3//POU4F1//PPARG//ZBTB4//RB1//RBBP8//CCND1//RNF2//SARS//PRDM16//SMARCA2//SUV39H1//ZEB1//TFAP2A//TGIF1//KLF10//TNF//UBE2I//WT1//ZNF217//LRP8//BHLHE41//TBL1XR1//E2F8//ARID5B//BHLHE40//TP63//RUNX3//NOG//FOXP2//PHF14//GTF2E1//SEPSECS//SECISBP2L//ST3GAL5//GTF2H1//CREBRF//ELK3//ETV5//ATF5//FOXF2//FLI1//GTF2A1//HIVEP2//IRF1//MEOX2//MYBL1//MYCN//NEUROD1//NHLH2//OTX2//KLF13//PKNOX1//POU3F2//FOXJ2//HIVEP3//BMPR2//SOX4//SOX5//BTF3//TFE3//TXK//FOSL1//KLF7//CBFB//FUBP3//CCNT2//NMI//CLOCK//ZNF516//POLR3H//SRSF2//SRSF6//SLBP//GFPT1//SLC35D1//NUP54//HPRT1//ADSS//ADCY3//IMPDH1//CMPK1//BAHD1//H2AFZ//ZMYND11//PNRC1//ZNF526//CREBL2//ZNF800//E2F5//ZNF367//ARID2//MED19//ZFP30//KDM2A//MYCBP2//PHF8//FLII//ADNP//ZMYND8//CNOT6L//ZNF521//EPC2//AGO1//HBP1//FOXP1//ZBTB11//ATAD2//MDFIC//HMGB3//HOXA3//HOXD1//ZNF680//ZNF662//NEUROG1//NFE2//NPAS2//ASCC1//IER5//COMMD10//ARID4B//BRWD1//BNC2//MED9//ZNF532//PRKAA1//TRERF1//MAPK1//SLC2A4RG//CCNL1//PHTF2//CNOT6//ZBTB26//MIER1//PURA//ATXN1//ATXN7//SMARCD2//SS18//MED22//ZNF3//ZNF708//ZNF131//ZNF227//ZNF655//ZSCAN5A//SAP30L//ANP32A//PPP1R1B//PCGF5//LCOR//ST18//HIPK3//PPARGC1B//ELAVL2//ELL2//NR5A2//NRBF2//AFF1//MLLT6//OGG1//PHF20L1//PPP2CA//PAK6//RORA//BTG1//UBE2V1//CSDE1//SNIP1//TEAD2//RPS6KA5//EMX2//ETS1//BOLA3//NR3C2//RFX7//TRAK2//BDP1//NRBP1//CPEB2//CPEB1//DDX3Y//GTPBP1//DDX6//TSC1//KBTBD8//PTGES3//GRM3//G3BP2//PSAT1//EREG//CRHR1//FMR1//SERP1//PRR16//FXR1//FLNA//BAMBI//IGF1//LRP6//WNT7A//ACVR1//DNAJB6//NRG1//RASD1//BTAF1//PPM1F//TOB1//CELF1//NANOS1//TIA1//MEX3B//SMURF1//BMP3//GDF5//SULF1//LEMD3//CTDSPL2//ADRB1//PTHLH//CALM2//EGLN1//RWDD3//TNFAIP3//DCP2//CCT6A//INSIG2//TNRC6B//NCK1//MYOG//C5AR1//INTS8//NABP1//FZD6//SMUG1//ADCYAP1//S1PR1//KPNA6//DLL1//TBK1//IL2//MET//PPP1R12A//ARMCX3//CYTL1//PPP3R1//MAVS//RAF1//YES1//PAIP2//BOLL//PDE5A//SPHK2//SPTSSB//SPTSSA//C8ORF44-SGK3//SGK3//SGK1//PTEN//RHEBL1//ROR1//IRAK4//YTHDF2//QKI//EGLN3//PSMA2//PSMD12//RAP2C//HSBP1L1//PPP1R15B//TMBIM6//DUSP1//GJA1//KCNK2//FBXW7//NPNT// |
| GO:0016477 | cell migration | Biological process | 106 | 1474 | 902 | 17653 | 1.40740913453294 | 0.000185231024866381 | 0.00421790377498766 | 3.73228627035487 | 0.117516629711752 | EDNRB//ISL1//SEMA4C//ACVR1//GJA1//GPM6A//LRP12//NR4A2//SPOCK1//FZD3//RAPGEF2//EFNB2//KDR//SLIT2//CCL21//CCL2//CCL8//EDN2//CXCL9//CD2AP//NCK1//TNFRSF12A//EDN1//ETS1//FOXP1//RHOB//BMPR2//WNT7A//NANOS1//STRAP//PFN2//PTPRG//IFNG//JUN//TGFBR2//PPM1F//C5AR1//TMEM201//TNS1//ARID5B//PRR5L//C5ORF30//IGF1//IGFBP3//POU4F1//POU3F2//ARX//GLI3//NRG1//EMX2//FLNA//C8ORF44-SGK3//SGK3//RND3//DOCK10//SGK1//PLXNA4//SLK//S1PR1//F3//FOXF1//FAM89B//NTF3//MAPK1//PTP4A1//CBLL1//ARID2//SULF1//ZMYND8//TMEFF2//CLIC4//PTEN//RAP2C//LDLRAD4//RECK//NAV3//DLG5//NOG//EPS8//EFNA1//SIRT1//PPARG//PIK3CA//APCDD1//SIRPA//MSN//PIK3CB//SDC1//YES1//TNF//LPAR1//VEGFB//SPRED1//MEOX2//IRAK4//WDR1//GCSAML//MET//PLAA//RASGEF1A//PALLD//ARC//BAMBI//PAK6//BTG1//FAM83D// |
| GO:2000027 | regulation of organ morphogenesis | Biological process | 27 | 249 | 902 | 17653 | 2.12215157748511 | 0.000189291659149047 | 0.00429590433162753 | 3.72286852214476 | 0.0299334811529933 | WT1//BMPR2//ISL1//EDN1//WNT10A//PRICKLE2//ROR1//PSMA2//SMURF1//PSMD12//FZD3//FZD6//SRSF6//FOXP2//AR//SULF1//ESR1//BTBD7//TNF//ETV5//TFAP2A//NOG//TGFBR2//ACVR1//FBXW7//TNFAIP3//NFIB// |
| GO:0050773 | regulation of dendrite development | Biological process | 18 | 136 | 902 | 17653 | 2.59027324898917 | 0.000195378482085544 | 0.00441921295767403 | 3.70912326879586 | 0.0199556541019956 | FMR1//HECW2//FXR1//RAPGEF2//RAB21//CPEB3//LPAR1//ZMYND8//NRG1//IL2//MAPK6//DLG5//ARC//EFNA1//PTEN//LRP8//DBN1//CAMSAP2// |
| GO:0021559 | trigeminal nerve development | Biological process | 5 | 12 | 902 | 17653 | 8.15456393200296 | 0.000201843540971298 | 0.00454214010671357 | 3.69498514353088 | 0.00554323725055432 | PLXNA4//ISL1//NEUROG1//POU4F1//TFAP2A// |
| GO:0097485 | neuron projection guidance | Biological process | 27 | 250 | 902 | 17653 | 2.11366297117517 | 0.000202156464900308 | 0.00454214010671357 | 3.69431236550698 | 0.0299334811529933 | RANBP9//ARX//DPYSL2//EFNA1//EFNB2//GLI3//OTX2//PIK3CA//PIK3CB//ENAH//MAPK1//ROBO2//SCN1B//WNT3//KLF7//RPS6KA5//SLIT2//NRXN3//FEZ2//NOG//PLXNA4//ISL1//UNC5B//FZD3//BMPR2//SEMA4C//NFIB// |
| GO:0001837 | epithelial to mesenchymal transition | Biological process | 18 | 137 | 902 | 17653 | 2.57136614498195 | 0.000214419463938439 | 0.0048017179954161 | 3.66873579408475 | 0.0199556541019956 | EFNA1//BAMBI//ISL1//TGFBR2//STRAP//FOXA1//PPP2CA//PTEN//LDLRAD4//LRP6//KBTBD8//GSC//NOG//ACVR1//FOXF2//PPP3R1//FAM83D//DLG5// |
| GO:0035306 | positive regulation of dephosphorylation | Biological process | 10 | 51 | 902 | 17653 | 3.83744185035433 | 0.00021805056219308 | 0.00486691733370231 | 3.66144278926096 | 0.0110864745011086 | PPARGC1B//NPNT//MTMR9//CALM2//PPP1R15B//SPPL3//CNEP1R1//PPP1R16B//PPP1R12A//IFNG// |
| GO:0043069 | negative regulation of programmed cell death | Biological process | 72 | 924 | 902 | 17653 | 1.52500935871224 | 0.000224105413872578 | 0.00498560826980344 | 3.64954765178004 | 0.0798226164079823 | FOXP1//IL2//HIPK3//DUSP1//EDNRB//ATF5//FLNA//ARL6IP1//SIRT1//KLHL20//GLI3//IGF1//KDR//MDM4//MET//ROR1//OGG1//ZFAND6//PRKAA1//SPHK2//PTEN//RAF1//RASA1//BCL2A1//BNIP2//TMBIM6//TFAP2A//WNT7A//WT1//BAG6//CASP2//TP63//CCND2//SOCS3//ADCYAP1//DLL1//CCL2//EDN1//LRP6//DNAJB6//CEBPB//CNTFR//EN1//UNC5B//ADNP//GABRA5//GRIK2//ISL1//JUN//NTF3//NR4A2//PIK3CA//POU4F1//ITSN1//C5AR1//GDF5//SYNGAP1//EFNA1//FZD3//TNFAIP3//SERPINB13//BAG5//CFDP1//CCL21//NEUROD1//SRSF6//CAST//NOG//ZMYND11//AR//ACVR1//TNF// |
| GO:0033158 | regulation of protein import into nucleus, translocation | Biological process | 6 | 19 | 902 | 17653 | 6.1803010853075 | 0.000267399022979911 | 0.00592924456528898 | 3.57284018389232 | 0.00665188470066519 | IFNG//IGF1//MAPK1//MAVS//SIRT1//OGG1// |
| GO:0048645 | animal organ formation | Biological process | 11 | 62 | 902 | 17653 | 3.47226593233674 | 0.000270414472439106 | 0.00596091852764766 | 3.56797006889813 | 0.0121951219512195 | ISL1//WT1//RDH10//MAPK1//TGFBR2//TP63//NOG//AR//GLI3//SULF1//HOXA3// |
| GO:0048863 | stem cell differentiation | Biological process | 28 | 268 | 902 | 17653 | 2.04472647847238 | 0.000270590268813815 | 0.00596091852764766 | 3.56768782590711 | 0.0310421286031042 | EDNRB//ISL1//SEMA4C//ACVR1//RDH10//EDN1//TAPT1//NRG1//KBTBD8//FRZB//LRP6//WNT10A//GSC//WNT3//WNT7A//TP63//MAPK1//MYB//YTHDF2//PSMA2//PSMD12//CBFB//FOXA1//TEAD2//SOX5//ESR1//GPM6A//JARID2// |
| GO:0007169 | transmembrane receptor protein tyrosine kinase signaling pathway | Biological process | 58 | 711 | 902 | 17653 | 1.59650534364952 | 0.000291102003814943 | 0.00639195731104045 | 3.5359548050635 | 0.0643015521064302 | EREG//FBXW7//ERRFI1//GPRC5A//SOCS5//NRG1//EPS8//EPS15//PIK3CA//RPS6KA5//APPL1//SPRED1//MAPK1//TIA1//SHCBP1//F3//SLC9A6//RAPGEF2//C2CD5//SULF1//KDR//CUL5//RASSF2//OTX2//SHISA2//IGFBP3//IGF1//AR//ATXN7//NPTN//NCK1//TSC1//SOCS1//SOCS3//SIRT1//PTEN//ARID5B//GIGYF1//PIK3CB//BMPR2//VEGFB//RAF1//MET//EFNA1//EFNB2//RASA1//ITSN1//SS18//YES1//NOG//DLL1//PHF14//NTF3//ROR1//PTPRG//TXK//CD3E//CD8A// |
| GO:0045665 | negative regulation of neuron differentiation | Biological process | 23 | 203 | 902 | 17653 | 2.21739866525401 | 0.000293290458449194 | 0.00641916948379255 | 3.53270206559179 | 0.0254988913525499 | STMN2//LPAR1//EFNB2//MYLIP//PTPRG//SPOCK1//TSC1//BAG5//EDNRB//DLL1//ULK2//PTEN//SEMA4C//WNT3//TRAK2//SYNGAP1//RAPGEF2//EFNA1//SLIT2//DDX6//GLI3//ID2//ISL1// |
| GO:0019538 | protein metabolic process | Biological process | 359 | 6078 | 902 | 17653 | 1.15596779924543 | 0.000317601397088283 | 0.00692883305970341 | 3.49811759583437 | 0.3980044345898 | GADD45A//GTF2H1//PTEN//CCNT2//DIRAS3//RANBP9//SPRED1//EREG//RASGEF1A//NRG1//IL2//MEF2A//MET//MAPK1//MAPK6//PSMA2//PSMD12//RAF1//RASA1//CCL2//TNF//SYNGAP1//RAPGEF2//CRKL//TAOK1//RAP1A//C1QTNF2//LPAR1//IGF1//NTF3//PIK3CB//PRKAA1//C5AR1//UBE2V1//DUSP1//PPP2CA//FBXO41//RNF145//ZNRF2//RNF167//MYLIP//ANKIB1//UBE2W//FBXW7//SMURF1//HECW2//KLHL42//SKP1//THOP1//LONRF3//FBXO30//CBFB//UBE3B//LONRF1//FBXO44//COPS8//NKTR//SEPSECS//SECISBP2L//SOCS1//IGFBP3//LRP6//PRR5L//PPP1R15B//SLIT2//EDNRB//EFNA1//FMR1//SIRT1//NPTN//IFNG//KDR//MAVS//CCND1//CCND2//ABHD17C//F3//ERRFI1//GPRC5A//SOCS5//ZER1//CACUL1//RNF38//MARCH10//PDZRN3//MYCBP2//FBXO33//KLHL3//RNF11//KLHL20//SPOPL//TRIM23//MDM4//DCAF8//CBLL1//CUL5//KBTBD8//SOCS3//CPEB2//CPEB3//SARS//CPEB1//DDX3Y//GTPBP1//DDX6//TSC1//PPP4R2//SERP1//RIMKLB//TGM3//UBE2I//YES1//SPPL3//IMMP2L//C8ORF44-SGK3//SGK2//HIPK3//FASTK//PDIK1L//CDK19//MORC3//MMD//SGK3//PAN3//GRK6//TBK1//NRBP1//CDK17//PIK3CA//PRKACB//CCL8//SGK1//CDK15//TGFBR2//TXK//MEX3B//RUNX3//ACVR1//RPS6KA5//RASSF2//GNAQ//NCK1//GSKIP//RB1//YWHAG//PPM1F//PTPN21//PPTC7//PPP1R12A//CTDSPL2//PPP2CB//PPP3R1//EP300//CLOCK//HS3ST5//PCMTD1//BTG1//FUT9//B3GALNT2//FUT2//FUT5//ST8SIA5//GFPT1//KCNE1//CCDC126//ALG2//EOGT//GCNT4//TRAK2//VEGFB//WDR45B//ALPI//RECK//PYURF//PIGP//PIGA//AMZ1//ADAMTS17//PRSS55//MEP1A//KLK15//NRIP3//NLN//LRP8//NPEPL1//ADAM12//CTSF//ADAM18//LMLN//CPD//GLI3//CASP2//FBXO8//USP27X//USP28//BAG6//USP38//GPHN//EGLN3//PMAIP1//PPARG//CR2//DLL1//IRAK4//MDFIC//PI15//CCL21//CALM2//ADNP//INHBB//BMP3//BMPR2//TTK//GDF5//SERPINI1//SERPINB13//SPOCK1//PRR16//SOX4//FXR1//GNPTG//EPC2//SIN3B//ARID4B//MIER1//SAP30L//TBL1XR1//ARID5B//ATXN7//MBD6//ESR1//OTUD3//AR//TNFAIP3//WDR20//NUP54//TOB1//CELF1//CNOT7//NANOS1//PURA//TIA1//CEP41//RICTOR//STK33//ROR1//ZDHHC22//ZDHHC7//ZDHHC18//ST3GAL5//MCFD2//CRTAP//EGLN1//DCUN1D4//KCNH4//PAK6//SLK//SULF1//DSEL//SLC35D1//DNAJB9//JKAMP//JARID2//ANAPC15//BAG5//NDFIP2//MOB1B//JUN//RAP2C//EDN1//WNT7A//ARPP19//PPP1R1B//MID1//SEMA4C//CREBL2//PFN2//IFNA1//PHF8//RWDD3//ADCY3//PRKAR2B//ESCO2//PRDM16//ISL1//CNOT6//TNRC6B//AGO1//PPP1R16B//CNEP1R1//PTPRG//PTP4A1//PTP4A2//CDC14A//RNF2//SUV39H1//PCGF5//LDLR//FAM83D//FLNA//NSG1//DNAJB6//ARL6IP1//UBXN2B//CD2AP//ARMC8//PCBP2//PLAA//VPS37A//VPS4B//TP63//PDE5A//RCAN3//PPP2R5E//CKAP4//CALU//RAB12//IRF1//CD3E//NMI//CEBPB//GJA1//CCNY//CCNL1//ADCYAP1//PAIP2//BOLL//GRIK2//ZMYND11//ULK2//CYTL1//SH3D19//CCM2//ARRDC3//DNMT3B//MYB//PAX5//STRAP//PMEPA1//LDLRAD4//NOG//CNOT6L//YTHDF2//QKI//ATG16L1//NPNT//GPR55//OTUD1//KDM2A//UBA5//ACSL1//GLRX//CAST//RAB3GAP1//MSN//ZMYND8//MYCN//ST18// |
| GO:0051270 | regulation of cellular component movement | Biological process | 75 | 985 | 902 | 17653 | 1.49017411955384 | 0.000333126869981154 | 0.00724417048772522 | 3.47739033574502 | 0.0831485587583149 | CCL8//SLIT2//EDN2//CXCL9//EDN1//ETS1//FOXP1//KDR//RHOB//BMPR2//WNT7A//STRAP//PFN2//PTPRG//IFNG//JUN//TGFBR2//PPM1F//C5AR1//PRR5L//C5ORF30//IGF1//IGFBP3//NRG1//C8ORF44-SGK3//FLNA//SGK3//RND3//NCK1//DOCK10//SGK1//PLXNA4//SLK//S1PR1//F3//FOXF1//FAM89B//NTF3//SEMA4C//MAPK1//PTP4A1//CBLL1//ACVR1//ARID2//SULF1//ZMYND8//TMEFF2//CLIC4//PTEN//RAP2C//LDLRAD4//RECK//NAV3//DLG5//NOG//EFNA1//SIRT1//PPARG//WNT3//VEGFB//LPAR1//TNF//KCNJ2//CCL21//SPRED1//MEOX2//EREG//MEMO1//RAF1//GCSAML//MSN//CCL2//MET//PLAA//RAPGEF2// |
| GO:1902904 | negative regulation of supramolecular fiber organization | Biological process | 17 | 130 | 902 | 17653 | 2.5592785263517 | 0.000336326242515256 | 0.00727809361491201 | 3.47323924468382 | 0.0188470066518847 | EPS8//STMN2//CAMSAP1//CAMSAP2//MID1//MID1IP1//NAV3//PFN2//SLIT2//S1PR1//TMEFF2//ARHGAP6//MET//PPFIA1//TMOD3//TMOD1//LDLR// |
| GO:0010243 | response to organonitrogen compound | Biological process | 72 | 937 | 902 | 17653 | 1.5038512779617 | 0.00033683916922482 | 0.00727809361491201 | 3.47257741245415 | 0.0798226164079823 | HPRT1//NR4A2//PPP1R1B//APPL1//HOMER1//ADSS//SLC6A1//CEBPB//CPEB3//SESN1//RRAGD//CPEB1//ZEB1//TNF//SOCS1//DNAJB9//JKAMP//BAG6//DNMT3B//PPARG//PRKAA1//C5AR1//TNFAIP3//SIRT1//IGF1//INSIG2//SRSF6//TSC1//CPEB2//INHBB//RAB8B//ERRFI1//PTEN//YWHAG//C2CD5//TRPC3//PIK3CA//EDN1//GLRB//GLRA3//EREG//GJA1//LRP6//BTG1//NCK1//SOCS3//CNGA3//PPARGC1B//DUSP1//JUN//SDC1//FOSL1//OGG1//MAPK1//KCNE1//RAP1A//RAP1B//WT1//RAPGEF2//CRHR1//ADCY3//PRKACB//PRKAR2B//LDLR//SOCS5//SLIT2//KLF3//KLF10//TMBIM6//EDNRB//MGST1//CCND1// |
| GO:0050896 | response to stimulus | Biological process | 516 | 9123 | 902 | 17653 | 1.1069398195103 | 0.000359647147472217 | 0.00773910318579272 | 3.44412337993983 | 0.572062084257206 | SIRT1//USP28//CLOCK//KCNH4//RANBP9//SPRED1//EREG//RASGEF1A//NRG1//IL2//MEF2A//MET//MAPK1//MAPK6//PSMA2//PSMD12//RAF1//RASA1//CCL2//TNF//SYNGAP1//RAPGEF2//GADD45A//CRKL//TAOK1//RAP1A//C1QTNF2//LPAR1//IGF1//NTF3//PIK3CB//PRKAA1//C5AR1//UBE2V1//DUSP1//PPP2CA//COPS8//GTF2H1//RBBP8//NABP1//ADCYAP1//GABRA5//GRIK2//EGLN3//DNMT3B//EP300//ETS1//MYB//NR4A2//EGLN1//TGFBR2//VEGFB//JUN//PMAIP1//BCL2A1//RAET1E//CBFB//GLRB//ID2//HPRT1//PPP1R1B//TBL1XR1//ADRB1//ARL6IP1//MAVS//PRKACB//SKP1//RPS6KA5//IRAK4//HIST2H2BE//TNFAIP3//TNFRSF21//IFNA1//IFNG//PIK3CA//TFE3//TSC1//TXK//CD3E//DLL1//DOCK10//CCL21//BAG6//CR2//CD8A//OGG1//ACVR1//F3//GJA1//CCL8//PPARG//SLIT2//EDN2//CXCL9//SKAP2//IRF1//S1PR1//SPHK2//DNAJB5//JKAMP//HSPA13//TMBIM6//INHBB//CEBPB//CHRM2//TBK1//FOSL1//ZFPM2//GNAQ//IRF2//NFE2//PRKAR2B//APCDD1//CCNY//HBP1//GRK6//HIC1//LRP6//STRN//WNT3//WNT7A//CXXC4//WNT10A//TNRC6B//AGO1//PPP3R1//FZD3//FZD6//CNIH1//CDS1//PTGES3//IQGAP2//ADCY3//RALBP1//TMED1//RASSF8//CHRNB4//ARAP2//CLCN6//CNGA3//CNTFR//CREBL2//DPYSL2//DTNA//ELK3//EPS8//ESR1//CD2AP//APPL1//ATP2C1//CNOT7//LRP12//HIVEP2//AR//NR3C2//PPP1R12A//HPCAL4//GULP1//RASD1//KCNK10//CYTL1//NDFIP2//PPP2R5E//PSD//ARRDC3//MIER1//HIVEP3//SMOC1//SMOC2//SPOCK1//ZNF217//ZYX//LRP8//GLRA3//SNX27//SYDE2//PPFIA1//OR6A2//PDE5A//GPRC5A//DLG5//PLAA//ARHGAP12//GTPBP1//FEZ2//ZNF516//CD69//ULK2//SNX17//KDR//ROR1//PTPRG//YES1//C8ORF44-SGK3//SGK2//AKAP11//DGKH//SGK3//NRBP1//ARHGEF4//SGK1//STK33//SS18//ANP32A//DGKE//RALGPS1//PALM2//BMPR2//FBXW7//ERRFI1//SOCS5//SYT1//CALM2//EPC2//ASTE1//UBE2W//SMUG1//ESCO2//KDM2A//ASCC1//MGST1//RORA//PLP2//KCNK2//GDF5//PXK//SDC1//CHST1//NMI//CRHR1//ZEB1//PAX5//YTHDF2//CACUL1//FMR1//NPAS2//ZBTB4//CCND1//ZMAT3//SUV39H1//CUL5//IMMP2L//CNOT6L//MDM4//CNOT6//SOX4//E2F8//CASP2//TP63//PON2//BTG1//SLC23A2//PPP1R15B//GABARAPL1//EDN1//EDNRB//IGSF3//TSPAN11//NCK1//EPS15//SMURF1//KLF10//NMUR1//RAPGEF4//TAPT1//FRZB//OR4N4//GRM3//RHOB//RGS7BP//PTHLH//ITSN1//SOS2//GPR55//FLNA//ANO1//HOMER1//NSG1//KCNB1//NXPH3//SORCS1//SORCS2//SUSD5//FOXA1//TMEM17//EVC//DZIP1//FOXF1//GLI3//ITGB8//CCM2//RCAN3//SAMD14//SNIP1//MDFIC//SOCS1//SOCS3//NEUROD1//RAB18//RGL1//RND3//ARHGAP1//RIN2//DIRAS3//RB1//G3BP2//ARHGAP6//PAK6//ARX//EFNA1//EFNB2//OTX2//ENAH//ROBO2//SCN1B//KLF7//NRXN3//ACSL1//ACSL4//ADIPOR2//TRPC3//NOG//GIT2//NPTN//TIA1//SHCBP1//GSKIP//RRAGD//PLEKHF2//EMX2//SLC6A11//TGIF1//SLC6A1//DNAJA2//SERPINB13//GPHN//SERINC3//MYOG//ANXA7//STEAP2//TUB//ADNP//RAP1B//PTEN//NR5A2//RWDD3//YWHAG//TOB1//LASP1//SH3BGRL//IGFBP3//PRR5L//FIBIN//SLC30A6//PPP2CB//TXLNA//OGN//BMP3//PPP4R2//JARID2//TTK//PMEPA1//LDLRAD4//PPP1R16B//UNC5B//ADSS//TNNI1//SLC25A6//SLC6A8//NREP//PLXNA4//CPEB3//SESN1//CPEB1//SPPL3//DENND1B//SLK//GSC//KCNJ2//SULF1//SHISA2//TSN//DNAJB9//NPNT//STRAP//LEMD3//FAM89B//BAMBI//PRDM16//CTDSPL2//PPARGC1B//CREBRF//SERP1//ISL1//NEUROG1//SLC9A6//RHEBL1//RICTOR//FAM83D//IQSEC2//FBXO8//AGFG1//RAB12//RAB21//RAB30//RAB9B//RAB8B//RAB28//RAP2C//FOXP1//INSIG2//SRSF6//CPEB2//C2CD5//MID1//SEMA4C//UBA5//SRSF2//EDARADD//TNFRSF12A//ST18//FOXP2//VPS4B//ETV5//IER5//NRBF2//PLEKHG4B//TEAD2//MOB1B//KLHL20//PPM1F//GSTA2//MSN//SYBU//GFPT1//ASNA1//SEC61A2//BRWD1//RASSF2//GSTM3//PHLDA3//ZFAND6//UBE2I//ZMYND11//BHLHE40//CKAP4//SIRPA//BRI3//ARMC8//IMPDH1//C6ORF120//NEU1//KCMF1//SNAP25//KCNAB2//HIPK3//ATXN7//PEX5L//TMEFF2//TMEM64//POLR3H//HMGB3//TRIM23//PCBP2//DLK2//ARID5B//GIGYF1//DHRS3//ELMOD2//CDK19//LDLR//NPDC1//GCSAML//ALG2//KCNJ3//IFI44L//OTUD3//KCNJ10//UGT2B28//PRICKLE2//RAB3GAP1//HSBP1//HSBP1L1//CLIC4//TFAP2A//SLC41A1//POU3F2//KCNE1//WT1//POU4F1//VAMP3//H2AFZ//CCND2//RUNX3//SOX5//NFIB//FASTK//SIVA1//ATG16L1//ZMYND8//BSN//LYPD6//C5ORF30//DNAJB6//NUP54//BAG5//DYNLL2//KLF3//MYCN//FAM168A//STMN2//DYNC1LI2//PIGA//PHF14//ARC//BBS9// |
| GO:0060389 | pathway-restricted SMAD protein phosphorylation | Biological process | 11 | 64 | 902 | 17653 | 3.36375762195122 | 0.00036046392185786 | 0.00773910318579272 | 3.4431381964706 | 0.0121951219512195 | INHBB//BMP3//BMPR2//TTK//GDF5//ACVR1//STRAP//PMEPA1//LDLRAD4//NOG//TGFBR2// |
| GO:0048589 | developmental growth | Biological process | 51 | 609 | 902 | 17653 | 1.63894683953557 | 0.000364577243947452 | 0.0077748787316508 | 3.43821044247335 | 0.0565410199556541 | ESR1//EREG//S1PR1//EVC//BNC2//TGFBR2//IGF1//WNT7A//DPYSL2//RAB21//EN1//ATF5//CLIC4//AR//TBL1XR1//CCM2//ARID5B//DLL1//PIK3CA//GDF5//ADRB1//POU3F2//TXK//GJA1//MYOG//ADNP//NRG1//TNFRSF12A//PTEN//ARX//SERP1//ULK2//SLC9A6//WNT3//PLXNA4//BMPR2//SEMA4C//SLIT2//ARID2//JARID2//KCNK2//NOG//ZFPM2//WT1//RDH10//EDN1//LRP6//SMURF1//SYT1//PLAA//GLI3// |
| GO:0033962 | cytoplasmic mRNA processing body assembly | Biological process | 6 | 20 | 902 | 17653 | 5.87128603104213 | 0.000365579097540286 | 0.0077748787316508 | 3.43701864357941 | 0.00665188470066519 | CNOT6L//PAN3//CNOT6//DDX6//PATL1//CNOT7// |
| GO:0060044 | negative regulation of cardiac muscle cell proliferation | Biological process | 6 | 20 | 902 | 17653 | 5.87128603104213 | 0.000365579097540286 | 0.0077748787316508 | 3.43701864357941 | 0.00665188470066519 | GJA1//JARID2//KCNK2//PTEN//TGFBR2//NOG// |
| GO:0010464 | regulation of mesenchymal cell proliferation | Biological process | 8 | 36 | 902 | 17653 | 4.34910076373491 | 0.00037955236211621 | 0.00804674804072705 | 3.42072830143005 | 0.00886917960088692 | FOXF1//MYCN//TGFBR2//TP63//FOXP2//NFIB//PHF14//ZEB1// |
| GO:0048562 | embryonic organ morphogenesis | Biological process | 29 | 288 | 902 | 17653 | 1.97068628356738 | 0.00039400461511321 | 0.00832704128753325 | 3.40449869110219 | 0.0321507760532151 | GJA1//DLL1//TGFBR2//TEAD2//ACVR1//TFAP2A//EFNA1//LRIG1//NEUROG1//INSIG2//FZD3//FZD6//MAPK1//TSHZ1//GSC//EDN1//NOG//FOXF1//GLI3//ID2//FOXF2//LRP6//ZEB1//PAX5//RDH10//HOXA3//MYCN//FRZB//NEUROD1// |
| GO:0008284 | positive regulation of cell proliferation | Biological process | 70 | 911 | 902 | 17653 | 1.50380542324533 | 0.000407036592376344 | 0.00857566502878883 | 3.39036654617338 | 0.0776053215077605 | F3//SIRT1//RICTOR//PPP1R16B//JUN//KDR//BMPR2//VEGFB//VASH2//GLI3//FZD3//FOXF1//MYCN//TGFBR2//TP63//FOXP2//IL2//CCND1//NCK1//CD3E//IGF1//EREG//ESR1//S1PR1//EDN1//FOXP1//ID2//MYB//IRAK4//TNF//C5AR1//NOG//ZFPM2//WNT7A//ETV5//SRSF6//AR//FOXJ2//FLNA//TNFAIP3//DNAJA2//ADCYAP1//CNTFR//CRKL//CACUL1//EDN2//EDNRB//EFNB2//ETS1//CNOT6L//BAMBI//DLL1//CNOT7//NRG1//HOXA3//IFNG//ISL1//NTF3//POU3F2//PRKAA1//MAPK1//SPHK2//PTEN//PTHLH//CNOT6//PURA//SOX4//TTK//FOSL1//CCND2// |
| GO:0048738 | cardiac muscle tissue development | Biological process | 24 | 221 | 902 | 17653 | 2.12535240942701 | 0.000411629113856808 | 0.00862439845608363 | 3.3854939158819 | 0.0266075388026608 | NRG1//ID2//ZFPM2//POU4F1//NOG//S1PR1//MEF2A//TSC1//EGLN1//ISL1//TNNI1//ARID2//GJA1//JARID2//KCNK2//PTEN//TGFBR2//WT1//ACVR1//EDN1//IGF1//DLL1//EFNB2//GJC1// |
| GO:0006928 | movement of cell or subcellular component | Biological process | 140 | 2090 | 902 | 17653 | 1.31097295748947 | 0.000412772079288566 | 0.00862439845608363 | 3.38428968691622 | 0.155210643015521 | EDNRB//ISL1//SEMA4C//ACVR1//GJA1//GPM6A//LRP12//NR4A2//SPOCK1//FZD3//RAPGEF2//EFNB2//KDR//SLIT2//CCL21//CCL2//CCL8//EDN2//CXCL9//FRMD6//CD2AP//NCK1//TNFRSF12A//KIF1C//DYNC1LI2//KIF13A//RANBP9//ARX//DPYSL2//EFNA1//GLI3//OTX2//PIK3CA//PIK3CB//ENAH//MAPK1//ROBO2//SCN1B//WNT3//KLF7//RPS6KA5//NRXN3//FEZ2//NOG//RAB21//FMR1//TRAK2//FXR1//EDN1//ETS1//FOXP1//RHOB//BMPR2//WNT7A//NANOS1//STRAP//PFN2//PTPRG//IFNG//JUN//TGFBR2//PPM1F//C5AR1//TMEM201//TNS1//ARID5B//PRR5L//C5ORF30//DYNLL2//IGF1//IGFBP3//S1PR1//RASGEF1A//PALLD//ARC//BAMBI//RND3//PAK6//PTEN//SDC1//BTG1//YES1//FAM83D//SYBU//POU4F1//PLXNA4//POU3F2//NRG1//EMX2//FLNA//TMOD1//TNNI1//ADCY3//C8ORF44-SGK3//SGK3//DOCK10//SGK1//SLK//F3//FOXF1//FAM89B//NTF3//PTP4A1//CBLL1//ARID2//SULF1//ZMYND8//TMEFF2//CLIC4//RAP2C//LDLRAD4//RECK//NAV3//DLG5//UNC5B//EPS8//SIRT1//PPARG//APCDD1//SIRPA//MSN//TNF//LPAR1//VEGFB//NFIB//KCNE1//KCNJ2//GJC1//SPRED1//MEOX2//KCNJ3//IRAK4//WDR1//EREG//MEMO1//RAF1//GCSAML//MET//PLAA//AKAP11// |
| GO:0007423 | sensory organ development | Biological process | 45 | 521 | 902 | 17653 | 1.69038945231539 | 0.000413175380714342 | 0.00862439845608363 | 3.38386556406057 | 0.0498891352549889 | RAB18//SMOC1//GJA1//TGFBR2//TFAP2A//GPM6A//CASP2//RDH10//LRIG1//NEUROD1//NEUROG1//INSIG2//FZD3//FZD6//MAPK1//TSHZ1//GSC//EDN1//NOG//RAB3GAP1//PKNOX1//WT1//FOXP2//GLI3//BNC2//PRDM16//WNT10A//MYCN//DLL1//FOXF2//LRP6//ZEB1//MAF//ROR1//CYTL1//CCM2//TUB//GABRA5//JUN//BMPR2//CLIC4//TMOD1//WNT7A//KCNK2//FRZB// |
| GO:0001525 | angiogenesis | Biological process | 48 | 567 | 902 | 17653 | 1.65680029094663 | 0.000422859745544945 | 0.00879938602806296 | 3.37380365587886 | 0.0532150776053215 | EDN1//PPP3R1//TGFBR2//ACVR1//E2F8//EFNB2//KDR//SLIT2//SPRED1//SULF1//AGO1//PPARG//FOXJ2//SARS//EFNA1//ETS1//TNFRSF12A//EGLN1//VASH2//F3//SIRT1//ISL1//ITGB8//RHOB//BTG1//C5AR1//VEGFB//ADAM12//PIK3CB//ADIPOR2//TNFAIP3//MEOX2//DLL1//PPP1R16B//S1PR1//ELK3//EREG//UNC5B//LEMD3//CLIC4//HOXA3//JUN//PIK3CA//PKNOX1//PTEN//RORA//CCL2//WNT7A// |
| GO:0044087 | regulation of cellular component biogenesis | Biological process | 68 | 881 | 902 | 17653 | 1.5105843742371 | 0.000434461662353301 | 0.00901308043710237 | 3.36204854041898 | 0.0753880266075388 | EPS8//GTF2H1//SLIT2//CNOT6L//PAN3//CNOT6//PMEPA1//LDLRAD4//PFN2//RASA1//RICTOR//NCK1//CCL21//CAMSAP1//CAMSAP2//STMN2//MET//CLIP1//NAV3//RAF1//IFNG//TNF//EP300//PMAIP1//IQGAP2//ARFIP1//TAPT1//ESR1//PRKAA1//FMR1//PPP1R16B//ZMYND8//GPM6A//TSC1//LPAR1//PPM1F//S1PR1//TMEFF2//ARHGAP6//PPFIA1//TMOD3//TMOD1//SLK//KDR//PTEN//ROBO2//SLITRK3//ADNP//WNT7A//DLG5//EDN1//MSN//BAG5//DNAJB6//RB1//VPS4B//RAP1A//RAP1B//RAPGEF2//SEPT9//FEZ2//SDC1//JUN//FOSL1//TBC1D12//RAB3GAP1//GJA1//CBFB// |
| GO:0060021 | roof of mouth development | Biological process | 13 | 87 | 902 | 17653 | 2.92439534113209 | 0.000453694463895897 | 0.00938328947806713 | 3.34323652008186 | 0.0144124168514412 | TSHZ1//FOXF2//GLI3//LRP6//MEOX2//INSIG2//BNC2//PRDM16//TFAP2A//TGFBR2//WNT7A//ARID5B//DHRS3// |
| GO:0044237 | cellular metabolic process | Biological process | 607 | 10957 | 902 | 17653 | 1.08419902675385 | 0.000459994101256725 | 0.00948457349633912 | 3.33724773746643 | 0.672949002217295 | SIRT1//UBXN2B//TRAPPC8//GABARAPL1//ATG16L1//WDR45B//ULK2//IGF1//NFIA//NFIB//NFIC//RBBP8//RBMS1//GADD45A//GTF2H1//PTEN//CCNT2//DIRAS3//NPAT//MTRR//TSHZ1//ZBTB18//CEBPB//TCERG1//STRAP//KLF12//GSC//ARX//DNMT3B//EDN1//EDNRB//EFNA1//CC2D1B//EN1//EP300//ESR1//JAZF1//CPEB3//FOXF1//MYT1L//SIN3B//ZFPM2//DNAJB5//GLI3//CNOT7//HIC1//FOXA1//HSBP1//ID2//IFNG//IRF2//AR//ISL1//JARID2//JUN//MAF//MDM4//MEF2A//MYB//NR4A2//PAX5//KLF3//POU4F1//PPARG//ZBTB4//RB1//CCND1//RNF2//SARS//PRDM16//SMARCA2//SUV39H1//ZEB1//TFAP2A//TGIF1//KLF10//TNF//UBE2I//WT1//ZNF217//LRP8//BHLHE41//TBL1XR1//E2F8//ARID5B//BHLHE40//TP63//RUNX3//NOG//FOXP2//PHF14//RANBP9//SPRED1//EREG//RASGEF1A//NRG1//IL2//MET//MAPK1//MAPK6//PSMA2//PSMD12//RAF1//RASA1//CCL2//SYNGAP1//RAPGEF2//PNRC1//DCP2//PPP2CA//CRKL//TAOK1//RAP1A//C1QTNF2//LPAR1//NTF3//PIK3CB//PRKAA1//C5AR1//UBE2V1//DUSP1//FBXO41//RNF145//ZNRF2//RNF167//MYLIP//ANKIB1//UBE2W//FBXW7//SMURF1//HECW2//KLHL42//SKP1//THOP1//LONRF3//FBXO30//CBFB//UBE3B//LONRF1//FBXO44//CNOT6L//PAN3//CNOT6//PATL1//COPS8//SRSF6//MBNL2//RBFOX3//FMR1//MBNL1//RBM25//SRSF2//ELAVL2//NOVA1//PCBP2//RBM41//NKTR//RRP15//PTGES3//NABP1//AGO1//GTF2E1//SEPSECS//SECISBP2L//EDN2//MGST1//ADH4//RDH10//SDC1//DHRS3//CHST1//ITGB8//ST3GAL5//ACSL1//ACSL4//RPP14//RPP25//NSG1//EPS15//VAMP3//SOCS1//IGFBP3//LRP6//PRR5L//PPP1R15B//SLIT2//NPTN//KDR//MAVS//CCND2//ABHD17C//CREBRF//ELK3//ETV5//ATF5//FOXF2//FLI1//GTF2A1//HIVEP2//IRF1//MEOX2//MYBL1//MYCN//NEUROD1//NHLH2//OTX2//KLF13//PKNOX1//POU3F2//FOXJ2//HIVEP3//BMPR2//SOX4//SOX5//BTF3//TFE3//TXK//FOSL1//KLF7//FUBP3//NMI//CLOCK//ZNF516//POLR3H//SLBP//ERRFI1//GPRC5A//SOCS5//ZER1//CACUL1//RNF38//MARCH10//PDZRN3//MYCBP2//FBXO33//KLHL3//RNF11//KLHL20//SPOPL//TRIM23//DCAF8//CBLL1//CUL5//KBTBD8//SOCS3//GFPT1//SLC35D1//NUP54//NLN//IMMP2L//COX8C//COX7A2//GLRX//GSTA2//GSTM3//DPYSL2//SLC23A2//HPRT1//CMPK1//ADSS//ADCY3//IMPDH1//PURA//GMNC//ESCO2//EPC2//ASTE1//USP28//SMUG1//OGG1//KDM2A//ASCC1//HMGB3//TSN//BAHD1//H2AFZ//ARID4B//ZMYND11//ZNF526//CREBL2//ZNF800//E2F5//ZNF367//ARID2//MED19//ZFP30//PHF8//FLII//ADNP//ZMYND8//ZNF521//HBP1//FOXP1//ZBTB11//ATAD2//MDFIC//HOXA3//HOXD1//ZNF680//ZNF662//NEUROG1//NFE2//NPAS2//IER5//COMMD10//BRWD1//BNC2//MED9//ZNF532//TRERF1//SLC2A4RG//CCNL1//PHTF2//ZBTB26//MIER1//ATXN1//ATXN7//SMARCD2//SS18//MED22//ZNF3//ZNF708//ZNF131//ZNF227//ZNF655//ZSCAN5A//SAP30L//ANP32A//PPP1R1B//PCGF5//LCOR//ST18//HIPK3//PPARGC1B//ELL2//NR5A2//NRBF2//AFF1//MLLT6//PHF20L1//PAK6//RORA//BTG1//CSDE1//SNIP1//TEAD2//RPS6KA5//EMX2//ETS1//BOLA3//NR3C2//RFX7//TRAK2//BDP1//NRBP1//CELF1//GRSF1//RTCA//PPP4R2//CPEB1//QKI//THG1L//CPEB2//DDX3Y//GTPBP1//DDX6//TSC1//SERP1//RIMKLB//TGM3//YES1//SPPL3//C8ORF44-SGK3//SGK2//FASTK//PDIK1L//CDK19//MORC3//MMD//SGK3//GRK6//TBK1//CDK17//PIK3CA//PRKACB//CCL8//SGK1//CDK15//TGFBR2//MEX3B//ACVR1//RASSF2//GNAQ//NCK1//GSKIP//YWHAG//PPM1F//PTPN21//PPTC7//PPP1R12A//CTDSPL2//PPP2CB//PPP3R1//HS3ST5//PCMTD1//FUT9//B3GALNT2//FUT2//FUT5//ST8SIA5//KCNE1//CCDC126//ALG2//EOGT//GCNT4//VEGFB//ALPI//RECK//PYURF//PIGP//PIGA//CTSF//FBXO8//USP27X//BAG6//USP38//CPD//SLC6A6//MCCC2//SERINC3//PSAT1//SLC6A8//ABHD5//PRKAR2B//MSMO1//ELOVL5//INSIG2//PLAA//LCLAT1//CDS1//ABHD3//SLC44A1//MTMR9//KDELC1//SPHK2//NEU1//GPHN//PRUNE2//BNIP2//SESN1//PMAIP1//VTI1A//RAB12//ANXA7//DRAM1//TMBIM6//EGLN3//F3//CASP2//GRM3//DLL1//G3BP2//IRAK4//UGT2B7//PTHLH//LPCAT3//DGKE//ACBD5//GLT6D1//PI15//RRAGD//CCL21//CRHR1//NPNT//CALM2//INHBB//BMP3//TTK//GDF5//CNEP1R1//LDLR//FKBP15//PLEKHF2//SERPINI1//SERPINB13//SPOCK1//ARPP19//PRR16//FXR1//INTS8//VPS37A//DYNLL2//CHMP3//VPS4B//EI24//SNX5//GNPTG//DGKH//HDHD2//FLNA//BAMBI//WNT7A//DNAJB6//RASD1//BTAF1//MBD6//OTUD3//TNFAIP3//WDR20//TOB1//NANOS1//TIA1//CEP41//RICTOR//STK33//ROR1//OGN//PRELP//ZDHHC22//ZDHHC7//ZDHHC18//MCFD2//CRTAP//EGLN1//ELOVL7//PON2//NAGA//ADIPOR2//DCUN1D4//KCNH4//SLK//SPTSSB//SPTSSA//SULF1//DSEL//DNAJB9//JKAMP//LEMD3//CXCL9//ADRB1//PDE5A//ANAPC15//BAG5//NDFIP2//MOB1B//RAP2C//RWDD3//CCT6A//MID1//SEMA4C//PFN2//IFNA1//TNRC6B//PPP1R16B//PTPRG//PTP4A1//PTP4A2//CDC14A//WDFY3//ITSN1//RDH14//MYOG//TDRD5//ARL6IP1//CD2AP//ARMC8//FZD6//YTHDF2//RCAN3//PPP2R5E//CKAP4//CALU//CD3E//MID1IP1//CCNY//ADCYAP1//S1PR1//KPNA6//ARMCX3//CYTL1//PAIP2//BOLL//GRIK2//GPCPD1//EFR3A//SH3D19//RHEBL1//CCM2//ARRDC3//UGT2B28//PMEPA1//LDLRAD4//FAM83D//GPR55//OTUD1//KCNAB2//UBA5//CAST//SNAP25//FEZ2//RAB3GAP1//MSN//HSBP1L1//FAM168A//GJA1//KCNK2//TBC1D12// |
| GO:0050890 | cognition | Biological process | 28 | 277 | 902 | 17653 | 1.97829132213213 | 0.000463038761351135 | 0.00950607545524885 | 3.33438265231617 | 0.0310421286031042 | MAPK1//PTEN//ARC//JUN//PRKAR2B//ATXN1//SLC6A1//FOSL1//NRXN3//CEBPB//KCNK2//NTF3//PAIP2//KCNK10//ADNP//CPEB3//LDLR//SGK1//CCND2//GABRA5//SNAP25//ADCY3//NPTN//PPP1R1B//SYNGAP1//FOXP2//EN1//NEUROG1// |
| GO:0003002 | regionalization | Biological process | 32 | 333 | 902 | 17653 | 1.88069222215564 | 0.000463848129562638 | 0.00950607545524885 | 3.33362419032718 | 0.0354767184035477 | EP300//FOXF1//DLL1//MEOX2//ISL1//CXXC4//NEUROG1//WT1//RNF2//WNT3//TSHZ1//CRKL//EMX2//ARC//GLI3//GRSF1//HOXA3//LRP6//NEUROD1//BMPR2//EDN1//EN1//WNT7A//NOG//TP63//GSC//FOXA1//TDRD5//AR//OTX2//PRKACB//ACVR1// |
| GO:0010921 | regulation of phosphatase activity | Biological process | 20 | 171 | 902 | 17653 | 2.28900040196574 | 0.000471617484022181 | 0.00963572846186554 | 3.32641010291197 | 0.0221729490022173 | TNF//PPARGC1B//NPNT//MTMR9//SPRED1//FKBP15//PPP1R12A//PLEKHF2//ARPP19//PPP1R1B//CALM2//PPP1R15B//RCAN3//UBXN2B//PPP4R2//PPP1R16B//IGFBP3//PPP2R5E//TSC1//IFNG// |
| GO:0045670 | regulation of osteoclast differentiation | Biological process | 11 | 66 | 902 | 17653 | 3.26182557280118 | 0.000474448850776464 | 0.00963572846186554 | 3.3238106009878 | 0.0121951219512195 | GPR55//PPARGC1B//TMEM64//IFNG//POU4F1//KLF10//TNF//FBXW7//CEBPB//TFE3//RASSF2// |
| GO:1902117 | positive regulation of organelle assembly | Biological process | 11 | 66 | 902 | 17653 | 3.26182557280118 | 0.000474448850776464 | 0.00963572846186554 | 3.3238106009878 | 0.0121951219512195 | CNOT6L//PAN3//CNOT6//TAPT1//EDN1//MSN//TNF//SEPT9//SDC1//VPS4B//RAB3GAP1// |
| GO:0045444 | fat cell differentiation | Biological process | 23 | 210 | 902 | 17653 | 2.14348537641221 | 0.000477241864022285 | 0.00966343331252309 | 3.32126146668567 | 0.0254988913525499 | DLK2//SIRT1//ZFPM2//RORA//TNF//CEBPB//CREBL2//TMEM64//FRZB//ID2//PPARG//PRDM16//TBL1XR1//ADRB1//ZNF516//EP300//ATF5//BBS9//INHBB//NR4A2//CCND1//ARID5B//SOCS1// |
| GO:0048585 | negative regulation of response to stimulus | Biological process | 112 | 1615 | 902 | 17653 | 1.35724259128322 | 0.000482898152429228 | 0.0097487767309817 | 3.31614445620936 | 0.124168514412417 | SPRED1//DUSP1//PPP2CA//PPARG//TNFAIP3//SLIT2//TNF//ADCYAP1//ERRFI1//GPRC5A//SOCS5//IGFBP3//KDR//RGS7BP//MET//ROR1//RAF1//PRR5L//ACVR1//PMEPA1//LDLRAD4//PTEN//GSC//APCDD1//FRZB//HIC1//SHISA2//CCND1//CXXC4//EDN1//STRAP//SIRT1//LEMD3//FAM89B//BAMBI//SMURF1//PRDM16//TGFBR2//TOB1//CTDSPL2//NOG//PRKAA1//TSC1//ISL1//TP63//DLG5//SULF1//EPS15//SOCS1//SOCS3//ZMYND11//ESR1//RORA//FOXF1//EFNA1//HIPK3//ATXN7//LPAR1//YTHDF2//FBXW7//DLK2//NMI//GLI3//RB1//CD3E//NEUROD1//PPP2CB//RASA1//SYNGAP1//NCK1//DHRS3//SEMA4C//WNT3//PCBP2//ETS1//IL2//ROBO2//CD2AP//ARHGAP12//OTUD3//PHLDA3//CALM2//CNOT7//FOXP1//GJA1//RANBP9//TMEM64//LRP6//PSMA2//PSMD12//FZD6//IGF1//C5ORF30//CREBRF//EREG//NRG1//RASSF2//OGG1//PRKACB//BAG5//TMBIM6//DNAJB9//PPP1R15B//SESN1//LYPD6//CLOCK//CCL2//PHF14//NR4A2//AR//UNC5B//BCL2A1// |
| GO:0021675 | nerve development | Biological process | 12 | 77 | 902 | 17653 | 3.05001871742448 | 0.000503269196034449 | 0.0101297903951815 | 3.29819965096918 | 0.0133037694013304 | LPAR1//ISL1//NEUROG1//POU4F1//TFAP2A//PLXNA4//HOXA3//GLI3//SULF1//GABRA5//LRIG1//NTF3// |
| GO:0051129 | negative regulation of cellular component organization | Biological process | 53 | 649 | 902 | 17653 | 1.59824427141876 | 0.000512243033750683 | 0.0102511408042851 | 3.29052393907457 | 0.0587583148558758 | EPS8//STMN2//CAMSAP1//CAMSAP2//MID1//MID1IP1//NAV3//TTK//SLIT2//LPAR1//EFNB2//MYLIP//PTPRG//SPOCK1//TSC1//BAG5//PMEPA1//LDLRAD4//PFN2//JARID2//RAF1//ATAD2//DCP2//EP300//TNF//TMEFF2//AR//ULK2//PTEN//SEMA4C//WNT3//TRAK2//SYNGAP1//RAPGEF2//S1PR1//ARHGAP6//MET//PPFIA1//DNMT3B//PAX5//TMOD3//TMOD1//ROBO2//EFNA1//PHF8//SIRT1//DNAJB6//IGF1//DUSP1//ANAPC15//FEZ2//LDLR//CCL21// |
| GO:0014074 | response to purine-containing compound | Biological process | 18 | 147 | 902 | 17653 | 2.39644327797638 | 0.000513304399840347 | 0.0102511408042851 | 3.2896250131244 | 0.0199556541019956 | DNMT3B//PPARG//PRKAA1//PTEN//TRPC3//CNGA3//PPARGC1B//DUSP1//JUN//SDC1//FOSL1//KCNE1//RAP1A//RAP1B//WT1//RAPGEF2//ADSS//SLC6A1// |
| GO:0030278 | regulation of ossification | Biological process | 21 | 185 | 902 | 17653 | 2.22156768742135 | 0.000515194405501723 | 0.0102511408042851 | 3.2880288616283 | 0.0232815964523282 | SMURF1//S1PR1//GJA1//BMPR2//TFAP2A//ACVR1//SMOC1//RASSF2//TOB1//TMEM64//ID2//TNF//NOG//CEBPB//NPNT//GLI3//IGF1//TP63//LRP6//MAPK1//DHRS3// |
| GO:0051567 | histone H3-K9 methylation | Biological process | 7 | 29 | 902 | 17653 | 4.72402324336723 | 0.000515361211512191 | 0.0102511408042851 | 3.28788827158283 | 0.00776053215077605 | SUV39H1//ARID4B//DNMT3B//PAX5//SIRT1//JARID2//MYB// |
[truncated: 360,044 more chars]
