# Supplementary material for: Altered microRNA Transcriptome in Cultured Human Liver Cells upon Infection with Ebola Virus
Source: Int J Mol Sci. 2021 Apr 6;22(7):3792. doi: 10.3390/ijms22073792 (PMC8038836; doi:10.3390/ijms22073792)
Supplement: Supplementary file 1 [file ijms-22-03792-s001.zip › Supplementary_File/C_ GO_Analysis_Results/16-30nt_go_Makona-24h-Huh7_vs_Control-24h-Huh7_up.mature_mirna_targets/CC_result(Human).html]

| GO.ID | Term | Ontology | Count | Pop.Hits | List.Total | Pop.Total | Fold.Enrichment | Pvalue | FDR | Enrichment.Score | Gene.Ratio | GENES |
| --- | --- | --- | --- | --- | --- | --- | --- | --- | --- | --- | --- | --- |
| GO:0005622 | intracellular | Cellular component | 817 | 14673 | 929 | 18698 | 1.12068247464625 | 1.28638526287882e-14 | 1.10500494081291e-11 | 13.8906289439432 | 0.879440258342304 | SIN3B//TRERF1//ZNF217//SAP30L//TBL1XR1//EP300//BDP1//GOLGA6A//YIPF6//CNIH1//STX6//RTN3//SEC23A//FUT9//SERINC3//CLCN5//VTI1A//B3GALNT2//RAB12//HS3ST5//FUT5//GJA1//ATP2C1//ARFIP1//RAB30//ST8SIA5//TRIM23//RND3//GCNT4//NDFIP2//ZDHHC7//SYBU//SLC30A6//PMEPA1//ACBD3//APOO//RIC3//GNPTG//CHST1//ST3GAL5//AP1S2//MCFD2//DSEL//MMGT1//HS3ST3A1//DCUN1D4//FBXO8//RNF11//ANKIB1//RNF2//UBE2V1//CBLL1//IER5//PPP2CA//PPP2CB//PPP2R5E//STRN//NCK1//PPP1R15B//GTPBP1//ZBTB18//JUN//SUV39H1//NEDD1//BBS9//GNAQ//CCNL1//CCND1//CCND2//CCNT2//CCNY//TRAPPC8//PLEKHF2//ULK2//GABARAPL1//ATG16L1//POLR3H//GTF2H1//PSMA2//PSMD12//PYURF//PIGP//PIGA//FMR1//DYNC1LI2//FBXO28//PPP1R12A//CLIP1//TTK//RASSF2//CFDP1//DCTN6//CENPO//DCTN5//CEBPB//PTGES3//PURA//NABP1//ESCO2//BAHD1//HIC1//MAF//MYCN//RB1//H2AFZ//HIST2H2BE//ESR1//SIRT1//IRF1//AR//MEF2A//POU4F1//SMARCA2//SMARCD2//TP63//RUNX3//DDX6//UBE2I//SYCE1//PCGF5//VPS37A//CHMP3//TMEM201//CALM2//KATNAL1//CDC14A//VPS4B//MZT1//PNRC1//DCP2//PATL1//TNRC6B//PAN3//AGO1//CNOT7//YTHDF2//CPEB1//MEX3B//MBD6//E2F5//TMEM217//JAZF1//SESN1//FOXA1//NFIB//NFIC//PAX5//FOXJ2//PAK6//ARC//DYNLL2//SEPT9//ROR1//ZYX//PALLD//WDR1//MCCC2//GLI3//KCNE1//LDLR//NAGA//NEU1//PON2//DRAM1//ARRDC3//RRAGD//SNX2//TNFAIP3//CTSF//CMC4//CCDC58//PPARGC1B//PPTC7//PRSS35//ELK3//ACSL1//SLC44A1//CLIC4//TIMM9//GLRX//TCAIM//GRSF1//SLC25A6//COX8C//JARID2//BOLA3//MGST1//NKTR//OGG1//DCAF8//RAB8B//PMAIP1//MIEF1//THG1L//PPP3R1//MAPK1//SMURF1//PTEN//MAVS//RDH14//RAF1//SGK1//TRAK2//SOX4//YWHAG//PRR5L//ABHD11//CASP2//CCM2//BAG5//RANBP9//DNAJB6//C8ORF44-SGK3//SGK2//HIPK3//DNAJA2//TACC2//IQGAP2//COPS8//RALBP1//CKAP4//RAPGEF4//STRAP//AKAP11//EGLN3//KLF12//ARAP2//CNN3//ADH4//EDARADD//UBXN2B//CRKL//KRT80//SYT6//FBXO41//SH3D19//ADSS//SPRED1//DENND1B//DPYSL2//TXLNA//DENND6A//PRSS55//EPS15//RASGEF1A//ZNRF2//ATF5//DZIP1//RAB3GAP1//RAB18//WDFY3//RAB21//PDZRN3//CDK19//FLI1//FLII//FLNA//EFR3A//RGL1//OTUD3//CAMSAP2//DNAJC16//KPNA6//SGK3//CNOT6L//RICTOR//LCLAT1//GABRA5//CNEP1R1//DNAJB5//ANAPC15//APPL1//TES//STEAP2//KLHL3//GFPT1//SNX5//SERP1//KLHL20//OSTM1//TBK1//MYLIP//GSTA2//GSTM3//GTF2A1//GTF2E1//NRBP1//PSAT1//ANXA7//HPRT1//AGFG1//HSBP1//ID2//IMPDH1//IRF2//RHOB//USP27X//ARHGAP1//ARHGAP6//MBNL1//MID1//NR3C2//KRTAP5-6//HSBP1L1//MSN//MTRR//NPAS2//ARHGEF4//PCBP2//MEMO1//CUTC//SEPSECS//ABHD5//IRAK4//RAB9B//PEX5L//CMPK1//SERPINB13//PIK3CA//PIK3CB//BRWD1//ERRFI1//RIN2//WDR44//EGLN1//PPARG//AFTPH//MTMR10//PXK//SEC61A2//FBXW7//DOCK10//PRKAA1//PRKACB//ENAH//PRKAR2B//MAPK6//NXF2//GPCPD1//WDR45B//SPHK2//KCMF1//PTHLH//CNOT6//RIMKLB//KLHL42//TAOK1//RANBP10//ZBTB4//RAP2C//MID1IP1//RAP1A//RAP1B//RASA1//RBMS1//SARS//ATXN1//ATXN7//PRDM16//SRSF2//ITSN1//S100PBP//SKP1//CDK15//MTMR9//SNAP25//BNIP2//SOS2//HSPA13//VAMP1//SYT1//BTF3//ZEB1//TFAP2A//TFE3//TGFBR2//THOP1//TIA1//TMOD1//TNNI1//TSN//TSC1//TUB//NXF2B//VBP1//WT1//YES1//CSDE1//SLBP//BAG6//E2F8//SNIP1//UBA5//CCDC6//KCNIP4//FOSL1//CUL5//PTP4A2//FXR1//SNX27//SBF2//CAST//FBXO30//SYDE2//PPP1R1B//KBTBD8//TEAD2//PPFIA1//KCNAB2//DGKE//KRTAP2-4//ALG2//SOCS1//DDX3Y//PDE5A//SYNGAP1//SKAP2//LMLN//SOCS3//CCT6A//NMI//LONRF1//MOB1B//FAM114A1//VAMP3//FBXO44//ARHGAP12//HOMER1//EI24//CEP41//CLOCK//PPM1F//SOCS5//RAPGEF2//SNX17//GIT2//C2CD5//G3BP2//ACBD5//TOB1//MBNL2//TSHZ1//CELF1//ZMYND11//TCERG1//RPP14//ZNF526//CPEB2//CREBL2//GSC//RBFOX3//PHF13//PPP4R2//RNF38//CREBRF//GADD45A//ZNF800//ARX//DNMT3B//DUSP1//ZNF367//CC2D1B//EMX2//EN1//ETS1//ETV5//MED19//ZFP30//CPEB3//FOXF1//FOXF2//MYT1L//MYCBP2//PHF8//ADNP//ZFPM2//ZMYND8//NR5A2//NUTM1//ZNF521//RWDD3//PPP1R16B//NSG1//FOXP1//ATAD2//PURG//MDFIC//TNPO2//HIVEP2//HMGB3//SPOPL//ZNF680//IGFBP3//ISL1//KCNK2//KDR//ZNF662//RGS7BP//MDM4//MEOX2//AFF1//MLLT6//ASNA1//MYB//MYBL1//MYOG//NAP1L3//NEUROD1//NEUROG1//NFIA//NFE2//NHLH2//NOVA1//NPAT//NR4A2//OTX2//ASCC1//PHF20L1//CDK17//COMMD10//GSKIP//ARMCX3//KLF13//RASD1//ARID4B//PKNOX1//POU3F2//CCNJ//C2ORF42//ZNF532//UBE2W//BTBD7//SLC2A4RG//PHTF2//ZBTB26//MIER1//HIVEP3//RBBP8//RORA//SH3BGRL//GMNC//RFX7//SOX5//SS18//BTG1//TMBIM6//KLF10//TXK//ZNF3//ZNF708//ZNF131//ZNF227//PTP4A1//ZNF655//ZSCAN5A//BHLHE41//NPEPL1//ANP32A//ARID5B//ZIC5//BHLHE40//KLF7//RTCA//BSN//FUBP3//RPS6KA5//NREP//PLAA//FOXP2//QKI//ZNF516//PHF14//ST18//LBR//NUP54//LEMD3//UGT2B28//NAV3//GPHN//SIVA1//ARPP19//WASF3//ADCY3//IFI44L//STMN2//PTPN21//RCAN3//SLC26A7//PCMTD1//RHEBL1//SLAIN1//FRMD6//CNGA3//LYPD6//RDH10//CAMSAP1//PRUNE2//DGKH//DBN1//PRICKLE2//LCA5//DTNA//EDN1//SAMD14//TAPT1//EVC//ACSL4//WDR47//IQSEC2//CD2AP//PHLDA3//FAM89B//BAMBI//RNF167//GLRB//NRBF2//NRG1//S100A7A//NANOS1//IGF1//LASP1//SNX19//DNAJB9//MET//PAIP2//ZMYND10//GULP1//PFN2//ZFAND6//ANO1//LRP2BP//HECW2//RBM25//SLC6A11//BMPR2//BOLL//SPOCK1//MED22//TGM3//TNS1//FZD3//SHCBP1//VASH2//CXXC4//FAM83D//UBE3B//C5ORF30//DLG5//SLIT2//RAB28//FEZ2//RALGPS1//SLK//DAZAP2//SLC23A2//PDIK1L//S1PR1//ARID2//ELAVL2//ELL2//KDM2A//MORC3//SMUG1//NAALADL2//HBP1//ZBTB11//HOXA3//HOXD1//KLF3//CTDSPL2//BNC2//RPP25//INTS8//USP28//SRSF6//ZMAT3//TGIF1//LDLRAD4//ADAM12//LCOR//CBFB//BTAF1//WDR20//RBM41//YPEL2//SLC14A1//GPRC5A//MTX3//ARMCX6//BCL2A1//ABCB7//SLC25A34//MICU3//SLC25A28//COX7A2//NLN//FASTK//CLCN6//SPPL3//MMD//CRHR1//LPAR1//TJAP1//ADRB1//FKBP15//LRP6//SLC9A6//SLC31A2//TBC1D12//CDS1//CRTAP//KIF1C//TMED1//TMEM64//SMIM14//SULF1//ZDHHC22//FIBIN//SHISA2//FAM19A1//IER3IP1//PLP2//STIM2//ELOVL5//RTN1//MSMO1//ELOVL7//CALU//ZDHHC18//ARL6IP1//EOGT//WNT3//WNT7A//KDELC1//GJC1//LPCAT3//SPTSSB//SPTSSA//REEP3//SLC35D1//INSIG2//JKAMP//UGT2B7//DHRS3//KCNJ2//CLIP4//CPD//FUT2//GLT6D1//SGCE//CCDC126//OGN//PRELP//SDC1//HS3ST1//TEX261//C16ORF70//KLK15//C5AR1//MARCKS//KIF13A//TMOD3//LRP8//EPS8//NTF3//FAM118B//CFL2//TMEM63B//MED9//FAM76B//NPNT//ZPLD1//FZD6//CHRM2//SEMA4C//NHLRC2//VEGFB//DLL1//CHIC1//CACUL1//ZER1//EDNRB//CABP7//CALN1//EPC2//GRIK2//TDRD5//ARMC8//SERPINI1//BRI3//C6ORF120//CHRNB4//IMMP2L//SESTD1//INHBB//STK33//RIMS4//TNF//ABHD17C//SIRPA//EMC7//PSD//NMUR1//ADCYAP1//EDN2//EREG//KCNH4//ARL5A//IFNG//IL2//HPCAL4//UBL3//ARL15//CCL2//LONRF3//GDF5//DIRAS3// |
| GO:0044424 | intracellular part | Cellular component | 802 | 14395 | 929 | 18698 | 1.1213524609931 | 1.63594396462598e-13 | 7.02637932806859e-11 | 12.7862315761357 | 0.863293864370291 | DCUN1D4//FBXO8//RNF11//ANKIB1//RNF2//UBE2V1//CBLL1//IER5//PPP2CA//PPP2CB//PPP2R5E//STRN//GNAQ//CCNL1//CCND1//CCND2//CCNT2//PSMA2//PSMD12//RANBP9//TOB1//MBNL2//GPHN//CEBPB//SIVA1//TACC2//CELF1//PTGES3//ARPP19//IQGAP2//SEPT9//WASF3//ADCY3//IFI44L//STMN2//PTPN21//RCAN3//STRAP//AKAP11//EGLN3//SLC26A7//PCMTD1//ARAP2//RHEBL1//SLAIN1//FRMD6//CNGA3//LYPD6//CPEB2//RBFOX3//PPP4R2//RDH10//CAMSAP1//PRUNE2//ADSS//DGKH//DBN1//GADD45A//DDX6//PRICKLE2//DCP2//LCA5//DNMT3B//DTNA//DUSP1//E2F5//EDN1//TMEM201//TXLNA//SAMD14//DENND6A//TAPT1//ESR1//ETS1//EVC//ACSL4//CCNY//ZNRF2//CPEB3//DZIP1//WDR47//WDFY3//IQSEC2//FLNA//ARC//OTUD3//SIN3B//FMR1//ADNP//SIRT1//ZFPM2//CD2AP//PHLDA3//ZMYND8//FAM89B//CNOT6L//NR5A2//CNEP1R1//NUTM1//BAMBI//CLIC4//RWDD3//RNF167//APPL1//AGO1//NSG1//ARFIP1//SESN1//KLHL20//GLI3//GLRB//TBK1//GRSF1//GSTM3//CNOT7//PSAT1//MDFIC//NRBF2//TNPO2//NRG1//HMGB3//HPRT1//AGFG1//HSBP1//S100A7A//SPOPL//ID2//NANOS1//IGF1//IMPDH1//IRF1//AR//ISL1//KDR//RHOB//RND3//ARHGAP1//LASP1//ARHGAP6//SNX19//RGS7BP//MAF//MBNL1//DNAJB9//MEOX2//MET//ASNA1//HSBP1L1//MSN//PPP1R12A//NAGA//NCK1//NEU1//NEUROD1//NFE2//NPAT//ROR1//NR4A2//DCAF8//PCBP2//MEMO1//CUTC//SEPSECS//IRAK4//PAIP2//CDK17//ZMYND10//COMMD10//GULP1//GSKIP//CMPK1//ARID4B//PFN2//SERPINB13//PIK3CA//PIK3CB//PKNOX1//ERRFI1//ZFAND6//POU4F1//EGLN1//NDFIP2//AFTPH//MTMR10//PXK//ANO1//UBE2W//FBXW7//DRAM1//DOCK10//PRKAA1//PRKAR2B//LRP2BP//MAPK1//MAPK6//NXF2//SLC2A4RG//PAK6//SMURF1//PTEN//PTHLH//RIMKLB//HECW2//TAOK1//MIER1//RAP2C//PURA//RBM25//RRAGD//RAF1//RAP1A//RASA1//HIVEP3//SARS//ATXN1//SGK1//CPEB1//SH3BGRL//SKP1//CDK15//SLC6A11//BMPR2//TRAK2//MTMR9//BOLL//SNAP25//BNIP2//SNX2//SOX4//SPOCK1//HSPA13//MED22//BTG1//TMBIM6//TFE3//TGM3//TIA1//TNFAIP3//TNS1//TSN//TSC1//TTK//TUB//NXF2B//TXK//UBE2I//VBP1//WT1//PTP4A1//SLBP//ZNF655//BAG6//NPEPL1//FZD3//SHCBP1//VASH2//UBA5//CXXC4//KCNIP4//CALM2//PTP4A2//FXR1//ANP32A//FAM83D//CASP2//CCM2//KATNAL1//SYDE2//PPP1R1B//PPP1R15B//PPFIA1//DGKE//ALG2//BHLHE40//CDC14A//TP63//RUNX3//SOCS1//SYNGAP1//SKAP2//FUBP3//UBE3B//SOCS3//C5ORF30//CCT6A//NMI//DLG5//RPS6KA5//MOB1B//NREP//SLIT2//RAB28//PLAA//QKI//HOMER1//VPS4B//FEZ2//RALGPS1//SOCS5//RAPGEF2//SLK//RASSF2//DAZAP2//GIT2//G3BP2//SLC23A2//GSC//EP300//ATF5//FOXF1//FOXF2//JUN//MEF2A//MYOG//NHLH2//NPAS2//ASCC1//POU3F2//TRERF1//RB1//ZEB1//E2F8//TEAD2//CLOCK//PRKACB//PPP3R1//BAG5//TRAPPC8//PATL1//CNOT6//SNX5//YES1//KCNAB2//ARMC8//CACUL1//CUL5//GTPBP1//EPS15//PRDM16//LEMD3//LBR//ABCB7//TIMM9//SLC25A34//MICU3//SLC25A6//CSDE1//SLC25A28//POLR3H//UGT2B28//NAV3//ACSL1//SLC44A1//GJA1//MTX3//MGST1//PMAIP1//ARMCX6//MIEF1//MAVS//BCL2A1//VAMP1//JARID2//RICTOR//PRR5L//SLC9A6//SPPL3//VPS37A//VTI1A//DENND1B//S1PR1//CC2D1B//MYCBP2//RAB30//NOVA1//PAX5//RAB8B//PPARG//SYBU//SPHK2//PMEPA1//RBBP8//SLC14A1//SMARCA2//TFAP2A//UGT2B7//LDLRAD4//RIC3//CLIP4//AP1S2//BTAF1//GPRC5A//VAMP3//SNX17//PLEKHF2//ULK2//ATG16L1//CMC4//PYURF//CCDC58//PPARGC1B//DYNLL2//PPTC7//PRSS35//ELK3//GABARAPL1//GLRX//TCAIM//COX8C//BOLA3//NKTR//OGG1//PON2//THG1L//RDH14//MCCC2//ACBD3//YWHAG//ZNF217//APOO//ABHD11//DNAJB6//C8ORF44-SGK3//SGK2//HIPK3//STX6//DNAJA2//SEC23A//DCTN6//COPS8//RALBP1//CKAP4//RAPGEF4//KLF12//CLCN5//NEDD1//CNN3//ADH4//EDARADD//UBXN2B//CRKL//KRT80//SYT6//FBXO41//SH3D19//SPRED1//DYNC1LI2//DPYSL2//RAB12//PRSS55//RASGEF1A//JAZF1//RAB3GAP1//RAB18//RAB21//PALLD//PDZRN3//CDK19//TNRC6B//FLI1//FLII//EFR3A//RGL1//CAMSAP2//DNAJC16//KPNA6//SGK3//LCLAT1//GABRA5//PAN3//DNAJB5//ANAPC15//TES//STEAP2//KLHL3//GFPT1//SERP1//BBS9//OSTM1//MYLIP//GSTA2//GTF2A1//GTF2E1//NRBP1//HIC1//ANXA7//IRF2//USP27X//MID1//NR3C2//KRTAP5-6//MZT1//MTRR//ARHGEF4//ABHD5//RAB9B//YTHDF2//PEX5L//CHMP3//BRWD1//RIN2//WDR44//SEC61A2//ENAH//GPCPD1//WDR45B//KCMF1//KLHL42//RANBP10//ZBTB4//MID1IP1//RAP1B//RBMS1//CLIP1//ATXN7//SRSF2//ITSN1//S100PBP//NABP1//SOS2//SYT1//BTF3//TGFBR2//THOP1//TMOD1//TNNI1//ZYX//CENPO//SNIP1//CCDC6//FOSL1//SNX27//SBF2//CAST//HIST2H2BE//FBXO30//MEX3B//DCTN5//KBTBD8//KRTAP2-4//DDX3Y//PDE5A//LMLN//LONRF1//FAM114A1//FBXO44//ARHGAP12//EI24//CEP41//PPM1F//C2CD5//WDR1//RTN3//CDS1//CRTAP//KIF1C//TMED1//B3GALNT2//TMEM64//SMIM14//SULF1//ZDHHC22//YIPF6//FIBIN//SHISA2//LRP6//FAM19A1//IER3IP1//PLP2//ZDHHC7//PHTF2//STIM2//ELOVL5//RTN1//MSMO1//ELOVL7//CALU//ZDHHC18//FUT9//CPD//ESCO2//MMD//FUT2//FUT5//ATP2C1//GOLGA6A//GLT6D1//KCNJ2//LDLR//SLC30A6//GNPTG//SGCE//CCDC126//MMGT1//TJAP1//SS18//DLL1//NTF3//CHIC1//CNIH1//MCFD2//SERINC3//CABP7//PPP1R16B//INHBB//RASD1//STK33//CALN1//FASTK//EOGT//IGFBP3//WNT3//WNT7A//KDELC1//OGN//PRELP//SDC1//HS3ST1//SERPINI1//ZER1//TMOD3//NPNT//KCNE1//ARRDC3//CTSF//TBC1D12//EPS8//MARCKS//RORA//C6ORF120//ACBD5//TSHZ1//ZBTB18//ZMYND11//TCERG1//PNRC1//RPP14//MBD6//ZNF526//CREBL2//PHF13//RNF38//CREBRF//ZNF800//ARX//ZNF367//EMX2//EN1//ETV5//MED19//ZFP30//MYT1L//PHF8//ZNF521//FOXP1//ATAD2//PURG//H2AFZ//HIVEP2//FOXA1//ZNF680//TRIM23//KCNK2//ZNF662//MDM4//AFF1//MLLT6//MYB//MYBL1//MYCN//NAP1L3//NEUROG1//NFIA//NFIB//NFIC//OTX2//PHF20L1//ARMCX3//KLF13//CCNJ//C2ORF42//ZNF532//BTBD7//FOXJ2//ZBTB26//GMNC//RFX7//SOX5//SUV39H1//KLF10//ZNF3//ZNF708//ZNF131//ZNF227//ZSCAN5A//BHLHE41//SAP30L//TBL1XR1//ARID5B//PCGF5//ZIC5//KLF7//RTCA//BSN//FOXP2//ZNF516//PHF14//ST18//PSD//CRHR1//LPAR1//ADRB1//FKBP15//SLC31A2//TEX261//KLK15//TNF//NUP54//NLN//C16ORF70//TDRD5//FBXO28//TMEM217//SMUG1//YPEL2//ZMAT3//DHRS3//CFDP1//CFL2//TMEM63B//SESTD1//BAHD1//SMARCD2//SYCE1//GTF2H1//PDIK1L//ARID2//ELAVL2//ELL2//KDM2A//MORC3//NAALADL2//HBP1//ZBTB11//HOXA3//HOXD1//KLF3//CTDSPL2//BNC2//RPP25//INTS8//BDP1//USP28//SRSF6//TGIF1//ADAM12//LCOR//CBFB//WDR20//RBM41//MED9//EDNRB//COX7A2//KIF13A//REEP3//LRP8//HS3ST5//ST8SIA5//GCNT4//CHST1//ST3GAL5//DSEL//HS3ST3A1//PIGP//PIGA//ARL6IP1//GJC1//LPCAT3//SPTSSB//SPTSSA//SLC35D1//INSIG2//JKAMP//EMC7//C5AR1//CLCN6//ZPLD1//FZD6//RIMS4//NHLRC2//VEGFB//EPC2//GRIK2//BRI3//SEMA4C//CHRNB4//SIRPA//ABHD17C//FAM118B//FAM76B//CHRM2//IMMP2L// |
| GO:0043229 | intracellular organelle | Cellular component | 713 | 12604 | 929 | 18698 | 1.13857220305956 | 9.60955349581165e-11 | 2.75153548430074e-08 | 10.0172967911879 | 0.767491926803014 | SLC9A6//ARAP2//SPPL3//VPS37A//VTI1A//DENND1B//POLR3H//DNMT3B//S1PR1//CC2D1B//DENND6A//EPS15//MYCBP2//APPL1//GJA1//RAB30//AGFG1//RHOB//RND3//NEU1//NOVA1//PAX5//RAB8B//UGT2B28//NDFIP2//PPARG//AFTPH//SYBU//SPHK2//PMEPA1//RBBP8//SLC14A1//SMARCA2//BNIP2//HSPA13//TFAP2A//TNFAIP3//UGT2B7//VBP1//LDLRAD4//BAG6//RIC3//CLIP4//UBA5//TEAD2//RUNX3//AP1S2//BTAF1//GPRC5A//VAMP3//CLOCK//SNX17//GIT2//CMC4//PYURF//CCDC58//PPARGC1B//DYNLL2//PPTC7//DDX6//PRSS35//ELK3//ACSL1//SIRT1//SLC44A1//GABARAPL1//CLIC4//TIMM9//GLRX//TCAIM//GRSF1//SLC25A6//COX8C//JARID2//BOLA3//MGST1//NKTR//OGG1//DCAF8//PMAIP1//PON2//MIEF1//THG1L//PPP2CA//PPP3R1//MAPK1//SMURF1//PTEN//MAVS//RDH14//RAF1//MCCC2//SGK1//ACBD3//TRAK2//SOX4//YWHAG//ZNF217//APOO//PRR5L//ABHD11//CASP2//CCM2//TP63//BAG5//RANBP9//DNAJB6//TOB1//MBNL2//TSHZ1//ZBTB18//CEBPB//CELF1//PTGES3//ZMYND11//TCERG1//COPS8//PNRC1//RPP14//EGLN3//MBD6//ZNF526//ADH4//CPEB2//UBXN2B//CREBL2//GSC//RBFOX3//PHF13//PPP4R2//RNF38//CREBRF//ESCO2//SPRED1//GADD45A//ZNF800//ARX//DUSP1//E2F5//ZNF367//EMX2//EN1//EP300//ESR1//ETS1//ETV5//MED19//CCNY//JAZF1//ATF5//ZFP30//CPEB3//DZIP1//FOXF1//FOXF2//WDFY3//PALLD//PDZRN3//MYT1L//CDK19//FLI1//PHF8//DCUN1D4//FLNA//SIN3B//FMR1//ADNP//ZFPM2//ZMYND8//CNOT6L//NR5A2//NUTM1//DNAJB5//ZNF521//RWDD3//PPP1R16B//TES//AGO1//RNF11//NSG1//FOXP1//SESN1//GLI3//ATAD2//GSTM3//GTF2E1//CNOT7//PURG//MDFIC//TNPO2//H2AFZ//HIVEP2//ANXA7//HMGB3//FOXA1//HSBP1//SPOPL//ID2//ZNF680//IGFBP3//IMPDH1//IRF1//AR//ISL1//JUN//TRIM23//KCNK2//KDR//ZNF662//USP27X//RGS7BP//MAF//MBNL1//MDM4//MEF2A//MEOX2//AFF1//MLLT6//ASNA1//HSBP1L1//MSN//MYB//MYBL1//MYCN//MYOG//NAP1L3//NCK1//NEUROD1//NEUROG1//NFIA//NFE2//NFIB//NFIC//NHLH2//NPAS2//NPAT//NR4A2//OTX2//PCBP2//ASCC1//MEMO1//CUTC//SEPSECS//PHF20L1//IRAK4//IER5//CDK17//COMMD10//YTHDF2//GSKIP//ARMCX3//KLF13//RASD1//CMPK1//ARID4B//PIK3CB//PKNOX1//BRWD1//ERRFI1//POU3F2//POU4F1//EGLN1//CCNJ//C2ORF42//PPP2CB//ZNF532//UBE2W//DOCK10//PRKAA1//BTBD7//TRERF1//FOXJ2//MAPK6//NXF2//SLC2A4RG//PSMA2//PAK6//CCNL1//PHTF2//CNOT6//TAOK1//RANBP10//ZBTB4//ZBTB26//MIER1//PURA//MID1IP1//RRAGD//RB1//HIVEP3//RBMS1//CCND1//RNF2//RORA//SARS//ATXN1//ATXN7//PRDM16//SRSF2//CPEB1//SH3BGRL//GMNC//S100PBP//NABP1//RFX7//SKP1//CDK15//SOX5//SS18//SUV39H1//BTF3//ZEB1//BTG1//TMBIM6//TFE3//KLF10//TSN//TSC1//TUB//NXF2B//TXK//UBE2I//UBE2V1//WT1//ZNF3//ZNF708//ZNF131//ZNF227//ZYX//PTP4A1//SLBP//ZNF655//ZSCAN5A//BHLHE41//SAP30L//NPEPL1//TBL1XR1//E2F8//SNIP1//CALM2//FOSL1//PTP4A2//FXR1//ANP32A//HIST2H2BE//KATNAL1//PPP1R1B//ARID5B//PCGF5//DGKE//ALG2//ZIC5//BHLHE40//CDC14A//KLF7//RTCA//DDX3Y//BSN//FUBP3//CCND2//CCNT2//ACBD5//RPS6KA5//MOB1B//FAM114A1//NREP//PLAA//FOXP2//QKI//VPS4B//ZNF516//PHF14//ST18//RASSF2//RTN3//CDS1//CRTAP//KIF1C//CKAP4//TMED1//B3GALNT2//TMEM64//SMIM14//TAPT1//SULF1//LCLAT1//SERP1//ZDHHC22//YIPF6//FIBIN//SHISA2//LRP6//FAM19A1//DNAJB9//IER3IP1//PLP2//ZDHHC7//STIM2//ELOVL5//RTN1//MSMO1//PLEKHF2//ELOVL7//KCNIP4//CALU//CAST//ZDHHC18//PPP1R15B//EI24//STX6//FUT9//ADCY3//STMN2//CLCN5//CPD//RAB12//RAB3GAP1//RAB18//CAMSAP2//MMD//FUT2//FUT5//ATP2C1//KLHL20//GOLGA6A//GLT6D1//KCNJ2//LDLR//WDR44//SLC30A6//PTHLH//SYT1//YES1//CSDE1//KBTBD8//GNPTG//SGCE//CCDC126//MMGT1//TJAP1//DYNC1LI2//FBXO28//PPP1R12A//CLIP1//TTK//WDR1//AKAP11//TMEM217//SMUG1//YPEL2//FBXW7//ZMAT3//RDH10//ACSL4//ABHD5//RAP1B//LMLN//DHRS3//NEDD1//CEP41//MID1//MZT1//KLHL42//SHCBP1//GPHN//WASF3//PTPN21//SLAIN1//FRMD6//DBN1//KLHL3//MYLIP//TMOD3//PFN2//ENAH//SNAP25//TMOD1//TNS1//CCDC6//C8ORF44-SGK3//TRAPPC8//SGK3//DLL1//NRBF2//NTF3//CHMP3//CHIC1//CXXC4//SOCS1//CFDP1//DCTN6//CENPO//DCTN5//NPNT//KCNE1//NAGA//DRAM1//ARRDC3//SNX2//CTSF//TBC1D12//ATG16L1//HECW2//SEPT9//CFL2//IQGAP2//CNN3//SAMD14//ARC//CD2AP//ARHGAP6//MARCKS//TMEM63B//TACC2//LASP1//KRT80//SESTD1//RIMS4//SLC26A7//CRHR1//CRKL//DGKH//LPAR1//RAB21//STEAP2//SNX5//SNX27//ADRB1//FKBP15//RAP1A//SLC31A2//RAPGEF2//TEX261//EDN1//KLK15//RAB9B//ITSN1//TNF//IGF1//ARHGAP1//DCP2//PATL1//TNRC6B//PAN3//MEX3B//TIA1//TDRD5//PEX5L//CLCN6//ZNRF2//GNAQ//OSTM1//OGN//SERPINB13//PRELP//SDC1//SNX19//CNIH1//MCFD2//SEC23A//C5AR1//SERPINI1//PSMD12//WNT3//WNT7A//TMEM201//SYT6//SEMA4C//VAMP1//C16ORF70//LCA5//FLII//PXK//RPP25//C2CD5//CAMSAP1//DPYSL2//REEP3//WDR47//KIF13A//KCNAB2//CCT6A//LRP8//HOMER1//CHRM2//NHLRC2//VEGFB//CHRNB4//ARMC8//BRI3//C6ORF120//RAP2C//ABHD17C//SIRPA//FAM83D//KCMF1//BDP1//SERINC3//HS3ST5//ARFIP1//ST8SIA5//GCNT4//CHST1//ST3GAL5//DSEL//HS3ST3A1//BBS9//GTF2H1//PIGP//PIGA//BAHD1//HIC1//SMARCD2//SYCE1//ROR1//CNEP1R1//LBR//NUP54//LEMD3//NAV3//KPNA6//SGK2//SIVA1//ARPP19//STRAP//KLF12//PDIK1L//SH3D19//ARID2//ELAVL2//ELL2//KDM2A//MORC3//NAALADL2//GABRA5//ANAPC15//HBP1//ZBTB11//TBK1//GTF2A1//NRBP1//NRG1//HOXA3//HOXD1//IRF2//NR3C2//KLF3//CTDSPL2//BNC2//INTS8//PRKACB//USP28//SRSF6//BMPR2//TGIF1//ADAM12//LCOR//CBFB//SKAP2//NMI//WDR20//RBM25//RBM41//MTX3//ARMCX6//BCL2A1//ABCB7//SLC25A34//MICU3//PRKAR2B//SLC25A28//COX7A2//NLN//THOP1//FASTK//SBF2//ARL6IP1//EOGT//KDELC1//GJC1//LPCAT3//SPTSSB//SPTSSA//SLC35D1//INSIG2//JKAMP//SEC61A2//HS3ST1//TNNI1//FAM118B//MED9//MED22//HIPK3//FAM76B//CBLL1//DAZAP2//ZPLD1//FZD6//ULK2//PRICKLE2//EDNRB//CABP7//CALN1//EPC2//ZMYND10//RAB28//EVC//DLG5//IMMP2L//KRTAP5-6//KRTAP2-4//EMC7//PSD// |
| GO:0005737 | cytoplasm | Cellular component | 661 | 11544 | 929 | 18698 | 1.15245660913045 | 4.40840600490171e-10 | 9.46705189552642e-08 | 9.35571841470786 | 0.711517761033369 | SPOCK1//EPS8//NRBP1//RHOB//RND3//MARCKS//TSC1//C2CD5//RIMS4//GABARAPL1//GRIK2//MAPK1//TRAK2//RAB21//SYBU//RASD1//DDX6//SEPT9//GLI3//ATG16L1//BSN//FMR1//FXR1//GOLGA6A//YIPF6//CNIH1//STX6//RTN3//SEC23A//FUT9//SERINC3//CLCN5//VTI1A//B3GALNT2//RAB12//HS3ST5//FUT5//GJA1//ATP2C1//ARFIP1//RAB30//ST8SIA5//TRIM23//GCNT4//NDFIP2//ZDHHC7//SLC30A6//PMEPA1//ACBD3//APOO//RIC3//GNPTG//CHST1//ST3GAL5//AP1S2//MCFD2//DSEL//MMGT1//HS3ST3A1//NCK1//PPP1R15B//GTPBP1//NEDD1//BBS9//CCNY//TRAPPC8//PLEKHF2//ULK2//PYURF//PIGP//PIGA//VPS37A//CHMP3//PNRC1//DCP2//PATL1//TNRC6B//PAN3//AGO1//CNOT7//YTHDF2//PSMA2//CPEB1//MEX3B//ARC//MCCC2//PPP1R12A//DYNC1LI2//KCNE1//LDLR//NAGA//NEU1//PON2//DRAM1//ARRDC3//RRAGD//SNX2//TNFAIP3//CTSF//CMC4//CCDC58//PPARGC1B//DYNLL2//PPTC7//PRSS35//ELK3//ACSL1//SIRT1//SLC44A1//CLIC4//TIMM9//GLRX//TCAIM//GRSF1//SLC25A6//COX8C//JARID2//BOLA3//MGST1//NKTR//OGG1//DCAF8//RAB8B//PMAIP1//MIEF1//THG1L//PPP2CA//PPP3R1//SMURF1//PTEN//MAVS//RDH14//RAF1//SGK1//SOX4//YWHAG//ZNF217//PRR5L//ABHD11//CASP2//CCM2//TP63//BAG5//RANBP9//DNAJB6//C8ORF44-SGK3//SGK2//HIPK3//DNAJA2//TACC2//DCTN6//PTGES3//IQGAP2//COPS8//RALBP1//CKAP4//RAPGEF4//STRAP//AKAP11//EGLN3//KLF12//ARAP2//CNN3//ADH4//EDARADD//UBXN2B//CRKL//KRT80//SYT6//FBXO41//SH3D19//ADSS//SPRED1//DENND1B//POLR3H//DPYSL2//TXLNA//DENND6A//PRSS55//EP300//EPS15//ESR1//RASGEF1A//JAZF1//ZNRF2//ATF5//DZIP1//RAB3GAP1//RAB18//WDFY3//PALLD//PDZRN3//CDK19//FLI1//FLII//FLNA//EFR3A//RGL1//OTUD3//CAMSAP2//DNAJC16//KPNA6//SGK3//CNOT6L//RICTOR//LCLAT1//GABRA5//CNEP1R1//DNAJB5//ANAPC15//APPL1//TES//STEAP2//KLHL3//GFPT1//SNX5//SERP1//SESN1//KLHL20//OSTM1//TBK1//MYLIP//GSTA2//GSTM3//GTF2A1//GTF2E1//PSAT1//HIC1//ANXA7//HPRT1//AGFG1//HSBP1//ID2//IMPDH1//IRF1//IRF2//AR//JUN//USP27X//ARHGAP1//ARHGAP6//MBNL1//MEF2A//MID1//NR3C2//KRTAP5-6//MZT1//HSBP1L1//MSN//MTRR//NPAS2//ARHGEF4//PAX5//PCBP2//MEMO1//CUTC//SEPSECS//ABHD5//IRAK4//RAB9B//PEX5L//CMPK1//SERPINB13//PIK3CA//PIK3CB//BRWD1//ERRFI1//RIN2//WDR44//EGLN1//PPARG//AFTPH//MTMR10//PXK//PPP2CB//SEC61A2//PPP2R5E//FBXW7//DOCK10//PRKAA1//PRKACB//ENAH//PRKAR2B//TRERF1//MAPK6//NXF2//GPCPD1//WDR45B//SPHK2//KCMF1//PSMD12//PTHLH//CNOT6//RIMKLB//KLHL42//TAOK1//RANBP10//ZBTB4//RAP2C//MID1IP1//RAP1A//RAP1B//RASA1//RBMS1//CCND1//CLIP1//SARS//ATXN1//ATXN7//PRDM16//SRSF2//ITSN1//S100PBP//NABP1//SKP1//CDK15//MTMR9//SNAP25//BNIP2//SOS2//HSPA13//VAMP1//SYT1//BTF3//ZEB1//TFAP2A//TFE3//TGFBR2//THOP1//TIA1//TMOD1//TNNI1//TSN//TUB//NXF2B//UBE2I//UBE2V1//VBP1//WT1//YES1//ZYX//CSDE1//SLBP//BAG6//CENPO//E2F8//SNIP1//CBLL1//UBA5//CCDC6//KCNIP4//FOSL1//CUL5//PTP4A2//SNX27//SBF2//CAST//HIST2H2BE//FBXO30//SYDE2//PPP1R1B//DCTN5//KBTBD8//TEAD2//PPFIA1//KCNAB2//DGKE//KRTAP2-4//ALG2//CDC14A//RUNX3//SOCS1//DDX3Y//PDE5A//SYNGAP1//SKAP2//CCND2//LMLN//SOCS3//CCNT2//CCT6A//NMI//LONRF1//MOB1B//FAM114A1//VAMP3//FBXO44//ARHGAP12//HOMER1//VPS4B//EI24//CEP41//CLOCK//PPM1F//SOCS5//RAPGEF2//RASSF2//SNX17//GIT2//G3BP2//WDR1//ACBD5//ACSL4//MTX3//ARMCX6//BCL2A1//ABCB7//SLC25A34//MICU3//SLC25A28//COX7A2//NLN//FASTK//CLCN6//SPPL3//MMD//GNAQ//STMN2//SLC26A7//CRHR1//DGKH//S1PR1//LPAR1//NSG1//KDR//TJAP1//ADRB1//FKBP15//RNF11//LRP6//PTP4A1//SLC9A6//SLC31A2//TBC1D12//NRBF2//CDS1//CRTAP//KIF1C//TMED1//TMEM64//SMIM14//TAPT1//SULF1//ZDHHC22//FIBIN//SHISA2//FAM19A1//DNAJB9//IER3IP1//PLP2//UGT2B28//PHTF2//STIM2//ELOVL5//RTN1//MSMO1//TMBIM6//ELOVL7//ANP32A//CALU//ZDHHC18//ARL6IP1//EOGT//IGFBP3//WNT3//WNT7A//KDELC1//GJC1//LPCAT3//RDH10//SPTSSB//SPTSSA//REEP3//SLC35D1//KCNK2//ASNA1//INSIG2//JKAMP//UGT2B7//DHRS3//KCNJ2//ADCY3//CPD//ESCO2//FUT2//GLT6D1//SGCE//CCDC126//OGN//PRELP//SDC1//HS3ST1//TEX261//C16ORF70//EDN1//KLK15//C5AR1//CD2AP//TMOD3//SS18//NTF3//KIF13A//CALM2//CFL2//LBR//NPNT//ZPLD1//SNX19//FZD6//GPRC5A//CHRM2//SEMA4C//DBN1//LASP1//IGF1//NHLRC2//VEGFB//ARMCX3//DLL1//CHIC1//CXXC4//LDLRAD4//CABP7//CALN1//TDRD5//ZMYND10//SERPINI1//BRI3//C6ORF120//CHRNB4//ARMC8//IMMP2L//PPP1R16B//NANOS1//INHBB//STK33//SLK//TNF//ABHD17C//SIRPA//EMC7//PSD//TOB1//MBNL2//GPHN//CEBPB//SIVA1//CELF1//ARPP19//WASF3//IFI44L//PTPN21//RCAN3//PCMTD1//RHEBL1//SLAIN1//FRMD6//CNGA3//LYPD6//CPEB2//RBFOX3//PPP4R2//CAMSAP1//PRUNE2//GADD45A//PRICKLE2//LCA5//DNMT3B//DTNA//DUSP1//E2F5//TMEM201//SAMD14//ETS1//EVC//CPEB3//WDR47//IQSEC2//SIN3B//ADNP//ZFPM2//PHLDA3//ZMYND8//FAM89B//NR5A2//NUTM1//BAMBI//RWDD3//RNF167//GLRB//MDFIC//TNPO2//NRG1//HMGB3//S100A7A//SPOPL//ISL1//RGS7BP//MAF//MEOX2//MET//NEUROD1//NFE2//NPAT//ROR1//NR4A2//PAIP2//IER5//CDK17//COMMD10//GULP1//GSKIP//ARID4B//PFN2//PKNOX1//ANKIB1//ZFAND6//POU4F1//ANO1//UBE2W//LRP2BP//SLC2A4RG//PAK6//HECW2//MIER1//PURA//RBM25//HIVEP3//SH3BGRL//SLC6A11//BMPR2//BOLL//STRN//MED22//BTG1//TGM3//TNS1//TTK//TXK//ZNF655//NPEPL1//FZD3//SHCBP1//VASH2//FAM83D//KATNAL1//BHLHE40//FUBP3//UBE3B//C5ORF30//DLG5//RPS6KA5//NREP//SLIT2//RAB28//PLAA//QKI//FEZ2//RALGPS1//DAZAP2//SLC23A2// |
| GO:0043226 | organelle | Cellular component | 751 | 13597 | 929 | 18698 | 1.11167101145357 | 2.3874078101889e-09 | 4.10156661790453e-07 | 8.62207338971249 | 0.808396124865447 | ADCY3//DYNLL2//LCA5//EVC//BBS9//GLI3//TUB//CEP41//GPHN//SEPT9//SLC9A6//ARAP2//SPPL3//VPS37A//VTI1A//DENND1B//POLR3H//DNMT3B//S1PR1//CC2D1B//DENND6A//EPS15//MYCBP2//APPL1//GJA1//RAB30//AGFG1//RHOB//RND3//NEU1//NOVA1//PAX5//RAB8B//UGT2B28//NDFIP2//PPARG//AFTPH//SYBU//SPHK2//PMEPA1//RBBP8//SLC14A1//SMARCA2//BNIP2//HSPA13//TFAP2A//TNFAIP3//UGT2B7//VBP1//LDLRAD4//BAG6//RIC3//CLIP4//UBA5//TEAD2//RUNX3//AP1S2//BTAF1//GPRC5A//VAMP3//CLOCK//SNX17//GIT2//EPS8//GLT6D1//MSN//SNAP25//CALM2//GPM6A//OGN//PRELP//CTSF//CNN3//SAMD14//CPEB3//ARC//FMR1//NPTN//GRIK2//GRM3//SEMA4C//PSD//ABHD17C//CPEB1//BMPR2//SPOCK1//STRN//LRP8//FXR1//KCNAB2//SYNGAP1//BSN//DLG5//HOMER1//CMC4//PYURF//CCDC58//PPARGC1B//PPTC7//DDX6//PRSS35//ELK3//ACSL1//SIRT1//SLC44A1//GABARAPL1//CLIC4//TIMM9//GLRX//TCAIM//GRSF1//SLC25A6//COX8C//JARID2//BOLA3//MGST1//NKTR//OGG1//DCAF8//PMAIP1//PON2//MIEF1//THG1L//PPP2CA//PPP3R1//MAPK1//SMURF1//PTEN//MAVS//RDH14//RAF1//MCCC2//SGK1//ACBD3//TRAK2//SOX4//YWHAG//ZNF217//APOO//PRR5L//ABHD11//CASP2//CCM2//TP63//BAG5//AKAP11//ACBD5//RANBP9//DNAJB6//TOB1//MBNL2//TSHZ1//ZBTB18//CEBPB//CELF1//PTGES3//ZMYND11//TCERG1//COPS8//PNRC1//RPP14//EGLN3//MBD6//ZNF526//ADH4//CPEB2//UBXN2B//CREBL2//GSC//RBFOX3//PHF13//PPP4R2//RNF38//CREBRF//ESCO2//SPRED1//GADD45A//ZNF800//ARX//DUSP1//E2F5//ZNF367//EMX2//EN1//EP300//ESR1//ETS1//ETV5//MED19//CCNY//JAZF1//ATF5//ZFP30//DZIP1//FOXF1//FOXF2//WDFY3//PALLD//PDZRN3//MYT1L//CDK19//FLI1//PHF8//DCUN1D4//FLNA//SIN3B//ADNP//ZFPM2//ZMYND8//CNOT6L//NR5A2//NUTM1//DNAJB5//ZNF521//RWDD3//PPP1R16B//TES//AGO1//RNF11//NSG1//FOXP1//SESN1//ATAD2//GSTM3//GTF2E1//CNOT7//PURG//MDFIC//TNPO2//H2AFZ//HIVEP2//ANXA7//HMGB3//FOXA1//HSBP1//SPOPL//ID2//ZNF680//IGFBP3//IMPDH1//IRF1//AR//ISL1//JUN//TRIM23//KCNK2//KDR//ZNF662//USP27X//RGS7BP//MAF//MBNL1//MDM4//MEF2A//MEOX2//AFF1//MLLT6//ASNA1//HSBP1L1//MYB//MYBL1//MYCN//MYOG//NAP1L3//NCK1//NEUROD1//NEUROG1//NFIA//NFE2//NFIB//NFIC//NHLH2//NPAS2//NPAT//NR4A2//OTX2//PCBP2//ASCC1//MEMO1//CUTC//SEPSECS//PHF20L1//IRAK4//IER5//CDK17//COMMD10//YTHDF2//GSKIP//ARMCX3//KLF13//RASD1//CMPK1//ARID4B//PIK3CB//PKNOX1//BRWD1//ERRFI1//POU3F2//POU4F1//EGLN1//CCNJ//C2ORF42//PPP2CB//ZNF532//UBE2W//DOCK10//PRKAA1//BTBD7//TRERF1//FOXJ2//MAPK6//NXF2//SLC2A4RG//PSMA2//PAK6//CCNL1//PHTF2//CNOT6//TAOK1//RANBP10//ZBTB4//ZBTB26//MIER1//PURA//MID1IP1//RRAGD//RB1//HIVEP3//RBMS1//CCND1//RNF2//RORA//SARS//ATXN1//ATXN7//PRDM16//SRSF2//SH3BGRL//GMNC//S100PBP//NABP1//RFX7//SKP1//CDK15//SOX5//SS18//SUV39H1//BTF3//ZEB1//BTG1//TMBIM6//TFE3//KLF10//TSN//TSC1//NXF2B//TXK//UBE2I//UBE2V1//WT1//ZNF3//ZNF708//ZNF131//ZNF227//ZYX//PTP4A1//SLBP//ZNF655//ZSCAN5A//BHLHE41//SAP30L//NPEPL1//TBL1XR1//E2F8//SNIP1//FOSL1//PTP4A2//ANP32A//HIST2H2BE//KATNAL1//PPP1R1B//ARID5B//PCGF5//DGKE//ALG2//ZIC5//BHLHE40//CDC14A//KLF7//RTCA//DDX3Y//FUBP3//CCND2//CCNT2//RPS6KA5//MOB1B//FAM114A1//NREP//PLAA//FOXP2//QKI//VPS4B//ZNF516//PHF14//ST18//RASSF2//RAB12//TBC1D12//NRBF2//ATG16L1//PLEKHF2//RTN3//CDS1//CRTAP//KIF1C//CKAP4//TMED1//B3GALNT2//TMEM64//SMIM14//TAPT1//SULF1//LCLAT1//SERP1//ZDHHC22//YIPF6//FIBIN//SHISA2//LRP6//FAM19A1//DNAJB9//IER3IP1//PLP2//ZDHHC7//STIM2//ELOVL5//RTN1//MSMO1//ELOVL7//KCNIP4//CALU//CAST//ZDHHC18//PPP1R15B//EI24//KCNJ2//STX6//FUT9//STMN2//CLCN5//CPD//RAB3GAP1//RAB18//CAMSAP2//MMD//FUT2//FUT5//ATP2C1//KLHL20//GOLGA6A//LDLR//WDR44//SLC30A6//PTHLH//SYT1//YES1//CSDE1//KBTBD8//GNPTG//SGCE//CCDC126//MMGT1//TJAP1//C8ORF44-SGK3//TRAPPC8//SGK3//DLL1//NTF3//CHMP3//CHIC1//CXXC4//SOCS1//DNAJA2//CFL2//IQGAP2//WASF3//COX7A2//CR2//CRKL//SIRPA//ADSS//DPYSL2//EFNA1//F3//ACSL4//RAB21//EFR3A//CD2AP//PHLDA3//NPNT//GFPT1//GNAQ//GSTA2//PSAT1//HPRT1//ITGB8//C6ORF120//ARHGAP1//LASP1//MARCKS//MEP1A//NAGA//PI15//SLC26A4//PFN2//SERPINI1//SERPINB13//UBL3//ARL15//ANO1//PRKACB//PRKAR2B//PSMD12//RAP2C//PTPRG//RAP1A//RAP1B//ROBO2//SDC1//BMP3//DLK2//SNX2//TGM3//WNT3//WNT7A//HDHD2//CCT6A//SLIT2//SLK//WDR1//DYNC1LI2//FBXO28//PPP1R12A//CLIP1//TTK//TMEM217//SMUG1//YPEL2//FBXW7//ZMAT3//RDH10//ABHD5//LMLN//DHRS3//NEDD1//MID1//MZT1//KLHL42//SHCBP1//PTPN21//SLAIN1//FRMD6//DBN1//KLHL3//MYLIP//TMOD3//ENAH//TMOD1//TNS1//CCDC6//CFDP1//DCTN6//CENPO//DCTN5//ARHGAP6//TMEM63B//TACC2//DCP2//PATL1//KRT80//SESTD1//HECW2//TIA1//SLC26A7//CRHR1//DGKH//LPAR1//STEAP2//SNX5//ARRDC3//SNX27//EDN1//RAB9B//ITSN1//RAPGEF2//KCNE1//DRAM1//ADRB1//FKBP15//SLC31A2//TNF//PEX5L//TEX261//IGF1//RIMS4//KLK15//TNRC6B//PAN3//MEX3B//TDRD5//CLCN6//ZNRF2//OSTM1//SNX19//CNIH1//MCFD2//SEC23A//C5AR1//TMEM201//SYT6//VAMP1//C16ORF70//FLII//PXK//RPP25//C2CD5//CAMSAP1//REEP3//WDR47//KIF13A//CHRM2//NHLRC2//VEGFB//CHRNB4//ARMC8//BRI3//FAM83D//KCMF1//BDP1//SERINC3//HS3ST5//ARFIP1//ST8SIA5//GCNT4//CHST1//ST3GAL5//DSEL//HS3ST3A1//GTF2H1//PIGP//PIGA//BAHD1//HIC1//SMARCD2//SYCE1//ROR1//CNEP1R1//LBR//NUP54//LEMD3//NAV3//KPNA6//SGK2//SIVA1//ARPP19//STRAP//KLF12//PDIK1L//SH3D19//ARID2//ELAVL2//ELL2//KDM2A//MORC3//NAALADL2//GABRA5//ANAPC15//HBP1//ZBTB11//TBK1//GTF2A1//NRBP1//NRG1//HOXA3//HOXD1//IRF2//NR3C2//KLF3//CTDSPL2//BNC2//INTS8//USP28//SRSF6//TGIF1//ADAM12//LCOR//CBFB//SKAP2//NMI//WDR20//RBM25//RBM41//MTX3//ARMCX6//BCL2A1//ABCB7//SLC25A34//MICU3//SLC25A28//NLN//THOP1//FASTK//SBF2//ARL6IP1//EOGT//KDELC1//GJC1//LPCAT3//SPTSSB//SPTSSA//SLC35D1//INSIG2//JKAMP//SEC61A2//HS3ST1//TNNI1//FAM118B//MED9//MED22//HIPK3//FAM76B//CBLL1//DAZAP2//ZPLD1//FZD6//ULK2//PRICKLE2//EDNRB//CABP7//CALN1//EPC2//ZMYND10//RAB28//TMEM17//C5ORF30//CNGA3//IMMP2L//KRTAP5-6//KRTAP2-4//EMC7//GSG1L// |
| GO:0005623 | cell | Cellular component | 886 | 16915 | 929 | 18698 | 1.05424405634835 | 8.50465729629319e-09 | 1.21758343625264e-06 | 8.07034318203113 | 0.953713670613563 | RANBP9//GJC1//STX6//GPHN//RTN3//NMUR1//SLC9A6//TACC2//IQGAP2//ADCY3//SERINC3//CKAP4//TMED1//RAPGEF4//AKAP11//CHRM2//PALM2//CHRNB4//SLC26A7//CLCN5//FAT3//SPPL3//FRMD6//CNTFR//LYPD6//RAET1E//CPD//CR2//CRHR1//DYNLL2//SIRPA//SYT6//SH3D19//ADRB1//ADSS//DGKH//SPRED1//DBN1//CABP7//ABHD3//DPYSL2//S1PR1//LPAR1//EDNRB//EFNA1//EFNB2//ARID2//EPS8//EPS15//ESR1//ETV5//F3//ACSL1//UNC5B//CCNY//ZNRF2//SLITRK3//RAB18//WDFY3//PALLD//FLNA//EFR3A//SULF1//ARC//KCNH4//SLC44A1//CD2AP//PHLDA3//ALPI//SLC41A1//GABRA5//BRI3//BAMBI//CLIC4//LRIG1//PPP1R16B//TES//STEAP2//GJA1//TNFRSF21//GLRB//GNAQ//GPM6A//CDH20//ZDHHC22//OR4N4//DLL1//GRK6//GRIK2//MYLIP//GRM3//ANXA7//IGSF3//IGF1//AR//ITGB8//KCNA4//KCNB1//KCNE1//KCNJ2//KCNJ3//KCNJ10//KCNK2//KDR//RHOB//RND3//LDLR//RGS7BP//LRP6//MARCKS//MET//MGST1//MSN//NCK1//NEU1//NKTR//ROR1//IRAK4//RAB9B//TNFRSF12A//CHMP3//RASD1//SLC26A4//RAB8B//PIK3CA//PIK3CB//CHIC1//PLP2//UBL3//KCNK10//PON2//PXK//ANO1//XKR8//SLC6A15//PPP2CA//TMEM63B//PRKACB//ENAH//PRKAR2B//MAPK1//NPDC1//PMEPA1//SMURF1//PTEN//SLC24A3//NLN//ARRDC3//STIM2//RAP2C//RAF1//RAP1A//RAP1B//ENPP5//ROBO2//MSMO1//SCN1B//SDC1//ZMAT3//SGK1//ITSN1//SLC6A1//SLC6A6//SLC6A8//SLC6A11//SLC10A2//SLC14A1//SLC16A2//BMPR2//TRAK2//SNAP25//HSPA13//SYT1//TGFBR2//TNF//TRPC3//TSC1//TUB//C5AR1//FAM155A//WNT3//WNT7A//YES1//ZYX//LRP8//CSDE1//ADIPOR2//FZD3//GLRA3//KCNIP4//ADAM12//CALM2//PTP4A2//GDF5//FZD6//CALN1//RECK//KCNAB2//DGKE//OR6A2//SGCE//SKAP2//CCNT2//GPRC5A//DIRAS3//PLXNA4//CD3E//DLG5//CD8A//GPR55//MMGT1//VAMP3//SLIT2//RAB28//TJAP1//NRXN3//HOMER1//RALGPS1//RAPGEF2//RASSF2//C2CD5//WDR1//SLC23A2//SIN3B//TRERF1//ZNF217//SAP30L//TBL1XR1//EP300//BDP1//GOLGA6A//YIPF6//CNIH1//SEC23A//FUT9//VTI1A//B3GALNT2//RAB12//HS3ST5//FUT5//ATP2C1//ARFIP1//RAB30//ST8SIA5//TRIM23//GCNT4//NDFIP2//ZDHHC7//SYBU//SLC30A6//ACBD3//APOO//RIC3//GNPTG//CHST1//ST3GAL5//AP1S2//MCFD2//DSEL//HS3ST3A1//DCUN1D4//FBXO8//RNF11//ANKIB1//RNF2//UBE2V1//CBLL1//IER5//PPP2CB//PPP2R5E//STRN//PPP1R15B//GTPBP1//ZBTB18//JUN//SUV39H1//NEDD1//BBS9//SGK2//ADCYAP1//EDN2//EREG//GABARAPL1//ANAPC15//ARL5A//IFNG//IL2//HPCAL4//ARL15//PRKAA1//CCND1//CCL2//STK33//LONRF3//LONRF1//CCNL1//CCND2//TRAPPC8//PLEKHF2//ULK2//ATG16L1//POLR3H//GTF2H1//PSMA2//PSMD12//PYURF//PIGP//PIGA//FMR1//DYNC1LI2//FBXO28//PPP1R12A//CLIP1//TTK//CFDP1//DCTN6//CENPO//DCTN5//CEBPB//PTGES3//PURA//NABP1//ESCO2//BAHD1//HIC1//MAF//MYCN//RB1//H2AFZ//HIST2H2BE//SIRT1//IRF1//MEF2A//POU4F1//SMARCA2//SMARCD2//TP63//RUNX3//DDX6//UBE2I//SYCE1//PCGF5//VPS37A//TMEM201//KATNAL1//CDC14A//VPS4B//MZT1//PNRC1//DCP2//PATL1//TNRC6B//PAN3//AGO1//CNOT7//YTHDF2//CPEB1//MEX3B//MBD6//E2F5//TMEM217//JAZF1//SESN1//FOXA1//NFIB//NFIC//PAX5//FOXJ2//PAK6//SEPT9//SNX5//RASA1//SNX27//ARHGAP12//MCCC2//GLI3//NAGA//DRAM1//RRAGD//SNX2//TNFAIP3//CTSF//CMC4//CCDC58//PPARGC1B//PPTC7//PRSS35//ELK3//TIMM9//GLRX//TCAIM//GRSF1//SLC25A6//COX8C//JARID2//BOLA3//OGG1//DCAF8//PMAIP1//MIEF1//THG1L//PPP3R1//MAVS//RDH14//SOX4//YWHAG//PRR5L//ABHD11//CASP2//CCM2//BAG5//DNAJB6//C8ORF44-SGK3//HIPK3//DNAJA2//COPS8//RALBP1//STRAP//EGLN3//KLF12//ARAP2//CNN3//ADH4//EDARADD//UBXN2B//CRKL//KRT80//FBXO41//DENND1B//TXLNA//DENND6A//PRSS55//RASGEF1A//ATF5//DZIP1//RAB3GAP1//RAB21//PDZRN3//CDK19//FLI1//FLII//RGL1//OTUD3//CAMSAP2//DNAJC16//KPNA6//SGK3//CNOT6L//RICTOR//LCLAT1//CNEP1R1//DNAJB5//APPL1//KLHL3//GFPT1//SERP1//KLHL20//OSTM1//TBK1//GSTA2//GSTM3//GTF2A1//GTF2E1//NRBP1//PSAT1//HPRT1//AGFG1//HSBP1//ID2//IMPDH1//IRF2//USP27X//ARHGAP1//ARHGAP6//MBNL1//MID1//NR3C2//KRTAP5-6//HSBP1L1//MTRR//NPAS2//ARHGEF4//PCBP2//MEMO1//CUTC//SEPSECS//ABHD5//PEX5L//CMPK1//SERPINB13//BRWD1//ERRFI1//RIN2//WDR44//EGLN1//PPARG//AFTPH//MTMR10//SEC61A2//FBXW7//DOCK10//MAPK6//NXF2//GPCPD1//WDR45B//SPHK2//KCMF1//PTHLH//CNOT6//RIMKLB//KLHL42//TAOK1//RANBP10//ZBTB4//MID1IP1//RBMS1//SARS//ATXN1//ATXN7//PRDM16//SRSF2//S100PBP//SKP1//CDK15//MTMR9//BNIP2//SOS2//VAMP1//BTF3//ZEB1//TFAP2A//TFE3//THOP1//TIA1//TMOD1//TNNI1//TSN//NXF2B//VBP1//WT1//SLBP//BAG6//E2F8//SNIP1//UBA5//CCDC6//FOSL1//CUL5//FXR1//SBF2//CAST//FBXO30//SYDE2//PPP1R1B//KBTBD8//TEAD2//PPFIA1//KRTAP2-4//ALG2//SOCS1//DDX3Y//PDE5A//SYNGAP1//LMLN//SOCS3//CCT6A//NMI//MOB1B//FAM114A1//FBXO44//EI24//CEP41//CLOCK//PPM1F//SOCS5//SNX17//GIT2//G3BP2//ACBD5//CNGA3//SLC31A2//APCDD1//MMD//LRP12//NRG1//MEP1A//TSPAN11//PCDHB10//PCDH10//PTPRG//SLC22A23//TMBIM6//ACVR1//CD69//TOB1//MBNL2//TSHZ1//CELF1//ZMYND11//TCERG1//RPP14//ZNF526//CPEB2//CREBL2//GSC//RBFOX3//PHF13//PPP4R2//RNF38//CREBRF//GADD45A//ZNF800//ARX//DNMT3B//DUSP1//ZNF367//CC2D1B//EMX2//EN1//ETS1//MED19//ZFP30//CPEB3//FOXF1//FOXF2//MYT1L//MYCBP2//PHF8//ADNP//ZFPM2//ZMYND8//NR5A2//NUTM1//ZNF521//RWDD3//NSG1//FOXP1//ATAD2//PURG//MDFIC//TNPO2//HIVEP2//HMGB3//SPOPL//ZNF680//IGFBP3//ISL1//ZNF662//MDM4//MEOX2//AFF1//MLLT6//ASNA1//MYB//MYBL1//MYOG//NAP1L3//NEUROD1//NEUROG1//NFIA//NFE2//NHLH2//NOVA1//NPAT//NR4A2//OTX2//ASCC1//PHF20L1//CDK17//COMMD10//GSKIP//ARMCX3//KLF13//ARID4B//PKNOX1//POU3F2//CCNJ//C2ORF42//ZNF532//UBE2W//BTBD7//SLC2A4RG//PHTF2//ZBTB26//MIER1//HIVEP3//RBBP8//RORA//SH3BGRL//GMNC//RFX7//SOX5//SS18//BTG1//KLF10//TXK//ZNF3//ZNF708//ZNF131//ZNF227//PTP4A1//ZNF655//ZSCAN5A//BHLHE41//NPEPL1//ANP32A//ARID5B//ZIC5//BHLHE40//KLF7//RTCA//BSN//FUBP3//RPS6KA5//NREP//PLAA//FOXP2//QKI//ZNF516//PHF14//ST18//LBR//NUP54//LEMD3//UGT2B28//NAV3//SIVA1//ARPP19//WASF3//IFI44L//STMN2//PTPN21//RCAN3//PCMTD1//RHEBL1//SLAIN1//RDH10//CAMSAP1//PRUNE2//PRICKLE2//LCA5//DTNA//EDN1//SAMD14//TAPT1//EVC//ACSL4//WDR47//IQSEC2//FAM89B//RNF167//NRBF2//S100A7A//NANOS1//LASP1//SNX19//DNAJB9//PAIP2//ZMYND10//GULP1//PFN2//ZFAND6//LRP2BP//HECW2//RBM25//BOLL//SPOCK1//MED22//TGM3//TNS1//SHCBP1//VASH2//CXXC4//FAM83D//UBE3B//C5ORF30//FEZ2//SLK//DAZAP2//PDIK1L//ELAVL2//ELL2//KDM2A//MORC3//SMUG1//NAALADL2//HBP1//ZBTB11//HOXA3//HOXD1//KLF3//CTDSPL2//BNC2//RPP25//INTS8//USP28//SRSF6//TGIF1//LDLRAD4//LCOR//CBFB//BTAF1//WDR20//RBM41//YPEL2//MTX3//ARMCX6//BCL2A1//ABCB7//SLC25A34//MICU3//SLC25A28//COX7A2//FASTK//CLCN6//FKBP15//TBC1D12//CDS1//CRTAP//KIF1C//TMEM64//SMIM14//FIBIN//SHISA2//FAM19A1//IER3IP1//ELOVL5//RTN1//ELOVL7//CALU//ZDHHC18//ARL6IP1//EOGT//KDELC1//LPCAT3//SPTSSB//SPTSSA//REEP3//SLC35D1//INSIG2//JKAMP//UGT2B7//DHRS3//CLIP4//FUT2//GLT6D1//CCDC126//OGN//PRELP//HS3ST1//TEX261//C16ORF70//KLK15//KIF13A//TMOD3//NTF3//CXCL9//NPTN//SEMA4C//PSD//ABHD17C//FAM118B//CFL2//MED9//FAM76B//TMEM185A//NPNT//ZPLD1//NHLRC2//VEGFB//CACUL1//ZER1//EPC2//TDRD5//ARMC8//SERPINI1//C6ORF120//TMEM17//IMMP2L//RIMS4//SESTD1//INHBB//EMC7//GSG1L//CCL8//CCL21// |
| GO:0044464 | cell part | Cellular component | 884 | 16886 | 929 | 18698 | 1.05367074360618 | 1.78456772997247e-08 | 2.18991954292336e-06 | 7.74846696455051 | 0.951560818083961 | RANBP9//GJC1//STX6//GPHN//RTN3//NMUR1//SLC9A6//TACC2//IQGAP2//ADCY3//SERINC3//CKAP4//TMED1//RAPGEF4//AKAP11//CHRM2//PALM2//CHRNB4//SLC26A7//CLCN5//FAT3//SPPL3//FRMD6//CNTFR//LYPD6//RAET1E//CPD//CR2//CRHR1//DYNLL2//SIRPA//SYT6//SH3D19//ADRB1//ADSS//DGKH//SPRED1//DBN1//CABP7//ABHD3//DPYSL2//S1PR1//LPAR1//EDNRB//EFNA1//EFNB2//ARID2//EPS8//EPS15//ESR1//ETV5//F3//ACSL1//UNC5B//CCNY//ZNRF2//SLITRK3//RAB18//WDFY3//PALLD//FLNA//EFR3A//SULF1//ARC//KCNH4//SLC44A1//CD2AP//PHLDA3//ALPI//SLC41A1//GABRA5//BRI3//BAMBI//CLIC4//LRIG1//PPP1R16B//TES//STEAP2//GJA1//TNFRSF21//GLRB//GNAQ//GPM6A//CDH20//ZDHHC22//OR4N4//DLL1//GRK6//GRIK2//MYLIP//GRM3//ANXA7//IGSF3//IGF1//AR//ITGB8//KCNA4//KCNB1//KCNE1//KCNJ2//KCNJ3//KCNJ10//KCNK2//KDR//RHOB//RND3//LDLR//RGS7BP//LRP6//MARCKS//MET//MGST1//MSN//NCK1//NEU1//NKTR//ROR1//IRAK4//RAB9B//TNFRSF12A//CHMP3//RASD1//SLC26A4//RAB8B//PIK3CA//PIK3CB//CHIC1//PLP2//UBL3//KCNK10//PON2//PXK//ANO1//XKR8//SLC6A15//PPP2CA//TMEM63B//PRKACB//ENAH//PRKAR2B//MAPK1//NPDC1//PMEPA1//SMURF1//PTEN//SLC24A3//NLN//ARRDC3//STIM2//RAP2C//RAF1//RAP1A//RAP1B//ENPP5//ROBO2//MSMO1//SCN1B//SDC1//ZMAT3//SGK1//ITSN1//SLC6A1//SLC6A6//SLC6A8//SLC6A11//SLC10A2//SLC14A1//SLC16A2//BMPR2//TRAK2//SNAP25//HSPA13//SYT1//TGFBR2//TNF//TRPC3//TSC1//TUB//C5AR1//FAM155A//WNT3//WNT7A//YES1//ZYX//LRP8//CSDE1//ADIPOR2//FZD3//GLRA3//KCNIP4//ADAM12//CALM2//PTP4A2//GDF5//FZD6//CALN1//RECK//KCNAB2//DGKE//OR6A2//SGCE//SKAP2//CCNT2//GPRC5A//DIRAS3//PLXNA4//CD3E//DLG5//CD8A//GPR55//MMGT1//VAMP3//SLIT2//RAB28//TJAP1//NRXN3//HOMER1//RALGPS1//RAPGEF2//RASSF2//C2CD5//WDR1//SLC23A2//SGK2//ADCYAP1//EDN2//EREG//GABARAPL1//ANAPC15//ARL5A//IFNG//IL2//HPCAL4//ARL15//PRKAA1//CCND1//CCL2//STK33//LONRF3//LONRF1//LRP12//AP1S2//NPTN//VAMP1//TNS1//BSN//RHEBL1//RAB12//RAB21//RNF167//RAB30//NRBP1//RTN1//KDELC1//KIF13A//SHCBP1//SNX5//SNX2//SNX27//SLK//SEPT9//FMR1//AGFG1//YWHAG//RDH10//NRG1//NEDD1//ACVR1//EDN1//INHBB//VPS4B//RAB3GAP1//DTNA//PPP3R1//IER5//PPP2CB//PPP2R5E//STRN//PPP1R15B//PPP4R2//CNEP1R1//PPP1R12A//STMN2//FKBP15//OTX2//MZT1//PSD//CNN3//VTI1A//ACSL4//ADNP//SERPINI1//PURA//ELOVL5//RIC3//FXR1//PPP1R1B//DCUN1D4//FBXO8//RNF11//ANKIB1//RNF2//UBE2V1//CBLL1//CCNL1//CCND2//PSMA2//PSMD12//TOB1//MBNL2//CEBPB//SIVA1//CELF1//PTGES3//ARPP19//WASF3//IFI44L//PTPN21//RCAN3//STRAP//EGLN3//PCMTD1//ARAP2//SLAIN1//CNGA3//CPEB2//RBFOX3//CAMSAP1//PRUNE2//GADD45A//DDX6//PRICKLE2//DCP2//LCA5//DNMT3B//DUSP1//E2F5//TMEM201//TXLNA//SAMD14//DENND6A//TAPT1//ETS1//EVC//CPEB3//DZIP1//WDR47//IQSEC2//OTUD3//SIN3B//SIRT1//ZFPM2//ZMYND8//FAM89B//CNOT6L//NR5A2//NUTM1//RWDD3//APPL1//AGO1//NSG1//ARFIP1//SESN1//KLHL20//GLI3//TBK1//GRSF1//GSTM3//CNOT7//PSAT1//MDFIC//NRBF2//TNPO2//HMGB3//HPRT1//HSBP1//S100A7A//SPOPL//ID2//NANOS1//IMPDH1//IRF1//ISL1//ARHGAP1//LASP1//ARHGAP6//SNX19//MAF//MBNL1//DNAJB9//MEOX2//ASNA1//HSBP1L1//NAGA//NEUROD1//NFE2//NPAT//NR4A2//DCAF8//PCBP2//MEMO1//CUTC//SEPSECS//PAIP2//CDK17//ZMYND10//COMMD10//GULP1//GSKIP//CMPK1//ARID4B//PFN2//SERPINB13//PKNOX1//ERRFI1//ZFAND6//POU4F1//EGLN1//NDFIP2//AFTPH//MTMR10//UBE2W//FBXW7//DRAM1//DOCK10//LRP2BP//MAPK6//NXF2//SLC2A4RG//PAK6//PTHLH//RIMKLB//HECW2//TAOK1//MIER1//RBM25//RRAGD//RASA1//HIVEP3//SARS//ATXN1//CPEB1//SH3BGRL//SKP1//CDK15//MTMR9//BOLL//BNIP2//SOX4//SPOCK1//MED22//BTG1//TMBIM6//TFE3//TGM3//TIA1//TNFAIP3//TSN//TTK//NXF2B//TXK//UBE2I//VBP1//WT1//PTP4A1//SLBP//ZNF655//BAG6//NPEPL1//VASH2//UBA5//CXXC4//ANP32A//FAM83D//CASP2//CCM2//KATNAL1//SYDE2//PPFIA1//ALG2//BHLHE40//CDC14A//TP63//RUNX3//SOCS1//SYNGAP1//FUBP3//UBE3B//SOCS3//C5ORF30//CCT6A//NMI//RPS6KA5//MOB1B//NREP//PLAA//QKI//FEZ2//SOCS5//DAZAP2//GIT2//G3BP2//GSC//EP300//ATF5//FOXF1//FOXF2//JUN//MEF2A//MYOG//NHLH2//NPAS2//ASCC1//POU3F2//TRERF1//RB1//ZEB1//E2F8//TEAD2//CLOCK//BAG5//TRAPPC8//PATL1//CNOT6//ARMC8//ARHGAP12//CXCL9//CD69//RNF38//NTF3//SEMA4C//ABHD17C//KIF1C//FOSL1//C16ORF70//DHRS3//RIMS4//NEUROG1//SMARCA2//SS18//FLII//FBXO44//CACUL1//CUL5//ZER1//KLHL3//KLHL42//KBTBD8//GTPBP1//PPARG//RORA//PRDM16//TMEM185A//NFIB//LEMD3//LBR//ABCB7//TIMM9//SLC25A34//MICU3//SLC25A6//SLC25A28//POLR3H//SLC31A2//APCDD1//MMD//MEP1A//TSPAN11//PCDHB10//PCDH10//PTPRG//SLC22A23//ST3GAL5//ARHGEF4//NUP54//CLIP1//UGT2B28//NAV3//MTX3//PMAIP1//ARMCX6//MIEF1//MAVS//BCL2A1//SYBU//JARID2//RICTOR//PRR5L//BBS9//CEP41//DYNC1LI2//FBXO28//CMC4//PYURF//CCDC58//PPARGC1B//PPTC7//PRSS35//ELK3//GLRX//TCAIM//COX8C//BOLA3//OGG1//THG1L//RDH14//MCCC2//ACBD3//ZNF217//APOO//ABHD11//SERP1//DNAJB6//TSHZ1//ZBTB18//ZMYND11//TCERG1//COPS8//PNRC1//RPP14//MBD6//ZNF526//ADH4//UBXN2B//CREBL2//PHF13//CREBRF//ESCO2//ZNF800//ARX//ZNF367//CC2D1B//EMX2//EN1//MED19//JAZF1//ZFP30//PDZRN3//MYT1L//MYCBP2//CDK19//FLI1//PHF8//DNAJB5//ZNF521//FOXP1//ATAD2//GTF2E1//PURG//H2AFZ//HIVEP2//FOXA1//ZNF680//IGFBP3//TRIM23//ZNF662//USP27X//MDM4//AFF1//MLLT6//MYB//MYBL1//MYCN//NAP1L3//NFIA//NFIC//NOVA1//PHF20L1//YTHDF2//ARMCX3//KLF13//BRWD1//CCNJ//C2ORF42//ZNF532//BTBD7//FOXJ2//PHTF2//RANBP10//ZBTB4//ZBTB26//MID1IP1//RBBP8//RBMS1//ATXN7//SRSF2//GMNC//S100PBP//NABP1//RFX7//SOX5//SUV39H1//BTF3//TFAP2A//KLF10//ZNF3//ZNF708//ZNF131//ZNF227//ZSCAN5A//BHLHE41//SAP30L//TBL1XR1//SNIP1//HIST2H2BE//ARID5B//PCGF5//ZIC5//KLF7//RTCA//DDX3Y//ACBD5//FAM114A1//FOXP2//ZNF516//PHF14//ST18//TMEM217//SMUG1//YPEL2//CDS1//CRTAP//B3GALNT2//TMEM64//SMIM14//LCLAT1//YIPF6//FIBIN//SHISA2//FAM19A1//IER3IP1//ZDHHC7//PLEKHF2//ELOVL7//CALU//CAST//ZDHHC18//EI24//VPS37A//DENND1B//PAX5//SPHK2//UGT2B7//LDLRAD4//CLIP4//BTAF1//SNX17//FUT9//CAMSAP2//FUT2//FUT5//ATP2C1//GOLGA6A//GLT6D1//WDR44//SLC30A6//GNPTG//CCDC126//ABHD5//LMLN//MID1//TMOD3//TMOD1//CCDC6//C8ORF44-SGK3//SGK3//ULK2//ATG16L1//HIPK3//DNAJA2//SEC23A//DCTN6//RALBP1//KLF12//EDARADD//CRKL//KRT80//FBXO41//PRSS55//RASGEF1A//TNRC6B//RGL1//DNAJC16//KPNA6//PAN3//GFPT1//OSTM1//GSTA2//GTF2A1//HIC1//IRF2//NR3C2//KRTAP5-6//MTRR//PEX5L//RIN2//SEC61A2//GPCPD1//WDR45B//KCMF1//SOS2//THOP1//TNNI1//CENPO//SBF2//FBXO30//MEX3B//DCTN5//KRTAP2-4//PDE5A//PPM1F//FASTK//EOGT//OGN//PRELP//HS3ST1//CNIH1//MCFD2//HS3ST5//ST8SIA5//GCNT4//CHST1//DSEL//HS3ST3A1//PIGP//PIGA//TBC1D12//LPCAT3//SPTSSB//SPTSSA//REEP3//SLC35D1//ARL6IP1//INSIG2//JKAMP//CFL2//NPNT//ZPLD1//TDRD5//EMC7//GTF2H1//BAHD1//PDIK1L//ELAVL2//ELL2//KDM2A//MORC3//NAALADL2//HBP1//ZBTB11//HOXA3//HOXD1//KLF3//CTDSPL2//BNC2//RPP25//INTS8//BDP1//USP28//SRSF6//SMARCD2//TGIF1//LCOR//CBFB//WDR20//C6ORF120//CTSF//GSG1L//TMEM17//EPC2//CFDP1//RBM41//CLCN6//TEX261//SESTD1//KLK15//NHLRC2//VEGFB//SYCE1//COX7A2//MED9//FAM118B//FAM76B//IMMP2L// |
| GO:0005829 | cytosol | Cellular component | 322 | 5022 | 929 | 18698 | 1.29050177068048 | 4.76664441976149e-08 | 4.62128122588613e-06 | 7.32178724419111 | 0.346609257265877 | CCT6A//ADCY3//SNAP25//TSC1//BAG6//RANBP9//DNAJB6//C8ORF44-SGK3//SGK2//HIPK3//STX6//DNAJA2//SEC23A//TACC2//DCTN6//PTGES3//IQGAP2//COPS8//RALBP1//CKAP4//RAPGEF4//STRAP//AKAP11//EGLN3//KLF12//ARAP2//CLCN5//NEDD1//CNN3//ADH4//EDARADD//VPS37A//UBXN2B//CRKL//DYNLL2//VTI1A//KRT80//SYT6//FBXO41//SH3D19//ADSS//SPRED1//DENND1B//DDX6//DCP2//POLR3H//DYNC1LI2//DPYSL2//TXLNA//RAB12//DENND6A//PRSS55//EP300//EPS15//ESR1//PATL1//RASGEF1A//JAZF1//ZNRF2//ATF5//DZIP1//TRAPPC8//RAB3GAP1//RAB18//WDFY3//RAB21//PALLD//PDZRN3//CDK19//TNRC6B//FLI1//FLII//FLNA//EFR3A//RGL1//OTUD3//CAMSAP2//FMR1//DNAJC16//SIRT1//KPNA6//SGK3//GABARAPL1//CNOT6L//RICTOR//LCLAT1//GABRA5//CNEP1R1//PAN3//DNAJB5//ANAPC15//CLIC4//APPL1//TES//STEAP2//KLHL3//AGO1//GFPT1//GJA1//SNX5//SERP1//ARFIP1//BBS9//SESN1//KLHL20//GLI3//GLRX//OSTM1//TBK1//MYLIP//GSTA2//GSTM3//GTF2A1//GTF2E1//CNOT7//NRBP1//PSAT1//HIC1//ANXA7//HPRT1//AGFG1//HSBP1//ID2//IMPDH1//IRF1//IRF2//AR//JUN//RHOB//USP27X//ARHGAP1//ARHGAP6//MBNL1//MEF2A//MID1//NR3C2//KRTAP5-6//MZT1//HSBP1L1//MSN//MTRR//PPP1R12A//NCK1//NKTR//NPAS2//ARHGEF4//DCAF8//PAX5//PCBP2//MEMO1//CUTC//SEPSECS//ABHD5//IRAK4//RAB9B//YTHDF2//PEX5L//CHMP3//CMPK1//SERPINB13//PIK3CA//PIK3CB//PMAIP1//BRWD1//ERRFI1//RIN2//WDR44//EGLN1//PPARG//AFTPH//MTMR10//PXK//THG1L//ATG16L1//PPP2CA//PPP2CB//SEC61A2//PPP2R5E//FBXW7//PPP3R1//DOCK10//PRKAA1//PRKACB//ENAH//PRKAR2B//TRERF1//MAPK1//MAPK6//NXF2//GPCPD1//WDR45B//PSMA2//SPHK2//KCMF1//SMURF1//PSMD12//PTEN//PTHLH//CNOT6//RIMKLB//KLHL42//TAOK1//RANBP10//ZBTB4//RAP2C//MID1IP1//RRAGD//RAF1//RAP1A//RAP1B//RASA1//RBMS1//CCND1//CLIP1//SARS//ATXN1//ATXN7//PRDM16//MCCC2//SRSF2//SGK1//CPEB1//ITSN1//S100PBP//NABP1//SKP1//CDK15//MTMR9//BNIP2//SNX2//SOS2//HSPA13//VAMP1//SYT1//BTF3//ZEB1//TFAP2A//TFE3//TGFBR2//THOP1//TIA1//TMOD1//TNFAIP3//TNNI1//TSN//TUB//NXF2B//UBE2I//UBE2V1//VBP1//WT1//YES1//YWHAG//ZYX//CSDE1//SLBP//APOO//CENPO//E2F8//SNIP1//CBLL1//UBA5//CCDC6//KCNIP4//FOSL1//CUL5//PTP4A2//FXR1//SNX27//SBF2//CAST//HIST2H2BE//CASP2//FBXO30//SYDE2//PPP1R1B//MEX3B//DCTN5//KBTBD8//TEAD2//PPFIA1//KCNAB2//DGKE//KRTAP2-4//ALG2//CDC14A//TP63//RUNX3//SOCS1//DDX3Y//PDE5A//SYNGAP1//AP1S2//SKAP2//CCND2//LMLN//SOCS3//CCNT2//NMI//LONRF1//MOB1B//FAM114A1//VAMP3//FBXO44//ARHGAP12//HOMER1//VPS4B//BAG5//EI24//GTPBP1//CEP41//CLOCK//PPM1F//SOCS5//RAPGEF2//ULK2//RASSF2//SNX17//GIT2//C2CD5//G3BP2//WDR1// |
| GO:0005654 | nucleoplasm | Cellular component | 237 | 3472 | 929 | 18698 | 1.37387768920548 | 4.84185460220898e-08 | 4.62128122588613e-06 | 7.31498825628347 | 0.255113024757804 | SIN3B//TRERF1//ZNF217//SAP30L//TBL1XR1//EP300//POLR3H//GTF2A1//GTF2E1//GTF2H1//BAHD1//SIRT1//SUV39H1//ELL2//AFF1//RB1//CCNT2//FMR1//NPAT//FAM118B//APPL1//PPARGC1B//MED19//CDK19//GLI3//MED9//MED22//HIPK3//GSC//CREBRF//FLI1//CNOT7//RAB8B//USP28//ZBTB4//RNF2//BHLHE40//CDC14A//PATL1//WDFY3//MORC3//KLHL20//NFE2//PTEN//UBE2I//CKAP4//FAM76B//DENND1B//GADD45A//PPP1R16B//HBP1//AR//MEOX2//NR4A2//OGG1//SERPINB13//PRKAA1//SLC2A4RG//CCNL1//RBM25//RAF1//SRSF2//SRSF6//SGK1//S100PBP//WT1//CBLL1//DAZAP2//INTS8//EPC2//JARID2//NXF2//ATXN1//NXF2B//DNAJB6//SGK2//MBNL2//STX6//CEBPB//SIVA1//TACC2//CELF1//PTGES3//ZMYND11//ARPP19//TCERG1//COPS8//RPP14//STRAP//EGLN3//KLF12//MBD6//VPS37A//CRKL//PHF13//PDIK1L//PPP4R2//RNF38//SH3D19//ESCO2//DCP2//DNMT3B//E2F5//S1PR1//ZNF367//ARID2//ELAVL2//CC2D1B//ELK3//ESR1//ETS1//ETV5//ATF5//DZIP1//KDM2A//PDZRN3//PHF8//FLII//ZFPM2//SLC44A1//SMUG1//KPNA6//NR5A2//NAALADL2//GABRA5//ANAPC15//AGO1//GJA1//RNF11//FOXP1//ZBTB11//ATAD2//TBK1//NRBP1//NRBF2//NRG1//HIC1//HIVEP2//FOXA1//HOXA3//HOXD1//HSBP1//IRF1//IRF2//ISL1//JUN//MBNL1//MDM4//MEF2A//NR3C2//MYB//MYOG//PPP1R12A//NEUROD1//NFIA//NKTR//NPAS2//DCAF8//PAX5//PCBP2//ASCC1//CUTC//KLF3//CTDSPL2//ARID4B//BRWD1//POU4F1//PPARG//BNC2//RPP25//UBE2W//FBXW7//PPP3R1//DOCK10//PRKACB//BDP1//MAPK1//MAPK6//PSMA2//SMURF1//PSMD12//PTHLH//MIER1//RRAGD//RBBP8//CCND1//RORA//ATXN7//PRDM16//ZMAT3//CPEB1//NABP1//SKP1//BMPR2//SMARCA2//SMARCD2//SOX4//ZEB1//TFAP2A//TFE3//TGIF1//TIA1//UBE2V1//LDLRAD4//ZNF131//SLBP//BAG6//CENPO//E2F8//SNIP1//ADAM12//FOSL1//ANP32A//SNX27//HIST2H2BE//ARID5B//MEX3B//PCGF5//LCOR//DCTN5//TEAD2//TP63//RTCA//RUNX3//CBFB//SOCS1//SKAP2//CCND2//BTAF1//NMI//ACBD5//WDR20//RPS6KA5//FAM114A1//CLOCK//RASSF2//GIT2// |
| GO:0031981 | nuclear lumen | Cellular component | 269 | 4071 | 929 | 18698 | 1.32993562331056 | 8.28563313216512e-08 | 7.11735886052984e-06 | 7.08167430012336 | 0.289558665231432 | ZBTB18//JUN//DNAJB6//SGK2//MBNL2//STX6//CEBPB//SIVA1//TACC2//CELF1//PTGES3//ZMYND11//ARPP19//TCERG1//COPS8//RPP14//STRAP//EGLN3//KLF12//MBD6//PPARGC1B//VPS37A//CRKL//PHF13//PDIK1L//PPP4R2//RNF38//SH3D19//CREBRF//ESCO2//GADD45A//DCP2//POLR3H//DNMT3B//E2F5//S1PR1//ZNF367//ARID2//ELAVL2//CC2D1B//ELK3//EP300//ESR1//ETS1//ETV5//ATF5//DZIP1//BAHD1//ELL2//KDM2A//PDZRN3//PHF8//FLII//SIN3B//FMR1//SIRT1//ZFPM2//SLC44A1//MORC3//SMUG1//KPNA6//NR5A2//NAALADL2//GABRA5//ANAPC15//AGO1//HBP1//GJA1//RNF11//FOXP1//ZBTB11//GLI3//ATAD2//TBK1//GTF2A1//GTF2E1//GTF2H1//NRBP1//NRBF2//NRG1//HIC1//HIVEP2//FOXA1//HOXA3//HOXD1//HSBP1//IRF1//IRF2//AR//ISL1//JARID2//MBNL1//MDM4//MEF2A//NR3C2//MYB//MYOG//PPP1R12A//NEUROD1//NFIA//NFE2//NKTR//NPAS2//NPAT//NR4A2//OGG1//DCAF8//PAX5//PCBP2//ASCC1//CUTC//KLF3//CTDSPL2//ARID4B//RAB8B//SERPINB13//BRWD1//POU4F1//PPARG//BNC2//RPP25//UBE2W//FBXW7//PPP3R1//DOCK10//PRKAA1//INTS8//PRKACB//TRERF1//BDP1//MAPK1//MAPK6//NXF2//PSMA2//SMURF1//PSMD12//PTEN//PTHLH//USP28//ZBTB4//MIER1//RRAGD//RB1//RBBP8//CCND1//RNF2//RORA//ATXN1//ATXN7//PRDM16//SRSF2//SRSF6//ZMAT3//CPEB1//NABP1//SKP1//BMPR2//SMARCA2//SMARCD2//SOX4//SUV39H1//ZEB1//TFAP2A//TFE3//TGIF1//TIA1//NXF2B//UBE2I//UBE2V1//WT1//LDLRAD4//ZNF131//ZNF217//SLBP//BAG6//CENPO//SAP30L//TBL1XR1//E2F8//SNIP1//ADAM12//FOSL1//ANP32A//SNX27//HIST2H2BE//ARID5B//MEX3B//PCGF5//LCOR//DCTN5//TEAD2//CDC14A//TP63//RTCA//RUNX3//CBFB//SOCS1//SKAP2//CCND2//BTAF1//CCNT2//NMI//ACBD5//WDR20//RPS6KA5//FAM114A1//CLOCK//RASSF2//GIT2//AKAP11//DDX6//TMEM217//WDFY3//FLNA//MDFIC//YPEL2//DNAJB9//ASNA1//MYCN//NOVA1//CMPK1//PIK3CB//SLC14A1//ZNF655//FXR1//DDX3Y//GPRC5A//MOB1B//TNPO2//CFL2//CLIC4//PURA//H2AFZ//SYCE1//APPL1//SS18//AFF1//FAM118B//MED19//CDK19//MED9//MED22//HIPK3//GSC//FLI1//CNOT7//BHLHE40//PATL1//KLHL20//CKAP4//FAM76B//DENND1B//PPP1R16B//MEOX2//SLC2A4RG//CCNL1//RBM25//RAF1//SGK1//S100PBP//CBLL1//DAZAP2//EPC2//JAZF1//SESN1//NFIB//NFIC//FOXJ2//PAK6// |
| GO:0044446 | intracellular organelle part | Cellular component | 529 | 9080 | 929 | 18698 | 1.17259831280852 | 9.28209326643593e-08 | 7.24847101442588e-06 | 7.03235407210585 | 0.569429494079656 | SNX5//PLEKHF2//WDFY3//SIRT1//CNEP1R1//ANXA7//LBR//NUP54//CLIP1//BNIP2//UBE2I//CMC4//TIMM9//NLN//THOP1//DYNC1LI2//FBXO28//PPP1R12A//TTK//RASSF2//ESCO2//BAHD1//SIN3B//HIC1//MAF//MYCN//RB1//CCND2//H2AFZ//HIST2H2BE//FLNA//ZBTB18//JUN//GTF2H1//KPNA6//AGFG1//SUV39H1//DNAJB6//SGK2//MBNL2//STX6//CEBPB//SIVA1//TACC2//CELF1//PTGES3//ZMYND11//ARPP19//TCERG1//COPS8//RPP14//STRAP//EGLN3//KLF12//MBD6//PPARGC1B//VPS37A//CRKL//PHF13//PDIK1L//PPP4R2//RNF38//SH3D19//CREBRF//GADD45A//DCP2//POLR3H//DNMT3B//E2F5//S1PR1//ZNF367//ARID2//ELAVL2//CC2D1B//ELK3//EP300//ESR1//ETS1//ETV5//ATF5//DZIP1//ELL2//KDM2A//PDZRN3//PHF8//FLII//FMR1//ZFPM2//SLC44A1//MORC3//SMUG1//NR5A2//NAALADL2//GABRA5//ANAPC15//AGO1//HBP1//GJA1//RNF11//FOXP1//ZBTB11//GLI3//ATAD2//TBK1//GTF2A1//GTF2E1//NRBP1//NRBF2//NRG1//HIVEP2//FOXA1//HOXA3//HOXD1//HSBP1//IRF1//IRF2//AR//ISL1//JARID2//MBNL1//MDM4//MEF2A//NR3C2//MYB//MYOG//NEUROD1//NFIA//NFE2//NKTR//NPAS2//NPAT//NR4A2//OGG1//DCAF8//PAX5//PCBP2//ASCC1//CUTC//KLF3//CTDSPL2//ARID4B//RAB8B//SERPINB13//BRWD1//POU4F1//PPARG//BNC2//RPP25//UBE2W//FBXW7//PPP3R1//DOCK10//PRKAA1//INTS8//PRKACB//TRERF1//BDP1//MAPK1//MAPK6//NXF2//PSMA2//SMURF1//PSMD12//PTEN//PTHLH//USP28//ZBTB4//MIER1//RRAGD//RBBP8//CCND1//RNF2//RORA//ATXN1//ATXN7//PRDM16//SRSF2//SRSF6//ZMAT3//CPEB1//NABP1//SKP1//BMPR2//SMARCA2//SMARCD2//SOX4//ZEB1//TFAP2A//TFE3//TGIF1//TIA1//NXF2B//UBE2V1//WT1//LDLRAD4//ZNF131//ZNF217//SLBP//BAG6//CENPO//SAP30L//TBL1XR1//E2F8//SNIP1//ADAM12//FOSL1//ANP32A//SNX27//ARID5B//MEX3B//PCGF5//LCOR//DCTN5//TEAD2//CDC14A//TP63//RTCA//RUNX3//CBFB//SOCS1//SKAP2//BTAF1//CCNT2//NMI//ACBD5//WDR20//RPS6KA5//FAM114A1//CLOCK//GIT2//RBM25//AKAP11//DDX6//TMEM217//MDFIC//YPEL2//DNAJB9//ASNA1//NOVA1//CMPK1//PIK3CB//SLC14A1//ZNF655//FXR1//DDX3Y//GPRC5A//MOB1B//TMOD1//CFL2//CLIC4//PRICKLE2//EDNRB//TMEM201//LEMD3//GNAQ//TNPO2//C2ORF42//EI24//FASTK//MCCC2//MAVS//TMBIM6//GRSF1//NEDD1//PPP2CA//PPP2CB//CALM2//KATNAL1//VPS4B//PALLD//WDR1//SEPT9//ATG16L1//LCA5//YTHDF2//PXK//YES1//C2CD5//MID1//MZT1//KLHL42//PTP4A1//SHCBP1//KBTBD8//TNNI1//TMOD3//RANBP9//LRP8//DYNLL2//LASP1//IQGAP2//CD2AP//MARCKS//RAB28//DBN1//CAMSAP2//NAV3//CNIH1//RTN3//SEC23A//FUT9//SERINC3//CLCN5//VTI1A//B3GALNT2//RAB12//HS3ST5//FUT5//ATP2C1//ARFIP1//RAB30//YIPF6//ST8SIA5//TRIM23//RND3//GCNT4//NDFIP2//ZDHHC7//SYBU//SLC30A6//PMEPA1//ACBD3//APOO//RIC3//GNPTG//CHST1//ST3GAL5//AP1S2//MCFD2//DSEL//MMGT1//HS3ST3A1//OGN//PRELP//SDC1//WNT3//WNT7A//HS3ST1//GOLGA6A//AFTPH//TRAPPC8//PYURF//PIGP//PIGA//CRTAP//CKAP4//EOGT//IGFBP3//KDELC1//CALU//GJC1//LPCAT3//CDS1//SLC9A6//TMED1//RDH10//SPTSSB//SPTSSA//SMIM14//ACSL1//ACSL4//REEP3//RAB3GAP1//RAB18//RAB21//SLC35D1//ARL6IP1//LCLAT1//NSG1//SERP1//KCNK2//SHISA2//MGST1//IER3IP1//INSIG2//JKAMP//PLP2//UGT2B28//SEC61A2//STIM2//RDH14//ELOVL5//MSMO1//SGK1//UGT2B7//ELOVL7//ALG2//DHRS3//EMC7//SYT6//EPS15//ZPLD1//ZNRF2//GABARAPL1//SNX19//YWHAG//FZD6//ULK2//SNX17//SPPL3//SERPINI1//ARMC8//IMPDH1//KCMF1//SBF2//FUT2//CABP7//KIF13A//CALN1//VAMP3//CFDP1//DCTN6//FAM83D//RBM41//CLCN6//MMD//OSTM1//NEU1//DRAM1//SPHK2//C6ORF120//CTSF//PEX5L//EDN1//TAPT1//PRKAR2B//CEP41//EVC//DLG5//HECW2//KIF1C//RAB9B//C5AR1//CHRM2//SYT1//LDLR//BRI3//RAP1B//CHRNB4//RAP1A//SNAP25//VAMP1//KCNAB2//SIRPA//RAP2C//TEX261//RTN1//STEAP2//PSD//MTX3//PMAIP1//ARMCX6//MIEF1//RAF1//BCL2A1//ABCB7//SLC25A34//MICU3//SLC25A6//CSDE1//SLC25A28//SULF1//KLHL20//C16ORF70//TJAP1//ROR1//ZYX//PURA//SYCE1//JAZF1//SESN1//NFIB//NFIC//FOXJ2//PAK6//AFF1//MED19//CDK19//MED9//MED22//HIPK3//GSC//FLI1//CNOT7//BHLHE40//COX7A2//BBS9//CAMSAP1//DPYSL2//WDR47//MID1IP1//CCT6A//FKBP15//ARHGAP6//TSC1//ZMYND10//APPL1//RHOB//ARHGAP1//IRAK4//WDR44//SNX2//CHMP3//LRP6//SLC26A7//ABHD17C//FAM118B//PATL1//FAM76B//DENND1B//PPP1R16B//MEOX2//SLC2A4RG//CCNL1//S100PBP//CBLL1//DAZAP2//SS18//COX8C//IMMP2L//IGF1//NHLRC2//VEGFB//KRT80//KRTAP5-6//KRTAP2-4//EPC2//SEMA4C//ARMCX3// |
| GO:0044422 | organelle part | Cellular component | 540 | 9321 | 929 | 18698 | 1.1660326018231 | 1.28904130135908e-07 | 9.1917950685787e-06 | 6.88973316746817 | 0.581270182992465 | RDH10//UGT2B7//EPS8//CDC14A//RAB21//APPL1//NCK1//PRICKLE2//EDNRB//TMEM201//WDFY3//PHF8//LEMD3//CNEP1R1//GNAQ//TNPO2//LBR//NUP54//C2ORF42//DCTN5//CCND2//EI24//MAVS//TMBIM6//ACSL4//PLEKHF2//DDX6//SEPT9//GLI3//ATG16L1//BBS9//RAB28//GSTM3//TMEM17//C5ORF30//NEDD1//TAPT1//EVC//DZIP1//DLG5//CEP41//EPS15//DYNLL2//PRKACB//PRKAR2B//TNNI1//TMOD1//CALM2//DNAJB6//CFL2//PALLD//FLNA//KCNE1//PPP1R12A//HOMER1//NPNT//FXR1//GRIK2//IQGAP2//RAB18//RAB9B//RAB8B//C5AR1//ZPLD1//ZNRF2//GABARAPL1//CLIC4//NSG1//SNX5//SNX19//CLIP1//YWHAG//FZD6//AP1S2//GPRC5A//ULK2//SNX17//C2CD5//WNT3//WNT7A//SIRT1//ABCB7//TIMM9//SLC25A34//MICU3//SLC25A6//CSDE1//SLC25A28//CNIH1//STX6//RTN3//SEC23A//FUT9//SERINC3//CLCN5//VTI1A//B3GALNT2//RAB12//HS3ST5//FUT5//GJA1//ATP2C1//ARFIP1//RAB30//YIPF6//ST8SIA5//TRIM23//RND3//GCNT4//NDFIP2//ZDHHC7//SYBU//SLC30A6//PMEPA1//ACBD3//APOO//RIC3//GNPTG//CHST1//ST3GAL5//MCFD2//DSEL//MMGT1//HS3ST3A1//UGT2B28//NAV3//ACSL1//SLC44A1//MTX3//MGST1//PMAIP1//ARMCX6//MIEF1//RAF1//BCL2A1//VAMP1//SBF2//CLCN6//VPS37A//STEAP2//TBK1//RHOB//ARHGAP1//LDLR//IRAK4//WDR44//KIF13A//SNX2//VPS4B//SPPL3//TMED1//TEX261//IER3IP1//SYT1//ARMCX3//PSD//ANXA7//BNIP2//UBE2I//CMC4//NLN//THOP1//IMPDH1//SERPINI1//PSMA2//PSMD12//SULF1//KLHL20//SNAP25//C16ORF70//TJAP1//RAB3GAP1//TMOD3//FASTK//MCCC2//CRTAP//CKAP4//EOGT//IGFBP3//DNAJB9//KDELC1//CALU//OGN//PRELP//SDC1//HS3ST1//ARMC8//MAPK1//KCMF1//FMR1//PPP2CA//PPP2CB//SUV39H1//DYNC1LI2//FBXO28//TTK//RASSF2//CFDP1//DCTN6//CENPO//PTGES3//PURA//NABP1//ESCO2//BAHD1//SIN3B//HIC1//MAF//MYCN//RB1//H2AFZ//HIST2H2BE//CEBPB//ESR1//IRF1//AR//JUN//MEF2A//POU4F1//SMARCA2//SMARCD2//TP63//RUNX3//RNF2//SYCE1//MBD6//TRERF1//ZNF217//SAP30L//TBL1XR1//EP300//ZBTB18//GTF2H1//E2F5//TMEM217//JAZF1//SESN1//FOXA1//NFIB//NFIC//PAX5//FOXJ2//PAK6//KPNA6//AGFG1//SGK2//MBNL2//SIVA1//TACC2//CELF1//ZMYND11//ARPP19//TCERG1//COPS8//RPP14//STRAP//EGLN3//KLF12//PPARGC1B//CRKL//PHF13//PDIK1L//PPP4R2//RNF38//SH3D19//CREBRF//GADD45A//DCP2//POLR3H//DNMT3B//S1PR1//ZNF367//ARID2//ELAVL2//CC2D1B//ELK3//ETS1//ETV5//ATF5//ELL2//KDM2A//PDZRN3//FLII//ZFPM2//MORC3//SMUG1//NR5A2//NAALADL2//GABRA5//ANAPC15//AGO1//HBP1//RNF11//FOXP1//ZBTB11//ATAD2//GTF2A1//GTF2E1//NRBP1//NRBF2//NRG1//HIVEP2//HOXA3//HOXD1//HSBP1//IRF2//ISL1//JARID2//MBNL1//MDM4//NR3C2//MYB//MYOG//NEUROD1//NFIA//NFE2//NKTR//NPAS2//NPAT//NR4A2//OGG1//DCAF8//PCBP2//ASCC1//CUTC//KLF3//CTDSPL2//ARID4B//SERPINB13//BRWD1//PPARG//BNC2//RPP25//UBE2W//FBXW7//PPP3R1//DOCK10//PRKAA1//INTS8//BDP1//MAPK6//NXF2//SMURF1//PTEN//PTHLH//USP28//ZBTB4//MIER1//RRAGD//RBBP8//CCND1//RORA//ATXN1//ATXN7//PRDM16//SRSF2//SRSF6//ZMAT3//CPEB1//SKP1//BMPR2//SOX4//ZEB1//TFAP2A//TFE3//TGIF1//TIA1//NXF2B//UBE2V1//WT1//LDLRAD4//ZNF131//SLBP//BAG6//E2F8//SNIP1//ADAM12//FOSL1//ANP32A//SNX27//ARID5B//MEX3B//PCGF5//LCOR//TEAD2//RTCA//CBFB//SOCS1//SKAP2//BTAF1//CCNT2//NMI//ACBD5//WDR20//RPS6KA5//FAM114A1//CLOCK//GIT2//RBM25//RBM41//AKAP11//MDFIC//YPEL2//ASNA1//NOVA1//CMPK1//PIK3CB//SLC14A1//ZNF655//DDX3Y//MOB1B//AFF1//MED19//CDK19//MED9//MED22//HIPK3//GSC//FLI1//CNOT7//BHLHE40//COX7A2//GRSF1//KATNAL1//MZT1//ROR1//ZYX//WDR1//CAMSAP2//MARCKS//LCA5//YTHDF2//PXK//YES1//MID1//KLHL42//PTP4A1//SHCBP1//KBTBD8//KIF1C//CAMSAP1//DPYSL2//REEP3//WDR47//MID1IP1//KCNAB2//CCT6A//RANBP9//LRP8//FKBP15//ARHGAP6//TSC1//LASP1//CD2AP//ZMYND10//DBN1//HECW2//FAM83D//GOLGA6A//AFTPH//FUT2//CABP7//CALN1//VAMP3//TRAPPC8//PYURF//PIGP//PIGA//ARL6IP1//GJC1//LPCAT3//CDS1//SLC9A6//SPTSSB//SPTSSA//SMIM14//SLC35D1//LCLAT1//SERP1//KCNK2//SHISA2//INSIG2//JKAMP//PLP2//SEC61A2//STIM2//RDH14//ELOVL5//MSMO1//SGK1//ELOVL7//ALG2//DHRS3//RTN1//EDN1//EMC7//SYT6//C6ORF120//NEU1//CTSF//PEX5L//CNGA3//CHRM2//LRP6//MMD//CHMP3//BRI3//RAP1B//CHRNB4//RAP1A//SLC26A7//RAP2C//ABHD17C//SIRPA//GSG1L//EPC2//KRT80//KRTAP5-6//KRTAP2-4//OSTM1//DRAM1//SPHK2//SEMA4C//SS18//FAM118B//PATL1//FAM76B//DENND1B//PPP1R16B//MEOX2//SLC2A4RG//CCNL1//S100PBP//CBLL1//DAZAP2//IGF1//NHLRC2//VEGFB//COX8C//IMMP2L// |
| GO:0036477 | somatodendritic compartment | Cellular component | 72 | 766 | 929 | 18698 | 1.89183461820594 | 1.39107492306779e-07 | 9.1917950685787e-06 | 6.85664947834826 | 0.077502691065662 | STMN2//CHRM2//CNN3//CPD//VTI1A//LPAR1//ACSL4//FLNA//ARC//FMR1//ADNP//GPM6A//AGFG1//KCNJ2//KCNK2//LRP6//SERPINI1//PRKAR2B//SMURF1//PURA//ELOVL5//BMPR2//TRAK2//STRN//LRP8//RIC3//FZD3//FXR1//PPP1R1B//RAPGEF2//GABRA5//KCNB1//CNGA3//RBFOX3//WDFY3//GRIK2//NEUROG1//MAPK1//GLRA3//GPHN//SLC9A6//KIF1C//DBN1//SAMD14//CPEB3//NPTN//NSG1//KLHL20//GLRB//GNAQ//NRG1//NTF3//KIF13A//CPEB1//C16ORF70//TMEM185A//TP63//BSN//HOMER1//GABARAPL1//SGCE//ZMYND8//GRM3//KCNA4//DOCK10//PSD//PTEN//ABHD17C//CD3E//SYNGAP1//LDLR//SNAP25// |
| GO:0044428 | nuclear part | Cellular component | 287 | 4435 | 929 | 18698 | 1.30246995532892 | 1.77543524421035e-07 | 1.08935633912621e-05 | 6.75069516317931 | 0.308934337997847 | ZBTB18//JUN//GTF2H1//WDFY3//SIRT1//CNEP1R1//ANXA7//LBR//NUP54//CLIP1//BNIP2//UBE2I//KPNA6//AGFG1//SUV39H1//DNAJB6//SGK2//MBNL2//STX6//CEBPB//SIVA1//TACC2//CELF1//PTGES3//ZMYND11//ARPP19//TCERG1//COPS8//RPP14//STRAP//EGLN3//KLF12//MBD6//PPARGC1B//VPS37A//CRKL//PHF13//PDIK1L//PPP4R2//RNF38//SH3D19//CREBRF//ESCO2//GADD45A//DCP2//POLR3H//DNMT3B//E2F5//S1PR1//ZNF367//ARID2//ELAVL2//CC2D1B//ELK3//EP300//ESR1//ETS1//ETV5//ATF5//DZIP1//BAHD1//ELL2//KDM2A//PDZRN3//PHF8//FLII//SIN3B//FMR1//ZFPM2//SLC44A1//MORC3//SMUG1//NR5A2//NAALADL2//GABRA5//ANAPC15//AGO1//HBP1//GJA1//RNF11//FOXP1//ZBTB11//GLI3//ATAD2//TBK1//GTF2A1//GTF2E1//NRBP1//NRBF2//NRG1//HIC1//HIVEP2//FOXA1//HOXA3//HOXD1//HSBP1//IRF1//IRF2//AR//ISL1//JARID2//MBNL1//MDM4//MEF2A//NR3C2//MYB//MYOG//PPP1R12A//NEUROD1//NFIA//NFE2//NKTR//NPAS2//NPAT//NR4A2//OGG1//DCAF8//PAX5//PCBP2//ASCC1//CUTC//KLF3//CTDSPL2//ARID4B//RAB8B//SERPINB13//BRWD1//POU4F1//PPARG//BNC2//RPP25//UBE2W//FBXW7//PPP3R1//DOCK10//PRKAA1//INTS8//PRKACB//TRERF1//BDP1//MAPK1//MAPK6//NXF2//PSMA2//SMURF1//PSMD12//PTEN//PTHLH//USP28//ZBTB4//MIER1//RRAGD//RB1//RBBP8//CCND1//RNF2//RORA//ATXN1//ATXN7//PRDM16//SRSF2//SRSF6//ZMAT3//CPEB1//NABP1//SKP1//BMPR2//SMARCA2//SMARCD2//SOX4//ZEB1//TFAP2A//TFE3//TGIF1//TIA1//NXF2B//UBE2V1//WT1//LDLRAD4//ZNF131//ZNF217//SLBP//BAG6//CENPO//SAP30L//TBL1XR1//E2F8//SNIP1//ADAM12//FOSL1//ANP32A//SNX27//HIST2H2BE//ARID5B//MEX3B//PCGF5//LCOR//DCTN5//TEAD2//CDC14A//TP63//RTCA//RUNX3//CBFB//SOCS1//SKAP2//CCND2//BTAF1//CCNT2//NMI//ACBD5//WDR20//RPS6KA5//FAM114A1//CLOCK//RASSF2//GIT2//RBM25//AKAP11//DDX6//TMEM217//FLNA//MDFIC//YPEL2//DNAJB9//ASNA1//MYCN//NOVA1//CMPK1//PIK3CB//SLC14A1//ZNF655//FXR1//DDX3Y//GPRC5A//MOB1B//TMOD1//CFL2//CLIC4//PRICKLE2//EDNRB//TMEM201//LEMD3//GNAQ//TNPO2//C2ORF42//EI24//RBM41//UGT2B28//NAV3//AFF1//MED19//CDK19//MED9//MED22//HIPK3//GSC//FLI1//CNOT7//BHLHE40//JAZF1//SESN1//NFIB//NFIC//FOXJ2//PAK6//PURA//H2AFZ//SYCE1//APPL1//FAM118B//PATL1//KLHL20//CKAP4//FAM76B//DENND1B//PPP1R16B//MEOX2//SLC2A4RG//CCNL1//RAF1//SGK1//S100PBP//CBLL1//DAZAP2//SS18//EPC2// |
| GO:0043227 | membrane-bounded organelle | Cellular component | 694 | 12616 | 929 | 18698 | 1.10717744924517 | 5.11300984192789e-07 | 2.92805030281071e-05 | 6.29132337130811 | 0.747039827771798 | SLC9A6//ARAP2//SPPL3//VPS37A//VTI1A//DENND1B//POLR3H//DNMT3B//S1PR1//CC2D1B//DENND6A//EPS15//MYCBP2//APPL1//GJA1//RAB30//AGFG1//RHOB//RND3//NEU1//NOVA1//PAX5//RAB8B//UGT2B28//NDFIP2//PPARG//AFTPH//SYBU//SPHK2//PMEPA1//RBBP8//SLC14A1//SMARCA2//BNIP2//HSPA13//TFAP2A//TNFAIP3//UGT2B7//VBP1//LDLRAD4//BAG6//RIC3//CLIP4//UBA5//TEAD2//RUNX3//AP1S2//BTAF1//GPRC5A//VAMP3//CLOCK//SNX17//GIT2//RDH10//EPS8//GLT6D1//MSN//SNAP25//CALM2//CMC4//PYURF//CCDC58//PPARGC1B//DYNLL2//PPTC7//DDX6//PRSS35//ELK3//ACSL1//SIRT1//SLC44A1//GABARAPL1//CLIC4//TIMM9//GLRX//TCAIM//GRSF1//SLC25A6//COX8C//JARID2//BOLA3//MGST1//NKTR//OGG1//DCAF8//PMAIP1//PON2//MIEF1//THG1L//PPP2CA//PPP3R1//MAPK1//SMURF1//PTEN//MAVS//RDH14//RAF1//MCCC2//SGK1//ACBD3//TRAK2//SOX4//YWHAG//ZNF217//APOO//PRR5L//ABHD11//CASP2//CCM2//TP63//BAG5//RANBP9//DNAJB6//TOB1//MBNL2//TSHZ1//ZBTB18//CEBPB//CELF1//PTGES3//ZMYND11//TCERG1//COPS8//PNRC1//RPP14//EGLN3//MBD6//ZNF526//ADH4//CPEB2//UBXN2B//CREBL2//GSC//RBFOX3//PHF13//PPP4R2//RNF38//CREBRF//ESCO2//SPRED1//GADD45A//ZNF800//ARX//DUSP1//E2F5//ZNF367//EMX2//EN1//EP300//ESR1//ETS1//ETV5//MED19//CCNY//JAZF1//ATF5//ZFP30//CPEB3//DZIP1//FOXF1//FOXF2//WDFY3//PALLD//PDZRN3//MYT1L//CDK19//FLI1//PHF8//DCUN1D4//FLNA//SIN3B//FMR1//ADNP//ZFPM2//ZMYND8//CNOT6L//NR5A2//NUTM1//DNAJB5//ZNF521//RWDD3//PPP1R16B//TES//AGO1//RNF11//NSG1//FOXP1//SESN1//GLI3//ATAD2//GSTM3//GTF2E1//CNOT7//PURG//MDFIC//TNPO2//H2AFZ//HIVEP2//ANXA7//HMGB3//FOXA1//HSBP1//SPOPL//ID2//ZNF680//IGFBP3//IMPDH1//IRF1//AR//ISL1//JUN//TRIM23//KCNK2//KDR//ZNF662//USP27X//RGS7BP//MAF//MBNL1//MDM4//MEF2A//MEOX2//AFF1//MLLT6//ASNA1//HSBP1L1//MYB//MYBL1//MYCN//MYOG//NAP1L3//NCK1//NEUROD1//NEUROG1//NFIA//NFE2//NFIB//NFIC//NHLH2//NPAS2//NPAT//NR4A2//OTX2//PCBP2//ASCC1//MEMO1//CUTC//SEPSECS//PHF20L1//IRAK4//IER5//CDK17//COMMD10//YTHDF2//GSKIP//ARMCX3//KLF13//RASD1//CMPK1//ARID4B//PIK3CB//PKNOX1//BRWD1//ERRFI1//POU3F2//POU4F1//EGLN1//CCNJ//C2ORF42//PPP2CB//ZNF532//UBE2W//DOCK10//PRKAA1//BTBD7//TRERF1//FOXJ2//MAPK6//NXF2//SLC2A4RG//PSMA2//PAK6//CCNL1//PHTF2//CNOT6//TAOK1//RANBP10//ZBTB4//ZBTB26//MIER1//PURA//MID1IP1//RRAGD//RB1//HIVEP3//RBMS1//CCND1//RNF2//RORA//SARS//ATXN1//ATXN7//PRDM16//SRSF2//CPEB1//SH3BGRL//GMNC//S100PBP//NABP1//RFX7//SKP1//CDK15//SOX5//SS18//SUV39H1//BTF3//ZEB1//BTG1//TMBIM6//TFE3//KLF10//TSN//TSC1//TUB//NXF2B//TXK//UBE2I//UBE2V1//WT1//ZNF3//ZNF708//ZNF131//ZNF227//ZYX//PTP4A1//SLBP//ZNF655//ZSCAN5A//BHLHE41//SAP30L//NPEPL1//TBL1XR1//E2F8//SNIP1//FOSL1//PTP4A2//FXR1//ANP32A//HIST2H2BE//KATNAL1//PPP1R1B//ARID5B//PCGF5//DGKE//ALG2//ZIC5//BHLHE40//CDC14A//KLF7//RTCA//DDX3Y//BSN//FUBP3//CCND2//CCNT2//ACBD5//RPS6KA5//MOB1B//FAM114A1//NREP//PLAA//FOXP2//QKI//VPS4B//ZNF516//PHF14//ST18//RASSF2//RTN3//CDS1//CRTAP//KIF1C//CKAP4//TMED1//B3GALNT2//TMEM64//SMIM14//TAPT1//SULF1//LCLAT1//SERP1//ZDHHC22//YIPF6//FIBIN//SHISA2//LRP6//FAM19A1//DNAJB9//IER3IP1//PLP2//ZDHHC7//STIM2//ELOVL5//RTN1//MSMO1//PLEKHF2//ELOVL7//KCNIP4//CALU//CAST//ZDHHC18//PPP1R15B//EI24//STX6//FUT9//ADCY3//STMN2//CLCN5//CPD//RAB12//RAB3GAP1//RAB18//CAMSAP2//MMD//FUT2//FUT5//ATP2C1//KLHL20//GOLGA6A//KCNJ2//LDLR//WDR44//SLC30A6//PTHLH//SYT1//YES1//CSDE1//KBTBD8//GNPTG//SGCE//CCDC126//MMGT1//TJAP1//GPM6A//OGN//PRELP//CTSF//DYNC1LI2//KCNE1//NAGA//DRAM1//ARRDC3//SNX2//TBC1D12//NRBF2//ATG16L1//AKAP11//C8ORF44-SGK3//TRAPPC8//SGK3//DLL1//NTF3//CHMP3//CHIC1//CXXC4//SOCS1//DNAJA2//CFL2//IQGAP2//WASF3//COX7A2//CR2//CRKL//SIRPA//ADSS//DPYSL2//EFNA1//F3//ACSL4//RAB21//EFR3A//CD2AP//PHLDA3//NPNT//GFPT1//GNAQ//GSTA2//PSAT1//HPRT1//ITGB8//C6ORF120//ARHGAP1//LASP1//MARCKS//MEP1A//PI15//SLC26A4//PFN2//SERPINI1//SERPINB13//UBL3//ARL15//ANO1//PRKACB//PRKAR2B//PSMD12//RAP2C//PTPRG//RAP1A//RAP1B//ROBO2//SDC1//BMP3//DLK2//TGM3//WNT3//WNT7A//HDHD2//CCT6A//SLIT2//SLK//WDR1//PEX5L//SLC26A7//CRHR1//DGKH//LPAR1//ARC//STEAP2//SNX5//CLIP1//SNX27//EDN1//RAB9B//ITSN1//RAPGEF2//ADRB1//FKBP15//SLC31A2//TNF//TEX261//IGF1//KLK15//CLCN6//ZNRF2//OSTM1//SNX19//CNIH1//MCFD2//SEC23A//C5AR1//SYT6//SEMA4C//VAMP1//C16ORF70//CHRM2//NHLRC2//VEGFB//CHRNB4//KCNAB2//ARMC8//BRI3//ABHD17C//KCMF1//SERINC3//HS3ST5//ARFIP1//ST8SIA5//GCNT4//CHST1//ST3GAL5//DSEL//HS3ST3A1//LEMD3//LBR//NAV3//MTX3//ARMCX6//BCL2A1//ABCB7//SLC25A34//MICU3//SLC25A28//SBF2//TBK1//KIF13A//ZPLD1//FZD6//ULK2//C2CD5//PRICKLE2//EDNRB//TMEM201//CNEP1R1//NUP54//DCTN5//CABP7//CALN1//CNGA3//DHRS3//TMEM17//EVC//BBS9//NLN//THOP1//FASTK//IMMP2L//BDP1//GTF2H1//SMARCD2//SYCE1//TMEM217//KPNA6//SGK2//SIVA1//TACC2//ARPP19//STRAP//KLF12//PDIK1L//SH3D19//DCP2//ARID2//ELAVL2//BAHD1//ELL2//KDM2A//FLII//MORC3//SMUG1//NAALADL2//GABRA5//ANAPC15//HBP1//ZBTB11//GTF2A1//NRBP1//NRG1//HIC1//HOXA3//HOXD1//IRF2//NR3C2//PPP1R12A//KLF3//CTDSPL2//BNC2//RPP25//FBXW7//INTS8//USP28//SRSF6//ZMAT3//BMPR2//TGIF1//TIA1//CENPO//ADAM12//MEX3B//LCOR//CBFB//SKAP2//NMI//WDR20//RBM25//RBM41//YPEL2//TMOD1//FAM118B//MED9//MED22//HIPK3//PATL1//FAM76B//CBLL1//DAZAP2//EPC2//PSD//PIGP//PIGA//ARL6IP1//EOGT//KDELC1//GJC1//LPCAT3//SPTSSB//SPTSSA//REEP3//SLC35D1//INSIG2//JKAMP//SEC61A2//EMC7//HS3ST1// |
| GO:0043231 | intracellular membrane-bounded organelle | Cellular component | 610 | 10864 | 929 | 18698 | 1.13010688167713 | 7.85811480028865e-07 | 4.21882538340497e-05 | 6.10468163080957 | 0.656620021528525 | CMC4//PYURF//CCDC58//PPARGC1B//DYNLL2//PPTC7//DDX6//PRSS35//ELK3//ACSL1//SIRT1//SLC44A1//GABARAPL1//CLIC4//TIMM9//GJA1//GLRX//TCAIM//GRSF1//SLC25A6//COX8C//JARID2//BOLA3//MGST1//NKTR//OGG1//DCAF8//RAB8B//PMAIP1//PON2//MIEF1//NDFIP2//THG1L//PPP2CA//PPP3R1//MAPK1//SMURF1//PTEN//MAVS//RDH14//RAF1//MCCC2//SGK1//ACBD3//TRAK2//SOX4//YWHAG//ZNF217//APOO//PRR5L//ABHD11//CASP2//CCM2//TP63//BAG5//RANBP9//DNAJB6//TOB1//MBNL2//TSHZ1//ZBTB18//CEBPB//CELF1//PTGES3//ZMYND11//TCERG1//COPS8//PNRC1//RPP14//EGLN3//MBD6//ZNF526//ADH4//CPEB2//UBXN2B//CREBL2//GSC//RBFOX3//PHF13//PPP4R2//RNF38//CREBRF//ESCO2//SPRED1//GADD45A//ZNF800//ARX//DNMT3B//DUSP1//E2F5//ZNF367//CC2D1B//EMX2//EN1//EP300//ESR1//ETS1//ETV5//MED19//CCNY//JAZF1//ATF5//ZFP30//CPEB3//DZIP1//FOXF1//FOXF2//WDFY3//PALLD//PDZRN3//MYT1L//MYCBP2//CDK19//FLI1//PHF8//DCUN1D4//FLNA//SIN3B//FMR1//ADNP//ZFPM2//ZMYND8//CNOT6L//NR5A2//NUTM1//DNAJB5//ZNF521//RWDD3//PPP1R16B//APPL1//TES//AGO1//RNF11//NSG1//FOXP1//SESN1//GLI3//ATAD2//GSTM3//GTF2E1//CNOT7//PURG//MDFIC//TNPO2//H2AFZ//HIVEP2//ANXA7//HMGB3//FOXA1//HSBP1//SPOPL//ID2//ZNF680//IGFBP3//IMPDH1//IRF1//AR//ISL1//JUN//TRIM23//KCNK2//KDR//RHOB//ZNF662//USP27X//RGS7BP//MAF//MBNL1//MDM4//MEF2A//MEOX2//AFF1//MLLT6//ASNA1//HSBP1L1//MSN//MYB//MYBL1//MYCN//MYOG//NAP1L3//NCK1//NEUROD1//NEUROG1//NFIA//NFE2//NFIB//NFIC//NHLH2//NOVA1//NPAS2//NPAT//NR4A2//OTX2//PCBP2//ASCC1//MEMO1//CUTC//SEPSECS//PHF20L1//IRAK4//IER5//CDK17//COMMD10//YTHDF2//GSKIP//ARMCX3//KLF13//RASD1//CMPK1//ARID4B//PIK3CB//PKNOX1//BRWD1//ERRFI1//POU3F2//POU4F1//EGLN1//CCNJ//PPARG//AFTPH//C2ORF42//PPP2CB//ZNF532//UBE2W//DOCK10//PRKAA1//BTBD7//TRERF1//FOXJ2//MAPK6//NXF2//SLC2A4RG//PSMA2//PAK6//CCNL1//PHTF2//CNOT6//TAOK1//RANBP10//ZBTB4//ZBTB26//MIER1//PURA//MID1IP1//RRAGD//RB1//HIVEP3//RBBP8//RBMS1//CCND1//RNF2//RORA//SARS//ATXN1//ATXN7//PRDM16//SRSF2//CPEB1//SH3BGRL//GMNC//S100PBP//NABP1//RFX7//SKP1//CDK15//SMARCA2//SOX5//SS18//HSPA13//SUV39H1//BTF3//ZEB1//BTG1//TMBIM6//TFAP2A//TFE3//KLF10//TNFAIP3//TSN//TSC1//TUB//NXF2B//TXK//UBE2I//UBE2V1//VBP1//WT1//ZNF3//ZNF708//ZNF131//ZNF227//ZYX//PTP4A1//SLBP//ZNF655//ZSCAN5A//BAG6//BHLHE41//SAP30L//NPEPL1//TBL1XR1//E2F8//SNIP1//UBA5//CALM2//FOSL1//PTP4A2//FXR1//ANP32A//HIST2H2BE//KATNAL1//PPP1R1B//ARID5B//PCGF5//TEAD2//DGKE//ALG2//ZIC5//BHLHE40//CDC14A//KLF7//RTCA//RUNX3//DDX3Y//BSN//FUBP3//CCND2//CCNT2//ACBD5//RPS6KA5//MOB1B//FAM114A1//NREP//PLAA//FOXP2//QKI//VPS4B//CLOCK//ZNF516//PHF14//ST18//RASSF2//RTN3//CDS1//CRTAP//KIF1C//CKAP4//TMED1//B3GALNT2//TMEM64//SMIM14//TAPT1//SULF1//LCLAT1//SERP1//ZDHHC22//YIPF6//FIBIN//SHISA2//LRP6//FAM19A1//DNAJB9//IER3IP1//PLP2//UGT2B28//ZDHHC7//STIM2//ELOVL5//RTN1//MSMO1//PLEKHF2//ELOVL7//KCNIP4//CALU//CAST//ZDHHC18//PPP1R15B//EI24//STX6//FUT9//ADCY3//STMN2//CLCN5//SPPL3//CPD//VTI1A//RAB12//RAB3GAP1//RAB18//CAMSAP2//MMD//FUT2//FUT5//ATP2C1//KLHL20//GOLGA6A//GLT6D1//KCNJ2//LDLR//WDR44//SLC30A6//PTHLH//SYT1//YES1//CSDE1//KBTBD8//GNPTG//AP1S2//SGCE//CCDC126//MMGT1//TJAP1//SNX17//RAB30//TBC1D12//NRBF2//ATG16L1//AKAP11//DYNC1LI2//KCNE1//NAGA//NEU1//DRAM1//ARRDC3//SNX2//CTSF//ACSL4//PEX5L//MARCKS//CLCN6//ZNRF2//GNAQ//OSTM1//SPHK2//OGN//SERPINB13//PRELP//SDC1//EDN1//CNIH1//MCFD2//BRI3//RAP1B//C6ORF120//MTX3//ARMCX6//BCL2A1//VAMP1//ABCB7//SLC25A34//MICU3//PRKAR2B//SLC25A28//COX7A2//NLN//THOP1//FASTK//IMMP2L//SBF2//BDP1//GTF2H1//SMARCD2//SYCE1//TMEM217//PAX5//CNEP1R1//LBR//NUP54//CLIP1//BNIP2//LEMD3//TMEM201//NAV3//KPNA6//AGFG1//SGK2//SIVA1//TACC2//ARPP19//STRAP//KLF12//VPS37A//CRKL//PDIK1L//SH3D19//DCP2//POLR3H//S1PR1//ARID2//ELAVL2//BAHD1//ELL2//KDM2A//FLII//MORC3//SMUG1//NAALADL2//GABRA5//ANAPC15//HBP1//ZBTB11//TBK1//GTF2A1//NRBP1//NRG1//HIC1//HOXA3//HOXD1//IRF2//NR3C2//PPP1R12A//KLF3//CTDSPL2//BNC2//RPP25//FBXW7//INTS8//PRKACB//PSMD12//USP28//SRSF6//ZMAT3//BMPR2//TGIF1//TIA1//LDLRAD4//CENPO//ADAM12//SNX27//MEX3B//LCOR//DCTN5//CBFB//SOCS1//SKAP2//BTAF1//NMI//WDR20//GIT2//RBM25//RBM41//YPEL2//SLC14A1//GPRC5A//TMOD1//FAM118B//CFL2//MED9//MED22//HIPK3//PATL1//FAM76B//DENND1B//CBLL1//DAZAP2//PRICKLE2//EDNRB//EPC2//PIGP//PIGA//ARL6IP1//EOGT//WNT3//WNT7A//KDELC1//GJC1//LPCAT3//SLC9A6//SEC23A//RDH10//SPTSSB//SPTSSA//REEP3//RAB21//SLC35D1//INSIG2//JKAMP//SEC61A2//UGT2B7//RIC3//DHRS3//TEX261//EMC7//SYT6//SERINC3//HS3ST5//ARFIP1//ST8SIA5//RND3//GCNT4//SYBU//PMEPA1//CHST1//ST3GAL5//DSEL//HS3ST3A1//HS3ST1//SNAP25//C16ORF70//STEAP2//CABP7//KIF13A//CALN1//VAMP3//TRAPPC8//ARAP2//DENND6A//EPS15//CLIP4// |
| GO:0005634 | nucleus | Cellular component | 431 | 7267 | 929 | 18698 | 1.19371747447024 | 9.99862906711131e-07 | 5.05224845214624e-05 | 6.00005954294042 | 0.463939720129171 | MARCKS//SIN3B//TRERF1//ZNF217//SAP30L//TBL1XR1//EP300//BDP1//ZBTB18//JUN//SUV39H1//GTF2H1//PURA//NABP1//CEBPB//ESR1//SIRT1//H2AFZ//IRF1//AR//MEF2A//POU4F1//SMARCA2//SMARCD2//TP63//RUNX3//UBE2I//SYCE1//MBD6//E2F5//TMEM217//JAZF1//SESN1//FOXA1//NFIB//NFIC//PAX5//FOXJ2//PAK6//RNF2//WDFY3//CNEP1R1//ANXA7//LBR//NUP54//CLIP1//BNIP2//LEMD3//TMEM201//UGT2B28//NAV3//KPNA6//AGFG1//DNAJB6//SGK2//MBNL2//STX6//SIVA1//TACC2//CELF1//PTGES3//ZMYND11//ARPP19//TCERG1//COPS8//RPP14//STRAP//EGLN3//KLF12//PPARGC1B//VPS37A//CRKL//PHF13//PDIK1L//PPP4R2//RNF38//SH3D19//CREBRF//ESCO2//GADD45A//DCP2//POLR3H//DNMT3B//S1PR1//ZNF367//ARID2//ELAVL2//CC2D1B//ELK3//ETS1//ETV5//ATF5//DZIP1//BAHD1//ELL2//KDM2A//PDZRN3//PHF8//FLII//FMR1//ZFPM2//SLC44A1//MORC3//SMUG1//NR5A2//NAALADL2//GABRA5//ANAPC15//AGO1//HBP1//GJA1//RNF11//FOXP1//ZBTB11//GLI3//ATAD2//TBK1//GTF2A1//GTF2E1//NRBP1//NRBF2//NRG1//HIC1//HIVEP2//HOXA3//HOXD1//HSBP1//IRF2//ISL1//JARID2//MBNL1//MDM4//NR3C2//MYB//MYOG//PPP1R12A//NEUROD1//NFIA//NFE2//NKTR//NPAS2//NPAT//NR4A2//OGG1//DCAF8//PCBP2//ASCC1//CUTC//KLF3//CTDSPL2//ARID4B//RAB8B//SERPINB13//BRWD1//PPARG//BNC2//RPP25//UBE2W//FBXW7//PPP3R1//DOCK10//PRKAA1//INTS8//PRKACB//MAPK1//MAPK6//NXF2//PSMA2//SMURF1//PSMD12//PTEN//PTHLH//USP28//ZBTB4//MIER1//RRAGD//RB1//RBBP8//CCND1//RORA//ATXN1//ATXN7//PRDM16//SRSF2//SRSF6//ZMAT3//CPEB1//SKP1//BMPR2//SOX4//ZEB1//TFAP2A//TFE3//TGIF1//TIA1//NXF2B//UBE2V1//WT1//LDLRAD4//ZNF131//SLBP//BAG6//CENPO//E2F8//SNIP1//ADAM12//FOSL1//ANP32A//SNX27//HIST2H2BE//ARID5B//MEX3B//PCGF5//LCOR//DCTN5//TEAD2//CDC14A//RTCA//CBFB//SOCS1//SKAP2//CCND2//BTAF1//CCNT2//NMI//ACBD5//WDR20//RPS6KA5//FAM114A1//CLOCK//RASSF2//GIT2//RBM25//RBM41//AKAP11//DDX6//FLNA//MDFIC//YPEL2//DNAJB9//ASNA1//MYCN//NOVA1//CMPK1//PIK3CB//SLC14A1//ZNF655//FXR1//DDX3Y//GPRC5A//MOB1B//AFF1//TMOD1//FAM118B//CFL2//CLIC4//APPL1//MED19//CDK19//MED9//MED22//HIPK3//GSC//FLI1//CNOT7//BHLHE40//PATL1//KLHL20//CKAP4//FAM76B//DENND1B//PPP1R16B//MEOX2//SLC2A4RG//CCNL1//RAF1//SGK1//S100PBP//CBLL1//DAZAP2//PRICKLE2//EDNRB//GNAQ//TNPO2//C2ORF42//EI24//EPC2//SS18//RANBP9//TOB1//TSHZ1//PNRC1//ZNF526//ADH4//CPEB2//UBXN2B//CREBL2//DYNLL2//RBFOX3//SPRED1//ZNF800//ARX//DUSP1//EMX2//EN1//CCNY//ZFP30//CPEB3//FOXF1//FOXF2//PALLD//MYT1L//MYCBP2//DCUN1D4//ADNP//ZMYND8//CNOT6L//NUTM1//DNAJB5//ZNF521//RWDD3//TES//NSG1//GLRX//SLC25A6//GSTM3//PURG//HMGB3//SPOPL//ID2//ZNF680//IGFBP3//IMPDH1//TRIM23//KCNK2//KDR//RHOB//ZNF662//USP27X//RGS7BP//MAF//MGST1//MLLT6//HSBP1L1//MSN//MYBL1//NAP1L3//NCK1//NEUROG1//NHLH2//OTX2//MEMO1//SEPSECS//PHF20L1//IRAK4//IER5//CDK17//COMMD10//YTHDF2//GSKIP//ARMCX3//KLF13//RASD1//PKNOX1//PMAIP1//ERRFI1//PON2//POU3F2//EGLN1//CCNJ//AFTPH//PPP2CA//PPP2CB//ZNF532//BTBD7//PHTF2//CNOT6//TAOK1//RANBP10//RDH14//ZBTB26//MID1IP1//HIVEP3//RBMS1//SARS//SH3BGRL//GMNC//RFX7//CDK15//TRAK2//SOX5//HSPA13//BTF3//BTG1//TMBIM6//KLF10//TNFAIP3//TSN//TSC1//TUB//TXK//VBP1//ZNF3//ZNF708//ZNF227//ZYX//PTP4A1//ZSCAN5A//BHLHE41//NPEPL1//UBA5//CALM2//PTP4A2//CASP2//KATNAL1//PPP1R1B//DGKE//ALG2//ZIC5//KLF7//BSN//FUBP3//NREP//PLAA//FOXP2//QKI//VPS4B//BAG5//ZNF516//PHF14//ST18// |
| GO:0097458 | neuron part | Cellular component | 117 | 1533 | 929 | 18698 | 1.53611294260394 | 1.63969096776694e-06 | 7.80549749512764e-05 | 5.7852379956353 | 0.125941872981701 | GNAQ//VTI1A//NTF3//RAB8B//SNAP25//SYT1//CNN3//SAMD14//EPS8//CPEB3//ARC//FMR1//NPTN//GRIK2//GRM3//SEMA4C//PSD//ABHD17C//CPEB1//BMPR2//SPOCK1//STRN//LRP8//FXR1//KCNAB2//SYNGAP1//BSN//DLG5//HOMER1//KIF1C//STMN2//CHRNB4//LYPD6//CPEB2//GABRA5//GLRB//GPM6A//POU4F1//PTEN//RAP1A//KIF13A//ITSN1//SLC6A1//SLC6A11//RIC3//GLRA3//FOSL1//VAMP3//RAPGEF2//SYT6//VAMP1//C16ORF70//KCNB1//NSG1//LDLR//CNGA3//DHRS3//RIMS4//ZNRF2//CHRM2//CPD//LPAR1//ACSL4//FLNA//ADNP//AGFG1//KCNJ2//KCNK2//LRP6//SERPINI1//PRKAR2B//SMURF1//PURA//ELOVL5//TRAK2//FZD3//PPP1R1B//ZMYND8//RBFOX3//WDFY3//NEUROG1//MAPK1//PPFIA1//SMARCA2//SS18//KCNJ10//WNT7A//DTNA//TNFRSF21//KLHL20//KCNA4//ROR1//FEZ2//GPHN//SLC9A6//DBN1//NRG1//TMEM185A//TP63//CDC14A//ROBO2//GABARAPL1//SGCE//PALLD//FKBP15//OTX2//TSC1//SCN1B//STX6//ADCYAP1//PFN2//RAB21//SYBU//GSG1L//DOCK10//CD3E//NFIB// |
| GO:0045202 | synapse | Cellular component | 76 | 880 | 929 | 18698 | 1.73824248948038 | 1.72647790928318e-06 | 7.80549749512764e-05 | 5.76283897418553 | 0.0818083961248654 | PDZRN3//NRG1//ASCC1//SPOCK1//SYT1//BSN//NPTN//GLRB//CHRM2//CNN3//SAMD14//EPS8//CPEB3//ARC//FMR1//GRIK2//GRM3//SEMA4C//PSD//ABHD17C//CPEB1//BMPR2//STRN//LRP8//FXR1//KCNAB2//SYNGAP1//DLG5//HOMER1//VTI1A//NTF3//RAB8B//SNAP25//STX6//SYT6//VAMP1//C16ORF70//RIMS4//ZNRF2//FOSL1//ADCYAP1//PFN2//LPAR1//ZMYND8//GPM6A//KCNA4//KCNJ2//DOCK10//PRKAR2B//PTEN//CD3E//SLC9A6//ROR1//KCNK2//GPHN//CHRNB4//GABRA5//NSG1//KCNB1//GLRA3//FZD3//PPFIA1//CPD//KCNJ10//WNT7A//RAB3GAP1//GSG1L//LYPD6//CPEB2//DTNA//LRP6//ENAH//ITSN1//VAMP3//PLAA//RAPGEF2// |
| GO:0030425 | dendrite | Cellular component | 55 | 569 | 929 | 18698 | 1.94549386020836 | 1.85161586876182e-06 | 7.95269015633202e-05 | 5.73244910587251 | 0.0592034445640474 | GABARAPL1//GABRA5//KCNB1//SGCE//GRIK2//MAPK1//TRAK2//CNN3//LPAR1//ARC//FMR1//ZMYND8//GPM6A//GRM3//KCNA4//KCNJ2//DOCK10//PRKAR2B//PSD//PTEN//ABHD17C//STRN//FXR1//CD3E//FLNA//SYNGAP1//CPEB3//GPHN//SLC9A6//KIF1C//CHRM2//CNGA3//DBN1//SAMD14//ADNP//NPTN//NSG1//KLHL20//GLRB//GNAQ//NRG1//NTF3//PURA//ELOVL5//KIF13A//CPEB1//BMPR2//LRP8//FZD3//GLRA3//C16ORF70//TMEM185A//TP63//BSN//HOMER1// |
| GO:0097447 | dendritic tree | Cellular component | 55 | 570 | 929 | 18698 | 1.94208071308519 | 1.95377077342974e-06 | 7.9918528303626e-05 | 5.70912639132058 | 0.0592034445640474 | GPHN//SLC9A6//KIF1C//CHRM2//CNGA3//DBN1//SAMD14//CPEB3//FMR1//ADNP//GABRA5//NPTN//NSG1//KLHL20//GLRB//GNAQ//NRG1//KCNB1//NTF3//PURA//ELOVL5//KIF13A//CPEB1//BMPR2//TRAK2//STRN//LRP8//FZD3//GLRA3//C16ORF70//TMEM185A//TP63//BSN//HOMER1//FLNA//GABARAPL1//SGCE//GRIK2//MAPK1//CNN3//LPAR1//ARC//ZMYND8//GPM6A//GRM3//KCNA4//KCNJ2//DOCK10//PRKAR2B//PSD//PTEN//ABHD17C//FXR1//CD3E//SYNGAP1// |
| GO:0043005 | neuron projection | Cellular component | 96 | 1224 | 929 | 18698 | 1.57858967052914 | 5.05411274568092e-06 | 0.000197340129479087 | 5.29635507419273 | 0.103336921420883 | KIF1C//DTNA//WDFY3//FMR1//ADNP//TNFRSF21//KLHL20//GRM3//KCNA4//KCNB1//NTF3//ROR1//MAPK1//SMURF1//KIF13A//SLC6A1//LRP8//FZD3//FXR1//BSN//HOMER1//FEZ2//GPHN//SLC9A6//CHRM2//CNGA3//DBN1//SAMD14//CPEB3//GABRA5//NPTN//NSG1//GLRB//GNAQ//NRG1//PURA//ELOVL5//CPEB1//BMPR2//TRAK2//STRN//GLRA3//C16ORF70//TMEM185A//TP63//EPS8//VTI1A//CDC14A//NFIB//FLNA//CNN3//LPAR1//ARC//ZMYND8//GPM6A//KCNJ2//DOCK10//PRKAR2B//PSD//PTEN//ABHD17C//CD3E//GABARAPL1//SGCE//GRIK2//SYNGAP1//ROBO2//KCNAB2//KCNK2//RAB21//SYBU//STMN2//PALLD//FKBP15//OTX2//SNAP25//TSC1//SCN1B//SPOCK1//STX6//ADCYAP1//PFN2//VAMP1//SYT1//DHRS3//CHRNB4//LYPD6//CPEB2//POU4F1//RAP1A//ITSN1//SLC6A11//RIC3//FOSL1//VAMP3//RAPGEF2// |
| GO:0044456 | synapse part | Cellular component | 64 | 740 | 929 | 18698 | 1.74071509615105 | 1.10897971481331e-05 | 0.000414179815228101 | 4.95507639778701 | 0.0688912809472551 | VTI1A//NTF3//RAB8B//SNAP25//SYT1//SYT6//SEMA4C//VAMP1//C16ORF70//FMR1//RIMS4//FZD3//PPFIA1//BSN//CPD//KCNJ10//WNT7A//RAB3GAP1//GABRA5//NSG1//PSD//GPHN//ZNRF2//NPTN//GRIK2//GRM3//FOSL1//CHRM2//CHRNB4//CPEB3//ARC//GLRB//KCNB1//PTEN//ABHD17C//CPEB1//STRN//GLRA3//DLG5//HOMER1//STX6//ADCYAP1//PFN2//SLC9A6//ROR1//KCNAB2//CNN3//LPAR1//ZMYND8//GPM6A//KCNA4//KCNJ2//DOCK10//PRKAR2B//FXR1//CD3E//SAMD14//EPS8//BMPR2//SPOCK1//LRP8//SYNGAP1//KCNK2//GSG1L// |
| GO:0044444 | cytoplasmic part | Cellular component | 541 | 9626 | 929 | 18698 | 1.13117773736675 | 1.32393278728139e-05 | 0.000473857610114464 | 4.87813406236894 | 0.582346609257266 | NCK1//PPP1R15B//GTPBP1//CCNY//TRAPPC8//PLEKHF2//ULK2//SEPT9//GLI3//ATG16L1//GJA1//PPP1R12A//CMC4//PYURF//CCDC58//PPARGC1B//DYNLL2//PPTC7//DDX6//PRSS35//ELK3//ACSL1//SIRT1//SLC44A1//GABARAPL1//CLIC4//TIMM9//GLRX//TCAIM//GRSF1//SLC25A6//COX8C//JARID2//BOLA3//MGST1//NKTR//OGG1//DCAF8//RAB8B//PMAIP1//PON2//MIEF1//NDFIP2//THG1L//PPP2CA//PPP3R1//MAPK1//SMURF1//PTEN//MAVS//RDH14//RAF1//MCCC2//SGK1//ACBD3//TRAK2//SOX4//YWHAG//ZNF217//APOO//PRR5L//ABHD11//CASP2//CCM2//TP63//BAG5//RANBP9//DNAJB6//C8ORF44-SGK3//SGK2//HIPK3//STX6//DNAJA2//SEC23A//TACC2//DCTN6//PTGES3//IQGAP2//COPS8//RALBP1//CKAP4//RAPGEF4//STRAP//AKAP11//EGLN3//KLF12//ARAP2//CLCN5//NEDD1//CNN3//ADH4//EDARADD//VPS37A//UBXN2B//CRKL//VTI1A//KRT80//SYT6//FBXO41//SH3D19//ADSS//SPRED1//DENND1B//DCP2//POLR3H//DYNC1LI2//DPYSL2//TXLNA//RAB12//DENND6A//PRSS55//EP300//EPS15//ESR1//PATL1//RASGEF1A//JAZF1//ZNRF2//ATF5//DZIP1//RAB3GAP1//RAB18//WDFY3//RAB21//PALLD//PDZRN3//CDK19//TNRC6B//FLI1//FLII//FLNA//EFR3A//RGL1//OTUD3//CAMSAP2//FMR1//DNAJC16//KPNA6//SGK3//CNOT6L//RICTOR//LCLAT1//GABRA5//CNEP1R1//PAN3//DNAJB5//ANAPC15//APPL1//TES//STEAP2//KLHL3//AGO1//GFPT1//SNX5//SERP1//ARFIP1//BBS9//SESN1//KLHL20//OSTM1//TBK1//MYLIP//GSTA2//GSTM3//GTF2A1//GTF2E1//CNOT7//NRBP1//PSAT1//HIC1//ANXA7//HPRT1//AGFG1//HSBP1//ID2//IMPDH1//IRF1//IRF2//AR//JUN//RHOB//USP27X//ARHGAP1//ARHGAP6//MBNL1//MEF2A//MID1//NR3C2//KRTAP5-6//MZT1//HSBP1L1//MSN//MTRR//NPAS2//ARHGEF4//PAX5//PCBP2//MEMO1//CUTC//SEPSECS//ABHD5//IRAK4//RAB9B//YTHDF2//PEX5L//CHMP3//CMPK1//SERPINB13//PIK3CA//PIK3CB//BRWD1//ERRFI1//RIN2//WDR44//EGLN1//PPARG//AFTPH//MTMR10//PXK//PPP2CB//SEC61A2//PPP2R5E//FBXW7//DOCK10//PRKAA1//PRKACB//ENAH//PRKAR2B//TRERF1//MAPK6//NXF2//GPCPD1//WDR45B//PSMA2//SPHK2//KCMF1//PSMD12//PTHLH//CNOT6//RIMKLB//KLHL42//TAOK1//RANBP10//ZBTB4//RAP2C//MID1IP1//RRAGD//RAP1A//RAP1B//RASA1//RBMS1//CCND1//CLIP1//SARS//ATXN1//ATXN7//PRDM16//SRSF2//CPEB1//ITSN1//S100PBP//NABP1//SKP1//CDK15//MTMR9//SNAP25//BNIP2//SNX2//SOS2//HSPA13//VAMP1//SYT1//BTF3//ZEB1//TFAP2A//TFE3//TGFBR2//THOP1//TIA1//TMOD1//TNFAIP3//TNNI1//TSN//TSC1//TUB//NXF2B//UBE2I//UBE2V1//VBP1//WT1//YES1//ZYX//CSDE1//SLBP//BAG6//CENPO//E2F8//SNIP1//CBLL1//UBA5//CCDC6//KCNIP4//FOSL1//CUL5//PTP4A2//FXR1//SNX27//SBF2//CAST//HIST2H2BE//FBXO30//SYDE2//PPP1R1B//MEX3B//DCTN5//KBTBD8//TEAD2//PPFIA1//KCNAB2//DGKE//KRTAP2-4//ALG2//CDC14A//RUNX3//SOCS1//DDX3Y//PDE5A//SYNGAP1//AP1S2//SKAP2//CCND2//LMLN//SOCS3//CCNT2//CCT6A//NMI//LONRF1//MOB1B//FAM114A1//VAMP3//FBXO44//ARHGAP12//HOMER1//VPS4B//EI24//CEP41//CLOCK//PPM1F//SOCS5//RAPGEF2//RASSF2//SNX17//GIT2//C2CD5//G3BP2//WDR1//RTN3//CDS1//CRTAP//KIF1C//TMED1//B3GALNT2//TMEM64//SMIM14//TAPT1//SULF1//NSG1//ZDHHC22//YIPF6//KDR//FIBIN//SHISA2//LRP6//FAM19A1//DNAJB9//IER3IP1//PLP2//UGT2B28//ZDHHC7//PHTF2//STIM2//ELOVL5//RTN1//MSMO1//TMBIM6//PTP4A1//ELOVL7//ANP32A//CALU//ZDHHC18//FUT9//ADCY3//STMN2//SPPL3//CPD//ESCO2//MMD//FUT2//FUT5//ATP2C1//GOLGA6A//GLT6D1//KCNJ2//LDLR//SLC30A6//GNPTG//SGCE//CCDC126//MMGT1//TJAP1//SS18//DLL1//NRBF2//RND3//NTF3//CHIC1//SYBU//CXXC4//CNIH1//MCFD2//SERINC3//CABP7//CD2AP//PPP1R16B//NANOS1//INHBB//RASD1//STK33//CALN1//SLK//TMOD3//NPNT//TBC1D12//SLC26A7//CRHR1//DGKH//S1PR1//LPAR1//ARC//ARRDC3//EDN1//PNRC1//TDRD5//ACBD5//NLN//FASTK//HS3ST5//RAB30//ST8SIA5//TRIM23//GCNT4//PMEPA1//RIC3//CHST1//ST3GAL5//DSEL//HS3ST3A1//OGN//PRELP//SDC1//WNT3//WNT7A//HS3ST1//PIGP//PIGA//EOGT//IGFBP3//KDELC1//GJC1//LPCAT3//SLC9A6//RDH10//SPTSSB//SPTSSA//ACSL4//REEP3//SLC35D1//ARL6IP1//KCNK2//ASNA1//INSIG2//JKAMP//UGT2B7//DHRS3//EMC7//ZPLD1//SNX19//FZD6//GPRC5A//SERPINI1//ARMC8//DBN1//CALM2//CFL2//KCNE1//ZMYND10//EPS8//MARCKS//KIF13A//NAGA//NEU1//DRAM1//CTSF//ADRB1//FKBP15//RNF11//SLC31A2//TNF//CLCN6//GNAQ//C6ORF120//TEX261//IGF1//RIMS4//C5AR1//CHRM2//BRI3//CHRNB4//SIRPA//LASP1//BSN//LBR//KLK15//MTX3//ARMCX6//BCL2A1//ABCB7//SLC25A34//MICU3//SLC25A28//GRIK2//COX7A2//C16ORF70//LDLRAD4//ABHD17C//PSD//IMMP2L//NHLRC2//VEGFB//SEMA4C//ARMCX3// |
| GO:0120025 | plasma membrane bounded cell projection | Cellular component | 138 | 1980 | 929 | 18698 | 1.40279218449294 | 2.17132408558501e-05 | 0.000746066955807009 | 4.66327535016288 | 0.148546824542519 | PALLD//CD2AP//SNX5//TNFRSF12A//RASA1//CLIP1//MSN//ADCY3//DYNLL2//LCA5//EVC//BBS9//GLI3//TUB//CEP41//KIF1C//STMN2//CHRNB4//LYPD6//CPEB2//CPEB3//FMR1//GABRA5//GLRB//GPM6A//POU4F1//PTEN//RAP1A//KIF13A//CPEB1//ITSN1//SLC6A1//SLC6A11//SNAP25//SYT1//RIC3//GLRA3//FOSL1//VAMP3//HOMER1//RAPGEF2//IQGAP2//WASF3//FAM89B//NRBP1//PIK3CA//ENAH//SNX2//TSC1//MAPK1//RAF1//SEPT9//DTNA//WDFY3//ADNP//TNFRSF21//KLHL20//GRM3//KCNA4//KCNB1//NTF3//ROR1//SMURF1//LRP8//FZD3//FXR1//BSN//FEZ2//GPHN//SLC9A6//CHRM2//CNGA3//DBN1//SAMD14//NPTN//NSG1//GNAQ//NRG1//PURA//ELOVL5//BMPR2//TRAK2//STRN//C16ORF70//TMEM185A//TP63//EPS8//CDC14A//KCNK2//CLIC4//FOXA1//SLC10A2//CNN3//LPAR1//ARC//ZMYND8//KCNJ2//DOCK10//PRKAR2B//PSD//ABHD17C//CD3E//NFIB//FLNA//RNF38//ARHGEF4//C2CD5//VTI1A//GABARAPL1//SGCE//GRIK2//SYNGAP1//DDX6//GSTM3//ATG16L1//FKBP15//OTX2//ROBO2//SLC26A4//SCN1B//SPOCK1//RAB28//TMEM17//C5ORF30//NEDD1//TAPT1//DZIP1//DLG5//DHRS3//STX6//ADCYAP1//PFN2//VAMP1//KCNAB2//EPS15//PRKACB//RAB21//SYBU// |
| GO:0008287 | protein serine/threonine phosphatase complex | Cellular component | 11 | 50 | 929 | 18698 | 4.42794402583423 | 2.69452906646352e-05 | 0.000857259432626727 | 4.56951712721487 | 0.0118406889128095 | IER5//PPP2CA//PPP2CB//PPP2R5E//STRN//NCK1//PPP1R15B//PPP3R1//PPP4R2//CNEP1R1//PPP1R12A// |
| GO:1903293 | phosphatase complex | Cellular component | 11 | 50 | 929 | 18698 | 4.42794402583423 | 2.69452906646352e-05 | 0.000857259432626727 | 4.56951712721487 | 0.0118406889128095 | IER5//PPP2CA//PPP2CB//PPP2R5E//STRN//NCK1//PPP1R15B//PPP3R1//PPP4R2//CNEP1R1//PPP1R12A// |
| GO:0098794 | postsynapse | Cellular component | 43 | 450 | 929 | 18698 | 1.92324841526133 | 3.21857569310433e-05 | 0.000964649833388788 | 4.49233627286064 | 0.046286329386437 | CNN3//LPAR1//ARC//FMR1//ZMYND8//GPM6A//GRM3//KCNA4//KCNJ2//DOCK10//PRKAR2B//PSD//PTEN//ABHD17C//STRN//FXR1//CD3E//GPHN//CHRM2//CHRNB4//CPEB3//GABRA5//NSG1//GLRB//GRIK2//KCNB1//SEMA4C//CPEB1//GLRA3//DLG5//HOMER1//SAMD14//EPS8//NPTN//BMPR2//SPOCK1//LRP8//KCNAB2//SYNGAP1//BSN//GSG1L//RAB3GAP1//SNAP25// |
| GO:0030424 | axon | Cellular component | 49 | 538 | 929 | 18698 | 1.8331299194481 | 3.25667580538706e-05 | 0.000964649833388788 | 4.48722547230378 | 0.0527448869752422 | NFIB//STMN2//DBN1//EPS8//PALLD//FKBP15//FMR1//OTX2//SNAP25//TSC1//NRG1//ROBO2//SCN1B//SPOCK1//STX6//ADCYAP1//GRIK2//PFN2//VAMP1//SYT1//SLC9A6//CHRM2//ROR1//KCNAB2//GPM6A//TRAK2//KCNK2//RAB21//SYBU//KIF1C//DTNA//WDFY3//ADNP//TNFRSF21//KLHL20//GRM3//KCNA4//KCNB1//NTF3//MAPK1//SMURF1//KIF13A//SLC6A1//LRP8//FZD3//FXR1//BSN//HOMER1//FEZ2// |
| GO:0042995 | cell projection | Cellular component | 141 | 2055 | 929 | 18698 | 1.3809778979045 | 3.77854080200427e-05 | 0.00108192218297389 | 4.42267588375727 | 0.151776103336921 | PALLD//CD2AP//SNX5//TNFRSF12A//RASA1//CLIP1//MSN//ADCY3//DYNLL2//LCA5//EVC//BBS9//GLI3//TUB//CEP41//KIF1C//STMN2//CHRNB4//LYPD6//CPEB2//CPEB3//FMR1//GABRA5//GLRB//GPM6A//POU4F1//PTEN//RAP1A//KIF13A//CPEB1//ITSN1//SLC6A1//SLC6A11//SNAP25//SYT1//RIC3//GLRA3//FOSL1//VAMP3//HOMER1//RAPGEF2//IQGAP2//WASF3//FAM89B//NRBP1//PIK3CA//ENAH//SNX2//TSC1//MAPK1//RAF1//SEPT9//DTNA//WDFY3//ADNP//TNFRSF21//KLHL20//GRM3//KCNA4//KCNB1//NTF3//ROR1//SMURF1//LRP8//FZD3//FXR1//BSN//FEZ2//GPHN//SLC9A6//CHRM2//CNGA3//DBN1//SAMD14//NPTN//NSG1//GNAQ//NRG1//PURA//ELOVL5//BMPR2//TRAK2//STRN//C16ORF70//TMEM185A//TP63//EPS8//CNN3//LPAR1//ARC//ZMYND8//KCNJ2//DOCK10//PRKAR2B//PSD//ABHD17C//CD3E//NFIB//CDC14A//FLNA//KCNK2//CLIC4//FOXA1//SLC10A2//RNF38//ARHGEF4//C2CD5//VTI1A//GABARAPL1//SGCE//GRIK2//SYNGAP1//DDX6//GSTM3//ATG16L1//FKBP15//OTX2//ROBO2//SLC26A4//SCN1B//SPOCK1//RAB28//TMEM17//C5ORF30//NEDD1//TAPT1//DZIP1//DLG5//DHRS3//STX6//ADCYAP1//PFN2//VAMP1//KCNAB2//EPS15//PRKACB//RAB21//SYBU//PPP1R16B//AGFG1//WDR1// |
| GO:0030014 | CCR4-NOT complex | Cellular component | 6 | 16 | 929 | 18698 | 7.54763186221744 | 7.71122246590885e-05 | 0.00213675487039216 | 4.11287676745961 | 0.00645855758880517 | CNOT7//TOB1//PATL1//CPEB3//CNOT6L//CNOT6// |
| GO:0098805 | whole membrane | Cellular component | 114 | 1630 | 929 | 18698 | 1.40765649454853 | 0.000106818156848242 | 0.00278591962130477 | 3.97135492007165 | 0.122712594187298 | PLEKHF2//SBF2//IQGAP2//RAB18//RAB9B//RAB8B//C5AR1//CLCN5//CLCN6//VPS37A//ZNRF2//APPL1//STEAP2//TBK1//RHOB//ARHGAP1//LDLR//IRAK4//WDR44//PMEPA1//KIF13A//SNX2//VPS4B//SNX17//SPPL3//GJA1//WNT3//WNT7A//ATG16L1//WDR45B//ULK2//GABARAPL1//CNIH1//SEC23A//VTI1A//MCFD2//SYT1//CKAP4//BRI3//MGST1//RAP1B//CHRNB4//RAP1A//SNAP25//VAMP1//KCNAB2//ANXA7//SIRPA//RAP2C//SLC9A6//EPS15//RAB21//NSG1//SNX5//SNX19//LRP6//LDLRAD4//SNX27//MMGT1//MMD//CHMP3//SLC26A7//RAB12//ABHD17C//VAMP3//CHRM2//ACSL1//ACSL4//PEX5L//MAVS//ACBD5//UGT2B28//NAV3//SLC44A1//MTX3//PMAIP1//ARMCX6//MIEF1//RAF1//BCL2A1//GNAQ//OSTM1//TRIM23//NEU1//DRAM1//SPHK2//RDH14//AP1S2//SYT6//SEMA4C//C16ORF70//NDFIP2//WDFY3//RRAGD//PSD//SPRED1//MAPK1//BMPR2//TGFBR2//LRP8//CD8A//LYPD6//S1PR1//EDNRB//UNC5B//SULF1//DLL1//KCNE1//KDR//PRKAR2B//TNF//ARMCX3//STX6//AFTPH// |
| GO:0005768 | endosome | Cellular component | 69 | 878 | 929 | 18698 | 1.58173606224147 | 0.000107026015719508 | 0.00278591962130477 | 3.97051064184652 | 0.0742734122712594 | C8ORF44-SGK3//STX6//ADRB1//RAB21//FKBP15//SGK3//APPL1//STEAP2//GJA1//RNF11//KDR//RHOB//LDLR//LRP6//MAPK1//ARRDC3//RAP1A//TRAK2//PTP4A1//PTP4A2//SNX27//MMGT1//SNX17//SLC9A6//SLC31A2//DYNC1LI2//NSG1//CHMP3//RAPGEF2//DENND6A//TBC1D12//TNF//VAMP3//ARHGAP1//CLIP1//EPS15//SNX5//SNX19//PMEPA1//SNX2//LDLRAD4//PLEKHF2//VPS37A//VTI1A//MMD//VPS4B//NDFIP2//SLC26A7//RAB12//RAB8B//RAP2C//ABHD17C//CLCN5//CLCN6//ZNRF2//TBK1//IRAK4//WDR44//KIF13A//RRAGD//PSD//STMN2//CRHR1//CRKL//DGKH//S1PR1//LPAR1//ARC//TJAP1// |
| GO:0044463 | cell projection part | Cellular component | 99 | 1377 | 929 | 18698 | 1.44704053131838 | 0.000115724471881382 | 0.00284020918131735 | 3.93657479248099 | 0.106566200215285 | GPHN//SLC9A6//KIF1C//CHRM2//CNGA3//DBN1//SAMD14//CPEB3//FMR1//ADNP//GABRA5//NPTN//NSG1//KLHL20//GLRB//GNAQ//NRG1//KCNB1//NTF3//PURA//ELOVL5//KIF13A//CPEB1//BMPR2//TRAK2//STRN//LRP8//FZD3//GLRA3//C16ORF70//TMEM185A//TP63//BSN//HOMER1//EPS8//CNN3//LPAR1//ARC//ZMYND8//GPM6A//GRM3//KCNA4//KCNJ2//DOCK10//PRKAR2B//PSD//PTEN//ABHD17C//FXR1//CD3E//FLNA//SYNGAP1//VTI1A//ROBO2//SLC26A4//MSN//ARHGEF4//C2CD5//GABARAPL1//SGCE//DHRS3//TMEM17//EPS15//EVC//BBS9//GRIK2//MAPK1//RAB21//SYBU//STMN2//PALLD//FKBP15//OTX2//SNAP25//TSC1//SCN1B//SPOCK1//STX6//ADCYAP1//PFN2//VAMP1//SYT1//ROR1//KCNAB2//KCNK2//DDX6//SEPT9//GLI3//ATG16L1//RAB28//GSTM3//C5ORF30//NEDD1//TAPT1//DZIP1//DLG5//CEP41//DYNLL2//PRKACB// |
| GO:0120038 | plasma membrane bounded cell projection part | Cellular component | 99 | 1377 | 929 | 18698 | 1.44704053131838 | 0.000115724471881382 | 0.00284020918131735 | 3.93657479248099 | 0.106566200215285 | GPHN//SLC9A6//KIF1C//CHRM2//CNGA3//DBN1//SAMD14//CPEB3//FMR1//ADNP//GABRA5//NPTN//NSG1//KLHL20//GLRB//GNAQ//NRG1//KCNB1//NTF3//PURA//ELOVL5//KIF13A//CPEB1//BMPR2//TRAK2//STRN//LRP8//FZD3//GLRA3//C16ORF70//TMEM185A//TP63//BSN//HOMER1//EPS8//CNN3//LPAR1//ARC//ZMYND8//GPM6A//GRM3//KCNA4//KCNJ2//DOCK10//PRKAR2B//PSD//PTEN//ABHD17C//FXR1//CD3E//FLNA//SYNGAP1//VTI1A//SLC26A4//MSN//ARHGEF4//C2CD5//TMEM17//EPS15//EVC//BBS9//STMN2//PALLD//FKBP15//OTX2//SNAP25//TSC1//ROBO2//SCN1B//SPOCK1//STX6//ADCYAP1//GRIK2//PFN2//VAMP1//SYT1//ROR1//KCNAB2//RAB21//SYBU//DDX6//SEPT9//GLI3//ATG16L1//RAB28//GSTM3//C5ORF30//NEDD1//TAPT1//DZIP1//DLG5//CEP41//DYNLL2//PRKACB//GABARAPL1//SGCE//DHRS3//MAPK1//KCNK2// |
| GO:0099572 | postsynaptic specialization | Cellular component | 25 | 229 | 929 | 18698 | 2.19727274009241 | 0.000191350088003319 | 0.00456582571096808 | 3.7181713337042 | 0.0269106566200215 | CNN3//SAMD14//EPS8//CPEB3//ARC//FMR1//NPTN//GRIK2//GRM3//SEMA4C//PSD//ABHD17C//CPEB1//BMPR2//SPOCK1//STRN//LRP8//FXR1//KCNAB2//SYNGAP1//BSN//DLG5//HOMER1//GSG1L//GPHN// |
| GO:0032279 | asymmetric synapse | Cellular component | 25 | 232 | 929 | 18698 | 2.16885973052225 | 0.000234278586199778 | 0.00543906231204349 | 3.63026740547167 | 0.0269106566200215 | CNN3//SAMD14//EPS8//CPEB3//ARC//FMR1//NPTN//GRIK2//GRM3//SEMA4C//PSD//ABHD17C//CPEB1//BMPR2//SPOCK1//STRN//LRP8//FXR1//KCNAB2//SYNGAP1//BSN//DLG5//HOMER1//GSG1L//CHRM2// |
| GO:0005667 | transcription factor complex | Cellular component | 33 | 345 | 929 | 18698 | 1.92519305471053 | 0.000253409052271395 | 0.00572837831318759 | 3.5961778753196 | 0.0355220667384284 | SOX4//NR5A2//NEUROD1//PPARG//RORA//BDP1//GTF2A1//GTF2E1//GTF2H1//RB1//JUN//CEBPB//TEAD2//GSC//E2F5//EP300//ETS1//ATF5//FOXF1//FOXF2//MEF2A//MYOG//NHLH2//NPAS2//NR4A2//ASCC1//PKNOX1//POU3F2//TRERF1//ZEB1//E2F8//TP63//CLOCK// |
| GO:0098984 | neuron to neuron synapse | Cellular component | 25 | 235 | 929 | 18698 | 2.14117215949431 | 0.00028551767407575 | 0.00628870979566844 | 3.5443670029488 | 0.0269106566200215 | CHRM2//CNN3//SAMD14//EPS8//CPEB3//ARC//FMR1//NPTN//GRIK2//GRM3//SEMA4C//PSD//ABHD17C//CPEB1//BMPR2//SPOCK1//STRN//LRP8//FXR1//KCNAB2//SYNGAP1//BSN//DLG5//HOMER1//GSG1L// |
| GO:0005769 | early endosome | Cellular component | 32 | 334 | 929 | 18698 | 1.92833708256254 | 0.000303394500360894 | 0.00649701426458594 | 3.51799229594403 | 0.0344456404736276 | SLC9A6//EPS15//RAB21//APPL1//NSG1//SNX5//SNX19//LRP6//PMEPA1//SNX2//LDLRAD4//PLEKHF2//SNX27//MMGT1//C8ORF44-SGK3//STX6//ADRB1//FKBP15//SGK3//STEAP2//GJA1//RNF11//KDR//RHOB//LDLR//MAPK1//ARRDC3//RAP1A//TRAK2//PTP4A1//PTP4A2//SNX17// |
| GO:0044297 | cell body | Cellular component | 44 | 513 | 929 | 18698 | 1.72629396718683 | 0.000310101961406314 | 0.00649701426458594 | 3.50849548682541 | 0.0473627556512379 | STMN2//CHRM2//CNN3//CPD//VTI1A//LPAR1//ACSL4//FLNA//ARC//FMR1//ADNP//GPM6A//AGFG1//KCNJ2//KCNK2//LRP6//SERPINI1//PRKAR2B//SMURF1//PURA//ELOVL5//BMPR2//TRAK2//STRN//LRP8//RIC3//FZD3//FXR1//PPP1R1B//RAPGEF2//GABRA5//KCNB1//CNGA3//RBFOX3//WDFY3//GRIK2//NEUROG1//MAPK1//GLRA3//RDH10//GABARAPL1//GNAQ//NRG1//CD3E// |
| GO:0035770 | ribonucleoprotein granule | Cellular component | 22 | 200 | 929 | 18698 | 2.21397201291711 | 0.000407803058273922 | 0.0081918284716574 | 3.38954952160224 | 0.0236813778256189 | CKAP4//DDX6//DCP2//PATL1//FMR1//YTHDF2//FXR1//SOCS1//DDX3Y//TIA1//PNRC1//TNRC6B//PAN3//AGO1//CNOT7//PSMA2//CPEB1//MEX3B//MBNL1//TDRD5//CLOCK//GRSF1// |
| GO:0014069 | postsynaptic density | Cellular component | 24 | 227 | 929 | 18698 | 2.12796669243135 | 0.00041006824712604 | 0.0081918284716574 | 3.3871438581981 | 0.0258342303552207 | GRIK2//GSG1L//CNN3//SAMD14//EPS8//CPEB3//ARC//FMR1//NPTN//GRM3//SEMA4C//PSD//ABHD17C//CPEB1//BMPR2//SPOCK1//STRN//LRP8//FXR1//KCNAB2//SYNGAP1//BSN//DLG5//HOMER1// |
| GO:0043025 | neuronal cell body | Cellular component | 39 | 449 | 929 | 18698 | 1.74822653378756 | 0.000522248270129616 | 0.0101957105463941 | 3.2821229898817 | 0.0419806243272336 | GABRA5//KCNB1//CNGA3//RBFOX3//WDFY3//FMR1//GRIK2//NEUROG1//SERPINI1//MAPK1//GLRA3//STMN2//CHRM2//CNN3//CPD//VTI1A//LPAR1//ACSL4//FLNA//ARC//ADNP//GPM6A//AGFG1//KCNJ2//KCNK2//LRP6//PRKAR2B//SMURF1//PURA//ELOVL5//BMPR2//TRAK2//STRN//LRP8//RIC3//FZD3//FXR1//PPP1R1B//RAPGEF2// |
| GO:0036464 | cytoplasmic ribonucleoprotein granule | Cellular component | 21 | 191 | 929 | 18698 | 2.21291824232553 | 0.000549945800126586 | 0.0104978542735275 | 3.25968011026182 | 0.0226049515608181 | PNRC1//DDX6//DCP2//PATL1//TNRC6B//PAN3//AGO1//CNOT7//YTHDF2//PSMA2//CPEB1//MEX3B//FMR1//MBNL1//TIA1//TDRD5//CLOCK//FXR1//CKAP4//SOCS1//DDX3Y// |
| GO:0010008 | endosome membrane | Cellular component | 39 | 451 | 929 | 18698 | 1.74047386623196 | 0.000569203674293076 | 0.0106292599177772 | 3.24473230514422 | 0.0419806243272336 | VPS37A//CHMP3//SNX5//SLC9A6//EPS15//RAB21//APPL1//NSG1//SNX19//LRP6//PMEPA1//SNX2//LDLRAD4//PLEKHF2//SNX27//MMGT1//VTI1A//MMD//RHOB//VPS4B//LDLR//SLC26A7//RAB12//RAB8B//RAP2C//ABHD17C//VAMP3//NDFIP2//PSD//CLCN5//CLCN6//ZNRF2//STEAP2//TBK1//ARHGAP1//IRAK4//WDR44//KIF13A//SNX17// |
| GO:0098588 | bounding membrane of organelle | Cellular component | 133 | 2040 | 929 | 18698 | 1.31220266362735 | 0.000582522907106758 | 0.0106465356852065 | 3.23468699178786 | 0.143164693218515 | CNIH1//STX6//RTN3//SEC23A//FUT9//SERINC3//CLCN5//VTI1A//B3GALNT2//RAB12//HS3ST5//FUT5//GJA1//ATP2C1//ARFIP1//RAB30//YIPF6//ST8SIA5//TRIM23//RND3//GCNT4//NDFIP2//ZDHHC7//SYBU//SLC30A6//PMEPA1//ACBD3//APOO//RIC3//GNPTG//CHST1//ST3GAL5//AP1S2//MCFD2//DSEL//MMGT1//HS3ST3A1//PLEKHF2//SBF2//IQGAP2//RAB18//RAB9B//RAB8B//C5AR1//CLCN6//VPS37A//ZNRF2//APPL1//STEAP2//TBK1//RHOB//ARHGAP1//LDLR//IRAK4//WDR44//KIF13A//SNX2//VPS4B//SNX17//SPPL3//WNT3//WNT7A//TMED1//TMEM17//EPS15//EVC//BBS9//RAB21//FUT2//NSG1//GOLGA6A//CABP7//CALN1//VAMP3//GABARAPL1//ATG16L1//SYT1//CKAP4//BRI3//MGST1//RAP1B//CHRNB4//RAP1A//SNAP25//VAMP1//KCNAB2//ANXA7//SIRPA//RAP2C//SLC9A6//SNX5//SNX19//LRP6//LDLRAD4//SNX27//MMD//CHMP3//SLC26A7//ABHD17C//CHRM2//ACSL1//ACSL4//PEX5L//MAVS//ACBD5//UGT2B28//NAV3//SLC44A1//MTX3//PMAIP1//ARMCX6//MIEF1//RAF1//BCL2A1//CNGA3//DHRS3//GNAQ//OSTM1//NEU1//DRAM1//SPHK2//RDH14//SYT6//SEMA4C//C16ORF70//WDFY3//RRAGD//PSD//TEX261//IER3IP1//ARMCX3//ARL6IP1//AFTPH// |
| GO:0044309 | neuron spine | Cellular component | 18 | 155 | 929 | 18698 | 2.33733115733185 | 0.000701671387864971 | 0.0125569942120002 | 3.15386623238626 | 0.0193756727664155 | CNN3//LPAR1//ARC//FMR1//ZMYND8//GPM6A//GRM3//KCNA4//KCNJ2//DOCK10//PRKAR2B//PSD//PTEN//ABHD17C//STRN//FXR1//CD3E//SLC9A6// |
| GO:0000932 | P-body | Cellular component | 12 | 84 | 929 | 18698 | 2.87528832846379 | 0.000877709402488832 | 0.0153867831987328 | 3.05664924922556 | 0.0129171151776103 | PNRC1//DDX6//DCP2//PATL1//TNRC6B//PAN3//AGO1//CNOT7//YTHDF2//PSMA2//CPEB1//MEX3B// |
| GO:0017053 | transcriptional repressor complex | Cellular component | 12 | 85 | 929 | 18698 | 2.84146140695245 | 0.000976713178521726 | 0.0167799324070033 | 3.01023295242594 | 0.0129171151776103 | BAHD1//SIRT1//SUV39H1//APPL1//JAZF1//GLI3//JUN//MIER1//RBBP8//CCND1//PRDM16//TBL1XR1// |
| GO:0031974 | membrane-enclosed lumen | Cellular component | 299 | 5188 | 929 | 18698 | 1.15998043012234 | 0.00124876472954377 | 0.0202394132580773 | 2.90351937610582 | 0.321851453175457 | IMPDH1//SERPINI1//PSMA2//PSMD12//FASTK//MCCC2//CRTAP//CKAP4//EOGT//IGFBP3//DNAJB9//WNT3//WNT7A//KDELC1//CALU//OGN//PRELP//SDC1//HS3ST1//ARMC8//MAPK1//KCMF1//CMC4//TIMM9//NLN//THOP1//C6ORF120//NSG1//NEU1//SERPINB13//CTSF//EDN1//IGF1//NHLRC2//VEGFB//GRSF1//ZBTB18//JUN//DNAJB6//SGK2//MBNL2//STX6//CEBPB//SIVA1//TACC2//CELF1//PTGES3//ZMYND11//ARPP19//TCERG1//COPS8//RPP14//STRAP//EGLN3//KLF12//MBD6//PPARGC1B//VPS37A//CRKL//PHF13//PDIK1L//PPP4R2//RNF38//SH3D19//CREBRF//ESCO2//GADD45A//DCP2//POLR3H//DNMT3B//E2F5//S1PR1//ZNF367//ARID2//ELAVL2//CC2D1B//ELK3//EP300//ESR1//ETS1//ETV5//ATF5//DZIP1//BAHD1//ELL2//KDM2A//PDZRN3//PHF8//FLII//SIN3B//FMR1//SIRT1//ZFPM2//SLC44A1//MORC3//SMUG1//KPNA6//NR5A2//NAALADL2//GABRA5//ANAPC15//AGO1//HBP1//GJA1//RNF11//FOXP1//ZBTB11//GLI3//ATAD2//TBK1//GTF2A1//GTF2E1//GTF2H1//NRBP1//NRBF2//NRG1//HIC1//HIVEP2//FOXA1//HOXA3//HOXD1//HSBP1//IRF1//IRF2//AR//ISL1//JARID2//MBNL1//MDM4//MEF2A//NR3C2//MYB//MYOG//PPP1R12A//NEUROD1//NFIA//NFE2//NKTR//NPAS2//NPAT//NR4A2//OGG1//DCAF8//PAX5//PCBP2//ASCC1//CUTC//KLF3//CTDSPL2//ARID4B//RAB8B//BRWD1//POU4F1//PPARG//BNC2//RPP25//UBE2W//FBXW7//PPP3R1//DOCK10//PRKAA1//INTS8//PRKACB//TRERF1//BDP1//MAPK6//NXF2//SMURF1//PTEN//PTHLH//USP28//ZBTB4//MIER1//RRAGD//RB1//RBBP8//CCND1//RNF2//RORA//ATXN1//ATXN7//PRDM16//SRSF2//SRSF6//ZMAT3//CPEB1//NABP1//SKP1//BMPR2//SMARCA2//SMARCD2//SOX4//SUV39H1//ZEB1//TFAP2A//TFE3//TGIF1//TIA1//NXF2B//UBE2I//UBE2V1//WT1//LDLRAD4//ZNF131//ZNF217//SLBP//BAG6//CENPO//SAP30L//TBL1XR1//E2F8//SNIP1//ADAM12//FOSL1//ANP32A//SNX27//HIST2H2BE//ARID5B//MEX3B//PCGF5//LCOR//DCTN5//TEAD2//CDC14A//TP63//RTCA//RUNX3//CBFB//SOCS1//SKAP2//CCND2//BTAF1//CCNT2//NMI//ACBD5//WDR20//RPS6KA5//FAM114A1//CLOCK//RASSF2//GIT2//AKAP11//DDX6//TMEM217//WDFY3//FLNA//MDFIC//YPEL2//ASNA1//MYCN//NOVA1//CMPK1//PIK3CB//SLC14A1//ZNF655//FXR1//DDX3Y//GPRC5A//MOB1B//TNPO2//CFL2//CLIC4//PURA//H2AFZ//SYCE1//APPL1//SS18//AFF1//FAM118B//MED19//CDK19//MED9//MED22//HIPK3//GSC//FLI1//CNOT7//BHLHE40//PATL1//KLHL20//FAM76B//DENND1B//PPP1R16B//MEOX2//SLC2A4RG//CCNL1//RBM25//RAF1//SGK1//S100PBP//CBLL1//DAZAP2//EPC2//JAZF1//SESN1//NFIB//NFIC//FOXJ2//PAK6// |
| GO:0043233 | organelle lumen | Cellular component | 299 | 5188 | 929 | 18698 | 1.15998043012234 | 0.00124876472954377 | 0.0202394132580773 | 2.90351937610582 | 0.321851453175457 | SERPINI1//FASTK//MCCC2//CRTAP//CKAP4//EOGT//IGFBP3//DNAJB9//WNT3//WNT7A//KDELC1//CALU//OGN//PRELP//SDC1//HS3ST1//ARMC8//IMPDH1//MAPK1//PSMA2//KCMF1//PSMD12//C6ORF120//NSG1//NEU1//SERPINB13//CTSF//EDN1//CMC4//TIMM9//NLN//THOP1//IGF1//NHLRC2//VEGFB//GRSF1//ZBTB18//JUN//DNAJB6//SGK2//MBNL2//STX6//CEBPB//SIVA1//TACC2//CELF1//PTGES3//ZMYND11//ARPP19//TCERG1//COPS8//RPP14//STRAP//EGLN3//KLF12//MBD6//PPARGC1B//VPS37A//CRKL//PHF13//PDIK1L//PPP4R2//RNF38//SH3D19//CREBRF//ESCO2//GADD45A//DCP2//POLR3H//DNMT3B//E2F5//S1PR1//ZNF367//ARID2//ELAVL2//CC2D1B//ELK3//EP300//ESR1//ETS1//ETV5//ATF5//DZIP1//BAHD1//ELL2//KDM2A//PDZRN3//PHF8//FLII//SIN3B//FMR1//SIRT1//ZFPM2//SLC44A1//MORC3//SMUG1//KPNA6//NR5A2//NAALADL2//GABRA5//ANAPC15//AGO1//HBP1//GJA1//RNF11//FOXP1//ZBTB11//GLI3//ATAD2//TBK1//GTF2A1//GTF2E1//GTF2H1//NRBP1//NRBF2//NRG1//HIC1//HIVEP2//FOXA1//HOXA3//HOXD1//HSBP1//IRF1//IRF2//AR//ISL1//JARID2//MBNL1//MDM4//MEF2A//NR3C2//MYB//MYOG//PPP1R12A//NEUROD1//NFIA//NFE2//NKTR//NPAS2//NPAT//NR4A2//OGG1//DCAF8//PAX5//PCBP2//ASCC1//CUTC//KLF3//CTDSPL2//ARID4B//RAB8B//BRWD1//POU4F1//PPARG//BNC2//RPP25//UBE2W//FBXW7//PPP3R1//DOCK10//PRKAA1//INTS8//PRKACB//TRERF1//BDP1//MAPK6//NXF2//SMURF1//PTEN//PTHLH//USP28//ZBTB4//MIER1//RRAGD//RB1//RBBP8//CCND1//RNF2//RORA//ATXN1//ATXN7//PRDM16//SRSF2//SRSF6//ZMAT3//CPEB1//NABP1//SKP1//BMPR2//SMARCA2//SMARCD2//SOX4//SUV39H1//ZEB1//TFAP2A//TFE3//TGIF1//TIA1//NXF2B//UBE2I//UBE2V1//WT1//LDLRAD4//ZNF131//ZNF217//SLBP//BAG6//CENPO//SAP30L//TBL1XR1//E2F8//SNIP1//ADAM12//FOSL1//ANP32A//SNX27//HIST2H2BE//ARID5B//MEX3B//PCGF5//LCOR//DCTN5//TEAD2//CDC14A//TP63//RTCA//RUNX3//CBFB//SOCS1//SKAP2//CCND2//BTAF1//CCNT2//NMI//ACBD5//WDR20//RPS6KA5//FAM114A1//CLOCK//RASSF2//GIT2//AKAP11//DDX6//TMEM217//WDFY3//FLNA//MDFIC//YPEL2//ASNA1//MYCN//NOVA1//CMPK1//PIK3CB//SLC14A1//ZNF655//FXR1//DDX3Y//GPRC5A//MOB1B//TNPO2//CFL2//CLIC4//PURA//H2AFZ//SYCE1//APPL1//SS18//AFF1//FAM118B//MED19//CDK19//MED9//MED22//HIPK3//GSC//FLI1//CNOT7//BHLHE40//PATL1//KLHL20//FAM76B//DENND1B//PPP1R16B//MEOX2//SLC2A4RG//CCNL1//RBM25//RAF1//SGK1//S100PBP//CBLL1//DAZAP2//EPC2//JAZF1//SESN1//NFIB//NFIC//FOXJ2//PAK6// |
| GO:0070013 | intracellular organelle lumen | Cellular component | 299 | 5188 | 929 | 18698 | 1.15998043012234 | 0.00124876472954377 | 0.0202394132580773 | 2.90351937610582 | 0.321851453175457 | FASTK//MCCC2//CRTAP//CKAP4//EOGT//IGFBP3//DNAJB9//WNT3//WNT7A//KDELC1//CALU//OGN//PRELP//SDC1//HS3ST1//SERPINI1//ARMC8//IMPDH1//MAPK1//PSMA2//KCMF1//PSMD12//C6ORF120//NSG1//NEU1//SERPINB13//CTSF//EDN1//CMC4//TIMM9//NLN//THOP1//IGF1//NHLRC2//VEGFB//GRSF1//ZBTB18//JUN//DNAJB6//SGK2//MBNL2//STX6//CEBPB//SIVA1//TACC2//CELF1//PTGES3//ZMYND11//ARPP19//TCERG1//COPS8//RPP14//STRAP//EGLN3//KLF12//MBD6//PPARGC1B//VPS37A//CRKL//PHF13//PDIK1L//PPP4R2//RNF38//SH3D19//CREBRF//ESCO2//GADD45A//DCP2//POLR3H//DNMT3B//E2F5//S1PR1//ZNF367//ARID2//ELAVL2//CC2D1B//ELK3//EP300//ESR1//ETS1//ETV5//ATF5//DZIP1//BAHD1//ELL2//KDM2A//PDZRN3//PHF8//FLII//SIN3B//FMR1//SIRT1//ZFPM2//SLC44A1//MORC3//SMUG1//KPNA6//NR5A2//NAALADL2//GABRA5//ANAPC15//AGO1//HBP1//GJA1//RNF11//FOXP1//ZBTB11//GLI3//ATAD2//TBK1//GTF2A1//GTF2E1//GTF2H1//NRBP1//NRBF2//NRG1//HIC1//HIVEP2//FOXA1//HOXA3//HOXD1//HSBP1//IRF1//IRF2//AR//ISL1//JARID2//MBNL1//MDM4//MEF2A//NR3C2//MYB//MYOG//PPP1R12A//NEUROD1//NFIA//NFE2//NKTR//NPAS2//NPAT//NR4A2//OGG1//DCAF8//PAX5//PCBP2//ASCC1//CUTC//KLF3//CTDSPL2//ARID4B//RAB8B//BRWD1//POU4F1//PPARG//BNC2//RPP25//UBE2W//FBXW7//PPP3R1//DOCK10//PRKAA1//INTS8//PRKACB//TRERF1//BDP1//MAPK6//NXF2//SMURF1//PTEN//PTHLH//USP28//ZBTB4//MIER1//RRAGD//RB1//RBBP8//CCND1//RNF2//RORA//ATXN1//ATXN7//PRDM16//SRSF2//SRSF6//ZMAT3//CPEB1//NABP1//SKP1//BMPR2//SMARCA2//SMARCD2//SOX4//SUV39H1//ZEB1//TFAP2A//TFE3//TGIF1//TIA1//NXF2B//UBE2I//UBE2V1//WT1//LDLRAD4//ZNF131//ZNF217//SLBP//BAG6//CENPO//SAP30L//TBL1XR1//E2F8//SNIP1//ADAM12//FOSL1//ANP32A//SNX27//HIST2H2BE//ARID5B//MEX3B//PCGF5//LCOR//DCTN5//TEAD2//CDC14A//TP63//RTCA//RUNX3//CBFB//SOCS1//SKAP2//CCND2//BTAF1//CCNT2//NMI//ACBD5//WDR20//RPS6KA5//FAM114A1//CLOCK//RASSF2//GIT2//AKAP11//DDX6//TMEM217//WDFY3//FLNA//MDFIC//YPEL2//ASNA1//MYCN//NOVA1//CMPK1//PIK3CB//SLC14A1//ZNF655//FXR1//DDX3Y//GPRC5A//MOB1B//TNPO2//CFL2//CLIC4//PURA//H2AFZ//SYCE1//APPL1//SS18//AFF1//FAM118B//MED19//CDK19//MED9//MED22//HIPK3//GSC//FLI1//CNOT7//BHLHE40//PATL1//KLHL20//FAM76B//DENND1B//PPP1R16B//MEOX2//SLC2A4RG//CCNL1//RBM25//RAF1//SGK1//S100PBP//CBLL1//DAZAP2//EPC2//JAZF1//SESN1//NFIB//NFIC//FOXJ2//PAK6// |
| GO:0005794 | Golgi apparatus | Cellular component | 100 | 1501 | 929 | 18698 | 1.34090728176193 | 0.00148311143150953 | 0.0235924577716053 | 2.82882621763538 | 0.107642626480086 | GOLGA6A//YIPF6//CNIH1//STX6//RTN3//SEC23A//FUT9//SERINC3//CLCN5//VTI1A//B3GALNT2//RAB12//HS3ST5//FUT5//GJA1//ATP2C1//ARFIP1//RAB30//ST8SIA5//TRIM23//RND3//GCNT4//NDFIP2//ZDHHC7//SYBU//SLC30A6//PMEPA1//ACBD3//APOO//RIC3//GNPTG//CHST1//ST3GAL5//AP1S2//MCFD2//DSEL//MMGT1//HS3ST3A1//SULF1//OGN//PRELP//SDC1//WNT3//WNT7A//HS3ST1//RAB21//KLHL20//SNAP25//C16ORF70//TJAP1//AFTPH//TEX261//STEAP2//IER3IP1//FUT2//NSG1//CABP7//KIF13A//CALN1//VAMP3//TRAPPC8//KIF1C//ADCY3//TMED1//STMN2//SPPL3//CPD//UBXN2B//ESCO2//ESR1//RAB3GAP1//RAB18//CAMSAP2//MMD//GABARAPL1//ZDHHC22//GLT6D1//KCNJ2//KDR//FIBIN//LDLR//LRP6//WDR44//MAPK1//PTHLH//RAF1//SYT1//TFAP2A//YES1//CSDE1//CALU//ZDHHC18//KBTBD8//TP63//SGCE//CCDC126//FAM114A1//EI24//RASSF2//SNX17// |
| GO:0043197 | dendritic spine | Cellular component | 17 | 153 | 929 | 18698 | 2.23633536658294 | 0.00159100249172565 | 0.0248485661889515 | 2.79832914018883 | 0.0182992465016146 | FMR1//CNN3//LPAR1//ARC//ZMYND8//GPM6A//GRM3//KCNA4//KCNJ2//DOCK10//PRKAR2B//PSD//PTEN//ABHD17C//STRN//FXR1//CD3E// |
| GO:0044440 | endosomal part | Cellular component | 40 | 496 | 929 | 18698 | 1.62314663703601 | 0.0018292003397745 | 0.0280586266404696 | 2.73773872662102 | 0.0430570505920344 | CLCN5//CLCN6//VPS37A//ZNRF2//APPL1//STEAP2//TBK1//RHOB//ARHGAP1//LDLR//IRAK4//WDR44//PMEPA1//KIF13A//SNX2//VPS4B//SNX17//SNX5//RRAGD//SLC9A6//EPS15//RAB21//NSG1//SNX19//LRP6//LDLRAD4//PLEKHF2//SNX27//MMGT1//VTI1A//MMD//CHMP3//SLC26A7//RAB12//RAB8B//RAP2C//ABHD17C//VAMP3//PSD//NDFIP2// |
| GO:0097060 | synaptic membrane | Cellular component | 28 | 317 | 929 | 18698 | 1.77778079614796 | 0.00238097405800969 | 0.0358816967689531 | 2.62324533644082 | 0.0301399354144241 | RIMS4//ZNRF2//FMR1//NPTN//GRIK2//GRM3//SNAP25//SYT1//FOSL1//GPHN//CHRM2//CHRNB4//CPEB3//ARC//GABRA5//NSG1//GLRB//KCNB1//SEMA4C//PTEN//ABHD17C//CPEB1//STRN//GLRA3//DLG5//HOMER1//GSG1L//CPD// |
| GO:0032991 | protein-containing complex | Cellular component | 290 | 5071 | 929 | 18698 | 1.15102254127026 | 0.00248654648393954 | 0.0368266108569666 | 2.60440341757805 | 0.31216361679225 | GTF2H1//DYNC1LI2//FBXO28//PPP1R12A//CLIP1//TTK//RASSF2//PALLD//WDR1//C1QTNF2//C1QTNF6//KPNA6//AGFG1//PURA//GSC//E2F5//EP300//ETS1//ATF5//FOXF1//FOXF2//JUN//MEF2A//MYOG//NHLH2//NPAS2//NR4A2//ASCC1//PKNOX1//POU3F2//TRERF1//RB1//ZEB1//E2F8//TEAD2//TP63//CLOCK//PSMD12//PSMA2//TNNI1//RANBP9//MID1//LRP8//PRKACB//PRKAR2B//ELL2//AFF1//ADCY3//COPS8//TMOD1//VBP1//DYNLL2//PPARGC1B//MED19//CDK19//GLI3//MED9//MED22//JAZF1//MIER1//RBBP8//CCND1//PRDM16//TBL1XR1//TOB1//PATL1//CPEB3//CNOT6L//CNOT7//CNOT6//NUP54//SEPT9//PAN3//RNF2//SKP1//PCGF5//FLNA//RICTOR//SESN1//PRR5L//IQGAP2//CD2AP//INTS8//RAP1A//STRAP//FMR1//NFE2//ST18//TSC1//BBS9//ARMC8//TMEM17//NXF2//ATXN1//NXF2B//CR2//KDR//LDLR//LRP6//MET//NR3C2//ROR1//PEX5L//TGFBR2//NABP1//CSDE1//SNX27//CNTFR//DZIP1//CALM2//RRAGD//UBE2I//WNT3//CFDP1//DCTN6//CENPO//DCTN5//SOX4//KIF1C//KIF13A//CCNT2//SNAP25//SIRT1//JARID2//H2AFZ//HIST2H2BE//ESR1//GTF2E1//VPS37A//CHMP3//IGF1//IGFBP3//PLXNA4//BAG6//SGCE//ITGB8//PYURF//PIGP//PIGA//AP1S2//PIK3CA//PIK3CB//MEP1A//SNX5//SNX2//STX6//VTI1A//VAMP1//SYT1//VAMP3//INSIG2//CNEP1R1//EMC7//MMGT1//EVC//CNGA3//MCCC2//TIMM9//TRAPPC8//CCT6A//SIN3B//ZNF217//SAP30L//RPP25//SERP1//NCK1//RBM25//SRSF2//PTGES3//AGO1//FXR1//GRSF1//SLBP//CPEB2//CPEB1//APPL1//MZT1//NPNT//NR5A2//NEUROD1//PPARG//RORA//RBM41//AFTPH//EPS15//NRBF2//BAHD1//SUV39H1//SEC23A//RPP14//RRP15//SPTSSB//SPTSSA//DDX6//DCP2//PNRC1//TNRC6B//YTHDF2//MEX3B//MBNL1//TIA1//TDRD5//CKAP4//SOCS1//DDX3Y//CHRNB4//CD3E//CD8A//ACVR1//GABRA5//BDP1//GTF2A1//CEBPB//COX8C//ARL6IP1//GNAQ//SCN1B//SLC25A6//GJC1//GJA1//KCNH4//KCNA4//KCNB1//KCNE1//KCNJ2//KCNJ3//KCNK2//KCNIP4//KCNAB2//CDH20//GLRB//GLRA3//IMMP2L//APOO//DCUN1D4//FBXO8//RNF11//ANKIB1//UBE2V1//CBLL1//WDFY3//F3//CLIC4//ANO1//IER5//PPP2CA//PPP2CB//PPP2R5E//STRN//PPP1R15B//PPP3R1//PPP4R2//SMARCA2//SMARCD2//SS18//GTPBP1//CACUL1//CUL5//FBXW7//ANAPC15//SPOPL//FBXO44//ZER1//KLHL3//KLHL20//KLHL42//KBTBD8//DCAF8//EPS8//GRIK2//POLR3H//SESTD1//CCNL1//CCND2//CCNY//PRKAA1//EPC2//CRTAP//RAB3GAP1//SLC41A1//TES//ID2//OTX2//USP28//GIGYF1//MTMR9//PPM1F//CD69//RAPGEF2//DAZAP2// |
| GO:0030054 | cell junction | Cellular component | 84 | 1247 | 929 | 18698 | 1.35578952456833 | 0.00261009725535284 | 0.0380012464804761 | 2.58334331002718 | 0.0904198062432723 | FLNA//CD2AP//CLIC4//NCK1//RAP1B//CD3E//RAPGEF2//WDR1//CNN3//CDH20//TMOD3//BMPR2//ZYX//DLG5//GJC1//DBN1//GJA1//FRMD6//RAP2C//DLL1//CCND1//STRN//TJAP1//KCNJ2//SCN1B//EFNB2//RAB21//PALLD//FLII//TES//IRF2//RHOB//RND3//LASP1//MARCKS//MSN//PPP1R12A//PCBP2//ENAH//MAPK1//TNS1//YES1//YWHAG//PPFIA1//LMLN//GIT2//GPHN//CHRM2//CHRNB4//LYPD6//RIMS4//GSG1L//SYT6//ESCO2//DCP2//DTNA//EPS8//ZNRF2//CPEB3//PDZRN3//ARC//FMR1//GABRA5//GLRB//GRIK2//KCNB1//KDR//NEU1//NFIA//SEMA4C//PAK6//ABHD17C//RAP1A//CPEB1//ITSN1//SNAP25//VAMP1//SYT1//GLRA3//KCNAB2//BSN//VAMP3//PLAA//HOMER1// |
| GO:0000159 | protein phosphatase type 2A complex | Cellular component | 5 | 21 | 929 | 18698 | 4.79214721410631 | 0.00313085644061031 | 0.0448234280414043 | 2.50433684566323 | 0.00538213132400431 | IER5//PPP2CA//PPP2CB//PPP2R5E//STRN// |
| GO:0012505 | endomembrane system | Cellular component | 255 | 4421 | 929 | 18698 | 1.16091148299205 | 0.00324589079351831 | 0.0457085277316759 | 2.48866609589926 | 0.27448869752422 | WDFY3//SIRT1//CNEP1R1//ANXA7//LBR//NUP54//CLIP1//BNIP2//UBE2I//STMN2//SLC26A7//CLCN5//CRHR1//CRKL//VTI1A//DGKH//S1PR1//LPAR1//RAB12//RAB21//ARC//STEAP2//NSG1//SNX5//KDR//RAB8B//ARRDC3//SNX2//SNX27//TJAP1//VPS4B//SNX17//RTN3//CDS1//CRTAP//KIF1C//CKAP4//TMED1//UBXN2B//B3GALNT2//TMEM64//SMIM14//TAPT1//SULF1//GABARAPL1//LCLAT1//SERP1//ZDHHC22//YIPF6//FIBIN//SHISA2//LRP6//FAM19A1//DNAJB9//MGST1//NCK1//IER3IP1//PLP2//UGT2B28//NDFIP2//ZDHHC7//PHTF2//STIM2//RDH14//ELOVL5//RTN1//MSMO1//HSPA13//TMBIM6//PTP4A1//PLEKHF2//ELOVL7//KCNIP4//ANP32A//CALU//CAST//ZDHHC18//PPP1R15B//EI24//STX6//FUT9//ADCY3//SPPL3//CPD//ESCO2//ESR1//RAB3GAP1//RAB18//CAMSAP2//MMD//FUT2//FUT5//GJA1//ATP2C1//KLHL20//GOLGA6A//GLT6D1//KCNJ2//LDLR//WDR44//AFTPH//SLC30A6//MAPK1//PTHLH//RAF1//ACBD3//SYT1//TFAP2A//YES1//CSDE1//KBTBD8//GNPTG//TP63//AP1S2//SGCE//CCDC126//FAM114A1//MMGT1//RASSF2//EDN1//VAMP3//KLK15//EPS15//LRP12//ITSN1//C8ORF44-SGK3//ADRB1//FKBP15//SGK3//APPL1//RNF11//RHOB//RAP1A//TRAK2//PTP4A2//SLC9A6//SLC31A2//DYNC1LI2//CHMP3//RAPGEF2//DENND6A//TBC1D12//TNF//ARHGAP1//RASD1//NTF3//SNAP25//IGF1//KPNA6//AGFG1//PRICKLE2//EDNRB//TMEM201//PHF8//LEMD3//GNAQ//TNPO2//C2ORF42//DCTN5//CCND2//SNX19//PMEPA1//LDLRAD4//VPS37A//IQGAP2//RAB9B//C5AR1//IMPDH1//SERPINI1//PSMA2//PSMD12//SYT6//SEMA4C//VAMP1//C16ORF70//NHLRC2//VEGFB//NAV3//GJC1//PYURF//LPCAT3//CNIH1//SEC23A//RDH10//SPTSSB//SPTSSA//ACSL1//ACSL4//REEP3//SLC35D1//ARL6IP1//KCNK2//NR3C2//ASNA1//INSIG2//PIGP//JKAMP//PIGA//SEC61A2//SGK1//UGT2B7//APOO//RIC3//ALG2//MCFD2//DHRS3//CHRNB4//KCNAB2//ARMC8//NEU1//BRI3//RAP1B//C6ORF120//RAP2C//ABHD17C//SIRPA//KCMF1//CLCN6//ZNRF2//TBK1//IRAK4//KIF13A//RRAGD//PSD//EOGT//IGFBP3//WNT3//WNT7A//KDELC1//TEX261//EMC7//SERINC3//HS3ST5//ARFIP1//RAB30//ST8SIA5//TRIM23//RND3//GCNT4//SYBU//CHST1//ST3GAL5//DSEL//HS3ST3A1//OGN//PRELP//SDC1//HS3ST1//CABP7//CALN1//TRAPPC8//RHEBL1//RNF167//NRBP1//RAB28// |
| GO:0098590 | plasma membrane region | Cellular component | 73 | 1069 | 929 | 18698 | 1.37443623558933 | 0.00350739408686258 | 0.0485943793647574 | 2.45501543453642 | 0.0785791173304629 | EPS15//MET//BMPR2//SLC23A2//SLC26A7//SLC41A1//KCNJ10//LDLR//MSN//SLC14A1//C5AR1//CNTFR//GJA1//DLL1//KCNE1//KCNK2//SLC26A4//ANO1//PRKAA1//PTEN//SLC10A2//FZD3//FZD6//VAMP3//RAPGEF2//RAB21//RHOB//PSD//CD8A//CPD//RIMS4//EPS8//ARHGEF4//C2CD5//TMEM17//EVC//BBS9//SPRED1//LRP6//MAPK1//TGFBR2//LRP8//ZNRF2//FMR1//NPTN//GRIK2//GRM3//SNAP25//SYT1//FOSL1//GPHN//CHRM2//CHRNB4//CPEB3//ARC//GABRA5//NSG1//GLRB//KCNB1//SEMA4C//ABHD17C//CPEB1//STRN//GLRA3//DLG5//HOMER1//NRG1//ROBO2//GABARAPL1//SGCE//CNGA3//DHRS3//GSG1L// |
| GO:0044451 | nucleoplasm part | Cellular component | 74 | 1113 | 929 | 18698 | 1.3381845050712 | 0.00628422873631558 | 0.0856849600713505 | 2.20174801568939 | 0.0796555435952637 | SIN3B//TRERF1//ZNF217//SAP30L//TBL1XR1//EP300//POLR3H//GTF2A1//GTF2E1//GTF2H1//ELL2//AFF1//PPARGC1B//MED19//CDK19//GLI3//MED9//MED22//HIPK3//GSC//CREBRF//FLI1//CNOT7//RAB8B//USP28//ZBTB4//RNF2//BHLHE40//CDC14A//INTS8//JARID2//NXF2//ATXN1//NXF2B//APPL1//RB1//CCNT2//FMR1//NPAT//FAM118B//PATL1//WDFY3//SIRT1//MORC3//KLHL20//NFE2//PTEN//UBE2I//CKAP4//FAM76B//DENND1B//GADD45A//PPP1R16B//HBP1//AR//MEOX2//NR4A2//OGG1//SERPINB13//PRKAA1//SLC2A4RG//CCNL1//RBM25//RAF1//SRSF2//SRSF6//SGK1//S100PBP//WT1//CBLL1//DAZAP2//BAHD1//SUV39H1//EPC2// |
| GO:0031984 | organelle subcompartment | Cellular component | 104 | 1655 | 929 | 18698 | 1.26477939765658 | 0.00713038646450229 | 0.0957031558282417 | 2.14688693089816 | 0.11194833153929 | SULF1//RAB30//STX6//RAB12//RAB21//ATP2C1//KLHL20//YIPF6//SNAP25//C16ORF70//TJAP1//RAB3GAP1//RAB18//GOLGA6A//VTI1A//CABP7//NSG1//ARFIP1//KIF13A//CALN1//AP1S2//VAMP3//FUT9//FUT2//FUT5//CNIH1//RTN3//SEC23A//SERINC3//CLCN5//B3GALNT2//HS3ST5//GJA1//ST8SIA5//TRIM23//RND3//GCNT4//NDFIP2//ZDHHC7//SYBU//SLC30A6//PMEPA1//ACBD3//APOO//RIC3//GNPTG//CHST1//ST3GAL5//MCFD2//DSEL//MMGT1//HS3ST3A1//GJC1//PYURF//LPCAT3//CDS1//SLC9A6//CKAP4//TMED1//RDH10//SPTSSB//SPTSSA//SMIM14//ACSL1//ACSL4//REEP3//SLC35D1//ARL6IP1//LCLAT1//SERP1//ANXA7//KCNK2//SHISA2//DNAJB9//MGST1//NR3C2//ASNA1//IER3IP1//INSIG2//PIGP//JKAMP//PIGA//PLP2//UGT2B28//SEC61A2//STIM2//RDH14//ELOVL5//MSMO1//SGK1//TMBIM6//UGT2B7//ELOVL7//CALU//ALG2//DHRS3//EI24//TEX261//STEAP2//TAPT1//LBR//RTN1//SPPL3//EMC7// |
| GO:0031410 | cytoplasmic vesicle | Cellular component | 138 | 2279 | 929 | 18698 | 1.21874880443002 | 0.00731869394174195 | 0.0967193553224052 | 2.13556641409224 | 0.148546824542519 | STMN2//SLC26A7//CLCN5//CRHR1//CRKL//VTI1A//DGKH//S1PR1//LPAR1//RAB12//RAB21//ARC//STEAP2//NSG1//SNX5//KDR//RAB8B//ARRDC3//CLIP1//SNX2//SNX27//TJAP1//VPS4B//SNX17//EDN1//PLEKHF2//VAMP3//CD2AP//RAB9B//ITSN1//RAPGEF2//C8ORF44-SGK3//STX6//ADRB1//FKBP15//SGK3//APPL1//GJA1//RNF11//RHOB//LDLR//LRP6//MAPK1//RAP1A//TRAK2//PTP4A1//PTP4A2//MMGT1//SLC9A6//SLC31A2//DYNC1LI2//CHMP3//DENND6A//TBC1D12//TNF//ARHGAP1//TMED1//TEX261//YIPF6//IER3IP1//IGF1//ZYX//DENND1B//CALU//CPD//KLK15//NTF3//SNAP25//SYT1//CKAP4//EPS15//SNX19//PMEPA1//LDLRAD4//VPS37A//MMD//NDFIP2//SPPL3//CNIH1//SEC23A//MCFD2//IQGAP2//RAB18//C5AR1//IMPDH1//SERPINI1//PSMA2//PSMD12//WNT3//WNT7A//SYT6//SEMA4C//VAMP1//C16ORF70//CHRM2//NHLRC2//VEGFB//CHRNB4//KCNAB2//ARMC8//NEU1//BRI3//MGST1//RAP1B//C6ORF120//ANXA7//RAP2C//ABHD17C//SIRPA//KCMF1//AFTPH//CLCN6//ZNRF2//TBK1//IRAK4//WDR44//KIF13A//ZPLD1//GABARAPL1//CLIC4//YWHAG//FZD6//AP1S2//GPRC5A//ULK2//C2CD5//RRAGD//PSD//SPRED1//TRAPPC8//DLL1//NRBF2//AGFG1//RND3//CHIC1//SYBU//CXXC4//SOCS1// |
| GO:0097708 | intracellular vesicle | Cellular component | 138 | 2282 | 929 | 18698 | 1.21714659303068 | 0.00764582885009295 | 0.0995116209428764 | 2.11657542777816 | 0.148546824542519 | C8ORF44-SGK3//SPRED1//TRAPPC8//SGK3//DLL1//NRBF2//AGFG1//RHOB//RND3//LRP6//NTF3//CHMP3//CHIC1//SYBU//TRAK2//CXXC4//SOCS1//SNX17//STMN2//SLC26A7//CLCN5//CRHR1//CRKL//VTI1A//DGKH//S1PR1//LPAR1//RAB12//RAB21//ARC//STEAP2//NSG1//SNX5//KDR//RAB8B//ARRDC3//CLIP1//SNX2//SNX27//TJAP1//VPS4B//EDN1//PLEKHF2//VAMP3//CD2AP//RAB9B//ITSN1//RAPGEF2//STX6//ADRB1//FKBP15//APPL1//GJA1//RNF11//LDLR//MAPK1//RAP1A//PTP4A1//PTP4A2//MMGT1//SLC9A6//SLC31A2//DYNC1LI2//DENND6A//TBC1D12//TNF//ARHGAP1//TMED1//TEX261//YIPF6//IER3IP1//SNAP25//SYT1//IGF1//ZYX//DENND1B//CPD//KLK15//CALU//CKAP4//EPS15//SNX19//PMEPA1//LDLRAD4//VPS37A//MMD//NDFIP2//SPPL3//CNIH1//SEC23A//MCFD2//IQGAP2//RAB18//C5AR1//IMPDH1//SERPINI1//PSMA2//PSMD12//WNT3//WNT7A//SYT6//SEMA4C//VAMP1//C16ORF70//CHRM2//NHLRC2//VEGFB//CHRNB4//KCNAB2//ARMC8//NEU1//BRI3//MGST1//RAP1B//C6ORF120//ANXA7//RAP2C//ABHD17C//SIRPA//KCMF1//CLCN6//ZNRF2//TBK1//IRAK4//WDR44//KIF13A//RRAGD//PSD//AFTPH//ZPLD1//GABARAPL1//CLIC4//YWHAG//FZD6//AP1S2//GPRC5A//ULK2//C2CD5// |
| GO:0016592 | mediator complex | Cellular component | 6 | 36 | 929 | 18698 | 3.35450304987442 | 0.00802893620273822 | 0.102938152211226 | 2.09534199293873 | 0.00645855758880517 | PPARGC1B//MED19//CDK19//GLI3//MED9//MED22// |
| GO:0031901 | early endosome membrane | Cellular component | 14 | 138 | 929 | 18698 | 2.04187142166269 | 0.00879187307793782 | 0.111062043734538 | 2.05591859014202 | 0.0150699677072121 | RAB21//SLC9A6//EPS15//APPL1//NSG1//SNX5//SNX19//LRP6//PMEPA1//SNX2//LDLRAD4//PLEKHF2//SNX27//MMGT1// |
| GO:0044306 | neuron projection terminus | Cellular component | 14 | 139 | 929 | 18698 | 2.02718169920468 | 0.00934418760053395 | 0.116328364476212 | 2.02945845094028 | 0.0150699677072121 | SLC9A6//CHRM2//FMR1//ROR1//KCNAB2//KCNK2//STX6//ADCYAP1//GRIK2//PFN2//VAMP1//SYT1//VTI1A//BSN// |
| GO:0034705 | potassium channel complex | Cellular component | 11 | 99 | 929 | 18698 | 2.23633536658294 | 0.0100241525121857 | 0.123010671542393 | 1.99895233440249 | 0.0118406889128095 | KCNH4//KCNA4//KCNB1//KCNE1//KCNJ2//KCNJ3//KCNK2//SNAP25//KCNIP4//KCNAB2//GRIK2// |
| GO:0033267 | axon part | Cellular component | 28 | 353 | 929 | 18698 | 1.59647737217819 | 0.0101875850725272 | 0.123255430666209 | 1.99192875161394 | 0.0301399354144241 | STMN2//DBN1//EPS8//PALLD//FKBP15//FMR1//OTX2//SNAP25//TSC1//NRG1//ROBO2//SCN1B//SPOCK1//STX6//ADCYAP1//GRIK2//PFN2//VAMP1//SYT1//SLC9A6//CHRM2//ROR1//KCNAB2//RAB21//SYBU//GPM6A//TRAK2//KCNK2// |
| GO:0045211 | postsynaptic membrane | Cellular component | 21 | 244 | 929 | 18698 | 1.73224337821384 | 0.0103574547274654 | 0.123570189040177 | 1.98474695633089 | 0.0226049515608181 | GSG1L//GRIK2//GPHN//CHRM2//CHRNB4//CPEB3//ARC//FMR1//GABRA5//NSG1//GLRB//GRM3//KCNB1//SEMA4C//PTEN//ABHD17C//CPEB1//STRN//GLRA3//DLG5//HOMER1// |
| GO:0005802 | trans-Golgi network | Cellular component | 18 | 202 | 929 | 18698 | 1.79349668013088 | 0.0120883884623042 | 0.142245557385196 | 1.91763159232188 | 0.0193756727664155 | STX6//VTI1A//CABP7//NSG1//ARFIP1//KIF13A//CALN1//AP1S2//VAMP3//RAB12//RAB21//ATP2C1//KLHL20//RAB30//YIPF6//SNAP25//C16ORF70//TJAP1// |
| GO:0005811 | lipid droplet | Cellular component | 9 | 77 | 929 | 18698 | 2.35250863237946 | 0.0138818745503382 | 0.16114230052352 | 1.85755188460893 | 0.00968783638320775 | CKAP4//RDH10//ACSL4//RAB3GAP1//ABHD5//RAP1B//TSC1//LMLN//DHRS3// |
| GO:0098793 | presynapse | Cellular component | 29 | 379 | 929 | 18698 | 1.54006208622203 | 0.0142991816698978 | 0.16349884087907 | 1.84468881613054 | 0.031216361679225 | VTI1A//NTF3//RAB8B//SNAP25//SYT1//RIMS4//ZNRF2//FMR1//NPTN//GRIK2//GRM3//FOSL1//STX6//ADCYAP1//PFN2//VAMP1//SLC9A6//CHRM2//ROR1//KCNAB2//FZD3//PPFIA1//BSN//KCNK2//SYT6//SEMA4C//C16ORF70//KCNJ10//WNT7A// |
| GO:0150034 | distal axon | Cellular component | 22 | 268 | 929 | 18698 | 1.6522179200874 | 0.0147446638714599 | 0.16349884087907 | 1.83136512344681 | 0.0236813778256189 | STMN2//DBN1//EPS8//PALLD//FKBP15//FMR1//OTX2//SNAP25//TSC1//SLC9A6//CHRM2//ROR1//KCNAB2//GPM6A//TRAK2//KCNK2//STX6//ADCYAP1//GRIK2//PFN2//VAMP1//SYT1// |
| GO:0016442 | RISC complex | Cellular component | 3 | 11 | 929 | 18698 | 5.48918680888541 | 0.014944857413 | 0.16349884087907 | 1.82550822415382 | 0.00322927879440258 | DDX6//DCP2//AGO1// |
| GO:0031332 | RNAi effector complex | Cellular component | 3 | 11 | 929 | 18698 | 5.48918680888541 | 0.014944857413 | 0.16349884087907 | 1.82550822415382 | 0.00322927879440258 | DDX6//DCP2//AGO1// |
| GO:0000307 | cyclin-dependent protein kinase holoenzyme complex | Cellular component | 6 | 41 | 929 | 18698 | 2.94541731208485 | 0.015036563945805 | 0.16349884087907 | 1.82285139445342 | 0.00645855758880517 | CCNY//CCND2//RB1//CCNT2//CCNL1//CCND1// |
| GO:0008076 | voltage-gated potassium channel complex | Cellular component | 10 | 92 | 929 | 18698 | 2.18771938035288 | 0.0158459706521056 | 0.170146109876984 | 1.80008115275377 | 0.0107642626480086 | KCNH4//KCNA4//KCNB1//KCNE1//KCNJ2//KCNJ3//KCNK2//SNAP25//KCNIP4//KCNAB2// |
| GO:0042734 | presynaptic membrane | Cellular component | 9 | 79 | 929 | 18698 | 2.29295145181289 | 0.0162326523441391 | 0.172146276094018 | 1.78961051256372 | 0.00968783638320775 | RIMS4//ZNRF2//FMR1//NPTN//GRIK2//GRM3//SNAP25//SYT1//FOSL1// |
| GO:0044433 | cytoplasmic vesicle part | Cellular component | 91 | 1472 | 929 | 18698 | 1.2442653975757 | 0.0171094922115193 | 0.179232363532867 | 1.76676287958851 | 0.0979547900968784 | AFTPH//EPS15//ZPLD1//ZNRF2//RAB21//GABARAPL1//CLIC4//NSG1//SNX5//SNX19//CLIP1//YWHAG//FZD6//AP1S2//GPRC5A//ULK2//SNX17//C2CD5//SPPL3//GJA1//SERPINI1//ARMC8//IMPDH1//MAPK1//PSMA2//KCMF1//PSMD12//SEC23A//IQGAP2//RAB18//RAB9B//RAB8B//C5AR1//WNT3//WNT7A//CNIH1//VTI1A//MCFD2//CLCN5//CLCN6//VPS37A//APPL1//STEAP2//TBK1//RHOB//ARHGAP1//LDLR//IRAK4//WDR44//PMEPA1//KIF13A//SNX2//VPS4B//RRAGD//STX6//SYT1//SYT6//SEMA4C//VAMP1//C16ORF70//CKAP4//BRI3//MGST1//RAP1B//CHRNB4//RAP1A//SNAP25//KCNAB2//ANXA7//SIRPA//RAP2C//SLC9A6//LRP6//LDLRAD4//PLEKHF2//SNX27//MMGT1//MMD//CHMP3//NDFIP2//SLC26A7//RAB12//ABHD17C//VAMP3//CHRM2//PSD//IGF1//NHLRC2//VEGFB//C6ORF120//NEU1// |
| GO:0055037 | recycling endosome | Cellular component | 15 | 165 | 929 | 18698 | 1.82972893629514 | 0.0176178061545835 | 0.182095712200907 | 1.75404817278424 | 0.0161463939720129 | SLC9A6//SLC26A7//RAB12//NSG1//RAB8B//RAP2C//ABHD17C//VAMP3//C8ORF44-SGK3//SLC31A2//DENND6A//TBC1D12//SGK3//RNF11//TNF// |
| GO:0044431 | Golgi apparatus part | Cellular component | 61 | 935 | 929 | 18698 | 1.31309958957651 | 0.0178067983991574 | 0.182095712200907 | 1.7494141581656 | 0.0656620021528525 | CNIH1//STX6//RTN3//SEC23A//FUT9//SERINC3//CLCN5//VTI1A//B3GALNT2//RAB12//HS3ST5//FUT5//GJA1//ATP2C1//ARFIP1//RAB30//YIPF6//ST8SIA5//TRIM23//RND3//GCNT4//NDFIP2//ZDHHC7//SYBU//SLC30A6//PMEPA1//ACBD3//APOO//RIC3//GNPTG//CHST1//ST3GAL5//AP1S2//MCFD2//DSEL//MMGT1//HS3ST3A1//OGN//PRELP//SDC1//WNT3//WNT7A//HS3ST1//GOLGA6A//AFTPH//TRAPPC8//RAB21//FUT2//NSG1//CABP7//KIF13A//CALN1//VAMP3//TEX261//STEAP2//IER3IP1//SULF1//KLHL20//SNAP25//C16ORF70//TJAP1// |
| GO:0031090 | organelle membrane | Cellular component | 170 | 2950 | 929 | 18698 | 1.159862071482 | 0.0184640856359931 | 0.186595877191977 | 1.73367219427214 | 0.182992465016146 | RAB21//APPL1//NCK1//PRICKLE2//EDNRB//TMEM201//WDFY3//PHF8//LEMD3//CNEP1R1//GNAQ//TNPO2//LBR//NUP54//C2ORF42//DCTN5//CCND2//EI24//MAVS//TMBIM6//ZPLD1//ZNRF2//GABARAPL1//CLIC4//NSG1//SNX5//SNX19//CLIP1//YWHAG//FZD6//AP1S2//GPRC5A//ULK2//SNX17//C2CD5//SIRT1//ABCB7//TIMM9//SLC25A34//MICU3//SLC25A6//PRKAR2B//CSDE1//SLC25A28//UGT2B28//NAV3//ACSL1//ACSL4//SLC44A1//GJA1//MTX3//MGST1//PMAIP1//ARMCX6//MIEF1//RAF1//BCL2A1//VAMP1//CNIH1//STX6//RTN3//SEC23A//FUT9//SERINC3//CLCN5//VTI1A//B3GALNT2//RAB12//HS3ST5//FUT5//ATP2C1//ARFIP1//RAB30//YIPF6//ST8SIA5//TRIM23//RND3//GCNT4//NDFIP2//ZDHHC7//SYBU//SLC30A6//PMEPA1//ACBD3//APOO//RIC3//GNPTG//CHST1//ST3GAL5//MCFD2//DSEL//MMGT1//HS3ST3A1//PLEKHF2//SBF2//IQGAP2//RAB18//RAB9B//RAB8B//C5AR1//CLCN6//VPS37A//STEAP2//TBK1//RHOB//ARHGAP1//LDLR//IRAK4//WDR44//KIF13A//SNX2//VPS4B//SPPL3//WNT3//WNT7A//TMED1//TMEM17//EPS15//EVC//BBS9//FUT2//GOLGA6A//CABP7//CALN1//VAMP3//ATG16L1//MMD//OSTM1//NEU1//DRAM1//SPHK2//RDH14//SYT6//SEMA4C//SYT1//C16ORF70//CKAP4//BRI3//RAP1B//CHRNB4//RAP1A//SNAP25//KCNAB2//ANXA7//SIRPA//RAP2C//SLC9A6//LRP6//LDLRAD4//SNX27//CHMP3//SLC26A7//ABHD17C//CHRM2//PEX5L//ACBD5//CNGA3//DHRS3//COX7A2//RRAGD//PSD//TEX261//IER3IP1//ARMCX3//COX8C//IMMP2L//ARL6IP1//AFTPH//RDH10//UGT2B7// |
| GO:0015630 | microtubule cytoskeleton | Cellular component | 73 | 1152 | 929 | 18698 | 1.27541001375434 | 0.0188403239822747 | 0.188184166288069 | 1.72491163325735 | 0.0785791173304629 | TACC2//AKAP11//LCA5//DZIP1//FLII//YTHDF2//PXK//RPP25//MAPK1//YES1//C2CD5//MID1//MZT1//KLHL42//RB1//TTK//PTP4A1//SHCBP1//KATNAL1//KBTBD8//KIF1C//SEPT9//DYNLL2//CAMSAP1//DYNC1LI2//DPYSL2//REEP3//WDR47//GABARAPL1//SYBU//MID1IP1//CLIP1//KIF13A//KCNAB2//CCT6A//RANBP9//LRP8//DCTN6//NEDD1//VPS37A//PPP4R2//POLR3H//TAPT1//ATF5//CAMSAP2//CLIC4//MARCKS//PPP1R12A//PRKACB//PRKAR2B//RRAGD//TFAP2A//CALM2//PCGF5//DCTN5//CDC14A//VPS4B//CEP41//EVC//DLG5//RAB28//HECW2//TBL1XR1//SERP1//SS18//BBS9//ZMYND10//TMEM201//PPP2CA//PPP2CB//NAV3//FAM83D//ATXN7// |
| GO:0120111 | neuron projection cytoplasm | Cellular component | 8 | 68 | 929 | 18698 | 2.36788450579371 | 0.0190837583027175 | 0.188424694046372 | 1.71933609245166 | 0.00861141011840689 | GABARAPL1//GRIK2//MAPK1//TRAK2//FMR1//FXR1//RAB21//SYBU// |
| GO:0043679 | axon terminus | Cellular component | 12 | 123 | 929 | 18698 | 1.9636115413899 | 0.0193752941315066 | 0.189129291579138 | 1.71275169585542 | 0.0129171151776103 | STX6//ADCYAP1//GRIK2//PFN2//VAMP1//SYT1//KCNK2//SLC9A6//CHRM2//FMR1//ROR1//KCNAB2// |
| GO:0031932 | TORC2 complex | Cellular component | 3 | 13 | 929 | 18698 | 4.64469653059535 | 0.0240567025003711 | 0.225402735983862 | 1.61876390251728 | 0.00322927879440258 | RICTOR//SESN1//PRR5L// |
| GO:0005778 | peroxisomal membrane | Cellular component | 7 | 58 | 929 | 18698 | 2.42912289818492 | 0.0242259464870834 | 0.225402735983862 | 1.61571924643044 | 0.00753498385360603 | ACSL1//ACSL4//MGST1//PEX5L//RAB8B//MAVS//ACBD5// |
| GO:0031903 | microbody membrane | Cellular component | 7 | 58 | 929 | 18698 | 2.42912289818492 | 0.0242259464870834 | 0.225402735983862 | 1.61571924643044 | 0.00753498385360603 | ACSL1//ACSL4//MGST1//PEX5L//RAB8B//MAVS//ACBD5// |
| GO:0032588 | trans-Golgi network membrane | Cellular component | 9 | 85 | 929 | 18698 | 2.13109605521434 | 0.025025698932276 | 0.225402735983862 | 1.60161378445037 | 0.00968783638320775 | STX6//VTI1A//CABP7//NSG1//ARFIP1//KIF13A//CALN1//AP1S2//VAMP3// |
| GO:1902554 | serine/threonine protein kinase complex | Cellular component | 9 | 85 | 929 | 18698 | 2.13109605521434 | 0.025025698932276 | 0.225402735983862 | 1.60161378445037 | 0.00968783638320775 | CCNL1//CCND1//CCND2//CCNT2//ACVR1//TGFBR2//CCNY//GTF2H1//RB1// |
| GO:0000407 | phagophore assembly site | Cellular component | 5 | 34 | 929 | 18698 | 2.95985563224213 | 0.025178435459055 | 0.225402735983862 | 1.59897125963758 | 0.00538213132400431 | ATG16L1//WDR45B//ULK2//TRAPPC8//PLEKHF2// |
| GO:0016605 | PML body | Cellular component | 10 | 99 | 929 | 18698 | 2.03303215143904 | 0.0251905269551231 | 0.225402735983862 | 1.5987627474956 | 0.0107642626480086 | HIPK3//PATL1//WDFY3//SIRT1//MORC3//KLHL20//NFE2//PTEN//RB1//UBE2I// |
| GO:1902911 | protein kinase complex | Cellular component | 10 | 99 | 929 | 18698 | 2.03303215143904 | 0.0251905269551231 | 0.225402735983862 | 1.5987627474956 | 0.0107642626480086 | PRKAA1//CCNL1//CCND1//CCND2//CCNT2//ACVR1//TGFBR2//CCNY//RB1//GTF2H1// |
| GO:0016604 | nuclear body | Cellular component | 49 | 742 | 929 | 18698 | 1.32914271787477 | 0.0259784840823215 | 0.228155207262785 | 1.58538619482923 | 0.0527448869752422 | FMR1//NPAT//FAM118B//HIPK3//PATL1//WDFY3//SIRT1//MORC3//KLHL20//NFE2//PTEN//RB1//UBE2I//CKAP4//FAM76B//DENND1B//GADD45A//PPP1R16B//HBP1//GLI3//CNOT7//AR//MEOX2//NR4A2//OGG1//SERPINB13//PRKAA1//SLC2A4RG//CCNL1//RBM25//RAF1//SRSF2//SRSF6//SGK1//S100PBP//WT1//ZNF217//CBLL1//DAZAP2//GSC//CREBRF//MED19//FLI1//RAB8B//USP28//ZBTB4//RNF2//BHLHE40//CDC14A// |
| GO:0055038 | recycling endosome membrane | Cellular component | 8 | 72 | 929 | 18698 | 2.23633536658294 | 0.0260293484420872 | 0.228155207262785 | 1.58453670286839 | 0.00861141011840689 | SLC9A6//SLC26A7//RAB12//NSG1//RAB8B//RAP2C//ABHD17C//VAMP3// |
| GO:0012506 | vesicle membrane | Cellular component | 51 | 781 | 929 | 18698 | 1.31431233452186 | 0.0280720298528726 | 0.24172908118976 | 1.55172618294701 | 0.0548977395048439 | ZPLD1//ZNRF2//RAB21//GABARAPL1//CLIC4//NSG1//SNX5//SNX19//CLIP1//YWHAG//FZD6//AP1S2//GPRC5A//ULK2//SNX17//C2CD5//IQGAP2//RAB18//RAB9B//RAB8B//C5AR1//WNT3//WNT7A//CNIH1//SEC23A//VTI1A//MCFD2//SYT6//SEMA4C//VAMP1//SYT1//C16ORF70//CKAP4//BRI3//MGST1//RAP1B//CHRNB4//RAP1A//SNAP25//KCNAB2//ANXA7//SIRPA//RAP2C//CHRM2//VAMP3//LDLR//STX6//EPS15//AFTPH//APPL1//NCK1// |
| GO:0005884 | actin filament | Cellular component | 10 | 101 | 929 | 18698 | 1.99277408903431 | 0.0284657456712745 | 0.24172908118976 | 1.54567743519063 | 0.0107642626480086 | IQGAP2//FLNA//CD2AP//WDR1//PALLD//FKBP15//ARHGAP6//TMOD1//TSC1//YES1// |
| GO:0000139 | Golgi membrane | Cellular component | 48 | 729 | 929 | 18698 | 1.32523577278989 | 0.0285838985133849 | 0.24172908118976 | 1.54387853875759 | 0.0516684607104413 | FUT9//RAB21//FUT2//FUT5//NSG1//GOLGA6A//STX6//VTI1A//CABP7//ARFIP1//KIF13A//CALN1//AP1S2//VAMP3//TEX261//STEAP2//IER3IP1//CNIH1//RTN3//SEC23A//SERINC3//CLCN5//B3GALNT2//RAB12//HS3ST5//GJA1//ATP2C1//RAB30//YIPF6//ST8SIA5//TRIM23//RND3//GCNT4//NDFIP2//ZDHHC7//SYBU//SLC30A6//PMEPA1//ACBD3//APOO//RIC3//GNPTG//CHST1//ST3GAL5//MCFD2//DSEL//MMGT1//HS3ST3A1// |
| GO:0043228 | non-membrane-bounded organelle | Cellular component | 229 | 4125 | 929 | 18698 | 1.1173544704309 | 0.0290730119874108 | 0.24172908118976 | 1.53650997264133 | 0.246501614639397 | DYNC1LI2//FBXO28//PPP1R12A//CLIP1//TTK//RASSF2//PALLD//WDR1//GJA1//SERP1//NCK1//ZMYND11//EP300//MYT1L//FMR1//HMGB3//ZBTB4//RBBP8//CLOCK//AKAP11//DDX6//E2F5//TMEM217//WDFY3//PHF8//FLNA//SIRT1//SMUG1//MDFIC//YPEL2//DNAJB9//ASNA1//MYCN//NOVA1//CUTC//CMPK1//PIK3CB//BRWD1//FBXW7//TRERF1//ATXN1//ATXN7//ZMAT3//SLC14A1//WT1//SLBP//ZNF655//SAP30L//E2F8//FXR1//PCGF5//RUNX3//DDX3Y//CCND2//GPRC5A//MOB1B//CKAP4//RDH10//ACSL4//RAB3GAP1//ABHD5//RAP1B//TSC1//LMLN//DHRS3//NEDD1//DZIP1//CEP41//MID1//MZT1//KLHL42//RB1//PTP4A1//SHCBP1//KATNAL1//KBTBD8//GPHN//WASF3//PTPN21//SLAIN1//FRMD6//DBN1//KLHL3//MYLIP//TMOD3//HSBP1//MSN//PFN2//ENAH//MAPK1//SNAP25//TMOD1//TNS1//CCDC6//SGCE//GRSF1//CFDP1//DCTN6//CENPO//DCTN5//NPNT//ZBTB18//JUN//SUV39H1//SIN3B//ESCO2//HECW2//CDC14A//SEPT9//CFL2//IQGAP2//CNN3//SAMD14//ARC//CD2AP//CLIC4//KLHL20//ARHGAP6//MARCKS//TMEM63B//ALG2//TACC2//PPP2CA//MID1IP1//LASP1//MAPK6//KRT80//SMARCA2//ZNF131//SESTD1//RIMS4//BSN//PNRC1//DCP2//PATL1//TNRC6B//PAN3//AGO1//CNOT7//YTHDF2//PSMA2//CPEB1//MEX3B//MBNL1//TIA1//TDRD5//SOCS1//CEBPB//RNF2//TMEM201//PPP2CB//CALM2//VPS4B//TBL1XR1//FKBP15//YES1//DYNLL2//LCA5//FLII//PXK//RPP25//C2CD5//KIF1C//CAMSAP1//DPYSL2//REEP3//WDR47//GABARAPL1//SYBU//KIF13A//KCNAB2//CCT6A//RANBP9//LRP8//HOMER1//FAM83D//PURA//NABP1//ESR1//H2AFZ//IRF1//AR//MEF2A//POU4F1//SMARCD2//TP63//UBE2I//SYCE1//APPL1//SS18//TNNI1//DNAJB6//KCNE1//PTGES3//BAHD1//HIC1//MAF//HIST2H2BE//MBD6//JAZF1//SESN1//FOXA1//NFIB//NFIC//PAX5//FOXJ2//PAK6//BBS9//ROR1//ZYX//GLI3//ATG16L1//VPS37A//PPP4R2//POLR3H//TAPT1//ATF5//CAMSAP2//PRKACB//PRKAR2B//RRAGD//TFAP2A//ZMYND10//RAB28//EVC//DLG5//KRTAP5-6//KRTAP2-4//NAV3// |
| GO:0043232 | intracellular non-membrane-bounded organelle | Cellular component | 229 | 4125 | 929 | 18698 | 1.1173544704309 | 0.0290730119874108 | 0.24172908118976 | 1.53650997264133 | 0.246501614639397 | DYNC1LI2//FBXO28//PPP1R12A//CLIP1//TTK//RASSF2//PALLD//WDR1//GJA1//SERP1//NCK1//ZMYND11//EP300//MYT1L//FMR1//HMGB3//ZBTB4//RBBP8//CLOCK//AKAP11//DDX6//E2F5//TMEM217//WDFY3//PHF8//FLNA//SIRT1//SMUG1//MDFIC//YPEL2//DNAJB9//ASNA1//MYCN//NOVA1//CUTC//CMPK1//PIK3CB//BRWD1//FBXW7//TRERF1//ATXN1//ATXN7//ZMAT3//SLC14A1//WT1//SLBP//ZNF655//SAP30L//E2F8//FXR1//PCGF5//RUNX3//DDX3Y//CCND2//GPRC5A//MOB1B//CKAP4//RDH10//ACSL4//RAB3GAP1//ABHD5//RAP1B//TSC1//LMLN//DHRS3//NEDD1//DZIP1//CEP41//MID1//MZT1//KLHL42//RB1//PTP4A1//SHCBP1//KATNAL1//KBTBD8//GPHN//WASF3//PTPN21//SLAIN1//FRMD6//DBN1//KLHL3//MYLIP//TMOD3//HSBP1//MSN//PFN2//ENAH//MAPK1//SNAP25//TMOD1//TNS1//CCDC6//SGCE//GRSF1//CFDP1//DCTN6//CENPO//DCTN5//NPNT//ZBTB18//JUN//SIN3B//HECW2//CDC14A//CFL2//IQGAP2//SEPT9//CNN3//SAMD14//ARC//CD2AP//CLIC4//KLHL20//ARHGAP6//MARCKS//TMEM63B//ALG2//TACC2//PPP2CA//MID1IP1//MAPK6//KRT80//SMARCA2//ZNF131//SESTD1//DCP2//PATL1//YTHDF2//SOCS1//TIA1//SUV39H1//ESCO2//LASP1//RIMS4//BSN//PNRC1//TNRC6B//PAN3//AGO1//CNOT7//PSMA2//CPEB1//MEX3B//MBNL1//TDRD5//CEBPB//RNF2//TMEM201//PPP2CB//CALM2//VPS4B//TBL1XR1//FKBP15//YES1//DYNLL2//LCA5//FLII//PXK//RPP25//C2CD5//KIF1C//CAMSAP1//DPYSL2//REEP3//WDR47//GABARAPL1//SYBU//KIF13A//KCNAB2//CCT6A//RANBP9//LRP8//HOMER1//FAM83D//PURA//NABP1//ESR1//H2AFZ//IRF1//AR//MEF2A//POU4F1//SMARCD2//TP63//UBE2I//SYCE1//APPL1//SS18//TNNI1//DNAJB6//KCNE1//PTGES3//BAHD1//HIC1//MAF//HIST2H2BE//MBD6//JAZF1//SESN1//FOXA1//NFIB//NFIC//PAX5//FOXJ2//PAK6//BBS9//ROR1//ZYX//GLI3//ATG16L1//VPS37A//PPP4R2//POLR3H//TAPT1//ATF5//CAMSAP2//PRKACB//PRKAR2B//RRAGD//TFAP2A//ZMYND10//RAB28//EVC//DLG5//KRTAP5-6//KRTAP2-4//NAV3// |
| GO:0010369 | chromocenter | Cellular component | 3 | 14 | 929 | 18698 | 4.31293249269568 | 0.0295101147736707 | 0.24172908118976 | 1.5300291013955 | 0.00322927879440258 | MBD6//ESCO2//FMR1// |
| GO:0098791 | Golgi subcompartment | Cellular component | 53 | 819 | 929 | 18698 | 1.30248103768018 | 0.0295477922292489 | 0.24172908118976 | 1.52947496346207 | 0.0570505920344456 | CNIH1//STX6//RTN3//SEC23A//FUT9//SERINC3//CLCN5//VTI1A//B3GALNT2//RAB12//HS3ST5//FUT5//GJA1//ATP2C1//ARFIP1//RAB30//YIPF6//ST8SIA5//TRIM23//RND3//GCNT4//NDFIP2//ZDHHC7//SYBU//SLC30A6//PMEPA1//ACBD3//APOO//RIC3//GNPTG//CHST1//ST3GAL5//AP1S2//MCFD2//DSEL//MMGT1//HS3ST3A1//SULF1//RAB21//KLHL20//SNAP25//C16ORF70//TJAP1//GOLGA6A//CABP7//NSG1//KIF13A//CALN1//VAMP3//FUT2//TEX261//STEAP2//IER3IP1// |
| GO:0042175 | nuclear outer membrane-endoplasmic reticulum membrane network | Cellular component | 67 | 1071 | 929 | 18698 | 1.25911318958872 | 0.0301664405842077 | 0.244462004356928 | 1.52047593032327 | 0.0721205597416577 | UGT2B28//NAV3//GJC1//PYURF//LPCAT3//CNIH1//RTN3//CDS1//SLC9A6//SEC23A//CKAP4//TMED1//VTI1A//B3GALNT2//RDH10//SPTSSB//SPTSSA//SMIM14//ACSL1//ACSL4//REEP3//RAB3GAP1//RAB18//RAB21//SLC35D1//ARL6IP1//LCLAT1//GJA1//NSG1//SERP1//ANXA7//KCNK2//SHISA2//DNAJB9//MGST1//NR3C2//ASNA1//IER3IP1//INSIG2//PIGP//JKAMP//PIGA//PLP2//SEC61A2//STIM2//RDH14//ELOVL5//MSMO1//SGK1//TMBIM6//UGT2B7//APOO//RIC3//ELOVL7//CALU//ALG2//MCFD2//DHRS3//EI24//CNEP1R1//TEX261//TAPT1//LBR//RTN1//SPPL3//EMC7//MMGT1// |
| GO:0005856 | cytoskeleton | Cellular component | 124 | 2126 | 929 | 18698 | 1.17391828274062 | 0.031063652144448 | 0.248074188855212 | 1.50774748573115 | 0.133476856835307 | CFL2//IQGAP2//SEPT9//CNN3//DBN1//SAMD14//PALLD//FLNA//ARC//CD2AP//CLIC4//KLHL20//ARHGAP6//MARCKS//PPP1R12A//TMEM63B//ALG2//TACC2//PPP2CA//MID1IP1//CLIP1//ATXN7//TMOD1//MAPK6//KRT80//SMARCA2//ZNF131//SESTD1//LASP1//WDR1//RIMS4//BSN//TMOD3//DCTN6//FKBP15//TSC1//YES1//DYNLL2//AKAP11//LCA5//DZIP1//FLII//YTHDF2//PXK//RPP25//MAPK1//C2CD5//MID1//MZT1//KLHL42//RB1//TTK//PTP4A1//SHCBP1//KATNAL1//KBTBD8//KIF1C//CAMSAP1//DYNC1LI2//DPYSL2//REEP3//WDR47//GABARAPL1//SYBU//KIF13A//KCNAB2//CCT6A//RANBP9//LRP8//GJA1//NEDD1//BBS9//TMEM201//PPP2CB//CALM2//CDC14A//VPS4B//DDX6//ROR1//ZYX//GLI3//ATG16L1//VPS37A//PPP4R2//POLR3H//TAPT1//ATF5//CAMSAP2//PRKACB//PRKAR2B//RRAGD//TFAP2A//PCGF5//DCTN5//CEP41//TNNI1//TBL1XR1//SERP1//SS18//ZMYND10//RAB28//EVC//DLG5//KRTAP5-6//KRTAP2-4//HECW2//FAM83D//NAV3//GPHN//WASF3//CKAP4//PTPN21//SLAIN1//FRMD6//KLHL3//MYLIP//HSBP1//MSN//PFN2//ENAH//SNAP25//TNS1//CCDC6//SGCE// |
| GO:0043198 | dendritic shaft | Cellular component | 5 | 36 | 929 | 18698 | 2.79541920822868 | 0.031467219613137 | 0.248074188855212 | 1.50214162886997 | 0.00538213132400431 | LPAR1//FLNA//ZMYND8//PRKAR2B//SYNGAP1// |
| GO:0019898 | extrinsic component of membrane | Cellular component | 22 | 289 | 929 | 18698 | 1.5321605625724 | 0.0314785641271456 | 0.248074188855212 | 1.50198508590254 | 0.0236813778256189 | PIK3CA//PIK3CB//FMR1//DTNA//SNX5//ERRFI1//SNAP25//TGM3//TXK//YES1//KCNAB2//PLEKHF2//WDFY3//PSD//CDH20//GNAQ//NRBF2//SEC23A//CNTFR//SYT6//WDR45B//SNX2// |
| GO:0000775 | chromosome, centromeric region | Cellular component | 16 | 194 | 929 | 18698 | 1.6599602721028 | 0.0327133259615173 | 0.255461336372212 | 1.48527529894126 | 0.0172228202368138 | DYNC1LI2//FBXO28//PPP1R12A//CLIP1//TTK//RASSF2//CEBPB//CFDP1//DCTN6//CENPO//DCTN5//ESCO2//FMR1//PPP2CA//PPP2CB//SUV39H1// |
| GO:0005639 | integral component of nuclear inner membrane | Cellular component | 3 | 15 | 929 | 18698 | 4.0254036598493 | 0.0355571056575354 | 0.270296935927636 | 1.44907359776142 | 0.00322927879440258 | TMEM201//LEMD3//LBR// |
| GO:0016514 | SWI/SNF complex | Cellular component | 3 | 15 | 929 | 18698 | 4.0254036598493 | 0.0355571056575354 | 0.270296935927636 | 1.44907359776142 | 0.00322927879440258 | RB1//SMARCA2//SMARCD2// |
| GO:0031229 | intrinsic component of nuclear inner membrane | Cellular component | 3 | 15 | 929 | 18698 | 4.0254036598493 | 0.0355571056575354 | 0.270296935927636 | 1.44907359776142 | 0.00322927879440258 | TMEM201//LEMD3//LBR// |
| GO:0000803 | sex chromosome | Cellular component | 4 | 26 | 929 | 18698 | 3.09646435373023 | 0.037870293081833 | 0.281961389209473 | 1.42170133381949 | 0.00430570505920344 | SIN3B//PCGF5//RNF2//ESCO2// |
| GO:1990752 | microtubule end | Cellular component | 4 | 26 | 929 | 18698 | 3.09646435373023 | 0.037870293081833 | 0.281961389209473 | 1.42170133381949 | 0.00430570505920344 | CLIP1//CAMSAP1//CAMSAP2//NAV3// |
| GO:0030659 | cytoplasmic vesicle membrane | Cellular component | 49 | 761 | 929 | 18698 | 1.29595781427474 | 0.0380762760748531 | 0.281961389209473 | 1.41934553295632 | 0.0527448869752422 | IQGAP2//RAB18//RAB9B//RAB8B//C5AR1//WNT3//WNT7A//CNIH1//SEC23A//VTI1A//MCFD2//SYT1//CKAP4//BRI3//MGST1//RAP1B//CHRNB4//RAP1A//SNAP25//VAMP1//KCNAB2//ANXA7//SIRPA//RAP2C//CHRM2//VAMP3//LDLR//SYT6//SEMA4C//C16ORF70//STX6//EPS15//AFTPH//ZPLD1//ZNRF2//RAB21//GABARAPL1//CLIC4//NSG1//SNX5//SNX19//CLIP1//YWHAG//FZD6//AP1S2//GPRC5A//ULK2//SNX17//C2CD5// |
| GO:0005789 | endoplasmic reticulum membrane | Cellular component | 65 | 1050 | 929 | 18698 | 1.24595827566764 | 0.0388892676097449 | 0.28552034937411 | 1.41017023571707 | 0.069967707212056 | PYURF//PIGP//PIGA//SPTSSB//SPTSSA//TEX261//TAPT1//ARL6IP1//LBR//ELOVL5//RTN1//ELOVL7//SPPL3//INSIG2//EMC7//MMGT1//GJC1//LPCAT3//CNIH1//RTN3//CDS1//SLC9A6//SEC23A//CKAP4//TMED1//VTI1A//B3GALNT2//RDH10//SMIM14//ACSL1//ACSL4//REEP3//RAB3GAP1//RAB18//RAB21//SLC35D1//LCLAT1//GJA1//NSG1//SERP1//ANXA7//KCNK2//SHISA2//DNAJB9//MGST1//NR3C2//ASNA1//IER3IP1//JKAMP//PLP2//UGT2B28//SEC61A2//STIM2//RDH14//MSMO1//SGK1//TMBIM6//UGT2B7//APOO//RIC3//CALU//ALG2//MCFD2//DHRS3//EI24// |
| GO:0016607 | nuclear speck | Cellular component | 27 | 381 | 929 | 18698 | 1.42632413144267 | 0.0409714226997014 | 0.292027082840122 | 1.38751895525843 | 0.0290635091496232 | CKAP4//FAM76B//DENND1B//GADD45A//PATL1//PPP1R16B//HBP1//GLI3//CNOT7//AR//MEOX2//NR4A2//OGG1//SERPINB13//PRKAA1//SLC2A4RG//CCNL1//RBM25//RAF1//SRSF2//SRSF6//SGK1//S100PBP//WT1//ZNF217//CBLL1//DAZAP2// |
| GO:0098827 | endoplasmic reticulum subcompartment | Cellular component | 65 | 1054 | 929 | 18698 | 1.24122978126283 | 0.0414992928204533 | 0.292027082840122 | 1.38195930393349 | 0.069967707212056 | GJC1//PYURF//LPCAT3//CNIH1//RTN3//CDS1//SLC9A6//SEC23A//CKAP4//TMED1//VTI1A//B3GALNT2//RDH10//SPTSSB//SPTSSA//SMIM14//ACSL1//ACSL4//REEP3//RAB3GAP1//RAB18//RAB21//SLC35D1//ARL6IP1//LCLAT1//GJA1//NSG1//SERP1//ANXA7//KCNK2//SHISA2//DNAJB9//MGST1//NR3C2//ASNA1//IER3IP1//INSIG2//PIGP//JKAMP//PIGA//PLP2//UGT2B28//SEC61A2//STIM2//RDH14//ELOVL5//MSMO1//SGK1//TMBIM6//UGT2B7//APOO//RIC3//ELOVL7//CALU//ALG2//MCFD2//DHRS3//EI24//TEX261//TAPT1//LBR//RTN1//SPPL3//EMC7//MMGT1// |
| GO:0034045 | phagophore assembly site membrane | Cellular component | 3 | 16 | 929 | 18698 | 3.77381593110872 | 0.0421885816471096 | 0.292027082840122 | 1.37480507507292 | 0.00322927879440258 | ATG16L1//WDR45B//ULK2// |
| GO:0038201 | TOR complex | Cellular component | 3 | 16 | 929 | 18698 | 3.77381593110872 | 0.0421885816471096 | 0.292027082840122 | 1.37480507507292 | 0.00322927879440258 | RICTOR//SESN1//PRR5L// |
| GO:0044453 | nuclear membrane part | Cellular component | 3 | 16 | 929 | 18698 | 3.77381593110872 | 0.0421885816471096 | 0.292027082840122 | 1.37480507507292 | 0.00322927879440258 | TMEM201//LEMD3//LBR// |
| GO:0032839 | dendrite cytoplasm | Cellular component | 4 | 27 | 929 | 18698 | 2.98178048877726 | 0.0427678276485752 | 0.292027082840122 | 1.36888280876112 | 0.00430570505920344 | GABARAPL1//GRIK2//MAPK1//TRAK2// |
| GO:0031968 | organelle outer membrane | Cellular component | 16 | 201 | 929 | 18698 | 1.60215071038778 | 0.0430471710998507 | 0.292027082840122 | 1.36605538349238 | 0.0172228202368138 | UGT2B28//NAV3//ACSL1//ACSL4//SLC44A1//GJA1//MTX3//MGST1//PMAIP1//ARMCX6//MIEF1//MAVS//RAF1//BCL2A1//VAMP1//ARMCX3// |
| GO:0098796 | membrane protein complex | Cellular component | 71 | 1166 | 929 | 18698 | 1.22557315544297 | 0.0431249549094008 | 0.292027082840122 | 1.36527134600815 | 0.0764262648008611 | PYURF//PIGP//PIGA//VPS37A//CHMP3//AP1S2//PIK3CA//PIK3CB//MEP1A//SNX5//SNX2//SNX27//STX6//VTI1A//SNAP25//VAMP1//SYT1//VAMP3//INSIG2//RRAGD//CNEP1R1//EMC7//MMGT1//EVC//CNGA3//NRBF2//SPTSSB//SPTSSA//ARL6IP1//GNAQ//SCN1B//EPS15//GJC1//GJA1//KCNH4//KCNA4//KCNB1//KCNE1//KCNJ2//KCNJ3//KCNK2//KCNIP4//KCNAB2//SGCE//CDH20//GLRB//GLRA3//IGF1//LRP6//SLC25A6//COX8C//IMMP2L//TIMM9//APOO//AFTPH//SEC23A//CHRNB4//EPS8//GRIK2//CALM2//SESTD1//GABRA5//CLIC4//ANO1//ITGB8//CD3E//CD8A//ACVR1//TGFBR2//CNTFR//NPNT// |
| GO:0031201 | SNARE complex | Cellular component | 6 | 52 | 929 | 18698 | 2.32234826529767 | 0.0431728119875058 | 0.292027082840122 | 1.36478966339181 | 0.00645855758880517 | SNAP25//STX6//VTI1A//VAMP1//SYT1//VAMP3// |
| GO:0045177 | apical part of cell | Cellular component | 26 | 366 | 929 | 18698 | 1.42978818519237 | 0.043175133318621 | 0.292027082840122 | 1.36476631271934 | 0.0279870828848224 | CNTFR//EPS15//GJA1//DLL1//KCNE1//KCNK2//MSN//SLC26A4//ANO1//PRKAA1//PTEN//SLC10A2//BMPR2//FZD3//FZD6//VAMP3//RAPGEF2//SLC23A2//CLCN5//NEDD1//CLIC4//LDLR//MGST1//C5AR1//ACVR1//HOMER1// |
| GO:0031252 | cell leading edge | Cellular component | 27 | 385 | 929 | 18698 | 1.41150517942768 | 0.0456022817300269 | 0.306034062547602 | 1.34101342667836 | 0.0290635091496232 | PALLD//CD2AP//SNX5//TNFRSF12A//RASA1//CLIP1//IQGAP2//WASF3//STMN2//FAM89B//NRBP1//PIK3CA//ENAH//ITSN1//SNX2//TSC1//NRG1//ROBO2//EPS8//ARHGEF4//PSD//C2CD5//GABARAPL1//GABRA5//KCNB1//SGCE//SLK// |
| GO:0019867 | outer membrane | Cellular component | 16 | 203 | 929 | 18698 | 1.58636597432485 | 0.0463854520056971 | 0.308876769557316 | 1.3336182070384 | 0.0172228202368138 | UGT2B28//NAV3//ACSL1//ACSL4//SLC44A1//GJA1//MTX3//MGST1//PMAIP1//ARMCX6//MIEF1//MAVS//RAF1//BCL2A1//VAMP1//ARMCX3// |
| GO:0031143 | pseudopodium | Cellular component | 3 | 17 | 929 | 18698 | 3.55182675869056 | 0.0493917558359166 | 0.325853639387101 | 1.30634553475573 | 0.00322927879440258 | MSN//MAPK1//RAF1// |
| GO:0048471 | perinuclear region of cytoplasm | Cellular component | 43 | 668 | 929 | 18698 | 1.29560147734671 | 0.0496936283582191 | 0.325853639387101 | 1.30369929227793 | 0.046286329386437 | SYT6//DNAJB6//STX6//SEC23A//SEPT9//COPS8//SERINC3//CKAP4//STMN2//VTI1A//CABP7//FLNA//FMR1//CD2AP//CLIC4//PPP1R16B//KLHL20//NANOS1//INHBB//ARHGAP1//MSN//RASD1//RAB8B//WDR44//NDFIP2//PPARG//PRKACB//PRKAR2B//RAP1A//STK33//SNAP25//BNIP2//TSC1//FXR1//ANP32A//CALN1//ALG2//CCNT2//VAMP3//BAG5//PPM1F//RAPGEF2//SLK// |
