# Supplementary material for: Altered microRNA Transcriptome in Cultured Human Liver Cells upon Infection with Ebola Virus
Source: Int J Mol Sci. 2021 Apr 6;22(7):3792. doi: 10.3390/ijms22073792 (PMC8038836; doi:10.3390/ijms22073792)
Supplement: Supplementary file 1 [file ijms-22-03792-s001.zip › Supplementary_File/C_ GO_Analysis_Results/16-30nt_go_Makona-24h-Huh7_vs_Control-24h-Huh7_up.mature_mirna_targets/MF_result(Human).html]

| GO.ID | Term | Ontology | Count | Pop.Hits | List.Total | Pop.Total | Fold.Enrichment | Pvalue | FDR | Enrichment.Score | Gene.Ratio | GENES |
| --- | --- | --- | --- | --- | --- | --- | --- | --- | --- | --- | --- | --- |
| GO:0001067 | regulatory region nucleic acid binding | Molecular function | 96 | 878 | 906 | 17548 | 2.11775709393715 | 1.68863319114908e-12 | 2.18846861572921e-09 | 11.7724646786473 | 0.105960264900662 | BAHD1//NR5A2//MEF2A//NFE2//PPARG//RORA//SOX4//SUV39H1//TFAP2A//CEBPB//ZMYND11//CREBRF//ETV5//ATF5//FOXF1//FOXF2//ZMYND8//GLI3//PURG//IRF2//JARID2//MYOG//NHLH2//NR4A2//KLF3//ZBTB4//PURA//ARID5B//RUNX3//GSC//CC2D1B//ELK3//EN1//EP300//ESR1//ETS1//FLI1//AGO1//H2AFZ//IRF1//AR//ISL1//JUN//MEOX2//MYB//MYBL1//MYCN//NFIA//NFIB//NFIC//OTX2//PAX5//KLF13//POU3F2//FOXJ2//RFX7//SMARCD2//TFE3//TGIF1//KLF10//TXK//ZNF217//BHLHE41//E2F8//FOSL1//KLF7//FUBP3//FOXP2//CLOCK//GADD45A//ARX//POU4F1//ZNF516//RB1//SIRT1//SARS//NPAS2//ST18//MBNL1//SLC2A4RG//HIVEP2//FOXA1//ARID4B//ZNF532//TRERF1//HIVEP3//SMARCA2//TNF//WT1//TBL1XR1//TEAD2//TP63//NEUROD1//NEUROG1//ZEB1//BHLHE40// |
| GO:0044212 | transcription regulatory region DNA binding | Molecular function | 95 | 877 | 906 | 17548 | 2.09808674464674 | 3.82551575743711e-12 | 2.47893421081925e-09 | 11.4173100048217 | 0.10485651214128 | BAHD1//NR5A2//MEF2A//NFE2//PPARG//RORA//SOX4//SUV39H1//TFAP2A//CEBPB//ZMYND11//CREBRF//ETV5//ATF5//FOXF1//FOXF2//ZMYND8//GLI3//PURG//IRF2//JARID2//MYOG//NHLH2//NR4A2//KLF3//ZBTB4//PURA//ARID5B//RUNX3//GSC//CC2D1B//ELK3//EN1//EP300//ESR1//ETS1//FLI1//AGO1//H2AFZ//IRF1//AR//ISL1//JUN//MEOX2//MYB//MYBL1//MYCN//NFIA//NFIB//NFIC//OTX2//PAX5//KLF13//POU3F2//FOXJ2//RFX7//SMARCD2//TFE3//TGIF1//KLF10//TXK//ZNF217//BHLHE41//E2F8//FOSL1//KLF7//FUBP3//FOXP2//CLOCK//GADD45A//ARX//POU4F1//ZNF516//RB1//SIRT1//SARS//NPAS2//ST18//SLC2A4RG//NEUROD1//NEUROG1//ZEB1//BHLHE40//HIVEP2//FOXA1//ARID4B//ZNF532//TRERF1//HIVEP3//SMARCA2//TNF//WT1//TBL1XR1//TEAD2//TP63// |
| GO:0005515 | protein binding | Molecular function | 688 | 11565 | 906 | 17548 | 1.15223809373834 | 1.16816951902201e-11 | 5.04649232217508e-09 | 10.9324941299686 | 0.759381898454746 | STX6//VTI1A//VAMP1//SYT1//VAMP3//AGO1//GTF2E1//GSC//ZFPM2//ID2//AR//MEF2A//TEAD2//ESR1//EP300//ISL1//JUN//NEUROD1//NHLH2//RB1//BHLHE41//BHLHE40//TCERG1//GTF2A1//RBBP8//BDP1//FAM89B//RORA//CCNT2//S1PR1//FLNA//GNAQ//CRKL//MAPK1//RASA1//YES1//SOCS3//CR2//C5AR1//LPAR1//IGFBP3//F3//ALPI//LDLR//SERPINB13//TNF//TNFAIP3//SIRT1//ZMAT3//TP63//IQGAP2//WASF3//CNN3//DBN1//EPS8//LIMCH1//PALLD//FLII//FKBP15//KLHL3//KLHL20//TMOD3//MSN//PFN2//PXK//ENAH//TMOD1//TNNI1//TNS1//TOB1//ARHGAP1//LASP1//ARHGAP6//NCK1//SH3BGRL//SKAP2//MARCKS//SRSF2//YWHAG//RAPGEF4//RAB3GAP1//ARHGEF4//DOCK10//ITSN1//SOS2//RALGPS1//IQSEC2//FBXO8//PSD//RANBP9//ADRB1//EREG//RASGEF1A//NRG1//IL2//MET//RAPGEF2//PLEKHG4B//ARPP19//TMED1//STRAP//NXPH3//C1QTNF2//ADCYAP1//CNTFR//EFNA1//GJA1//NSG1//ITGB8//LRP6//NTF3//RAB8B//CYTL1//CCL2//BMP3//TRAK2//TXK//WNT7A//BAG6//SNX17//NPTN//BAMBI//WNT3//WNT10A//DLL1//EDN1//TXLNA//IFNA1//IFNG//INHBB//CXCL9//GDF5//IRAK4//SNX2//SOCS5//IGF1//SOCS1//PTEN//SIVA1//CD2AP//SPRED1//NPNT//ANXA7//KDR//EDN2//PTHLH//SNAP25//ADCY3//CAMSAP1//CAMSAP2//PPP3R1//STRN//TMEM201//LBR//CCL8//CCL21//GLI3//NR4A2//SKP1//TBL1XR1//DLG5//KIF1C//DPYSL2//FMR1//MID1//OGG1//SYBU//CLIP1//KIF13A//FAM83D//KATNAL1//NAV3//CNGA3//NPAT//HPCAL4//PPARG//PPP2CA//PPP2CB//PRKAA1//MID1IP1//ATXN1//SDC1//VPS4B//OGN//VEGFB//DYNLL2//CDH20//MYLIP//PPARGC1B//E2F5//ETS1//MED19//FOXF2//CNOT7//MDFIC//FOXA1//JARID2//NFIA//INSIG2//TRERF1//PURA//CCND1//ZEB1//UBE2I//AKAP11//RAF1//CALM2//OTX2//RGL1//TNPO2//STMN2//ATF5//VBP1//SPHK2//RAP1A//DENND1B//DENND6A//TRAPPC8//RIN2//SBF2//SIRPA//EPS15//ERRFI1//CCDC6//ADAM12//SYNGAP1//CD3E//QKI//RIMS4//TBC1D12//APCDD1//FRZB//ROR1//FZD3//FZD6//RALBP1//DUSP1//BMPR2//LRP8//CDK19//GSTM3//MDM4//PCBP2//EGLN1//BTG1//TMBIM6//CASP2//HDHD2//CEBPB//GADD45A//MYCN//MOB1B//CACUL1//CCNY//RICTOR//RHOB//RND3//PPP1R12A//MAPK6//MAVS//ZBTB4//CCND2//CD8A//BAG5//RCAN3//MTMR9//DDX6//ZNF521//ARFIP1//PRKAR2B//SYT6//NOG//PLP2//G3BP2//DTNA//GRIK2//CXXC4//SEMA4C//AFTPH//FBXW7//CDK15//DNAJB9//PPP4R2//GABARAPL1//DNAJB6//DNAJA2//HSPA13//ARAP2//APPL1//AGFG1//PEX5L//GIT2//KCNE1//TGFBR2//ZFAND6//DCUN1D4//ANKIB1//LONRF3//LONRF1//SPOPL//JKAMP//UBE2W//PRKACB//UBE2V1//PRR5L//CUL5//ARRDC3//EDNRB//PPP1R1B//TSC1//SEPT9//RIC3//PRDM16//ZNF516//GSKIP//ACBD3//SNX5//ZMYND11//PHF13//PHF8//ZMYND8//HOMER1//TACC2//SHCBP1//ATAD2//SMARCA2//SAP30L//ANP32A//C1QTNF6//CLCN5//ARL6IP1//BRI3//FOXP1//YIPF6//TBK1//HPRT1//HSBP1//TRIM23//KCNJ2//RAB9B//IER5//PON2//MIEF1//POU3F2//THG1L//ATG16L1//FOXJ2//ZBTB26//PTPRG//ROBO2//BNIP2//TSN//ZNF3//ADIPOR2//CBLL1//ACCS//NMI//SLIT2//FOXP2//SLK//DAZAP2//SPPL3//TIMM9//NRBP1//FIBIN//MGST1//NAGA//NEUROG1//CHMP3//BCL2A1//SARS//DLK2//TFAP2A//TTK//E2F8//FXR1//GNPTG//ACVR1//CD69//DNMT3B//HIC1//MIER1//MZT1//UBXN2B//PLAA//PIK3CA//PIK3CB//S100A7A//CCT6A//KCNB1//MB21D2//TES//PAK6//CAST//GPRC5A//DYNC1LI2//RAET1E//EFNB2//CHRNB4//H2AFZ//MYOG//PKNOX1//RRAGD//SOX4//HIST2H2BE//ALG2//NPAS2//ANO1//TFE3//CLOCK//GLRX//NFE2//SUV39H1//S100PBP//ADNP//NDFIP2//PMEPA1//GABRA5//NRXN3//CFL2//SAMD14//WDR1//PALM2//POU4F1//PTGES3//DNAJB5//TGIF1//SMURF1//LDLRAD4//WT1//RNF2//HOXA3//TEX261//CHRM2//C8ORF44-SGK3//PYURF//GPHN//RTN3//ZBTB18//SEC23A//CELF1//DCTN6//RAI2//COPS8//FASTK//PNRC1//PTPN21//RASSF8//EGLN3//KLF12//SORCS1//RHEBL1//NEDD1//SLAIN1//FRMD6//EDARADD//LYPD6//VPS37A//CRHR1//FAM76B//KRT80//GSG1L//PDIK1L//SH3D19//CREBRF//ADSS//C12ORF50//MARCH10//SPTSSB//DCP2//LCA5//SPTSSA//ARID2//ELAVL2//CC2D1B//ELK3//RAB12//EMX2//SMIM14//ETV5//UNC5B//PATL1//ATXN7L1//HS3ST5//CPEB3//DZIP1//BAHD1//WDR47//RAB18//ELL2//KDM2A//WDFY3//RAB21//MYCBP2//TNRC6B//FLI1//FAM168A//FBXO28//OTUD3//SIN3B//MORC3//TTC33//SMUG1//LEMD3//KPNA6//SGK3//CNOT6L//NR5A2//LCLAT1//FBXO33//NAALADL2//CNEP1R1//PAN3//NUTM1//ARMC8//CLIC4//RWDD3//RNF167//PPP1R16B//HBP1//RNF11//SERP1//BBS9//SESN1//GLRB//GPM6A//ZDHHC22//GRK6//ASTE1//C16ORF72//GRSF1//SLC25A6//GSTA2//GTF2H1//ST8SIA5//LRP12//NRBF2//HMGB3//NANOS1//IRF1//IRF2//KCNA4//NHLRC2//KCNJ3//KCNJ10//SNX19//FAM19A1//MAF//MBNL1//MEOX2//MEP1A//AFF1//MLLT6//NR3C2//ASNA1//KRTAP5-6//MTRR//MYB//NEU1//DCAF8//PAX5//ASCC1//MEMO1//CUTC//SEPSECS//PHF20L1//PAIP2//KLF3//CDK17//FAM53C//TNFRSF12A//PRR16//ZMYND10//COMMD10//YTHDF2//KLF13//RASD1//ARID4B//PIGA//NUP54//PMAIP1//RPP25//C2ORF42//MED9//C19ORF73//RBM41//PPP2R5E//DRAM1//PPP3R2//KLK15//INTS8//LRP2BP//NPDC1//NRIP3//PSMA2//FSTL5//CCNL1//TMEM159//PSMD12//CNOT6//HECW2//KLHL42//TAOK1//RANBP10//STIM2//USP28//RBM25//RAP1B//RBMS1//ELOVL5//RTN1//SLC22A23//ATXN7//SCN1B//MCCC2//SMOC1//SRSF6//SGK1//GIGYF1//NABP1//SMARCD2//BOLL//SOX5//SS18//MED22//BTF3//KLF10//TIA1//TRPC3//MPPED2//ZNF217//ZNF227//ZYX//PTP4A1//CSDE1//SLBP//ZNF655//APOO//CENPO//FAM118B//PLEKHF2//CLIP4//SNIP1//UBA5//ELOVL7//C16ORF70//KCNIP4//FOSL1//PTP4A2//CALU//SNX27//CCM2//ARID5B//PCGF5//RECK//LCOR//DCTN5//KBTBD8//TMEM185A//PPP1R15B//FAM104A//PPFIA1//KRTAP2-4//CDC14A//RUNX3//CBFB//PDE5A//AP1S2//FUBP3//C5ORF30//MCFD2//DIRAS3//SESTD1//BTF3L4//WDR20//RPS6KA5//FAM114A1//NREP//SYCE1//FBXO44//TJAP1//CEP41//FEZ2//PPM1F//PHF14//ULK2//SECISBP2L//RASSF2// |
| GO:0000982 | transcription factor activity, RNA polymerase II proximal promoter sequence-specific DNA binding | Molecular function | 55 | 415 | 906 | 17548 | 2.5669299715418 | 1.19393144206684e-10 | 3.86833787229656e-08 | 9.92302061054911 | 0.0607064017660044 | CEBPB//ELK3//ESR1//ETS1//FLI1//SIRT1//NR5A2//GLI3//FOXA1//IRF1//AR//ISL1//JUN//MEF2A//MEOX2//MYB//MYBL1//MYCN//MYOG//NEUROD1//NFIA//NFIB//NFIC//NHLH2//NR4A2//OTX2//PAX5//KLF13//POU3F2//POU4F1//FOXJ2//SOX4//TFAP2A//TFE3//KLF10//TXK//WT1//FOSL1//KLF7//TP63//FUBP3//TSHZ1//GSC//CC2D1B//EN1//MYT1L//ZFPM2//RB1//TGIF1//ZNF217//BHLHE41//E2F8//BHLHE40//FOXP2//CLOCK// |
| GO:0000976 | transcription regulatory region sequence-specific DNA binding | Molecular function | 80 | 740 | 906 | 17548 | 2.09390847801444 | 2.4103281831683e-10 | 6.24757065077223e-08 | 9.61792382114484 | 0.0883002207505519 | CEBPB//ZMYND11//CREBRF//ETV5//ATF5//FOXF1//FOXF2//ZMYND8//GLI3//PURG//IRF2//JARID2//MEF2A//MYOG//NHLH2//NR4A2//KLF3//PPARG//ZBTB4//PURA//RORA//TFAP2A//ARID5B//RUNX3//GSC//CC2D1B//ELK3//EN1//EP300//ESR1//ETS1//FLI1//NR5A2//AGO1//H2AFZ//IRF1//AR//ISL1//JUN//MEOX2//MYB//MYBL1//MYCN//NFIA//NFIB//NFIC//OTX2//PAX5//KLF13//POU3F2//FOXJ2//RFX7//SMARCD2//TFE3//TGIF1//KLF10//TXK//ZNF217//BHLHE41//E2F8//FOSL1//KLF7//FUBP3//FOXP2//CLOCK//GADD45A//ARX//POU4F1//NFE2//ZNF516//SIRT1//SARS//SOX4//SLC2A4RG//NEUROD1//NEUROG1//ZEB1//BHLHE40//BAHD1//SUV39H1// |
| GO:0000981 | RNA polymerase II transcription factor activity, sequence-specific DNA binding | Molecular function | 106 | 1108 | 906 | 17548 | 1.85295781831512 | 3.61795525308139e-10 | 7.8147833466558e-08 | 9.44153680880291 | 0.116997792494481 | ETS1//JUN//MEF2A//TFAP2A//FOSL1//CLOCK//CEBPB//ELK3//ESR1//FLI1//SIRT1//NR5A2//GLI3//FOXA1//IRF1//AR//ISL1//MEOX2//MYB//MYBL1//MYCN//MYOG//NEUROD1//NFIA//NFIB//NFIC//NHLH2//NR4A2//OTX2//PAX5//KLF13//POU3F2//POU4F1//FOXJ2//SOX4//TFE3//KLF10//TXK//WT1//KLF7//TP63//FUBP3//TSHZ1//GSC//CC2D1B//EN1//MYT1L//ZFPM2//RB1//TGIF1//ZNF217//BHLHE41//E2F8//BHLHE40//FOXP2//ARX//PURG//HIC1//JARID2//ZBTB4//PURA//ZEB1//ARID5B//CREBRF//EP300//ETV5//ATF5//FOXF1//FOXF2//IRF2//MAF//PKNOX1//RORA//TEAD2//PPARG//ZBTB18//KLF12//CREBL2//E2F5//EMX2//JAZF1//ADNP//HBP1//FOXP1//HIVEP2//HOXA3//HOXD1//ID2//BOLA3//NR3C2//NEUROG1//NFE2//NPAS2//TRERF1//MIER1//HIVEP3//RFX7//SOX5//ZNF3//ZNF708//ZNF131//ZSCAN5A//LCOR//RUNX3//CBFB//ST18// |
| GO:0001012 | RNA polymerase II regulatory region DNA binding | Molecular function | 73 | 665 | 906 | 17548 | 2.12618300718684 | 7.85265816413042e-10 | 1.45386356867329e-07 | 9.10498330750923 | 0.0805739514348786 | CEBPB//ZMYND11//CREBRF//ETV5//ATF5//FOXF1//FOXF2//ZMYND8//GLI3//PURG//IRF2//JARID2//MEF2A//MYOG//NHLH2//NR4A2//KLF3//PPARG//ZBTB4//PURA//RORA//TFAP2A//ARID5B//RUNX3//GSC//CC2D1B//ELK3//EN1//EP300//ESR1//ETS1//FLI1//NR5A2//AGO1//H2AFZ//IRF1//AR//ISL1//JUN//MEOX2//MYB//MYBL1//MYCN//NFIA//NFIB//NFIC//OTX2//PAX5//KLF13//POU3F2//FOXJ2//RFX7//SMARCD2//TFE3//TGIF1//KLF10//TXK//ZNF217//BHLHE41//E2F8//FOSL1//KLF7//FUBP3//FOXP2//CLOCK//GADD45A//ARX//POU4F1//NEUROD1//NEUROG1//ZEB1//BHLHE40//RB1// |
| GO:0001077 | transcriptional activator activity, RNA polymerase II proximal promoter sequence-specific DNA binding | Molecular function | 41 | 279 | 906 | 17548 | 2.84628957092106 | 1.34973842369121e-09 | 2.18657624637976e-07 | 8.86975038865656 | 0.0452538631346578 | CEBPB//ELK3//ESR1//ETS1//FLI1//SIRT1//NR5A2//GLI3//FOXA1//IRF1//AR//ISL1//JUN//MEF2A//MEOX2//MYB//MYBL1//MYCN//MYOG//NEUROD1//NFIA//NFIB//NFIC//NHLH2//NR4A2//OTX2//PAX5//KLF13//POU3F2//POU4F1//FOXJ2//SOX4//TFAP2A//TFE3//KLF10//TXK//WT1//FOSL1//KLF7//TP63//FUBP3// |
| GO:0000977 | RNA polymerase II regulatory region sequence-specific DNA binding | Molecular function | 72 | 662 | 906 | 17548 | 2.10656049298734 | 1.54668985478334e-09 | 2.22723339088801e-07 | 8.81059676313313 | 0.0794701986754967 | CEBPB//GSC//CC2D1B//ELK3//EN1//EP300//ESR1//ETS1//FLI1//NR5A2//AGO1//GLI3//H2AFZ//IRF1//AR//ISL1//JUN//MEF2A//MEOX2//MYB//MYBL1//MYCN//MYOG//NFIA//NFIB//NFIC//NHLH2//NR4A2//OTX2//PAX5//KLF13//POU3F2//FOXJ2//RFX7//SMARCD2//TFAP2A//TFE3//TGIF1//KLF10//TXK//ZNF217//BHLHE41//E2F8//FOSL1//KLF7//FUBP3//FOXP2//CLOCK//GADD45A//ARX//POU4F1//NEUROD1//NEUROG1//ZEB1//BHLHE40//ZMYND11//CREBRF//ETV5//ATF5//FOXF1//FOXF2//ZMYND8//PURG//IRF2//JARID2//KLF3//PPARG//ZBTB4//PURA//RORA//ARID5B//RUNX3// |
| GO:1990837 | sequence-specific double-stranded DNA binding | Molecular function | 80 | 776 | 906 | 17548 | 1.99676839398284 | 2.25975816639393e-09 | 2.92864658364653e-07 | 8.64593803546336 | 0.0883002207505519 | BAHD1//NR5A2//MEF2A//NFE2//PPARG//RORA//SOX4//SUV39H1//TFAP2A//CEBPB//ZMYND11//CREBRF//ETV5//ATF5//FOXF1//FOXF2//ZMYND8//GLI3//PURG//IRF2//JARID2//MYOG//NHLH2//NR4A2//KLF3//ZBTB4//PURA//ARID5B//RUNX3//GSC//CC2D1B//ELK3//EN1//EP300//ESR1//ETS1//FLI1//AGO1//H2AFZ//IRF1//AR//ISL1//JUN//MEOX2//MYB//MYBL1//MYCN//NFIA//NFIB//NFIC//OTX2//PAX5//KLF13//POU3F2//FOXJ2//RFX7//SMARCD2//TFE3//TGIF1//KLF10//TXK//ZNF217//BHLHE41//E2F8//FOSL1//KLF7//FUBP3//FOXP2//CLOCK//GADD45A//ARX//POU4F1//ZNF516//SIRT1//SARS//SLC2A4RG//NEUROD1//NEUROG1//ZEB1//BHLHE40// |
| GO:0003690 | double-stranded DNA binding | Molecular function | 85 | 860 | 906 | 17548 | 1.9143436521382 | 5.20579522592449e-09 | 6.13337328436194e-07 | 8.28351291920798 | 0.0938189845474614 | BAHD1//NR5A2//MEF2A//NFE2//PPARG//RORA//SOX4//SUV39H1//TFAP2A//CEBPB//ZMYND11//CREBRF//ETV5//ATF5//FOXF1//FOXF2//ZMYND8//GLI3//PURG//IRF2//JARID2//MYOG//NHLH2//NR4A2//KLF3//ZBTB4//PURA//ARID5B//RUNX3//GSC//CC2D1B//ELK3//EN1//EP300//ESR1//ETS1//FLI1//AGO1//H2AFZ//IRF1//AR//ISL1//JUN//MEOX2//MYB//MYBL1//MYCN//NFIA//NFIB//NFIC//OTX2//PAX5//KLF13//POU3F2//FOXJ2//RFX7//SMARCD2//TFE3//TGIF1//KLF10//TXK//ZNF217//BHLHE41//E2F8//FOSL1//KLF7//FUBP3//FOXP2//CLOCK//GADD45A//ARX//POU4F1//ZNF516//SIRT1//SARS//SLC2A4RG//WT1//NEUROD1//NEUROG1//ZEB1//BHLHE40//HMGB3//AFF1//RBMS1//TP63// |
| GO:0000987 | proximal promoter sequence-specific DNA binding | Molecular function | 54 | 451 | 906 | 17548 | 2.31908488862131 | 7.11086407976685e-09 | 7.6797332061482e-07 | 8.14807762257917 | 0.0596026490066225 | CEBPB//GSC//CC2D1B//ELK3//EN1//EP300//ESR1//ETS1//FLI1//NR5A2//AGO1//GLI3//H2AFZ//IRF1//AR//ISL1//JUN//MEF2A//MEOX2//MYB//MYBL1//MYCN//MYOG//NFIA//NFIB//NFIC//NHLH2//NR4A2//OTX2//PAX5//KLF13//POU3F2//FOXJ2//RFX7//SMARCD2//TFAP2A//TFE3//TGIF1//KLF10//TXK//ZNF217//BHLHE41//E2F8//FOSL1//KLF7//FUBP3//FOXP2//CLOCK//NEUROD1//NEUROG1//ZEB1//BHLHE40//NFE2//ZNF516// |
| GO:0001228 | transcriptional activator activity, RNA polymerase II transcription regulatory region sequence-specific DNA binding | Molecular function | 51 | 417 | 906 | 17548 | 2.3688281163149 | 9.23348347171153e-09 | 9.20507275333703e-07 | 8.03463442387842 | 0.0562913907284768 | CEBPB//ELK3//ESR1//ETS1//FLI1//SIRT1//NR5A2//GLI3//FOXA1//IRF1//AR//ISL1//JUN//MEF2A//MEOX2//MYB//MYBL1//MYCN//MYOG//NEUROD1//NFIA//NFIB//NFIC//NHLH2//NR4A2//OTX2//PAX5//KLF13//POU3F2//POU4F1//FOXJ2//SOX4//TFAP2A//TFE3//KLF10//TXK//WT1//FOSL1//KLF7//TP63//FUBP3//CREBRF//EP300//ETV5//ATF5//FOXF1//FOXF2//IRF2//MAF//PKNOX1//RORA// |
| GO:0000978 | RNA polymerase II proximal promoter sequence-specific DNA binding | Molecular function | 52 | 436 | 906 | 17548 | 2.31002288514896 | 1.54914080905058e-08 | 1.43406177752111e-06 | 7.80990910527865 | 0.0573951434878587 | ESR1//JUN//MYOG//NEUROD1//NEUROG1//ZEB1//BHLHE41//BHLHE40//CLOCK//CEBPB//GSC//CC2D1B//ELK3//EN1//EP300//ETS1//FLI1//NR5A2//AGO1//GLI3//H2AFZ//IRF1//AR//ISL1//MEF2A//MEOX2//MYB//MYBL1//MYCN//NFIA//NFIB//NFIC//NHLH2//NR4A2//OTX2//PAX5//KLF13//POU3F2//FOXJ2//RFX7//SMARCD2//TFAP2A//TFE3//TGIF1//KLF10//TXK//ZNF217//E2F8//FOSL1//KLF7//FUBP3//FOXP2// |
| GO:0000989 | transcription factor activity, transcription factor binding | Molecular function | 66 | 643 | 906 | 17548 | 1.98807329055648 | 7.21893190644885e-08 | 6.23715716717181e-06 | 7.14152705470875 | 0.0728476821192053 | BDP1//GTF2E1//PPARG//RORA//TCERG1//PPARGC1B//MED19//MED9//TRERF1//MED22//ZFPM2//ISL1//MEF2A//NEUROD1//POU3F2//SMARCA2//SOX4//SIN3B//ZMYND8//RBBP8//TEAD2//MYB//MYBL1//JUN//CLOCK//CNOT7//ID2//BHLHE41//BHLHE40//ZMYND11//NRG1//SS18//BTG1//SAP30L//CBFB//NMI//ZBTB18//EP300//GTF2A1//NFE2//NPAT//POU4F1//RAP2C//RB1//PRDM16//SMARCD2//ZEB1//TFAP2A//ARID5B//KLF7//TOB1//KLF12//DNMT3B//ELK3//JAZF1//ATF5//SIRT1//HSBP1//HSBP1L1//CCND1//SRSF2//TGIF1//TBL1XR1//E2F8//NEUROG1//TP63// |
| GO:0000988 | transcription factor activity, protein binding | Molecular function | 66 | 648 | 906 | 17548 | 1.97273321887008 | 9.6576218014654e-08 | 7.82267365918697e-06 | 7.01512980584732 | 0.0728476821192053 | NEUROG1//TP63//BDP1//GTF2E1//PPARG//RORA//TCERG1//PPARGC1B//MED19//MED9//TRERF1//MED22//ZFPM2//ISL1//MEF2A//NEUROD1//POU3F2//SMARCA2//SOX4//SIN3B//ZMYND8//RBBP8//TEAD2//MYB//MYBL1//JUN//CLOCK//CNOT7//ID2//BHLHE41//BHLHE40//ZMYND11//NRG1//SS18//BTG1//SAP30L//CBFB//NMI//ZBTB18//EP300//GTF2A1//NFE2//NPAT//POU4F1//RAP2C//RB1//PRDM16//SMARCD2//ZEB1//TFAP2A//ARID5B//KLF7//TOB1//KLF12//DNMT3B//ELK3//JAZF1//ATF5//SIRT1//HSBP1//HSBP1L1//CCND1//SRSF2//TGIF1//TBL1XR1//E2F8// |
| GO:0043565 | sequence-specific DNA binding | Molecular function | 99 | 1139 | 906 | 17548 | 1.68349138607702 | 1.83878032043183e-07 | 1.40179958545862e-05 | 6.73547015293869 | 0.109271523178808 | BAHD1//NR5A2//MEF2A//NFE2//PPARG//RORA//SOX4//SUV39H1//TFAP2A//CEBPB//ZMYND11//CREBRF//ETV5//ATF5//FOXF1//FOXF2//ZMYND8//GLI3//PURG//IRF2//JARID2//MYOG//NHLH2//NR4A2//KLF3//ZBTB4//PURA//ARID5B//RUNX3//GSC//CC2D1B//ELK3//EN1//EP300//ESR1//ETS1//FLI1//AGO1//H2AFZ//IRF1//AR//ISL1//JUN//MEOX2//MYB//MYBL1//MYCN//NFIA//NFIB//NFIC//OTX2//PAX5//KLF13//POU3F2//FOXJ2//RFX7//SMARCD2//TFE3//TGIF1//KLF10//TXK//ZNF217//BHLHE41//E2F8//FOSL1//KLF7//FUBP3//FOXP2//CLOCK//GADD45A//ARX//POU4F1//ZNF516//SIRT1//SARS//SLC2A4RG//NEUROD1//NEUROG1//ZEB1//BHLHE40//ZBTB18//EMX2//FOXP1//GTF2E1//HIC1//HIVEP2//FOXA1//HOXA3//HOXD1//MAF//NR3C2//PKNOX1//ZNF532//HIVEP3//PRDM16//TSN//WT1//TEAD2//TP63// |
| GO:0140110 | transcription regulator activity | Molecular function | 146 | 1921 | 906 | 17548 | 1.47205799040005 | 7.9458826884635e-07 | 5.72103553569372e-05 | 6.09985785107641 | 0.161147902869757 | TSHZ1//ZBTB18//CEBPB//KLF12//CREBL2//GSC//CREBRF//ARX//E2F5//CC2D1B//ELK3//EMX2//EN1//ESR1//ETS1//ETV5//JAZF1//ATF5//FOXF1//FOXF2//MYT1L//FLI1//ADNP//ZFPM2//NR5A2//HBP1//FOXP1//GLI3//HIC1//HIVEP2//FOXA1//HOXA3//HOXD1//ID2//IRF1//IRF2//AR//ISL1//JUN//BOLA3//MAF//MEF2A//MEOX2//NR3C2//MYB//MYBL1//MYCN//MYOG//NEUROD1//NEUROG1//NFIA//NFE2//NFIB//NFIC//NHLH2//NPAS2//NR4A2//OTX2//PAX5//KLF13//PKNOX1//POU3F2//POU4F1//PPARG//TRERF1//FOXJ2//ZBTB4//MIER1//HIVEP3//RORA//RFX7//SOX4//SOX5//ZEB1//TFAP2A//TFE3//TGIF1//KLF10//WT1//ZNF3//ZNF708//ZNF131//ZSCAN5A//BHLHE41//E2F8//FOSL1//LCOR//TEAD2//BHLHE40//TP63//RUNX3//CBFB//FOXP2//CLOCK//ST18//BDP1//GTF2E1//SIRT1//TXK//KLF7//FUBP3//RB1//ZNF217//TCERG1//PPARGC1B//MED19//MED9//MED22//SMARCA2//SIN3B//ZMYND8//RBBP8//CNOT7//PURG//JARID2//PURA//ARID5B//EP300//ZNF367//ZFP30//AFF1//KLF3//ZNF532//SLC2A4RG//PRDM16//ZNF227//ZNF655//BTAF1//ZNF516//ZMYND11//NRG1//SS18//BTG1//SAP30L//NMI//GTF2A1//NPAT//RAP2C//SMARCD2//TOB1//DNMT3B//HSBP1//HSBP1L1//CCND1//SRSF2//TBL1XR1// |
| GO:0001076 | transcription factor activity, RNA polymerase II transcription factor binding | Molecular function | 27 | 191 | 906 | 17548 | 2.7379771852571 | 1.88745965119239e-06 | 0.000128744616207649 | 5.72412232364675 | 0.0298013245033113 | TCERG1//PPARGC1B//MED19//MED9//TRERF1//MED22//ZFPM2//ISL1//MEF2A//NEUROD1//POU3F2//SMARCA2//SOX4//SIN3B//ZMYND8//RBBP8//MYB//MYBL1//JUN//CLOCK//CNOT7//ID2//BHLHE41//BHLHE40//GTF2E1//PPARG//RORA// |
| GO:0003712 | transcription cofactor activity | Molecular function | 56 | 566 | 906 | 17548 | 1.91633320072699 | 2.28879531477817e-06 | 0.000148313936397625 | 5.64039304421366 | 0.0618101545253863 | TCERG1//PPARGC1B//MED19//MED9//TRERF1//MED22//ZFPM2//ISL1//MEF2A//NEUROD1//POU3F2//SMARCA2//SOX4//SIN3B//ZMYND8//RBBP8//ZBTB18//EP300//GTF2A1//JUN//NFE2//NPAT//POU4F1//RAP2C//RB1//PRDM16//SMARCD2//ZEB1//TFAP2A//ARID5B//KLF7//CBFB//TOB1//ZMYND11//KLF12//DNMT3B//ELK3//JAZF1//ATF5//SIRT1//HSBP1//HSBP1L1//CCND1//SRSF2//TGIF1//BHLHE41//TBL1XR1//E2F8//BHLHE40//PPARG//RORA//SS18//NRG1//BTG1//SAP30L//NMI// |
| GO:0003700 | DNA binding transcription factor activity | Molecular function | 118 | 1551 | 906 | 17548 | 1.47356615328998 | 1.0192202596006e-05 | 0.000629004503067799 | 4.9917319522131 | 0.130242825607064 | TSHZ1//ZBTB18//CEBPB//KLF12//CREBL2//GSC//CREBRF//ARX//E2F5//CC2D1B//ELK3//EMX2//EN1//ESR1//ETS1//ETV5//JAZF1//ATF5//FOXF1//FOXF2//MYT1L//FLI1//ADNP//ZFPM2//NR5A2//HBP1//FOXP1//GLI3//HIC1//HIVEP2//FOXA1//HOXA3//HOXD1//ID2//IRF1//IRF2//AR//ISL1//JUN//BOLA3//MAF//MEF2A//MEOX2//NR3C2//MYB//MYBL1//MYCN//MYOG//NEUROD1//NEUROG1//NFIA//NFE2//NFIB//NFIC//NHLH2//NPAS2//NR4A2//OTX2//PAX5//KLF13//PKNOX1//POU3F2//POU4F1//PPARG//TRERF1//FOXJ2//ZBTB4//MIER1//HIVEP3//RORA//RFX7//SOX4//SOX5//ZEB1//TFAP2A//TFE3//TGIF1//KLF10//WT1//ZNF3//ZNF708//ZNF131//ZSCAN5A//BHLHE41//E2F8//FOSL1//LCOR//TEAD2//BHLHE40//TP63//RUNX3//CBFB//FOXP2//CLOCK//ST18//SIRT1//TXK//KLF7//FUBP3//RB1//ZNF217//PURG//JARID2//PURA//ARID5B//EP300//ZNF367//ZFP30//CNOT7//AFF1//KLF3//ZNF532//SLC2A4RG//PRDM16//ZNF227//ZNF655//BTAF1//ZNF516// |
| GO:0030371 | translation repressor activity | Molecular function | 8 | 25 | 906 | 17548 | 6.19796909492274 | 2.41113140784306e-05 | 0.00142037559298391 | 4.61777911971548 | 0.00883002207505519 | CELF1//CPEB2//CPEB3//PURA//CPEB1//FMR1//NANOS1//PAIP2// |
| GO:0019003 | GDP binding | Molecular function | 12 | 59 | 906 | 17548 | 3.93938713660343 | 3.9452802886546e-05 | 0.00222307967569407 | 4.40392213732353 | 0.0132450331125828 | RAB12//RAB18//RAB21//TRIM23//RHOB//RAB9B//RAB8B//MIEF1//RAP2C//RRAGD//RAP1B//RAB28// |
| GO:0019904 | protein domain specific binding | Molecular function | 60 | 698 | 906 | 17548 | 1.66492722822065 | 7.30716714563167e-05 | 0.00380179408000545 | 4.1362509584006 | 0.0662251655629139 | SIRPA//EPS15//CD2AP//GJA1//ARHGAP1//ARHGAP6//ERRFI1//ENAH//SH3BGRL//CCDC6//ADAM12//SYNGAP1//CD3E//QKI//ADRB1//DTNA//LPAR1//GRIK2//PTEN//FZD3//CXXC4//RAPGEF2//RAB8B//TRAK2//SHCBP1//SIRT1//UBE2I//PPARGC1B//PPARG//NFE2//NDFIP2//PMEPA1//TP63//DAZAP2//MAVS//STRN//LBR//WT1//AR//RNF2//HOXA3//JUN//CCT6A//RB1//CALM2//TEAD2//DDX6//ZNF521//ARFIP1//FOXA1//NCK1//ARHGEF4//HPCAL4//PPP3R1//PRKAR2B//SKP1//WNT3//YWHAG//CASP2//BHLHE40// |
| GO:0001104 | RNA polymerase II transcription cofactor activity | Molecular function | 16 | 103 | 906 | 17548 | 3.00872286161298 | 7.33370771605989e-05 | 0.00380179408000545 | 4.13467640277899 | 0.0176600441501104 | ZFPM2//ISL1//MEF2A//NEUROD1//POU3F2//SMARCA2//SOX4//TCERG1//SIN3B//ZMYND8//RBBP8//PPARGC1B//MED19//MED9//TRERF1//MED22// |
| GO:0099106 | ion channel regulator activity | Molecular function | 15 | 95 | 906 | 17548 | 3.05820843499477 | 0.000101012340237089 | 0.00502268410841227 | 3.99562556711341 | 0.0165562913907285 | C8ORF44-SGK3//SGK2//SGK3//GRM3//HPCAL4//STIM2//SGK1//ARPP19//FLNA//KCNE1//KCNIP4//KCNAB2//CALM2//RASA1//SCN1B// |
| GO:0003677 | DNA binding | Molecular function | 170 | 2520 | 906 | 17548 | 1.30661550860226 | 0.000104639252258589 | 0.00502268410841227 | 3.98030537243274 | 0.187637969094923 | HMGB3//BAHD1//NR5A2//MEF2A//NFE2//PPARG//RORA//SOX4//SUV39H1//TFAP2A//CEBPB//ZMYND11//CREBRF//ETV5//ATF5//FOXF1//FOXF2//ZMYND8//GLI3//PURG//IRF2//JARID2//MYOG//NHLH2//NR4A2//KLF3//ZBTB4//PURA//ARID5B//RUNX3//GSC//CC2D1B//ELK3//EN1//EP300//ESR1//ETS1//FLI1//AGO1//H2AFZ//IRF1//AR//ISL1//JUN//MEOX2//MYB//MYBL1//MYCN//NFIA//NFIB//NFIC//OTX2//PAX5//KLF13//POU3F2//FOXJ2//RFX7//SMARCD2//TFE3//TGIF1//KLF10//TXK//ZNF217//BHLHE41//E2F8//FOSL1//KLF7//FUBP3//FOXP2//CLOCK//GADD45A//ARX//POU4F1//ZNF516//RB1//SIRT1//SARS//NPAS2//ST18//SLC2A4RG//OGG1//RBBP8//TP63//AFF1//RBMS1//NABP1//TSN//TRERF1//WT1//LEMD3//ZBTB18//EMX2//FOXP1//GTF2E1//HIC1//HIVEP2//FOXA1//HOXA3//HOXD1//MAF//NR3C2//NEUROD1//PKNOX1//ZNF532//HIVEP3//PRDM16//TEAD2//ARID4B//SMARCA2//TNF//TBL1XR1//SAP30L//NEUROG1//ZEB1//BHLHE40//DNAJB6//TSHZ1//KLF12//ZNF526//CR2//CREBL2//RBFOX3//ZNF800//POLR3H//DNMT3B//E2F5//ZNF367//ARID2//ZFP30//KDM2A//MYT1L//ADNP//ZFPM2//SMUG1//ZNF521//HBP1//RNF11//ZBTB11//GTF2A1//AGFG1//ZNF680//IMPDH1//ZNF662//LBR//PCBP2//ZFAND6//BNC2//BDP1//MAPK1//PHTF2//ZBTB26//MIER1//ATXN1//SOS2//SOX5//TNFAIP3//ZNF3//ZNF708//ZNF131//ZNF227//CSDE1//ZNF655//ZSCAN5A//CXXC4//HIST2H2BE//LCOR//ZIC5//CBFB//DDX3Y//BTAF1// |
| GO:0001085 | RNA polymerase II transcription factor binding | Molecular function | 19 | 141 | 906 | 17548 | 2.6099603901492 | 0.000117903045931922 | 0.00532119841662737 | 3.9284749751056 | 0.0209713024282561 | ESR1//GTF2E1//EP300//ISL1//JUN//MEF2A//NEUROD1//NHLH2//RB1//BHLHE41//BHLHE40//TCERG1//GSC//GTF2A1//RBBP8//ZFPM2//ID2//AR//TEAD2// |
| GO:0008134 | transcription factor binding | Molecular function | 54 | 620 | 906 | 17548 | 1.68694723349712 | 0.000119070026297989 | 0.00532119841662737 | 3.92419755045422 | 0.0596026490066225 | GSC//ZFPM2//ID2//AR//MEF2A//TEAD2//ESR1//GTF2E1//EP300//ISL1//JUN//NEUROD1//NHLH2//RB1//BHLHE41//BHLHE40//TCERG1//GTF2A1//RBBP8//BDP1//FAM89B//RORA//CCNT2//PPARGC1B//PPARG//STRN//GABARAPL1//DLL1//MDFIC//PRDM16//ZNF516//TACC2//SIRT1//CEBPB//NR4A2//FOXP1//FOXP2//ZMYND8//E2F5//ETS1//MED19//FOXF2//FLNA//CNOT7//FOXA1//JARID2//NFIA//INSIG2//TRERF1//MAPK1//PURA//CCND1//ZEB1//UBE2I// |
| GO:0001047 | core promoter binding | Molecular function | 19 | 142 | 906 | 17548 | 2.59158038740167 | 0.000129666909626206 | 0.0056016104958521 | 3.88717083964674 | 0.0209713024282561 | CEBPB//GADD45A//H2AFZ//TFAP2A//ESR1//SIRT1//PPARG//RORA//SARS//SOX4//KLF10//CLOCK//EP300//AGO1//MYOG//NPAS2//RB1//E2F8//ST18// |
| GO:0015459 | potassium channel regulator activity | Molecular function | 10 | 49 | 906 | 17548 | 3.95278641257828 | 0.000167439267159463 | 0.00700004162060207 | 3.77614268546546 | 0.011037527593819 | RASA1//C8ORF44-SGK3//SGK2//ARPP19//FLNA//SGK3//KCNE1//SGK1//KCNIP4//KCNAB2// |
| GO:0003682 | chromatin binding | Molecular function | 48 | 540 | 906 | 17548 | 1.72165808192298 | 0.000175064167571034 | 0.00709009878662688 | 3.75680273694725 | 0.0529801324503311 | EP300//LEMD3//H2AFZ//MYOG//CLOCK//SAP30L//SMARCD2//ISL1//TSHZ1//CEBPB//ZMYND11//MBD6//PHF13//ARX//DNMT3B//ESR1//ATF5//BAHD1//FLI1//PHF8//SIN3B//FMR1//ADNP//ZMYND8//NR5A2//GLI3//ATAD2//GTF2H1//HMGB3//AR//JARID2//JUN//MEF2A//NEUROD1//NEUROG1//NFIA//POU4F1//PPARG//PRKAA1//RNF2//ATXN7//GMNC//SMARCA2//SUV39H1//ZEB1//TFAP2A//TP63//CCNT2// |
| GO:0003705 | transcription factor activity, RNA polymerase II distal enhancer sequence-specific binding | Molecular function | 15 | 101 | 906 | 17548 | 2.87653268638122 | 0.000204141803626646 | 0.00801720537879192 | 3.69006805248445 | 0.0165562913907285 | MEF2A//MEOX2//POU4F1//ARX//CEBPB//FOXF1//FOXF2//NR5A2//JUN//MYOG//PKNOX1//PURA//BHLHE41//TEAD2//BHLHE40// |
| GO:0005488 | binding | Molecular function | 810 | 15006 | 906 | 17548 | 1.04548908913256 | 0.00024179483439392 | 0.00921665015807413 | 3.61655298146606 | 0.894039735099338 | SEPSECS//THG1L//ACBD3//ACBD5//STX6//VTI1A//VAMP1//SYT1//VAMP3//HPRT1//DHRS3//PEX5L//ALPI//IRAK4//PRKACB//PTEN//RPS6KA5//HMGB3//BAG5//CELF1//CPEB2//CPEB3//PURA//CPEB1//BAHD1//NR5A2//MEF2A//NFE2//PPARG//RORA//SOX4//SUV39H1//TFAP2A//CEBPB//ZMYND11//CREBRF//ETV5//ATF5//FOXF1//FOXF2//ZMYND8//GLI3//PURG//IRF2//JARID2//MYOG//NHLH2//NR4A2//KLF3//ZBTB4//ARID5B//RUNX3//GSC//CC2D1B//ELK3//EN1//EP300//ESR1//ETS1//FLI1//AGO1//H2AFZ//IRF1//AR//ISL1//JUN//MEOX2//MYB//MYBL1//MYCN//NFIA//NFIB//NFIC//OTX2//PAX5//KLF13//POU3F2//FOXJ2//RFX7//SMARCD2//TFE3//TGIF1//KLF10//TXK//ZNF217//BHLHE41//E2F8//FOSL1//KLF7//FUBP3//FOXP2//CLOCK//GADD45A//ARX//POU4F1//ZNF516//GTF2E1//RB1//SIRT1//SARS//NPAS2//ST18//MBNL1//ZFPM2//ID2//TEAD2//NEUROD1//BHLHE40//TCERG1//GTF2A1//RBBP8//BDP1//SLC2A4RG//FAM89B//CCNT2//LDLR//S1PR1//FLNA//GNAQ//CRKL//MAPK1//RASA1//YES1//SOCS3//SYT6//CR2//C5AR1//LPAR1//IGFBP3//F3//SERPINB13//TNF//TNFAIP3//ZMAT3//TP63//FMR1//FXR1//ADAMTS17//JAZF1//DZIP1//ZFR2//TBK1//IMPDH1//TRIM23//KCMF1//DNAJB6//TSHZ1//ZBTB18//KLF12//ZNF526//CREBL2//RBFOX3//ZNF800//POLR3H//DNMT3B//E2F5//ZNF367//ARID2//ZFP30//KDM2A//MYT1L//ADNP//SMUG1//ZNF521//HBP1//RNF11//ZBTB11//HIVEP2//FOXA1//AGFG1//ZNF680//ZNF662//LBR//NEUROG1//PCBP2//ZFAND6//BNC2//ZNF532//PHTF2//ZBTB26//MIER1//ATXN1//SOS2//SOX5//TSN//ZNF3//ZNF708//ZNF131//ZNF227//CSDE1//ZNF655//ZSCAN5A//SAP30L//CXXC4//HIST2H2BE//LCOR//ZIC5//CBFB//DDX3Y//BTAF1//MBD6//PHF13//PHF8//SIN3B//ATAD2//GTF2H1//PRKAA1//RNF2//ATXN7//GMNC//SMARCA2//ZEB1//OGG1//AFF1//RBMS1//NABP1//MBNL2//KIF1C//FASTK//CKAP4//RPP14//RCAN3//STRAP//PPARGC1B//DDX6//ELAVL2//PATL1//TNRC6B//MORC3//PAN3//TES//GRSF1//CNOT7//ANXA7//NANOS1//NOVA1//ASCC1//YTHDF2//RPP25//NXF2//CNOT6//RBM25//SRSF2//SRSF6//BTF3//TIA1//TNS1//NXF2B//UBE2I//WT1//YWHAG//ZYX//SLBP//SNIP1//ANP32A//CAST//MEX3B//RTCA//CCT6A//QKI//GTPBP1//SECISBP2L//G3BP2//MSN//PAIP2//BOLL//IQGAP2//WASF3//CNN3//DBN1//EPS8//LIMCH1//PALLD//FLII//FKBP15//KLHL3//KLHL20//TMOD3//PFN2//PXK//ENAH//TMOD1//TNNI1//TOB1//ARHGAP1//LASP1//ARHGAP6//NCK1//SH3BGRL//SKAP2//MARCKS//RAPGEF4//RAB3GAP1//ARHGEF4//DOCK10//ITSN1//RALGPS1//IQSEC2//FBXO8//PSD//RANBP9//ADRB1//EREG//RASGEF1A//NRG1//IL2//MET//RAPGEF2//PLEKHG4B//ARPP19//TMED1//NXPH3//C1QTNF2//ADCYAP1//CNTFR//EFNA1//GJA1//NSG1//ITGB8//LRP6//NTF3//RAB8B//CYTL1//CCL2//BMP3//TRAK2//WNT7A//BAG6//SNX17//NPTN//BAMBI//WNT3//WNT10A//DLL1//EDN1//TXLNA//IFNA1//IFNG//INHBB//CXCL9//GDF5//SNX2//SOCS5//IGF1//SOCS1//SIVA1//CD2AP//SPRED1//NPNT//KDR//EDN2//PTHLH//SNAP25//NR3C2//ADH4//EGLN3//EGLN1//MSMO1//CUTC//FAT3//CABP7//EPS15//SULF1//CDH20//MICU3//S100A7A//HPCAL4//PPP3R1//PPP3R2//PCDHB10//FSTL5//SLC24A3//PCDH10//STIM2//SMOC1//SMOC2//DLK2//BNIP2//SPOCK1//TGM3//LRP8//KCNIP4//CALM2//CALU//CALN1//MCFD2//SLIT2//CD69//C2CD5//C8ORF44-SGK3//PYURF//GPHN//DNAJA2//RTN3//SEC23A//DCTN6//PTGES3//CFL2//RAI2//SEPT9//COPS8//RALBP1//PNRC1//STMN2//PTPN21//AKAP11//RASSF8//CHRNB4//SORCS1//C1QTNF6//CLCN5//RHEBL1//NEDD1//SPPL3//SLAIN1//FRMD6//EDARADD//LYPD6//RAET1E//VPS37A//UBXN2B//CRHR1//DYNLL2//CACUL1//FAM76B//KRT80//GSG1L//APCDD1//PDIK1L//PPP4R2//SH3D19//ADSS//C12ORF50//MARCH10//SPTSSB//DCP2//LCA5//SPTSSA//DPYSL2//DTNA//DUSP1//EDNRB//EFNB2//RAB12//EMX2//SMIM14//MED19//UNC5B//CCNY//ATXN7L1//HS3ST5//TRAPPC8//WDR47//RAB18//ELL2//WDFY3//RAB21//MYCBP2//DCUN1D4//RGL1//FAM168A//ARL6IP1//FBXO28//OTUD3//CAMSAP2//TTC33//LEMD3//KPNA6//SGK3//GABARAPL1//CNOT6L//RICTOR//LCLAT1//FBXO33//NAALADL2//CNEP1R1//NUTM1//DNAJB5//ARMC8//CLIC4//RWDD3//RNF167//PPP1R16B//APPL1//TIMM9//FOXP1//SNX5//SERP1//BBS9//SESN1//GLRB//GPM6A//ZDHHC22//YIPF6//GRK6//ASTE1//C16ORF72//MYLIP//SLC25A6//GSTA2//GSTM3//ST8SIA5//NRBP1//LRP12//MDFIC//NRBF2//TNPO2//HIC1//HOXA3//HSBP1//SPOPL//KCNA4//NHLRC2//KCNB1//KCNE1//KCNJ3//KCNJ10//RHOB//RND3//SNX19//FAM19A1//MAF//DNAJB9//MDM4//MEP1A//MGST1//MID1//MLLT6//ASNA1//KRTAP5-6//MZT1//MTRR//PPP1R12A//NEU1//NPAT//ROR1//OGN//DCAF8//MEMO1//PHF20L1//INSIG2//RAB9B//IER5//CDK17//FAM53C//TNFRSF12A//PRR16//ZMYND10//COMMD10//GSKIP//CHMP3//RASD1//ARID4B//PIGA//PIK3CA//PIK3CB//PKNOX1//NUP54//PLP2//PMAIP1//ERRFI1//ANKIB1//MIEF1//NDFIP2//SEMA4C//C2ORF42//ATG16L1//MED9//ANO1//PPP2CA//C19ORF73//PPP2CB//UBE2W//RBM41//PPP2R5E//FBXW7//DRAM1//KLK15//SYBU//INTS8//PRKAR2B//LRP2BP//MAPK6//NPDC1//NRIP3//PSMA2//SPHK2//PAK6//PMEPA1//CCNL1//TMEM159//SMURF1//PSMD12//MAVS//HECW2//KLHL42//TAOK1//ARRDC3//RANBP10//USP28//PTPRG//MID1IP1//RRAGD//RAF1//RAP1A//RAP1B//CCND1//BCL2A1//ELOVL5//ROBO2//CLIP1//RTN1//SLC22A23//SCN1B//CCL21//SDC1//KIF13A//PRDM16//MCCC2//SGK1//GIGYF1//SKP1//CDK15//BMPR2//MTMR9//SS18//HSPA13//STRN//MED22//BTG1//TMBIM6//TGFBR2//TRPC3//TSC1//TTK//UBE2V1//VBP1//VEGFB//MPPED2//PTP4A1//APOO//CENPO//FAM118B//PLEKHF2//TBL1XR1//CLIP4//FZD3//SHCBP1//LONRF3//CBLL1//UBA5//PRR5L//ELOVL7//C16ORF70//CCDC6//ADAM12//CUL5//PTP4A2//SNX27//FAM83D//SBF2//FZD6//CASP2//CCM2//KATNAL1//HDHD2//PPP1R1B//PCGF5//RECK//DCTN5//KBTBD8//TMEM185A//ACCS//PPP1R15B//FAM104A//PPFIA1//KRTAP2-4//ALG2//CDC14A//PDE5A//AP1S2//CCND2//ACVR1//C5ORF30//GPRC5A//DIRAS3//NMI//SESTD1//BTF3L4//CD3E//LONRF1//WDR20//DLG5//NOG//CD8A//MOB1B//FAM114A1//NREP//SYCE1//FBXO44//TJAP1//PLAA//HOMER1//VPS4B//CEP41//FEZ2//PPM1F//PHF14//ULK2//SLK//RASSF2//DAZAP2//GIT2//ADCY3//CAMSAP1//TMEM201//SGK2//HIPK3//CLCN6//DGKH//DYNC1LI2//ACSL1//ACSL4//ABCB7//CDK19//ATP2C1//CMPK1//RIMKLB//STK33//DGKE//NAV3//IFI44L//ARL5A//RAB30//ARL15//RAP2C//RAB28//HAPLN1//SUSD5//ARFIP1//PHLDA3//KCNJ2//ARAP2//CCL8//CNGA3//TRERF1//CHRM2//PRELP//CPD//RNF145//AMZ1//PRICKLE2//PDZRN3//FBXO30//NKTR//GLRA3//DENND1B//DENND6A//RIN2//SIRPA//SYNGAP1//RIMS4//TBC1D12//FRZB//NPEPL1//GRIK2//EMC7//AFTPH//JKAMP//WDR45B//RIC3//TUB//TACC2//SLC6A11//NLN//THOP1//BRI3//PON2//ADIPOR2//FIBIN//NAGA//GNPTG//NMUR1//SEC61A2//HOXD1//HIVEP3//CRTAP//MB21D2//RNF38//ESCO2//PRUNE2//PPTC7//ZNRF2//STEAP2//YPEL2//ENPP5//SLC6A1//BSN//LMLN//NRXN3//GLRX//S100PBP//GABRA5//SAMD14//WDR1//PALM2//LDLRAD4//TEX261//GFPT1//GPCPD1// |
| GO:0045182 | translation regulator activity | Molecular function | 10 | 53 | 906 | 17548 | 3.65446290974218 | 0.000329552674330283 | 0.0120097754452339 | 3.48207515969617 | 0.011037527593819 | CELF1//CPEB2//CPEB3//PURA//CPEB1//BOLL//FMR1//NANOS1//PAIP2//FXR1// |
| GO:0033613 | activating transcription factor binding | Molecular function | 12 | 73 | 906 | 17548 | 3.18388823369319 | 0.00033360487347872 | 0.0120097754452339 | 3.47676761358149 | 0.0132450331125828 | EP300//ISL1//JUN//MEF2A//NEUROD1//NHLH2//RB1//BHLHE41//BHLHE40//PPARG//PRDM16//ZNF516// |
| GO:0046332 | SMAD binding | Molecular function | 12 | 74 | 906 | 17548 | 3.14086271702166 | 0.000379785751967951 | 0.0133027657986612 | 3.42046133227348 | 0.0132450331125828 | TGIF1//SMURF1//JUN//PMEPA1//LDLRAD4//TOB1//FLNA//MEF2A//PURA//PRDM16//TGFBR2//ACVR1// |
| GO:0005246 | calcium channel regulator activity | Molecular function | 8 | 36 | 906 | 17548 | 4.30414520480746 | 0.000406935233032552 | 0.0137270341443597 | 3.39047470668349 | 0.00883002207505519 | CALM2//C8ORF44-SGK3//SGK2//SGK3//GRM3//HPCAL4//STIM2//SGK1// |
| GO:0003714 | transcription corepressor activity | Molecular function | 25 | 232 | 906 | 17548 | 2.0871393773312 | 0.000413082046010825 | 0.0137270341443597 | 3.38396368056898 | 0.0275938189845475 | TCERG1//SIN3B//ZMYND8//RBBP8//TOB1//ZMYND11//KLF12//DNMT3B//ELK3//JAZF1//ATF5//SIRT1//ZFPM2//HSBP1//HSBP1L1//NPAT//POU4F1//CCND1//SRSF2//ZEB1//TGIF1//BHLHE41//TBL1XR1//E2F8//BHLHE40// |
| GO:0098772 | molecular function regulator | Molecular function | 127 | 1848 | 906 | 17548 | 1.33107087908412 | 0.00043431170156043 | 0.0140716991305579 | 3.36219846938365 | 0.140176600441501 | BAG5//DNAJB6//DNAJA2//PPP1R12A//NCK1//GSKIP//PPP1R1B//SOCS1//SOCS3//SOCS5//PRKAR2B//ARPP19//CAST//RECK//SERPINI1//SERPINB13//SPOCK1//RAPGEF4//RAB3GAP1//ARHGEF4//DOCK10//ITSN1//SOS2//RALGPS1//IQSEC2//FBXO8//PSD//RANBP9//ADRB1//EREG//RASGEF1A//NRG1//IL2//MET//RAPGEF2//PLEKHG4B//IQGAP2//CPEB2//SLIT2//RALBP1//ARAP2//TBC1D12//ELMOD2//APPL1//GNAQ//AGFG1//JUN//ARHGAP1//ARHGAP6//ERRFI1//RIN2//RASA1//BNIP2//SYDE2//SYNGAP1//ARHGAP12//GIT2//EDN1//TXLNA//IFNA1//IFNG//INHBB//CXCL9//BMP3//TNF//WNT7A//GDF5//EDN2//IGF1//PTHLH//ADCYAP1//C8ORF44-SGK3//SGK2//SGK3//GRM3//HPCAL4//STIM2//SGK1//CCL2//CCL8//CCL21//RICTOR//TRIM23//NTF3//OGN//VEGFB//IGFBP3//RGL1//YWHAG//RCAN3//RAF1//FLNA//KCNE1//KCNIP4//KCNAB2//PLAA//CCNY//CCNL1//CCND1//CCNT2//RAP1A//SCN1B//DENND1B//DENND6A//TRAPPC8//SBF2//MOB1B//LRP6//CALM2//UBXN2B//PPP4R2//PPP1R16B//PPP2R5E//PPP1R15B//MTMR9//ESR1//SPRED1//PIK3CA//PI15//LYPD6//EPS8//TAOK1//SEMA4C//WNT3//WNT10A//TMBIM6//FBXW7// |
| GO:0000900 | translation repressor activity, mRNA regulatory element binding | Molecular function | 5 | 14 | 906 | 17548 | 6.91737622201198 | 0.000491266378596933 | 0.0155288104063811 | 3.30868295719526 | 0.00551876379690949 | CELF1//CPEB2//CPEB3//PURA//CPEB1// |
| GO:0005343 | organic acid:sodium symporter activity | Molecular function | 7 | 30 | 906 | 17548 | 4.51935246504783 | 0.000683885278355203 | 0.0211027457321034 | 3.16501674499912 | 0.00772626931567329 | SLC6A15//SLC6A8//SLC6A1//SLC6A11//SLC6A6//SLC10A2//SLC23A2// |
| GO:0001102 | RNA polymerase II activating transcription factor binding | Molecular function | 9 | 49 | 906 | 17548 | 3.55750777132045 | 0.000797477661709446 | 0.02403560580408 | 3.09828147320143 | 0.00993377483443709 | EP300//ISL1//JUN//MEF2A//NEUROD1//NHLH2//RB1//BHLHE41//BHLHE40// |
| GO:0050699 | WW domain binding | Molecular function | 7 | 31 | 906 | 17548 | 4.37356690165919 | 0.000844436529362784 | 0.0248724941375947 | 3.07343298787322 | 0.00772626931567329 | NFE2//NDFIP2//ENAH//PMEPA1//TP63//RAPGEF2//DAZAP2// |
| GO:0001046 | core promoter sequence-specific DNA binding | Molecular function | 12 | 87 | 906 | 17548 | 2.67153840298394 | 0.00166313678447023 | 0.0478983393927426 | 2.77907203081795 | 0.0132450331125828 | CEBPB//GADD45A//H2AFZ//TFAP2A//ESR1//SIRT1//PPARG//RORA//SARS//SOX4//KLF10//CLOCK// |
| GO:0003713 | transcription coactivator activity | Molecular function | 29 | 317 | 906 | 17548 | 1.77189573888761 | 0.00208503509792838 | 0.0587435975416344 | 2.68088663003875 | 0.0320088300220751 | ZFPM2//ISL1//MEF2A//NEUROD1//POU3F2//SMARCA2//SOX4//PPARGC1B//PPARG//TRERF1//RORA//SS18//ZBTB18//TCERG1//EP300//GTF2A1//JUN//NFE2//NPAT//POU4F1//RAP2C//RB1//PRDM16//SMARCD2//ZEB1//TFAP2A//ARID5B//KLF7//CBFB// |
| GO:0090079 | translation regulator activity, nucleic acid binding | Molecular function | 6 | 27 | 906 | 17548 | 4.30414520480746 | 0.00216651240185065 | 0.0597404270808179 | 2.66423882057381 | 0.00662251655629139 | CELF1//CPEB2//CPEB3//PURA//CPEB1//BOLL// |
| GO:0001227 | transcriptional repressor activity, RNA polymerase II transcription regulatory region sequence-specific DNA binding | Molecular function | 23 | 233 | 906 | 17548 | 1.91192716179215 | 0.00223164268692711 | 0.060254352547032 | 2.65137533998456 | 0.0253863134657837 | TSHZ1//CEBPB//GSC//CC2D1B//EN1//MYT1L//ZFPM2//RB1//TFAP2A//TGIF1//ZNF217//BHLHE41//E2F8//BHLHE40//FOXP2//ARX//PURG//HIC1//JARID2//ZBTB4//PURA//ZEB1//ARID5B// |
| GO:0005070 | SH3/SH2 adaptor activity | Molecular function | 9 | 57 | 906 | 17548 | 3.05820843499477 | 0.00241646794592735 | 0.0635475604268613 | 2.61681896133586 | 0.00993377483443709 | TOB1//CRKL//EPS8//ARHGAP1//LASP1//ARHGAP6//NCK1//SH3BGRL//SKAP2// |
| GO:0019208 | phosphatase regulator activity | Molecular function | 12 | 91 | 906 | 17548 | 2.55410814351212 | 0.00245168057202397 | 0.0635475604268613 | 2.61053611443267 | 0.0132450331125828 | ARPP19//PPP1R1B//IGFBP3//RCAN3//UBXN2B//PPP4R2//PPP1R16B//PPP2R5E//PPP1R15B//CALM2//PPP1R12A//SBF2// |
| GO:0070888 | E-box binding | Molecular function | 7 | 37 | 906 | 17548 | 3.66433983652527 | 0.00252520446839734 | 0.0641699017851559 | 2.59770345085166 | 0.00772626931567329 | MYOG//NEUROD1//NEUROG1//ZEB1//BHLHE41//BHLHE40//CLOCK// |
| GO:0035091 | phosphatidylinositol binding | Molecular function | 23 | 237 | 906 | 17548 | 1.8796583489349 | 0.00277252042087339 | 0.0690997397202291 | 2.55712524639072 | 0.0253863134657837 | WDFY3//SYT1//PHLDA3//KCNJ2//PFN2//SESTD1//IQGAP2//ARAP2//SAP30L//SNX19//WDR45B//PLEKHF2//SNX27//ARFIP1//DENND1B//C8ORF44-SGK3//SGK3//SNX5//PXK//SNX2//TUB//SBF2//SNX17// |
| GO:0004535 | poly(A)-specific ribonuclease activity | Molecular function | 4 | 13 | 906 | 17548 | 5.95958566819494 | 0.00346899826190807 | 0.0848268254232615 | 2.45979591775492 | 0.00441501103752759 | CNOT6L//PAN3//CNOT7//CNOT6// |
| GO:0001191 | transcriptional repressor activity, RNA polymerase II transcription factor binding | Molecular function | 8 | 50 | 906 | 17548 | 3.09898454746137 | 0.00381638546658824 | 0.0914948478701699 | 2.41834776668343 | 0.00883002207505519 | TCERG1//SIN3B//ZMYND8//RBBP8//CNOT7//ID2//BHLHE41//BHLHE40// |
| GO:0016247 | channel regulator activity | Molecular function | 15 | 134 | 906 | 17548 | 2.16813284570525 | 0.00388288320436678 | 0.0914948478701699 | 2.41084567266845 | 0.0165562913907285 | C8ORF44-SGK3//SGK2//SGK3//GRM3//HPCAL4//STIM2//SGK1//ARPP19//FLNA//KCNE1//KCNIP4//KCNAB2//SCN1B//CALM2//RASA1// |
| GO:0017147 | Wnt-protein binding | Molecular function | 6 | 31 | 906 | 17548 | 3.74877162999359 | 0.00452040370121002 | 0.104128930271403 | 2.34482277815791 | 0.00662251655629139 | APCDD1//FRZB//LRP6//ROR1//FZD3//FZD6// |
| GO:0042813 | Wnt-activated receptor activity | Molecular function | 5 | 22 | 906 | 17548 | 4.4019666867349 | 0.004580540030513 | 0.104128930271403 | 2.33908331709525 | 0.00551876379690949 | FRZB//LRP6//ROR1//FZD3//FZD6// |
| GO:0005041 | low-density lipoprotein particle receptor activity | Molecular function | 4 | 14 | 906 | 17548 | 5.53390097760959 | 0.00466009101523253 | 0.104128930271403 | 2.33160560111531 | 0.00441501103752759 | LRP12//LDLR//LRP6//LRP8// |
| GO:0017124 | SH3 domain binding | Molecular function | 14 | 125 | 906 | 17548 | 2.16928918322296 | 0.00515208976694169 | 0.113171327761973 | 2.28801657869409 | 0.0154525386313466 | SIRPA//EPS15//CD2AP//GJA1//ARHGAP1//ARHGAP6//ERRFI1//ENAH//SH3BGRL//CCDC6//ADAM12//SYNGAP1//CD3E//QKI// |
| GO:0019207 | kinase regulator activity | Molecular function | 21 | 222 | 906 | 17548 | 1.83216991826263 | 0.00554470054233621 | 0.119268027405321 | 2.25612190421686 | 0.0231788079470199 | NCK1//GSKIP//PPP1R1B//SOCS1//SOCS3//SOCS5//PRKAR2B//YWHAG//CCNY//CCNL1//CCND1//CCNT2//IL2//ITSN1//MOB1B//LRP6//SPRED1//PIK3CA//NRG1//TAOK1//CALM2// |
| GO:0005088 | Ras guanyl-nucleotide exchange factor activity | Molecular function | 23 | 251 | 906 | 17548 | 1.77481684740068 | 0.0056136957343554 | 0.119268027405321 | 2.25075113008268 | 0.0253863134657837 | PLEKHG4B//ARHGEF4//ITSN1//SOS2//RGL1//RALGPS1//RAP1A//RAPGEF2//DENND1B//DENND6A//TRAPPC8//RAB3GAP1//RIN2//SBF2//EPS8//RANBP9//RAPGEF4//ADRB1//EREG//RASGEF1A//NRG1//IL2//MET// |
| GO:0001190 | transcriptional activator activity, RNA polymerase II transcription factor binding | Molecular function | 9 | 65 | 906 | 17548 | 2.68181355068772 | 0.00595325404561354 | 0.122301405396259 | 2.22524558422903 | 0.00993377483443709 | ZFPM2//ISL1//MEF2A//NEUROD1//POU3F2//SMARCA2//SOX4//JUN//CLOCK// |
| GO:0035258 | steroid hormone receptor binding | Molecular function | 11 | 89 | 906 | 17548 | 2.39387851278617 | 0.00601490658799677 | 0.122301405396259 | 2.22077111290145 | 0.0121412803532009 | PPARGC1B//ESR1//ISL1//PPARG//STRN//CEBPB//NR4A2//EP300//FOXP1//RB1//FOXP2// |
| GO:0050811 | GABA receptor binding | Molecular function | 4 | 15 | 906 | 17548 | 5.16497424576895 | 0.00609804307422489 | 0.122301405396259 | 2.21480951227105 | 0.00441501103752759 | GABARAPL1//GABRA5//PPP2CA//TRAK2// |
| GO:0061630 | ubiquitin protein ligase activity | Molecular function | 21 | 224 | 906 | 17548 | 1.81581125827815 | 0.00613394394348519 | 0.122301405396259 | 2.21226019723946 | 0.0231788079470199 | CACUL1//RNF38//RNF145//ZNRF2//PDZRN3//RNF11//MYLIP//MDM4//ANKIB1//UBE2W//SMURF1//HECW2//RNF2//SKP1//UBE2V1//LONRF3//CBLL1//CUL5//FBXO30//UBE3B//LONRF1// |
| GO:0001078 | transcriptional repressor activity, RNA polymerase II proximal promoter sequence-specific DNA binding | Molecular function | 15 | 141 | 906 | 17548 | 2.06049504485463 | 0.00623626419507403 | 0.122457551466908 | 2.20507549450431 | 0.0165562913907285 | TSHZ1//CEBPB//GSC//CC2D1B//EN1//MYT1L//ZFPM2//RB1//TFAP2A//TGIF1//ZNF217//BHLHE41//E2F8//BHLHE40//FOXP2// |
| GO:0019888 | protein phosphatase regulator activity | Molecular function | 10 | 78 | 906 | 17548 | 2.48316069508123 | 0.0066723231629893 | 0.127316661153498 | 2.17572292752257 | 0.011037527593819 | ARPP19//PPP1R1B//IGFBP3//RCAN3//CALM2//UBXN2B//PPP4R2//PPP1R16B//PPP2R5E//PPP1R15B// |
| GO:0030234 | enzyme regulator activity | Molecular function | 72 | 1041 | 906 | 17548 | 1.3396186804588 | 0.00672209188731609 | 0.127316661153498 | 2.17249555525815 | 0.0794701986754967 | BAG5//DNAJB6//DNAJA2//PPP1R12A//NCK1//GSKIP//PPP1R1B//SOCS1//SOCS3//SOCS5//PRKAR2B//ARPP19//CAST//RECK//SERPINI1//SERPINB13//SPOCK1//IQGAP2//CPEB2//SLIT2//RALBP1//ARAP2//RAB3GAP1//TBC1D12//ELMOD2//APPL1//GNAQ//AGFG1//JUN//ARHGAP1//ARHGAP6//ERRFI1//RIN2//RASA1//BNIP2//SYDE2//SYNGAP1//ARHGAP12//RAPGEF2//GIT2//RICTOR//TRIM23//IGFBP3//YWHAG//RCAN3//RAF1//CCL8//PLAA//CCNY//CCNL1//CCND1//CCNT2//SBF2//IL2//ITSN1//MOB1B//LRP6//UBXN2B//PPP4R2//PPP1R16B//PPP2R5E//PPP1R15B//ESR1//SPRED1//PIK3CA//NRG1//PI15//TAOK1//CALM2//TMBIM6//FBXW7//MTMR9// |
| GO:0042802 | identical protein binding | Molecular function | 108 | 1661 | 906 | 17548 | 1.25937060176787 | 0.00684200690374145 | 0.127316661153498 | 2.16481649193767 | 0.119205298013245 | CEBPB//SPPL3//CR2//DYNLL2//SYT6//GADD45A//FLNA//FMR1//TIMM9//CDH20//GRIK2//GSTM3//NRBP1//HPRT1//INHBB//JUN//FIBIN//LRP6//MGST1//MID1//NAGA//NEUROG1//NR4A2//CHMP3//ZBTB4//BCL2A1//CLIP1//SARS//DLK2//SNX2//TFAP2A//TTK//VEGFB//BHLHE41//E2F8//FXR1//SBF2//GNPTG//ACCS//BHLHE40//ACVR1//CD3E//NOG//SLIT2//FOXP2//VPS4B//CD69//SLK//TCERG1//C1QTNF2//C1QTNF6//CLCN5//CRKL//APCDD1//DPYSL2//EPS15//ESR1//ETS1//ARL6IP1//SIRT1//BRI3//APPL1//FOXP1//YIPF6//TBK1//HSBP1//TRIM23//KCNJ2//KDR//LDLR//MARCKS//MET//RAB9B//IER5//PON2//MIEF1//POU3F2//PPARG//THG1L//ATG16L1//FBXW7//FOXJ2//MAPK1//PTEN//ZBTB26//PTPRG//MID1IP1//RAF1//RB1//RBBP8//ROBO2//ATXN1//SDC1//BNIP2//SYT1//TNF//TNFAIP3//TSN//YWHAG//ZNF3//ADIPOR2//CBLL1//GDF5//CASP2//KATNAL1//TP63//NMI//DAZAP2// |
| GO:0001105 | RNA polymerase II transcription coactivator activity | Molecular function | 7 | 44 | 906 | 17548 | 3.08137668071443 | 0.00687667151292044 | 0.127316661153498 | 2.16262172068245 | 0.00772626931567329 | ZFPM2//ISL1//MEF2A//NEUROD1//POU3F2//SMARCA2//SOX4// |
| GO:0019899 | enzyme binding | Molecular function | 144 | 2301 | 906 | 17548 | 1.2121191189549 | 0.00727470689813613 | 0.132789016056119 | 2.13818449994844 | 0.158940397350993 | AGO1//GTF2E1//F3//ALPI//LDLR//SERPINB13//TNF//TNFAIP3//MARCKS//SRSF2//YWHAG//RAPGEF4//RAB3GAP1//ARHGEF4//DOCK10//ITSN1//SOS2//RALGPS1//IQSEC2//FBXO8//PSD//RANBP9//ADRB1//EREG//RASGEF1A//NRG1//IL2//MET//RAPGEF2//PLEKHG4B//AKAP11//RAF1//CALM2//RGL1//TNPO2//SPHK2//RAP1A//IQGAP2//FLNA//DENND1B//DENND6A//TRAPPC8//RIN2//SBF2//RIMS4//TBC1D12//ARHGAP1//RALBP1//CEBPB//GADD45A//ATF5//MYCN//RB1//BTG1//MOB1B//CACUL1//SPRED1//ESR1//CCNY//RICTOR//RHOB//RND3//MEF2A//MSN//PPP1R12A//ERRFI1//MAPK6//MAVS//ZBTB4//CCND1//FAM83D//SOCS1//CCND2//CD3E//CD8A//BAG5//RCAN3//PPP3R1//MAPK1//PPARG//MTMR9//EPS8//TOB1//NCK1//SOCS5//ARAP2//APPL1//AGFG1//PEX5L//GIT2//KDR//ROR1//TGFBR2//DCUN1D4//GRIK2//ANKIB1//LONRF3//LONRF1//GABARAPL1//SPOPL//JUN//MID1//PCBP2//JKAMP//UBE2W//FBXW7//PRKACB//PRKAR2B//TMBIM6//UBE2V1//BAG6//PRR5L//CUL5//FZD6//ETS1//GLI3//DNMT3B//HIC1//MIER1//BHLHE41//UBE2I//PAK6//SIRT1//POU4F1//ATG16L1//RRAGD//RASA1//AR//ARPP19//STRN//TCERG1//CCNT2//PTGES3//CHMP3//PTEN//GJA1//CDK19//GSTM3//MDM4//EGLN1//TRAK2//YES1//CASP2//HDHD2// |
| GO:0017160 | Ral GTPase binding | Molecular function | 4 | 16 | 906 | 17548 | 4.84216335540839 | 0.00780301232457436 | 0.137699000204219 | 2.10773770713157 | 0.00441501103752759 | RGL1//RALGPS1//RALBP1//FLNA// |
| GO:0061659 | ubiquitin-like protein ligase activity | Molecular function | 21 | 229 | 906 | 17548 | 1.77616472425461 | 0.00783466314000139 | 0.137699000204219 | 2.10597967176509 | 0.0231788079470199 | CACUL1//RNF38//RNF145//ZNRF2//PDZRN3//RNF11//MYLIP//MDM4//ANKIB1//UBE2W//SMURF1//HECW2//RNF2//SKP1//UBE2V1//LONRF3//CBLL1//CUL5//FBXO30//UBE3B//LONRF1// |
| GO:0005543 | phospholipid binding | Molecular function | 33 | 410 | 906 | 17548 | 1.55894039735099 | 0.00786244291289525 | 0.137699000204219 | 2.10444249483184 | 0.0364238410596026 | SYT6//SYT1//ANXA7//C2CD5//WDFY3//PHLDA3//KCNJ2//PFN2//SESTD1//IQGAP2//ARAP2//SAP30L//CHMP3//SNX19//WDR45B//PLEKHF2//SNX27//C8ORF44-SGK3//SGK3//SNX5//PXK//SNX2//TUB//SBF2//SNX17//LPAR1//ARFIP1//RAPGEF2//DENND1B//F3//NR5A2//PSD//SMURF1// |
| GO:0008013 | beta-catenin binding | Molecular function | 10 | 82 | 906 | 17548 | 2.36203090507726 | 0.00943862062116231 | 0.156695594875353 | 2.02509146972621 | 0.011037527593819 | EP300//ESR1//CD2AP//GLI3//AR//NR4A2//RORA//SKP1//TBL1XR1//DLG5// |
| GO:0035591 | signaling adaptor activity | Molecular function | 10 | 82 | 906 | 17548 | 2.36203090507726 | 0.00943862062116231 | 0.156695594875353 | 2.02509146972621 | 0.011037527593819 | TOB1//CRKL//EPS8//ARHGAP1//LASP1//ARHGAP6//NCK1//SH3BGRL//SKAP2//HOMER1// |
| GO:0005085 | guanyl-nucleotide exchange factor activity | Molecular function | 27 | 323 | 906 | 17548 | 1.619051524409 | 0.00957352389484697 | 0.156695594875353 | 2.01892817440809 | 0.0298013245033113 | IQSEC2//FBXO8//PSD//RANBP9//RAPGEF4//ADRB1//EREG//RASGEF1A//NRG1//IL2//MET//RAPGEF2//PLEKHG4B//ARHGEF4//ITSN1//SOS2//RGL1//RALGPS1//RAP1A//DENND1B//DENND6A//TRAPPC8//RAB3GAP1//RIN2//SBF2//EPS8//DOCK10// |
| GO:0032266 | phosphatidylinositol-3-phosphate binding | Molecular function | 6 | 36 | 906 | 17548 | 3.22810890360559 | 0.00961131302751297 | 0.156695594875353 | 2.01721727813264 | 0.00662251655629139 | PHLDA3//SNX19//WDR45B//PLEKHF2//SNX27//SESTD1// |
| GO:0005283 | amino acid:sodium symporter activity | Molecular function | 4 | 17 | 906 | 17548 | 4.55733021685495 | 0.00979347467970956 | 0.156695594875353 | 2.00906319517404 | 0.00441501103752759 | SLC6A15//SLC6A8//SLC6A1//SLC6A11// |
| GO:0017081 | chloride channel regulator activity | Molecular function | 4 | 17 | 906 | 17548 | 4.55733021685495 | 0.00979347467970956 | 0.156695594875353 | 2.00906319517404 | 0.00441501103752759 | C8ORF44-SGK3//SGK2//SGK3//SGK1// |
| GO:0030228 | lipoprotein particle receptor activity | Molecular function | 4 | 17 | 906 | 17548 | 4.55733021685495 | 0.00979347467970956 | 0.156695594875353 | 2.00906319517404 | 0.00441501103752759 | LRP12//LDLR//LRP6//LRP8// |
| GO:0036002 | pre-mRNA binding | Molecular function | 6 | 37 | 906 | 17548 | 3.14086271702166 | 0.0109828179099224 | 0.173582097698286 | 1.9592862167422 | 0.00662251655629139 | SLBP//EP300//RBM41//CELF1//SRSF2//SRSF6// |
| GO:0005328 | neurotransmitter:sodium symporter activity | Molecular function | 5 | 27 | 906 | 17548 | 3.58678767067288 | 0.0113479430148207 | 0.1771919776772 | 1.94508285373722 | 0.00551876379690949 | SLC6A1//SLC6A11//SLC6A15//SLC6A6//SLC6A8// |
| GO:0015467 | G-protein activated inward rectifier potassium channel activity | Molecular function | 3 | 10 | 906 | 17548 | 5.81059602649007 | 0.012526287292105 | 0.188768236401954 | 1.9021776319174 | 0.0033112582781457 | KCNJ2//KCNJ3//KCNJ10// |
| GO:0030957 | Tat protein binding | Molecular function | 3 | 10 | 906 | 17548 | 5.81059602649007 | 0.012526287292105 | 0.188768236401954 | 1.9021776319174 | 0.0033112582781457 | GABARAPL1//DLL1//MDFIC// |
| GO:0140161 | monocarboxylate:sodium symporter activity | Molecular function | 3 | 10 | 906 | 17548 | 5.81059602649007 | 0.012526287292105 | 0.188768236401954 | 1.9021776319174 | 0.0033112582781457 | SLC6A1//SLC6A11//SLC10A2// |
| GO:0004033 | aldo-keto reductase (NADP) activity | Molecular function | 5 | 29 | 906 | 17548 | 3.33942300372992 | 0.0153365465550675 | 0.228461659027212 | 1.81427242271605 | 0.00551876379690949 | ADH4//RDH10//RDH14//DHRS3//KCNAB2// |
| GO:0005267 | potassium channel activity | Molecular function | 13 | 129 | 906 | 17548 | 1.95187980218013 | 0.0159521443976156 | 0.233860081322511 | 1.79718092781352 | 0.0143487858719647 | KCNJ2//KCNJ3//KCNJ10//CNGA3//KCNH4//KCNA4//KCNB1//KCNE1//KCNAB2//KCNK2//GRIK2//KCNK10//KCNIP4// |
| GO:0051219 | phosphoprotein binding | Molecular function | 9 | 76 | 906 | 17548 | 2.29365632624608 | 0.0160908855335859 | 0.233860081322511 | 1.79342005461027 | 0.00993377483443709 | CRKL//MAPK1//RASA1//YES1//SOCS3//FBXW7//TBK1//MID1//RB1// |
| GO:0004672 | protein kinase activity | Molecular function | 46 | 644 | 906 | 17548 | 1.3834752444024 | 0.0162402834251744 | 0.233860081322511 | 1.78940639572814 | 0.0507726269315673 | KCNH4//C8ORF44-SGK3//SGK2//HIPK3//FASTK//PDIK1L//SGK3//TBK1//NRBP1//IRAK4//CDK17//PIK3CA//PRKAA1//PRKACB//MAPK1//MAPK6//PAK6//TAOK1//RAF1//SGK1//CDK15//STK33//TTK//ACVR1//CCNT2//RPS6KA5//ULK2//SLK//TGFBR2//CDK19//CCND1//BMPR2//GRK6//KDR//MET//ROR1//EREG//NRG1//YES1//TXK//GTF2H1//MMD//PAN3//CCL2//CCL8//RASSF2// |
| GO:0001134 | transcription factor activity, transcription factor recruiting | Molecular function | 5 | 30 | 906 | 17548 | 3.22810890360559 | 0.0176420706292653 | 0.25125410478602 | 1.75345044357556 | 0.00551876379690949 | PPARG//RORA//MYB//MYBL1//TEAD2// |
| GO:0046982 | protein heterodimerization activity | Molecular function | 37 | 500 | 906 | 17548 | 1.43328035320088 | 0.0179006279950727 | 0.252165368278415 | 1.74713173270667 | 0.0408388520971302 | CEBPB//CHRNB4//DYNLL2//ADRB1//GADD45A//FMR1//SNX5//GTF2A1//H2AFZ//JUN//KCNB1//MEF2A//MID1//MYOG//NEUROD1//NR4A2//PKNOX1//PPARG//PPP2CA//MAPK6//RRAGD//RAF1//BCL2A1//SNX2//SOS2//SOX4//SYT1//VEGFB//BHLHE41//ADIPOR2//FXR1//HIST2H2BE//TEAD2//ALG2//BHLHE40//CD3E//FOXP2// |
| GO:0043022 | ribosome binding | Molecular function | 7 | 53 | 906 | 17548 | 2.55812403681953 | 0.0185693337060029 | 0.258772650354621 | 1.73120367908185 | 0.00772626931567329 | CPEB2//CPEB3//FMR1//RICTOR//SEC61A2//CPEB1//BAG6// |
| GO:0003730 | mRNA 3'-UTR binding | Molecular function | 8 | 66 | 906 | 17548 | 2.34771556625861 | 0.0198783804136594 | 0.271870830200739 | 1.70161900253748 | 0.00883002207505519 | CPEB2//CPEB3//CPEB1//ELAVL2//FMR1//BOLL//FXR1//SECISBP2L// |
| GO:1901981 | phosphatidylinositol phosphate binding | Molecular function | 14 | 147 | 906 | 17548 | 1.8446336592032 | 0.0199288031397147 | 0.271870830200739 | 1.70051878285624 | 0.0154525386313466 | PHLDA3//KCNJ2//PFN2//SYT1//SESTD1//IQGAP2//ARAP2//SAP30L//SNX19//WDR45B//PLEKHF2//SNX27//ARFIP1//DENND1B// |
| GO:0001223 | transcription coactivator binding | Molecular function | 4 | 21 | 906 | 17548 | 3.68926731840639 | 0.0209153420106643 | 0.272812017502805 | 1.6795350293798 | 0.00441501103752759 | ESR1//RORA//TEAD2//CCNT2// |
| GO:0005416 | amino acid:cation symporter activity | Molecular function | 4 | 21 | 906 | 17548 | 3.68926731840639 | 0.0209153420106643 | 0.272812017502805 | 1.6795350293798 | 0.00441501103752759 | SLC6A15//SLC6A8//SLC6A1//SLC6A11// |
| GO:0046873 | metal ion transmembrane transporter activity | Molecular function | 34 | 457 | 906 | 17548 | 1.44099390883051 | 0.0210085831461535 | 0.272812017502805 | 1.67760323614979 | 0.0375275938189845 | KCNJ2//KCNJ3//KCNJ10//SCN1B//CNGA3//KCNH4//KCNA4//KCNB1//KCNE1//KCNAB2//GPM6A//SLC24A3//TRPC3//CUL5//KCNK10//KCNIP4//SLC6A15//SLC6A8//SLC6A6//SLC6A1//SLC6A11//SLC31A2//SLC25A28//SLC30A6//ATP2C1//SLC10A2//SLC41A1//MMGT1//KCNK2//FAM155A//GRIK2//STIM2//SLC9A6//SLC23A2// |
| GO:0005247 | voltage-gated chloride channel activity | Molecular function | 3 | 12 | 906 | 17548 | 4.84216335540839 | 0.0212608130924254 | 0.272812017502805 | 1.67242013046177 | 0.0033112582781457 | CLCN5//CLCN6//ANO1// |
| GO:0035925 | mRNA 3'-UTR AU-rich region binding | Molecular function | 3 | 12 | 906 | 17548 | 4.84216335540839 | 0.0212608130924254 | 0.272812017502805 | 1.67242013046177 | 0.0033112582781457 | CPEB2//CPEB3//CPEB1// |
| GO:0086008 | voltage-gated potassium channel activity involved in cardiac muscle cell action potential repolarization | Molecular function | 3 | 12 | 906 | 17548 | 4.84216335540839 | 0.0212608130924254 | 0.272812017502805 | 1.67242013046177 | 0.0033112582781457 | KCNJ3//KCNE1//KCNJ2// |
| GO:0001158 | enhancer sequence-specific DNA binding | Molecular function | 10 | 94 | 906 | 17548 | 2.06049504485463 | 0.0230473655706787 | 0.289994036695142 | 1.6373787094719 | 0.011037527593819 | ARX//FLI1//NR5A2//H2AFZ//JUN//MEOX2//POU4F1//SMARCD2//ISL1//SLC2A4RG// |
| GO:0005249 | voltage-gated potassium channel activity | Molecular function | 10 | 94 | 906 | 17548 | 2.06049504485463 | 0.0230473655706787 | 0.289994036695142 | 1.6373787094719 | 0.011037527593819 | KCNJ2//KCNJ3//KCNJ10//KCNA4//KCNB1//KCNE1//KCNK2//CNGA3//KCNH4//KCNAB2// |
| GO:0140096 | catalytic activity, acting on a protein | Molecular function | 143 | 2370 | 906 | 17548 | 1.16865714738127 | 0.0238815434827198 | 0.294722963374857 | 1.62193760784561 | 0.157836644591611 | KCNH4//NKTR//TGM3//GCNT4//PSMA2//NPEPL1//CPD//NRIP3//CASP2//CTSF//ADAMTS17//MEP1A//NLN//THOP1//ADAM12//ADAM18//LMLN//PRSS55//F3//KLK15//SIN3B//SIRT1//ARID4B//MIER1//SAP30L//C8ORF44-SGK3//HIPK3//MMD//SGK3//PAN3//TBK1//GTF2H1//IRAK4//CDK17//PRKAA1//TAOK1//RAF1//CCL2//CCL8//ACVR1//RPS6KA5//RASSF2//SGK2//FASTK//PDIK1L//NRBP1//PIK3CA//PRKACB//MAPK1//MAPK6//PAK6//SGK1//CDK15//STK33//TTK//CCNT2//ULK2//SLK//TGFBR2//CDK19//CCND1//BMPR2//GRK6//KDR//MET//ROR1//EREG//NRG1//YES1//TXK//PCMTD1//PPTC7//CTDSPL2//PPP2CA//PTEN//CDC14A//PPP2CB//PPM1F//PPP3R1//PTPN21//PTPRG//PTP4A1//PTP4A2//DUSP1//ZER1//FBXO41//RNF38//ZNRF2//PDZRN3//RNF167//KLHL3//RNF11//KLHL20//MYLIP//TRIM23//UBE2W//FBXW7//SMURF1//KLHL42//RNF2//SKP1//TNFAIP3//CBLL1//CUL5//FBXO30//KBTBD8//LONRF1//FBXO44//OTUD1//OTUD3//USP27X//USP28//USP38//AMZ1//IMMP2L//ABHD17C//KATNAL1//VPS4B//EOGT//PRDM16//SUV39H1//ZDHHC22//ZDHHC7//ZDHHC18//ATG16L1//UBE2I//CCDC126//EGLN1//EGLN3//KDM2A//PHF8//ARID5B//ATXN7//WDR20//SPPL3//CACUL1//RNF145//MDM4//ANKIB1//HECW2//UBE2V1//LONRF3//UBE3B// |
| GO:0001664 | G-protein coupled receptor binding | Molecular function | 22 | 271 | 906 | 17548 | 1.5723630083983 | 0.0243137668257573 | 0.294722963374857 | 1.61414775259591 | 0.0242825607064018 | BAMBI//LRP6//WNT3//WNT7A//WNT10A//CXCL9//CCL2//CCL8//CCL21//ADRB1//RAPGEF2//ARRDC3//EDNRB//EDN1//EDN2//PPP1R1B//GNAQ//IL2//FLNA//ADCYAP1//HOMER1//S1PR1// |
| GO:0008106 | alcohol dehydrogenase (NADP+) activity | Molecular function | 4 | 22 | 906 | 17548 | 3.52157334938792 | 0.0245452980062535 | 0.294722963374857 | 1.61003169073112 | 0.00441501103752759 | ADH4//RDH10//RDH14//DHRS3// |
| GO:0017075 | syntaxin-1 binding | Molecular function | 4 | 22 | 906 | 17548 | 3.52157334938792 | 0.0245452980062535 | 0.294722963374857 | 1.61003169073112 | 0.00441501103752759 | SYBU//SNAP25//SYT1//VAMP3// |
| GO:0045309 | protein phosphorylated amino acid binding | Molecular function | 6 | 44 | 906 | 17548 | 2.64118001204094 | 0.0246258064107063 | 0.294722963374857 | 1.60860953894261 | 0.00662251655629139 | CRKL//MAPK1//RASA1//YES1//SOCS3//FBXW7// |
| GO:0015267 | channel activity | Molecular function | 34 | 463 | 906 | 17548 | 1.42232012167503 | 0.0248498695082344 | 0.294722963374857 | 1.60467588749413 | 0.0375275938189845 | GJC1//CNGA3//ANO1//CHRNB4//GABRA5//GLRB//GLRA3//GRIK2//KCNJ2//KCNJ3//KCNJ10//GJA1//CLIC4//KCNK10//SCN1B//KCNIP4//CLCN5//CLCN6//KCNH4//KCNA4//KCNB1//KCNE1//KCNAB2//SLC26A7//SLC26A4//GPM6A//SLC24A3//TRPC3//CUL5//SLC14A1//KCNK2//FAM155A//STIM2//BCL2A1// |
| GO:0015079 | potassium ion transmembrane transporter activity | Molecular function | 15 | 166 | 906 | 17548 | 1.75017952605122 | 0.0250478900656414 | 0.294722963374857 | 1.60122885149103 | 0.0165562913907285 | KCNJ2//KCNJ3//KCNJ10//CNGA3//KCNH4//KCNA4//KCNB1//KCNE1//KCNAB2//KCNK10//KCNIP4//SLC24A3//KCNK2//GRIK2//SLC9A6// |
| GO:0005254 | chloride channel activity | Molecular function | 9 | 82 | 906 | 17548 | 2.12582781456954 | 0.025242476029791 | 0.294722963374857 | 1.5978680474733 | 0.00993377483443709 | ANO1//CLCN5//CLCN6//GLRB//GLRA3//GABRA5//SLC26A7//CLIC4//SLC26A4// |
| GO:0022803 | passive transmembrane transporter activity | Molecular function | 34 | 464 | 906 | 17548 | 1.41925477658522 | 0.0255404450382533 | 0.295539435442645 | 1.59277153949337 | 0.0375275938189845 | GJC1//CNGA3//ANO1//CHRNB4//GABRA5//GLRB//GLRA3//GRIK2//KCNJ2//KCNJ3//KCNJ10//GJA1//CLIC4//KCNK10//SCN1B//KCNIP4//CLCN5//CLCN6//KCNH4//KCNA4//KCNB1//KCNE1//KCNAB2//SLC26A7//SLC26A4//GPM6A//SLC24A3//TRPC3//CUL5//SLC14A1//BCL2A1//KCNK2//FAM155A//STIM2// |
| GO:0016896 | exoribonuclease activity, producing 5'-phosphomonoesters | Molecular function | 5 | 33 | 906 | 17548 | 2.93464445782327 | 0.0258929360556445 | 0.295738639952159 | 1.5868187011886 | 0.00551876379690949 | CNOT7//DCP2//CNOT6L//PAN3//CNOT6// |
| GO:0008417 | fucosyltransferase activity | Molecular function | 3 | 13 | 906 | 17548 | 4.4696892511462 | 0.0265983575415806 | 0.295738639952159 | 1.5751451803859 | 0.0033112582781457 | FUT2//FUT5//FUT9// |
| GO:0010314 | phosphatidylinositol-5-phosphate binding | Molecular function | 3 | 13 | 906 | 17548 | 4.4696892511462 | 0.0265983575415806 | 0.295738639952159 | 1.5751451803859 | 0.0033112582781457 | PHLDA3//SAP30L//SESTD1// |
| GO:0015355 | secondary active monocarboxylate transmembrane transporter activity | Molecular function | 3 | 13 | 906 | 17548 | 4.4696892511462 | 0.0265983575415806 | 0.295738639952159 | 1.5751451803859 | 0.0033112582781457 | SLC6A1//SLC6A11//SLC10A2// |
| GO:0051020 | GTPase binding | Molecular function | 46 | 665 | 906 | 17548 | 1.3397865524739 | 0.0266986272179032 | 0.295738639952159 | 1.57351106848624 | 0.0507726269315673 | RAPGEF4//RAB3GAP1//ARHGEF4//DOCK10//ITSN1//SOS2//RALGPS1//IQSEC2//FBXO8//PSD//RANBP9//ADRB1//EREG//RASGEF1A//NRG1//IL2//MET//RAPGEF2//PLEKHG4B//RGL1//TNPO2//SPHK2//RAP1A//IQGAP2//FLNA//DENND1B//DENND6A//TRAPPC8//RIN2//SBF2//RIMS4//TBC1D12//ARHGAP1//RALBP1//EPS8//ARAP2//APPL1//AGFG1//PEX5L//ERRFI1//GIT2//PAK6//POU4F1//ATG16L1//RRAGD//RASA1// |
| GO:0005244 | voltage-gated ion channel activity | Molecular function | 17 | 198 | 906 | 17548 | 1.66296519276652 | 0.0277688226091475 | 0.302423479844161 | 1.5564425338324 | 0.0187637969094923 | KCNJ2//KCNJ3//KCNJ10//CLCN5//CLCN6//ANO1//CNGA3//KCNH4//KCNA4//KCNB1//KCNE1//KCNAB2//KCNK2//CLIC4//KCNK10//SCN1B//KCNIP4// |
| GO:0022832 | voltage-gated channel activity | Molecular function | 17 | 198 | 906 | 17548 | 1.66296519276652 | 0.0277688226091475 | 0.302423479844161 | 1.5564425338324 | 0.0187637969094923 | KCNJ2//KCNJ3//KCNJ10//CLIC4//KCNK10//SCN1B//KCNIP4//CLCN5//CLCN6//ANO1//CNGA3//KCNH4//KCNA4//KCNB1//KCNE1//KCNAB2//KCNK2// |
| GO:0070412 | R-SMAD binding | Molecular function | 4 | 23 | 906 | 17548 | 3.36846146463192 | 0.0285320861956949 | 0.305599865368765 | 1.54466647264175 | 0.00441501103752759 | JUN//PMEPA1//SMURF1//LDLRAD4// |
| GO:0070717 | poly-purine tract binding | Molecular function | 4 | 23 | 906 | 17548 | 3.36846146463192 | 0.0285320861956949 | 0.305599865368765 | 1.54466647264175 | 0.00441501103752759 | TIA1//PATL1//FMR1//ATXN1// |
| GO:0031624 | ubiquitin conjugating enzyme binding | Molecular function | 5 | 34 | 906 | 17548 | 2.84833138553435 | 0.0291087262886239 | 0.309220567787349 | 1.53597679758161 | 0.00551876379690949 | DCUN1D4//GRIK2//ANKIB1//LONRF3//LONRF1// |
| GO:0022838 | substrate-specific channel activity | Molecular function | 32 | 437 | 906 | 17548 | 1.41829956405555 | 0.0298283658524825 | 0.314289123128596 | 1.5253705388166 | 0.0353200883002208 | GJC1//CNGA3//ANO1//CHRNB4//GABRA5//GLRB//GLRA3//GRIK2//KCNJ2//KCNJ3//KCNJ10//CLCN5//CLCN6//SCN1B//KCNH4//KCNA4//KCNB1//KCNE1//KCNAB2//SLC26A7//CLIC4//SLC26A4//GPM6A//SLC24A3//TRPC3//CUL5//KCNK10//KCNIP4//SLC14A1//KCNK2//FAM155A//STIM2// |
| GO:0019887 | protein kinase regulator activity | Molecular function | 17 | 200 | 906 | 17548 | 1.64633554083885 | 0.0301968043801751 | 0.315605310296024 | 1.52003901444314 | 0.0187637969094923 | NCK1//GSKIP//PPP1R1B//SOCS1//SOCS3//SOCS5//PRKAR2B//YWHAG//CCNY//CCNL1//CCND1//CCNT2//SPRED1//PIK3CA//NRG1//TAOK1//CALM2// |
| GO:0003707 | steroid hormone receptor activity | Molecular function | 7 | 59 | 906 | 17548 | 2.29797582968534 | 0.0315766914853094 | 0.317898626644853 | 1.50063337606763 | 0.00772626931567329 | AR//ESR1//NR5A2//NR3C2//NR4A2//PPARG//RORA// |
| GO:0048306 | calcium-dependent protein binding | Molecular function | 7 | 59 | 906 | 17548 | 2.29797582968534 | 0.0315766914853094 | 0.317898626644853 | 1.50063337606763 | 0.00772626931567329 | STMN2//SYT6//ANXA7//S100PBP//SNAP25//SYT1//ALG2// |
| GO:0004674 | protein serine/threonine kinase activity | Molecular function | 36 | 505 | 906 | 17548 | 1.38073568946299 | 0.0315816722091556 | 0.317898626644853 | 1.50056487838149 | 0.0397350993377483 | TGFBR2//ACVR1//PRKAA1//PRKACB//CDK19//CDK17//CCND1//CDK15//BMPR2//GRK6//MAPK1//MAPK6//KDR//MET//ROR1//TAOK1//RAF1//GTF2H1//FASTK//C8ORF44-SGK3//SGK2//HIPK3//PDIK1L//SGK3//TBK1//NRBP1//IRAK4//PIK3CA//PAK6//SGK1//STK33//TTK//CCNT2//RPS6KA5//ULK2//SLK// |
| GO:0019787 | ubiquitin-like protein transferase activity | Molecular function | 34 | 472 | 906 | 17548 | 1.39519961088038 | 0.0316193455617643 | 0.317898626644853 | 1.5000471230773 | 0.0375275938189845 | ZER1//FBXO41//RNF38//ZNRF2//PDZRN3//RNF167//KLHL3//RNF11//KLHL20//MYLIP//TRIM23//UBE2W//FBXW7//SMURF1//KLHL42//RNF2//SKP1//TNFAIP3//CBLL1//CUL5//FBXO30//KBTBD8//LONRF1//FBXO44//UBE2I//CACUL1//RNF145//MDM4//ANKIB1//HECW2//UBE2V1//LONRF3//UBE3B//ATG16L1// |
| GO:0019210 | kinase inhibitor activity | Molecular function | 10 | 99 | 906 | 17548 | 1.95642963854884 | 0.0316426873743719 | 0.317898626644853 | 1.49972663951385 | 0.011037527593819 | NCK1//GSKIP//PPP1R1B//SOCS1//SOCS3//SOCS5//PRKAR2B//YWHAG//SPRED1//LRP6// |
| GO:0001784 | phosphotyrosine residue binding | Molecular function | 5 | 35 | 906 | 17548 | 2.76695048880479 | 0.0325657956555619 | 0.318012438423585 | 1.48723830657024 | 0.00551876379690949 | CRKL//MAPK1//RASA1//YES1//SOCS3// |
| GO:0004532 | exoribonuclease activity | Molecular function | 5 | 35 | 906 | 17548 | 2.76695048880479 | 0.0325657956555619 | 0.318012438423585 | 1.48723830657024 | 0.00551876379690949 | CNOT7//DCP2//CNOT6L//PAN3//CNOT6// |
| GO:0005095 | GTPase inhibitor activity | Molecular function | 3 | 14 | 906 | 17548 | 4.15042573320719 | 0.0325815211396756 | 0.318012438423585 | 1.48702864356509 | 0.0033112582781457 | IQGAP2//CPEB2//SLIT2// |
| GO:0005216 | ion channel activity | Molecular function | 31 | 424 | 906 | 17548 | 1.41610437752509 | 0.0326908959627494 | 0.318012438423585 | 1.48557317650297 | 0.0342163355408389 | CNGA3//ANO1//CHRNB4//GABRA5//GLRB//GLRA3//GRIK2//KCNJ2//KCNJ3//KCNJ10//CLCN5//CLCN6//SCN1B//KCNH4//KCNA4//KCNB1//KCNE1//KCNAB2//SLC26A7//CLIC4//SLC26A4//GPM6A//SLC24A3//TRPC3//CUL5//KCNK10//KCNIP4//KCNK2//FAM155A//STIM2//GJC1// |
| GO:0030159 | receptor signaling complex scaffold activity | Molecular function | 4 | 24 | 906 | 17548 | 3.22810890360559 | 0.032880915701204 | 0.318012438423585 | 1.48305609628885 | 0.00441501103752759 | NCK1//CD3E//DLG5//G3BP2// |
| GO:0030165 | PDZ domain binding | Molecular function | 9 | 87 | 906 | 17548 | 2.00365380223795 | 0.0352872440803934 | 0.338757543171777 | 1.45238225854041 | 0.00993377483443709 | ADRB1//DTNA//LPAR1//GJA1//GRIK2//PTEN//FZD3//CXXC4//RAPGEF2// |
| GO:0008022 | protein C-terminus binding | Molecular function | 16 | 189 | 906 | 17548 | 1.63967436373617 | 0.0358502897250789 | 0.341632172674281 | 1.44550733021948 | 0.0176600441501104 | CNGA3//EP300//SIRT1//CD2AP//NPAT//HPCAL4//PPARG//PPP2CA//PPP2CB//PRKAA1//MID1IP1//ATXN1//SDC1//SYT1//VPS4B//SNX17// |
| GO:0017091 | AU-rich element binding | Molecular function | 4 | 25 | 906 | 17548 | 3.09898454746137 | 0.0375951847226649 | 0.353959035809298 | 1.42486777694191 | 0.00441501103752759 | CPEB2//CPEB3//CPEB1//TIA1// |
| GO:0004842 | ubiquitin-protein transferase activity | Molecular function | 32 | 446 | 906 | 17548 | 1.38967916926519 | 0.0379159182641371 | 0.353959035809298 | 1.42117842161811 | 0.0353200883002208 | CACUL1//RNF38//RNF145//ZNRF2//PDZRN3//RNF11//MYLIP//MDM4//ANKIB1//UBE2W//SMURF1//HECW2//RNF2//SKP1//UBE2V1//LONRF3//CBLL1//CUL5//FBXO30//UBE3B//LONRF1//ZER1//FBXO41//RNF167//KLHL3//KLHL20//TRIM23//FBXW7//KLHL42//TNFAIP3//KBTBD8//FBXO44// |
| GO:0005102 | signaling receptor binding | Molecular function | 98 | 1594 | 906 | 17548 | 1.19079550521963 | 0.0379631990567071 | 0.353959035809298 | 1.42063719787446 | 0.108167770419426 | S1PR1//FLNA//GNAQ//NPTN//BAMBI//LRP6//WNT3//WNT7A//WNT10A//DLL1//EDN1//TXLNA//NRG1//IFNA1//IFNG//IL2//INHBB//CXCL9//BMP3//TNF//GDF5//IRAK4//EREG//SNX2//YES1//SOCS5//IGF1//YWHAG//SOCS1//PTEN//SIVA1//NTF3//CD2AP//SPRED1//NPNT//ANXA7//KDR//EDN2//PTHLH//ADCYAP1//CCL2//CCL8//CCL21//OGN//VEGFB//ISL1//SEMA4C//PPARGC1B//ESR1//PPARG//STRN//TOB1//NCK1//ADRB1//RAPGEF2//ARRDC3//EDNRB//PPP1R1B//RIC3//TGFBR2//HOMER1//TACC2//EP300//SIRT1//CEBPB//NR4A2//CD8A//CD3E//RAET1E//EFNA1//EFNB2//SLIT2//FOXP1//RB1//FOXP2//SYT1//SNX17//GABARAPL1//GABRA5//PPP2CA//TRAK2//NRXN3//ARPP19//TMED1//STRAP//NXPH3//C1QTNF2//CNTFR//GJA1//NSG1//AR//ITGB8//MSN//RAB8B//CYTL1//RASA1//TXK//BAG6// |
| GO:0000980 | RNA polymerase II distal enhancer sequence-specific DNA binding | Molecular function | 8 | 75 | 906 | 17548 | 2.06598969830758 | 0.0391314079647013 | 0.354161546042009 | 1.40747452571803 | 0.00883002207505519 | ARX//FLI1//NR5A2//H2AFZ//JUN//MEOX2//POU4F1//SMARCD2// |
| GO:0034483 | heparan sulfate sulfotransferase activity | Molecular function | 3 | 15 | 906 | 17548 | 3.87373068432671 | 0.0392023906560383 | 0.354161546042009 | 1.40668744785027 | 0.0033112582781457 | HS3ST5//HS3ST3A1//HS3ST1// |
| GO:0045125 | bioactive lipid receptor activity | Molecular function | 3 | 15 | 906 | 17548 | 3.87373068432671 | 0.0392023906560383 | 0.354161546042009 | 1.40668744785027 | 0.0033112582781457 | S1PR1//SPHK2//LPAR1// |
| GO:0004879 | nuclear receptor activity | Molecular function | 6 | 49 | 906 | 17548 | 2.37167184754697 | 0.0393512828935565 | 0.354161546042009 | 1.40504110461342 | 0.00662251655629139 | AR//ESR1//NR5A2//NR4A2//PPARG//RORA// |
| GO:0098531 | transcription factor activity, direct ligand regulated sequence-specific DNA binding | Molecular function | 6 | 49 | 906 | 17548 | 2.37167184754697 | 0.0393512828935565 | 0.354161546042009 | 1.40504110461342 | 0.00662251655629139 | NR5A2//AR//NR4A2//PPARG//RORA//ESR1// |
| GO:0005109 | frizzled binding | Molecular function | 5 | 37 | 906 | 17548 | 2.61738559751805 | 0.0402189618216539 | 0.357012154252489 | 1.39556914410591 | 0.00551876379690949 | BAMBI//LRP6//WNT3//WNT7A//WNT10A// |
| GO:0017080 | sodium channel regulator activity | Molecular function | 5 | 37 | 906 | 17548 | 2.61738559751805 | 0.0402189618216539 | 0.357012154252489 | 1.39556914410591 | 0.00551876379690949 | SCN1B//C8ORF44-SGK3//SGK2//SGK3//SGK1// |
| GO:0003676 | nucleic acid binding | Molecular function | 242 | 4252 | 906 | 17548 | 1.10235515711085 | 0.0411649479676845 | 0.362923622898769 | 1.38547242919845 | 0.267108167770419 | SEPSECS//THG1L//HMGB3//CELF1//CPEB2//CPEB3//PURA//CPEB1//BAHD1//NR5A2//MEF2A//NFE2//PPARG//RORA//SOX4//SUV39H1//TFAP2A//CEBPB//ZMYND11//CREBRF//ETV5//ATF5//FOXF1//FOXF2//ZMYND8//GLI3//PURG//IRF2//JARID2//MYOG//NHLH2//NR4A2//KLF3//ZBTB4//ARID5B//RUNX3//GSC//CC2D1B//ELK3//EN1//EP300//ESR1//ETS1//FLI1//AGO1//H2AFZ//IRF1//AR//ISL1//JUN//MEOX2//MYB//MYBL1//MYCN//NFIA//NFIB//NFIC//OTX2//PAX5//KLF13//POU3F2//FOXJ2//RFX7//SMARCD2//TFE3//TGIF1//KLF10//TXK//ZNF217//BHLHE41//E2F8//FOSL1//KLF7//FUBP3//FOXP2//CLOCK//GADD45A//ARX//POU4F1//ZNF516//RB1//SIRT1//SARS//NPAS2//ST18//MBNL1//SLC2A4RG//FMR1//FXR1//DNAJB6//TSHZ1//ZBTB18//KLF12//ZNF526//CR2//CREBL2//RBFOX3//ZNF800//POLR3H//DNMT3B//E2F5//ZNF367//ARID2//ZFP30//KDM2A//MYT1L//ADNP//ZFPM2//SMUG1//ZNF521//HBP1//RNF11//ZBTB11//GTF2A1//HIVEP2//FOXA1//AGFG1//ZNF680//IMPDH1//ZNF662//LBR//NEUROG1//PCBP2//ZFAND6//BNC2//ZNF532//BDP1//MAPK1//PHTF2//ZBTB26//MIER1//ATXN1//SOS2//SOX5//TNFAIP3//TSN//ZNF3//ZNF708//ZNF131//ZNF227//CSDE1//ZNF655//ZSCAN5A//SAP30L//CXXC4//HIST2H2BE//LCOR//ZIC5//TP63//CBFB//DDX3Y//BTAF1//OGG1//RBBP8//AFF1//RBMS1//NABP1//MBNL2//KIF1C//TCERG1//FASTK//CKAP4//RPP14//RCAN3//STRAP//PPARGC1B//CRKL//DDX6//ELAVL2//PATL1//TNRC6B//FLNA//MORC3//PAN3//TES//GRSF1//CNOT7//ANXA7//NANOS1//NOVA1//ASCC1//YTHDF2//RPP25//NXF2//CNOT6//RBM25//SRSF2//SRSF6//ZMAT3//BTF3//TIA1//TNS1//NXF2B//UBE2I//WT1//YWHAG//ZYX//SLBP//SNIP1//ANP32A//CAST//MEX3B//RTCA//CCT6A//QKI//GTPBP1//SECISBP2L//G3BP2//MSN//PAIP2//BOLL//TRERF1//RBM41//LEMD3//EMX2//FOXP1//GTF2E1//HIC1//HOXA3//HOXD1//MAF//NR3C2//NEUROD1//PKNOX1//HIVEP3//PRDM16//TEAD2//ARID4B//SMARCA2//TNF//TBL1XR1//DCP2//ZEB1//BHLHE40//CCNT2//ADAMTS17//JAZF1//DZIP1//ZFR2//TBK1//TRIM23//KCMF1// |
| GO:0016740 | transferase activity | Molecular function | 144 | 2437 | 906 | 17548 | 1.14447521243957 | 0.0423737623662048 | 0.371056729909469 | 1.37290297328154 | 0.158940397350993 | ALG2//KCNH4//POLR3H//CHST1//PTGES3//TGM3//ST8SIA5//GCNT4//WDFY3//ST3GAL5//LPCAT3//LCLAT1//ABHD5//DNMT3B//DGKH//SPHK2//DGKE//GNPTG//CMPK1//CDS1//GFPT1//GSTA2//GSTM3//MGST1//EP300//EPC2//CLOCK//HPRT1//ESCO2//PSAT1//C8ORF44-SGK3//HIPK3//MMD//SGK3//PAN3//TBK1//GTF2H1//IRAK4//CDK17//PRKAA1//TAOK1//RAF1//CCL2//CCL8//ACVR1//RPS6KA5//RASSF2//SGK2//FASTK//PDIK1L//NRBP1//PIK3CA//PRKACB//MAPK1//MAPK6//PAK6//SGK1//CDK15//STK33//TTK//CCNT2//ULK2//SLK//TGFBR2//CDK19//CCND1//BMPR2//GRK6//KDR//MET//ROR1//EREG//NRG1//YES1//TXK//PCMTD1//SPTSSB//SPTSSA//ZER1//FBXO41//RNF38//ZNRF2//PDZRN3//RNF167//KLHL3//RNF11//KLHL20//MYLIP//TRIM23//UBE2W//FBXW7//SMURF1//KLHL42//RNF2//SKP1//TNFAIP3//CBLL1//CUL5//FBXO30//KBTBD8//LONRF1//FBXO44//FUT2//DSEL//HS3ST3A1//HS3ST1//THG1L//PIGA//UGT2B28//UGT2B7//B3GALNT2//FUT9//FUT5//HS3ST5//SUV39H1//ELOVL5//ELOVL7//EOGT//PIK3CB//ZDHHC7//ZDHHC18//GLT6D1//PIGP//PRDM16//ZDHHC22//ATG16L1//UBE2I//CCDC126//KDELC1//ESR1//GPHN//CACUL1//RNF145//MDM4//ANKIB1//HECW2//UBE2V1//LONRF3//UBE3B//SEPSECS//MARCH10//MYCBP2//MID1//KCMF1// |
| GO:0019900 | kinase binding | Molecular function | 49 | 739 | 906 | 17548 | 1.28425442173213 | 0.0431835864590324 | 0.375610255375208 | 1.36468129172189 | 0.054083885209713 | MARCKS//SRSF2//YWHAG//CACUL1//SPRED1//ESR1//CCNY//RICTOR//RHOB//RND3//MEF2A//MSN//PPP1R12A//ERRFI1//MAPK6//MAVS//ZBTB4//CCND1//CALM2//FAM83D//SOCS1//CCND2//CD3E//CD8A//BAG5//TOB1//NRG1//NCK1//SOCS5//KDR//MET//ROR1//RAF1//MAPK1//TGFBR2//PRKAR2B//APPL1//SIRT1//GJA1//PTEN//CEBPB//GADD45A//ATF5//FLNA//MYCN//RB1//BTG1//TNFAIP3//MOB1B// |
| GO:0051427 | hormone receptor binding | Molecular function | 15 | 179 | 906 | 17548 | 1.62307151577935 | 0.044393599298618 | 0.376269938491452 | 1.35267964226549 | 0.0165562913907285 | ISL1//PPARGC1B//ESR1//PPARG//STRN//TACC2//EP300//SIRT1//CEBPB//NR4A2//FOXP1//RB1//FOXP2//ADCYAP1//PTHLH// |
| GO:0005484 | SNAP receptor activity | Molecular function | 5 | 38 | 906 | 17548 | 2.54850702916231 | 0.0444207566274631 | 0.376269938491452 | 1.35241404830142 | 0.00551876379690949 | STX6//VTI1A//SNAP25//VAMP1//VAMP3// |
| GO:0016409 | palmitoyltransferase activity | Molecular function | 5 | 38 | 906 | 17548 | 2.54850702916231 | 0.0444207566274631 | 0.376269938491452 | 1.35241404830142 | 0.00551876379690949 | SPTSSB//SPTSSA//ZDHHC22//ZDHHC7//ZDHHC18// |
| GO:0030551 | cyclic nucleotide binding | Molecular function | 5 | 38 | 906 | 17548 | 2.54850702916231 | 0.0444207566274631 | 0.376269938491452 | 1.35241404830142 | 0.00551876379690949 | RAPGEF4//PRKAR2B//RAPGEF2//CNGA3//PDE5A// |
| GO:0005237 | inhibitory extracellular ligand-gated ion channel activity | Molecular function | 3 | 16 | 906 | 17548 | 3.63162251655629 | 0.0464484010402222 | 0.386520260001808 | 1.33302923175345 | 0.0033112582781457 | GLRB//GLRA3//GABRA5// |
| GO:1990381 | ubiquitin-specific protease binding | Molecular function | 3 | 16 | 906 | 17548 | 3.63162251655629 | 0.0464484010402222 | 0.386520260001808 | 1.33302923175345 | 0.0033112582781457 | CHMP3//PTEN//BAG6// |
| GO:0017112 | Rab guanyl-nucleotide exchange factor activity | Molecular function | 6 | 51 | 906 | 17548 | 2.27866510842748 | 0.0465255868520695 | 0.386520260001808 | 1.33230814017445 | 0.00662251655629139 | DENND1B//DENND6A//TRAPPC8//RAB3GAP1//RIN2//SBF2// |
| GO:0016773 | phosphotransferase activity, alcohol group as acceptor | Molecular function | 51 | 778 | 906 | 17548 | 1.26966751221505 | 0.0470507704769068 | 0.3883936212616 | 1.32743326041676 | 0.0562913907284768 | KCNH4//DGKH//SPHK2//DGKE//C8ORF44-SGK3//HIPK3//MMD//SGK3//PAN3//TBK1//GTF2H1//IRAK4//CDK17//PRKAA1//TAOK1//RAF1//CCL2//CCL8//ACVR1//RPS6KA5//RASSF2//SGK2//FASTK//PDIK1L//NRBP1//PIK3CA//PRKACB//MAPK1//MAPK6//PAK6//SGK1//CDK15//STK33//TTK//CCNT2//ULK2//SLK//TGFBR2//CDK19//CCND1//BMPR2//GRK6//KDR//MET//ROR1//EREG//NRG1//YES1//TXK//PIK3CB//ESR1// |
| GO:0043425 | bHLH transcription factor binding | Molecular function | 4 | 27 | 906 | 17548 | 2.8694301365383 | 0.0481250028787853 | 0.394746859056365 | 1.31762923150442 | 0.00441501103752759 | BHLHE41//BHLHE40//SIRT1//ISL1// |
| GO:0035257 | nuclear hormone receptor binding | Molecular function | 13 | 151 | 906 | 17548 | 1.6674999634519 | 0.0489582194796547 | 0.399055675758695 | 1.31017438506637 | 0.0143487858719647 | ISL1//PPARGC1B//ESR1//PPARG//STRN//CEBPB//NR4A2//EP300//FOXP1//RB1//FOXP2//TACC2//SIRT1// |
