# Supplementary material for: Altered microRNA Transcriptome in Cultured Human Liver Cells upon Infection with Ebola Virus
Source: Int J Mol Sci. 2021 Apr 6;22(7):3792. doi: 10.3390/ijms22073792 (PMC8038836; doi:10.3390/ijms22073792)
Supplement: Supplementary file 1 [file ijms-22-03792-s001.zip › Supplementary_File/C_ GO_Analysis_Results/16-30nt_go_Makona-96h-Huh7_vs_Control-96h-Huh7_down.mature_mirna_targets/BP_result(Human).html]

| GO.ID | Term | Ontology | Count | Pop.Hits | List.Total | Pop.Total | Fold.Enrichment | Pvalue | FDR | Enrichment.Score | Gene.Ratio | GENES |
| --- | --- | --- | --- | --- | --- | --- | --- | --- | --- | --- | --- | --- |
| GO:0007267 | cell-cell signaling | Biological process | 84 | 1658 | 509 | 17653 | 1.75709603494162 | 2.06062130107648e-07 | 0.00139359818591802 | 6.68600181509349 | 0.165029469548134 | WNT11//FERMT2//CTNND2//HIC1//TRABD2B//MARK1//CCDC88C//NDP//PRKAA1//CALCOCO1//SKP1//TNKS2//KREMEN1//VPS26A//FZD2//GNAO1//PPP3CB//FZD3//UNC13B//SLC12A7//CPLX1//CHRNA3//DLG4//HTR3E//GRIN2B//GRM2//KCNIP2//NOVA1//NPTX1//NPTX2//EXOC4//BSN//CHRFAM7A//SNPH//CHRM1//P2RX2//SYT9//DPP4//SLC25A5//IL1B//ABCC8//CACNA2D2//SYT2//SYT14//CPLX3//EDN1//SNX19//FOXP3//FGFR2//SFRP1//NFATC4//SIRT3//USP46//CA7//RAF1//VSNL1//CNTN2//KIT//SHANK3//SHISA9//SLC6A3//LRRK2//PTPRU//DIXDC1//TIAM1//LRRTM1//SCN3B//RBMS3//DAPK3//DLX5//NFKB1//CELF4//SH3GL1//CRTC1//NRGN//PAX8//GPNMB//EFNA3//FGF11//FGFR3//FSHB//TAC4//C1QA//CXCL14// |
| GO:0007399 | nervous system development | Biological process | 105 | 2296 | 509 | 17653 | 1.58605467439743 | 7.64128324185842e-07 | 0.00258389992823442 | 6.11683370188768 | 0.206286836935167 | FBXO45//SATB2//MARK1//ASCL1//FZD3//CCK//OTP//PHOX2A//FGFR2//RTN4//LRRN1//PARD6B//EFNA3//NCAM1//SPTBN2//CNTN2//VLDLR//SHANK3//UNC5A//DBNL//BSN//NPTX1//SH3GL1//PAX8//CTNS//FOXG1//GRIN2B//MED1//CA10//BCR//SNPH//SLC5A3//MMP24//SFRP1//ZNF804A//TRIM67//LRRK2//NF2//RFX4//NRGN//AQP1//PAX5//UCHL5//SYPL2//MAPKAP1//DIXDC1//RAX//DLX5//ALDH1A2//SLC6A3//SEMA7A//DAPK3//FZD2//WNT11//TTL//GNAO1//SMAD1//JAM3//NREP//UBE4B//NPTXR//NPTX2//TBC1D24//PRDM12//PLLP//GAL3ST1//KCNIP2//HEYL//KIT//TLX2//DGKG//SKIL//HS6ST1//SEMA5B//PPP3CB//RGMA//PACSIN1//CHRNA3//SEMA4G//ONECUT2//TIAM1//NFATC4//CUX1//LRRTM1//PALM//ABCC8//UNC13B//WDR19//PAK2//CTNND2//DLG4//ACTR2//SPRY3//CRTC1//ZSWIM6//SYT2//CHRM1//FGF11//KIF2A//NDP//NRN1//RBFOX1//SCN3B//PURA//SMPD1// |
| GO:0120036 | plasma membrane bounded cell projection organization | Biological process | 72 | 1444 | 509 | 17653 | 1.72928288045105 | 3.11492733191577e-06 | 0.00610615367847119 | 5.50655208053486 | 0.141453831041257 | FGFR2//RTN4//LRRN1//PARD6B//CCK//EFNA3//NCAM1//SPTBN2//CNTN2//VLDLR//SHANK3//UNC5A//ACTR3//ACTR2//UBXN10//EHD3//MCIDAS//PARVA//WDR19//RFX4//ONECUT2//SEPT7//WAS//BRK1//SFRP1//ZNF804A//TRIM67//LRRK2//FBXO45//ARFIP2//KIT//ARHGEF4//ARHGEF6//TTL//JAM3//NREP//UBE4B//NPTXR//GNAO1//NPTX2//TBC1D24//PRDM12//CDC42EP1//EMP2//CCDC103//FZD3//SEMA7A//SEMA5B//PPP3CB//RGMA//DBNL//PACSIN1//CHRNA3//SEMA4G//SKIL//TIAM1//TLX2//NFATC4//CUX1//PALM//NPTX1//PAK2//CTNND2//DLG4//SPRY3//DLX5//MAPRE1//CRTC1//EPS8L2//ZSWIM6//GRIN2B//SYT2// |
| GO:0030030 | cell projection organization | Biological process | 73 | 1477 | 509 | 17653 | 1.7141274260335 | 3.61150594616069e-06 | 0.00610615367847119 | 5.44231166575934 | 0.143418467583497 | FGFR2//RTN4//LRRN1//PARD6B//CCK//EFNA3//NCAM1//SPTBN2//CNTN2//VLDLR//SHANK3//UNC5A//ACTR3//ACTR2//UBXN10//EHD3//MCIDAS//PARVA//WDR19//RFX4//ONECUT2//SEPT7//WAS//BRK1//SFRP1//ZNF804A//TRIM67//LRRK2//FBXO45//ARFIP2//KIT//ARHGEF4//ARHGEF6//TTL//JAM3//NREP//UBE4B//NPTXR//GNAO1//NPTX2//TBC1D24//PRDM12//CDC42EP1//EMP2//CCDC103//FZD3//SEMA7A//SEMA5B//PPP3CB//RGMA//DBNL//PACSIN1//CHRNA3//SEMA4G//SKIL//TIAM1//TLX2//NFATC4//CUX1//PALM//NPTX1//PAK2//CTNND2//DLG4//SPRY3//DLX5//MAPRE1//CRTC1//EPS8L2//ZSWIM6//GRIN2B//SYT2//FAM92B// |
| GO:0030182 | neuron differentiation | Biological process | 66 | 1303 | 509 | 17653 | 1.75671074911003 | 5.04893683075687e-06 | 0.00630576277882959 | 5.29680006290034 | 0.129666011787819 | PHOX2A//ASCL1//FGFR2//RTN4//LRRN1//PARD6B//CCK//EFNA3//NCAM1//SPTBN2//CNTN2//VLDLR//SHANK3//UNC5A//SFRP1//ZNF804A//TRIM67//LRRK2//OTP//DLX5//SATB2//FBXO45//TTL//JAM3//NREP//UBE4B//NPTXR//GNAO1//NPTX2//TBC1D24//PRDM12//FZD3//KCNIP2//MED1//DIXDC1//HEYL//SEMA7A//DGKG//SKIL//HS6ST1//SEMA5B//PPP3CB//RGMA//DBNL//PACSIN1//CHRNA3//SEMA4G//ONECUT2//TIAM1//TLX2//NFATC4//CUX1//FZD2//NPTX1//PAK2//CTNND2//DLG4//ACTR2//SPRY3//CRTC1//ZSWIM6//SYT2//DAPK3//WNT11//ALDH1A2//SNPH// |
| GO:0023052 | signaling | Biological process | 242 | 6724 | 509 | 17653 | 1.24821213399733 | 6.47508830075761e-06 | 0.00630576277882959 | 5.18875430473392 | 0.475442043222004 | CAMKK2//LRRK2//DLG4//FGFR3//FGFR2//GRIN2B//IL1B//KIT//SMAD1//NCAM1//MAPK4//RAF1//SPTBN2//YWHAB//SHANK3//TAB1//PRKAA1//THBS1//PROK1//CD74//CD81//CCK//AGPAT1//MED1//TRIM5//CD209//NFKB1//PAK2//SKP1//TIRAP//UBE2D3//GPNMB//EDN1//EFNA3//FGF11//FSHB//TAC4//NDP//C1QA//CXCL14//WNT11//FERMT2//CTNND2//HIC1//TRABD2B//MARK1//CCDC88C//CALCOCO1//TNKS2//KREMEN1//VPS26A//FZD2//GNAO1//PPP3CB//FZD3//FAM13A//EBP//RALBP1//PRDM4//PTPRT//AKAP10//CHRM1//CHRNA3//DGKG//HTR3E//KCNIP3//KCNIP2//ARHGAP40//IL4R//IMPA2//LGALS3BP//MRC1//NRGN//CC2D1A//PRKAB1//HIVEP3//BCR//SH3GL1//RASL11B//SMPD1//ABCC8//TNFSF4//VLDLR//RERGL//NDFIP1//OGT//TNFSF14//CHRFAM7A//SH3BP5//SGK2//UNC13B//DAPK3//CARHSP1//HMOX1//ARHGEF4//POLR2M//PLA2G4C//OXSR1//CDKN1B//PML//PARP16//CLEC1A//TSPAN14//TSPAN18//SKIL//PTPRU//TPRA1//GPR160//GNAZ//GPR34//GRM2//PTAFR//SFRP1//TIAM1//ARHGEF6//GPR3//PALM//HEYL//ASCL1//EVC//ITGA5//TLN1//FERMT3//SEMA7A//REL//TRIB1//MAP3K9//DBNL//DDAH1//ARFIP2//RHOF//RALGPS2//CDKN2A//RASSF1//RALGDS//CDC42EP1//EPS8L2//SLC12A7//CPLX1//NOVA1//NPTX1//NPTX2//EXOC4//BSN//SNPH//P2RX2//SYT9//DPP4//SLC25A5//CACNA2D2//ATP6V1G2//GALNT3//POLR2E//PTBP1//UBE4B//NFATC4//SPRY3//PPP1R2//RGS9BP//BMP10//TTK//PIP4K2C//PIK3IP1//SYT2//SYT14//CPLX3//MGRN1//BRK1//NREP//LRRTM1//PIM1//JAM3//SCN1A//SCN7A//RFX4//FOXP3//WAS//SNX19//DLX5//RGMA//HTRA4//ONECUT2//NRIP1//CTH//EDEM3//CREB3L3//MAPKAP1//LARP1//IQSEC3//SIRT3//USP46//CA7//RABL3//INSIG2//RELT//WDFY1//ARHGDIB//TEAD1//NF2//VSNL1//KDELR3//ASNA1//GOSR2//UNC5A//ACTR3//ACTR2//PTPN18//PAX8//RNF125//S100A12//TGM2//RNF149//SHKBP1//ENHO//ZMIZ1//COPS5//TRIM67//CNTN2//SHISA9//ALDH1A2//CD79A//PAX5//RALGAPB//CGNL1//SLC6A3//RTN4//DIXDC1//TRIM68//TRIM21//WDR19//KCNJ12//CACNG1//SEMA4G//SCN3B//RBMS3//CELF4//LYPD6//CRTC1//LDOC1//EHD3//EMP2//LIMS2// |
| GO:0048666 | neuron development | Biological process | 56 | 1060 | 509 | 17653 | 1.83224228046113 | 8.43166561055021e-06 | 0.00630576277882959 | 5.07408662537305 | 0.110019646365422 | ASCL1//FGFR2//RTN4//LRRN1//PARD6B//CCK//EFNA3//NCAM1//SPTBN2//CNTN2//VLDLR//SHANK3//UNC5A//SFRP1//ZNF804A//TRIM67//LRRK2//FBXO45//TTL//JAM3//NREP//UBE4B//NPTXR//GNAO1//NPTX2//TBC1D24//PRDM12//FZD3//KCNIP2//SEMA7A//SEMA5B//PPP3CB//RGMA//DBNL//PACSIN1//CHRNA3//SEMA4G//ONECUT2//SKIL//TIAM1//TLX2//NFATC4//CUX1//FZD2//NPTX1//PAK2//CTNND2//DLG4//ACTR2//SPRY3//DLX5//CRTC1//ZSWIM6//SYT2//DGKG//HS6ST1// |
| GO:0048699 | generation of neurons | Biological process | 70 | 1437 | 509 | 17653 | 1.68943703661169 | 9.61541112898067e-06 | 0.00630576277882959 | 5.01703214177075 | 0.137524557956778 | FBXO45//SATB2//MARK1//ASCL1//FZD3//CCK//OTP//PHOX2A//FGFR2//RTN4//LRRN1//PARD6B//EFNA3//NCAM1//SPTBN2//CNTN2//VLDLR//SHANK3//UNC5A//SFRP1//ZNF804A//TRIM67//LRRK2//NF2//DLX5//DAPK3//FZD2//WNT11//ALDH1A2//SNPH//TTL//JAM3//NREP//UBE4B//NPTXR//GNAO1//NPTX2//TBC1D24//PRDM12//KCNIP2//MED1//DIXDC1//HEYL//SEMA7A//KIT//DGKG//SKIL//HS6ST1//SEMA5B//PPP3CB//RGMA//DBNL//PACSIN1//CHRNA3//SEMA4G//ONECUT2//TIAM1//TLX2//NFATC4//CUX1//ABCC8//NPTX1//PAK2//CTNND2//DLG4//ACTR2//SPRY3//CRTC1//ZSWIM6//SYT2// |
| GO:0007268 | chemical synaptic transmission | Biological process | 40 | 667 | 509 | 17653 | 2.07986380090898 | 1.05460919390222e-05 | 0.00630576277882959 | 4.97690844688318 | 0.0785854616895874 | UNC13B//CPLX1//SNPH//CHRM1//CHRNA3//CHRFAM7A//P2RX2//SYT2//SYT9//SYT14//CPLX3//USP46//CA7//EDN1//GRIN2B//NFATC4//PPP3CB//CNTN2//DLG4//KIT//SHANK3//SHISA9//IL1B//SLC6A3//LRRK2//GRM2//CACNA2D2//LRRTM1//CELF4//SH3GL1//CRTC1//NRGN//SLC12A7//HTR3E//KCNIP2//NOVA1//NPTX1//NPTX2//EXOC4//BSN// |
| GO:0098916 | anterograde trans-synaptic signaling | Biological process | 40 | 667 | 509 | 17653 | 2.07986380090898 | 1.05460919390222e-05 | 0.00630576277882959 | 4.97690844688318 | 0.0785854616895874 | UNC13B//SLC12A7//CPLX1//CHRNA3//DLG4//HTR3E//GRIN2B//GRM2//KCNIP2//NOVA1//NPTX1//NPTX2//EXOC4//BSN//CHRFAM7A//SNPH//CHRM1//P2RX2//SYT2//SYT9//SYT14//CPLX3//USP46//CA7//EDN1//NFATC4//PPP3CB//CNTN2//KIT//SHANK3//SHISA9//IL1B//SLC6A3//LRRK2//CACNA2D2//LRRTM1//CELF4//SH3GL1//CRTC1//NRGN// |
| GO:1905114 | cell surface receptor signaling pathway involved in cell-cell signaling | Biological process | 38 | 620 | 509 | 17653 | 2.12565435071931 | 1.08193664487572e-05 | 0.00630576277882959 | 4.96579816953116 | 0.0746561886051081 | WNT11//FERMT2//CTNND2//HIC1//TRABD2B//MARK1//CCDC88C//NDP//PRKAA1//CALCOCO1//SKP1//TNKS2//KREMEN1//VPS26A//FZD2//GNAO1//PPP3CB//FZD3//FOXP3//FGFR2//SFRP1//NFATC4//PTPRU//LRRK2//DIXDC1//TIAM1//CHRNA3//P2RX2//GRIN2B//RBMS3//DAPK3//DLX5//NFKB1//CELF4//UNC13B//SH3GL1//DLG4//SHANK3// |
| GO:0099537 | trans-synaptic signaling | Biological process | 40 | 670 | 509 | 17653 | 2.07055097791983 | 1.17082243995818e-05 | 0.00630576277882959 | 4.9315089624796 | 0.0785854616895874 | UNC13B//SLC12A7//CPLX1//CHRNA3//DLG4//HTR3E//GRIN2B//GRM2//KCNIP2//NOVA1//NPTX1//NPTX2//EXOC4//BSN//CHRFAM7A//SNPH//CHRM1//P2RX2//SYT2//SYT9//SYT14//CPLX3//USP46//CA7//EDN1//NFATC4//PPP3CB//CNTN2//KIT//SHANK3//SHISA9//IL1B//SLC6A3//LRRK2//CACNA2D2//LRRTM1//CELF4//SH3GL1//CRTC1//NRGN// |
| GO:0099536 | synaptic signaling | Biological process | 40 | 671 | 509 | 17653 | 2.06746520895125 | 1.2121087701432e-05 | 0.00630576277882959 | 4.91645840644578 | 0.0785854616895874 | UNC13B//SLC12A7//CPLX1//CHRNA3//DLG4//HTR3E//GRIN2B//GRM2//KCNIP2//NOVA1//NPTX1//NPTX2//EXOC4//BSN//CHRFAM7A//SNPH//CHRM1//P2RX2//SYT2//SYT9//SYT14//CPLX3//USP46//CA7//EDN1//NFATC4//PPP3CB//CNTN2//KIT//SHANK3//SHISA9//IL1B//SLC6A3//LRRK2//CACNA2D2//LRRTM1//CELF4//SH3GL1//CRTC1//NRGN// |
| GO:0007154 | cell communication | Biological process | 240 | 6744 | 509 | 17653 | 1.23422522705186 | 1.8503136044542e-05 | 0.00892337105706897 | 4.73275465800698 | 0.471512770137525 | CAMKK2//LRRK2//DLG4//FGFR3//FGFR2//GRIN2B//IL1B//KIT//SMAD1//NCAM1//MAPK4//RAF1//SPTBN2//YWHAB//SHANK3//TAB1//PRKAA1//THBS1//PROK1//CD74//CD81//CCK//AGPAT1//MED1//TRIM5//CD209//NFKB1//PAK2//SKP1//TIRAP//UBE2D3//GPNMB//EDN1//EFNA3//FGF11//FSHB//TAC4//NDP//C1QA//CXCL14//WNT11//FERMT2//CTNND2//HIC1//TRABD2B//MARK1//CCDC88C//CALCOCO1//TNKS2//KREMEN1//VPS26A//FZD2//GNAO1//PPP3CB//FZD3//FAM13A//EBP//RALBP1//PRDM4//PTPRT//AKAP10//CHRM1//CHRNA3//DGKG//HTR3E//KCNIP3//KCNIP2//ARHGAP40//IL4R//IMPA2//LGALS3BP//MRC1//NRGN//CC2D1A//PRKAB1//HIVEP3//BCR//SH3GL1//RASL11B//SMPD1//ABCC8//TNFSF4//VLDLR//RERGL//NDFIP1//OGT//TNFSF14//CHRFAM7A//SH3BP5//SGK2//UNC13B//DAPK3//CARHSP1//HMOX1//ARHGEF4//POLR2M//PLA2G4C//OXSR1//CDKN1B//PML//PARP16//CLEC1A//TSPAN14//TSPAN18//SKIL//PTPRU//TPRA1//GPR160//GNAZ//GPR34//GRM2//PTAFR//SFRP1//TIAM1//ARHGEF6//GPR3//PALM//HEYL//ASCL1//EVC//ITGA5//TLN1//FERMT3//SEMA7A//REL//TRIB1//MAP3K9//DBNL//DDAH1//ARFIP2//RHOF//RALGPS2//CDKN2A//RASSF1//RALGDS//CDC42EP1//EPS8L2//SLC12A7//CPLX1//NOVA1//NPTX1//NPTX2//EXOC4//BSN//SNPH//P2RX2//SYT9//DPP4//SLC25A5//CACNA2D2//ATP6V1G2//GALNT3//POLR2E//PTBP1//UBE4B//NFATC4//SPRY3//PPP1R2//RGS9BP//BMP10//TTK//PIP4K2C//PIK3IP1//SYT2//SYT14//CPLX3//MGRN1//BRK1//NREP//LRRTM1//PIM1//JAM3//SCN1A//SCN7A//RFX4//FOXP3//WAS//SNX19//DLX5//RGMA//HTRA4//ONECUT2//NRIP1//CTH//EDEM3//CREB3L3//MAPKAP1//FOSL1//LARP1//IQSEC3//SIRT3//USP46//CA7//RABL3//INSIG2//RELT//WDFY1//ARHGDIB//TEAD1//NF2//VSNL1//KDELR3//ASNA1//GOSR2//UNC5A//ACTR3//ACTR2//PTPN18//PAX8//RNF125//S100A12//TGM2//RNF149//SHKBP1//ENHO//ZMIZ1//COPS5//TRIM67//CNTN2//SHISA9//ALDH1A2//CD79A//PAX5//RALGAPB//CGNL1//SLC6A3//RTN4//DIXDC1//TRIM68//TRIM21//WDR19//SEMA4G//SCN3B//RBMS3//CELF4//LYPD6//CRTC1//LDOC1//EMP2//LIMS2// |
| GO:0031175 | neuron projection development | Biological process | 50 | 936 | 509 | 17653 | 1.85265645727335 | 1.97915963117011e-05 | 0.00892337105706897 | 4.70351917590961 | 0.0982318271119843 | FGFR2//RTN4//LRRN1//PARD6B//CCK//EFNA3//NCAM1//SPTBN2//CNTN2//VLDLR//SHANK3//UNC5A//SFRP1//ZNF804A//TRIM67//LRRK2//FBXO45//TTL//JAM3//NREP//FZD3//SEMA7A//SEMA5B//PPP3CB//RGMA//DBNL//PACSIN1//CHRNA3//SEMA4G//SKIL//TIAM1//TLX2//NFATC4//CUX1//NPTX1//PAK2//CTNND2//DLG4//ACTR2//SPRY3//DLX5//CRTC1//ZSWIM6//SYT2//UBE4B//NPTXR//GNAO1//NPTX2//TBC1D24//PRDM12// |
| GO:0050804 | modulation of chemical synaptic transmission | Biological process | 24 | 335 | 509 | 17653 | 2.4846611735038 | 4.29109052668606e-05 | 0.0162142184787004 | 4.36743232330804 | 0.0471512770137525 | UNC13B//CHRNA3//USP46//CA7//EDN1//CPLX1//CPLX3//GRIN2B//NFATC4//PPP3CB//CNTN2//DLG4//KIT//SHANK3//SHISA9//IL1B//LRRK2//GRM2//CACNA2D2//LRRTM1//CELF4//SH3GL1//CRTC1//NRGN// |
| GO:0099177 | regulation of trans-synaptic signaling | Biological process | 24 | 335 | 509 | 17653 | 2.4846611735038 | 4.29109052668606e-05 | 0.0162142184787004 | 4.36743232330804 | 0.0471512770137525 | UNC13B//CHRNA3//USP46//CA7//EDN1//CPLX1//CPLX3//GRIN2B//NFATC4//PPP3CB//CNTN2//DLG4//KIT//SHANK3//SHISA9//IL1B//LRRK2//GRM2//CACNA2D2//LRRTM1//CELF4//SH3GL1//CRTC1//NRGN// |
| GO:0022008 | neurogenesis | Biological process | 71 | 1536 | 509 | 17653 | 1.60312679068435 | 4.62087338708074e-05 | 0.0162142184787004 | 4.33527593107997 | 0.139489194499018 | FBXO45//SATB2//MARK1//ASCL1//FZD3//CCK//OTP//PHOX2A//FGFR2//RTN4//LRRN1//PARD6B//EFNA3//NCAM1//SPTBN2//CNTN2//VLDLR//SHANK3//UNC5A//MMP24//SFRP1//ZNF804A//TRIM67//LRRK2//NF2//DLX5//DAPK3//FZD2//WNT11//ALDH1A2//SNPH//TTL//JAM3//NREP//UBE4B//NPTXR//GNAO1//NPTX2//TBC1D24//PRDM12//KCNIP2//MED1//DIXDC1//HEYL//SEMA7A//KIT//DGKG//SKIL//HS6ST1//SEMA5B//PPP3CB//RGMA//DBNL//PACSIN1//CHRNA3//SEMA4G//ONECUT2//TIAM1//TLX2//NFATC4//CUX1//ABCC8//NPTX1//PAK2//CTNND2//DLG4//ACTR2//SPRY3//CRTC1//ZSWIM6//SYT2// |
| GO:0048522 | positive regulation of cellular process | Biological process | 189 | 5142 | 509 | 17653 | 1.27476599734533 | 4.79457006779724e-05 | 0.0162142184787004 | 4.3192503302148 | 0.371316306483301 | TAB1//LRRK2//RAF1//IL1B//KIT//PRKAA1//THBS1//PROK1//CD74//CD81//GPNMB//CAMKK2//AGPAT1//OTP//FZD3//FGFR2//PML//CCK//CDKN1B//PARP16//CHRNA3//HEYL//TIRAP//MAP3K9//DBNL//DDX39B//CHRM1//DPP4//EDN1//EMP2//FGFR3//FSHB//SLC25A5//PGGT1B//BRK1//PURA//SFRP1//TIAM1//TTK//TNFSF4//FOSL1//ALDH1A2//CASP7//TP53INP2//TRIM21//P2RX2//RTN4//MICAL2//NFKB1//UNC13B//TNFSF14//BMP10//ABHD5//CNTN2//TRIM67//ZNF804A//LARP1//RBMS3//PTAFR//RBM3//HMOX1//TAF8//DLX5//ELK1//ZNF281//FZD2//MYBL2//NDP//NFYA//FOXP3//MED1//PPP3CB//KMT2E//CALCOCO1//HIVEP3//GPBP1L1//TEAD1//WNT11//PAX8//NRIP1//CREB5//EIF4G2//DAPK3//MAPRE1//ITGA5//SEMA4G//FERMT3//SEMA7A//ONECUT2//CDC42EP1//LMOD1//PAXBP1//PAK2//NDFIP1//SIRT3//OXSR1//MAPKAP1//NEK7//TNKS2//CA7//TRIB1//RNF217//RNF125//RNF114//TRABD2B//INSIG2//WDFY1//ACTR3//ACTR2//ARFIP2//CDKN2A//SMPD1//VSNL1//CD209//IL4R//ARHGEF4//UBE2Z//TGM2//ARHGEF6//CTH//CC2D1A//REL//S100A12//TRIM5//HIC1//ASCL1//DDAH1//ZMIZ1//RAG1//NF2//SMAD1//SHKBP1//ENHO//TSPAN14//DAZL//VLDLR//OGT//EVC//TTL//MED6//ARID3B//COPS5//PRDM4//TET3//SATB2//CRTC1//ATP1B4//RAX//TLX2//MCIDAS//PHOX2A//NFATC4//PAX5//RGMA//RFX4//HLTF//TCF20//CREB3L3//SYT9//CELF4//AQP1//SHANK3//BCR//SKIL//CUX1//DLG4//WAS//PALM//PRDM12//LRRTM1//LRRN1//PIM1//GRIN2B//DIXDC1//PACSIN1//EPS8L2//LSM11//NRGN//YWHAB//TAC4//SEPT7//EFNA3//ABCC8//SYT2//UBE2D3//FAM168A//CXCL14//PIP4K2C//LIMS2// |
| GO:0032879 | regulation of localization | Biological process | 111 | 2700 | 509 | 17653 | 1.42580440951757 | 4.79497810992175e-05 | 0.0162142184787004 | 4.31921337113396 | 0.218074656188605 | NKAIN1//DLG4//LRRTM1//HMOX1//BCR//CD74//EDN1//SYT9//DPP4//SLC25A5//IL1B//PPP3CB//ABCC8//CACNA2D2//AQP1//PML//P2RX2//EMP2//THBS1//VASH1//BMP10//RTN4//SCN3B//PRSS8//UNC13B//TNFSF14//RSC1A1//MEST//NFKB1//ABHD5//CCK//CHRNA3//TRIB1//CPLX1//LGI3//SYT2//SYT14//PACSIN1//KIF2A//RHOF//BEX4//PARD6B//GPNMB//DAPK3//MAPRE1//FSHB//ITGA5//KIT//SEMA4G//TIAM1//WNT11//FERMT3//SEMA7A//ONECUT2//PTPRU//NF2//SFRP1//SIRT3//CYP4F2//REEP2//NDFIP1//SGK2//TRIM5//LRRK2//MED1//KCNA6//KCNJ12//KCNQ5//SCN1A//SCN7A//CACNG1//KCNQ4//VSNL1//CHRM1//IL4R//PTAFR//NUP214//CPLX3//FOXP3//TNFSF4//CDKN1B//BEST1//GNAO1//PRKAA1//SLC30A6//JAM3//TIRAP//TTL//SH3GL1//YWHAB//OXSR1//ARHGDIB//KCNIP3//KCNIP2//EHD3//TAC4//SLMAP//ZSWIM6//NUCB1//TP53INP2//UBE2D3//SESTD1//RAF1//CD81//WAS//SHISA9//SHANK3//CXCL14//PAX8//GLRX//CA7// |
| GO:0048856 | anatomical structure development | Biological process | 210 | 5836 | 509 | 17653 | 1.24797173831957 | 5.23667548660812e-05 | 0.0168645887218718 | 4.28094433821272 | 0.412573673870334 | MED1//WDR19//EBP//DLX5//EVC//FGFR3//FGFR2//VASH1//HMOX1//ITGA5//RTN4//JAM3//PROK1//HS6ST1//KIT//NRIP1//DAZL//FOXS1//ALDH1A2//EDN1//NFATC4//ZMIZ1//PAX8//SMAD1//SFRP1//TAB1//BRK1//FOSL1//TLX2//NF2//FZD3//SEMA4G//SEMA7A//FBXO45//SATB2//MARK1//ASCL1//CCK//SKIL//TAF8//WNT11//DDX39B//ONECUT2//CDKN1B//NDP//LDOC1//PPP3CB//CCDC103//MICAL2//PARVA//THBS1//OTP//SNX19//CTNS//RAG1//FOXP3//NDFIP1//FZD2//HEYL//UBE4B//KRT2//PHOX2A//SPRY3//CUX1//LCLAT1//GRIN2B//HIC1//HMGB3//HOXC6//ARHGDIB//PAX5//PIM1//ASF1B//MBNL3//RNF114//RAF1//HIVEP3//RELT//TMEFF1//CELF4//FOXG1//FOXI2//DLG4//RAX//CHRM1//CHRNA3//FGF11//KIF2A//NRGN//NRN1//RBFOX1//SCN3B//PURA//SMPD1//VLDLR//LRRN1//PARD6B//EFNA3//NCAM1//SPTBN2//CNTN2//SHANK3//UNC5A//DBNL//BSN//NPTX1//SH3GL1//CA10//BCR//SNPH//SLC5A3//MEST//BMP10//PAXBP1//EMP2//IL1B//CSN3//SLC6A3//LRRK2//FERMT2//CDC42EP1//DAPK3//PALM//RHOF//WAS//KRT9//EVPLL//RNF38//MEA1//HOXC9//MMP24//ZNF281//TIAM1//ZNF804A//TRIM67//SEPT7//PML//ABCC8//RFX4//AQP1//UCHL5//SYPL2//MAPKAP1//DIXDC1//TIRAP//CD79A//KMT2E//LMOD1//MYOM3//GPNMB//TTL//ALDOC//GNAO1//PTPRU//NREP//NPTXR//NPTX2//TBC1D24//PRDM12//KRTAP1-1//KRT38//FERMT3//PRKAB1//TPRA1//INSIG2//KCNQ4//PLLP//GAL3ST1//FSHB//GPD2//MAEA//TNFSF4//SLC27A4//TET3//CD74//KCNIP2//IL4R//TRIB1//CXCL14//TNFSF14//DDAH1//EXOC4//TEAD1//C2ORF49//PRKAA1//DGKG//SEMA5B//RGMA//P2RX2//C11ORF88//CACNA2D2//PACSIN1//PTBP1//LRRTM1//KREMEN1//CRISPLD1//UNC13B//TGM2//PAK2//CTNND2//ACTR2//PRSS8//NPHS2//MYO19//CRTC1//ZSWIM6//MCIDAS//SYT2//CTH//LIMS2// |
| GO:0007166 | cell surface receptor signaling pathway | Biological process | 121 | 3016 | 509 | 17653 | 1.39140888411771 | 5.90597377069485e-05 | 0.0181555002778224 | 4.22870848613284 | 0.237721021611002 | AGPAT1//CD74//TAB1//CD209//NFKB1//PAK2//RAF1//SKP1//WNT11//FERMT2//CTNND2//HIC1//TRABD2B//MARK1//CCDC88C//NDP//PRKAA1//CALCOCO1//TNKS2//KREMEN1//VPS26A//FZD2//GNAO1//PPP3CB//FZD3//PTPRT//CHRNA3//FSHB//SMAD1//PML//SKIL//PTPRU//GRM2//GRIN2B//CDKN1B//HEYL//ASCL1//EVC//ITGA5//TLN1//FERMT3//SEMA7A//ATP6V1G2//FGFR3//FGFR2//GALNT3//POLR2E//PTBP1//BMP10//TTK//NREP//HMOX1//LRRTM1//IL1B//IL4R//KIT//PIM1//PTAFR//RFX4//FOXP3//WAS//SFRP1//NFATC4//DLX5//RGMA//UBE2D3//THBS1//HTRA4//RASL11B//ONECUT2//TRIM5//TRIB1//TNFSF4//RELT//TNFSF14//GPR34//P2RX2//UNC5A//VLDLR//ACTR3//ACTR2//BRK1//PTPN18//SPRY3//SHKBP1//ENHO//ZMIZ1//TSPAN14//MGRN1//OGT//BCR//EFNA3//TIAM1//CD79A//PAX5//LRRK2//DIXDC1//CNTN2//NCAM1//TRIM68//TRIM21//MED1//WDR19//EDN1//SEMA4G//RBMS3//DAPK3//CELF4//UNC13B//SH3GL1//SHANK3//RTN4//DLG4//SHISA9//EMP2//LIMS2//TIRAP//CLEC1A//MAPK4//TSPAN18//CD81// |
| GO:0006811 | ion transport | Biological process | 73 | 1610 | 509 | 17653 | 1.57252559518725 | 6.63045802335018e-05 | 0.0194964293964858 | 4.17845647006537 | 0.143418467583497 | SLC27A4//EDN1//NKAIN1//P2RX2//CDKN1B//KCNIP2//AQP1//KCNA6//KCNJ12//ABCC8//KCNQ4//SCN3B//SCN1A//SCN7A//SLC5A3//SLC10A7//SLC5A6//SLC23A2//SLC26A1//ANO4//BEST1//NDFIP1//SLC39A2//SLC39A8//SLC12A7//ATP1B4//PML//THBS1//PRSS8//UNC13B//CPLX1//GRM2//CCK//CHRNA3//STEAP2//ASNA1//CA7//CTNS//SLC25A5//SLC6A3//SLC25A2//PCTP//SLC52A2//CYP4F2//IL1B//ATP6V1G2//SGK2//DLG4//HTR3E//RAF1//ANO3//CHRFAM7A//KCNQ5//CACNG1//CACNA2D2//CHRM1//GNAO1//SLC30A6//KCNIP3//SLC9A5//SLC35A2//GRIN2B//RALBP1//OXSR1//EHD3//SLMAP//PTAFR//SESTD1//SHISA9//SHANK3//GLRX//PLLP//TOMM40L// |
| GO:0010557 | positive regulation of macromolecule biosynthetic process | Biological process | 76 | 1698 | 509 | 17653 | 1.55230353056063 | 7.03678032361867e-05 | 0.0198290605535971 | 4.15262600670024 | 0.149312377210216 | HEYL//DDX39B//MICAL2//EDN1//LARP1//RBMS3//PTAFR//RBM3//THBS1//CAMKK2//TAF8//DLX5//ELK1//ZNF281//FZD2//BMP10//IL1B//MYBL2//NDP//NFKB1//NFYA//FOXP3//MED1//PPP3CB//KMT2E//CALCOCO1//TP53INP2//HIVEP3//GPBP1L1//SFRP1//TEAD1//WNT11//PAX8//NRIP1//CREB5//CD74//NEK7//TNKS2//INSIG2//HMOX1//PML//TIRAP//MED6//ARID3B//COPS5//PRDM4//TET3//FGFR2//SATB2//CRTC1//ATP1B4//FSHB//RAX//TLX2//MCIDAS//PHOX2A//SMAD1//ASCL1//NFATC4//PAX5//RGMA//ZMIZ1//RAF1//REL//RFX4//HLTF//TCF20//FOSL1//CREB3L3//OGT//PAXBP1//ONECUT2//CD81//DAZL//LRRK2//PRKAA1// |
| GO:0048731 | system development | Biological process | 176 | 4760 | 509 | 17653 | 1.28234963926632 | 7.5830681662554e-05 | 0.0200249661918283 | 4.12015504000506 | 0.345776031434185 | EBP//DLX5//EVC//FGFR3//FGFR2//VASH1//HMOX1//ITGA5//MED1//RTN4//JAM3//PROK1//HS6ST1//KIT//NRIP1//FOXS1//ALDH1A2//EDN1//NFATC4//ZMIZ1//PAX8//SMAD1//SFRP1//SEMA4G//SEMA7A//FBXO45//SATB2//MARK1//ASCL1//FZD3//CCK//WNT11//DDX39B//ONECUT2//CDKN1B//NDP//LDOC1//PPP3CB//CCDC103//MICAL2//PARVA//THBS1//OTP//SNX19//CTNS//RAG1//FOXP3//NDFIP1//TAB1//FZD2//HEYL//UBE4B//KRT2//PHOX2A//PAX5//CHRM1//CHRNA3//DLG4//FGF11//KIF2A//NRGN//NRN1//RBFOX1//SCN3B//PURA//SMPD1//VLDLR//LRRN1//PARD6B//EFNA3//NCAM1//SPTBN2//CNTN2//SHANK3//UNC5A//DBNL//BSN//NPTX1//SH3GL1//FOXG1//GRIN2B//CA10//BCR//SNPH//SLC5A3//RAF1//BMP10//PAXBP1//CSN3//SLC6A3//WDR19//RNF38//MEA1//MMP24//TIAM1//IL1B//ZNF804A//TRIM67//LRRK2//NF2//PML//ABCC8//RFX4//AQP1//UCHL5//SYPL2//MAPKAP1//DIXDC1//RAX//TIRAP//DAPK3//CD79A//KMT2E//GPNMB//TTL//GNAO1//PTPRU//NREP//NPTXR//NPTX2//TBC1D24//PRDM12//KRT9//KRTAP1-1//KRT38//HIVEP3//INSIG2//KCNQ4//PLLP//GAL3ST1//FSHB//GPD2//MAEA//TNFSF4//SLC27A4//CD74//KCNIP2//HMGB3//IL4R//TRIB1//EMP2//DDAH1//EXOC4//TLX2//SPRY3//TEAD1//PRKAA1//DGKG//SKIL//SEMA5B//RGMA//HOXC9//HOXC6//MYOM3//P2RX2//C11ORF88//PACSIN1//CXCL14//CUX1//LRRTM1//PIM1//PALM//UNC13B//TGM2//FOSL1//PAK2//CTNND2//ACTR2//PRSS8//NPHS2//CRTC1//ZSWIM6//CELF4//SYT2//CTH//LIMS2// |
| GO:0048518 | positive regulation of biological process | Biological process | 209 | 5841 | 509 | 17653 | 1.24096581680412 | 8.07599245938796e-05 | 0.0200249661918283 | 4.09280409523208 | 0.410609037328094 | TAB1//LRRK2//RAF1//IL1B//KIT//PRKAA1//THBS1//PROK1//CD74//CD81//AGPAT1//TNFSF4//GPNMB//CAMKK2//EMP2//SFRP1//OTP//FZD3//FGFR2//TRIM5//CD209//NFKB1//PAK2//SKP1//IL4R//FOXP3//CREB3L3//TIRAP//UBE2D3//EDN1//C1QA//PML//CCK//VSIG4//CDKN1B//PARP16//CHRNA3//HEYL//MAP3K9//DBNL//DDX39B//CHRM1//DPP4//FGFR3//FSHB//SLC25A5//PGGT1B//BRK1//PURA//TIAM1//TTK//FOSL1//ALDH1A2//CASP7//AQP1//PYURF//SCN3B//TP53INP2//TRIM21//P2RX2//BMP10//RBMS3//SMAD1//MED1//PRKAB1//CALCOCO1//WNT11//ZNF804A//RTN4//MICAL2//PRSS8//UNC13B//ITGA5//TNFSF14//ABHD5//CNTN2//TRIM67//LARP1//PTAFR//RBM3//HMOX1//TAF8//DLX5//ELK1//ZNF281//FZD2//MYBL2//NDP//NFYA//PPP3CB//KMT2E//HIVEP3//GPBP1L1//TEAD1//PAX8//NRIP1//CREB5//WAS//EIF4G2//DAPK3//MAPRE1//SEMA4G//FERMT3//SEMA7A//ONECUT2//CDC42EP1//LMOD1//PAXBP1//NDFIP1//SIRT3//OXSR1//MAPKAP1//NEK7//TNKS2//CA7//CYP4F2//SGK2//TRIB1//RNF217//RNF125//RNF114//TRABD2B//POLR2E//RNF135//NFATC4//ABCC8//CRTC1//INSIG2//WDFY1//ACTR3//ACTR2//ARFIP2//CDKN2A//SMPD1//VSNL1//FOXS1//SLC6A3//ARHGEF4//UBE2Z//TGM2//ARHGEF6//CTH//CC2D1A//REL//S100A12//HIC1//ASCL1//PPIH//DDAH1//ZMIZ1//RAG1//NF2//SHKBP1//ENHO//TSPAN14//DAZL//VLDLR//OGT//EVC//TTL//MED6//ARID3B//COPS5//PRDM4//TET3//SATB2//ATP1B4//RAX//TLX2//MCIDAS//PHOX2A//PAX5//RGMA//RFX4//HLTF//TCF20//SYT9//TMPRSS2//CACNA2D2//CELF4//SHANK3//BCR//SKIL//CUX1//DLG4//CD79A//PALM//PRDM12//LRRTM1//LRRN1//PIM1//GRIN2B//DIXDC1//PACSIN1//EPS8L2//LSM11//NRGN//YWHAB//EHD3//TAC4//ZNF322//SEPT7//EFNA3//SYT2//FAM168A//CXCL14//GLRX//PIP4K2C//LIMS2// |
| GO:0022603 | regulation of anatomical structure morphogenesis | Biological process | 55 | 1123 | 509 | 17653 | 1.69857087124545 | 8.19114750192116e-05 | 0.0200249661918283 | 4.08665525344796 | 0.108055009823183 | WNT11//FERMT2//CDC42EP1//DAPK3//KIT//PALM//RHOF//PARVA//WAS//SEPT7//EFNA3//VASH1//PML//ABCC8//THBS1//TTL//RTN4//CNTN2//EDN1//GPNMB//EMP2//HMOX1//SFRP1//PROK1//DDAH1//IL1B//AQP1//SEMA7A//CHRNA3//SEMA4G//SKIL//TIAM1//TLX2//NFATC4//CUX1//FZD2//FZD3//BMP10//BCR//FGFR2//LRRK2//SHANK3//ACTR2//PAX8//MYO19//DLG4//IL4R//TNFSF14//ZSWIM6//ITGA5//SMAD1//SYT2//PIM1//LIMS2//MED1// |
| GO:0007275 | multicellular organism development | Biological process | 194 | 5351 | 509 | 17653 | 1.25738280746599 | 8.29068539658719e-05 | 0.0200249661918283 | 4.08140956454606 | 0.381139489194499 | EBP//DLX5//EVC//FGFR3//FGFR2//VASH1//HMOX1//ITGA5//MED1//RTN4//JAM3//PROK1//HS6ST1//KIT//NRIP1//FOXS1//ALDH1A2//EDN1//NFATC4//ZMIZ1//PAX8//SMAD1//SFRP1//TAB1//BRK1//WDR19//FOSL1//TLX2//NF2//SEMA4G//SEMA7A//FBXO45//SATB2//MARK1//ASCL1//FZD3//CCK//SKIL//TAF8//WNT11//DDX39B//ONECUT2//CDKN1B//NDP//LDOC1//PPP3CB//CCDC103//MICAL2//PARVA//THBS1//OTP//SNX19//CTNS//RAG1//FOXP3//NDFIP1//FZD2//HEYL//UBE4B//KRT2//PHOX2A//DLG4//RAX//PAX5//CHRM1//CHRNA3//FGF11//KIF2A//NRGN//NRN1//RBFOX1//SCN3B//PURA//SMPD1//VLDLR//LRRN1//PARD6B//EFNA3//NCAM1//SPTBN2//CNTN2//SHANK3//UNC5A//DBNL//BSN//NPTX1//SH3GL1//FOXG1//GRIN2B//CA10//BCR//SNPH//SLC5A3//RAF1//BMP10//PAXBP1//EMP2//IL1B//CSN3//SLC6A3//LRRK2//RNF38//MEA1//CELF4//HOXC6//HOXC9//MMP24//ZNF281//TIAM1//ZNF804A//TRIM67//SEPT7//PML//ABCC8//RFX4//AQP1//UCHL5//SYPL2//MAPKAP1//DIXDC1//TIRAP//DAPK3//CD79A//KMT2E//GPNMB//TTL//GNAO1//PTPRU//NREP//NPTXR//NPTX2//TBC1D24//PRDM12//KRT9//KRTAP1-1//KRT38//PRKAB1//HIVEP3//TPRA1//INSIG2//KCNQ4//PLLP//GAL3ST1//FSHB//GPD2//MAEA//TNFSF4//SLC27A4//TET3//CD74//KCNIP2//HMGB3//IL4R//TRIB1//DDAH1//EXOC4//SPRY3//TEAD1//C2ORF49//PRKAA1//DGKG//SEMA5B//RGMA//MYOM3//P2RX2//C11ORF88//PACSIN1//CXCL14//CUX1//LRRTM1//PIM1//PALM//KREMEN1//UNC13B//TGM2//PAK2//CTNND2//ACTR2//PRSS8//NPHS2//CRTC1//ARHGDIB//ZSWIM6//SYT2//CTH//LIMS2//DAZL//LCLAT1//HIC1//ASF1B//MBNL3//RNF114//RELT//TMEFF1// |
| GO:0065008 | regulation of biological quality | Biological process | 144 | 3770 | 509 | 17653 | 1.32471325165587 | 0.000106372014278745 | 0.0248066873299018 | 3.97317261680595 | 0.282907662082515 | SLC6A3//IL1B//ABCC8//JAM3//FOXP3//TP53INP2//SKIL//CD74//P2RX2//CHRM1//DDAH1//EDN1//AQP1//CYP4F2//EMP2//AKAP10//VPS45//EHD3//PAFAH2//WAS//GLRX//MED1//KDELR3//FSHB//SYPL2//LETM2//HMOX1//SKP1//NDFIP1//SLC39A8//ATP1B4//SLC12A7//TLN1//DLG4//TAC4//UNC13B//CPLX1//SNPH//CSN3//SYT9//DPP4//SLC25A5//PPP3CB//CACNA2D2//BRK1//FERMT2//CDC42EP1//DAPK3//KIT//PALM//RHOF//PARVA//PTAFR//SCN3B//PML//MEST//NFKB1//ABHD5//CTNS//CHRNA3//SYT2//SYT14//CPLX3//SEPT7//RTN4//SCN1A//SCN7A//CHDH//DPYS//NRIP1//SNX19//DGKG//RAF1//THBS1//KMT2E//TTL//ARHGAP40//EPS8L2//LMOD1//CNTN2//RNF149//NF2//CD81//FBXL3//CCDC88C//TRIM21//SIRT3//CCK//NEK7//TNKS2//TGM2//CA7//ACTR3//ACTR2//ARFIP2//LRRK2//VSNL1//PRIMA1//SLC26A1//HTR3E//CHRFAM7A//ALDH1A2//SMAD1//PRKAA1//RAG1//TNFSF14//BCR//MAEA//CARHSP1//YWHAB//NUP214//SEMA7A//SHANK3//SFRP1//GRIN2B//NFATC4//SHISA9//LARP1//SEMA4G//SLC9A5//TAF8//POLR2M//SPTBN2//LRRTM1//LRRN1//STEAP2//TIAM1//SLC30A6//ANO4//ANO3//HAAO//FERMT3//TIRAP//DAZL//KCNIP2//ATP6V1G2//CELF4//CRTC1//SH3GL1//GRIPAP1//NRGN//SLMAP//ZNF804A//PAX8// |
| GO:0032502 | developmental process | Biological process | 221 | 6267 | 509 | 17653 | 1.22301932065019 | 0.000110199718594634 | 0.0248426898951837 | 3.9578195144947 | 0.434184675834971 | MED1//WDR19//EBP//DLX5//EVC//FGFR3//FGFR2//VASH1//HMOX1//ITGA5//RTN4//JAM3//PROK1//HS6ST1//KIT//NRIP1//DAZL//FOXS1//ALDH1A2//EDN1//NFATC4//ZMIZ1//GPNMB//TP53INP2//SFRP1//WNT11//SEMA7A//PAX8//SMAD1//TAB1//BRK1//FOSL1//TLX2//NF2//FZD3//SEMA4G//FBXO45//SATB2//MARK1//ASCL1//CCK//SKIL//TAF8//DDX39B//ONECUT2//CDKN1B//NDP//LDOC1//PPP3CB//CCDC103//MICAL2//PARVA//THBS1//OTP//SNX19//CTNS//RAG1//FOXP3//NDFIP1//FZD2//HEYL//UBE4B//KRT2//PHOX2A//SPRY3//CUX1//LCLAT1//GRIN2B//HIC1//HMGB3//HOXC6//ARHGDIB//PAX5//PIM1//ASF1B//MBNL3//RNF114//RAF1//HIVEP3//RELT//TMEFF1//CELF4//FOXG1//FOXI2//DLG4//RAX//CHRM1//CHRNA3//FGF11//KIF2A//NRGN//NRN1//RBFOX1//SCN3B//PURA//SMPD1//VLDLR//LRRN1//PARD6B//EFNA3//NCAM1//SPTBN2//CNTN2//SHANK3//UNC5A//DBNL//BSN//NPTX1//SH3GL1//CA10//BCR//SNPH//SLC5A3//MEST//BMP10//PAXBP1//EMP2//IL1B//TPRA1//SIRT3//GNAO1//SLC6A3//CSN3//LRRK2//FERMT2//CDC42EP1//DAPK3//PALM//RHOF//WAS//KRT9//EVPLL//RNF38//MEA1//HOXC9//MMP24//ZNF281//TIAM1//NFKB1//ZNF804A//TRIM67//UNC13B//SEPT7//PML//ABCC8//RFX4//AQP1//UCHL5//SYPL2//MAPKAP1//DIXDC1//TIRAP//PTPRU//ELK1//MYBL2//ABHD5//CD79A//KMT2E//LMOD1//MYOM3//TTL//ALDOC//NREP//NPTXR//NPTX2//TBC1D24//PRDM12//KRTAP1-1//KRT38//FERMT3//POLR2E//GPD2//TNKS2//PRKAB1//CACNA2D2//INSIG2//KCNQ4//PLLP//GAL3ST1//FSHB//MAEA//TNFSF4//SLC27A4//TET3//CD74//KCNIP2//FAM57B//IL4R//TRIB1//CXCL14//TNFSF14//DDAH1//EXOC4//TEAD1//C2ORF49//PRKAA1//DGKG//SEMA5B//RGMA//P2RX2//C11ORF88//PACSIN1//PTBP1//LRRTM1//KREMEN1//CRISPLD1//TGM2//PAK2//CTNND2//ACTR2//PRSS8//NPHS2//MYO19//CDKN2A//CRTC1//ZNF322//ZSWIM6//MCIDAS//SYT2//CTH//LIMS2// |
| GO:0043269 | regulation of ion transport | Biological process | 35 | 622 | 509 | 17653 | 1.95154422959084 | 0.000134327207129464 | 0.0293049968327924 | 3.87183601481084 | 0.068762278978389 | NKAIN1//PML//P2RX2//THBS1//SCN3B//PRSS8//CCK//CHRNA3//CYP4F2//EDN1//IL1B//KCNA6//KCNJ12//KCNQ5//SCN1A//SCN7A//CACNG1//KCNQ4//CACNA2D2//CHRM1//BEST1//GNAO1//OXSR1//KCNIP3//KCNIP2//EHD3//SLMAP//PTAFR//ABCC8//SESTD1//DLG4//SHISA9//SHANK3//GLRX//CA7// |
| GO:0016055 | Wnt signaling pathway | Biological process | 30 | 504 | 509 | 17653 | 2.06438862381888 | 0.000154083061421365 | 0.0318950125359629 | 3.81224510129173 | 0.0589390962671906 | WNT11//FZD2//GNAO1//PPP3CB//FZD3//FOXP3//FGFR2//SFRP1//HIC1//TRABD2B//NFATC4//PTPRU//LRRK2//NDP//DIXDC1//TIAM1//CTNND2//KREMEN1//RBMS3//DAPK3//DLX5//NFKB1//TNKS2//FERMT2//MARK1//CCDC88C//PRKAA1//CALCOCO1//SKP1//VPS26A// |
| GO:0048812 | neuron projection morphogenesis | Biological process | 35 | 628 | 509 | 17653 | 1.93289890255653 | 0.00016157066159665 | 0.0318950125359629 | 3.79163749667672 | 0.068762278978389 | FGFR2//RTN4//LRRN1//PARD6B//CCK//EFNA3//NCAM1//SPTBN2//CNTN2//VLDLR//SHANK3//UNC5A//FBXO45//TTL//FZD3//SEMA7A//SEMA5B//PPP3CB//CHRNA3//SEMA4G//SKIL//TIAM1//TLX2//NFATC4//CUX1//NPTX1//CTNND2//DLG4//LRRK2//ACTR2//DLX5//ZSWIM6//SYT2//DBNL//PACSIN1// |
| GO:0010646 | regulation of cell communication | Biological process | 135 | 3522 | 509 | 17653 | 1.32936780205032 | 0.000164518875478295 | 0.0318950125359629 | 3.7837842676484 | 0.265225933202358 | TAB1//LRRK2//RAF1//IL1B//KIT//PRKAA1//THBS1//PROK1//CD74//CD81//AGPAT1//TIRAP//MAP3K9//DBNL//SYT9//DPP4//SLC25A5//PPP3CB//ABCC8//CACNA2D2//FGFR2//SPRY3//PPP1R2//FGFR3//RGS9BP//GPNMB//EDN1//FGF11//FSHB//BMP10//NDP//TNFSF4//TNFSF14//CCK//CXCL14//UNC13B//TTK//CHRNA3//PIP4K2C//PIK3IP1//MGRN1//YWHAB//NREP//RFX4//SMPD1//FOXP3//SFRP1//HIC1//TRABD2B//NFATC4//HTRA4//SKIL//RASL11B//ONECUT2//ITGA5//TRIM5//TRIB1//IQSEC3//SIRT3//USP46//CA7//RALGPS2//MED1//WDFY1//OGT//ARHGDIB//ARHGEF4//BCR//EPS8L2//TIAM1//ARHGEF6//NF2//VSNL1//RNF125//CTH//HMOX1//CC2D1A//REL//S100A12//TGM2//NDFIP1//PAK2//RNF149//SHKBP1//ENHO//ASCL1//ZMIZ1//TSPAN14//EVC//COPS5//LRRTM1//TRIM67//MAPKAP1//CPLX1//CPLX3//GRIN2B//CNTN2//DLG4//SHANK3//SHISA9//P2RX2//PAX5//FAM13A//RALBP1//ARHGAP40//RHOF//RALGAPB//CGNL1//RTN4//GRM2//PALM//TRIM68//HEYL//CTNND2//KREMEN1//SEMA7A//WNT11//RBMS3//DAPK3//DLX5//NFKB1//TNKS2//DIXDC1//CELF4//SH3GL1//LYPD6//CRTC1//NRGN//PTPN18//PML//PRKAB1//LDOC1//PAX8//EMP2//LIMS2// |
| GO:0198738 | cell-cell signaling by wnt | Biological process | 30 | 506 | 509 | 17653 | 2.05622898498956 | 0.000165063646127266 | 0.0318950125359629 | 3.78234856589417 | 0.0589390962671906 | WNT11//FERMT2//CTNND2//HIC1//TRABD2B//MARK1//CCDC88C//NDP//PRKAA1//CALCOCO1//SKP1//TNKS2//KREMEN1//VPS26A//FZD2//GNAO1//PPP3CB//FZD3//FOXP3//FGFR2//SFRP1//NFATC4//PTPRU//LRRK2//DIXDC1//TIAM1//RBMS3//DAPK3//DLX5//NFKB1// |
| GO:0065007 | biological regulation | Biological process | 392 | 12351 | 509 | 17653 | 1.1007398365332 | 0.000192496649067681 | 0.0345111495640306 | 3.71557682617669 | 0.770137524557957 | CDKN1B//CUX1//EDN1//FGFR2//FOXS1//SATB2//ZNF281//DNAJB5//HEYL//KCNIP3//HIC1//ASCL1//NFKB1//PAX5//FOXP3//RLIM//MED1//CC2D1A//REL//SKIL//UBE2D3//VLDLR//NRIP1//BHLHE40//PAXBP1//CAMKK2//LRRK2//DLG4//FGFR3//GRIN2B//IL1B//KIT//SMAD1//NCAM1//MAPK4//RAF1//SPTBN2//YWHAB//SHANK3//RBM8A//TAB1//PRKAA1//THBS1//PROK1//CD74//CD81//RBFOX1//MBNL3//CELF4//PTBP1//SLC6A3//SGK2//HTRA4//ABCC8//JAM3//GPNMB//AGPAT1//TNFSF4//CCK//EHD3//TP53INP2//PPP3CB//CCDC88C//PML//ALDH1A2//VASH1//ONECUT2//CDKN2A//NF2//EMP2//SFRP1//NKAIN1//OTP//FZD3//LRRTM1//TRIM5//CD209//PAK2//SKP1//RAG1//WAS//IL4R//CREB3L3//HMOX1//BCR//CD96//TIRAP//NDFIP1//P2RX2//CHRM1//DDAH1//AQP1//CYP4F2//WNT11//COPS5//LIN9//MYBL2//NUP214//AKAP10//VPS45//PAFAH2//C1QA//FERMT2//CTNND2//TRABD2B//MARK1//NDP//CALCOCO1//TNKS2//KREMEN1//VPS26A//FZD2//GNAO1//FAM13A//EBP//RALBP1//PRDM4//PTPRT//CHRNA3//DGKG//FGF11//FSHB//HTR3E//KCNIP2//ARHGAP40//IMPA2//LGALS3BP//MRC1//NRGN//PRKAB1//HIVEP3//SH3GL1//RASL11B//SMPD1//RERGL//OGT//TNFSF14//CHRFAM7A//SH3BP5//CXCL14//UNC13B//DAPK3//CARHSP1//ARHGEF4//POLR2M//PLA2G4C//OXSR1//PPP1R3F//GLRX//MCIDAS//USP37//PARG//KRBOX1//ZNF618//EWSR1//ZBTB43//ATP1B4//MYCBP//NRBF2//HMGB3//MAP3K9//NFYA//PHF20L1//UCHL5//PFDN1//ASF1B//GPBP1L1//RSC1A1//HLTF//TCF20//ZNF322//MED6//ELK1//FOXG1//HOXC6//HOXC9//FOXI2//PTAFR//PURA//CREBZF//PRDM12//RFX4//TCF19//FOSL1//CREB5//RBM3//EIF4G2//TRIB1//CEP85//KDELR3//SYPL2//LETM2//SLC39A8//SLC12A7//VSIG4//PARP16//TLN1//RASSF1//KMT2E//TTK//KLHL22//ARHGDIB//CLEC1A//TSPAN14//TSPAN18//PTPRU//TPRA1//GPR160//GNAZ//GPR34//GRM2//TIAM1//ARHGEF6//GPR3//PALM//TAC4//EVC//ITGA5//FERMT3//SEMA7A//DBNL//ARFIP2//RHOF//RALGPS2//RALGDS//CDC42EP1//EPS8L2//CPLX1//SNPH//MAEA//NEK7//TTC28//PIM1//CSN3//KCNJ12//SYT9//DPP4//SLC25A5//CACNA2D2//BRK1//DDX39B//LIMS2//PGGT1B//LDOC1//DEC1//ATP6V1G2//PARVA//GALNT3//POLR2E//UBE4B//NFATC4//CASP7//PYURF//SPRY3//PPP1R2//RGS9BP//MAPRE1//SCN3B//UBXN2A//BMP10//DRAM1//PIP4K2C//TRIM21//DLAT//RBMS3//ZNF804A//RTN4//PAX8//SIRT3//CTNS//MICAL2//PRSS8//MEST//ABHD5//INSIG2//PPP1R26//CNTN2//TRIM67//PIK3IP1//SYT2//SYT14//CPLX3//LARP1//EXOC4//MGRN1//FBXO10//TNFAIP8L2//RELT//SEPT7//EFNA3//TAF8//DLX5//TEAD1//PASD1//NREP//MEX3B//LGI3//SCN1A//SCN7A//CHDH//DPYS//SNX19//PACSIN1//KIF2A//BEX4//PARD6B//SEMA4G//RGMA//TTL//LMOD1//CTH//EDEM3//EXD1//MAPKAP1//RNF149//FBXL3//IQSEC3//USP46//CA7//REEP2//RNF217//RNF125//RNF114//MYO19//TGM2//RNF135//RABL3//CRTC1//DIXDC1//TBC1D24//WDFY1//ACTR3//ACTR2//KCNA6//KCNQ5//CACNG1//KCNQ4//VSNL1//ASNA1//GOSR2//UNC5A//PTPN18//PRIMA1//SLC26A1//UBE2Z//MMP24//S100A12//WDR91//PHOX2A//SLC5A3//UBXN2B//PPIH//IDI1//ZMIZ1//FAM57B//SHKBP1//ENHO//DAZL//ARID3B//TET3//RAX//TLX2//TMPRSS2//SRSF9//SHISA9//CD79A//RALGAPB//CGNL1//NOVA1//SLC9A5//BEST1//LRRN1//STEAP2//TRIM68//WDR19//SLC30A6//ANO4//ANO3//HAAO//GRIPAP1//BSN//LYPD6//LSM11//SNRPA//SLMAP//PEX5//ALAD//ZSWIM6//NUCB1//SESTD1//FAM168A//DYNC1I1// |
| GO:0051090 | regulation of DNA binding transcription factor activity | Biological process | 26 | 416 | 509 | 17653 | 2.16760805500982 | 0.000197009586977629 | 0.0345111495640306 | 3.70551263947203 | 0.0510805500982318 | CDKN2A//FOXP3//TRIM21//CRTC1//TRIB1//FOXS1//HMOX1//RLIM//PIM1//TNFSF4//BHLHE40//COPS5//EDN1//FZD2//IL1B//KIT//NDP//FOSL1//TRIM5//TAB1//TIRAP//CTH//NFKB1//S100A12//HEYL//CREBZF// |
| GO:0031325 | positive regulation of cellular metabolic process | Biological process | 120 | 3074 | 509 | 17653 | 1.35387360625207 | 0.000206678540271664 | 0.0345111495640306 | 3.68470461444881 | 0.235756385068762 | TAB1//LRRK2//RAF1//IL1B//KIT//PRKAA1//THBS1//PROK1//CD74//CD81//GPNMB//CAMKK2//PML//CCK//PARP16//CHRNA3//HEYL//TIRAP//MAP3K9//DBNL//DDX39B//CASP7//TP53INP2//TRIM21//MICAL2//BMP10//TTK//EDN1//ABHD5//CNTN2//LARP1//RBMS3//PTAFR//RBM3//HMOX1//TAF8//DLX5//ELK1//ZNF281//FZD2//MYBL2//NDP//NFKB1//NFYA//FOXP3//MED1//PPP3CB//KMT2E//CALCOCO1//HIVEP3//GPBP1L1//SFRP1//TEAD1//WNT11//PAX8//NRIP1//CREB5//PAXBP1//NDFIP1//EMP2//PAK2//OXSR1//MAPKAP1//NEK7//TNKS2//TRIB1//RNF217//RNF125//RNF114//INSIG2//FGFR3//FGFR2//SMPD1//S100A12//TRIM5//TIAM1//DDAH1//CDKN1B//VLDLR//OGT//MED6//ARID3B//COPS5//PRDM4//TET3//SATB2//CRTC1//ATP1B4//FSHB//RAX//TLX2//MCIDAS//PHOX2A//SMAD1//ASCL1//NFATC4//PAX5//RGMA//ZMIZ1//REL//RFX4//HLTF//TCF20//FOSL1//CREB3L3//ONECUT2//DAZL//CELF4//ITGA5//PRDM12//DLG4//SEMA7A//EFNA3//TRABD2B//FAM168A//TRIM67//TNFSF4//PIP4K2C//GRIN2B//AGPAT1// |
| GO:0050808 | synapse organization | Biological process | 21 | 304 | 509 | 17653 | 2.39577732395823 | 0.000210961480056732 | 0.0345111495640306 | 3.67579683629475 | 0.0412573673870334 | DBNL//SPTBN2//SHANK3//BSN//UNC13B//LRRK2//P2RX2//CACNA2D2//LRRTM1//LRRN1//PALM//FBXO45//CTNND2//DLG4//ACTR2//TIAM1//CNTN2//GRIN2B//ZNF804A//GRIPAP1//NFATC4// |
| GO:0030029 | actin filament-based process | Biological process | 39 | 739 | 509 | 17653 | 1.83029421695011 | 0.000213023408035164 | 0.0345111495640306 | 3.67157267157563 | 0.0766208251473477 | WAS//RHOF//MICAL2//ARFIP2//IQSEC3//NF2//PAK2//BCR//MYOM3//EMP2//LMOD1//PACSIN1//BRK1//CGNL1//MLPH//ARHGAP40//EPS8L2//CDC42EP1//TLN1//AQP1//MAPKAP1//KIT//PARVA//NPHS2//SHANK3//DIXDC1//ACTR3//ACTR2//MYO19//DAPK3//BMP10//SFRP1//WNT11//SPTBN2//EDN1//SCN3B//SCN1A//JAM3//ARHGDIB// |
| GO:0051130 | positive regulation of cellular component organization | Biological process | 57 | 1221 | 509 | 17653 | 1.61904876836115 | 0.000214590726387626 | 0.0345111495640306 | 3.66838905015061 | 0.111984282907662 | BRK1//UNC13B//TRIM67//ZNF804A//CDC42EP1//LMOD1//PAXBP1//CDKN1B//MAPRE1//KIT//PML//NEK7//TNKS2//TRABD2B//CCK//IL1B//ACTR3//ACTR2//ARFIP2//FOXP3//SEMA7A//DAZL//EDN1//RTN4//BCR//SKIL//TIAM1//CUX1//WAS//PALM//NF2//SFRP1//WNT11//PRDM12//SHANK3//LRRTM1//LRRN1//BMP10//OGT//DLG4//CRTC1//PACSIN1//VLDLR//EPS8L2//LRRK2//YWHAB//IL4R//TNFSF14//SEPT7//CAMKK2//SYT2//PRKAA1//UBE2D3//ABCC8//KMT2E//FOSL1//PIP4K2C// |
| GO:0023051 | regulation of signaling | Biological process | 136 | 3575 | 509 | 17653 | 1.3193608748815 | 0.000217201743641691 | 0.0345111495640306 | 3.66313669266088 | 0.267190569744597 | TAB1//LRRK2//RAF1//IL1B//KIT//PRKAA1//THBS1//PROK1//CD74//CD81//EHD3//AGPAT1//TIRAP//MAP3K9//DBNL//SYT9//DPP4//SLC25A5//PPP3CB//ABCC8//CACNA2D2//FGFR2//SPRY3//PPP1R2//FGFR3//RGS9BP//GPNMB//EDN1//FGF11//FSHB//BMP10//NDP//TNFSF4//TNFSF14//CCK//CXCL14//UNC13B//TTK//CHRNA3//PIP4K2C//PIK3IP1//MGRN1//YWHAB//NREP//RFX4//SMPD1//FOXP3//SFRP1//HIC1//TRABD2B//NFATC4//HTRA4//SKIL//RASL11B//ONECUT2//ITGA5//TRIM5//TRIB1//IQSEC3//SIRT3//USP46//CA7//RALGPS2//MED1//WDFY1//OGT//ARHGDIB//ARHGEF4//BCR//EPS8L2//TIAM1//ARHGEF6//NF2//VSNL1//RNF125//CTH//HMOX1//CC2D1A//REL//S100A12//TGM2//NDFIP1//PAK2//RNF149//SHKBP1//ENHO//ASCL1//ZMIZ1//TSPAN14//EVC//COPS5//LRRTM1//TRIM67//MAPKAP1//CPLX1//CPLX3//GRIN2B//CNTN2//DLG4//SHANK3//SHISA9//P2RX2//PAX5//FAM13A//RALBP1//ARHGAP40//RHOF//RALGAPB//CGNL1//RTN4//GRM2//PALM//TRIM68//HEYL//CTNND2//KREMEN1//SEMA7A//WNT11//RBMS3//DAPK3//DLX5//NFKB1//TNKS2//DIXDC1//CELF4//SH3GL1//LYPD6//CRTC1//NRGN//PTPN18//PML//PRKAB1//LDOC1//PAX8//EMP2//LIMS2// |
| GO:0045893 | positive regulation of transcription, DNA-templated | Biological process | 65 | 1450 | 509 | 17653 | 1.55469819117946 | 0.000238520063077849 | 0.0345111495640306 | 3.62247508447489 | 0.12770137524558 | HEYL//DDX39B//MICAL2//INSIG2//MED6//ARID3B//COPS5//PRDM4//EDN1//ELK1//TET3//FGFR2//SATB2//CRTC1//ATP1B4//FSHB//RAX//TLX2//MCIDAS//IL1B//PHOX2A//SMAD1//ASCL1//MYBL2//NFATC4//NFKB1//PAX5//FOXP3//PML//MED1//PPP3CB//RGMA//ZMIZ1//CALCOCO1//RAF1//REL//RFX4//HLTF//TCF20//TEAD1//PAX8//FOSL1//NRIP1//CREB3L3//OGT//PAXBP1//ONECUT2//CD81//DLX5//PRKAA1//CAMKK2//TAF8//ZNF281//FZD2//BMP10//NDP//NFYA//KMT2E//TP53INP2//HIVEP3//GPBP1L1//SFRP1//WNT11//CREB5//CD74// |
| GO:1903508 | positive regulation of nucleic acid-templated transcription | Biological process | 65 | 1450 | 509 | 17653 | 1.55469819117946 | 0.000238520063077849 | 0.0345111495640306 | 3.62247508447489 | 0.12770137524558 | HEYL//DDX39B//MICAL2//CAMKK2//TAF8//DLX5//ELK1//ZNF281//FZD2//BMP10//IL1B//MYBL2//NDP//NFKB1//NFYA//FOXP3//MED1//PPP3CB//KMT2E//CALCOCO1//TP53INP2//HIVEP3//GPBP1L1//SFRP1//TEAD1//WNT11//PAX8//NRIP1//CREB5//CD74//INSIG2//MED6//ARID3B//COPS5//PRDM4//EDN1//TET3//FGFR2//SATB2//CRTC1//ATP1B4//FSHB//RAX//TLX2//MCIDAS//PHOX2A//SMAD1//ASCL1//NFATC4//PAX5//PML//RGMA//ZMIZ1//RAF1//REL//RFX4//HLTF//TCF20//FOSL1//CREB3L3//OGT//PAXBP1//ONECUT2//CD81//PRKAA1// |
| GO:1902680 | positive regulation of RNA biosynthetic process | Biological process | 65 | 1451 | 509 | 17653 | 1.55362672447293 | 0.000243112796231453 | 0.0345111495640306 | 3.61419218148389 | 0.12770137524558 | HEYL//DDX39B//MICAL2//CAMKK2//TAF8//DLX5//ELK1//ZNF281//FZD2//BMP10//IL1B//MYBL2//NDP//NFKB1//NFYA//FOXP3//MED1//PPP3CB//KMT2E//CALCOCO1//TP53INP2//HIVEP3//GPBP1L1//SFRP1//TEAD1//WNT11//PAX8//NRIP1//CREB5//CD74//INSIG2//MED6//ARID3B//COPS5//PRDM4//EDN1//TET3//FGFR2//SATB2//CRTC1//ATP1B4//FSHB//RAX//TLX2//MCIDAS//PHOX2A//SMAD1//ASCL1//NFATC4//PAX5//PML//RGMA//ZMIZ1//RAF1//REL//RFX4//HLTF//TCF20//FOSL1//CREB3L3//OGT//PAXBP1//ONECUT2//CD81//PRKAA1// |
| GO:0030036 | actin cytoskeleton organization | Biological process | 35 | 642 | 509 | 17653 | 1.8907484591986 | 0.000245386041292415 | 0.0345111495640306 | 3.61015014561104 | 0.068762278978389 | WAS//RHOF//MICAL2//MYOM3//EMP2//LMOD1//PACSIN1//BRK1//CGNL1//ARHGAP40//EPS8L2//CDC42EP1//TLN1//AQP1//MAPKAP1//KIT//PARVA//NPHS2//SHANK3//DIXDC1//ACTR3//ACTR2//ARFIP2//DAPK3//BMP10//NF2//SFRP1//WNT11//SPTBN2//EDN1//JAM3//ARHGDIB//IQSEC3//PAK2//BCR// |
| GO:0120039 | plasma membrane bounded cell projection morphogenesis | Biological process | 35 | 642 | 509 | 17653 | 1.8907484591986 | 0.000245386041292415 | 0.0345111495640306 | 3.61015014561104 | 0.068762278978389 | FGFR2//RTN4//LRRN1//PARD6B//CCK//EFNA3//NCAM1//SPTBN2//CNTN2//VLDLR//SHANK3//UNC5A//FBXO45//TTL//FZD3//SEMA7A//SEMA5B//PPP3CB//LRRK2//DBNL//PACSIN1//CHRNA3//SEMA4G//SKIL//TIAM1//TLX2//NFATC4//CUX1//NPTX1//CTNND2//DLG4//ACTR2//DLX5//ZSWIM6//SYT2// |
| GO:0048667 | cell morphogenesis involved in neuron differentiation | Biological process | 32 | 567 | 509 | 17653 | 1.95734625073197 | 0.000245664192585556 | 0.0345111495640306 | 3.60965814061306 | 0.0628683693516699 | FGFR2//RTN4//LRRN1//PARD6B//CCK//EFNA3//NCAM1//SPTBN2//CNTN2//VLDLR//SHANK3//UNC5A//FBXO45//TTL//FZD3//SEMA7A//SEMA5B//PPP3CB//CHRNA3//SEMA4G//SKIL//TIAM1//TLX2//NFATC4//CUX1//NPTX1//CTNND2//DLG4//LRRK2//ACTR2//DLX5//ZSWIM6// |
| GO:0051173 | positive regulation of nitrogen compound metabolic process | Biological process | 116 | 2965 | 509 | 17653 | 1.35685684657613 | 0.000250043816152225 | 0.0345111495640306 | 3.60198388154493 | 0.227897838899804 | TAB1//LRRK2//RAF1//IL1B//KIT//PRKAA1//THBS1//PROK1//CD74//CD81//GPNMB//CAMKK2//PML//CCK//PARP16//CHRNA3//HEYL//TIRAP//MAP3K9//DBNL//DDX39B//CASP7//MICAL2//BMP10//TTK//CNTN2//LARP1//RBMS3//PTAFR//RBM3//TAF8//DLX5//ELK1//ZNF281//FZD2//MYBL2//NDP//NFKB1//NFYA//FOXP3//MED1//PPP3CB//KMT2E//CALCOCO1//TP53INP2//HIVEP3//GPBP1L1//SFRP1//TEAD1//WNT11//PAX8//NRIP1//CREB5//PAXBP1//NDFIP1//EMP2//PAK2//OXSR1//MAPKAP1//NEK7//TNKS2//TRIB1//RNF217//RNF125//RNF114//INSIG2//SMPD1//FGFR3//EDN1//S100A12//FGFR2//TRIM5//TIAM1//HMOX1//DDAH1//CDKN1B//VLDLR//OGT//MED6//ARID3B//COPS5//PRDM4//TET3//SATB2//CRTC1//ATP1B4//FSHB//RAX//TLX2//MCIDAS//PHOX2A//SMAD1//ASCL1//NFATC4//PAX5//RGMA//ZMIZ1//REL//RFX4//HLTF//TCF20//FOSL1//CREB3L3//ONECUT2//DAZL//CELF4//ITGA5//PRDM12//DLG4//SEMA7A//EFNA3//TRABD2B//FAM168A//TRIM67//TNFSF4//GRIN2B// |
| GO:0048858 | cell projection morphogenesis | Biological process | 35 | 644 | 509 | 17653 | 1.88487656957376 | 0.000260104089399481 | 0.0351816791321738 | 3.58485281970083 | 0.068762278978389 | FGFR2//RTN4//LRRN1//PARD6B//CCK//EFNA3//NCAM1//SPTBN2//CNTN2//VLDLR//SHANK3//UNC5A//FBXO45//TTL//FZD3//SEMA7A//SEMA5B//PPP3CB//LRRK2//DBNL//PACSIN1//CHRNA3//SEMA4G//SKIL//TIAM1//TLX2//NFATC4//CUX1//NPTX1//CTNND2//DLG4//ACTR2//DLX5//ZSWIM6//SYT2// |
| GO:0008284 | positive regulation of cell proliferation | Biological process | 45 | 911 | 509 | 17653 | 1.71314796883323 | 0.000308627896137645 | 0.0409264796388018 | 3.51056482165881 | 0.0884086444007859 | OTP//FZD3//FGFR2//TIRAP//CD74//CD81//FOXP3//MED1//RTN4//CD209//IL1B//TNFSF4//AQP1//PML//ZMIZ1//EDN1//HMOX1//PTAFR//TGM2//THBS1//DLX5//SFRP1//BMP10//PIM1//DDX39B//ASCL1//PAXBP1//CDKN1B//CHRM1//DPP4//EMP2//FGFR3//FSHB//SLC25A5//KIT//PGGT1B//PRKAA1//BRK1//PURA//TIAM1//TTK//FOSL1//PROK1//CCK//ALDH1A2// |
| GO:0010628 | positive regulation of gene expression | Biological process | 79 | 1877 | 509 | 17653 | 1.4596998303316 | 0.000360328977611974 | 0.0468635552998035 | 3.44330081056427 | 0.155206286836935 | HEYL//DDX39B//MICAL2//CNTN2//LARP1//RBMS3//PTAFR//RBM3//THBS1//CAMKK2//TAF8//DLX5//ELK1//ZNF281//FZD2//BMP10//IL1B//MYBL2//NDP//NFKB1//NFYA//FOXP3//MED1//PPP3CB//KMT2E//CALCOCO1//TP53INP2//HIVEP3//GPBP1L1//SFRP1//TEAD1//WNT11//PAX8//NRIP1//CREB5//CD74//INSIG2//POLR2E//MED6//ARID3B//COPS5//PRDM4//EDN1//TET3//FGFR2//SATB2//CRTC1//ATP1B4//FSHB//RAX//TLX2//MCIDAS//PHOX2A//SMAD1//ASCL1//NFATC4//PAX5//PML//RGMA//ZMIZ1//RAF1//REL//RFX4//HLTF//TCF20//FOSL1//CREB3L3//OGT//PAXBP1//ONECUT2//CD81//DAZL//CELF4//TIRAP//PRKAA1//KIT//PRKAB1//ALDH1A2//ZNF804A// |
| GO:0040012 | regulation of locomotion | Biological process | 47 | 973 | 509 | 17653 | 1.6752736458041 | 0.000373653725666447 | 0.0469229938671028 | 3.42753068307645 | 0.0923379174852652 | HMOX1//BCR//EMP2//EDN1//THBS1//VASH1//BMP10//RTN4//TNFSF14//TRIB1//KIF2A//RHOF//BEX4//PARD6B//GPNMB//DAPK3//MAPRE1//FSHB//IL1B//ITGA5//KIT//SEMA4G//TIAM1//WNT11//FERMT3//SEMA7A//ONECUT2//PTPRU//NF2//SFRP1//ABCC8//JAM3//TIRAP//CD74//ARHGDIB//TAC4//PML//TRIM21//TRIM5//ZSWIM6//PTAFR//RAF1//CD81//WAS//CXCL14//CHRM1//LRRK2// |
| GO:0006464 | cellular protein modification process | Biological process | 154 | 4192 | 509 | 17653 | 1.2740902308073 | 0.000381600571150474 | 0.0469229938671028 | 3.41839098394878 | 0.302554027504912 | CDKN1B//CAMKK2//LRRK2//DLG4//FGFR3//FGFR2//GRIN2B//IL1B//KIT//SMAD1//NCAM1//MAPK4//RAF1//SPTBN2//YWHAB//SHANK3//TAB1//PRKAA1//THBS1//PROK1//CD74//CD81//UBE4B//RNF217//MGRN1//FBXL3//FBXO10//RLIM//RNF125//RNF114//SKP1//TRIM21//UBE2D3//UBE2E1//TNKS2//FBXL20//COPS5//PPIH//CCDC88C//PML//GPNMB//MAEA//RNF38//FBXO45//RNF149//MED1//DCAF16//TULP4//KLHL14//PEX5//RNF123//UBE2Z//HLTF//TRIM7//RNF135//KLHL22//SGK2//GLYCTK//NEK7//DAPK3//MARK1//MAP3K9//PAK2//PIM1//PPP3CB//PRKAB1//BCR//WNT11//MEX3B//AATK//OXSR1//TRIB1//LRRTM1//NF2//CEP85//PTPRU//PTPRT//PTPN18//PARP16//SIRT3//SNRPB//ST8SIA5//EDEM3//MGAT5//MAN1C1//GAL3ST1//TET3//GALNT10//OGT//SPRN//NRN1//VNN2//PGGT1B//PYURF//PGAP3//CHRNA3//TIRAP//ARHGEF6//DBNL//RAG1//BMP10//TTK//GALNT3//ST3GAL3//USP54//UCHL5//USP37//USP46//USP45//NUP214//CUX1//KRT2//TGM4//TGM2//TTL//GOLGA7B//CTH//ST6GALNAC6//NFS1//PARG//PAXBP1//FOXP3//UBXN2A//NDFIP1//CDKN2A//EMP2//MAPKAP1//PPP1R2//PPP1R26//KMT2E//SMPD1//EDN1//S100A12//SPRY3//TRIM5//TIAM1//SFRP1//UBXN2B//TGOLN2//NUCB1//PRDM4//VLDLR//ITGA5//CCK//NFKB1//PAX5//PRDM12//TRIM68//SH3BP5//SEMA7A//GLRX//BEX4//TRABD2B//CHRM1// |
| GO:0036211 | protein modification process | Biological process | 154 | 4192 | 509 | 17653 | 1.2740902308073 | 0.000381600571150474 | 0.0469229938671028 | 3.41839098394878 | 0.302554027504912 | CDKN1B//CAMKK2//LRRK2//DLG4//FGFR3//FGFR2//GRIN2B//IL1B//KIT//SMAD1//NCAM1//MAPK4//RAF1//SPTBN2//YWHAB//SHANK3//TAB1//PRKAA1//THBS1//PROK1//CD74//CD81//UBE4B//RNF217//MGRN1//FBXL3//FBXO10//RLIM//RNF125//RNF114//SKP1//TRIM21//UBE2D3//UBE2E1//TNKS2//FBXL20//COPS5//PPIH//CCDC88C//PML//GPNMB//MAEA//RNF38//FBXO45//RNF149//MED1//DCAF16//TULP4//KLHL14//PEX5//RNF123//UBE2Z//HLTF//TRIM7//RNF135//KLHL22//CHRM1//SGK2//GLYCTK//NEK7//DAPK3//MARK1//MAP3K9//PAK2//PIM1//PPP3CB//PRKAB1//BCR//WNT11//MEX3B//AATK//OXSR1//TRIB1//LRRTM1//NF2//CEP85//PTPRU//PTPRT//PTPN18//PARP16//SIRT3//SNRPB//ST8SIA5//EDEM3//MGAT5//MAN1C1//GAL3ST1//TET3//GALNT10//OGT//SPRN//NRN1//VNN2//PGGT1B//PYURF//PGAP3//CHRNA3//TIRAP//ARHGEF6//DBNL//RAG1//BMP10//TTK//GALNT3//ST3GAL3//USP54//UCHL5//USP37//USP46//USP45//NUP214//CUX1//KRT2//TGM4//TGM2//TTL//GOLGA7B//CTH//ST6GALNAC6//NFS1//PARG//PAXBP1//FOXP3//UBXN2A//NDFIP1//CDKN2A//EMP2//MAPKAP1//PPP1R2//PPP1R26//KMT2E//SMPD1//EDN1//S100A12//SPRY3//TRIM5//TIAM1//SFRP1//UBXN2B//TGOLN2//NUCB1//PRDM4//VLDLR//ITGA5//CCK//NFKB1//PAX5//PRDM12//TRIM68//SH3BP5//SEMA7A//GLRX//BEX4//TRABD2B// |
| GO:0032990 | cell part morphogenesis | Biological process | 35 | 663 | 509 | 17653 | 1.83086049895249 | 0.000444544241420322 | 0.0530926000886647 | 3.35208501118736 | 0.068762278978389 | FGFR2//RTN4//LRRN1//PARD6B//CCK//EFNA3//NCAM1//SPTBN2//CNTN2//VLDLR//SHANK3//UNC5A//FBXO45//TTL//FZD3//SEMA7A//SEMA5B//PPP3CB//LRRK2//DBNL//PACSIN1//CHRNA3//SEMA4G//SKIL//TIAM1//TLX2//NFATC4//CUX1//NPTX1//CTNND2//DLG4//ACTR2//DLX5//ZSWIM6//SYT2// |
| GO:0060322 | head development | Biological process | 38 | 741 | 509 | 17653 | 1.77855019898242 | 0.000447475706794897 | 0.0530926000886647 | 3.34923053731188 | 0.0746561886051081 | CTNS//FOXG1//GRIN2B//MED1//CA10//BCR//SNPH//NRGN//RFX4//AQP1//PAX5//UCHL5//SPTBN2//PHOX2A//ASCL1//LRRK2//SYPL2//MAPKAP1//NF2//FGFR2//SHANK3//FBXO45//DIXDC1//RTN4//CNTN2//RAX//OTP//DLX5//SATB2//ALDH1A2//SLC6A3//SEMA7A//GNAO1//SMAD1//FZD3//RAF1//CRISPLD1//SFRP1// |
| GO:0010604 | positive regulation of macromolecule metabolic process | Biological process | 119 | 3106 | 509 | 17653 | 1.32875909102985 | 0.000470928936679448 | 0.0545698288259241 | 3.32704462309709 | 0.233791748526523 | TAB1//LRRK2//RAF1//IL1B//KIT//PRKAA1//THBS1//PROK1//CD74//CD81//GPNMB//CAMKK2//PML//CCK//PARP16//CHRNA3//HEYL//TIRAP//MAP3K9//DBNL//DDX39B//CASP7//RBMS3//SMAD1//MED1//PRKAB1//CALCOCO1//WNT11//ALDH1A2//ZNF804A//MICAL2//BMP10//TTK//EDN1//CNTN2//LARP1//PTAFR//RBM3//TAF8//DLX5//ELK1//ZNF281//FZD2//MYBL2//NDP//NFKB1//NFYA//FOXP3//PPP3CB//KMT2E//TP53INP2//HIVEP3//GPBP1L1//SFRP1//TEAD1//PAX8//NRIP1//CREB5//PAXBP1//NDFIP1//EMP2//PAK2//OXSR1//MAPKAP1//NEK7//TNKS2//TRIB1//RNF217//RNF125//RNF114//INSIG2//SMPD1//FGFR3//S100A12//FGFR2//TRIM5//TIAM1//HMOX1//CDKN1B//POLR2E//VLDLR//OGT//MED6//ARID3B//COPS5//PRDM4//TET3//SATB2//CRTC1//ATP1B4//FSHB//RAX//TLX2//MCIDAS//PHOX2A//ASCL1//NFATC4//PAX5//RGMA//ZMIZ1//REL//RFX4//HLTF//TCF20//FOSL1//CREB3L3//ONECUT2//DAZL//CELF4//ITGA5//PRDM12//DLG4//SEMA7A//EFNA3//TRABD2B//FAM168A//TRIM67//TNFSF4//GRIN2B// |
| GO:0007165 | signal transduction | Biological process | 215 | 6210 | 509 | 17653 | 1.2007361850618 | 0.000482833981406068 | 0.0545698288259241 | 3.31620217225778 | 0.422396856581532 | CAMKK2//LRRK2//DLG4//FGFR3//FGFR2//GRIN2B//IL1B//KIT//SMAD1//NCAM1//MAPK4//RAF1//SPTBN2//YWHAB//SHANK3//TAB1//PRKAA1//THBS1//PROK1//CD74//CD81//CCK//AGPAT1//MED1//TRIM5//CD209//NFKB1//PAK2//SKP1//TIRAP//UBE2D3//WNT11//FERMT2//CTNND2//HIC1//TRABD2B//MARK1//CCDC88C//NDP//CALCOCO1//TNKS2//KREMEN1//VPS26A//FZD2//GNAO1//PPP3CB//FZD3//PTPRT//SGK2//UNC13B//DGKG//DAPK3//CARHSP1//HMOX1//ARHGEF4//BCR//POLR2M//PLA2G4C//SH3BP5//OXSR1//CDKN1B//PML//PARP16//EDN1//CLEC1A//TSPAN14//TSPAN18//CHRNA3//FSHB//SKIL//PTPRU//CHRM1//TPRA1//GPR160//GNAZ//GPR34//GRM2//PTAFR//SFRP1//TIAM1//ARHGEF6//GPR3//PALM//TAC4//HEYL//ASCL1//EVC//ITGA5//TLN1//FERMT3//SEMA7A//REL//TRIB1//MAP3K9//DBNL//DDAH1//RALBP1//ARFIP2//RHOF//RALGPS2//CDKN2A//RASSF1//RALGDS//CDC42EP1//EPS8L2//ATP6V1G2//GALNT3//POLR2E//PTBP1//UBE4B//NFATC4//SPRY3//PPP1R2//RGS9BP//GPNMB//FGF11//BMP10//TNFSF4//TNFSF14//CXCL14//TTK//PIP4K2C//PIK3IP1//MGRN1//BRK1//NREP//LRRTM1//IL4R//PIM1//RFX4//FOXP3//WAS//SMPD1//DLX5//RGMA//HTRA4//RASL11B//ONECUT2//NRIP1//CTH//EDEM3//CREB3L3//MAPKAP1//LARP1//IQSEC3//RABL3//INSIG2//RELT//WDFY1//OGT//ARHGDIB//TEAD1//NF2//P2RX2//KDELR3//ASNA1//GOSR2//UNC5A//VLDLR//ACTR3//ACTR2//PTPN18//PAX8//RNF125//CC2D1A//S100A12//TGM2//NDFIP1//RNF149//SHKBP1//ENHO//ZMIZ1//COPS5//TRIM67//EFNA3//ALDH1A2//CD79A//PAX5//FAM13A//ARHGAP40//RALGAPB//CGNL1//RTN4//DIXDC1//CNTN2//TRIM68//TRIM21//WDR19//SIRT3//SEMA4G//RBMS3//CELF4//SH3GL1//BSN//LYPD6//SLC25A5//PRKAB1//LDOC1//SHISA9//EMP2//LIMS2//EBP//PRDM4//AKAP10//HTR3E//KCNIP3//KCNIP2//IMPA2//LGALS3BP//MRC1//NRGN//HIVEP3//ABCC8//RERGL//CHRFAM7A// |
| GO:0051128 | regulation of cellular component organization | Biological process | 96 | 2403 | 509 | 17653 | 1.38553723366421 | 0.000484132741321225 | 0.0545698288259241 | 3.31503554554687 | 0.18860510805501 | SGK2//HTRA4//DLG4//LRRTM1//DAPK3//TTK//KLHL22//CDKN1B//BRK1//WAS//FERMT2//CDC42EP1//KIT//PALM//RHOF//PARVA//DPP4//UNC13B//SFRP1//ZNF804A//TRIM67//LRRK2//SEPT7//PACSIN1//EIF4G2//CDKN2A//BMP10//PML//RTN4//WNT11//TTL//ARHGAP40//EPS8L2//LMOD1//PARG//PAXBP1//FOXP3//MAPRE1//CNTN2//RAF1//NEK7//TNKS2//SHANK3//TRABD2B//CCK//DIXDC1//IL1B//ACTR3//ACTR2//ARFIP2//GPR3//SEMA7A//DAZL//EDN1//CEP85//RGMA//CHRNA3//SEMA4G//BCR//SKIL//TIAM1//TLX2//NFATC4//CUX1//NF2//PAX5//PRDM12//SPTBN2//THBS1//LRRN1//DDX39B//OGT//RASSF1//JAM3//MYO19//CRTC1//VLDLR//SH3GL1//YWHAB//SLC25A5//PEX5//IL4R//TNFSF14//ZSWIM6//GRIN2B//EHD3//CAMKK2//SYT2//PRKAA1//UBE2D3//ABCC8//GRIPAP1//KMT2E//FOSL1//ARHGDIB//PIP4K2C// |
| GO:0009891 | positive regulation of biosynthetic process | Biological process | 77 | 1841 | 509 | 17653 | 1.45056660715486 | 0.000519943530620265 | 0.0561443471444483 | 3.28404382111851 | 0.151277013752456 | HEYL//DDX39B//MICAL2//EDN1//LARP1//RBMS3//PTAFR//RBM3//THBS1//CAMKK2//TAF8//DLX5//ELK1//ZNF281//FZD2//BMP10//IL1B//MYBL2//NDP//NFKB1//NFYA//FOXP3//MED1//PPP3CB//KMT2E//CALCOCO1//TP53INP2//HIVEP3//GPBP1L1//SFRP1//TEAD1//WNT11//PAX8//NRIP1//CREB5//CD74//NEK7//TNKS2//INSIG2//HMOX1//PML//TIRAP//DDAH1//PRKAA1//MED6//ARID3B//COPS5//PRDM4//TET3//FGFR2//SATB2//CRTC1//ATP1B4//FSHB//RAX//TLX2//MCIDAS//PHOX2A//SMAD1//ASCL1//NFATC4//PAX5//RGMA//ZMIZ1//RAF1//REL//RFX4//HLTF//TCF20//FOSL1//CREB3L3//OGT//PAXBP1//ONECUT2//CD81//DAZL//LRRK2// |
| GO:0050789 | regulation of biological process | Biological process | 372 | 11709 | 509 | 17653 | 1.10185354372008 | 0.000521996561686664 | 0.0561443471444483 | 3.28233235762116 | 0.730844793713163 | CDKN1B//CUX1//EDN1//FGFR2//FOXS1//SATB2//ZNF281//DNAJB5//HEYL//KCNIP3//HIC1//ASCL1//NFKB1//PAX5//FOXP3//RLIM//MED1//CC2D1A//REL//SKIL//UBE2D3//VLDLR//NRIP1//BHLHE40//PAXBP1//CAMKK2//LRRK2//DLG4//FGFR3//GRIN2B//IL1B//KIT//SMAD1//NCAM1//MAPK4//RAF1//SPTBN2//YWHAB//SHANK3//RBM8A//TAB1//PRKAA1//THBS1//PROK1//CD74//CD81//RBFOX1//MBNL3//CELF4//PTBP1//SGK2//HTRA4//GPNMB//AGPAT1//TNFSF4//CCK//EHD3//PPP3CB//CCDC88C//PML//ALDH1A2//VASH1//ONECUT2//CDKN2A//NF2//EMP2//SFRP1//NKAIN1//OTP//FZD3//LRRTM1//TRIM5//CD209//PAK2//SKP1//RAG1//WAS//IL4R//CREB3L3//HMOX1//BCR//CD96//TIRAP//NDFIP1//CHRM1//WNT11//COPS5//LIN9//MYBL2//NUP214//C1QA//FERMT2//CTNND2//TRABD2B//MARK1//NDP//CALCOCO1//TNKS2//KREMEN1//VPS26A//FZD2//GNAO1//FAM13A//EBP//RALBP1//PRDM4//PTPRT//AKAP10//CHRNA3//DGKG//FGF11//FSHB//HTR3E//KCNIP2//ARHGAP40//IMPA2//LGALS3BP//MRC1//NRGN//PRKAB1//HIVEP3//SH3GL1//RASL11B//SMPD1//ABCC8//RERGL//OGT//TNFSF14//CHRFAM7A//SH3BP5//CXCL14//UNC13B//DAPK3//CARHSP1//ARHGEF4//POLR2M//PLA2G4C//OXSR1//PPP1R3F//GLRX//MCIDAS//USP37//PARG//KRBOX1//ZNF618//EWSR1//ZBTB43//ATP1B4//MYCBP//NRBF2//HMGB3//MAP3K9//NFYA//PHF20L1//UCHL5//PFDN1//ASF1B//GPBP1L1//RSC1A1//HLTF//TCF20//ZNF322//MED6//ELK1//FOXG1//HOXC6//HOXC9//FOXI2//PTAFR//PURA//CREBZF//PRDM12//RFX4//TCF19//FOSL1//CREB5//RBM3//EIF4G2//TRIB1//CEP85//VSIG4//PARP16//RASSF1//KMT2E//TTK//KLHL22//ARHGDIB//CLEC1A//TSPAN14//TSPAN18//PTPRU//TPRA1//GPR160//GNAZ//GPR34//GRM2//TIAM1//ARHGEF6//GPR3//PALM//TAC4//EVC//ITGA5//TLN1//FERMT3//SEMA7A//DBNL//DDAH1//ARFIP2//RHOF//RALGPS2//RALGDS//CDC42EP1//EPS8L2//MAEA//NEK7//TTC28//PIM1//KCNJ12//SYT9//DPP4//SLC25A5//CACNA2D2//BRK1//DDX39B//AQP1//PAFAH2//LIMS2//PGGT1B//LDOC1//DEC1//ATP6V1G2//PARVA//GALNT3//POLR2E//UBE4B//NFATC4//CASP7//PYURF//SPRY3//PPP1R2//RGS9BP//MAPRE1//SCN3B//UBXN2A//BMP10//DRAM1//PIP4K2C//TP53INP2//TRIM21//DLAT//P2RX2//RBMS3//ZNF804A//RTN4//PAX8//SIRT3//CTNS//MICAL2//PRSS8//MEST//ABHD5//INSIG2//PPP1R26//CNTN2//TRIM67//PIK3IP1//LARP1//EXOC4//MGRN1//FBXO10//TNFAIP8L2//RELT//SEPT7//EFNA3//TAF8//DLX5//TEAD1//PASD1//NREP//MEX3B//CPLX1//LGI3//SYT2//SYT14//PACSIN1//KIF2A//BEX4//PARD6B//SEMA4G//RGMA//TTL//LMOD1//CTH//EDEM3//EXD1//MAPKAP1//IQSEC3//USP46//CA7//CYP4F2//REEP2//RNF217//RNF125//RNF114//MYO19//RNF135//RABL3//CRTC1//DIXDC1//WDFY1//ACTR3//ACTR2//KCNA6//KCNQ5//SCN1A//SCN7A//CACNG1//KCNQ4//VSNL1//KDELR3//ASNA1//GOSR2//UNC5A//PTPN18//SLC6A3//FBXL3//UBE2Z//TGM2//S100A12//RNF149//WDR91//PHOX2A//SLC5A3//UBXN2B//PPIH//IDI1//ZMIZ1//FAM57B//SHKBP1//ENHO//DAZL//ARID3B//TET3//RAX//TLX2//TMPRSS2//CPLX3//SRSF9//SHISA9//CD79A//RALGAPB//CGNL1//NOVA1//BEST1//LRRN1//TRIM68//WDR19//SLC30A6//JAM3//BSN//LYPD6//LSM11//SNRPA//SLMAP//PEX5//ALAD//ZSWIM6//NUCB1//SESTD1//FAM168A//GRIPAP1// |
| GO:0031328 | positive regulation of cellular biosynthetic process | Biological process | 76 | 1812 | 509 | 17653 | 1.45464205016112 | 0.000523006634644424 | 0.0561443471444483 | 3.28149280181879 | 0.149312377210216 | HEYL//DDX39B//MICAL2//LARP1//RBMS3//PTAFR//RBM3//THBS1//CAMKK2//TAF8//DLX5//ELK1//ZNF281//FZD2//BMP10//IL1B//MYBL2//NDP//NFKB1//NFYA//FOXP3//MED1//PPP3CB//KMT2E//CALCOCO1//TP53INP2//HIVEP3//GPBP1L1//SFRP1//TEAD1//WNT11//PAX8//NRIP1//CREB5//CD74//NEK7//TNKS2//INSIG2//HMOX1//TIRAP//EDN1//DDAH1//PRKAA1//MED6//ARID3B//COPS5//PRDM4//TET3//FGFR2//SATB2//CRTC1//ATP1B4//FSHB//RAX//TLX2//MCIDAS//PHOX2A//SMAD1//ASCL1//NFATC4//PAX5//PML//RGMA//ZMIZ1//RAF1//REL//RFX4//HLTF//TCF20//FOSL1//CREB3L3//OGT//PAXBP1//ONECUT2//CD81//DAZL// |
| GO:0051254 | positive regulation of RNA metabolic process | Biological process | 66 | 1523 | 509 | 17653 | 1.50295082474745 | 0.000537826267796105 | 0.0568331101422665 | 3.26935799036056 | 0.129666011787819 | HEYL//DDX39B//MICAL2//CAMKK2//TAF8//DLX5//ELK1//ZNF281//FZD2//BMP10//IL1B//MYBL2//NDP//NFKB1//NFYA//FOXP3//MED1//PPP3CB//KMT2E//CALCOCO1//TP53INP2//HIVEP3//GPBP1L1//SFRP1//TEAD1//WNT11//PAX8//NRIP1//CREB5//CD74//INSIG2//MED6//ARID3B//COPS5//PRDM4//EDN1//TET3//FGFR2//SATB2//CRTC1//ATP1B4//FSHB//RAX//TLX2//MCIDAS//PHOX2A//SMAD1//ASCL1//NFATC4//PAX5//PML//RGMA//ZMIZ1//RAF1//REL//RFX4//HLTF//TCF20//FOSL1//CREB3L3//OGT//PAXBP1//ONECUT2//CD81//CELF4//PRKAA1// |
| GO:0009653 | anatomical structure morphogenesis | Biological process | 104 | 2656 | 509 | 17653 | 1.3580195043435 | 0.000553219401925317 | 0.0570970832133728 | 3.25710259717924 | 0.204322200392927 | MED1//WDR19//FGFR2//VASH1//HMOX1//ITGA5//RTN4//JAM3//PROK1//HS6ST1//EDN1//NFATC4//ZMIZ1//PAX8//TLX2//NF2//SMAD1//FZD3//SKIL//PPP3CB//CCDC103//MICAL2//DLX5//FGFR3//PARVA//THBS1//TAB1//ALDH1A2//FZD2//HEYL//WNT11//UBE4B//PAX5//ONECUT2//LRRN1//PARD6B//CCK//EFNA3//NCAM1//SPTBN2//CNTN2//VLDLR//SHANK3//UNC5A//FERMT2//CDC42EP1//DAPK3//KIT//PALM//RHOF//WAS//ZNF281//SFRP1//SEPT7//PML//ABCC8//PHOX2A//RFX4//FBXO45//LMOD1//MYOM3//TTL//FERMT3//LRRK2//INSIG2//BCR//KCNQ4//AQP1//BMP10//GPNMB//EMP2//DDAH1//IL1B//SEMA7A//EXOC4//C2ORF49//SEMA5B//SATB2//HOXC9//DBNL//PACSIN1//CHRNA3//SEMA4G//UCHL5//TIAM1//CUX1//CRISPLD1//NPTX1//TGM2//CTNND2//DLG4//ACTR2//NDP//MYO19//IL4R//TNFSF14//ZSWIM6//SYT2//PIM1//LIMS2//FOXG1//FOXS1//FOXI2//FOXP3// |
| GO:0032989 | cellular component morphogenesis | Biological process | 51 | 1101 | 509 | 17653 | 1.60651060207813 | 0.000557209447298921 | 0.0570970832133728 | 3.25398152886866 | 0.100196463654224 | MED1//WDR19//FGFR2//RTN4//LRRN1//PARD6B//CCK//EFNA3//NCAM1//SPTBN2//CNTN2//VLDLR//SHANK3//UNC5A//FERMT2//CDC42EP1//DAPK3//KIT//PALM//RHOF//PARVA//WAS//SEPT7//FBXO45//LMOD1//MYOM3//TTL//FERMT3//FZD3//BMP10//SEMA7A//SEMA5B//PPP3CB//LRRK2//DBNL//PACSIN1//CHRNA3//SEMA4G//SKIL//TIAM1//TLX2//NFATC4//CUX1//EDN1//NPTX1//CTNND2//DLG4//ACTR2//DLX5//ZSWIM6//SYT2// |
| GO:0007612 | learning | Biological process | 12 | 136 | 509 | 17653 | 3.06015254824916 | 0.000577479425688563 | 0.0577826397846477 | 3.23846348410958 | 0.0235756385068762 | ACTR2//NPTX2//NRGN//CTNS//KIT//RAG1//ABCC8//SHANK3//DLG4//PPP3CB//CNTN2//FOSL1// |
| GO:0051179 | localization | Biological process | 223 | 6497 | 509 | 17653 | 1.19039949827229 | 0.000581225197171277 | 0.0577826397846477 | 3.23565556650884 | 0.43811394891945 | SPRN//PML//NUP214//CUX1//SLC27A4//SEMA4G//SEMA7A//FBXO45//SATB2//MARK1//ASCL1//FZD3//CCK//PLLP//EDN1//NKAIN1//DLG4//LRRTM1//JAM3//S100A12//KIT//LGALS3BP//THBS1//TLN1//FERMT3//HMOX1//BCR//CD74//AQP1//SH3GL1//SPTBN2//AKAP10//MAPRE1//CD81//SYT2//SYT9//SYT14//EXOC4//U2AF1//DDX39B//SRSF9//RBM8A//YWHAB//GOLGA7B//KDELR3//GOSR2//TIMM23//TOMM34//TIMM22//SLC6A3//SLC12A7//SLC26A1//CHRNA3//CTNS//SLC9A5//TOMM40L//P2RX2//CDKN1B//KCNIP2//KCNA6//KCNJ12//ABCC8//KCNQ4//SCN3B//SCN1A//SCN7A//SLC5A3//SLC10A7//SLC5A6//SLC23A2//ANO4//BEST1//NDFIP1//SLC39A2//SLC39A8//SLC25A5//SLC25A2//EBP//RALBP1//ESYT1//PCTP//ANO3//VLDLR//VPS45//AP1S1//ARFIP2//RABL3//KCNIP3//MLPH//VPS26A//CPLX1//LGI3//SNX19//DYNC1I1//KIF2A//SCAMP1//STEAP2//TGOLN2//SYS1//LRRK2//CD209//WAS//DBNL//MRC1//TMPRSS2//BIN2//VPS53//UNC13B//SNPH//CSN3//MED1//DPP4//IL1B//PPP3CB//CACNA2D2//MGRN1//FAM160A2//SLC35A2//ATP1B4//EMP2//VASH1//BMP10//RTN4//PRSS8//TNFSF14//RSC1A1//MEST//NFKB1//ABHD5//GRM2//TRIB1//EHD3//ASNA1//CA7//CPLX3//ACTR3//ACTR2//AVL9//PAK2//RHOF//TIAM1//PEX5//NRIP1//DIXDC1//CNTN2//PTAFR//PACSIN1//BEX4//PARD6B//GPNMB//DAPK3//FSHB//ITGA5//WNT11//ONECUT2//PTPRU//NF2//SFRP1//CCDC88C//SIRT3//SLC52A2//CYP4F2//REEP2//SGK2//ARL4C//TRIM5//ATP6V1G2//HTR3E//RAF1//CHRFAM7A//TTK//MIS12//SEPT7//MYO19//KCNQ5//CACNG1//BSN//WDR19//VSNL1//BRK1//CHRM1//TGM2//IL4R//ALAD//ALDOC//TSPAN14//WDR91//RBFOX1//FOXP3//MOGAT2//TNFSF4//SNRPB//TAF8//SKP1//KRT2//POLR2M//GNAO1//CXCL14//PRKAA1//SLC30A6//TULP4//TNKS2//PARVA//FERMT2//PALM//SLMAP//TIRAP//TTL//SHANK3//GRIN2B//GRIPAP1//OXSR1//ARHGDIB//TAC4//ZSWIM6//NUCB1//TP53INP2//UBE2D3//SESTD1//COPS5//SHISA9//PAX8//GLRX// |
| GO:0007420 | brain development | Biological process | 36 | 700 | 509 | 17653 | 1.78363177097951 | 0.000597897574555636 | 0.0577826397846477 | 3.22337320834476 | 0.0707269155206287 | NRGN//RFX4//AQP1//PAX5//UCHL5//SPTBN2//PHOX2A//ASCL1//LRRK2//SYPL2//MAPKAP1//NF2//FGFR2//SHANK3//FBXO45//DIXDC1//RTN4//CNTN2//RAX//OTP//DLX5//SATB2//ALDH1A2//SLC6A3//SEMA7A//GNAO1//SMAD1//FZD3//SFRP1//CTNS//FOXG1//GRIN2B//MED1//CA10//BCR//SNPH// |
| GO:0006513 | protein monoubiquitination | Biological process | 8 | 66 | 509 | 17653 | 4.20384592486754 | 0.000598075526382573 | 0.0577826397846477 | 3.22324396882091 | 0.0157170923379175 | RAG1//UBE2E1//SKP1//UBE4B//MGRN1//TRIM21//UBE2D3//KLHL22// |
| GO:0061564 | axon development | Biological process | 28 | 497 | 509 | 17653 | 1.9539002186004 | 0.000608489654448278 | 0.0579607821554043 | 3.21574680125351 | 0.0550098231827112 | FGFR2//RTN4//LRRN1//PARD6B//CCK//EFNA3//NCAM1//SPTBN2//CNTN2//VLDLR//SHANK3//UNC5A//FBXO45//TTL//JAM3//NREP//FZD3//SEMA7A//SEMA5B//PPP3CB//RGMA//SEMA4G//SKIL//TIAM1//NPTX1//DLX5//ZSWIM6//SPRY3// |
| GO:0000902 | cell morphogenesis | Biological process | 47 | 997 | 509 | 17653 | 1.63494609565435 | 0.000634074610434908 | 0.0595589804218234 | 3.19785963644997 | 0.0923379174852652 | FGFR2//RTN4//LRRN1//PARD6B//CCK//EFNA3//NCAM1//SPTBN2//CNTN2//VLDLR//SHANK3//UNC5A//FERMT2//CDC42EP1//DAPK3//KIT//PALM//RHOF//PARVA//WAS//SEPT7//FBXO45//TTL//FERMT3//FZD3//SEMA7A//SEMA5B//PPP3CB//LRRK2//DBNL//PACSIN1//CHRNA3//SEMA4G//SKIL//TIAM1//TLX2//NFATC4//CUX1//NPTX1//CTNND2//DLG4//ACTR2//DLX5//ZSWIM6//SYT2//MED1//WDR19// |
| GO:0032956 | regulation of actin cytoskeleton organization | Biological process | 21 | 331 | 509 | 17653 | 2.20035137910363 | 0.000652685807791336 | 0.059708135366533 | 3.18529583063928 | 0.0412573673870334 | BRK1//WAS//ARHGAP40//EPS8L2//CDC42EP1//LMOD1//SHANK3//ACTR3//ACTR2//ARFIP2//DAPK3//NF2//SFRP1//WNT11//SPTBN2//EDN1//BMP10//JAM3//ARHGDIB//RHOF//DIXDC1// |
| GO:0046718 | viral entry into host cell | Biological process | 11 | 119 | 509 | 17653 | 3.20587409816579 | 0.0006561915362914 | 0.059708135366533 | 3.18296937554346 | 0.0216110019646365 | TRIM5//TRIM21//TMPRSS2//CD74//DPP4//CD209//ITGA5//MRC1//NCAM1//SLC52A2//CD81// |
| GO:0007010 | cytoskeleton organization | Biological process | 58 | 1306 | 509 | 17653 | 1.54022991963945 | 0.000662148477375421 | 0.059708135366533 | 3.17904461528144 | 0.113948919449902 | SEPT7//WAS//TTL//KIF2A//MARK1//MAP7D1//CNTN2//RHOF//MICAL2//ARFIP2//IQSEC3//NF2//PAK2//BCR//MYOM3//EMP2//LMOD1//PACSIN1//BRK1//CGNL1//TLN1//TTK//ARHGAP40//EPS8L2//CDC42EP1//AQP1//MAPKAP1//MAPRE1//CDKN1B//CCDC88C//KIT//PARVA//NPHS2//SHANK3//DIXDC1//ACTR3//ACTR2//CCDC103//DAPK3//RAF1//KRT2//KRT9//BMP10//CEP85//NEK7//SFRP1//WNT11//SPTBN2//EDN1//RASSF1//JAM3//MYBL2//MCIDAS//ARHGDIB//MAEA//PALM//PFDN1//CCDC6// |
| GO:0120035 | regulation of plasma membrane bounded cell projection organization | Biological process | 32 | 602 | 509 | 17653 | 1.84354705010802 | 0.000689955800767189 | 0.0613969879024803 | 3.16117872969425 | 0.0628683693516699 | WAS//BRK1//SFRP1//ZNF804A//TRIM67//LRRK2//TTL//RTN4//CNTN2//CDC42EP1//KIT//SEMA7A//RGMA//CHRNA3//SEMA4G//SKIL//TIAM1//TLX2//NFATC4//CUX1//PALM//SHANK3//ACTR2//DLG4//CRTC1//PACSIN1//VLDLR//EPS8L2//ZSWIM6//SEPT7//GRIN2B//SYT2// |
| GO:0030072 | peptide hormone secretion | Biological process | 17 | 243 | 509 | 17653 | 2.42629378996984 | 0.000715459235086824 | 0.0628396208687298 | 3.14541510611273 | 0.0333988212180747 | SYT9//DPP4//SLC25A5//IL1B//PPP3CB//ABCC8//CACNA2D2//CPLX1//SNX19//CPLX3//SIRT3//RAF1//VSNL1//SFRP1//TIAM1//PAX8//EDN1// |
| GO:0009893 | positive regulation of metabolic process | Biological process | 125 | 3329 | 509 | 17653 | 1.30225776810443 | 0.000729751895844147 | 0.0632732316871021 | 3.13682476807194 | 0.245579567779961 | TAB1//LRRK2//RAF1//IL1B//KIT//PRKAA1//THBS1//PROK1//CD74//CD81//GPNMB//CAMKK2//PML//CCK//PARP16//CHRNA3//HEYL//TIRAP//MAP3K9//DBNL//DDX39B//CASP7//TP53INP2//TRIM21//RBMS3//SMAD1//MED1//PRKAB1//CALCOCO1//WNT11//ALDH1A2//ZNF804A//MICAL2//BMP10//TTK//EDN1//ABHD5//CNTN2//LARP1//PTAFR//RBM3//HMOX1//TAF8//DLX5//ELK1//ZNF281//FZD2//MYBL2//NDP//NFKB1//NFYA//FOXP3//PPP3CB//KMT2E//HIVEP3//GPBP1L1//SFRP1//TEAD1//PAX8//NRIP1//CREB5//PAXBP1//AGPAT1//NDFIP1//EMP2//PAK2//OXSR1//MAPKAP1//NEK7//TNKS2//TRIB1//RNF217//RNF125//RNF114//INSIG2//FGFR3//FGFR2//SMPD1//S100A12//TRIM5//TIAM1//DDAH1//CDKN1B//POLR2E//VLDLR//OGT//MED6//ARID3B//COPS5//PRDM4//TET3//SATB2//CRTC1//ATP1B4//FSHB//RAX//TLX2//MCIDAS//PHOX2A//ASCL1//NFATC4//PAX5//RGMA//ZMIZ1//REL//RFX4//HLTF//TCF20//FOSL1//CREB3L3//ONECUT2//DAZL//CELF4//ITGA5//PRDM12//DLG4//SEMA7A//EFNA3//TRABD2B//FAM168A//TRIM67//TNFSF4//PIP4K2C//GRIN2B//PYURF// |
| GO:0048468 | cell development | Biological process | 83 | 2045 | 509 | 17653 | 1.40762029195748 | 0.000776670304837977 | 0.0653269195472909 | 3.10976329931507 | 0.163064833005894 | DAZL//SEMA4G//SEMA7A//OTP//FZD3//ONECUT2//SATB2//KRT2//ASCL1//CELF4//KIT//JAM3//FGFR2//RTN4//LRRN1//PARD6B//CCK//EFNA3//NCAM1//SPTBN2//CNTN2//VLDLR//SHANK3//UNC5A//SFRP1//ZNF804A//TRIM67//LRRK2//NF2//HEYL//EDN1//ALDH1A2//FBXO45//LMOD1//MYOM3//TTL//NREP//UBE4B//NPTXR//GNAO1//NPTX2//TBC1D24//PRDM12//FERMT2//PARVA//FERMT3//IL1B//MED1//MAEA//KCNIP2//BMP10//TRIB1//DIXDC1//DGKG//SKIL//HS6ST1//SEMA5B//PPP3CB//RGMA//P2RX2//C11ORF88//CACNA2D2//DBNL//PACSIN1//CHRNA3//TIAM1//TLX2//NFATC4//CUX1//FZD2//ABCC8//NPTX1//PAK2//CTNND2//DLG4//ACTR2//DDX39B//SPRY3//NPHS2//DLX5//CRTC1//ZSWIM6//SYT2// |
| GO:0021879 | forebrain neuron differentiation | Biological process | 7 | 53 | 509 | 17653 | 4.58060570115283 | 0.000777255843719484 | 0.0653269195472909 | 3.10943600407334 | 0.0137524557956778 | SHANK3//CNTN2//FGFR2//DLX5//ASCL1//SATB2//OTP// |
| GO:0030073 | insulin secretion | Biological process | 15 | 202 | 509 | 17653 | 2.57537590694236 | 0.000790127022203333 | 0.0653269195472909 | 3.10230308515572 | 0.0294695481335953 | SYT9//DPP4//SLC25A5//IL1B//PPP3CB//ABCC8//CACNA2D2//SIRT3//RAF1//VSNL1//SFRP1//TIAM1//CPLX1//SNX19//CPLX3// |
| GO:0003012 | muscle system process | Biological process | 26 | 456 | 509 | 17653 | 1.97746699755282 | 0.000792075617755117 | 0.0653269195472909 | 3.10123335527145 | 0.0510805500982318 | CHRM1//TIAM1//LMOD1//GNAO1//KCNIP2//KCNJ12//SCN7A//TLN1//CACNG1//SLMAP//SSPN//CHRNA3//DAPK3//MYOM3//EDN1//BMP10//IL1B//HMOX1//P2RX2//EHD3//SCN3B//DDX39B//SCN1A//KIT//ABCC8//PTAFR// |
| GO:0050793 | regulation of developmental process | Biological process | 100 | 2563 | 509 | 17653 | 1.35316928912045 | 0.000817766365170481 | 0.0666331798511803 | 3.08737075599892 | 0.196463654223969 | OTP//FZD3//FOXP3//NDFIP1//WNT11//FERMT2//CDC42EP1//DAPK3//KIT//PALM//RHOF//PARVA//WAS//FGFR2//TIAM1//IL1B//SFRP1//NFKB1//ZNF804A//TRIM67//LRRK2//NF2//SEPT7//EFNA3//VASH1//PML//ABCC8//THBS1//RFX4//TTL//RTN4//ASCL1//CNTN2//MED1//TNKS2//CACNA2D2//FOXS1//SLC6A3//EDN1//TNFSF4//HMGB3//CD74//ZMIZ1//RAG1//RAF1//SKIL//TAF8//FAM57B//IL4R//TRIB1//KMT2E//MBNL3//CXCL14//TNFSF14//NREP//DIXDC1//HEYL//SMAD1//FSHB//GPNMB//EMP2//HMOX1//PROK1//DDAH1//AQP1//SEMA7A//SHANK3//FGFR3//PRKAA1//RGMA//CHRNA3//SEMA4G//TLX2//NFATC4//CUX1//PTBP1//LRRTM1//LRRN1//BMP10//PIM1//FZD2//BCR//CDKN1B//ACTR2//DDX39B//PAX8//MYO19//DLG4//CRTC1//PACSIN1//VLDLR//ARHGDIB//ZNF322//ZSWIM6//CELF4//ITGA5//SYT2//CTH//LIMS2//CDKN2A// |
| GO:0007610 | behavior | Biological process | 31 | 583 | 509 | 17653 | 1.84413995760699 | 0.000828557443194359 | 0.0667087379562315 | 3.08167737692777 | 0.0609037328094303 | DPP4//USP46//FBXL20//CCK//GRIN2B//DLG4//PPP3CB//CNTN2//FOSL1//SHANK3//CRTC1//ITGA5//ABCC8//VLDLR//CTNS//CHRNA3//GNAO1//LRRTM1//NOVA1//SLC6A3//SCN1A//PAX5//SPTBN2//ACTR2//NPTX2//NRGN//KIT//RAG1//LRRK2//P2RX2//THBS1// |
| GO:0016043 | cellular component organization | Biological process | 215 | 6269 | 509 | 17653 | 1.18943558928598 | 0.000850425170249428 | 0.0676638285458457 | 3.07036389437062 | 0.422396856581532 | UBXN2B//UBXN2A//TP53INP2//MIS12//KLHL22//SEPT7//WAS//TTL//KIF2A//MARK1//MAP7D1//CNTN2//DDX39B//SNRPB//COPS5//MED1//WDR19//SGK2//HTRA4//EMP2//PLLP//CCK//DLG4//LRRTM1//RHOF//MICAL2//ARFIP2//IQSEC3//NF2//PAK2//BCR//SH3GL1//MAEA//PACSIN1//PALM//PFDN1//SPTBN2//CCDC6//NDP//SYT2//SYT9//SYT14//PURA//DAPK3//HLTF//ASF1B//SATB2//HMGB3//FOXP3//CELF4//SRSF9//TIMM23//TOMM34//TIMM22//SLC12A7//AQP1//BIN2//LRRK2//SIRT3//MYOM3//LMOD1//BRK1//CGNL1//TLN1//FAM160A2//PARD6B//ITGA5//TTK//DYNC1I1//MAPRE1//CENPO//CHTF8//NUP214//CDKN1B//DAZL//CHRNA3//SMAD1//ZMIZ1//FOSL1//FGFR2//RTN4//LRRN1//EFNA3//NCAM1//VLDLR//SHANK3//UNC5A//DBNL//BSN//UNC13B//P2RX2//CACNA2D2//FERMT2//CDC42EP1//KIT//PARVA//ACTR3//ACTR2//UBXN10//EHD3//MCIDAS//RFX4//ONECUT2//SKP1//RAG1//UBE2E1//DPP4//SFRP1//ZNF804A//TRIM67//PEX5//NFS1//FBXO45//FAM92B//ARHGEF4//ARHGEF6//TOMM40L//THBS1//JAM3//EIF4G2//CDKN2A//BMP10//PML//WNT11//ARHGAP40//EPS8L2//MAPKAP1//PARG//PAXBP1//NREP//CCDC88C//UBE4B//NPTXR//GNAO1//NPTX2//TBC1D24//PRDM12//RAF1//NPHS2//NEK7//TNKS2//TRABD2B//REEP2//DIXDC1//IL1B//FERMT3//LIMS2//DNAJB12//KMT2E//PRKAA1//CCDC103//FZD3//GPR3//KCTD18//ALAD//HMOX1//KCNA6//TGM2//SHKBP1//OGT//PRDM4//KRT2//KRT9//KCNIP2//SEMA7A//EDN1//CEP85//GOSR2//SEMA5B//PPP3CB//RGMA//SEMA4G//SKIL//TIAM1//TLX2//NFATC4//CUX1//CTNND2//MED6//CTH//DPYS//CRTC1//KCNJ12//CD79A//GRIN2B//CD74//PRKAB1//YWHAB//PAX5//NPTX1//TGOLN2//SPRY3//ANO4//ANO3//PPIH//TEAD1//TRIM21//RASSF1//TET3//MYO19//MYBL2//ATP6V1G2//DLX5//GRIPAP1//SLC25A5//IL4R//TNFSF14//ZSWIM6//CAMKK2//UBE2D3//FZD2//ABCC8//SNX19//ARHGDIB//PIP4K2C// |
| GO:0097061 | dendritic spine organization | Biological process | 8 | 70 | 509 | 17653 | 3.96362615773225 | 0.000889215271265472 | 0.0699274753438184 | 3.050993087393 | 0.0157170923379175 | CTNND2//DLG4//SHANK3//LRRK2//ACTR2//TIAM1//GRIN2B//ZNF804A// |
| GO:0031344 | regulation of cell projection organization | Biological process | 32 | 612 | 509 | 17653 | 1.8134237322958 | 0.000907086235623572 | 0.0705129219715197 | 3.04235142311062 | 0.0628683693516699 | WAS//BRK1//SFRP1//ZNF804A//TRIM67//LRRK2//TTL//RTN4//CNTN2//CDC42EP1//KIT//SEMA7A//RGMA//CHRNA3//SEMA4G//SKIL//TIAM1//TLX2//NFATC4//CUX1//PALM//SHANK3//ACTR2//DLG4//CRTC1//PACSIN1//VLDLR//EPS8L2//ZSWIM6//SEPT7//GRIN2B//SYT2// |
| GO:0007567 | parturition | Biological process | 4 | 16 | 509 | 17653 | 8.67043222003929 | 0.000943273019618301 | 0.0724926753599838 | 3.02536258748269 | 0.00785854616895874 | EDN1//LDOC1//PTAFR//PLA2G4C// |
| GO:0030900 | forebrain development | Biological process | 22 | 366 | 509 | 17653 | 2.08469408569251 | 0.000999932747225581 | 0.0759836535897371 | 3.00002920849101 | 0.0432220039292731 | NRGN//RFX4//AQP1//PAX5//UCHL5//LRRK2//NF2//FGFR2//SHANK3//FBXO45//DIXDC1//RTN4//CNTN2//RAX//OTP//DLX5//ASCL1//SATB2//ALDH1A2//SLC6A3//SEMA7A//GNAO1// |
| GO:0016482 | cytosolic transport | Biological process | 12 | 146 | 509 | 17653 | 2.85055305864305 | 0.00108235171103887 | 0.0813327180195098 | 2.96563159196214 | 0.0235756385068762 | TGOLN2//SYS1//EHD3//AP1S1//VPS53//VPS26A//GOSR2//EMP2//WDR91//FAM160A2//LRRK2//ACTR2// |
| GO:0043412 | macromolecule modification | Biological process | 157 | 4389 | 509 | 17653 | 1.24060866579737 | 0.00113191963934047 | 0.0841227749545011 | 2.94618440479955 | 0.308447937131631 | CDKN1B//THUMPD1//CAMKK2//LRRK2//DLG4//FGFR3//FGFR2//GRIN2B//IL1B//KIT//SMAD1//NCAM1//MAPK4//RAF1//SPTBN2//YWHAB//SHANK3//TAB1//PRKAA1//THBS1//PROK1//CD74//CD81//UBE4B//RNF217//MGRN1//FBXL3//FBXO10//RLIM//RNF125//RNF114//SKP1//TRIM21//UBE2D3//UBE2E1//TNKS2//FBXL20//COPS5//PPIH//FTSJ1//CCDC88C//PML//GPNMB//URM1//MAEA//RNF38//FBXO45//RNF149//MED1//DCAF16//TULP4//KLHL14//PEX5//RNF123//UBE2Z//HLTF//TRIM7//RNF135//KLHL22//KMT2E//CHRM1//SGK2//GLYCTK//NEK7//DAPK3//MARK1//MAP3K9//PAK2//PIM1//PPP3CB//PRKAB1//BCR//WNT11//MEX3B//AATK//OXSR1//TRIB1//LRRTM1//NF2//CEP85//PTPRU//PTPRT//PTPN18//PARP16//SIRT3//SNRPB//ST8SIA5//EDEM3//MGAT5//MAN1C1//GAL3ST1//TET3//GALNT10//OGT//SPRN//NRN1//VNN2//PGGT1B//PYURF//PGAP3//CHRNA3//TIRAP//ARHGEF6//DBNL//RAG1//BMP10//TTK//GALNT3//ST3GAL3//USP54//UCHL5//USP37//USP46//USP45//NUP214//CUX1//KRT2//TGM4//TGM2//TTL//GOLGA7B//CTH//ST6GALNAC6//NFS1//PARG//PAXBP1//FOXP3//UBXN2A//NDFIP1//CDKN2A//EMP2//MAPKAP1//PPP1R2//PPP1R26//SMPD1//EDN1//S100A12//SPRY3//TRIM5//TIAM1//SFRP1//UBXN2B//TGOLN2//NUCB1//PRDM4//VLDLR//ITGA5//CCK//NFKB1//PAX5//PRDM12//TRIM68//SH3BP5//SEMA7A//GLRX//BEX4//TRABD2B// |
| GO:0022604 | regulation of cell morphogenesis | Biological process | 26 | 468 | 509 | 17653 | 1.92676271556429 | 0.00115205004535975 | 0.084586197198729 | 2.93852865463546 | 0.0510805500982318 | FERMT2//CDC42EP1//DAPK3//KIT//PALM//RHOF//PARVA//WAS//SEPT7//TTL//RTN4//CNTN2//SEMA7A//CHRNA3//SEMA4G//SKIL//TIAM1//TLX2//NFATC4//CUX1//LRRK2//SHANK3//ACTR2//DLG4//ZSWIM6//SYT2// |
| GO:0106027 | neuron projection organization | Biological process | 8 | 73 | 509 | 17653 | 3.80073741152407 | 0.00117453030138753 | 0.084586197198729 | 2.93013577449672 | 0.0157170923379175 | CTNND2//DLG4//SHANK3//LRRK2//ACTR2//TIAM1//GRIN2B//ZNF804A// |
| GO:0000165 | MAPK cascade | Biological process | 44 | 944 | 509 | 17653 | 1.61652126136326 | 0.00118045224160181 | 0.084586197198729 | 2.92795157879141 | 0.0864440078585462 | TAB1//LRRK2//RAF1//IL1B//KIT//PRKAA1//THBS1//PROK1//CD74//CD81//TRIB1//ARHGEF6//MAP3K9//DBNL//EDN1//S100A12//SPRY3//SMPD1//BMP10//PAK2//RNF149//NF2//FGFR3//FGFR2//TRIM5//TIAM1//SFRP1//COPS5//TIRAP//NFKB1//SKP1//MED1//SIRT3//GPNMB//SEMA7A//CAMKK2//DLG4//GRIN2B//SMAD1//NCAM1//MAPK4//SPTBN2//YWHAB//SHANK3// |
| GO:0000904 | cell morphogenesis involved in differentiation | Biological process | 35 | 701 | 509 | 17653 | 1.73161271156277 | 0.00118818405055142 | 0.084586197198729 | 2.925116281621 | 0.068762278978389 | FGFR2//RTN4//LRRN1//PARD6B//CCK//EFNA3//NCAM1//SPTBN2//CNTN2//VLDLR//SHANK3//UNC5A//FBXO45//TTL//FERMT2//PARVA//FERMT3//FZD3//SEMA7A//SEMA5B//PPP3CB//CHRNA3//SEMA4G//SKIL//TIAM1//TLX2//NFATC4//CUX1//NPTX1//CTNND2//DLG4//LRRK2//ACTR2//DLX5//ZSWIM6// |
| GO:0023014 | signal transduction by protein phosphorylation | Biological process | 45 | 973 | 509 | 17653 | 1.60398540555711 | 0.00121537963478251 | 0.085620963229522 | 2.91528804507911 | 0.0884086444007859 | CAMKK2//LRRK2//DLG4//FGFR3//FGFR2//GRIN2B//IL1B//KIT//SMAD1//NCAM1//MAPK4//RAF1//SPTBN2//YWHAB//SHANK3//TAB1//PRKAA1//THBS1//PROK1//CD74//CD81//TRIB1//ARHGEF6//MAP3K9//DBNL//EDN1//S100A12//SPRY3//SMPD1//BMP10//PAK2//RNF149//NF2//TRIM5//TIAM1//SFRP1//COPS5//TIRAP//NFKB1//SKP1//MED1//SIRT3//GPNMB//SEMA7A//OXSR1// |
| GO:0048583 | regulation of response to stimulus | Biological process | 152 | 4243 | 509 | 17653 | 1.24242818519535 | 0.00132695496346732 | 0.0925174888446339 | 2.87714381673582 | 0.298624754420432 | TAB1//LRRK2//RAF1//IL1B//KIT//PRKAA1//THBS1//PROK1//CD74//CD81//PPP3CB//AGPAT1//TRIM5//CD209//NFKB1//PAK2//SKP1//WAS//CREB3L3//FOXP3//TNFSF4//CD96//TIRAP//UBE2D3//NDFIP1//C1QA//PARG//VSIG4//MAP3K9//DBNL//FGFR2//SPRY3//PPP1R2//FGFR3//RGS9BP//GPNMB//EDN1//FGF11//FSHB//BMP10//NDP//TNFSF14//CCK//CXCL14//TTK//CHRNA3//PIP4K2C//PIK3IP1//MGRN1//YWHAB//NREP//RFX4//SMPD1//SFRP1//HIC1//TRABD2B//NFATC4//HTRA4//SKIL//RASL11B//ONECUT2//ITGA5//TRIB1//IQSEC3//RALGPS2//HMOX1//MED1//WDFY1//OGT//ARHGDIB//ARHGEF4//BCR//EPS8L2//TIAM1//ARHGEF6//NF2//VSNL1//ACTR3//ACTR2//BRK1//RNF125//CTH//CC2D1A//REL//S100A12//TGM2//IL4R//PTAFR//RNF149//RNF135//SHKBP1//ENHO//ASCL1//ZMIZ1//TSPAN14//EVC//COPS5//LRRTM1//TRIM67//MAPKAP1//RGMA//RTN4//SEMA4G//SEMA7A//TNFAIP8L2//P2RX2//CD79A//PAX5//FAM13A//RALBP1//ARHGAP40//RHOF//RALGAPB//CGNL1//SLC6A3//PALM//CNTN2//TRIM68//HEYL//CTNND2//KREMEN1//ABCC8//DDX39B//SIRT3//JAM3//WNT11//RBMS3//DAPK3//DLX5//TNKS2//DIXDC1//CELF4//UNC13B//SH3GL1//LYPD6//NUP214//DDAH1//SHANK3//SLC25A5//PTPN18//PML//PRKAB1//ZSWIM6//LDOC1//FAM168A//DLG4//SHISA9//VASH1//CDKN2A//RAG1//EMP2//LIMS2// |
| GO:0040011 | locomotion | Biological process | 75 | 1845 | 509 | 17653 | 1.40982637724216 | 0.00137070913380593 | 0.0945929170605051 | 2.86305469326658 | 0.147347740667976 | SEMA4G//SEMA7A//FBXO45//SATB2//MARK1//ASCL1//FZD3//CCK//JAM3//S100A12//KIT//HMOX1//BCR//RALBP1//PTAFR//FOSL1//CXCL14//EFNA3//NCAM1//SPTBN2//CNTN2//VLDLR//SHANK3//UNC5A//EMP2//EDN1//THBS1//VASH1//BMP10//RTN4//PML//TNFSF14//TRIB1//AVL9//PAK2//RHOF//TIAM1//DIXDC1//LRRK2//KIF2A//BEX4//PARD6B//GPNMB//DAPK3//MAPRE1//FSHB//IL1B//ITGA5//WNT11//FERMT3//ONECUT2//PTPRU//NF2//SFRP1//PPP3CB//CHRM1//DPP4//BRK1//CD74//SEMA5B//KRT2//BIN2//ABCC8//ACTR3//PARVA//TIRAP//DLX5//ARHGDIB//TAC4//TRIM21//TRIM5//ZSWIM6//RAF1//CD81//WAS// |
| GO:0071346 | cellular response to interferon-gamma | Biological process | 13 | 171 | 509 | 17653 | 2.63662266340376 | 0.00139955609033661 | 0.0956080589792575 | 2.85400969152755 | 0.0255402750491159 | NCAM1//PML//TRIM68//PTAFR//TRIM21//TRIM5//MED1//ACTR3//ACTR2//DAPK3//EDN1//MRC1//WAS// |
| GO:0007264 | small GTPase mediated signal transduction | Biological process | 30 | 578 | 509 | 17653 | 1.80008973426421 | 0.00145628720378435 | 0.0984887035919356 | 2.83675296656253 | 0.0589390962671906 | CDKN2A//RASSF1//RALGDS//CDC42EP1//PAK2//RHOF//EPS8L2//DBNL//BRK1//TIAM1//IQSEC3//RABL3//RALGPS2//WAS//OGT//ARHGDIB//ARHGEF4//RAF1//BCR//ARHGEF6//SPRY3//TRIM67//MAPKAP1//FAM13A//RALBP1//ARHGAP40//RALGAPB//CGNL1//ARFIP2//HMOX1// |
| GO:0070633 | transepithelial transport | Biological process | 4 | 18 | 509 | 17653 | 7.70705086225715 | 0.00151501695632329 | 0.101446135402123 | 2.81958250643796 | 0.00785854616895874 | BEST1//AQP1//EDN1//SLC23A2// |
| GO:0051960 | regulation of nervous system development | Biological process | 39 | 820 | 509 | 17653 | 1.64949686137333 | 0.00155322700980975 | 0.102208044159903 | 2.80876506590519 | 0.0766208251473477 | OTP//FZD3//SFRP1//ZNF804A//TRIM67//LRRK2//NF2//RFX4//TTL//RTN4//CNTN2//EFNA3//NREP//ASCL1//MED1//DIXDC1//HEYL//SEMA7A//KIT//SHANK3//RGMA//CHRNA3//SEMA4G//SKIL//TIAM1//TLX2//NFATC4//CUX1//LRRTM1//LRRN1//ABCC8//ACTR2//FZD2//DLG4//CRTC1//PACSIN1//VLDLR//ZSWIM6//SYT2// |
| GO:0030901 | midbrain development | Biological process | 9 | 94 | 509 | 17653 | 3.32059106299377 | 0.00155662110727044 | 0.102208044159903 | 2.80781708495693 | 0.0176817288801572 | SYPL2//MAPKAP1//FZD3//SFRP1//FGFR2//PHOX2A//SMAD1//UCHL5//RFX4// |
| GO:0006936 | muscle contraction | Biological process | 21 | 355 | 509 | 17653 | 2.05159522953042 | 0.00157216707322842 | 0.102236210733114 | 2.80350130363653 | 0.0412573673870334 | CHRM1//CHRNA3//DAPK3//MYOM3//EDN1//P2RX2//BMP10//EHD3//SCN3B//SCN1A//KIT//PTAFR//LMOD1//GNAO1//KCNIP2//KCNJ12//SCN7A//TLN1//CACNG1//SLMAP//SSPN// |
| GO:1902905 | positive regulation of supramolecular fiber organization | Biological process | 14 | 195 | 509 | 17653 | 2.48997027857539 | 0.00160968338657547 | 0.103678940413428 | 2.79325953824471 | 0.0275049115913556 | CDC42EP1//LMOD1//CDKN1B//MAPRE1//ACTR3//ACTR2//ARFIP2//WAS//NF2//SFRP1//WNT11//EDN1//BMP10//BRK1// |
| GO:0007611 | learning or memory | Biological process | 16 | 240 | 509 | 17653 | 2.31211525867714 | 0.00167621209459496 | 0.10659893068041 | 2.77567103004676 | 0.031434184675835 | DLG4//PPP3CB//CNTN2//FOSL1//SHANK3//CRTC1//ITGA5//ABCC8//VLDLR//CTNS//ACTR2//NPTX2//NRGN//KIT//RAG1//GRIN2B// |
| GO:0009966 | regulation of signal transduction | Biological process | 119 | 3215 | 509 | 17653 | 1.2837094048954 | 0.00172627677860831 | 0.10659893068041 | 2.76288957143337 | 0.233791748526523 | TAB1//LRRK2//RAF1//IL1B//KIT//PRKAA1//THBS1//PROK1//CD74//CD81//AGPAT1//TIRAP//MAP3K9//DBNL//FGFR2//FGFR3//RGS9BP//GPNMB//EDN1//FGF11//FSHB//BMP10//NDP//TNFSF4//TNFSF14//CCK//CXCL14//TTK//PIP4K2C//PIK3IP1//MGRN1//YWHAB//NREP//RFX4//SMPD1//FOXP3//SFRP1//HIC1//TRABD2B//NFATC4//HTRA4//SKIL//RASL11B//ONECUT2//ITGA5//TRIM5//TRIB1//IQSEC3//RALGPS2//MED1//WDFY1//OGT//ARHGDIB//ARHGEF4//BCR//EPS8L2//TIAM1//ARHGEF6//NF2//RNF125//SPRY3//CTH//HMOX1//CC2D1A//REL//S100A12//TGM2//NDFIP1//PAK2//RNF149//SHKBP1//ENHO//ASCL1//ZMIZ1//TSPAN14//EVC//COPS5//LRRTM1//TRIM67//MAPKAP1//P2RX2//PAX5//FAM13A//RALBP1//ARHGAP40//RHOF//RALGAPB//CGNL1//RTN4//PALM//CNTN2//TRIM68//HEYL//CTNND2//KREMEN1//SIRT3//SEMA7A//WNT11//RBMS3//DAPK3//DLX5//NFKB1//TNKS2//DIXDC1//CELF4//UNC13B//SH3GL1//LYPD6//SHANK3//SLC25A5//PTPN18//PML//PRKAB1//LDOC1//DLG4//SHISA9//EMP2//LIMS2//PPP1R2// |
| GO:0065009 | regulation of molecular function | Biological process | 124 | 3373 | 509 | 17653 | 1.27498795764586 | 0.00172833589482809 | 0.10659893068041 | 2.76237185034433 | 0.243614931237721 | CDKN1B//TAB1//LRRK2//RAF1//IL1B//KIT//PRKAA1//THBS1//PROK1//CD74//CD81//TRIB1//LRRTM1//NF2//PAK2//CEP85//PML//CCK//PARP16//CHRNA3//GRM2//PALM//TIRAP//MAP3K9//DBNL//CASP7//GPNMB//EDN1//FGF11//FSHB//BMP10//NDP//TNFSF4//TNFSF14//CXCL14//FGFR3//FGFR2//PTAFR//PPP1R26//UCHL5//NFKB1//CDKN2A//FOXP3//TRIM21//NFATC4//TIAM1//EMP2//WNT11//OXSR1//MAPKAP1//NDFIP1//SGK2//PPP1R2//CRTC1//RALBP1//FAM13A//CDC42EP1//GNAO1//ARHGAP40//ARHGDIB//TBC1D24//BCR//SFRP1//ARHGEF6//MMP24//YWHAB//AQP1//RAG1//HMOX1//S100A12//SPRY3//SMPD1//FOXS1//RLIM//PIM1//BHLHE40//NRBF2//WDR91//PIK3IP1//UBXN2B//UBXN2A//CAMKK2//VLDLR//PRKAB1//RALGDS//WAS//DDAH1//ABHD5//CREBZF//COPS5//FZD2//FOSL1//TRIM5//CTH//NEK7//DLG4//SH3BP5//RALGAPB//LYPD6//EHD3//EFNA3//MAPRE1//LDOC1//ABCC8//SLMAP//MED1//SHISA9//SHANK3//PPP1R3F//DYNC1I1//SCN3B//GLRX//HEYL//GRIN2B//KCNIP2//KCNIP3//IQSEC3//NCAM1//ARHGEF4//RALGPS2//PRSS8//RSC1A1//EPS8L2//SPTBN2// |
| GO:0045935 | positive regulation of nucleobase-containing compound metabolic process | Biological process | 71 | 1742 | 509 | 17653 | 1.41354922531065 | 0.00173451137452178 | 0.10659893068041 | 2.76082284781048 | 0.139489194499018 | PARP16//HEYL//DDX39B//MICAL2//CAMKK2//TAF8//DLX5//ELK1//ZNF281//FZD2//BMP10//IL1B//MYBL2//NDP//NFKB1//NFYA//FOXP3//MED1//PPP3CB//KMT2E//CALCOCO1//TP53INP2//HIVEP3//GPBP1L1//SFRP1//TEAD1//WNT11//PAX8//NRIP1//CREB5//CD74//PML//NEK7//TNKS2//INSIG2//PRKAA1//MED6//ARID3B//COPS5//PRDM4//EDN1//TET3//FGFR2//SATB2//CRTC1//ATP1B4//FSHB//RAX//TLX2//MCIDAS//PHOX2A//SMAD1//ASCL1//NFATC4//PAX5//RGMA//ZMIZ1//RAF1//REL//RFX4//HLTF//TCF20//FOSL1//CREB3L3//OGT//PAXBP1//ONECUT2//CD81//CELF4//FAM168A//TNFSF4// |
| GO:0048813 | dendrite morphogenesis | Biological process | 11 | 134 | 509 | 17653 | 2.84700759463977 | 0.00174088675376483 | 0.10659893068041 | 2.75922947920701 | 0.0216110019646365 | CHRNA3//TLX2//NFATC4//CUX1//CTNND2//DLG4//SHANK3//LRRK2//ACTR2//TIAM1//VLDLR// |
| GO:0002704 | negative regulation of leukocyte mediated immunity | Biological process | 6 | 45 | 509 | 17653 | 4.62423051735429 | 0.00174959061149276 | 0.10659893068041 | 2.757063560439 | 0.0117878192534381 | PPP3CB//FOXP3//CD96//HMOX1//BCR//NDFIP1// |
| GO:0030260 | entry into host cell | Biological process | 11 | 135 | 509 | 17653 | 2.82591864949429 | 0.00184738844131936 | 0.108642504596894 | 2.73344177798442 | 0.0216110019646365 | DPP4//CD209//ITGA5//MRC1//NCAM1//SLC52A2//CD81//TRIM5//TRIM21//TMPRSS2//CD74// |
| GO:0044409 | entry into host | Biological process | 11 | 135 | 509 | 17653 | 2.82591864949429 | 0.00184738844131936 | 0.108642504596894 | 2.73344177798442 | 0.0216110019646365 | DPP4//CD209//ITGA5//MRC1//NCAM1//SLC52A2//CD81//TRIM5//TRIM21//TMPRSS2//CD74// |
| GO:0051806 | entry into cell of other organism involved in symbiotic interaction | Biological process | 11 | 135 | 509 | 17653 | 2.82591864949429 | 0.00184738844131936 | 0.108642504596894 | 2.73344177798442 | 0.0216110019646365 | DPP4//CD209//ITGA5//MRC1//NCAM1//SLC52A2//CD81//TRIM5//TRIM21//TMPRSS2//CD74// |
| GO:0051828 | entry into other organism involved in symbiotic interaction | Biological process | 11 | 135 | 509 | 17653 | 2.82591864949429 | 0.00184738844131936 | 0.108642504596894 | 2.73344177798442 | 0.0216110019646365 | DPP4//CD209//ITGA5//MRC1//NCAM1//SLC52A2//CD81//TRIM5//TRIM21//TMPRSS2//CD74// |
| GO:0003007 | heart morphogenesis | Biological process | 16 | 243 | 509 | 17653 | 2.28357062585397 | 0.00190267125832765 | 0.110929014828189 | 2.7206362422056 | 0.031434184675835 | CCDC103//MICAL2//FGFR2//PARVA//FZD2//HEYL//WNT11//UBE4B//MED1//MYOM3//BMP10//RTN4//PIM1//TAB1//ZMIZ1//ALDH1A2// |
| GO:0060284 | regulation of cell development | Biological process | 40 | 858 | 509 | 17653 | 1.61686381725674 | 0.00195071222543777 | 0.111610858952204 | 2.70980679422546 | 0.0785854616895874 | OTP//FZD3//SFRP1//ZNF804A//TRIM67//LRRK2//NF2//TTL//RTN4//CNTN2//TRIB1//EFNA3//NREP//ASCL1//MED1//DIXDC1//HEYL//SEMA7A//KIT//SHANK3//RGMA//CHRNA3//SEMA4G//SKIL//TIAM1//TLX2//NFATC4//CUX1//ABCC8//EDN1//BMP10//ACTR2//DDX39B//DLG4//CRTC1//PACSIN1//VLDLR//ZSWIM6//IL1B//SYT2// |
| GO:0050773 | regulation of dendrite development | Biological process | 11 | 136 | 509 | 17653 | 2.80513983589507 | 0.00195914043469487 | 0.111610858952204 | 2.70793443188323 | 0.0216110019646365 | CHRNA3//TLX2//NFATC4//CUX1//SHANK3//LRRK2//ACTR2//TIAM1//CRTC1//PACSIN1//VLDLR// |
| GO:0045620 | negative regulation of lymphocyte differentiation | Biological process | 6 | 46 | 509 | 17653 | 4.52370376697702 | 0.00196387582660244 | 0.111610858952204 | 2.70688597557464 | 0.0117878192534381 | HMGB3//SFRP1//CD74//TNFSF4//IL4R//FOXP3// |
| GO:0042471 | ear morphogenesis | Biological process | 10 | 117 | 509 | 17653 | 2.96425033163736 | 0.00206296748459913 | 0.116265409152866 | 2.68550761709251 | 0.0196463654223969 | FGFR2//DLX5//INSIG2//BCR//PAX8//FZD3//KCNQ4//EDN1//FZD2//WDR19// |
| GO:0043549 | regulation of kinase activity | Biological process | 41 | 889 | 509 | 17653 | 1.59949480774628 | 0.00209350969983528 | 0.117011620661041 | 2.67912502255628 | 0.0805500982318271 | CDKN1B//TAB1//LRRK2//RAF1//IL1B//KIT//PRKAA1//THBS1//PROK1//CD74//CD81//TRIB1//LRRTM1//NF2//PAK2//CEP85//PARP16//CHRNA3//TIRAP//MAP3K9//DBNL//CDKN2A//EMP2//WNT11//OXSR1//MAPKAP1//FGFR3//FGFR2//EDN1//S100A12//SPRY3//SMPD1//TIAM1//SFRP1//NRBF2//WDR91//PIK3IP1//CAMKK2//VLDLR//DLG4//SH3BP5// |
| GO:0006935 | chemotaxis | Biological process | 30 | 594 | 509 | 17653 | 1.75160246869481 | 0.00220093371874624 | 0.122007497867876 | 2.65739303605465 | 0.0589390962671906 | S100A12//KIT//EFNA3//NCAM1//SPTBN2//CNTN2//VLDLR//SHANK3//UNC5A//THBS1//TNFSF14//IL1B//FZD3//SEMA4G//SEMA7A//GPNMB//SEMA5B//BIN2//CXCL14//PARVA//JAM3//TIRAP//EDN1//CD74//DLX5//ZSWIM6//TIAM1//RALBP1//PTAFR//FOSL1// |
| GO:0042330 | taxis | Biological process | 30 | 595 | 509 | 17653 | 1.74865859899952 | 0.00225675841662677 | 0.124085017655665 | 2.64651492915503 | 0.0589390962671906 | S100A12//KIT//RALBP1//PTAFR//FOSL1//CXCL14//EFNA3//NCAM1//SPTBN2//CNTN2//VLDLR//SHANK3//UNC5A//THBS1//TNFSF14//IL1B//FZD3//SEMA4G//SEMA7A//GPNMB//SEMA5B//BIN2//PARVA//JAM3//TIRAP//EDN1//CD74//DLX5//ZSWIM6//TIAM1// |
| GO:0070542 | response to fatty acid | Biological process | 8 | 81 | 509 | 17653 | 3.42535593878095 | 0.002301255679844 | 0.125511227119234 | 2.63803512658272 | 0.0157170923379175 | EDN1//PRKAA1//SFRP1//TNFSF4//PTAFR//NFATC4//ALAD//INSIG2// |
| GO:0021872 | forebrain generation of neurons | Biological process | 7 | 64 | 509 | 17653 | 3.79331409626719 | 0.00238772612219289 | 0.129185534115124 | 2.6220154892854 | 0.0137524557956778 | SHANK3//FGFR2//CNTN2//OTP//DLX5//ASCL1//SATB2// |
| GO:0050767 | regulation of neurogenesis | Biological process | 35 | 732 | 509 | 17653 | 1.65827938634631 | 0.00244564009853724 | 0.130898006722565 | 2.61160745356284 | 0.068762278978389 | OTP//FZD3//SFRP1//ZNF804A//TRIM67//LRRK2//NF2//TTL//RTN4//CNTN2//EFNA3//NREP//ASCL1//MED1//DIXDC1//HEYL//SEMA7A//KIT//SHANK3//RGMA//CHRNA3//SEMA4G//SKIL//TIAM1//TLX2//NFATC4//CUX1//ABCC8//ACTR2//DLG4//CRTC1//PACSIN1//VLDLR//ZSWIM6//SYT2// |
| GO:0021670 | lateral ventricle development | Biological process | 3 | 10 | 509 | 17653 | 10.4045186640472 | 0.00245808766135824 | 0.130898006722565 | 2.60940263318052 | 0.00589390962671906 | AQP1//PAX5//UCHL5// |
| GO:0045664 | regulation of neuron differentiation | Biological process | 30 | 600 | 509 | 17653 | 1.73408644400786 | 0.00255449564722736 | 0.134969172360927 | 2.59269483300393 | 0.0589390962671906 | SFRP1//ZNF804A//TRIM67//LRRK2//TTL//RTN4//CNTN2//ASCL1//MED1//DIXDC1//HEYL//SEMA7A//RGMA//CHRNA3//SEMA4G//SKIL//TIAM1//TLX2//NFATC4//CUX1//SHANK3//ACTR2//DLG4//CRTC1//PACSIN1//VLDLR//ZSWIM6//SYT2//EFNA3//NREP// |
| GO:0045860 | positive regulation of protein kinase activity | Biological process | 27 | 522 | 509 | 17653 | 1.79388252828399 | 0.00259122874453258 | 0.135848682164913 | 2.58649424733709 | 0.0530451866404715 | TAB1//LRRK2//RAF1//IL1B//KIT//PRKAA1//THBS1//PROK1//CD74//CD81//PARP16//CHRNA3//TIRAP//MAP3K9//DBNL//EMP2//PAK2//WNT11//OXSR1//MAPKAP1//EDN1//S100A12//TIAM1//CDKN1B//DLG4//CAMKK2//VLDLR// |
| GO:0048814 | regulation of dendrite morphogenesis | Biological process | 8 | 83 | 509 | 17653 | 3.34281724146093 | 0.00268476572078741 | 0.139669773612964 | 2.57109360590798 | 0.0157170923379175 | TLX2//NFATC4//CUX1//LRRK2//SHANK3//ACTR2//TIAM1//CHRNA3// |
| GO:0043153 | entrainment of circadian clock by photoperiod | Biological process | 4 | 21 | 509 | 17653 | 6.60604359622041 | 0.00276714198869899 | 0.142856345569246 | 2.55796855559474 | 0.00785854616895874 | CRTC1//FBXL3//PML//BHLHE40// |
| GO:2000145 | regulation of cell motility | Biological process | 41 | 904 | 509 | 17653 | 1.57295451779474 | 0.00282488821761568 | 0.144732719816173 | 2.54899873277664 | 0.0805500982318271 | HMOX1//BCR//EMP2//EDN1//THBS1//VASH1//BMP10//RTN4//TNFSF14//TRIB1//KIF2A//RHOF//BEX4//PARD6B//GPNMB//DAPK3//MAPRE1//FSHB//IL1B//ITGA5//KIT//SEMA4G//TIAM1//WNT11//FERMT3//SEMA7A//ONECUT2//PTPRU//NF2//SFRP1//ABCC8//JAM3//TIRAP//CD74//ARHGDIB//TAC4//PTAFR//WAS//CXCL14//RAF1//CD81// |
| GO:0050890 | cognition | Biological process | 17 | 277 | 509 | 17653 | 2.12848155582192 | 0.00291765640188434 | 0.14836173117251 | 2.534965854167 | 0.0333988212180747 | GRIN2B//DLG4//PPP3CB//CNTN2//FOSL1//SHANK3//CRTC1//ITGA5//ABCC8//VLDLR//CTNS//ACTR2//NPTX2//NRGN//KIT//RAG1//CHRM1// |
| GO:0010975 | regulation of neuron projection development | Biological process | 24 | 450 | 509 | 17653 | 1.84969220694172 | 0.00298312247194861 | 0.150558636401406 | 2.525328916333 | 0.0471512770137525 | TRIM67//ZNF804A//LRRK2//TTL//RTN4//CNTN2//SEMA7A//RGMA//CHRNA3//SEMA4G//SKIL//TIAM1//TLX2//NFATC4//CUX1//SHANK3//ACTR2//DLG4//CRTC1//PACSIN1//VLDLR//ZSWIM6//SYT2//SFRP1// |
| GO:2001238 | positive regulation of extrinsic apoptotic signaling pathway | Biological process | 6 | 50 | 509 | 17653 | 4.16180746561886 | 0.00302445635889173 | 0.151514061890258 | 2.51935267772276 | 0.0117878192534381 | SFRP1//SKIL//THBS1//DAPK3//PAK2//PML// |
| GO:0001662 | behavioral fear response | Biological process | 5 | 35 | 509 | 17653 | 4.95453269716531 | 0.00310383679462813 | 0.152110494507754 | 2.50810112280556 | 0.00982318271119843 | RAG1//DPP4//USP46//FBXL20//CCK// |
| GO:0002209 | behavioral defense response | Biological process | 5 | 35 | 509 | 17653 | 4.95453269716531 | 0.00310383679462813 | 0.152110494507754 | 2.50810112280556 | 0.00982318271119843 | DPP4//USP46//FBXL20//CCK//RAG1// |
| GO:0002823 | negative regulation of adaptive immune response based on somatic recombination of immune receptors built from immunoglobulin superfamily domains | Biological process | 5 | 35 | 509 | 17653 | 4.95453269716531 | 0.00310383679462813 | 0.152110494507754 | 2.50810112280556 | 0.00982318271119843 | PPP3CB//FOXP3//IL4R//TNFSF4//NDFIP1// |
| GO:0071840 | cellular component organization or biogenesis | Biological process | 217 | 6486 | 509 | 17653 | 1.16033536339718 | 0.00317982263260048 | 0.152895349136764 | 2.49759710386184 | 0.426326129666012 | UBXN2B//UBXN2A//TP53INP2//MIS12//KLHL22//SEPT7//WAS//THUMPD1//TTL//KIF2A//MARK1//MAP7D1//CNTN2//DDX39B//SNRPB//COPS5//MED1//WDR19//SGK2//HTRA4//EMP2//PLLP//CCK//DLG4//LRRTM1//RHOF//MICAL2//ARFIP2//IQSEC3//NF2//PAK2//BCR//SH3GL1//MAEA//PACSIN1//PALM//PFDN1//SPTBN2//CCDC6//NDP//SYT2//SYT9//SYT14//PURA//DAPK3//HLTF//ASF1B//SATB2//HMGB3//FOXP3//RPP14//CELF4//SRSF9//TIMM23//TOMM34//TIMM22//SLC12A7//AQP1//BIN2//LRRK2//SIRT3//MYOM3//LMOD1//BRK1//CGNL1//TLN1//FAM160A2//PARD6B//ITGA5//TTK//DYNC1I1//MAPRE1//CENPO//CHTF8//NUP214//CDKN1B//DAZL//CHRNA3//SMAD1//ZMIZ1//FOSL1//FGFR2//RTN4//LRRN1//EFNA3//NCAM1//VLDLR//SHANK3//UNC5A//DBNL//BSN//UNC13B//P2RX2//CACNA2D2//FERMT2//CDC42EP1//KIT//PARVA//ACTR3//ACTR2//UBXN10//EHD3//MCIDAS//RFX4//ONECUT2//SKP1//RAG1//UBE2E1//DPP4//SFRP1//ZNF804A//TRIM67//PEX5//NFS1//FBXO45//FAM92B//ARHGEF4//ARHGEF6//TOMM40L//THBS1//JAM3//EIF4G2//CDKN2A//BMP10//PML//WNT11//ARHGAP40//EPS8L2//MAPKAP1//PARG//PAXBP1//NREP//CCDC88C//UBE4B//NPTXR//GNAO1//NPTX2//TBC1D24//PRDM12//RAF1//NPHS2//NEK7//TNKS2//TRABD2B//REEP2//DIXDC1//IL1B//FERMT3//LIMS2//DNAJB12//KMT2E//PRKAA1//CCDC103//FZD3//GPR3//KCTD18//ALAD//HMOX1//KCNA6//TGM2//SHKBP1//OGT//PRDM4//KRT2//KRT9//KCNIP2//SEMA7A//EDN1//CEP85//GOSR2//SEMA5B//PPP3CB//RGMA//SEMA4G//SKIL//TIAM1//TLX2//NFATC4//CUX1//CTNND2//MED6//CTH//DPYS//CRTC1//KCNJ12//CD79A//GRIN2B//CD74//PRKAB1//YWHAB//PAX5//NPTX1//TGOLN2//SPRY3//ANO4//ANO3//PPIH//TEAD1//TRIM21//RASSF1//TET3//MYO19//MYBL2//ATP6V1G2//DLX5//GRIPAP1//SLC25A5//IL4R//TNFSF14//ZSWIM6//CAMKK2//UBE2D3//FZD2//ABCC8//SNX19//ARHGDIB//PIP4K2C// |
| GO:0006812 | cation transport | Biological process | 48 | 1109 | 509 | 17653 | 1.50110278291032 | 0.00321632368768012 | 0.152895349136764 | 2.49264025075089 | 0.0943025540275049 | EDN1//NKAIN1//CDKN1B//KCNIP2//AQP1//KCNA6//KCNJ12//ABCC8//KCNQ4//SCN3B//SCN1A//SCN7A//SLC5A3//SLC10A7//SLC5A6//SLC23A2//NDFIP1//SLC39A2//SLC39A8//SLC12A7//ATP1B4//PML//P2RX2//PRSS8//CHRNA3//STEAP2//SLC6A3//SLC25A2//ATP6V1G2//BEST1//GNAO1//CACNG1//CACNA2D2//SLC30A6//KCNIP3//KCNQ5//SLC9A5//GRIN2B//HTR3E//RALBP1//OXSR1//EHD3//SLMAP//SESTD1//DLG4//SHISA9//SHANK3//GLRX// |
| GO:0032970 | regulation of actin filament-based process | Biological process | 21 | 377 | 509 | 17653 | 1.93187349199814 | 0.00322055881565622 | 0.152895349136764 | 2.49206876510559 | 0.0412573673870334 | BRK1//WAS//ARHGAP40//EPS8L2//CDC42EP1//LMOD1//SHANK3//RHOF//DIXDC1//ACTR3//ACTR2//ARFIP2//DAPK3//NF2//SFRP1//WNT11//SPTBN2//EDN1//BMP10//JAM3//ARHGDIB// |
| GO:0032147 | activation of protein kinase activity | Biological process | 19 | 328 | 509 | 17653 | 2.00900258757008 | 0.00324656684600807 | 0.152895349136764 | 2.48857565068324 | 0.037328094302554 | TAB1//LRRK2//RAF1//IL1B//KIT//PRKAA1//THBS1//PROK1//CD74//CD81//CHRNA3//TIRAP//MAP3K9//DBNL//MAPKAP1//EMP2//PAK2//WNT11//OXSR1// |
| GO:0010638 | positive regulation of organelle organization | Biological process | 31 | 637 | 509 | 17653 | 1.68780784189148 | 0.00327178553904682 | 0.152895349136764 | 2.48521517146192 | 0.0609037328094303 | CDC42EP1//LMOD1//PAXBP1//CDKN1B//MAPRE1//PML//NEK7//TNKS2//IL1B//ACTR3//ACTR2//ARFIP2//FOXP3//DAZL//EDN1//WAS//NF2//SFRP1//WNT11//PRDM12//BMP10//OGT//YWHAB//LRRK2//SEPT7//CAMKK2//PRKAA1//UBE2D3//KMT2E//BRK1//PIP4K2C// |
| GO:0031346 | positive regulation of cell projection organization | Biological process | 20 | 353 | 509 | 17653 | 1.96497047479644 | 0.00329937778562764 | 0.152895349136764 | 2.48156795398485 | 0.0392927308447937 | BRK1//TRIM67//ZNF804A//CDC42EP1//KIT//SEMA7A//RTN4//SKIL//TIAM1//CUX1//PALM//SHANK3//ACTR2//DLG4//CRTC1//PACSIN1//VLDLR//EPS8L2//SEPT7//SYT2// |
| GO:0061082 | myeloid leukocyte cytokine production | Biological process | 4 | 22 | 509 | 17653 | 6.3057688873013 | 0.00330592160975797 | 0.152895349136764 | 2.48070744866329 | 0.00785854616895874 | KIT//HMOX1//SEMA7A//CD74// |
| GO:0002698 | negative regulation of immune effector process | Biological process | 9 | 105 | 509 | 17653 | 2.97271961829919 | 0.00333295294433369 | 0.152895349136764 | 2.47717081780076 | 0.0176817288801572 | PPP3CB//FOXP3//CD96//HMOX1//RNF125//BCR//IL4R//TNFSF4//NDFIP1// |
| GO:0045010 | actin nucleation | Biological process | 6 | 51 | 509 | 17653 | 4.08020339766555 | 0.00334592808993657 | 0.152895349136764 | 2.47548339704946 | 0.0117878192534381 | ACTR3//ACTR2//ARFIP2//WAS//BRK1//LMOD1// |
| GO:0060997 | dendritic spine morphogenesis | Biological process | 6 | 51 | 509 | 17653 | 4.08020339766555 | 0.00334592808993657 | 0.152895349136764 | 2.47548339704946 | 0.0117878192534381 | LRRK2//SHANK3//ACTR2//TIAM1//CTNND2//DLG4// |
| GO:0033555 | multicellular organismal response to stress | Biological process | 7 | 68 | 509 | 17653 | 3.57017797295736 | 0.00337713319148633 | 0.153285582375987 | 2.47145181079282 | 0.0137524557956778 | DPP4//USP46//FBXL20//CCK//P2RX2//THBS1//RAG1// |
| GO:0051049 | regulation of transport | Biological process | 72 | 1821 | 509 | 17653 | 1.37127099361412 | 0.00347048115584903 | 0.154838609934519 | 2.45961030940396 | 0.141453831041257 | NKAIN1//DLG4//LRRTM1//CD74//EDN1//SYT9//DPP4//SLC25A5//IL1B//PPP3CB//ABCC8//CACNA2D2//AQP1//PML//P2RX2//THBS1//SCN3B//PRSS8//UNC13B//RSC1A1//CCK//CHRNA3//CPLX1//LGI3//SYT2//SYT14//PACSIN1//SIRT3//CYP4F2//NFKB1//REEP2//NDFIP1//SGK2//LRRK2//MED1//KCNA6//KCNJ12//KCNQ5//SCN1A//SCN7A//CACNG1//KCNQ4//VSNL1//CHRM1//HMOX1//IL4R//BCR//PTAFR//SFRP1//NUP214//CPLX3//FOXP3//TNFSF4//BEST1//GNAO1//PRKAA1//TIAM1//SH3GL1//YWHAB//OXSR1//KCNIP3//KCNIP2//EHD3//SLMAP//NUCB1//UBE2D3//SESTD1//SHISA9//SHANK3//PAX8//GLRX//CA7// |
| GO:0046879 | hormone secretion | Biological process | 18 | 306 | 509 | 17653 | 2.04010169883277 | 0.00350291185752626 | 0.154838609934519 | 2.45557079053603 | 0.0353634577603143 | SYT9//DPP4//SLC25A5//IL1B//PPP3CB//ABCC8//CACNA2D2//CHRNA3//EDN1//CPLX1//SNX19//CPLX3//SIRT3//RAF1//VSNL1//SFRP1//TIAM1//PAX8// |
| GO:0034314 | Arp2/3 complex-mediated actin nucleation | Biological process | 5 | 36 | 509 | 17653 | 4.81690678891072 | 0.00352047987464457 | 0.154838609934519 | 2.45339813404869 | 0.00982318271119843 | ARFIP2//BRK1//WAS//ACTR3//ACTR2// |
| GO:0048167 | regulation of synaptic plasticity | Biological process | 12 | 168 | 509 | 17653 | 2.47726634858265 | 0.00352282369687318 | 0.154838609934519 | 2.45310909096325 | 0.0235756385068762 | UNC13B//CNTN2//DLG4//KIT//SHANK3//SHISA9//GRIN2B//LRRTM1//CRTC1//NRGN//NFATC4//PPP3CB// |
| GO:0007409 | axonogenesis | Biological process | 24 | 456 | 509 | 17653 | 1.82535415158722 | 0.00352582373649504 | 0.154838609934519 | 2.45273940280839 | 0.0471512770137525 | EFNA3//NCAM1//SPTBN2//CNTN2//VLDLR//SHANK3//UNC5A//FBXO45//TTL//RTN4//FZD3//SEMA7A//SEMA5B//PPP3CB//SEMA4G//SKIL//TIAM1//NPTX1//DLX5//ZSWIM6//FGFR2//LRRN1//PARD6B//CCK// |
| GO:0000187 | activation of MAPK activity | Biological process | 11 | 147 | 509 | 17653 | 2.59523141280088 | 0.00359181895997655 | 0.156719171782719 | 2.44468556138798 | 0.0216110019646365 | DBNL//MAP3K9//TAB1//LRRK2//IL1B//KIT//PRKAA1//THBS1//PROK1//CD74//CD81// |
| GO:1900006 | positive regulation of dendrite development | Biological process | 7 | 69 | 509 | 17653 | 3.51843626320435 | 0.00366724454456015 | 0.158171146743351 | 2.43566012881444 | 0.0137524557956778 | CUX1//SHANK3//ACTR2//TIAM1//CRTC1//PACSIN1//VLDLR// |
| GO:0048168 | regulation of neuronal synaptic plasticity | Biological process | 6 | 52 | 509 | 17653 | 4.00173794771044 | 0.0036921573988416 | 0.158171146743351 | 2.43271979302227 | 0.0117878192534381 | DLG4//KIT//SHANK3//UNC13B//SHISA9//CNTN2// |
| GO:0034341 | response to interferon-gamma | Biological process | 13 | 191 | 509 | 17653 | 2.36053652063897 | 0.00369525967550634 | 0.158171146743351 | 2.43235503719422 | 0.0255402750491159 | NCAM1//PML//TRIM68//PTAFR//TRIM21//TRIM5//MED1//ACTR3//ACTR2//DAPK3//EDN1//MRC1//WAS// |
| GO:0021537 | telencephalon development | Biological process | 15 | 237 | 509 | 17653 | 2.19504613165552 | 0.00377681329110135 | 0.16064520935672 | 2.42287448423487 | 0.0294695481335953 | AQP1//PAX5//UCHL5//LRRK2//NF2//SHANK3//FBXO45//DIXDC1//RTN4//CNTN2//DLX5//ASCL1//SEMA7A//NRGN//RFX4// |
| GO:0033674 | positive regulation of kinase activity | Biological process | 29 | 590 | 509 | 17653 | 1.70469514834671 | 0.00380876841163631 | 0.160991879799352 | 2.41921543337899 | 0.0569744597249509 | TAB1//LRRK2//RAF1//IL1B//KIT//PRKAA1//THBS1//PROK1//CD74//CD81//PARP16//CHRNA3//TIRAP//MAP3K9//DBNL//EMP2//PAK2//WNT11//OXSR1//MAPKAP1//EDN1//S100A12//TIAM1//FGFR3//CDKN1B//CAMKK2//VLDLR//DLG4//FGFR2// |
| GO:0016079 | synaptic vesicle exocytosis | Biological process | 8 | 88 | 509 | 17653 | 3.15288444365065 | 0.00386299290781177 | 0.162269695872863 | 2.41307608918913 | 0.0157170923379175 | UNC13B//SNPH//SYT2//SYT9//SYT14//LRRK2//CPLX1//CPLX3// |
| GO:2000191 | regulation of fatty acid transport | Biological process | 4 | 23 | 509 | 17653 | 6.03160502263603 | 0.00391190292671354 | 0.16314122494763 | 2.40761193070338 | 0.00785854616895874 | THBS1//CYP4F2//EDN1//IL1B// |
| GO:0051051 | negative regulation of transport | Biological process | 24 | 460 | 509 | 17653 | 1.80948150679081 | 0.00393198575579827 | 0.16314122494763 | 2.40538806413324 | 0.0471512770137525 | DLG4//LRRTM1//CD74//THBS1//IL1B//RSC1A1//CYP4F2//NFKB1//NDFIP1//HMOX1//BCR//PACSIN1//SFRP1//ABCC8//VSNL1//EDN1//FOXP3//PML//GNAO1//OXSR1//SLC25A5//LRRK2//SESTD1//PRKAA1// |
| GO:0042596 | fear response | Biological process | 5 | 37 | 509 | 17653 | 4.68672011894016 | 0.00397579375651217 | 0.163953007166413 | 2.40057615262781 | 0.00982318271119843 | DPP4//USP46//FBXL20//CCK//RAG1// |
| GO:0023061 | signal release | Biological process | 23 | 435 | 509 | 17653 | 1.83374658446808 | 0.00400893889476209 | 0.164317901486521 | 2.39697056332291 | 0.0451866404715128 | UNC13B//CPLX1//SNPH//SYT9//DPP4//SLC25A5//IL1B//PPP3CB//ABCC8//CACNA2D2//CHRNA3//SYT2//SYT14//CPLX3//EDN1//SNX19//SIRT3//RAF1//VSNL1//SFRP1//TIAM1//LRRK2//PAX8// |
| GO:0043550 | regulation of lipid kinase activity | Biological process | 6 | 53 | 509 | 17653 | 3.926233458131 | 0.00406427249098485 | 0.164590867404374 | 2.3910171822783 | 0.0117878192534381 | CD81//WDR91//FGFR3//KIT//PIK3IP1//NRBF2// |
| GO:0071398 | cellular response to fatty acid | Biological process | 6 | 53 | 509 | 17653 | 3.926233458131 | 0.00406427249098485 | 0.164590867404374 | 2.3910171822783 | 0.0117878192534381 | PRKAA1//SFRP1//TNFSF4//NFATC4//EDN1//PTAFR// |
| GO:0016358 | dendrite development | Biological process | 14 | 216 | 509 | 17653 | 2.247889834825 | 0.00409775393107945 | 0.164958987118395 | 2.38745412440797 | 0.0275049115913556 | VLDLR//CHRNA3//TLX2//NFATC4//CUX1//PAK2//CTNND2//DLG4//SHANK3//LRRK2//ACTR2//TIAM1//CRTC1//PACSIN1// |
| GO:0030001 | metal ion transport | Biological process | 39 | 868 | 509 | 17653 | 1.55828044507619 | 0.00415500362299444 | 0.166273902380541 | 2.38142859319255 | 0.0766208251473477 | NKAIN1//CDKN1B//KCNIP2//AQP1//KCNA6//KCNJ12//ABCC8//KCNQ4//SCN3B//SCN1A//SCN7A//SLC5A3//SLC10A7//SLC5A6//SLC23A2//SLC39A2//SLC39A8//SLC12A7//ATP1B4//PML//P2RX2//PRSS8//STEAP2//ATP6V1G2//BEST1//GNAO1//CACNG1//CACNA2D2//SLC30A6//KCNIP3//KCNQ5//SLC9A5//GRIN2B//OXSR1//EHD3//SLMAP//SESTD1//GLRX//NDFIP1// |
| GO:0051495 | positive regulation of cytoskeleton organization | Biological process | 14 | 217 | 509 | 17653 | 2.23753089549401 | 0.00426837316347386 | 0.16973712352609 | 2.36973761929603 | 0.0275049115913556 | CDC42EP1//LMOD1//CDKN1B//MAPRE1//ACTR3//ACTR2//ARFIP2//WAS//NF2//SFRP1//WNT11//EDN1//BMP10//BRK1// |
| GO:0008306 | associative learning | Biological process | 7 | 71 | 509 | 17653 | 3.41932538255071 | 0.00430388338475046 | 0.16973712352609 | 2.36613950454555 | 0.0137524557956778 | CTNS//KIT//RAG1//ABCC8//ACTR2//NPTX2//NRGN// |
| GO:0033631 | cell-cell adhesion mediated by integrin | Biological process | 3 | 12 | 509 | 17653 | 8.67043222003929 | 0.00431683945682204 | 0.16973712352609 | 2.36483410244699 | 0.00589390962671906 | DPP4//FERMT3//ITGA5// |
| GO:0042391 | regulation of membrane potential | Biological process | 23 | 438 | 509 | 17653 | 1.82118667635529 | 0.00435507231899925 | 0.170250601695907 | 2.36100462883507 | 0.0451866404715128 | CTNS//SCN1A//SCN7A//GLRX//EDN1//SCN3B//LRRK2//CCK//CHRNA3//P2RX2//GRIN2B//KCNIP2//EHD3//CELF4//UNC13B//SH3GL1//SLMAP//CRTC1//DLG4//SHANK3//SLC26A1//HTR3E//CHRFAM7A// |
| GO:0002820 | negative regulation of adaptive immune response | Biological process | 5 | 38 | 509 | 17653 | 4.56338537896805 | 0.004471691515473 | 0.170923442176329 | 2.3495281643678 | 0.00982318271119843 | PPP3CB//FOXP3//IL4R//TNFSF4//NDFIP1// |
| GO:0007223 | Wnt signaling pathway, calcium modulating pathway | Biological process | 5 | 38 | 509 | 17653 | 4.56338537896805 | 0.004471691515473 | 0.170923442176329 | 2.3495281643678 | 0.00982318271119843 | FZD2//GNAO1//PPP3CB//WNT11//FZD3// |
| GO:0098815 | modulation of excitatory postsynaptic potential | Biological process | 5 | 38 | 509 | 17653 | 4.56338537896805 | 0.004471691515473 | 0.170923442176329 | 2.3495281643678 | 0.00982318271119843 | LRRK2//CELF4//DLG4//SHANK3//SH3GL1// |
| GO:0051270 | regulation of cellular component movement | Biological process | 43 | 985 | 509 | 17653 | 1.51402471253478 | 0.00447337709081919 | 0.170923442176329 | 2.349364490703 | 0.0844793713163065 | HMOX1//BCR//EMP2//EDN1//THBS1//VASH1//BMP10//RTN4//TNFSF14//TRIB1//KIF2A//RHOF//BEX4//PARD6B//GPNMB//DAPK3//MAPRE1//FSHB//IL1B//ITGA5//KIT//SEMA4G//TIAM1//WNT11//FERMT3//SEMA7A//ONECUT2//PTPRU//NF2//SFRP1//CDKN1B//ABCC8//JAM3//TIRAP//CD74//ARHGDIB//TAC4//ZSWIM6//PTAFR//RAF1//CD81//WAS//CXCL14// |
| GO:0006688 | glycosphingolipid biosynthetic process | Biological process | 4 | 24 | 509 | 17653 | 5.78028814669286 | 0.00458881980480736 | 0.174349372696136 | 2.33829899598029 | 0.00785854616895874 | ST6GALNAC6//GAL3ST1//PRKAA1//ST8SIA5// |
| GO:0051056 | regulation of small GTPase mediated signal transduction | Biological process | 19 | 339 | 509 | 17653 | 1.94381371304716 | 0.00463098761936017 | 0.174968543406329 | 2.3343263800622 | 0.037328094302554 | IQSEC3//RALGPS2//OGT//ARHGDIB//ARHGEF4//RAF1//BCR//EPS8L2//TIAM1//ARHGEF6//SPRY3//TRIM67//MAPKAP1//CGNL1//FAM13A//RALBP1//ARHGAP40//RHOF//RALGAPB// |
| GO:0009790 | embryo development | Biological process | 42 | 959 | 509 | 17653 | 1.51890783416747 | 0.00467298687793806 | 0.175574501419417 | 2.33040543850213 | 0.0825147347740668 | TAB1//EDN1//FGFR2//BRK1//ZMIZ1//WDR19//FOSL1//TLX2//NF2//SMAD1//SKIL//TAF8//FZD3//MED1//CCDC103//MICAL2//SATB2//CELF4//ZNF281//SFRP1//ALDH1A2//SEPT7//DLX5//KIT//ITGA5//TPRA1//INSIG2//BCR//PAX8//KCNQ4//TET3//EXOC4//WNT11//TEAD1//C2ORF49//PAX5//HOXC9//HOXC6//VASH1//HS6ST1//FZD2//DLG4// |
| GO:0051338 | regulation of transferase activity | Biological process | 43 | 988 | 509 | 17653 | 1.50942747150482 | 0.00471777081611504 | 0.17627781231705 | 2.32626316046487 | 0.0844793713163065 | CDKN1B//TAB1//LRRK2//RAF1//IL1B//KIT//PRKAA1//THBS1//PROK1//CD74//CD81//TRIB1//LRRTM1//NF2//PAK2//CEP85//PARP16//CHRNA3//TIRAP//MAP3K9//DBNL//CDKN2A//EMP2//WNT11//OXSR1//MAPKAP1//FGFR3//FGFR2//EDN1//S100A12//SPRY3//SMPD1//TIAM1//SFRP1//NRBF2//WDR91//PIK3IP1//CAMKK2//VLDLR//NEK7//DLG4//SH3BP5//PPP1R3F// |
| GO:0045944 | positive regulation of transcription by RNA polymerase II | Biological process | 48 | 1132 | 509 | 17653 | 1.47060334474165 | 0.00474683277715036 | 0.176389176219054 | 2.3235960674685 | 0.0943025540275049 | HEYL//MICAL2//INSIG2//DLX5//SMAD1//TEAD1//CREB3L3//MED6//ARID3B//COPS5//PRDM4//EDN1//ELK1//TET3//FGFR2//SATB2//CRTC1//ATP1B4//FSHB//RAX//TLX2//MCIDAS//IL1B//PHOX2A//ASCL1//MYBL2//NFATC4//NFKB1//PAX5//FOXP3//PML//MED1//PPP3CB//RGMA//ZMIZ1//CALCOCO1//RAF1//REL//RFX4//HLTF//TCF20//PAX8//FOSL1//NRIP1//OGT//PAXBP1//ONECUT2//CD81// |
| GO:0035556 | intracellular signal transduction | Biological process | 105 | 2871 | 509 | 17653 | 1.26840178767555 | 0.00496552448661569 | 0.183507333896076 | 2.3040348721207 | 0.206286836935167 | CAMKK2//LRRK2//DLG4//FGFR3//FGFR2//GRIN2B//IL1B//KIT//SMAD1//NCAM1//MAPK4//RAF1//SPTBN2//YWHAB//SHANK3//TAB1//PRKAA1//THBS1//PROK1//CD74//CD81//CDKN1B//PML//GPR3//EDN1//NFKB1//REL//TIRAP//TRIB1//ARHGEF6//MAP3K9//DBNL//DDAH1//RALBP1//ARFIP2//HMOX1//RHOF//RALGPS2//CDKN2A//RASSF1//RALGDS//CDC42EP1//PAK2//EPS8L2//HIC1//NFATC4//PIP4K2C//PIK3IP1//BRK1//TIAM1//CCDC88C//MAPKAP1//OXSR1//LARP1//IQSEC3//RABL3//WAS//PPP3CB//OGT//ARHGDIB//ARHGEF4//BCR//TEAD1//NF2//SKP1//RNF125//CTH//CC2D1A//S100A12//TGM2//NDFIP1//TRIM5//SPRY3//SMPD1//BMP10//RNF149//SFRP1//MGRN1//COPS5//LRRTM1//TRIM67//PTAFR//P2RX2//FAM13A//ARHGAP40//RALGAPB//CGNL1//RTN4//MED1//SIRT3//GPNMB//SEMA7A//WNT11//PRKAB1//SKIL//SGK2//UNC13B//DGKG//DAPK3//CARHSP1//CD209//MARK1//POLR2M//PLA2G4C//SH3BP5// |
| GO:0006687 | glycosphingolipid metabolic process | Biological process | 7 | 73 | 509 | 17653 | 3.32564523508356 | 0.00502070954007258 | 0.184538362062559 | 2.29923490286236 | 0.0137524557956778 | ST6GALNAC6//GAL3ST1//ST8SIA5//PRKAA1//ESYT1//KIT//SMPD1// |
| GO:0002695 | negative regulation of leukocyte activation | Biological process | 11 | 154 | 509 | 17653 | 2.47726634858265 | 0.00510221550431937 | 0.185525397175221 | 2.29224120186889 | 0.0216110019646365 | GPNMB//FOXP3//NDFIP1//HMOX1//BCR//HMGB3//SFRP1//CD74//TNFSF4//IL4R//TNFAIP8L2// |
| GO:0051091 | positive regulation of DNA binding transcription factor activity | Biological process | 15 | 245 | 509 | 17653 | 2.12337115592799 | 0.00512446769289121 | 0.185525397175221 | 2.29035124052518 | 0.0294695481335953 | CRTC1//TAB1//TIRAP//CTH//IL1B//NFKB1//S100A12//TRIM5//COPS5//EDN1//FZD2//KIT//NDP//TRIM21//FOSL1// |
| GO:0006366 | transcription by RNA polymerase II | Biological process | 90 | 2405 | 509 | 17653 | 1.2978609560142 | 0.00517663744580038 | 0.185525397175221 | 2.28595225047149 | 0.176817288801572 | CUX1//EDN1//FGFR2//FOXS1//SATB2//ZNF281//DNAJB5//HEYL//KCNIP3//HIC1//ASCL1//NFKB1//PAX5//FOXP3//RLIM//MED1//CC2D1A//REL//SKIL//UBE2D3//VLDLR//NRIP1//BHLHE40//PAXBP1//LSM11//SNRPB//U2AF1//DDX39B//SRSF9//RBM8A//MED6//ELK1//FOXG1//OTP//CARHSP1//LIN9//HOXC6//HOXC9//KIT//FOXI2//MYBL2//NFYA//MBNL3//PTAFR//PURA//CREBZF//HIVEP3//PRDM12//RFX4//TCF19//ZNF322//FOSL1//OGT//CREB5//NRBF2//POLR2E//TEAD1//MICAL2//DLX5//BMP10//SMAD1//RGMA//SFRP1//INSIG2//HMOX1//TAF8//ARID3B//COPS5//PRDM4//TET3//CRTC1//ATP1B4//FSHB//RAX//TLX2//MCIDAS//IL1B//PHOX2A//NFATC4//PML//PPP3CB//ZMIZ1//CALCOCO1//RAF1//HLTF//TCF20//PAX8//CREB3L3//ONECUT2//CD81// |
| GO:0055085 | transmembrane transport | Biological process | 62 | 1550 | 509 | 17653 | 1.38726915520629 | 0.00518246995676228 | 0.185525397175221 | 2.28546320687041 | 0.121807465618861 | EBP//RALBP1//SLC12A7//ATP1B4//KCNJ12//THBS1//IL1B//RSC1A1//SLC23A2//SLC5A6//PEX5//TIMM23//TOMM40L//SGK2//CHRNA3//ANO4//DLG4//P2RX2//HTR3E//ATP6V1G2//RAF1//ANO3//BEST1//CHRFAM7A//KCNA6//KCNQ5//SCN1A//SCN7A//CACNG1//KCNQ4//CACNA2D2//AQP1//SCN3B//TIMM22//BCR//SLC39A8//KCNIP2//SLC39A2//SLC30A6//KCNIP3//SLC9A5//ABCC8//SLC35A2//GRIN2B//STEAP2//OXSR1//EHD3//SLMAP//SLC26A1//PTAFR//SLC25A2//SESTD1//SHISA9//SHANK3//GLRX//PRKAA1//CSN3//CTNS//SLC25A5//SLC5A3//SLC6A3//SLC10A7// |
| GO:0099504 | synaptic vesicle cycle | Biological process | 10 | 133 | 509 | 17653 | 2.60764878798174 | 0.00520093490931757 | 0.185525397175221 | 2.2839185814686 | 0.0196463654223969 | SH3GL1//UNC13B//CPLX1//SYT2//SYT9//SYT14//CPLX3//SNPH//PACSIN1//LRRK2// |
| GO:0009914 | hormone transport | Biological process | 18 | 318 | 509 | 17653 | 1.9631167290655 | 0.00521215813445099 | 0.185525397175221 | 2.2829824164672 | 0.0353634577603143 | SYT9//DPP4//SLC25A5//IL1B//PPP3CB//ABCC8//CACNA2D2//CHRNA3//EDN1//CPLX1//SNX19//CPLX3//SIRT3//RAF1//VSNL1//SFRP1//TIAM1//PAX8// |
| GO:0048869 | cellular developmental process | Biological process | 150 | 4323 | 509 | 17653 | 1.20339100902697 | 0.0052950125416062 | 0.186166495800932 | 2.27613300689853 | 0.294695481335953 | MED1//WDR19//DAZL//ZMIZ1//GPNMB//DLX5//TP53INP2//SFRP1//WNT11//SEMA7A//SMAD1//SEMA4G//FBXO45//SATB2//MARK1//ASCL1//FZD3//CCK//FGFR2//OTP//FGFR3//SNX19//ONECUT2//KIT//JAM3//RAG1//FOXP3//NDFIP1//HEYL//KRT2//PAX8//PHOX2A//CELF4//RTN4//LRRN1//PARD6B//EFNA3//NCAM1//SPTBN2//CNTN2//VLDLR//SHANK3//UNC5A//FERMT2//CDC42EP1//DAPK3//PALM//RHOF//PARVA//WAS//MMP24//TIAM1//IL1B//NFKB1//ZNF804A//TRIM67//LRRK2//NF2//EDN1//ALDH1A2//SEPT7//TIRAP//PML//PTPRU//TAF8//ELK1//FOXG1//FOXS1//KIF2A//FOXI2//MEA1//MYBL2//ABHD5//ASF1B//RNF114//PURA//RAF1//FZD2//SNPH//CD79A//PPP3CB//KMT2E//LMOD1//MYOM3//TTL//ALDOC//NREP//UBE4B//NPTXR//GNAO1//NPTX2//TBC1D24//PRDM12//KRT9//KRTAP1-1//KRT38//FERMT3//PAX5//HIVEP3//ITGA5//MAEA//TNFSF4//CD74//KCNIP2//BMP10//HMGB3//SKIL//FAM57B//IL4R//TRIB1//THBS1//MBNL3//CXCL14//TNFSF14//DIXDC1//FSHB//DGKG//HS6ST1//SEMA5B//RGMA//P2RX2//C11ORF88//CACNA2D2//DBNL//PACSIN1//CHRNA3//ZNF281//TLX2//NFATC4//CUX1//PTBP1//ABCC8//NPTX1//PAK2//CTNND2//DLG4//ACTR2//DDX39B//SPRY3//PRSS8//NPHS2//CDKN2A//CRTC1//ZSWIM6//CDKN1B//MCIDAS//SYT2//CTH//VASH1//EMP2// |
| GO:0045859 | regulation of protein kinase activity | Biological process | 36 | 797 | 509 | 17653 | 1.56655237099832 | 0.00533160221758802 | 0.186166495800932 | 2.27314226007416 | 0.0707269155206287 | CDKN1B//TAB1//LRRK2//RAF1//IL1B//KIT//PRKAA1//THBS1//PROK1//CD74//CD81//TRIB1//LRRTM1//NF2//PAK2//CEP85//PARP16//CHRNA3//TIRAP//MAP3K9//DBNL//CDKN2A//EMP2//WNT11//OXSR1//MAPKAP1//EDN1//S100A12//SPRY3//SMPD1//TIAM1//SFRP1//CAMKK2//VLDLR//DLG4//SH3BP5// |
| GO:0009648 | photoperiodism | Biological process | 4 | 25 | 509 | 17653 | 5.54907662082515 | 0.0053402780105546 | 0.186166495800932 | 2.2724361333634 | 0.00785854616895874 | CRTC1//FBXL3//PML//BHLHE40// |
| GO:0035640 | exploration behavior | Biological process | 4 | 25 | 509 | 17653 | 5.54907662082515 | 0.0053402780105546 | 0.186166495800932 | 2.2724361333634 | 0.00785854616895874 | LRRK2//DLG4//DPP4//LRRTM1// |
| GO:0043405 | regulation of MAP kinase activity | Biological process | 19 | 344 | 509 | 17653 | 1.91556060675287 | 0.0054046830012551 | 0.18744549301276 | 2.267229773467 | 0.037328094302554 | TAB1//LRRK2//RAF1//IL1B//KIT//PRKAA1//THBS1//PROK1//CD74//CD81//MAP3K9//DBNL//EDN1//S100A12//SPRY3//SMPD1//TIAM1//SFRP1//TRIB1// |
| GO:0035641 | locomotory exploration behavior | Biological process | 3 | 13 | 509 | 17653 | 8.00347589542089 | 0.00549280182761217 | 0.18952968755174 | 2.26020606941611 | 0.00589390962671906 | LRRK2//DLG4//DPP4// |
| GO:0010647 | positive regulation of cell communication | Biological process | 68 | 1737 | 509 | 17653 | 1.35771880474996 | 0.0056583404859377 | 0.193522490622921 | 2.24731092278309 | 0.133595284872299 | TAB1//LRRK2//RAF1//IL1B//KIT//PRKAA1//THBS1//PROK1//CD74//CD81//AGPAT1//TIRAP//MAP3K9//DBNL//UNC13B//BMP10//TTK//EDN1//FGFR2//SFRP1//ITGA5//SIRT3//CA7//MED1//WDFY1//PPP3CB//VSNL1//FGFR3//CTH//HMOX1//CC2D1A//REL//S100A12//TGM2//NDFIP1//TRIM5//TIAM1//HIC1//SHKBP1//ENHO//ASCL1//ZMIZ1//TSPAN14//EVC//DLG4//P2RX2//RTN4//SHANK3//CNTN2//GRIN2B//LRRTM1//GPNMB//SEMA7A//WNT11//DAPK3//DLX5//NFKB1//TNKS2//DIXDC1//CRTC1//NRGN//YWHAB//SKIL//EMP2//LIMS2//NFATC4//PAK2//PML// |
| GO:0031098 | stress-activated protein kinase signaling cascade | Biological process | 17 | 296 | 509 | 17653 | 1.99185605054957 | 0.00566574791414142 | 0.193522490622921 | 2.24674275242992 | 0.0333988212180747 | TRIB1//TAB1//ARHGEF6//MAP3K9//DBNL//EDN1//TIAM1//SFRP1//COPS5//TIRAP//IL1B//NFKB1//SKP1//CCDC88C//PAK2//MAPKAP1//OXSR1// |
| GO:0051641 | cellular localization | Biological process | 101 | 2761 | 509 | 17653 | 1.26869055302277 | 0.00588067118481925 | 0.19985416694941 | 2.23057310330683 | 0.198428290766208 | SPRN//PML//NUP214//PLLP//SH3GL1//EXOC4//U2AF1//DDX39B//SRSF9//RBM8A//YWHAB//GOLGA7B//KDELR3//GOSR2//TIMM23//TOMM34//TIMM22//VPS45//AP1S1//ARFIP2//RABL3//KCNIP3//MLPH//VPS26A//CD74//DYNC1I1//SPTBN2//TGOLN2//SYS1//TLN1//UNC13B//CPLX1//SNPH//MGRN1//FAM160A2//P2RX2//CHRNA3//SYT2//SYT9//SYT14//CPLX3//WAS//ACTR3//ACTR2//PEX5//TOMM40L//CCDC88C//REEP2//ARL4C//EHD3//VPS53//LRRK2//MED1//PTPRU//EMP2//WNT11//TTK//MIS12//SEPT7//MYO19//MAPRE1//WDR19//RAF1//PPP3CB//VSNL1//DLG4//KIT//HMOX1//IL4R//WDR91//IL1B//PACSIN1//SNRPB//TAF8//SKP1//SLC6A3//POLR2M//PARD6B//TIAM1//CD81//TULP4//ESYT1//BCR//TNKS2//CNTN2//FERMT2//PALM//SCN3B//SLMAP//TSPAN14//TTL//CHRM1//SHANK3//GRIN2B//GRIPAP1//NF2//NUCB1//TP53INP2//PRKAA1//UBE2D3//RTN4// |
| GO:0023056 | positive regulation of signaling | Biological process | 68 | 1743 | 509 | 17653 | 1.35304507392466 | 0.0061170163618525 | 0.206846908276042 | 2.21346035784571 | 0.133595284872299 | TAB1//LRRK2//RAF1//IL1B//KIT//PRKAA1//THBS1//PROK1//CD74//CD81//AGPAT1//TIRAP//MAP3K9//DBNL//UNC13B//BMP10//TTK//EDN1//FGFR2//SFRP1//ITGA5//SIRT3//CA7//MED1//WDFY1//PPP3CB//VSNL1//FGFR3//CTH//HMOX1//CC2D1A//REL//S100A12//TGM2//NDFIP1//TRIM5//TIAM1//HIC1//SHKBP1//ENHO//ASCL1//ZMIZ1//TSPAN14//EVC//DLG4//P2RX2//RTN4//SHANK3//CNTN2//GRIN2B//LRRTM1//GPNMB//SEMA7A//WNT11//DAPK3//DLX5//NFKB1//TNKS2//DIXDC1//CRTC1//NRGN//YWHAB//SKIL//EMP2//LIMS2//NFATC4//PAK2//PML// |
| GO:0051865 | protein autoubiquitination | Biological process | 6 | 58 | 509 | 17653 | 3.58776505656798 | 0.0063528862018519 | 0.212695888035269 | 2.19702892403675 | 0.0117878192534381 | LRRK2//UBE4B//TRIM68//RAG1//TRIM21//UBE2D3// |
| GO:0099175 | regulation of postsynapse organization | Biological process | 6 | 58 | 509 | 17653 | 3.58776505656798 | 0.0063528862018519 | 0.212695888035269 | 2.19702892403675 | 0.0117878192534381 | LRRK2//SHANK3//ACTR2//TIAM1//GRIN2B//ZNF804A// |
| GO:0017156 | calcium ion regulated exocytosis | Biological process | 9 | 116 | 509 | 17653 | 2.69082379242599 | 0.00641525125427965 | 0.213725833658588 | 2.1927863297473 | 0.0176817288801572 | UNC13B//CPLX1//SYT2//SYT9//SYT14//CPLX3//SNPH//LRRK2//PPP3CB// |
| GO:0010498 | proteasomal protein catabolic process | Biological process | 23 | 453 | 509 | 17653 | 1.76088248177398 | 0.00648178014040772 | 0.21488372102734 | 2.18830570416266 | 0.0451866404715128 | UCHL5//UBE4B//UBE2E1//FBXL3//SKP1//FBXL20//OGT//TRIB1//LRRK2//RNF217//RNF125//RNF114//DNAJB12//MAEA//UBXN2B//RNF38//UBXN2A//FBXO45//RLIM//PML//UBE2D3//ALAD//EDEM3// |
| GO:0051493 | regulation of cytoskeleton organization | Biological process | 25 | 506 | 509 | 17653 | 1.71352415415796 | 0.00651983899860099 | 0.21509107876848 | 2.18576312863656 | 0.0491159135559921 | BRK1//WAS//ARHGAP40//EPS8L2//CDC42EP1//LMOD1//MAPRE1//CDKN1B//SHANK3//RHOF//DIXDC1//ACTR3//ACTR2//ARFIP2//DAPK3//CEP85//NF2//SFRP1//WNT11//SPTBN2//EDN1//BMP10//RASSF1//JAM3//ARHGDIB// |
| GO:0048568 | embryonic organ development | Biological process | 22 | 428 | 509 | 17653 | 1.78270569010154 | 0.00666875684541471 | 0.218935934687086 | 2.17595511743212 | 0.0432220039292731 | MED1//CCDC103//MICAL2//FGFR2//WDR19//ALDH1A2//KIT//DLX5//INSIG2//BCR//PAX8//FZD3//KCNQ4//EDN1//WNT11//PAX5//SATB2//HOXC9//VASH1//HS6ST1//FZD2//TEAD1// |
| GO:0071560 | cellular response to transforming growth factor beta stimulus | Biological process | 14 | 229 | 509 | 17653 | 2.12028036821922 | 0.0068026309160822 | 0.22055594812399 | 2.16732309150557 | 0.0275049115913556 | TAB1//FERMT2//FSHB//SMAD1//PML//SKIL//NREP//THBS1//HTRA4//RASL11B//ONECUT2//EDN1//FGFR2//SFRP1// |
| GO:0030950 | establishment or maintenance of actin cytoskeleton polarity | Biological process | 3 | 14 | 509 | 17653 | 7.43179904574797 | 0.00684271014751805 | 0.22055594812399 | 2.16477185602876 | 0.00589390962671906 | AQP1//RHOF//MAPKAP1// |
| GO:0045623 | negative regulation of T-helper cell differentiation | Biological process | 3 | 14 | 509 | 17653 | 7.43179904574797 | 0.00684271014751805 | 0.22055594812399 | 2.16477185602876 | 0.00589390962671906 | IL4R//TNFSF4//FOXP3// |
| GO:0030154 | cell differentiation | Biological process | 143 | 4124 | 509 | 17653 | 1.20259147183862 | 0.00684855080674818 | 0.22055594812399 | 2.1644013180284 | 0.280943025540275 | DAZL//ZMIZ1//GPNMB//DLX5//TP53INP2//SFRP1//WNT11//SEMA7A//SMAD1//SEMA4G//FBXO45//SATB2//MARK1//ASCL1//FZD3//CCK//FGFR2//OTP//FGFR3//SNX19//ONECUT2//KIT//JAM3//RAG1//FOXP3//NDFIP1//HEYL//KRT2//PAX8//PHOX2A//CELF4//RTN4//LRRN1//PARD6B//EFNA3//NCAM1//SPTBN2//CNTN2//VLDLR//SHANK3//UNC5A//MMP24//TIAM1//IL1B//NFKB1//ZNF804A//TRIM67//LRRK2//NF2//EDN1//ALDH1A2//TIRAP//PML//DAPK3//FZD2//SNPH//CD79A//MED1//PPP3CB//KMT2E//LMOD1//MYOM3//TTL//ALDOC//NREP//UBE4B//NPTXR//GNAO1//NPTX2//TBC1D24//PRDM12//KRT9//KRTAP1-1//KRT38//FERMT2//PARVA//FERMT3//PAX5//HIVEP3//ITGA5//MAEA//TNFSF4//CD74//KCNIP2//BMP10//HMGB3//RAF1//SKIL//TAF8//FAM57B//IL4R//TRIB1//THBS1//MBNL3//CXCL14//TNFSF14//DIXDC1//FSHB//DGKG//HS6ST1//SEMA5B//RGMA//P2RX2//C11ORF88//CACNA2D2//DBNL//PACSIN1//CHRNA3//ZNF281//TLX2//NFATC4//CUX1//PTBP1//ABCC8//NPTX1//PAK2//CTNND2//DLG4//ACTR2//DDX39B//SPRY3//PRSS8//NPHS2//CRTC1//ZSWIM6//CDKN1B//MCIDAS//SYT2//CTH//EMP2//PTPRU//ELK1//FOXG1//FOXS1//KIF2A//FOXI2//MEA1//MYBL2//ABHD5//ASF1B//RNF114//PURA//SEPT7// |
| GO:1903793 | positive regulation of anion transport | Biological process | 5 | 42 | 509 | 17653 | 4.12877724763776 | 0.00689879951607338 | 0.221121237569688 | 2.16122647577064 | 0.00982318271119843 | CCK//CYP4F2//EDN1//IL1B//PTAFR// |
| GO:0050794 | regulation of cellular process | Biological process | 346 | 11074 | 509 | 17653 | 1.0836082890134 | 0.00699206647379104 | 0.22305351680306 | 2.15539445150303 | 0.679764243614931 | CDKN1B//CUX1//EDN1//FGFR2//FOXS1//SATB2//ZNF281//DNAJB5//HEYL//KCNIP3//HIC1//ASCL1//NFKB1//PAX5//FOXP3//RLIM//MED1//CC2D1A//REL//SKIL//UBE2D3//VLDLR//NRIP1//BHLHE40//PAXBP1//CAMKK2//LRRK2//DLG4//FGFR3//GRIN2B//IL1B//KIT//SMAD1//NCAM1//MAPK4//RAF1//SPTBN2//YWHAB//SHANK3//TAB1//PRKAA1//THBS1//PROK1//CD74//CD81//RBFOX1//MBNL3//CELF4//PTBP1//RBM8A//SGK2//HTRA4//CCK//CCDC88C//PML//GPNMB//ALDH1A2//VASH1//CDKN2A//NF2//AGPAT1//OTP//FZD3//LRRTM1//TRIM5//CD209//PAK2//SKP1//HMOX1//BCR//TIRAP//NDFIP1//WNT11//COPS5//LIN9//MYBL2//NUP214//FERMT2//CTNND2//TRABD2B//MARK1//NDP//CALCOCO1//TNKS2//KREMEN1//VPS26A//FZD2//GNAO1//PPP3CB//FAM13A//EBP//RALBP1//PRDM4//PTPRT//AKAP10//CHRM1//CHRNA3//DGKG//FGF11//FSHB//HTR3E//KCNIP2//ARHGAP40//IL4R//IMPA2//LGALS3BP//MRC1//NRGN//PRKAB1//HIVEP3//SH3GL1//RASL11B//SMPD1//ABCC8//TNFSF4//RERGL//OGT//TNFSF14//CHRFAM7A//SH3BP5//CXCL14//UNC13B//DAPK3//CARHSP1//ARHGEF4//POLR2M//PLA2G4C//OXSR1//PPP1R3F//GLRX//MCIDAS//USP37//PARG//KRBOX1//ZNF618//EWSR1//ZBTB43//ATP1B4//MYCBP//NRBF2//HMGB3//MAP3K9//NFYA//PHF20L1//UCHL5//PFDN1//ASF1B//GPBP1L1//RSC1A1//HLTF//TCF20//ZNF322//MED6//ELK1//FOXG1//HOXC6//HOXC9//FOXI2//PTAFR//PURA//CREBZF//PRDM12//RFX4//TCF19//FOSL1//CREB5//RBM3//EIF4G2//TRIB1//CEP85//PARP16//RASSF1//KMT2E//TTK//KLHL22//ARHGDIB//CLEC1A//TSPAN14//TSPAN18//PTPRU//TPRA1//GPR160//GNAZ//GPR34//GRM2//SFRP1//TIAM1//ARHGEF6//GPR3//PALM//TAC4//EVC//ITGA5//TLN1//FERMT3//SEMA7A//DBNL//DDAH1//ARFIP2//RHOF//RALGPS2//RALGDS//CDC42EP1//EPS8L2//MAEA//NEK7//TTC28//PIM1//SYT9//DPP4//SLC25A5//CACNA2D2//BRK1//WAS//DDX39B//AQP1//PAFAH2//LIMS2//EMP2//PGGT1B//LDOC1//DEC1//ATP6V1G2//PARVA//GALNT3//POLR2E//UBE4B//NFATC4//CASP7//SPRY3//PPP1R2//RGS9BP//MAPRE1//BMP10//DRAM1//PIP4K2C//TP53INP2//TRIM21//DLAT//P2RX2//RTN4//PAX8//SIRT3//CTNS//MICAL2//ABHD5//PPP1R26//CNTN2//ZNF804A//TRIM67//PIK3IP1//LARP1//RBMS3//EXOC4//MGRN1//FBXO10//TNFAIP8L2//RELT//SEPT7//TAF8//DLX5//TEAD1//PASD1//NREP//MEX3B//CPLX1//LGI3//SYT2//SYT14//PACSIN1//KIF2A//BEX4//PARD6B//SEMA4G//ONECUT2//RGMA//TTL//LMOD1//CTH//EDEM3//CREB3L3//MAPKAP1//UBXN2A//IQSEC3//USP46//CA7//RNF217//RNF125//RNF114//MYO19//RABL3//CRTC1//INSIG2//DIXDC1//WDFY1//ACTR3//ACTR2//VSNL1//KDELR3//ASNA1//GOSR2//UNC5A//PTPN18//SLC6A3//UBE2Z//TGM2//S100A12//RAG1//RNF149//WDR91//UBXN2B//ZMIZ1//FAM57B//EFNA3//SHKBP1//ENHO//DAZL//ARID3B//TET3//RAX//TLX2//PHOX2A//CPLX3//SRSF9//SHISA9//CD79A//RALGAPB//CGNL1//NOVA1//LRRN1//TRIM68//SCN3B//WDR19//SLC30A6//JAM3//BSN//LYPD6//LSM11//SNRPA//SLMAP//PEX5//ALAD//ZSWIM6//EHD3//FAM168A//GRIPAP1// |
| GO:0099003 | vesicle-mediated transport in synapse | Biological process | 10 | 139 | 509 | 17653 | 2.4950884086444 | 0.00705510538056193 | 0.223440563975545 | 2.15149649489975 | 0.0196463654223969 | SH3GL1//UNC13B//CPLX1//SYT2//SYT9//SYT14//CPLX3//SNPH//PACSIN1//LRRK2// |
| GO:0009649 | entrainment of circadian clock | Biological process | 4 | 27 | 509 | 17653 | 5.13803390817143 | 0.00708053961219068 | 0.223440563975545 | 2.14993364320479 | 0.00785854616895874 | CRTC1//FBXL3//PML//BHLHE40// |
| GO:0006810 | transport | Biological process | 171 | 5048 | 509 | 17653 | 1.17483669542529 | 0.00710331528238093 | 0.223440563975545 | 2.14853890863366 | 0.335952848722986 | SPRN//PML//NUP214//CUX1//SLC27A4//EDN1//NKAIN1//DLG4//LRRTM1//LGALS3BP//THBS1//TLN1//FERMT3//CD74//AQP1//SH3GL1//SPTBN2//SYT2//SYT9//SYT14//EXOC4//U2AF1//DDX39B//SRSF9//RBM8A//YWHAB//GOLGA7B//GOSR2//TIMM23//TOMM34//TIMM22//SLC12A7//SLC26A1//CHRNA3//CTNS//PLLP//SLC9A5//TOMM40L//P2RX2//CDKN1B//KCNIP2//KCNA6//KCNJ12//ABCC8//KCNQ4//SCN3B//SCN1A//SCN7A//SLC5A3//SLC10A7//SLC5A6//SLC23A2//ANO4//BEST1//NDFIP1//SLC39A2//SLC39A8//SLC25A5//SLC25A2//EBP//RALBP1//ESYT1//PCTP//ANO3//VLDLR//VPS45//AP1S1//ARFIP2//RABL3//KCNIP3//MLPH//VPS26A//CPLX1//LGI3//SNX19//DYNC1I1//KDELR3//KIF2A//SCAMP1//STEAP2//TGOLN2//SYS1//LRRK2//CD209//WAS//DBNL//MRC1//TMPRSS2//BIN2//VPS53//UNC13B//SNPH//CSN3//MED1//SLC6A3//DPP4//IL1B//PPP3CB//CACNA2D2//MGRN1//FAM160A2//SLC35A2//ATP1B4//PRSS8//RSC1A1//GRM2//CCK//EHD3//ASNA1//CA7//CPLX3//ACTR2//PEX5//PTAFR//PACSIN1//CCDC88C//CNTN2//CD81//SIRT3//SLC52A2//CYP4F2//NFKB1//REEP2//SGK2//ARL4C//ATP6V1G2//HTR3E//RAF1//CHRFAM7A//MYO19//KCNQ5//CACNG1//WDR19//VSNL1//ACTR3//BRK1//CHRM1//TGM2//KIT//HMOX1//IL4R//ALAD//ALDOC//RHOF//S100A12//TSPAN14//BCR//EMP2//WDR91//SFRP1//RBFOX1//FOXP3//MOGAT2//TNFSF4//SNRPB//GNAO1//PRKAA1//TIAM1//SLC30A6//GRIN2B//GRIPAP1//OXSR1//SLMAP//NUCB1//UBE2D3//SESTD1//COPS5//SHISA9//SHANK3//PAX8//GLRX// |
| GO:0044089 | positive regulation of cellular component biogenesis | Biological process | 25 | 511 | 509 | 17653 | 1.69675777300182 | 0.00734645067741155 | 0.229171151938288 | 2.13392243282311 | 0.0491159135559921 | BRK1//UNC13B//CDC42EP1//LMOD1//CDKN1B//MAPRE1//KIT//TRABD2B//CCK//ACTR3//ACTR2//ARFIP2//WAS//PALM//NF2//SFRP1//WNT11//LRRTM1//LRRN1//EDN1//BMP10//EPS8L2//SEPT7//FOSL1//PIP4K2C// |
| GO:0042472 | inner ear morphogenesis | Biological process | 8 | 98 | 509 | 17653 | 2.83116154123732 | 0.00737899173663403 | 0.229171151938288 | 2.13200297599957 | 0.0157170923379175 | FGFR2//FZD2//DLX5//INSIG2//BCR//PAX8//FZD3//KCNQ4// |
| GO:0044257 | cellular protein catabolic process | Biological process | 33 | 730 | 509 | 17653 | 1.56780418225368 | 0.00738715231739564 | 0.229171151938288 | 2.13152294606871 | 0.0648330058939096 | RNF123//UBE4B//RNF217//FBXO45//FBXO10//RLIM//UCHL5//RNF125//USP37//TP53INP2//USP46//UBE2D3//UBE2E1//USP45//FBXL3//SKP1//FBXL20//OGT//TRIB1//LRRK2//RNF114//VLDLR//DNAJB12//MAEA//UBXN2B//RNF38//UBXN2A//PML//ALAD//WDR91//CD81//EDEM3//TRIM67// |
| GO:0042127 | regulation of cell proliferation | Biological process | 65 | 1668 | 509 | 17653 | 1.35150622134905 | 0.0075100878299169 | 0.231921114126612 | 2.12435498392457 | 0.12770137524558 | ALDH1A2//VASH1//THBS1//OTP//FZD3//FGFR2//CDKN1B//CHRM1//DPP4//EDN1//EMP2//FGFR3//FSHB//SLC25A5//IL1B//KIT//PGGT1B//PRKAA1//BRK1//PURA//SFRP1//TIAM1//TTK//TNFSF4//FOSL1//PROK1//CCK//CD81//PTPRU//CDKN2A//GPNMB//DDAH1//LDOC1//SMAD1//NF2//DEC1//FOXP3//PML//RAF1//MED1//PAXBP1//TIRAP//CD74//RTN4//CD209//NDFIP1//AQP1//ZMIZ1//HMOX1//PTAFR//TGM2//TRIB1//DLX5//BMP10//PIM1//ABCC8//WNT11//LRRK2//DDX39B//LIMS2//ASCL1//SGK2//IL4R//BEX4//RELT// |
| GO:0009612 | response to mechanical stimulus | Biological process | 13 | 209 | 509 | 17653 | 2.15723672460308 | 0.00777580147672434 | 0.238793640959669 | 2.1092548358729 | 0.0255402750491159 | IL1B//AQP1//NFKB1//WNT11//TNFSF14//EDN1//RAF1//SHANK3//KIT//SCN1A//ANO3//THBS1//FOSL1// |
| GO:0017157 | regulation of exocytosis | Biological process | 12 | 186 | 509 | 17653 | 2.23753089549401 | 0.00783932397201977 | 0.238793640959669 | 2.10572138730028 | 0.0235756385068762 | UNC13B//SYT2//SYT9//SYT14//HMOX1//IL4R//BCR//PTAFR//VSNL1//LRRK2//CPLX1//LGI3// |
| GO:2000026 | regulation of multicellular organismal development | Biological process | 76 | 2008 | 509 | 17653 | 1.31265507713742 | 0.00786678456997259 | 0.238793640959669 | 2.10420274271929 | 0.149312377210216 | OTP//FZD3//FOXP3//NDFIP1//WNT11//TIAM1//IL1B//SFRP1//ZNF804A//TRIM67//LRRK2//NF2//SEPT7//EFNA3//VASH1//PML//ABCC8//THBS1//RFX4//TTL//RTN4//ASCL1//CNTN2//MED1//EDN1//TNFSF4//HMGB3//CD74//ZMIZ1//RAG1//IL4R//TRIB1//KMT2E//NREP//DIXDC1//HEYL//FSHB//GPNMB//EMP2//HMOX1//PROK1//DDAH1//AQP1//SEMA7A//KIT//SHANK3//PRKAA1//RGMA//CHRNA3//SEMA4G//SKIL//TLX2//NFATC4//CUX1//LRRTM1//LRRN1//FGFR2//BMP10//PIM1//FZD2//CDKN1B//ACTR2//SMAD1//DDX39B//PAX8//DLG4//CRTC1//PACSIN1//VLDLR//ARHGDIB//ZSWIM6//CELF4//ITGA5//SYT2//CTH//LIMS2// |
| GO:0071559 | response to transforming growth factor beta | Biological process | 14 | 233 | 509 | 17653 | 2.0838807052455 | 0.00787386987047259 | 0.238793640959669 | 2.10381176696549 | 0.0275049115913556 | TAB1//FERMT2//FSHB//SMAD1//PML//SKIL//NREP//THBS1//HTRA4//RASL11B//ONECUT2//EDN1//FGFR2//SFRP1// |
| GO:0007417 | central nervous system development | Biological process | 40 | 932 | 509 | 17653 | 1.4884862180325 | 0.00791786227058693 | 0.239055814892765 | 2.10139205697183 | 0.0785854616895874 | CTNS//FOXG1//GRIN2B//MED1//CA10//BCR//SNPH//ASCL1//RFX4//VLDLR//PHOX2A//NRGN//AQP1//PAX5//UCHL5//SPTBN2//LRRK2//SYPL2//MAPKAP1//NF2//FGFR2//SHANK3//FBXO45//DIXDC1//RTN4//CNTN2//RAX//OTP//DLX5//SATB2//ALDH1A2//SLC6A3//SEMA7A//GNAO1//SMAD1//FZD3//SFRP1//NPTX1//SH3GL1//PAX8// |
| GO:0046622 | positive regulation of organ growth | Biological process | 6 | 61 | 509 | 17653 | 3.41131759476956 | 0.00810933347637197 | 0.24090484185857 | 2.09101483992317 | 0.0117878192534381 | FGFR2//BMP10//PIM1//EDN1//DDX39B//CACNA2D2// |
| GO:0032535 | regulation of cellular component size | Biological process | 19 | 358 | 509 | 17653 | 1.84065041542734 | 0.00815105219988664 | 0.24090484185857 | 2.08878632560246 | 0.037328094302554 | SLC12A7//AQP1//BRK1//WAS//TTL//RTN4//ARHGAP40//EPS8L2//CDC42EP1//LMOD1//CNTN2//ACTR3//ACTR2//ARFIP2//SEMA7A//EDN1//SHANK3//SEMA4G//SPTBN2// |
| GO:0048585 | negative regulation of response to stimulus | Biological process | 63 | 1615 | 509 | 17653 | 1.352909547647 | 0.00827970819362565 | 0.24090484185857 | 2.08198496902808 | 0.1237721021611 | PPP3CB//FOXP3//CD96//NDFIP1//FGFR3//FGFR2//KIT//RGS9BP//RAF1//THBS1//PIK3IP1//MGRN1//YWHAB//RFX4//SMPD1//HIC1//TRABD2B//NFATC4//SFRP1//EDN1//HTRA4//SKIL//RASL11B//ONECUT2//TRIB1//PRKAA1//CCK//HMOX1//RNF125//SPRY3//NF2//BCR//IL1B//RNF149//CD74//IL4R//TNFSF4//LRRTM1//TRIM67//MAPKAP1//RGMA//SEMA4G//SEMA7A//NFKB1//TNFAIP8L2//CGNL1//SLC6A3//PALM//HEYL//ABCC8//SIRT3//RBMS3//WNT11//KREMEN1//LRRK2//CELF4//DDAH1//SLC25A5//PTPN18//RTN4//DDX39B//LYPD6//CTH// |
| GO:0007265 | Ras protein signal transduction | Biological process | 23 | 463 | 509 | 17653 | 1.72285046272919 | 0.00832743989428679 | 0.24090484185857 | 2.07948849327188 | 0.0451866404715128 | CDC42EP1//PAK2//RHOF//EPS8L2//DBNL//BRK1//TIAM1//IQSEC3//RABL3//RALGPS2//WAS//OGT//ARHGDIB//ARHGEF4//RAF1//BCR//ARHGEF6//SPRY3//TRIM67//MAPKAP1//CDKN2A//RASSF1//RALGDS// |
| GO:0002830 | positive regulation of type 2 immune response | Biological process | 3 | 15 | 509 | 17653 | 6.93634577603143 | 0.00837242512299505 | 0.24090484185857 | 2.07714872779114 | 0.00589390962671906 | IL4R//TNFSF4//CD74// |
| GO:0032305 | positive regulation of icosanoid secretion | Biological process | 3 | 15 | 509 | 17653 | 6.93634577603143 | 0.00837242512299505 | 0.24090484185857 | 2.07714872779114 | 0.00589390962671906 | EDN1//IL1B//CYP4F2// |
| GO:0043371 | negative regulation of CD4-positive, alpha-beta T cell differentiation | Biological process | 3 | 15 | 509 | 17653 | 6.93634577603143 | 0.00837242512299505 | 0.24090484185857 | 2.07714872779114 | 0.00589390962671906 | IL4R//TNFSF4//FOXP3// |
| GO:0045408 | regulation of interleukin-6 biosynthetic process | Biological process | 3 | 15 | 509 | 17653 | 6.93634577603143 | 0.00837242512299505 | 0.24090484185857 | 2.07714872779114 | 0.00589390962671906 | TIRAP//IL1B//PTAFR// |
| GO:1902187 | negative regulation of viral release from host cell | Biological process | 3 | 15 | 509 | 17653 | 6.93634577603143 | 0.00837242512299505 | 0.24090484185857 | 2.07714872779114 | 0.00589390962671906 | PML//TRIM21//TRIM5// |
| GO:0033002 | muscle cell proliferation | Biological process | 13 | 211 | 509 | 17653 | 2.13678898313765 | 0.0083906989405468 | 0.24090484185857 | 2.0762018611761 | 0.0255402750491159 | PAXBP1//EDN1//FGFR2//HMOX1//PTAFR//TGM2//THBS1//TRIB1//BMP10//SMAD1//PIM1//CDKN1B//DDX39B// |
| GO:0051701 | interaction with host | Biological process | 13 | 211 | 509 | 17653 | 2.13678898313765 | 0.0083906989405468 | 0.24090484185857 | 2.0762018611761 | 0.0255402750491159 | CD209//DPP4//ITGA5//MRC1//NCAM1//SLC52A2//CD81//TRIM5//TRIM21//TMPRSS2//CD74//DDX39B//PML// |
| GO:0032501 | multicellular organismal process | Biological process | 243 | 7496 | 509 | 17653 | 1.12428763578951 | 0.00841233684908317 | 0.24090484185857 | 2.07508334549961 | 0.477406679764244 | EBP//DLX5//EVC//FGFR3//FGFR2//VASH1//HMOX1//ITGA5//MED1//RTN4//JAM3//PROK1//HS6ST1//KIT//NRIP1//DAZL//FOXS1//ALDH1A2//EDN1//NFATC4//ZMIZ1//GPNMB//TP53INP2//SFRP1//WNT11//SEMA7A//PAX8//SMAD1//IL1B//DPP4//USP46//FBXL20//CCK//TAB1//BRK1//WDR19//FOSL1//TLX2//NF2//SEMA4G//FBXO45//SATB2//MARK1//ASCL1//FZD3//FOXP3//PTAFR//AGPAT1//TNFSF4//SKIL//TAF8//DDX39B//ONECUT2//CDKN1B//NDP//LDOC1//PPP3CB//CCDC103//MICAL2//TGM2//PARVA//THBS1//OTP//SNX19//CTNS//RAG1//IL4R//CD96//NDFIP1//BCR//P2RX2//CHRM1//DDAH1//AQP1//CYP4F2//EMP2//FZD2//HEYL//UBE4B//TIAM1//KRT2//PHOX2A//LMOD1//GNAO1//KCNIP2//KCNJ12//SCN7A//TLN1//CACNG1//SLMAP//SSPN//AKAP10//VPS45//EHD3//PAFAH2//WAS//CHRNA3//DAPK3//MYOM3//SPRY3//CUX1//LCLAT1//GRIN2B//HIC1//HMGB3//HOXC6//ARHGDIB//PAX5//PIM1//ASF1B//MBNL3//RNF114//RAF1//HIVEP3//RELT//TMEFF1//SPIN3//CELF4//LRRK2//MYCBP//MEA1//CABS1//GAL3ST1//SEPT7//FSHB//DLG4//RAX//FGF11//KIF2A//NRGN//NRN1//RBFOX1//SCN3B//PURA//SMPD1//VLDLR//HTR3E//CHRFAM7A//LRRN1//PARD6B//EFNA3//NCAM1//SPTBN2//CNTN2//SHANK3//UNC5A//DBNL//BSN//NPTX1//SH3GL1//FOXG1//CA10//SNPH//SLC5A3//BMP10//PAXBP1//ABCC8//PLA2G4C//NPHS2//CSN3//SLC6A3//BEST1//EPS8L2//KCNQ4//CRTC1//SCN1A//TAC4//ACTR2//NPTX2//RNF38//HOXC9//MMP24//ZNF281//ZNF804A//TRIM67//TTK//PML//PRDM12//RFX4//UCHL5//SYPL2//MAPKAP1//DIXDC1//TIRAP//DGKG//CD79A//KMT2E//KREMEN1//DLAT//TTL//PTPRU//NREP//NPTXR//TBC1D24//KRT9//KRTAP1-1//KRT38//TRIM21//RNF125//RNF135//NFKB1//POLR2E//REL//OGT//BHLHE40//GPD2//TNKS2//PRKAB1//CACNA2D2//TPRA1//INSIG2//PLLP//MAEA//SLC27A4//TET3//CD74//TRIB1//EXOC4//TEAD1//C2ORF49//PRKAA1//SEMA5B//RGMA//C11ORF88//PACSIN1//CXCL14//S100A12//MOGAT2//RGS9BP//REEP2//LRRTM1//PALM//UNC13B//PAK2//CTNND2//KCNIP3//PRSS8//FERMT3//ZNF322//ZSWIM6//SYT2//CTH//LIMS2// |
| GO:0033036 | macromolecule localization | Biological process | 106 | 2955 | 509 | 17653 | 1.2440823219278 | 0.00844217766087255 | 0.24090484185857 | 2.07354551260275 | 0.208251473477407 | SPRN//PML//NUP214//SLC27A4//AKAP10//MAPRE1//PLLP//CD81//U2AF1//DDX39B//SRSF9//RBM8A//YWHAB//GOLGA7B//EXOC4//KDELR3//GOSR2//TIMM23//TOMM34//TIMM22//ANO4//ESYT1//PCTP//ANO3//VLDLR//VPS45//AP1S1//ARFIP2//RABL3//KCNIP3//MLPH//VPS26A//CD74//TLN1//SYT9//DPP4//SLC25A5//IL1B//PPP3CB//ABCC8//CACNA2D2//THBS1//MEST//NFKB1//ABHD5//EHD3//VPS53//FAM160A2//SCAMP1//PEX5//NRIP1//CPLX1//SNX19//CPLX3//TOMM40L//SIRT3//CYP4F2//EDN1//REEP2//TRIM5//LRRK2//MED1//ATP6V1G2//PTPRU//EMP2//WNT11//TTK//MIS12//SEPT7//DLG4//LRRTM1//BSN//RAF1//VSNL1//SYS1//SFRP1//RBFOX1//S100A12//FOXP3//UNC13B//TNFSF4//NDFIP1//TAF8//SKP1//WDR19//TIAM1//TULP4//BCR//TNKS2//CNTN2//FERMT2//PACSIN1//PALM//SCN3B//SLMAP//TSPAN14//IL4R//CHRM1//SHANK3//GRIPAP1//NF2//NUCB1//TP53INP2//PRKAA1//UBE2D3//RTN4// |
| GO:1903305 | regulation of regulated secretory pathway | Biological process | 9 | 122 | 509 | 17653 | 2.55848819607717 | 0.00883257037721951 | 0.250925706845194 | 2.05391289345538 | 0.0176817288801572 | UNC13B//SYT2//SYT9//SYT14//HMOX1//IL4R//BCR//PTAFR//LRRK2// |
| GO:0044093 | positive regulation of molecular function | Biological process | 67 | 1742 | 509 | 17653 | 1.33391264923681 | 0.00886755048587926 | 0.250925706845194 | 2.05219633026776 | 0.131630648330059 | TAB1//LRRK2//RAF1//IL1B//KIT//PRKAA1//THBS1//PROK1//CD74//CD81//PML//CCK//PARP16//CHRNA3//TIRAP//MAP3K9//DBNL//CASP7//FGFR3//FGFR2//PTAFR//TIAM1//EMP2//PAK2//WNT11//OXSR1//MAPKAP1//SGK2//CRTC1//FAM13A//RALBP1//CDC42EP1//GNAO1//ARHGAP40//ARHGDIB//TBC1D24//BCR//SFRP1//ARHGEF6//MMP24//YWHAB//EDN1//S100A12//CDKN1B//CAMKK2//VLDLR//ABHD5//COPS5//FZD2//NDP//TRIM21//FOSL1//TRIM5//CTH//NFKB1//NEK7//DLG4//RALGAPB//EHD3//EFNA3//MAPRE1//ABCC8//MED1//DYNC1I1//GLRX//SHANK3//GRIN2B// |
| GO:0030334 | regulation of cell migration | Biological process | 37 | 853 | 509 | 17653 | 1.50436573102675 | 0.00891281066175036 | 0.251155577105907 | 2.04998531926265 | 0.0726915520628684 | HMOX1//BCR//EMP2//EDN1//THBS1//VASH1//BMP10//RTN4//TNFSF14//TRIB1//GPNMB//DAPK3//MAPRE1//FSHB//IL1B//ITGA5//KIT//SEMA4G//TIAM1//WNT11//FERMT3//SEMA7A//ONECUT2//PTPRU//NF2//SFRP1//ABCC8//JAM3//TIRAP//CD74//ARHGDIB//PTAFR//CXCL14//KIF2A//RHOF//BEX4//PARD6B// |
| GO:1902903 | regulation of supramolecular fiber organization | Biological process | 18 | 336 | 509 | 17653 | 1.85794976143699 | 0.0090058006776592 | 0.251971611254847 | 2.04547766935961 | 0.0353634577603143 | BRK1//WAS//ARHGAP40//EPS8L2//CDC42EP1//LMOD1//MAPRE1//CDKN1B//SHANK3//ACTR3//ACTR2//ARFIP2//NF2//SFRP1//WNT11//SPTBN2//EDN1//BMP10// |
| GO:0007015 | actin filament organization | Biological process | 20 | 388 | 509 | 17653 | 1.78771798351326 | 0.00915450475731982 | 0.251971611254847 | 2.03836514529793 | 0.0392927308447937 | RHOF//MICAL2//BRK1//WAS//MYOM3//ARHGAP40//EPS8L2//CDC42EP1//LMOD1//SHANK3//ACTR3//ACTR2//ARFIP2//NF2//SFRP1//WNT11//SPTBN2//EMP2//PACSIN1//CGNL1// |
| GO:0007205 | protein kinase C-activating G-protein coupled receptor signaling pathway | Biological process | 4 | 29 | 509 | 17653 | 4.78368674209064 | 0.00915860912090517 | 0.251971611254847 | 2.03817047578421 | 0.00785854616895874 | CHRM1//DGKG//EDN1//CCK// |
| GO:0032892 | positive regulation of organic acid transport | Biological process | 4 | 29 | 509 | 17653 | 4.78368674209064 | 0.00915860912090517 | 0.251971611254847 | 2.03817047578421 | 0.00785854616895874 | CCK//CYP4F2//EDN1//IL1B// |
| GO:0007167 | enzyme linked receptor protein signaling pathway | Biological process | 43 | 1028 | 509 | 17653 | 1.45069488506494 | 0.00922909004150708 | 0.251971611254847 | 2.03484111689758 | 0.0844793713163065 | PTPRT//CHRNA3//TAB1//FERMT2//FSHB//SMAD1//PML//SKIL//PTPRU//ATP6V1G2//FGFR3//FGFR2//GALNT3//POLR2E//PTBP1//BMP10//TTK//NREP//DLX5//RGMA//UBE2D3//THBS1//HTRA4//RASL11B//ONECUT2//SFRP1//IL1B//ITGA5//KIT//PTPN18//SPRY3//SHKBP1//OGT//BCR//PAK2//BRK1//RAF1//ACTR3//ACTR2//EFNA3//GRIN2B//TIAM1//RTN4// |
| GO:0000381 | regulation of alternative mRNA splicing, via spliceosome | Biological process | 5 | 45 | 509 | 17653 | 3.85352543112857 | 0.00923596772984828 | 0.251971611254847 | 2.03451759316211 | 0.00982318271119843 | RBFOX1//MBNL3//CELF4//PTBP1//RBM8A// |
| GO:0055023 | positive regulation of cardiac muscle tissue growth | Biological process | 5 | 45 | 509 | 17653 | 3.85352543112857 | 0.00923596772984828 | 0.251971611254847 | 2.03451759316211 | 0.00982318271119843 | FGFR2//BMP10//PIM1//EDN1//DDX39B// |
| GO:0060359 | response to ammonium ion | Biological process | 9 | 123 | 509 | 17653 | 2.53768747903589 | 0.00929468043179586 | 0.251971611254847 | 2.03176553747826 | 0.0176817288801572 | CHRM1//SLC6A3//SMPD1//TIAM1//GNAO1//CASP7//CHRNA3//LRRK2//PTAFR// |
| GO:0099173 | postsynapse organization | Biological process | 8 | 102 | 509 | 17653 | 2.7201355984437 | 0.00931394031360225 | 0.251971611254847 | 2.03086654947783 | 0.0157170923379175 | CTNND2//DLG4//SHANK3//LRRK2//ACTR2//TIAM1//GRIN2B//ZNF804A// |
| GO:0016567 | protein ubiquitination | Biological process | 36 | 827 | 509 | 17653 | 1.5097245945413 | 0.00931434316334641 | 0.251971611254847 | 2.03084776563001 | 0.0707269155206287 | UBE4B//RNF217//MGRN1//FBXL3//FBXO10//RLIM//RNF125//RNF114//SKP1//TRIM21//UBE2D3//UBE2E1//TNKS2//FBXL20//KLHL22//RAG1//UBXN2A//OGT//LRRK2//NDFIP1//TRIM68//TRIM5//MAEA//RNF38//FBXO45//RNF149//MED1//DCAF16//TULP4//KLHL14//PEX5//RNF123//UBE2Z//HLTF//TRIM7//RNF135// |
| GO:0002260 | lymphocyte homeostasis | Biological process | 6 | 63 | 509 | 17653 | 3.30302179811021 | 0.00945868416359819 | 0.252656783878636 | 2.02416927588527 | 0.0117878192534381 | FOXP3//CD74//PPP3CB//RAG1//TNFSF14//SKIL// |
| GO:0030032 | lamellipodium assembly | Biological process | 6 | 63 | 509 | 17653 | 3.30302179811021 | 0.00945868416359819 | 0.252656783878636 | 2.02416927588527 | 0.0117878192534381 | WAS//BRK1//ARFIP2//KIT//ARHGEF4//ARHGEF6// |
| GO:0007626 | locomotory behavior | Biological process | 12 | 191 | 509 | 17653 | 2.17895678828213 | 0.00957740480011005 | 0.252656783878636 | 2.01875215623263 | 0.0235756385068762 | CTNS//SCN1A//CNTN2//PPP3CB//LRRK2//DLG4//DPP4//CHRNA3//GNAO1//LRRTM1//NOVA1//SLC6A3// |
| GO:0002703 | regulation of leukocyte mediated immunity | Biological process | 11 | 168 | 509 | 17653 | 2.27082748620077 | 0.00960125869489935 | 0.252656783878636 | 2.01767182858377 | 0.0216110019646365 | PPP3CB//WAS//IL1B//FOXP3//TNFSF4//CD96//HMOX1//IL4R//BCR//PTAFR//NDFIP1// |
| GO:0048562 | embryonic organ morphogenesis | Biological process | 16 | 288 | 509 | 17653 | 1.92676271556429 | 0.00966935869292725 | 0.252656783878636 | 2.01460232895307 | 0.031434184675835 | CCDC103//MICAL2//FGFR2//WDR19//DLX5//INSIG2//BCR//PAX8//FZD3//KCNQ4//EDN1//WNT11//PAX5//SATB2//HOXC9//FZD2// |
| GO:0034220 | ion transmembrane transport | Biological process | 46 | 1119 | 509 | 17653 | 1.42570109784382 | 0.00971258984693215 | 0.252656783878636 | 2.01266495070363 | 0.0903732809430255 | SLC12A7//ATP1B4//KCNJ12//THBS1//SLC23A2//SLC5A6//KCNA6//KCNQ5//SCN1A//SCN7A//CACNG1//KCNQ4//CACNA2D2//SCN3B//SLC39A8//KCNIP2//SLC39A2//SLC30A6//KCNIP3//AQP1//SLC9A5//ABCC8//SLC35A2//GRIN2B//CHRNA3//P2RX2//HTR3E//STEAP2//OXSR1//EHD3//SLMAP//SLC26A1//BEST1//ATP6V1G2//PTAFR//SLC25A2//SESTD1//DLG4//SHISA9//SHANK3//GLRX//SGK2//ANO4//RAF1//ANO3//CHRFAM7A// |
| GO:0099565 | chemical synaptic transmission, postsynaptic | Biological process | 9 | 124 | 509 | 17653 | 2.51722225743076 | 0.00977483981581855 | 0.252656783878636 | 2.00989035082991 | 0.0176817288801572 | CHRNA3//LRRK2//P2RX2//GRIN2B//CELF4//UNC13B//SH3GL1//DLG4//SHANK3// |
| GO:0051234 | establishment of localization | Biological process | 173 | 5155 | 509 | 17653 | 1.16390671120605 | 0.00980620721634198 | 0.252656783878636 | 2.00849893385518 | 0.339882121807466 | SPRN//PML//NUP214//CUX1//SLC27A4//EDN1//NKAIN1//DLG4//LRRTM1//LGALS3BP//THBS1//TLN1//FERMT3//CD74//AQP1//SH3GL1//SPTBN2//SYT2//SYT9//SYT14//EXOC4//U2AF1//DDX39B//SRSF9//RBM8A//YWHAB//GOLGA7B//GOSR2//TIMM23//TOMM34//TIMM22//SLC6A3//SLC12A7//SLC26A1//CHRNA3//CTNS//PLLP//SLC9A5//TOMM40L//P2RX2//CDKN1B//KCNIP2//KCNA6//KCNJ12//ABCC8//KCNQ4//SCN3B//SCN1A//SCN7A//SLC5A3//SLC10A7//SLC5A6//SLC23A2//ANO4//BEST1//NDFIP1//SLC39A2//SLC39A8//SLC25A5//SLC25A2//EBP//RALBP1//ESYT1//PCTP//ANO3//VLDLR//VPS45//AP1S1//ARFIP2//RABL3//KCNIP3//MLPH//VPS26A//CPLX1//LGI3//SNX19//DYNC1I1//KDELR3//KIF2A//SCAMP1//STEAP2//TGOLN2//SYS1//LRRK2//CD209//WAS//DBNL//MRC1//TMPRSS2//BIN2//VPS53//UNC13B//SNPH//CSN3//MED1//DPP4//IL1B//PPP3CB//CACNA2D2//MGRN1//FAM160A2//SLC35A2//ATP1B4//PRSS8//RSC1A1//GRM2//CCK//EHD3//ASNA1//CA7//CPLX3//ACTR3//ACTR2//PEX5//PTAFR//PACSIN1//CCDC88C//CNTN2//CD81//SIRT3//SLC52A2//CYP4F2//NFKB1//REEP2//SGK2//ARL4C//ATP6V1G2//HTR3E//RAF1//CHRFAM7A//MYO19//KCNQ5//CACNG1//WDR19//VSNL1//BRK1//CHRM1//TGM2//KIT//HMOX1//IL4R//ALAD//ALDOC//RHOF//S100A12//TSPAN14//BCR//EMP2//WDR91//SFRP1//RBFOX1//FOXP3//MOGAT2//TNFSF4//SNRPB//MIS12//GNAO1//PRKAA1//TIAM1//SLC30A6//TTL//GRIN2B//GRIPAP1//OXSR1//SLMAP//NUCB1//UBE2D3//SESTD1//COPS5//SHISA9//SHANK3//PAX8//GLRX// |
| GO:0006928 | movement of cell or subcellular component | Biological process | 78 | 2090 | 509 | 17653 | 1.29434203476185 | 0.00990565705896513 | 0.252656783878636 | 2.00411671168338 | 0.153241650294695 | SEMA4G//SEMA7A//FBXO45//SATB2//MARK1//ASCL1//FZD3//CCK//JAM3//S100A12//KIT//HMOX1//BCR//CCDC103//DYNC1I1//KIF2A//MYO19//EFNA3//NCAM1//SPTBN2//CNTN2//VLDLR//SHANK3//UNC5A//WAS//EMP2//EDN1//THBS1//VASH1//BMP10//RTN4//PML//TNFSF14//TRIB1//ACTR3//ACTR2//AVL9//PAK2//RHOF//TIAM1//DIXDC1//LRRK2//MLPH//BEX4//PARD6B//GPNMB//DAPK3//MAPRE1//FSHB//IL1B//ITGA5//WNT11//FERMT3//ONECUT2//PTPRU//NF2//SFRP1//WDR19//DPP4//BRK1//CD74//CDKN1B//KRT2//BIN2//CXCL14//ABCC8//PARVA//SCN3B//SCN1A//TIRAP//DLX5//SEMA5B//ARHGDIB//TAC4//ZSWIM6//PTAFR//RAF1//CD81// |
| GO:0015672 | monovalent inorganic cation transport | Biological process | 25 | 524 | 509 | 17653 | 1.65466263741208 | 0.00990654826942608 | 0.252656783878636 | 2.00407764003242 | 0.0491159135559921 | NKAIN1//CDKN1B//KCNIP2//AQP1//KCNA6//KCNJ12//ABCC8//KCNQ4//SCN3B//SCN1A//SCN7A//SLC5A3//SLC10A7//SLC5A6//SLC23A2//SLC12A7//ATP1B4//PRSS8//KCNIP3//KCNQ5//SLC9A5//OXSR1//SLMAP//ATP6V1G2//GLRX// |
| GO:0043433 | negative regulation of DNA binding transcription factor activity | Biological process | 11 | 169 | 509 | 17653 | 2.25739063716999 | 0.0100123879914125 | 0.252656783878636 | 1.99946232933303 | 0.0216110019646365 | CDKN2A//FOXP3//TRIM21//HEYL//TRIB1//FOXS1//HMOX1//RLIM//PIM1//TNFSF4//BHLHE40// |
| GO:0031058 | positive regulation of histone modification | Biological process | 7 | 83 | 509 | 17653 | 2.92496508627832 | 0.010019983853509 | 0.252656783878636 | 1.99913297830286 | 0.0137524557956778 | PAXBP1//IL1B//FOXP3//PRDM12//OGT//LRRK2//KMT2E// |
| GO:0044070 | regulation of anion transport | Biological process | 7 | 83 | 509 | 17653 | 2.92496508627832 | 0.010019983853509 | 0.252656783878636 | 1.99913297830286 | 0.0137524557956778 | THBS1//CCK//CYP4F2//EDN1//IL1B//PTAFR//CA7// |
| GO:0051094 | positive regulation of developmental process | Biological process | 53 | 1329 | 509 | 17653 | 1.38309377776398 | 0.0100833206014394 | 0.252656783878636 | 1.9963964241009 | 0.104125736738703 | OTP//FZD3//FOXP3//IL1B//TIAM1//NFKB1//TRIM67//ZNF804A//MED1//RTN4//FOXS1//SLC6A3//EDN1//TNFSF4//ZMIZ1//RAG1//CD74//NF2//SFRP1//IL4R//TRIB1//TNFSF14//HEYL//ASCL1//SMAD1//DDAH1//HMOX1//AQP1//THBS1//SEMA7A//CACNA2D2//KIT//SHANK3//PRKAA1//SKIL//CUX1//LRRTM1//LRRN1//FGFR2//BMP10//PIM1//ACTR2//DDX39B//PAX8//DLG4//CRTC1//PACSIN1//VLDLR//ZNF322//ITGA5//SYT2//CTH//CDKN2A// |
| GO:0034315 | regulation of Arp2/3 complex-mediated actin nucleation | Biological process | 3 | 16 | 509 | 17653 | 6.50282416502947 | 0.0100868448391589 | 0.252656783878636 | 1.9962446596531 | 0.00589390962671906 | BRK1//WAS//ARFIP2// |
| GO:0043173 | nucleotide salvage | Biological process | 3 | 16 | 509 | 17653 | 6.50282416502947 | 0.0100868448391589 | 0.252656783878636 | 1.9962446596531 | 0.00589390962671906 | UCK2//PRTFDC1//UCK1// |
| GO:0050908 | detection of light stimulus involved in visual perception | Biological process | 3 | 16 | 509 | 17653 | 6.50282416502947 | 0.0100868448391589 | 0.252656783878636 | 1.9962446596531 | 0.00589390962671906 | RGS9BP//SEMA5B//BEST1// |
| GO:0050962 | detection of light stimulus involved in sensory perception | Biological process | 3 | 16 | 509 | 17653 | 6.50282416502947 | 0.0100868448391589 | 0.252656783878636 | 1.9962446596531 | 0.00589390962671906 | RGS9BP//SEMA5B//BEST1// |
| GO:0080182 | histone H3-K4 trimethylation | Biological process | 3 | 16 | 509 | 17653 | 6.50282416502947 | 0.0100868448391589 | 0.252656783878636 | 1.9962446596531 | 0.00589390962671906 | KMT2E//TET3//OGT// |
| GO:2000193 | positive regulation of fatty acid transport | Biological process | 3 | 16 | 509 | 17653 | 6.50282416502947 | 0.0100868448391589 | 0.252656783878636 | 1.9962446596531 | 0.00589390962671906 | CYP4F2//EDN1//IL1B// |
| GO:1902930 | regulation of alcohol biosynthetic process | Biological process | 6 | 64 | 509 | 17653 | 3.25141208251473 | 0.0101901625816746 | 0.254302839630499 | 1.99181888687082 | 0.0117878192534381 | NFKB1//IDI1//NFYA//PRKAA1//IL1B//PTAFR// |
| GO:0002828 | regulation of type 2 immune response | Biological process | 4 | 30 | 509 | 17653 | 4.62423051735429 | 0.0103317216496644 | 0.255946642918243 | 1.98582730281262 | 0.00785854616895874 | NDFIP1//TNFSF4//CD74//IL4R// |
| GO:0046596 | regulation of viral entry into host cell | Biological process | 4 | 30 | 509 | 17653 | 4.62423051735429 | 0.0103317216496644 | 0.255946642918243 | 1.98582730281262 | 0.00785854616895874 | TRIM5//TRIM21//TMPRSS2//CD74// |
| GO:0051347 | positive regulation of transferase activity | Biological process | 30 | 664 | 509 | 17653 | 1.56694558193481 | 0.0104393918841253 | 0.257670099680071 | 1.98132479913652 | 0.0589390962671906 | TAB1//LRRK2//RAF1//IL1B//KIT//PRKAA1//THBS1//PROK1//CD74//CD81//PARP16//CHRNA3//TIRAP//MAP3K9//DBNL//EMP2//PAK2//WNT11//OXSR1//MAPKAP1//FGFR3//FGFR2//EDN1//S100A12//TIAM1//CDKN1B//CAMKK2//VLDLR//NEK7//DLG4// |
| GO:0034765 | regulation of ion transmembrane transport | Biological process | 22 | 446 | 509 | 17653 | 1.7107579268239 | 0.0105051728223242 | 0.258350850172286 | 1.97859679854938 | 0.0432220039292731 | THBS1//OXSR1//KCNIP3//KCNIP2//EHD3//SLMAP//PTAFR//ABCC8//SESTD1//DLG4//SHISA9//SHANK3//SCN3B//GLRX//KCNA6//KCNJ12//KCNQ5//SCN1A//SCN7A//CACNG1//KCNQ4//CACNA2D2// |
| GO:0048589 | developmental growth | Biological process | 28 | 609 | 509 | 17653 | 1.59456224736355 | 0.0105472837841442 | 0.258446667507852 | 1.97685936874917 | 0.0550098231827112 | TAF8//WNT11//EVC//FGFR3//FGFR2//TTL//RTN4//GPD2//SPTBN2//TNKS2//CACNA2D2//FOXS1//SLC6A3//SEMA7A//SEMA5B//PPP3CB//SEMA4G//SHANK3//BMP10//SMAD1//PIM1//MED1//EDN1//DDX39B//SFRP1//SYT2//TIAM1//ZMIZ1// |
| GO:0001776 | leukocyte homeostasis | Biological process | 7 | 84 | 509 | 17653 | 2.89014407334643 | 0.0106677693509442 | 0.260455321734425 | 1.9719263828111 | 0.0137524557956778 | JAM3//FOXP3//SKIL//CD74//PPP3CB//RAG1//TNFSF14// |
| GO:0006664 | glycolipid metabolic process | Biological process | 9 | 126 | 509 | 17653 | 2.47726634858265 | 0.0107910041505278 | 0.261494954276568 | 1.96693814041976 | 0.0176817288801572 | ST6GALNAC6//PGAP3//PYURF//GAL3ST1//ESYT1//KIT//SMPD1//ST8SIA5//PRKAA1// |
| GO:1903509 | liposaccharide metabolic process | Biological process | 9 | 126 | 509 | 17653 | 2.47726634858265 | 0.0107910041505278 | 0.261494954276568 | 1.96693814041976 | 0.0176817288801572 | ST6GALNAC6//PGAP3//PYURF//GAL3ST1//ESYT1//KIT//SMPD1//ST8SIA5//PRKAA1// |
| GO:0051259 | protein complex oligomerization | Biological process | 25 | 528 | 509 | 17653 | 1.64212731440138 | 0.0108263473602601 | 0.261494954276568 | 1.96551804280712 | 0.0491159135559921 | TRABD2B//CCK//KCTD18//ALAD//P2RX2//EHD3//HMOX1//KCNA6//CCDC88C//TGM2//SHKBP1//CTH//DPYS//CRTC1//KCNJ12//CD79A//GRIN2B//CD74//PRKAA1//PRKAB1//YWHAB//SEPT7//TRIM21//BRK1//PEX5// |
| GO:0032270 | positive regulation of cellular protein metabolic process | Biological process | 60 | 1546 | 509 | 17653 | 1.34599206520662 | 0.0109574910500274 | 0.261694940325345 | 1.96028887540139 | 0.117878192534381 | TAB1//LRRK2//RAF1//IL1B//KIT//PRKAA1//THBS1//PROK1//CD74//CD81//GPNMB//CAMKK2//PML//CCK//PARP16//CHRNA3//TIRAP//MAP3K9//DBNL//CASP7//BMP10//TTK//CNTN2//LARP1//RBMS3//PTAFR//RBM3//DDX39B//PAXBP1//NDFIP1//EMP2//PAK2//WNT11//OXSR1//MAPKAP1//TRIB1//RNF217//RNF125//RNF114//FOXP3//SMPD1//FGFR3//EDN1//S100A12//FGFR2//TRIM5//TIAM1//CDKN1B//VLDLR//OGT//DAZL//ITGA5//PRDM12//DLG4//SEMA7A//EFNA3//TRABD2B//KMT2E//TRIM67//GRIN2B// |
| GO:0006940 | regulation of smooth muscle contraction | Biological process | 6 | 65 | 509 | 17653 | 3.20139035816835 | 0.0109609395561899 | 0.261694940325345 | 1.96015221714912 | 0.0117878192534381 | CHRM1//EDN1//KIT//PTAFR//CHRNA3//DAPK3// |
| GO:0010508 | positive regulation of autophagy | Biological process | 8 | 105 | 509 | 17653 | 2.64241743848817 | 0.0109962071955837 | 0.261694940325345 | 1.95875708557549 | 0.0157170923379175 | LARP1//HMOX1//CAMKK2//PIP4K2C//LRRK2//PRKAA1//TP53INP2//TRIM21// |
| GO:0035094 | response to nicotine | Biological process | 5 | 47 | 509 | 17653 | 3.68954562554863 | 0.0110663061955762 | 0.261694940325345 | 1.95599731738507 | 0.00982318271119843 | CHRNA3//NFKB1//EDN1//HMOX1//SLC6A3// |
| GO:0071622 | regulation of granulocyte chemotaxis | Biological process | 5 | 47 | 509 | 17653 | 3.68954562554863 | 0.0110663061955762 | 0.261694940325345 | 1.95599731738507 | 0.00982318271119843 | THBS1//JAM3//TIRAP//EDN1//CD74// |
| GO:0034762 | regulation of transmembrane transport | Biological process | 25 | 529 | 509 | 17653 | 1.63902310397718 | 0.0110667977130044 | 0.261694940325345 | 1.9559780283321 | 0.0491159135559921 | THBS1//IL1B//RSC1A1//KCNA6//KCNJ12//KCNQ5//SCN1A//SCN7A//CACNG1//KCNQ4//CACNA2D2//OXSR1//KCNIP3//KCNIP2//EHD3//SLMAP//PTAFR//ABCC8//SESTD1//DLG4//SHISA9//SHANK3//SCN3B//GLRX//PRKAA1// |
| GO:0006468 | protein phosphorylation | Biological process | 73 | 1946 | 509 | 17653 | 1.30101038450744 | 0.0111940603404543 | 0.263781986350148 | 1.95101235640234 | 0.143418467583497 | CDKN1B//CAMKK2//LRRK2//DLG4//FGFR3//FGFR2//GRIN2B//IL1B//KIT//SMAD1//NCAM1//MAPK4//RAF1//SPTBN2//YWHAB//SHANK3//TAB1//PRKAA1//THBS1//PROK1//CD74//CD81//CCDC88C//PML//GPNMB//TRIB1//LRRTM1//NF2//PAK2//CEP85//PARP16//CHRNA3//TIRAP//ARHGEF6//MAP3K9//DBNL//BMP10//TTK//SGK2//DAPK3//OXSR1//CUX1//BCR//CDKN2A//EMP2//WNT11//MAPKAP1//EDN1//S100A12//SPRY3//SMPD1//RNF149//TRIM5//TIAM1//SFRP1//VLDLR//COPS5//PIM1//MEX3B//ITGA5//CCK//NFKB1//SKP1//SH3BP5//MED1//SIRT3//SEMA7A//GLYCTK//NEK7//MARK1//PPP3CB//PRKAB1//AATK// |
| GO:0043406 | positive regulation of MAP kinase activity | Biological process | 15 | 268 | 509 | 17653 | 1.94114154179984 | 0.0112703103697824 | 0.26458375357106 | 1.94806412388044 | 0.0294695481335953 | TAB1//LRRK2//RAF1//IL1B//KIT//PRKAA1//THBS1//PROK1//CD74//CD81//MAP3K9//DBNL//EDN1//TIAM1//S100A12// |
| GO:0050796 | regulation of insulin secretion | Biological process | 11 | 172 | 509 | 17653 | 2.21801754466121 | 0.0113273372167022 | 0.26458375357106 | 1.94587217027841 | 0.0216110019646365 | SIRT3//PPP3CB//VSNL1//SFRP1//ABCC8//TIAM1//SYT9//DPP4//SLC25A5//IL1B//CACNA2D2// |
| GO:0002367 | cytokine production involved in immune response | Biological process | 7 | 85 | 509 | 17653 | 2.85614237836588 | 0.0113454515060783 | 0.26458375357106 | 1.94517821615661 | 0.0137524557956778 | KIT//FOXP3//TNFSF4//CD96//HMOX1//SEMA7A//CD74// |
| GO:0003401 | axis elongation | Biological process | 4 | 31 | 509 | 17653 | 4.47506179098802 | 0.0115978284587774 | 0.264987730252458 | 1.93562331910542 | 0.00785854616895874 | WNT11//FGFR2//MED1//SFRP1// |
| GO:0034694 | response to prostaglandin | Biological process | 4 | 31 | 509 | 17653 | 4.47506179098802 | 0.0115978284587774 | 0.264987730252458 | 1.93562331910542 | 0.00785854616895874 | EDN1//PRKAA1//SFRP1//TNFSF4// |
| GO:0050869 | negative regulation of B cell activation | Biological process | 4 | 31 | 509 | 17653 | 4.47506179098802 | 0.0115978284587774 | 0.264987730252458 | 1.93562331910542 | 0.00785854616895874 | HMGB3//SFRP1//FOXP3//NDFIP1// |
| GO:0071353 | cellular response to interleukin-4 | Biological process | 4 | 31 | 509 | 17653 | 4.47506179098802 | 0.0115978284587774 | 0.264987730252458 | 1.93562331910542 | 0.00785854616895874 | IL4R//ALAD//MRC1//PML// |
| GO:0002718 | regulation of cytokine production involved in immune response | Biological process | 6 | 66 | 509 | 17653 | 3.15288444365065 | 0.0117720675029184 | 0.264987730252458 | 1.92914725625125 | 0.0117878192534381 | FOXP3//TNFSF4//CD96//HMOX1//SEMA7A//CD74// |
| GO:0050866 | negative regulation of cell activation | Biological process | 11 | 173 | 509 | 17653 | 2.20519663399843 | 0.0117937576074805 | 0.264987730252458 | 1.9283478023523 | 0.0216110019646365 | GPNMB//FOXP3//NDFIP1//HMOX1//BCR//HMGB3//SFRP1//CD74//TNFSF4//IL4R//TNFAIP8L2// |
| GO:0006511 | ubiquitin-dependent protein catabolic process | Biological process | 27 | 587 | 509 | 17653 | 1.59524136246038 | 0.0118356467374874 | 0.264987730252458 | 1.92680800585083 | 0.0530451866404715 | UBE4B//UBE2E1//FBXL3//SKP1//FBXL20//OGT//TRIB1//LRRK2//RNF217//RNF125//RNF114//MAEA//UBXN2B//RNF38//UBXN2A//FBXO45//RLIM//PML//UBE2D3//EDEM3//TRIM67//FBXO10//UCHL5//USP37//TP53INP2//USP46//USP45// |
| GO:0002577 | regulation of antigen processing and presentation | Biological process | 3 | 17 | 509 | 17653 | 6.12030509649832 | 0.0119899688986509 | 0.264987730252458 | 1.92118194343675 | 0.00589390962671906 | THBS1//CD74//WAS// |
| GO:0032303 | regulation of icosanoid secretion | Biological process | 3 | 17 | 509 | 17653 | 6.12030509649832 | 0.0119899688986509 | 0.264987730252458 | 1.92118194343675 | 0.00589390962671906 | CYP4F2//EDN1//IL1B// |
| GO:0042226 | interleukin-6 biosynthetic process | Biological process | 3 | 17 | 509 | 17653 | 6.12030509649832 | 0.0119899688986509 | 0.264987730252458 | 1.92118194343675 | 0.00589390962671906 | TIRAP//IL1B//PTAFR// |
| GO:0043174 | nucleoside salvage | Biological process | 3 | 17 | 509 | 17653 | 6.12030509649832 | 0.0119899688986509 | 0.264987730252458 | 1.92118194343675 | 0.00589390962671906 | PRTFDC1//UCK2//UCK1// |
| GO:0071380 | cellular response to prostaglandin E stimulus | Biological process | 3 | 17 | 509 | 17653 | 6.12030509649832 | 0.0119899688986509 | 0.264987730252458 | 1.92118194343675 | 0.00589390962671906 | PRKAA1//SFRP1//TNFSF4// |
| GO:0071636 | positive regulation of transforming growth factor beta production | Biological process | 3 | 17 | 509 | 17653 | 6.12030509649832 | 0.0119899688986509 | 0.264987730252458 | 1.92118194343675 | 0.00589390962671906 | FOXP3//THBS1//WNT11// |
| GO:1900273 | positive regulation of long-term synaptic potentiation | Biological process | 3 | 17 | 509 | 17653 | 6.12030509649832 | 0.0119899688986509 | 0.264987730252458 | 1.92118194343675 | 0.00589390962671906 | CRTC1//NRGN//SHANK3// |
| GO:1902043 | positive regulation of extrinsic apoptotic signaling pathway via death domain receptors | Biological process | 3 | 17 | 509 | 17653 | 6.12030509649832 | 0.0119899688986509 | 0.264987730252458 | 1.92118194343675 | 0.00589390962671906 | SFRP1//SKIL//THBS1// |
| GO:0048661 | positive regulation of smooth muscle cell proliferation | Biological process | 7 | 86 | 509 | 17653 | 2.82293142047791 | 0.0120537679149827 | 0.264987730252458 | 1.91887717475733 | 0.0137524557956778 | DDX39B//EDN1//FGFR2//HMOX1//PTAFR//TGM2//THBS1// |
| GO:0007528 | neuromuscular junction development | Biological process | 5 | 48 | 509 | 17653 | 3.61268009168304 | 0.0120680498177964 | 0.264987730252458 | 1.91836290569382 | 0.00982318271119843 | SHANK3//UNC13B//LRRK2//P2RX2//CACNA2D2// |
| GO:0045747 | positive regulation of Notch signaling pathway | Biological process | 5 | 48 | 509 | 17653 | 3.61268009168304 | 0.0120680498177964 | 0.264987730252458 | 1.91836290569382 | 0.00982318271119843 | ENHO//KIT//ASCL1//ZMIZ1//TSPAN14// |
| GO:0033043 | regulation of organelle organization | Biological process | 50 | 1253 | 509 | 17653 | 1.38394768077243 | 0.0121674218348036 | 0.266305093426462 | 1.91480143504316 | 0.0982318271119843 | DAPK3//TTK//KLHL22//CDKN1B//BRK1//WAS//ARHGAP40//EPS8L2//CDC42EP1//LMOD1//PARG//PAXBP1//FOXP3//MAPRE1//PML//NEK7//TNKS2//SHANK3//RHOF//DIXDC1//IL1B//ACTR3//ACTR2//ARFIP2//GPR3//DAZL//EDN1//CEP85//NF2//SFRP1//WNT11//PAX5//PRDM12//SPTBN2//BMP10//OGT//RASSF1//JAM3//LRRK2//MYO19//YWHAB//SLC25A5//SEPT7//EHD3//CAMKK2//PRKAA1//UBE2D3//KMT2E//ARHGDIB//PIP4K2C// |
| GO:0060078 | regulation of postsynaptic membrane potential | Biological process | 9 | 129 | 509 | 17653 | 2.41965550326678 | 0.0124612227119638 | 0.271758387168094 | 1.90443934202629 | 0.0176817288801572 | CHRNA3//LRRK2//P2RX2//GRIN2B//CELF4//UNC13B//SH3GL1//DLG4//SHANK3// |
| GO:0035966 | response to topologically incorrect protein | Biological process | 12 | 198 | 509 | 17653 | 2.10192296243377 | 0.012496947864746 | 0.271758387168094 | 1.90319604198014 | 0.0235756385068762 | DNAJB5//THBS1//PARP16//CTH//EDEM3//CREB3L3//KDELR3//ASNA1//TLN1//GOSR2//DNAJB12//COPS5// |
| GO:0032760 | positive regulation of tumor necrosis factor production | Biological process | 6 | 67 | 509 | 17653 | 3.10582646687975 | 0.0126245810021461 | 0.273653978581776 | 1.89878302679152 | 0.0117878192534381 | THBS1//LRRK2//TIRAP//NFATC4//PTAFR//ABCC8// |
| GO:0051726 | regulation of cell cycle | Biological process | 48 | 1197 | 509 | 17653 | 1.39074602025693 | 0.0127943950942158 | 0.276448862690676 | 1.89298024228593 | 0.0943025540275049 | CDKN1B//PML//CDKN2A//RASSF1//EIF4G2//VASH1//PRKAA1//PRKAB1//KMT2E//SKIL//THBS1//DAPK3//TTK//KLHL22//MAEA//NEK7//TTC28//MCIDAS//ASCL1//PAK2//PIM1//OXSR1//MAPRE1//SFRP1//SKP1//MYO19//GPR3//FGFR2//PGGT1B//TRIM21//FOSL1//DAZL//EDN1//IL1B//TTL//FZD3//CEP85//MED1//LSM11//TPRA1//DDX39B//GPNMB//COPS5//LIN9//MYBL2//NF2//BCR//NUP214// |
| GO:0060079 | excitatory postsynaptic potential | Biological process | 8 | 108 | 509 | 17653 | 2.56901695408572 | 0.0128930594951333 | 0.276850041642595 | 1.88964401327577 | 0.0157170923379175 | LRRK2//CELF4//SH3GL1//DLG4//SHANK3//CHRNA3//P2RX2//GRIN2B// |
| GO:0006814 | sodium ion transport | Biological process | 13 | 223 | 509 | 17653 | 2.02180482261006 | 0.012923933877537 | 0.276850041642595 | 1.88860527262083 | 0.0255402750491159 | NKAIN1//SCN3B//PRSS8//SCN1A//SCN7A//ATP1B4//SLC9A5//SLMAP//GLRX//SLC5A3//SLC10A7//SLC5A6//SLC23A2// |
| GO:0035587 | purinergic receptor signaling pathway | Biological process | 4 | 32 | 509 | 17653 | 4.33521611001965 | 0.0129594187959356 | 0.276850041642595 | 1.88741447526929 | 0.00785854616895874 | GPR34//PTAFR//P2RX2//CNTN2// |
| GO:0051239 | regulation of multicellular organismal process | Biological process | 105 | 2969 | 509 | 17653 | 1.22653470273375 | 0.0130424155907193 | 0.276850041642595 | 1.88464196530932 | 0.206286836935167 | GPNMB//AGPAT1//TNFSF4//OTP//FZD3//FOXP3//CD96//NDFIP1//CHRM1//EDN1//EMP2//WNT11//CHRNA3//DAPK3//GNAO1//KCNIP2//KCNJ12//AQP1//SCN3B//IL1B//THBS1//VASH1//BMP10//RTN4//TIAM1//SFRP1//ZNF804A//TRIM67//LRRK2//ABCC8//NF2//SEPT7//EFNA3//PML//RFX4//KREMEN1//TTL//ASCL1//CNTN2//TRIM21//RNF125//RNF135//NFKB1//POLR2E//TIRAP//REL//NFATC4//PTAFR//HMOX1//MED1//FGFR2//TNKS2//CACNA2D2//FOXS1//SLC6A3//PHOX2A//SLC5A3//HMGB3//CD74//ZMIZ1//RAG1//IL4R//TRIB1//KMT2E//NREP//DIXDC1//HEYL//SMAD1//FSHB//PROK1//DDAH1//SEMA7A//KIT//SHANK3//PRKAA1//RGMA//SEMA4G//SKIL//TLX2//CUX1//CCK//LRRTM1//LRRN1//EHD3//PIM1//FZD2//BCR//CDKN1B//ACTR2//DDX39B//KCNIP3//CACNG1//PAX8//DLG4//CRTC1//PACSIN1//VLDLR//ARHGDIB//ZNF322//ZSWIM6//CELF4//ITGA5//SYT2//CTH//LIMS2// |
| GO:0030968 | endoplasmic reticulum unfolded protein response | Biological process | 9 | 130 | 509 | 17653 | 2.40104276862627 | 0.0130585780399213 | 0.276850041642595 | 1.88410411119549 | 0.0176817288801572 | PARP16//KDELR3//ASNA1//TLN1//GOSR2//COPS5//CTH//EDEM3//CREB3L3// |
| GO:0032273 | positive regulation of protein polymerization | Biological process | 9 | 130 | 509 | 17653 | 2.40104276862627 | 0.0130585780399213 | 0.276850041642595 | 1.88410411119549 | 0.0176817288801572 | CDC42EP1//LMOD1//CDKN1B//MAPRE1//ACTR3//ACTR2//ARFIP2//WAS//BRK1// |
| GO:0060421 | positive regulation of heart growth | Biological process | 5 | 49 | 509 | 17653 | 3.53895192654665 | 0.0131295757902891 | 0.276954098927882 | 1.88174930551089 | 0.00982318271119843 | FGFR2//BMP10//PIM1//EDN1//DDX39B// |
| GO:0001944 | vasculature development | Biological process | 33 | 761 | 509 | 17653 | 1.50393830886358 | 0.0131453889924368 | 0.276954098927882 | 1.88122655792055 | 0.0648330058939096 | FGFR2//VASH1//HMOX1//ITGA5//MED1//RTN4//JAM3//PROK1//HS6ST1//FOXS1//ALDH1A2//EDN1//NFATC4//ZMIZ1//PPP3CB//PARVA//THBS1//EFNA3//PML//ABCC8//TAB1//GPNMB//EMP2//SFRP1//DDAH1//IL1B//AQP1//WNT11//FOSL1//NDP//SMAD1//CTH//KIT// |
| GO:0043161 | proteasome-mediated ubiquitin-dependent protein catabolic process | Biological process | 20 | 403 | 509 | 17653 | 1.72117761191847 | 0.0134419807871293 | 0.282323341811663 | 1.8715367296799 | 0.0392927308447937 | UBE4B//UBE2E1//FBXL3//SKP1//FBXL20//OGT//TRIB1//LRRK2//RNF217//RNF125//RNF114//EDEM3//MAEA//UBXN2B//RNF38//UBXN2A//FBXO45//RLIM//PML//UBE2D3// |
| GO:0009247 | glycolipid biosynthetic process | Biological process | 6 | 68 | 509 | 17653 | 3.06015254824916 | 0.0135194953064035 | 0.283072280982065 | 1.8690395206529 | 0.0117878192534381 | ST6GALNAC6//PYURF//PGAP3//GAL3ST1//ST8SIA5//PRKAA1// |
| GO:0034976 | response to endoplasmic reticulum stress | Biological process | 15 | 274 | 509 | 17653 | 1.89863479270933 | 0.0135737990775052 | 0.283332108522122 | 1.86729858362375 | 0.0294695481335953 | PARP16//UBE4B//CTH//EDEM3//CREB3L3//KDELR3//ASNA1//TLN1//GOSR2//DNAJB12//PML//LRRK2//COPS5//NRBF2//THBS1// |
| GO:0051603 | proteolysis involved in cellular protein catabolic process | Biological process | 30 | 679 | 509 | 17653 | 1.53232970015422 | 0.0139008336318443 | 0.286055794771078 | 1.85695915436073 | 0.0589390962671906 | UBE4B//RNF217//FBXO45//FBXO10//RLIM//UCHL5//RNF125//USP37//TP53INP2//USP46//UBE2D3//UBE2E1//USP45//FBXL3//SKP1//FBXL20//OGT//TRIB1//LRRK2//RNF114//DNAJB12//MAEA//UBXN2B//RNF38//UBXN2A//PML//ALAD//EDEM3//TRIM67//RNF123// |
| GO:0010769 | regulation of cell morphogenesis involved in differentiation | Biological process | 15 | 275 | 509 | 17653 | 1.89173066619039 | 0.0139909168747768 | 0.286055794771078 | 1.85415382370629 | 0.0294695481335953 | TTL//RTN4//CNTN2//SEMA7A//CHRNA3//SEMA4G//SKIL//TIAM1//TLX2//NFATC4//CUX1//LRRK2//SHANK3//ACTR2//ZSWIM6// |
| GO:0006206 | pyrimidine nucleobase metabolic process | Biological process | 3 | 18 | 509 | 17653 | 5.78028814669286 | 0.0140849592871165 | 0.286055794771078 | 1.85124440401411 | 0.00589390962671906 | DPYS//UCK2//UCK1// |
| GO:0006677 | glycosylceramide metabolic process | Biological process | 3 | 18 | 509 | 17653 | 5.78028814669286 | 0.0140849592871165 | 0.286055794771078 | 1.85124440401411 | 0.00589390962671906 | GAL3ST1//PRKAA1//ST6GALNAC6// |
| GO:0010829 | negative regulation of glucose transmembrane transport | Biological process | 3 | 18 | 509 | 17653 | 5.78028814669286 | 0.0140849592871165 | 0.286055794771078 | 1.85124440401411 | 0.00589390962671906 | PRKAA1//IL1B//RSC1A1// |
| GO:0030952 | establishment or maintenance of cytoskeleton polarity | Biological process | 3 | 18 | 509 | 17653 | 5.78028814669286 | 0.0140849592871165 | 0.286055794771078 | 1.85124440401411 | 0.00589390962671906 | AQP1//RHOF//MAPKAP1// |
| GO:0046639 | negative regulation of alpha-beta T cell differentiation | Biological process | 3 | 18 | 509 | 17653 | 5.78028814669286 | 0.0140849592871165 | 0.286055794771078 | 1.85124440401411 | 0.00589390962671906 | IL4R//TNFSF4//FOXP3// |
| GO:0051767 | nitric-oxide synthase biosynthetic process | Biological process | 3 | 18 | 509 | 17653 | 5.78028814669286 | 0.0140849592871165 | 0.286055794771078 | 1.85124440401411 | 0.00589390962671906 | LRRK2//EDN1//PGGT1B// |
| GO:0051769 | regulation of nitric-oxide synthase biosynthetic process | Biological process | 3 | 18 | 509 | 17653 | 5.78028814669286 | 0.0140849592871165 | 0.286055794771078 | 1.85124440401411 | 0.00589390962671906 | LRRK2//EDN1//PGGT1B// |
| GO:0032890 | regulation of organic acid transport | Biological process | 5 | 50 | 509 | 17653 | 3.46817288801572 | 0.0142523668382221 | 0.286295655118915 | 1.84611300797656 | 0.00982318271119843 | THBS1//CCK//CYP4F2//EDN1//IL1B// |
| GO:0051250 | negative regulation of lymphocyte activation | Biological process | 9 | 132 | 509 | 17653 | 2.36466333273799 | 0.0143167309522174 | 0.286295655118915 | 1.84415613646179 | 0.0176817288801572 | GPNMB//FOXP3//NDFIP1//HMGB3//SFRP1//CD74//TNFSF4//IL4R//TNFAIP8L2// |
| GO:0048639 | positive regulation of developmental growth | Biological process | 11 | 178 | 509 | 17653 | 2.14325290832432 | 0.0143490456573544 | 0.286295655118915 | 1.84317698252075 | 0.0216110019646365 | FOXS1//SLC6A3//SEMA7A//CACNA2D2//RTN4//FGFR2//BMP10//PIM1//EDN1//DDX39B//SYT2// |
| GO:0060333 | interferon-gamma-mediated signaling pathway | Biological process | 7 | 89 | 509 | 17653 | 2.72777642877641 | 0.0143697846296717 | 0.286295655118915 | 1.84254974090169 | 0.0137524557956778 | MED1//NCAM1//PML//TRIM68//PTAFR//TRIM21//TRIM5// |
| GO:0040013 | negative regulation of locomotion | Biological process | 18 | 353 | 509 | 17653 | 1.7684734273168 | 0.0143731657854229 | 0.286295655118915 | 1.84244756507974 | 0.0353634577603143 | HMOX1//BCR//VASH1//BMP10//THBS1//TRIB1//PTPRU//NF2//SFRP1//WNT11//SEMA4G//SEMA7A//ABCC8//ARHGDIB//PML//TRIM21//TRIM5//WAS// |
| GO:0002701 | negative regulation of production of molecular mediator of immune response | Biological process | 4 | 33 | 509 | 17653 | 4.20384592486754 | 0.0144188047690703 | 0.286295655118915 | 1.84107073848699 | 0.00785854616895874 | FOXP3//CD96//HMOX1//NDFIP1// |
| GO:0010039 | response to iron ion | Biological process | 4 | 33 | 509 | 17653 | 4.20384592486754 | 0.0144188047690703 | 0.286295655118915 | 1.84107073848699 | 0.00785854616895874 | HMOX1//ALAD//SLC6A3//C1QA// |
| GO:0006369 | termination of RNA polymerase II transcription | Biological process | 6 | 69 | 509 | 17653 | 3.01580251131801 | 0.0144578049027695 | 0.286295655118915 | 1.83989764002772 | 0.0117878192534381 | LSM11//SNRPB//U2AF1//DDX39B//SRSF9//RBM8A// |
| GO:0019941 | modification-dependent protein catabolic process | Biological process | 27 | 597 | 509 | 17653 | 1.56852040161515 | 0.0144777634260933 | 0.286295655118915 | 1.83929852423897 | 0.0530451866404715 | UBE4B//RNF217//FBXO45//FBXO10//RLIM//UCHL5//RNF125//USP37//TP53INP2//USP46//UBE2D3//UBE2E1//USP45//FBXL3//SKP1//FBXL20//OGT//TRIB1//LRRK2//RNF114//MAEA//UBXN2B//RNF38//UBXN2A//PML//EDEM3//TRIM67// |
| GO:0006986 | response to unfolded protein | Biological process | 11 | 179 | 509 | 17653 | 2.13127942838955 | 0.0149066692572128 | 0.293779997238152 | 1.8266193843677 | 0.0216110019646365 | PARP16//CTH//EDEM3//CREB3L3//KDELR3//ASNA1//TLN1//GOSR2//COPS5//DNAJB5//THBS1// |
| GO:0090276 | regulation of peptide hormone secretion | Biological process | 12 | 203 | 509 | 17653 | 2.05015146089599 | 0.0149685800118185 | 0.293779997238152 | 1.82481939687016 | 0.0235756385068762 | SYT9//DPP4//SLC25A5//IL1B//PPP3CB//ABCC8//CACNA2D2//SIRT3//VSNL1//SFRP1//TIAM1//PAX8// |
| GO:0070647 | protein modification by small protein conjugation or removal | Biological process | 45 | 1119 | 509 | 17653 | 1.39470759571678 | 0.0149865590783916 | 0.293779997238152 | 1.82429806994081 | 0.0884086444007859 | UBE4B//RNF217//MGRN1//FBXL3//FBXO10//RLIM//RNF125//RNF114//SKP1//TRIM21//UBE2D3//UBE2E1//TNKS2//FBXL20//COPS5//MAEA//RNF38//FBXO45//RNF149//MED1//DCAF16//TULP4//KLHL14//PEX5//RNF123//UBE2Z//HLTF//TRIM7//RNF135//KLHL22//RAG1//TAB1//USP54//SMAD1//UCHL5//USP37//USP46//OGT//USP45//NUP214//UBXN2A//LRRK2//NDFIP1//TRIM68//TRIM5// |
| GO:0072358 | cardiovascular system development | Biological process | 33 | 769 | 509 | 17653 | 1.48829265675577 | 0.0151126999148866 | 0.293859233829478 | 1.82065794112817 | 0.0648330058939096 | FGFR2//VASH1//HMOX1//ITGA5//MED1//RTN4//JAM3//PROK1//HS6ST1//FOXS1//ALDH1A2//EDN1//NFATC4//ZMIZ1//PPP3CB//PARVA//THBS1//EFNA3//PML//ABCC8//TAB1//GPNMB//EMP2//SFRP1//DDAH1//IL1B//AQP1//WNT11//FOSL1//NDP//SMAD1//CTH//KIT// |
| GO:0099531 | presynaptic process involved in chemical synaptic transmission | Biological process | 10 | 156 | 509 | 17653 | 2.22318774872802 | 0.0151259468251183 | 0.293859233829478 | 1.82027743068844 | 0.0196463654223969 | UNC13B//CPLX1//SNPH//CHRNA3//SYT2//SYT9//SYT14//CPLX3//SLC6A3//LRRK2// |
| GO:0071902 | positive regulation of protein serine/threonine kinase activity | Biological process | 18 | 355 | 509 | 17653 | 1.75851019674036 | 0.0151419424103482 | 0.293859233829478 | 1.8198184099095 | 0.0353634577603143 | TAB1//LRRK2//RAF1//IL1B//KIT//PRKAA1//THBS1//PROK1//CD74//CD81//TIRAP//MAP3K9//DBNL//EDN1//S100A12//TIAM1//CDKN1B//PARP16// |
| GO:0060996 | dendritic spine development | Biological process | 7 | 90 | 509 | 17653 | 2.69746780179 | 0.015207856253189 | 0.293859233829478 | 1.81793200113616 | 0.0137524557956778 | CTNND2//DLG4//SHANK3//LRRK2//ACTR2//TIAM1//PAK2// |
| GO:1905269 | positive regulation of chromatin organization | Biological process | 7 | 90 | 509 | 17653 | 2.69746780179 | 0.015207856253189 | 0.293859233829478 | 1.81793200113616 | 0.0137524557956778 | PAXBP1//IL1B//FOXP3//PRDM12//OGT//LRRK2//KMT2E// |
| GO:0051046 | regulation of secretion | Biological process | 32 | 741 | 509 | 17653 | 1.49772648335362 | 0.0152733290846887 | 0.294283545868233 | 1.81606629067575 | 0.0628683693516699 | CD74//EDN1//SYT9//DPP4//SLC25A5//IL1B//PPP3CB//ABCC8//CACNA2D2//AQP1//UNC13B//CCK//CHRNA3//CPLX1//LGI3//SYT2//SYT14//SIRT3//CYP4F2//VSNL1//HMOX1//IL4R//BCR//PTAFR//SFRP1//CPLX3//FOXP3//PML//TNFSF4//TIAM1//LRRK2//PAX8// |
| GO:1903557 | positive regulation of tumor necrosis factor superfamily cytokine production | Biological process | 6 | 70 | 509 | 17653 | 2.97271961829919 | 0.0154404821314215 | 0.296659035951147 | 1.81133914287678 | 0.0117878192534381 | TIRAP//NFATC4//PTAFR//ABCC8//THBS1//LRRK2// |
| GO:0006357 | regulation of transcription by RNA polymerase II | Biological process | 81 | 2227 | 509 | 17653 | 1.26143692828591 | 0.0157117906082941 | 0.30062398164489 | 1.80377431737971 | 0.159135559921415 | CUX1//EDN1//FGFR2//FOXS1//SATB2//ZNF281//DNAJB5//HEYL//KCNIP3//HIC1//ASCL1//NFKB1//PAX5//FOXP3//RLIM//MED1//CC2D1A//REL//SKIL//UBE2D3//VLDLR//NRIP1//BHLHE40//PAXBP1//MICAL2//DLX5//BMP10//SMAD1//RGMA//SFRP1//INSIG2//HMOX1//MED6//ARID3B//COPS5//PRDM4//ELK1//TET3//CRTC1//ATP1B4//FSHB//RAX//TLX2//MCIDAS//IL1B//PHOX2A//MYBL2//NFATC4//PML//PPP3CB//ZMIZ1//CALCOCO1//RAF1//RFX4//HLTF//TCF20//TEAD1//PAX8//FOSL1//CREB3L3//OGT//ONECUT2//CD81//FOXG1//OTP//CARHSP1//LIN9//HOXC6//HOXC9//KIT//FOXI2//NFYA//MBNL3//PTAFR//PURA//CREBZF//HIVEP3//PRDM12//TCF19//ZNF322//CREB5// |
| GO:0051240 | positive regulation of multicellular organismal process | Biological process | 61 | 1606 | 509 | 17653 | 1.31730103467596 | 0.0157357518116651 | 0.30062398164489 | 1.80311250283925 | 0.119842829076621 | AGPAT1//TNFSF4//OTP//FZD3//FOXP3//EDN1//AQP1//SCN3B//IL1B//THBS1//BMP10//RTN4//TIAM1//TRIM67//ZNF804A//NFKB1//POLR2E//RNF135//TIRAP//NFATC4//PTAFR//ABCC8//WNT11//MED1//RNF125//FOXS1//SLC6A3//HMOX1//ZMIZ1//RAG1//CD74//IL4R//TRIB1//HEYL//ASCL1//SMAD1//DDAH1//SEMA7A//FSHB//CACNA2D2//KIT//SHANK3//PRKAA1//SKIL//CUX1//LRRTM1//LRRN1//FGFR2//PIM1//ACTR2//DDX39B//PAX8//DLG4//CRTC1//PACSIN1//VLDLR//ZNF322//ITGA5//SYT2//LRRK2//CTH// |
| GO:0090066 | regulation of anatomical structure size | Biological process | 23 | 491 | 509 | 17653 | 1.62460237116826 | 0.0158647987947873 | 0.301002852066817 | 1.79956543146573 | 0.0451866404715128 | CHRM1//SLC12A7//AQP1//BRK1//WAS//HMOX1//PTAFR//EDN1//TTL//RTN4//ARHGAP40//EPS8L2//CDC42EP1//LMOD1//CNTN2//ACTR3//ACTR2//ARFIP2//LRRK2//SEMA7A//SHANK3//SEMA4G//SPTBN2// |
| GO:0110053 | regulation of actin filament organization | Biological process | 14 | 254 | 509 | 17653 | 1.91159135559921 | 0.0158697032824355 | 0.301002852066817 | 1.7994311932203 | 0.0275049115913556 | BRK1//WAS//ARHGAP40//EPS8L2//CDC42EP1//LMOD1//SHANK3//ACTR3//ACTR2//ARFIP2//NF2//SFRP1//WNT11//SPTBN2// |
| GO:0009416 | response to light stimulus | Biological process | 16 | 305 | 509 | 17653 | 1.8193693838771 | 0.0159561392458226 | 0.301002852066817 | 1.79707218235038 | 0.031434184675835 | CTNS//KIT//RAG1//ABCC8//UBE4B//PML//PRKAA1//AQP1//NFATC4//CRTC1//FBXL3//BHLHE40//RGS9BP//SEMA5B//BEST1//ELK1// |
| GO:0031062 | positive regulation of histone methylation | Biological process | 4 | 34 | 509 | 17653 | 4.08020339766555 | 0.015978119753362 | 0.301002852066817 | 1.79647432819998 | 0.00785854616895874 | PRDM12//OGT//KMT2E//PAXBP1// |
| GO:0070670 | response to interleukin-4 | Biological process | 4 | 34 | 509 | 17653 | 4.08020339766555 | 0.015978119753362 | 0.301002852066817 | 1.79647432819998 | 0.00785854616895874 | IL4R//ALAD//MRC1//PML// |
| GO:0097327 | response to antineoplastic agent | Biological process | 7 | 91 | 509 | 17653 | 2.66782529847363 | 0.0160801246564573 | 0.302083008476724 | 1.79371058883373 | 0.0137524557956778 | EDN1//PTAFR//AQP1//NUCB1//HMOX1//LARP1//PRKAA1// |
| GO:0016048 | detection of temperature stimulus | Biological process | 3 | 19 | 509 | 17653 | 5.47606245476166 | 0.0163741981110195 | 0.303082948356892 | 1.78583995939644 | 0.00589390962671906 | MMP24//PRDM12//ANO3// |
| GO:0033622 | integrin activation | Biological process | 3 | 19 | 509 | 17653 | 5.47606245476166 | 0.0163741981110195 | 0.303082948356892 | 1.78583995939644 | 0.00589390962671906 | FERMT2//TLN1//FERMT3// |
| GO:0035994 | response to muscle stretch | Biological process | 3 | 19 | 509 | 17653 | 5.47606245476166 | 0.0163741981110195 | 0.303082948356892 | 1.78583995939644 | 0.00589390962671906 | EDN1//NFKB1//RAF1// |
| GO:0048025 | negative regulation of mRNA splicing, via spliceosome | Biological process | 3 | 19 | 509 | 17653 | 5.47606245476166 | 0.0163741981110195 | 0.303082948356892 | 1.78583995939644 | 0.00589390962671906 | CELF4//PTBP1//SRSF9// |
| GO:0003205 | cardiac chamber development | Biological process | 10 | 158 | 509 | 17653 | 2.19504613165552 | 0.0164022414754728 | 0.303082948356892 | 1.78509679866503 | 0.0196463654223969 | FGFR2//PARVA//FZD2//HEYL//UBE4B//MED1//TAB1//PAX8//BMP10//WNT11// |
| GO:0055067 | monovalent inorganic cation homeostasis | Biological process | 10 | 158 | 509 | 17653 | 2.19504613165552 | 0.0164022414754728 | 0.303082948356892 | 1.78509679866503 | 0.0196463654223969 | EDN1//ATP1B4//CA7//LRRK2//SLC26A1//SLC9A5//SLC12A7//SCN7A//CYP4F2//ATP6V1G2// |
| GO:0007179 | transforming growth factor beta receptor signaling pathway | Biological process | 11 | 182 | 509 | 17653 | 2.09614844880071 | 0.0166776119232917 | 0.306676155862905 | 1.77786613611955 | 0.0216110019646365 | NREP//THBS1//HTRA4//SKIL//RASL11B//ONECUT2//TAB1//FERMT2//FSHB//SMAD1//PML// |
| GO:0065002 | intracellular protein transmembrane transport | Biological process | 5 | 52 | 509 | 17653 | 3.33478162309204 | 0.0166873910036299 | 0.306676155862905 | 1.77761155794613 | 0.00982318271119843 | PEX5//TIMM23//TOMM40L//TIMM22//BCR// |
| GO:0006793 | phosphorus metabolic process | Biological process | 116 | 3352 | 509 | 17653 | 1.20020302807226 | 0.0168181432149811 | 0.30747174480273 | 1.77422195360353 | 0.227897838899804 | CDKN1B//CAMKK2//LRRK2//DLG4//FGFR3//FGFR2//GRIN2B//IL1B//KIT//SMAD1//NCAM1//MAPK4//RAF1//SPTBN2//YWHAB//SHANK3//TAB1//PRKAA1//THBS1//PROK1//CD74//CD81//CCDC88C//PML//GPNMB//DLAT//NUP214//OGT//GPD2//AQP1//UCK2//SGK2//GLYCTK//NEK7//DAPK3//MARK1//MAP3K9//PAK2//PIM1//PPP3CB//PRKAB1//BCR//WNT11//MEX3B//AATK//OXSR1//TRIB1//LRRTM1//NF2//CEP85//PTPRU//PTPRT//PTPN18//PGAP3//PYURF//DBI//ACBD6//AGPAT1//PLA2G4C//LCLAT1//ABHD5//PCTP//PIP4K2C//SMPD1//NFS1//IMPA2//PRUNE2//PARP16//CHRNA3//GRM2//PALM//TIRAP//ARHGEF6//DBNL//IDI1//HAAO//BMP10//TTK//PPP1R26//UCK1//CUX1//ALDOC//FZD2//CDKN2A//EMP2//MAPKAP1//PRTFDC1//PPP1R2//PTAFR//EDN1//S100A12//SPRY3//RNF149//TRIM5//TIAM1//SFRP1//NRBF2//WDR91//PIK3IP1//UBXN2B//UBXN2A//VLDLR//CTNS//COPS5//DGKG//SULT4A1//SLC26A1//ITGA5//CCK//NFKB1//SKP1//SH3BP5//MED1//SIRT3//SEMA7A//PARG// |
| GO:0006796 | phosphate-containing compound metabolic process | Biological process | 113 | 3255 | 509 | 17653 | 1.2040047199563 | 0.0168216095781473 | 0.30747174480273 | 1.77413245101949 | 0.222003929273084 | CDKN1B//CAMKK2//LRRK2//DLG4//FGFR3//FGFR2//GRIN2B//IL1B//KIT//SMAD1//NCAM1//MAPK4//RAF1//SPTBN2//YWHAB//SHANK3//TAB1//PRKAA1//THBS1//PROK1//CD74//CD81//CCDC88C//PML//GPNMB//NUP214//OGT//GPD2//AQP1//UCK2//SGK2//GLYCTK//NEK7//DAPK3//MARK1//MAP3K9//PAK2//PIM1//PPP3CB//PRKAB1//BCR//WNT11//MEX3B//AATK//OXSR1//TRIB1//LRRTM1//NF2//CEP85//PTPRU//PTPRT//PTPN18//PGAP3//PYURF//AGPAT1//PLA2G4C//LCLAT1//ABHD5//PCTP//PIP4K2C//SMPD1//NFS1//PARP16//CHRNA3//GRM2//PALM//TIRAP//ARHGEF6//DBNL//IDI1//HAAO//BMP10//TTK//PPP1R26//UCK1//CUX1//ALDOC//FZD2//CDKN2A//EMP2//MAPKAP1//PRTFDC1//PPP1R2//PTAFR//DBI//EDN1//S100A12//SPRY3//RNF149//TRIM5//TIAM1//SFRP1//NRBF2//WDR91//PIK3IP1//IMPA2//UBXN2B//UBXN2A//VLDLR//CTNS//COPS5//DGKG//SULT4A1//SLC26A1//ITGA5//CCK//NFKB1//SKP1//SH3BP5//MED1//SIRT3//SEMA7A//PARG// |
| GO:1902106 | negative regulation of leukocyte differentiation | Biological process | 7 | 92 | 509 | 17653 | 2.63882719740326 | 0.0169872716418188 | 0.309188647566806 | 1.76987636839717 | 0.0137524557956778 | HMGB3//SFRP1//CD74//TNFSF4//IL4R//TRIB1//FOXP3// |
| GO:0008283 | cell proliferation | Biological process | 76 | 2078 | 509 | 17653 | 1.26843666741672 | 0.0170240402481021 | 0.309188647566806 | 1.76893736262823 | 0.149312377210216 | TAF8//HMOX1//ALDH1A2//VASH1//THBS1//OTP//FZD3//FGFR2//ASCL1//CDKN1B//CHRM1//DPP4//EDN1//EMP2//FGFR3//FSHB//SLC25A5//IL1B//KIT//PGGT1B//PRKAA1//BRK1//PURA//SFRP1//TIAM1//TTK//TNFSF4//FOSL1//PROK1//CCK//CD81//PTPRU//CDKN2A//GPNMB//DDAH1//LDOC1//SMAD1//NF2//DEC1//FOXP3//PML//RAF1//MED1//PAXBP1//DIXDC1//TIRAP//CD74//RTN4//PPP3CB//TNFSF14//CD79A//CD209//SGK2//IL4R//BEX4//RELT//NDFIP1//KRT2//AQP1//ZMIZ1//PTAFR//TGM2//TRIB1//DLX5//BMP10//PIM1//ABCC8//WNT11//LRRK2//DDX39B//LIMS2//PRDM4//MAPRE1//LARP1//NDP//TCF19// |
| GO:2001252 | positive regulation of chromosome organization | Biological process | 10 | 159 | 509 | 17653 | 2.18124081007278 | 0.0170695260595698 | 0.309188647566806 | 1.76777853703237 | 0.0196463654223969 | PAXBP1//PML//NEK7//TNKS2//IL1B//FOXP3//PRDM12//OGT//LRRK2//KMT2E// |
| GO:0031056 | regulation of histone modification | Biological process | 9 | 136 | 509 | 17653 | 2.29511441118687 | 0.0170984110882723 | 0.309188647566806 | 1.76704424561119 | 0.0176817288801572 | PAXBP1//FOXP3//IL1B//PAX5//PRDM12//OGT//LRRK2//KMT2E//PARG// |
| GO:0043632 | modification-dependent macromolecule catabolic process | Biological process | 27 | 606 | 509 | 17653 | 1.54522554416542 | 0.0172423137423379 | 0.31095938090515 | 1.76340445671856 | 0.0530451866404715 | UBE4B//RNF217//FBXO45//FBXO10//RLIM//UCHL5//RNF125//USP37//TP53INP2//USP46//UBE2D3//UBE2E1//USP45//FBXL3//SKP1//FBXL20//OGT//TRIB1//LRRK2//RNF114//MAEA//UBXN2B//RNF38//UBXN2A//PML//EDEM3//TRIM67// |
| GO:0001505 | regulation of neurotransmitter levels | Biological process | 16 | 308 | 509 | 17653 | 1.80164825351466 | 0.0173358227207766 | 0.311814279416522 | 1.76105554283857 | 0.031434184675835 | SLC6A3//UNC13B//CPLX1//SNPH//CHRNA3//SYT2//SYT9//SYT14//CPLX3//CHDH//DPYS//PRIMA1//EDN1//DDAH1//IL1B//LRRK2// |
| GO:0032680 | regulation of tumor necrosis factor production | Biological process | 8 | 114 | 509 | 17653 | 2.43380553544963 | 0.0173888565747332 | 0.311938559721275 | 1.75972897461662 | 0.0157170923379175 | GPNMB//FOXP3//TIRAP//NFATC4//PTAFR//ABCC8//THBS1//LRRK2// |
| GO:0002707 | negative regulation of lymphocyte mediated immunity | Biological process | 4 | 35 | 509 | 17653 | 3.96362615773225 | 0.017639317554489 | 0.314761753617438 | 1.75351822124801 | 0.00785854616895874 | PPP3CB//FOXP3//CD96//NDFIP1// |
| GO:0090022 | regulation of neutrophil chemotaxis | Biological process | 4 | 35 | 509 | 17653 | 3.96362615773225 | 0.017639317554489 | 0.314761753617438 | 1.75351822124801 | 0.00785854616895874 | TIRAP//EDN1//CD74//JAM3// |
| GO:0007159 | leukocyte cell-cell adhesion | Biological process | 17 | 335 | 509 | 17653 | 1.75996833123186 | 0.0177878528570375 | 0.315837204505583 | 1.74987647173721 | 0.0333988212180747 | DPP4//PAK2//TNFSF14//FOXP3//CD209//IL1B//TNFSF4//GPNMB//NDFIP1//CD74//ZMIZ1//RAG1//IL4R//TNFAIP8L2//PTAFR//ITGA5//FERMT3// |
| GO:0043583 | ear development | Biological process | 12 | 208 | 509 | 17653 | 2.00086897385522 | 0.0177947149445576 | 0.315837204505583 | 1.74970896461418 | 0.0235756385068762 | FGFR2//WDR19//DLX5//INSIG2//BCR//PAX8//FZD3//KCNQ4//EDN1//CDKN1B//CXCL14//FZD2// |
| GO:0009967 | positive regulation of signal transduction | Biological process | 60 | 1586 | 509 | 17653 | 1.31204522875752 | 0.0178396883219182 | 0.315837204505583 | 1.7486127374741 | 0.117878192534381 | TAB1//LRRK2//RAF1//IL1B//KIT//PRKAA1//THBS1//PROK1//CD74//CD81//AGPAT1//TIRAP//MAP3K9//DBNL//BMP10//TTK//FGFR2//SFRP1//ITGA5//MED1//WDFY1//FGFR3//CTH//HMOX1//CC2D1A//REL//S100A12//TGM2//NDFIP1//TRIM5//EDN1//TIAM1//HIC1//SHKBP1//ENHO//ASCL1//ZMIZ1//TSPAN14//EVC//P2RX2//RTN4//CNTN2//GPNMB//SEMA7A//WNT11//DAPK3//DLX5//NFKB1//TNKS2//DIXDC1//UNC13B//SHANK3//YWHAB//SKIL//DLG4//EMP2//LIMS2//NFATC4//PAK2//PML// |
| GO:0002709 | regulation of T cell mediated immunity | Biological process | 5 | 53 | 509 | 17653 | 3.27186121510917 | 0.0180022991820305 | 0.317883940908805 | 1.74467202499101 | 0.00982318271119843 | PPP3CB//WAS//IL1B//FOXP3//TNFSF4// |
| GO:0002700 | regulation of production of molecular mediator of immune response | Biological process | 8 | 115 | 509 | 17653 | 2.41264200905441 | 0.0182353008829531 | 0.320160469641425 | 1.73908706642049 | 0.0157170923379175 | IL4R//FOXP3//TNFSF4//CD96//HMOX1//NDFIP1//SEMA7A//CD74// |
| GO:0007178 | transmembrane receptor protein serine/threonine kinase signaling pathway | Biological process | 17 | 336 | 509 | 17653 | 1.75473033024605 | 0.0182558619073492 | 0.320160469641425 | 1.73859765804325 | 0.0333988212180747 | TAB1//FERMT2//FSHB//SMAD1//PML//SKIL//BMP10//TTK//NREP//DLX5//RGMA//UBE2D3//THBS1//HTRA4//RASL11B//ONECUT2//SFRP1// |
| GO:0032102 | negative regulation of response to external stimulus | Biological process | 16 | 310 | 509 | 17653 | 1.79002471639521 | 0.0183054570716364 | 0.320160469641425 | 1.73741942268459 | 0.031434184675835 | FOXP3//EDN1//TRIB1//CCK//RNF125//RGMA//SEMA4G//SEMA7A//NFKB1//BCR//TNFAIP8L2//NDFIP1//THBS1//SLC6A3//ABCC8//RTN4// |
| GO:0045597 | positive regulation of cell differentiation | Biological process | 38 | 926 | 509 | 17653 | 1.42322429529803 | 0.0183205828406375 | 0.320160469641425 | 1.73706071404992 | 0.0746561886051081 | OTP//FZD3//IL1B//TIAM1//NFKB1//TRIM67//ZNF804A//FOXP3//TNFSF4//ZMIZ1//RAG1//CD74//SFRP1//MED1//IL4R//TRIB1//TNFSF14//HEYL//ASCL1//SMAD1//SEMA7A//KIT//SHANK3//RTN4//SKIL//CUX1//EDN1//BMP10//ACTR2//DDX39B//PAX8//DLG4//CRTC1//PACSIN1//VLDLR//SYT2//CTH//NF2// |
| GO:0009605 | response to external stimulus | Biological process | 83 | 2306 | 509 | 17653 | 1.24830160323202 | 0.0184240133087264 | 0.320883647062169 | 1.73461576132778 | 0.163064833005894 | S100A12//KIT//CREB3L3//FOXP3//AP1S1//CREBZF//TNFSF4//FOSL1//RALBP1//PTAFR//CXCL14//THBS1//EFNA3//NCAM1//SPTBN2//CNTN2//VLDLR//SHANK3//UNC5A//SLC27A4//OGT//TIRAP//LRRK2//SFRP1//ALAD//TNFSF14//ANO3//IL1B//AQP1//NFKB1//WNT11//EDN1//C1QA//CD81//TRIM5//TRIB1//PRKAA1//HMOX1//CCK//CD96//FGFR2//ABCC8//RELT//ALDH1A2//P2RX2//RAF1//FZD3//RNF125//IL4R//CRTC1//FBXL3//PML//BHLHE40//RGMA//RTN4//SEMA4G//SEMA7A//BCR//TNFAIP8L2//NDFIP1//TGM2//ASCL1//RGS9BP//SEMA5B//BEST1//GPNMB//MMP24//PRDM12//SCN1A//USP46//SLC6A3//BIN2//PIM1//MED1//MRC1//PARVA//JAM3//CD74//DLX5//UNC13B//ZSWIM6//TIAM1//LARP1// |
| GO:0048513 | animal organ development | Biological process | 118 | 3428 | 509 | 17653 | 1.19382847370436 | 0.0184568591907709 | 0.320883647062169 | 1.7338422010441 | 0.231827111984283 | KIT//NRIP1//FGFR2//SMAD1//SFRP1//PAX8//SEMA4G//SEMA7A//ALDH1A2//WNT11//MED1//DDX39B//ONECUT2//CDKN1B//NDP//LDOC1//FZD3//CCDC103//MICAL2//DLX5//FGFR3//SNX19//CTNS//JAM3//RAG1//FOXP3//NDFIP1//TAB1//ZMIZ1//PARVA//FZD2//HEYL//UBE4B//KRT2//PAX5//FOXG1//GRIN2B//CA10//BCR//SNPH//EDN1//NFATC4//PPP3CB//RAF1//BMP10//EVC//PAXBP1//CSN3//SLC6A3//WDR19//RNF38//MEA1//TIAM1//IL1B//RFX4//VLDLR//PHOX2A//ASCL1//NRGN//AQP1//UCHL5//SPTBN2//LRRK2//SYPL2//MAPKAP1//NF2//SHANK3//FBXO45//DIXDC1//RTN4//CNTN2//RAX//OTP//SATB2//EBP//TIRAP//PML//CD79A//KMT2E//GPNMB//GNAO1//PTPRU//KRT9//KRTAP1-1//KRT38//HIVEP3//INSIG2//KCNQ4//FSHB//GPD2//MAEA//TNFSF4//SLC27A4//CD74//HMGB3//IL4R//TRIB1//THBS1//HS6ST1//EXOC4//TEAD1//PRKAA1//HOXC9//MYOM3//P2RX2//C11ORF88//CXCL14//PIM1//TGM2//FOSL1//VASH1//PRSS8//SKIL//NPHS2//HMOX1//CELF4//LIMS2//SPRY3// |
| GO:0045582 | positive regulation of T cell differentiation | Biological process | 6 | 73 | 509 | 17653 | 2.85055305864305 | 0.0186640834241663 | 0.323417961933289 | 1.72899333301338 | 0.0117878192534381 | FOXP3//TNFSF4//IL4R//ZMIZ1//RAG1//CD74// |
| GO:0072359 | circulatory system development | Biological process | 44 | 1105 | 509 | 17653 | 1.38099191920988 | 0.0186982734165187 | 0.323417961933289 | 1.72819849401417 | 0.0864440078585462 | FGFR2//VASH1//HMOX1//ITGA5//MED1//RTN4//JAM3//PROK1//HS6ST1//FOXS1//ALDH1A2//EDN1//NFATC4//ZMIZ1//PPP3CB//CCDC103//MICAL2//PARVA//THBS1//TAB1//FZD2//HEYL//WNT11//UBE4B//PAX8//RAF1//BMP10//EFNA3//PML//ABCC8//GPNMB//EMP2//SFRP1//DDAH1//IL1B//AQP1//MYOM3//SMAD1//PIM1//FOSL1//DDX39B//NDP//CTH//KIT// |
| GO:0071305 | cellular response to vitamin D | Biological process | 3 | 20 | 509 | 17653 | 5.20225933202358 | 0.0188593423475558 | 0.324543848082748 | 1.72447345580905 | 0.00589390962671906 | PIM1//MED1//SFRP1// |
| GO:2000515 | negative regulation of CD4-positive, alpha-beta T cell activation | Biological process | 3 | 20 | 509 | 17653 | 5.20225933202358 | 0.0188593423475558 | 0.324543848082748 | 1.72447345580905 | 0.00589390962671906 | IL4R//TNFSF4//FOXP3// |
| GO:0010976 | positive regulation of neuron projection development | Biological process | 14 | 260 | 509 | 17653 | 1.86747770893154 | 0.0190257940092608 | 0.325014142148891 | 1.72065720963519 | 0.0275049115913556 | SEMA7A//RTN4//SKIL//TIAM1//CUX1//SHANK3//ACTR2//DLG4//CRTC1//PACSIN1//VLDLR//SYT2//TRIM67//ZNF804A// |
| GO:0071804 | cellular potassium ion transport | Biological process | 12 | 210 | 509 | 17653 | 1.98181307886612 | 0.0190308443428894 | 0.325014142148891 | 1.72054194290988 | 0.0235756385068762 | SLC12A7//ATP1B4//KCNJ12//KCNIP2//KCNIP3//AQP1//KCNA6//KCNQ5//SLC9A5//ABCC8//KCNQ4//OXSR1// |
| GO:0071805 | potassium ion transmembrane transport | Biological process | 12 | 210 | 509 | 17653 | 1.98181307886612 | 0.0190308443428894 | 0.325014142148891 | 1.72054194290988 | 0.0235756385068762 | SLC12A7//ATP1B4//KCNJ12//KCNIP2//OXSR1//KCNIP3//ABCC8//AQP1//KCNA6//KCNQ5//SLC9A5//KCNQ4// |
| GO:0046777 | protein autophosphorylation | Biological process | 13 | 235 | 509 | 17653 | 1.91856372528529 | 0.0191461895986942 | 0.325984863882441 | 1.71791764471988 | 0.0255402750491159 | GPNMB//CAMKK2//LRRK2//DAPK3//FGFR3//FGFR2//KIT//MAP3K9//PAK2//PIM1//BCR//TTK//MEX3B// |
| GO:0030833 | regulation of actin filament polymerization | Biological process | 10 | 162 | 509 | 17653 | 2.1408474617381 | 0.0191918446384698 | 0.325984863882441 | 1.71688328072011 | 0.0196463654223969 | CDC42EP1//LMOD1//ACTR3//ACTR2//ARFIP2//WAS//SPTBN2//BRK1//ARHGAP40//EPS8L2// |
| GO:0008104 | protein localization | Biological process | 93 | 2628 | 509 | 17653 | 1.22732145580465 | 0.0192322875482913 | 0.325984863882441 | 1.71596905634839 | 0.182711198428291 | SPRN//PML//NUP214//U2AF1//DDX39B//SRSF9//RBM8A//YWHAB//GOLGA7B//EXOC4//KDELR3//GOSR2//TIMM23//TOMM34//TIMM22//VPS45//AP1S1//ARFIP2//RABL3//KCNIP3//MLPH//VPS26A//CD74//TLN1//SYT9//DPP4//SLC25A5//IL1B//PPP3CB//ABCC8//CACNA2D2//EHD3//VPS53//FAM160A2//SCAMP1//PEX5//CPLX1//SNX19//CPLX3//TOMM40L//SIRT3//REEP2//TRIM5//LRRK2//MED1//ATP6V1G2//PTPRU//EMP2//WNT11//VLDLR//TTK//MIS12//SEPT7//MAPRE1//DLG4//LRRTM1//BSN//RAF1//VSNL1//SYS1//SFRP1//S100A12//FOXP3//UNC13B//TNFSF4//NDFIP1//TAF8//SKP1//WDR19//TIAM1//CD81//TULP4//BCR//TNKS2//CNTN2//FERMT2//PACSIN1//PALM//SCN3B//SLMAP//TSPAN14//IL4R//CHRM1//SHANK3//GRIPAP1//NF2//NUCB1//TP53INP2//PRKAA1//UBE2D3//RTN4//AKAP10//PLLP// |
| GO:0007405 | neuroblast proliferation | Biological process | 5 | 54 | 509 | 17653 | 3.21127119260715 | 0.0193838188034945 | 0.326443823444297 | 1.71256065855948 | 0.00982318271119843 | OTP//FZD3//FGFR2//LRRK2//ASCL1// |
| GO:0022029 | telencephalon cell migration | Biological process | 5 | 54 | 509 | 17653 | 3.21127119260715 | 0.0193838188034945 | 0.326443823444297 | 1.71256065855948 | 0.00982318271119843 | FBXO45//DIXDC1//RTN4//CNTN2//LRRK2// |
| GO:0035418 | protein localization to synapse | Biological process | 4 | 36 | 509 | 17653 | 3.85352543112857 | 0.0194041722644695 | 0.326443823444297 | 1.71210487849163 | 0.00785854616895874 | GRIPAP1//DLG4//LRRTM1//BSN// |
| GO:0097435 | supramolecular fiber organization | Biological process | 28 | 641 | 509 | 17653 | 1.5149585158259 | 0.0195577338991929 | 0.328022154410543 | 1.7086814671399 | 0.0550098231827112 | RHOF//MICAL2//MYOM3//EMP2//LMOD1//PACSIN1//BRK1//CGNL1//KIF2A//WAS//ARHGAP40//EPS8L2//CDC42EP1//MAPRE1//CDKN1B//CCDC88C//SHANK3//ACTR3//ACTR2//ARFIP2//KRT2//KRT9//BMP10//NF2//SFRP1//WNT11//SPTBN2//EDN1// |
| GO:0046883 | regulation of hormone secretion | Biological process | 14 | 261 | 509 | 17653 | 1.86032262192414 | 0.0195949948812449 | 0.328022154410543 | 1.7078548456356 | 0.0275049115913556 | SYT9//DPP4//SLC25A5//IL1B//PPP3CB//ABCC8//CACNA2D2//CHRNA3//EDN1//SIRT3//VSNL1//SFRP1//TIAM1//PAX8// |
| GO:0032271 | regulation of protein polymerization | Biological process | 12 | 211 | 509 | 17653 | 1.97242059981937 | 0.0196724611510709 | 0.328505814233809 | 1.70614130365639 | 0.0235756385068762 | ARHGAP40//EPS8L2//CDC42EP1//LMOD1//MAPRE1//CDKN1B//ACTR3//ACTR2//ARFIP2//WAS//SPTBN2//BRK1// |
| GO:0001707 | mesoderm formation | Biological process | 6 | 74 | 509 | 17653 | 2.81203207136409 | 0.0198334665591727 | 0.328564078632009 | 1.70260137172796 | 0.0117878192534381 | SMAD1//FGFR2//EXOC4//WNT11//TLX2//NF2// |
| GO:0016126 | sterol biosynthetic process | Biological process | 6 | 74 | 509 | 17653 | 2.81203207136409 | 0.0198334665591727 | 0.328564078632009 | 1.70260137172796 | 0.0117878192534381 | EBP//IDI1//INSIG2//PRKAA1//NFYA//FAXDC2// |
| GO:0006892 | post-Golgi vesicle-mediated transport | Biological process | 7 | 95 | 509 | 17653 | 2.55549581222211 | 0.0199246174522303 | 0.328564078632009 | 1.70061000819937 | 0.0137524557956778 | STEAP2//EXOC4//TGOLN2//SYS1//GOSR2//EHD3//SCAMP1// |
| GO:0019233 | sensory perception of pain | Biological process | 7 | 95 | 509 | 17653 | 2.55549581222211 | 0.0199246174522303 | 0.328564078632009 | 1.70061000819937 | 0.0137524557956778 | MMP24//PRDM12//SCN1A//EDN1//CCK//PTAFR//SCN3B// |
| GO:0042035 | regulation of cytokine biosynthetic process | Biological process | 7 | 95 | 509 | 17653 | 2.55549581222211 | 0.0199246174522303 | 0.328564078632009 | 1.70061000819937 | 0.0137524557956778 | FOXP3//THBS1//HMOX1//IL1B//NFKB1//TIRAP//PTAFR// |
| GO:2000147 | positive regulation of cell motility | Biological process | 23 | 502 | 509 | 17653 | 1.58900351442951 | 0.0200046667396536 | 0.328564078632009 | 1.69886867919301 | 0.0451866404715128 | EDN1//THBS1//RTN4//TNFSF14//GPNMB//DAPK3//MAPRE1//FSHB//IL1B//ITGA5//KIT//SEMA4G//TIAM1//WNT11//FERMT3//SEMA7A//ONECUT2//TIRAP//CD74//HMOX1//TAC4//PTAFR//CXCL14// |
| GO:0032640 | tumor necrosis factor production | Biological process | 8 | 117 | 509 | 17653 | 2.37140026530989 | 0.0200160284483791 | 0.328564078632009 | 1.69862209039619 | 0.0157170923379175 | GPNMB//FOXP3//TIRAP//NFATC4//PTAFR//ABCC8//THBS1//LRRK2// |
| GO:0048598 | embryonic morphogenesis | Biological process | 26 | 586 | 509 | 17653 | 1.53877977966568 | 0.020115331129453 | 0.329394635420074 | 1.69647281387519 | 0.0510805500982318 | TLX2//NF2//SMAD1//FZD3//CCDC103//MICAL2//ZNF281//FGFR2//ALDH1A2//DLX5//WDR19//MED1//ITGA5//INSIG2//BCR//PAX8//KCNQ4//EDN1//EXOC4//WNT11//PAX5//SATB2//HOXC9//FZD2//SFRP1//C2ORF49// |
| GO:0046165 | alcohol biosynthetic process | Biological process | 9 | 140 | 509 | 17653 | 2.22953971372439 | 0.0202555917428158 | 0.330890258349428 | 1.69345506490272 | 0.0176817288801572 | IMPA2//EBP//IDI1//INSIG2//PRKAA1//NFKB1//PTAFR//NFYA//IL1B// |
| GO:0030163 | protein catabolic process | Biological process | 36 | 875 | 509 | 17653 | 1.42690541678361 | 0.0205876021240424 | 0.33478694133529 | 1.68639423351506 | 0.0707269155206287 | RNF123//UBE4B//RNF217//FBXO45//FBXO10//RLIM//UCHL5//RNF125//USP37//TP53INP2//USP46//UBE2D3//UBE2E1//USP45//FBXL3//SKP1//FBXL20//OGT//TRIB1//LRRK2//RNF114//VLDLR//DNAJB12//UBXN2A//MAEA//UBXN2B//RNF38//PML//CDKN1B//NDFIP1//IL1B//ALAD//WDR91//CD81//EDEM3//TRIM67// |
| GO:0070848 | response to growth factor | Biological process | 30 | 701 | 509 | 17653 | 1.4842394670538 | 0.0205978862224855 | 0.33478694133529 | 1.68617734511687 | 0.0589390962671906 | TAB1//FERMT2//FSHB//SMAD1//PML//SKIL//FGFR3//FGFR2//GALNT3//POLR2E//PTBP1//NREP//DLX5//BMP10//RGMA//UBE2D3//THBS1//HTRA4//RASL11B//ONECUT2//SFRP1//IL1B//ITGA5//MED1//SPRY3//RAF1//ASCL1//EDN1//HEYL//ELK1// |
| GO:0032722 | positive regulation of chemokine production | Biological process | 5 | 55 | 509 | 17653 | 3.15288444365065 | 0.020833130932777 | 0.33478694133529 | 1.68124545666543 | 0.00982318271119843 | HMOX1//IL1B//IL4R//TIRAP//CD74// |
| GO:0046513 | ceramide biosynthetic process | Biological process | 5 | 55 | 509 | 17653 | 3.15288444365065 | 0.020833130932777 | 0.33478694133529 | 1.68124545666543 | 0.00982318271119843 | ST6GALNAC6//GAL3ST1//PRKAA1//SMPD1//FAM57B// |
| GO:0046928 | regulation of neurotransmitter secretion | Biological process | 5 | 55 | 509 | 17653 | 3.15288444365065 | 0.020833130932777 | 0.33478694133529 | 1.68124545666543 | 0.00982318271119843 | UNC13B//CHRNA3//LRRK2//CPLX1//CPLX3// |
| GO:0060688 | regulation of morphogenesis of a branching structure | Biological process | 5 | 55 | 509 | 17653 | 3.15288444365065 | 0.020833130932777 | 0.33478694133529 | 1.68124545666543 | 0.00982318271119843 | FGFR2//SFRP1//PAX8//LRRK2//RTN4// |
| GO:0010720 | positive regulation of cell development | Biological process | 23 | 504 | 509 | 17653 | 1.58269794492781 | 0.0208406479819839 | 0.33478694133529 | 1.6810887819836 | 0.0451866404715128 | OTP//FZD3//TRIM67//ZNF804A//TRIB1//HEYL//ASCL1//SEMA7A//KIT//SHANK3//RTN4//SKIL//TIAM1//CUX1//EDN1//BMP10//ACTR2//DDX39B//DLG4//CRTC1//PACSIN1//VLDLR//SYT2// |
| GO:1903555 | regulation of tumor necrosis factor superfamily cytokine production | Biological process | 8 | 118 | 509 | 17653 | 2.35130365289201 | 0.0209512922589303 | 0.334906492262861 | 1.67878918493566 | 0.0157170923379175 | GPNMB//FOXP3//TIRAP//NFATC4//PTAFR//ABCC8//THBS1//LRRK2// |
| GO:0048872 | homeostasis of number of cells | Biological process | 13 | 238 | 509 | 17653 | 1.89438014891615 | 0.0210052743631478 | 0.334906492262861 | 1.67767164149457 | 0.0255402750491159 | JAM3//FOXP3//SKIL//CD74//KIT//KMT2E//HMOX1//PPP3CB//RAG1//TNFSF14//MAEA//MED1//BCR// |
| GO:1903312 | negative regulation of mRNA metabolic process | Biological process | 6 | 75 | 509 | 17653 | 2.77453831041257 | 0.0210517025757878 | 0.334906492262861 | 1.67671277444265 | 0.0117878192534381 | CELF4//PTBP1//SRSF9//LARP1//TIRAP//DAZL// |
| GO:0010033 | response to organic substance | Biological process | 111 | 3218 | 509 | 17653 | 1.19629332060207 | 0.0212230753412014 | 0.334906492262861 | 1.67319168419807 | 0.218074656188605 | AGPAT1//CD74//MED1//DNAJB5//THBS1//PARP16//TAB1//FERMT2//FSHB//SMAD1//PML//SKIL//CHRM1//ATP6V1G2//FGFR3//FGFR2//GALNT3//POLR2E//PTBP1//STEAP2//P2RX2//IL1B//CDKN1B//ALAD//SFRP1//NREP//HMOX1//LRRTM1//IL4R//KIT//PIM1//PTAFR//UBE4B//DLX5//BMP10//RGMA//UBE2D3//HTRA4//RASL11B//ONECUT2//CALCOCO1//NRIP1//ITGA5//CTH//EDEM3//CREB3L3//PRKAA1//TRIM5//TRIB1//ALDH1A2//CD96//TIRAP//EDN1//ABCC8//RELT//ASCL1//FOSL1//INSIG2//OGT//TNFSF4//TNFSF14//GNAO1//PGGT1B//PPP3CB//REL//TRIM21//PAK2//RAF1//VSNL1//ACTR2//KDELR3//ASNA1//TLN1//GOSR2//DNAJB12//SPRY3//SLC6A3//SMPD1//TIAM1//SRSF9//GRIN2B//USP46//PTPRU//NCAM1//TRIM68//HEYL//NFKB1//SKP1//MRC1//BCR//AQP1//WNT11//SATB2//MAPK4//UNC13B//PTPN18//ACTR3//DAPK3//WAS//PAX8//ELK1//CD81//CASP7//LARP1//CHRNA3//LRRK2//COPS5//NFATC4//UBXN2A//SLC25A5//MYBL2// |
| GO:0042092 | type 2 immune response | Biological process | 4 | 37 | 509 | 17653 | 3.74937609515213 | 0.0212742787502825 | 0.334906492262861 | 1.67214515463148 | 0.00785854616895874 | NDFIP1//TNFSF4//CD74//IL4R// |
| GO:0045581 | negative regulation of T cell differentiation | Biological process | 4 | 37 | 509 | 17653 | 3.74937609515213 | 0.0212742787502825 | 0.334906492262861 | 1.67214515463148 | 0.00785854616895874 | TNFSF4//IL4R//FOXP3//CD74// |
| GO:0061001 | regulation of dendritic spine morphogenesis | Biological process | 4 | 37 | 509 | 17653 | 3.74937609515213 | 0.0212742787502825 | 0.334906492262861 | 1.67214515463148 | 0.00785854616895874 | ACTR2//TIAM1//LRRK2//SHANK3// |
| GO:0042493 | response to drug | Biological process | 39 | 966 | 509 | 17653 | 1.40019402311194 | 0.0214158290137511 | 0.334906492262861 | 1.66926510926046 | 0.0766208251473477 | EBP//RALBP1//CHRM1//EDN1//ALAD//CHRNA3//AQP1//SLC25A5//SLC6A3//SLC5A6//PRKAA1//SLC52A2//THBS1//FOSL1//P2RX2//IL1B//HMOX1//RNF149//PPP3CB//SMPD1//TIAM1//GNAO1//CYP2C18//CYP2S1//ACTR2//FGFR2//GRIN2B//USP46//ASCL1//NFKB1//SFRP1//PTAFR//NUCB1//LARP1//LRRK2//SLC25A2//CDKN1B//ABCC8//FZD3// |
| GO:0050807 | regulation of synapse organization | Biological process | 10 | 165 | 509 | 17653 | 2.10192296243377 | 0.0215015999457038 | 0.334906492262861 | 1.66752922279426 | 0.0196463654223969 | SHANK3//LRRTM1//LRRN1//LRRK2//ACTR2//TIAM1//GRIN2B//ZNF804A//GRIPAP1//NFATC4// |
| GO:0014821 | phasic smooth muscle contraction | Biological process | 3 | 21 | 509 | 17653 | 4.95453269716531 | 0.0215413757406986 | 0.334906492262861 | 1.66672656392037 | 0.00589390962671906 | EDN1//P2RX2//KIT// |
| GO:0019373 | epoxygenase P450 pathway | Biological process | 3 | 21 | 509 | 17653 | 4.95453269716531 | 0.0215413757406986 | 0.334906492262861 | 1.66672656392037 | 0.00589390962671906 | CYP2C18//CYP2S1//CYP4F2// |
| GO:0048485 | sympathetic nervous system development | Biological process | 3 | 21 | 509 | 17653 | 4.95453269716531 | 0.0215413757406986 | 0.334906492262861 | 1.66672656392037 | 0.00589390962671906 | ASCL1//FZD3//PHOX2A// |
| GO:0097503 | sialylation | Biological process | 3 | 21 | 509 | 17653 | 4.95453269716531 | 0.0215413757406986 | 0.334906492262861 | 1.66672656392037 | 0.00589390962671906 | ST8SIA5//ST6GALNAC6//ST3GAL3// |
| GO:1902932 | positive regulation of alcohol biosynthetic process | Biological process | 3 | 21 | 509 | 17653 | 4.95453269716531 | 0.0215413757406986 | 0.334906492262861 | 1.66672656392037 | 0.00589390962671906 | PRKAA1//IL1B//PTAFR// |
| GO:0010941 | regulation of cell death | Biological process | 63 | 1696 | 509 | 17653 | 1.28829535344923 | 0.0217380405654067 | 0.337188918219829 | 1.66277960514315 | 0.1237721021611 | CD74//PML//CCK//CDKN1B//MAEA//COPS5//FGFR3//FGFR2//AQP1//KIT//ASCL1//NFKB1//PAFAH2//PAK2//PIM1//MED1//PRKAA1//LIMS2//RAF1//SFRP1//THBS1//WNT11//CASP7//HMOX1//PAX8//SGK2//DAPK3//FBXO10//BMP10//NF2//RTN4//TNFAIP8L2//RELT//OXSR1//EDN1//CDKN2A//UNC13B//MAP3K9//ARHGEF4//UBE2Z//SMPD1//TGM2//TIAM1//FOSL1//ALDH1A2//ARHGEF6//LRRK2//RAG1//TNFSF14//MYBL2//PARP16//FZD3//YWHAB//SLC25A5//GPNMB//REL//ELK1//GRIN2B//SKIL//ITGA5//CTH//NFATC4//IL1B// |
| GO:0060070 | canonical Wnt signaling pathway | Biological process | 16 | 317 | 509 | 17653 | 1.75049735672718 | 0.0220297348522813 | 0.340762043265206 | 1.65699072994338 | 0.031434184675835 | LRRK2//CTNND2//KREMEN1//RBMS3//SFRP1//WNT11//DAPK3//DLX5//FGFR2//NFKB1//TNKS2//DIXDC1//FZD2//PTPRU//NDP//FZD3// |
| GO:0007229 | integrin-mediated signaling pathway | Biological process | 7 | 97 | 509 | 17653 | 2.50280517691856 | 0.0220691667825167 | 0.340762043265206 | 1.65621406324007 | 0.0137524557956778 | EMP2//LIMS2//FERMT2//ITGA5//TLN1//FERMT3//SEMA7A// |
| GO:0035967 | cellular response to topologically incorrect protein | Biological process | 10 | 166 | 509 | 17653 | 2.08926077591308 | 0.022314642467425 | 0.341968599972796 | 1.6514100671774 | 0.0196463654223969 | PARP16//CTH//EDEM3//CREB3L3//KDELR3//ASNA1//TLN1//GOSR2//DNAJB12//COPS5// |
| GO:0043270 | positive regulation of ion transport | Biological process | 13 | 240 | 509 | 17653 | 1.87859364767518 | 0.0223175940512306 | 0.341968599972796 | 1.65135262633815 | 0.0255402750491159 | P2RX2//SCN3B//PRSS8//CCK//CYP4F2//EDN1//IL1B//EHD3//PTAFR//ABCC8//GLRX//SHANK3//CHRM1// |
| GO:0048332 | mesoderm morphogenesis | Biological process | 6 | 76 | 509 | 17653 | 2.73803122738083 | 0.0223196052290863 | 0.341968599972796 | 1.65131349111195 | 0.0117878192534381 | TLX2//NF2//SMAD1//FGFR2//EXOC4//WNT11// |
| GO:0030111 | regulation of Wnt signaling pathway | Biological process | 17 | 344 | 509 | 17653 | 1.7139226481473 | 0.0223495669359716 | 0.341968599972796 | 1.65073088770609 | 0.0333988212180747 | FGFR2//SFRP1//HIC1//TRABD2B//NFATC4//LRRK2//CTNND2//KREMEN1//RBMS3//WNT11//DAPK3//DLX5//NFKB1//TNKS2//DIXDC1//TIAM1//FOXP3// |
| GO:0045595 | regulation of cell differentiation | Biological process | 65 | 1761 | 509 | 17653 | 1.28013195752994 | 0.0224362354784839 | 0.342519775487554 | 1.64905001052072 | 0.12770137524558 | OTP//FZD3//NDFIP1//FGFR2//TIAM1//IL1B//SFRP1//NFKB1//ZNF804A//TRIM67//LRRK2//NF2//TTL//RTN4//ASCL1//CNTN2//FOXP3//TNFSF4//HMGB3//CD74//ZMIZ1//RAG1//SKIL//TAF8//FAM57B//MED1//IL4R//TRIB1//KMT2E//THBS1//MBNL3//CXCL14//TNFSF14//EFNA3//NREP//DIXDC1//HEYL//SMAD1//FSHB//SEMA7A//KIT//SHANK3//RGMA//CHRNA3//SEMA4G//TLX2//NFATC4//CUX1//PTBP1//ABCC8//EDN1//BMP10//ACTR2//DDX39B//PAX8//DLG4//CRTC1//PACSIN1//VLDLR//ZSWIM6//CDKN1B//SYT2//CTH//EMP2//RAF1// |
| GO:0048519 | negative regulation of biological process | Biological process | 174 | 5303 | 509 | 17653 | 1.13796357253391 | 0.0225753851500075 | 0.343379525783837 | 1.64636483161928 | 0.341846758349705 | CUX1//EDN1//FGFR2//FOXS1//SATB2//ZNF281//DNAJB5//HEYL//KCNIP3//HIC1//ASCL1//NFKB1//PAX5//FOXP3//RLIM//MED1//CC2D1A//REL//SKIL//UBE2D3//VLDLR//NRIP1//BHLHE40//PAXBP1//RBM8A//GPNMB//PPP3CB//LRRK2//VASH1//THBS1//CDKN2A//NF2//DLG4//LRRTM1//HMOX1//BCR//CD96//CD74//NDFIP1//TRIB1//PAK2//CEP85//CDKN1B//PML//RASSF1//EIF4G2//PRKAA1//PRKAB1//KMT2E//TTK//KLHL22//ARHGDIB//GRM2//PALM//MAEA//COPS5//FGFR3//AQP1//KIT//PAFAH2//PIM1//LIMS2//RAF1//SFRP1//WNT11//PTPRU//DDAH1//LDOC1//IL1B//SMAD1//DEC1//FOSL1//ALDH1A2//RGS9BP//BMP10//RBMS3//PAX8//SIRT3//DPP4//CTNS//RSC1A1//ABHD5//INSIG2//PPP1R26//UCHL5//SKP1//ABCC8//PIK3IP1//MGRN1//YWHAB//EFNA3//PASD1//FOXG1//PURA//CREBZF//TNFSF4//DAPK3//LARP1//CELF4//MEX3B//RFX4//SMPD1//TRABD2B//NFATC4//KREMEN1//RTN4//HTRA4//RASL11B//ONECUT2//EXD1//MAPRE1//OGT//TRIM21//CCK//SHANK3//CYP4F2//RNF125//RNF135//PPP1R2//PTBP1//TIRAP//SPRY3//RAG1//TNFSF14//RNF149//CARHSP1//NUP214//HMGB3//FAM57B//IL4R//MBNL3//CXCL14//CNTN2//DIXDC1//PACSIN1//FZD3//TRIM67//MAPKAP1//TRIM5//VSNL1//SRSF9//RGMA//SEMA4G//SEMA7A//TNFAIP8L2//TLX2//CGNL1//WAS//SPTBN2//LMOD1//PGGT1B//GNAO1//SLC6A3//PARP16//POLR2E//SH3BP5//DAZL//OXSR1//SLC25A5//PEX5//PTPN18//ALAD//TPRA1//CRTC1//GRIN2B//HOXC6//TP53INP2//TNKS2//BEX4//SESTD1//DDX39B//LYPD6//ITGA5//CTH// |
| GO:0071900 | regulation of protein serine/threonine kinase activity | Biological process | 23 | 508 | 509 | 17653 | 1.57023575638507 | 0.0225940986209977 | 0.343379525783837 | 1.64600497992407 | 0.0451866404715128 | CDKN1B//TAB1//LRRK2//RAF1//IL1B//KIT//PRKAA1//THBS1//PROK1//CD74//CD81//TIRAP//MAP3K9//DBNL//CDKN2A//TRIB1//EDN1//S100A12//SPRY3//SMPD1//TIAM1//SFRP1//PARP16// |
| GO:0048660 | regulation of smooth muscle cell proliferation | Biological process | 9 | 143 | 509 | 17653 | 2.18276615329661 | 0.022885061647442 | 0.345909489061571 | 1.64044791333903 | 0.0176817288801572 | EDN1//FGFR2//HMOX1//PTAFR//TGM2//THBS1//TRIB1//CDKN1B//DDX39B// |
| GO:0048489 | synaptic vesicle transport | Biological process | 8 | 120 | 509 | 17653 | 2.31211525867714 | 0.0229140102172976 | 0.345909489061571 | 1.63989889759221 | 0.0157170923379175 | UNC13B//CPLX1//SYT2//SYT9//SYT14//CPLX3//SNPH//LRRK2// |
| GO:0097480 | establishment of synaptic vesicle localization | Biological process | 8 | 120 | 509 | 17653 | 2.31211525867714 | 0.0229140102172976 | 0.345909489061571 | 1.63989889759221 | 0.0157170923379175 | UNC13B//CPLX1//SYT2//SYT9//SYT14//CPLX3//SNPH//LRRK2// |
| GO:0009887 | animal organ morphogenesis | Biological process | 40 | 1001 | 509 | 17653 | 1.38588327193435 | 0.0231054299939478 | 0.347123347245392 | 1.63628594481413 | 0.0785854616895874 | PAX8//CCDC103//MICAL2//DLX5//FGFR3//TAB1//ZMIZ1//ALDH1A2//FGFR2//PARVA//FZD2//HEYL//WNT11//UBE4B//MED1//LRRK2//WDR19//INSIG2//BCR//FZD3//KCNQ4//EDN1//NF2//AQP1//EXOC4//PAX5//SATB2//HOXC9//UCHL5//SHANK3//MYOM3//BMP10//TIAM1//RTN4//SFRP1//PML//TGM2//PIM1//LIMS2//ONECUT2// |
| GO:0043123 | positive regulation of I-kappaB kinase/NF-kappaB signaling | Biological process | 10 | 167 | 509 | 17653 | 2.07675023234474 | 0.0231497619922819 | 0.347123347245392 | 1.63545346969873 | 0.0196463654223969 | TIRAP//CTH//HMOX1//CC2D1A//REL//S100A12//TGM2//NDFIP1//TRIM5//CD74// |
| GO:0016601 | Rac protein signal transduction | Biological process | 4 | 38 | 509 | 17653 | 3.65070830317444 | 0.0232510537190836 | 0.347123347245392 | 1.63355736045086 | 0.00785854616895874 | OGT//DBNL//BRK1//TIAM1// |
| GO:0061614 | pri-miRNA transcription by RNA polymerase II | Biological process | 4 | 38 | 509 | 17653 | 3.65070830317444 | 0.0232510537190836 | 0.347123347245392 | 1.63355736045086 | 0.00785854616895874 | NFATC4//SMAD1//TEAD1//FOSL1// |
| GO:1902622 | regulation of neutrophil migration | Biological process | 4 | 38 | 509 | 17653 | 3.65070830317444 | 0.0232510537190836 | 0.347123347245392 | 1.63355736045086 | 0.00785854616895874 | JAM3//TIRAP//EDN1//CD74// |
| GO:0051247 | positive regulation of protein metabolic process | Biological process | 61 | 1641 | 509 | 17653 | 1.28920503454576 | 0.0234621669665647 | 0.349503601750831 | 1.62963187891443 | 0.119842829076621 | TAB1//LRRK2//RAF1//IL1B//KIT//PRKAA1//THBS1//PROK1//CD74//CD81//GPNMB//CAMKK2//PML//CCK//PARP16//CHRNA3//TIRAP//MAP3K9//DBNL//CASP7//BMP10//TTK//CNTN2//LARP1//RBMS3//PTAFR//RBM3//DDX39B//PAXBP1//NDFIP1//EMP2//PAK2//WNT11//OXSR1//MAPKAP1//TRIB1//RNF217//RNF125//RNF114//FOXP3//SMPD1//FGFR3//EDN1//S100A12//FGFR2//TRIM5//TIAM1//HMOX1//CDKN1B//VLDLR//OGT//DAZL//ITGA5//PRDM12//DLG4//SEMA7A//EFNA3//TRABD2B//KMT2E//TRIM67//GRIN2B// |
| GO:2000177 | regulation of neural precursor cell proliferation | Biological process | 6 | 77 | 509 | 17653 | 2.70247238027199 | 0.0236379581283087 | 0.349724432894394 | 1.62639004098483 | 0.0117878192534381 | OTP//FZD3//LRRK2//LIMS2//ASCL1//NF2// |
| GO:0006813 | potassium ion transport | Biological process | 13 | 242 | 509 | 17653 | 1.86306808033902 | 0.0236900274079394 | 0.349724432894394 | 1.6254344368243 | 0.0255402750491159 | SLC12A7//ATP1B4//KCNJ12//KCNIP2//KCNIP3//AQP1//KCNA6//KCNQ5//SLC9A5//ABCC8//KCNQ4//OXSR1//CDKN1B// |
| GO:0051260 | protein homooligomerization | Biological process | 16 | 320 | 509 | 17653 | 1.73408644400786 | 0.0237910839720449 | 0.349724432894394 | 1.62358577013789 | 0.031434184675835 | CTH//DPYS//CRTC1//KCNJ12//CD79A//BRK1//PEX5//KCTD18//ALAD//P2RX2//EHD3//HMOX1//KCNA6//CCDC88C//TGM2//SHKBP1// |
| GO:0010942 | positive regulation of cell death | Biological process | 29 | 681 | 509 | 17653 | 1.47690181721668 | 0.0239083182117262 | 0.349724432894394 | 1.62145097248546 | 0.0569744597249509 | PML//CCK//CASP7//CDKN2A//UNC13B//DAPK3//HMOX1//MAP3K9//ARHGEF4//SFRP1//UBE2Z//SMPD1//TGM2//TIAM1//WNT11//FOSL1//ALDH1A2//ARHGEF6//LRRK2//ASCL1//MYBL2//YWHAB//ELK1//GRIN2B//SKIL//THBS1//NFATC4//PAK2//CDKN1B// |
| GO:0000380 | alternative mRNA splicing, via spliceosome | Biological process | 5 | 57 | 509 | 17653 | 3.04225691931203 | 0.023939529319557 | 0.349724432894394 | 1.62088439260666 | 0.00982318271119843 | RBFOX1//MBNL3//CELF4//PTBP1//RBM8A// |
| GO:0021885 | forebrain cell migration | Biological process | 5 | 57 | 509 | 17653 | 3.04225691931203 | 0.023939529319557 | 0.349724432894394 | 1.62088439260666 | 0.00982318271119843 | FBXO45//DIXDC1//RTN4//CNTN2//LRRK2// |
| GO:0042108 | positive regulation of cytokine biosynthetic process | Biological process | 5 | 57 | 509 | 17653 | 3.04225691931203 | 0.023939529319557 | 0.349724432894394 | 1.62088439260666 | 0.00982318271119843 | THBS1//HMOX1//IL1B//TIRAP//PTAFR// |
| GO:1903902 | positive regulation of viral life cycle | Biological process | 5 | 57 | 509 | 17653 | 3.04225691931203 | 0.023939529319557 | 0.349724432894394 | 1.62088439260666 | 0.00982318271119843 | PPIH//LARP1//TRIM21//TMPRSS2//CD74// |
| GO:0003231 | cardiac ventricle development | Biological process | 8 | 121 | 509 | 17653 | 2.29300686810956 | 0.0239423942673524 | 0.349724432894394 | 1.6208324218032 | 0.0157170923379175 | FGFR2//FZD2//HEYL//UBE4B//MED1//PAX8//BMP10//WNT11// |
| GO:0048584 | positive regulation of response to stimulus | Biological process | 83 | 2334 | 509 | 17653 | 1.23332626266197 | 0.024058865067644 | 0.350651613299275 | 1.61872486355 | 0.163064833005894 | TAB1//LRRK2//RAF1//IL1B//KIT//PRKAA1//THBS1//PROK1//CD74//CD81//AGPAT1//TRIM5//CD209//NFKB1//PAK2//SKP1//CREB3L3//TNFSF4//TIRAP//UBE2D3//FOXP3//C1QA//VSIG4//MAP3K9//DBNL//TNFSF14//BMP10//TTK//WAS//FGFR2//SFRP1//ITGA5//MED1//WDFY1//PPP3CB//VSNL1//ACTR3//ACTR2//BRK1//RNF125//FGFR3//CTH//HMOX1//CC2D1A//REL//S100A12//TGM2//NDFIP1//IL4R//PTAFR//EDN1//TIAM1//HIC1//SHKBP1//ENHO//ASCL1//ZMIZ1//TSPAN14//EVC//RTN4//P2RX2//CD79A//PAX5//CNTN2//SEMA7A//GPNMB//WNT11//DAPK3//DLX5//TNKS2//DIXDC1//UNC13B//SHANK3//YWHAB//SKIL//FAM168A//DLG4//CXCL14//CDKN2A//EMP2//LIMS2//NFATC4//PML// |
| GO:0032446 | protein modification by small protein conjugation | Biological process | 37 | 915 | 509 | 17653 | 1.4024305667386 | 0.0241135702342819 | 0.350651613299275 | 1.61773848359779 | 0.0726915520628684 | UBE4B//RNF217//MGRN1//FBXL3//FBXO10//RLIM//RNF125//RNF114//SKP1//TRIM21//UBE2D3//UBE2E1//TNKS2//FBXL20//MAEA//RNF38//FBXO45//RNF149//MED1//DCAF16//TULP4//KLHL14//PEX5//RNF123//UBE2Z//HLTF//TRIM7//RNF135//KLHL22//RAG1//NUP214//UBXN2A//OGT//LRRK2//NDFIP1//TRIM68//TRIM5// |
| GO:0016310 | phosphorylation | Biological process | 84 | 2367 | 509 | 17653 | 1.23078378788897 | 0.0243332491565081 | 0.350651613299275 | 1.61379989696608 | 0.165029469548134 | CDKN1B//CAMKK2//LRRK2//DLG4//FGFR3//FGFR2//GRIN2B//IL1B//KIT//SMAD1//NCAM1//MAPK4//RAF1//SPTBN2//YWHAB//SHANK3//TAB1//PRKAA1//THBS1//PROK1//CD74//CD81//CCDC88C//PML//GPNMB//NUP214//OGT//SGK2//GLYCTK//NEK7//DAPK3//MARK1//MAP3K9//PAK2//PIM1//PPP3CB//PRKAB1//BCR//WNT11//MEX3B//AATK//OXSR1//TRIB1//LRRTM1//NF2//CEP85//PARP16//CHRNA3//TIRAP//ARHGEF6//DBNL//BMP10//TTK//CUX1//CDKN2A//EMP2//MAPKAP1//EDN1//S100A12//SPRY3//SMPD1//RNF149//TRIM5//TIAM1//SFRP1//NRBF2//WDR91//PIK3IP1//VLDLR//COPS5//DGKG//IMPA2//PIP4K2C//ITGA5//CCK//NFKB1//SKP1//SH3BP5//ALDOC//MED1//SIRT3//SEMA7A//UCK2//UCK1// |
| GO:0031399 | regulation of protein modification process | Biological process | 66 | 1800 | 509 | 17653 | 1.27166339227243 | 0.0243810941289691 | 0.350651613299275 | 1.61294680882791 | 0.129666011787819 | CDKN1B//TAB1//LRRK2//RAF1//IL1B//KIT//PRKAA1//THBS1//PROK1//CD74//CD81//CCDC88C//PML//GPNMB//CAMKK2//TRIB1//LRRTM1//NF2//PAK2//CEP85//PARP16//CHRNA3//TIRAP//MAP3K9//DBNL//BMP10//TTK//PARG//PAXBP1//FOXP3//UBXN2A//OGT//NDFIP1//CDKN2A//EMP2//WNT11//OXSR1//MAPKAP1//PPP1R2//PPP1R26//SMPD1//YWHAB//FGFR3//EDN1//S100A12//SPRY3//RNF149//FGFR2//TRIM5//TIAM1//SFRP1//UBXN2B//VLDLR//COPS5//ITGA5//CCK//PAX5//PRDM12//DLG4//SH3BP5//SIRT3//SEMA7A//TRIM21//BEX4//TRABD2B//KMT2E// |
| GO:0003416 | endochondral bone growth | Biological process | 3 | 22 | 509 | 17653 | 4.72932666547598 | 0.0244206579718998 | 0.350651613299275 | 1.61224263892858 | 0.00589390962671906 | EVC//FGFR3//FGFR2// |
| GO:0010226 | response to lithium ion | Biological process | 3 | 22 | 509 | 17653 | 4.72932666547598 | 0.0244206579718998 | 0.350651613299275 | 1.61224263892858 | 0.00589390962671906 | CDKN1B//NFATC4//ASCL1// |
| GO:0035590 | purinergic nucleotide receptor signaling pathway | Biological process | 3 | 22 | 509 | 17653 | 4.72932666547598 | 0.0244206579718998 | 0.350651613299275 | 1.61224263892858 | 0.00589390962671906 | GPR34//PTAFR//P2RX2// |
| GO:0071379 | cellular response to prostaglandin stimulus | Biological process | 3 | 22 | 509 | 17653 | 4.72932666547598 | 0.0244206579718998 | 0.350651613299275 | 1.61224263892858 | 0.00589390962671906 | PRKAA1//SFRP1//TNFSF4// |
| GO:0060560 | developmental growth involved in morphogenesis | Biological process | 12 | 218 | 509 | 17653 | 1.90908599340315 | 0.0246241959060254 | 0.352040384539 | 1.60863794232386 | 0.0235756385068762 | WNT11//TTL//RTN4//SEMA7A//SEMA5B//PPP3CB//SEMA4G//FGFR2//MED1//SFRP1//SYT2//TIAM1// |
| GO:0007269 | neurotransmitter secretion | Biological process | 9 | 145 | 509 | 17653 | 2.15265903394079 | 0.0247682828562487 | 0.352040384539 | 1.60610410128279 | 0.0176817288801572 | UNC13B//CHRNA3//CPLX1//SYT2//SYT9//SYT14//CPLX3//SNPH//LRRK2// |
| GO:0099643 | signal release from synapse | Biological process | 9 | 145 | 509 | 17653 | 2.15265903394079 | 0.0247682828562487 | 0.352040384539 | 1.60610410128279 | 0.0176817288801572 | UNC13B//CPLX1//SNPH//CHRNA3//SYT2//SYT9//SYT14//CPLX3//LRRK2// |
| GO:0048870 | cell motility | Biological process | 60 | 1615 | 509 | 17653 | 1.28848528347333 | 0.0247776464646701 | 0.352040384539 | 1.60593994799826 | 0.117878192534381 | SEMA4G//SEMA7A//FBXO45//SATB2//MARK1//ASCL1//FZD3//CCK//JAM3//S100A12//KIT//HMOX1//BCR//EMP2//EDN1//THBS1//VASH1//BMP10//RTN4//PML//TNFSF14//TRIB1//AVL9//PAK2//RHOF//TIAM1//DIXDC1//CNTN2//LRRK2//KIF2A//BEX4//PARD6B//GPNMB//DAPK3//MAPRE1//FSHB//IL1B//ITGA5//WNT11//FERMT3//ONECUT2//PTPRU//NF2//SFRP1//DPP4//CD74//KRT2//BIN2//CXCL14//ABCC8//ACTR3//PARVA//TIRAP//ARHGDIB//TAC4//PTAFR//RAF1//CD81//WAS//BRK1// |
| GO:0051674 | localization of cell | Biological process | 60 | 1615 | 509 | 17653 | 1.28848528347333 | 0.0247776464646701 | 0.352040384539 | 1.60593994799826 | 0.117878192534381 | SEMA4G//SEMA7A//FBXO45//SATB2//MARK1//ASCL1//FZD3//CCK//JAM3//S100A12//KIT//HMOX1//BCR//EMP2//EDN1//THBS1//VASH1//BMP10//RTN4//PML//TNFSF14//TRIB1//AVL9//PAK2//RHOF//TIAM1//DIXDC1//CNTN2//LRRK2//KIF2A//BEX4//PARD6B//GPNMB//DAPK3//MAPRE1//FSHB//IL1B//ITGA5//WNT11//FERMT3//ONECUT2//PTPRU//NF2//SFRP1//DPP4//BRK1//CD74//KRT2//BIN2//CXCL14//ABCC8//ACTR3//PARVA//TIRAP//ARHGDIB//TAC4//PTAFR//RAF1//CD81//WAS// |
| GO:0051716 | cellular response to stimulus | Biological process | 240 | 7556 | 509 | 17653 | 1.101590117951 | 0.024909705500257 | 0.353174713413497 | 1.60363140696246 | 0.471512770137525 | CAMKK2//LRRK2//DLG4//FGFR3//FGFR2//GRIN2B//IL1B//KIT//SMAD1//NCAM1//MAPK4//RAF1//SPTBN2//YWHAB//SHANK3//TAB1//PRKAA1//THBS1//PROK1//CD74//CD81//COPS5//CCK//AGPAT1//MED1//TRIM5//CD209//NFKB1//PAK2//SKP1//S100A12//TIRAP//UBE2D3//P2RX2//WNT11//FERMT2//CTNND2//HIC1//TRABD2B//MARK1//CCDC88C//NDP//CALCOCO1//TNKS2//KREMEN1//VPS26A//FZD2//GNAO1//PPP3CB//FZD3//FAM13A//GPNMB//EBP//RALBP1//PRDM4//PTPRT//AKAP10//CHRM1//CHRNA3//DGKG//FGF11//FSHB//HTR3E//KCNIP3//KCNIP2//ARHGAP40//IL4R//IMPA2//LGALS3BP//MRC1//NRGN//CC2D1A//PRKAB1//HIVEP3//BCR//SH3GL1//RASL11B//SMPD1//ABCC8//TNFSF4//VLDLR//RERGL//NDFIP1//OGT//TNFSF14//CHRFAM7A//SH3BP5//CXCL14//SGK2//UNC13B//DAPK3//CARHSP1//HMOX1//ARHGEF4//POLR2M//PLA2G4C//OXSR1//UCHL5//USP45//PARG//POLR2E//CYP2C18//CYP2S1//FBXO45//CDKN1B//PML//PARP16//EDN1//CLEC1A//TSPAN14//TSPAN18//SKIL//PTPRU//TPRA1//GPR160//GNAZ//GPR34//GRM2//PTAFR//SFRP1//TIAM1//ARHGEF6//GPR3//PALM//TAC4//HEYL//ASCL1//EVC//ITGA5//TLN1//FERMT3//SEMA7A//REL//TRIB1//MAP3K9//DBNL//DDAH1//ARFIP2//RHOF//RALGPS2//CDKN2A//RASSF1//RALGDS//CDC42EP1//EPS8L2//ATP6V1G2//GALNT3//PTBP1//UBE4B//NFATC4//SPRY3//PPP1R2//RGS9BP//BMP10//TTK//PIP4K2C//PIK3IP1//MGRN1//BRK1//NREP//LRRTM1//PIM1//RFX4//FOXP3//WAS//AQP1//DLX5//RGMA//HTRA4//ONECUT2//NRIP1//CTH//EDEM3//CREB3L3//MAPKAP1//JAM3//FOSL1//LARP1//IQSEC3//RABL3//INSIG2//RELT//WDFY1//GPX8//NRBF2//ARHGDIB//TEAD1//NF2//RNF149//VSNL1//ACTR2//KDELR3//ASNA1//GOSR2//DNAJB12//UNC5A//ACTR3//PTPN18//PAX8//RNF125//TGM2//SHKBP1//ENHO//ZMIZ1//TRIM67//EFNA3//ALDH1A2//RTN4//CD79A//PAX5//RALGAPB//CGNL1//DIXDC1//CNTN2//BIN2//TRIM68//TRIM21//WDR19//SIRT3//ALAD//SATB2//ELK1//SEMA4G//PARVA//CASP7//RBMS3//CELF4//BSN//LYPD6//NUP214//SLC25A5//LDOC1//FAM168A//UBXN2A//MYBL2//DDX39B//SHISA9//VASH1//EMP2//LIMS2// |
| GO:0048732 | gland development | Biological process | 20 | 430 | 509 | 17653 | 1.61310366884452 | 0.0250264751744507 | 0.35408797406864 | 1.60160031379903 | 0.0392927308447937 | MED1//DDX39B//ALDH1A2//ONECUT2//CSN3//SLC6A3//OTP//FGFR2//WNT11//RAF1//PAX8//RTN4//SFRP1//RAG1//PML//TGM2//CDKN1B//ASCL1//HMOX1//LIMS2// |
| GO:0046578 | regulation of Ras protein signal transduction | Biological process | 13 | 244 | 509 | 17653 | 1.84779703050018 | 0.0251240706891182 | 0.354726701608573 | 1.59990999333472 | 0.0255402750491159 | IQSEC3//RALGPS2//OGT//ARHGDIB//ARHGEF4//RAF1//BCR//EPS8L2//TIAM1//ARHGEF6//SPRY3//TRIM67//MAPKAP1// |
| GO:0043029 | T cell homeostasis | Biological process | 4 | 39 | 509 | 17653 | 3.55710039796484 | 0.0253357373072838 | 0.356955973032402 | 1.59626645258012 | 0.00785854616895874 | FOXP3//PPP3CB//RAG1//TNFSF14// |
| GO:0030335 | positive regulation of cell migration | Biological process | 22 | 486 | 509 | 17653 | 1.5699548052746 | 0.0253980314621654 | 0.356955973032402 | 1.59519994315367 | 0.0432220039292731 | EDN1//THBS1//RTN4//TNFSF14//TIRAP//CD74//HMOX1//PTAFR//TIAM1//CXCL14//GPNMB//DAPK3//MAPRE1//FSHB//IL1B//ITGA5//KIT//SEMA4G//WNT11//FERMT3//SEMA7A//ONECUT2// |
| GO:0003279 | cardiac septum development | Biological process | 7 | 100 | 509 | 17653 | 2.427721021611 | 0.0255775999901195 | 0.356955973032402 | 1.59214020887798 | 0.0137524557956778 | FGFR2//PARVA//FZD2//PAX8//HEYL//WNT11//TAB1// |
| GO:0030838 | positive regulation of actin filament polymerization | Biological process | 7 | 100 | 509 | 17653 | 2.427721021611 | 0.0255775999901195 | 0.356955973032402 | 1.59214020887798 | 0.0137524557956778 | ACTR3//ACTR2//ARFIP2//LMOD1//WAS//BRK1//CDC42EP1// |
| GO:0050868 | negative regulation of T cell activation | Biological process | 7 | 100 | 509 | 17653 | 2.427721021611 | 0.0255775999901195 | 0.356955973032402 | 1.59214020887798 | 0.0137524557956778 | GPNMB//FOXP3//NDFIP1//CD74//TNFSF4//IL4R//TNFAIP8L2// |
| GO:0055025 | positive regulation of cardiac muscle tissue development | Biological process | 5 | 58 | 509 | 17653 | 2.98980421380665 | 0.0255986465948122 | 0.356955973032402 | 1.5917829953109 | 0.00982318271119843 | FGFR2//BMP10//PIM1//EDN1//DDX39B// |
| GO:0048638 | regulation of developmental growth | Biological process | 16 | 323 | 509 | 17653 | 1.71798037796444 | 0.0256566008548209 | 0.357027966216366 | 1.5908008821692 | 0.031434184675835 | TTL//RTN4//FGFR2//TNKS2//CACNA2D2//FOXS1//SLC6A3//SEMA7A//FGFR3//SEMA4G//BMP10//PIM1//EDN1//DDX39B//SYT2//SFRP1// |
| GO:0048659 | smooth muscle cell proliferation | Biological process | 9 | 146 | 509 | 17653 | 2.13791479398229 | 0.0257500598920125 | 0.357592720841233 | 1.58922175649695 | 0.0176817288801572 | EDN1//FGFR2//HMOX1//PTAFR//TGM2//THBS1//TRIB1//CDKN1B//DDX39B// |
| GO:0001818 | negative regulation of cytokine production | Biological process | 13 | 245 | 509 | 17653 | 1.84025500180426 | 0.0258646578767478 | 0.358448117255011 | 1.58729326182181 | 0.0255402750491159 | FOXP3//CD96//RNF125//RNF135//REL//TNFSF4//THBS1//NDFIP1//GPNMB//HMOX1//NFKB1//PML//WNT11// |
| GO:0051272 | positive regulation of cellular component movement | Biological process | 23 | 515 | 509 | 17653 | 1.54889274610411 | 0.0259353939835779 | 0.358693393682898 | 1.58610715026942 | 0.0451866404715128 | EDN1//THBS1//RTN4//TNFSF14//GPNMB//DAPK3//MAPRE1//FSHB//IL1B//ITGA5//KIT//SEMA4G//TIAM1//WNT11//FERMT3//SEMA7A//ONECUT2//TIRAP//CD74//HMOX1//TAC4//PTAFR//CXCL14// |
| GO:0006820 | anion transport | Biological process | 26 | 600 | 509 | 17653 | 1.50287491814014 | 0.026028616077774 | 0.35924802149793 | 1.58454892239265 | 0.0510805500982318 | SLC27A4//SLC26A1//ANO4//BEST1//THBS1//UNC13B//CPLX1//GRM2//CCK//ASNA1//AQP1//CA7//CTNS//SLC25A5//SLC5A6//SLC23A2//PCTP//SLC52A2//CYP4F2//EDN1//IL1B//SLC35A2//ABCC8//SLC12A7//PTAFR//SLC25A2// |
| GO:0071706 | tumor necrosis factor superfamily cytokine production | Biological process | 8 | 123 | 509 | 17653 | 2.25572220358746 | 0.0260954668627694 | 0.359307273106364 | 1.58343492896331 | 0.0157170923379175 | GPNMB//FOXP3//TIRAP//NFATC4//PTAFR//ABCC8//THBS1//LRRK2// |
| GO:0098655 | cation transmembrane transport | Biological process | 34 | 832 | 509 | 17653 | 1.41728218981412 | 0.0261391658093052 | 0.359307273106364 | 1.58270827636513 | 0.0667976424361493 | SLC12A7//ATP1B4//KCNJ12//SCN3B//SCN1A//SCN7A//SLC39A8//CACNG1//CACNA2D2//KCNIP2//SLC39A2//SLC30A6//KCNIP3//AQP1//KCNA6//KCNQ5//SLC9A5//ABCC8//KCNQ4//GRIN2B//STEAP2//OXSR1//EHD3//SLMAP//ATP6V1G2//SLC25A2//SESTD1//DLG4//SHISA9//SHANK3//GLRX//CHRNA3//P2RX2//HTR3E// |
| GO:0015711 | organic anion transport | Biological process | 21 | 460 | 509 | 17653 | 1.58329631844196 | 0.0263307467929563 | 0.360598403805886 | 1.57953682325395 | 0.0412573673870334 | SLC27A4//THBS1//UNC13B//CPLX1//GRM2//CCK//SLC26A1//AQP1//BEST1//CA7//CTNS//SLC25A5//SLC5A6//SLC23A2//PCTP//SLC52A2//CYP4F2//EDN1//IL1B//SLC35A2//SLC25A2// |
| GO:0048024 | regulation of mRNA splicing, via spliceosome | Biological process | 6 | 79 | 509 | 17653 | 2.63405535798662 | 0.0264289930589571 | 0.360598403805886 | 1.57791938311381 | 0.0117878192534381 | RBFOX1//MBNL3//CELF4//PTBP1//RBM8A//SRSF9// |
| GO:0051289 | protein homotetramerization | Biological process | 6 | 79 | 509 | 17653 | 2.63405535798662 | 0.0264289930589571 | 0.360598403805886 | 1.57791938311381 | 0.0117878192534381 | PEX5//CTH//DPYS//CRTC1//KCNJ12//CD79A// |
| GO:1903530 | regulation of secretion by cell | Biological process | 29 | 687 | 509 | 17653 | 1.46400311138946 | 0.0264463711796125 | 0.360598403805886 | 1.57763391095539 | 0.0569744597249509 | SYT9//DPP4//SLC25A5//IL1B//PPP3CB//ABCC8//CACNA2D2//UNC13B//CCK//CHRNA3//EDN1//CPLX1//LGI3//SYT2//SYT14//SIRT3//VSNL1//HMOX1//IL4R//BCR//PTAFR//SFRP1//CPLX3//FOXP3//PML//TNFSF4//TIAM1//LRRK2//PAX8// |
| GO:0000209 | protein polyubiquitination | Biological process | 15 | 298 | 509 | 17653 | 1.7457246080616 | 0.0266116196803051 | 0.362121496776466 | 1.57492869189686 | 0.0294695481335953 | TRIM5//UBE2D3//UBE2E1//UBE4B//RNF217//MGRN1//FBXL3//FBXO10//RLIM//RNF125//RNF114//SKP1//TRIM21//TNKS2//FBXL20// |
| GO:0021953 | central nervous system neuron differentiation | Biological process | 10 | 171 | 509 | 17653 | 2.02817127954136 | 0.0267170254990069 | 0.362462828406051 | 1.57321189504664 | 0.0196463654223969 | PHOX2A//ASCL1//SHANK3//CNTN2//FGFR2//OTP//DLX5//SATB2//FBXO45//SFRP1// |
| GO:0046903 | secretion | Biological process | 59 | 1591 | 509 | 17653 | 1.28612319543009 | 0.0267438934458997 | 0.362462828406051 | 1.57277536674565 | 0.115913555992141 | EDN1//LGALS3BP//THBS1//TLN1//FERMT3//CD74//CPLX1//LGI3//SNX19//EXOC4//VPS45//UNC13B//SNPH//CSN3//MED1//SLC6A3//SYT9//DPP4//SLC25A5//IL1B//PPP3CB//ABCC8//CACNA2D2//AQP1//GRM2//CCK//CHRNA3//SYT2//SYT14//CPLX3//SIRT3//CYP4F2//RAF1//VSNL1//KIT//HMOX1//IL4R//ACTR2//ALAD//ALDOC//DBNL//NFKB1//BIN2//RHOF//PTAFR//S100A12//TSPAN14//SCAMP1//BCR//STEAP2//CHRM1//SFRP1//FOXP3//PML//TNFSF4//TIAM1//LRRK2//COPS5//PAX8// |
| GO:0051899 | membrane depolarization | Biological process | 7 | 101 | 509 | 17653 | 2.40368417981287 | 0.0268271221110583 | 0.362863653674175 | 1.57142591392231 | 0.0137524557956778 | LRRK2//CCK//SCN3B//SCN1A//SCN7A//SLMAP//EDN1// |
| GO:0003206 | cardiac chamber morphogenesis | Biological process | 8 | 124 | 509 | 17653 | 2.23753089549401 | 0.027221027348766 | 0.363635350310652 | 1.56509548811577 | 0.0157170923379175 | FGFR2//PARVA//FZD2//HEYL//UBE4B//MED1//BMP10//WNT11// |
| GO:0031060 | regulation of histone methylation | Biological process | 5 | 59 | 509 | 17653 | 2.93912956611501 | 0.0273296165193282 | 0.363635350310652 | 1.56336646214957 | 0.00982318271119843 | PAXBP1//PAX5//PRDM12//OGT//KMT2E// |
| GO:0071806 | protein transmembrane transport | Biological process | 5 | 59 | 509 | 17653 | 2.93912956611501 | 0.0273296165193282 | 0.363635350310652 | 1.56336646214957 | 0.00982318271119843 | PEX5//TIMM23//TOMM40L//TIMM22//BCR// |
| GO:1902041 | regulation of extrinsic apoptotic signaling pathway via death domain receptors | Biological process | 5 | 59 | 509 | 17653 | 2.93912956611501 | 0.0273296165193282 | 0.363635350310652 | 1.56336646214957 | 0.00982318271119843 | HMOX1//RAF1//SFRP1//SKIL//THBS1// |
| GO:0006884 | cell volume homeostasis | Biological process | 3 | 23 | 509 | 17653 | 4.52370376697702 | 0.0274969712286777 | 0.363635350310652 | 1.56071514075569 | 0.00589390962671906 | SHANK3//SLC12A7//AQP1// |
| GO:0021544 | subpallium development | Biological process | 3 | 23 | 509 | 17653 | 4.52370376697702 | 0.0274969712286777 | 0.363635350310652 | 1.56071514075569 | 0.00589390962671906 | LRRK2//SHANK3//ASCL1// |
| GO:0043032 | positive regulation of macrophage activation | Biological process | 3 | 23 | 509 | 17653 | 4.52370376697702 | 0.0274969712286777 | 0.363635350310652 | 1.56071514075569 | 0.00589390962671906 | LRRK2//IL4R//THBS1// |
| GO:0051457 | maintenance of protein location in nucleus | Biological process | 3 | 23 | 509 | 17653 | 4.52370376697702 | 0.0274969712286777 | 0.363635350310652 | 1.56071514075569 | 0.00589390962671906 | TAF8//PML//SKP1// |
| GO:0071577 | zinc ion transmembrane transport | Biological process | 3 | 23 | 509 | 17653 | 4.52370376697702 | 0.0274969712286777 | 0.363635350310652 | 1.56071514075569 | 0.00589390962671906 | SLC39A8//SLC39A2//SLC30A6// |
| GO:0043030 | regulation of macrophage activation | Biological process | 4 | 40 | 509 | 17653 | 3.46817288801572 | 0.0275293951440269 | 0.363635350310652 | 1.56020333050631 | 0.00785854616895874 | IL4R//THBS1//LRRK2//CD74// |
| GO:0044743 | protein transmembrane import into intracellular organelle | Biological process | 4 | 40 | 509 | 17653 | 3.46817288801572 | 0.0275293951440269 | 0.363635350310652 | 1.56020333050631 | 0.00785854616895874 | PEX5//TIMM23//TOMM40L//TIMM22// |
| GO:0060412 | ventricular septum morphogenesis | Biological process | 4 | 40 | 509 | 17653 | 3.46817288801572 | 0.0275293951440269 | 0.363635350310652 | 1.56020333050631 | 0.00785854616895874 | FGFR2//FZD2//HEYL//WNT11// |
| GO:0051149 | positive regulation of muscle cell differentiation | Biological process | 7 | 102 | 509 | 17653 | 2.38011864863824 | 0.0281175670856077 | 0.370680518908314 | 1.55102226000379 | 0.0137524557956778 | EDN1//BMP10//DDX39B//IL4R//TNFSF14//CTH//KIT// |
| GO:0002790 | peptide secretion | Biological process | 25 | 576 | 509 | 17653 | 1.5052833715346 | 0.0282057616533183 | 0.370895788989353 | 1.54966216832408 | 0.0491159135559921 | CD74//SYT9//DPP4//SLC25A5//IL1B//PPP3CB//ABCC8//CACNA2D2//EDN1//CPLX1//SNX19//CPLX3//SIRT3//RAF1//VSNL1//SFRP1//S100A12//FOXP3//PML//UNC13B//TNFSF4//TIAM1//IL4R//LRRK2//PAX8// |
| GO:0035270 | endocrine system development | Biological process | 8 | 125 | 509 | 17653 | 2.21963064833006 | 0.0283798344881381 | 0.370895788989353 | 1.54699014168679 | 0.0157170923379175 | ALDH1A2//SLC6A3//OTP//WNT11//RAF1//PAX8//ONECUT2//ASCL1// |
| GO:0042981 | regulation of apoptotic process | Biological process | 58 | 1566 | 509 | 17653 | 1.28450847704286 | 0.0285478490322506 | 0.370895788989353 | 1.54442660856054 | 0.113948919449902 | CD74//PML//CCK//CDKN1B//MAEA//COPS5//FGFR3//FGFR2//AQP1//KIT//ASCL1//NFKB1//PAFAH2//PAK2//PIM1//MED1//PRKAA1//LIMS2//RAF1//SFRP1//THBS1//WNT11//CASP7//HMOX1//PAX8//EDN1//CDKN2A//UNC13B//DAPK3//MAP3K9//ARHGEF4//UBE2Z//SMPD1//TGM2//TIAM1//FOSL1//ALDH1A2//ARHGEF6//RAG1//TNFSF14//MYBL2//FZD3//YWHAB//SLC25A5//SKIL//LRRK2//ITGA5//CTH//NFATC4//IL1B//SGK2//FBXO10//BMP10//NF2//RTN4//TNFAIP8L2//RELT//OXSR1// |
| GO:0034620 | cellular response to unfolded protein | Biological process | 9 | 149 | 509 | 17653 | 2.09486952967392 | 0.0288602646859892 | 0.370895788989353 | 1.53969969018163 | 0.0176817288801572 | PARP16//CTH//EDEM3//CREB3L3//KDELR3//ASNA1//TLN1//GOSR2//COPS5// |
| GO:0007423 | sensory organ development | Biological process | 23 | 521 | 509 | 17653 | 1.53105520968064 | 0.0290905594965661 | 0.370895788989353 | 1.53624792591916 | 0.0451866404715128 | CTNS//MED1//FGFR2//WDR19//ALDH1A2//DLX5//INSIG2//BCR//PAX8//FZD3//KCNQ4//EDN1//GPD2//RAX//KIT//CDKN1B//CXCL14//FZD2//ASCL1//NDP//NF2//SKIL//CELF4// |
| GO:0071242 | cellular response to ammonium ion | Biological process | 5 | 60 | 509 | 17653 | 2.89014407334643 | 0.0291332833526378 | 0.370895788989353 | 1.53561056715479 | 0.00982318271119843 | CHRM1//CASP7//CHRNA3//LRRK2//PTAFR// |
| GO:0006887 | exocytosis | Biological process | 35 | 869 | 509 | 17653 | 1.39684753832624 | 0.0292043103826229 | 0.370895788989353 | 1.53455304453668 | 0.068762278978389 | LGALS3BP//THBS1//TLN1//FERMT3//VPS45//EXOC4//UNC13B//CPLX1//SYT2//SYT9//SYT14//CPLX3//SNPH//PPP3CB//LGI3//KIT//HMOX1//IL4R//ACTR2//ALAD//ALDOC//DBNL//NFKB1//BIN2//RHOF//PTAFR//S100A12//TSPAN14//SCAMP1//BCR//STEAP2//VSNL1//COPS5//LRRK2//SNX19// |
| GO:0022607 | cellular component assembly | Biological process | 101 | 2934 | 509 | 17653 | 1.19388364584045 | 0.029357433883725 | 0.370895788989353 | 1.53228190852584 | 0.198428290766208 | UBXN2B//UBXN2A//TP53INP2//SEPT7//WAS//DDX39B//SNRPB//EMP2//RHOF//ASF1B//CELF4//SRSF9//TLN1//PARD6B//ITGA5//CHRNA3//SMAD1//DBNL//SPTBN2//SHANK3//BSN//ACTR3//ACTR2//UBXN10//EHD3//MCIDAS//PARVA//WDR19//RFX4//ONECUT2//SKP1//BRK1//UNC13B//NFS1//ARFIP2//KIT//ARHGEF4//ARHGEF6//LMOD1//MYOM3//ARHGAP40//EPS8L2//CDC42EP1//MAPRE1//CDKN1B//RAF1//TRABD2B//CCK//FERMT2//FERMT3//CENPO//LIMS2//DNAJB12//CDKN2A//CCDC103//KCTD18//ALAD//P2RX2//HMOX1//KCNA6//CCDC88C//TGM2//SHKBP1//BMP10//GOSR2//MED6//NEK7//CTH//DPYS//CRTC1//KCNJ12//CD79A//GRIN2B//CD74//PRKAA1//PRKAB1//YWHAB//RTN4//MIS12//PALM//NF2//SFRP1//WNT11//DAPK3//THBS1//LRRTM1//LRRN1//EDN1//FBXO45//PPIH//DLG4//PML//TEAD1//TRIM21//BIN2//KIF2A//MYBL2//PEX5//LRRK2//FOSL1//PIP4K2C// |
| GO:0002456 | T cell mediated immunity | Biological process | 6 | 81 | 509 | 17653 | 2.56901695408572 | 0.0294304408369191 | 0.370895788989353 | 1.53120323257903 | 0.0117878192534381 | EMP2//PPP3CB//WAS//IL1B//FOXP3//TNFSF4// |
| GO:0032602 | chemokine production | Biological process | 6 | 81 | 509 | 17653 | 2.56901695408572 | 0.0294304408369191 | 0.370895788989353 | 1.53120323257903 | 0.0117878192534381 | HMOX1//IL1B//TNFSF4//IL4R//TIRAP//CD74// |
| GO:0060415 | muscle tissue morphogenesis | Biological process | 6 | 81 | 509 | 17653 | 2.56901695408572 | 0.0294304408369191 | 0.370895788989353 | 1.53120323257903 | 0.0117878192534381 | FZD2//UBE4B//MED1//MYOM3//BMP10//FGFR2// |
| GO:0097581 | lamellipodium organization | Biological process | 6 | 81 | 509 | 17653 | 2.56901695408572 | 0.0294304408369191 | 0.370895788989353 | 1.53120323257903 | 0.0117878192534381 | WAS//BRK1//ARFIP2//KIT//ARHGEF4//ARHGEF6// |
| GO:0033559 | unsaturated fatty acid metabolic process | Biological process | 7 | 103 | 509 | 17653 | 2.35701070059321 | 0.0294494482402021 | 0.370895788989353 | 1.53092283766738 | 0.0137524557956778 | EDN1//CD74//CYP4F2//PLA2G4C//CYP2C18//CYP2S1//CYP4F3// |
| GO:1903037 | regulation of leukocyte cell-cell adhesion | Biological process | 15 | 302 | 509 | 17653 | 1.72260242782238 | 0.0294724861284283 | 0.370895788989353 | 1.53058322803479 | 0.0294695481335953 | DPP4//PAK2//TNFSF14//FOXP3//CD209//IL1B//TNFSF4//GPNMB//NDFIP1//CD74//ZMIZ1//RAG1//IL4R//TNFAIP8L2//PTAFR// |
| GO:1903039 | positive regulation of leukocyte cell-cell adhesion | Biological process | 12 | 224 | 509 | 17653 | 1.85794976143699 | 0.0295492004757001 | 0.370895788989353 | 1.52945426549979 | 0.0235756385068762 | DPP4//PAK2//TNFSF14//FOXP3//CD209//IL1B//TNFSF4//ZMIZ1//RAG1//CD74//IL4R//PTAFR// |
| GO:0097485 | neuron projection guidance | Biological process | 13 | 250 | 509 | 17653 | 1.80344990176817 | 0.0298104873192333 | 0.370895788989353 | 1.52563092439259 | 0.0255402750491159 | EFNA3//NCAM1//SPTBN2//CNTN2//VLDLR//SHANK3//UNC5A//FZD3//SEMA4G//SEMA7A//DLX5//ZSWIM6//SEMA5B// |
| GO:0009112 | nucleobase metabolic process | Biological process | 4 | 41 | 509 | 17653 | 3.38358330538119 | 0.0298329208427048 | 0.370895788989353 | 1.52530422421858 | 0.00785854616895874 | PRTFDC1//UCK2//UCK1//DPYS// |
| GO:0048488 | synaptic vesicle endocytosis | Biological process | 4 | 41 | 509 | 17653 | 3.38358330538119 | 0.0298329208427048 | 0.370895788989353 | 1.52530422421858 | 0.00785854616895874 | SH3GL1//LRRK2//SYT2//PACSIN1// |
| GO:0072595 | maintenance of protein localization in organelle | Biological process | 4 | 41 | 509 | 17653 | 3.38358330538119 | 0.0298329208427048 | 0.370895788989353 | 1.52530422421858 | 0.00785854616895874 | KDELR3//TAF8//PML//SKP1// |
| GO:0001819 | positive regulation of cytokine production | Biological process | 19 | 411 | 509 | 17653 | 1.60329160273233 | 0.0300052437643317 | 0.370895788989353 | 1.52280284065012 | 0.037328094302554 | TNFSF4//IL1B//NFKB1//POLR2E//RNF135//TIRAP//FOXP3//NFATC4//PTAFR//ABCC8//THBS1//WNT11//RNF125//HMOX1//SEMA7A//CD74//IL4R//LRRK2//AGPAT1// |
| GO:0065003 | protein-containing complex assembly | Biological process | 67 | 1852 | 509 | 17653 | 1.254684576118 | 0.0301133401516808 | 0.370895788989353 | 1.52124107016009 | 0.131630648330059 | SEPT7//DDX39B//SNRPB//ASF1B//CELF4//SRSF9//CHRNA3//SMAD1//WAS//SKP1//UNC13B//MYOM3//ARHGAP40//EPS8L2//CDC42EP1//LMOD1//MAPRE1//CDKN1B//RAF1//TRABD2B//CCK//FERMT2//TLN1//FERMT3//CENPO//ACTR3//ACTR2//ARFIP2//DNAJB12//CCDC103//KCTD18//ALAD//P2RX2//EHD3//HMOX1//KCNA6//CCDC88C//TGM2//SHKBP1//GOSR2//MED6//CTH//DPYS//CRTC1//KCNJ12//CD79A//GRIN2B//CD74//PRKAA1//PRKAB1//YWHAB//RTN4//MIS12//SPTBN2//TRIM21//BRK1//DBNL//BIN2//PEX5//LRRK2//FOSL1//PPIH//DLG4//PML//TEAD1//PARD6B//NFS1// |
| GO:0007254 | JNK cascade | Biological process | 11 | 200 | 509 | 17653 | 1.90749508840864 | 0.0307610727883501 | 0.370895788989353 | 1.51199852264381 | 0.0216110019646365 | MAP3K9//DBNL//EDN1//TIAM1//SFRP1//COPS5//TIRAP//IL1B//TRIB1//TAB1//ARHGEF6// |
| GO:0002719 | negative regulation of cytokine production involved in immune response | Biological process | 3 | 24 | 509 | 17653 | 4.33521611001965 | 0.0307695642892306 | 0.370895788989353 | 1.51187865351278 | 0.00589390962671906 | FOXP3//CD96//HMOX1// |
| GO:0021591 | ventricular system development | Biological process | 3 | 24 | 509 | 17653 | 4.33521611001965 | 0.0307695642892306 | 0.370895788989353 | 1.51187865351278 | 0.00589390962671906 | AQP1//PAX5//UCHL5// |
| GO:0033119 | negative regulation of RNA splicing | Biological process | 3 | 24 | 509 | 17653 | 4.33521611001965 | 0.0307695642892306 | 0.370895788989353 | 1.51187865351278 | 0.00589390962671906 | CELF4//PTBP1//SRSF9// |
| GO:0034695 | response to prostaglandin E | Biological process | 3 | 24 | 509 | 17653 | 4.33521611001965 | 0.0307695642892306 | 0.370895788989353 | 1.51187865351278 | 0.00589390962671906 | PRKAA1//SFRP1//TNFSF4// |
| GO:0035588 | G-protein coupled purinergic receptor signaling pathway | Biological process | 3 | 24 | 509 | 17653 | 4.33521611001965 | 0.0307695642892306 | 0.370895788989353 | 1.51187865351278 | 0.00589390962671906 | GPR34//PTAFR//CNTN2// |
| GO:0098868 | bone growth | Biological process | 3 | 24 | 509 | 17653 | 4.33521611001965 | 0.0307695642892306 | 0.370895788989353 | 1.51187865351278 | 0.00589390962671906 | EVC//FGFR3//FGFR2// |
| GO:0097479 | synaptic vesicle localization | Biological process | 8 | 127 | 509 | 17653 | 2.18467583497053 | 0.0307988145433444 | 0.370895788989353 | 1.51146599931748 | 0.0157170923379175 | UNC13B//CPLX1//SYT2//SYT9//SYT14//CPLX3//SNPH//LRRK2// |
| GO:0042147 | retrograde transport, endosome to Golgi | Biological process | 6 | 82 | 509 | 17653 | 2.53768747903589 | 0.0310116847714962 | 0.370895788989353 | 1.50847463920967 | 0.0117878192534381 | EHD3//LRRK2//AP1S1//VPS53//VPS26A//GOSR2// |
| GO:0050810 | regulation of steroid biosynthetic process | Biological process | 6 | 82 | 509 | 17653 | 2.53768747903589 | 0.0310116847714962 | 0.370895788989353 | 1.50847463920967 | 0.0117878192534381 | INSIG2//NFKB1//IDI1//NFYA//PRKAA1//IL1B// |
| GO:0031329 | regulation of cellular catabolic process | Biological process | 33 | 814 | 509 | 17653 | 1.40601603568205 | 0.0310203259905088 | 0.370895788989353 | 1.50835364252708 | 0.0648330058939096 | NUP214//OGT//UCHL5//LRRK2//DAPK3//DRAM1//PIP4K2C//EIF4G2//PRKAA1//TP53INP2//TRIM21//ABHD5//LARP1//HMOX1//ATP6V1G2//PRKAB1//EXOC4//VPS26A//TRIB1//RNF217//RNF125//RNF114//CARHSP1//YWHAB//IL1B//TIRAP//DAZL//ALAD//WDR91//CAMKK2//CD81//PML//TRIM67// |
| GO:0008610 | lipid biosynthetic process | Biological process | 29 | 697 | 509 | 17653 | 1.44299876258904 | 0.0311354939713605 | 0.370895788989353 | 1.50674423971183 | 0.0569744597249509 | EDN1//CD74//ST6GALNAC6//PYURF//PGAP3//PRKAA1//PRKAB1//MOGAT2//AGPAT1//LCLAT1//ABHD5//PCTP//PIP4K2C//GAL3ST1//ST8SIA5//EBP//IDI1//INSIG2//FSHB//MED1//NFKB1//FAXDC2//FGFR3//FGFR2//ALDH1A2//NFYA//SMPD1//FAM57B//IL1B// |
| GO:0002694 | regulation of leukocyte activation | Biological process | 23 | 525 | 509 | 17653 | 1.5193900271307 | 0.0313508887311863 | 0.370895788989353 | 1.50375014333153 | 0.0451866404715128 | TIRAP//CD74//CD81//DPP4//PAK2//TNFSF14//FOXP3//CD209//IL1B//TNFSF4//GPNMB//NDFIP1//IL4R//THBS1//HMOX1//BCR//PTAFR//HMGB3//SFRP1//ZMIZ1//RAG1//TNFAIP8L2//LRRK2// |
| GO:0034613 | cellular protein localization | Biological process | 64 | 1762 | 509 | 17653 | 1.25972227487518 | 0.0313741812143182 | 0.370895788989353 | 1.5034275993354 | 0.12573673870334 | SPRN//PML//NUP214//U2AF1//DDX39B//SRSF9//RBM8A//YWHAB//GOLGA7B//EXOC4//KDELR3//GOSR2//TIMM23//TOMM34//TIMM22//VPS45//AP1S1//ARFIP2//RABL3//KCNIP3//MLPH//VPS26A//CD74//TLN1//PEX5//TOMM40L//REEP2//LRRK2//MED1//PTPRU//EMP2//WNT11//TTK//MIS12//MAPRE1//SYS1//DLG4//IL1B//TAF8//SKP1//WDR19//CD81//TULP4//BCR//TNKS2//CNTN2//FERMT2//PACSIN1//TIAM1//EHD3//PALM//SCN3B//SLMAP//TSPAN14//CHRM1//SHANK3//GRIPAP1//NF2//NUCB1//TP53INP2//PRKAA1//UBE2D3//RTN4//SEPT7// |
| GO:1901615 | organic hydroxy compound metabolic process | Biological process | 22 | 497 | 509 | 17653 | 1.5352073146146 | 0.0314962465412422 | 0.370895788989353 | 1.50174119870417 | 0.0432220039292731 | IMPA2//SLC5A3//MOGAT2//MED1//EBP//IDI1//INSIG2//PRKAA1//VLDLR//NFKB1//FAXDC2//GPD2//PTAFR//CYP4F3//CYP4F2//SLC6A3//CTNS//ALDH1A2//NFYA//IL1B//KIT//PAX8// |
| GO:0044267 | cellular protein metabolic process | Biological process | 176 | 5421 | 509 | 17653 | 1.12598861518311 | 0.0315997172381482 | 0.370895788989353 | 1.50031680353544 | 0.345776031434185 | CDKN1B//CAMKK2//LRRK2//DLG4//FGFR3//FGFR2//GRIN2B//IL1B//KIT//SMAD1//NCAM1//MAPK4//RAF1//SPTBN2//YWHAB//SHANK3//TAB1//PRKAA1//THBS1//PROK1//CD74//CD81//UBE4B//RNF217//MGRN1//FBXL3//FBXO10//RLIM//RNF125//RNF114//SKP1//TRIM21//UBE2D3//UBE2E1//TNKS2//FBXL20//COPS5//PPIH//CCDC88C//PML//GPNMB//FTSJ1//MAEA//RNF38//FBXO45//RNF149//MED1//DCAF16//TULP4//KLHL14//PEX5//RNF123//UBE2Z//HLTF//TRIM7//RNF135//KLHL22//EIF4G2//LARP1//RBM3//RBM8A//CHRM1//SGK2//GLYCTK//NEK7//DAPK3//MARK1//MAP3K9//PAK2//PIM1//PPP3CB//PRKAB1//BCR//WNT11//MEX3B//AATK//OXSR1//TRIB1//LRRTM1//NF2//CEP85//PTPRU//PTPRT//PTPN18//PARP16//SIRT3//SNRPB//ST8SIA5//EDEM3//MGAT5//MAN1C1//GAL3ST1//TET3//GALNT10//OGT//SPRN//NRN1//VNN2//PGGT1B//PYURF//PGAP3//UCHL5//USP37//TP53INP2//USP46//USP45//NFS1//CCK//CHRNA3//TIRAP//ARHGEF6//DBNL//CASP7//RAG1//BMP10//TTK//CNTN2//RBMS3//PTAFR//DDX39B//GALNT3//ST3GAL3//USP54//NUP214//CELF4//PURA//CUX1//KRT2//TGM4//TGM2//TTL//GOLGA7B//CTH//ST6GALNAC6//PARG//PAXBP1//FOXP3//UBXN2A//NDFIP1//CDKN2A//EMP2//MAPKAP1//EDN1//NFKB1//PPP1R2//PPP1R26//VLDLR//KMT2E//SMPD1//DNAJB12//AQP1//TNFSF14//UBXN2B//S100A12//SPRY3//TRIM5//TIAM1//SFRP1//TGOLN2//NUCB1//PRDM4//DAZL//ITGA5//PAX5//PRDM12//TRIM68//POLR2E//SH3BP5//SEMA7A//GLRX//ALAD//EFNA3//WDR91//BEX4//TRABD2B//TRIM67// |
| GO:0061061 | muscle structure development | Biological process | 27 | 640 | 509 | 17653 | 1.46313543713163 | 0.031646173575648 | 0.370895788989353 | 1.49967879419144 | 0.0530451866404715 | FZD2//UBE4B//MED1//EVC//PAXBP1//LMOD1//MYOM3//HEYL//PAX5//HIVEP3//BMP10//MBNL3//CXCL14//TNFSF14//PRKAA1//P2RX2//C11ORF88//CACNA2D2//NFATC4//PTBP1//FGFR2//PIM1//EDN1//DDX39B//IL4R//CTH//KIT// |
| GO:0002685 | regulation of leukocyte migration | Biological process | 10 | 176 | 509 | 17653 | 1.97055277728166 | 0.0317080662608184 | 0.370895788989353 | 1.49883024292604 | 0.0196463654223969 | HMOX1//BCR//THBS1//TNFSF14//JAM3//TIRAP//EDN1//CD74//PTAFR//CXCL14// |
| GO:0030041 | actin filament polymerization | Biological process | 10 | 176 | 509 | 17653 | 1.97055277728166 | 0.0317080662608184 | 0.370895788989353 | 1.49883024292604 | 0.0196463654223969 | ARHGAP40//EPS8L2//CDC42EP1//LMOD1//ACTR3//ACTR2//ARFIP2//WAS//SPTBN2//BRK1// |
| GO:0050803 | regulation of synapse structure or activity | Biological process | 10 | 176 | 509 | 17653 | 1.97055277728166 | 0.0317080662608184 | 0.370895788989353 | 1.49883024292604 | 0.0196463654223969 | SHANK3//LRRTM1//LRRN1//LRRK2//ACTR2//TIAM1//GRIN2B//ZNF804A//GRIPAP1//NFATC4// |
| GO:0043408 | regulation of MAPK cascade | Biological process | 31 | 757 | 509 | 17653 | 1.42025574013854 | 0.0318981963449029 | 0.370895788989353 | 1.49623387304318 | 0.0609037328094303 | TAB1//LRRK2//RAF1//IL1B//KIT//PRKAA1//THBS1//PROK1//CD74//CD81//MAP3K9//DBNL//TRIB1//EDN1//S100A12//SPRY3//SMPD1//RNF149//NF2//FGFR3//FGFR2//TRIM5//TIAM1//SFRP1//COPS5//TIRAP//SIRT3//GPNMB//SEMA7A//BMP10//PAK2// |
| GO:0002638 | negative regulation of immunoglobulin production | Biological process | 2 | 10 | 509 | 17653 | 6.93634577603143 | 0.0320316698956391 | 0.370895788989353 | 1.49442041982643 | 0.00392927308447937 | FOXP3//NDFIP1// |
| GO:0002713 | negative regulation of B cell mediated immunity | Biological process | 2 | 10 | 509 | 17653 | 6.93634577603143 | 0.0320316698956391 | 0.370895788989353 | 1.49442041982643 | 0.00392927308447937 | FOXP3//NDFIP1// |
| GO:0002887 | negative regulation of myeloid leukocyte mediated immunity | Biological process | 2 | 10 | 509 | 17653 | 6.93634577603143 | 0.0320316698956391 | 0.370895788989353 | 1.49442041982643 | 0.00392927308447937 | HMOX1//BCR// |
| GO:0002890 | negative regulation of immunoglobulin mediated immune response | Biological process | 2 | 10 | 509 | 17653 | 6.93634577603143 | 0.0320316698956391 | 0.370895788989353 | 1.49442041982643 | 0.00392927308447937 | FOXP3//NDFIP1// |
| GO:0003149 | membranous septum morphogenesis | Biological process | 2 | 10 | 509 | 17653 | 6.93634577603143 | 0.0320316698956391 | 0.370895788989353 | 1.49442041982643 | 0.00392927308447937 | FGFR2//FZD2// |
| GO:0006222 | UMP biosynthetic process | Biological process | 2 | 10 | 509 | 17653 | 6.93634577603143 | 0.0320316698956391 | 0.370895788989353 | 1.49442041982643 | 0.00392927308447937 | UCK2//UCK1// |
| GO:0007028 | cytoplasm organization | Biological process | 2 | 10 | 509 | 17653 | 6.93634577603143 | 0.0320316698956391 | 0.370895788989353 | 1.49442041982643 | 0.00392927308447937 | ZMIZ1//FOSL1// |
| GO:0009173 | pyrimidine ribonucleoside monophosphate metabolic process | Biological process | 2 | 10 | 509 | 17653 | 6.93634577603143 | 0.0320316698956391 | 0.370895788989353 | 1.49442041982643 | 0.00392927308447937 | UCK2//UCK1// |
| GO:0009174 | pyrimidine ribonucleoside monophosphate biosynthetic process | Biological process | 2 | 10 | 509 | 17653 | 6.93634577603143 | 0.0320316698956391 | 0.370895788989353 | 1.49442041982643 | 0.00392927308447937 | UCK2//UCK1// |
| GO:0009629 | response to gravity | Biological process | 2 | 10 | 509 | 17653 | 6.93634577603143 | 0.0320316698956391 | 0.370895788989353 | 1.49442041982643 | 0.00392927308447937 | PTAFR//FOSL1// |
| GO:0021548 | pons development | Biological process | 2 | 10 | 509 | 17653 | 6.93634577603143 | 0.0320316698956391 | 0.370895788989353 | 1.49442041982643 | 0.00392927308447937 | PHOX2A//ASCL1// |
| GO:0021877 | forebrain neuron fate commitment | Biological process | 2 | 10 | 509 | 17653 | 6.93634577603143 | 0.0320316698956391 | 0.370895788989353 | 1.49442041982643 | 0.00392927308447937 | SATB2//ASCL1// |
| GO:0021892 | cerebral cortex GABAergic interneuron differentiation | Biological process | 2 | 10 | 509 | 17653 | 6.93634577603143 | 0.0320316698956391 | 0.370895788989353 | 1.49442041982643 | 0.00392927308447937 | CNTN2//ASCL1// |
| GO:0032306 | regulation of prostaglandin secretion | Biological process | 2 | 10 | 509 | 17653 | 6.93634577603143 | 0.0320316698956391 | 0.370895788989353 | 1.49442041982643 | 0.00392927308447937 | EDN1//IL1B// |
| GO:0032308 | positive regulation of prostaglandin secretion | Biological process | 2 | 10 | 509 | 17653 | 6.93634577603143 | 0.0320316698956391 | 0.370895788989353 | 1.49442041982643 | 0.00392927308447937 | EDN1//IL1B// |
| GO:0032905 | transforming growth factor beta1 production | Biological process | 2 | 10 | 509 | 17653 | 6.93634577603143 | 0.0320316698956391 | 0.370895788989353 | 1.49442041982643 | 0.00392927308447937 | FOXP3//THBS1// |
| GO:0032908 | regulation of transforming growth factor beta1 production | Biological process | 2 | 10 | 509 | 17653 | 6.93634577603143 | 0.0320316698956391 | 0.370895788989353 | 1.49442041982643 | 0.00392927308447937 | FOXP3//THBS1// |
| GO:0042758 | long-chain fatty acid catabolic process | Biological process | 2 | 10 | 509 | 17653 | 6.93634577603143 | 0.0320316698956391 | 0.370895788989353 | 1.49442041982643 | 0.00392927308447937 | CYP4F3//CYP4F2// |
| GO:0045625 | regulation of T-helper 1 cell differentiation | Biological process | 2 | 10 | 509 | 17653 | 6.93634577603143 | 0.0320316698956391 | 0.370895788989353 | 1.49442041982643 | 0.00392927308447937 | IL4R//TNFSF4// |
| GO:0046049 | UMP metabolic process | Biological process | 2 | 10 | 509 | 17653 | 6.93634577603143 | 0.0320316698956391 | 0.370895788989353 | 1.49442041982643 | 0.00392927308447937 | UCK2//UCK1// |
| GO:0048340 | paraxial mesoderm morphogenesis | Biological process | 2 | 10 | 509 | 17653 | 6.93634577603143 | 0.0320316698956391 | 0.370895788989353 | 1.49442041982643 | 0.00392927308447937 | EXOC4//WNT11// |
| GO:0060159 | regulation of dopamine receptor signaling pathway | Biological process | 2 | 10 | 509 | 17653 | 6.93634577603143 | 0.0320316698956391 | 0.370895788989353 | 1.49442041982643 | 0.00392927308447937 | LRRK2//PALM// |
| GO:0072578 | neurotransmitter-gated ion channel clustering | Biological process | 2 | 10 | 509 | 17653 | 6.93634577603143 | 0.0320316698956391 | 0.370895788989353 | 1.49442041982643 | 0.00392927308447937 | DLG4//SHANK3// |
| GO:0090394 | negative regulation of excitatory postsynaptic potential | Biological process | 2 | 10 | 509 | 17653 | 6.93634577603143 | 0.0320316698956391 | 0.370895788989353 | 1.49442041982643 | 0.00392927308447937 | LRRK2//CELF4// |
| GO:1901569 | fatty acid derivative catabolic process | Biological process | 2 | 10 | 509 | 17653 | 6.93634577603143 | 0.0320316698956391 | 0.370895788989353 | 1.49442041982643 | 0.00392927308447937 | CYP4F3//CYP4F2// |
| GO:1902950 | regulation of dendritic spine maintenance | Biological process | 2 | 10 | 509 | 17653 | 6.93634577603143 | 0.0320316698956391 | 0.370895788989353 | 1.49442041982643 | 0.00392927308447937 | GRIN2B//ZNF804A// |
| GO:1904338 | regulation of dopaminergic neuron differentiation | Biological process | 2 | 10 | 509 | 17653 | 6.93634577603143 | 0.0320316698956391 | 0.370895788989353 | 1.49442041982643 | 0.00392927308447937 | SFRP1//TIAM1// |
| GO:2000341 | regulation of chemokine (C-X-C motif) ligand 2 production | Biological process | 2 | 10 | 509 | 17653 | 6.93634577603143 | 0.0320316698956391 | 0.370895788989353 | 1.49442041982643 | 0.00392927308447937 | TIRAP//CD74// |
| GO:2001225 | regulation of chloride transport | Biological process | 2 | 10 | 509 | 17653 | 6.93634577603143 | 0.0320316698956391 | 0.370895788989353 | 1.49442041982643 | 0.00392927308447937 | PTAFR//CA7// |
| GO:0071774 | response to fibroblast growth factor | Biological process | 9 | 152 | 509 | 17653 | 2.05352342053562 | 0.0322248201130341 | 0.370895788989353 | 1.49180949830768 | 0.0176817288801572 | FGFR3//FGFR2//GALNT3//POLR2E//PTBP1//SPRY3//THBS1//SFRP1//ELK1// |
| GO:0042089 | cytokine biosynthetic process | Biological process | 7 | 105 | 509 | 17653 | 2.31211525867714 | 0.0322394782291732 | 0.370895788989353 | 1.49161199552776 | 0.0137524557956778 | FOXP3//THBS1//HMOX1//IL1B//NFKB1//TIRAP//PTAFR// |
| GO:0071621 | granulocyte chemotaxis | Biological process | 7 | 105 | 509 | 17653 | 2.31211525867714 | 0.0322394782291732 | 0.370895788989353 | 1.49161199552776 | 0.0137524557956778 | THBS1//IL1B//S100A12//JAM3//TIRAP//EDN1//CD74// |
| GO:0014047 | glutamate secretion | Biological process | 4 | 42 | 509 | 17653 | 3.30302179811021 | 0.032247038877087 | 0.370895788989353 | 1.49151015880805 | 0.00785854616895874 | CCK//UNC13B//CPLX1//GRM2// |
| GO:0032101 | regulation of response to external stimulus | Biological process | 33 | 817 | 509 | 17653 | 1.40085318610182 | 0.0324220407716652 | 0.372029061803858 | 1.48915965241227 | 0.0648330058939096 | CREB3L3//FOXP3//THBS1//TNFSF14//EDN1//C1QA//CD81//IL1B//TRIM5//TRIB1//CCK//RNF125//RGMA//RTN4//SEMA4G//SEMA7A//TNFSF4//NFKB1//BCR//TNFAIP8L2//NDFIP1//S100A12//TGM2//SLC6A3//ABCC8//MED1//JAM3//TIRAP//CD74//UNC13B//ZSWIM6//TIAM1//CXCL14// |
| GO:0071310 | cellular response to organic substance | Biological process | 92 | 2656 | 509 | 17653 | 1.20132494615002 | 0.0325064198872255 | 0.372029061803858 | 1.4880308591363 | 0.180746561886051 | AGPAT1//CD74//MED1//PARP16//TAB1//FERMT2//FSHB//SMAD1//PML//SKIL//CHRM1//ATP6V1G2//FGFR3//FGFR2//GALNT3//POLR2E//PTBP1//NREP//HMOX1//LRRTM1//IL1B//IL4R//KIT//PIM1//PTAFR//DLX5//BMP10//RGMA//UBE2D3//THBS1//HTRA4//RASL11B//ONECUT2//SFRP1//CALCOCO1//NRIP1//ITGA5//CTH//EDEM3//CREB3L3//TRIM5//TRIB1//TNFSF4//RELT//TNFSF14//PAK2//RAF1//PPP3CB//VSNL1//ACTR2//KDELR3//ASNA1//TLN1//GOSR2//SPRY3//OGT//NCAM1//TRIM68//TRIM21//HEYL//TIAM1//EDN1//NFKB1//SKP1//DNAJB12//MRC1//BCR//AQP1//WNT11//ALDH1A2//UNC13B//PTPN18//ACTR3//DAPK3//WAS//ALAD//PRKAA1//PAX8//ELK1//CDKN1B//CASP7//LARP1//CHRNA3//LRRK2//COPS5//NFATC4//UBXN2A//SLC25A5//MYBL2//SATB2//MAPK4//ABCC8// |
| GO:0045844 | positive regulation of striated muscle tissue development | Biological process | 6 | 83 | 509 | 17653 | 2.5071129310957 | 0.0326474017261446 | 0.372029061803858 | 1.48615137674418 | 0.0117878192534381 | PRKAA1//FGFR2//BMP10//PIM1//EDN1//DDX39B// |
| GO:0048636 | positive regulation of muscle organ development | Biological process | 6 | 83 | 509 | 17653 | 2.5071129310957 | 0.0326474017261446 | 0.372029061803858 | 1.48615137674418 | 0.0117878192534381 | PRKAA1//FGFR2//BMP10//PIM1//EDN1//DDX39B// |
| GO:0050896 | response to stimulus | Biological process | 284 | 9123 | 509 | 17653 | 1.07964605962563 | 0.0327002890182652 | 0.372029061803858 | 1.48544840885399 | 0.557956777996071 | CAMKK2//LRRK2//DLG4//FGFR3//FGFR2//GRIN2B//IL1B//KIT//SMAD1//NCAM1//MAPK4//RAF1//SPTBN2//YWHAB//SHANK3//TAB1//PRKAA1//THBS1//PROK1//CD74//CD81//COPS5//DPP4//USP46//FBXL20//CCK//CDKN1B//ALAD//P2RX2//PML//EMP2//PPP3CB//AGPAT1//MED1//TNFSF4//TRIM5//CD209//NFKB1//PAK2//SKP1//HMOX1//DBNL//RNF125//RAG1//JAM3//CD79A//RBMS3//FOXP3//IL4R//CD96//S100A12//WAS//CREB3L3//TIRAP//UBE2D3//NDFIP1//WNT11//DNAJB5//CLEC1A//AP1S1//CREBZF//FOSL1//AKAP10//VPS45//EHD3//PAFAH2//CYP4F2//C1QA//FERMT2//CTNND2//HIC1//TRABD2B//MARK1//CCDC88C//NDP//CALCOCO1//TNKS2//KREMEN1//VPS26A//FZD2//GNAO1//FZD3//FAM13A//GPNMB//EBP//RALBP1//PRDM4//PTPRT//CHRM1//CHRNA3//DGKG//FGF11//FSHB//HTR3E//KCNIP3//KCNIP2//ARHGAP40//IMPA2//LGALS3BP//MRC1//NRGN//CC2D1A//PRKAB1//HIVEP3//BCR//SH3GL1//RASL11B//SMPD1//ABCC8//VLDLR//RERGL//OGT//TNFSF14//CHRFAM7A//SH3BP5//CXCL14//SGK2//UNC13B//DAPK3//CARHSP1//ARHGEF4//POLR2M//PLA2G4C//OXSR1//UCHL5//USP45//PARG//POLR2E//CYP2C18//CYP2S1//PTAFR//TAC4//NFATC4//REL//SEMA7A//RELT//VSIG4//PAX5//FBXO45//SLC23A2//PARP16//EDN1//TSPAN14//TSPAN18//SKIL//PTPRU//TPRA1//GPR160//GNAZ//GPR34//GRM2//SFRP1//TIAM1//ARHGEF6//GPR3//PALM//HEYL//ASCL1//EVC//ITGA5//TLN1//FERMT3//TRIB1//MAP3K9//DDAH1//ARFIP2//RHOF//RALGPS2//CDKN2A//RASSF1//RALGDS//CDC42EP1//EPS8L2//EFNA3//CNTN2//UNC5A//SLC27A4//ATP6V1G2//CTNS//GALNT3//PTBP1//UBE4B//AQP1//SNN//SRSF9//ELK1//VASH1//PLLP//STEAP2//SPRY3//PPP1R2//RGS9BP//SLC6A3//HAAO//SLC30A6//BMP10//TTK//PIP4K2C//PIK3IP1//SLC25A5//SLC5A6//ANO3//MGRN1//BRK1//NREP//LRRTM1//PIM1//RFX4//DLX5//RGMA//HTRA4//ONECUT2//NRIP1//CTH//EDEM3//MAPKAP1//LARP1//IQSEC3//SLC52A2//ALDH1A2//RABL3//INSIG2//PGGT1B//WDFY1//TRIM21//GPX8//NRBF2//ARHGDIB//TEAD1//NF2//RNF149//VSNL1//ACTR2//KDELR3//ASNA1//GOSR2//DNAJB12//ACTR3//PTPN18//PAX8//TGM2//CRTC1//FBXL3//BHLHE40//ALDOC//BIN2//SCAMP1//HMGB3//TNFAIP8L2//RNF135//SHKBP1//ENHO//ZMIZ1//TRIM67//RTN4//SEMA4G//SEMA5B//BEST1//MMP24//PRDM12//SCN1A//RALGAPB//CGNL1//DIXDC1//TRIM68//WDR19//DDX39B//SIRT3//SATB2//PARVA//NUCB1//CASP7//CELF4//BSN//LYPD6//NUP214//ZSWIM6//SLC25A2//LDOC1//FAM168A//UBXN2A//MYBL2//SHISA9//LIMS2// |
| GO:0007266 | Rho protein signal transduction | Biological process | 11 | 202 | 509 | 17653 | 1.8886089984244 | 0.0327306360747147 | 0.372029061803858 | 1.48504555469644 | 0.0216110019646365 | WAS//ARHGDIB//ARHGEF4//RAF1//BCR//EPS8L2//TIAM1//ARHGEF6//CDC42EP1//PAK2//RHOF// |
| GO:0050920 | regulation of chemotaxis | Biological process | 11 | 202 | 509 | 17653 | 1.8886089984244 | 0.0327306360747147 | 0.372029061803858 | 1.48504555469644 | 0.0216110019646365 | THBS1//TNFSF14//SEMA4G//SEMA7A//JAM3//TIRAP//EDN1//CD74//ZSWIM6//TIAM1//CXCL14// |
| GO:0043067 | regulation of programmed cell death | Biological process | 58 | 1579 | 509 | 17653 | 1.27393304309634 | 0.0328149853018148 | 0.372361989255325 | 1.48392778593219 | 0.113948919449902 | CD74//PML//CCK//CDKN1B//MAEA//COPS5//FGFR3//FGFR2//AQP1//KIT//ASCL1//NFKB1//PAFAH2//PAK2//PIM1//MED1//PRKAA1//LIMS2//RAF1//SFRP1//THBS1//WNT11//CASP7//HMOX1//PAX8//SGK2//DAPK3//FBXO10//BMP10//NF2//RTN4//TNFAIP8L2//RELT//OXSR1//EDN1//CDKN2A//UNC13B//MAP3K9//ARHGEF4//UBE2Z//SMPD1//TGM2//TIAM1//FOSL1//ALDH1A2//ARHGEF6//LRRK2//RAG1//TNFSF14//MYBL2//FZD3//YWHAB//SLC25A5//SKIL//ITGA5//CTH//NFATC4//IL1B// |
| GO:0060193 | positive regulation of lipase activity | Biological process | 5 | 62 | 509 | 17653 | 2.79691361936751 | 0.0329617363080274 | 0.373400707958441 | 1.48198991927061 | 0.00982318271119843 | FGFR3//FGFR2//KIT//PTAFR//ABHD5// |
| GO:0051093 | negative regulation of developmental process | Biological process | 38 | 968 | 509 | 17653 | 1.36147282794005 | 0.0333549609118161 | 0.376600236054744 | 1.47683956395436 | 0.0746561886051081 | SFRP1//LRRK2//EFNA3//VASH1//PML//ABCC8//THBS1//RFX4//RTN4//HMGB3//CD74//TNFSF4//SKIL//FAM57B//IL4R//TRIB1//MBNL3//CXCL14//ASCL1//MED1//CNTN2//DIXDC1//FZD3//FOXP3//NDFIP1//FGFR3//RGMA//SEMA4G//SEMA7A//TLX2//NFATC4//PTBP1//BCR//CDKN1B//WNT11//PAX8//ARHGDIB//LIMS2// |
| GO:0009581 | detection of external stimulus | Biological process | 8 | 129 | 509 | 17653 | 2.15080489179269 | 0.0333555436044347 | 0.376600236054744 | 1.47683197713754 | 0.0157170923379175 | ANO3//RGS9BP//SEMA5B//BEST1//KIT//MMP24//PRDM12//SCN1A// |
| GO:0010038 | response to metal ion | Biological process | 16 | 334 | 509 | 17653 | 1.66140018587579 | 0.0334427761346982 | 0.376632740796548 | 1.47569767830428 | 0.031434184675835 | KCNIP2//ALAD//SLC6A3//C1QA//HAAO//SLC30A6//ABCC8//ASCL1//CDKN1B//KIT//THBS1//HMOX1//EDN1//AQP1//NFATC4//LRRK2// |
| GO:0001952 | regulation of cell-matrix adhesion | Biological process | 7 | 106 | 509 | 17653 | 2.29030285057642 | 0.033698558193548 | 0.376632740796548 | 1.47238868019636 | 0.0137524557956778 | CDKN2A//NF2//THBS1//EMP2//SFRP1//DAPK3//ONECUT2// |
| GO:0006939 | smooth muscle contraction | Biological process | 7 | 106 | 509 | 17653 | 2.29030285057642 | 0.033698558193548 | 0.376632740796548 | 1.47238868019636 | 0.0137524557956778 | CHRM1//CHRNA3//DAPK3//EDN1//P2RX2//KIT//PTAFR// |
| GO:0042107 | cytokine metabolic process | Biological process | 7 | 106 | 509 | 17653 | 2.29030285057642 | 0.033698558193548 | 0.376632740796548 | 1.47238868019636 | 0.0137524557956778 | FOXP3//THBS1//HMOX1//IL1B//NFKB1//TIRAP//PTAFR// |
| GO:0008154 | actin polymerization or depolymerization | Biological process | 11 | 203 | 509 | 17653 | 1.87930550582132 | 0.0337482538699849 | 0.376632740796548 | 1.47174869258845 | 0.0216110019646365 | MICAL2//BRK1//WAS//ARHGAP40//EPS8L2//CDC42EP1//LMOD1//ACTR3//ACTR2//ARFIP2//SPTBN2// |
| GO:0045665 | negative regulation of neuron differentiation | Biological process | 11 | 203 | 509 | 17653 | 1.87930550582132 | 0.0337482538699849 | 0.376632740796548 | 1.47174869258845 | 0.0216110019646365 | LRRK2//RTN4//RGMA//SEMA4G//SEMA7A//TLX2//NFATC4//ASCL1//MED1//CNTN2//DIXDC1// |
| GO:0048762 | mesenchymal cell differentiation | Biological process | 11 | 203 | 509 | 17653 | 1.87930550582132 | 0.0337482538699849 | 0.376632740796548 | 1.47174869258845 | 0.0216110019646365 | SEMA4G//SEMA7A//FGFR2//WNT11//HEYL//TIAM1//IL1B//SFRP1//EDN1//ALDH1A2//RTN4// |
| GO:0032409 | regulation of transporter activity | Biological process | 13 | 255 | 509 | 17653 | 1.7680881389884 | 0.0341772038903799 | 0.378839900378263 | 1.46626347071567 | 0.0255402750491159 | NDFIP1//SGK2//OXSR1//EHD3//PTAFR//ABCC8//SLMAP//DLG4//SHISA9//SHANK3//SCN3B//GLRX//KCNIP2// |
| GO:0031331 | positive regulation of cellular catabolic process | Biological process | 16 | 335 | 509 | 17653 | 1.65644078233586 | 0.0342279760483114 | 0.378839900378263 | 1.46561878058861 | 0.031434184675835 | LRRK2//PRKAA1//TP53INP2//TRIM21//ABHD5//LARP1//HMOX1//TRIB1//RNF217//RNF125//RNF114//IL1B//CAMKK2//CD81//TRIM67//PIP4K2C// |
| GO:0043372 | positive regulation of CD4-positive, alpha-beta T cell differentiation | Biological process | 3 | 25 | 509 | 17653 | 4.16180746561886 | 0.0342371942363228 | 0.378839900378263 | 1.46550183329427 | 0.00589390962671906 | FOXP3//IL4R//TNFSF4// |
| GO:0060384 | innervation | Biological process | 3 | 25 | 509 | 17653 | 4.16180746561886 | 0.0342371942363228 | 0.378839900378263 | 1.46550183329427 | 0.00589390962671906 | NPTX1//FBXO45//UNC13B// |
| GO:0071363 | cellular response to growth factor stimulus | Biological process | 28 | 674 | 509 | 17653 | 1.44078398908665 | 0.0343363224765691 | 0.378839900378263 | 1.46424622092987 | 0.0550098231827112 | TAB1//FERMT2//FSHB//SMAD1//PML//SKIL//FGFR3//FGFR2//GALNT3//POLR2E//PTBP1//NREP//DLX5//BMP10//RGMA//UBE2D3//THBS1//HTRA4//RASL11B//ONECUT2//SFRP1//IL1B//ITGA5//MED1//SPRY3//RAF1//EDN1//HEYL// |
| GO:0045621 | positive regulation of lymphocyte differentiation | Biological process | 6 | 84 | 509 | 17653 | 2.47726634858265 | 0.034338142678083 | 0.378839900378263 | 1.46422319917144 | 0.0117878192534381 | FOXP3//TNFSF4//ZMIZ1//RAG1//CD74//IL4R// |
| GO:1901863 | positive regulation of muscle tissue development | Biological process | 6 | 84 | 509 | 17653 | 2.47726634858265 | 0.034338142678083 | 0.378839900378263 | 1.46422319917144 | 0.0117878192534381 | PRKAA1//FGFR2//BMP10//PIM1//EDN1//DDX39B// |
| GO:0032268 | regulation of cellular protein metabolic process | Biological process | 96 | 2792 | 509 | 17653 | 1.19249497582202 | 0.0344226596568768 | 0.37915382289814 | 1.46315557707409 | 0.18860510805501 | CDKN1B//TAB1//LRRK2//RAF1//IL1B//KIT//PRKAA1//THBS1//PROK1//CD74//CD81//CCDC88C//PML//GPNMB//CAMKK2//RBM3//RBM8A//EIF4G2//TRIB1//LRRTM1//NF2//PAK2//CEP85//CCK//PARP16//CHRNA3//TIRAP//MAP3K9//DBNL//UCHL5//CASP7//BMP10//TTK//CNTN2//LARP1//RBMS3//PTAFR//DDX39B//DAPK3//CELF4//PURA//MEX3B//SMAD1//PARG//PAXBP1//FOXP3//UBXN2A//OGT//NDFIP1//CDKN2A//EMP2//WNT11//OXSR1//MAPKAP1//EDN1//NFKB1//RNF217//RNF125//RNF114//PPP1R2//PPP1R26//SMPD1//YWHAB//FGFR3//AQP1//RAG1//TNFSF14//S100A12//SPRY3//RNF149//FGFR2//TRIM5//TIAM1//SFRP1//UBXN2B//VLDLR//DAZL//COPS5//ITGA5//PAX5//PRDM12//POLR2E//NUP214//DLG4//SH3BP5//SIRT3//SEMA7A//TRIM21//ALAD//EFNA3//WDR91//BEX4//TRABD2B//KMT2E//TRIM67//GRIN2B// |
| GO:0070727 | cellular macromolecule localization | Biological process | 64 | 1772 | 509 | 17653 | 1.25261323269191 | 0.0346747808211063 | 0.380698372318164 | 1.45998627541646 | 0.12573673870334 | SPRN//PML//NUP214//U2AF1//DDX39B//SRSF9//RBM8A//YWHAB//GOLGA7B//EXOC4//KDELR3//GOSR2//TIMM23//TOMM34//TIMM22//VPS45//AP1S1//ARFIP2//RABL3//KCNIP3//MLPH//VPS26A//CD74//TLN1//PEX5//TOMM40L//REEP2//LRRK2//MED1//PTPRU//EMP2//WNT11//TTK//MIS12//SEPT7//MAPRE1//SYS1//DLG4//IL1B//TAF8//SKP1//WDR19//CD81//TULP4//BCR//TNKS2//CNTN2//FERMT2//PACSIN1//TIAM1//EHD3//PALM//SCN3B//SLMAP//TSPAN14//CHRM1//SHANK3//GRIPAP1//NF2//NUCB1//TP53INP2//PRKAA1//UBE2D3//RTN4// |
| GO:0043300 | regulation of leukocyte degranulation | Biological process | 4 | 43 | 509 | 17653 | 3.22620733768904 | 0.0347723078014306 | 0.380698372318164 | 1.45876648469304 | 0.00785854616895874 | HMOX1//IL4R//BCR//PTAFR// |
| GO:0048483 | autonomic nervous system development | Biological process | 4 | 43 | 509 | 17653 | 3.22620733768904 | 0.0347723078014306 | 0.380698372318164 | 1.45876648469304 | 0.00785854616895874 | PHOX2A//TLX2//ASCL1//FZD3// |
| GO:0007163 | establishment or maintenance of cell polarity | Biological process | 11 | 204 | 509 | 17653 | 1.87009322393004 | 0.0347880517658769 | 0.380698372318164 | 1.45856989235103 | 0.0216110019646365 | MARK1//JAM3//AQP1//RHOF//MAPKAP1//DLG4//WNT11//ACTR3//ACTR2//PARVA//PARD6B// |
| GO:0008219 | cell death | Biological process | 78 | 2216 | 509 | 17653 | 1.22074677466257 | 0.0349729124110604 | 0.381649850445852 | 1.45626819979297 | 0.153241650294695 | CCK//CD74//PRUNE2//DAPK3//FGFR2//FBXO10//KCNIP3//IL1B//MAP3K9//NFKB1//PAK2//PIM1//DRAM1//RTN4//RAF1//UBE2Z//TIAM1//UBE2D3//KREMEN1//CASP7//OGT//TNFSF14//UNC5A//ARHGEF6//PML//CDKN1B//MAEA//COPS5//FGFR3//AQP1//KIT//ASCL1//PAFAH2//MED1//PRKAA1//LIMS2//SFRP1//THBS1//WNT11//UBE4B//HIC1//HMOX1//NFATC4//PAX8//SGK2//BMP10//NF2//TNFAIP8L2//RELT//OXSR1//EDN1//CDKN2A//UNC13B//ARHGEF4//SMPD1//TGM2//FOSL1//ALDH1A2//LRRK2//RAG1//MYBL2//PARP16//KRT2//KRT9//PRSS8//KRT38//FZD3//YWHAB//SLC25A5//GPNMB//REL//ELK1//GRIN2B//SKIL//ITGA5//CTH//EIF4G2//EMP2// |
| GO:0046888 | negative regulation of hormone secretion | Biological process | 5 | 63 | 509 | 17653 | 2.75251816509184 | 0.0349878614928919 | 0.381649850445852 | 1.45608260139303 | 0.00982318271119843 | SFRP1//ABCC8//VSNL1//IL1B//EDN1// |
| GO:0007249 | I-kappaB kinase/NF-kappaB signaling | Biological process | 13 | 256 | 509 | 17653 | 1.76118154469548 | 0.0351029161964143 | 0.381673669190273 | 1.45465680275348 | 0.0255402750491159 | IL1B//TIRAP//CTH//HMOX1//CC2D1A//REL//S100A12//TGM2//NDFIP1//TRIM5//CD74//TAB1//NFKB1// |
| GO:0021700 | developmental maturation | Biological process | 13 | 256 | 509 | 17653 | 1.76118154469548 | 0.0351029161964143 | 0.381673669190273 | 1.45465680275348 | 0.0255402750491159 | DAZL//LRRK2//UNC13B//DLG4//ALDH1A2//MAEA//KCNIP2//CNTN2//ASCL1//PALM//FGFR3//SNX19//NFATC4// |
| GO:0051174 | regulation of phosphorus metabolic process | Biological process | 65 | 1806 | 509 | 17653 | 1.24823498184397 | 0.0355596685892661 | 0.386019323706592 | 1.44904229524186 | 0.12770137524558 | CDKN1B//TAB1//LRRK2//RAF1//IL1B//KIT//PRKAA1//THBS1//PROK1//CD74//CD81//CCDC88C//PML//GPNMB//CAMKK2//NUP214//OGT//TRIB1//LRRTM1//NF2//PAK2//CEP85//PARP16//CHRNA3//GRM2//PALM//TIRAP//MAP3K9//DBNL//DLAT//BMP10//TTK//PPP1R26//FZD2//CDKN2A//EMP2//WNT11//OXSR1//MAPKAP1//PPP1R2//FGFR3//FGFR2//SMPD1//YWHAB//EDN1//S100A12//SPRY3//RNF149//TRIM5//TIAM1//SFRP1//NRBF2//WDR91//PIK3IP1//UBXN2B//UBXN2A//VLDLR//COPS5//ITGA5//CCK//PTAFR//DLG4//SH3BP5//SIRT3//SEMA7A// |
| GO:0040017 | positive regulation of locomotion | Biological process | 23 | 532 | 509 | 17653 | 1.4993980530895 | 0.035622718826218 | 0.386084050355308 | 1.44827293702991 | 0.0451866404715128 | EDN1//THBS1//RTN4//TNFSF14//GPNMB//DAPK3//MAPRE1//FSHB//IL1B//ITGA5//KIT//SEMA4G//TIAM1//WNT11//FERMT3//SEMA7A//ONECUT2//TIRAP//CD74//HMOX1//TAC4//PTAFR//CXCL14// |
| GO:2001236 | regulation of extrinsic apoptotic signaling pathway | Biological process | 9 | 155 | 509 | 17653 | 2.01377780594461 | 0.0358522754617334 | 0.387466923290273 | 1.44548327544628 | 0.0176817288801572 | HMOX1//RAF1//SFRP1//SKIL//THBS1//PAK2//PML//IL1B//DAPK3// |
| GO:0010506 | regulation of autophagy | Biological process | 15 | 310 | 509 | 17653 | 1.67814817162051 | 0.0358648963447746 | 0.387466923290273 | 1.4453304200065 | 0.0294695481335953 | EIF4G2//LRRK2//PRKAA1//TP53INP2//TRIM21//LARP1//HMOX1//ATP6V1G2//PRKAB1//EXOC4//VPS26A//CAMKK2//PIP4K2C//DAPK3//DRAM1// |
| GO:0031334 | positive regulation of protein complex assembly | Biological process | 13 | 257 | 509 | 17653 | 1.75432869821807 | 0.0360464646655723 | 0.388082995741527 | 1.44313732321983 | 0.0255402750491159 | UNC13B//CDC42EP1//LMOD1//CDKN1B//MAPRE1//TRABD2B//CCK//ACTR3//ACTR2//ARFIP2//WAS//FOSL1//BRK1// |
| GO:0007215 | glutamate receptor signaling pathway | Biological process | 6 | 85 | 509 | 17653 | 2.44812203859933 | 0.0360844230202606 | 0.388082995741527 | 1.44268023455688 | 0.0117878192534381 | GRM2//GRIN2B//TIAM1//SHANK3//DLG4//SHISA9// |
| GO:0009894 | regulation of catabolic process | Biological process | 36 | 914 | 509 | 17653 | 1.3660199558924 | 0.036146478707624 | 0.388082995741527 | 1.44193400410485 | 0.0707269155206287 | NUP214//OGT//UCHL5//LRRK2//DAPK3//DRAM1//PIP4K2C//EIF4G2//PRKAA1//TP53INP2//TRIM21//ABHD5//LARP1//HMOX1//ATP6V1G2//PRKAB1//EXOC4//VPS26A//TRIB1//RNF217//RNF125//RNF114//UBXN2A//CARHSP1//YWHAB//CDKN1B//NDFIP1//IL1B//TIRAP//DAZL//ALAD//WDR91//CAMKK2//CD81//PML//TRIM67// |
| GO:1903362 | regulation of cellular protein catabolic process | Biological process | 12 | 231 | 509 | 17653 | 1.80164825351466 | 0.0361514545789091 | 0.388082995741527 | 1.44187422387748 | 0.0235756385068762 | UCHL5//OGT//TRIB1//LRRK2//RNF217//RNF125//RNF114//ALAD//CD81//PML//TRIM67//WDR91// |
| GO:0019218 | regulation of steroid metabolic process | Biological process | 7 | 108 | 509 | 17653 | 2.247889834825 | 0.0367470261889457 | 0.392356652456552 | 1.43477780112369 | 0.0137524557956778 | INSIG2//NFKB1//IDI1//NFYA//PRKAA1//IL1B//KIT// |
| GO:0043279 | response to alkaloid | Biological process | 7 | 108 | 509 | 17653 | 2.247889834825 | 0.0367470261889457 | 0.392356652456552 | 1.43477780112369 | 0.0137524557956778 | PRKAA1//SLC6A3//SMPD1//TIAM1//GNAO1//CASP7//SRSF9// |
| GO:0009896 | positive regulation of catabolic process | Biological process | 18 | 393 | 509 | 17653 | 1.5884761319156 | 0.0367489904723372 | 0.392356652456552 | 1.43475458687408 | 0.0353634577603143 | LRRK2//PRKAA1//TP53INP2//TRIM21//ABHD5//LARP1//HMOX1//TRIB1//RNF217//RNF125//RNF114//CDKN1B//NDFIP1//IL1B//CAMKK2//CD81//TRIM67//PIP4K2C// |
| GO:0019915 | lipid storage | Biological process | 5 | 64 | 509 | 17653 | 2.70951006876228 | 0.0370893614006222 | 0.392356652456552 | 1.43075064422797 | 0.00982318271119843 | MEST//NFKB1//ABHD5//IL1B//NRIP1// |
| GO:0046686 | response to cadmium ion | Biological process | 5 | 64 | 509 | 17653 | 2.70951006876228 | 0.0370893614006222 | 0.392356652456552 | 1.43075064422797 | 0.00982318271119843 | HMOX1//CDKN1B//ALAD//HAAO//KIT// |
| GO:0045619 | regulation of lymphocyte differentiation | Biological process | 9 | 156 | 509 | 17653 | 2.00086897385522 | 0.0371212343578527 | 0.392356652456552 | 1.43037759102292 | 0.0176817288801572 | FOXP3//TNFSF4//HMGB3//SFRP1//CD74//ZMIZ1//RAG1//IL4R//NDFIP1// |
| GO:0030155 | regulation of cell adhesion | Biological process | 27 | 650 | 509 | 17653 | 1.44062566117576 | 0.0372727266684189 | 0.392356652456552 | 1.42860883547977 | 0.0530451866404715 | ONECUT2//CDKN2A//NF2//THBS1//EMP2//SFRP1//ARHGDIB//ITGA5//DPP4//PAK2//TNFSF14//FOXP3//FERMT3//IL1B//CD209//TNFSF4//GPNMB//NDFIP1//CD74//ZMIZ1//RAG1//IL4R//TGM2//TNFAIP8L2//DAPK3//PTAFR//PML// |
| GO:0008064 | regulation of actin polymerization or depolymerization | Biological process | 10 | 181 | 509 | 17653 | 1.91611761768824 | 0.0373218331306008 | 0.392356652456552 | 1.42803703322798 | 0.0196463654223969 | ARHGAP40//EPS8L2//CDC42EP1//LMOD1//ACTR3//ACTR2//ARFIP2//WAS//SPTBN2//BRK1// |
| GO:0006775 | fat-soluble vitamin metabolic process | Biological process | 4 | 44 | 509 | 17653 | 3.15288444365065 | 0.0374091237765705 | 0.392356652456552 | 1.42702246404069 | 0.00785854616895874 | ALDH1A2//NFKB1//CYP4F2//IL1B// |
| GO:0015701 | bicarbonate transport | Biological process | 4 | 44 | 509 | 17653 | 3.15288444365065 | 0.0374091237765705 | 0.392356652456552 | 1.42702246404069 | 0.00785854616895874 | SLC26A1//AQP1//BEST1//CA7// |
| GO:0009582 | detection of abiotic stimulus | Biological process | 8 | 132 | 509 | 17653 | 2.10192296243377 | 0.0374553048078743 | 0.392356652456552 | 1.42648666432948 | 0.0157170923379175 | ANO3//RGS9BP//SEMA5B//BEST1//KIT//MMP24//PRDM12//SCN1A// |
| GO:0051656 | establishment of organelle localization | Biological process | 20 | 450 | 509 | 17653 | 1.54141017245143 | 0.0376490557589977 | 0.392356652456552 | 1.42424591146326 | 0.0392927308447937 | EXOC4//UNC13B//CPLX1//SYT2//SYT9//SYT14//CPLX3//SNPH//ACTR3//ACTR2//MLPH//MYO19//KIT//HMOX1//IL4R//DYNC1I1//GOSR2//MIS12//TTL//LRRK2// |
| GO:1901655 | cellular response to ketone | Biological process | 6 | 86 | 509 | 17653 | 2.41965550326678 | 0.0378867221531565 | 0.392356652456552 | 1.42151296697034 | 0.0117878192534381 | PRKAA1//SFRP1//TNFSF4//ELK1//AQP1//LARP1// |
| GO:0006829 | zinc ion transport | Biological process | 3 | 26 | 509 | 17653 | 4.00173794771044 | 0.0378981659089899 | 0.392356652456552 | 1.42138180731106 | 0.00589390962671906 | SLC39A2//SLC30A6//SLC39A8// |
| GO:0048169 | regulation of long-term neuronal synaptic plasticity | Biological process | 3 | 26 | 509 | 17653 | 4.00173794771044 | 0.0378981659089899 | 0.392356652456552 | 1.42138180731106 | 0.00589390962671906 | KIT//SHANK3//DLG4// |
| GO:0060765 | regulation of androgen receptor signaling pathway | Biological process | 3 | 26 | 509 | 17653 | 4.00173794771044 | 0.0378981659089899 | 0.392356652456552 | 1.42138180731106 | 0.00589390962671906 | HEYL//SFRP1//TRIM68// |
| GO:0007584 | response to nutrient | Biological process | 11 | 207 | 509 | 17653 | 1.84299042358323 | 0.0380426233604925 | 0.392356652456552 | 1.41972954242594 | 0.0216110019646365 | ALAD//HMOX1//ALDH1A2//P2RX2//IL1B//ASCL1//PIM1//MED1//SFRP1//SLC27A4//OGT// |
| GO:0006353 | DNA-templated transcription, termination | Biological process | 7 | 109 | 509 | 17653 | 2.22726699230367 | 0.0383372239887427 | 0.392356652456552 | 1.41637933772866 | 0.0137524557956778 | POLR2E//LSM11//SNRPB//U2AF1//DDX39B//SRSF9//RBM8A// |
| GO:0051271 | negative regulation of cellular component movement | Biological process | 16 | 340 | 509 | 17653 | 1.63208135906622 | 0.0383573281266532 | 0.392356652456552 | 1.41615165229033 | 0.031434184675835 | HMOX1//BCR//VASH1//BMP10//THBS1//TRIB1//PTPRU//NF2//SFRP1//WNT11//SEMA4G//SEMA7A//ABCC8//ARHGDIB//WAS//CDKN1B// |
| GO:0015791 | polyol transport | Biological process | 2 | 11 | 509 | 17653 | 6.3057688873013 | 0.0384147227295189 | 0.392356652456552 | 1.41550229712315 | 0.00392927308447937 | AQP1//SLC5A3// |
| GO:0016188 | synaptic vesicle maturation | Biological process | 2 | 11 | 509 | 17653 | 6.3057688873013 | 0.0384147227295189 | 0.392356652456552 | 1.41550229712315 | 0.00392927308447937 | UNC13B//DLG4// |
| GO:0042368 | vitamin D biosynthetic process | Biological process | 2 | 11 | 509 | 17653 | 6.3057688873013 | 0.0384147227295189 | 0.392356652456552 | 1.41550229712315 | 0.00392927308447937 | NFKB1//IL1B// |
| GO:0043301 | negative regulation of leukocyte degranulation | Biological process | 2 | 11 | 509 | 17653 | 6.3057688873013 | 0.0384147227295189 | 0.392356652456552 | 1.41550229712315 | 0.00392927308447937 | HMOX1//BCR// |
| GO:0044650 | adhesion of symbiont to host cell | Biological process | 2 | 11 | 509 | 17653 | 6.3057688873013 | 0.0384147227295189 | 0.392356652456552 | 1.41550229712315 | 0.00392927308447937 | CD209//CD81// |
| GO:0045073 | regulation of chemokine biosynthetic process | Biological process | 2 | 11 | 509 | 17653 | 6.3057688873013 | 0.0384147227295189 | 0.392356652456552 | 1.41550229712315 | 0.00392927308447937 | HMOX1//IL1B// |
| GO:0045472 | response to ether | Biological process | 2 | 11 | 509 | 17653 | 6.3057688873013 | 0.0384147227295189 | 0.392356652456552 | 1.41550229712315 | 0.00392927308447937 | LARP1//NFATC4// |
| GO:0045628 | regulation of T-helper 2 cell differentiation | Biological process | 2 | 11 | 509 | 17653 | 6.3057688873013 | 0.0384147227295189 | 0.392356652456552 | 1.41550229712315 | 0.00392927308447937 | IL4R//TNFSF4// |
| GO:0046689 | response to mercury ion | Biological process | 2 | 11 | 509 | 17653 | 6.3057688873013 | 0.0384147227295189 | 0.392356652456552 | 1.41550229712315 | 0.00392927308447937 | AQP1//ALAD// |
| GO:0060525 | prostate glandular acinus development | Biological process | 2 | 11 | 509 | 17653 | 6.3057688873013 | 0.0384147227295189 | 0.392356652456552 | 1.41550229712315 | 0.00392927308447937 | FGFR2//SFRP1// |
| GO:0061081 | positive regulation of myeloid leukocyte cytokine production involved in immune response | Biological process | 2 | 11 | 509 | 17653 | 6.3057688873013 | 0.0384147227295189 | 0.392356652456552 | 1.41550229712315 | 0.00392927308447937 | SEMA7A//CD74// |
| GO:0099515 | actin filament-based transport | Biological process | 2 | 11 | 509 | 17653 | 6.3057688873013 | 0.0384147227295189 | 0.392356652456552 | 1.41550229712315 | 0.00392927308447937 | MLPH//MYO19// |
| GO:1902563 | regulation of neutrophil activation | Biological process | 2 | 11 | 509 | 17653 | 6.3057688873013 | 0.0384147227295189 | 0.392356652456552 | 1.41550229712315 | 0.00392927308447937 | BCR//PTAFR// |
| GO:0030832 | regulation of actin filament length | Biological process | 10 | 182 | 509 | 17653 | 1.90558949890973 | 0.0385220785496304 | 0.392356652456552 | 1.41429028753191 | 0.0196463654223969 | BRK1//WAS//ARHGAP40//EPS8L2//CDC42EP1//LMOD1//ACTR3//ACTR2//ARFIP2//SPTBN2// |
| GO:0048839 | inner ear development | Biological process | 10 | 182 | 509 | 17653 | 1.90558949890973 | 0.0385220785496304 | 0.392356652456552 | 1.41429028753191 | 0.0196463654223969 | FGFR2//DLX5//INSIG2//BCR//PAX8//FZD3//KCNQ4//FZD2//CDKN1B//CXCL14// |
| GO:0045927 | positive regulation of growth | Biological process | 13 | 260 | 509 | 17653 | 1.73408644400786 | 0.0389856947480104 | 0.396481584332022 | 1.40909472199124 | 0.0255402750491159 | EIF4G2//SFRP1//FOXS1//SLC6A3//SEMA7A//CACNA2D2//RTN4//FGFR2//BMP10//PIM1//EDN1//DDX39B//SYT2// |
| GO:0009987 | cellular process | Biological process | 477 | 16158 | 509 | 17653 | 1.02383863571203 | 0.0391595361531751 | 0.397194105151696 | 1.40716246108007 | 0.93713163064833 | CYP4F2//UBXN2B//UBXN2A//TP53INP2//SLC25A2//DDAH1//SLC25A5//BEX4//CEP85//MIS12//KLHL22//CDKN1B//CDKN2A//USP37//MAPRE1//SKP1//SEPT7//NFS1//CTH//CUX1//EDN1//FGFR2//FOXS1//SATB2//ZNF281//DNAJB5//HEYL//KCNIP3//HIC1//ASCL1//NFKB1//PAX5//FOXP3//RLIM//MED1//CC2D1A//REL//SKIL//UBE2D3//VLDLR//NRIP1//BHLHE40//PAXBP1//WAS//THUMPD1//CAMKK2//LRRK2//DLG4//FGFR3//GRIN2B//IL1B//KIT//SMAD1//NCAM1//MAPK4//RAF1//SPTBN2//YWHAB//SHANK3//RBM8A//TAB1//PRKAA1//THBS1//PROK1//CD74//CD81//UBE4B//RNF217//MGRN1//FBXL3//FBXO10//RNF125//RNF114//TRIM21//UBE2E1//TNKS2//FBXL20//TTL//KIF2A//MARK1//MAP7D1//CNTN2//DDX39B//MYBL2//COPS5//CELF4//RBFOX1//MBNL3//PTBP1//SNRPB//PPIH//NOVA1//POLR2E//SNRPA//U2AF1//SRSF9//WDR19//PACSIN1//RHOF//SLC6A3//FTSJ1//PAX8//DAZL//SGK2//HTRA4//ZMIZ1//ST6GALNAC6//GPNMB//DLX5//SFRP1//WNT11//SEMA7A//SLC27A4//ABCC8//RPP14//SEMA4G//FBXO45//FZD3//CCK//EMP2//PLLP//EHD3//CCDC88C//PML//ALDH1A2//VASH1//NF2//AGPAT1//OTP//SNX19//ONECUT2//LRRTM1//URM1//TRIM5//CD209//PAK2//JAM3//RAG1//S100A12//LGALS3BP//TLN1//FERMT3//HMOX1//BCR//TIRAP//NDFIP1//EFNA3//FGF11//FSHB//TAC4//NDP//C1QA//CXCL14//P2RX2//KRT2//CCDC103//PHOX2A//ARID3B//PRDM4//ELK1//RAX//TLX2//NFATC4//NFYA//HIVEP3//RFX4//HLTF//FOSL1//CREB3L3//CREB5//LSM11//LIN9//NUP214//TOMM34//GNAO1//GNAZ//PFDN1//RPAP3//MICAL2//ARFIP2//IQSEC3//SH3GL1//MAEA//PALM//CCDC6//PRUNE2//DAPK3//MAP3K9//PIM1//DRAM1//RTN4//UBE2Z//TIAM1//KREMEN1//CASP7//OGT//TNFSF14//UNC5A//ARHGEF6//FERMT2//CTNND2//TRABD2B//CALCOCO1//VPS26A//FZD2//PPP3CB//FAM13A//EBP//RALBP1//PTPRT//AKAP10//CHRM1//CHRNA3//DGKG//HTR3E//KCNIP2//ARHGAP40//IL4R//IMPA2//MRC1//NRGN//PRKAB1//RASL11B//SMPD1//TNFSF4//RERGL//CHRFAM7A//SH3BP5//UNC13B//CARHSP1//ARHGEF4//POLR2M//PLA2G4C//OXSR1//SYT2//SYT9//SYT14//EXOC4//RNF38//RNF149//DCAF16//TULP4//KLHL14//PEX5//RNF123//TRIM7//RNF135//PPP1R2//PGM5//PPP1R3F//SLC5A3//MOGAT2//CYP2C18//CYP2S1//DLAT//GLRX//HAAO//GPD2//CTNS//SLC23A2//PRTFDC1//AQP1//UCK2//UCK1//DPYS//TET3//PURA//MCIDAS//UCHL5//USP45//PARG//KMT2E//HMGB3//ASF1B//ZNF618//EWSR1//FOXG1//ZBTB43//CRTC1//ATP1B4//MYCBP//HOXC6//HOXC9//FOXI2//CREBZF//PRDM12//GPBP1L1//RSC1A1//TCF19//TCF20//ZNF322//KRBOX1//NRBF2//PHF20L1//MED6//PTAFR//TEAD1//C2ORF49//RBM3//EIF4G2//LARP1//GLYCTK//NEK7//MEX3B//AATK//TRIB1//PTPRU//PTPN18//PARP16//SIRT3//ST8SIA5//EDEM3//MGAT5//MAN1C1//GAL3ST1//GALNT10//SPRN//NRN1//VNN2//PGGT1B//PGAP3//PYURF//USP46//UROC1//KDELR3//TIMM23//TIMM22//ABHD5//DBI//ACBD6//INSIG2//LCLAT1//PCTP//PIP4K2C//ESYT1//CYP4F3//CA7//SLC5A6//SLC52A2//ALAD//SYPL2//LETM2//SLC39A8//SLC12A7//CPLX1//LGI3//VPS45//BIN2//MYOM3//LMOD1//BRK1//CGNL1//DYNC1I1//MYO19//FAM160A2//PARD6B//ITGA5//TTC28//DIXDC1//RASSF1//TTK//CENPO//CHTF8//ARHGDIB//ACTR3//ACTR2//PARVA//CLEC1A//TSPAN14//TSPAN18//TPRA1//GPR160//GPR34//GRM2//GPR3//EVC//DBNL//RALGPS2//RALGDS//CDC42EP1//EPS8L2//NPTX1//NPTX2//BSN//SNPH//LRRN1//CACNA2D2//DPP4//PAFAH2//LIMS2//LDOC1//DEC1//ATP6V1G2//GALNT3//IDI1//UBXN10//SPRY3//RGS9BP//MMP24//KCNJ12//BMP10//PPP1R26//ZNF804A//TRIM67//PIK3IP1//HS6ST1//PABPC3//CPLX3//RBMS3//ST3GAL3//TNFAIP8L2//RELT//AVL9//TAF8//PASD1//USP54//NREP//TGM4//TGM2//GOLGA7B//SCN1A//SCN7A//CHDH//FAM92B//MLPH//TOMM40L//MEA1//CD79A//ALDOC//RGMA//MAPKAP1//EXD1//NPTXR//TBC1D24//KRT9//KRTAP1-1//KRT38//NPHS2//RABL3//REEP2//WDFY1//GPX8//DNAJB12//VSNL1//ASNA1//GOSR2//PRIMA1//KCTD18//KCNA6//SHKBP1//SCAMP1//WDR91//TGOLN2//NUCB1//STEAP2//FAM57B//ENHO//SHISA9//SEMA5B//C11ORF88//SULT4A1//SLC26A1//RALGAPB//SLC9A5//TRIM68//SCN3B//SLC30A6//ANO4//ANO3//PRSS8//KCNQ5//KCNQ4//GRIPAP1//LYPD6//SLMAP//ZSWIM6//FAM168A// |
| GO:0051048 | negative regulation of secretion | Biological process | 11 | 208 | 509 | 17653 | 1.83412989270062 | 0.039173217231433 | 0.397194105151696 | 1.40701075910108 | 0.0216110019646365 | CD74//CYP4F2//HMOX1//BCR//SFRP1//ABCC8//VSNL1//EDN1//FOXP3//PML//IL1B// |
| GO:1901616 | organic hydroxy compound catabolic process | Biological process | 5 | 65 | 509 | 17653 | 2.66782529847363 | 0.0392667298876583 | 0.397546248847654 | 1.40597526503669 | 0.00982318271119843 | GPD2//CYP4F3//CYP4F2//SLC6A3//IMPA2// |
| GO:0002690 | positive regulation of leukocyte chemotaxis | Biological process | 6 | 87 | 509 | 17653 | 2.39184337104532 | 0.0397454831343742 | 0.400477823610945 | 1.4007122194925 | 0.0117878192534381 | THBS1//TNFSF14//TIRAP//EDN1//CD74//CXCL14// |
| GO:0048644 | muscle organ morphogenesis | Biological process | 6 | 87 | 509 | 17653 | 2.39184337104532 | 0.0397454831343742 | 0.400477823610945 | 1.4007122194925 | 0.0117878192534381 | FZD2//UBE4B//MED1//MYOM3//BMP10//FGFR2// |
| GO:1901654 | response to ketone | Biological process | 10 | 183 | 509 | 17653 | 1.89517644153864 | 0.0397487001305697 | 0.400477823610945 | 1.40067706915471 | 0.0196463654223969 | THBS1//FOSL1//EDN1//PRKAA1//SFRP1//TNFSF4//ELK1//PTAFR//AQP1//LARP1// |
| GO:0034097 | response to cytokine | Biological process | 44 | 1164 | 509 | 17653 | 1.31099318790972 | 0.0397931535511689 | 0.400477823610945 | 1.40019164226599 | 0.0864440078585462 | AGPAT1//CD74//HMOX1//LRRTM1//IL1B//IL4R//KIT//PIM1//PTAFR//TNFSF4//RELT//TNFSF14//TRIM21//PAK2//NCAM1//PML//TRIM68//TRIM5//MED1//EDN1//TAB1//NFKB1//SKP1//PTPN18//ACTR3//ACTR2//DAPK3//MRC1//WAS//SFRP1//ALAD//THBS1//CTH//UBXN2A//SLC25A5//MYBL2//PARP16//GNAO1//PGGT1B//PPP3CB//REL//SKIL//FOSL1//ALDH1A2// |
| GO:0006690 | icosanoid metabolic process | Biological process | 7 | 110 | 509 | 17653 | 2.20701911055546 | 0.0399719025381823 | 0.400570339071687 | 1.39824518018197 | 0.0137524557956778 | EDN1//CD74//CYP4F3//CYP4F2//PLA2G4C//CYP2C18//CYP2S1// |
| GO:0007613 | memory | Biological process | 7 | 110 | 509 | 17653 | 2.20701911055546 | 0.0399719025381823 | 0.400570339071687 | 1.39824518018197 | 0.0137524557956778 | CTNS//CRTC1//ITGA5//PPP3CB//ABCC8//VLDLR//SHANK3// |
| GO:0010817 | regulation of hormone levels | Biological process | 22 | 510 | 509 | 17653 | 1.49607457914403 | 0.0400562745116681 | 0.400570339071687 | 1.39732944531361 | 0.0432220039292731 | MED1//FSHB//SYT9//DPP4//SLC25A5//IL1B//PPP3CB//ABCC8//CACNA2D2//NFKB1//CHRNA3//EDN1//CPLX1//SNX19//CPLX3//SIRT3//RAF1//VSNL1//ALDH1A2//SFRP1//TIAM1//PAX8// |
| GO:0002886 | regulation of myeloid leukocyte mediated immunity | Biological process | 4 | 45 | 509 | 17653 | 3.08282034490286 | 0.0401577243664947 | 0.400570339071687 | 1.39623090550138 | 0.00785854616895874 | HMOX1//IL4R//BCR//PTAFR// |
| GO:0031103 | axon regeneration | Biological process | 4 | 45 | 509 | 17653 | 3.08282034490286 | 0.0401577243664947 | 0.400570339071687 | 1.39623090550138 | 0.00785854616895874 | RGMA//RTN4//JAM3//NREP// |
| GO:0043551 | regulation of phosphatidylinositol 3-kinase activity | Biological process | 4 | 45 | 509 | 17653 | 3.08282034490286 | 0.0401577243664947 | 0.400570339071687 | 1.39623090550138 | 0.00785854616895874 | FGFR3//KIT//PIK3IP1//WDR91// |
| GO:0016477 | cell migration | Biological process | 54 | 1474 | 509 | 17653 | 1.27056537281444 | 0.0402977533540259 | 0.401075804896086 | 1.39471916559952 | 0.106090373280943 | SEMA4G//SEMA7A//FBXO45//SATB2//MARK1//ASCL1//FZD3//CCK//JAM3//S100A12//KIT//HMOX1//BCR//EMP2//EDN1//THBS1//VASH1//BMP10//RTN4//PML//TNFSF14//TRIB1//DIXDC1//CNTN2//LRRK2//KIF2A//RHOF//BEX4//PARD6B//GPNMB//DAPK3//MAPRE1//FSHB//IL1B//ITGA5//TIAM1//WNT11//FERMT3//ONECUT2//PTPRU//NF2//SFRP1//DPP4//CD74//KRT2//BIN2//CXCL14//ABCC8//PARVA//TIRAP//ARHGDIB//PTAFR//AVL9//PAK2// |
| GO:0007160 | cell-matrix adhesion | Biological process | 11 | 209 | 509 | 17653 | 1.82535415158722 | 0.0403270068504123 | 0.401075804896086 | 1.39440401098492 | 0.0216110019646365 | ONECUT2//CDKN2A//NF2//THBS1//EMP2//SFRP1//FERMT2//DAPK3//CD96//TIAM1//JAM3// |
| GO:0045666 | positive regulation of neuron differentiation | Biological process | 16 | 343 | 509 | 17653 | 1.61780659499275 | 0.0410016187784806 | 0.407186413801563 | 1.38719899662945 | 0.031434184675835 | TRIM67//ZNF804A//SEMA7A//RTN4//SKIL//TIAM1//CUX1//SHANK3//ACTR2//DLG4//CRTC1//PACSIN1//VLDLR//SYT2//HEYL//ASCL1// |
| GO:0019058 | viral life cycle | Biological process | 15 | 316 | 509 | 17653 | 1.64628459874164 | 0.0412787186397666 | 0.409081210314325 | 1.38427379239625 | 0.0294695481335953 | CD209//DPP4//ITGA5//MRC1//NCAM1//SLC52A2//CD81//PPIH//LARP1//TRIM5//TRIM21//TMPRSS2//CD74//NUP214//PML// |
| GO:0032103 | positive regulation of response to external stimulus | Biological process | 14 | 289 | 509 | 17653 | 1.68008375197993 | 0.0413133914896768 | 0.409081210314325 | 1.38390915155467 | 0.0275049115913556 | CREB3L3//THBS1//TNFSF14//IL1B//RTN4//S100A12//TGM2//TNFSF4//TIRAP//EDN1//CD74//UNC13B//TIAM1//CXCL14// |
| GO:0002688 | regulation of leukocyte chemotaxis | Biological process | 7 | 111 | 509 | 17653 | 2.18713605550541 | 0.0416514138092296 | 0.409214125903123 | 1.38037025238142 | 0.0137524557956778 | THBS1//TNFSF14//JAM3//TIRAP//EDN1//CD74//CXCL14// |
| GO:0030593 | neutrophil chemotaxis | Biological process | 6 | 88 | 509 | 17653 | 2.36466333273799 | 0.0416611123850046 | 0.409214125903123 | 1.38026913822362 | 0.0117878192534381 | JAM3//TIRAP//EDN1//CD74//IL1B//S100A12// |
| GO:0110020 | regulation of actomyosin structure organization | Biological process | 6 | 88 | 509 | 17653 | 2.36466333273799 | 0.0416611123850046 | 0.409214125903123 | 1.38026913822362 | 0.0117878192534381 | WAS//NF2//SFRP1//WNT11//EDN1//BMP10// |
| GO:0019220 | regulation of phosphate metabolic process | Biological process | 64 | 1791 | 509 | 17653 | 1.23932476176999 | 0.0416921291050926 | 0.409214125903123 | 1.37994592605425 | 0.12573673870334 | CDKN1B//TAB1//LRRK2//RAF1//IL1B//KIT//PRKAA1//THBS1//PROK1//CD74//CD81//CCDC88C//PML//GPNMB//CAMKK2//NUP214//OGT//TRIB1//LRRTM1//NF2//PAK2//CEP85//PARP16//CHRNA3//GRM2//PALM//TIRAP//MAP3K9//DBNL//BMP10//TTK//PPP1R26//FZD2//CDKN2A//EMP2//WNT11//OXSR1//MAPKAP1//PPP1R2//FGFR3//FGFR2//SMPD1//YWHAB//EDN1//S100A12//SPRY3//RNF149//TRIM5//TIAM1//SFRP1//NRBF2//WDR91//PIK3IP1//UBXN2B//UBXN2A//VLDLR//COPS5//ITGA5//CCK//PTAFR//DLG4//SH3BP5//SIRT3//SEMA7A// |
| GO:0007628 | adult walking behavior | Biological process | 3 | 27 | 509 | 17653 | 3.85352543112857 | 0.0417503691960897 | 0.409214125903123 | 1.37933967972252 | 0.00589390962671906 | CTNS//SCN1A//CNTN2// |
| GO:0032673 | regulation of interleukin-4 production | Biological process | 3 | 27 | 509 | 17653 | 3.85352543112857 | 0.0417503691960897 | 0.409214125903123 | 1.37933967972252 | 0.00589390962671906 | FOXP3//NDFIP1//TNFSF4// |
| GO:1902692 | regulation of neuroblast proliferation | Biological process | 3 | 27 | 509 | 17653 | 3.85352543112857 | 0.0417503691960897 | 0.409214125903123 | 1.37933967972252 | 0.00589390962671906 | OTP//FZD3//LRRK2// |
| GO:0001961 | positive regulation of cytokine-mediated signaling pathway | Biological process | 4 | 46 | 509 | 17653 | 3.01580251131801 | 0.04301819257227 | 0.421030443366515 | 1.36634784063334 | 0.00785854616895874 | MED1//EDN1//AGPAT1//CD74// |
| GO:1903038 | negative regulation of leukocyte cell-cell adhesion | Biological process | 7 | 112 | 509 | 17653 | 2.16760805500982 | 0.0433760880867511 | 0.421958023081912 | 1.36274961781156 | 0.0137524557956778 | GPNMB//FOXP3//NDFIP1//CD74//TNFSF4//IL4R//TNFAIP8L2// |
| GO:0031323 | regulation of cellular metabolic process | Biological process | 205 | 6454 | 509 | 17653 | 1.10160434156062 | 0.0434397013453038 | 0.421958023081912 | 1.36211316925099 | 0.402750491159136 | CDKN1B//CUX1//EDN1//FGFR2//FOXS1//SATB2//ZNF281//DNAJB5//HEYL//KCNIP3//HIC1//ASCL1//NFKB1//PAX5//FOXP3//RLIM//MED1//CC2D1A//REL//SKIL//UBE2D3//VLDLR//NRIP1//BHLHE40//PAXBP1//TAB1//LRRK2//RAF1//IL1B//KIT//PRKAA1//THBS1//PROK1//CD74//CD81//RBFOX1//MBNL3//CELF4//PTBP1//RBM8A//CCDC88C//PML//GPNMB//CAMKK2//DLG4//LRRTM1//PPP1R3F//NUP214//OGT//MCIDAS//USP37//PARG//KRBOX1//ZNF618//CTNND2//DAPK3//EWSR1//ZBTB43//ATP1B4//MYCBP//NRBF2//HMGB3//MAP3K9//NFYA//PHF20L1//UCHL5//PFDN1//ASF1B//HIVEP3//GPBP1L1//RSC1A1//HLTF//TCF20//ZNF322//MED6//ELK1//FOXG1//OTP//CARHSP1//LIN9//HOXC6//HOXC9//FOXI2//MYBL2//PTAFR//PURA//CREBZF//PRDM12//RFX4//TCF19//FOSL1//CREB5//RBM3//EIF4G2//TRIB1//NF2//PAK2//CEP85//CCK//PARP16//CHRNA3//GRM2//PALM//TIRAP//DBNL//DDX39B//CASP7//DRAM1//PIP4K2C//TP53INP2//TRIM21//DLAT//SIRT3//BCR//CTNS//MICAL2//BMP10//TTK//ABHD5//PPP1R26//CNTN2//LARP1//RBMS3//HMOX1//ATP6V1G2//PRKAB1//EXOC4//VPS26A//TAF8//DLX5//FZD2//NDP//PPP3CB//KMT2E//CALCOCO1//SFRP1//TEAD1//WNT11//PAX8//CDKN2A//PASD1//TNFSF4//YWHAB//MEX3B//SMAD1//RGMA//AGPAT1//UBXN2A//NDFIP1//EMP2//OXSR1//MAPKAP1//NEK7//TNKS2//RNF217//RNF125//RNF114//PPP1R2//CRTC1//INSIG2//FGFR3//SMPD1//SLC6A3//AQP1//RAG1//TNFSF14//S100A12//SPRY3//RNF149//TRIM5//PIM1//TIAM1//WDR91//PIK3IP1//UBXN2B//DDAH1//MAEA//ARID3B//COPS5//PRDM4//TET3//FSHB//RAX//TLX2//PHOX2A//NFATC4//ZMIZ1//CREB3L3//ONECUT2//DAZL//SRSF9//ITGA5//CTH//NOVA1//POLR2E//SH3BP5//SEMA7A//SNRPA//ALAD//EFNA3//BEX4//TRABD2B//FAM168A//TRIM67//GRIN2B// |
| GO:0006694 | steroid biosynthetic process | Biological process | 10 | 186 | 509 | 17653 | 1.86460907957834 | 0.0435890017062761 | 0.421958023081912 | 1.36062307726404 | 0.0196463654223969 | EBP//IDI1//INSIG2//PRKAA1//FSHB//MED1//NFKB1//FAXDC2//NFYA//IL1B// |
| GO:0007044 | cell-substrate junction assembly | Biological process | 6 | 89 | 509 | 17653 | 2.33809408180835 | 0.0436339794516552 | 0.421958023081912 | 1.36017517722442 | 0.0117878192534381 | FERMT2//DAPK3//SFRP1//THBS1//ITGA5//TLN1// |
| GO:0003281 | ventricular septum development | Biological process | 5 | 67 | 509 | 17653 | 2.58818872239979 | 0.0438506979756987 | 0.421958023081912 | 1.35802348953666 | 0.00982318271119843 | FGFR2//FZD2//HEYL//WNT11//PAX8// |
| GO:0006695 | cholesterol biosynthetic process | Biological process | 5 | 67 | 509 | 17653 | 2.58818872239979 | 0.0438506979756987 | 0.421958023081912 | 1.35802348953666 | 0.00982318271119843 | EBP//IDI1//NFYA//PRKAA1//INSIG2// |
| GO:0030521 | androgen receptor signaling pathway | Biological process | 5 | 67 | 509 | 17653 | 2.58818872239979 | 0.0438506979756987 | 0.421958023081912 | 1.35802348953666 | 0.00982318271119843 | TRIM68//HEYL//SFRP1//MED1//NRIP1// |
| GO:0040014 | regulation of multicellular organism growth | Biological process | 5 | 67 | 509 | 17653 | 2.58818872239979 | 0.0438506979756987 | 0.421958023081912 | 1.35802348953666 | 0.00982318271119843 | FOXS1//SLC6A3//FGFR2//TNKS2//CACNA2D2// |
| GO:0050769 | positive regulation of neurogenesis | Biological process | 19 | 430 | 509 | 17653 | 1.53244848540229 | 0.0440724378518379 | 0.421958023081912 | 1.3558329259748 | 0.037328094302554 | OTP//FZD3//TRIM67//ZNF804A//HEYL//ASCL1//SEMA7A//KIT//SHANK3//RTN4//SKIL//TIAM1//CUX1//ACTR2//DLG4//CRTC1//PACSIN1//VLDLR//SYT2// |
| GO:0051649 | establishment of localization in cell | Biological process | 74 | 2117 | 509 | 17653 | 1.21230417436544 | 0.0449828384192667 | 0.421958023081912 | 1.34695314403307 | 0.145383104125737 | SPRN//PML//NUP214//SH3GL1//EXOC4//U2AF1//DDX39B//SRSF9//RBM8A//YWHAB//GOLGA7B//GOSR2//TIMM23//TOMM34//TIMM22//VPS45//AP1S1//ARFIP2//RABL3//KCNIP3//MLPH//VPS26A//CD74//DYNC1I1//SPTBN2//TGOLN2//SYS1//UNC13B//CPLX1//SNPH//MGRN1//FAM160A2//P2RX2//CHRNA3//SYT2//SYT9//SYT14//CPLX3//WAS//ACTR3//ACTR2//PEX5//TOMM40L//CCDC88C//REEP2//ARL4C//EHD3//VPS53//LRRK2//MED1//MYO19//WDR19//RAF1//PPP3CB//VSNL1//KIT//HMOX1//IL4R//EMP2//WDR91//IL1B//PACSIN1//SNRPB//MIS12//SLC6A3//TIAM1//BCR//TTL//CHRM1//GRIN2B//GRIPAP1//NUCB1//PRKAA1//UBE2D3// |
| GO:0006766 | vitamin metabolic process | Biological process | 8 | 137 | 509 | 17653 | 2.02521044555662 | 0.045015791750915 | 0.421958023081912 | 1.34663510695389 | 0.0157170923379175 | SLC5A6//SLC52A2//ALDH1A2//NFKB1//VNN2//SLC23A2//CYP4F2//IL1B// |
| GO:0090263 | positive regulation of canonical Wnt signaling pathway | Biological process | 8 | 137 | 509 | 17653 | 2.02521044555662 | 0.045015791750915 | 0.421958023081912 | 1.34663510695389 | 0.0157170923379175 | LRRK2//DAPK3//DLX5//FGFR2//NFKB1//SFRP1//TNKS2//DIXDC1// |
| GO:0050870 | positive regulation of T cell activation | Biological process | 11 | 213 | 509 | 17653 | 1.7910752003837 | 0.0451778387440397 | 0.421958023081912 | 1.34507454906834 | 0.0216110019646365 | DPP4//PAK2//TNFSF14//FOXP3//CD209//IL1B//TNFSF4//ZMIZ1//RAG1//CD74//IL4R// |
| GO:0043068 | positive regulation of programmed cell death | Biological process | 26 | 633 | 509 | 17653 | 1.42452598875843 | 0.0452204954811468 | 0.421958023081912 | 1.34466468339824 | 0.0510805500982318 | PML//CCK//CASP7//CDKN2A//UNC13B//DAPK3//HMOX1//MAP3K9//ARHGEF4//SFRP1//UBE2Z//SMPD1//TGM2//TIAM1//WNT11//FOSL1//ALDH1A2//ARHGEF6//ASCL1//MYBL2//YWHAB//SKIL//THBS1//NFATC4//PAK2//LRRK2// |
| GO:0006020 | inositol metabolic process | Biological process | 2 | 12 | 509 | 17653 | 5.78028814669286 | 0.0452342993840583 | 0.421958023081912 | 1.34453213190844 | 0.00392927308447937 | IMPA2//SLC5A3// |
| GO:0010623 | programmed cell death involved in cell development | Biological process | 2 | 12 | 509 | 17653 | 5.78028814669286 | 0.0452342993840583 | 0.421958023081912 | 1.34453213190844 | 0.00392927308447937 | IL1B//KIT// |
| GO:0010935 | regulation of macrophage cytokine production | Biological process | 2 | 12 | 509 | 17653 | 5.78028814669286 | 0.0452342993840583 | 0.421958023081912 | 1.34453213190844 | 0.00392927308447937 | SEMA7A//CD74// |
| GO:0014831 | gastro-intestinal system smooth muscle contraction | Biological process | 2 | 12 | 509 | 17653 | 5.78028814669286 | 0.0452342993840583 | 0.421958023081912 | 1.34453213190844 | 0.00392927308447937 | KIT//PTAFR// |
| GO:0017121 | phospholipid scrambling | Biological process | 2 | 12 | 509 | 17653 | 5.78028814669286 | 0.0452342993840583 | 0.421958023081912 | 1.34453213190844 | 0.00392927308447937 | ANO4//ANO3// |
| GO:0030656 | regulation of vitamin metabolic process | Biological process | 2 | 12 | 509 | 17653 | 5.78028814669286 | 0.0452342993840583 | 0.421958023081912 | 1.34453213190844 | 0.00392927308447937 | NFKB1//IL1B// |
| GO:0032700 | negative regulation of interleukin-17 production | Biological process | 2 | 12 | 509 | 17653 | 5.78028814669286 | 0.0452342993840583 | 0.421958023081912 | 1.34453213190844 | 0.00392927308447937 | FOXP3//TNFSF4// |
| GO:0033160 | positive regulation of protein import into nucleus, translocation | Biological process | 2 | 12 | 509 | 17653 | 5.78028814669286 | 0.0452342993840583 | 0.421958023081912 | 1.34453213190844 | 0.00392927308447937 | LRRK2//MED1// |
| GO:0042033 | chemokine biosynthetic process | Biological process | 2 | 12 | 509 | 17653 | 5.78028814669286 | 0.0452342993840583 | 0.421958023081912 | 1.34453213190844 | 0.00392927308447937 | HMOX1//IL1B// |
| GO:0043097 | pyrimidine nucleoside salvage | Biological process | 2 | 12 | 509 | 17653 | 5.78028814669286 | 0.0452342993840583 | 0.421958023081912 | 1.34453213190844 | 0.00392927308447937 | UCK2//UCK1// |
| GO:0045161 | neuronal ion channel clustering | Biological process | 2 | 12 | 509 | 17653 | 5.78028814669286 | 0.0452342993840583 | 0.421958023081912 | 1.34453213190844 | 0.00392927308447937 | KCNIP2//CNTN2// |
| GO:0048266 | behavioral response to pain | Biological process | 2 | 12 | 509 | 17653 | 5.78028814669286 | 0.0452342993840583 | 0.421958023081912 | 1.34453213190844 | 0.00392927308447937 | P2RX2//THBS1// |
| GO:0048302 | regulation of isotype switching to IgG isotypes | Biological process | 2 | 12 | 509 | 17653 | 5.78028814669286 | 0.0452342993840583 | 0.421958023081912 | 1.34453213190844 | 0.00392927308447937 | FOXP3//NDFIP1// |
| GO:0050755 | chemokine metabolic process | Biological process | 2 | 12 | 509 | 17653 | 5.78028814669286 | 0.0452342993840583 | 0.421958023081912 | 1.34453213190844 | 0.00392927308447937 | HMOX1//IL1B// |
| GO:0060442 | branching involved in prostate gland morphogenesis | Biological process | 2 | 12 | 509 | 17653 | 5.78028814669286 | 0.0452342993840583 | 0.421958023081912 | 1.34453213190844 | 0.00392927308447937 | FGFR2//SFRP1// |
| GO:0060766 | negative regulation of androgen receptor signaling pathway | Biological process | 2 | 12 | 509 | 17653 | 5.78028814669286 | 0.0452342993840583 | 0.421958023081912 | 1.34453213190844 | 0.00392927308447937 | HEYL//SFRP1// |
| GO:0071285 | cellular response to lithium ion | Biological process | 2 | 12 | 509 | 17653 | 5.78028814669286 | 0.0452342993840583 | 0.421958023081912 | 1.34453213190844 | 0.00392927308447937 | CDKN1B//NFATC4// |
| GO:0072567 | chemokine (C-X-C motif) ligand 2 production | Biological process | 2 | 12 | 509 | 17653 | 5.78028814669286 | 0.0452342993840583 | 0.421958023081912 | 1.34453213190844 | 0.00392927308447937 | TIRAP//CD74// |
| GO:0099563 | modification of synaptic structure | Biological process | 2 | 12 | 509 | 17653 | 5.78028814669286 | 0.0452342993840583 | 0.421958023081912 | 1.34453213190844 | 0.00392927308447937 | GRIPAP1//TIAM1// |
| GO:1905065 | positive regulation of vascular smooth muscle cell differentiation | Biological process | 2 | 12 | 509 | 17653 | 5.78028814669286 | 0.0452342993840583 | 0.421958023081912 | 1.34453213190844 | 0.00392927308447937 | CTH//KIT// |
| GO:0031124 | mRNA 3'-end processing | Biological process | 6 | 90 | 509 | 17653 | 2.31211525867714 | 0.0456644168229743 | 0.422871901178296 | 1.34042208432466 | 0.0117878192534381 | LSM11//SNRPA//U2AF1//DDX39B//SRSF9//RBM8A// |
| GO:0033273 | response to vitamin | Biological process | 6 | 90 | 509 | 17653 | 2.31211525867714 | 0.0456644168229743 | 0.422871901178296 | 1.34042208432466 | 0.0117878192534381 | ALAD//ALDH1A2//ASCL1//PIM1//MED1//SFRP1// |
| GO:0021799 | cerebral cortex radially oriented cell migration | Biological process | 3 | 28 | 509 | 17653 | 3.71589952287398 | 0.0457913142713854 | 0.422871901178296 | 1.33921689146642 | 0.00589390962671906 | RTN4//FBXO45//DIXDC1// |
| GO:0046636 | negative regulation of alpha-beta T cell activation | Biological process | 3 | 28 | 509 | 17653 | 3.71589952287398 | 0.0457913142713854 | 0.422871901178296 | 1.33921689146642 | 0.00589390962671906 | IL4R//TNFSF4//FOXP3// |
| GO:0051125 | regulation of actin nucleation | Biological process | 3 | 28 | 509 | 17653 | 3.71589952287398 | 0.0457913142713854 | 0.422871901178296 | 1.33921689146642 | 0.00589390962671906 | ARFIP2//WAS//BRK1// |
| GO:0071634 | regulation of transforming growth factor beta production | Biological process | 3 | 28 | 509 | 17653 | 3.71589952287398 | 0.0457913142713854 | 0.422871901178296 | 1.33921689146642 | 0.00589390962671906 | FOXP3//THBS1//WNT11// |
| GO:0090659 | walking behavior | Biological process | 3 | 28 | 509 | 17653 | 3.71589952287398 | 0.0457913142713854 | 0.422871901178296 | 1.33921689146642 | 0.00589390962671906 | CTNS//SCN1A//CNTN2// |
| GO:1901565 | organonitrogen compound catabolic process | Biological process | 46 | 1238 | 509 | 17653 | 1.28865874675867 | 0.0459205542325079 | 0.422871901178296 | 1.33799287891437 | 0.0903732809430255 | CTH//DPYS//TET3//RNF123//UBE4B//RNF217//FBXO45//FBXO10//RLIM//UCHL5//RNF125//USP37//TP53INP2//USP46//UBE2D3//UBE2E1//USP45//DDAH1//UROC1//HAAO//SMPD1//HMOX1//FBXL3//SKP1//FBXL20//OGT//TRIB1//LRRK2//RNF114//VLDLR//DNAJB12//UBXN2A//SLC6A3//CHDH//MAEA//UBXN2B//RNF38//PML//CDKN1B//NDFIP1//IL1B//ALAD//WDR91//CD81//EDEM3//TRIM67// |
| GO:0003254 | regulation of membrane depolarization | Biological process | 4 | 47 | 509 | 17653 | 2.95163650043891 | 0.0459904610724276 | 0.422871901178296 | 1.33733223643146 | 0.00785854616895874 | LRRK2//CCK//SCN3B//SLMAP// |
| GO:0099601 | regulation of neurotransmitter receptor activity | Biological process | 4 | 47 | 509 | 17653 | 2.95163650043891 | 0.0459904610724276 | 0.422871901178296 | 1.33733223643146 | 0.00785854616895874 | DLG4//SHISA9//SHANK3//LYPD6// |
| GO:0071702 | organic substance transport | Biological process | 91 | 2667 | 509 | 17653 | 1.1833660772757 | 0.046080216921059 | 0.422871901178296 | 1.33648548515348 | 0.178781925343811 | SPRN//PML//NUP214//SLC27A4//EDN1//CD74//U2AF1//DDX39B//SRSF9//RBM8A//YWHAB//GOLGA7B//EXOC4//GOSR2//TIMM23//TOMM34//TIMM22//ANO4//ESYT1//PCTP//ANO3//VLDLR//VPS45//AP1S1//ARFIP2//RABL3//KCNIP3//MLPH//VPS26A//SYT9//DPP4//SLC25A5//IL1B//PPP3CB//ABCC8//CACNA2D2//SLC35A2//THBS1//RSC1A1//UNC13B//CPLX1//GRM2//CCK//CHRNA3//KDELR3//EHD3//VPS53//FAM160A2//SCAMP1//AQP1//SLC26A1//BEST1//CA7//SLC5A3//CTNS//SLC6A3//SLC23A2//SLC5A6//SLC25A2//PEX5//SNX19//CPLX3//TOMM40L//SIRT3//SLC52A2//CYP4F2//NFKB1//REEP2//LRRK2//MED1//ATP6V1G2//RAF1//VSNL1//SYS1//SFRP1//CD209//RBFOX1//S100A12//FOXP3//TNFSF4//NDFIP1//TIAM1//BCR//IL4R//CHRM1//GRIPAP1//RALBP1//NUCB1//PRKAA1//UBE2D3//PAX8// |
| GO:1901617 | organic hydroxy compound biosynthetic process | Biological process | 12 | 240 | 509 | 17653 | 1.73408644400786 | 0.0460934684032244 | 0.422871901178296 | 1.33636061121357 | 0.0235756385068762 | IMPA2//EBP//IDI1//INSIG2//PRKAA1//NFKB1//FAXDC2//PTAFR//SLC6A3//CTNS//NFYA//IL1B// |
| GO:0006952 | defense response | Biological process | 61 | 1707 | 509 | 17653 | 1.23935879419425 | 0.0461451224411626 | 0.422871901178296 | 1.33587419732142 | 0.119842829076621 | IL1B//DPP4//USP46//FBXL20//CCK//TNFSF4//TRIM5//TAB1//CD209//NFKB1//PAK2//RAF1//SKP1//HMOX1//RBMS3//JAM3//IL4R//CD96//THBS1//CREB3L3//FOXP3//TIRAP//UBE2D3//S100A12//TAC4//KIT//SMAD1//NFATC4//PTAFR//REL//SEMA7A//RELT//PLA2G4C//VSIG4//LGALS3BP//FOSL1//C1QA//CD81//WDFY1//TRIM21//RNF125//HMGB3//PML//TNFAIP8L2//RNF135//BCR//NDFIP1//TGM2//NCAM1//TRIM68//MED1//ACTR3//ACTR2//DAPK3//EDN1//MRC1//WAS//UNC13B//RAG1//CLEC1A//CD74// |
| GO:0055008 | cardiac muscle tissue morphogenesis | Biological process | 5 | 68 | 509 | 17653 | 2.55012712354097 | 0.0462579362827857 | 0.423332101597401 | 1.33481374642469 | 0.00982318271119843 | UBE4B//MED1//BMP10//FGFR2//MYOM3// |
| GO:0051403 | stress-activated MAPK cascade | Biological process | 13 | 267 | 509 | 17653 | 1.68862350352825 | 0.0464964061817103 | 0.424939452712036 | 1.33258061347066 | 0.0255402750491159 | TRIB1//TAB1//ARHGEF6//MAP3K9//DBNL//EDN1//TIAM1//SFRP1//COPS5//TIRAP//IL1B//NFKB1//SKP1// |
| GO:0061351 | neural precursor cell proliferation | Biological process | 8 | 138 | 509 | 17653 | 2.01053500754534 | 0.0466396271202703 | 0.425673141989727 | 1.33124493038147 | 0.0157170923379175 | OTP//FZD3//ASCL1//FGFR2//DIXDC1//LRRK2//NF2//LIMS2// |
| GO:0008360 | regulation of cell shape | Biological process | 9 | 163 | 509 | 17653 | 1.91494208540745 | 0.0468685999891386 | 0.42661149626722 | 1.32911801908642 | 0.0176817288801572 | SEPT7//FERMT2//CDC42EP1//DAPK3//KIT//PALM//RHOF//PARVA//WAS// |
| GO:1902275 | regulation of chromatin organization | Biological process | 9 | 163 | 509 | 17653 | 1.91494208540745 | 0.0468685999891386 | 0.42661149626722 | 1.32911801908642 | 0.0176817288801572 | PARG//PAXBP1//FOXP3//IL1B//PAX5//PRDM12//OGT//LRRK2//KMT2E// |
| GO:0043154 | negative regulation of cysteine-type endopeptidase activity involved in apoptotic process | Biological process | 6 | 91 | 509 | 17653 | 2.28670739869168 | 0.0477527197994342 | 0.433492139602112 | 1.32100188773997 | 0.0117878192534381 | PAK2//AQP1//RAF1//RAG1//THBS1//TNFSF14// |
| GO:0120034 | positive regulation of plasma membrane bounded cell projection assembly | Biological process | 6 | 91 | 509 | 17653 | 2.28670739869168 | 0.0477527197994342 | 0.433492139602112 | 1.32100188773997 | 0.0117878192534381 | BRK1//CDC42EP1//KIT//PALM//EPS8L2//SEPT7// |
| GO:0016241 | regulation of macroautophagy | Biological process | 9 | 164 | 509 | 17653 | 1.90326560927692 | 0.0483874674450782 | 0.436123000582508 | 1.31526710786473 | 0.0176817288801572 | LARP1//HMOX1//LRRK2//PIP4K2C//ATP6V1G2//PRKAA1//PRKAB1//EXOC4//VPS26A// |
| GO:0007507 | heart development | Biological process | 23 | 550 | 509 | 17653 | 1.4503268440793 | 0.048591300515722 | 0.436123000582508 | 1.31344147716166 | 0.0451866404715128 | CCDC103//MICAL2//TAB1//ZMIZ1//ALDH1A2//FGFR2//PARVA//FZD2//HEYL//WNT11//UBE4B//MED1//PAX8//BMP10//MYOM3//SMAD1//PIM1//RTN4//EDN1//DDX39B//NFATC4//PPP3CB//RAF1// |
| GO:0120031 | plasma membrane bounded cell projection assembly | Biological process | 23 | 550 | 509 | 17653 | 1.4503268440793 | 0.048591300515722 | 0.436123000582508 | 1.31344147716166 | 0.0451866404715128 | ACTR3//ACTR2//UBXN10//EHD3//MCIDAS//PARVA//WDR19//RFX4//ONECUT2//SEPT7//WAS//BRK1//ARFIP2//KIT//ARHGEF4//ARHGEF6//CDC42EP1//EMP2//CCDC103//PALM//DBNL//MAPRE1//EPS8L2// |
| GO:0002696 | positive regulation of leukocyte activation | Biological process | 16 | 351 | 509 | 17653 | 1.58093351020659 | 0.0486888806960048 | 0.436123000582508 | 1.31257020929342 | 0.031434184675835 | TIRAP//CD74//CD81//DPP4//PAK2//TNFSF14//FOXP3//CD209//IL1B//TNFSF4//IL4R//THBS1//PTAFR//ZMIZ1//RAG1//LRRK2// |
| GO:0002088 | lens development in camera-type eye | Biological process | 5 | 69 | 509 | 17653 | 2.51316875943168 | 0.0487423215468495 | 0.436123000582508 | 1.31209378965734 | 0.00982318271119843 | NF2//SKIL//CDKN1B//CTNS//MED1// |
| GO:0036498 | IRE1-mediated unfolded protein response | Biological process | 5 | 69 | 509 | 17653 | 2.51316875943168 | 0.0487423215468495 | 0.436123000582508 | 1.31209378965734 | 0.00982318271119843 | COPS5//KDELR3//ASNA1//TLN1//GOSR2// |
| GO:0051155 | positive regulation of striated muscle cell differentiation | Biological process | 5 | 69 | 509 | 17653 | 2.51316875943168 | 0.0487423215468495 | 0.436123000582508 | 1.31209378965734 | 0.00982318271119843 | EDN1//BMP10//DDX39B//IL4R//TNFSF14// |
| GO:0055021 | regulation of cardiac muscle tissue growth | Biological process | 5 | 69 | 509 | 17653 | 2.51316875943168 | 0.0487423215468495 | 0.436123000582508 | 1.31209378965734 | 0.00982318271119843 | FGFR2//BMP10//PIM1//EDN1//DDX39B// |
| GO:0060411 | cardiac septum morphogenesis | Biological process | 5 | 69 | 509 | 17653 | 2.51316875943168 | 0.0487423215468495 | 0.436123000582508 | 1.31209378965734 | 0.00982318271119843 | FGFR2//PARVA//FZD2//HEYL//WNT11// |
| GO:1902653 | secondary alcohol biosynthetic process | Biological process | 5 | 69 | 509 | 17653 | 2.51316875943168 | 0.0487423215468495 | 0.436123000582508 | 1.31209378965734 | 0.00982318271119843 | EBP//IDI1//INSIG2//PRKAA1//NFYA// |
| GO:0046717 | acid secretion | Biological process | 7 | 115 | 509 | 17653 | 2.11106175792261 | 0.0488240614536591 | 0.436123000582508 | 1.31136609641521 | 0.0137524557956778 | UNC13B//CPLX1//GRM2//CCK//CYP4F2//EDN1//IL1B// |
| GO:0008542 | visual learning | Biological process | 4 | 48 | 509 | 17653 | 2.89014407334643 | 0.0490743166410304 | 0.436123000582508 | 1.30914573922864 | 0.00785854616895874 | CTNS//KIT//RAG1//ABCC8// |
| GO:0055010 | ventricular cardiac muscle tissue morphogenesis | Biological process | 4 | 48 | 509 | 17653 | 2.89014407334643 | 0.0490743166410304 | 0.436123000582508 | 1.30914573922864 | 0.00785854616895874 | UBE4B//MED1//FGFR2//BMP10// |
| GO:0060324 | face development | Biological process | 4 | 48 | 509 | 17653 | 2.89014407334643 | 0.0490743166410304 | 0.436123000582508 | 1.30914573922864 | 0.00785854616895874 | DLX5//CRISPLD1//RAF1//ALDH1A2// |
| GO:1903307 | positive regulation of regulated secretory pathway | Biological process | 4 | 48 | 509 | 17653 | 2.89014407334643 | 0.0490743166410304 | 0.436123000582508 | 1.30914573922864 | 0.00785854616895874 | UNC13B//IL4R//PTAFR//SYT9// |
| GO:1905953 | negative regulation of lipid localization | Biological process | 4 | 48 | 509 | 17653 | 2.89014407334643 | 0.0490743166410304 | 0.436123000582508 | 1.30914573922864 | 0.00785854616895874 | THBS1//ABHD5//CYP4F2//NFKB1// |
| GO:0048523 | negative regulation of cellular process | Biological process | 156 | 4821 | 509 | 17653 | 1.12224636077671 | 0.0491533756392855 | 0.436252335234236 | 1.30844665135833 | 0.306483300589391 | CUX1//EDN1//FGFR2//FOXS1//SATB2//ZNF281//DNAJB5//HEYL//KCNIP3//HIC1//ASCL1//NFKB1//PAX5//FOXP3//RLIM//MED1//CC2D1A//REL//SKIL//UBE2D3//VLDLR//NRIP1//BHLHE40//PAXBP1//LRRK2//VASH1//THBS1//CDKN2A//NF2//DLG4//LRRTM1//HMOX1//BCR//CD74//TRIB1//PAK2//CEP85//CDKN1B//PML//RASSF1//EIF4G2//PRKAA1//PRKAB1//KMT2E//TTK//KLHL22//ARHGDIB//GRM2//PALM//MAEA//COPS5//FGFR3//AQP1//KIT//PAFAH2//PIM1//LIMS2//RAF1//SFRP1//WNT11//PTPRU//GPNMB//DDAH1//LDOC1//IL1B//SMAD1//DEC1//FOSL1//ALDH1A2//RGS9BP//BMP10//PAX8//SIRT3//DPP4//CTNS//PPP1R26//UCHL5//SKP1//PIK3IP1//MGRN1//YWHAB//PASD1//FOXG1//PURA//CREBZF//TNFSF4//DAPK3//LARP1//CELF4//MEX3B//RFX4//SMPD1//TRABD2B//NFATC4//RTN4//HTRA4//RASL11B//ONECUT2//MAPRE1//OGT//SHANK3//PPP1R2//PTBP1//RNF125//SPRY3//NDFIP1//RAG1//TNFSF14//RNF149//HMGB3//FAM57B//IL4R//MBNL3//CXCL14//CNTN2//DIXDC1//INSIG2//PACSIN1//FZD3//TRIM67//MAPKAP1//ABCC8//VSNL1//SRSF9//RGMA//SEMA4G//SEMA7A//TLX2//TNFAIP8L2//CGNL1//WAS//SPTBN2//LMOD1//PARP16//POLR2E//NUP214//SH3BP5//TIRAP//DAZL//TRIM21//RBMS3//KREMEN1//OXSR1//SLC25A5//PEX5//PTPN18//ALAD//TPRA1//GRIN2B//HOXC6//TNKS2//BEX4//DDX39B//LYPD6//ITGA5//CTH// |
| GO:0051962 | positive regulation of nervous system development | Biological process | 21 | 493 | 509 | 17653 | 1.4773150232927 | 0.0492292810398344 | 0.436353378338663 | 1.30777650675645 | 0.0412573673870334 | OTP//FZD3//TRIM67//ZNF804A//HEYL//ASCL1//SEMA7A//KIT//SHANK3//RTN4//SKIL//TIAM1//CUX1//LRRTM1//LRRN1//ACTR2//DLG4//CRTC1//PACSIN1//VLDLR//SYT2// |
| GO:0007589 | body fluid secretion | Biological process | 6 | 92 | 509 | 17653 | 2.26185188348851 | 0.0498991464150818 | 0.438177265526602 | 1.30190688344255 | 0.0117878192534381 | CSN3//MED1//SLC6A3//AQP1//CHRM1//EDN1// |
