# Supplementary material for: Altered microRNA Transcriptome in Cultured Human Liver Cells upon Infection with Ebola Virus
Source: Int J Mol Sci. 2021 Apr 6;22(7):3792. doi: 10.3390/ijms22073792 (PMC8038836; doi:10.3390/ijms22073792)
Supplement: Supplementary file 1 [file ijms-22-03792-s001.zip › Supplementary_File/C_ GO_Analysis_Results/16-30nt_go_Makona-96h-Huh7_vs_Control-96h-Huh7_down.mature_mirna_targets/CC_result(Human).html]

| GO.ID | Term | Ontology | Count | Pop.Hits | List.Total | Pop.Total | Fold.Enrichment | Pvalue | FDR | Enrichment.Score | Gene.Ratio | GENES |
| --- | --- | --- | --- | --- | --- | --- | --- | --- | --- | --- | --- | --- |
| GO:0045202 | synapse | Cellular component | 59 | 880 | 543 | 18698 | 2.30868491545287 | 1.8395056772885e-09 | 1.58013537679082e-06 | 8.7352988674625 | 0.10865561694291 | UNC13B//DLG4//TBC1D24//FGFR2//LRRTM1//BSN//IQSEC3//ACTR2//CHRM1//CHRNA3//FBXO45//DBNL//GRIN2B//SH2D5//NRGN//PALM//RTN4//BCR//TIAM1//SHISA9//SHANK3//COPS5//LGI3//SYNPR//SYPL2//ATP6V1G2//LRRK2//SYT2//SYT9//ABCC8//SCAMP1//GRM2//FOSL1//SNPH//CPLX1//AP1S1//CPLX3//CCK//ZNF804A//ELK1//PACSIN1//KCNIP3//KCNA6//SSPN//FZD3//SLC6A3//P2RX2//GRIPAP1//LYPD6//PRIMA1//CRTC1//HTR3E//ITGA5//NRN1//EXOC4//SH3GL1//CNTN2//NDFIP1//CHRFAM7A// |
| GO:0044456 | synapse part | Cellular component | 47 | 740 | 543 | 18698 | 2.18706385943955 | 4.41549759062184e-07 | 0.000189645621517208 | 6.35502034787702 | 0.0865561694290976 | COPS5//DLG4//LGI3//UNC13B//LRRK2//SYT2//SYT9//ABCC8//SCAMP1//NRGN//FZD3//BSN//CPLX3//SLC6A3//ZNF804A//P2RX2//PALM//SHISA9//FBXO45//GRM2//FOSL1//SNPH//CHRM1//CHRNA3//GRIN2B//LRRTM1//SH2D5//IQSEC3//BCR//SSPN//SHANK3//SYNPR//SYPL2//ATP6V1G2//CPLX1//AP1S1//TBC1D24//CCK//ELK1//PACSIN1//KCNIP3//KCNA6//TIAM1//GRIPAP1//ACTR2//DBNL//RTN4// |
| GO:0098793 | presynapse | Cellular component | 30 | 379 | 543 | 18698 | 2.72569570985 | 7.45607341312606e-07 | 0.000213492235395843 | 6.12748982453302 | 0.0552486187845304 | COPS5//DLG4//LGI3//UNC13B//FBXO45//GRM2//FOSL1//SNPH//CPLX1//AP1S1//LRRK2//TBC1D24//CPLX3//CCK//CHRM1//ELK1//PACSIN1//KCNIP3//KCNA6//FZD3//BSN//SYT2//SYT9//ABCC8//SCAMP1//SYNPR//SYPL2//ATP6V1G2//SLC6A3//ZNF804A// |
| GO:0030054 | cell junction | Cellular component | 66 | 1247 | 543 | 18698 | 1.82252211938487 | 1.55755495838352e-06 | 0.000334484927312861 | 5.8075566203018 | 0.121546961325967 | PTPRU//ACTR3//ITGA5//KIT//TIAM1//WAS//NPHS2//CDC42EP1//EIF4G2//PGM5//JAM3//DPP4//CD96//CTNND2//NF2//PARD6B//CGNL1//SCN1A//ACTR2//FERMT2//FGFR3//FZD2//EHD3//LIMS2//PARVA//TGM2//TLN1//YWHAB//NUP214//DIXDC1//CD81//UNC13B//COPS5//CHRM1//CHRNA3//LRRK2//SYT2//LYPD6//SYNPR//SYT9//PRIMA1//DLG4//FBXO45//LGI3//DBNL//GRIN2B//GRM2//PACSIN1//LRRTM1//SH2D5//NRN1//BIN2//GRIPAP1//TBC1D24//CPLX3//BCR//RSC1A1//SH3GL1//SPTBN2//SHISA9//NDFIP1//SSPN//FERMT3//SHANK3//BSN//SNPH// |
| GO:0005737 | cytoplasm | Cellular component | 383 | 11544 | 543 | 18698 | 1.14245152504821 | 8.38130664443011e-06 | 0.00143990848151309 | 5.07668826964784 | 0.705340699815838 | FERMT2//FGFR2//ARFIP2//DBNL//BIN2//RHOF//NDFIP1//PARD6B//PLA2G4C//UNC13B//LRRK2//DLG4//THBS1//EXD1//CARHSP1//CCDC103//SEPT7//BSN//KDELR3//VPS45//GBP4//AP1S1//CUX1//EMP2//GALNT3//ST8SIA5//ST6GALNAC6//GOLGA7B//MGAT5//NCAM1//RNF125//GALNT10//SLC30A6//MAN1C1//ST3GAL3//SLC35A2//KIAA0319L//TNKS2//FAM57B//CREB3L3//PGAP3//HS6ST1//GAL3ST1//GOSR2//CD74//EXOC4//PPP1R2//CEP85//PYURF//TRIM21//MEX3B//TRIM5//VPS53//KIT//SKIL//CTNS//IL1B//DRAM1//SMPD1//SHKBP1//VPS26A//TIMM23//EMC8//TOMM34//AKAP10//GLYCTK//DLAT//ELK1//ALDOC//SIRT3//DDAH1//MYCBP//GLRX//SLC25A5//NFKB1//UCHL5//CREBZF//RAF1//ABCC8//TGM2//YWHAB//MYO19//OGT//NFS1//SH3BP5//ACTR3//ACTR2//SGK2//ARL4C//FAM13A//SPRY3//CDKN1B//CDKN2A//TAB1//TGOLN2//CAMKK2//CPLX1//RALBP1//COPS5//TIRAP//UROC1//UBXN2B//CTH//RNF217//UBXN2A//DYNC1I1//DPYS//EIF4G2//ALAD//MAPRE1//MGRN1//CRTC1//HAAO//FTSJ1//LCLAT1//LMOD1//DNAJB5//SULT4A1//STEAP2//FBXL3//FBXO10//PTPN18//GNAZ//WDR91//PACSIN1//KCNIP3//EHD3//HIC1//HMOX1//HOXC6//IDI1//ARHGAP40//IMPA2//KIF2A//KRT2//KRT9//ARHGDIB//SMAD1//MAGEA11//NF2//NFATC4//SPRN//PABPC3//PAK2//ARHGEF4//PAX5//ABHD5//RLIM//C9ORF78//PGM5//PIM1//ATP6V1G2//PML//POLR2E//CC2D1A//PARP16//TRIM68//PPP3CB//PRKAA1//PRKAB1//LIMS2//MAP7D1//PARVA//BRK1//RNF114//MAPK4//GRIPAP1//SH3GLB2//TULP4//KLHL14//WDFY1//RANBP10//CALCOCO1//PEX5//TP53INP2//PCTP//RALGDS//CPLX3//REL//BCR//S100A12//RNF123//SFRP1//SH3GL1//EPS8L2//SKP1//UBE2Z//SNRPB//SPTBN2//TIAM1//TLN1//UBE2D3//UBE2E1//UCK2//BEST1//VSNL1//WAS//CA7//MIS12//MAPKAP1//CENPO//RPAP3//ZNF322//PIP4K2C//NUP214//CCDC6//FOSL1//URM1//KRTAP1-1//UCK1//CASP7//FAM160A2//RNF135//DDI2//ACBD6//KLHL22//FBXL20//PARG//SHANK3//DIXDC1//KRT38//ALDH1A2//SYS1//PAXBP1//ARHGEF6//RBM8A//OXSR1//SNN//TOMM40L//LETM2//GPD2//TIMM22//CHDH//SLC25A2//CABS1//DPP4//CYB561A3//VLDLR//NUCB1//CD79A//NRBF2//AGPAT1//EBP//FGFR3//VASH1//ESYT1//CYP2S1//LRRTM1//ITGA5//MEST//REEP2//DNAJB12//LRRC59//RGMA//APMAP//RTN4//NPHS2//MOGAT2//EDEM3//DBI//GPX8//FAXDC2//SLC27A4//CYP2C18//CYP4F3//ASNA1//PAFAH2//INSIG2//SLC10A7//CYP4F2//SLMAP//RSC1A1//TGM4//JAM3//CXCL14//RBFOX1//SCAMP1//EDN1//NPTX1//SSPN//SYT9//PTAFR//FAM92B//MAEA//MYOM3//SNPH//LGI3//MRC1//LARP1//NRGN//RBM3//BMP10//SCN3B//SCN1A//KCNQ5//SYNPR//SYPL2//SNX19//SYT2//FZD2//MLPH//POLR2M//LGALS3BP//FERMT3//SLC39A2//PALM//MMP24//CABP7//CALN1//TSPAN14//GPNMB//TAF8//FAM168B//AATK//AVL9//EMC10//TRIB1//CD96//UBE4B//PPIH//PRDM4//CDC42EP1//RASSF1//SLAIN1//LYPD6//CAPSL//NEK7//CTNND2//PRUNE2//DGKG//DAPK3//DAZL//DLX5//TET3//EVC//EWSR1//KLHDC10//TTC28//FSHB//HEYL//RBMS3//C11ORF21//KCNIP2//CD209//HMGB3//TLX2//AQP1//MARK1//MEA1//MAP3K9//IQSEC3//CCDC88C//TRIM67//FOXP3//PFDN1//RALGPS2//MBNL3//KMT2E//BEX4//CELF4//PRTFDC1//ZMIZ1//TBC1D24//WDR19//PURA//HIVEP3//SLC6A3//HLTF//TTK//WNT11//DDX39B//TNFAIP8L2//FZD3//TRIM7//CCDC70//RELT//USP45//BHLHE40//TNFSF14//EMILIN3//ZNF804A//NREP//SLC23A2// |
| GO:0042995 | cell projection | Cellular component | 91 | 2055 | 543 | 18698 | 1.52484216280643 | 2.57480503539878e-05 | 0.00368626254234592 | 4.58925565021739 | 0.167587476979742 | ARFIP2//DBNL//ITGA5//TLN1//UBXN10//EVC//IQCE//TULP4//WDR19//CHRNA3//LRRK2//LYPD6//SYNPR//DLG4//LGI3//HTR3E//GRIN2B//NF2//NPTX1//KLHL14//SLC6A3//FOSL1//SHANK3//CHRFAM7A//SNPH//ACTR3//ACTR2//DPP4//BRK1//ARHGEF6//RAF1//SCN7A//CCDC103//CABS1//SEPT7//FAM168B//GRM2//LRRTM1//KCNA6//NRGN//PALM//GRIPAP1//FZD3//CCK//BSN//CPLX1//CHRM1//CTNND2//ELK1//CRTC1//NPTXR//GNAO1//GNAZ//KCNIP3//NPTX2//PURA//RBM3//MLPH//NDFIP1//RBM8A//TIAM1//ZNF804A//SLC27A4//EXOC4//ARL4C//RNF38//TIRAP//PACSIN1//ARHGEF4//EPS8L2//UNC5A//FERMT2//KIF2A//SHISA9//RGS9BP//EHD3//MAPRE1//AQP1//SLC5A6//SCN1A//CNTN2//UNC13B//AP1S1//TBC1D24//CPLX3//RTN4//CAMKK2//FAM92B//BIN2//SH3GL1//FERMT3// |
| GO:0097458 | neuron part | Cellular component | 72 | 1533 | 543 | 18698 | 1.6172816814609 | 3.23318766591327e-05 | 0.00396758315002785 | 4.49036908663336 | 0.132596685082873 | RGS9BP//WDR19//COPS5//DLG4//LGI3//ACTR2//CHRM1//CHRNA3//FBXO45//DBNL//GRIN2B//SH2D5//NRGN//PALM//RTN4//BCR//TIAM1//SHISA9//SHANK3//BSN//LRRK2//LYPD6//SYNPR//HTR3E//NF2//NPTX1//KLHL14//SLC6A3//FOSL1//CHRFAM7A//SNPH//UNC13B//SYT2//SYT9//ABCC8//SCAMP1//EPS8L2//UNC5A//GRM2//CPLX1//ELK1//CRTC1//NPTXR//ASCL1//NPTX2//PURA//SCN1A//SPTBN2//CNTN2//FZD3//POLR2M//ZNF804A//RBM8A//CTNND2//CCK//CPLX3//FAM168B//LRRTM1//KCNA6//GRIPAP1//GNAO1//GNAZ//KCNIP3//RBM3//MLPH//NDFIP1//AP1S1//TBC1D24//PACSIN1//SYPL2//ATP6V1G2//EXOC4// |
| GO:0044459 | plasma membrane part | Cellular component | 116 | 2830 | 543 | 18698 | 1.41145448984506 | 5.32115910963562e-05 | 0.00497025580922082 | 4.27399375490658 | 0.213627992633518 | CD81//BIN2//CD209//ITGA5//KIT//NCAM1//THBS1//SEMA7A//CD74//CD79A//NPTXR//NPTX2//PGM5//FZD3//PRSS8//PPP3CB//SCN1A//TGM2//UNC5A//EXOC4//EVC//FERMT2//DLG4//TIAM1//PTPRU//CD96//MAEA//GPNMB//EBP//SLC12A7//SLC26A1//MMP24//CHRM1//CHRNA3//EFNA3//FGFR3//FGFR2//P2RX2//ATP1B4//STEAP2//GPR3//HTR3E//GPR34//GRIN2B//GRM2//SLC25A5//SLC39A2//IL4R//AQP1//KCNA6//KCNJ12//TRABD2B//MRC1//PALM//CLEC1A//REEP2//KCNQ5//PTAFR//SLC39A8//SLC5A3//SLC6A3//ABCC8//CNTN2//TMPRSS2//TNFSF4//NPHS2//SLMAP//SLC52A2//SSPN//TSPAN14//RELT//SLC5A6//CHRFAM7A//TSPAN18//HS6ST1//GAL3ST1//SLC23A2//DPP4//TIRAP//PACSIN1//NF2//ARHGEF4//EPS8L2//TLN1//KCNQ4//BEST1//EMP2//PRKAA1//SPTBN2//PARD6B//CYP4F2//SEPT7//MAPRE1//LRRK2//GNAO1//GNAZ//SCN3B//SCN7A//CACNG1//CACNA2D2//KCNIP3//KCNIP2//EHD3//SLC27A4//SHISA9//IQCE//SHANK3//HMOX1//UNC13B//FBXO45//FOSL1//SNPH//LRRTM1//SH2D5//IQSEC3//BCR// |
| GO:0044306 | neuron projection terminus | Cellular component | 14 | 139 | 543 | 18698 | 3.46823535646621 | 5.40609618460629e-05 | 0.00497025580922082 | 4.26711623165391 | 0.0257826887661142 | CHRM1//ELK1//PACSIN1//KCNIP3//KCNA6//UNC13B//CPLX1//AP1S1//LRRK2//TBC1D24//CPLX3//CCK//DLG4//BSN// |
| GO:0005938 | cell cortex | Cellular component | 22 | 296 | 543 | 18698 | 2.55933004828032 | 5.78609523774251e-05 | 0.00497025580922082 | 4.237614422246 | 0.0405156537753223 | UNC13B//BSN//EXOC4//MAEA//SEPT7//SPTBN2//ACTR2//WAS//DLG4//NF2//MLPH//POLR2M//MAPRE1//FERMT2//FGFR2//ARFIP2//DBNL//BIN2//RHOF//NDFIP1//PARD6B//PLA2G4C// |
| GO:0044425 | membrane part | Cellular component | 240 | 6780 | 543 | 18698 | 1.21892468912466 | 6.82312006204393e-05 | 0.00532823648481431 | 4.16601698665291 | 0.441988950276243 | PYURF//AGPAT1//EBP//FAXDC2//SLC27A4//KDELR3//CYP2C18//ESYT1//LCLAT1//CYP2S1//HMOX1//CYP4F3//MEST//ASNA1//PAFAH2//INSIG2//REEP2//PARP16//LRRC59//RTN4//KLHL14//MOGAT2//FAM57B//SLC10A7//CREB3L3//CYP4F2//PLA2G4C//GOSR2//AP1S1//VLDLR//KCNJ12//PRSS8//MUC22//GPNMB//TGOLN2//SLC26A1//TOMM34//PTPRT//VPS45//VSIG4//SMIM12//CHRNA3//PIK3IP1//TM4SF18//ANO4//TMEM132E//TEDDM1//SYT2//FAM168B//TPRA1//LETM2//SYT9//PRIMA1//CTNS//RNF217//CCDC167//CABP7//DPP4//EMP2//EVC//CYB561A3//FGFR2//P2RX2//AVL9//FAM189A1//NPTXR//FZD2//SYT14//FAM19A5//GALNT3//GPR160//C16ORF54//EMC10//RNF149//HTR3E//SLC25A5//ST8SIA5//TIMM22//ST6GALNAC6//CD209//XKR7//LRRTM1//KCNA6//KIT//RGS9BP//SMAD1//MGAT5//SMCO3//NCAM1//GPX8//PLLP//SEMA5B//DRAM1//GALNT10//SLC30A6//SCN3B//KCNQ5//APMAP//PTAFR//LRRN1//SEMA4G//MS4A7//ANO3//SFRP1//ST3GAL3//SLC5A3//SLC6A3//SLC9A5//SLC35E2B//SLC35A2//BEST1//NKAIN1//FZD3//KIAA0319L//NDFIP1//SNN//CALN1//JAM3//SLC25A2//KREMEN1//TMEM164//TMEM246//TMEFF1//TNFSF14//PPP1R3F//CHRFAM7A//UNC5A//KCNQ4//PGAP3//CD22//FAM189A2//SCAMP1//AATK//CD74//SNPH//SLC23A2//LYPD6//EFNA3//SPRN//NRN1//RGMA//CNTN2//SEMA7A//VNN2//TGM2//LRRK2//CD81//BIN2//ITGA5//THBS1//CD79A//NPTX2//PGM5//PPP3CB//SCN1A//EXOC4//TOMM40L//VPS26A//CPLX1//CPLX3//RNF125//EMC8//ATP6V1G2//PTPRU//CD96//MAEA//SLC12A7//MMP24//CHRM1//FGFR3//ATP1B4//STEAP2//GPR3//GPR34//GRIN2B//GRM2//SLC39A2//IL4R//AQP1//TRABD2B//MRC1//PALM//CLEC1A//SLC39A8//ABCC8//TMPRSS2//TNFSF4//NPHS2//SLMAP//SLC52A2//SSPN//TSPAN14//RELT//SLC5A6//TSPAN18//HS6ST1//GAL3ST1//MAN1C1//SYS1//DNAJB12//SYNPR//SYPL2//FERMT2//DLG4//TIAM1//WDR91//PML//NUCB1//NRBF2//TIRAP//PACSIN1//NF2//ARHGEF4//EPS8L2//TLN1//SHISA9//TIMM23//GNAO1//GNAZ//SCN7A//CACNG1//CACNA2D2//KCNIP3//KCNIP2//PRKAA1//SPTBN2//PARD6B//SEPT7//MAPRE1//DAPK3//UNC13B//FBXO45//FOSL1//SH2D5//IQSEC3//BCR//SHANK3//IQCE//EHD3//SESTD1// |
| GO:0120025 | plasma membrane bounded cell projection | Cellular component | 86 | 1980 | 543 | 18698 | 1.49564521829715 | 8.83044106219444e-05 | 0.00632112406035419 | 4.05401760377313 | 0.158379373848987 | ARFIP2//DBNL//ITGA5//TLN1//UBXN10//EVC//IQCE//TULP4//WDR19//CHRNA3//LRRK2//LYPD6//SYNPR//DLG4//LGI3//HTR3E//GRIN2B//NF2//NPTX1//KLHL14//SLC6A3//FOSL1//SHANK3//CHRFAM7A//SNPH//ACTR3//ACTR2//DPP4//BRK1//ARHGEF6//RAF1//SCN7A//CCDC103//CABS1//SEPT7//FAM168B//GRM2//LRRTM1//KCNA6//NRGN//PALM//GRIPAP1//FZD3//CCK//BSN//CPLX1//CHRM1//CTNND2//ELK1//CRTC1//NPTXR//GNAO1//GNAZ//KCNIP3//NPTX2//PURA//RBM3//MLPH//NDFIP1//RBM8A//SLC27A4//EXOC4//ARL4C//TIAM1//ZNF804A//RNF38//TIRAP//PACSIN1//ARHGEF4//EPS8L2//UNC5A//FERMT2//KIF2A//SHISA9//RGS9BP//EHD3//MAPRE1//AQP1//SLC5A6//SCN1A//CNTN2//UNC13B//AP1S1//TBC1D24//CPLX3//RTN4// |
| GO:0043005 | neuron projection | Cellular component | 58 | 1224 | 543 | 18698 | 1.63170596661009 | 0.000159254791010732 | 0.0105230665752476 | 3.79790749350925 | 0.106813996316759 | LRRK2//FAM168B//GRM2//LRRTM1//KCNA6//NRGN//PALM//GRIPAP1//SLC6A3//FZD3//CCK//BSN//CPLX1//CHRM1//CHRNA3//CTNND2//ELK1//CRTC1//NPTXR//GNAO1//GNAZ//DBNL//KCNIP3//NPTX2//PURA//RBM3//MLPH//NDFIP1//SHANK3//RBM8A//UNC5A//DLG4//TIAM1//ZNF804A//EPS8L2//SHISA9//RGS9BP//WDR19//PACSIN1//UNC13B//EXOC4//SCN1A//CNTN2//AP1S1//TBC1D24//CPLX3//RTN4//LYPD6//SYNPR//LGI3//HTR3E//GRIN2B//NF2//NPTX1//KLHL14//FOSL1//CHRFAM7A//SNPH// |
| GO:0005829 | cytosol | Cellular component | 183 | 5022 | 543 | 18698 | 1.25478612337758 | 0.000209817683270159 | 0.0128738135663619 | 3.67815791260089 | 0.337016574585635 | UCHL5//ACTR3//ACTR2//SGK2//ARL4C//FAM13A//SPRY3//CDKN1B//CDKN2A//EMC8//TAB1//UNC13B//TGOLN2//CAMKK2//CPLX1//RALBP1//TOMM34//FERMT2//COPS5//AKAP10//TIRAP//AP1S1//LRRK2//UROC1//GLYCTK//UBXN2B//CTH//CUX1//RNF217//UBXN2A//DLG4//DYNC1I1//DPYS//EIF4G2//EMP2//ALAD//MAPRE1//ALDOC//MGRN1//CRTC1//HAAO//DDAH1//CARHSP1//ARFIP2//FTSJ1//LCLAT1//LMOD1//DNAJB5//SULT4A1//STEAP2//FBXL3//FBXO10//PTPN18//GLRX//GNAZ//DBNL//WDR91//PACSIN1//KCNIP3//EHD3//HIC1//HMOX1//HOXC6//IDI1//ARHGAP40//IL1B//IMPA2//KIF2A//KRT2//KRT9//ARHGDIB//SMAD1//MAGEA11//NCAM1//NF2//NFATC4//NFKB1//SPRN//PABPC3//PAK2//ARHGEF4//PAX5//ABHD5//RLIM//C9ORF78//PGM5//PIM1//ATP6V1G2//PML//POLR2E//RHOF//CC2D1A//PARP16//TRIM68//VPS53//PPP3CB//PRKAA1//PRKAB1//LIMS2//MAP7D1//PARVA//BRK1//RNF114//MAPK4//GRIPAP1//SH3GLB2//TULP4//KLHL14//WDFY1//RANBP10//CALCOCO1//PEX5//TP53INP2//PCTP//RAF1//RALGDS//CPLX3//REL//EXOC4//BCR//S100A12//RNF123//SFRP1//SH3GL1//EPS8L2//CEP85//SKP1//UBE2Z//SNRPB//SPTBN2//TRIM21//TGM2//TIAM1//TLN1//UBE2D3//UBE2E1//UCK2//BEST1//VSNL1//WAS//YWHAB//CA7//MIS12//MAPKAP1//CENPO//RPAP3//ZNF322//PIP4K2C//MYO19//NUP214//CCDC6//TNKS2//FOSL1//URM1//KRTAP1-1//UCK1//CASP7//FAM160A2//MEX3B//RNF135//DDI2//ACBD6//PARD6B//CREB3L3//OGT//KLHL22//FBXL20//PARG//SHANK3//TRIM5//DIXDC1//PLA2G4C//KRT38//ALDH1A2//SYS1//NFS1//PAXBP1//ARHGEF6//VPS26A//GOSR2//SEPT7//RBM8A//OXSR1// |
| GO:0043679 | axon terminus | Cellular component | 12 | 123 | 543 | 18698 | 3.35947536270943 | 0.000245735795217212 | 0.0131303410529385 | 3.60953157720724 | 0.0220994475138122 | UNC13B//CPLX1//AP1S1//LRRK2//TBC1D24//CPLX3//CCK//CHRM1//ELK1//PACSIN1//KCNIP3//KCNA6// |
| GO:0016020 | membrane | Cellular component | 314 | 9426 | 543 | 18698 | 1.14709011827714 | 0.000259793219156996 | 0.0131303410529385 | 3.58537218860222 | 0.578268876611418 | PTPRU//ARL4C//CD96//GPNMB//UNC13B//TGOLN2//SLC12A7//SLC26A1//SLC27A4//PTPRT//CDC42EP1//AKAP10//CHRM1//CHRNA3//PIK3IP1//TIRAP//LRRK2//ANO4//SYT2//FAM168B//LYPD6//SYT9//PRIMA1//CTNS//DGKG//CABP7//DLG4//DPP4//EFNA3//EMP2//EWSR1//FGFR3//FGFR2//P2RX2//MGRN1//ESYT1//CRTC1//NPTXR//ARFIP2//FZD2//SYT14//STEAP2//GPR160//GNAO1//GNAZ//HTR3E//DBNL//GRIN2B//GRM2//SLC39A2//PACSIN1//ST6GALNAC6//KCNIP3//KCNIP2//CD209//IL4R//AQP1//ITGA5//KCNA6//ENHO//KCNJ12//KIT//MARK1//MRC1//NCAM1//NF2//NPTX1//NPTX2//SPRN//PAK2//PALM//NRN1//PIM1//RHOF//CC2D1A//ENOX1//RALGPS2//PPP3CB//LIMS2//PARVA//SCN3B//KMT2E//RNF114//KCNQ5//PRSS8//RGMA//RTN4//PTAFR//TBC1D24//SEMA4G//RAF1//CPLX3//EXOC4//RSC1A1//S100A12//SCN1A//SCN7A//ANO3//SLC39A8//SFRP1//EPS8L2//SLC5A3//SLC6A3//SLC9A5//SMPD1//ABCC8//CNTN2//TIAM1//TMPRSS2//TNFSF4//UBE2D3//VLDLR//BEST1//NPHS2//CACNG1//MAPKAP1//NKAIN1//SLC52A2//FZD3//KIAA0319L//TSPAN14//CCDC70//CALN1//JAM3//KREMEN1//SLC10A7//PARD6B//OGT//SEMA7A//SHANK3//TMEFF1//TNFSF14//VNN2//SLC5A6//UNC5A//KCNQ4//ZNF804A//CACNA2D2//CD22//SCAMP1//CD74//CD79A//CD81//SLC23A2//PYURF//AGPAT1//EBP//FAXDC2//KDELR3//CYP2C18//LCLAT1//CYP2S1//HMOX1//CYP4F3//MEST//ASNA1//PAFAH2//INSIG2//REEP2//PARP16//LRRC59//KLHL14//MOGAT2//FAM57B//CREB3L3//CYP4F2//PLA2G4C//GOSR2//KRT2//EVPLL//PGM5//SLMAP//SSPN//WAS//DNAJB12//RNF123//NRGN//SNPH//PRKAA1//SPTBN2//SEPT7//MAPRE1//TAB1//VPS45//EHD3//VPS53//NDFIP1//VPS26A//AP1S1//CYB561A3//DRAM1//WDR91//SNX19//PML//GRIPAP1//SH3GL1//YWHAB//ATP1B4//SMAD1//TIMM23//LETM2//GPD2//SLC25A5//TIMM22//CHDH//SLC25A2//CABS1//TOMM34//MYO19//SNN//GBP4//CUX1//GALNT3//ST8SIA5//GOLGA7B//MGAT5//RNF125//GALNT10//SLC30A6//MAN1C1//ST3GAL3//SLC35A2//TNKS2//PGAP3//HS6ST1//GAL3ST1//EVC//IQCE//FERMT2//ARHGEF4//TLN1//SHISA9//PEX5//FBXO45//FOSL1//LRRTM1//SH2D5//IQSEC3//BCR//NUCB1//MMP24//SYS1//DAPK3//PLLP//ATP6V1G2//MUC22//VSIG4//SMIM12//TM4SF18//TMEM132E//TEDDM1//TPRA1//RNF217//CCDC167//AVL9//FAM189A1//FAM19A5//C16ORF54//EMC10//RNF149//XKR7//RGS9BP//SMCO3//GPX8//SEMA5B//APMAP//LRRN1//MS4A7//SLC35E2B//TMEM164//TMEM246//PPP1R3F//CHRFAM7A//FAM189A2//AATK//BIN2//MAEA//GPR3//GPR34//TRABD2B//CLEC1A//RELT//TSPAN18//TOMM40L//THBS1//SYNPR//SYPL2//CPLX1//TGM2//SESTD1//NRBF2//EMC8//MED6//ACTR3//ACTR2//SPRY3//RALBP1//EIF4G2//LARP1//LMOD1//KIF2A//KRT9//LGALS3BP//ARHGDIB//MED1//PTBP1//HLTF//RASL11B//TTK//VSNL1//RERGL//PIP4K2C//EDEM3//FERMT3// |
| GO:0036477 | somatodendritic compartment | Cellular component | 40 | 766 | 543 | 18698 | 1.79815260928311 | 0.00025985541082649 | 0.0131303410529385 | 3.58526823567152 | 0.0736648250460405 | CPLX1//CHRNA3//LRRK2//ELK1//CRTC1//NPTXR//ASCL1//NPTX2//NRGN//RTN4//KLHL14//PURA//SCN1A//SLC6A3//SPTBN2//CNTN2//TIAM1//FZD3//POLR2M//ZNF804A//SNPH//RBM8A//UNC5A//CCK//CTNND2//CHRM1//GNAO1//GNAZ//DBNL//GRM2//KCNIP3//GRIPAP1//RBM3//MLPH//NDFIP1//SHANK3//BSN//DLG4//PALM//SHISA9// |
| GO:0150034 | distal axon | Cellular component | 19 | 268 | 543 | 18698 | 2.44126054808829 | 0.000331638645714194 | 0.0152920035833379 | 3.47933486701654 | 0.0349907918968692 | LRRK2//NPTXR//LRRTM1//NPTX2//ZNF804A//CHRM1//ELK1//PACSIN1//KCNIP3//KCNA6//RTN4//TIAM1//UNC13B//CPLX1//AP1S1//TBC1D24//CPLX3//CCK//EXOC4// |
| GO:0098590 | plasma membrane region | Cellular component | 51 | 1069 | 543 | 18698 | 1.64281173606768 | 0.000342747815407673 | 0.0152920035833379 | 3.46502530461844 | 0.0939226519337017 | AQP1//KCNQ4//SLC23A2//SLC26A1//DLG4//PALM//BEST1//CD81//DPP4//EMP2//P2RX2//PRKAA1//SPTBN2//FZD3//PARD6B//CYP4F2//SEPT7//MAPRE1//NF2//CHRNA3//LRRK2//FERMT2//SLC27A4//SLC5A6//TIRAP//PACSIN1//ITGA5//ARHGEF4//EPS8L2//TIAM1//TLN1//UNC5A//EVC//IQCE//SHANK3//HMOX1//SHISA9//UNC13B//FBXO45//GRM2//FOSL1//SNPH//CHRM1//GRIN2B//LRRTM1//SH2D5//IQSEC3//BCR//SSPN//EHD3//SLC6A3// |
| GO:0044463 | cell projection part | Cellular component | 62 | 1377 | 543 | 18698 | 1.55043325562951 | 0.000373844092258552 | 0.0152920035833379 | 3.42730947798315 | 0.114180478821363 | CPLX1//CHRM1//CHRNA3//LRRK2//CTNND2//ELK1//CRTC1//NPTXR//GNAO1//GNAZ//DBNL//GRM2//KCNIP3//NPTX2//GRIPAP1//PURA//RBM3//MLPH//FZD3//NDFIP1//SHANK3//CCK//BSN//RBM8A//MAPRE1//EPS8L2//DLG4//TIAM1//ZNF804A//NRGN//EHD3//FERMT2//DPP4//SLC27A4//AQP1//SLC5A6//NF2//PALM//TIRAP//PACSIN1//ITGA5//ARHGEF4//TLN1//UNC5A//SHISA9//EVC//IQCE//LRRTM1//EXOC4//SCN1A//CNTN2//UNC13B//AP1S1//TBC1D24//CPLX3//KCNA6//RTN4//RGS9BP//WDR19//CCDC103//SEPT7//KIF2A// |
| GO:0120038 | plasma membrane bounded cell projection part | Cellular component | 62 | 1377 | 543 | 18698 | 1.55043325562951 | 0.000373844092258552 | 0.0152920035833379 | 3.42730947798315 | 0.114180478821363 | CPLX1//CHRM1//CHRNA3//LRRK2//CTNND2//ELK1//CRTC1//NPTXR//GNAO1//GNAZ//DBNL//GRM2//KCNIP3//NPTX2//GRIPAP1//PURA//RBM3//MLPH//FZD3//NDFIP1//SHANK3//CCK//BSN//RBM8A//MAPRE1//EPS8L2//DLG4//TIAM1//ZNF804A//NRGN//FERMT2//DPP4//SLC27A4//AQP1//SLC5A6//NF2//PALM//TIRAP//PACSIN1//ITGA5//ARHGEF4//TLN1//UNC5A//EVC//IQCE//LRRTM1//SCN1A//CNTN2//UNC13B//AP1S1//TBC1D24//CPLX3//KCNA6//RTN4//RGS9BP//WDR19//CCDC103//SEPT7//KIF2A//EHD3//SHISA9//EXOC4// |
| GO:0034702 | ion channel complex | Cellular component | 20 | 296 | 543 | 18698 | 2.32666368025484 | 0.000435155708795436 | 0.0169908524479673 | 3.36135531460726 | 0.0368324125230203 | CHRNA3//CHRFAM7A//DLG4//SHISA9//SHANK3//BEST1//GRIN2B//SESTD1//KCNIP2//KCNA6//SCN3B//SCN1A//SCN7A//CACNG1//CACNA2D2//KCNIP3//KCNQ5//ABCC8//CNTN2//KCNQ4// |
| GO:1902495 | transmembrane transporter complex | Cellular component | 21 | 321 | 543 | 18698 | 2.25273231097571 | 0.000484193076706864 | 0.0180835588213564 | 3.31498142466195 | 0.0386740331491713 | CHRNA3//CHRFAM7A//DLG4//SHISA9//SHANK3//BEST1//GRIN2B//SESTD1//KCNIP2//KCNA6//SCN3B//SCN1A//SCN7A//CACNG1//CACNA2D2//KCNIP3//KCNQ5//ABCC8//CNTN2//KCNQ4//ATP1B4// |
| GO:0098791 | Golgi subcompartment | Cellular component | 41 | 819 | 543 | 18698 | 1.72383335919248 | 0.000514202524696029 | 0.0182099966241913 | 3.28886579533442 | 0.0755064456721915 | KDELR3//VPS45//GBP4//AP1S1//CUX1//EMP2//GALNT3//DBNL//ST8SIA5//ST6GALNAC6//GOLGA7B//MGAT5//NCAM1//RNF125//GALNT10//SLC30A6//MAN1C1//ST3GAL3//SLC35A2//KIAA0319L//TNKS2//NDFIP1//FAM57B//CREB3L3//PGAP3//HS6ST1//GAL3ST1//GOSR2//CD74//TGOLN2//LRRK2//NUCB1//RBFOX1//SYS1//SCAMP1//MMP24//CABP7//ARFIP2//VPS53//CALN1//STEAP2// |
| GO:0030424 | axon | Cellular component | 30 | 538 | 543 | 18698 | 1.92014623426236 | 0.000529976618864707 | 0.0182099966241913 | 3.27574328987473 | 0.0552486187845304 | DLG4//LRRK2//NPTXR//LRRTM1//NPTX2//ZNF804A//EXOC4//SCN1A//CNTN2//CCK//UNC13B//CPLX1//AP1S1//TBC1D24//CPLX3//CHRM1//ELK1//PACSIN1//KCNIP3//KCNA6//RTN4//TIAM1//FAM168B//GRM2//NRGN//PALM//GRIPAP1//SLC6A3//FZD3//BSN// |
| GO:0034703 | cation channel complex | Cellular component | 16 | 217 | 543 | 18698 | 2.53895833863754 | 0.000627417984949567 | 0.0197267676988849 | 3.2024430363995 | 0.0294659300184162 | GRIN2B//DLG4//SHISA9//SESTD1//KCNIP2//KCNA6//CACNG1//CACNA2D2//KCNIP3//KCNQ5//ABCC8//CNTN2//KCNQ4//SCN3B//SCN1A//SCN7A// |
| GO:0030425 | dendrite | Cellular component | 31 | 569 | 543 | 18698 | 1.87605148769933 | 0.000633676248347925 | 0.0197267676988849 | 3.1981325709318 | 0.0570902394106814 | LRRK2//DLG4//TIAM1//SHANK3//ZNF804A//RTN4//PALM//SHISA9//NRGN//CPLX1//CHRM1//CHRNA3//CTNND2//ELK1//CRTC1//NPTXR//GNAO1//GNAZ//DBNL//GRM2//KCNIP3//NPTX2//GRIPAP1//PURA//RBM3//MLPH//FZD3//NDFIP1//CCK//BSN//RBM8A// |
| GO:0097447 | dendritic tree | Cellular component | 31 | 570 | 543 | 18698 | 1.87276016929986 | 0.000652273824979934 | 0.0197267676988849 | 3.18557004888034 | 0.0570902394106814 | CPLX1//CHRM1//CHRNA3//LRRK2//CTNND2//ELK1//CRTC1//NPTXR//GNAO1//GNAZ//DBNL//GRM2//KCNIP3//NPTX2//GRIPAP1//PURA//RBM3//MLPH//FZD3//NDFIP1//SHANK3//CCK//BSN//RBM8A//DLG4//TIAM1//ZNF804A//RTN4//PALM//SHISA9//NRGN// |
| GO:1990351 | transporter complex | Cellular component | 21 | 329 | 543 | 18698 | 2.19795462560245 | 0.000666510038888549 | 0.0197267676988849 | 3.17619330491319 | 0.0386740331491713 | CHRNA3//CHRFAM7A//DLG4//SHISA9//SHANK3//GRIN2B//SESTD1//KCNIP2//KCNA6//BEST1//ATP1B4//SCN3B//SCN1A//SCN7A//CACNG1//CACNA2D2//KCNIP3//KCNQ5//ABCC8//CNTN2//KCNQ4// |
| GO:0033267 | axon part | Cellular component | 22 | 353 | 543 | 18698 | 2.14606712263733 | 0.000688944157120544 | 0.0197267676988849 | 3.16181597872701 | 0.0405156537753223 | LRRK2//NPTXR//LRRTM1//NPTX2//ZNF804A//SCN1A//CNTN2//CCK//UNC13B//CPLX1//AP1S1//TBC1D24//CPLX3//CHRM1//ELK1//PACSIN1//KCNIP3//KCNA6//DLG4//TIAM1//RTN4//EXOC4// |
| GO:0000139 | Golgi membrane | Cellular component | 37 | 729 | 543 | 18698 | 1.74771060536015 | 0.000744843325928118 | 0.0201008533849386 | 3.12793506931962 | 0.0681399631675875 | GALNT3//NUCB1//ST3GAL3//MMP24//AP1S1//CABP7//ARFIP2//VPS53//CALN1//SYS1//CD74//STEAP2//MAN1C1//KDELR3//VPS45//GBP4//CUX1//EMP2//DBNL//ST8SIA5//ST6GALNAC6//GOLGA7B//MGAT5//NCAM1//RNF125//GALNT10//SLC30A6//SLC35A2//KIAA0319L//TNKS2//NDFIP1//FAM57B//CREB3L3//PGAP3//HS6ST1//GAL3ST1//GOSR2// |
| GO:0031253 | cell projection membrane | Cellular component | 21 | 332 | 543 | 18698 | 2.17809358982893 | 0.000748809439252662 | 0.0201008533849386 | 3.1256286896579 | 0.0386740331491713 | FERMT2//DPP4//SLC27A4//AQP1//SLC5A6//NF2//PALM//TIRAP//PACSIN1//ITGA5//ARHGEF4//EPS8L2//TIAM1//TLN1//UNC5A//EVC//IQCE//SHANK3//SHISA9//EHD3//MAPRE1// |
| GO:0030133 | transport vesicle | Cellular component | 23 | 381 | 543 | 18698 | 2.07873049018044 | 0.000811468499504419 | 0.0211227709416453 | 3.09072833444149 | 0.0423572744014733 | CD74//TGOLN2//STEAP2//COPS5//DLG4//LGI3//UNC13B//LRRK2//SYT2//SYT9//ABCC8//SCAMP1//NRGN//GOSR2//SYNPR//SYPL2//ATP6V1G2//AP1S1//KDELR3//EDN1//FGFR3//NPTX1//SSPN// |
| GO:0044444 | cytoplasmic part | Cellular component | 316 | 9626 | 543 | 18698 | 1.13041145853063 | 0.000842296013587637 | 0.02128036104917 | 3.07453525472932 | 0.58195211786372 | PPP1R2//CARHSP1//CCDC103//SEPT7//TIMM23//PYURF//EMC8//TOMM34//AKAP10//LRRK2//GLYCTK//DLAT//ELK1//ALDOC//SIRT3//DDAH1//MYCBP//GLRX//SLC25A5//NFKB1//UCHL5//CREBZF//RAF1//CEP85//ABCC8//TGM2//YWHAB//MYO19//OGT//NFS1//SH3BP5//CD74//ACTR3//ACTR2//SGK2//ARL4C//FAM13A//SPRY3//CDKN1B//CDKN2A//TAB1//UNC13B//TGOLN2//CAMKK2//CPLX1//RALBP1//FERMT2//COPS5//TIRAP//AP1S1//UROC1//UBXN2B//CTH//CUX1//RNF217//UBXN2A//DLG4//DYNC1I1//DPYS//EIF4G2//EMP2//ALAD//MAPRE1//MGRN1//CRTC1//HAAO//ARFIP2//FTSJ1//LCLAT1//LMOD1//DNAJB5//SULT4A1//STEAP2//FBXL3//FBXO10//PTPN18//GNAZ//DBNL//WDR91//PACSIN1//KCNIP3//EHD3//HIC1//HMOX1//HOXC6//IDI1//ARHGAP40//IL1B//IMPA2//KIF2A//KRT2//KRT9//ARHGDIB//SMAD1//MAGEA11//NCAM1//NF2//NFATC4//SPRN//PABPC3//PAK2//ARHGEF4//PAX5//ABHD5//RLIM//C9ORF78//PGM5//PIM1//ATP6V1G2//PML//POLR2E//RHOF//CC2D1A//PARP16//TRIM68//VPS53//PPP3CB//PRKAA1//PRKAB1//LIMS2//MAP7D1//PARVA//BRK1//RNF114//MAPK4//GRIPAP1//SH3GLB2//TULP4//KLHL14//WDFY1//RANBP10//CALCOCO1//PEX5//TP53INP2//PCTP//RALGDS//CPLX3//REL//EXOC4//BCR//S100A12//RNF123//SFRP1//SH3GL1//EPS8L2//SKP1//UBE2Z//SNRPB//SPTBN2//TRIM21//TIAM1//TLN1//UBE2D3//UBE2E1//UCK2//BEST1//VSNL1//WAS//CA7//MIS12//MAPKAP1//CENPO//RPAP3//ZNF322//PIP4K2C//NUP214//CCDC6//TNKS2//FOSL1//URM1//KRTAP1-1//UCK1//CASP7//FAM160A2//MEX3B//RNF135//DDI2//ACBD6//PARD6B//CREB3L3//KLHL22//FBXL20//PARG//SHANK3//TRIM5//DIXDC1//PLA2G4C//KRT38//ALDH1A2//SYS1//PAXBP1//ARHGEF6//VPS26A//GOSR2//RBM8A//OXSR1//AGPAT1//EBP//KDELR3//FGFR3//VASH1//ESYT1//CYP2S1//LRRTM1//ITGA5//MEST//REEP2//DNAJB12//LRRC59//RGMA//MAN1C1//APMAP//RTN4//THBS1//SLC35A2//NPHS2//MOGAT2//EDEM3//FAM57B//NUCB1//VPS45//DBI//FGFR2//GALNT3//MGAT5//GALNT10//SLC30A6//RSC1A1//TGM4//NDFIP1//JAM3//CXCL14//SNPH//GPD2//NRBF2//SLC39A2//PALM//KIAA0319L//GBP4//TAF8//FAM168B//CABP7//MLPH//CALN1//AATK//SLMAP//KCNQ5//SMPD1//EDN1//NPTX1//SSPN//DPP4//LARP1//EXD1//RBM3//NRGN//ST8SIA5//ST6GALNAC6//GOLGA7B//RNF125//ST3GAL3//PGAP3//HS6ST1//GAL3ST1//GPX8//FAXDC2//SLC27A4//CYP2C18//CYP4F3//ASNA1//PAFAH2//INSIG2//SLC10A7//CYP4F2//EMC10//SNX19//BIN2//CTNS//MAEA//MYOM3//BMP10//SCN3B//SCN1A//POLR2M//FAM92B//MMP24//DRAM1//SHKBP1//KIT//CD79A//AVL9//CYB561A3//VLDLR//LGI3//SCAMP1//SYT9//PTAFR//SYT2//FZD2//TSPAN14//BSN//GPNMB//SNN//LETM2//TIMM22//CHDH//SLC25A2//CABS1//RBFOX1//MRC1//SYNPR//SYPL2//TOMM40L//LGALS3BP//FERMT3//SKIL// |
| GO:0030863 | cortical cytoskeleton | Cellular component | 10 | 105 | 543 | 18698 | 3.27948785407349 | 0.000958589252980083 | 0.0235265190945683 | 3.01836744431733 | 0.0184162062615101 | NF2//MLPH//POLR2M//MAPRE1//BSN//MAEA//SPTBN2//ACTR2//WAS//DLG4// |
| GO:0014069 | postsynaptic density | Cellular component | 16 | 227 | 543 | 18698 | 2.42710995367553 | 0.00101587554275396 | 0.0242399192007125 | 2.99315949520763 | 0.0294659300184162 | DLG4//GRIN2B//ACTR2//CHRM1//CHRNA3//FBXO45//DBNL//SH2D5//NRGN//PALM//RTN4//BCR//TIAM1//SHISA9//SHANK3//BSN// |
| GO:0031256 | leading edge membrane | Cellular component | 13 | 165 | 543 | 18698 | 2.71303086109716 | 0.0010814223096706 | 0.0245396197418007 | 2.96600467520159 | 0.0239410681399632 | FERMT2//DPP4//TIRAP//PACSIN1//ITGA5//NF2//ARHGEF4//EPS8L2//TIAM1//TLN1//UNC5A//PALM//SHISA9// |
| GO:0044297 | cell body | Cellular component | 28 | 513 | 543 | 18698 | 1.87947257134036 | 0.0011114387490398 | 0.0245396197418007 | 2.95411446610669 | 0.0515653775322284 | CPLX1//CHRNA3//LRRK2//ELK1//CRTC1//NPTXR//ASCL1//NPTX2//NRGN//RTN4//KLHL14//PURA//SCN1A//SLC6A3//SPTBN2//CNTN2//TIAM1//FZD3//POLR2M//ZNF804A//SNPH//RBM8A//UNC5A//CCK//CTNND2//GNAO1//GNAZ//NF2// |
| GO:0099572 | postsynaptic specialization | Cellular component | 16 | 229 | 543 | 18698 | 2.40591248683121 | 0.00111413873100143 | 0.0245396197418007 | 2.95306072805178 | 0.0294659300184162 | ACTR2//CHRM1//CHRNA3//DLG4//FBXO45//DBNL//GRIN2B//SH2D5//NRGN//PALM//RTN4//BCR//TIAM1//SHISA9//SHANK3//BSN// |
| GO:0044448 | cell cortex part | Cellular component | 13 | 166 | 543 | 18698 | 2.69668730169296 | 0.00114302819778861 | 0.0245465305475104 | 2.94194305570013 | 0.0239410681399632 | EXOC4//MAEA//SEPT7//SPTBN2//ACTR2//DLG4//NF2//MLPH//POLR2M//MAPRE1//BSN//WAS//UNC13B// |
| GO:0032279 | asymmetric synapse | Cellular component | 16 | 232 | 543 | 18698 | 2.37480154950149 | 0.00127655328222582 | 0.0267453480349263 | 2.89396105340675 | 0.0294659300184162 | ACTR2//CHRM1//CHRNA3//DLG4//FBXO45//DBNL//GRIN2B//SH2D5//NRGN//PALM//RTN4//BCR//TIAM1//SHISA9//SHANK3//BSN// |
| GO:0031984 | organelle subcompartment | Cellular component | 69 | 1655 | 543 | 18698 | 1.43564287025755 | 0.00141044032161912 | 0.028846862768353 | 2.85064528493564 | 0.12707182320442 | TGOLN2//LRRK2//NUCB1//RBFOX1//SYS1//SCAMP1//PARP16//RTN4//MMP24//AP1S1//CABP7//ARFIP2//VPS53//CALN1//CD74//GALNT3//ST3GAL3//KDELR3//VPS45//GBP4//CUX1//EMP2//DBNL//ST8SIA5//ST6GALNAC6//GOLGA7B//MGAT5//NCAM1//RNF125//GALNT10//SLC30A6//MAN1C1//SLC35A2//KIAA0319L//TNKS2//NDFIP1//FAM57B//CREB3L3//PGAP3//HS6ST1//GAL3ST1//GOSR2//PYURF//AGPAT1//EBP//FAXDC2//SLC27A4//CYP2C18//ESYT1//LCLAT1//CYP2S1//HMOX1//CYP4F3//MEST//ASNA1//PAFAH2//INSIG2//REEP2//LRRC59//KLHL14//MOGAT2//SLC10A7//CYP4F2//PLA2G4C//PML//STEAP2//DNAJB12//EMC8//EMC10// |
| GO:0098984 | neuron to neuron synapse | Cellular component | 16 | 235 | 543 | 18698 | 2.34448493397594 | 0.00145851429195429 | 0.0291363669020636 | 2.83608931087789 | 0.0294659300184162 | ACTR2//CHRM1//CHRNA3//DLG4//FBXO45//DBNL//GRIN2B//SH2D5//NRGN//PALM//RTN4//BCR//TIAM1//SHISA9//SHANK3//BSN// |
| GO:0043025 | neuronal cell body | Cellular component | 25 | 449 | 543 | 18698 | 1.91729523762648 | 0.00153517981234029 | 0.0299708967909161 | 2.81384074922025 | 0.0460405156537753 | UNC5A//CCK//LRRK2//CTNND2//CPLX1//CHRNA3//ELK1//CRTC1//NPTXR//ASCL1//NPTX2//NRGN//RTN4//KLHL14//PURA//SCN1A//SLC6A3//SPTBN2//CNTN2//TIAM1//FZD3//POLR2M//ZNF804A//SNPH//RBM8A// |
| GO:0031224 | intrinsic component of membrane | Cellular component | 191 | 5506 | 543 | 18698 | 1.19451741579084 | 0.00197236445846206 | 0.0376502459959758 | 2.70501283195355 | 0.351749539594843 | MUC22//GPNMB//AGPAT1//TGOLN2//FAXDC2//SLC26A1//TOMM34//SLC27A4//KDELR3//PTPRT//VPS45//VSIG4//SMIM12//CHRNA3//PIK3IP1//TM4SF18//ANO4//TMEM132E//TEDDM1//SYT2//FAM168B//TPRA1//LETM2//SYT9//PRIMA1//CTNS//RNF217//CCDC167//CABP7//DPP4//EMP2//EVC//CYB561A3//FGFR2//P2RX2//AVL9//FAM189A1//NPTXR//FZD2//LCLAT1//SYT14//FAM19A5//GALNT3//GPR160//C16ORF54//EMC10//RNF149//HTR3E//SLC25A5//ST8SIA5//TIMM22//ST6GALNAC6//CD209//XKR7//LRRTM1//KCNA6//KIT//RGS9BP//CYP4F3//SMAD1//MEST//MGAT5//SMCO3//NCAM1//GPX8//PLLP//SEMA5B//PARP16//DRAM1//LRRC59//GALNT10//SLC30A6//SCN3B//KCNQ5//PRSS8//APMAP//PTAFR//LRRN1//SEMA4G//MS4A7//ANO3//SFRP1//ST3GAL3//SLC5A3//SLC6A3//SLC9A5//SLC35E2B//SLC35A2//VLDLR//BEST1//NKAIN1//FZD3//KIAA0319L//MOGAT2//NDFIP1//SNN//CALN1//JAM3//FAM57B//SLC25A2//KREMEN1//SLC10A7//TMEM164//TMEM246//CREB3L3//TMEFF1//TNFSF14//PPP1R3F//CHRFAM7A//UNC5A//KCNQ4//PGAP3//CD22//FAM189A2//SCAMP1//GOSR2//AATK//CD74//SNPH//SLC23A2//LYPD6//EFNA3//SPRN//NRN1//RGMA//CNTN2//SEMA7A//VNN2//TGM2//ESYT1//PTPRU//CD96//MAEA//EBP//SLC12A7//MMP24//CHRM1//FGFR3//ATP1B4//STEAP2//GPR3//GPR34//GRIN2B//GRM2//SLC39A2//IL4R//AQP1//KCNJ12//TRABD2B//MRC1//PALM//CLEC1A//REEP2//SLC39A8//ABCC8//TMPRSS2//TNFSF4//NPHS2//SLMAP//SLC52A2//SSPN//TSPAN14//RELT//SLC5A6//TSPAN18//HS6ST1//GAL3ST1//CD79A//CD81//DNAJB12//RTN4//MAN1C1//SYS1//SYNPR//SYPL2//ATP6V1G2//TIMM23//TOMM40L//SCN1A//SCN7A//ITGA5//DLG4//KCNIP3//KCNIP2//SHISA9//SHANK3//INSIG2//EMC8//CACNG1//CACNA2D2//SESTD1// |
| GO:0071944 | cell periphery | Cellular component | 194 | 5614 | 543 | 18698 | 1.18993885976981 | 0.00214813348257774 | 0.0391753824037779 | 2.66793873556755 | 0.357274401473296 | PTPRU//ARL4C//CD96//GPNMB//UNC13B//TGOLN2//SLC12A7//SLC26A1//SLC27A4//PTPRT//CDC42EP1//AKAP10//CHRM1//CHRNA3//PIK3IP1//TIRAP//LRRK2//ANO4//SYT2//FAM168B//LYPD6//SYT9//PRIMA1//CTNS//DGKG//CABP7//DLG4//DPP4//EFNA3//EMP2//EWSR1//FGFR3//FGFR2//P2RX2//MGRN1//ESYT1//CRTC1//NPTXR//ARFIP2//FZD2//SYT14//STEAP2//GPR160//GNAO1//GNAZ//HTR3E//DBNL//GRIN2B//GRM2//SLC39A2//PACSIN1//ST6GALNAC6//KCNIP3//KCNIP2//CD209//IL4R//AQP1//ITGA5//KCNA6//ENHO//KCNJ12//KIT//MARK1//MRC1//NCAM1//NF2//NPTX1//NPTX2//SPRN//PAK2//PALM//NRN1//PIM1//RHOF//CC2D1A//ENOX1//RALGPS2//PPP3CB//LIMS2//PARVA//SCN3B//KMT2E//RNF114//KCNQ5//PRSS8//RGMA//RTN4//PTAFR//TBC1D24//SEMA4G//RAF1//CPLX3//EXOC4//RSC1A1//S100A12//SCN1A//SCN7A//ANO3//SLC39A8//SFRP1//EPS8L2//SLC5A3//SLC6A3//SLC9A5//SMPD1//ABCC8//CNTN2//TIAM1//TMPRSS2//TNFSF4//UBE2D3//VLDLR//BEST1//NPHS2//CACNG1//MAPKAP1//NKAIN1//SLC52A2//FZD3//KIAA0319L//TSPAN14//CCDC70//CALN1//JAM3//KREMEN1//SLC10A7//PARD6B//OGT//SEMA7A//SHANK3//TMEFF1//TNFSF14//VNN2//SLC5A6//UNC5A//KCNQ4//ZNF804A//CACNA2D2//CD22//SCAMP1//CD74//CD79A//CD81//SLC23A2//FERMT2//BIN2//NDFIP1//PLA2G4C//KRT2//EVPLL//PGM5//SLMAP//SSPN//BSN//MAEA//EBP//MMP24//ATP1B4//GPR3//GPR34//SLC25A5//TRABD2B//CLEC1A//REEP2//RELT//CHRFAM7A//TSPAN18//HS6ST1//GAL3ST1//HMOX1//SHISA9//THBS1//PRKAA1//SPTBN2//CYP4F2//SEPT7//EHD3//TGM2//MAPRE1//ARHGEF4//TLN1//FBXO45//FOSL1//SNPH//LRRTM1//SH2D5//IQSEC3//BCR//EVC//IQCE//ACTR2//WAS//MLPH//POLR2M// |
| GO:0030672 | synaptic vesicle membrane | Cellular component | 9 | 98 | 543 | 18698 | 3.16236328785658 | 0.00218907841138689 | 0.0391753824037779 | 2.65973868200322 | 0.0165745856353591 | SYNPR//SYPL2//ATP6V1G2//UNC13B//LRRK2//SYT2//SYT9//ABCC8//SCAMP1// |
| GO:0099501 | exocytic vesicle membrane | Cellular component | 9 | 98 | 543 | 18698 | 3.16236328785658 | 0.00218907841138689 | 0.0391753824037779 | 2.65973868200322 | 0.0165745856353591 | UNC13B//LRRK2//SYT2//SYT9//ABCC8//SCAMP1//SYNPR//SYPL2//ATP6V1G2// |
| GO:0012505 | endomembrane system | Cellular component | 157 | 4421 | 543 | 18698 | 1.2228535913685 | 0.00235667400913 | 0.0413139382416871 | 2.62770048783096 | 0.289134438305709 | EBP//ATP1B4//GNAZ//PARP16//LRRC59//RTN4//TNKS2//POLR2M//PLA2G4C//CDKN1B//TGOLN2//LRRK2//STEAP2//PACSIN1//SMPD1//VPS26A//AGPAT1//KDELR3//UBXN2B//UBXN2A//DLG4//FGFR3//VASH1//ESYT1//LCLAT1//CYP2S1//KCNIP3//HMOX1//LRRTM1//ITGA5//MEST//REEP2//DNAJB12//RGMA//MAN1C1//APMAP//KLHL14//TGM2//THBS1//SLC35A2//NPHS2//MOGAT2//EDEM3//FAM57B//CREB3L3//UNC13B//VPS45//AP1S1//GLYCTK//CUX1//DBI//EMP2//FGFR2//MAPRE1//GALNT3//MGAT5//TRIM68//VPS53//GALNT10//SLC30A6//PEX5//RAF1//RSC1A1//CEP85//TGM4//MAPKAP1//NDFIP1//JAM3//CXCL14//GOSR2//EDN1//NPTX1//SSPN//IL1B//VLDLR//TRIM5//MGRN1//DBNL//NF2//NUCB1//WDFY1//CTNS//CD74//CD79A//DYNC1I1//AVL9//GRIPAP1//SLMAP//COPS5//LGI3//KIT//SKIL//SYT9//AQP1//RNF123//WDR91//SNX19//PML//SH3GL1//CYB561A3//RHOF//PTAFR//ALAD//ALDOC//NFKB1//BIN2//S100A12//SYT2//ABCC8//SCAMP1//NRGN//FERMT3//PYURF//FAXDC2//SLC27A4//CYP2C18//CYP4F3//ASNA1//PAFAH2//INSIG2//SLC10A7//CYP4F2//TSPAN14//ACTR2//LGALS3BP//EHD3//SMAD1//TAB1//MRC1//UBE2D3//GPX8//PGAP3//RNF125//EMC8//EMC10//GBP4//ST8SIA5//ST6GALNAC6//GOLGA7B//NCAM1//ST3GAL3//KIAA0319L//HS6ST1//GAL3ST1//RBFOX1//SYS1//MMP24//CABP7//ARFIP2//CALN1//NUP214//P2RX2//SYNPR//SYPL2//ATP6V1G2//CDC42EP1//RABL3// |
| GO:0002102 | podosome | Cellular component | 5 | 34 | 543 | 18698 | 5.06391506878995 | 0.0028113747210829 | 0.0482994177082042 | 2.55108126451262 | 0.00920810313075507 | ACTR2//DBNL//BIN2//SH3GL1//FERMT3// |
| GO:0032588 | trans-Golgi network membrane | Cellular component | 8 | 85 | 543 | 18698 | 3.24090564402557 | 0.00325986093308813 | 0.0549062851278962 | 2.48680092670528 | 0.0147329650092081 | MMP24//AP1S1//CABP7//ARFIP2//VPS53//CALN1//SYS1//CD74// |
| GO:0044431 | Golgi apparatus part | Cellular component | 42 | 935 | 543 | 18698 | 1.54679587555766 | 0.003453339265373 | 0.0570465082491424 | 2.46176075329214 | 0.0773480662983425 | KDELR3//VPS45//GBP4//AP1S1//CUX1//EMP2//GALNT3//DBNL//ST8SIA5//ST6GALNAC6//GOLGA7B//MGAT5//NCAM1//RNF125//GALNT10//SLC30A6//MAN1C1//ST3GAL3//SLC35A2//KIAA0319L//TNKS2//NDFIP1//FAM57B//CREB3L3//PGAP3//HS6ST1//GAL3ST1//GOSR2//CD74//VPS53//UBXN2A//NUCB1//MMP24//CABP7//ARFIP2//CALN1//SYS1//STEAP2//TGOLN2//LRRK2//RBFOX1//SCAMP1// |
| GO:0016021 | integral component of membrane | Cellular component | 184 | 5369 | 543 | 18698 | 1.18010253940585 | 0.00439701872130564 | 0.0712563747008887 | 2.35684168526898 | 0.338858195211786 | PTPRU//CD96//MAEA//GPNMB//EBP//SLC12A7//SLC26A1//MMP24//CHRM1//CHRNA3//EFNA3//FGFR3//FGFR2//P2RX2//ATP1B4//STEAP2//GPR3//HTR3E//GPR34//GRIN2B//GRM2//SLC25A5//SLC39A2//IL4R//AQP1//KCNA6//KCNJ12//KIT//TRABD2B//MRC1//PALM//CLEC1A//REEP2//KCNQ5//PTAFR//SLC39A8//SLC5A3//SLC6A3//ABCC8//CNTN2//TMPRSS2//TNFSF4//NPHS2//SLMAP//SLC52A2//SSPN//TSPAN14//RELT//SLC5A6//CHRFAM7A//TSPAN18//HS6ST1//GAL3ST1//CD79A//CD81//SLC23A2//TOMM40L//ESYT1//DNAJB12//RTN4//CD74//MAN1C1//SYS1//SYNPR//SYPL2//ATP6V1G2//TIMM23//SCN3B//SCN1A//SCN7A//ITGA5//DLG4//KCNIP3//KCNIP2//KCNQ4//SHISA9//SHANK3//INSIG2//EMC8//EMC10//CACNG1//CACNA2D2//SESTD1//BEST1//MUC22//AGPAT1//TGOLN2//FAXDC2//TOMM34//SLC27A4//KDELR3//PTPRT//VPS45//VSIG4//SMIM12//PIK3IP1//TM4SF18//ANO4//TMEM132E//TEDDM1//SYT2//FAM168B//TPRA1//LETM2//SYT9//PRIMA1//CTNS//RNF217//CCDC167//CABP7//DPP4//EMP2//EVC//CYB561A3//AVL9//FAM189A1//NPTXR//FZD2//LCLAT1//SYT14//FAM19A5//GALNT3//GPR160//C16ORF54//RNF149//ST8SIA5//TIMM22//ST6GALNAC6//CD209//XKR7//LRRTM1//RGS9BP//CYP4F3//SMAD1//MEST//MGAT5//SMCO3//NCAM1//GPX8//PLLP//SEMA5B//PARP16//DRAM1//LRRC59//GALNT10//SLC30A6//PRSS8//APMAP//LRRN1//SEMA4G//MS4A7//ANO3//SFRP1//ST3GAL3//SLC9A5//SLC35E2B//SLC35A2//VLDLR//NKAIN1//FZD3//KIAA0319L//MOGAT2//NDFIP1//SNN//CALN1//JAM3//FAM57B//SLC25A2//KREMEN1//SLC10A7//TMEM164//TMEM246//CREB3L3//TMEFF1//TNFSF14//PPP1R3F//UNC5A//PGAP3//CD22//FAM189A2//SCAMP1//GOSR2//AATK//SNPH// |
| GO:0043195 | terminal bouton | Cellular component | 7 | 71 | 543 | 18698 | 3.3949627785127 | 0.00447944613952036 | 0.0712563747008887 | 2.3487756809617 | 0.0128913443830571 | UNC13B//CPLX1//AP1S1//LRRK2//TBC1D24//CPLX3//CCK// |
| GO:0032587 | ruffle membrane | Cellular component | 8 | 90 | 543 | 18698 | 3.06085533046859 | 0.00463216694310406 | 0.0723460255295707 | 2.33421579704349 | 0.0147329650092081 | TIRAP//PACSIN1//ITGA5//NF2//ARHGEF4//EPS8L2//TIAM1//TLN1// |
| GO:0031226 | intrinsic component of plasma membrane | Cellular component | 69 | 1741 | 543 | 18698 | 1.36472656535109 | 0.00485314072653392 | 0.07444371221594 | 2.31397711526394 | 0.12707182320442 | PRSS8//PTPRU//CD96//MAEA//GPNMB//EBP//SLC12A7//SLC26A1//MMP24//CHRM1//CHRNA3//EFNA3//FGFR3//FGFR2//P2RX2//ATP1B4//STEAP2//GPR3//HTR3E//GPR34//GRIN2B//GRM2//SLC25A5//SLC39A2//IL4R//AQP1//KCNA6//KCNJ12//KIT//TRABD2B//MRC1//PALM//CLEC1A//REEP2//KCNQ5//PTAFR//SLC39A8//SLC5A3//SLC6A3//ABCC8//CNTN2//TMPRSS2//TNFSF4//NPHS2//SLMAP//SLC52A2//SSPN//TSPAN14//RELT//SLC5A6//CHRFAM7A//TSPAN18//HS6ST1//GAL3ST1//CD79A//CD81//SLC23A2//SCN3B//SCN1A//SCN7A//ITGA5//DLG4//KCNIP3//KCNIP2//KCNQ4//SHISA9//SHANK3//TGM2//UNC5A// |
| GO:0008076 | voltage-gated potassium channel complex | Cellular component | 8 | 92 | 543 | 18698 | 2.99431499719753 | 0.00529071317950653 | 0.0778210255708116 | 2.27648578183101 | 0.0147329650092081 | DLG4//KCNIP3//KCNIP2//KCNA6//KCNQ5//ABCC8//CNTN2//KCNQ4// |
| GO:0099568 | cytoplasmic region | Cellular component | 24 | 468 | 543 | 18698 | 1.76587807527034 | 0.00533863818083579 | 0.0778210255708116 | 2.27256951188065 | 0.0441988950276243 | FERMT2//FGFR2//ARFIP2//DBNL//BIN2//RHOF//NDFIP1//PARD6B//PLA2G4C//UNC13B//LRRK2//DLG4//CCDC103//SEPT7//BSN//EXOC4//MAEA//SPTBN2//ACTR2//WAS//NF2//MLPH//POLR2M//MAPRE1// |
| GO:0008021 | synaptic vesicle | Cellular component | 12 | 176 | 543 | 18698 | 2.34781516825716 | 0.00541437289523267 | 0.0778210255708116 | 2.26645183708324 | 0.0220994475138122 | UNC13B//LRRK2//SYT2//SYT9//ABCC8//SCAMP1//SYNPR//SYPL2//ATP6V1G2//COPS5//DLG4//LGI3// |
| GO:0098797 | plasma membrane protein complex | Cellular component | 28 | 574 | 543 | 18698 | 1.67973768135471 | 0.00543569445197753 | 0.0778210255708116 | 2.26474496355647 | 0.0515653775322284 | GNAO1//GNAZ//SCN3B//SCN1A//SCN7A//ATP1B4//CACNG1//CACNA2D2//DLG4//KCNIP3//KCNIP2//KCNA6//KCNQ5//ABCC8//CNTN2//KCNQ4//PGM5//SSPN//SLC6A3//ITGA5//CD74//CHRNA3//CHRFAM7A//CD79A//SHISA9//SHANK3//GRIN2B//EVC// |
| GO:0005886 | plasma membrane | Cellular component | 187 | 5503 | 543 | 18698 | 1.17013890631897 | 0.00586960287670604 | 0.0826555552637785 | 2.23139128108449 | 0.344383057090239 | KRT2//EVPLL//AQP1//PGM5//ABCC8//SLMAP//SSPN//PPP3CB//SCN1A//GNAO1//GNAZ//SCN3B//SCN7A//CD81//BIN2//CD74//PRSS8//PTPRU//CD96//MAEA//GPNMB//EBP//SLC12A7//SLC26A1//MMP24//CHRM1//CHRNA3//EFNA3//FGFR3//FGFR2//P2RX2//ATP1B4//STEAP2//GPR3//HTR3E//GPR34//GRIN2B//GRM2//SLC25A5//SLC39A2//IL4R//KCNA6//KCNJ12//KIT//TRABD2B//MRC1//PALM//CLEC1A//REEP2//KCNQ5//PTAFR//SLC39A8//SLC5A3//SLC6A3//CNTN2//TMPRSS2//TNFSF4//NPHS2//SLC52A2//TSPAN14//RELT//SLC5A6//CHRFAM7A//TSPAN18//HS6ST1//GAL3ST1//CD79A//SLC23A2//ITGA5//CACNG1//CACNA2D2//HMOX1//DLG4//KCNIP3//KCNIP2//KCNQ4//SHISA9//SHANK3//CD209//NCAM1//THBS1//SEMA7A//NPTXR//NPTX2//BEST1//DPP4//EMP2//PRKAA1//SPTBN2//FZD3//PARD6B//CYP4F2//SEPT7//EHD3//TGM2//UNC5A//FERMT2//TIAM1//MAPRE1//SLC27A4//NF2//EXOC4//TIRAP//PACSIN1//ARHGEF4//EPS8L2//TLN1//UNC13B//FBXO45//FOSL1//SNPH//LRRTM1//SH2D5//IQSEC3//BCR//EVC//IQCE//LRRK2//ARL4C//TGOLN2//PTPRT//CDC42EP1//AKAP10//PIK3IP1//ANO4//SYT2//FAM168B//LYPD6//SYT9//PRIMA1//CTNS//DGKG//CABP7//EWSR1//MGRN1//ESYT1//CRTC1//ARFIP2//FZD2//SYT14//GPR160//DBNL//ST6GALNAC6//ENHO//MARK1//NPTX1//SPRN//PAK2//NRN1//PIM1//RHOF//CC2D1A//ENOX1//RALGPS2//LIMS2//PARVA//KMT2E//RNF114//RGMA//RTN4//TBC1D24//SEMA4G//RAF1//CPLX3//RSC1A1//S100A12//ANO3//SFRP1//SLC9A5//SMPD1//UBE2D3//VLDLR//MAPKAP1//NKAIN1//KIAA0319L//CCDC70//CALN1//JAM3//KREMEN1//SLC10A7//OGT//TMEFF1//TNFSF14//VNN2//ZNF804A//CD22//SCAMP1// |
| GO:0030864 | cortical actin cytoskeleton | Cellular component | 7 | 75 | 543 | 18698 | 3.21389809699202 | 0.00605677101438499 | 0.0839155855057533 | 2.21775884520229 | 0.0128913443830571 | MAEA//SPTBN2//ACTR2//WAS//NF2//MLPH//POLR2M// |
| GO:0005802 | trans-Golgi network | Cellular component | 13 | 202 | 543 | 18698 | 2.21608956475758 | 0.00626776686312811 | 0.0854605037369372 | 2.20288716599094 | 0.0239410681399632 | MMP24//AP1S1//CABP7//ARFIP2//VPS53//CALN1//SYS1//CD74//TGOLN2//LRRK2//NUCB1//RBFOX1//SCAMP1// |
| GO:0030658 | transport vesicle membrane | Cellular component | 13 | 203 | 543 | 18698 | 2.20517286739424 | 0.0065251288622221 | 0.0866420699075924 | 2.18541090719284 | 0.0239410681399632 | GOSR2//CD74//NRGN//UNC13B//LRRK2//SYT2//SYT9//ABCC8//SCAMP1//SYNPR//SYPL2//ATP6V1G2//AP1S1// |
| GO:0098794 | postsynapse | Cellular component | 23 | 450 | 543 | 18698 | 1.75999181501944 | 0.00655735598140216 | 0.0866420699075924 | 2.18327123900207 | 0.0423572744014733 | DLG4//TIAM1//SHANK3//ZNF804A//CHRM1//CHRNA3//FBXO45//GRIN2B//LRRTM1//SH2D5//IQSEC3//BCR//SHISA9//SSPN//PALM//NRGN//GRIPAP1//ACTR2//DBNL//RTN4//BSN//LRRK2//P2RX2// |
| GO:0044424 | intracellular part | Cellular component | 442 | 14395 | 543 | 18698 | 1.05731873086176 | 0.0066570158485461 | 0.0866420699075924 | 2.17672040910993 | 0.813996316758748 | UBE4B//RNF217//FBXL3//FBXO10//MED1//UBE2E1//GNAO1//GNAZ//ACTR2//ARL4C//TRIB1//CD96//SPRY3//CDKN1B//CDKN2A//MAEA//EMC8//PPIH//CAMKK2//TOMM34//FERMT2//COPS5//PRDM4//CDC42EP1//RASSF1//TIRAP//LRRK2//SLAIN1//LYPD6//GLYCTK//CAPSL//NEK7//CTH//CTNND2//CYP2C18//PRUNE2//DGKG//DAPK3//DAZL//DLG4//DLX5//DYNC1I1//EDN1//TET3//EMP2//EVC//EWSR1//FGFR3//FGFR2//VASH1//KLHDC10//AVL9//MGRN1//TTC28//LARP1//CRTC1//HAAO//CARHSP1//ARFIP2//FTSJ1//FSHB//FZD2//MYCBP//HEYL//BMP10//RBMS3//DBNL//C11ORF21//CYP2S1//NRBF2//PACSIN1//ST6GALNAC6//KCNIP2//CD209//EHD3//HMGB3//TLX2//IDI1//ARHGAP40//AQP1//IMPA2//KIF2A//KIT//CCDC103//ARHGDIB//SNX19//SMAD1//MARK1//MEA1//MAP3K9//ASNA1//IQSEC3//CCDC88C//TRIM67//NF2//NFKB1//PABPC3//PAK2//FOXP3//UCHL5//PFDN1//PIM1//PML//RHOF//RBFOX1//RALGPS2//TRIM68//PPP3CB//DRAM1//PRKAA1//PARVA//MBNL3//KMT2E//MAPK4//BEX4//CELF4//SH3GLB2//PRTFDC1//TULP4//ZMIZ1//TBC1D24//WDR19//PURA//PEX5//RAF1//HIVEP3//RBM3//EXOC4//S100A12//RNF123//SH3GL1//SKP1//UBE2Z//SLC6A3//HLTF//SNRPB//TRIM21//TGM4//TTK//WNT11//YWHAB//MAPKAP1//DDX39B//TNFAIP8L2//RPAP3//FZD3//TNKS2//TRIM7//SNN//CCDC70//CASP7//RNF135//KLHL22//RELT//USP45//PARG//CYP4F2//TRIM5//BHLHE40//TNFSF14//ALDH1A2//EMILIN3//ZNF804A//NREP//SH3BP5//CD79A//SNPH//RBM8A//OXSR1//SLC23A2//SATB2//NFATC4//REL//TEAD1//ALAD//PGGT1B//TIAM1//PRKAB1//C2ORF49//HOXC9//KLHL14//ATP1B4//TIMM23//LETM2//GPD2//SLC25A5//TIMM22//CHDH//SLC25A2//CABS1//POLR2E//OGT//MYO19//AP1S1//CTNS//NOVA1//PALM//PAX5//RNF125//GRIPAP1//CALCOCO1//NUP214//PPP1R2//SEPT7//PYURF//AKAP10//DLAT//ELK1//ALDOC//SIRT3//DDAH1//GLRX//CREBZF//CEP85//ABCC8//TGM2//NFS1//CD74//ACTR3//SGK2//FAM13A//TAB1//UNC13B//TGOLN2//CPLX1//RALBP1//UROC1//UBXN2B//CUX1//UBXN2A//DPYS//EIF4G2//MAPRE1//LCLAT1//LMOD1//DNAJB5//SULT4A1//STEAP2//PTPN18//WDR91//KCNIP3//HIC1//HMOX1//HOXC6//IL1B//KRT2//KRT9//MAGEA11//NCAM1//SPRN//ARHGEF4//ABHD5//RLIM//C9ORF78//PGM5//ATP6V1G2//CC2D1A//PARP16//VPS53//LIMS2//MAP7D1//BRK1//RNF114//WDFY1//RANBP10//TP53INP2//PCTP//RALGDS//CPLX3//BCR//SFRP1//EPS8L2//SPTBN2//TLN1//UBE2D3//UCK2//BEST1//VSNL1//WAS//CA7//MIS12//CENPO//ZNF322//PIP4K2C//CCDC6//FOSL1//URM1//KRTAP1-1//UCK1//FAM160A2//MEX3B//DDI2//ACBD6//PARD6B//CREB3L3//FBXL20//SHANK3//DIXDC1//PLA2G4C//KRT38//SYS1//PAXBP1//ARHGEF6//VPS26A//GOSR2//AGPAT1//EBP//KDELR3//ESYT1//LRRTM1//ITGA5//MEST//REEP2//DNAJB12//LRRC59//RGMA//MAN1C1//APMAP//RTN4//THBS1//SLC35A2//NPHS2//MOGAT2//EDEM3//FAM57B//NUCB1//VPS45//DBI//GALNT3//MGAT5//GALNT10//SLC30A6//RSC1A1//NDFIP1//JAM3//CXCL14//SLC39A2//KIAA0319L//GBP4//TAF8//FAM168B//CABP7//MLPH//CALN1//AATK//GPX8//BIN2//FBXO45//ZSWIM6//DCAF16//POLR2M//SMPD1//SHKBP1//ASCL1//NFYA//SLMAP//MED6//ARID3B//RPP14//ZNF618//LSM11//PASD1//RNF38//FOXG1//FOXS1//ZBTB43//OTP//ZNF281//LDOC1//RAX//MCIDAS//FOXI2//VWA5A//MYBL2//NRGN//PHF20L1//USP37//RAG1//PRDM12//RFX4//GPBP1L1//TCF19//TCF20//PAX8//NRIP1//BSN//ONECUT2//CREB5//MICAL2//KCNQ5//NPTX1//SSPN//DPP4//SCAMP1//GPNMB//EXD1//FERMT3//PTBP1//SRSF9//PPP1R26//FAM92B//SESTD1//PHOX2A//ASF1B//CHTF8//LIN9//THUMPD1//SCN1A//SKIL//SNRPA//U2AF1//MYOM3//CGNL1//ST8SIA5//GOLGA7B//ST3GAL3//PGAP3//HS6ST1//GAL3ST1//MMP24//FAXDC2//SLC27A4//CYP4F3//PAFAH2//INSIG2//SLC10A7//EMC10//SYT9//PTAFR//MRC1//SYNPR//SYPL2//SCN3B//LGALS3BP//CCDC97//CYB561A3//VLDLR//LGI3//SYT2//TSPAN14//P2RX2//TOMM40L// |
| GO:0005887 | integral component of plasma membrane | Cellular component | 66 | 1678 | 543 | 18698 | 1.35440112209352 | 0.00691437157746369 | 0.0881874849397727 | 2.16024728523534 | 0.121546961325967 | SCN3B//SCN1A//SCN7A//ITGA5//ATP1B4//CHRNA3//CHRFAM7A//DLG4//KCNIP3//KCNIP2//KCNA6//KCNQ5//ABCC8//CNTN2//KCNQ4//SHISA9//SHANK3//GRIN2B//PTPRU//CD96//MAEA//GPNMB//EBP//SLC12A7//SLC26A1//MMP24//CHRM1//EFNA3//FGFR3//FGFR2//P2RX2//STEAP2//GPR3//HTR3E//GPR34//GRM2//SLC25A5//SLC39A2//IL4R//AQP1//KCNJ12//KIT//TRABD2B//MRC1//PALM//CLEC1A//REEP2//PTAFR//SLC39A8//SLC5A3//SLC6A3//TMPRSS2//TNFSF4//NPHS2//SLMAP//SLC52A2//SSPN//TSPAN14//RELT//SLC5A6//TSPAN18//HS6ST1//GAL3ST1//CD79A//CD81//SLC23A2// |
| GO:0001518 | voltage-gated sodium channel complex | Cellular component | 3 | 14 | 543 | 18698 | 7.37884767166535 | 0.00698108146205418 | 0.0881874849397727 | 2.15607729419326 | 0.00552486187845304 | SCN3B//SCN1A//SCN7A// |
| GO:0005794 | Golgi apparatus | Cellular component | 60 | 1501 | 543 | 18698 | 1.37646725387495 | 0.00713261102580638 | 0.0887958387125751 | 2.14675145941575 | 0.110497237569061 | KDELR3//VPS45//GBP4//AP1S1//CUX1//EMP2//GALNT3//DBNL//ST8SIA5//ST6GALNAC6//GOLGA7B//MGAT5//NCAM1//RNF125//GALNT10//SLC30A6//MAN1C1//ST3GAL3//SLC35A2//KIAA0319L//TNKS2//NDFIP1//FAM57B//CREB3L3//PGAP3//HS6ST1//GAL3ST1//GOSR2//CD74//VPS53//UBXN2A//NUCB1//TGOLN2//LRRK2//RBFOX1//SYS1//SCAMP1//STEAP2//MMP24//CABP7//ARFIP2//CALN1//UNC13B//GLYCTK//UBXN2B//DBI//FGFR3//FGFR2//MAPRE1//KCNIP3//ITGA5//TRIM68//PEX5//RAF1//RSC1A1//CEP85//TGM4//MAPKAP1//JAM3//CXCL14// |
| GO:0034705 | potassium channel complex | Cellular component | 8 | 99 | 543 | 18698 | 2.78259575497145 | 0.00816987608792552 | 0.1002560508504 | 2.08778453033886 | 0.0147329650092081 | DLG4//KCNIP3//KCNIP2//KCNA6//KCNQ5//ABCC8//CNTN2//KCNQ4// |
| GO:0005623 | cell | Cellular component | 507 | 16915 | 543 | 18698 | 1.03212258889508 | 0.00921374814013983 | 0.111473375385635 | 2.0355636634576 | 0.933701657458563 | PTPRU//ARL4C//CD96//GPNMB//UNC13B//TGOLN2//SLC12A7//SLC26A1//SLC27A4//PTPRT//CDC42EP1//AKAP10//CHRM1//CHRNA3//PIK3IP1//TIRAP//LRRK2//ANO4//SYT2//FAM168B//LYPD6//SYT9//PRIMA1//CTNS//DGKG//CABP7//DLG4//DPP4//EFNA3//EMP2//EWSR1//FGFR3//FGFR2//P2RX2//MGRN1//ESYT1//CRTC1//NPTXR//ARFIP2//FZD2//SYT14//STEAP2//GPR160//GNAO1//GNAZ//HTR3E//DBNL//GRIN2B//GRM2//SLC39A2//PACSIN1//ST6GALNAC6//KCNIP3//KCNIP2//CD209//IL4R//AQP1//ITGA5//KCNA6//ENHO//KCNJ12//KIT//MARK1//MRC1//NCAM1//NF2//NPTX1//NPTX2//SPRN//PAK2//PALM//NRN1//PIM1//RHOF//CC2D1A//ENOX1//RALGPS2//PPP3CB//LIMS2//PARVA//SCN3B//KMT2E//RNF114//KCNQ5//PRSS8//RGMA//RTN4//PTAFR//TBC1D24//SEMA4G//RAF1//CPLX3//EXOC4//RSC1A1//S100A12//SCN1A//SCN7A//ANO3//SLC39A8//SFRP1//EPS8L2//SLC5A3//SLC6A3//SLC9A5//SMPD1//ABCC8//CNTN2//TIAM1//TMPRSS2//TNFSF4//UBE2D3//VLDLR//BEST1//NPHS2//CACNG1//MAPKAP1//NKAIN1//SLC52A2//FZD3//KIAA0319L//TSPAN14//CCDC70//CALN1//JAM3//KREMEN1//SLC10A7//PARD6B//OGT//SEMA7A//SHANK3//TMEFF1//TNFSF14//VNN2//SLC5A6//UNC5A//KCNQ4//ZNF804A//CACNA2D2//CD22//SCAMP1//CD74//CD79A//CD81//SLC23A2//SATB2//NRIP1//KDELR3//VPS45//GBP4//AP1S1//CUX1//GALNT3//ST8SIA5//GOLGA7B//MGAT5//RNF125//GALNT10//SLC30A6//MAN1C1//ST3GAL3//SLC35A2//TNKS2//NDFIP1//FAM57B//CREB3L3//PGAP3//HS6ST1//GAL3ST1//GOSR2//UBE4B//RNF217//FBXL3//FBXO10//MED1//UBE2E1//PPP1R2//CARHSP1//CEP85//KRBOX1//SGK2//CAMKK2//DAZL//GPR3//SMAD1//CLEC1A//PARP16//PRKAA1//POLR2M//DDX39B//POLR2E//MIS12//PYURF//DYNC1I1//TTK//CENPO//SEPT7//PML//PURA//COPS5//HIC1//DLX5//PHOX2A//ASF1B//CALCOCO1//RASSF1//NEK7//TTC28//KIF2A//BEX4//TRIM21//MEX3B//TRIM5//VPS53//KRT2//EVPLL//PAX5//SKIL//KRT9//KRT38//FERMT2//PGM5//TLN1//RGS9BP//WDR19//BIN2//TET3//SH3GL1//FERMT3//SNRPB//CCDC103//IL1B//DRAM1//SHKBP1//VPS26A//TIMM23//EMC8//TOMM34//GLYCTK//DLAT//ELK1//ALDOC//SIRT3//DDAH1//MYCBP//GLRX//SLC25A5//NFKB1//UCHL5//CREBZF//TGM2//YWHAB//MYO19//NFS1//SH3BP5//ACTR3//ACTR2//FAM13A//SPRY3//CDKN1B//CDKN2A//TAB1//CPLX1//RALBP1//UROC1//UBXN2B//CTH//UBXN2A//DPYS//EIF4G2//ALAD//MAPRE1//HAAO//FTSJ1//LCLAT1//LMOD1//DNAJB5//SULT4A1//PTPN18//WDR91//EHD3//HMOX1//HOXC6//IDI1//ARHGAP40//IMPA2//ARHGDIB//MAGEA11//NFATC4//PABPC3//ARHGEF4//ABHD5//RLIM//C9ORF78//ATP6V1G2//TRIM68//PRKAB1//MAP7D1//BRK1//MAPK4//GRIPAP1//SH3GLB2//TULP4//KLHL14//WDFY1//RANBP10//PEX5//TP53INP2//PCTP//RALGDS//REL//BCR//RNF123//SKP1//UBE2Z//SPTBN2//UCK2//VSNL1//WAS//CA7//RPAP3//ZNF322//PIP4K2C//NUP214//CCDC6//FOSL1//URM1//KRTAP1-1//UCK1//CASP7//FAM160A2//RNF135//DDI2//ACBD6//KLHL22//FBXL20//PARG//DIXDC1//PLA2G4C//ALDH1A2//SYS1//PAXBP1//ARHGEF6//RBM8A//OXSR1//MAEA//EBP//MMP24//ATP1B4//GPR34//TRABD2B//REEP2//SLMAP//SSPN//RELT//CHRFAM7A//TSPAN18//MED6//TRIB1//ARID3B//RPP14//ZNF618//TAF8//LSM11//PASD1//CTNND2//RNF38//DAPK3//FOXG1//FOXS1//ZBTB43//OTP//ZNF281//LDOC1//HEYL//RAX//HMGB3//TLX2//MCIDAS//FOXI2//VWA5A//ASCL1//ASNA1//MYBL2//NFYA//NOVA1//NRGN//NUCB1//FOXP3//PHF20L1//RBFOX1//MBNL3//CELF4//USP37//RAG1//HIVEP3//PRDM12//RBM3//RFX4//GPBP1L1//HLTF//TCF19//TCF20//TEAD1//PAX8//TRIM7//USP45//BHLHE40//BSN//NREP//ONECUT2//CREB5//MICAL2//LRRC59//PPIH//PRDM4//SLAIN1//CAPSL//CYP2C18//PRUNE2//EDN1//EVC//VASH1//KLHDC10//AVL9//LARP1//FSHB//BMP10//RBMS3//C11ORF21//CYP2S1//NRBF2//SNX19//MEA1//MAP3K9//IQSEC3//CCDC88C//TRIM67//PFDN1//PRTFDC1//ZMIZ1//TGM4//WNT11//TNFAIP8L2//SNN//CYP4F2//EMILIN3//SNPH//LIN9//HOXC9//DCAF16//CHTF8//THUMPD1//PTBP1//SNRPA//U2AF1//C2ORF49//SRSF9//CCDC97//PPP1R26//TOMM40L//LETM2//GPD2//TIMM22//CHDH//SLC25A2//CABS1//CYB561A3//AGPAT1//LRRTM1//MEST//DNAJB12//APMAP//THBS1//MOGAT2//EDEM3//DBI//GPX8//FAXDC2//CYP4F3//PAFAH2//INSIG2//CXCL14//FAM92B//MYOM3//UBXN10//IQCE//PGGT1B//LGI3//SHISA9//FBXO45//NDP//RABL3//SH2D5//CGNL1//SYNPR//SYPL2//CCK//MLPH//LGALS3BP//ZSWIM6//EXD1//PLLP//SESTD1//AATK//EMC10//TTL//TAC4// |
| GO:0098588 | bounding membrane of organelle | Cellular component | 77 | 2040 | 543 | 18698 | 1.29973820098942 | 0.0096884712821586 | 0.114511682786598 | 2.01374474370246 | 0.141804788213628 | KDELR3//VPS45//GBP4//AP1S1//CUX1//EMP2//GALNT3//DBNL//ST8SIA5//ST6GALNAC6//GOLGA7B//MGAT5//NCAM1//RNF125//GALNT10//SLC30A6//MAN1C1//ST3GAL3//SLC35A2//KIAA0319L//TNKS2//NDFIP1//FAM57B//CREB3L3//PGAP3//HS6ST1//GAL3ST1//GOSR2//CD74//CTNS//SYT9//RHOF//PTAFR//TAB1//STEAP2//EHD3//MRC1//VPS53//UBE2D3//VPS26A//DLG4//EVC//IQCE//SHANK3//NUCB1//MMP24//CABP7//ARFIP2//CALN1//SYS1//NRGN//TSPAN14//SCAMP1//SYT2//WDR91//SNX19//PML//GRIPAP1//SH3GL1//CYB561A3//TGOLN2//FZD2//PEX5//TOMM34//LRRK2//RAF1//MYO19//SNN//DPP4//DRAM1//VLDLR//UNC13B//ABCC8//ATP6V1G2//TOMM40L//SYNPR//SYPL2// |
| GO:0070382 | exocytic vesicle | Cellular component | 12 | 190 | 543 | 18698 | 2.17481826112242 | 0.00973149341492624 | 0.114511682786598 | 2.0118205068958 | 0.0220994475138122 | COPS5//DLG4//LGI3//UNC13B//LRRK2//SYT2//SYT9//ABCC8//SCAMP1//SYNPR//SYPL2//ATP6V1G2// |
| GO:0005622 | intracellular | Cellular component | 448 | 14673 | 543 | 18698 | 1.05136719590825 | 0.0104494794769855 | 0.121298687442305 | 1.98090534265371 | 0.825046040515654 | SATB2//NRIP1//OGT//KDELR3//VPS45//GBP4//AP1S1//CUX1//EMP2//GALNT3//DBNL//ST8SIA5//ST6GALNAC6//GOLGA7B//MGAT5//NCAM1//RNF125//GALNT10//SLC30A6//MAN1C1//ST3GAL3//SLC35A2//KIAA0319L//TNKS2//NDFIP1//FAM57B//CREB3L3//PGAP3//HS6ST1//GAL3ST1//GOSR2//CD74//EXOC4//UBE4B//RNF217//FBXL3//FBXO10//MED1//UBE2E1//PPP1R2//CARHSP1//CEP85//GNAO1//GNAZ//DDX39B//POLR2E//MIS12//PYURF//DYNC1I1//TTK//CENPO//SEPT7//PML//PURA//COPS5//HIC1//KMT2E//DLX5//PHOX2A//ASF1B//CALCOCO1//RASSF1//NEK7//TTC28//KIF2A//BEX4//TRIM21//MEX3B//TRIM5//VPS53//PAX5//CC2D1A//KIT//SKIL//KRT2//KRT9//KRT38//FERMT2//PGM5//TET3//BIN2//SH3GL1//FERMT3//SNRPB//CCDC103//LRRK2//CTNS//IL1B//DRAM1//SMPD1//SHKBP1//VPS26A//TIMM23//EMC8//TOMM34//AKAP10//GLYCTK//DLAT//ELK1//ALDOC//SIRT3//DDAH1//MYCBP//GLRX//SLC25A5//NFKB1//UCHL5//CREBZF//RAF1//ABCC8//TGM2//YWHAB//MYO19//NFS1//SH3BP5//ACTR3//ACTR2//SGK2//ARL4C//FAM13A//SPRY3//CDKN1B//CDKN2A//TAB1//UNC13B//TGOLN2//CAMKK2//CPLX1//RALBP1//TIRAP//UROC1//UBXN2B//CTH//UBXN2A//DLG4//DPYS//EIF4G2//ALAD//MAPRE1//MGRN1//CRTC1//HAAO//ARFIP2//FTSJ1//LCLAT1//LMOD1//DNAJB5//SULT4A1//STEAP2//PTPN18//WDR91//PACSIN1//KCNIP3//EHD3//HMOX1//HOXC6//IDI1//ARHGAP40//IMPA2//ARHGDIB//SMAD1//MAGEA11//NF2//NFATC4//SPRN//PABPC3//PAK2//ARHGEF4//ABHD5//RLIM//C9ORF78//PIM1//ATP6V1G2//RHOF//PARP16//TRIM68//PPP3CB//PRKAA1//PRKAB1//LIMS2//MAP7D1//PARVA//BRK1//RNF114//MAPK4//GRIPAP1//SH3GLB2//TULP4//KLHL14//WDFY1//RANBP10//PEX5//TP53INP2//PCTP//RALGDS//CPLX3//REL//BCR//S100A12//RNF123//SFRP1//EPS8L2//SKP1//UBE2Z//SPTBN2//TIAM1//TLN1//UBE2D3//UCK2//BEST1//VSNL1//WAS//CA7//MAPKAP1//RPAP3//ZNF322//PIP4K2C//NUP214//CCDC6//FOSL1//URM1//KRTAP1-1//UCK1//CASP7//FAM160A2//RNF135//DDI2//ACBD6//PARD6B//KLHL22//FBXL20//PARG//SHANK3//DIXDC1//PLA2G4C//ALDH1A2//SYS1//PAXBP1//ARHGEF6//RBM8A//OXSR1//MED6//TRIB1//MAEA//ARID3B//RPP14//ZNF618//TAF8//LSM11//PASD1//CTNND2//RNF38//DAPK3//DAZL//EWSR1//FGFR3//FGFR2//FOXG1//FOXS1//ZBTB43//ATP1B4//OTP//ZNF281//LDOC1//HEYL//RAX//HMGB3//TLX2//MCIDAS//AQP1//FOXI2//VWA5A//ASCL1//ASNA1//MYBL2//NFYA//NOVA1//NRGN//NUCB1//PALM//FOXP3//PHF20L1//RBFOX1//MBNL3//CELF4//USP37//RAG1//HIVEP3//PRDM12//RBM3//RFX4//GPBP1L1//HLTF//TCF19//TCF20//TEAD1//PAX8//TRIM7//RELT//USP45//BHLHE40//BSN//ZNF804A//NREP//ONECUT2//CREB5//MICAL2//EBP//LRRC59//RTN4//POLR2M//P2RX2//CD96//PPIH//PRDM4//CDC42EP1//SLAIN1//LYPD6//CAPSL//CYP2C18//PRUNE2//DGKG//EDN1//EVC//VASH1//KLHDC10//AVL9//LARP1//FSHB//FZD2//BMP10//RBMS3//C11ORF21//CYP2S1//NRBF2//KCNIP2//CD209//SNX19//MARK1//MEA1//MAP3K9//IQSEC3//CCDC88C//TRIM67//PFDN1//RALGPS2//PRTFDC1//ZMIZ1//TBC1D24//WDR19//SLC6A3//TGM4//WNT11//TNFAIP8L2//FZD3//SNN//CCDC70//CYP4F2//TNFSF14//EMILIN3//CD79A//SNPH//SLC23A2//LIN9//HOXC9//DCAF16//CHTF8//THUMPD1//PTBP1//RSC1A1//SCN1A//SNRPA//U2AF1//C2ORF49//SRSF9//CCDC97//PPP1R26//TOMM40L//LETM2//GPD2//TIMM22//CHDH//SLC25A2//CABS1//DPP4//CYB561A3//VLDLR//AGPAT1//ESYT1//LRRTM1//ITGA5//MEST//REEP2//DNAJB12//RGMA//APMAP//THBS1//NPHS2//MOGAT2//EDEM3//DBI//GPX8//FAXDC2//SLC27A4//CYP4F3//PAFAH2//INSIG2//SLC10A7//SLMAP//JAM3//CXCL14//SCAMP1//NPTX1//SSPN//SYT9//PTAFR//FAM92B//MYOM3//PGGT1B//LGI3//FBXO45//MRC1//CGNL1//SCN3B//KCNQ5//SYNPR//SYPL2//SYT2//MLPH//LGALS3BP//SLC39A2//ZSWIM6//MMP24//CABP7//CALN1//TSPAN14//GPNMB//EXD1//SESTD1//FAM168B//AATK//EMC10//KRBOX1//ANO4//GPR3//GRIN2B//GRM2//CLEC1A// |
| GO:0060076 | excitatory synapse | Cellular component | 4 | 30 | 543 | 18698 | 4.59128299570289 | 0.0105911232638516 | 0.121303665115314 | 1.97505797743522 | 0.00736648250460405 | DLG4//FGFR2//LRRTM1//BSN// |
| GO:0030665 | clathrin-coated vesicle membrane | Cellular component | 8 | 104 | 543 | 18698 | 2.64881711290551 | 0.0108521695663055 | 0.122658074440216 | 1.96448342895988 | 0.0147329650092081 | NRGN//FZD2//CD74//AP1S1//TGOLN2//SYT2//SYT9//DBNL// |
| GO:0097060 | synaptic membrane | Cellular component | 17 | 317 | 543 | 18698 | 1.84665167808239 | 0.0116153119968101 | 0.12957861045792 | 1.93496912020097 | 0.0313075506445672 | PALM//SHISA9//UNC13B//FBXO45//GRM2//FOSL1//SNPH//CHRM1//CHRNA3//DLG4//GRIN2B//LRRTM1//SH2D5//IQSEC3//BCR//SSPN//SHANK3// |
| GO:0030140 | trans-Golgi network transport vesicle | Cellular component | 4 | 32 | 543 | 18698 | 4.30432780847145 | 0.0132803390914874 | 0.146253990763945 | 1.87679083583534 | 0.00736648250460405 | NRGN//AP1S1//TGOLN2//STEAP2// |
| GO:0030285 | integral component of synaptic vesicle membrane | Cellular component | 3 | 18 | 543 | 18698 | 5.73910374462861 | 0.0143606655567971 | 0.156149515358085 | 1.84282543189412 | 0.00552486187845304 | SYNPR//SYPL2//ATP6V1G2// |
| GO:0030136 | clathrin-coated vesicle | Cellular component | 11 | 179 | 543 | 18698 | 2.11609411813122 | 0.0156677265864832 | 0.165102525598076 | 1.80499401582073 | 0.0202578268876611 | TGOLN2//STEAP2//SYT2//SYT9//DBNL//EDN1//NRGN//FZD2//CD74//AP1S1//SCAMP1// |
| GO:0044464 | cell part | Cellular component | 505 | 16886 | 543 | 18698 | 1.02981667335217 | 0.0157553918849926 | 0.165102525598076 | 1.80257079011783 | 0.930018416206261 | PTPRU//ARL4C//CD96//GPNMB//UNC13B//TGOLN2//SLC12A7//SLC26A1//SLC27A4//PTPRT//CDC42EP1//AKAP10//CHRM1//CHRNA3//PIK3IP1//TIRAP//LRRK2//ANO4//SYT2//FAM168B//LYPD6//SYT9//PRIMA1//CTNS//DGKG//CABP7//DLG4//DPP4//EFNA3//EMP2//EWSR1//FGFR3//FGFR2//P2RX2//MGRN1//ESYT1//CRTC1//NPTXR//ARFIP2//FZD2//SYT14//STEAP2//GPR160//GNAO1//GNAZ//HTR3E//DBNL//GRIN2B//GRM2//SLC39A2//PACSIN1//ST6GALNAC6//KCNIP3//KCNIP2//CD209//IL4R//AQP1//ITGA5//KCNA6//ENHO//KCNJ12//KIT//MARK1//MRC1//NCAM1//NF2//NPTX1//NPTX2//SPRN//PAK2//PALM//NRN1//PIM1//RHOF//CC2D1A//ENOX1//RALGPS2//PPP3CB//LIMS2//PARVA//SCN3B//KMT2E//RNF114//KCNQ5//PRSS8//RGMA//RTN4//PTAFR//TBC1D24//SEMA4G//RAF1//CPLX3//EXOC4//RSC1A1//S100A12//SCN1A//SCN7A//ANO3//SLC39A8//SFRP1//EPS8L2//SLC5A3//SLC6A3//SLC9A5//SMPD1//ABCC8//CNTN2//TIAM1//TMPRSS2//TNFSF4//UBE2D3//VLDLR//BEST1//NPHS2//CACNG1//MAPKAP1//NKAIN1//SLC52A2//FZD3//KIAA0319L//TSPAN14//CCDC70//CALN1//JAM3//KREMEN1//SLC10A7//PARD6B//OGT//SEMA7A//SHANK3//TMEFF1//TNFSF14//VNN2//SLC5A6//UNC5A//KCNQ4//ZNF804A//CACNA2D2//CD22//SCAMP1//CD74//CD79A//CD81//SLC23A2//KRBOX1//SGK2//CAMKK2//DAZL//GPR3//SMAD1//CLEC1A//RNF125//PARP16//PRKAA1//POLR2M//AP1S1//FERMT2//BMP10//NDP//APMAP//THBS1//TLN1//BSN//RABL3//TTC28//SEPT7//VPS26A//FAM92B//BIN2//SH3GL1//FERMT3//DLAT//SLC25A5//EHD3//PLLP//VASH1//EDN1//KRT2//EVPLL//PGM5//SLMAP//SSPN//PPP1R2//LRRTM1//MAEA//CPLX1//ELK1//ASCL1//NRGN//KLHL14//PURA//SPTBN2//SNPH//RBM8A//UBE4B//RNF217//FBXL3//FBXO10//MED1//UBE2E1//ACTR2//TRIB1//SPRY3//CDKN1B//CDKN2A//EMC8//PPIH//TOMM34//COPS5//PRDM4//RASSF1//SLAIN1//GLYCTK//CAPSL//NEK7//CTH//CTNND2//CYP2C18//PRUNE2//DAPK3//DLX5//DYNC1I1//TET3//EVC//KLHDC10//AVL9//LARP1//HAAO//CARHSP1//FTSJ1//FSHB//MYCBP//HEYL//RBMS3//C11ORF21//CYP2S1//NRBF2//HMGB3//TLX2//IDI1//ARHGAP40//IMPA2//KIF2A//CCDC103//ARHGDIB//SNX19//MEA1//MAP3K9//ASNA1//IQSEC3//CCDC88C//TRIM67//NFKB1//PABPC3//FOXP3//UCHL5//PFDN1//PML//RBFOX1//TRIM68//DRAM1//MBNL3//MAPK4//BEX4//CELF4//SH3GLB2//PRTFDC1//TULP4//ZMIZ1//WDR19//PEX5//HIVEP3//RBM3//RNF123//SKP1//UBE2Z//HLTF//SNRPB//TRIM21//TGM4//TTK//WNT11//YWHAB//DDX39B//TNFAIP8L2//RPAP3//TNKS2//TRIM7//SNN//CASP7//RNF135//KLHL22//RELT//USP45//PARG//CYP4F2//TRIM5//BHLHE40//ALDH1A2//EMILIN3//NREP//SH3BP5//OXSR1//SATB2//NFATC4//REL//TEAD1//ALAD//PGGT1B//PRKAB1//C2ORF49//TGM2//SKIL//RNF38//RGS9BP//LGI3//FBXO45//SH2D5//BCR//SHISA9//SYNPR//FOSL1//CHRFAM7A//CCK//ACTR3//RALGDS//GPD2//FBXL20//ZSWIM6//DCAF16//NDFIP1//PLA2G4C//NFYA//HOXC9//GRIPAP1//MLPH//ATP1B4//TIMM23//LETM2//TIMM22//CHDH//SLC25A2//CABS1//POLR2E//EBP//MMP24//GPR34//TRABD2B//REEP2//TSPAN18//HS6ST1//GAL3ST1//ARHGEF4//LRRC59//MYO19//UBXN10//IQCE//BRK1//ARHGEF6//PYURF//ALDOC//SIRT3//DDAH1//GLRX//CREBZF//CEP85//NFS1//MED6//ARID3B//RPP14//ZNF618//GBP4//TAF8//LSM11//UBXN2B//PASD1//CUX1//UBXN2A//FOXG1//FOXS1//ZBTB43//OTP//ZNF281//LDOC1//DNAJB5//RAX//HMOX1//MCIDAS//KRT9//FOXI2//VWA5A//MYBL2//NOVA1//NUCB1//PHF20L1//RLIM//WDFY1//RANBP10//CALCOCO1//USP37//TP53INP2//RAG1//PRDM12//RFX4//GPBP1L1//TCF19//TCF20//SLC35A2//PAX8//MIS12//ZNF322//NUP214//NRIP1//CREB3L3//PAXBP1//ONECUT2//CREB5//MICAL2//PTBP1//SRSF9//PPP1R26//AGPAT1//KDELR3//LCLAT1//MEST//DNAJB12//MAN1C1//MOGAT2//EDEM3//FAM57B//PAX5//VPS45//DBI//MAPRE1//GALNT3//MGAT5//VPS53//GALNT10//SLC30A6//CXCL14//GOSR2//ABHD5//MAP7D1//LMOD1//CCDC6//FAM13A//TAB1//RALBP1//UROC1//DPYS//EIF4G2//SULT4A1//PTPN18//WDR91//HIC1//HOXC6//IL1B//MAGEA11//C9ORF78//ATP6V1G2//PCTP//UCK2//VSNL1//WAS//CA7//CENPO//PIP4K2C//URM1//KRTAP1-1//UCK1//FAM160A2//MEX3B//DDI2//ACBD6//DIXDC1//KRT38//SYS1//GPX8//AATK//ST8SIA5//GOLGA7B//ST3GAL3//PGAP3//FAXDC2//CYP4F3//PAFAH2//INSIG2//MYOM3//EXD1//EMC10//LIN9//CHTF8//THUMPD1//ASF1B//SNRPA//U2AF1//CGNL1//SYPL2//SHKBP1//PHOX2A//CCDC97//CYB561A3//SESTD1//LGALS3BP//TOMM40L// |
| GO:0015629 | actin cytoskeleton | Cellular component | 23 | 487 | 543 | 18698 | 1.62627580443275 | 0.0157659410424009 | 0.165102525598076 | 1.80228010183807 | 0.0423572744014733 | DBNL//BIN2//SH3GL1//FERMT3//MYOM3//LMOD1//DAPK3//WAS//ACTR3//ACTR2//MYO19//CGNL1//NF2//MLPH//POLR2M//FERMT2//PGM5//SEPT7//MAEA//SPTBN2//PARVA//KLHL14//ONECUT2// |
| GO:0030532 | small nuclear ribonucleoprotein complex | Cellular component | 6 | 70 | 543 | 18698 | 2.95153906866614 | 0.0159528633581377 | 0.165102525598076 | 1.79716135467379 | 0.0110497237569061 | LSM11//SNRPB//SNRPA//CCDC97//DDX39B//PPIH// |
| GO:0031252 | cell leading edge | Cellular component | 19 | 385 | 543 | 18698 | 1.69937097892899 | 0.017787767131734 | 0.180634367497803 | 1.74987856474508 | 0.0349907918968692 | ARFIP2//DBNL//ITGA5//TLN1//ACTR3//ACTR2//DPP4//NF2//BRK1//ARHGEF6//FERMT2//TIRAP//PACSIN1//ARHGEF4//EPS8L2//TIAM1//UNC5A//PALM//SHISA9// |
| GO:0031225 | anchored component of membrane | Cellular component | 10 | 159 | 543 | 18698 | 2.16569952627495 | 0.0178741807186417 | 0.180634367497803 | 1.74777385540254 | 0.0184162062615101 | PRSS8//LYPD6//EFNA3//NCAM1//SPRN//NRN1//RGMA//CNTN2//SEMA7A//VNN2// |
| GO:0031674 | I band | Cellular component | 9 | 138 | 543 | 18698 | 2.24573624789815 | 0.0194376054761492 | 0.194150036093165 | 1.71135723696875 | 0.0165745856353591 | MYOM3//BMP10//PGM5//PPP3CB//PARVA//SCN3B//SCN1A//FERMT2//POLR2M// |
| GO:0001726 | ruffle | Cellular component | 10 | 164 | 543 | 18698 | 2.09967210169339 | 0.0216660858851635 | 0.213921468682247 | 1.66421953962677 | 0.0184162062615101 | TIRAP//PACSIN1//ITGA5//NF2//ARHGEF4//EPS8L2//TIAM1//TLN1//ARFIP2//DBNL// |
| GO:0019005 | SCF ubiquitin ligase complex | Cellular component | 5 | 56 | 543 | 18698 | 3.0745198631939 | 0.0229799646838141 | 0.222570406768594 | 1.63865064308187 | 0.00920810313075507 | FBXO45//FBXL3//SKP1//TRIM21//FBXL20// |
| GO:0031410 | cytoplasmic vesicle | Cellular component | 82 | 2279 | 543 | 18698 | 1.23898158945032 | 0.0230602633322525 | 0.222570406768594 | 1.6371357376705 | 0.151012891344383 | CDKN1B//TGOLN2//LRRK2//STEAP2//PACSIN1//SMPD1//VPS26A//NUCB1//KDELR3//EDN1//FGFR3//NPTX1//SSPN//TIRAP//DPP4//EHD3//MGRN1//DBNL//NF2//WDFY1//CTNS//DYNC1I1//AVL9//VPS53//WAS//SCAMP1//GPNMB//ATP6V1G2//YWHAB//IL1B//THBS1//CD74//CD79A//GRIPAP1//COPS5//DLG4//LGI3//KIT//SKIL//SYT9//UNC13B//WDR91//SNX19//PML//SH3GL1//CYB561A3//GOSR2//RHOF//PTAFR//ALAD//ALDOC//NFKB1//BIN2//S100A12//SYT2//ABCC8//NRGN//FERMT3//TSPAN14//ACTR2//LGALS3BP//FZD2//AP1S1//TAB1//VPS45//MRC1//UBE2D3//NDFIP1//SYNPR//SYPL2//EBP//EMP2//FGFR2//NRBF2//SLC39A2//ITGA5//ARHGDIB//PALM//TP53INP2//TRIM21//MAPKAP1//KIAA0319L// |
| GO:0031982 | vesicle | Cellular component | 147 | 4372 | 543 | 18698 | 1.15779723301977 | 0.0235175165650814 | 0.223933416277724 | 1.62860854139802 | 0.270718232044199 | WAS//SLC5A6//EBP//LRRK2//EMP2//FGFR2//NRBF2//SLC39A2//ITGA5//ARHGDIB//PALM//RHOF//TP53INP2//TRIM21//MAPKAP1//KIAA0319L//ACTR3//ACTR2//MMP24//VSIG4//PIK3IP1//AP1S1//FAM168B//CPNE8//CTH//CTNS//DBI//DPP4//DPYS//ALAD//ALDOC//MGRN1//HAAO//DDAH1//CARHSP1//FSHB//GALNT3//GLRX//GNAZ//RNF149//DBNL//SLC25A5//CD209//IL1B//AQP1//KRT2//KRT9//TRABD2B//LGALS3BP//MEST//MGAT5//ASNA1//NCAM1//NUCB1//PABPC3//PLLP//REEP2//CC2D1A//CHTF8//BRK1//PRSS8//RGMA//MAN1C1//APMAP//RTN4//PTBP1//BCR//SFRP1//EPS8L2//SKP1//SMPD1//SNRPB//TGM4//TGM2//THBS1//TLN1//TMPRSS2//C1QA//UBE2D3//YWHAB//NPHS2//MLPH//PIP4K2C//URM1//CRISPLD1//FERMT3//PRADC1//PARD6B//RELT//KRT38//CD22//VPS26A//CREB5//CD74//CD81//SEPT7//OXSR1//CDKN1B//TGOLN2//STEAP2//PACSIN1//NF2//WDFY1//KDELR3//EDN1//FGFR3//NPTX1//SSPN//TIRAP//EHD3//SCAMP1//GPNMB//ATP6V1G2//DYNC1I1//AVL9//VPS53//CD79A//GRIPAP1//COPS5//DLG4//LGI3//KIT//SKIL//SYT9//UNC13B//WDR91//SNX19//PML//SH3GL1//CYB561A3//GOSR2//PTAFR//NFKB1//BIN2//S100A12//SYT2//ABCC8//NRGN//TSPAN14//FZD2//TAB1//VPS45//MRC1//NDFIP1//SYNPR//SYPL2//SPRN// |
| GO:0097708 | intracellular vesicle | Cellular component | 82 | 2282 | 543 | 18698 | 1.23735277929767 | 0.0237228648210394 | 0.223933416277724 | 1.62483286586252 | 0.151012891344383 | EBP//LRRK2//EMP2//FGFR2//NRBF2//SLC39A2//ITGA5//ARHGDIB//PALM//RHOF//TP53INP2//TRIM21//MAPKAP1//KIAA0319L//CDKN1B//TGOLN2//STEAP2//PACSIN1//SMPD1//VPS26A//NUCB1//KDELR3//EDN1//FGFR3//NPTX1//SSPN//TIRAP//DPP4//EHD3//MGRN1//DBNL//NF2//WDFY1//CTNS//CD74//CD79A//DYNC1I1//AVL9//VPS53//GRIPAP1//COPS5//DLG4//LGI3//UNC13B//WAS//SCAMP1//IL1B//THBS1//GPNMB//ATP6V1G2//YWHAB//KIT//SKIL//SYT9//WDR91//SNX19//PML//SH3GL1//CYB561A3//GOSR2//PTAFR//ALAD//ALDOC//NFKB1//BIN2//S100A12//SYT2//ABCC8//NRGN//FERMT3//TSPAN14//ACTR2//LGALS3BP//FZD2//TAB1//VPS45//MRC1//UBE2D3//NDFIP1//AP1S1//SYNPR//SYPL2// |
| GO:0031090 | organelle membrane | Cellular component | 103 | 2950 | 543 | 18698 | 1.20229359802728 | 0.0241152334791943 | 0.225162886506825 | 1.61770852896354 | 0.189686924493554 | WAS//SLC5A6//AQP1//DNAJB12//RNF123//LRRK2//NRGN//SNPH//AP1S1//PACSIN1//SNX19//YWHAB//ATP1B4//SMAD1//TIMM23//LETM2//GPD2//SLC25A5//TIMM22//CHDH//SLC25A2//CABS1//TOMM34//RAF1//MYO19//SNN//KDELR3//VPS45//GBP4//CUX1//EMP2//GALNT3//DBNL//ST8SIA5//ST6GALNAC6//GOLGA7B//MGAT5//NCAM1//RNF125//GALNT10//SLC30A6//MAN1C1//ST3GAL3//SLC35A2//KIAA0319L//TNKS2//NDFIP1//FAM57B//CREB3L3//PGAP3//HS6ST1//GAL3ST1//GOSR2//CD74//CTNS//SYT9//RHOF//PTAFR//TAB1//STEAP2//EHD3//MRC1//VPS53//UBE2D3//VPS26A//DLG4//EVC//IQCE//SHANK3//NUCB1//MMP24//CABP7//ARFIP2//CALN1//SYS1//DPP4//CYB561A3//DRAM1//VLDLR//UNC13B//SYT2//ABCC8//SCAMP1//TSPAN14//WDR91//PML//GRIPAP1//SH3GL1//TGOLN2//FZD2//PEX5//ATP6V1G2//P2RX2//SYNPR//SYPL2//TRABD2B//TOMM40L//CYP2C18//CYP2S1//CYP4F3//LRRC59//SLC39A8//CYP4F2// |
| GO:0034706 | sodium channel complex | Cellular component | 3 | 23 | 543 | 18698 | 4.4914724957963 | 0.0280133557094274 | 0.258747016713958 | 1.55263486408628 | 0.00552486187845304 | SCN3B//SCN1A//SCN7A// |
| GO:0120114 | Sm-like protein family complex | Cellular component | 6 | 81 | 543 | 18698 | 2.55071277539049 | 0.0303483413745031 | 0.277332183411683 | 1.51786503940206 | 0.0110497237569061 | SNRPB//LSM11//SNRPA//CCDC97//DDX39B//PPIH// |
| GO:0098562 | cytoplasmic side of membrane | Cellular component | 11 | 199 | 543 | 18698 | 1.90342134243964 | 0.0312112908892366 | 0.281647999167449 | 1.50568826867916 | 0.0202578268876611 | NPTXR//KIT//NPTX2//PGM5//LRRK2//FERMT2//DLG4//TIAM1//NPHS2//GNAO1//GNAZ// |
| GO:0098796 | membrane protein complex | Cellular component | 45 | 1166 | 543 | 18698 | 1.32895198203235 | 0.0315067429572194 | 0.281647999167449 | 1.50159649015567 | 0.0828729281767956 | PYURF//TOMM40L//VPS26A//CPLX1//CPLX3//GOSR2//INSIG2//RNF125//EMC8//EMC10//EVC//NRBF2//KCNQ5//GNAO1//GNAZ//SCN3B//SCN1A//SCN7A//ATP1B4//CACNG1//CACNA2D2//DLG4//KCNIP3//KCNIP2//KCNA6//ABCC8//CNTN2//KCNQ4//PGM5//SSPN//SLC6A3//ITGA5//TIMM23//TIMM22//ATP6V1G2//AP1S1//CHRNA3//CHRFAM7A//SHISA9//SHANK3//GRIN2B//SESTD1//BEST1//CD74//CD79A// |
| GO:0031588 | nucleotide-activated protein kinase complex | Cellular component | 2 | 10 | 543 | 18698 | 6.88692449355433 | 0.0324600138737805 | 0.281647999167449 | 1.48865129888733 | 0.00368324125230203 | PRKAA1//PRKAB1// |
| GO:0044224 | juxtaparanode region of axon | Cellular component | 2 | 10 | 543 | 18698 | 6.88692449355433 | 0.0324600138737805 | 0.281647999167449 | 1.48865129888733 | 0.00368324125230203 | DLG4//CNTN2// |
| GO:0072546 | ER membrane protein complex | Cellular component | 2 | 10 | 543 | 18698 | 6.88692449355433 | 0.0324600138737805 | 0.281647999167449 | 1.48865129888733 | 0.00368324125230203 | EMC8//EMC10// |
| GO:0009898 | cytoplasmic side of plasma membrane | Cellular component | 10 | 176 | 543 | 18698 | 1.9565126402143 | 0.0330970311800187 | 0.284303497836361 | 1.48021096090589 | 0.0184162062615101 | FERMT2//DLG4//TIAM1//NPHS2//GNAO1//GNAZ//NPTXR//KIT//NPTX2//PGM5// |
| GO:0044304 | main axon | Cellular component | 5 | 63 | 543 | 18698 | 2.73290654506124 | 0.035929268479949 | 0.302580800238002 | 1.44455162494274 | 0.00920810313075507 | SCN1A//CNTN2//CCK//DLG4//TIAM1// |
| GO:0097525 | spliceosomal snRNP complex | Cellular component | 5 | 63 | 543 | 18698 | 2.73290654506124 | 0.035929268479949 | 0.302580800238002 | 1.44455162494274 | 0.00920810313075507 | SNRPB//SNRPA//CCDC97//DDX39B//PPIH// |
| GO:0000151 | ubiquitin ligase complex | Cellular component | 15 | 311 | 543 | 18698 | 1.66083388108223 | 0.0387848419005588 | 0.319569132620175 | 1.41133797453963 | 0.0276243093922652 | FBXO45//FBXL3//SKP1//TRIM21//FBXL20//ZSWIM6//KLHL14//KLHL22//DCAF16//CDKN1B//UBE4B//RNF217//FBXO10//MED1//UBE2E1// |
| GO:0005885 | Arp2/3 protein complex | Cellular component | 2 | 11 | 543 | 18698 | 6.26084044868575 | 0.0389230277784275 | 0.319569132620175 | 1.40979338381175 | 0.00368324125230203 | ACTR3//ACTR2// |
| GO:0005925 | focal adhesion | Cellular component | 18 | 393 | 543 | 18698 | 1.57715828096664 | 0.0390625831491483 | 0.319569132620175 | 1.4082390408657 | 0.0331491712707182 | ACTR3//ACTR2//FERMT2//CDC42EP1//DPP4//FGFR3//FZD2//EHD3//ITGA5//PGM5//LIMS2//PARVA//TGM2//TLN1//YWHAB//NUP214//DIXDC1//CD81// |
| GO:0005924 | cell-substrate adherens junction | Cellular component | 18 | 396 | 543 | 18698 | 1.56521011217144 | 0.0415490727686314 | 0.336704278379758 | 1.38143866372007 | 0.0331491712707182 | ACTR3//ACTR2//FERMT2//CDC42EP1//DPP4//FGFR3//FZD2//EHD3//ITGA5//PGM5//LIMS2//PARVA//TGM2//TLN1//YWHAB//NUP214//DIXDC1//CD81// |
| GO:0044422 | organelle part | Cellular component | 291 | 9321 | 543 | 18698 | 1.07504292866876 | 0.0421566020648111 | 0.338098924875758 | 1.37513440168483 | 0.535911602209945 | AQP1//CYP2C18//CYP2S1//CYP4F3//LRRC59//SLC39A8//CYP4F2//EPS8L2//ESYT1//WAS//SLC5A6//DNAJB12//RNF123//LRRK2//NRGN//SNPH//TRABD2B//RGS9BP//WDR19//CCDC103//SEPT7//EVC//IQCE//SHANK3//KIF2A//MYOM3//LMOD1//BMP10//PGM5//PPP3CB//PARVA//SCN3B//SCN1A//FERMT2//POLR2M//NUCB1//DLG4//GRIN2B//CD74//SYT9//RHOF//PTAFR//AP1S1//PACSIN1//SNX19//YWHAB//ATP1B4//SMAD1//TIMM23//LETM2//GPD2//SLC25A5//TIMM22//CHDH//SLC25A2//CABS1//KDELR3//VPS45//GBP4//CUX1//EMP2//GALNT3//DBNL//ST8SIA5//ST6GALNAC6//GOLGA7B//MGAT5//NCAM1//RNF125//GALNT10//SLC30A6//MAN1C1//ST3GAL3//SLC35A2//KIAA0319L//TNKS2//NDFIP1//FAM57B//CREB3L3//PGAP3//HS6ST1//GAL3ST1//GOSR2//TOMM34//RAF1//MYO19//SNN//CTNS//TAB1//STEAP2//EHD3//MRC1//VPS53//UBE2D3//VPS26A//SYS1//P2RX2//SYNPR//SYPL2//ATP6V1G2//WDR91//PML//EBP//GNAZ//PARP16//RTN4//PLA2G4C//ALAD//ALDOC//NFKB1//BIN2//S100A12//TGOLN2//RBFOX1//SCAMP1//DLAT//SIRT3//PARG//NFS1//DBI//GPX8//THBS1//EDEM3//ACTR2//RBM3//MIS12//DYNC1I1//TTK//CENPO//PURA//COPS5//HIC1//MED1//KMT2E//DLX5//PHOX2A//ASF1B//CALCOCO1//NRIP1//CHTF8//MYBL2//SATB2//OGT//DDX39B//PAX5//CC2D1A//SNRPB//MED6//SGK2//CDKN1B//MAEA//PPIH//ARID3B//RPP14//TAF8//LSM11//NEK7//RNF38//ELK1//FGFR2//KLHDC10//CRTC1//ZNF281//PTPN18//HEYL//LIN9//NRBF2//HOXC6//HOXC9//VWA5A//MAGEA11//NFYA//FOXP3//RLIM//UCHL5//C9ORF78//PIM1//POLR2E//DCAF16//PRKAA1//THUMPD1//PRKAB1//MBNL3//MAPK4//GRIPAP1//SH3GLB2//ZMIZ1//PTBP1//USP37//RAG1//REL//RSC1A1//SKIL//SKP1//UBE2Z//HLTF//SNRPA//TRIM21//TCF20//TEAD1//U2AF1//UBE2E1//PAX8//C2ORF49//MAPKAP1//NUP214//FOSL1//CASP7//MEX3B//DDI2//USP45//SRSF9//SH3BP5//ONECUT2//RBM8A//EWSR1//LDOC1//HMOX1//ASNA1//NF2//NOVA1//SPRN//TRIM68//CEP85//PPP1R26//FBXL3//MCIDAS//BHLHE40//PRDM4//NFATC4//ASCL1//RASSF1//TTC28//BEX4//KRT2//KRT9//KRT38//SH3GL1//FERMT3//MAPRE1//CCDC88C//ZNF322//KLHL22//FAM92B//SLMAP//MAP7D1//TIAM1//DAPK3//ACTR3//SPTBN2//CGNL1//MLPH//UBXN2A//MMP24//CABP7//ARFIP2//CALN1//PYURF//AGPAT1//FAXDC2//SLC27A4//LCLAT1//MEST//PAFAH2//INSIG2//REEP2//KLHL14//MOGAT2//SLC10A7//EDN1//EMC8//EMC10//SMPD1//PEX5//SYT2//FZD2//CYB561A3//TSPAN14//CDKN2A//KRTAP1-1//CCDC97//DPP4//DRAM1//VLDLR//UNC13B//ABCC8//ZNF804A//TP53INP2//PASD1//LGALS3BP//TOMM40L// |
| GO:0098563 | intrinsic component of synaptic vesicle membrane | Cellular component | 3 | 27 | 543 | 18698 | 3.82606916308574 | 0.0425083630810033 | 0.338098924875758 | 1.37152561859381 | 0.00552486187845304 | SYNPR//SYPL2//ATP6V1G2// |
| GO:0005687 | U4 snRNP | Cellular component | 2 | 12 | 543 | 18698 | 5.73910374462861 | 0.0458265240777317 | 0.355554453761552 | 1.33888308255686 | 0.00368324125230203 | SNRPB//DDX39B// |
| GO:0043194 | axon initial segment | Cellular component | 2 | 12 | 543 | 18698 | 5.73910374462861 | 0.0458265240777317 | 0.355554453761552 | 1.33888308255686 | 0.00368324125230203 | SCN1A//CCK// |
| GO:0030055 | cell-substrate junction | Cellular component | 18 | 401 | 543 | 18698 | 1.5456937766082 | 0.0459447547934019 | 0.355554453761552 | 1.33776406195593 | 0.0331491712707182 | ACTR3//ACTR2//FERMT2//CDC42EP1//DPP4//FGFR3//FZD2//EHD3//ITGA5//PGM5//LIMS2//PARVA//TGM2//TLN1//YWHAB//NUP214//DIXDC1//CD81// |
| GO:0031461 | cullin-RING ubiquitin ligase complex | Cellular component | 10 | 188 | 543 | 18698 | 1.8316288546687 | 0.048206261336085 | 0.369724807925866 | 1.3168965491656 | 0.0184162062615101 | FBXO45//FBXL3//SKP1//TRIM21//FBXL20//ZSWIM6//KLHL14//KLHL22//DCAF16//CDKN1B// |
| GO:0070161 | anchoring junction | Cellular component | 23 | 546 | 543 | 18698 | 1.45054270468635 | 0.0486809787114113 | 0.370061599230994 | 1.31264069903909 | 0.0423572744014733 | CD96//CTNND2//NF2//JAM3//CDC42EP1//EIF4G2//PGM5//ACTR3//ACTR2//FERMT2//DPP4//FGFR3//FZD2//EHD3//ITGA5//LIMS2//PARVA//TGM2//TLN1//YWHAB//NUP214//DIXDC1//CD81// |
