# Supplementary figures and images for: Altered microRNA Transcriptome in Cultured Human Liver Cells upon Infection with Ebola Virus (part 2 of 2)
Source: Int J Mol Sci. 2021 Apr 6;22(7):3792. doi: 10.3390/ijms22073792 (PMC8038836; doi:10.3390/ijms22073792)

# Sig GO terms of DE gene-MF

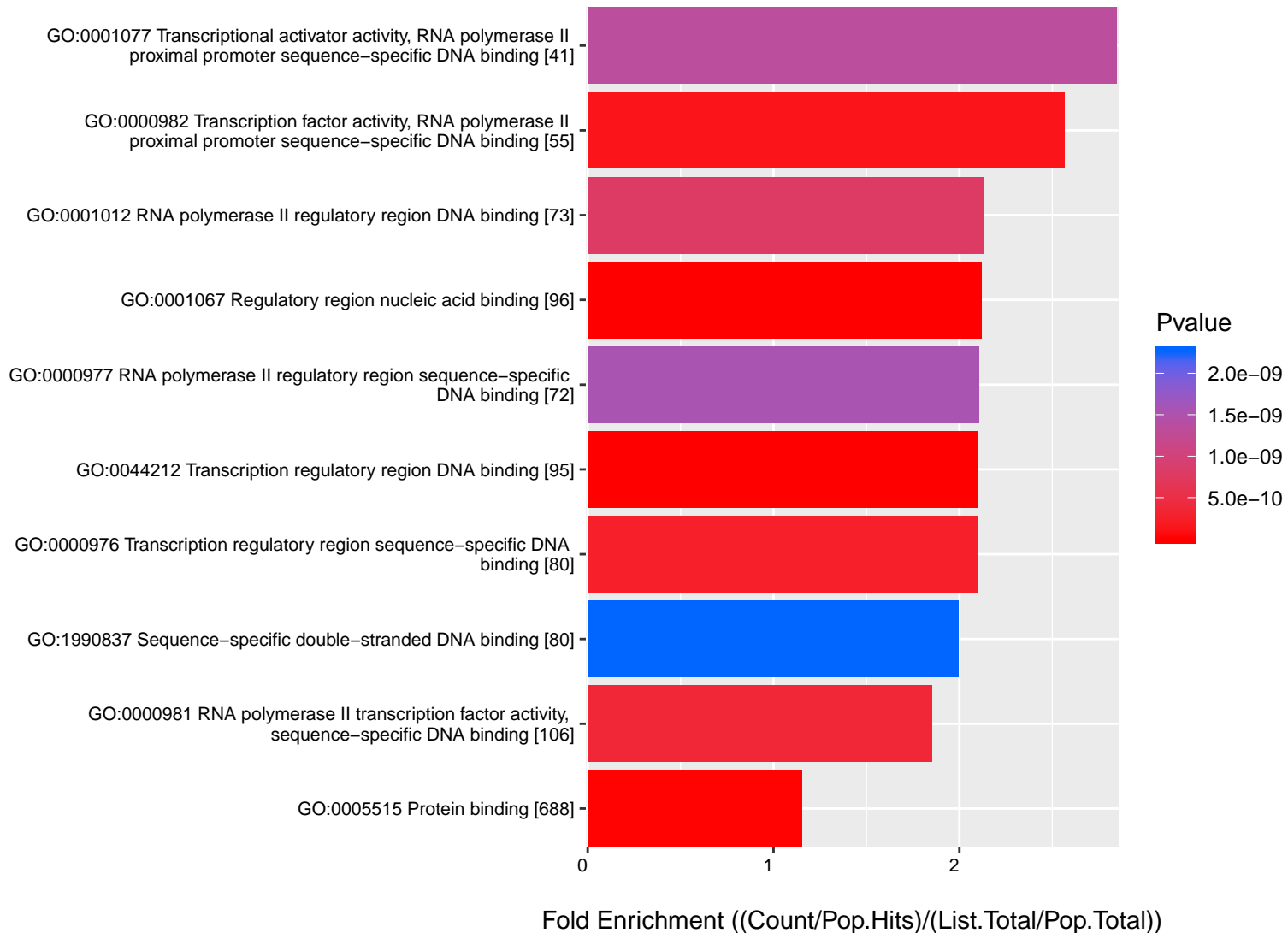

Supplement: Supplementary file 1 [file ijms-22-03792-s001.zip › Supplementary_File/C_ GO_Analysis_Results/16-30nt_go_Makona-24h-Huh7_vs_Control-24h-Huh7_up.mature_mirna_targets/MF_FoldEnrichment.pdf]

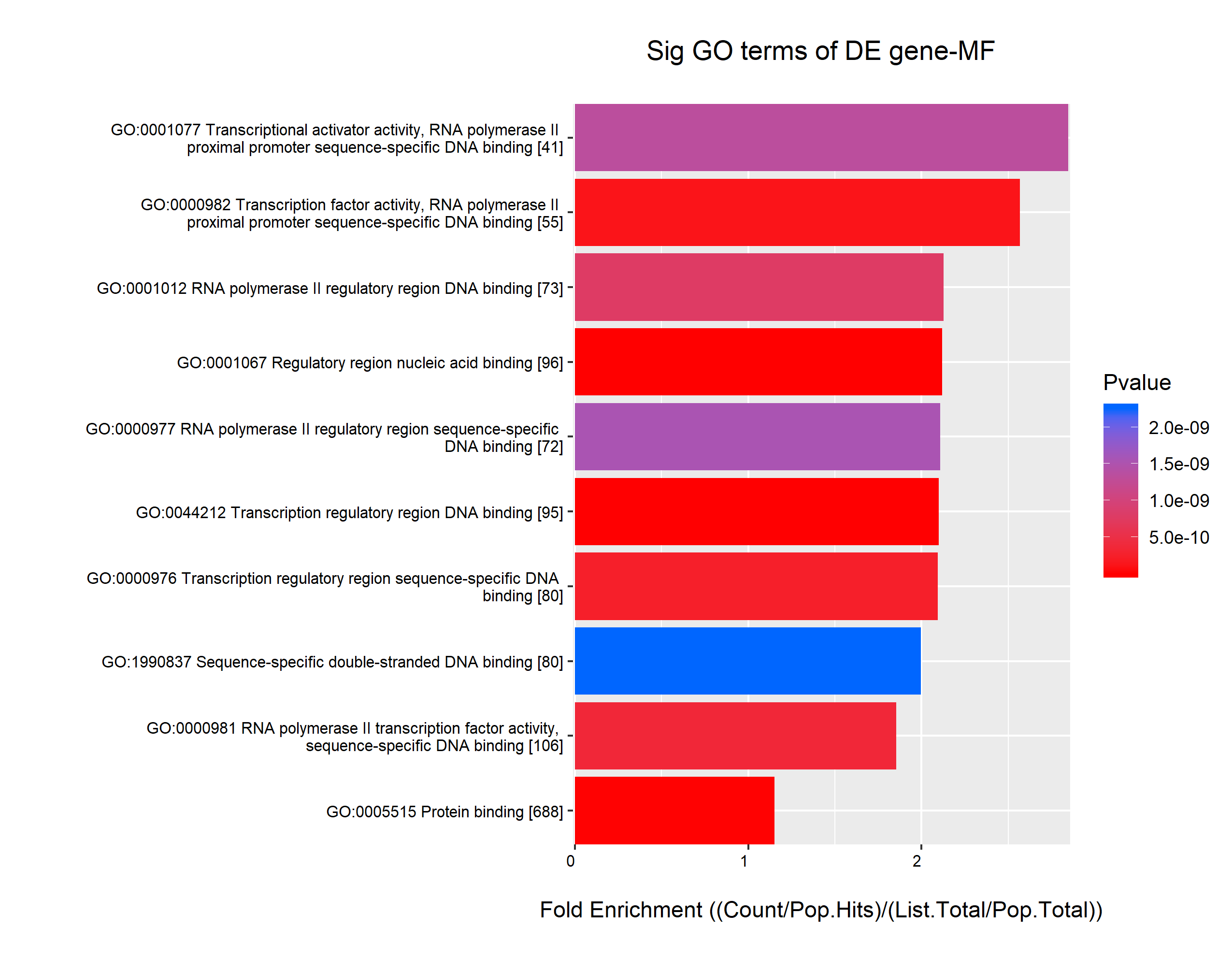

Supplement: Supplementary file 1 [file ijms-22-03792-s001.zip › Supplementary_File/C_ GO_Analysis_Results/16-30nt_go_Makona-24h-Huh7_vs_Control-24h-Huh7_up.mature_mirna_targets/MF_FoldEnrichment.png]

# Sig GO terms of DE gene-MF

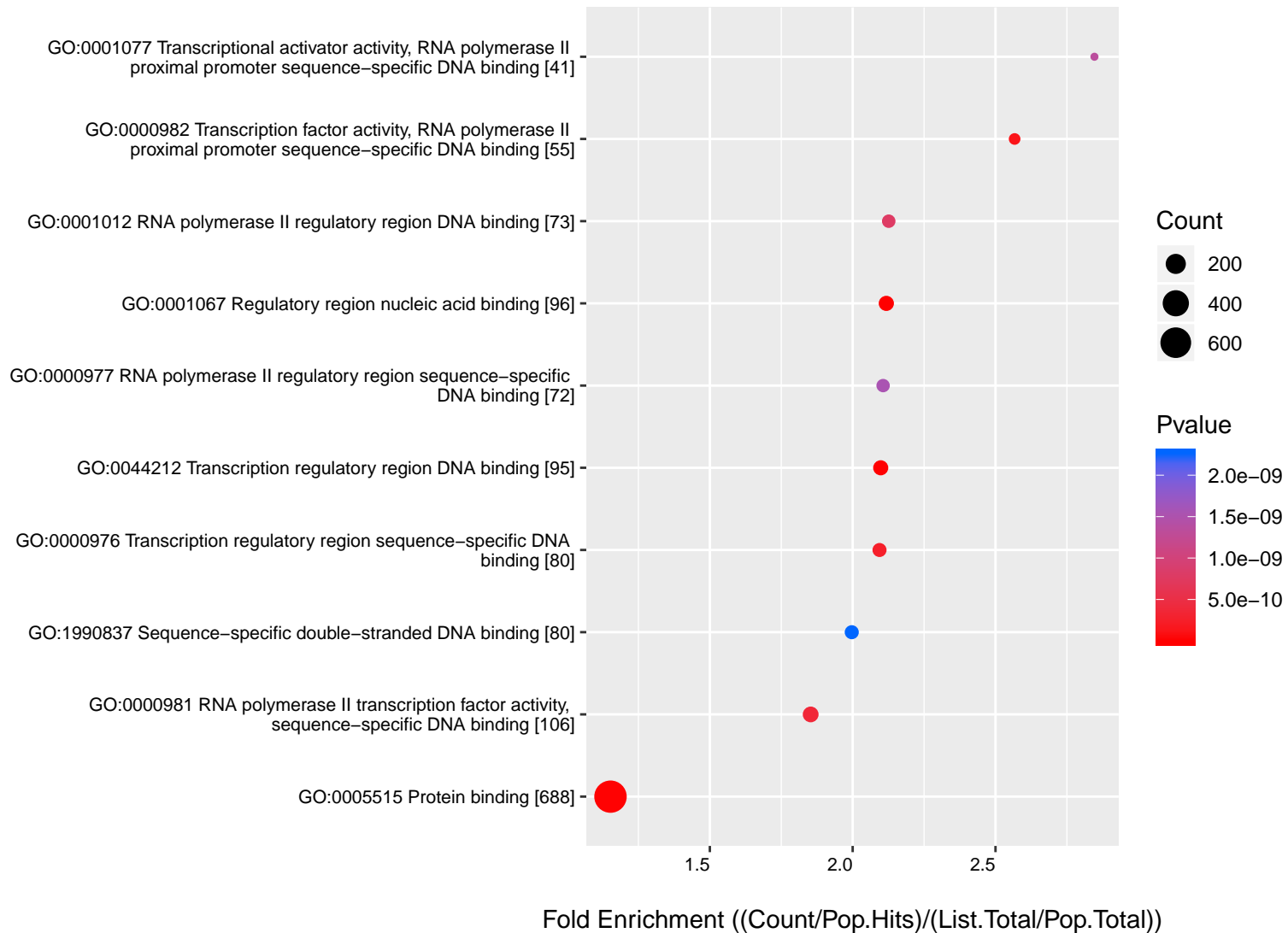

Supplement: Supplementary file 1 [file ijms-22-03792-s001.zip › Supplementary_File/C_ GO_Analysis_Results/16-30nt_go_Makona-24h-Huh7_vs_Control-24h-Huh7_up.mature_mirna_targets/MF_FoldEnrichmentDotPlot.pdf]

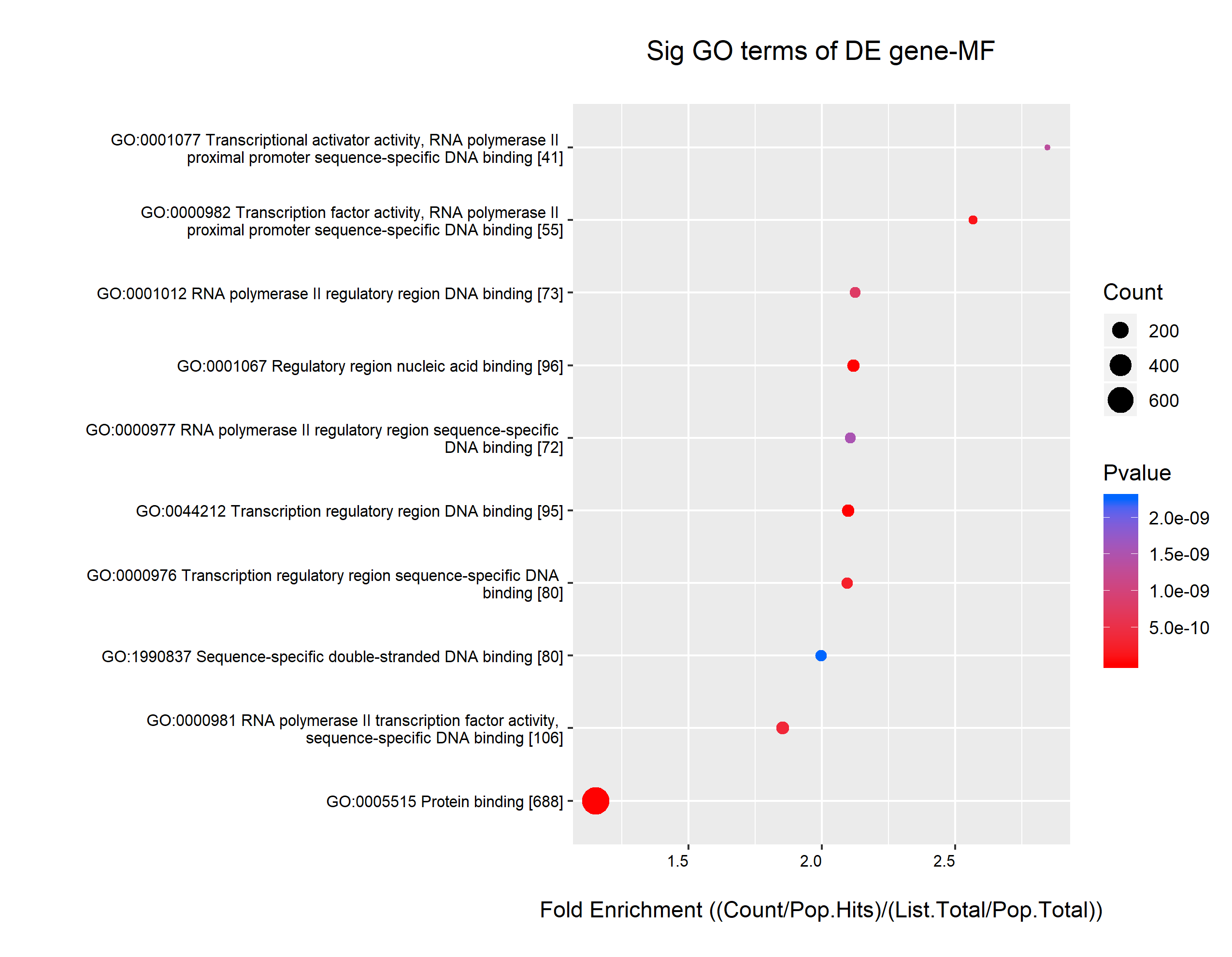

Supplement: Supplementary file 1 [file ijms-22-03792-s001.zip › Supplementary_File/C_ GO_Analysis_Results/16-30nt_go_Makona-24h-Huh7_vs_Control-24h-Huh7_up.mature_mirna_targets/MF_FoldEnrichmentDotPlot.png]

# Sig GO terms of DE gene-MF

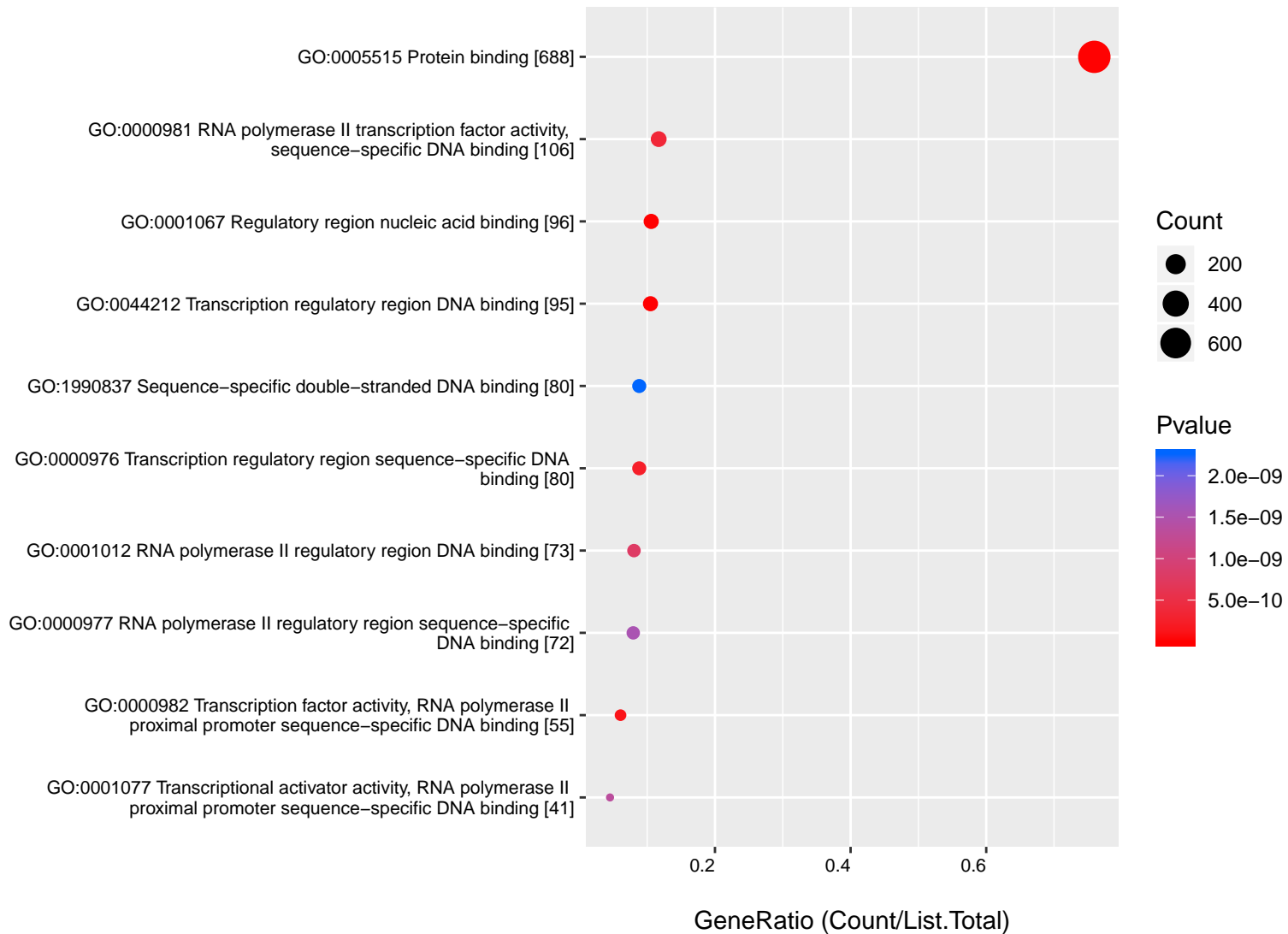

Supplement: Supplementary file 1 [file ijms-22-03792-s001.zip › Supplementary_File/C_ GO_Analysis_Results/16-30nt_go_Makona-24h-Huh7_vs_Control-24h-Huh7_up.mature_mirna_targets/MF_GeneRatioDotPlot.pdf]

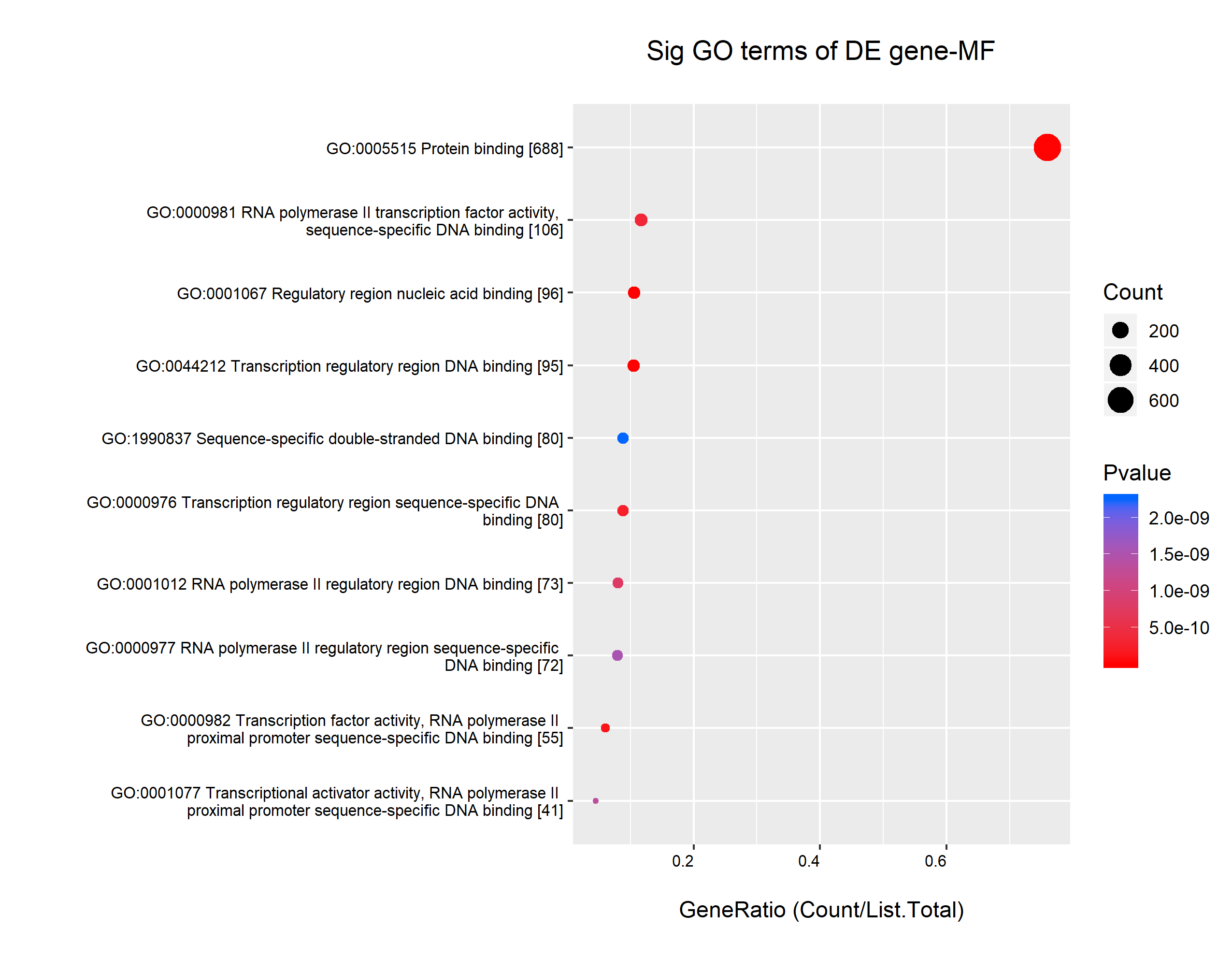

Supplement: Supplementary file 1 [file ijms-22-03792-s001.zip › Supplementary_File/C_ GO_Analysis_Results/16-30nt_go_Makona-24h-Huh7_vs_Control-24h-Huh7_up.mature_mirna_targets/MF_GeneRatioDotPlot.png]

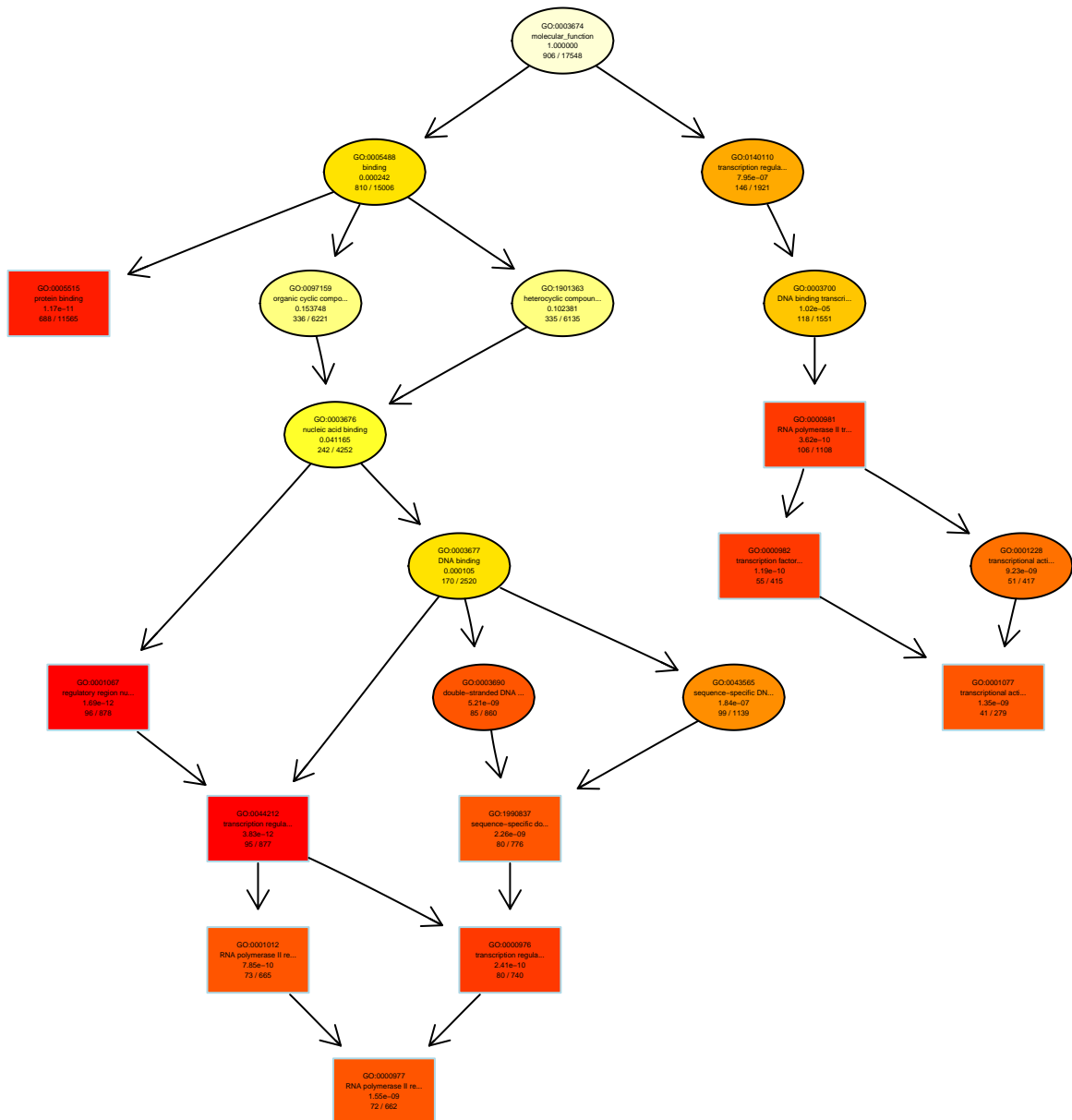

Supplement: Supplementary file 1 [file ijms-22-03792-s001.zip › Supplementary_File/C_ GO_Analysis_Results/16-30nt_go_Makona-24h-Huh7_vs_Control-24h-Huh7_up.mature_mirna_targets/MF_Pvalue_tree.pdf]

# GO Biological Process Classification

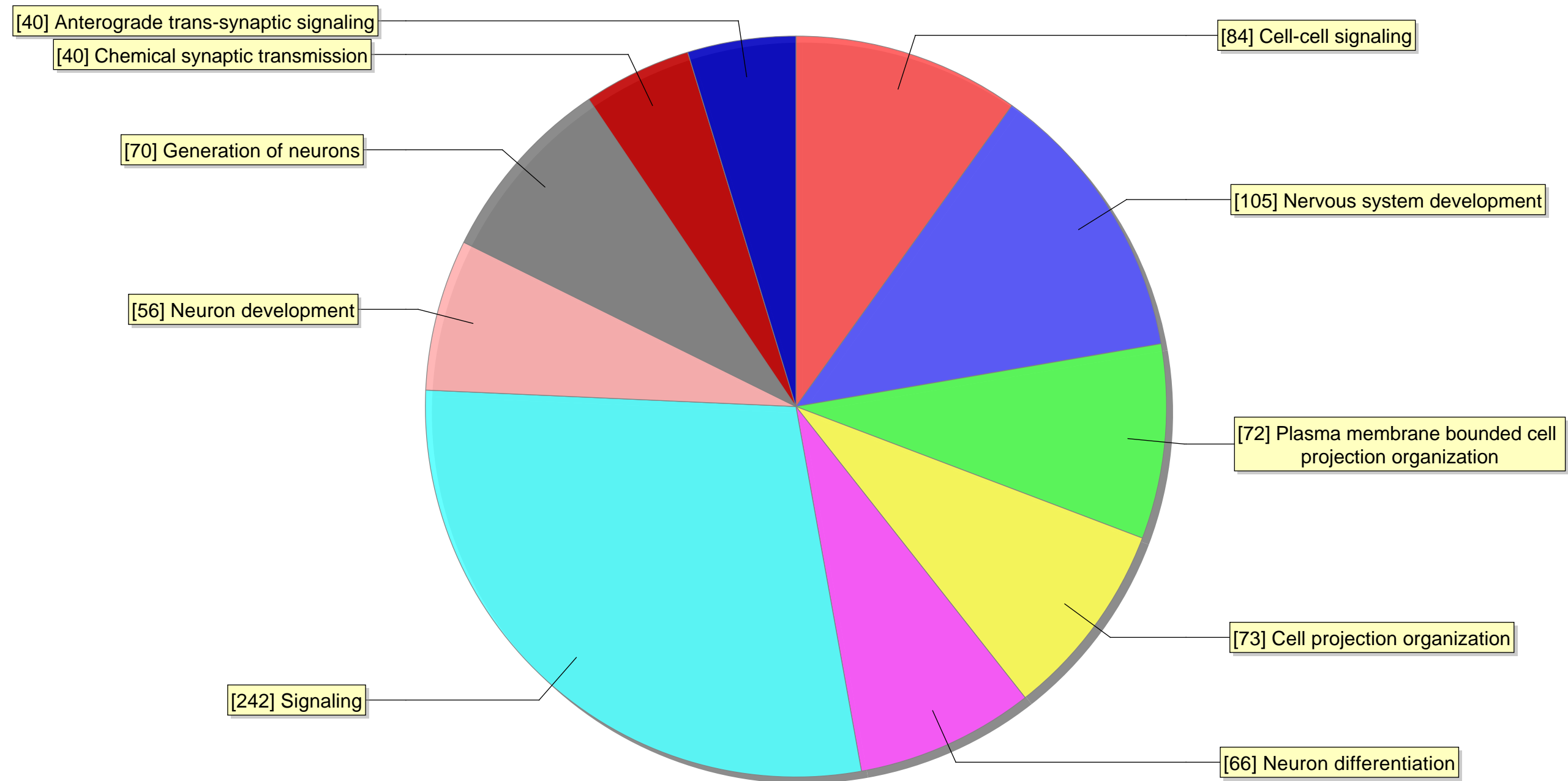

Supplement: Supplementary file 1 [file ijms-22-03792-s001.zip › Supplementary_File/C_ GO_Analysis_Results/16-30nt_go_Makona-96h-Huh7_vs_Control-96h-Huh7_down.mature_mirna_targets/BP_Count.pdf]

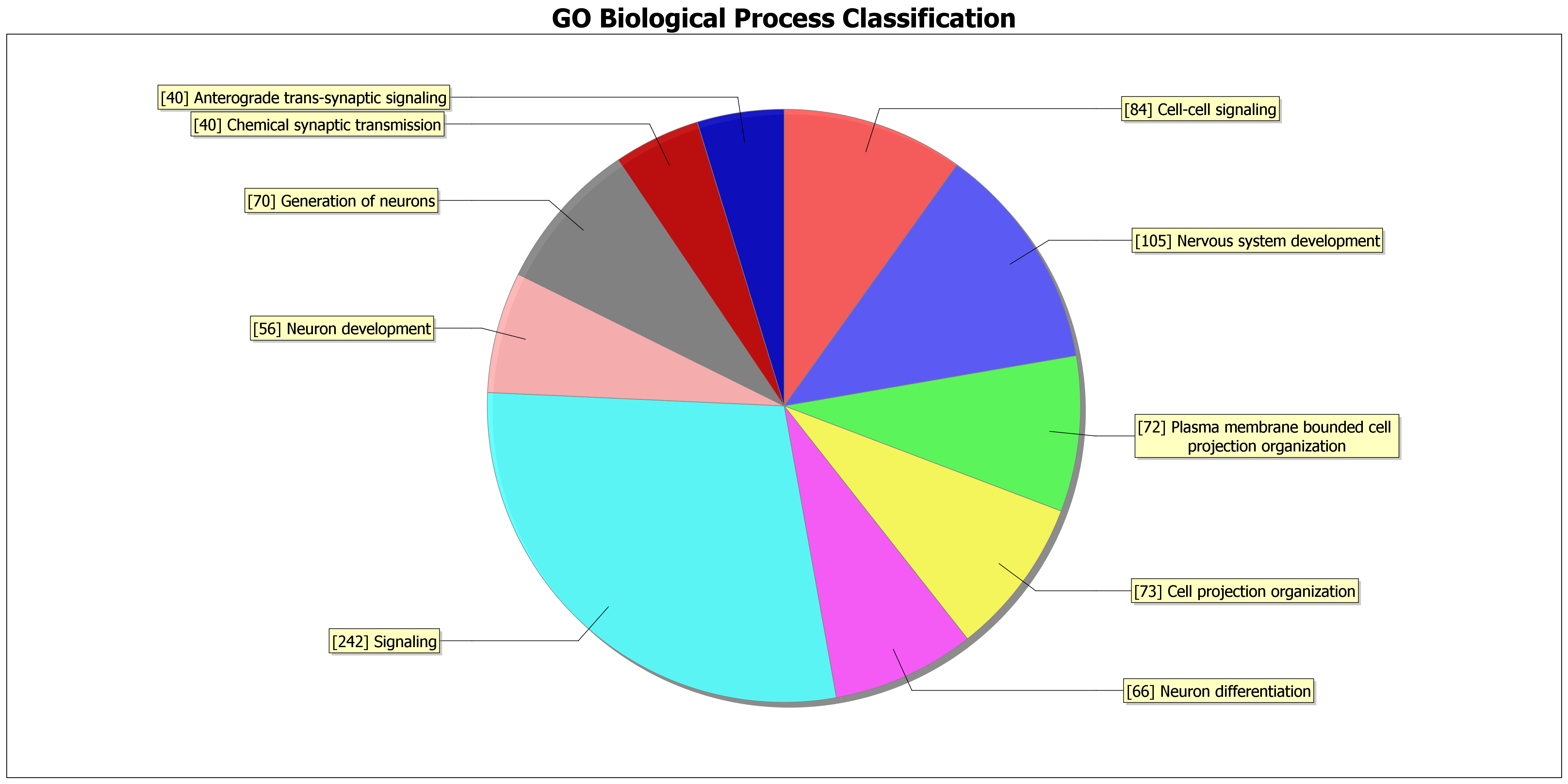

Supplement: Supplementary file 1 [file ijms-22-03792-s001.zip › Supplementary_File/C_ GO_Analysis_Results/16-30nt_go_Makona-96h-Huh7_vs_Control-96h-Huh7_down.mature_mirna_targets/BP_Count.png]

## Sig GO terms of DE gene-BP

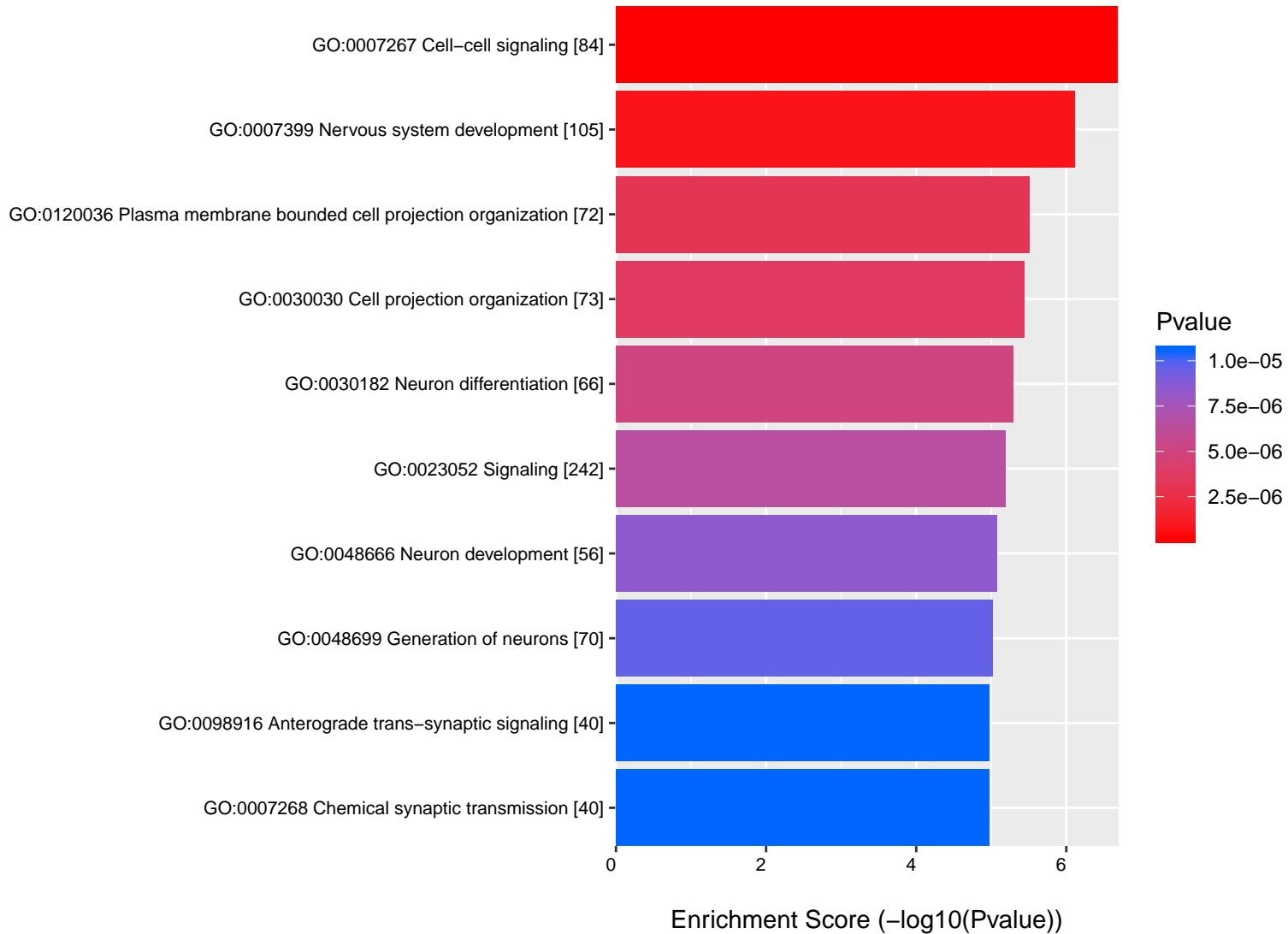

Supplement: Supplementary file 1 [file ijms-22-03792-s001.zip › Supplementary_File/C_ GO_Analysis_Results/16-30nt_go_Makona-96h-Huh7_vs_Control-96h-Huh7_down.mature_mirna_targets/BP_EnrichmentScore.pdf]

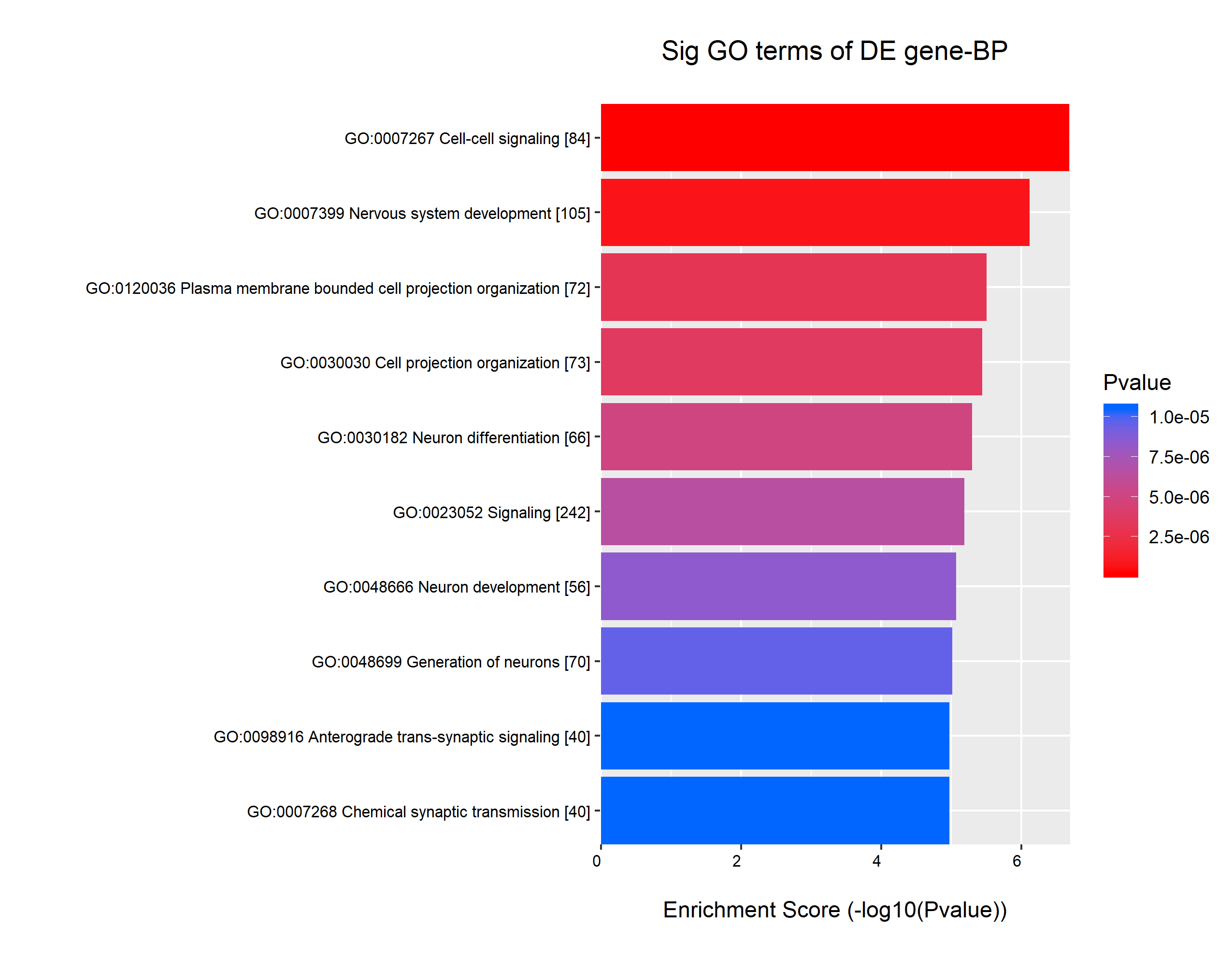

Supplement: Supplementary file 1 [file ijms-22-03792-s001.zip › Supplementary_File/C_ GO_Analysis_Results/16-30nt_go_Makona-96h-Huh7_vs_Control-96h-Huh7_down.mature_mirna_targets/BP_EnrichmentScore.png]

## Sig GO terms of DE gene-BP

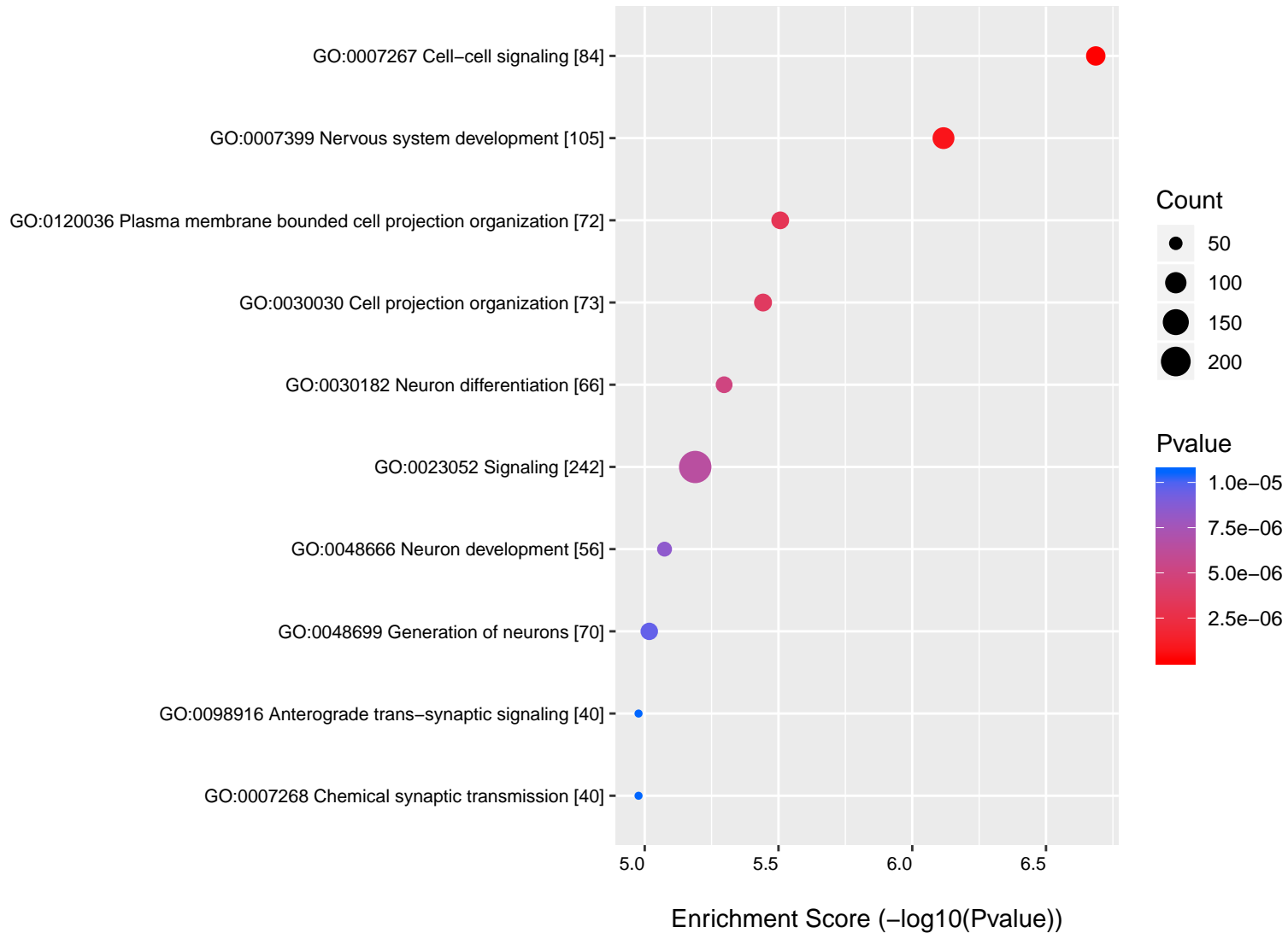

Supplement: Supplementary file 1 [file ijms-22-03792-s001.zip › Supplementary_File/C_ GO_Analysis_Results/16-30nt_go_Makona-96h-Huh7_vs_Control-96h-Huh7_down.mature_mirna_targets/BP_EnrichmentScoreDotPlot.pdf]

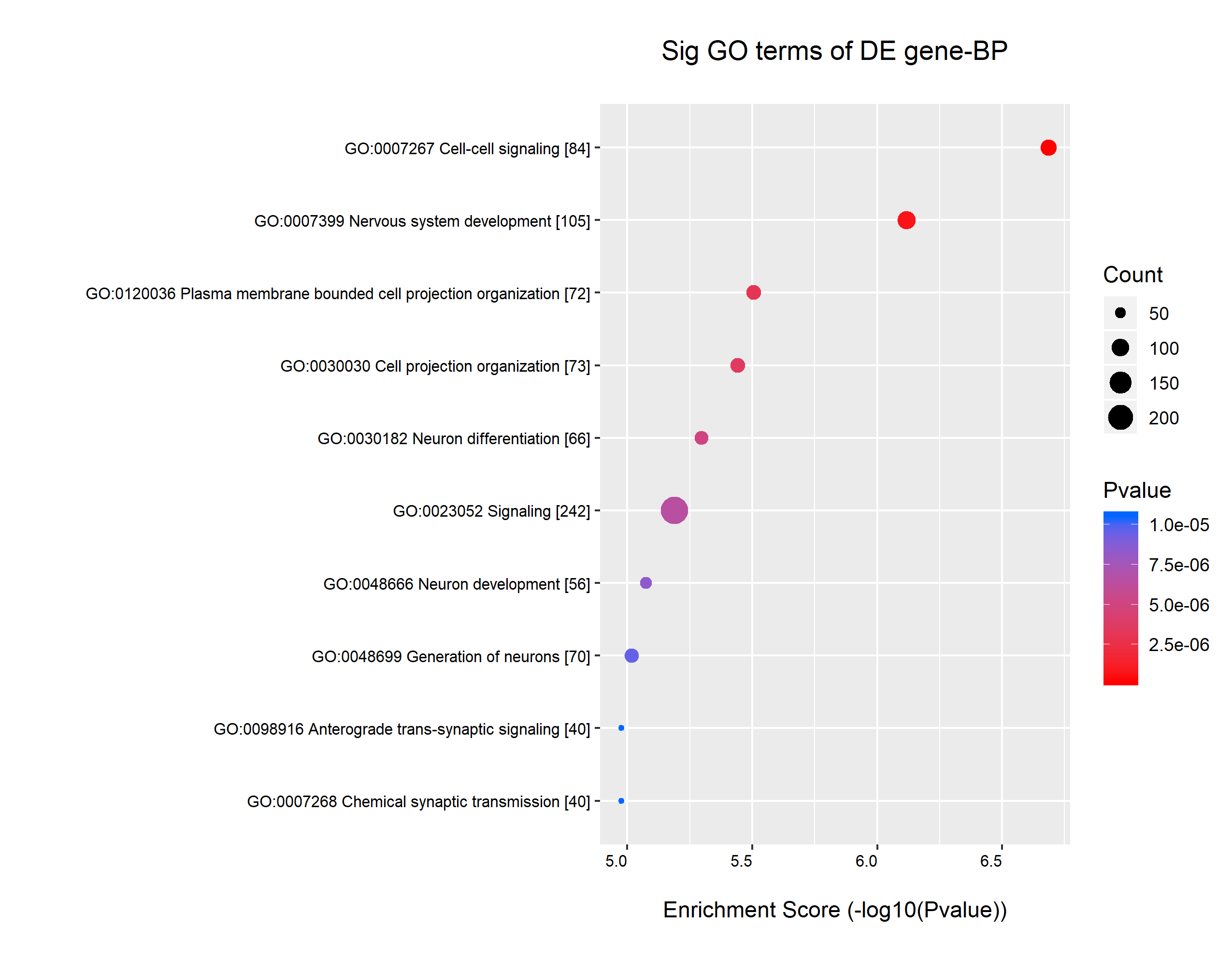

Supplement: Supplementary file 1 [file ijms-22-03792-s001.zip › Supplementary_File/C_ GO_Analysis_Results/16-30nt_go_Makona-96h-Huh7_vs_Control-96h-Huh7_down.mature_mirna_targets/BP_EnrichmentScoreDotPlot.png]

## Sig GO terms of DE gene-BP

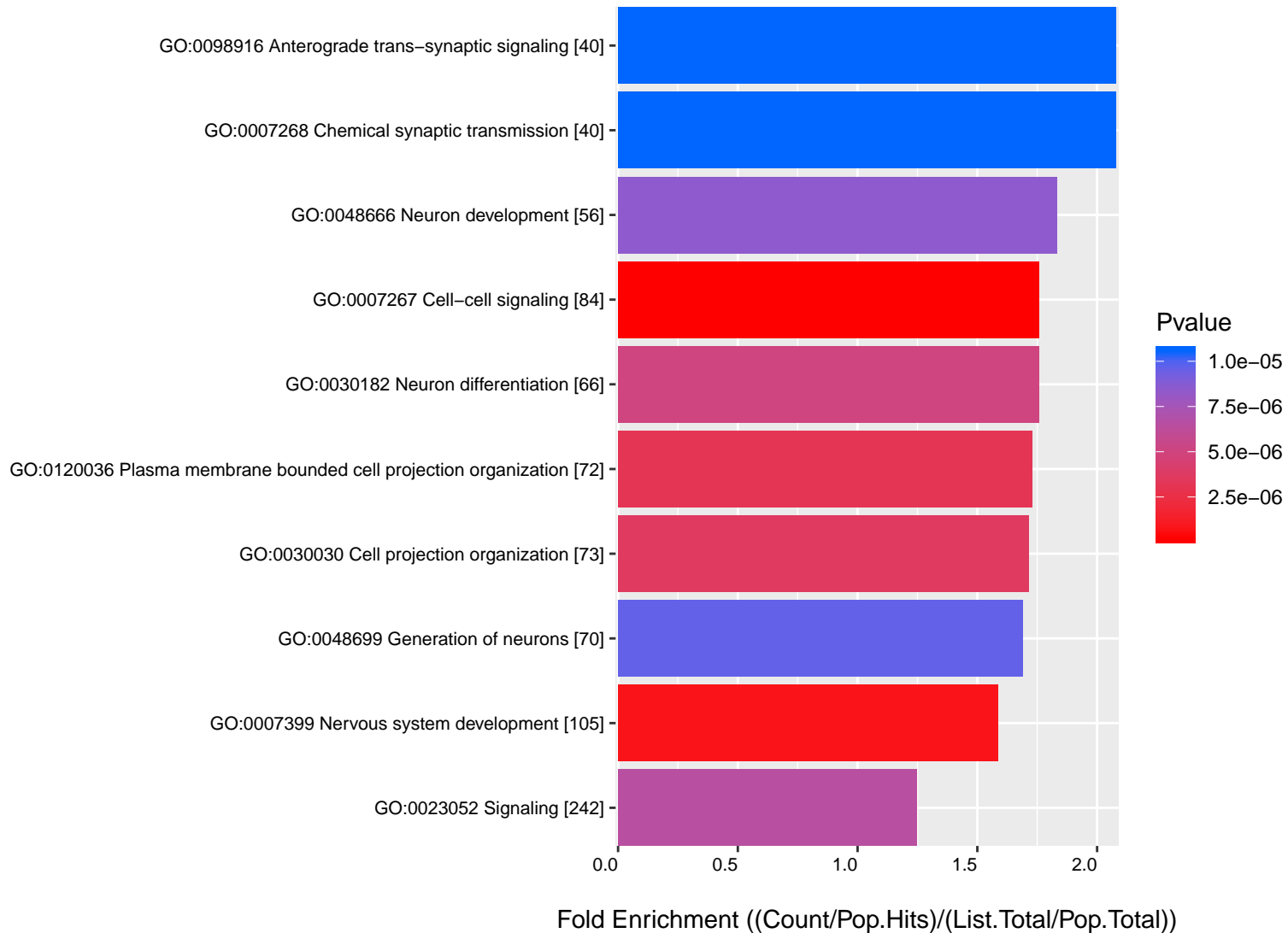

Supplement: Supplementary file 1 [file ijms-22-03792-s001.zip › Supplementary_File/C_ GO_Analysis_Results/16-30nt_go_Makona-96h-Huh7_vs_Control-96h-Huh7_down.mature_mirna_targets/BP_FoldEnrichment.pdf]

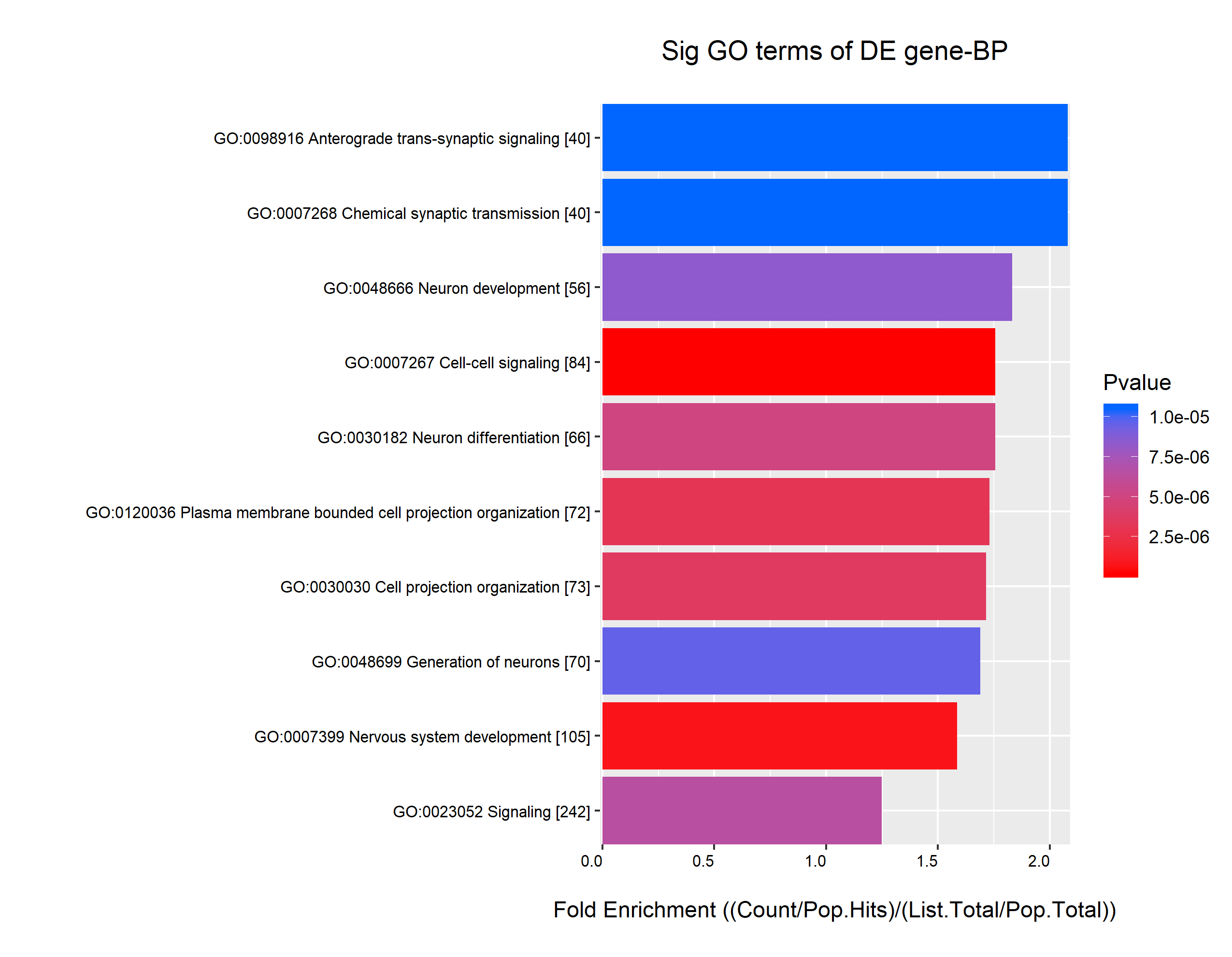

Supplement: Supplementary file 1 [file ijms-22-03792-s001.zip › Supplementary_File/C_ GO_Analysis_Results/16-30nt_go_Makona-96h-Huh7_vs_Control-96h-Huh7_down.mature_mirna_targets/BP_FoldEnrichment.png]

# Sig GO terms of DE gene-BP

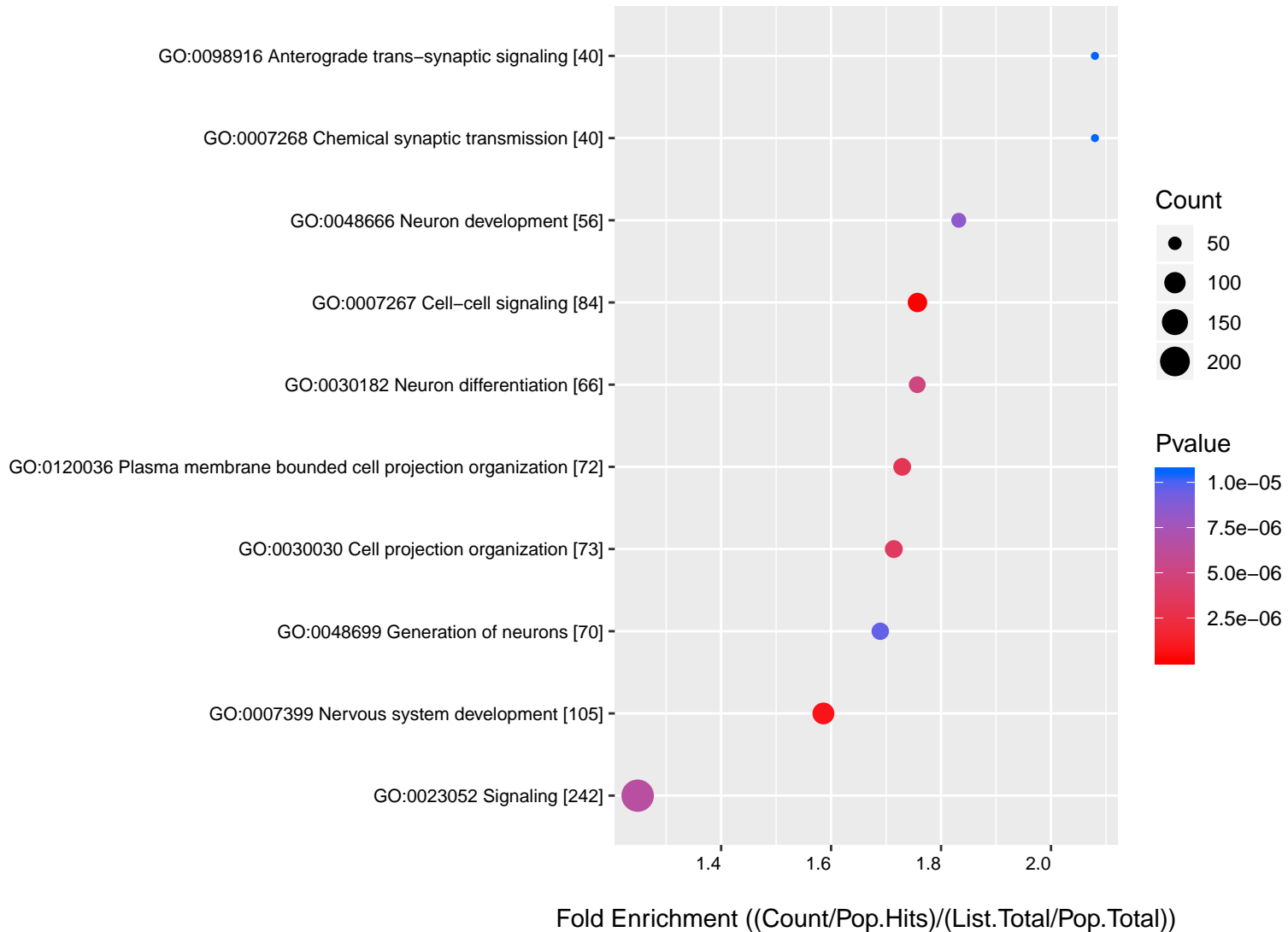

Supplement: Supplementary file 1 [file ijms-22-03792-s001.zip › Supplementary_File/C_ GO_Analysis_Results/16-30nt_go_Makona-96h-Huh7_vs_Control-96h-Huh7_down.mature_mirna_targets/BP_FoldEnrichmentDotPlot.pdf]

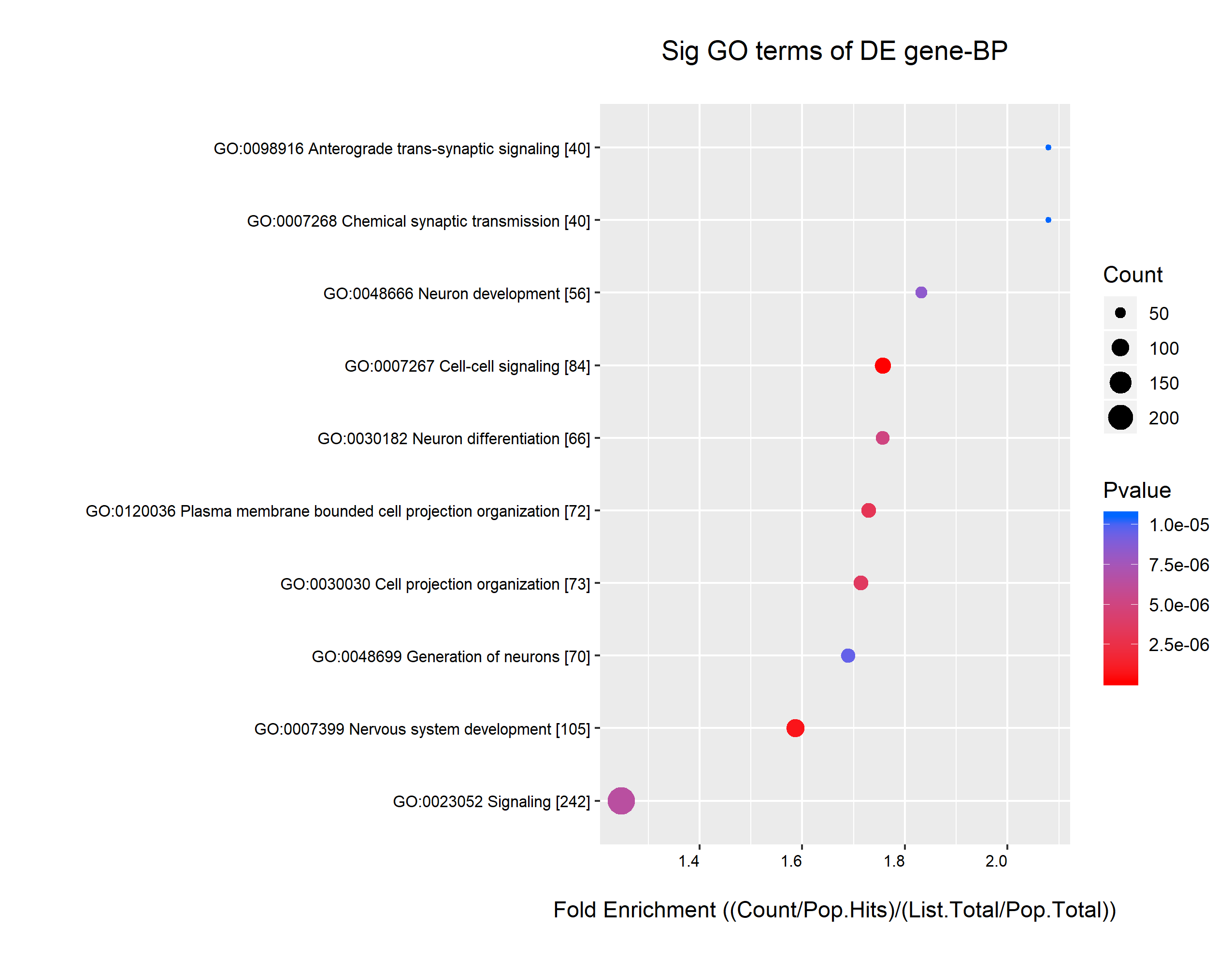

Supplement: Supplementary file 1 [file ijms-22-03792-s001.zip › Supplementary_File/C_ GO_Analysis_Results/16-30nt_go_Makona-96h-Huh7_vs_Control-96h-Huh7_down.mature_mirna_targets/BP_FoldEnrichmentDotPlot.png]

## Sig GO terms of DE gene-BP

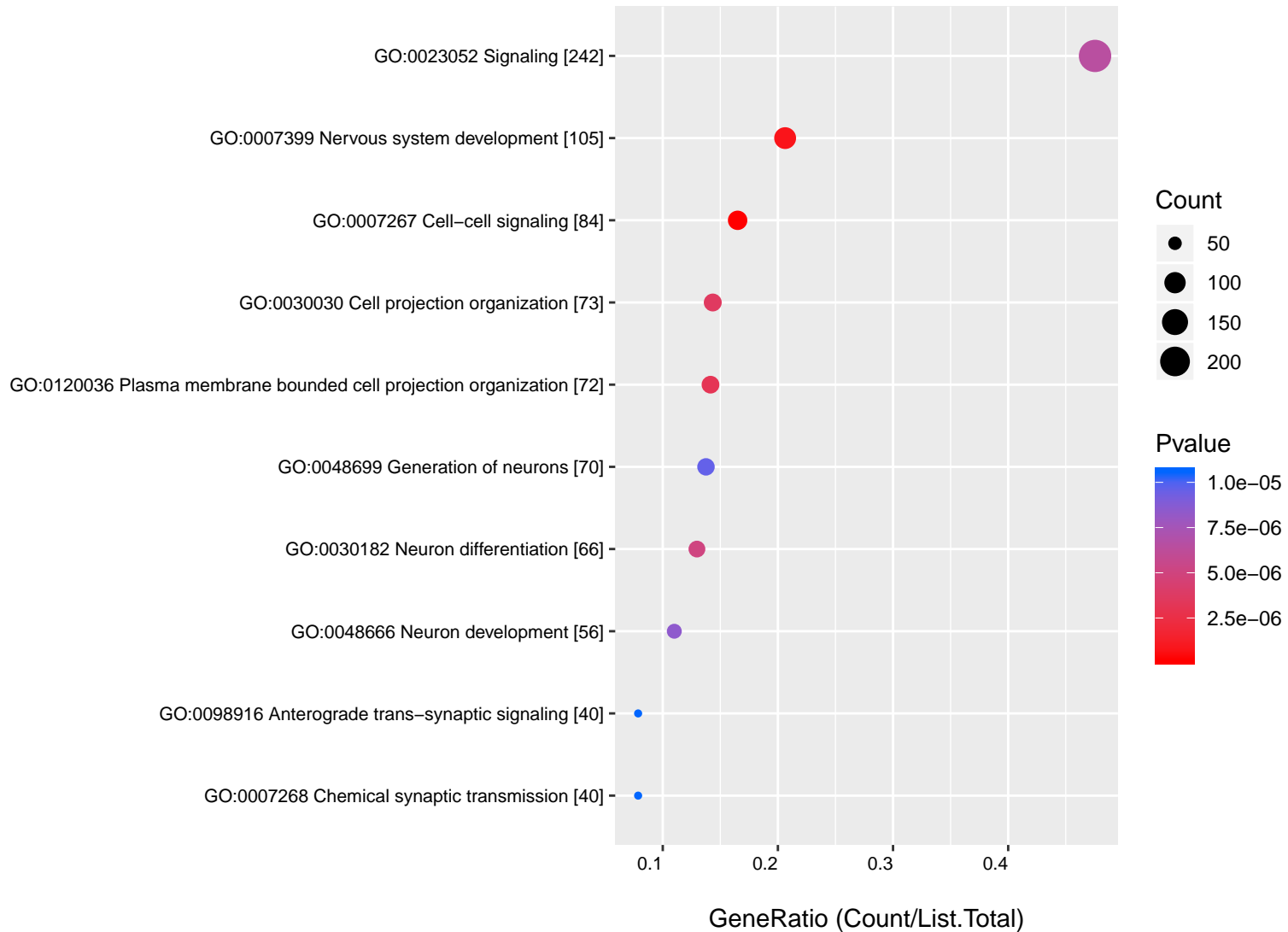

Supplement: Supplementary file 1 [file ijms-22-03792-s001.zip › Supplementary_File/C_ GO_Analysis_Results/16-30nt_go_Makona-96h-Huh7_vs_Control-96h-Huh7_down.mature_mirna_targets/BP_GeneRatioDotPlot.pdf]

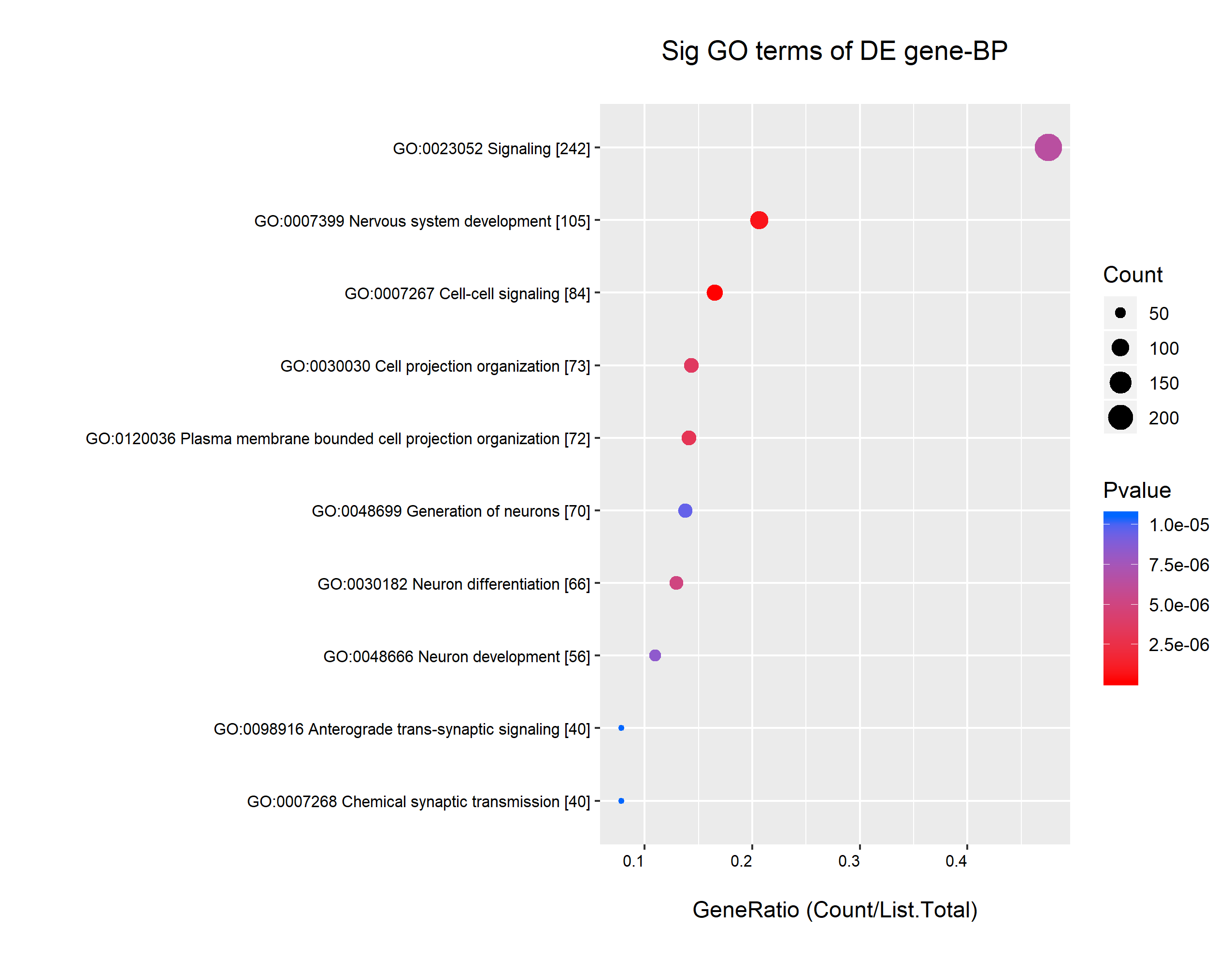

Supplement: Supplementary file 1 [file ijms-22-03792-s001.zip › Supplementary_File/C_ GO_Analysis_Results/16-30nt_go_Makona-96h-Huh7_vs_Control-96h-Huh7_down.mature_mirna_targets/BP_GeneRatioDotPlot.png]

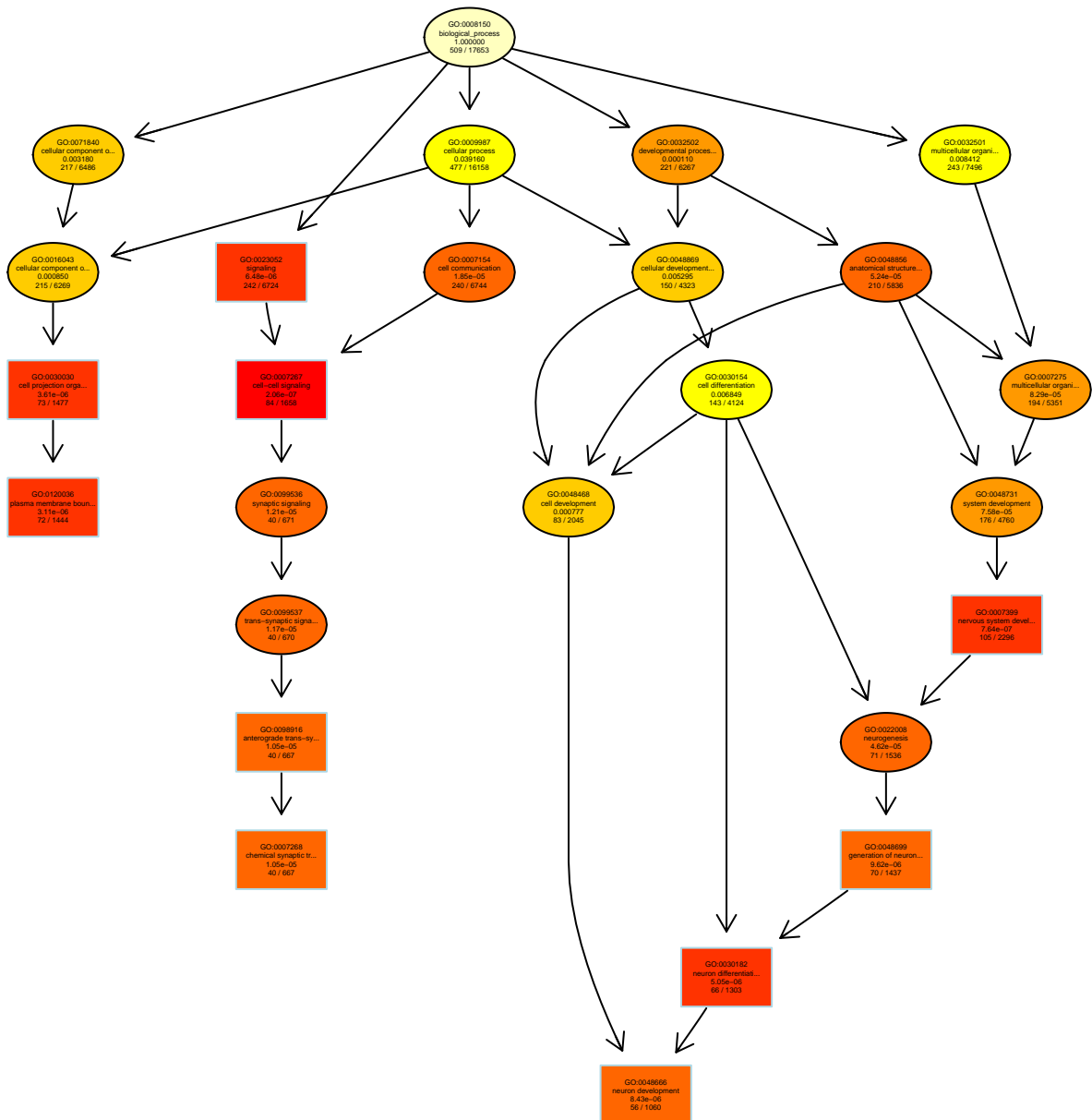

Supplement: Supplementary file 1 [file ijms-22-03792-s001.zip › Supplementary_File/C_ GO_Analysis_Results/16-30nt_go_Makona-96h-Huh7_vs_Control-96h-Huh7_down.mature_mirna_targets/BP_Pvalue_tree.pdf]

# GO Cellular Component Classification

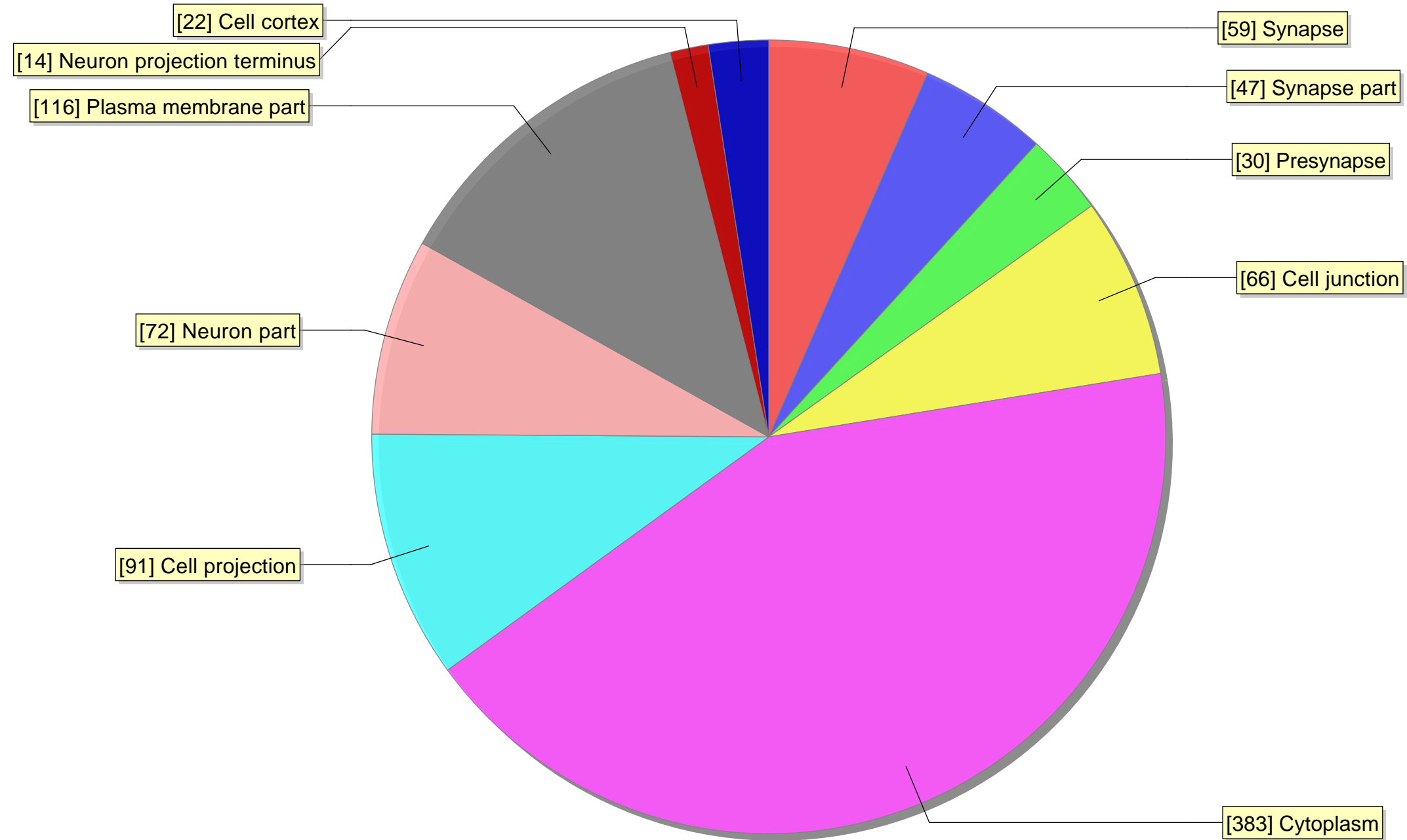

Supplement: Supplementary file 1 [file ijms-22-03792-s001.zip › Supplementary_File/C_ GO_Analysis_Results/16-30nt_go_Makona-96h-Huh7_vs_Control-96h-Huh7_down.mature_mirna_targets/CC_Count.pdf]

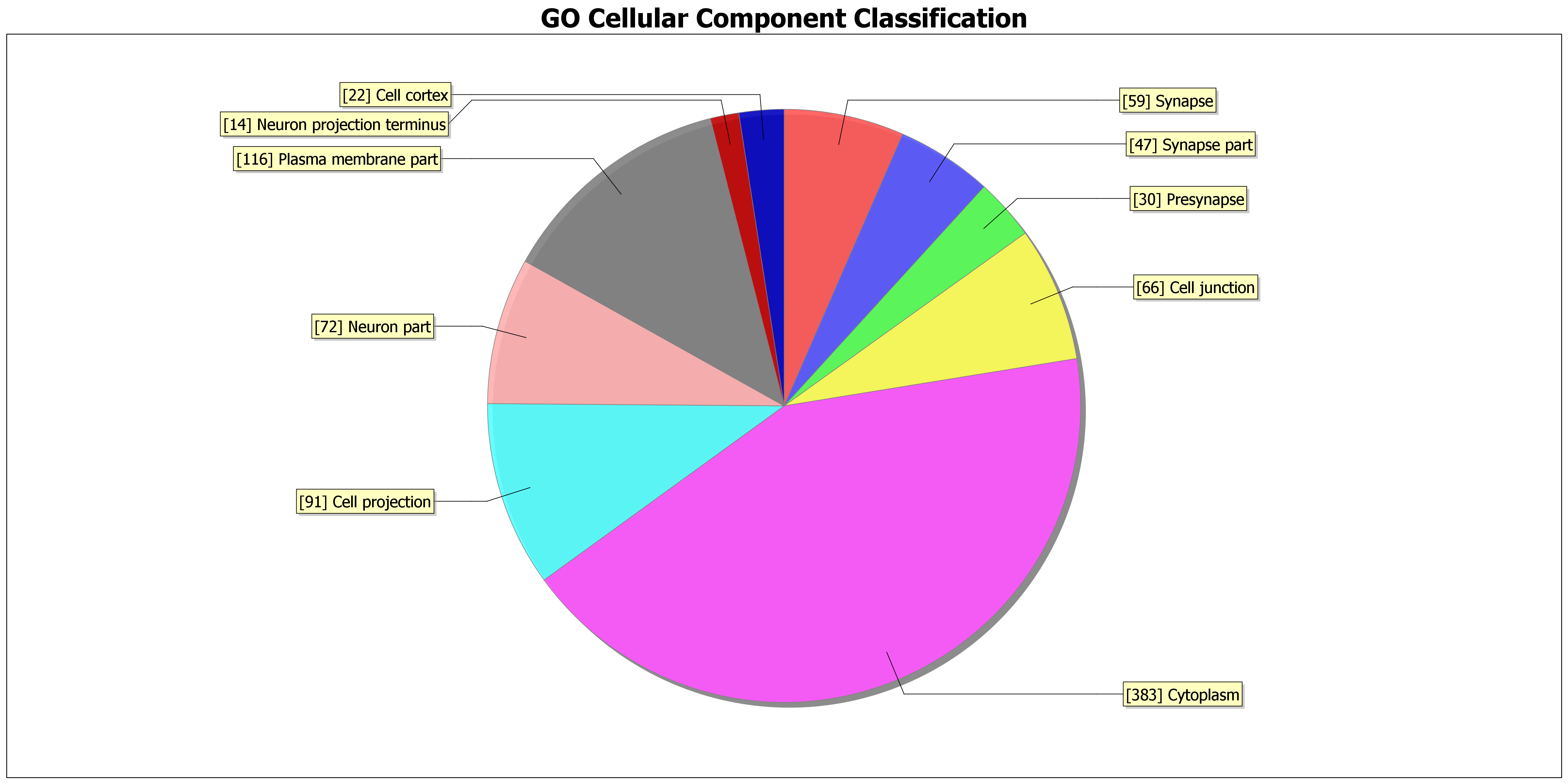

Supplement: Supplementary file 1 [file ijms-22-03792-s001.zip › Supplementary_File/C_ GO_Analysis_Results/16-30nt_go_Makona-96h-Huh7_vs_Control-96h-Huh7_down.mature_mirna_targets/CC_Count.png]

# Sig GO terms of DE gene-CC

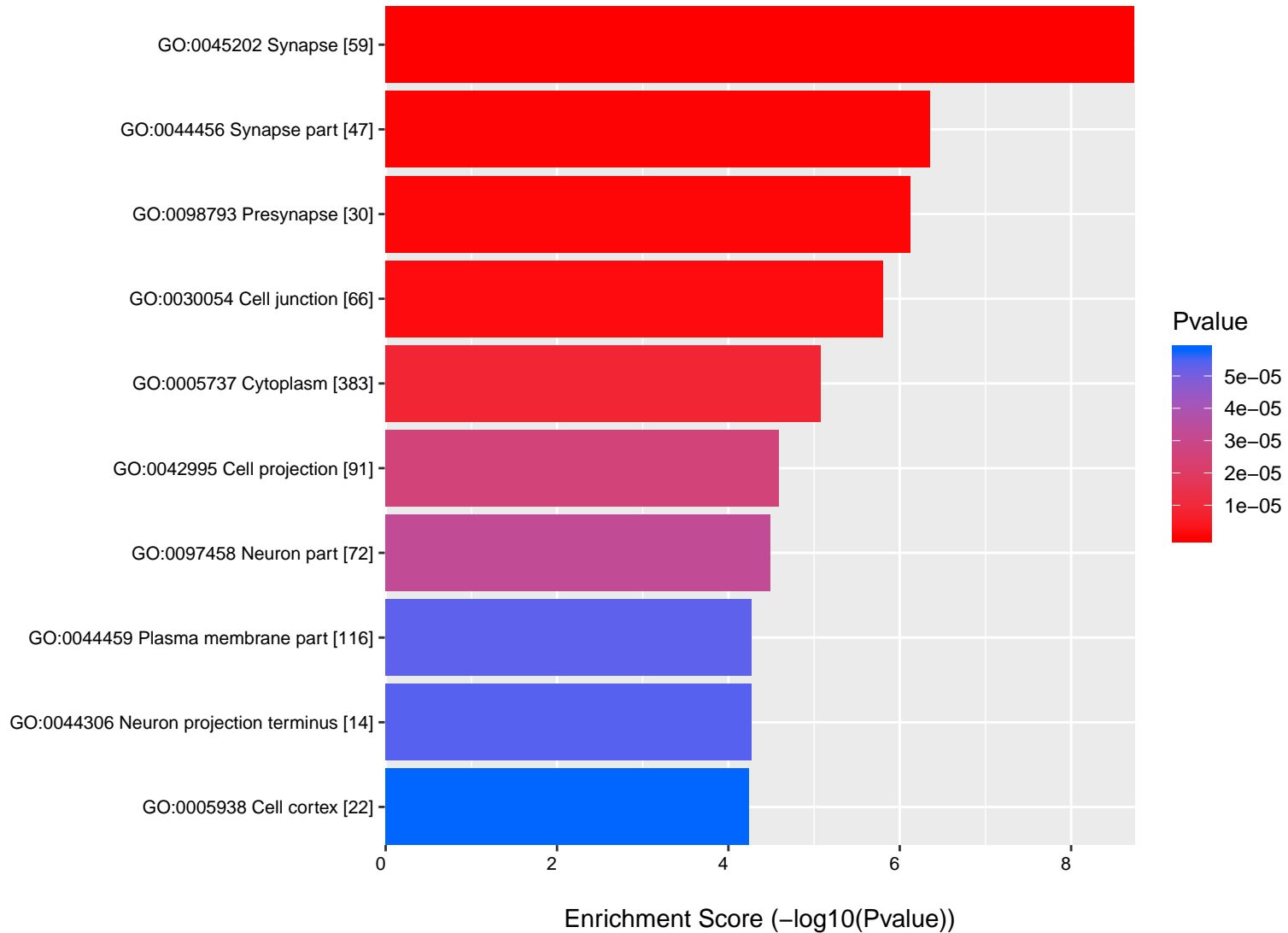

Supplement: Supplementary file 1 [file ijms-22-03792-s001.zip › Supplementary_File/C_ GO_Analysis_Results/16-30nt_go_Makona-96h-Huh7_vs_Control-96h-Huh7_down.mature_mirna_targets/CC_EnrichmentScore.pdf]

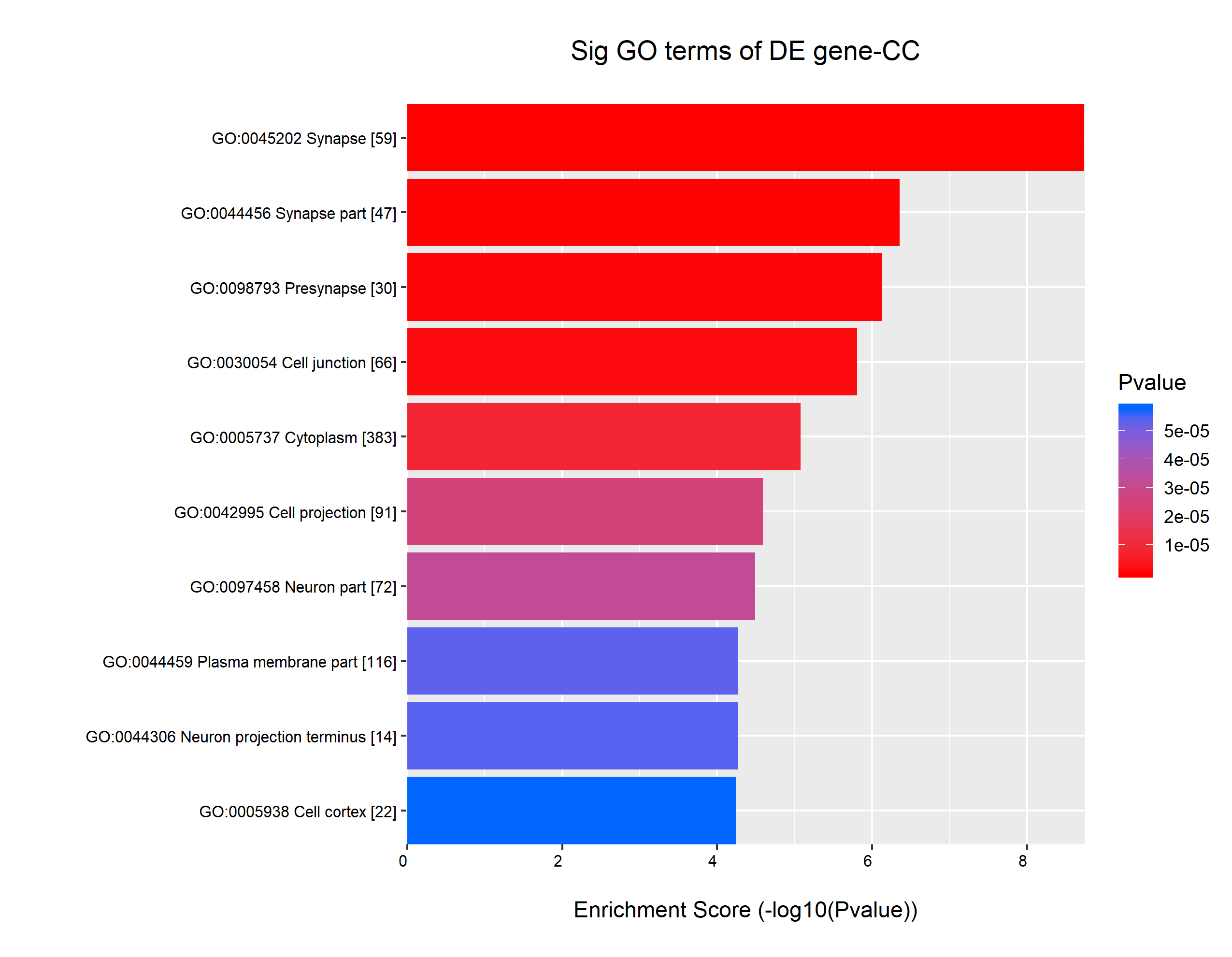

Supplement: Supplementary file 1 [file ijms-22-03792-s001.zip › Supplementary_File/C_ GO_Analysis_Results/16-30nt_go_Makona-96h-Huh7_vs_Control-96h-Huh7_down.mature_mirna_targets/CC_EnrichmentScore.png]

## Sig GO terms of DE gene-CC

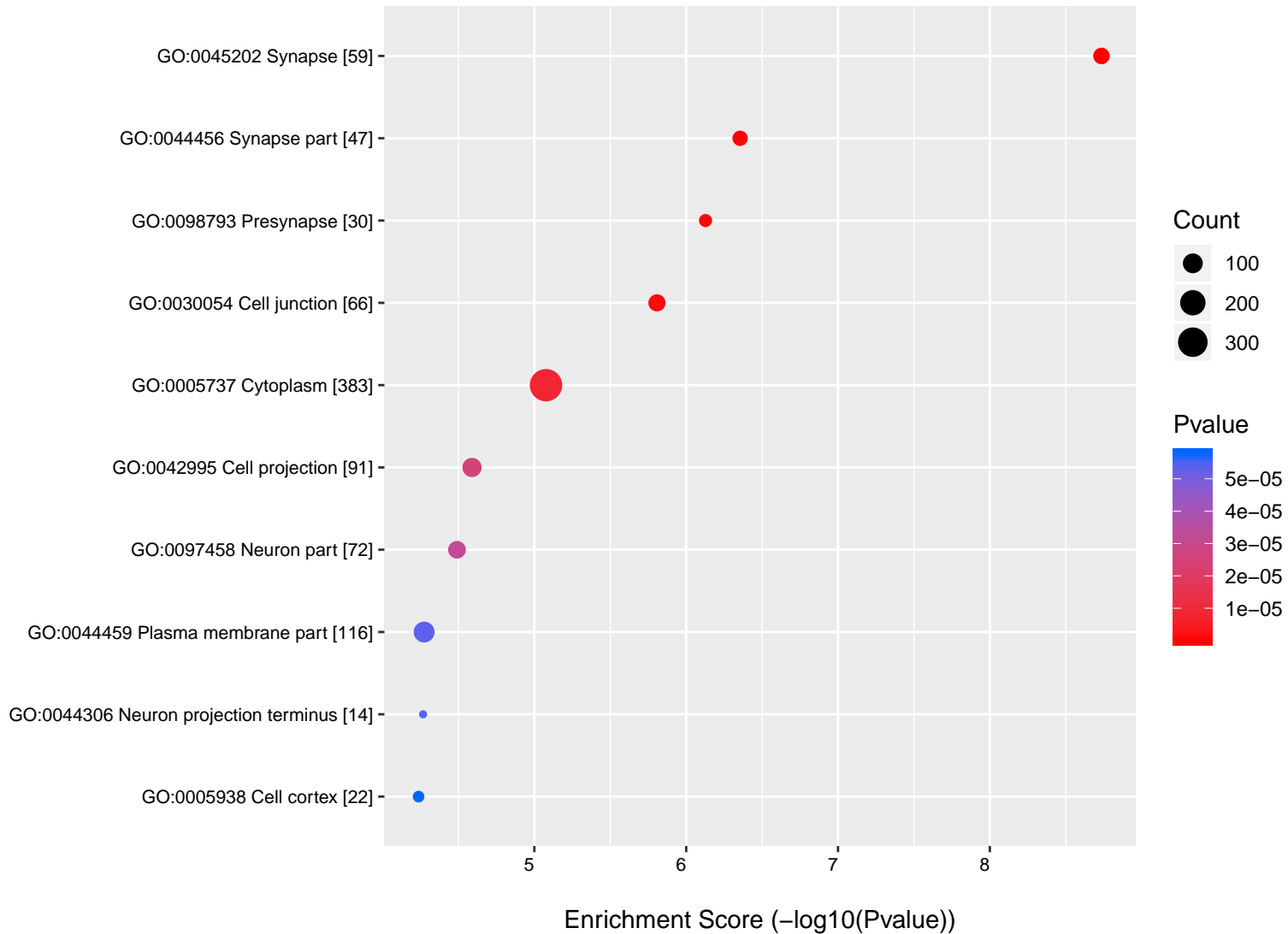

Supplement: Supplementary file 1 [file ijms-22-03792-s001.zip › Supplementary_File/C_ GO_Analysis_Results/16-30nt_go_Makona-96h-Huh7_vs_Control-96h-Huh7_down.mature_mirna_targets/CC_EnrichmentScoreDotPlot.pdf]

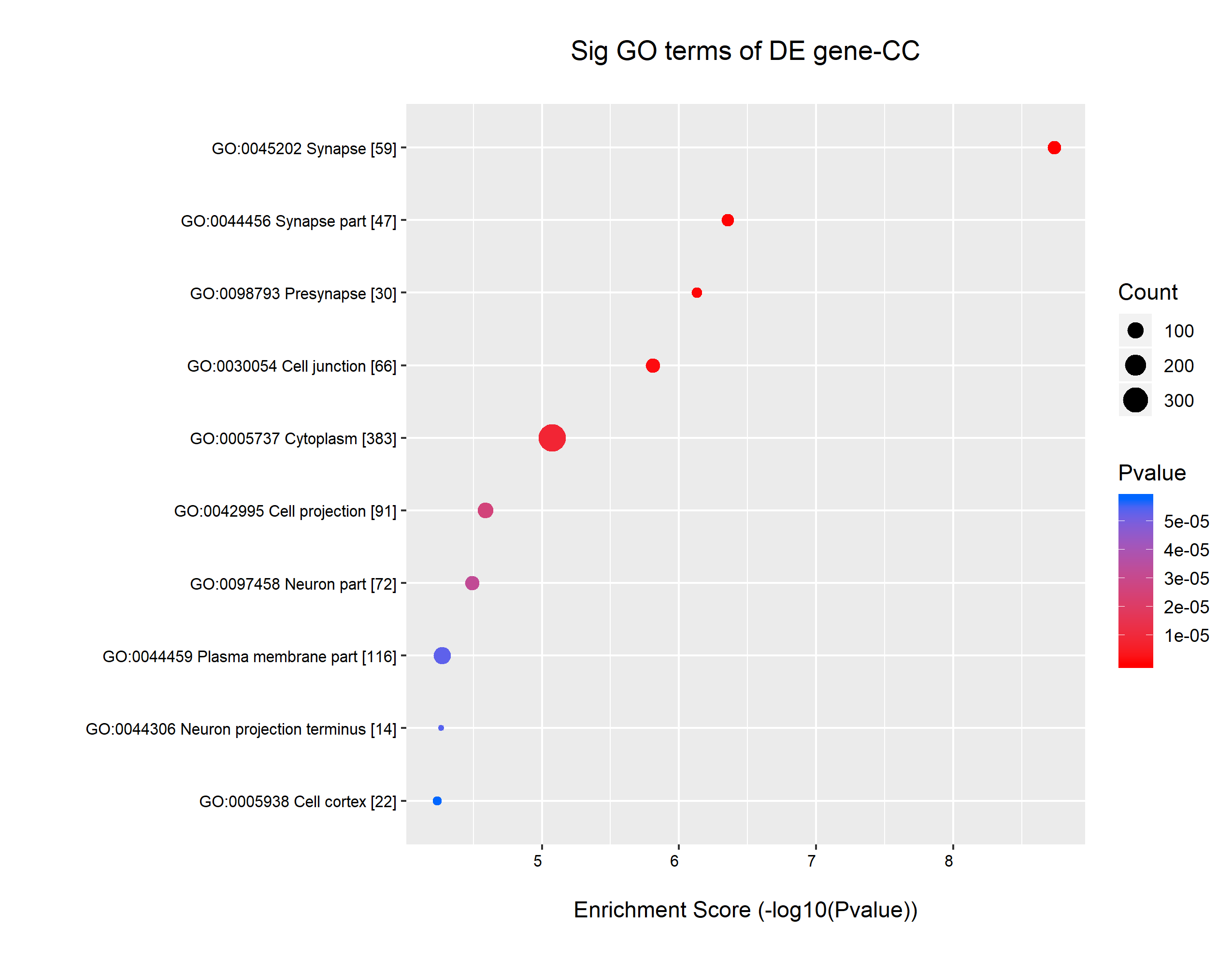

Supplement: Supplementary file 1 [file ijms-22-03792-s001.zip › Supplementary_File/C_ GO_Analysis_Results/16-30nt_go_Makona-96h-Huh7_vs_Control-96h-Huh7_down.mature_mirna_targets/CC_EnrichmentScoreDotPlot.png]

## Sig GO terms of DE gene-CC

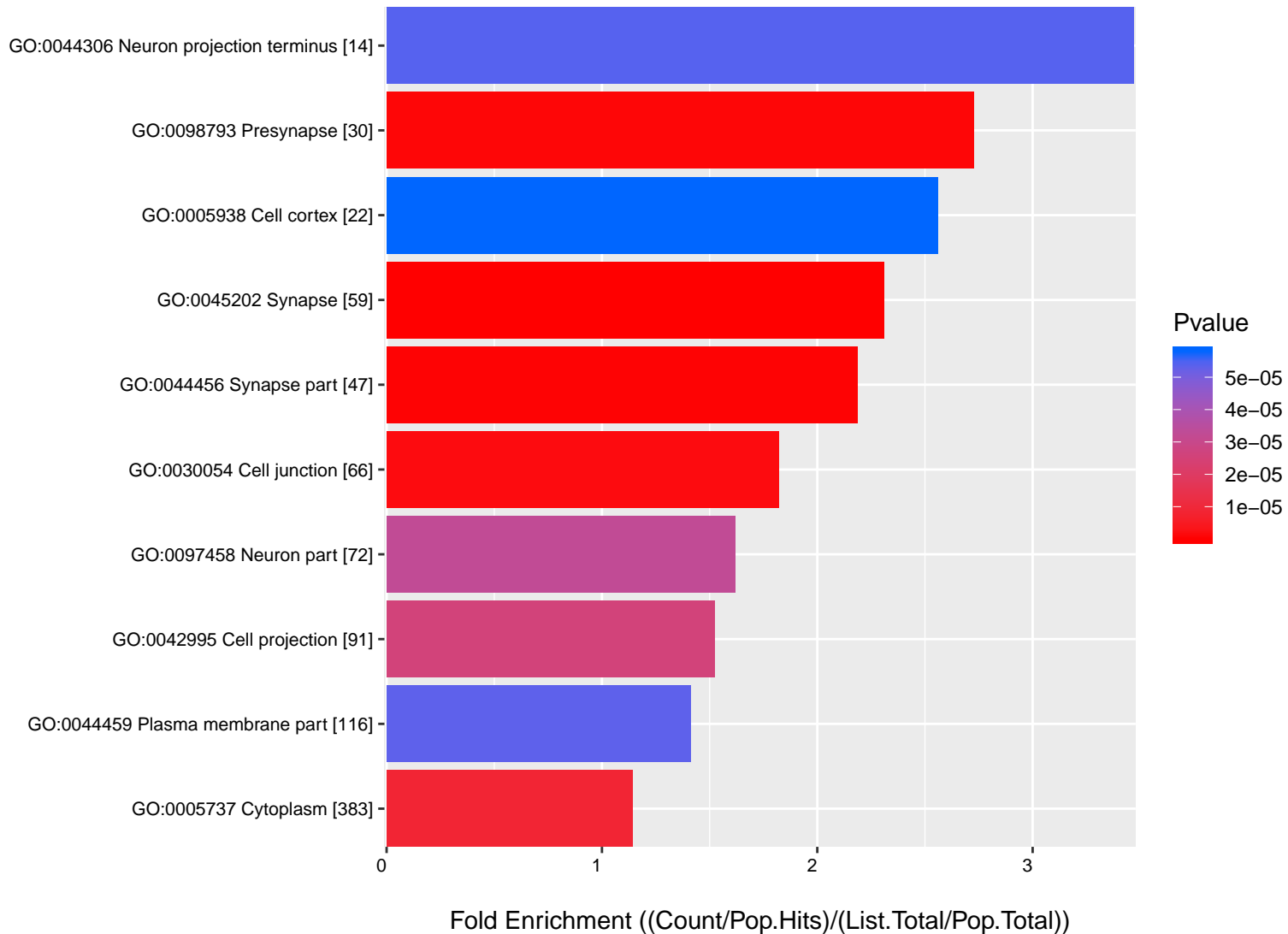

Supplement: Supplementary file 1 [file ijms-22-03792-s001.zip › Supplementary_File/C_ GO_Analysis_Results/16-30nt_go_Makona-96h-Huh7_vs_Control-96h-Huh7_down.mature_mirna_targets/CC_FoldEnrichment.pdf]

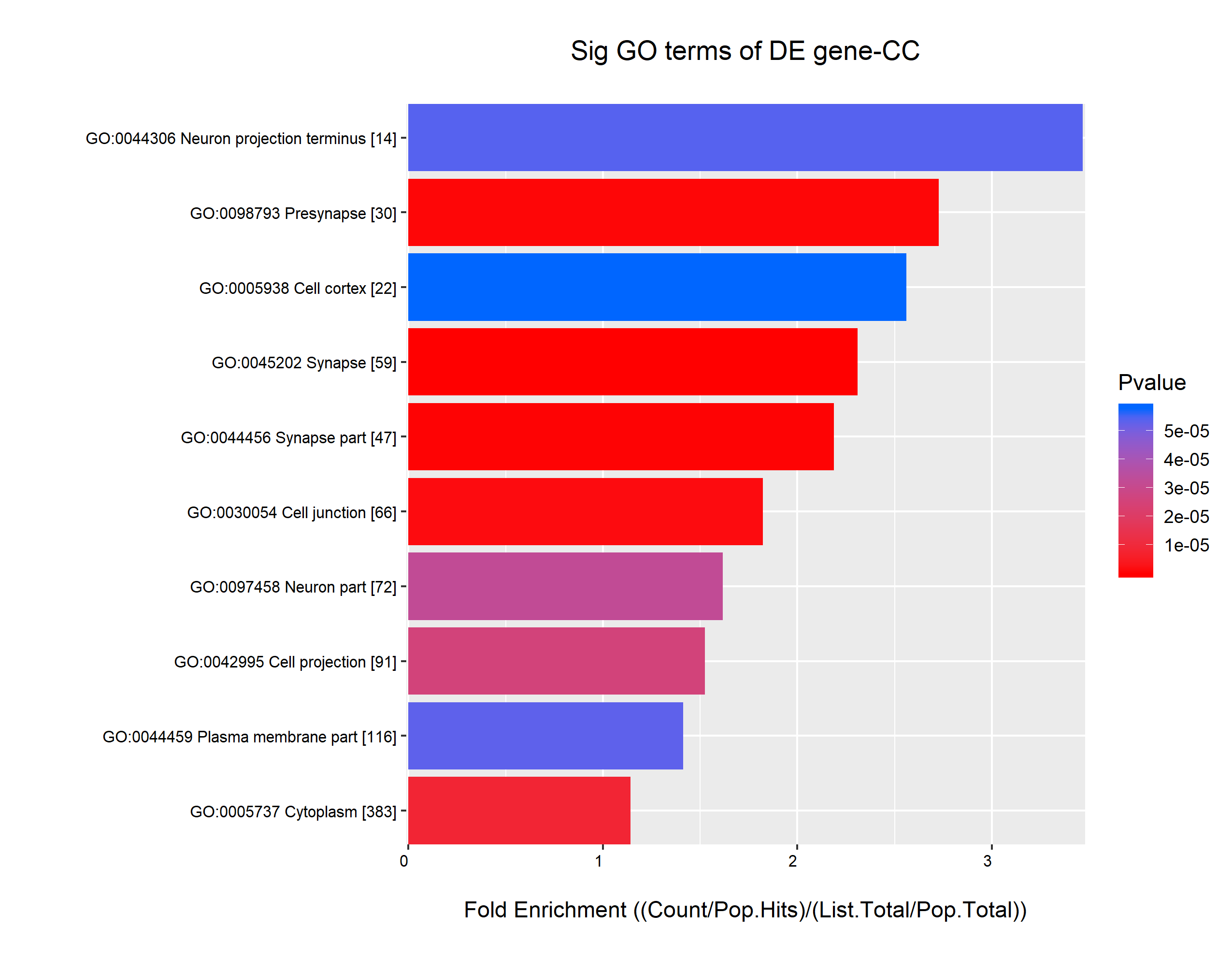

Supplement: Supplementary file 1 [file ijms-22-03792-s001.zip › Supplementary_File/C_ GO_Analysis_Results/16-30nt_go_Makona-96h-Huh7_vs_Control-96h-Huh7_down.mature_mirna_targets/CC_FoldEnrichment.png]

## Sig GO terms of DE gene-CC

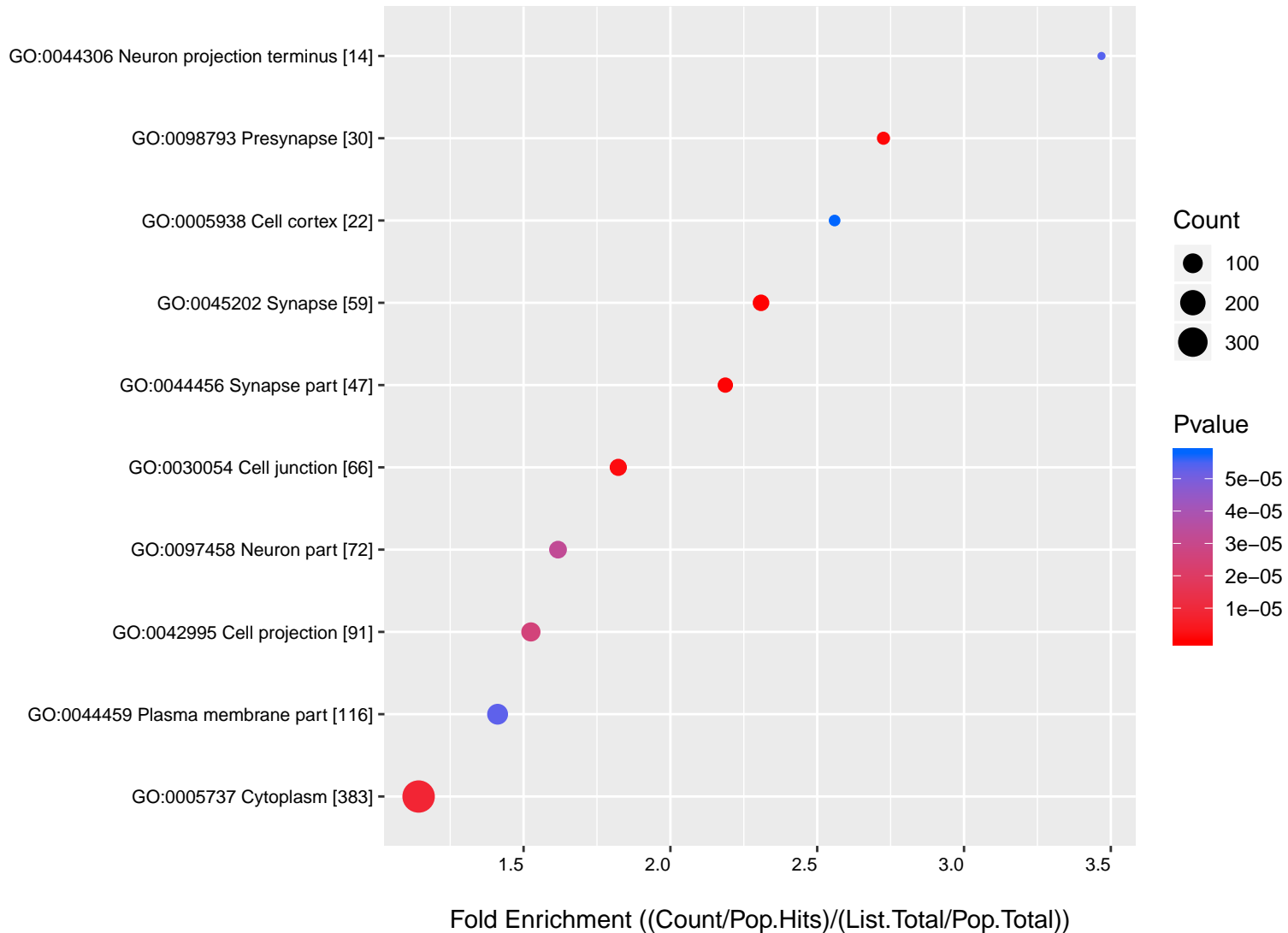

Supplement: Supplementary file 1 [file ijms-22-03792-s001.zip › Supplementary_File/C_ GO_Analysis_Results/16-30nt_go_Makona-96h-Huh7_vs_Control-96h-Huh7_down.mature_mirna_targets/CC_FoldEnrichmentDotPlot.pdf]

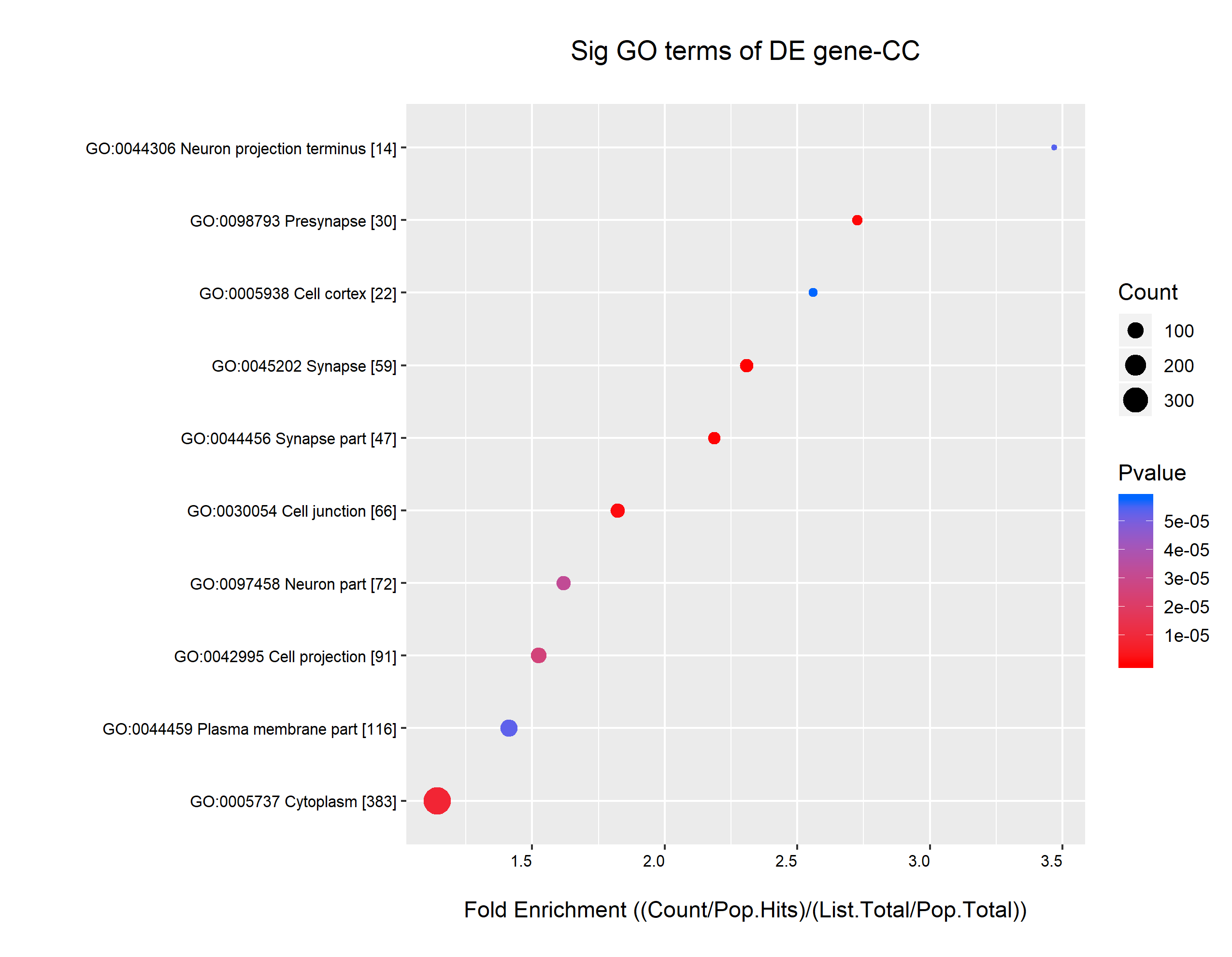

Supplement: Supplementary file 1 [file ijms-22-03792-s001.zip › Supplementary_File/C_ GO_Analysis_Results/16-30nt_go_Makona-96h-Huh7_vs_Control-96h-Huh7_down.mature_mirna_targets/CC_FoldEnrichmentDotPlot.png]

## Sig GO terms of DE gene-CC

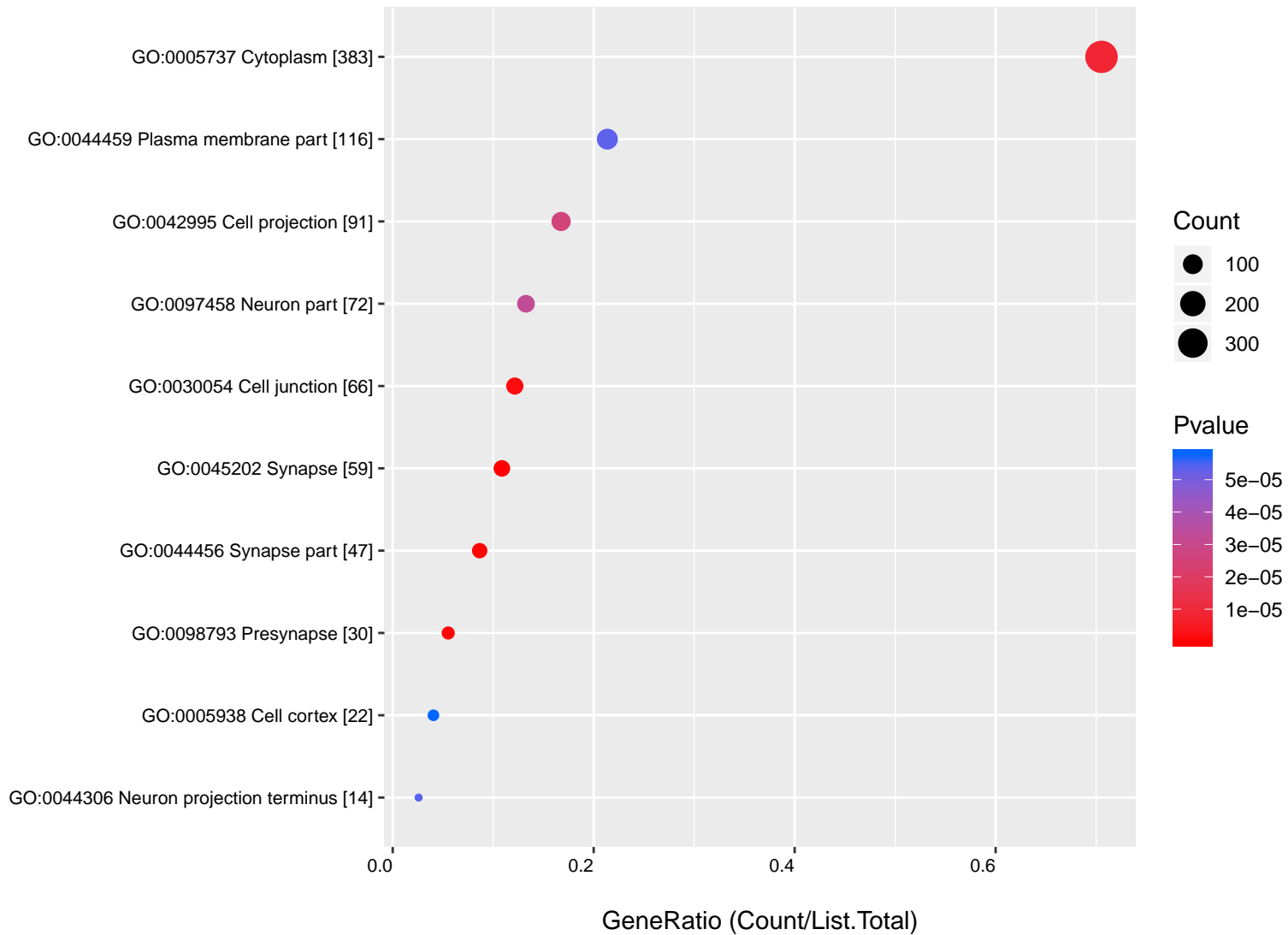

Supplement: Supplementary file 1 [file ijms-22-03792-s001.zip › Supplementary_File/C_ GO_Analysis_Results/16-30nt_go_Makona-96h-Huh7_vs_Control-96h-Huh7_down.mature_mirna_targets/CC_GeneRatioDotPlot.pdf]

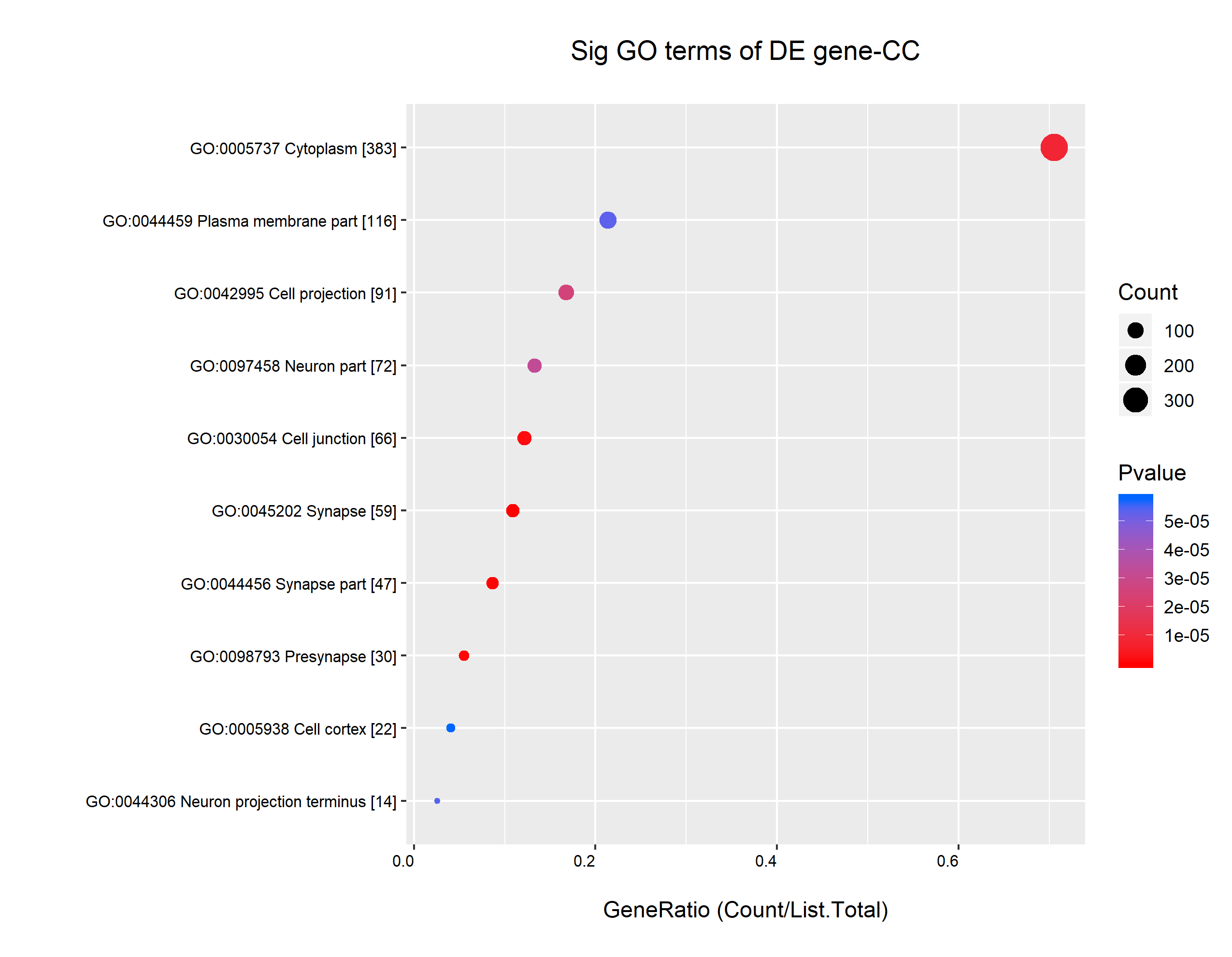

Supplement: Supplementary file 1 [file ijms-22-03792-s001.zip › Supplementary_File/C_ GO_Analysis_Results/16-30nt_go_Makona-96h-Huh7_vs_Control-96h-Huh7_down.mature_mirna_targets/CC_GeneRatioDotPlot.png]

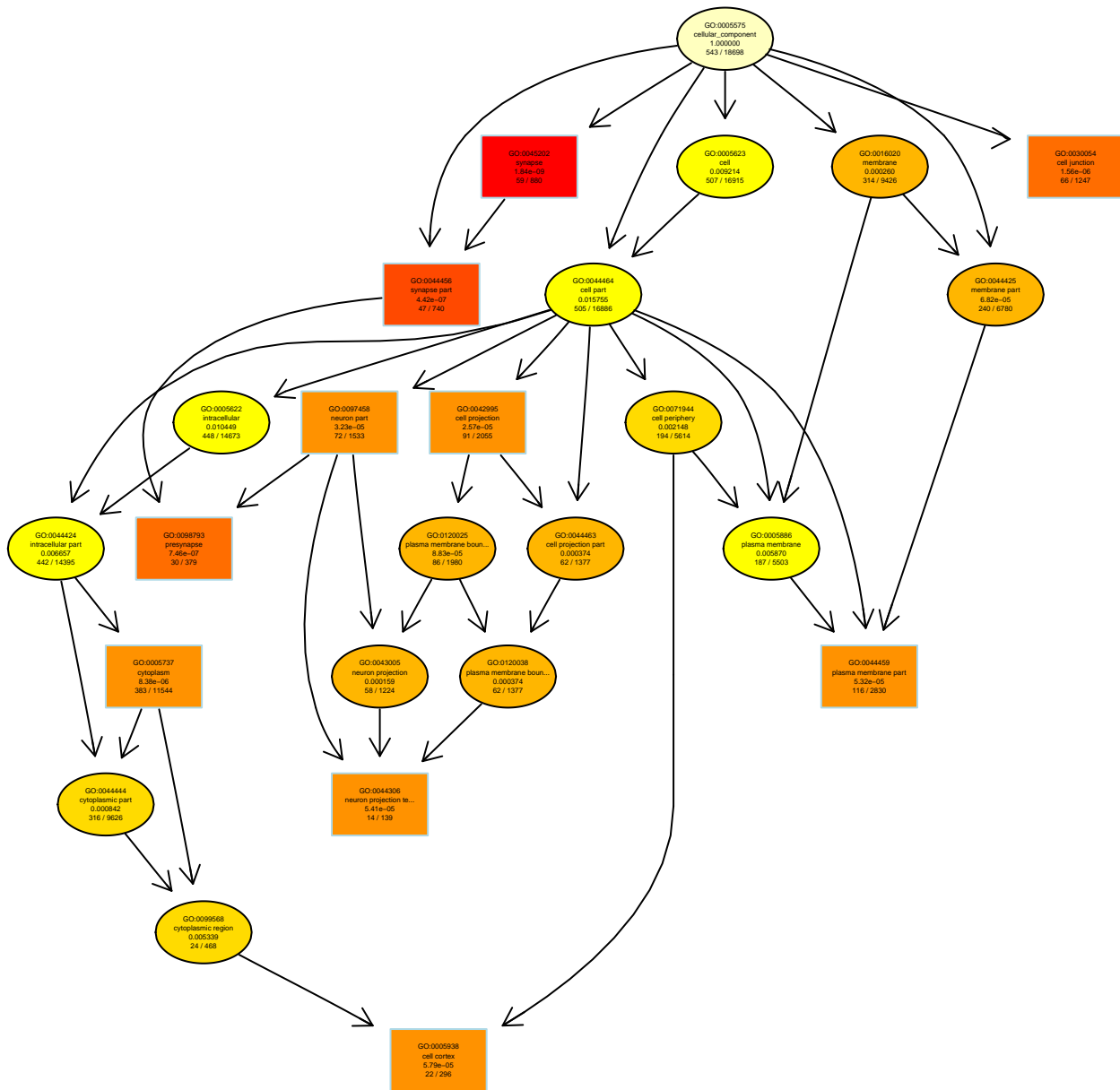

Supplement: Supplementary file 1 [file ijms-22-03792-s001.zip › Supplementary_File/C_ GO_Analysis_Results/16-30nt_go_Makona-96h-Huh7_vs_Control-96h-Huh7_down.mature_mirna_targets/CC_Pvalue_tree.pdf]

Sig GO terms of DE gene

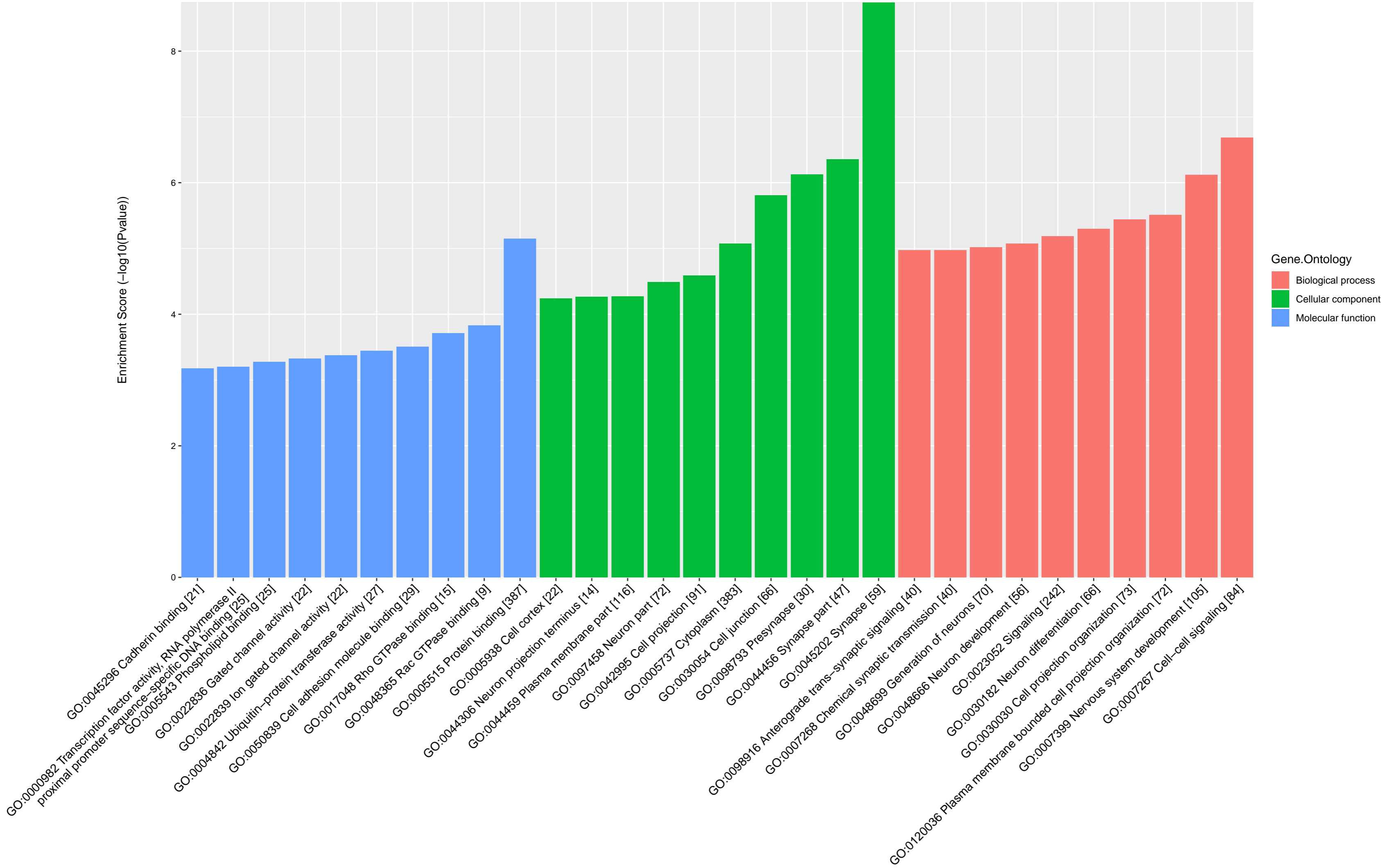

Supplement: Supplementary file 1 [file ijms-22-03792-s001.zip › Supplementary_File/C_ GO_Analysis_Results/16-30nt_go_Makona-96h-Huh7_vs_Control-96h-Huh7_down.mature_mirna_targets/GeneOntology_EnrichmentScore.pdf]

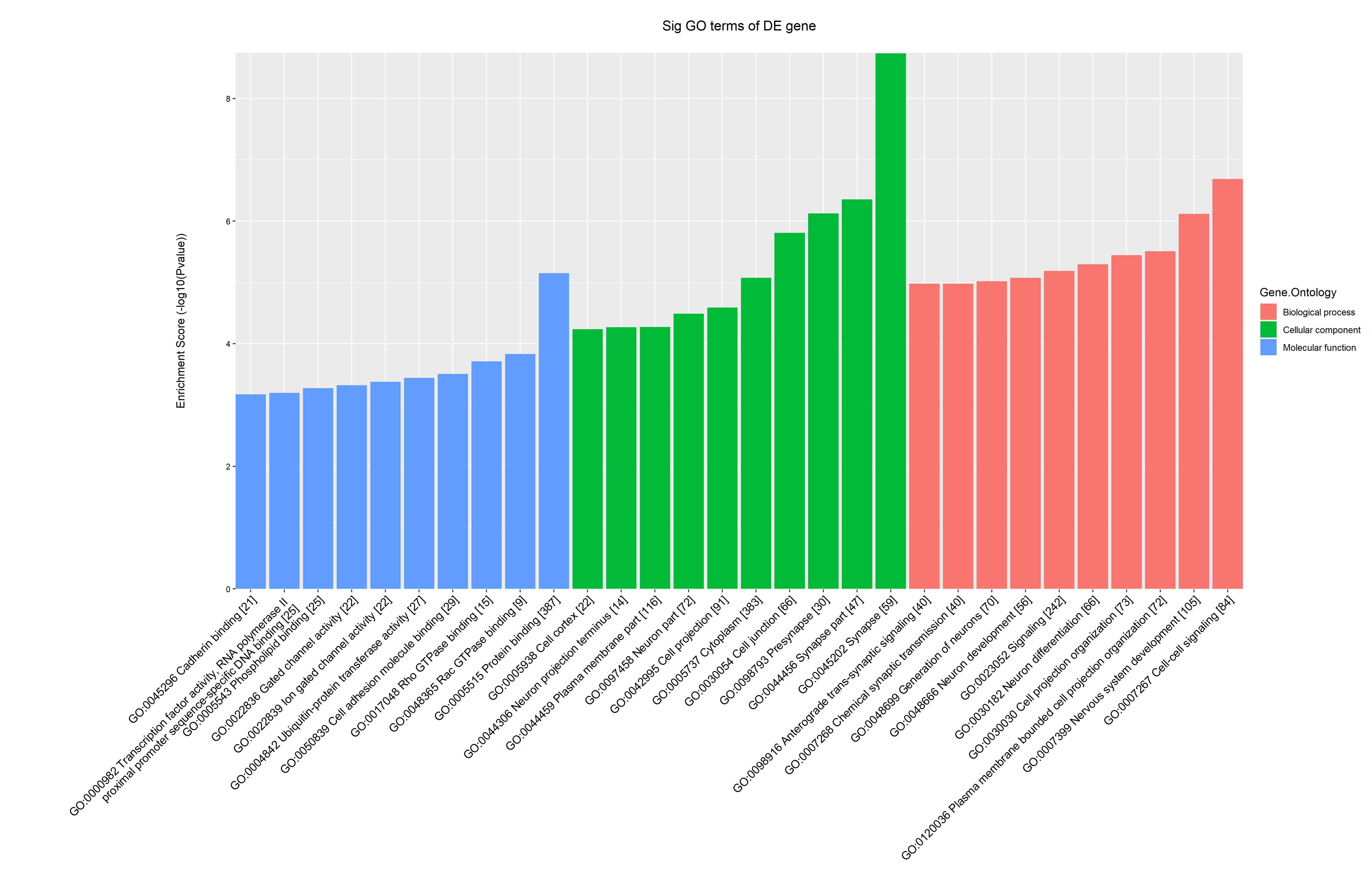

Supplement: Supplementary file 1 [file ijms-22-03792-s001.zip › Supplementary_File/C_ GO_Analysis_Results/16-30nt_go_Makona-96h-Huh7_vs_Control-96h-Huh7_down.mature_mirna_targets/GeneOntology_EnrichmentScore.png]

Sig GO terms of DE gene

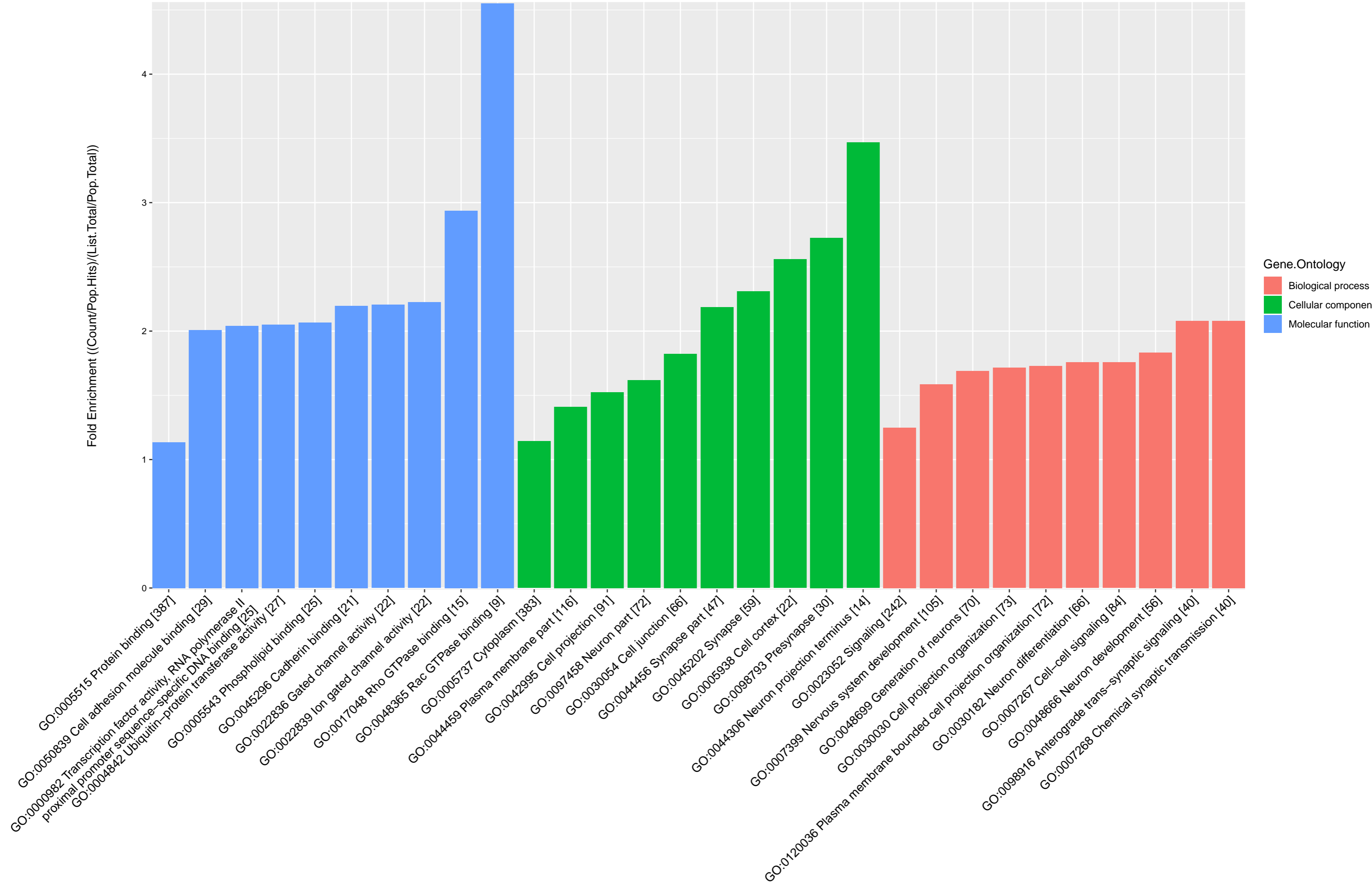

Supplement: Supplementary file 1 [file ijms-22-03792-s001.zip › Supplementary_File/C_ GO_Analysis_Results/16-30nt_go_Makona-96h-Huh7_vs_Control-96h-Huh7_down.mature_mirna_targets/GeneOntology_FoldEnrichment.pdf]

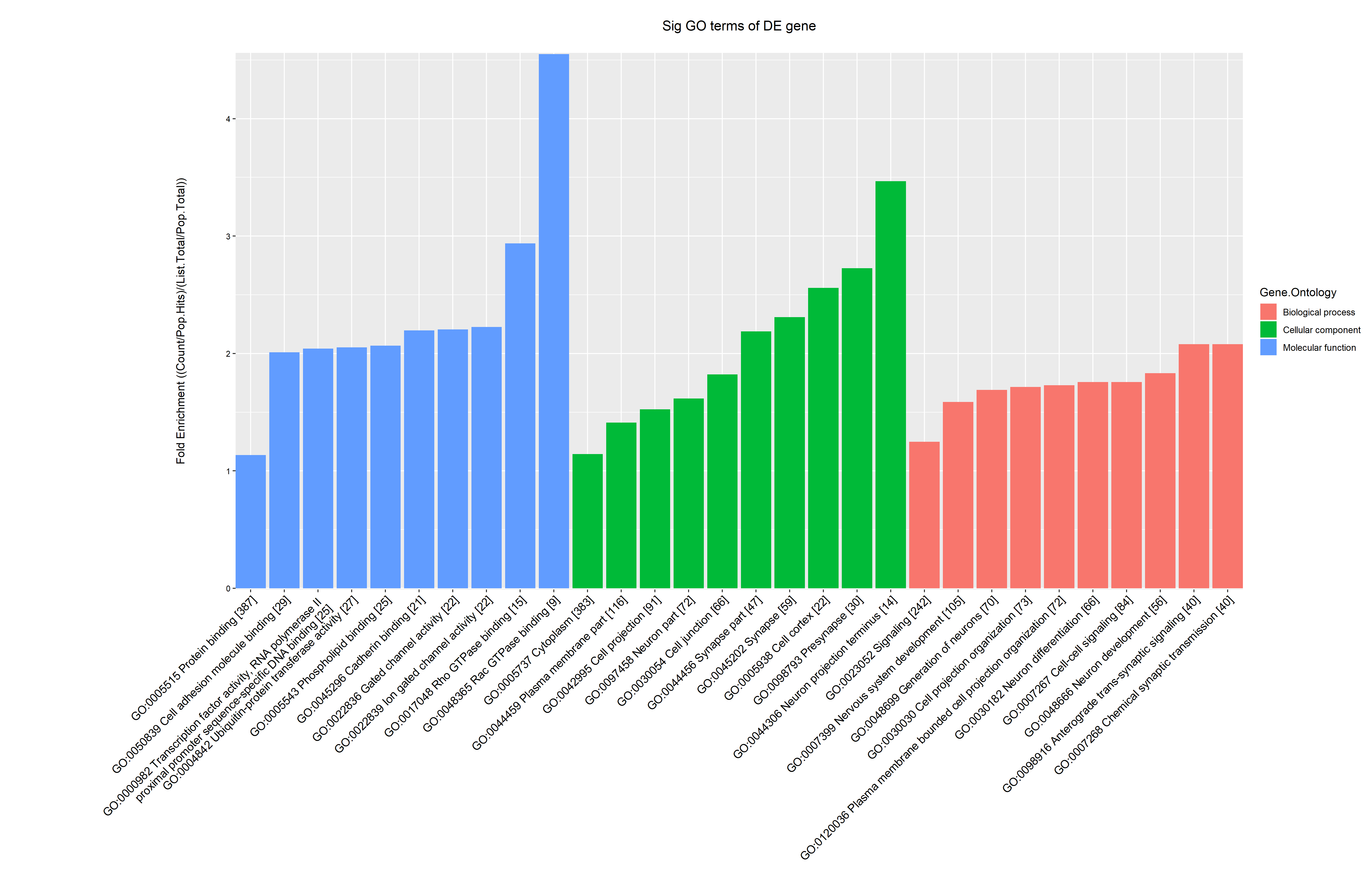

Supplement: Supplementary file 1 [file ijms-22-03792-s001.zip › Supplementary_File/C_ GO_Analysis_Results/16-30nt_go_Makona-96h-Huh7_vs_Control-96h-Huh7_down.mature_mirna_targets/GeneOntology_FoldEnrichment.png]

# GO Molecular Function Classification

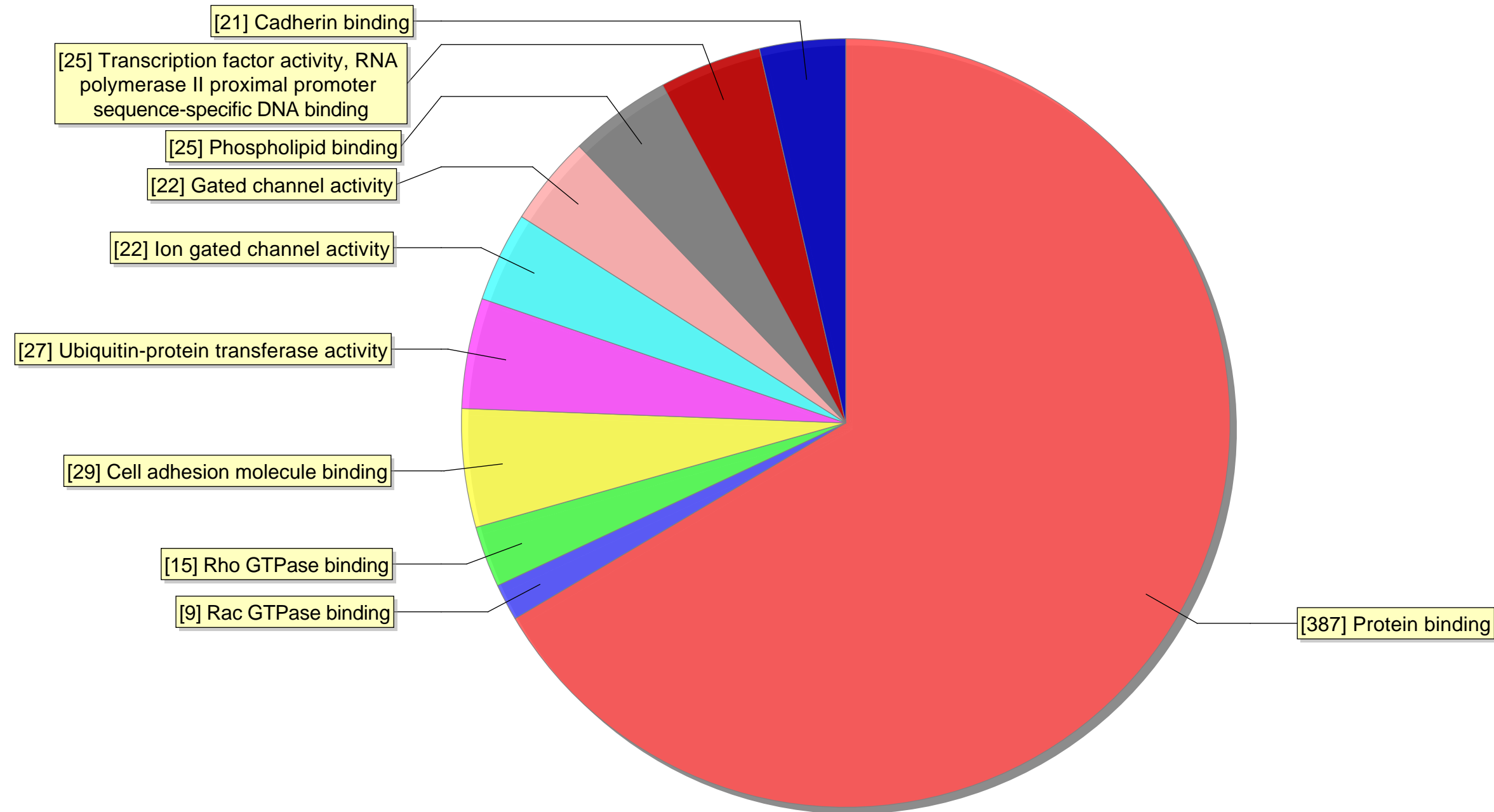

Supplement: Supplementary file 1 [file ijms-22-03792-s001.zip › Supplementary_File/C_ GO_Analysis_Results/16-30nt_go_Makona-96h-Huh7_vs_Control-96h-Huh7_down.mature_mirna_targets/MF_Count.pdf]

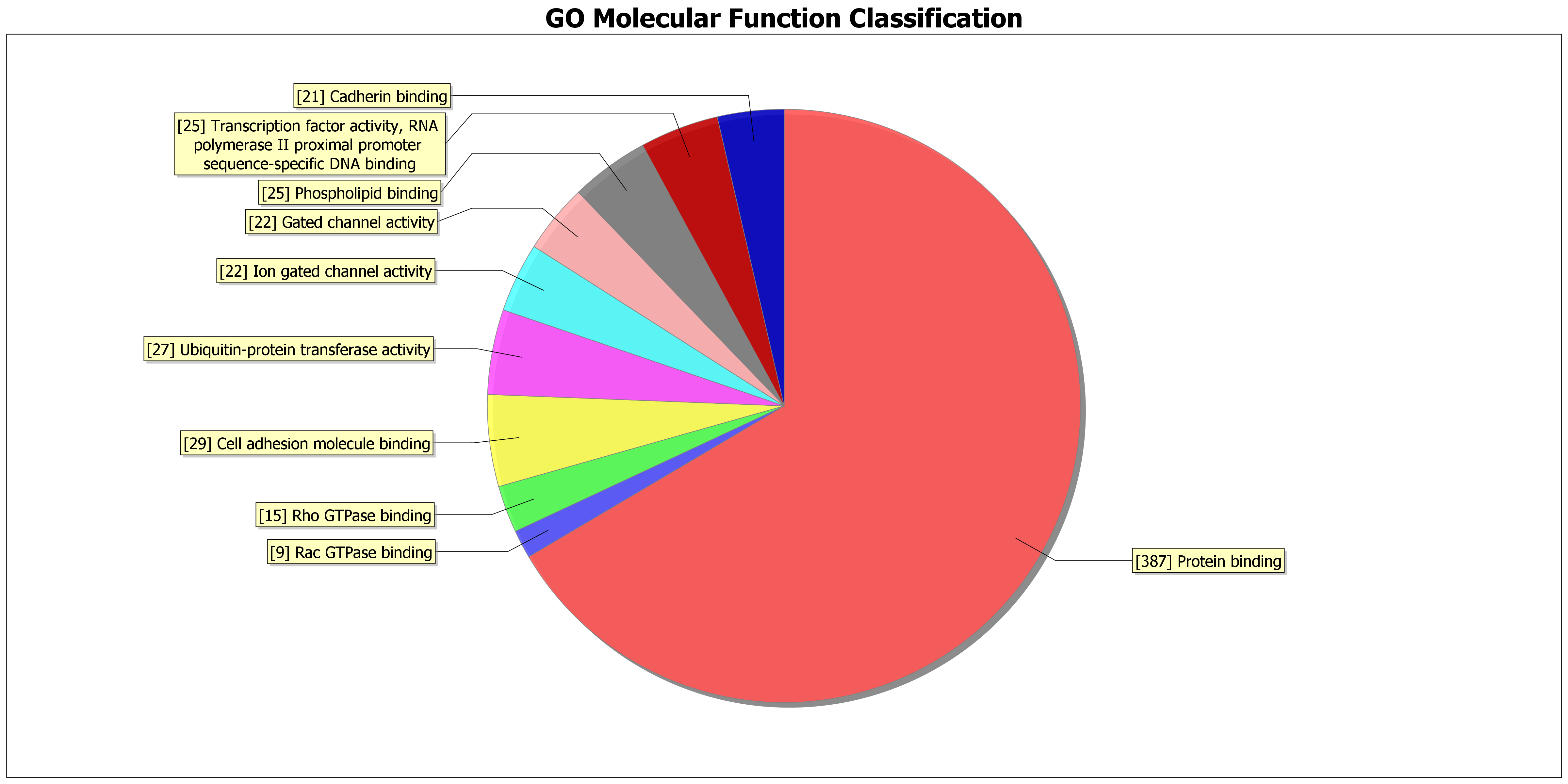

Supplement: Supplementary file 1 [file ijms-22-03792-s001.zip › Supplementary_File/C_ GO_Analysis_Results/16-30nt_go_Makona-96h-Huh7_vs_Control-96h-Huh7_down.mature_mirna_targets/MF_Count.png]

## Sig GO terms of DE gene-MF

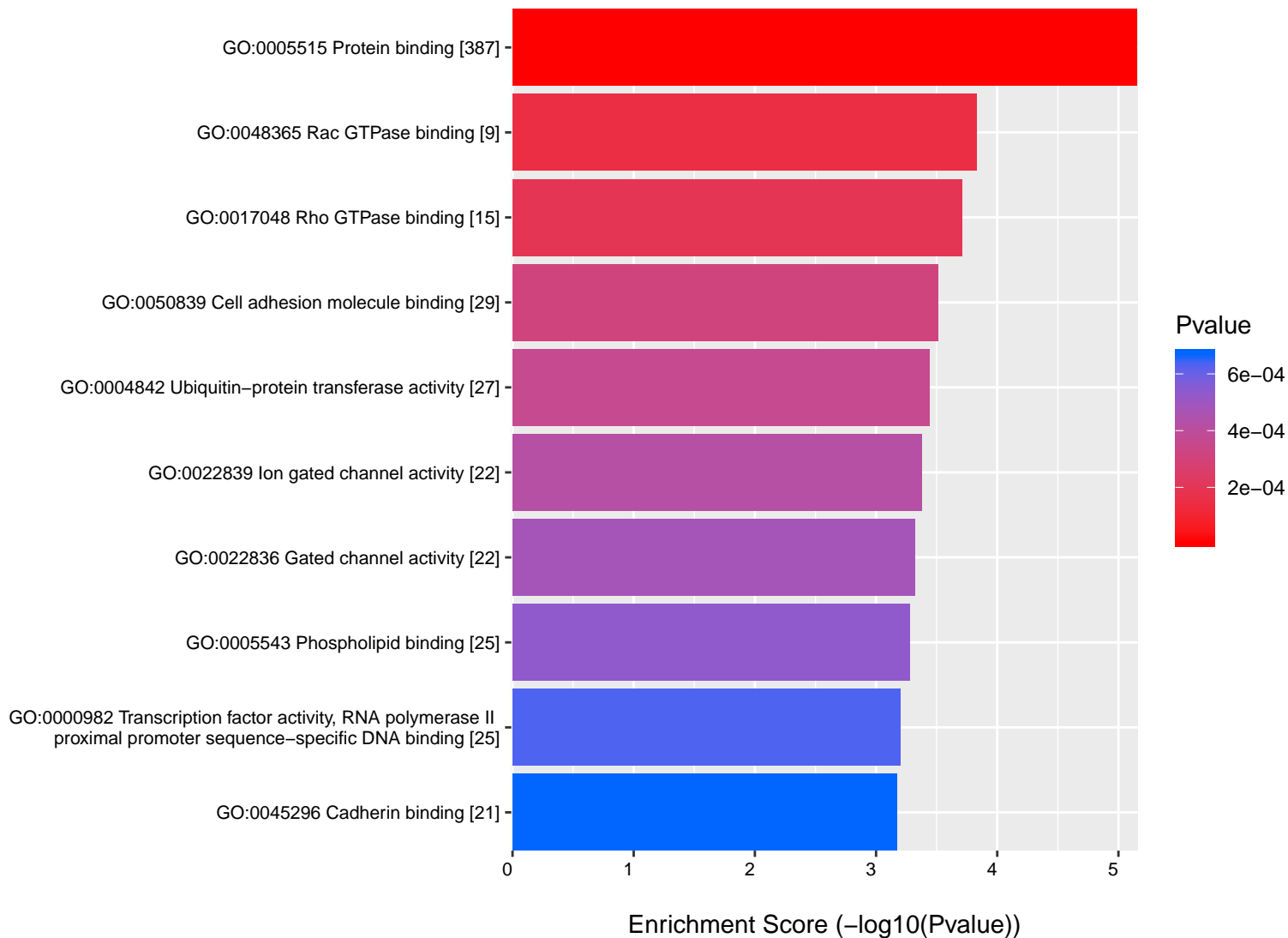

Supplement: Supplementary file 1 [file ijms-22-03792-s001.zip › Supplementary_File/C_ GO_Analysis_Results/16-30nt_go_Makona-96h-Huh7_vs_Control-96h-Huh7_down.mature_mirna_targets/MF_EnrichmentScore.pdf]

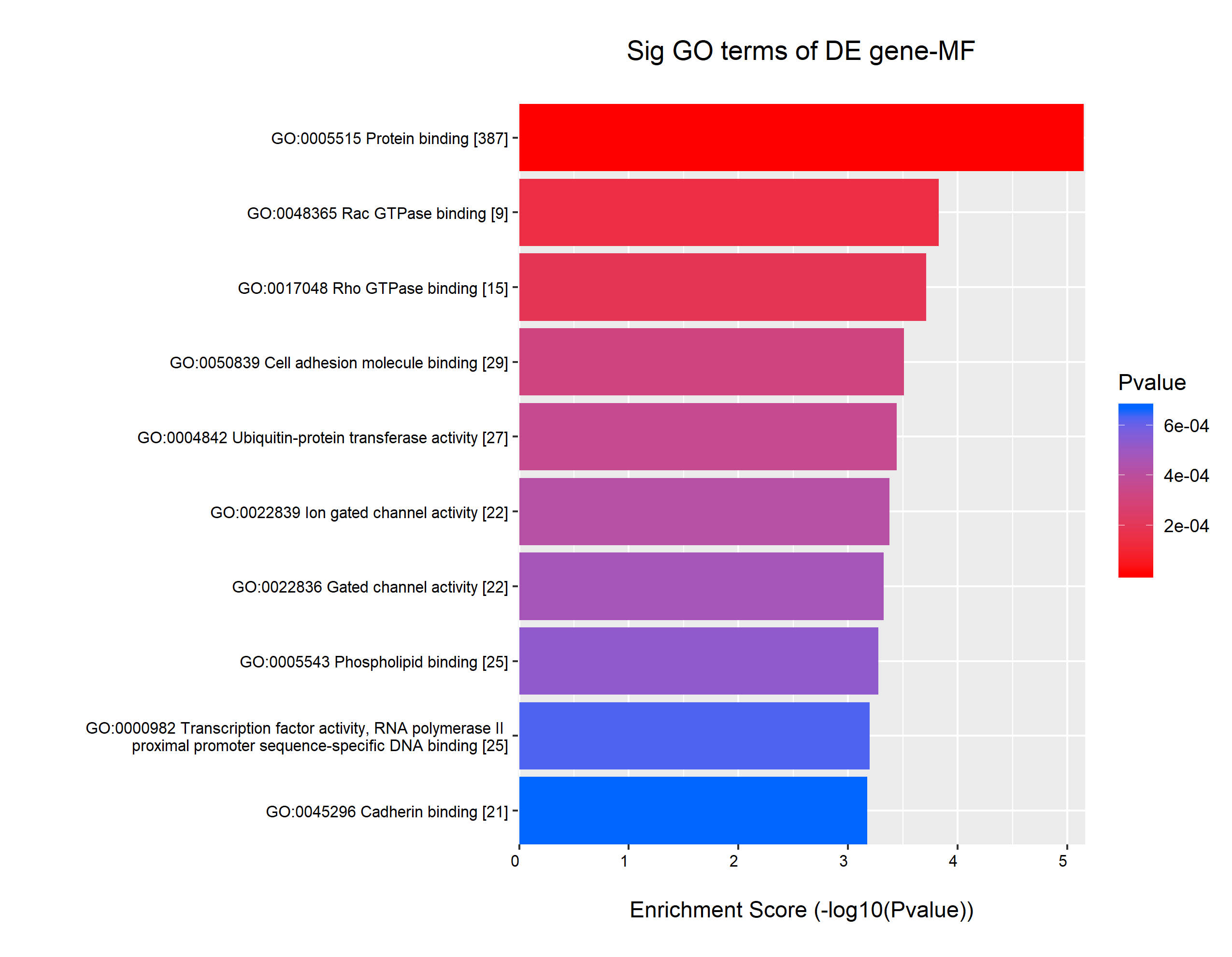

Supplement: Supplementary file 1 [file ijms-22-03792-s001.zip › Supplementary_File/C_ GO_Analysis_Results/16-30nt_go_Makona-96h-Huh7_vs_Control-96h-Huh7_down.mature_mirna_targets/MF_EnrichmentScore.png]

## Sig GO terms of DE gene-MF

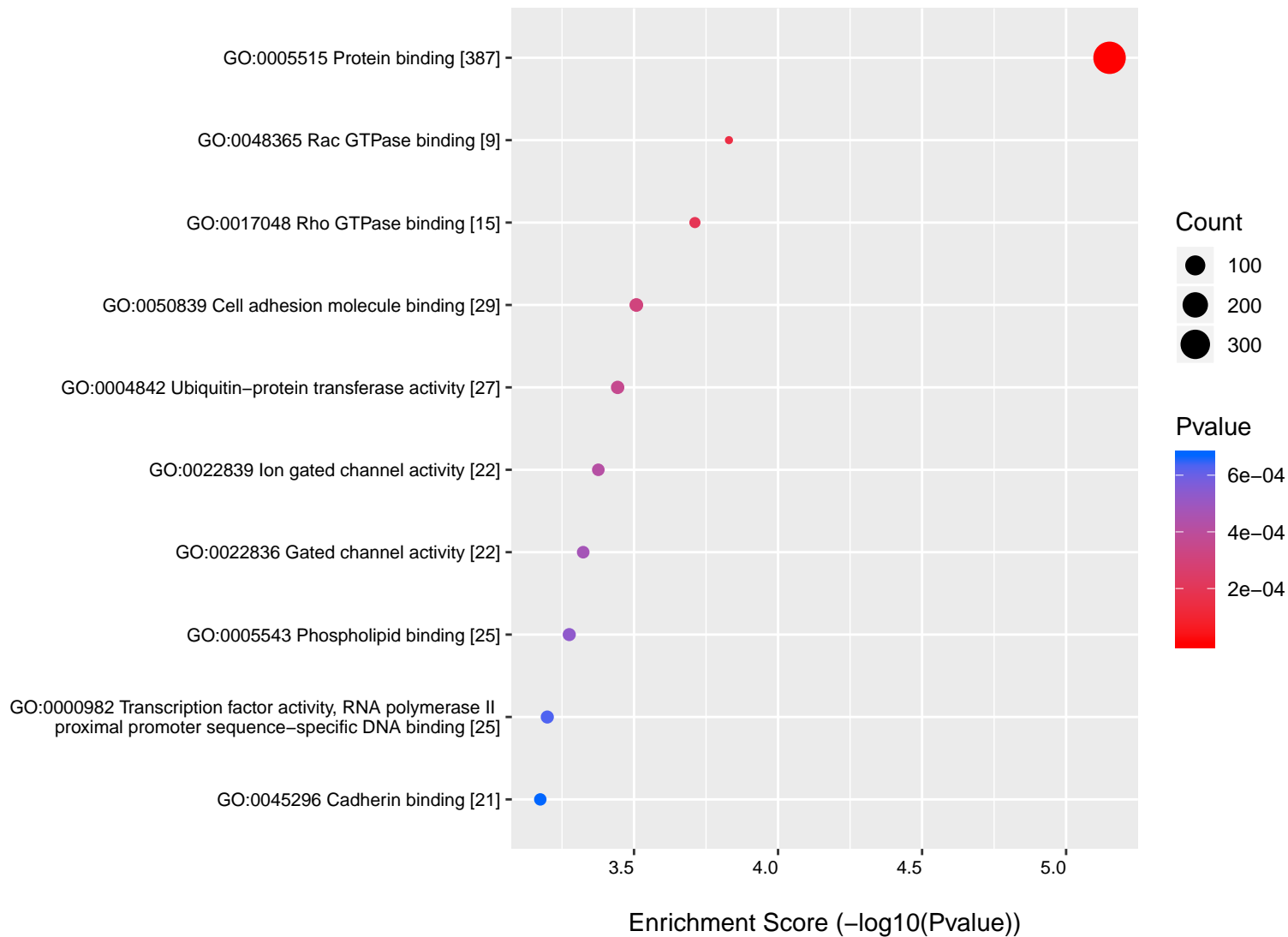

Supplement: Supplementary file 1 [file ijms-22-03792-s001.zip › Supplementary_File/C_ GO_Analysis_Results/16-30nt_go_Makona-96h-Huh7_vs_Control-96h-Huh7_down.mature_mirna_targets/MF_EnrichmentScoreDotPlot.pdf]

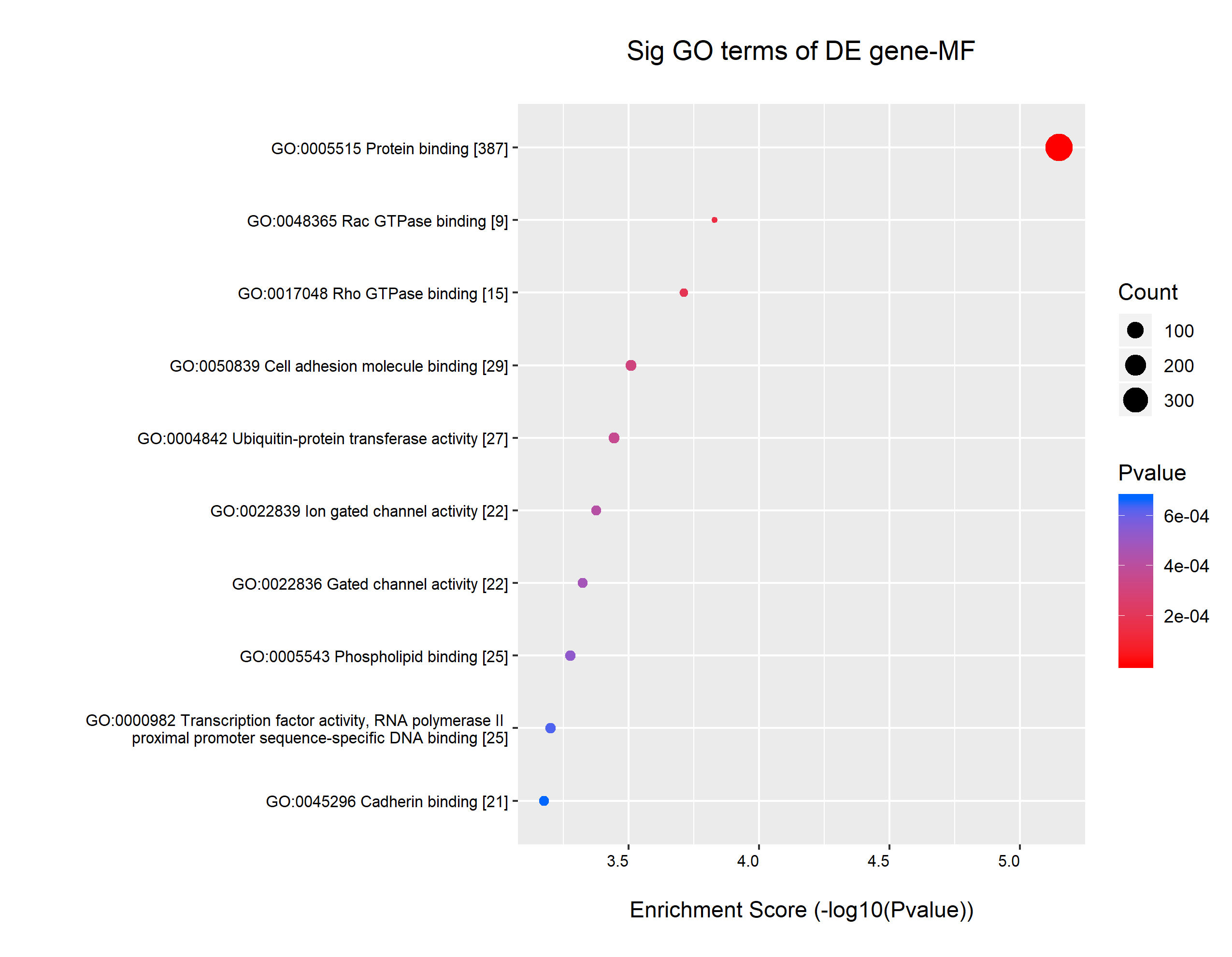

Supplement: Supplementary file 1 [file ijms-22-03792-s001.zip › Supplementary_File/C_ GO_Analysis_Results/16-30nt_go_Makona-96h-Huh7_vs_Control-96h-Huh7_down.mature_mirna_targets/MF_EnrichmentScoreDotPlot.png]

## Sig GO terms of DE gene-MF

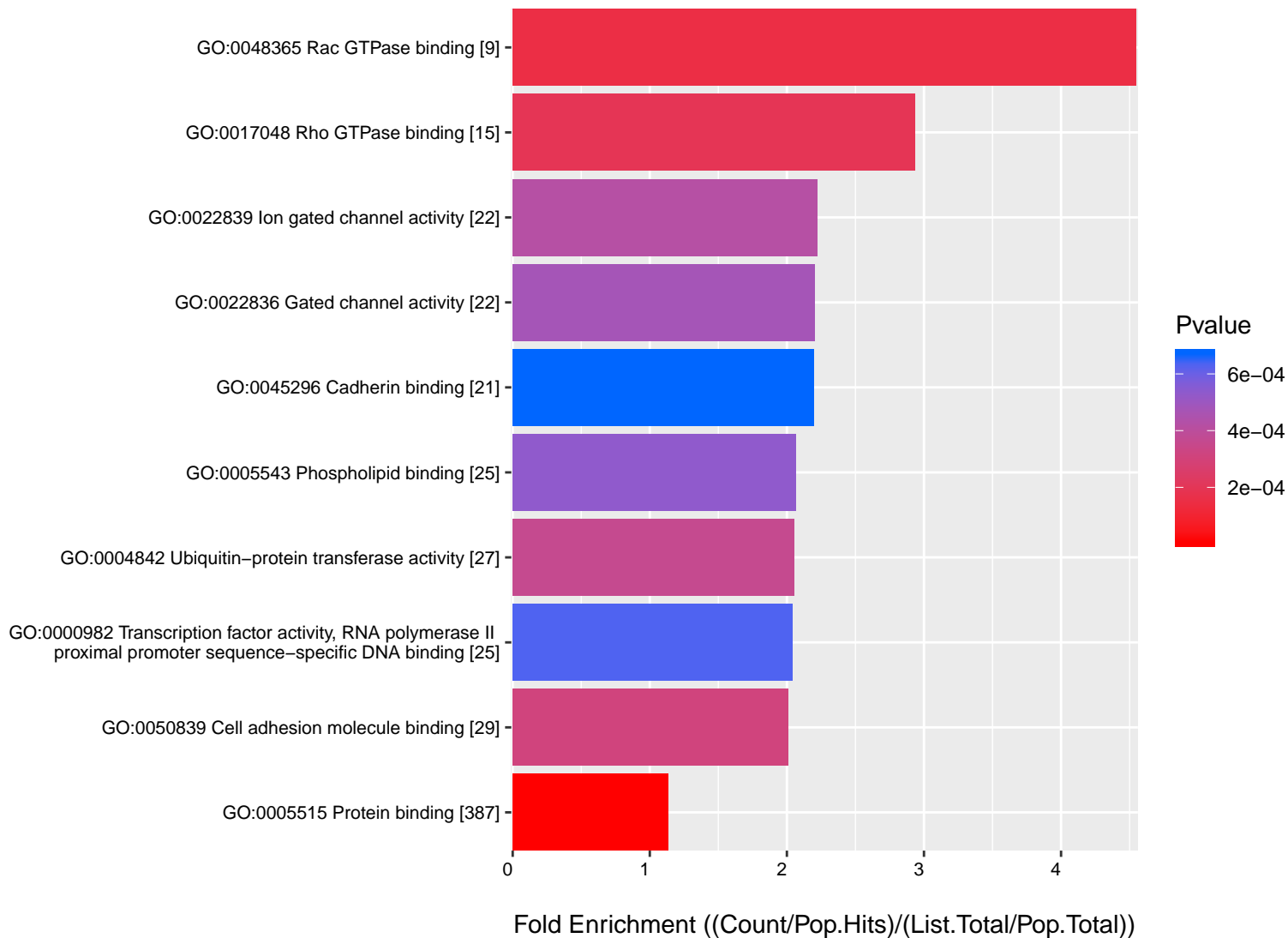

Supplement: Supplementary file 1 [file ijms-22-03792-s001.zip › Supplementary_File/C_ GO_Analysis_Results/16-30nt_go_Makona-96h-Huh7_vs_Control-96h-Huh7_down.mature_mirna_targets/MF_FoldEnrichment.pdf]

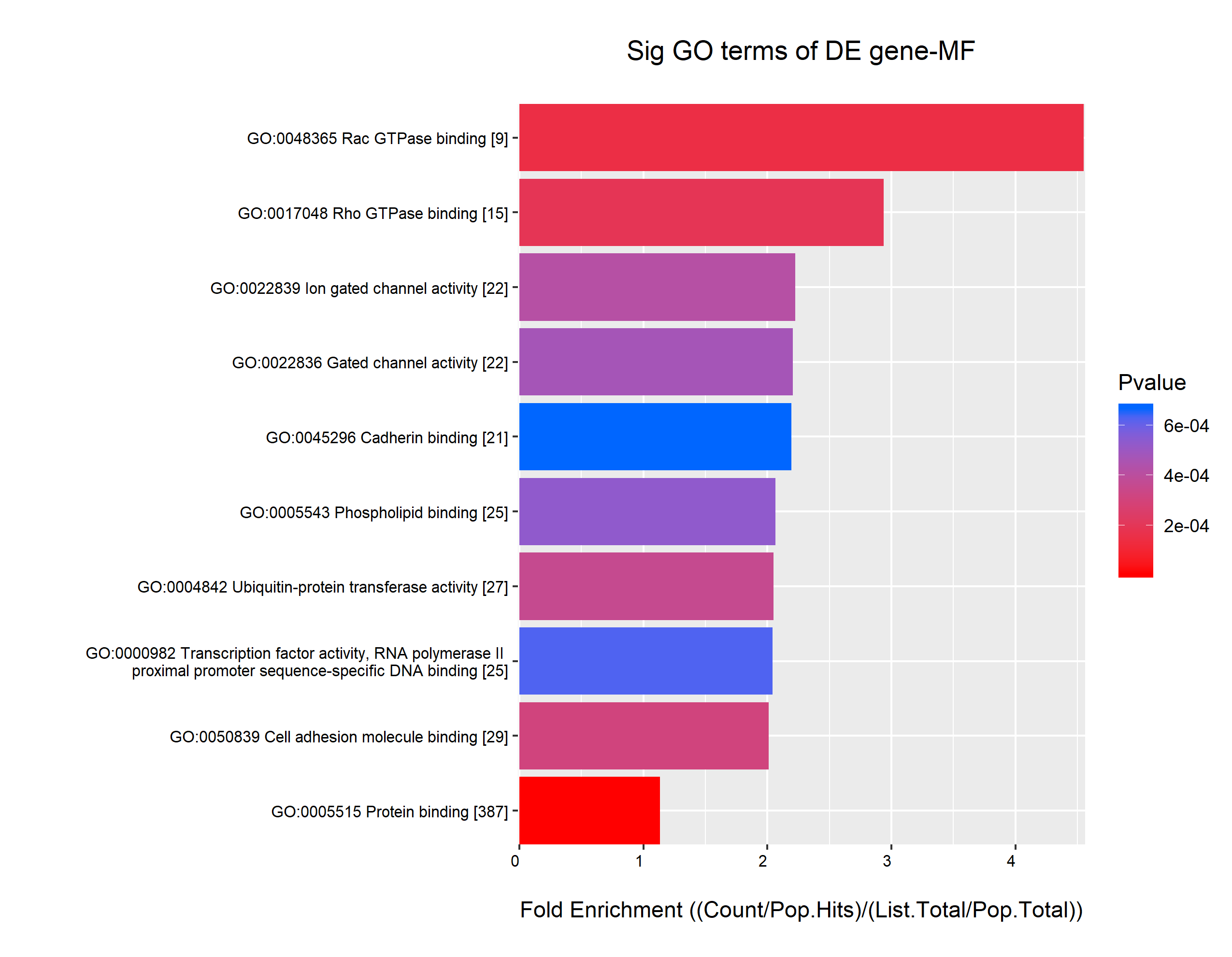

Supplement: Supplementary file 1 [file ijms-22-03792-s001.zip › Supplementary_File/C_ GO_Analysis_Results/16-30nt_go_Makona-96h-Huh7_vs_Control-96h-Huh7_down.mature_mirna_targets/MF_FoldEnrichment.png]

# Sig GO terms of DE gene–MF

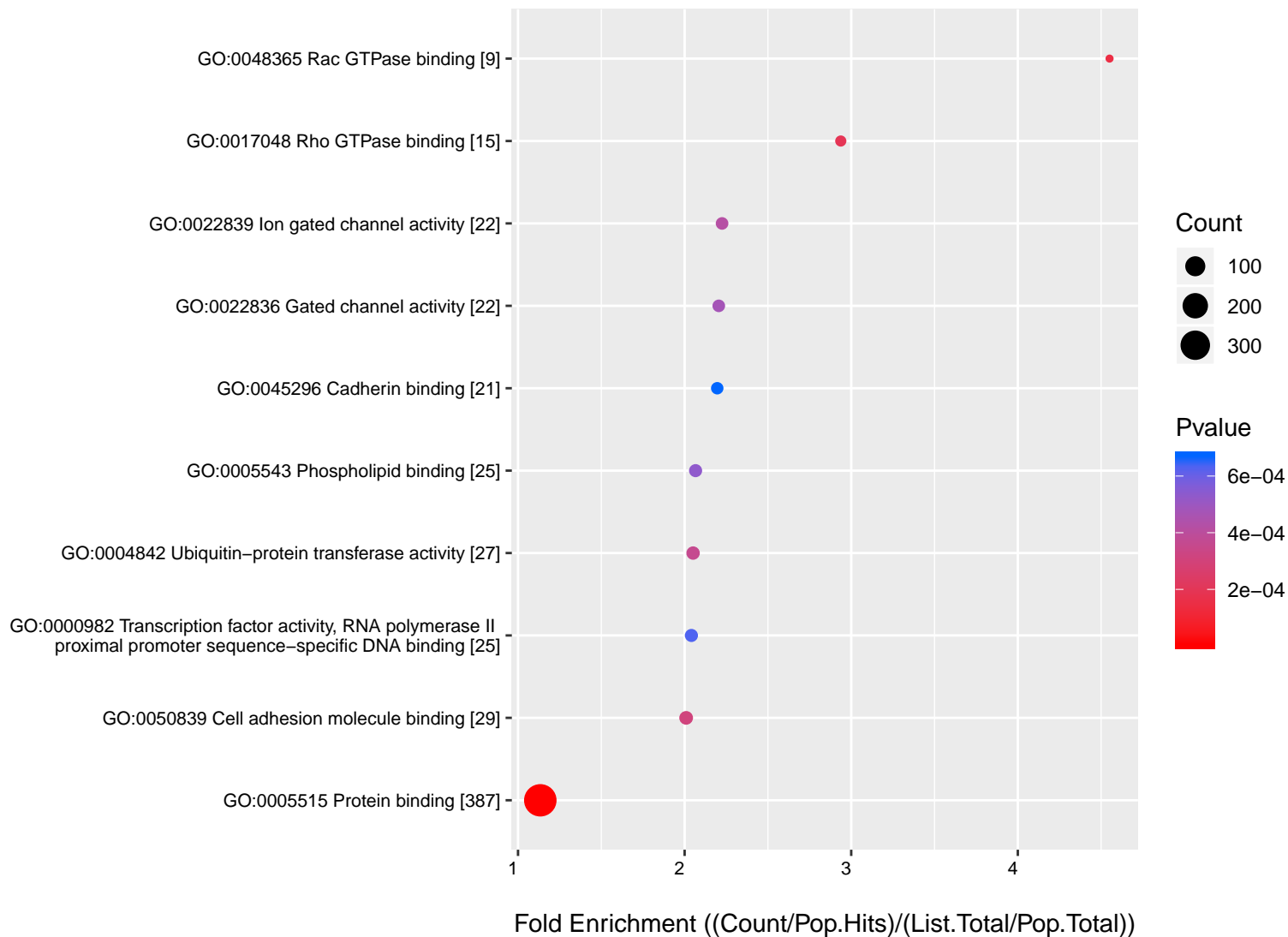

Supplement: Supplementary file 1 [file ijms-22-03792-s001.zip › Supplementary_File/C_ GO_Analysis_Results/16-30nt_go_Makona-96h-Huh7_vs_Control-96h-Huh7_down.mature_mirna_targets/MF_FoldEnrichmentDotPlot.pdf]

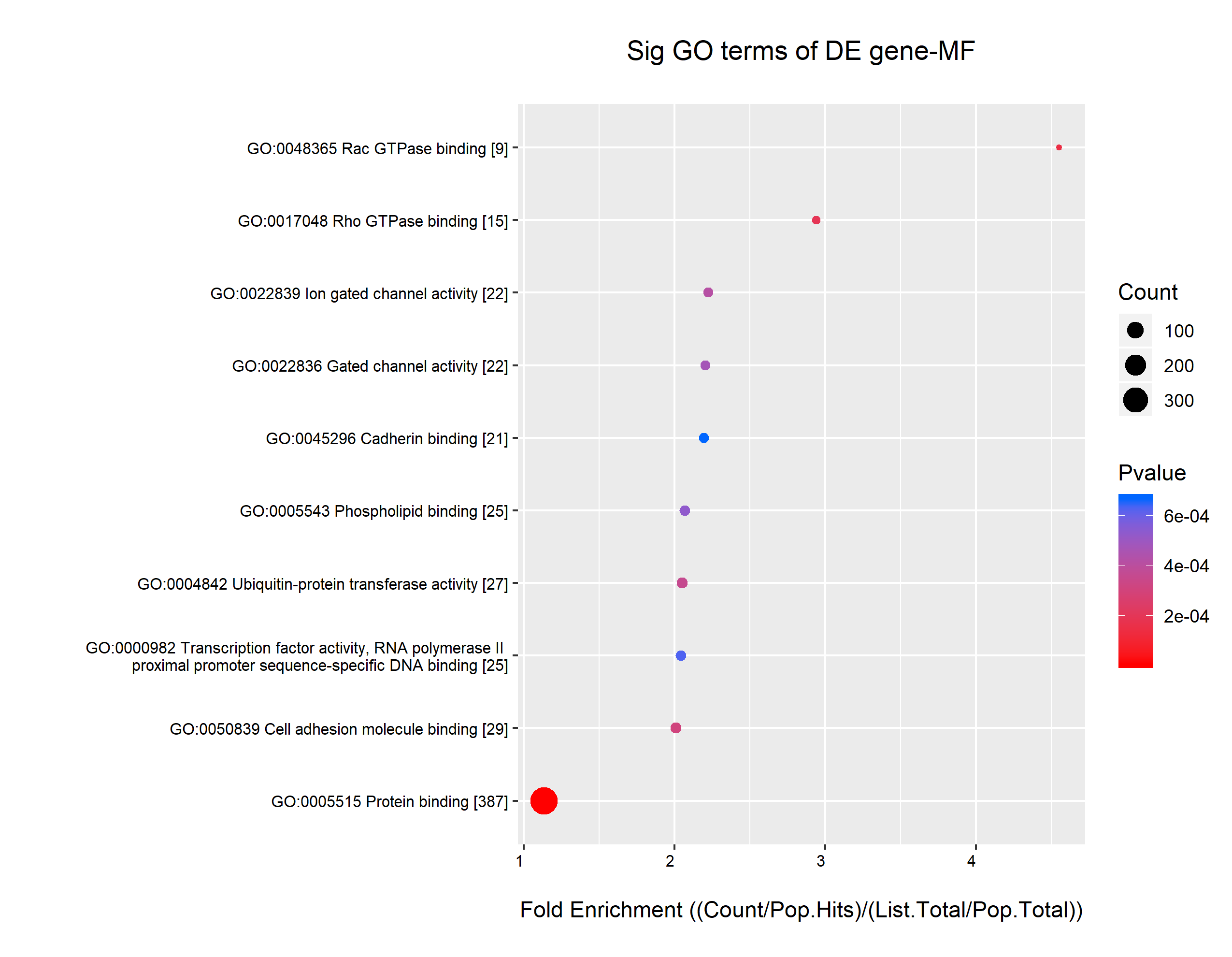

Supplement: Supplementary file 1 [file ijms-22-03792-s001.zip › Supplementary_File/C_ GO_Analysis_Results/16-30nt_go_Makona-96h-Huh7_vs_Control-96h-Huh7_down.mature_mirna_targets/MF_FoldEnrichmentDotPlot.png]

## Sig GO terms of DE gene-MF

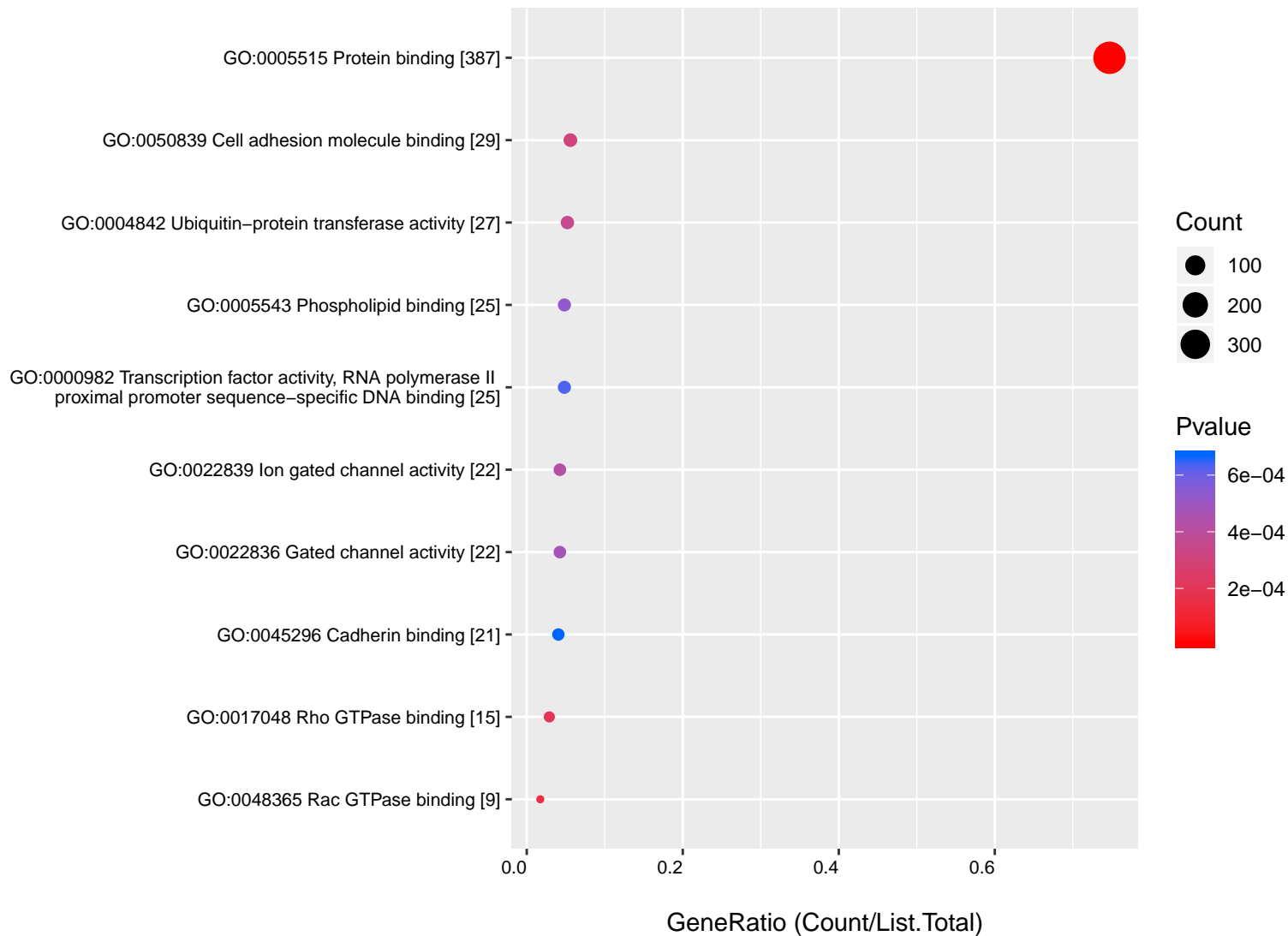

Supplement: Supplementary file 1 [file ijms-22-03792-s001.zip › Supplementary_File/C_ GO_Analysis_Results/16-30nt_go_Makona-96h-Huh7_vs_Control-96h-Huh7_down.mature_mirna_targets/MF_GeneRatioDotPlot.pdf]
