# Supplementary material for: Altered microRNA Transcriptome in Cultured Human Liver Cells upon Infection with Ebola Virus
Source: Int J Mol Sci. 2021 Apr 6;22(7):3792. doi: 10.3390/ijms22073792 (PMC8038836; doi:10.3390/ijms22073792)
Supplement: Supplementary file 1 [file miRNA-target_Network.pdf › Supplementary_File/B_ miRNA_Targets_Prediction/Makona-24h-Huh7_vs_Control-24h-Huh7_down.mature_mirna/miRNA-target_Network.pdf]

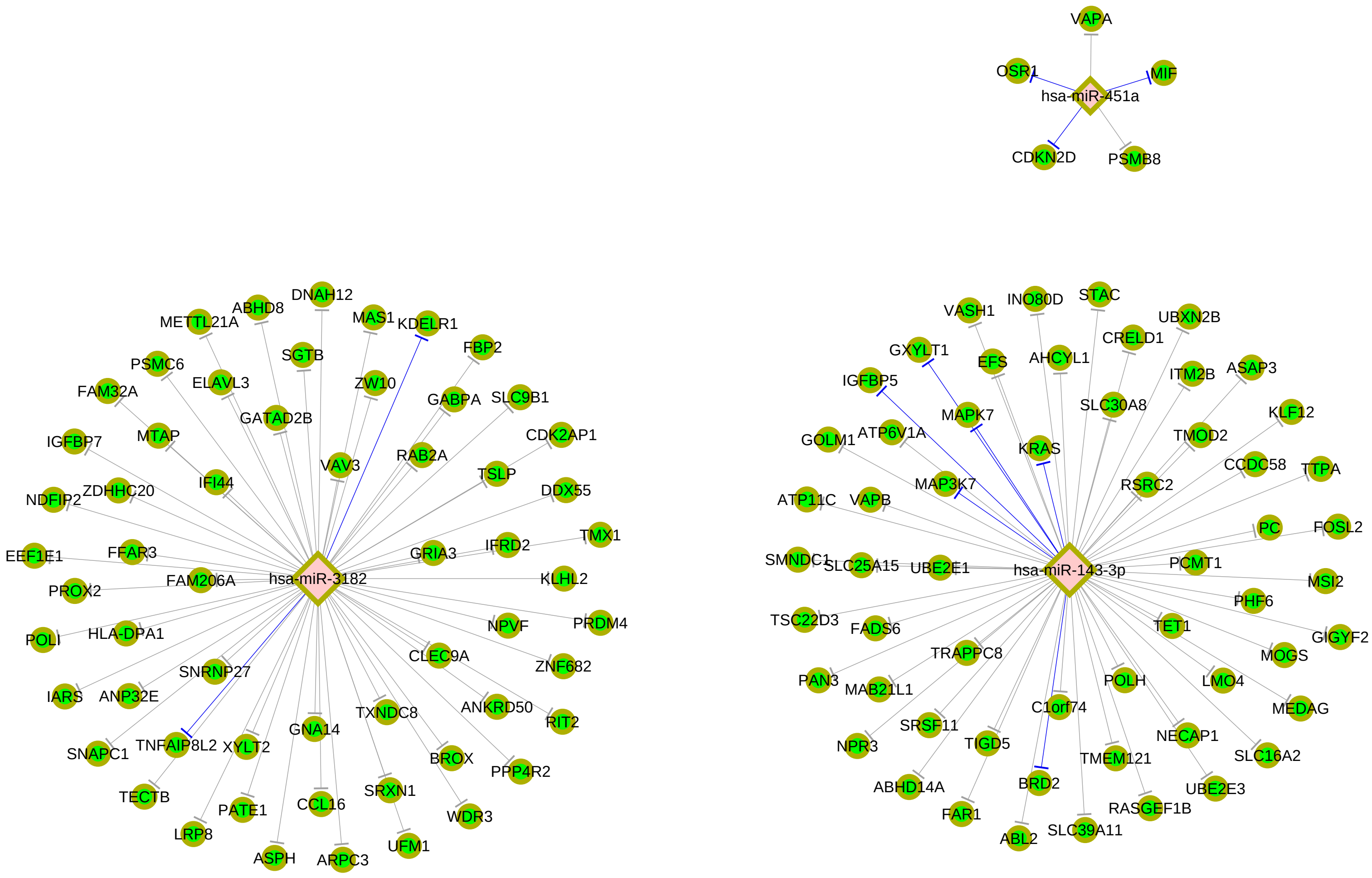
VAPA
OSR1
MIF
hsa-miR-451a
CDKN2D
PSMB8
DNAH12
ABHD8
METTL21A
MAS1
KDELR1
SGTB
FBP2
PSMC6
ELAVL3
ZW10
FAM32A
GATAD2B
GABPA
SLC9B1
IGFBP7
MTAP
CDK2AP1
VAV3
RAB2A
TSLP
IFI44
ZDHHC20
NDFIP2
DDX55
TMX1
IFRD2
GRIA3
EEF1E1
FFAR3
FAM206A
hsa-miR-3182
KLHL2
PROX2
PRDM4
NPVF
POLI
HLA-DPA1
CLEC9A
ZNF682
SNRNP27
IARS
ANP32E
TXNDC8
ANKRD50
RIT2
GNA14
SNAPC1
TNFAIP8L2
XYLT2
BROX
PPP4R2
TECTB
SRXN1
PATE1
CCL16
WDR3
LRP8
ASPH
ARPC3
UFM1
STAC
INO80D
VASH1
UBXN2B
GXYLT1
CRELD1
EFS
AHCYL1
IGFBP5
ITM2B
ASAP3
SLC30A8
MAPK7
KLF12
GOLM1
ATP6V1A
TMOD2
KRAS
CCDC58
TTPA
MAP3K7
RSRC2
ATP11C
VAPB
PC
FOSL2
SMNDC1
SLC25A15
UBE2E1
PCMT1
hsa-miR-143-3p
MSI2
PHF6
TSC22D3
FADS6
TET1
GIGYF2
TRAPPC8
MOGS
PAN3
MAB21L1
POLH
LMO4
MEDAG
C1orf74
SRSF11
NECAP1
NPR3
TIGD5
SLC16A2
TMEM121
ABHD14A
BRD2
UBE2E3
RASGEF1B
FAR1
ABL2
SLC39A11
