# Supplementary material for: Altered microRNA Transcriptome in Cultured Human Liver Cells upon Infection with Ebola Virus
Source: Int J Mol Sci. 2021 Apr 6;22(7):3792. doi: 10.3390/ijms22073792 (PMC8038836; doi:10.3390/ijms22073792)
Supplement: Supplementary file 1 [file miRNA-target_Network.pdf › Supplementary_File/B_ miRNA_Targets_Prediction/Mayinga-24h-Huh7_vs_Control-24h-Huh7_down.mature_mirna/miRNA-target_Network.pdf]

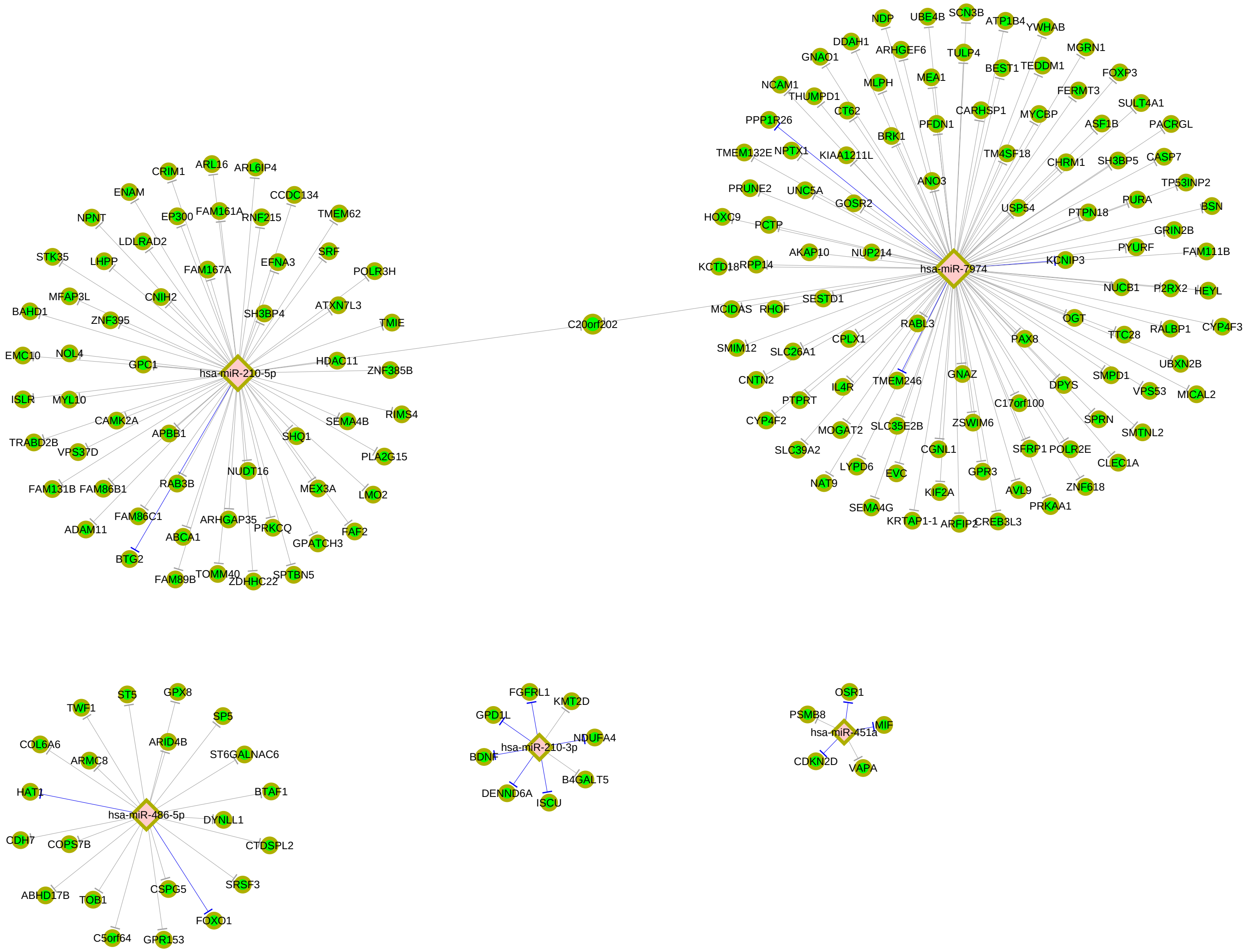
hsa-miR-210-5p
CRIM1
ARL16
ARL6IP4
ENAM
CCDC134
NPNT
EP300
FAM161A
RNF215
TMEM62
LDLRAD2
STK35
LHPP
FAM167A
EFNA3
SRF
POLR3H
MFAP3L
CNIH2
BAHD1
ZNF395
SH3BP4
ATXN7L3
TMIE
EMC10
NOL4
GPC1
HDAC11
ZNF385B
ISLR
MYL10
CAMK2A
RIMS4
TRABD2B
VPS37D
APBB1
SEMA4B
SHQ1
PLA2G15
FAM131B
FAM86B1
RAB3B
NUDT16
MEX3A
LMO2
FAM86C1
ADAM11
ARHGAP35
PRKCQ
FAF2
ABCA1
GPATCH3
BTG2
FAM89B
TOMM40
ZDHHC22
SPTBN5
C20orf202
hsa-miR-7974
NDP
UBE4B
SCN3B
ATP1B4
YWHAB
DDAH1
ARHGEF6
TULP4
MGRN1
GNAO1
MEA1
BEST1
TEDDM1
FOXP3
NCAM1
MLPH
THUMPD1
FERMT3
SULT4A1
CT62
CARHSP1
MYCBP
PPP1R26
PFDN1
ASF1B
PACRGL
BRK1
TMEM132E
NPTX1
KIAA1211L
TM4SF18
CHRM1
SH3BP5
CASP7
ANO3
TP53INP2
PRUNE2
UNC5A
GOSR2
USP54
PURA
BSN
PTPN18
HOXC9
PCTP
GRIN2B
PYURF
AKAP10
NUP214
FAM111B
KCNIP3
KCTD18
RPP14
NUCB1
P2RX2
HEYL
MCIDAS
RHOF
SESTD1
OGT
RABL3
CYP4F3
TTC28
RALBP1
SMIM12
CPLX1
PAX8
SLC26A1
UBXN2B
TMEM246
GNAZ
SMPD1
CNTN2
IL4R
DPYS
VPS53
MICAL2
PTPRT
C17orf100
SPRN
ZSWIM6
SMTNL2
CYP4F2
MOGAT2
SLC35E2B
SFRP1
POLR2E
SLC39A2
CGNL1
CLEC1A
LYPD6
EVC
GPR3
NAT9
ZNF618
AVL9
KIF2A
PRKAA1
SEMA4G
KRTAP1-1
ARFIP2
CREB3L3
hsa-miR-486-5p
GPX8
ST5
TWF1
SP5
ARID4B
COL6A6
ARMC8
ST6GALNAC6
HAT1
BTAF1
DYNLL1
CDH7
COPS7B
CTDSPL2
SRSF3
ABHD17B
TOB1
CSPG5
FOXO1
C5orf64
GPR153
hsa-miR-210-3p
FGFRL1
KMT2D
GPD1L
NDUFA4
BDNF
B4GALT5
DENND6A
ISCU
hsa-miR-451a
OSR1
PSMB8
MIF
CDKN2D
VAPA
