# Supplementary material for: Altered microRNA Transcriptome in Cultured Human Liver Cells upon Infection with Ebola Virus
Source: Int J Mol Sci. 2021 Apr 6;22(7):3792. doi: 10.3390/ijms22073792 (PMC8038836; doi:10.3390/ijms22073792)
Supplement: Supplementary file 1 [file miRNA-target_Network.pdf › Supplementary_File/B_ miRNA_Targets_Prediction/Reston-24h-Huh7_vs_Control-24h-Huh7_down.mature_mirna/miRNA-target_Network.pdf]

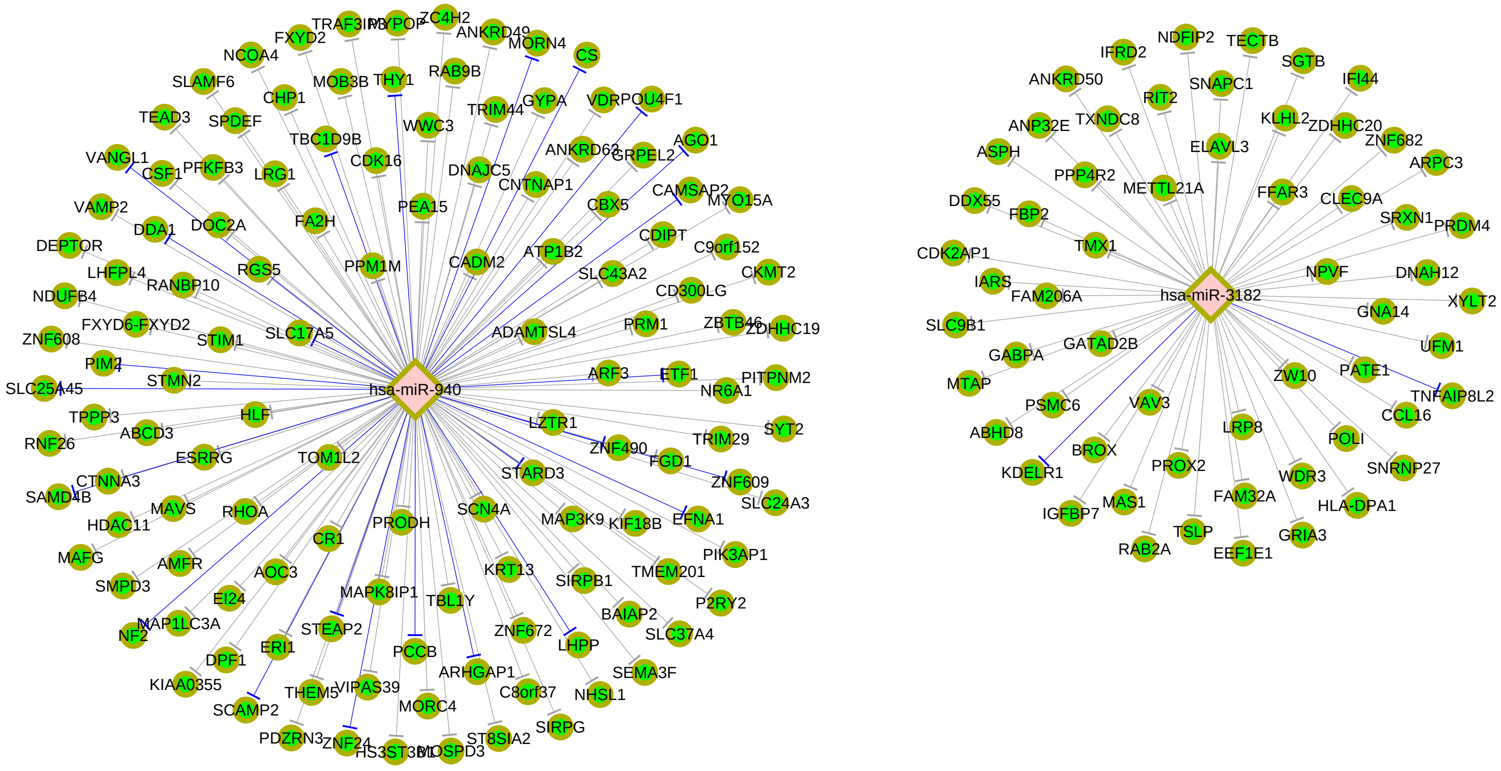
hsa-miR-940
TRAF3IP3
MYPOP
ZC4H2
ANKRD49
FXYD2
NCOA4
MORN4
CS
SLAMF6
CHP1
MOB3B
THY1
RAB9B
GYPA
VDR
POU4F1
TEAD3
SPDEF
TRIM44
WWC3
AGO1
VANGL1
TBC1D9B
CDK16
ANKRD63
GRPEL2
CSF1
PFKFB3
LRG1
DNAJC5
CNTNAP1
CAMSAP2
MYO15A
VAMP2
DDA1
DOC2A
FA2H
PEA15
CBX5
DEPTOR
CDIPT
C9orf152
LHFPL4
RGS5
PPM1M
CADM2
ATP1B2
CKMT2
NDUFB4
RANBP10
SLC43A2
CD300LG
FXYD6-FXYD2
ZBTB46
ZDHHC19
ZNF608
STIM1
SLC17A5
ADAMTSL4
PRM1
PIM2
ARF3
ETF1
PITPNM2
SLC25A45
STMN2
NR6A1
TPPP3
HLF
SYT2
LZTR1
RNF26
ABCD3
TRIM29
TOM1L2
ZNF490
FGD1
ESRRG
STARD3
ZNF609
SAMD4B
CTNNA3
SLC24A3
MAVS
RHOA
SCN4A
MAP3K9
HDAC11
PRODH
KIF18B
EFNA1
MAFG
CR1
PIK3AP1
AMFR
AOC3
KRT13
TMEM201
SMPD3
MAPK8IP1
SIRPB1
EI24
TBL1Y
P2RY2
BAIAP2
NF2
MAP1LC3A
STEAP2
SLC37A4
ZNF672
ERI1
PCCB
LHPP
DPF1
ARHGAP1
SEMA3F
KIAA0355
THEM5
VIPAS39
C8orf37
NHSL1
SCAMP2
MORC4
SIRPG
PDZRN3
ZNF24
ST8SIA2
HS3ST3B1
MOSPD3
hsa-miR-3182
NDFIP2
TECTB
IFRD2
SGTB
SNAPC1
IFI44
ANKRD50
RIT2
KLHL2
TXNDC8
ZDHHC20
ANP32E
ZNF682
ELAVL3
ASPH
ARPC3
PPP4R2
METTL21A
FFAR3
CLEC9A
DDX55
FBP2
SRXN1
PRDM4
TMX1
CDK2AP1
NPVF
DNAH12
IARS
FAM206A
XYLT2
GNA14
SLC9B1
UFM1
GATAD2B
GABPA
PATE1
MTAP
ZW10
TNFAIP8L2
VAV3
PSMC6
CCL16
LRP8
ABHD8
POLI
BROX
KDELR1
PROX2
SNRNP27
WDR3
FAM32A
IGFBP7
MAS1
HLA-DPA1
TSLP
GRIA3
RAB2A
EEF1E1
